# Genome-wide identification and functional prediction of cold and/or drought-responsive lncRNAs in cassava

**DOI:** 10.1038/srep45981

**Published:** 2017-04-07

**Authors:** Shuxia Li, Xiang Yu, Ning Lei, Zhihao Cheng, Pingjuan Zhao, Yuke He, Wenquan Wang, Ming Peng

**Affiliations:** 1 Institute of Tropical Bioscience and Biotechnology, Chinese Academy of Tropical Agricultural Sciences, Haikou 571101, China; 2 National Key Laboratory of Plant Molecular Genetics and National Center for Plant Gene Research (Shanghai), Institute of Plant Physiology and Ecology, Shanghai Institutes for Biological Sciences, Chinese Academy of Sciences, Shanghai 200032, China; 3 Haikou Experimental Station, Chinese Academy of Tropical Agricultural Sciences, Haikou 571101, China

## Abstract

Cold and drought stresses seriously affect cassava (*Manihot esculenta*) plant growth and yield. Recently, long noncoding RNAs (lncRNAs) have emerged as key regulators of diverse cellular processes in mammals and plants. To date, no systematic screening of lncRNAs under abiotic stress and their regulatory roles in cassava has been reported. In this study, we present the first reference catalog of 682 high-confidence lncRNAs based on analysis of strand-specific RNA-seq data from cassava shoot apices and young leaves under cold, drought stress and control conditions. Among them, 16 lncRNAs were identified as putative target mimics of cassava known miRNAs. Additionally, by comparing with small RNA-seq data, we found 42 lncNATs and sense gene pairs can generate nat-siRNAs. We identified 318 lncRNAs responsive to cold and/or drought stress, which were typically co-expressed concordantly or discordantly with their neighboring genes. Trans-regulatory network analysis suggested that many lncRNAs were associated with hormone signal transduction, secondary metabolites biosynthesis, and sucrose metabolism pathway. The study provides an opportunity for future computational and experimental studies to uncover the functions of lncRNAs in cassava.

Plants are sessile organisms and are constantly exposed to a wide range of environmental stresses during their life cycle. Cold and drought are the most severe abiotic stresses that seriously influence plant growth and development, and are major limiters of crop productivity worldwide^1^. To overcome the stress-induced loss of crop yield, it is imperative to develop crop cultivars that are stress-tolerant. Plants respond and adapt to cold and/or drought stresses through complex physiological and biochemical processes that include altered gene expression levels, signal transition pathways, and cellular metabolic rate, thus acquiring resistance^2^. Currently, numerous studies have been conducted to investigate the cold and/or drought-responsive transcriptome and regulatory networks in multiple plant species, including *Arabidopsis*^3^, rice^4^, maize^5^ and wheat^6^. These studies have identified a large number of genes that are induced by cold and/or drought stresses, and are thought to function as key regulators in stress response and tolerance, such as *DEHYDRATION RESPONSE ELEMENT-BINDING PROTEINs* (DREBs) and COLD REGULATED (CORs) genes^7^,^8^.

Long noncoding RNAs (lncRNAs) are generally defined as RNA transcripts that length are more than 200 nucleotide (nt) but lack a coding sequence (CDS) or open reading frame (ORF)^9^. In plants, although some lncRNAs are transcribed by RNA polymerase III or produced by plant-specific RNA polymerase IV/V, the majority of lncRNAs shows a clear signature of RNA polymerase II transcription^10^,^11^. Some lncRNAs are transcribed from intergenic regions (lincRNAs) or introns, while others are long noncoding nature antisense transcripts (lncNATs) that overlap with protein-coding regions on the opposite strand^12^. Early studies attributed lncRNAs to transcriptional noise, because of their low expression and primary sequence conservation compared with mRNAs. However, they have emerged as an important class of regulators in diverse biological processes across eukaryotes^13^,^14^. Studies on the plant lncRNAs have revealed that they play key roles in almost all developmental process, including flowering time^15^, root organogenesis^16^, photo morphogenesis^17^, and reproduction^18^. In particular, plant lncRNAs are recognized as emerging regulatory components in response to abiotic stresses^19^. However, to date, only a few plant lncRNAs with potential roles in abiotic stresses have been characterized. For examples, in *Arabidopsis*, more than 300 lncRNAs that were regulated by various stress stimuli including cold, drought, salt, heat and highlight have been detected, many of which contained conserved elements that might be responsible for the stress-responsive functions of lncRNAs^20^,^21^. Overexpression of npc536 lead to obvious visible differences compared with wild-type plants under salt stress conditions^21^.

Functional analyses of lncRNAs have shown that they are involved in the transcriptional and post-transcriptional regulation of gene expression *via* a number of complex mechanisms. They can function in either cis or trans by sequence complementarity or homology with RNAs or DNAs, and/or by structure, forming molecular frames and scaffolds for assembly of macromolecular complexes^9^. Thus far, although lncRNAs have received more attention in recent years, only a few lncRNAs have been sufficiently described in plant. Interestingly, target mimicry was a recently identified regulatory mechanism for microRNAs (miRNAs) functions and first found in plants. As an endogenous lncRNA, *INDUCED BY PHOSPHATE STARVATION 1 (IPS1*) interacts with miRNAs, which usually post-transcriptionally regulate the abundance of their mRNA targets through cleavage in plants, and function as miRNA target mimics. Pairing with a three-nucleotide bulge, *IPS1* binds to miR399 and destroys the miR399-mediated cleavage of its targets *PHO2* gene^22^. Many endogenous miRNA target mimics have also been predicted by bioinformatics approaches^23^. Some of target mimics of miR160 and miR166 have been experimentally confirmed for their roles in the regulation of plant development^18^,^24^.

To detect and discover novel lncRNAs, several strategies have been employed including both computational and experimental screenings^25^. Recent genome-wide transcriptome analysis approaches, such as tiling arrays and next generation sequencing of full-length cDNA libraries, in model organisms have revealed that much larger portions of non-coding transcripts than previously believed. To date, by analyzing RNA-sequencing (RNA-seq) data, thousands of lncRNAs have been identified in a number of species. For examples, more than 120,000 lncRNAs were identified and annotated across 37 plant species and six algae in the Green Non-Coding Database (GreeNC Database)^26^. In *Arabidopsis*, approximately 6500 lncRNAs were uncovered from 200 transcriptomic data sets, with either organ-specific or stress-induced expression patterns^19^. Wang *et al*. discovered a total of 37,238 lncNATs, which associated with 70% of annotated mRNAs^27^. In rice, by performing whole transcriptome strand-specific RNA sequencing (ssRNA-seq), 2224 lncRNAs involved in the reproductive process were verified^18^. Similarly, by exploiting EST and RNA-seq datasets from 30 different experiments, 1,704 high-confidence lncRNAs were identified in maize^28^.

Cassava is one of the important root crops with stronger stress resistance, while the regulation mechanism response to cold and/or drought stress was largely unknown. The improved RNA-seq technologies make it possible for us to obtain a global view of various RNAs in response to stresses. Thus far, using molecular technologies and ‘omics’ tools, much work like identification of mRNAs and miRNAs has been performed and numbers of RNA data sets were available from various experiments conducted by different laboratories. For examples, the expression patterns of miRNAs were identified in cassava under condition of chilling, heat or drought treatment^29^,^30^. By analyzing oligonucleotide microarray and RNA-seq data, the transcriptomic identification of candidate genes involved cold or drought stress responses have also been reported^31^,^32^,^33^,^34^,^35^. iTRAQ-based proteomic analysis was employed to study different strategies of cassava in response to drought stress^36^. However, these data sets have not yet been utilized to explore and study lncRNAs in cassava, and the mechanism of lncRNAs participating cold- and/or drought-tolerance remains obscure, and thus needs to be further explored.

Here, we used a strand-specific RNA sequencing approach to investigate the genome-wide transcriptome reconfiguration of cassava challenged by low-temperature and polyethylene glycol-simulated drought stress in a high-throughput manner. We systematically identified first landscape of novel lncRNAs with a specific focus on the lncRNAs exhibited stress-regulated expression patterns. The potential function of lncRNAs and their target genes were also predicted and analyzed. Our results indicated that a number of cassava lncRNAs response to cold and/or drought stress, and enhanced our understanding of putative regulatory functions of lncRNAs in plants.

## Results

### Experiments to explore cold and/or drought response and transcriptome sequencing of cassava seedlings

To systematically identify lncRNAs related to cold and/or drought stress in cassava, we performed whole transcriptome ssRNA-seq of cassava shoot apices and young leaves from 15-day-old seedlings under low temperature (4 °C, 24 hour), PEG-simulated drought conditions (20% PEG, 6 hour) and control conditions. This protocol was chosen for two reasons: (i) we quantified the levels of transcripts of cold and/or drought responsive genes (known DREB/CBFs and CORs/RD29) using RNA derived from samples exposed to cold stress at 4 °C or drought stress for different time points, the expression of the stress-induced genes is maximal at 24 hour after cold stress and at 6 hour after PEG treatment, respectively (Supplemental Figure S1). (ii) We observed visible stem bending and leave wilting in PEG-induced drought stress treatments (Supplemental Figure S1). The phenotypic changes under cold stress were less severe than those under drought stress. These physiological differences indicated that plants under stress conditions may show significant changes in gene expression (including lncRNAs). Moreover, according to the precious study, soluble protein content of cassava leaves showed steady increase during the first 6 to 8 h 20%PEG treatment and decreased in prolonged drought stress conditions^32^, indicating the accumulation of the stress-induced transcripts reached a high peak at 6 h PEG treatment. Hence, 24 hour cold stress treatment and 6 hour PEG-induced drought stress treatments were the sole conditions that allowed a clear discrimination of expression of stress responded transcripts. We generated three independent biological replicates in these cases.

### Genome-wide identification of novel lncRNAs in cassava

**Figure 1:**
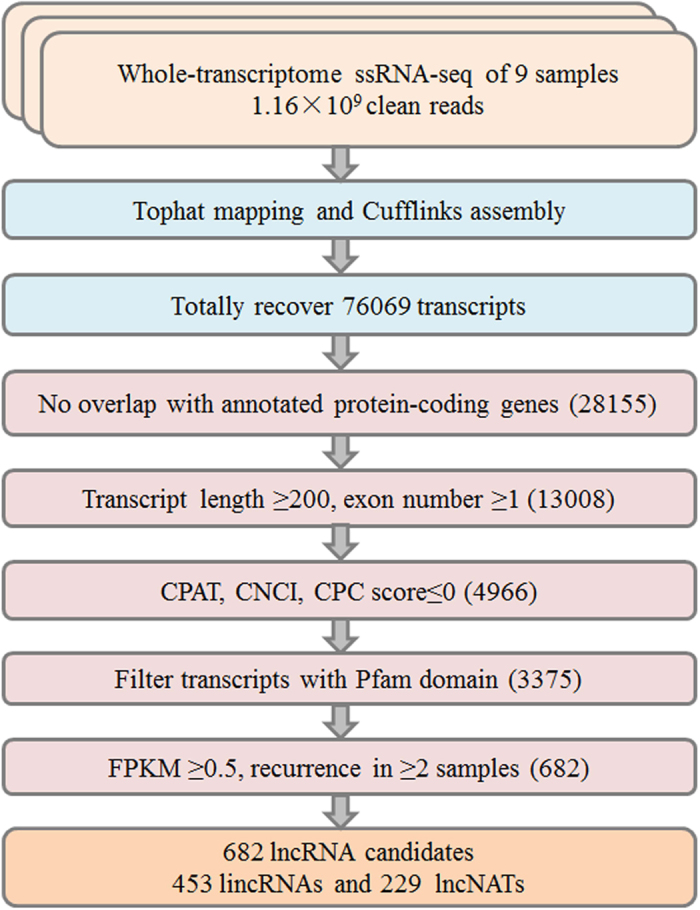
An integrative computational pipeline for the systematic identification of lncRNAs in cassava. CPAT, Coding-Potential Assessment Tool; CNCI, Coding-Non-Coding Index; CPC, Coding Potential Calculator; lincRNA, long intergenic non-coding RNA; lncNAT, long non-coding natural antisense transcript.

In total, 140 gigabases (Gb) raw reads of 125-bp length were generated of 9 samples by paired end sequencing with Illumina HiSeq 2500 machine. After trimming adapters and filtering out low quality reads, approximately 116 million clean reads with 62–64% mapping to the *Manihot esculenta* genome were obtained and were used for further analysis. To characterize cassava lncRNAs, we developed a computational identification pipeline based on whole transcriptome ssRNA-seq data (Fig. 1). The cassava transcriptome was reconstructed from all of the RNA-seq datasets using cufflink 2.0^37^. A total of ~7.2 million transcripts were obtained among 9 samples. Four filter processes were applied to distinguish lncRNAs from protein-coding transcript units. First, we removed transcripts that were overlapping with known protein-coding genes in sense. We totally discovered 76,069 transcripts and most of the transcripts (63.08%) were mRNAs. Second, we filtered transcripts with length <200nt. Then, we evaluated the coding potential of the remaining transcripts and obtained novel expressed lncRNAs. We used the Coding Potential Calculator (CPC)^38^, Coding-Potential Assessment Tool (CPAT)^39^ and Coding-Non-Coding Index (CNCI)^40^ to predict the coding potential of each transcript. All transcripts with scores >0 were discarded. To guarantee the thorough elimination of protein-coding transcripts, we also employed HMMER to scan each transcript to exclude transcripts that encoded any of the known protein domains cataloged in the Pfam protein family database^41^,^42^. Finally, after filtering out those FPKM (fragments per kilobase of transcript per million mapped reads)^43^ scores <0.5, which indicated infrequently expressed transcripts, and transcripts contained in only one sample, we obtained 682 reliably expressed novel lncRNAs, including 453 lincRNAs and 229 lncNATs (Supplemental Data S1). In addition, we aligned the 682 lncRNAs with GreeNC database, and found all these lncRNAs have not been annotated in cassava.

### Characterization of cassava lncRNAs

**Figure 2:**
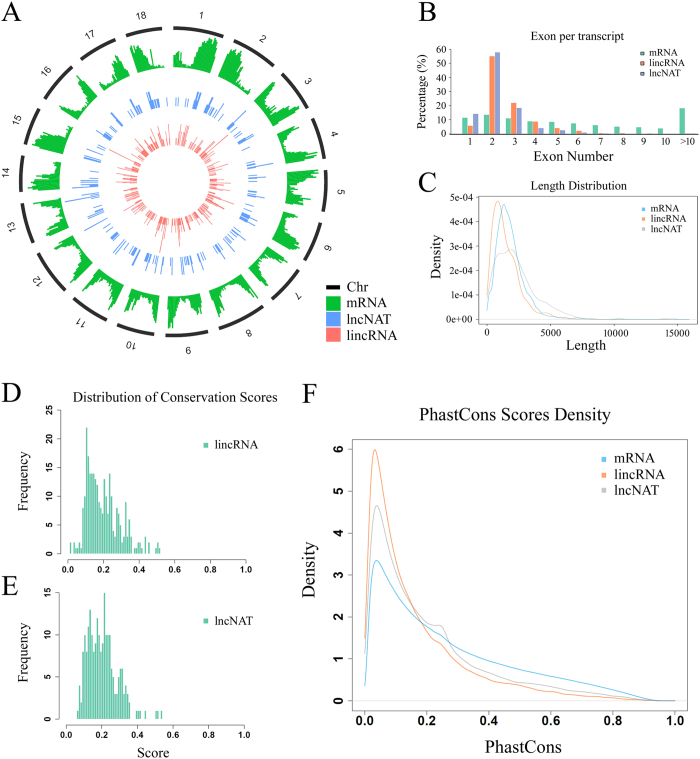
Characteristics of cassava lncRNAs. (**A**) Distribution of mRNAs, lincRNAs and lncNATs along each chromosome. The abundance of mRNAs, lincRNAs and lncNATs in physical bins of 1 Mb for each chromosome (generated using ggbio R package). (**B**) The number of exons per transcript for all mRNAs, lincRNAs and lncNATs. (**C**) Transcript size distributions for all mRNAs, lincRNAs and lncNATs. (**D**,**E**) Conservation scores distributions for lincRNAs (**D**) and lncNATs (**E**), respectively. (**F**) The level of conservation of mRNAs, lincRNAs and lncNATs.

We characterized the basic genomic features of the obtained lncRNAs and compared these features with cassava protein-coding genes where appropriate. We found that the pericentromeric regions of most cassava chromosomes have lower density of mRNAs and lncNATs than chromosome ‘arms’, while cassava lincRNAs are more evenly distributed across chromosomes (Fig. 2A). Most (~55%) of the cassava lincRNAs and lncNATs contains two exons, while the majority of mRNAs consist of more than 10 exons (Fig. 2B). Full-length cassava lincRNAs are generally shorter than protein-coding transcripts, while the majority of lncNATs are relatively longer than mRNAs and lincRNAs (Fig. 2C). We further measured the conservation of lncRNAs through genomic alignment between the genomes of cassava and *Arabidopsis*, and obtained the conservation score of cassava lincRNAs and lncNATs (Fig. 2D,E). By comparing phastCons^44^ scores of mRNA and lncRNAs, as expected, the conservation of lncRNAs is substantially lower than that of protein coding genes. However, we found lncNATs were more conserved than lincRNAs (Fig. 2F).

### Variation in lncRNA expression among stresses

**Figure 3:**
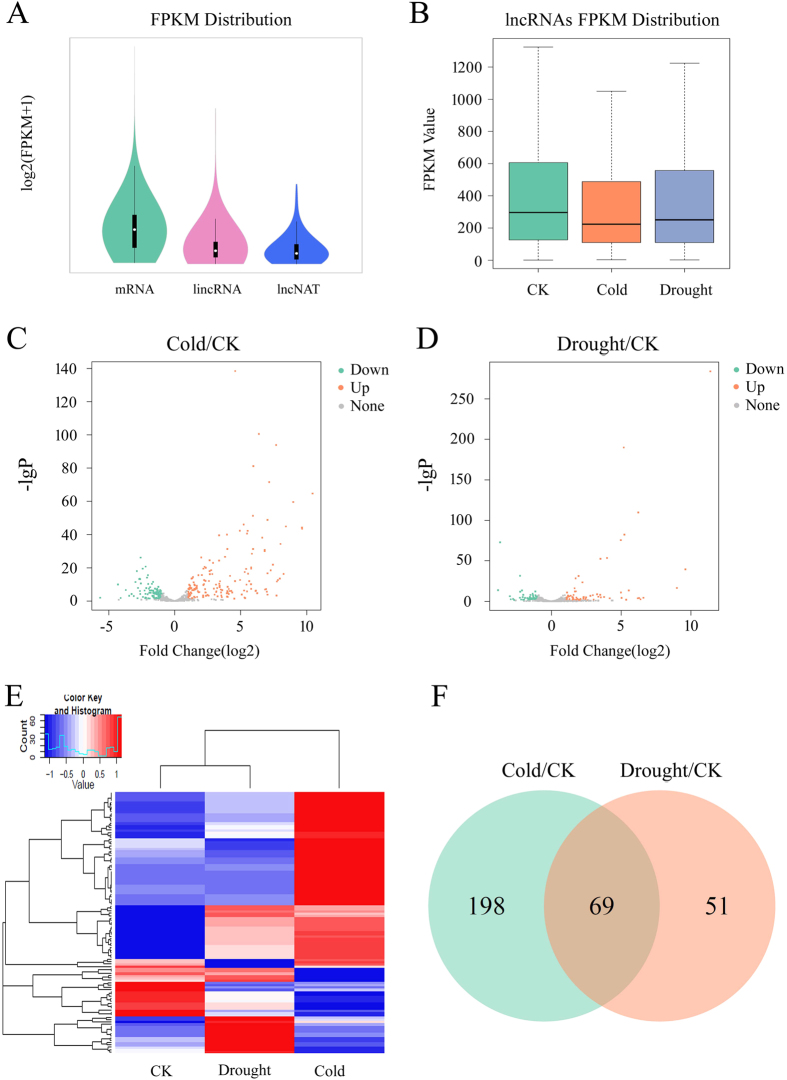
Variation in lncRNA expression under cold or drought stress. (**A**) The violin plot of expression levels of mRNAs, lincRNAs and lncNATs. FPKM, fragments per kilobase of exons per million fragments mapped. (**B**) The box plot of expression levels of lncRNAs under control, cold and drought stress. (**C**,**D**) The Volcano plot of differential expressed (**D**,**E**) lncRNAs between cold stress and control (**C**), drought stress and control conditions (**D**), respectively. (**E**) Hierarchical clustering of the maximal JS (Jensen-Shannon) specificity score of lncRNA under cold or drought stress. (**F**) Venn diagram of DE-lncRNAs under cold and/or drought stress.

We then systematically estimated the expression level of all transcripts identified including lncRNAs and mRNAs using FPKM. The results showed that the majority of lincRNAs and lncNATs were expressed at similar levels and were relatively lower than mRNAs (Fig. 3A). The lower expression level and highly differentiated expression pattern of lncRNAs were also found in *Arabidopsis* and rice^18^,^19^, suggesting that both of these characteristics are conserved for lncRNAs. To explore the transcriptional activity of lncRNAs from all samples in response to different stresses, we compared global expression levels of lncRNAs among normal, cold and drought conditions (Supplemental Data S2). Intriguingly, we found that average abundance of lncRNAs under cold stress tend to be lower as compared to control condition, while lncRNAs under drought stress exhibited a similar level of expression to that of control (Fig. 3B). Next, we identified significantly differentially expressed lncRNAs (DE-lncRNAs) under cold or drought stress as compared to normal condition (Supplemental Data S2). The results indicated that approximately 24.05% lncRNAs presented a significantly up-regulated pattern while only about 14.81% lncRNAs were significantly down-regulated under cold treatment (Fig. 3C). Similarly, nearly 9.82% lncRNAs were strongly induced and 7.78% lncRNAs were repressed by drought treatment (Fig. 3D). We further estimated the degree of the differential expression of lncRNAs based on the JS (Jensen-Shannon) score^45^. We observed that lncRNAs have highly stress-specific expression pattern (Fig. 3E). We clustered the lncRNAs based on their expression patterns under cold stress, drought stress and control conditions. Remarkably, various expression differences of these lncRNAs were found among stress and control conditions, suggesting that these DE-lncRNAs may play an important role in cold and/or drought stress response (Fig. 3E). Finally, we surveyed the union of DE-lncRNAs response to both cold and drought stress. We found that there were 69 DE-lncRNAs under both stresses compared to control, among which, 40 lncRNAs showed an acquired expression pattern (up-regulated) for the induction of both cold and drought; and there were 198 cold-specific DE-lncRNAs and 51 drought-specific DE-lncRNAs respectively (Fig. 3F, Supplemental Data S2). Taken together, we identified a total of 318 lncRNAs that respond to cold and/or drought stress.

### Validation of lncRNA expression using qRT-PCR

**Figure 4:**
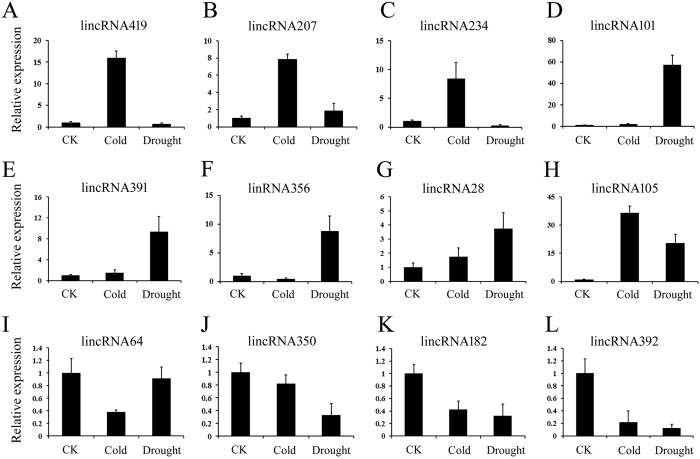
Confirmation of the expression patterns of lncRNAs using quantitative RT-PCR. (**A**–**L**) The expression pattern of lncRNAs under cold or drought stress. The values shown are the means ± standard deviation of three replicates. *MeACTIN* was used as the reference gene.

To further characterize cold- and/or drought- responsive lncRNAs listed in supplemental data S2 in cassava, 12 lncRNAs under cold and/or drought conditions were randomly selected and analyzed by real-time quantitative PCR (qRT-PCR). As shown in Fig. 4, the expression patterns of the stress-responsive lncRNAs as investigated by RNA-seq and qRT-PCR were relatively consistent with similar trends, suggesting that the lncRNA expression patterns based on RNA-seq data are reliable. The qRT-PCR results confirmed that 85% of lncRNAs (8 of the 12 detected lncRNAs) were cold- or drought- inducible in cassava. Among them, we found that three lncRNAs (lincRNA419, 207 and 234) were specifically up-regulated under the cold condition (Fig. 4A,B and C), while lincRNA101, 391 and 356 were uniquely up-regulated under the drought condition (Fig. 4D,E and F). The striking differences in the response pattern between the two treatments suggested that these lncRNAs may play different roles in cassava under the two types of stresses. Moreover, lincRNA28 and lincRNA105 were up-regulated after both cold and drought treatment (Fig. 4G,H), while four lncRNAs (lincRNA64, 350,182 and 392) were down-regulated under cold and/or drought treatment (Fig. 4I–L). The action mode of these lncRNAs in cassava will be further studied.

### LncRNAs as potential miRNA precursors

MiRNAs are small RNAs of 20–22 nt in length, which generated via sequential cleavage of long precursor transcripts with stem-loop by Dicer-like 1 (DCL1), and play important roles in the regulation of gene expression at both transcriptional and post-transcriptional levels^46^. By aligning miRNA precursors to the 682 lncRNAs, we identified 12 lncRNAs as 11 known cassava miRNA precursors, including miR156g, miR160d, miR166h, miR167g and miR169d (Supplemental Data S3). Among these lncRNAs, 7 lncRNAs are differentially expressed in response to cold and/or drought stress, suggesting these miRNAs may be involved in stress responses.

### Functional prediction of several lncRNAs acting as miRNA target mimics

**Figure 5:**
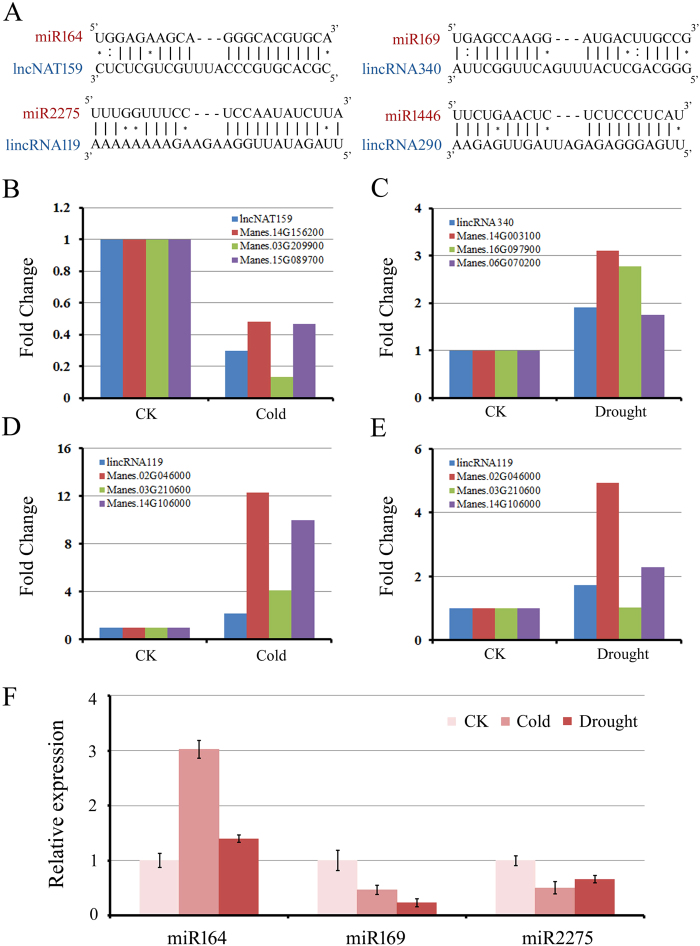
Functional prediction of cassava lncRNAs as miRNA target mimics. (**A**) Predicted base-pairing interaction of miR164-lncNAT159, miR169-lincRNA340, miR2275-lincRNA119 and miR1446-lincRNA290. (B-E) Relative transcript abundances of lncNAT159 (**B**), lincRNA340 (**C**), lincRNA119 (**D**,**E**) and targets of miR164 (**B**), miR169 (**C**), and miR2275 (**D**,**E**) under cold or drought stress, respectively. (**F**) Relative expression of miR164, miR169 and miR2275 under cold or drought stress. U6 was used as the reference gene.

It has been shown that lncRNAs can serve as miRNA target mimicry to interfere with the miRNA-mediated regulation of their targets in plants. Similar to the interactions of miRNAs with their targets, miRNA target mimicry also rely on the sequence-dependent interaction of miRNAs with lncRNAs, except for the bulges in the middle of miRNA-lincRNA duplexes^22^. We predicted lncRNAs that might act as miRNA target mimicry using the algorithm developed by Wu *et al*.^24^. In total, we found that 16 of the identified lncRNAs that may act as miRNA mimics bound by conserved miRNAs, such as miR164, miR169, miR2275 and miR1446 (Fig. 5A, Supplemental Data S4). Considering the possible role of these lncRNAs in regulating the transcript levels of miRNA targets, we further investigated coordination of lncRNAs and miRNA-targeted genes expression after cold or drought treatment using RNA-seq data. Interestingly, we found lncRNAs was positively correlated with miRNA-targeted genes expression. For example, we found a cold-repressive lincRNA159, binding miRNA164 with three mismatches, coordinated with decrease of the expression of miR164-targeted *NAC (NAM, ATAF1*/*2, CUC2*) genes under cold treatment (Fig. 5B). On the other hand, expression of lincRNA340 was induced by drought stress, accompanied by an increase of miR169-targeted NUCLEAR FACTOR Y (NF-Y) genes after drought treatment (Fig. 5C). It is known that miR164 and miR169 participate in regulating cold and/or drought stress responses in plants^47^,^48^. Thus, these two miRNA-lncRNAs functional pairs might be important regulators of abiotic stresses responses. In addition, in response to both cold and drought stress, induction of lincRNA119 was positively correlated with dramatically increased the mRNA abundance of corresponding miR2275 targets (Fig. 5D,E). To further understand the relationship of miRNA target mimics and correlated miRNAs, we analyzed the expression level of miR164, miR169 and miR2275 under stress conditions by qRT-PCR, respectively. We found miR164 was significantly up-regulated by cold and drought stress, especially after cold treatment, while miR169 and miR2275 exhibited decreased expression pattern during all stress conditions (Fig. 5F). This result is consistent with the expression pattern of miRNA targets and miRNA target mimics. Taken together, these results strengthen our assumption that lncRNAs may be involved in inhibiting the functions of miRNAs.

### Identification of stresses responsive lncNAT-siRNAs in cassava

**Figure 6:**
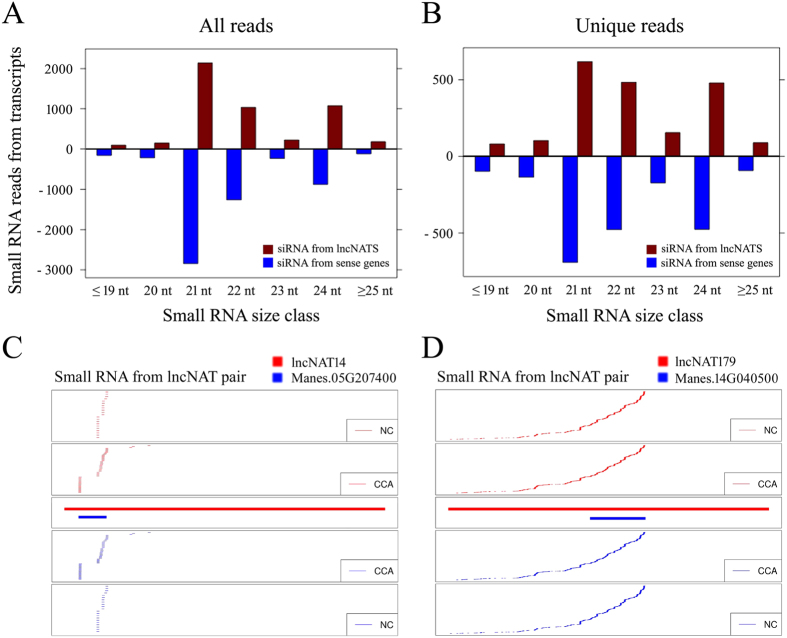
Small RNAs generated from lncNAT-sense pairs. (**A**,**B**) Number of total (**A**) and unique (**B**) small RNA reads derived from lncNATs and the corresponding sense transcripts, respectively. (**C**,**D**) Small RNAs distribution along lncNAT14-Manes.05G207400 (**C**) and lncNAT179-Manes.14G040500 (**D**) are shown for both NC and CCA libraries, respectively.

Previous research has suggested that NAT, which pairs of endogenous coding or non-coding RNAs with perfect complementarity to each other, may generate a double stranded RNA duplex to produce siRNAs^49^,^50^. Nat-siRNAs has been documented to regulate gene expression in plants^51^. Since nat-siRNAs generated from pairs of endogenous coding RNAs has been investigated in cassava^52^, the question arises whether the lncNAT-siRNAs from lncNAT and sense RNA pairs are indeed produced in response to cold and/or drought stress. To investigate abiotic stress effects on siRNA generation from lncNAT pairs, we used public small RNA-seq data of three kinds of cold treatment, including chilling acclimation (CA, 24 to 14 °C), chilling stress after chilling acclimation (CCA, 14 to 4 °C), chilling shock (CS, 24 °C to 4 °C) and normal control (NC, 24 °C)^53^. This small RNA-seq data set was derived from cassava leaves exposed to low temperature, using experimental conditions similar to those used for our ssRNA-seq data. Therefore, we aligned siRNA enrichments at the gene body regions of lncNATs and sense genes. All 19- to 25-nt siRNAs mapped to lncNATs and their complementary regions were taken as potential lncNAT-siRNAs. Our results showed that a large number of lncNATs (18.34%) may serve as precursors of lncNAT-siRNAs under CCA and/or CS conditions (Supplemental Data S5). Among them, 45.2% of lncNATs were significantly regulated by cold and/or drought stress (Table 1). The total and unique mapped lncNAT-siRNAs reads had lengths peaked at 21-nt, 22 nt and 24-nt (Fig. 6A,B), which is well consistent with previous studies^52^. Focusing on cold-regulated lncNATs, for example, a total of 2127 siRNAs reads aligned to the overlap region of the two lncNAT pairs, lncNAT14-Manes.05G207400 and lncNAT179-Manes.14G040500, arranged in a convergent fashion (Fig. 6C,D, Table 1). Specifically, siRNAs from lncNAT14 pairs were induced under CCA treatment compared to NC (Fig. 6C). These results suggest that siRNA-mediated regulation of lncNATs pairs may contribute to cold signaling pathways.

### Functional prediction of stress-responsive lincRNA targets in cis-regulatory relationships

**Figure 7:**
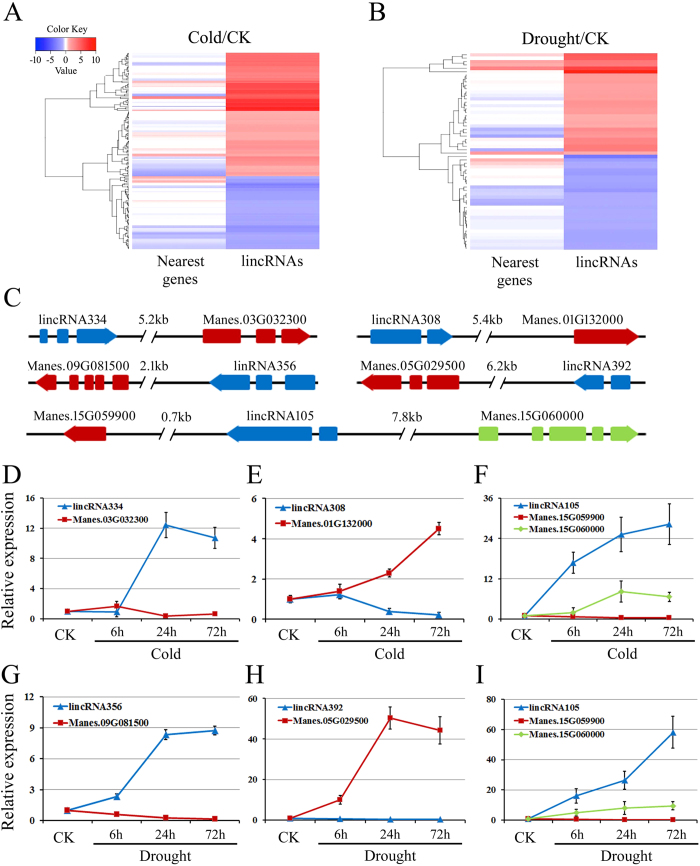
Comparison of the expression pattern of lincRNAs and adjacent protein-coding genes. (**A**,**B**) A heatmap was generated from the fold change values in the RNA-seq data, and was used to visualize the lincRNA and neighbors expression pattern under cold (**A**) and drought stress (**B**), respectively. (**C**) Gene structures of five lincRNAs and nearby protein-coding genes. (**D**–**I**) Expression patterns analysis of lincRNA334 (**D**), lincRNA308 (**E**), lincRNA356 (**G**), lincRNA392 (**H**), lincRNA105 (**F**,**I**) and nearby protein-coding genes by qRT-PCR under cold or drought stress, respectively. Data are presented as means ± SD of three independent replicates. *MeACTIN* was used as the reference gene.

There are relatively few functionally characterized lncRNAs in cassava. Some mammalian lncRNAs can function in cis as scaffolds to recruit co-activator complexes that mediate chromosome looping between enhancer and promoter regions, controlling chromatin topology and modulating gene expression at the transcriptional level^54^. Emerging evidence showed that lncRNAs tend to influence the expression of adjacent genes or overlapping genes^55^,^56^. To investigate the possible functions of cassava lncRNAs, we predicted the potential targets of lincRNAs in cis-regulatory relationships. We searched for known protein-coding genes located within 10 Kb upstream and downstream of all the identified cassava lincRNAs. Interestingly, we found most of lincRNAs (76.6%) that were transcribed close to 524 protein-coding neighbors (Supplemental Data S6). We classified stress-responsive lincRNAs-neighbors pairs into two groups: concordant regulated group and discordant regulated group. For concordant lincRNAs-neighbors pairs, the transcripts abundance of neighbor genes should change in the same direction with its lincRNAs partner no less than 1.5 fold. For discordant pairs, the expression of adjacent genes should change in the opposite direction no less than 1.5 fold. We identified 98 and 81 concordant lincRNAs-neighbors pairs potentially involved in cold and drought stress response pathways, respectively. Interestingly, we also found 40 and 35 cold- and drought-responsive discordant lincRNAs-neighbors pairs, respectively (Fig. 7A,B, Supplemental Data S6). We carried out qRT-PCR to confirm the transcription of five lincRNAs and their adjacent protein-coding genes under cold and/or drought stress conditions (Fig. 7C). The results showed that the regulation of these discordant lincRNAs pairs displayed a similar pattern as RNA-seq data (Fig. 7D–I). For examples, the expression of Manes.09G081500, coding a protein tyrosine kinase, was specifically down-regulated after 6 to 72 h of drought treatment accompanied by its up-regulated adjacent lincRNA356 (Fig. 7C,G). During cold treatment, this discordant pair did not show more than two fold expression differences. Moreover, Manes.15G059900 and Manes.15G060000 were transcribed oppositely with their adjacent lincRNA105 under cold and drought stress (Fig. 7C,F and I). It will be interesting to check the function of this lincRNAs in abiotic stress responses. Therefore, a number of discordant lincRNA-neighbor pairs that were identified suggested a potentially widespread occurrence of negative regulation by lincRNAs.

### Functional prediction of protein-coding genes co-expressed with DE-lncRNAs in trans-regulatory relationships

**Figure 8:**
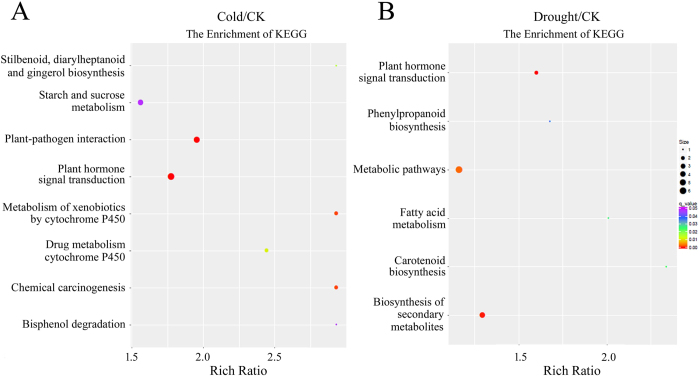
Statistics of KEGG pathway enrichment. (**A**,**B**) KEGG pathway classification of DE-lncRNA-correlated differentially expressed genes under cold (**A**) and drought stress (**B**), respectively. The y-axis corresponds to KEGG pathway with a q-value ≤ 0.05, and the x-axis shows the enrichment ratio between the number of DEGs and all unigenes enriched in a particular pathway. The color of the dot represents q value, and the size of the dot represents the number of DEGs mapped to the reference pathways.

To further understand the function of stress-responsive lncRNAs, we performed co-expression analysis to identify trans-regulatory network of lncRNAs. In total, there were 12651 and 11133 protein-coding genes that were co-expressed with cold and drought responsive lncRNAs, respectively (Supplemental Data S7). GO (Gene Ontology) analysis of the genes in lncRNA trans-regulatory network was performed to explore their functions. We found 45 GO terms that were significantly enriched (P < 0.05), and 6 of these terms were associated with both cold and drought stress. For example, the top 10 enriched terms included sequence-specific DNA binding transcription factor activity, monooxygenase activity, tetrapyrrole binding activity, protein kinase activity, and phosphotransferase activity (Supplemental Figure S2). We also analyze the statistical enrichment of differentially expressed lncRNA target genes in KEGG (Kyoto Encyclopedia of Genes and Genomes) pathway. Based on the results of significantly differentially expressed lncRNAs analysis, 8 and 6 pathways were found responsive to cold and drought stress, respectively (Fig. 8A,B, Supplemental Data S8). The most enriched pathways including plant hormone signal transduction, starch and sucrose metabolism, plant-pathogen interaction, biosynthesis of secondary metabolites and metabolic pathways. These results suggest that one of the principal roles of lncRNAs may be transcriptional regulation of gene expression, especially genes that involved in hormone signal transduction and secondary metabolic pathways. These findings provide a comprehensive view of cassava lncRNAs, which will enable in-depth functional analysis.

## Discussion

Understanding the mechanism of gene regulation will provide molecular basis for the resistance research of cassava, contributing to make cassava better adapt to cold and/or drought stress. Over the past decade, emerging evidence reflected that the existence of long non-coding RNAs revealed the complexity of eukaryotic genome expression^9^. Recently, with the advance of next-generation sequencing technology, many novel non-coding RNA transcripts have been found in different species^18^,^19^,^26^,^28^,^57^,^58^,^59^. However, although plants exhibit complicated physiological, biochemical and molecular responses to cold and/or drought stress, a genome-wide identification and characterization of known and novel lncRNAs involved in these responses is still lacking, especially in cassava. In the research, we systematically identified and analyzed cassava lncRNAs to find novel lncRNAs dynamically regulated by cold and/or drought stress with ssRNA-seq method. This is the first work to globally identify lncRNAs that respond to cold and/or drought stress in cassava. Based on strict screen criteria, we totally identified 682 high-confidence lncRNAs, of which 453 were lincRNAs and 229 were lncNATs. The number of lncRNAs was far less than that of lncRNAs identified in *Arabidopsis* or rice, which may be due to the rigorous filtration criteria we used to identify lncRNAs. These lncRNAs have a median length of 1899 nucleotides, and usually have 2–3 exons. By analyzing the expression patterns of lncRNAs, we found most lncRNAs were expressed at low levels compared with mRNAs. Our analysis generated a relatively robust list of potential lncRNAs for cassava, which will be useful for functional genomics research.

To understand the crosstalk and specificity of the responses of cassava to cold and drought stress, we performed the integrated analysis of the effects of the two stresses on the levels of lncRNA transcripts. Finally, a total of 318 cold and/or drought stress responsive lncRNAs, which were significantly up-regulated or down-regulated, were identified from RNA-seq data. We further identified 69 common differentially expressed lncRNAs, 198 cold-specific DE-lncRNAs and 51 drought-specific DE-lncRNAs, respectively. For example, lincRNA419 were specifically up-regulated under cold stress and unchanged under drought stress, whereas the lincRNA101 was specifically identified under drought stress. Unexpectedly, we also noticed that the drought responsive lncRNAs was less than the cold responsive ones. These differences in expression of lncRNAs indicated that different lncRNAs members executed different functions to cope with various stresses.

One important class of noncoding RNAs is lncRNAs that act as target mimics of miRNAs, which were first studied in plant^22^. Consequently, several studies found that lncRNAs function as putative miRNAs target mimics by using computational methods in plant^23^,^24^. In our study, we globally analyzed the regulatory network of miRNAs. Using bioinformatics, we found that 12 lncRNAs act as the precursor of miRNAs, and a portion of lncRNAs could not be directly cleaved by the miRNAs due to the existence of three nucleotide bulges at the 10th nucleotide positions of the miRNA-lncRNA pairing site. We have predicted 16 lncRNAs act as potential target mimics of conserved miRNAs in cassava. By analyzing the expression of lincRNAs candidates and miRNAs targets, two of these cold and/or drought stress-related lincRNAs (linRNA159 and 340) were predicted to be target mimics of miR164 and miR169, respectively. It has been reported that a decrease in miR164-targeted NAC genes cause drought tolerance in plant and that miR169-targeted NF-Y genes also plays a role in abiotic stress responses^47^,^48^. Considering the coordinate expression pattern of linRNAs and miR164/169 targets, it is intriguing to associate these two lincRNAs with the important functions of miR164/miR169 in regulating cold and/or drought stress responses.

The sensitive strand-specific RNA library construction protocol enabled us to identify many novel lncRNAs transcribed from antisense strand of protein-coding genes. Compared with previous genomic studies, our results represent more comprehensive findings to date and extend our knowledge of antisense transcripts in plants. Among a total of 229 lncNAT candidates, we found 153 cold and/or drought responsive lncNATs. This observation indicated these lncNATs may be very important to the gene regulation in response to cold and/or drought stress. Meanwhile, by comparing small RNAs associated with lncNAT pairs, we observed dozens of cold and/or drought responsive lncNAT pairs may serve as precursors of siRNAs (Table 1, Fig. 6, Supplemental Data S5), suggesting that these lncNATs may function through short siRNAs.

Emerging evidence revealed that lncRNAs could regulate various stages of gene expression in cis. Cis-acting lncRNAs were reported to control the expression of genes that are positioned in the vicinity of their transcription sites55. Further analyses provided evidence for the importance of lncRNA also act as long-distance regulatory elements. In maize, the conserved non-coding RNA *Vgt1 (Vegetative to generative transition1*), located 70 kb upstream of the *ZmRap2* gene and influenced the expression of *ZmRap2*^28^. In this study, we conducted a genome-wide investigation of the lincRNAs and protein-coding transcripts which adjacent to lincRNAs (<10 kb). We found that the expression of many lincRNA was correlated with their neighboring protein-coding genes, which showed differentially expression pattern under stress conditions. This phenomenon has also been proved by qRT-PCR. For example, lincRNA356 is up-regulated by drought treatment, while its neighboring genes (Manes.09G081500) was down-regulated, suggesting the potential for antagonistic expression of this lincRNA and the nearby coding gene. Our findings, together with previous reports in plant, provide a starting point for future investigation into the molecular mechanisms and regulatory functions of lncRNAs. Considering that more and more trans-acting lncRNAs have also been discovered, which can regulate gene expression at independent loci, we constructed a gene co-expression network of stress-responsive lncRNAs and coding genes. Based on the GO and KEGG enrichment analyses, we found that many genes encoded involved in hormone signal transduction and secondary metabolic pathways were differentially expressed under cold and/or drought stress. However, we noticed that a great number of DE-lncRNAs and correlated DEGs involved in the plant-pathogen interaction and starch and sucrose metabolism pathways were specifically enriched in the cold treatment, while lncRNAs-correlated DEGs belonged to metabolic pathways were specifically modulated by drought stress. These findings support the above viewpoint regarding the functional specialization of lncRNAs when encountering different stresses. Taken together, our results suggest that cassava implements divergent mechanisms to modulate the response to various environmental stimuli. The function of these lncRNAs should be further investigated.

In summary, our study provides a comprehensive resource for functional investigation of lncRNAs under stress conditions, and also complements the reference genome annotation of cassava, which might further aid the gene cloning and trigger more functional studies on lncRNAs in cassava. The discovery of lncRNAs has filled gaps in our knowledge of cold and/or drought regulatory pathways. Although increasing number of the lncRNAs was identified and characterized, their roles in regulation of targeted genes were far from being understood in many plant species. By predicting lncRNA targets, we provided a set of research clues concerning the potential roles of the lncRNAs related to the signaling regulatory network under cold and/or drought stress condition. Therefore, the lncRNA investigation may provide new insights into complicate regulations of abiotic responses and thus could be conducted for improvements in cassava yield.

## Materials and Methods

### Plant materials and stress treatment

Cassava (*Manihot esculenta*) cultivar (TMS60444) was used in this study. Their stems were cut into approximately 1.5 cm in length with one buds and planted in MS plate for two weeks in a greenhouse at 26 ± 2 °C, with a photoperiod of 16 h light and 8 h dark. Plants with a uniform growth status (about 5 cm height and with 2–3 leaves) were transferred to a chamber for cold treatment at 4 °C under light. The shoot apices and youngest expanded leaf were collected after 1 h, 6 h, 12 h, 24 h, 48 h and 72 h of treatment, and then were frozen in liquid nitrogen and held at −80 °C for RNA extraction. For drought treatment, seedlings were treated with 20% PEG6000 and harvested at 1 h, 3 h, 6 h, 12 h, 24 h and 48 h after treatment. Cassava plants used for RNA-seq sequencing were planted as above, but cold and drought stress treated only at 24 h and 6 h time point, respectively. In all cases, parallel and untreated plants at the same stage were used as controls. More than ten plants were harvested and pooled for each time point, and the collection was repeated three times as biological replicates.

### Whole transcriptome library construction and high-throughput sequencing

The total RNA isolation, whole transcriptome libraries preparation and deep sequencing were performed by the Annoroad Gene Technology Corporation (Beijing, PR China). Whole transcriptome libraries were constructed using TruSeq Stranded Total RNA with Ribo-Zero Gold (Illumina, San Diego, CA, USA) for rRNA depletion according to the manufacturer’s instructions. The total RNA-seq libraries were sequenced initially on a HiSeq 2500 instrument that generated paired-end reads of 125 nucleotides.

### Bioinformatics identification of cassava lncRNAs

Reads with more than 10% N (Unable to determine base information), with adapter sequence, or low quality were removed from the raw reads to obtain clean reads for further analysis. Cleaned reads were aligned to the cassava reference genome using Tophat 2.0 program with parameter “–library-type fr-firststrand”^60^. After the alignment, Cufflinks^37^ was employed to assemble reads into transcripts according to the instructions provided. The assembled transcripts detected in two or more samples were selected for further analysis. The number of fragments per kilobase per million mapped reads (FPKM) per gene was calculated^43^. Next, we discarded transcripts that overlapped with known protein-coding genes on the same strand, transcripts with FPKM scores <0.5 and transcripts shorter than 200 nt. We used the CPC^38^, CPAT^39^ and CNCI^40^ to filter transcripts with coding potential. The remaining transcripts with known protein domains were excluded by Pfam Scan according to Pfam HMM^41^,^42^. The transcripts that remained were considered reliably expressed lncRNAs. Signifiant differently expressed lncRNAs between cold or drought stresses and normal condition were extracted. The cassava mRNA, lincRNAs, lncNATs were aligned to the cassava genome separately to obtain the distribution of all these kinds of transcripts along chromosome with a summarized size of every 1 Mb.

### Quantitative real time PCR (qRT-PCR) validation of lncRNAs and genes

Total RNA were isolated respectively from cassava shoot apices and youngest expanded leaf after stress treatments for qRT-PCR using the RNAiso reagent (OMEGA). First-strand cDNA was reverse transcribed by PrimeScriptTM RT reagent Kit with gDNA Eraser (Takara). The qRT-PCR was performed using SYBR Premix Ex TaqTM (Takara). The *MeACTIN* was used as the reference gene and all the primers used were as listed in Supplemental Data S9. Quantification of lncRNA and gene expression was performed using the comparative Ct method, and the specificity of the amplified product was evaluated by melting curve. This experiment was performed three technical replicates and three biological replicates.

### Prediction of lncRNAs as miRNA target mimics

All lincRNAs and lncNATs candidates were used to predict miRNAs mimic target sites according to the standard of miR399 target mimic sites in *IPS1*. That is, the pairing is less than 4 mismatches between miRNA and lncRNA and is interrupted by a 3 nt mismatched loop at the expected miRNA cleavage site^22^,^24^. Mature cassava miRNAs were downloaded from miRBase database (http://www.mirbase.org/).

### Analysis of small RNAs derived from lncNATs

The 4 small RNAs dataset from samples under NC, CA, CCA and CS condition were downloaded from GEO under accession number GSE52178. The small RNAs were mapped to lncNATs using STAR program^61^. Abundance of small RNAs was normalized to reads per 6 million (RP6M). The length distribution of total and unique small RNAs from lncNATs and sense genes was plotted respectively, and the small RNAs distribution along given pairs of lncNATs and sense genes were visualized using customized script.

### Gene Ontology (GO) and Kyoto Encyclopedia of Genes and Genomes (KEGG) enrichment analysis

GO enrichment analysis of protein-coding genes correlated with lincRNAs was implemented by the GO seq R package^62^. KOBAS software was used for testing the statistical enrichment of differential expressed lncRNA target genes in KEGG pathways (http://www.genome.ad.jp/kegg/)^63^.

## Additional Information

**How to cite this article:** Li, S. *et al*. Genome-wide identification and functional prediction of cold and/or drought-responsive lncRNAs in cassava. *Sci. Rep.* **7**, 45981; doi: 10.1038/srep45981 (2017).

**Publisher's note:** Springer Nature remains neutral with regard to jurisdictional claims in published maps and institutional affiliations.

## Supplementary Material

Supplementary Information

Supplementary Figure

## Figures and Tables

**Table 1:** Differential expressed lncNATs acting as precursor of small RNAs.

lncNAT ID	siRNA reads				siRNA Fold Change			lncNAT Fold Change	
	CA	CCA	CS	NC	CCA/NC	CA/NC	CS/NC	Cold/CK	Drought/CK
lncNAT_229	15	146	13	12	11.23	1.15	1.00	2.07	1.62
lncNAT_76	558	318	120	44	7.07	12.40	2.67	47.34	6.09
lncNAT_43	258	794	181	141	5.59	1.82	1.27	2.53	1.18
lncNAT_222	129	219	67	63	3.42	2.02	1.05	2.23	0.25
lncNAT_36	58	215	68	88	2.42	0.65	0.76	0.28	0.40
lncNAT_95	35	58	34	24	2.32	1.40	1.36	0.22	0.59
lncNAT_110	33	54	35	26	2.00	1.22	1.30	55.30	2.22
lncNAT_149	56	75	29	41	1.79	1.33	0.69	0.45	0.70
lncNAT_94	268	382	226	267	1.43	1.00	0.84	0.48	1.11
lncNAT_179	701	1152	761	876	1.31	0.80	0.87	3.11	2.61
lncNAT_73	47	47	44	36	1.27	1.27	1.19	42.70	1.65
lncNAT_61	13	24	16	22	1.04	0.57	0.70	2.83	2.29
lncNAT_195	37	30	36	29	1.00	1.23	1.20	62.09	74.88
lncNAT_212	18	24	24	26	0.89	0.67	0.89	0.41	0.89
lncNAT_185	17	16	24	23	0.67	0.71	1.00	0.74	0.07
lncNAT_156	168	139	190	213	0.65	0.79	0.89	0.17	1.02
lncNAT_109	29	10	19	15	0.63	1.81	1.19	0.22	1.33
lncNAT_64	15	7	7	14	0.47	1.00	0.47	2.41	1.78
lncNAT_33	15	14	30	41	0.33	0.36	0.71	0.48	0.96
