# Corrigendum: Genome-wide identification and functional prediction of cold and/or drought-responsive lncRNAs in cassava

**DOI:** 10.1038/srep46795

**Published:** 2017-05-04

**Authors:** Shuxia Li, Xiang Yu, Ning Lei, Zhihao Cheng, Pingjuan Zhao, Yuke He, Wenquan Wang, Ming Peng

Scientific Reports 7: Article number: 4598110.1038/srep45981; published online: 04 07 2017; updated: 05 04 2017

The original version of this Article contained errors.

An affiliation for Xiang Yu was omitted. The correct affiliations are listed below:

National Key Laboratory of Plant Molecular Genetics and National Center for Plant Gene Research (Shanghai), Institute of Plant Physiology and Ecology, Shanghai Institutes for Biological Sciences, Chinese Academy of Sciences, Shanghai 200032, China.

Department of Biology, University of Pennsylvania, Philadelphia, PA 19104, USA.

In addition, the original version omitted Shuxia Li and Xiang Yu as equally contributing authors.

There was a typographical error in the Results section under subheading ‘Genome-wide identification of novel lncRNAs in cassava’,

“In addition, we aligned the 682 lncRNAs with GreeNC database, and found all these lncRNAs have not been annotated in cassava”.

now reads:

“In addition, we aligned the 682 lncRNAs with GreeNC database^26^, and found all these lncRNAs have not been annotated in cassava”.

In addition, there was a typographical error in the Discussion section,

“Cis-acting lncRNAs were reported to control the expression of genes that are positioned in the vicinity of their transcription sites55”.

now reads:

“Cis-acting lncRNAs were reported to control the expression of genes that are positioned in the vicinity of their transcription sites^55^”.

These errors have been corrected in both the HTML and PDF versions of the Article.

