# Supplementary material for: Genome-wide identification and functional prediction of cold and/or drought-responsive lncRNAs in cassava
Source: Sci Rep. 2017 Apr 7;7:45981. doi: 10.1038/srep45981 (PMC5384091; doi:10.1038/srep45981)
Supplement: Supplementary Information [file srep45981-s1.pdf]

Supplementary information

Genome-wide identification and functional prediction of cold and/or drought-responsive lncRNAs in cassava

Shuxia Li[1†], Xiang Yu[2†], Ning Lei[1], Zhihao Cheng[3], Pingjuan Zhao[1], Yuke He[2], Wenquan Wang[1]& Ming Peng[1*]

Supplemental Data S1. The information of 682 identified lncRNAs in this study

lncRNA ID	Chromosome N(	Exon start	Exon end	strand	Transcript ID	Exon Number
lincRNA_1	Scaffold00833	61860	62614	+	"TCONS_LINC_0000129(	exon_number "1"
lincRNA_1	Scaffold00833	65121	65192	+	"TCONS_LINC_0000129(	exon_number "2"
lincRNA_10	Chromosome10	17455266	17455667	-	"TCONS_LINC_0000072(	exon_number "1"
lincRNA_10	Chromosome10	17457713	17457795	-	"TCONS_LINC_0000072(	exon_number "2"
lincRNA_10	Chromosome10	17457960	17458830	-	"TCONS_LINC_0000072(	exon_number "3"
lincRNA_10	Chromosome10	17456507	17456982	-	"TCONS_LINC_0000072I	exon_number "1"
lincRNA_10	Chromosome10	17457073	17457201	-	"TCONS_LINC_0000072I	exon_number "2"
lincRNA_10	Chromosome10	17457292	17457442	-	"TCONS_LINC_0000072I	exon_number "3"
lincRNA_10	Chromosome10	17457713	17457795	-	"TCONS_LINC_0000072I	exon_number "4"
lincRNA_10	Chromosome10	17458036	17458109	-	"TCONS_LINC_0000072I	exon_number "5"
lincRNA_10	Chromosome10	17458908	17458971	-	"TCONS_LINC_0000072I	exon_number "6"
lincRNA_100	Chromosome15	2648079	2648223	-	"TCONS_LINC_0000104(	exon_number "1"
lincRNA_100	Chromosome15	2660414	2660490	-	"TCONS_LINC_0000104(	exon_number "2"
lincRNA_101	Chromosome15	275056	277228	+	"TCONS_LINC_0000101!	exon_number "1"
lincRNA_101	Chromosome15	280310	280888	+	"TCONS_LINC_0000101!	exon_number "2"
lincRNA_102	Chromosome15	3498537	3500112	-	"TCONS_LINC_0000104'	exon_number "1"
lincRNA_102	Chromosome15	3500328	3500460	-	"TCONS_LINC_0000104'	exon_number "2"
lincRNA_102	Chromosome15	3500593	3500663	-	"TCONS_LINC_0000104'	exon_number "3"
lincRNA_102	Chromosome15	3500775	3501557	-	"TCONS_LINC_0000104'	exon_number "4"
lincRNA_103	Chromosome15	3585329	3587760	-	"TCONS_LINC_0000104{	exon_number "1"
lincRNA_104	Chromosome15	38256	38689	+	"TCONS_LINC_0000101:	exon_number "1"
lincRNA_104	Chromosome15	39619	40344	+	"TCONS_LINC_0000101:	exon_number "2"
lincRNA_105	Chromosome15	4452059	4453000	-	"TCONS_LINC_0000105(	exon_number "1"
lincRNA_105	Chromosome15	4453260	4454245	-	"TCONS_LINC_0000105(	exon_number "2"
lincRNA_106	Chromosome15	5442135	5443058	-	"TCONS_LINC_0000105:	exon_number "1"
lincRNA_106	Chromosome15	5445285	5445537	-	"TCONS_LINC_0000105:	exon_number "2"
lincRNA_107	Chromosome15	5529132	5529800	+	"TCONS_LINC_0000101'	exon_number "1"
lincRNA_107	Chromosome15	5548970	5549206	+	"TCONS_LINC_0000101'	exon_number "2"
lincRNA_108	Chromosome15	581701	581854	-	"TCONS_LINC_0000104I	exon_number "1"
lincRNA_108	Chromosome15	594304	594418	-	"TCONS_LINC_0000104I	exon_number "2"
lincRNA_108	Chromosome15	595337	596288	-	"TCONS_LINC_0000104I	exon_number "3"
lincRNA_108	Chromosome15	596656	596728	-	"TCONS_LINC_0000104I	exon_number "4"
lincRNA_108	Chromosome15	593853	594418	-	"TCONS_LINC_0000104:	exon_number "1"
lincRNA_108	Chromosome15	595337	596457	-	"TCONS_LINC_0000104:	exon_number "2"
lincRNA_108	Chromosome15	596656	596814	-	"TCONS_LINC_0000104:	exon_number "3"
lincRNA_109	Chromosome15	6118621	6118958	+	"TCONS_LINC_0000101{	exon_number "1"
lincRNA_109	Chromosome15	6120808	6121814	+	"TCONS_LINC_0000101{	exon_number "2"
lincRNA_109	Chromosome15	6118625	6118958	+	"TCONS_LINC_0000101!	exon_number "1"
lincRNA_109	Chromosome15	6124893	6124959	+	"TCONS_LINC_0000101!	exon_number "2"
lincRNA_109	Chromosome15	6125117	6125215	+	"TCONS_LINC_0000101!	exon_number "3"
lincRNA_109	Chromosome15	6125308	6125465	+	"TCONS_LINC_0000101!	exon_number "4"
lincRNA_109	Chromosome15	6127038	6127109	+	"TCONS_LINC_0000101!	exon_number "5"
lincRNA_109	Chromosome15	6127235	6127295	+	"TCONS_LINC_0000101!	exon_number "6"
lincRNA_109	Chromosome15	6127401	6128186	+	"TCONS_LINC_0000101!	exon_number "7"
lincRNA_109	Chromosome15	6128445	6129620	+	"TCONS_LINC_0000101!	exon_number "8"
lincRNA_11	Chromosome10	18403881	18403891	+	"TCONS_LINC_0000067!	exon_number "1"
lincRNA_11	Chromosome10	18431428	18431875	+	"TCONS_LINC_0000067!	exon_number "2"
lincRNA_110	Chromosome15	7796474	7797013	+	"TCONS_LINC_0000102I	exon_number "1"
lincRNA_110	Chromosome15	7797158	7797241	+	"TCONS_LINC_0000102I	exon_number "2"
lincRNA_110	Chromosome15	7798327	7798385	+	"TCONS_LINC_0000102I	exon_number "3"
lincRNA_110	Chromosome15	7798690	7799826	+	"TCONS_LINC_0000102I	exon_number "4"
lincRNA_111	Chromosome15	9643756	9644527	+	"TCONS_LINC_0000102·	exon_number "1"
lincRNA_111	Chromosome15	9647657	9647978	+	"TCONS_LINC_0000102·	exon_number "2"
lincRNA_112	Chromosome12	10607945	10607991	+	"TCONS_LINC_0000080'	exon_number "1"
lincRNA_112	Chromosome12	10610807	10611835	+	"TCONS_LINC_0000080'	exon_number "2"

lincRNA_113	Chromosome12	14050830	14051361	+	"TCONS_LINC_00000811	exon_number "1"
lincRNA_113	Chromosome12	14054091	14054243	+	"TCONS_LINC_00000811	exon_number "2"
lincRNA_113	Chromosome12	14059686	14059709	+	"TCONS_LINC_00000811	exon_number "3"
lincRNA_113	Chromosome12	14050860	14051361	+	"TCONS_LINC_00000812	exon_number "1"
lincRNA_113	Chromosome12	14054091	14054243	+	"TCONS_LINC_00000812	exon_number "2"
lincRNA_113	Chromosome12	14055699	14055752	+	"TCONS_LINC_00000812	exon_number "3"
lincRNA_114	Chromosome12	14339998	14340005	-	"TCONS_LINC_00000835	exon_number "1"
lincRNA_114	Chromosome12	14354489	14355012	-	"TCONS_LINC_00000835	exon_number "2"
lincRNA_115	Chromosome12	15249153	15250397	-	"TCONS_LINC_00000836	exon_number "1"
lincRNA_115	Chromosome12	15251383	15251505	-	"TCONS_LINC_00000836	exon_number "2"
lincRNA_116	Chromosome12	18520189	18521017	-	"TCONS_LINC_00000839	exon_number "1"
lincRNA_116	Chromosome12	18522050	18522212	-	"TCONS_LINC_00000839	exon_number "2"
lincRNA_117	Chromosome12	19266328	19267228	+	"TCONS_LINC_00000815	exon_number "1"
lincRNA_117	Chromosome12	19279506	19279557	+	"TCONS_LINC_00000815	exon_number "2"
lincRNA_118	Chromosome12	19426138	19429461	+	"TCONS_LINC_00000818	exon_number "1"
lincRNA_118	Chromosome12	19432486	19432515	+	"TCONS_LINC_00000818	exon_number "2"
lincRNA_119	Chromosome12	21548623	21549785	+	"TCONS_LINC_00000820	exon_number "1"
lincRNA_119	Chromosome12	21553496	21554818	+	"TCONS_LINC_00000820	exon_number "2"
lincRNA_12	Chromosome10	18627406	18627620	-	"TCONS_LINC_00000723	exon_number "1"
lincRNA_12	Chromosome10	18686621	18686623	-	"TCONS_LINC_00000723	exon_number "2"
lincRNA_120	Chromosome12	21705909	21706208	+	"TCONS_LINC_00000822	exon_number "1"
lincRNA_120	Chromosome12	21707118	21707176	+	"TCONS_LINC_00000822	exon_number "2"
lincRNA_120	Chromosome12	21710496	21710553	+	"TCONS_LINC_00000822	exon_number "3"
lincRNA_120	Chromosome12	21710638	21710647	+	"TCONS_LINC_00000822	exon_number "4"
lincRNA_120	Chromosome12	21710393	21710459	+	"TCONS_LINC_00000823	exon_number "1"
lincRNA_120	Chromosome12	21710482	21710543	+	"TCONS_LINC_00000823	exon_number "2"
lincRNA_120	Chromosome12	21710628	21711110	+	"TCONS_LINC_00000823	exon_number "3"
lincRNA_120	Chromosome12	21711196	21711494	+	"TCONS_LINC_00000823	exon_number "4"
lincRNA_121	Chromosome12	27172631	27173132	+	"TCONS_LINC_00000826	exon_number "1"
lincRNA_121	Chromosome12	27175862	27176304	+	"TCONS_LINC_00000826	exon_number "2"
lincRNA_121	Chromosome12	27176395	27177399	+	"TCONS_LINC_00000826	exon_number "3"
lincRNA_122	Chromosome12	27372648	27372661	-	"TCONS_LINC_00000841	exon_number "1"
lincRNA_122	Chromosome12	27373516	27378975	-	"TCONS_LINC_00000841	exon_number "2"
lincRNA_123	Chromosome12	27727632	27728668	+	"TCONS_LINC_00000827	exon_number "1"
lincRNA_123	Chromosome12	27732776	27732782	+	"TCONS_LINC_00000827	exon_number "2"
lincRNA_124	Chromosome12	28598538	28598554	+	"TCONS_LINC_00000829	exon_number "1"
lincRNA_124	Chromosome12	28602677	28603348	+	"TCONS_LINC_00000829	exon_number "2"
lincRNA_125	Chromosome12	29407321	29407523	-	"TCONS_LINC_00000842	exon_number "1"
lincRNA_125	Chromosome12	29408390	29408514	-	"TCONS_LINC_00000842	exon_number "2"
lincRNA_125	Chromosome12	29409512	29409566	-	"TCONS_LINC_00000842	exon_number "3"
lincRNA_126	Chromosome12	30142431	30142461	-	"TCONS_LINC_00000843	exon_number "1"
lincRNA_126	Chromosome12	30143552	30145507	-	"TCONS_LINC_00000843	exon_number "2"
lincRNA_127	Chromosome12	3580588	3580900	+	"TCONS_LINC_00000798	exon_number "1"
lincRNA_127	Chromosome12	3583067	3583114	+	"TCONS_LINC_00000798	exon_number "2"
lincRNA_127	Chromosome12	3583207	3583433	+	"TCONS_LINC_00000798	exon_number "3"
lincRNA_127	Chromosome12	3582481	3583114	+	"TCONS_LINC_00000799	exon_number "1"
lincRNA_127	Chromosome12	3583207	3583356	+	"TCONS_LINC_00000799	exon_number "2"
lincRNA_127	Chromosome12	3583535	3583652	+	"TCONS_LINC_00000799	exon_number "3"
lincRNA_127	Chromosome12	3583798	3584204	+	"TCONS_LINC_00000799	exon_number "4"
lincRNA_128	Chromosome12	6450920	6450959	+	"TCONS_LINC_00000802	exon_number "1"
lincRNA_128	Chromosome12	6450981	6452986	+	"TCONS_LINC_00000802	exon_number "2"
lincRNA_129	Chromosome12	9238858	9238870	-	"TCONS_LINC_00000830	exon_number "1"
lincRNA_129	Chromosome12	9247430	9247955	-	"TCONS_LINC_00000830	exon_number "2"
lincRNA_13	Chromosome10	19349060	19349091	+	"TCONS_LINC_00000676	exon_number "1"
lincRNA_13	Chromosome10	19349255	19352309	+	"TCONS_LINC_00000676	exon_number "2"
lincRNA_130	Chromosome12	9266720	9267227	-	"TCONS_LINC_00000831	exon_number "1"
lincRNA_130	Chromosome12	9268812	9269119	-	"TCONS_LINC_00000831	exon_number "2"

lincRNA_130	Chromosome12	9266720	9267227	-	"TCONS_LINC_0000083[illegible]	exon_number "1"
lincRNA_130	Chromosome12	9274280	9274354	-	"TCONS_LINC_0000083[illegible]	exon_number "2"
lincRNA_130	Chromosome12	9274469	9277533	-	"TCONS_LINC_0000083[illegible]	exon_number "3"
lincRNA_131	Chromosome13	1045081	1045681	-	"TCONS_LINC_0000088[illegible]	exon_number "1"
lincRNA_131	Chromosome13	1046221	1046293	-	"TCONS_LINC_0000088[illegible]	exon_number "2"
lincRNA_131	Chromosome13	1047825	1047995	-	"TCONS_LINC_0000088[illegible]	exon_number "3"
lincRNA_131	Chromosome13	1049064	1049184	-	"TCONS_LINC_0000088[illegible]	exon_number "4"
lincRNA_131	Chromosome13	1049382	1049517	-	"TCONS_LINC_0000088[illegible]	exon_number "5"
lincRNA_131	Chromosome13	1049690	1049925	-	"TCONS_LINC_0000088[illegible]	exon_number "6"
lincRNA_131	Chromosome13	1046206	1046298	-	"TCONS_LINC_0000089[illegible]	exon_number "1"
lincRNA_131	Chromosome13	1047825	1047995	-	"TCONS_LINC_0000089[illegible]	exon_number "2"
lincRNA_131	Chromosome13	1049064	1049184	-	"TCONS_LINC_0000089[illegible]	exon_number "3"
lincRNA_131	Chromosome13	1049382	1049941	-	"TCONS_LINC_0000089[illegible]	exon_number "4"
lincRNA_132	Chromosome13	10690525	10690545	-	"TCONS_LINC_0000090[illegible]	exon_number "1"
lincRNA_132	Chromosome13	10751656	10752133	-	"TCONS_LINC_0000090[illegible]	exon_number "2"
lincRNA_132	Chromosome13	10761498	10761672	-	"TCONS_LINC_0000090[illegible]	exon_number "3"
lincRNA_133	Chromosome13	10944515	10946613	-	"TCONS_LINC_0000090[illegible]	exon_number "1"
lincRNA_133	Chromosome13	10950165	10951422	-	"TCONS_LINC_0000090[illegible]	exon_number "2"
lincRNA_134	Chromosome13	11220046	11220299	+	"TCONS_LINC_0000086[illegible]	exon_number "1"
lincRNA_134	Chromosome13	11223479	11223778	+	"TCONS_LINC_0000086[illegible]	exon_number "2"
lincRNA_135	Chromosome13	11269886	11269986	+	"TCONS_LINC_0000087[illegible]	exon_number "1"
lincRNA_135	Chromosome13	11273533	11274254	+	"TCONS_LINC_0000087[illegible]	exon_number "2"
lincRNA_136	Chromosome13	13515892	13516775	+	"TCONS_LINC_0000087[illegible]	exon_number "1"
lincRNA_136	Chromosome13	13519164	13519203	+	"TCONS_LINC_0000087[illegible]	exon_number "2"
lincRNA_137	Chromosome13	13712676	13712700	-	"TCONS_LINC_0000090[illegible]	exon_number "1"
lincRNA_137	Chromosome13	13713454	13714897	-	"TCONS_LINC_0000090[illegible]	exon_number "2"
lincRNA_137	Chromosome13	13715519	13715547	-	"TCONS_LINC_0000090[illegible]	exon_number "3"
lincRNA_138	Chromosome13	1439343	1440089	+	"TCONS_LINC_0000084[illegible]	exon_number "1"
lincRNA_138	Chromosome13	1449636	1449826	+	"TCONS_LINC_0000084[illegible]	exon_number "2"
lincRNA_139	Chromosome13	15279612	15279654	+	"TCONS_LINC_0000088[illegible]	exon_number "1"
lincRNA_139	Chromosome13	15283690	15284094	+	"TCONS_LINC_0000088[illegible]	exon_number "2"
lincRNA_14	Chromosome10	19763805	19763828	+	"TCONS_LINC_0000067[illegible]	exon_number "1"
lincRNA_14	Chromosome10	19763952	19764002	+	"TCONS_LINC_0000067[illegible]	exon_number "2"
lincRNA_14	Chromosome10	19764808	19764928	+	"TCONS_LINC_0000067[illegible]	exon_number "3"
lincRNA_14	Chromosome10	19765039	19765101	+	"TCONS_LINC_0000067[illegible]	exon_number "4"
lincRNA_14	Chromosome10	19765261	19765332	+	"TCONS_LINC_0000067[illegible]	exon_number "5"
lincRNA_14	Chromosome10	19766272	19766358	+	"TCONS_LINC_0000067[illegible]	exon_number "6"
lincRNA_14	Chromosome10	19767464	19767763	+	"TCONS_LINC_0000067[illegible]	exon_number "7"
lincRNA_14	Chromosome10	19767891	19767941	+	"TCONS_LINC_0000067[illegible]	exon_number "8"
lincRNA_14	Chromosome10	19768041	19768538	+	"TCONS_LINC_0000067[illegible]	exon_number "9"
lincRNA_140	Chromosome13	15583085	15583512	-	"TCONS_LINC_0000090[illegible]	exon_number "1"
lincRNA_140	Chromosome13	15583698	15585008	-	"TCONS_LINC_0000090[illegible]	exon_number "2"
lincRNA_140	Chromosome13	15585190	15585514	-	"TCONS_LINC_0000090[illegible]	exon_number "3"
lincRNA_141	Chromosome13	16163551	16164521	-	"TCONS_LINC_0000090[illegible]	exon_number "1"
lincRNA_141	Chromosome13	16166327	16166949	-	"TCONS_LINC_0000090[illegible]	exon_number "2"
lincRNA_141	Chromosome13	16163551	16164291	-	"TCONS_LINC_0000091[illegible]	exon_number "1"
lincRNA_141	Chromosome13	16166969	16168076	-	"TCONS_LINC_0000091[illegible]	exon_number "2"
lincRNA_141	Chromosome13	16168159	16168356	-	"TCONS_LINC_0000091[illegible]	exon_number "3"
lincRNA_142	Chromosome13	22865361	22865643	+	"TCONS_LINC_0000088[illegible]	exon_number "1"
lincRNA_142	Chromosome13	22865701	22865829	+	"TCONS_LINC_0000088[illegible]	exon_number "2"
lincRNA_142	Chromosome13	22867274	22867912	+	"TCONS_LINC_0000088[illegible]	exon_number "3"
lincRNA_143	Chromosome13	24246954	24247540	+	"TCONS_LINC_0000088[illegible]	exon_number "1"
lincRNA_143	Chromosome13	24251567	24251990	+	"TCONS_LINC_0000088[illegible]	exon_number "2"
lincRNA_144	Chromosome13	24319702	24319755	-	"TCONS_LINC_0000091[illegible]	exon_number "1"
lincRNA_144	Chromosome13	24319792	24320261	-	"TCONS_LINC_0000091[illegible]	exon_number "2"
lincRNA_144	Chromosome13	24328077	24328086	-	"TCONS_LINC_0000091[illegible]	exon_number "3"
lincRNA_145	Chromosome13	25646111	25647436	-	"TCONS_LINC_0000092[illegible]	exon_number "1"

lincRNA_145	Chromosome13	25647564	25648048	-	"TCONS_LINC_00000921	exon_number "2"
lincRNA_145	Chromosome13	25648252	25648289	-	"TCONS_LINC_00000921	exon_number "3"
lincRNA_146	Chromosome13	25755879	25756489	-	"TCONS_LINC_00000922	exon_number "1"
lincRNA_146	Chromosome13	25756849	25757251	-	"TCONS_LINC_00000922	exon_number "2"
lincRNA_146	Chromosome13	25757349	25757542	-	"TCONS_LINC_00000922	exon_number "3"
lincRNA_146	Chromosome13	25758020	25758030	-	"TCONS_LINC_00000922	exon_number "4"
lincRNA_147	Chromosome13	26981862	26982290	-	"TCONS_LINC_00000923	exon_number "1"
lincRNA_147	Chromosome13	26983860	26985007	-	"TCONS_LINC_00000923	exon_number "2"
lincRNA_148	Chromosome13	27275240	27275717	+	"TCONS_LINC_00000885	exon_number "1"
lincRNA_148	Chromosome13	27275818	27275905	+	"TCONS_LINC_00000885	exon_number "2"
lincRNA_148	Chromosome13	27286284	27286324	+	"TCONS_LINC_00000885	exon_number "3"
lincRNA_148	Chromosome13	27275615	27275717	+	"TCONS_LINC_00000886	exon_number "1"
lincRNA_148	Chromosome13	27275818	27276513	+	"TCONS_LINC_00000886	exon_number "2"
lincRNA_149	Chromosome13	3235896	3236328	-	"TCONS_LINC_00000894	exon_number "1"
lincRNA_149	Chromosome13	3238346	3238373	-	"TCONS_LINC_00000894	exon_number "2"
lincRNA_15	Chromosome10	20661891	20662371	+	"TCONS_LINC_00000682	exon_number "1"
lincRNA_15	Chromosome10	20663208	20663340	+	"TCONS_LINC_00000682	exon_number "2"
lincRNA_15	Chromosome10	20663759	20664088	+	"TCONS_LINC_00000682	exon_number "3"
lincRNA_150	Chromosome13	3865996	3866352	-	"TCONS_LINC_00000896	exon_number "1"
lincRNA_150	Chromosome13	3873741	3876348	-	"TCONS_LINC_00000896	exon_number "2"
lincRNA_150	Chromosome13	3866550	3866579	-	"TCONS_LINC_00000897	exon_number "1"
lincRNA_150	Chromosome13	3873855	3875481	-	"TCONS_LINC_00000897	exon_number "2"
lincRNA_151	Chromosome13	6054517	6055665	-	"TCONS_LINC_00000898	exon_number "1"
lincRNA_151	Chromosome13	6055760	6068557	-	"TCONS_LINC_00000898	exon_number "2"
lincRNA_152	Chromosome13	6120570	6120858	-	"TCONS_LINC_00000899	exon_number "1"
lincRNA_152	Chromosome13	6128203	6128224	-	"TCONS_LINC_00000899	exon_number "2"
lincRNA_153	Chromosome13	8001072	8001113	-	"TCONS_LINC_00000902	exon_number "1"
lincRNA_153	Chromosome13	8004286	8004579	-	"TCONS_LINC_00000902	exon_number "2"
lincRNA_154	Chromosome13	949389	950137	+	"TCONS_LINC_00000845	exon_number "1"
lincRNA_154	Chromosome13	950249	951422	+	"TCONS_LINC_00000845	exon_number "2"
lincRNA_155	Scaffold01331	44	371	-	"TCONS_LINC_00001330	exon_number "1"
lincRNA_155	Scaffold01331	21166	28454	-	"TCONS_LINC_00001330	exon_number "2"
lincRNA_155	Scaffold01331	16717	16723	-	"TCONS_LINC_00001331	exon_number "1"
lincRNA_155	Scaffold01331	18067	28456	-	"TCONS_LINC_00001331	exon_number "2"
lincRNA_156	Chromosome11	10428200	10428797	-	"TCONS_LINC_00000774	exon_number "1"
lincRNA_156	Chromosome11	10430667	10430683	-	"TCONS_LINC_00000774	exon_number "2"
lincRNA_157	Chromosome11	10607066	10608222	+	"TCONS_LINC_00000740	exon_number "1"
lincRNA_157	Chromosome11	10644957	10645016	+	"TCONS_LINC_00000740	exon_number "2"
lincRNA_158	Chromosome11	12540887	12541288	+	"TCONS_LINC_00000744	exon_number "1"
lincRNA_158	Chromosome11	12541832	12542166	+	"TCONS_LINC_00000744	exon_number "2"
lincRNA_158	Chromosome11	12542493	12542981	+	"TCONS_LINC_00000744	exon_number "3"
lincRNA_159	Chromosome11	15638407	15642882	-	"TCONS_LINC_00000780	exon_number "1"
lincRNA_16	Chromosome10	21546988	21546999	-	"TCONS_LINC_00000726	exon_number "1"
lincRNA_16	Chromosome10	21574456	21576638	-	"TCONS_LINC_00000726	exon_number "2"
lincRNA_160	Chromosome11	1625419	1625679	+	"TCONS_LINC_00000735	exon_number "1"
lincRNA_160	Chromosome11	1627543	1629140	+	"TCONS_LINC_00000735	exon_number "2"
lincRNA_161	Chromosome11	17803778	17804379	-	"TCONS_LINC_00000784	exon_number "1"
lincRNA_161	Chromosome11	17815255	17815344	-	"TCONS_LINC_00000784	exon_number "2"
lincRNA_161	Chromosome11	17818139	17818594	-	"TCONS_LINC_00000784	exon_number "3"
lincRNA_162	Chromosome11	17889931	17890412	+	"TCONS_LINC_00000748	exon_number "1"
lincRNA_162	Chromosome11	17897814	17898075	+	"TCONS_LINC_00000748	exon_number "2"
lincRNA_163	Chromosome11	19317343	19318020	-	"TCONS_LINC_00000790	exon_number "1"
lincRNA_163	Chromosome11	19324249	19324332	-	"TCONS_LINC_00000790	exon_number "2"
lincRNA_163	Chromosome11	19324403	19324532	-	"TCONS_LINC_00000790	exon_number "3"
lincRNA_163	Chromosome11	19325543	19325640	-	"TCONS_LINC_00000790	exon_number "4"
lincRNA_163	Chromosome11	19319441	19319482	-	"TCONS_LINC_00000791	exon_number "1"
lincRNA_163	Chromosome11	19324052	19325692	-	"TCONS_LINC_00000791	exon_number "2"

lincRNA_164	Chromosome11	20129516	20129548	+	"TCONS_LINC_00000749	exon_number "1"
lincRNA_164	Chromosome11	20135306	20135583	+	"TCONS_LINC_00000749	exon_number "2"
lincRNA_165	Chromosome11	21170740	21172212	-	"TCONS_LINC_00000792	exon_number "1"
lincRNA_165	Chromosome11	21186138	21186370	-	"TCONS_LINC_00000792	exon_number "2"
lincRNA_166	Chromosome11	24942228	24942365	+	"TCONS_LINC_00000754	exon_number "1"
lincRNA_166	Chromosome11	24942463	24944575	+	"TCONS_LINC_00000754	exon_number "2"
lincRNA_166	Chromosome11	24944684	24945391	+	"TCONS_LINC_00000754	exon_number "3"
lincRNA_166	Chromosome11	24945882	24946183	+	"TCONS_LINC_00000754	exon_number "4"
lincRNA_166	Chromosome11	24943385	24944575	+	"TCONS_LINC_00000755	exon_number "1"
lincRNA_166	Chromosome11	24944663	24946511	+	"TCONS_LINC_00000755	exon_number "2"
lincRNA_166	Chromosome11	24946673	24947132	+	"TCONS_LINC_00000755	exon_number "3"
lincRNA_167	Chromosome11	26398432	26399353	+	"TCONS_LINC_00000757	exon_number "1"
lincRNA_167	Chromosome11	26403813	26404565	+	"TCONS_LINC_00000757	exon_number "2"
lincRNA_167	Chromosome11	26399274	26400518	+	"TCONS_LINC_00000758	exon_number "1"
lincRNA_167	Chromosome11	26405145	26405937	+	"TCONS_LINC_00000758	exon_number "2"
lincRNA_167	Chromosome11	26401700	26404565	+	"TCONS_LINC_00000759	exon_number "1"
lincRNA_168	Chromosome11	6559595	6560049	-	"TCONS_LINC_00000767	exon_number "1"
lincRNA_168	Chromosome11	6566863	6568504	-	"TCONS_LINC_00000767	exon_number "2"
lincRNA_168	Chromosome11	6568968	6570017	-	"TCONS_LINC_00000767	exon_number "3"
lincRNA_168	Chromosome11	6559595	6559951	-	"TCONS_LINC_00000768	exon_number "1"
lincRNA_168	Chromosome11	6566863	6566951	-	"TCONS_LINC_00000768	exon_number "2"
lincRNA_168	Chromosome11	6569512	6569851	-	"TCONS_LINC_00000768	exon_number "3"
lincRNA_168	Chromosome11	6570513	6570576	-	"TCONS_LINC_00000768	exon_number "4"
lincRNA_168	Chromosome11	6570659	6570679	-	"TCONS_LINC_00000768	exon_number "5"
lincRNA_168	Chromosome11	6565954	6569851	-	"TCONS_LINC_00000769	exon_number "1"
lincRNA_168	Chromosome11	6570226	6570476	-	"TCONS_LINC_00000769	exon_number "2"
lincRNA_168	Chromosome11	6565954	6569851	-	"TCONS_LINC_00000770	exon_number "1"
lincRNA_168	Chromosome11	6570513	6570729	-	"TCONS_LINC_00000770	exon_number "2"
lincRNA_169	Chromosome11	8222978	8223021	+	"TCONS_LINC_00000739	exon_number "1"
lincRNA_169	Chromosome11	8229211	8229990	+	"TCONS_LINC_00000739	exon_number "2"
lincRNA_17	Chromosome10	21650521	21651122	-	"TCONS_LINC_00000728	exon_number "1"
lincRNA_17	Chromosome10	21652498	21652528	-	"TCONS_LINC_00000728	exon_number "2"
lincRNA_170	Chromosome11	9760591	9760597	-	"TCONS_LINC_00000773	exon_number "1"
lincRNA_170	Chromosome11	9763086	9763359	-	"TCONS_LINC_00000773	exon_number "2"
lincRNA_171	Chromosome14	10728419	10728470	-	"TCONS_LINC_00000985	exon_number "1"
lincRNA_171	Chromosome14	10729221	10729286	-	"TCONS_LINC_00000985	exon_number "2"
lincRNA_171	Chromosome14	10729652	10730000	-	"TCONS_LINC_00000985	exon_number "3"
lincRNA_171	Chromosome14	10728746	10729286	-	"TCONS_LINC_00000986	exon_number "1"
lincRNA_171	Chromosome14	10729652	10729835	-	"TCONS_LINC_00000986	exon_number "2"
lincRNA_171	Chromosome14	10730816	10730865	-	"TCONS_LINC_00000986	exon_number "3"
lincRNA_172	Chromosome14	11986453	11991568	-	"TCONS_LINC_00000990	exon_number "1"
lincRNA_172	Chromosome14	11991681	11992422	-	"TCONS_LINC_00000990	exon_number "2"
lincRNA_172	Chromosome14	11992448	11993528	-	"TCONS_LINC_00000990	exon_number "3"
lincRNA_173	Chromosome14	12572034	12573059	+	"TCONS_LINC_00000951	exon_number "1"
lincRNA_173	Chromosome14	12573936	12575320	+	"TCONS_LINC_00000951	exon_number "2"
lincRNA_173	Chromosome14	12575352	12575621	+	"TCONS_LINC_00000951	exon_number "3"
lincRNA_174	Chromosome14	12844755	12845002	+	"TCONS_LINC_00000953	exon_number "1"
lincRNA_174	Chromosome14	12846376	12846443	+	"TCONS_LINC_00000953	exon_number "2"
lincRNA_174	Chromosome14	12850376	12850869	+	"TCONS_LINC_00000953	exon_number "3"
lincRNA_174	Chromosome14	12850943	12851069	+	"TCONS_LINC_00000953	exon_number "4"
lincRNA_174	Chromosome14	12851155	12851448	+	"TCONS_LINC_00000953	exon_number "5"
lincRNA_174	Chromosome14	12851538	12852194	+	"TCONS_LINC_00000953	exon_number "6"
lincRNA_174	Chromosome14	12850209	12850285	+	"TCONS_LINC_00000954	exon_number "1"
lincRNA_174	Chromosome14	12850376	12850869	+	"TCONS_LINC_00000954	exon_number "2"
lincRNA_174	Chromosome14	12850943	12851069	+	"TCONS_LINC_00000954	exon_number "3"
lincRNA_174	Chromosome14	12851155	12851448	+	"TCONS_LINC_00000954	exon_number "4"
lincRNA_174	Chromosome14	12851538	12852194	+	"TCONS_LINC_00000954	exon_number "5"

lincRNA_175	Chromosome14	13155182	13156326	-	"TCONS_LINC_00000991	exon_number "1"
lincRNA_175	Chromosome14	13160875	13161893	-	"TCONS_LINC_00000991	exon_number "2"
lincRNA_176	Chromosome14	13479464	13479882	-	"TCONS_LINC_00000995	exon_number "1"
lincRNA_176	Chromosome14	13480881	13481703	-	"TCONS_LINC_00000995	exon_number "2"
lincRNA_177	Chromosome14	14770498	14772664	+	"TCONS_LINC_00000955	exon_number "1"
lincRNA_177	Chromosome14	14772727	14772954	+	"TCONS_LINC_00000955	exon_number "2"
lincRNA_177	Chromosome14	14780266	14780897	+	"TCONS_LINC_00000955	exon_number "3"
lincRNA_178	Chromosome14	14848764	14849507	+	"TCONS_LINC_00000956	exon_number "1"
lincRNA_178	Chromosome14	14850783	14851294	+	"TCONS_LINC_00000956	exon_number "2"
lincRNA_178	Chromosome14	14848764	14849507	+	"TCONS_LINC_00000957	exon_number "1"
lincRNA_178	Chromosome14	14854832	14854901	+	"TCONS_LINC_00000957	exon_number "2"
lincRNA_178	Chromosome14	14855327	14855693	+	"TCONS_LINC_00000957	exon_number "3"
lincRNA_179	Chromosome14	15622732	15623415	-	"TCONS_LINC_00000997	exon_number "1"
lincRNA_179	Chromosome14	15624238	15624395	-	"TCONS_LINC_00000997	exon_number "2"
lincRNA_179	Chromosome14	15624584	15624924	-	"TCONS_LINC_00000997	exon_number "3"
lincRNA_18	Chromosome10	21679028	21679349	-	"TCONS_LINC_00000729	exon_number "1"
lincRNA_18	Chromosome10	21684892	21684908	-	"TCONS_LINC_00000729	exon_number "2"
lincRNA_180	Chromosome14	20156581	20156633	+	"TCONS_LINC_00000961	exon_number "1"
lincRNA_180	Chromosome14	20165761	20165926	+	"TCONS_LINC_00000961	exon_number "2"
lincRNA_180	Chromosome14	20166286	20166820	+	"TCONS_LINC_00000961	exon_number "3"
lincRNA_180	Chromosome14	20166128	20166517	+	"TCONS_LINC_00000963	exon_number "1"
lincRNA_180	Chromosome14	20167093	20167188	+	"TCONS_LINC_00000963	exon_number "2"
lincRNA_180	Chromosome14	20167973	20172542	+	"TCONS_LINC_00000963	exon_number "3"
lincRNA_181	Chromosome14	20237460	20239628	-	"TCONS_LINC_00001003	exon_number "1"
lincRNA_181	Chromosome14	20237460	20239424	-	"TCONS_LINC_00001004	exon_number "1"
lincRNA_181	Chromosome14	20240300	20240837	-	"TCONS_LINC_00001004	exon_number "2"
lincRNA_181	Chromosome14	20241286	20241357	-	"TCONS_LINC_00001004	exon_number "3"
lincRNA_181	Chromosome14	20243170	20243577	-	"TCONS_LINC_00001004	exon_number "4"
lincRNA_181	Chromosome14	20242734	20243260	-	"TCONS_LINC_00001005	exon_number "1"
lincRNA_181	Chromosome14	20243369	20243496	-	"TCONS_LINC_00001005	exon_number "2"
lincRNA_181	Chromosome14	20243583	20243918	-	"TCONS_LINC_00001005	exon_number "3"
lincRNA_182	Chromosome14	21217248	21217606	-	"TCONS_LINC_00001006	exon_number "1"
lincRNA_182	Chromosome14	21219381	21220090	-	"TCONS_LINC_00001006	exon_number "2"
lincRNA_183	Chromosome14	22382789	22383163	-	"TCONS_LINC_00001008	exon_number "1"
lincRNA_183	Chromosome14	22384012	22384186	-	"TCONS_LINC_00001008	exon_number "2"
lincRNA_183	Chromosome14	22386019	22386220	-	"TCONS_LINC_00001008	exon_number "3"
lincRNA_184	Chromosome14	22658378	22660072	+	"TCONS_LINC_00000965	exon_number "1"
lincRNA_184	Chromosome14	22669583	22669725	+	"TCONS_LINC_00000965	exon_number "2"
lincRNA_185	Chromosome14	22810245	22810289	-	"TCONS_LINC_00001011	exon_number "1"
lincRNA_185	Chromosome14	22833012	22834284	-	"TCONS_LINC_00001011	exon_number "2"
lincRNA_186	Chromosome14	3214497	3214611	-	"TCONS_LINC_00000972	exon_number "1"
lincRNA_186	Chromosome14	3215893	3215955	-	"TCONS_LINC_00000972	exon_number "2"
lincRNA_186	Chromosome14	3216070	3216200	-	"TCONS_LINC_00000972	exon_number "3"
lincRNA_186	Chromosome14	3224662	3224740	-	"TCONS_LINC_00000972	exon_number "4"
lincRNA_186	Chromosome14	3224921	3225017	-	"TCONS_LINC_00000972	exon_number "5"
lincRNA_186	Chromosome14	3225362	3225461	-	"TCONS_LINC_00000972	exon_number "6"
lincRNA_186	Chromosome14	3225638	3225892	-	"TCONS_LINC_00000972	exon_number "7"
lincRNA_186	Chromosome14	3214655	3215764	-	"TCONS_LINC_00000973	exon_number "1"
lincRNA_186	Chromosome14	3215893	3215955	-	"TCONS_LINC_00000973	exon_number "2"
lincRNA_186	Chromosome14	3216070	3216200	-	"TCONS_LINC_00000973	exon_number "3"
lincRNA_186	Chromosome14	3224662	3224740	-	"TCONS_LINC_00000973	exon_number "4"
lincRNA_186	Chromosome14	3224921	3225017	-	"TCONS_LINC_00000973	exon_number "5"
lincRNA_186	Chromosome14	3225362	3225461	-	"TCONS_LINC_00000973	exon_number "6"
lincRNA_186	Chromosome14	3225638	3225783	-	"TCONS_LINC_00000973	exon_number "7"
lincRNA_186	Chromosome14	3222625	3224751	-	"TCONS_LINC_00000975	exon_number "1"
lincRNA_186	Chromosome14	3224921	3225018	-	"TCONS_LINC_00000975	exon_number "2"
lincRNA_186	Chromosome14	3225363	3225461	-	"TCONS_LINC_00000975	exon_number "3"

lincRNA_186	Chromosome14	3225638	3225691	-	"TCONS_LINC_0000097[illegible]	exon_number "4"
lincRNA_187	Chromosome14	5592159	5593671	-	"TCONS_LINC_0000097[illegible]	exon_number "1"
lincRNA_187	Chromosome14	5593865	5594684	-	"TCONS_LINC_0000097[illegible]	exon_number "2"
lincRNA_188	Chromosome14	5868514	5868899	+	"TCONS_LINC_0000093[illegible]	exon_number "1"
lincRNA_188	Chromosome14	5868976	5869290	+	"TCONS_LINC_0000093[illegible]	exon_number "2"
lincRNA_188	Chromosome14	5870155	5870350	+	"TCONS_LINC_0000093[illegible]	exon_number "3"
lincRNA_188	Chromosome14	5870669	5870717	+	"TCONS_LINC_0000093[illegible]	exon_number "4"
lincRNA_188	Chromosome14	5870843	5870958	+	"TCONS_LINC_0000093[illegible]	exon_number "5"
lincRNA_188	Chromosome14	5871053	5871879	+	"TCONS_LINC_0000093[illegible]	exon_number "6"
lincRNA_188	Chromosome14	5869302	5870717	+	"TCONS_LINC_0000093[illegible]	exon_number "1"
lincRNA_188	Chromosome14	5870843	5870958	+	"TCONS_LINC_0000093[illegible]	exon_number "2"
lincRNA_188	Chromosome14	5871053	5871879	+	"TCONS_LINC_0000093[illegible]	exon_number "3"
lincRNA_189	Chromosome14	6116199	6116225	-	"TCONS_LINC_0000098[illegible]	exon_number "1"
lincRNA_189	Chromosome14	6124692	6124771	-	"TCONS_LINC_0000098[illegible]	exon_number "2"
lincRNA_189	Chromosome14	6124946	6126198	-	"TCONS_LINC_0000098[illegible]	exon_number "3"
lincRNA_19	Chromosome10	22671258	22671467	+	"TCONS_LINC_0000068[illegible]	exon_number "1"
lincRNA_19	Chromosome10	22671503	22671610	+	"TCONS_LINC_0000068[illegible]	exon_number "2"
lincRNA_19	Chromosome10	22684418	22684501	+	"TCONS_LINC_0000068[illegible]	exon_number "3"
lincRNA_190	Chromosome14	6209003	6209504	+	"TCONS_LINC_0000093[illegible]	exon_number "1"
lincRNA_190	Chromosome14	6212965	6214955	+	"TCONS_LINC_0000093[illegible]	exon_number "2"
lincRNA_190	Chromosome14	6209096	6209504	+	"TCONS_LINC_0000093[illegible]	exon_number "1"
lincRNA_190	Chromosome14	6209617	6209723	+	"TCONS_LINC_0000093[illegible]	exon_number "2"
lincRNA_190	Chromosome14	6212965	6214955	+	"TCONS_LINC_0000093[illegible]	exon_number "3"
lincRNA_191	Chromosome14	6837615	6837621	+	"TCONS_LINC_0000094[illegible]	exon_number "1"
lincRNA_191	Chromosome14	6839035	6839078	+	"TCONS_LINC_0000094[illegible]	exon_number "2"
lincRNA_191	Chromosome14	6839426	6840098	+	"TCONS_LINC_0000094[illegible]	exon_number "3"
lincRNA_191	Chromosome14	6838489	6839350	+	"TCONS_LINC_0000094[illegible]	exon_number "1"
lincRNA_192	Chromosome18	133332	133421	+	"TCONS_LINC_0001120[illegible]	exon_number "1"
lincRNA_192	Chromosome18	135888	136278	+	"TCONS_LINC_0001120[illegible]	exon_number "2"
lincRNA_193	Chromosome18	1590561	1593849	-	"TCONS_LINC_0001123[illegible]	exon_number "1"
lincRNA_194	Chromosome18	16678138	16678378	+	"TCONS_LINC_0001122[illegible]	exon_number "1"
lincRNA_194	Chromosome18	16693524	16693540	+	"TCONS_LINC_0001122[illegible]	exon_number "2"
lincRNA_195	Chromosome18	1692151	1692766	+	"TCONS_LINC_0001120[illegible]	exon_number "1"
lincRNA_195	Chromosome18	1693084	1693161	+	"TCONS_LINC_0001120[illegible]	exon_number "2"
lincRNA_195	Chromosome18	1694769	1694903	+	"TCONS_LINC_0001120[illegible]	exon_number "3"
lincRNA_195	Chromosome18	1695008	1695119	+	"TCONS_LINC_0001120[illegible]	exon_number "4"
lincRNA_195	Chromosome18	1695250	1695848	+	"TCONS_LINC_0001120[illegible]	exon_number "5"
lincRNA_195	Chromosome18	1692653	1692766	+	"TCONS_LINC_0001121[illegible]	exon_number "1"
lincRNA_195	Chromosome18	1693084	1694081	+	"TCONS_LINC_0001121[illegible]	exon_number "2"
lincRNA_195	Chromosome18	1694769	1694903	+	"TCONS_LINC_0001121[illegible]	exon_number "3"
lincRNA_195	Chromosome18	1695008	1695119	+	"TCONS_LINC_0001121[illegible]	exon_number "4"
lincRNA_195	Chromosome18	1695250	1695848	+	"TCONS_LINC_0001121[illegible]	exon_number "5"
lincRNA_196	Chromosome18	17543138	17543159	-	"TCONS_LINC_0001124[illegible]	exon_number "1"
lincRNA_196	Chromosome18	17546033	17546340	-	"TCONS_LINC_0001124[illegible]	exon_number "2"
lincRNA_197	Chromosome18	17637169	17637329	-	"TCONS_LINC_0001124[illegible]	exon_number "1"
lincRNA_197	Chromosome18	17658914	17659602	-	"TCONS_LINC_0001124[illegible]	exon_number "2"
lincRNA_198	Chromosome18	23153483	23154224	+	"TCONS_LINC_0001122[illegible]	exon_number "1"
lincRNA_198	Chromosome18	23163207	23163218	+	"TCONS_LINC_0001122[illegible]	exon_number "2"
lincRNA_199	Chromosome18	24253125	24253273	+	"TCONS_LINC_0001123[illegible]	exon_number "1"
lincRNA_199	Chromosome18	24303204	24304097	+	"TCONS_LINC_0001123[illegible]	exon_number "2"
lincRNA_2	Scaffold00833	6362	10098	+	"TCONS_LINC_0001129[illegible]	exon_number "1"
lincRNA_2	Scaffold00833	10168	11115	+	"TCONS_LINC_0001129[illegible]	exon_number "2"
lincRNA_20	Chromosome10	23536211	23537725	+	"TCONS_LINC_0000068[illegible]	exon_number "1"
lincRNA_20	Chromosome10	23537801	23538419	+	"TCONS_LINC_0000068[illegible]	exon_number "2"
lincRNA_200	Chromosome18	24404062	24404066	+	"TCONS_LINC_0001123[illegible]	exon_number "1"
lincRNA_200	Chromosome18	24404155	24404331	+	"TCONS_LINC_0001123[illegible]	exon_number "2"
lincRNA_200	Chromosome18	24404468	24404886	+	"TCONS_LINC_0001123[illegible]	exon_number "3"

lincRNA_200	Chromosome18	24404968	24405150	+	"TCONS_LINC_0000123(	exon_number "4"
lincRNA_200	Chromosome18	24405225	24406183	+	"TCONS_LINC_0000123(	exon_number "5"
lincRNA_201	Chromosome18	24466024	24466214	+	"TCONS_LINC_0000123	exon_number "1"
lincRNA_201	Chromosome18	24479957	24479966	+	"TCONS_LINC_0000123	exon_number "2"
lincRNA_202	Chromosome18	3712066	3712114	-	"TCONS_LINC_0000124(	exon_number "1"
lincRNA_202	Chromosome18	3717637	3718018	-	"TCONS_LINC_0000124(	exon_number "2"
lincRNA_203	Chromosome18	3864871	3865490	+	"TCONS_LINC_0000121	exon_number "1"
lincRNA_203	Chromosome18	3866821	3867259	+	"TCONS_LINC_0000121	exon_number "2"
lincRNA_204	Chromosome18	4073548	4075447	+	"TCONS_LINC_0000121	exon_number "1"
lincRNA_204	Chromosome18	4075924	4075980	+	"TCONS_LINC_0000121	exon_number "2"
lincRNA_204	Chromosome18	4076700	4076766	+	"TCONS_LINC_0000121	exon_number "3"
lincRNA_204	Chromosome18	4077375	4077972	+	"TCONS_LINC_0000121	exon_number "4"
lincRNA_204	Chromosome18	4074264	4074813	+	"TCONS_LINC_0000121	exon_number "1"
lincRNA_204	Chromosome18	4075384	4075468	+	"TCONS_LINC_0000121	exon_number "2"
lincRNA_204	Chromosome18	4075924	4075980	+	"TCONS_LINC_0000121	exon_number "3"
lincRNA_204	Chromosome18	4076700	4076766	+	"TCONS_LINC_0000121	exon_number "4"
lincRNA_204	Chromosome18	4077375	4077972	+	"TCONS_LINC_0000121	exon_number "5"
lincRNA_205	Chromosome18	4319119	4319759	+	"TCONS_LINC_0000121	exon_number "1"
lincRNA_205	Chromosome18	4319912	4321159	+	"TCONS_LINC_0000121	exon_number "2"
lincRNA_206	Chromosome18	4619996	4620186	+	"TCONS_LINC_0000121(	exon_number "1"
lincRNA_206	Chromosome18	4620356	4620530	+	"TCONS_LINC_0000121(	exon_number "2"
lincRNA_206	Chromosome18	4621955	4622720	+	"TCONS_LINC_0000121(	exon_number "3"
lincRNA_207	Chromosome18	538139	538264	+	"TCONS_LINC_0000120	exon_number "1"
lincRNA_207	Chromosome18	538995	539225	+	"TCONS_LINC_0000120	exon_number "2"
lincRNA_207	Chromosome18	539428	539729	+	"TCONS_LINC_0000120	exon_number "3"
lincRNA_207	Chromosome18	540397	540609	+	"TCONS_LINC_0000120	exon_number "4"
lincRNA_207	Chromosome18	540691	540833	+	"TCONS_LINC_0000120	exon_number "5"
lincRNA_208	Chromosome18	6597681	6598456	+	"TCONS_LINC_0000122	exon_number "1"
lincRNA_208	Chromosome18	6602648	6602721	+	"TCONS_LINC_0000122	exon_number "2"
lincRNA_209	Chromosome18	7063207	7063224	+	"TCONS_LINC_0000122	exon_number "1"
lincRNA_209	Chromosome18	7067104	7071465	+	"TCONS_LINC_0000122	exon_number "2"
lincRNA_209	Chromosome18	7066250	7071465	+	"TCONS_LINC_0000122	exon_number "1"
lincRNA_21	Chromosome10	23922234	23922526	+	"TCONS_LINC_0000068	exon_number "1"
lincRNA_21	Chromosome10	23928778	23928789	+	"TCONS_LINC_0000068	exon_number "2"
lincRNA_210	Chromosome18	8560422	8561195	-	"TCONS_LINC_0000124	exon_number "1"
lincRNA_210	Chromosome18	8561525	8562430	-	"TCONS_LINC_0000124	exon_number "2"
lincRNA_211	Scaffold00587	210467	211308	+	"TCONS_LINC_0000127	exon_number "1"
lincRNA_211	Scaffold00587	218813	218878	+	"TCONS_LINC_0000127	exon_number "2"
lincRNA_212	Scaffold00742	164957	165377	-	"TCONS_LINC_0000128	exon_number "1"
lincRNA_212	Scaffold00742	170532	170692	-	"TCONS_LINC_0000128	exon_number "2"
lincRNA_213	Scaffold00766	95567	97471	+	"TCONS_LINC_0000128	exon_number "1"
lincRNA_213	Scaffold00766	97613	97705	+	"TCONS_LINC_0000128	exon_number "2"
lincRNA_214	Scaffold00927	25401	25967	-	"TCONS_LINC_0000130	exon_number "1"
lincRNA_214	Scaffold00927	26329	27576	-	"TCONS_LINC_0000130	exon_number "2"
lincRNA_215	Scaffold00640	90578	90822	+	"TCONS_LINC_0000127	exon_number "1"
lincRNA_215	Scaffold00640	91681	91854	+	"TCONS_LINC_0000127	exon_number "2"
lincRNA_215	Scaffold00640	94934	95194	+	"TCONS_LINC_0000127	exon_number "3"
lincRNA_215	Scaffold00640	103324	103395	+	"TCONS_LINC_0000127	exon_number "4"
lincRNA_216	Scaffold00994	48779	49317	+	"TCONS_LINC_0000130	exon_number "1"
lincRNA_216	Scaffold00994	50249	50331	+	"TCONS_LINC_0000130	exon_number "2"
lincRNA_216	Scaffold00994	52719	55301	+	"TCONS_LINC_0000130	exon_number "3"
lincRNA_217	Scaffold00806	66665	66687	-	"TCONS_LINC_0000128	exon_number "1"
lincRNA_217	Scaffold00806	147630	147817	-	"TCONS_LINC_0000128	exon_number "2"
lincRNA_218	Scaffold00346	111820	113325	+	"TCONS_LINC_0000126(	exon_number "1"
lincRNA_218	Scaffold00346	118125	118167	+	"TCONS_LINC_0000126(	exon_number "2"
lincRNA_218	Scaffold00346	117509	118167	+	"TCONS_LINC_0000126	exon_number "1"
lincRNA_219	Chromosome07	10229606	10229639	+	"TCONS_LINC_0000044(	exon_number "1"

lincRNA_219	Chromosome07	10251121	10251819	+	"TCONS_LINC_00000446	exon_number "2"
lincRNA_22	Chromosome10	24205774	24205837	-	"TCONS_LINC_00000731	exon_number "1"
lincRNA_22	Chromosome10	24211947	24212155	-	"TCONS_LINC_00000731	exon_number "2"
lincRNA_220	Chromosome07	10919217	10919876	+	"TCONS_LINC_00000448	exon_number "1"
lincRNA_220	Chromosome07	10924822	10925281	+	"TCONS_LINC_00000448	exon_number "2"
lincRNA_221	Chromosome07	12169339	12170511	+	"TCONS_LINC_00000449	exon_number "1"
lincRNA_221	Chromosome07	12174687	12174769	+	"TCONS_LINC_00000449	exon_number "2"
lincRNA_222	Chromosome07	12550726	12550844	-	"TCONS_LINC_00000486	exon_number "1"
lincRNA_222	Chromosome07	12554771	12555470	-	"TCONS_LINC_00000486	exon_number "2"
lincRNA_223	Chromosome07	15100441	15102050	-	"TCONS_LINC_00000487	exon_number "1"
lincRNA_223	Chromosome07	15100441	15100582	-	"TCONS_LINC_00000488	exon_number "1"
lincRNA_223	Chromosome07	15102195	15103525	-	"TCONS_LINC_00000488	exon_number "2"
lincRNA_224	Chromosome07	16401350	16401620	+	"TCONS_LINC_00000451	exon_number "1"
lincRNA_224	Chromosome07	16407862	16408246	+	"TCONS_LINC_00000451	exon_number "2"
lincRNA_224	Chromosome07	16408594	16408823	+	"TCONS_LINC_00000451	exon_number "3"
lincRNA_224	Chromosome07	16401390	16403583	+	"TCONS_LINC_00000452	exon_number "1"
lincRNA_225	Chromosome07	1710117	1710578	+	"TCONS_LINC_00000434	exon_number "1"
lincRNA_225	Chromosome07	1711383	1711818	+	"TCONS_LINC_00000434	exon_number "2"
lincRNA_225	Chromosome07	1713623	1713739	+	"TCONS_LINC_00000434	exon_number "3"
lincRNA_225	Chromosome07	1714517	1714621	+	"TCONS_LINC_00000434	exon_number "4"
lincRNA_225	Chromosome07	1711745	1713739	+	"TCONS_LINC_00000436	exon_number "1"
lincRNA_225	Chromosome07	1713992	1714077	+	"TCONS_LINC_00000436	exon_number "2"
lincRNA_225	Chromosome07	1714245	1714360	+	"TCONS_LINC_00000436	exon_number "3"
lincRNA_225	Chromosome07	1714526	1714621	+	"TCONS_LINC_00000436	exon_number "4"
lincRNA_225	Chromosome07	1714252	1714351	+	"TCONS_LINC_00000437	exon_number "1"
lincRNA_225	Chromosome07	1714517	1714608	+	"TCONS_LINC_00000437	exon_number "2"
lincRNA_225	Chromosome07	1714689	1714765	+	"TCONS_LINC_00000437	exon_number "3"
lincRNA_225	Chromosome07	1714973	1715533	+	"TCONS_LINC_00000437	exon_number "4"
lincRNA_226	Chromosome07	18125864	18126288	-	"TCONS_LINC_00000491	exon_number "1"
lincRNA_226	Chromosome07	18145518	18145573	-	"TCONS_LINC_00000491	exon_number "2"
lincRNA_227	Chromosome07	18846040	18846350	-	"TCONS_LINC_00000492	exon_number "1"
lincRNA_227	Chromosome07	18846433	18846519	-	"TCONS_LINC_00000492	exon_number "2"
lincRNA_227	Chromosome07	18850469	18850555	-	"TCONS_LINC_00000492	exon_number "3"
lincRNA_227	Chromosome07	18862859	18862936	-	"TCONS_LINC_00000492	exon_number "4"
lincRNA_227	Chromosome07	18863316	18863494	-	"TCONS_LINC_00000492	exon_number "5"
lincRNA_227	Chromosome07	18865000	18865119	-	"TCONS_LINC_00000492	exon_number "6"
lincRNA_227	Chromosome07	18866151	18866315	-	"TCONS_LINC_00000492	exon_number "7"
lincRNA_228	Chromosome07	19046176	19047186	-	"TCONS_LINC_00000493	exon_number "1"
lincRNA_228	Chromosome07	19047672	19048953	-	"TCONS_LINC_00000493	exon_number "2"
lincRNA_229	Chromosome07	22895210	22895456	+	"TCONS_LINC_00000459	exon_number "1"
lincRNA_229	Chromosome07	22896245	22896296	+	"TCONS_LINC_00000459	exon_number "2"
lincRNA_229	Chromosome07	22897769	22897775	+	"TCONS_LINC_00000459	exon_number "3"
lincRNA_229	Chromosome07	22895210	22895795	+	"TCONS_LINC_00000458	exon_number "1"
lincRNA_229	Chromosome07	22896242	22896293	+	"TCONS_LINC_00000458	exon_number "2"
lincRNA_229	Chromosome07	22897766	22897775	+	"TCONS_LINC_00000458	exon_number "3"
lincRNA_23	Chromosome10	4518160	4518266	+	"TCONS_LINC_00000667	exon_number "1"
lincRNA_23	Chromosome10	4530537	4530858	+	"TCONS_LINC_00000667	exon_number "2"
lincRNA_230	Chromosome07	22984810	22985619	-	"TCONS_LINC_00000497	exon_number "1"
lincRNA_230	Chromosome07	22986297	22987533	-	"TCONS_LINC_00000497	exon_number "2"
lincRNA_231	Chromosome07	24096263	24096372	+	"TCONS_LINC_00000461	exon_number "1"
lincRNA_231	Chromosome07	24099914	24100171	+	"TCONS_LINC_00000461	exon_number "2"
lincRNA_232	Chromosome07	24115163	24115839	+	"TCONS_LINC_00000462	exon_number "1"
lincRNA_232	Chromosome07	24116219	24116344	+	"TCONS_LINC_00000462	exon_number "2"
lincRNA_232	Chromosome07	24116432	24116998	+	"TCONS_LINC_00000462	exon_number "3"
lincRNA_232	Chromosome07	24117088	24117170	+	"TCONS_LINC_00000462	exon_number "4"
lincRNA_232	Chromosome07	24118345	24119026	+	"TCONS_LINC_00000462	exon_number "5"
lincRNA_233	Chromosome07	24463555	24464320	+	"TCONS_LINC_00000464	exon_number "1"

lincRNA_233	Chromosome07	24464698	24465561	+	"TCONS_LINC_00000464	exon_number "2"
lincRNA_234	Chromosome07	25560600	25561950	+	"TCONS_LINC_00000466	exon_number "1"
lincRNA_234	Chromosome07	25593162	25593391	+	"TCONS_LINC_00000466	exon_number "2"
lincRNA_234	Chromosome07	25594513	25594661	+	"TCONS_LINC_00000466	exon_number "3"
lincRNA_234	Chromosome07	25593190	25593894	+	"TCONS_LINC_00000467	exon_number "1"
lincRNA_235	Chromosome07	26168504	26168811	+	"TCONS_LINC_00000468	exon_number "1"
lincRNA_235	Chromosome07	26168881	26169544	+	"TCONS_LINC_00000468	exon_number "2"
lincRNA_235	Chromosome07	26169697	26171825	+	"TCONS_LINC_00000468	exon_number "3"
lincRNA_236	Chromosome07	4618218	4618950	-	"TCONS_LINC_00000475	exon_number "1"
lincRNA_236	Chromosome07	4619859	4620432	-	"TCONS_LINC_00000475	exon_number "2"
lincRNA_237	Chromosome07	5079957	5081342	-	"TCONS_LINC_00000477	exon_number "1"
lincRNA_237	Chromosome07	5081751	5082948	-	"TCONS_LINC_00000477	exon_number "2"
lincRNA_238	Chromosome07	6828197	6828206	+	"TCONS_LINC_00000440	exon_number "1"
lincRNA_238	Chromosome07	6866311	6866868	+	"TCONS_LINC_00000440	exon_number "2"
lincRNA_239	Chromosome07	9040003	9041031	+	"TCONS_LINC_00000444	exon_number "1"
lincRNA_239	Chromosome07	9041189	9042159	+	"TCONS_LINC_00000444	exon_number "2"
lincRNA_24	Chromosome10	4559460	4559540	+	"TCONS_LINC_00000668	exon_number "1"
lincRNA_24	Chromosome10	4566133	4566275	+	"TCONS_LINC_00000668	exon_number "2"
lincRNA_240	Chromosome02	13198998	13200783	-	"TCONS_LINC_00000138	exon_number "1"
lincRNA_240	Chromosome02	13201133	13203262	-	"TCONS_LINC_00000138	exon_number "2"
lincRNA_241	Chromosome02	15429372	15429615	-	"TCONS_LINC_00000139	exon_number "1"
lincRNA_241	Chromosome02	15433289	15434783	-	"TCONS_LINC_00000139	exon_number "2"
lincRNA_242	Chromosome02	21340388	21340451	+	"TCONS_LINC_00000101	exon_number "1"
lincRNA_242	Chromosome02	21345158	21345337	+	"TCONS_LINC_00000101	exon_number "2"
lincRNA_243	Chromosome02	22663567	22663628	+	"TCONS_LINC_00000105	exon_number "1"
lincRNA_243	Chromosome02	22667368	22667733	+	"TCONS_LINC_00000105	exon_number "2"
lincRNA_244	Chromosome02	22700249	22700823	+	"TCONS_LINC_00000107	exon_number "1"
lincRNA_244	Chromosome02	22706182	22706215	+	"TCONS_LINC_00000107	exon_number "2"
lincRNA_245	Chromosome02	24509281	24510164	-	"TCONS_LINC_00000144	exon_number "1"
lincRNA_245	Chromosome02	24521739	24521809	-	"TCONS_LINC_00000144	exon_number "2"
lincRNA_246	Chromosome02	24530550	24530969	-	"TCONS_LINC_00000145	exon_number "1"
lincRNA_246	Chromosome02	24532945	24532969	-	"TCONS_LINC_00000145	exon_number "2"
lincRNA_247	Chromosome02	25890846	25890996	+	"TCONS_LINC_00000111	exon_number "1"
lincRNA_247	Chromosome02	25893741	25893952	+	"TCONS_LINC_00000111	exon_number "2"
lincRNA_248	Chromosome02	2746172	2749808	+	"TCONS_LINC_00000076	exon_number "1"
lincRNA_249	Chromosome02	27875634	27875860	+	"TCONS_LINC_00000112	exon_number "1"
lincRNA_249	Chromosome02	27895963	27895979	+	"TCONS_LINC_00000112	exon_number "2"
lincRNA_25	Chromosome10	4584308	4585339	-	"TCONS_LINC_00000693	exon_number "1"
lincRNA_25	Chromosome10	4584308	4584559	-	"TCONS_LINC_00000694	exon_number "1"
lincRNA_25	Chromosome10	4593283	4593641	-	"TCONS_LINC_00000694	exon_number "2"
lincRNA_250	Chromosome02	28057116	28060025	-	"TCONS_LINC_00000147	exon_number "1"
lincRNA_250	Chromosome02	28060867	28062453	-	"TCONS_LINC_00000147	exon_number "2"
lincRNA_250	Chromosome02	28057116	28061072	-	"TCONS_LINC_00000148	exon_number "1"
lincRNA_250	Chromosome02	28067019	28067213	-	"TCONS_LINC_00000148	exon_number "2"
lincRNA_250	Chromosome02	28067319	28067419	-	"TCONS_LINC_00000148	exon_number "3"
lincRNA_250	Chromosome02	28068588	28068733	-	"TCONS_LINC_00000148	exon_number "4"
lincRNA_250	Chromosome02	28069094	28069357	-	"TCONS_LINC_00000148	exon_number "5"
lincRNA_250	Chromosome02	28057116	28060020	-	"TCONS_LINC_00000149	exon_number "1"
lincRNA_250	Chromosome02	28060862	28061072	-	"TCONS_LINC_00000149	exon_number "2"
lincRNA_250	Chromosome02	28067019	28067213	-	"TCONS_LINC_00000149	exon_number "3"
lincRNA_250	Chromosome02	28067319	28067419	-	"TCONS_LINC_00000149	exon_number "4"
lincRNA_250	Chromosome02	28068592	28068733	-	"TCONS_LINC_00000149	exon_number "5"
lincRNA_250	Chromosome02	28069094	28069367	-	"TCONS_LINC_00000149	exon_number "6"
lincRNA_251	Chromosome02	29380963	29383185	+	"TCONS_LINC_00000114	exon_number "1"
lincRNA_251	Chromosome02	29382518	29382526	+	"TCONS_LINC_00000115	exon_number "1"
lincRNA_251	Chromosome02	29419888	29421081	+	"TCONS_LINC_00000115	exon_number "2"
lincRNA_252	Chromosome02	30158404	30158749	+	"TCONS_LINC_00000120	exon_number "1"

lincRNA_252	Chromosome02	30164980	30165062	+	"TCONS_LINC_0000120	exon_number "2"
lincRNA_253	Chromosome02	32175672	32177887	-	"TCONS_LINC_0000152	exon_number "1"
lincRNA_253	Chromosome02	32185226	32187378	-	"TCONS_LINC_0000152	exon_number "2"
lincRNA_254	Chromosome02	3401114	3401482	+	"TCONS_LINC_0000077	exon_number "1"
lincRNA_254	Chromosome02	3401610	3402060	+	"TCONS_LINC_0000077	exon_number "2"
lincRNA_254	Chromosome02	3401303	3401482	+	"TCONS_LINC_0000078	exon_number "1"
lincRNA_254	Chromosome02	3401610	3401685	+	"TCONS_LINC_0000078	exon_number "2"
lincRNA_254	Chromosome02	3402250	3402299	+	"TCONS_LINC_0000078	exon_number "3"
lincRNA_254	Chromosome02	3402415	3403594	+	"TCONS_LINC_0000078	exon_number "4"
lincRNA_255	Chromosome02	6122934	6123619	+	"TCONS_LINC_0000083	exon_number "1"
lincRNA_255	Chromosome02	6125381	6125461	+	"TCONS_LINC_0000083	exon_number "2"
lincRNA_256	Chromosome02	8589165	8589190	-	"TCONS_LINC_0000134	exon_number "1"
lincRNA_256	Chromosome02	8591545	8592798	-	"TCONS_LINC_0000134	exon_number "2"
lincRNA_257	Chromosome02	9012674	9012698	-	"TCONS_LINC_0000135	exon_number "1"
lincRNA_257	Chromosome02	9021210	9022157	-	"TCONS_LINC_0000135	exon_number "2"
lincRNA_257	Chromosome02	9012674	9012716	-	"TCONS_LINC_0000136	exon_number "1"
lincRNA_257	Chromosome02	9021228	9022623	-	"TCONS_LINC_0000136	exon_number "2"
lincRNA_258	Chromosome02	9695152	9695403	+	"TCONS_LINC_0000091	exon_number "1"
lincRNA_258	Chromosome02	9695748	9695883	+	"TCONS_LINC_0000091	exon_number "2"
lincRNA_258	Chromosome02	9697253	9697350	+	"TCONS_LINC_0000091	exon_number "3"
lincRNA_258	Chromosome02	9697798	9697991	+	"TCONS_LINC_0000091	exon_number "4"
lincRNA_258	Chromosome02	9698859	9699196	+	"TCONS_LINC_0000091	exon_number "5"
lincRNA_258	Chromosome02	9695788	9697350	+	"TCONS_LINC_0000092	exon_number "1"
lincRNA_258	Chromosome02	9697798	9698030	+	"TCONS_LINC_0000092	exon_number "2"
lincRNA_258	Chromosome02	9698544	9698653	+	"TCONS_LINC_0000092	exon_number "3"
lincRNA_259	Scaffold00824	78697	80911	-	"TCONS_LINC_0001291	exon_number "1"
lincRNA_259	Scaffold00824	86471	87251	-	"TCONS_LINC_0001291	exon_number "2"
lincRNA_259	Scaffold00824	93341	94917	-	"TCONS_LINC_0001291	exon_number "3"
lincRNA_26	Chromosome10	5598578	5598819	+	"TCONS_LINC_0000670	exon_number "1"
lincRNA_26	Chromosome10	5598916	5599048	+	"TCONS_LINC_0000670	exon_number "2"
lincRNA_26	Chromosome10	5600316	5600821	+	"TCONS_LINC_0000670	exon_number "3"
lincRNA_260	Scaffold00817	118598	118671	-	"TCONS_LINC_0001290	exon_number "1"
lincRNA_260	Scaffold00817	140694	140958	-	"TCONS_LINC_0001290	exon_number "2"
lincRNA_261	Scaffold00334	265428	265494	-	"TCONS_LINC_0001256	exon_number "1"
lincRNA_261	Scaffold00334	273132	273711	-	"TCONS_LINC_0001256	exon_number "2"
lincRNA_262	Scaffold00334	278573	280666	-	"TCONS_LINC_0001258	exon_number "1"
lincRNA_263	Scaffold00334	282351	285596	-	"TCONS_LINC_0001259	exon_number "1"
lincRNA_263	Scaffold00334	286055	286688	-	"TCONS_LINC_0001259	exon_number "2"
lincRNA_263	Scaffold00334	286781	286986	-	"TCONS_LINC_0001259	exon_number "3"
lincRNA_264	Scaffold00334	67029	67569	-	"TCONS_LINC_0001253	exon_number "1"
lincRNA_264	Scaffold00334	90829	91738	-	"TCONS_LINC_0001253	exon_number "2"
lincRNA_265	Scaffold00829	13332	13574	+	"TCONS_LINC_0001292	exon_number "1"
lincRNA_265	Scaffold00829	25374	26269	+	"TCONS_LINC_0001292	exon_number "2"
lincRNA_265	Scaffold00829	13334	13574	+	"TCONS_LINC_0001293	exon_number "1"
lincRNA_265	Scaffold00829	20591	20648	+	"TCONS_LINC_0001293	exon_number "2"
lincRNA_265	Scaffold00829	21278	23466	+	"TCONS_LINC_0001293	exon_number "3"
lincRNA_266	Scaffold00476	235855	235985	+	"TCONS_LINC_0001266	exon_number "1"
lincRNA_266	Scaffold00476	236070	236212	+	"TCONS_LINC_0001266	exon_number "2"
lincRNA_266	Scaffold00476	241076	241385	+	"TCONS_LINC_0001266	exon_number "3"
lincRNA_266	Scaffold00476	259735	259746	+	"TCONS_LINC_0001266	exon_number "4"
lincRNA_267	Scaffold01245	11444	11571	-	"TCONS_LINC_0001327	exon_number "1"
lincRNA_267	Scaffold01245	13385	13533	-	"TCONS_LINC_0001327	exon_number "2"
lincRNA_267	Scaffold01245	12661	13109	-	"TCONS_LINC_0001328	exon_number "1"
lincRNA_267	Scaffold01245	13385	13524	-	"TCONS_LINC_0001328	exon_number "2"
lincRNA_268	Chromosome06	10208924	10210223	-	"TCONS_LINC_0000401	exon_number "1"
lincRNA_268	Chromosome06	10210358	10210435	-	"TCONS_LINC_0000401	exon_number "2"
lincRNA_268	Chromosome06	10210537	10211264	-	"TCONS_LINC_0000401	exon_number "3"

lincRNA_268	Chromosome06	10211285	10211350	-	"TCONS_LINC_00000401	exon_number "4"
lincRNA_268	Chromosome06	10208924	10210070	-	"TCONS_LINC_00000402	exon_number "1"
lincRNA_268	Chromosome06	10210358	10211264	-	"TCONS_LINC_00000402	exon_number "2"
lincRNA_268	Chromosome06	10211285	10211375	-	"TCONS_LINC_00000402	exon_number "3"
lincRNA_269	Chromosome06	10922028	10922041	-	"TCONS_LINC_00000404	exon_number "1"
lincRNA_269	Chromosome06	10967207	10969884	-	"TCONS_LINC_00000404	exon_number "2"
lincRNA_27	Chromosome10	7216793	7222522	-	"TCONS_LINC_00000697	exon_number "1"
lincRNA_27	Chromosome10	7222575	7224126	-	"TCONS_LINC_00000697	exon_number "2"
lincRNA_27	Chromosome10	7216793	7218399	-	"TCONS_LINC_00000698	exon_number "1"
lincRNA_27	Chromosome10	7223167	7224806	-	"TCONS_LINC_00000698	exon_number "2"
lincRNA_270	Chromosome06	13256992	13257007	+	"TCONS_LINC_00000365	exon_number "1"
lincRNA_270	Chromosome06	13330091	13330357	+	"TCONS_LINC_00000365	exon_number "2"
lincRNA_271	Chromosome06	15905297	15905940	-	"TCONS_LINC_00000410	exon_number "1"
lincRNA_271	Chromosome06	15907345	15907455	-	"TCONS_LINC_00000410	exon_number "2"
lincRNA_271	Chromosome06	15907647	15908534	-	"TCONS_LINC_00000410	exon_number "3"
lincRNA_272	Chromosome06	16333951	16333984	-	"TCONS_LINC_00000411	exon_number "1"
lincRNA_272	Chromosome06	16334619	16334696	-	"TCONS_LINC_00000411	exon_number "2"
lincRNA_272	Chromosome06	16334892	16335105	-	"TCONS_LINC_00000411	exon_number "3"
lincRNA_272	Chromosome06	16336158	16336862	-	"TCONS_LINC_00000411	exon_number "4"
lincRNA_273	Chromosome06	1828751	1829448	-	"TCONS_LINC_00000389	exon_number "1"
lincRNA_273	Chromosome06	1829555	1830085	-	"TCONS_LINC_00000389	exon_number "2"
lincRNA_273	Chromosome06	1830629	1830701	-	"TCONS_LINC_00000389	exon_number "3"
lincRNA_273	Chromosome06	1830882	1830882	-	"TCONS_LINC_00000389	exon_number "4"
lincRNA_274	Chromosome06	20525127	20526346	-	"TCONS_LINC_00000414	exon_number "1"
lincRNA_274	Chromosome06	20527088	20527236	-	"TCONS_LINC_00000414	exon_number "2"
lincRNA_274	Chromosome06	20525127	20526234	-	"TCONS_LINC_00000415	exon_number "1"
lincRNA_274	Chromosome06	20527166	20527242	-	"TCONS_LINC_00000415	exon_number "2"
lincRNA_275	Chromosome06	2134862	2136354	+	"TCONS_LINC_00000359	exon_number "1"
lincRNA_275	Chromosome06	2136975	2137018	+	"TCONS_LINC_00000359	exon_number "2"
lincRNA_276	Chromosome06	21835688	21836100	-	"TCONS_LINC_00000419	exon_number "1"
lincRNA_276	Chromosome06	21836945	21837712	-	"TCONS_LINC_00000419	exon_number "2"
lincRNA_277	Chromosome06	22674073	22675381	+	"TCONS_LINC_00000370	exon_number "1"
lincRNA_277	Chromosome06	22675636	22676322	+	"TCONS_LINC_00000370	exon_number "2"
lincRNA_278	Chromosome06	22917129	22917597	-	"TCONS_LINC_00000421	exon_number "1"
lincRNA_278	Chromosome06	22917709	22918115	-	"TCONS_LINC_00000421	exon_number "2"
lincRNA_278	Chromosome06	22920505	22920962	-	"TCONS_LINC_00000421	exon_number "3"
lincRNA_279	Chromosome06	23307514	23308056	+	"TCONS_LINC_00000371	exon_number "1"
lincRNA_279	Chromosome06	23310210	23310618	+	"TCONS_LINC_00000371	exon_number "2"
lincRNA_28	Chromosome10	7229138	7230209	-	"TCONS_LINC_00000700	exon_number "1"
lincRNA_28	Chromosome10	7237796	7237853	-	"TCONS_LINC_00000700	exon_number "2"
lincRNA_280	Chromosome06	23440553	23440947	+	"TCONS_LINC_00000372	exon_number "1"
lincRNA_280	Chromosome06	23441989	23442152	+	"TCONS_LINC_00000372	exon_number "2"
lincRNA_280	Chromosome06	23442240	23442395	+	"TCONS_LINC_00000372	exon_number "3"
lincRNA_280	Chromosome06	23442587	23443838	+	"TCONS_LINC_00000372	exon_number "4"
lincRNA_281	Chromosome06	23739168	23740578	+	"TCONS_LINC_00000373	exon_number "1"
lincRNA_281	Chromosome06	23740678	23741432	+	"TCONS_LINC_00000373	exon_number "2"
lincRNA_281	Chromosome06	23739206	23739307	+	"TCONS_LINC_00000374	exon_number "1"
lincRNA_281	Chromosome06	23740678	23741432	+	"TCONS_LINC_00000374	exon_number "2"
lincRNA_282	Chromosome06	241979	242521	-	"TCONS_LINC_00000388	exon_number "1"
lincRNA_282	Chromosome06	296613	296659	-	"TCONS_LINC_00000388	exon_number "2"
lincRNA_283	Chromosome06	24220198	24220259	+	"TCONS_LINC_00000376	exon_number "1"
lincRNA_283	Chromosome06	24221128	24221307	+	"TCONS_LINC_00000376	exon_number "2"
lincRNA_283	Chromosome06	24222410	24223084	+	"TCONS_LINC_00000376	exon_number "3"
lincRNA_284	Chromosome06	24623421	24623999	+	"TCONS_LINC_00000377	exon_number "1"
lincRNA_284	Chromosome06	24636469	24637126	+	"TCONS_LINC_00000377	exon_number "2"
lincRNA_284	Chromosome06	24624163	24624173	+	"TCONS_LINC_00000378	exon_number "1"
lincRNA_284	Chromosome06	24636469	24637126	+	"TCONS_LINC_00000378	exon_number "2"

lincRNA_285	Chromosome06	24972483	24973982	+	"TCONS_LINC_0000038[illegible]	exon_number "1"
lincRNA_285	Chromosome06	24974122	24974516	+	"TCONS_LINC_0000038[illegible]	exon_number "2"
lincRNA_285	Chromosome06	24975755	24975971	+	"TCONS_LINC_0000038[illegible]	exon_number "3"
lincRNA_285	Chromosome06	24979852	24980968	+	"TCONS_LINC_0000038[illegible]	exon_number "4"
lincRNA_286	Chromosome06	26443800	26443874	-	"TCONS_LINC_0000042[illegible]	exon_number "1"
lincRNA_286	Chromosome06	26445385	26445545	-	"TCONS_LINC_0000042[illegible]	exon_number "2"
lincRNA_286	Chromosome06	26445685	26446989	-	"TCONS_LINC_0000042[illegible]	exon_number "3"
lincRNA_287	Chromosome06	26683522	26684093	+	"TCONS_LINC_0000038[illegible]	exon_number "1"
lincRNA_287	Chromosome06	26684316	26684492	+	"TCONS_LINC_0000038[illegible]	exon_number "2"
lincRNA_287	Chromosome06	26690027	26691452	+	"TCONS_LINC_0000038[illegible]	exon_number "3"
lincRNA_287	Chromosome06	26683548	26684093	+	"TCONS_LINC_0000038[illegible]	exon_number "1"
lincRNA_287	Chromosome06	26684316	26684492	+	"TCONS_LINC_0000038[illegible]	exon_number "2"
lincRNA_287	Chromosome06	26689445	26691452	+	"TCONS_LINC_0000038[illegible]	exon_number "3"
lincRNA_287	Chromosome06	26683560	26684093	+	"TCONS_LINC_0000038[illegible]	exon_number "1"
lincRNA_287	Chromosome06	26684316	26684496	+	"TCONS_LINC_0000038[illegible]	exon_number "2"
lincRNA_287	Chromosome06	26689449	26691452	+	"TCONS_LINC_0000038[illegible]	exon_number "3"
lincRNA_288	Chromosome06	27861074	27861089	+	"TCONS_LINC_0000038[illegible]	exon_number "1"
lincRNA_288	Chromosome06	27866504	27867552	+	"TCONS_LINC_0000038[illegible]	exon_number "2"
lincRNA_289	Chromosome06	7475096	7476479	-	"TCONS_LINC_0000039[illegible]	exon_number "1"
lincRNA_289	Chromosome06	7476746	7477376	-	"TCONS_LINC_0000039[illegible]	exon_number "2"
lincRNA_29	Chromosome10	7291278	7292001	-	"TCONS_LINC_0000070[illegible]	exon_number "1"
lincRNA_29	Chromosome10	7294028	7294150	-	"TCONS_LINC_0000070[illegible]	exon_number "2"
lincRNA_290	Chromosome06	7844407	7844985	+	"TCONS_LINC_0000036[illegible]	exon_number "1"
lincRNA_290	Chromosome06	7847136	7847142	+	"TCONS_LINC_0000036[illegible]	exon_number "2"
lincRNA_291	Chromosome06	8517885	8519569	+	"TCONS_LINC_0000036[illegible]	exon_number "1"
lincRNA_291	Chromosome06	8519672	8520314	+	"TCONS_LINC_0000036[illegible]	exon_number "2"
lincRNA_292	Chromosome06	9409013	9409168	-	"TCONS_LINC_0000039[illegible]	exon_number "1"
lincRNA_292	Chromosome06	9428858	9428957	-	"TCONS_LINC_0000039[illegible]	exon_number "2"
lincRNA_293	Chromosome06	9701803	9702609	+	"TCONS_LINC_0000036[illegible]	exon_number "1"
lincRNA_293	Chromosome06	9706322	9706328	+	"TCONS_LINC_0000036[illegible]	exon_number "2"
lincRNA_294	Chromosome06	9772485	9772503	-	"TCONS_LINC_0000039[illegible]	exon_number "1"
lincRNA_294	Chromosome06	9775896	9777836	-	"TCONS_LINC_0000039[illegible]	exon_number "2"
lincRNA_295	Chromosome06	9778143	9779356	-	"TCONS_LINC_0000039[illegible]	exon_number "1"
lincRNA_295	Chromosome06	9778143	9779068	-	"TCONS_LINC_0000040[illegible]	exon_number "1"
lincRNA_295	Chromosome06	9781400	9781481	-	"TCONS_LINC_0000040[illegible]	exon_number "2"
lincRNA_296	Chromosome01	12205503	12205848	+	"TCONS_LINC_0000001[illegible]	exon_number "1"
lincRNA_296	Chromosome01	12212443	12212513	+	"TCONS_LINC_0000001[illegible]	exon_number "2"
lincRNA_297	Chromosome01	12830717	12830729	+	"TCONS_LINC_0000001[illegible]	exon_number "1"
lincRNA_297	Chromosome01	12896121	12896814	+	"TCONS_LINC_0000001[illegible]	exon_number "2"
lincRNA_298	Chromosome01	13122894	13123284	+	"TCONS_LINC_0000001[illegible]	exon_number "1"
lincRNA_298	Chromosome01	13124796	13125290	+	"TCONS_LINC_0000001[illegible]	exon_number "2"
lincRNA_299	Chromosome01	14261223	14261535	+	"TCONS_LINC_0000001[illegible]	exon_number "1"
lincRNA_299	Chromosome01	14322830	14323231	+	"TCONS_LINC_0000001[illegible]	exon_number "2"
lincRNA_3	Scaffold00833	79830	79864	+	"TCONS_LINC_0001300[illegible]	exon_number "1"
lincRNA_3	Scaffold00833	93576	95871	+	"TCONS_LINC_0001300[illegible]	exon_number "2"
lincRNA_30	Chromosome10	7417182	7421739	+	"TCONS_LINC_0000067[illegible]	exon_number "1"
lincRNA_30	Chromosome10	7421874	7423433	+	"TCONS_LINC_0000067[illegible]	exon_number "2"
lincRNA_300	Chromosome01	14322389	14322783	-	"TCONS_LINC_0000004[illegible]	exon_number "1"
lincRNA_300	Chromosome01	14327140	14327146	-	"TCONS_LINC_0000004[illegible]	exon_number "2"
lincRNA_301	Chromosome01	14701645	14701663	+	"TCONS_LINC_0000001[illegible]	exon_number "1"
lincRNA_301	Chromosome01	14804538	14804869	+	"TCONS_LINC_0000001[illegible]	exon_number "2"
lincRNA_302	Chromosome01	17561509	17566214	-	"TCONS_LINC_0000005[illegible]	exon_number "1"
lincRNA_303	Chromosome01	17722472	17725020	+	"TCONS_LINC_0000001[illegible]	exon_number "1"
lincRNA_303	Chromosome01	17725043	17725838	+	"TCONS_LINC_0000001[illegible]	exon_number "2"
lincRNA_303	Chromosome01	17722472	17723940	+	"TCONS_LINC_0000001[illegible]	exon_number "1"
lincRNA_303	Chromosome01	17744062	17744113	+	"TCONS_LINC_0000001[illegible]	exon_number "2"
lincRNA_304	Chromosome01	22897040	22897503	+	"TCONS_LINC_0000002[illegible]	exon_number "1"

lincRNA_304	Chromosome01	22899068	22899289	+	"TCONS_LINC_0000002[illegible]	exon_number "2"
lincRNA_305	Chromosome01	22898343	22898387	+	"TCONS_LINC_0000002[illegible]	exon_number "1"
lincRNA_305	Chromosome01	22901027	22901569	+	"TCONS_LINC_0000002[illegible]	exon_number "2"
lincRNA_306	Chromosome01	24067001	24069440	+	"TCONS_LINC_0000002[illegible]	exon_number "1"
lincRNA_307	Chromosome01	24543457	24543513	+	"TCONS_LINC_0000002[illegible]	exon_number "1"
lincRNA_307	Chromosome01	24544536	24544636	+	"TCONS_LINC_0000002[illegible]	exon_number "2"
lincRNA_307	Chromosome01	24544830	24544842	+	"TCONS_LINC_0000002[illegible]	exon_number "3"
lincRNA_307	Chromosome01	24545349	24545436	+	"TCONS_LINC_0000002[illegible]	exon_number "4"
lincRNA_307	Chromosome01	24551735	24551821	+	"TCONS_LINC_0000002[illegible]	exon_number "5"
lincRNA_307	Chromosome01	24552413	24552507	+	"TCONS_LINC_0000002[illegible]	exon_number "6"
lincRNA_307	Chromosome01	24553314	24553443	+	"TCONS_LINC_0000002[illegible]	exon_number "7"
lincRNA_307	Chromosome01	24553537	24553885	+	"TCONS_LINC_0000002[illegible]	exon_number "8"
lincRNA_307	Chromosome01	24544778	24544842	+	"TCONS_LINC_0000003[illegible]	exon_number "1"
lincRNA_307	Chromosome01	24545349	24551821	+	"TCONS_LINC_0000003[illegible]	exon_number "2"
lincRNA_307	Chromosome01	24552413	24552507	+	"TCONS_LINC_0000003[illegible]	exon_number "3"
lincRNA_307	Chromosome01	24553314	24553443	+	"TCONS_LINC_0000003[illegible]	exon_number "4"
lincRNA_307	Chromosome01	24553537	24553885	+	"TCONS_LINC_0000003[illegible]	exon_number "5"
lincRNA_308	Chromosome01	24682413	24683350	+	"TCONS_LINC_0000003[illegible]	exon_number "1"
lincRNA_308	Chromosome01	24684708	24685475	+	"TCONS_LINC_0000003[illegible]	exon_number "2"
lincRNA_309	Chromosome01	25602210	25602585	+	"TCONS_LINC_0000003[illegible]	exon_number "1"
lincRNA_309	Chromosome01	25605935	25606531	+	"TCONS_LINC_0000003[illegible]	exon_number "2"
lincRNA_31	Chromosome10	7735038	7735676	-	"TCONS_LINC_0000070[illegible]	exon_number "1"
lincRNA_31	Chromosome10	7735992	7737241	-	"TCONS_LINC_0000070[illegible]	exon_number "2"
lincRNA_310	Chromosome01	25725376	25725918	-	"TCONS_LINC_0000005[illegible]	exon_number "1"
lincRNA_310	Chromosome01	25726132	25726382	-	"TCONS_LINC_0000005[illegible]	exon_number "2"
lincRNA_310	Chromosome01	25726909	25726971	-	"TCONS_LINC_0000005[illegible]	exon_number "3"
lincRNA_310	Chromosome01	25727602	25728057	-	"TCONS_LINC_0000005[illegible]	exon_number "4"
lincRNA_310	Chromosome01	25725960	25726218	-	"TCONS_LINC_0000005[illegible]	exon_number "1"
lincRNA_310	Chromosome01	25726290	25726378	-	"TCONS_LINC_0000005[illegible]	exon_number "2"
lincRNA_310	Chromosome01	25726719	25726792	-	"TCONS_LINC_0000005[illegible]	exon_number "3"
lincRNA_311	Chromosome01	27169349	27169767	-	"TCONS_LINC_0000006[illegible]	exon_number "1"
lincRNA_311	Chromosome01	27169897	27170679	-	"TCONS_LINC_0000006[illegible]	exon_number "2"
lincRNA_311	Chromosome01	27170983	27171416	-	"TCONS_LINC_0000006[illegible]	exon_number "3"
lincRNA_311	Chromosome01	27169914	27171210	-	"TCONS_LINC_0000006[illegible]	exon_number "1"
lincRNA_311	Chromosome01	27172036	27172301	-	"TCONS_LINC_0000006[illegible]	exon_number "2"
lincRNA_312	Chromosome01	27221945	27223337	-	"TCONS_LINC_0000006[illegible]	exon_number "1"
lincRNA_312	Chromosome01	27224413	27224650	-	"TCONS_LINC_0000006[illegible]	exon_number "2"
lincRNA_313	Chromosome01	27771982	27774245	+	"TCONS_LINC_0000003[illegible]	exon_number "1"
lincRNA_314	Chromosome01	29689323	29691751	+	"TCONS_LINC_0000004[illegible]	exon_number "1"
lincRNA_314	Chromosome01	29692081	29693454	+	"TCONS_LINC_0000004[illegible]	exon_number "2"
lincRNA_315	Chromosome01	29956371	29957929	-	"TCONS_LINC_0000006[illegible]	exon_number "1"
lincRNA_315	Chromosome01	29958015	29958213	-	"TCONS_LINC_0000006[illegible]	exon_number "2"
lincRNA_315	Chromosome01	29958384	29958522	-	"TCONS_LINC_0000006[illegible]	exon_number "3"
lincRNA_315	Chromosome01	29962528	29962910	-	"TCONS_LINC_0000006[illegible]	exon_number "4"
lincRNA_315	Chromosome01	29963083	29963314	-	"TCONS_LINC_0000006[illegible]	exon_number "5"
lincRNA_315	Chromosome01	29963730	29963811	-	"TCONS_LINC_0000006[illegible]	exon_number "6"
lincRNA_315	Chromosome01	29964463	29964518	-	"TCONS_LINC_0000006[illegible]	exon_number "7"
lincRNA_315	Chromosome01	29964644	29965411	-	"TCONS_LINC_0000006[illegible]	exon_number "8"
lincRNA_316	Chromosome01	29969179	29971421	-	"TCONS_LINC_0000006[illegible]	exon_number "1"
lincRNA_316	Chromosome01	29971455	29971529	-	"TCONS_LINC_0000006[illegible]	exon_number "2"
lincRNA_317	Chromosome01	30071306	30075768	-	"TCONS_LINC_0000006[illegible]	exon_number "1"
lincRNA_317	Chromosome01	30082739	30083167	-	"TCONS_LINC_0000006[illegible]	exon_number "2"
lincRNA_318	Chromosome01	32700298	32700389	-	"TCONS_LINC_0000007[illegible]	exon_number "1"
lincRNA_318	Chromosome01	32701175	32701311	-	"TCONS_LINC_0000007[illegible]	exon_number "2"
lincRNA_318	Chromosome01	32701465	32701542	-	"TCONS_LINC_0000007[illegible]	exon_number "3"
lincRNA_318	Chromosome01	32701757	32701925	-	"TCONS_LINC_0000007[illegible]	exon_number "4"
lincRNA_318	Chromosome01	32702165	32702643	-	"TCONS_LINC_0000007[illegible]	exon_number "5"

lincRNA_319	Chromosome01	6700602	6700763	+	"TCONS_LINC_0000000[illegible]	exon_number "1"
lincRNA_319	Chromosome01	6705178	6705315	+	"TCONS_LINC_0000000[illegible]	exon_number "2"
lincRNA_32	Chromosome10	8110867	8111745	-	"TCONS_LINC_0000070[illegible]	exon_number "1"
lincRNA_32	Chromosome10	8116710	8116855	-	"TCONS_LINC_0000070[illegible]	exon_number "2"
lincRNA_32	Chromosome10	8116886	8117489	-	"TCONS_LINC_0000070[illegible]	exon_number "3"
lincRNA_320	Chromosome01	8813133	8813474	-	"TCONS_LINC_0000004[illegible]	exon_number "1"
lincRNA_320	Chromosome01	8864363	8864369	-	"TCONS_LINC_0000004[illegible]	exon_number "2"
lincRNA_321	Chromosome03	10796787	10797133	+	"TCONS_LINC_0000017[illegible]	exon_number "1"
lincRNA_321	Chromosome03	10809648	10809652	+	"TCONS_LINC_0000017[illegible]	exon_number "2"
lincRNA_322	Chromosome03	11371090	11371244	-	"TCONS_LINC_0000021[illegible]	exon_number "1"
lincRNA_322	Chromosome03	11371316	11371613	-	"TCONS_LINC_0000021[illegible]	exon_number "2"
lincRNA_322	Chromosome03	11378751	11383309	-	"TCONS_LINC_0000021[illegible]	exon_number "3"
lincRNA_323	Chromosome03	121853	123094	+	"TCONS_LINC_0000015[illegible]	exon_number "1"
lincRNA_323	Chromosome03	123249	123404	+	"TCONS_LINC_0000015[illegible]	exon_number "2"
lincRNA_323	Chromosome03	123797	123890	+	"TCONS_LINC_0000015[illegible]	exon_number "3"
lincRNA_324	Chromosome03	14415262	14416356	+	"TCONS_LINC_0000018[illegible]	exon_number "1"
lincRNA_324	Chromosome03	14416727	14417443	+	"TCONS_LINC_0000018[illegible]	exon_number "2"
lincRNA_325	Chromosome03	14652820	14653721	-	"TCONS_LINC_0000021[illegible]	exon_number "1"
lincRNA_325	Chromosome03	14657145	14657166	-	"TCONS_LINC_0000021[illegible]	exon_number "2"
lincRNA_326	Chromosome03	1484604	1485353	+	"TCONS_LINC_0000016[illegible]	exon_number "1"
lincRNA_326	Chromosome03	1486203	1486276	+	"TCONS_LINC_0000016[illegible]	exon_number "2"
lincRNA_326	Chromosome03	1486364	1486865	+	"TCONS_LINC_0000016[illegible]	exon_number "3"
lincRNA_327	Chromosome03	15716956	15719312	-	"TCONS_LINC_0000022[illegible]	exon_number "1"
lincRNA_327	Chromosome03	15721243	15721574	-	"TCONS_LINC_0000022[illegible]	exon_number "2"
lincRNA_327	Chromosome03	15720306	15721599	-	"TCONS_LINC_0000022[illegible]	exon_number "1"
lincRNA_328	Chromosome03	19197963	19198342	+	"TCONS_LINC_0000018[illegible]	exon_number "1"
lincRNA_328	Chromosome03	19201915	19202796	+	"TCONS_LINC_0000018[illegible]	exon_number "2"
lincRNA_329	Chromosome03	19283957	19284579	+	"TCONS_LINC_0000018[illegible]	exon_number "1"
lincRNA_329	Chromosome03	19283960	19284343	+	"TCONS_LINC_0000018[illegible]	exon_number "1"
lincRNA_329	Chromosome03	19291805	19291944	+	"TCONS_LINC_0000018[illegible]	exon_number "2"
lincRNA_33	Chromosome10	914445	914583	-	"TCONS_LINC_0000069[illegible]	exon_number "1"
lincRNA_33	Chromosome10	914743	915015	-	"TCONS_LINC_0000069[illegible]	exon_number "2"
lincRNA_33	Chromosome10	915089	915211	-	"TCONS_LINC_0000069[illegible]	exon_number "3"
lincRNA_33	Chromosome10	916918	917269	-	"TCONS_LINC_0000069[illegible]	exon_number "4"
lincRNA_330	Chromosome03	2050412	2050996	+	"TCONS_LINC_0000016[illegible]	exon_number "1"
lincRNA_330	Chromosome03	2051658	2052826	+	"TCONS_LINC_0000016[illegible]	exon_number "2"
lincRNA_331	Chromosome03	23905070	23905911	-	"TCONS_LINC_0000022[illegible]	exon_number "1"
lincRNA_331	Chromosome03	23907063	23907513	-	"TCONS_LINC_0000022[illegible]	exon_number "2"
lincRNA_332	Chromosome03	24817842	24819499	-	"TCONS_LINC_0000023[illegible]	exon_number "1"
lincRNA_332	Chromosome03	24819573	24819995	-	"TCONS_LINC_0000023[illegible]	exon_number "2"
lincRNA_333	Chromosome03	2547183	2548410	+	"TCONS_LINC_0000016[illegible]	exon_number "1"
lincRNA_333	Chromosome03	2550208	2551527	+	"TCONS_LINC_0000016[illegible]	exon_number "2"
lincRNA_334	Chromosome03	2553402	2553483	+	"TCONS_LINC_0000016[illegible]	exon_number "1"
lincRNA_334	Chromosome03	2553612	2553737	+	"TCONS_LINC_0000016[illegible]	exon_number "2"
lincRNA_334	Chromosome03	2554389	2555526	+	"TCONS_LINC_0000016[illegible]	exon_number "3"
lincRNA_335	Chromosome03	27818390	27818544	-	"TCONS_LINC_0000023[illegible]	exon_number "1"
lincRNA_335	Chromosome03	27820016	27820111	-	"TCONS_LINC_0000023[illegible]	exon_number "2"
lincRNA_335	Chromosome03	27820225	27820408	-	"TCONS_LINC_0000023[illegible]	exon_number "3"
lincRNA_335	Chromosome03	27818665	27820111	-	"TCONS_LINC_0000023[illegible]	exon_number "1"
lincRNA_335	Chromosome03	27820225	27820400	-	"TCONS_LINC_0000023[illegible]	exon_number "2"
lincRNA_336	Chromosome03	29275231	29275478	+	"TCONS_LINC_0000019[illegible]	exon_number "1"
lincRNA_336	Chromosome03	29279915	29280070	+	"TCONS_LINC_0000019[illegible]	exon_number "2"
lincRNA_337	Chromosome03	4759121	4759348	+	"TCONS_LINC_0000016[illegible]	exon_number "1"
lincRNA_337	Chromosome03	4760318	4762370	+	"TCONS_LINC_0000016[illegible]	exon_number "2"
lincRNA_338	Chromosome03	6479541	6479659	-	"TCONS_LINC_0000020[illegible]	exon_number "1"
lincRNA_338	Chromosome03	6480414	6481192	-	"TCONS_LINC_0000020[illegible]	exon_number "2"
lincRNA_338	Chromosome03	6481581	6481709	-	"TCONS_LINC_0000020[illegible]	exon_number "3"

lincRNA_338	Chromosome03	6482302	6482363	-	"TCONS_LINC_0000020!	exon_number "4"
lincRNA_338	Chromosome03	6482813	6482826	-	"TCONS_LINC_0000020!	exon_number "5"
lincRNA_338	Chromosome03	6480379	6481192	-	"TCONS_LINC_0000020(	exon_number "1"
lincRNA_338	Chromosome03	6481581	6481709	-	"TCONS_LINC_0000020(	exon_number "2"
lincRNA_338	Chromosome03	6482302	6482362	-	"TCONS_LINC_0000020(	exon_number "3"
lincRNA_338	Chromosome03	6482686	6482807	-	"TCONS_LINC_0000020(	exon_number "4"
lincRNA_339	Chromosome03	7105681	7105770	+	"TCONS_LINC_0000016!	exon_number "1"
lincRNA_339	Chromosome03	7106278	7108924	+	"TCONS_LINC_0000016!	exon_number "2"
lincRNA_339	Chromosome03	7111086	7111135	+	"TCONS_LINC_0000016!	exon_number "3"
lincRNA_339	Chromosome03	7105900	7105935	+	"TCONS_LINC_0000016!	exon_number "1"
lincRNA_339	Chromosome03	7106443	7109804	+	"TCONS_LINC_0000016!	exon_number "2"
lincRNA_339	Chromosome03	7112007	7112328	+	"TCONS_LINC_0000016!	exon_number "3"
lincRNA_34	Chromosome10	9551687	9553659	-	"TCONS_LINC_0000070(	exon_number "1"
lincRNA_34	Chromosome10	9554347	9555634	-	"TCONS_LINC_0000070(	exon_number "2"
lincRNA_340	Chromosome03	773378	773913	+	"TCONS_LINC_0000015(	exon_number "1"
lincRNA_340	Chromosome03	773378	773589	+	"TCONS_LINC_0000015'	exon_number "1"
lincRNA_340	Chromosome03	773957	774038	+	"TCONS_LINC_0000015'	exon_number "2"
lincRNA_340	Chromosome03	774129	774208	+	"TCONS_LINC_0000015'	exon_number "3"
lincRNA_340	Chromosome03	774333	774423	+	"TCONS_LINC_0000015'	exon_number "4"
lincRNA_340	Chromosome03	774504	774560	+	"TCONS_LINC_0000015'	exon_number "5"
lincRNA_340	Chromosome03	774934	775010	+	"TCONS_LINC_0000015'	exon_number "6"
lincRNA_340	Chromosome03	775102	775708	+	"TCONS_LINC_0000015'	exon_number "7"
lincRNA_340	Chromosome03	775829	775976	+	"TCONS_LINC_0000015'	exon_number "8"
lincRNA_340	Chromosome03	776612	776769	+	"TCONS_LINC_0000015'	exon_number "9"
lincRNA_340	Chromosome03	776923	776987	+	"TCONS_LINC_0000015'	exon_number "10"
lincRNA_340	Chromosome03	777075	778236	+	"TCONS_LINC_0000015'	exon_number "11"
lincRNA_340	Chromosome03	774565	775010	+	"TCONS_LINC_0000015!	exon_number "1"
lincRNA_340	Chromosome03	775102	775205	+	"TCONS_LINC_0000015!	exon_number "2"
lincRNA_340	Chromosome03	775612	775708	+	"TCONS_LINC_0000015!	exon_number "3"
lincRNA_340	Chromosome03	775829	775976	+	"TCONS_LINC_0000015!	exon_number "4"
lincRNA_340	Chromosome03	776612	776769	+	"TCONS_LINC_0000015!	exon_number "5"
lincRNA_340	Chromosome03	776923	778236	+	"TCONS_LINC_0000015!	exon_number "6"
lincRNA_341	Chromosome03	780142	780442	-	"TCONS_LINC_0000019(	exon_number "1"
lincRNA_341	Chromosome03	780552	782926	-	"TCONS_LINC_0000019(	exon_number "2"
lincRNA_342	Chromosome03	8396277	8398178	-	"TCONS_LINC_0000021	exon_number "1"
lincRNA_342	Chromosome03	8398498	8398734	-	"TCONS_LINC_0000021	exon_number "2"
lincRNA_343	Chromosome03	8424907	8425211	+	"TCONS_LINC_0000017:	exon_number "1"
lincRNA_343	Chromosome03	8428035	8428519	+	"TCONS_LINC_0000017:	exon_number "2"
lincRNA_343	Chromosome03	8428799	8428977	+	"TCONS_LINC_0000017:	exon_number "3"
lincRNA_343	Chromosome03	8424963	8425211	+	"TCONS_LINC_0000017<	exon_number "1"
lincRNA_343	Chromosome03	8428035	8428115	+	"TCONS_LINC_0000017<	exon_number "2"
lincRNA_343	Chromosome03	8428264	8428519	+	"TCONS_LINC_0000017<	exon_number "3"
lincRNA_343	Chromosome03	8429251	8429491	+	"TCONS_LINC_0000017<	exon_number "4"
lincRNA_344	Chromosome03	8634854	8634874	-	"TCONS_LINC_0000021:	exon_number "1"
lincRNA_344	Chromosome03	8641772	8646680	-	"TCONS_LINC_0000021:	exon_number "2"
lincRNA_344	Chromosome03	8635393	8635403	-	"TCONS_LINC_0000021<	exon_number "1"
lincRNA_344	Chromosome03	8642301	8644479	-	"TCONS_LINC_0000021<	exon_number "2"
lincRNA_345	Chromosome03	8664206	8664729	-	"TCONS_LINC_0000021!	exon_number "1"
lincRNA_345	Chromosome03	8669254	8669345	-	"TCONS_LINC_0000021!	exon_number "2"
lincRNA_346	Chromosome03	8915066	8915499	+	"TCONS_LINC_0000017(	exon_number "1"
lincRNA_346	Chromosome03	8939861	8939972	+	"TCONS_LINC_0000017(	exon_number "2"
lincRNA_347	Chromosome03	9263744	9263801	+	"TCONS_LINC_0000017'	exon_number "1"
lincRNA_347	Chromosome03	9307860	9308313	+	"TCONS_LINC_0000017'	exon_number "2"
lincRNA_348	Chromosome09	10271508	10271590	-	"TCONS_LINC_0000063	exon_number "1"
lincRNA_348	Chromosome09	10276642	10277063	-	"TCONS_LINC_0000063	exon_number "2"
lincRNA_349	Chromosome09	11331739	11332416	+	"TCONS_LINC_0000059'	exon_number "1"
lincRNA_349	Chromosome09	11336691	11336819	+	"TCONS_LINC_0000059'	exon_number "2"

lincRNA_35	Scaffold01189	38720	38961	+	"TCONS_LINC_0000132[illegible]	exon_number "1"
lincRNA_35	Scaffold01189	45829	45906	+	"TCONS_LINC_0000132[illegible]	exon_number "2"
lincRNA_350	Chromosome09	11337442	11337526	+	"TCONS_LINC_0000059[illegible]	exon_number "1"
lincRNA_350	Chromosome09	11337624	11337723	+	"TCONS_LINC_0000059[illegible]	exon_number "2"
lincRNA_350	Chromosome09	11339087	11339552	+	"TCONS_LINC_0000059[illegible]	exon_number "3"
lincRNA_350	Chromosome09	11341166	11341201	+	"TCONS_LINC_0000059[illegible]	exon_number "4"
lincRNA_350	Chromosome09	11341305	11342048	+	"TCONS_LINC_0000059[illegible]	exon_number "5"
lincRNA_351	Chromosome09	12043491	12043504	-	"TCONS_LINC_0000063[illegible]	exon_number "1"
lincRNA_351	Chromosome09	12049786	12050393	-	"TCONS_LINC_0000063[illegible]	exon_number "2"
lincRNA_352	Chromosome09	12462288	12462637	+	"TCONS_LINC_0000059[illegible]	exon_number "1"
lincRNA_352	Chromosome09	12463291	12463400	+	"TCONS_LINC_0000059[illegible]	exon_number "2"
lincRNA_352	Chromosome09	12464238	12464465	+	"TCONS_LINC_0000059[illegible]	exon_number "3"
lincRNA_352	Chromosome09	12471737	12472530	+	"TCONS_LINC_0000059[illegible]	exon_number "4"
lincRNA_352	Chromosome09	12462433	12462637	+	"TCONS_LINC_0000060[illegible]	exon_number "1"
lincRNA_352	Chromosome09	12463286	12464461	+	"TCONS_LINC_0000060[illegible]	exon_number "2"
lincRNA_352	Chromosome09	12471733	12472530	+	"TCONS_LINC_0000060[illegible]	exon_number "3"
lincRNA_352	Chromosome09	12462443	12462637	+	"TCONS_LINC_0000060[illegible]	exon_number "1"
lincRNA_352	Chromosome09	12463291	12464461	+	"TCONS_LINC_0000060[illegible]	exon_number "2"
lincRNA_352	Chromosome09	12471210	12472530	+	"TCONS_LINC_0000060[illegible]	exon_number "3"
lincRNA_353	Chromosome09	12620129	12620362	-	"TCONS_LINC_0000063[illegible]	exon_number "1"
lincRNA_353	Chromosome09	12622120	12622976	-	"TCONS_LINC_0000063[illegible]	exon_number "2"
lincRNA_353	Chromosome09	12620129	12623023	-	"TCONS_LINC_0000063[illegible]	exon_number "1"
lincRNA_354	Chromosome09	14202877	14203229	+	"TCONS_LINC_0000060[illegible]	exon_number "1"
lincRNA_354	Chromosome09	14204207	14204262	+	"TCONS_LINC_0000060[illegible]	exon_number "2"
lincRNA_354	Chromosome09	14204344	14204391	+	"TCONS_LINC_0000060[illegible]	exon_number "3"
lincRNA_354	Chromosome09	14204595	14205376	+	"TCONS_LINC_0000060[illegible]	exon_number "4"
lincRNA_354	Chromosome09	14203981	14204262	+	"TCONS_LINC_0000060[illegible]	exon_number "1"
lincRNA_354	Chromosome09	14204344	14204391	+	"TCONS_LINC_0000060[illegible]	exon_number "2"
lincRNA_354	Chromosome09	14204595	14205376	+	"TCONS_LINC_0000060[illegible]	exon_number "3"
lincRNA_355	Chromosome09	14477338	14478186	-	"TCONS_LINC_0000063[illegible]	exon_number "1"
lincRNA_355	Chromosome09	14479632	14479763	-	"TCONS_LINC_0000063[illegible]	exon_number "2"
lincRNA_355	Chromosome09	14477338	14478182	-	"TCONS_LINC_0000063[illegible]	exon_number "1"
lincRNA_355	Chromosome09	14479632	14480212	-	"TCONS_LINC_0000063[illegible]	exon_number "2"
lincRNA_356	Chromosome09	14586329	14586755	-	"TCONS_LINC_0000063[illegible]	exon_number "1"
lincRNA_356	Chromosome09	14589840	14589995	-	"TCONS_LINC_0000063[illegible]	exon_number "2"
lincRNA_356	Chromosome09	14590397	14590713	-	"TCONS_LINC_0000063[illegible]	exon_number "3"
lincRNA_357	Chromosome09	14592291	14592413	-	"TCONS_LINC_0000064[illegible]	exon_number "1"
lincRNA_357	Chromosome09	14592527	14594757	-	"TCONS_LINC_0000064[illegible]	exon_number "2"
lincRNA_358	Chromosome09	15566340	15566437	-	"TCONS_LINC_0000064[illegible]	exon_number "1"
lincRNA_358	Chromosome09	15568628	15569692	-	"TCONS_LINC_0000064[illegible]	exon_number "2"
lincRNA_359	Chromosome09	19420501	19420702	-	"TCONS_LINC_0000064[illegible]	exon_number "1"
lincRNA_359	Chromosome09	19424582	19425023	-	"TCONS_LINC_0000064[illegible]	exon_number "2"
lincRNA_36	Scaffold00460	337370	337746	+	"TCONS_LINC_0000126[illegible]	exon_number "1"
lincRNA_36	Scaffold00460	365626	365632	+	"TCONS_LINC_0000126[illegible]	exon_number "2"
lincRNA_360	Chromosome09	19624231	19624897	-	"TCONS_LINC_0000064[illegible]	exon_number "1"
lincRNA_360	Chromosome09	19624953	19625097	-	"TCONS_LINC_0000064[illegible]	exon_number "2"
lincRNA_360	Chromosome09	19626114	19626396	-	"TCONS_LINC_0000064[illegible]	exon_number "3"
lincRNA_360	Chromosome09	19626431	19626463	-	"TCONS_LINC_0000064[illegible]	exon_number "4"
lincRNA_360	Chromosome09	19625842	19626396	-	"TCONS_LINC_0000064[illegible]	exon_number "1"
lincRNA_360	Chromosome09	19626431	19626463	-	"TCONS_LINC_0000064[illegible]	exon_number "2"
lincRNA_361	Chromosome09	1985852	1986885	-	"TCONS_LINC_0000061[illegible]	exon_number "1"
lincRNA_361	Chromosome09	1987057	1988005	-	"TCONS_LINC_0000061[illegible]	exon_number "2"
lincRNA_362	Chromosome09	1991504	1991720	-	"TCONS_LINC_0000061[illegible]	exon_number "1"
lincRNA_362	Chromosome09	1991763	1992375	-	"TCONS_LINC_0000061[illegible]	exon_number "2"
lincRNA_362	Chromosome09	1993750	1993980	-	"TCONS_LINC_0000061[illegible]	exon_number "3"
lincRNA_363	Chromosome09	21733910	21733951	-	"TCONS_LINC_0000064[illegible]	exon_number "1"
lincRNA_363	Chromosome09	21735850	21737465	-	"TCONS_LINC_0000064[illegible]	exon_number "2"

lincRNA_364	Chromosome09	22797252	22797274	-	"TCONS_LINC_0000065[illegible]	exon_number "1"
lincRNA_364	Chromosome09	22799582	22800361	-	"TCONS_LINC_0000065[illegible]	exon_number "2"
lincRNA_365	Chromosome09	26611553	26611610	-	"TCONS_LINC_0000065[illegible]	exon_number "1"
lincRNA_365	Chromosome09	26618896	26620633	-	"TCONS_LINC_0000065[illegible]	exon_number "2"
lincRNA_366	Chromosome09	27273975	27275189	-	"TCONS_LINC_0000065[illegible]	exon_number "1"
lincRNA_366	Chromosome09	27275313	27275397	-	"TCONS_LINC_0000065[illegible]	exon_number "2"
lincRNA_366	Chromosome09	27275575	27275976	-	"TCONS_LINC_0000065[illegible]	exon_number "3"
lincRNA_367	Chromosome09	2761927	2762282	+	"TCONS_LINC_0000058[illegible]	exon_number "1"
lincRNA_367	Chromosome09	2769940	2770737	+	"TCONS_LINC_0000058[illegible]	exon_number "2"
lincRNA_368	Chromosome09	28119088	28119606	+	"TCONS_LINC_0000060[illegible]	exon_number "1"
lincRNA_368	Chromosome09	28119841	28119958	+	"TCONS_LINC_0000060[illegible]	exon_number "2"
lincRNA_368	Chromosome09	28120448	28120563	+	"TCONS_LINC_0000060[illegible]	exon_number "3"
lincRNA_368	Chromosome09	28121310	28121850	+	"TCONS_LINC_0000060[illegible]	exon_number "4"
lincRNA_368	Chromosome09	28123383	28123433	+	"TCONS_LINC_0000060[illegible]	exon_number "5"
lincRNA_368	Chromosome09	28121073	28121161	+	"TCONS_LINC_0000060[illegible]	exon_number "1"
lincRNA_368	Chromosome09	28121310	28121853	+	"TCONS_LINC_0000060[illegible]	exon_number "2"
lincRNA_368	Chromosome09	28122594	28122671	+	"TCONS_LINC_0000060[illegible]	exon_number "3"
lincRNA_368	Chromosome09	28124444	28124937	+	"TCONS_LINC_0000060[illegible]	exon_number "4"
lincRNA_368	Chromosome09	28122641	28122671	+	"TCONS_LINC_0000061[illegible]	exon_number "1"
lincRNA_368	Chromosome09	28124290	28124349	+	"TCONS_LINC_0000061[illegible]	exon_number "2"
lincRNA_368	Chromosome09	28124444	28124937	+	"TCONS_LINC_0000061[illegible]	exon_number "3"
lincRNA_369	Chromosome09	28443451	28443568	-	"TCONS_LINC_0000066[illegible]	exon_number "1"
lincRNA_369	Chromosome09	28443652	28445067	-	"TCONS_LINC_0000066[illegible]	exon_number "2"
lincRNA_369	Chromosome09	28443451	28443568	-	"TCONS_LINC_0000066[illegible]	exon_number "1"
lincRNA_369	Chromosome09	28443652	28444113	-	"TCONS_LINC_0000066[illegible]	exon_number "2"
lincRNA_369	Chromosome09	28450805	28451561	-	"TCONS_LINC_0000066[illegible]	exon_number "3"
lincRNA_369	Chromosome09	28448741	28448786	-	"TCONS_LINC_0000066[illegible]	exon_number "1"
lincRNA_369	Chromosome09	28448931	28450805	-	"TCONS_LINC_0000066[illegible]	exon_number "2"
lincRNA_37	Scaffold01371	90	286	+	"TCONS_LINC_0001133[illegible]	exon_number "1"
lincRNA_37	Scaffold01371	1182	3073	+	"TCONS_LINC_0001133[illegible]	exon_number "2"
lincRNA_37	Scaffold01371	3320	4164	+	"TCONS_LINC_0001133[illegible]	exon_number "3"
lincRNA_370	Chromosome09	413252	413649	+	"TCONS_LINC_0000057[illegible]	exon_number "1"
lincRNA_370	Chromosome09	419326	419332	+	"TCONS_LINC_0000057[illegible]	exon_number "2"
lincRNA_371	Chromosome09	5582370	5582982	+	"TCONS_LINC_0000058[illegible]	exon_number "1"
lincRNA_371	Chromosome09	5584881	5584918	+	"TCONS_LINC_0000058[illegible]	exon_number "2"
lincRNA_371	Chromosome09	5582399	5583367	+	"TCONS_LINC_0000058[illegible]	exon_number "1"
lincRNA_372	Chromosome09	6130933	6131069	-	"TCONS_LINC_0000062[illegible]	exon_number "1"
lincRNA_372	Chromosome09	6134505	6134685	-	"TCONS_LINC_0000062[illegible]	exon_number "2"
lincRNA_373	Chromosome09	6605159	6605946	-	"TCONS_LINC_0000062[illegible]	exon_number "1"
lincRNA_373	Chromosome09	6606453	6607339	-	"TCONS_LINC_0000062[illegible]	exon_number "2"
lincRNA_374	Chromosome09	7187474	7188128	-	"TCONS_LINC_0000062[illegible]	exon_number "1"
lincRNA_374	Chromosome09	7189030	7189101	-	"TCONS_LINC_0000062[illegible]	exon_number "2"
lincRNA_374	Chromosome09	7189485	7189494	-	"TCONS_LINC_0000062[illegible]	exon_number "3"
lincRNA_374	Chromosome09	7187474	7188126	-	"TCONS_LINC_0000062[illegible]	exon_number "1"
lincRNA_374	Chromosome09	7189485	7189644	-	"TCONS_LINC_0000062[illegible]	exon_number "2"
lincRNA_374	Chromosome09	7190145	7190179	-	"TCONS_LINC_0000062[illegible]	exon_number "3"
lincRNA_375	Chromosome09	81672	81783	-	"TCONS_LINC_0000061[illegible]	exon_number "1"
lincRNA_375	Chromosome09	81804	82178	-	"TCONS_LINC_0000061[illegible]	exon_number "2"
lincRNA_375	Chromosome09	90354	90659	-	"TCONS_LINC_0000061[illegible]	exon_number "3"
lincRNA_376	Chromosome09	8414260	8414317	+	"TCONS_LINC_0000059[illegible]	exon_number "1"
lincRNA_376	Chromosome09	8433752	8435826	+	"TCONS_LINC_0000059[illegible]	exon_number "2"
lincRNA_377	Chromosome09	9693419	9695450	+	"TCONS_LINC_0000059[illegible]	exon_number "1"
lincRNA_377	Chromosome09	9727786	9728134	+	"TCONS_LINC_0000059[illegible]	exon_number "2"
lincRNA_378	Scaffold01367	19519	22128	-	"TCONS_LINC_0001133[illegible]	exon_number "1"
lincRNA_378	Scaffold01367	24089	24395	-	"TCONS_LINC_0001133[illegible]	exon_number "2"
lincRNA_378	Scaffold01367	24496	24528	-	"TCONS_LINC_0001133[illegible]	exon_number "3"
lincRNA_379	Scaffold01404	12711	12990	-	"TCONS_LINC_0001134[illegible]	exon_number "1"

lincRNA_379	Scaffold01404	14771	14892	-	"TCONS_LINC_00001340	exon_number "2"
lincRNA_379	Scaffold01404	16935	17925	-	"TCONS_LINC_00001340	exon_number "3"
lincRNA_38	Scaffold00600	176287	176413	+	"TCONS_LINC_00001273	exon_number "1"
lincRNA_38	Scaffold00600	188739	188971	+	"TCONS_LINC_00001273	exon_number "2"
lincRNA_380	Scaffold01045	42233	42267	-	"TCONS_LINC_00001314	exon_number "1"
lincRNA_380	Scaffold01045	42309	43739	-	"TCONS_LINC_00001314	exon_number "2"
lincRNA_380	Scaffold01045	45540	45963	-	"TCONS_LINC_00001314	exon_number "3"
lincRNA_381	Chromosome05	11726601	11727102	-	"TCONS_LINC_00000340	exon_number "1"
lincRNA_381	Chromosome05	11735963	11736665	-	"TCONS_LINC_00000340	exon_number "2"
lincRNA_382	Chromosome05	11794103	11796382	+	"TCONS_LINC_00000294	exon_number "1"
lincRNA_382	Chromosome05	11796424	11796448	+	"TCONS_LINC_00000294	exon_number "2"
lincRNA_383	Chromosome05	11920689	11923029	+	"TCONS_LINC_00000296	exon_number "1"
lincRNA_383	Chromosome05	11923229	11923390	+	"TCONS_LINC_00000296	exon_number "2"
lincRNA_383	Chromosome05	11923488	11926950	+	"TCONS_LINC_00000296	exon_number "3"
lincRNA_384	Chromosome05	1351845	1351862	-	"TCONS_LINC_00000321	exon_number "1"
lincRNA_384	Chromosome05	1351900	1353733	-	"TCONS_LINC_00000321	exon_number "2"
lincRNA_384	Chromosome05	1354007	1354449	-	"TCONS_LINC_00000321	exon_number "3"
lincRNA_385	Chromosome05	14476672	14477628	+	"TCONS_LINC_00000299	exon_number "1"
lincRNA_385	Chromosome05	14518005	14518018	+	"TCONS_LINC_00000299	exon_number "2"
lincRNA_386	Chromosome05	15371301	15371673	-	"TCONS_LINC_00000342	exon_number "1"
lincRNA_386	Chromosome05	15378527	15378651	-	"TCONS_LINC_00000342	exon_number "2"
lincRNA_387	Chromosome05	15668813	15668847	-	"TCONS_LINC_00000345	exon_number "1"
lincRNA_387	Chromosome05	15676340	15676925	-	"TCONS_LINC_00000345	exon_number "2"
lincRNA_388	Chromosome05	16343587	16345291	+	"TCONS_LINC_00000301	exon_number "1"
lincRNA_388	Chromosome05	16352985	16353374	+	"TCONS_LINC_00000301	exon_number "2"
lincRNA_389	Chromosome05	16422715	16422726	+	"TCONS_LINC_00000302	exon_number "1"
lincRNA_389	Chromosome05	16466572	16466816	+	"TCONS_LINC_00000302	exon_number "2"
lincRNA_39	Chromosome16	1147643	1148041	+	"TCONS_LINC_00001077	exon_number "1"
lincRNA_39	Chromosome16	1148505	1149193	+	"TCONS_LINC_00001077	exon_number "2"
lincRNA_39	Chromosome16	1151346	1152655	+	"TCONS_LINC_00001077	exon_number "3"
lincRNA_390	Chromosome05	17729450	17730753	+	"TCONS_LINC_00000306	exon_number "1"
lincRNA_390	Chromosome05	17759951	17759967	+	"TCONS_LINC_00000306	exon_number "2"
lincRNA_391	Chromosome05	18345278	18347212	+	"TCONS_LINC_00000307	exon_number "1"
lincRNA_391	Chromosome05	18349351	18349361	+	"TCONS_LINC_00000307	exon_number "2"
lincRNA_392	Chromosome05	2110140	2110646	-	"TCONS_LINC_00000323	exon_number "1"
lincRNA_392	Chromosome05	2112177	2112828	-	"TCONS_LINC_00000323	exon_number "2"
lincRNA_393	Chromosome05	22337454	22338243	-	"TCONS_LINC_00000348	exon_number "1"
lincRNA_393	Chromosome05	22340873	22340938	-	"TCONS_LINC_00000348	exon_number "2"
lincRNA_394	Chromosome05	23994731	23994741	-	"TCONS_LINC_00000349	exon_number "1"
lincRNA_394	Chromosome05	23995376	23997056	-	"TCONS_LINC_00000349	exon_number "2"
lincRNA_395	Chromosome05	25170926	25171957	-	"TCONS_LINC_00000350	exon_number "1"
lincRNA_395	Chromosome05	25172033	25172118	-	"TCONS_LINC_00000350	exon_number "2"
lincRNA_395	Chromosome05	25173096	25173143	-	"TCONS_LINC_00000350	exon_number "3"
lincRNA_395	Chromosome05	25173259	25173291	-	"TCONS_LINC_00000350	exon_number "4"
lincRNA_395	Chromosome05	25173841	25174194	-	"TCONS_LINC_00000350	exon_number "5"
lincRNA_396	Chromosome05	25651906	25652209	+	"TCONS_LINC_00000312	exon_number "1"
lincRNA_396	Chromosome05	25652859	25653451	+	"TCONS_LINC_00000312	exon_number "2"
lincRNA_396	Chromosome05	25654952	25654989	+	"TCONS_LINC_00000312	exon_number "3"
lincRNA_397	Chromosome05	25917520	25917906	+	"TCONS_LINC_00000313	exon_number "1"
lincRNA_397	Chromosome05	25919691	25919957	+	"TCONS_LINC_00000313	exon_number "2"
lincRNA_398	Chromosome05	26898456	26899087	-	"TCONS_LINC_00000351	exon_number "1"
lincRNA_398	Chromosome05	26899855	26900009	-	"TCONS_LINC_00000351	exon_number "2"
lincRNA_398	Chromosome05	26900144	26900577	-	"TCONS_LINC_00000351	exon_number "3"
lincRNA_399	Chromosome05	27394465	27394708	+	"TCONS_LINC_00000317	exon_number "1"
lincRNA_399	Chromosome05	27396151	27396314	+	"TCONS_LINC_00000317	exon_number "2"
lincRNA_399	Chromosome05	27396711	27396783	+	"TCONS_LINC_00000317	exon_number "3"
lincRNA_399	Chromosome05	27394465	27394705	+	"TCONS_LINC_00000318	exon_number "1"

lincRNA_399	Chromosome05	27396154	27396314	+	"TCONS_LINC_0000031[illegible]	exon_number "2"
lincRNA_399	Chromosome05	27397240	27399203	+	"TCONS_LINC_0000031[illegible]	exon_number "3"
lincRNA_4	Chromosome10	10320409	10320614	-	"TCONS_LINC_0000070[illegible]	exon_number "1"
lincRNA_4	Chromosome10	10332130	10332170	-	"TCONS_LINC_0000070[illegible]	exon_number "2"
lincRNA_4	Chromosome10	10332964	10333303	-	"TCONS_LINC_0000070[illegible]	exon_number "3"
lincRNA_4	Chromosome10	10326086	10326089	-	"TCONS_LINC_0000071[illegible]	exon_number "1"
lincRNA_4	Chromosome10	10332130	10332170	-	"TCONS_LINC_0000071[illegible]	exon_number "2"
lincRNA_4	Chromosome10	10332964	10333292	-	"TCONS_LINC_0000071[illegible]	exon_number "3"
lincRNA_4	Chromosome10	10332289	10333284	-	"TCONS_LINC_0000071[illegible]	exon_number "1"
lincRNA_4	Chromosome10	10333387	10333514	-	"TCONS_LINC_0000071[illegible]	exon_number "2"
lincRNA_4	Chromosome10	10333631	10333707	-	"TCONS_LINC_0000071[illegible]	exon_number "3"
lincRNA_4	Chromosome10	10334352	10334808	-	"TCONS_LINC_0000071[illegible]	exon_number "4"
lincRNA_40	Chromosome16	16078068	16078085	-	"TCONS_LINC_0000112[illegible]	exon_number "1"
lincRNA_40	Chromosome16	16080629	16081666	-	"TCONS_LINC_0000112[illegible]	exon_number "2"
lincRNA_400	Chromosome05	27702806	27703019	-	"TCONS_LINC_0000035[illegible]	exon_number "1"
lincRNA_400	Chromosome05	27706031	27706902	-	"TCONS_LINC_0000035[illegible]	exon_number "2"
lincRNA_400	Chromosome05	27705094	27706092	-	"TCONS_LINC_0000035[illegible]	exon_number "1"
lincRNA_401	Chromosome05	3611165	3612586	-	"TCONS_LINC_0000032[illegible]	exon_number "1"
lincRNA_401	Chromosome05	3614033	3614157	-	"TCONS_LINC_0000032[illegible]	exon_number "2"
lincRNA_401	Chromosome05	3614713	3615318	-	"TCONS_LINC_0000032[illegible]	exon_number "3"
lincRNA_401	Chromosome05	3615685	3615900	-	"TCONS_LINC_0000032[illegible]	exon_number "4"
lincRNA_401	Chromosome05	3615995	3616027	-	"TCONS_LINC_0000032[illegible]	exon_number "5"
lincRNA_401	Chromosome05	3611165	3611542	-	"TCONS_LINC_0000032[illegible]	exon_number "1"
lincRNA_401	Chromosome05	3612512	3612586	-	"TCONS_LINC_0000032[illegible]	exon_number "2"
lincRNA_401	Chromosome05	3614033	3614157	-	"TCONS_LINC_0000032[illegible]	exon_number "3"
lincRNA_401	Chromosome05	3614988	3615318	-	"TCONS_LINC_0000032[illegible]	exon_number "4"
lincRNA_401	Chromosome05	3615685	3616288	-	"TCONS_LINC_0000032[illegible]	exon_number "5"
lincRNA_401	Chromosome05	3611165	3611542	-	"TCONS_LINC_0000032[illegible]	exon_number "1"
lincRNA_401	Chromosome05	3612512	3612586	-	"TCONS_LINC_0000032[illegible]	exon_number "2"
lincRNA_401	Chromosome05	3614033	3614157	-	"TCONS_LINC_0000032[illegible]	exon_number "3"
lincRNA_401	Chromosome05	3614713	3614831	-	"TCONS_LINC_0000032[illegible]	exon_number "4"
lincRNA_401	Chromosome05	3614988	3615318	-	"TCONS_LINC_0000032[illegible]	exon_number "5"
lincRNA_401	Chromosome05	3615685	3615900	-	"TCONS_LINC_0000032[illegible]	exon_number "6"
lincRNA_401	Chromosome05	3615995	3616034	-	"TCONS_LINC_0000032[illegible]	exon_number "7"
lincRNA_401	Chromosome05	3616341	3616394	-	"TCONS_LINC_0000032[illegible]	exon_number "8"
lincRNA_401	Chromosome05	3613752	3614157	-	"TCONS_LINC_0000032[illegible]	exon_number "1"
lincRNA_401	Chromosome05	3614988	3615318	-	"TCONS_LINC_0000032[illegible]	exon_number "2"
lincRNA_401	Chromosome05	3615685	3616027	-	"TCONS_LINC_0000032[illegible]	exon_number "3"
lincRNA_402	Chromosome05	4391453	4392285	+	"TCONS_LINC_0000028[illegible]	exon_number "1"
lincRNA_402	Chromosome05	4393676	4395686	+	"TCONS_LINC_0000028[illegible]	exon_number "2"
lincRNA_402	Chromosome05	4396280	4396412	+	"TCONS_LINC_0000028[illegible]	exon_number "3"
lincRNA_403	Chromosome05	4879921	4880158	+	"TCONS_LINC_0000028[illegible]	exon_number "1"
lincRNA_403	Chromosome05	4886617	4886734	+	"TCONS_LINC_0000028[illegible]	exon_number "2"
lincRNA_404	Chromosome05	5350191	5350383	-	"TCONS_LINC_0000033[illegible]	exon_number "1"
lincRNA_404	Chromosome05	5352665	5353150	-	"TCONS_LINC_0000033[illegible]	exon_number "2"
lincRNA_405	Chromosome05	6659643	6659820	-	"TCONS_LINC_0000033[illegible]	exon_number "1"
lincRNA_405	Chromosome05	6659919	6659976	-	"TCONS_LINC_0000033[illegible]	exon_number "2"
lincRNA_405	Chromosome05	6665832	6665911	-	"TCONS_LINC_0000033[illegible]	exon_number "3"
lincRNA_406	Chromosome05	8348731	8348831	-	"TCONS_LINC_0000033[illegible]	exon_number "1"
lincRNA_406	Chromosome05	8349032	8349212	-	"TCONS_LINC_0000033[illegible]	exon_number "2"
lincRNA_406	Chromosome05	8350280	8350353	-	"TCONS_LINC_0000033[illegible]	exon_number "3"
lincRNA_406	Chromosome05	8350459	8350550	-	"TCONS_LINC_0000033[illegible]	exon_number "4"
lincRNA_406	Chromosome05	8350962	8352965	-	"TCONS_LINC_0000033[illegible]	exon_number "5"
lincRNA_407	Chromosome04	10799991	10800056	+	"TCONS_LINC_0000023[illegible]	exon_number "1"
lincRNA_407	Chromosome04	10802069	10802700	+	"TCONS_LINC_0000023[illegible]	exon_number "2"
lincRNA_408	Chromosome04	11214069	11214830	+	"TCONS_LINC_0000024[illegible]	exon_number "1"
lincRNA_408	Chromosome04	11222884	11223039	+	"TCONS_LINC_0000024[illegible]	exon_number "2"

lincRNA_409	Chromosome04	1213246	1213286	-	"TCONS_LINC_00000258	exon_number "1"
lincRNA_409	Chromosome04	1217763	1217972	-	"TCONS_LINC_00000258	exon_number "2"
lincRNA_41	Chromosome16	16338403	16338510	+	"TCONS_LINC_00001083	exon_number "1"
lincRNA_41	Chromosome16	16339223	16339312	+	"TCONS_LINC_00001083	exon_number "2"
lincRNA_41	Chromosome16	16340097	16342992	+	"TCONS_LINC_00001083	exon_number "3"
lincRNA_41	Chromosome16	16339585	16339709	+	"TCONS_LINC_00001084	exon_number "1"
lincRNA_41	Chromosome16	16339767	16342992	+	"TCONS_LINC_00001084	exon_number "2"
lincRNA_410	Chromosome04	12662949	12662965	-	"TCONS_LINC_00000265	exon_number "1"
lincRNA_410	Chromosome04	12690138	12690747	-	"TCONS_LINC_00000265	exon_number "2"
lincRNA_411	Chromosome04	12942331	12943406	+	"TCONS_LINC_00000242	exon_number "1"
lincRNA_411	Chromosome04	12970723	12971124	+	"TCONS_LINC_00000242	exon_number "2"
lincRNA_412	Chromosome04	12971650	12972046	+	"TCONS_LINC_00000243	exon_number "1"
lincRNA_412	Chromosome04	12974316	12974375	+	"TCONS_LINC_00000243	exon_number "2"
lincRNA_413	Chromosome04	13131926	13132330	-	"TCONS_LINC_00000266	exon_number "1"
lincRNA_413	Chromosome04	13134214	13134472	-	"TCONS_LINC_00000266	exon_number "2"
lincRNA_413	Chromosome04	13143376	13143701	-	"TCONS_LINC_00000266	exon_number "3"
lincRNA_414	Chromosome04	1529961	1530548	-	"TCONS_LINC_00000259	exon_number "1"
lincRNA_414	Chromosome04	1532180	1532403	-	"TCONS_LINC_00000259	exon_number "2"
lincRNA_414	Chromosome04	1529961	1531925	-	"TCONS_LINC_00000260	exon_number "1"
lincRNA_414	Chromosome04	1531978	1532231	-	"TCONS_LINC_00000260	exon_number "2"
lincRNA_414	Chromosome04	1532591	1533066	-	"TCONS_LINC_00000260	exon_number "3"
lincRNA_414	Chromosome04	1533162	1533725	-	"TCONS_LINC_00000260	exon_number "4"
lincRNA_414	Chromosome04	1532573	1533066	-	"TCONS_LINC_00000261	exon_number "1"
lincRNA_414	Chromosome04	1533162	1533363	-	"TCONS_LINC_00000261	exon_number "2"
lincRNA_414	Chromosome04	1533920	1534278	-	"TCONS_LINC_00000261	exon_number "3"
lincRNA_415	Chromosome04	16013302	16013349	-	"TCONS_LINC_00000270	exon_number "1"
lincRNA_415	Chromosome04	16020920	16024263	-	"TCONS_LINC_00000270	exon_number "2"
lincRNA_416	Chromosome04	18326843	18330522	+	"TCONS_LINC_00000244	exon_number "1"
lincRNA_416	Chromosome04	18328835	18329213	+	"TCONS_LINC_00000245	exon_number "1"
lincRNA_416	Chromosome04	18336493	18336914	+	"TCONS_LINC_00000245	exon_number "2"
lincRNA_417	Chromosome04	18717980	18719288	+	"TCONS_LINC_00000246	exon_number "1"
lincRNA_417	Chromosome04	18719794	18720015	+	"TCONS_LINC_00000246	exon_number "2"
lincRNA_418	Chromosome04	22606193	22609547	-	"TCONS_LINC_00000278	exon_number "1"
lincRNA_418	Chromosome04	22610024	22610126	-	"TCONS_LINC_00000278	exon_number "2"
lincRNA_418	Chromosome04	22606193	22610485	-	"TCONS_LINC_00000279	exon_number "1"
lincRNA_418	Chromosome04	22610562	22610583	-	"TCONS_LINC_00000279	exon_number "2"
lincRNA_419	Chromosome04	25523115	25523118	-	"TCONS_LINC_00000280	exon_number "1"
lincRNA_419	Chromosome04	25523322	25523392	-	"TCONS_LINC_00000280	exon_number "2"
lincRNA_419	Chromosome04	25525043	25525430	-	"TCONS_LINC_00000280	exon_number "3"
lincRNA_42	Chromosome16	17005384	17005395	-	"TCONS_LINC_00001126	exon_number "1"
lincRNA_42	Chromosome16	17011739	17012196	-	"TCONS_LINC_00001126	exon_number "2"
lincRNA_420	Chromosome04	27545765	27545854	-	"TCONS_LINC_00000285	exon_number "1"
lincRNA_420	Chromosome04	27545952	27546024	-	"TCONS_LINC_00000285	exon_number "2"
lincRNA_420	Chromosome04	27546406	27546510	-	"TCONS_LINC_00000285	exon_number "3"
lincRNA_420	Chromosome04	27547136	27548382	-	"TCONS_LINC_00000285	exon_number "4"
lincRNA_421	Chromosome04	28445682	28447491	+	"TCONS_LINC_00000255	exon_number "1"
lincRNA_421	Chromosome04	28446728	28447036	+	"TCONS_LINC_00000256	exon_number "1"
lincRNA_421	Chromosome04	28454136	28456821	+	"TCONS_LINC_00000256	exon_number "2"
lincRNA_422	Chromosome04	3477396	3477518	+	"TCONS_LINC_00000234	exon_number "1"
lincRNA_422	Chromosome04	3477618	3477801	+	"TCONS_LINC_00000234	exon_number "2"
lincRNA_422	Chromosome04	3479846	3480886	+	"TCONS_LINC_00000234	exon_number "3"
lincRNA_423	Chromosome04	7388431	7390017	-	"TCONS_LINC_00000263	exon_number "1"
lincRNA_423	Chromosome04	7392069	7392388	-	"TCONS_LINC_00000263	exon_number "2"
lincRNA_424	Scaffold00584	51480	51541	-	"TCONS_LINC_00001271	exon_number "1"
lincRNA_424	Scaffold00584	54213	54559	-	"TCONS_LINC_00001271	exon_number "2"
lincRNA_425	Scaffold00259	146498	146559	-	"TCONS_LINC_00001251	exon_number "1"
lincRNA_425	Scaffold00259	147569	147671	-	"TCONS_LINC_00001251	exon_number "2"

lincRNA_425	Scaffold00259	147765	147806	-	"TCONS_LINC_00001251	exon_number "3"
lincRNA_425	Scaffold00259	148585	148606	-	"TCONS_LINC_00001251	exon_number "4"
lincRNA_425	Scaffold00259	146830	148699	-	"TCONS_LINC_00001252	exon_number "1"
lincRNA_425	Scaffold00259	149021	149066	-	"TCONS_LINC_00001252	exon_number "2"
lincRNA_425	Scaffold00259	149830	150187	-	"TCONS_LINC_00001252	exon_number "3"
lincRNA_426	Scaffold00755	28830	29062	+	"TCONS_LINC_00001283	exon_number "1"
lincRNA_426	Scaffold00755	29089	29169	+	"TCONS_LINC_00001283	exon_number "2"
lincRNA_426	Scaffold00755	73002	73062	+	"TCONS_LINC_00001283	exon_number "3"
lincRNA_427	Scaffold01336	14759	16566	-	"TCONS_LINC_00001332	exon_number "1"
lincRNA_427	Scaffold01336	14759	15358	-	"TCONS_LINC_00001333	exon_number "1"
lincRNA_427	Scaffold01336	16760	18204	-	"TCONS_LINC_00001333	exon_number "2"
lincRNA_428	Scaffold00997	55574	56815	+	"TCONS_LINC_00001310	exon_number "1"
lincRNA_428	Scaffold00997	79101	80043	+	"TCONS_LINC_00001310	exon_number "2"
lincRNA_428	Scaffold00997	77387	77743	+	"TCONS_LINC_00001311	exon_number "1"
lincRNA_428	Scaffold00997	78721	78901	+	"TCONS_LINC_00001311	exon_number "2"
lincRNA_428	Scaffold00997	79268	79335	+	"TCONS_LINC_00001311	exon_number "3"
lincRNA_428	Scaffold00997	79464	80043	+	"TCONS_LINC_00001311	exon_number "4"
lincRNA_429	Chromosome08	11749192	11749844	+	"TCONS_LINC_00000515	exon_number "1"
lincRNA_429	Chromosome08	11749950	11750030	+	"TCONS_LINC_00000515	exon_number "2"
lincRNA_429	Chromosome08	11761938	11761987	+	"TCONS_LINC_00000515	exon_number "3"
lincRNA_43	Chromosome16	18647664	18647792	+	"TCONS_LINC_00001087	exon_number "1"
lincRNA_43	Chromosome16	18737316	18737429	+	"TCONS_LINC_00001087	exon_number "2"
lincRNA_430	Chromosome08	1382037	1382714	+	"TCONS_LINC_00000506	exon_number "1"
lincRNA_430	Chromosome08	1385514	1385562	+	"TCONS_LINC_00000506	exon_number "2"
lincRNA_430	Chromosome08	1382037	1382724	+	"TCONS_LINC_00000507	exon_number "1"
lincRNA_430	Chromosome08	1385590	1387413	+	"TCONS_LINC_00000507	exon_number "2"
lincRNA_431	Chromosome08	17971707	17972639	+	"TCONS_LINC_00000518	exon_number "1"
lincRNA_431	Chromosome08	18040812	18040819	+	"TCONS_LINC_00000518	exon_number "2"
lincRNA_432	Chromosome08	18552311	18552680	-	"TCONS_LINC_00000549	exon_number "1"
lincRNA_432	Chromosome08	18554439	18554723	-	"TCONS_LINC_00000549	exon_number "2"
lincRNA_432	Chromosome08	18554800	18554924	-	"TCONS_LINC_00000549	exon_number "3"
lincRNA_432	Chromosome08	18554994	18555489	-	"TCONS_LINC_00000549	exon_number "4"
lincRNA_432	Chromosome08	18562555	18562715	-	"TCONS_LINC_00000549	exon_number "5"
lincRNA_432	Chromosome08	18553466	18554322	-	"TCONS_LINC_00000550	exon_number "1"
lincRNA_432	Chromosome08	18554439	18554723	-	"TCONS_LINC_00000550	exon_number "2"
lincRNA_432	Chromosome08	18554813	18554924	-	"TCONS_LINC_00000550	exon_number "3"
lincRNA_432	Chromosome08	18562166	18562175	-	"TCONS_LINC_00000550	exon_number "4"
lincRNA_433	Chromosome08	20829798	20829806	-	"TCONS_LINC_00000552	exon_number "1"
lincRNA_433	Chromosome08	20875861	20876261	-	"TCONS_LINC_00000552	exon_number "2"
lincRNA_434	Chromosome08	23915086	23915110	-	"TCONS_LINC_00000553	exon_number "1"
lincRNA_434	Chromosome08	23915566	23915637	-	"TCONS_LINC_00000553	exon_number "2"
lincRNA_434	Chromosome08	23923157	23923228	-	"TCONS_LINC_00000553	exon_number "3"
lincRNA_434	Chromosome08	23923836	23927556	-	"TCONS_LINC_00000553	exon_number "4"
lincRNA_434	Chromosome08	23922527	23922656	-	"TCONS_LINC_00000554	exon_number "1"
lincRNA_434	Chromosome08	23923157	23923228	-	"TCONS_LINC_00000554	exon_number "2"
lincRNA_434	Chromosome08	23923836	23923935	-	"TCONS_LINC_00000554	exon_number "3"
lincRNA_434	Chromosome08	23924020	23925720	-	"TCONS_LINC_00000554	exon_number "4"
lincRNA_434	Chromosome08	23922729	23923352	-	"TCONS_LINC_00000555	exon_number "1"
lincRNA_434	Chromosome08	23925873	23926694	-	"TCONS_LINC_00000556	exon_number "1"
lincRNA_434	Chromosome08	23930905	23931276	-	"TCONS_LINC_00000556	exon_number "2"
lincRNA_435	Chromosome08	266658	267071	+	"TCONS_LINC_00000502	exon_number "1"
lincRNA_435	Chromosome08	267176	267330	+	"TCONS_LINC_00000502	exon_number "2"
lincRNA_435	Chromosome08	267453	267523	+	"TCONS_LINC_00000502	exon_number "3"
lincRNA_435	Chromosome08	268705	268823	+	"TCONS_LINC_00000502	exon_number "4"
lincRNA_435	Chromosome08	266677	267071	+	"TCONS_LINC_00000503	exon_number "1"
lincRNA_435	Chromosome08	267176	268823	+	"TCONS_LINC_00000503	exon_number "2"
lincRNA_436	Chromosome08	27818373	27818696	+	"TCONS_LINC_00000522	exon_number "1"

lincRNA_436	Chromosome08	27831387	27831613	+	"TCONS_LINC_0000052[illegible]	exon_number "2"
lincRNA_437	Chromosome08	29455420	29455742	+	"TCONS_LINC_0000052[illegible]	exon_number "1"
lincRNA_437	Chromosome08	29456098	29456175	+	"TCONS_LINC_0000052[illegible]	exon_number "2"
lincRNA_437	Chromosome08	29457177	29457286	+	"TCONS_LINC_0000052[illegible]	exon_number "3"
lincRNA_437	Chromosome08	29457442	29457528	+	"TCONS_LINC_0000052[illegible]	exon_number "4"
lincRNA_437	Chromosome08	29457673	29457729	+	"TCONS_LINC_0000052[illegible]	exon_number "5"
lincRNA_437	Chromosome08	29457843	29458035	+	"TCONS_LINC_0000052[illegible]	exon_number "6"
lincRNA_438	Chromosome08	2963596	2964412	-	"TCONS_LINC_0000053[illegible]	exon_number "1"
lincRNA_438	Chromosome08	2965971	2966032	-	"TCONS_LINC_0000053[illegible]	exon_number "2"
lincRNA_438	Chromosome08	2966175	2966378	-	"TCONS_LINC_0000053[illegible]	exon_number "3"
lincRNA_439	Chromosome08	30096435	30096453	+	"TCONS_LINC_0000052[illegible]	exon_number "1"
lincRNA_439	Chromosome08	30099258	30100384	+	"TCONS_LINC_0000052[illegible]	exon_number "2"
lincRNA_44	Chromosome16	1906991	1907243	-	"TCONS_LINC_0000111[illegible]	exon_number "1"
lincRNA_44	Chromosome16	1907650	1909190	-	"TCONS_LINC_0000111[illegible]	exon_number "2"
lincRNA_440	Chromosome08	3037846	3040048	-	"TCONS_LINC_0000053[illegible]	exon_number "1"
lincRNA_440	Chromosome08	3037846	3039451	-	"TCONS_LINC_0000053[illegible]	exon_number "1"
lincRNA_440	Chromosome08	3041311	3041539	-	"TCONS_LINC_0000053[illegible]	exon_number "2"
lincRNA_441	Chromosome08	30956037	30957189	-	"TCONS_LINC_0000056[illegible]	exon_number "1"
lincRNA_441	Chromosome08	30958052	30958672	-	"TCONS_LINC_0000056[illegible]	exon_number "2"
lincRNA_441	Chromosome08	30956037	30957186	-	"TCONS_LINC_0000056[illegible]	exon_number "1"
lincRNA_441	Chromosome08	30958049	30958346	-	"TCONS_LINC_0000056[illegible]	exon_number "2"
lincRNA_441	Chromosome08	30958905	30958931	-	"TCONS_LINC_0000056[illegible]	exon_number "3"
lincRNA_442	Chromosome08	31107540	31108404	-	"TCONS_LINC_0000056[illegible]	exon_number "1"
lincRNA_442	Chromosome08	31108561	31108853	-	"TCONS_LINC_0000056[illegible]	exon_number "2"
lincRNA_442	Chromosome08	31109662	31111434	-	"TCONS_LINC_0000056[illegible]	exon_number "3"
lincRNA_442	Chromosome08	31109396	31109692	-	"TCONS_LINC_0000056[illegible]	exon_number "1"
lincRNA_442	Chromosome08	31109714	31111089	-	"TCONS_LINC_0000056[illegible]	exon_number "2"
lincRNA_443	Chromosome08	31933469	31933871	-	"TCONS_LINC_0000057[illegible]	exon_number "1"
lincRNA_443	Chromosome08	31935450	31935729	-	"TCONS_LINC_0000057[illegible]	exon_number "2"
lincRNA_444	Chromosome08	32727487	32727519	+	"TCONS_LINC_0000053[illegible]	exon_number "1"
lincRNA_444	Chromosome08	32728836	32731120	+	"TCONS_LINC_0000053[illegible]	exon_number "2"
lincRNA_445	Chromosome08	32743349	32743408	+	"TCONS_LINC_0000053[illegible]	exon_number "1"
lincRNA_445	Chromosome08	32743851	32746051	+	"TCONS_LINC_0000053[illegible]	exon_number "2"
lincRNA_446	Chromosome08	33216800	33216979	-	"TCONS_LINC_0000057[illegible]	exon_number "1"
lincRNA_446	Chromosome08	33223235	33223497	-	"TCONS_LINC_0000057[illegible]	exon_number "2"
lincRNA_446	Chromosome08	33228806	33228868	-	"TCONS_LINC_0000057[illegible]	exon_number "3"
lincRNA_446	Chromosome08	33229005	33229277	-	"TCONS_LINC_0000057[illegible]	exon_number "4"
lincRNA_446	Chromosome08	33216980	33217336	-	"TCONS_LINC_0000057[illegible]	exon_number "1"
lincRNA_446	Chromosome08	33222840	33223497	-	"TCONS_LINC_0000057[illegible]	exon_number "2"
lincRNA_446	Chromosome08	33223682	33224858	-	"TCONS_LINC_0000057[illegible]	exon_number "3"
lincRNA_447	Chromosome08	4585331	4585886	-	"TCONS_LINC_0000053[illegible]	exon_number "1"
lincRNA_447	Chromosome08	4586016	4586102	-	"TCONS_LINC_0000053[illegible]	exon_number "2"
lincRNA_447	Chromosome08	4586283	4586375	-	"TCONS_LINC_0000053[illegible]	exon_number "3"
lincRNA_447	Chromosome08	4591621	4591707	-	"TCONS_LINC_0000053[illegible]	exon_number "4"
lincRNA_447	Chromosome08	4596548	4596776	-	"TCONS_LINC_0000053[illegible]	exon_number "5"
lincRNA_447	Chromosome08	4591691	4591952	-	"TCONS_LINC_0000053[illegible]	exon_number "1"
lincRNA_448	Chromosome08	5435036	5435049	+	"TCONS_LINC_0000051[illegible]	exon_number "1"
lincRNA_448	Chromosome08	5435209	5436407	+	"TCONS_LINC_0000051[illegible]	exon_number "2"
lincRNA_448	Chromosome08	5436501	5437347	+	"TCONS_LINC_0000051[illegible]	exon_number "3"
lincRNA_449	Chromosome08	625046	625072	+	"TCONS_LINC_0000050[illegible]	exon_number "1"
lincRNA_449	Chromosome08	625154	627640	+	"TCONS_LINC_0000050[illegible]	exon_number "2"
lincRNA_45	Chromosome16	19170893	19171352	-	"TCONS_LINC_0000113[illegible]	exon_number "1"
lincRNA_45	Chromosome16	19178563	19178944	-	"TCONS_LINC_0000113[illegible]	exon_number "2"
lincRNA_450	Chromosome08	7803698	7804012	+	"TCONS_LINC_0000051[illegible]	exon_number "1"
lincRNA_450	Chromosome08	7805444	7805631	+	"TCONS_LINC_0000051[illegible]	exon_number "2"
lincRNA_450	Chromosome08	7805719	7805779	+	"TCONS_LINC_0000051[illegible]	exon_number "3"
lincRNA_450	Chromosome08	7806119	7806297	+	"TCONS_LINC_0000051[illegible]	exon_number "4"

lincRNA_450	Chromosome08	7803698	7804012	+	"TCONS_LINC_0000051	exon_number "1"
lincRNA_450	Chromosome08	7805444	7805631	+	"TCONS_LINC_0000051	exon_number "2"
lincRNA_450	Chromosome08	7805719	7805779	+	"TCONS_LINC_0000051	exon_number "3"
lincRNA_450	Chromosome08	7807335	7807415	+	"TCONS_LINC_0000051	exon_number "4"
lincRNA_451	Chromosome08	8069321	8069327	-	"TCONS_LINC_0000054	exon_number "1"
lincRNA_451	Chromosome08	8072009	8072494	-	"TCONS_LINC_0000054	exon_number "2"
lincRNA_452	Chromosome08	8756697	8756780	-	"TCONS_LINC_0000054	exon_number "1"
lincRNA_452	Chromosome08	8757545	8757607	-	"TCONS_LINC_0000054	exon_number "2"
lincRNA_452	Chromosome08	8761968	8762271	-	"TCONS_LINC_0000054	exon_number "3"
lincRNA_453	Scaffold01351	9023	9063	+	"TCONS_LINC_0001333	exon_number "1"
lincRNA_453	Scaffold01351	12848	13773	+	"TCONS_LINC_0001333	exon_number "2"
lincRNA_453	Scaffold01351	17404	17499	+	"TCONS_LINC_0001333	exon_number "3"
lincRNA_46	Chromosome16	20101572	20101802	-	"TCONS_LINC_0001113	exon_number "1"
lincRNA_46	Chromosome16	20103821	20103904	-	"TCONS_LINC_0001113	exon_number "2"
lincRNA_47	Chromosome16	20941514	20941780	-	"TCONS_LINC_0001113	exon_number "1"
lincRNA_47	Chromosome16	20959647	20959658	-	"TCONS_LINC_0001113	exon_number "2"
lincRNA_48	Chromosome16	21462133	21462268	+	"TCONS_LINC_0001108	exon_number "1"
lincRNA_48	Chromosome16	21465163	21465289	+	"TCONS_LINC_0001108	exon_number "2"
lincRNA_48	Chromosome16	21465410	21467925	+	"TCONS_LINC_0001108	exon_number "3"
lincRNA_48	Chromosome16	21464414	21464717	+	"TCONS_LINC_0001109	exon_number "1"
lincRNA_48	Chromosome16	21464915	21465289	+	"TCONS_LINC_0001109	exon_number "2"
lincRNA_48	Chromosome16	21465410	21465517	+	"TCONS_LINC_0001109	exon_number "3"
lincRNA_48	Chromosome16	21465917	21465980	+	"TCONS_LINC_0001109	exon_number "4"
lincRNA_48	Chromosome16	21466075	21466215	+	"TCONS_LINC_0001109	exon_number "5"
lincRNA_48	Chromosome16	21467240	21467925	+	"TCONS_LINC_0001109	exon_number "6"
lincRNA_49	Chromosome16	22797327	22797636	-	"TCONS_LINC_0001113	exon_number "1"
lincRNA_49	Chromosome16	22799444	22799817	-	"TCONS_LINC_0001113	exon_number "2"
lincRNA_49	Chromosome16	22799976	22800474	-	"TCONS_LINC_0001113	exon_number "3"
lincRNA_5	Chromosome10	11906991	11907185	+	"TCONS_LINC_0000067	exon_number "1"
lincRNA_5	Chromosome10	11909919	11910046	+	"TCONS_LINC_0000067	exon_number "2"
lincRNA_50	Chromosome16	23840360	23840438	+	"TCONS_LINC_0001091	exon_number "1"
lincRNA_50	Chromosome16	23843185	23844399	+	"TCONS_LINC_0001091	exon_number "2"
lincRNA_50	Chromosome16	23844565	23844589	+	"TCONS_LINC_0001091	exon_number "3"
lincRNA_50	Chromosome16	23842577	23843914	+	"TCONS_LINC_0001092	exon_number "1"
lincRNA_51	Chromosome16	24516650	24517046	+	"TCONS_LINC_0001093	exon_number "1"
lincRNA_51	Chromosome16	24518543	24519178	+	"TCONS_LINC_0001093	exon_number "2"
lincRNA_51	Chromosome16	24520384	24520443	+	"TCONS_LINC_0001093	exon_number "3"
lincRNA_51	Chromosome16	24520521	24520571	+	"TCONS_LINC_0001093	exon_number "4"
lincRNA_51	Chromosome16	24521344	24521349	+	"TCONS_LINC_0001093	exon_number "5"
lincRNA_51	Chromosome16	24517318	24517342	+	"TCONS_LINC_0001094	exon_number "1"
lincRNA_51	Chromosome16	24517420	24517780	+	"TCONS_LINC_0001094	exon_number "2"
lincRNA_51	Chromosome16	24518543	24518732	+	"TCONS_LINC_0001094	exon_number "3"
lincRNA_51	Chromosome16	24518968	24520571	+	"TCONS_LINC_0001095	exon_number "1"
lincRNA_51	Chromosome16	24520708	24521240	+	"TCONS_LINC_0001095	exon_number "2"
lincRNA_52	Chromosome16	25100556	25100561	+	"TCONS_LINC_0001097	exon_number "1"
lincRNA_52	Chromosome16	25100636	25100768	+	"TCONS_LINC_0001097	exon_number "2"
lincRNA_52	Chromosome16	25110019	25110090	+	"TCONS_LINC_0001097	exon_number "3"
lincRNA_52	Chromosome16	25110598	25111121	+	"TCONS_LINC_0001097	exon_number "4"
lincRNA_52	Chromosome16	25100748	25100768	+	"TCONS_LINC_0001098	exon_number "1"
lincRNA_52	Chromosome16	25100851	25100930	+	"TCONS_LINC_0001098	exon_number "2"
lincRNA_52	Chromosome16	25110019	25111121	+	"TCONS_LINC_0001098	exon_number "3"
lincRNA_52	Chromosome16	25101623	25101812	+	"TCONS_LINC_0001099	exon_number "1"
lincRNA_52	Chromosome16	25109304	25109966	+	"TCONS_LINC_0001099	exon_number "2"
lincRNA_52	Chromosome16	25109223	25109369	+	"TCONS_LINC_0001100	exon_number "1"
lincRNA_52	Chromosome16	25110021	25111121	+	"TCONS_LINC_0001100	exon_number "2"
lincRNA_53	Chromosome16	26366611	26367762	+	"TCONS_LINC_0001101	exon_number "1"
lincRNA_53	Chromosome16	26368062	26368714	+	"TCONS_LINC_0001101	exon_number "2"

lincRNA_53	Chromosome16	26368746	26369162	+	"TCONS_LINC_00001101	exon_number "3"
lincRNA_53	Chromosome16	26367338	26369162	+	"TCONS_LINC_00001102	exon_number "1"
lincRNA_54	Chromosome16	27804168	27804242	+	"TCONS_LINC_00001107	exon_number "1"
lincRNA_54	Chromosome16	27806807	27807334	+	"TCONS_LINC_00001107	exon_number "2"
lincRNA_55	Chromosome16	27935066	27935445	+	"TCONS_LINC_00001108	exon_number "1"
lincRNA_55	Chromosome16	27936627	27936706	+	"TCONS_LINC_00001108	exon_number "2"
lincRNA_55	Chromosome16	27937227	27937288	+	"TCONS_LINC_00001108	exon_number "3"
lincRNA_55	Chromosome16	27943467	27943988	+	"TCONS_LINC_00001108	exon_number "4"
lincRNA_55	Chromosome16	27935251	27936706	+	"TCONS_LINC_00001109	exon_number "1"
lincRNA_55	Chromosome16	27937227	27937288	+	"TCONS_LINC_00001109	exon_number "2"
lincRNA_55	Chromosome16	27938550	27938921	+	"TCONS_LINC_00001109	exon_number "3"
lincRNA_55	Chromosome16	27936536	27937288	+	"TCONS_LINC_00001110	exon_number "1"
lincRNA_55	Chromosome16	27943467	27943988	+	"TCONS_LINC_00001110	exon_number "2"
lincRNA_56	Chromosome16	28068913	28069840	-	"TCONS_LINC_00001139	exon_number "1"
lincRNA_56	Chromosome16	28070417	28071249	-	"TCONS_LINC_00001139	exon_number "2"
lincRNA_57	Chromosome16	28212936	28213385	-	"TCONS_LINC_00001140	exon_number "1"
lincRNA_57	Chromosome16	28213703	28215471	-	"TCONS_LINC_00001140	exon_number "2"
lincRNA_58	Chromosome16	28591836	28591872	-	"TCONS_LINC_00001141	exon_number "1"
lincRNA_58	Chromosome16	28596052	28596792	-	"TCONS_LINC_00001141	exon_number "2"
lincRNA_59	Chromosome16	5948927	5953190	+	"TCONS_LINC_00001078	exon_number "1"
lincRNA_59	Chromosome16	5953227	5957306	+	"TCONS_LINC_00001078	exon_number "2"
lincRNA_6	Chromosome10	12486684	12488478	-	"TCONS_LINC_00000714	exon_number "1"
lincRNA_6	Chromosome10	12489068	12489650	-	"TCONS_LINC_00000714	exon_number "2"
lincRNA_60	Chromosome16	6064757	6065251	-	"TCONS_LINC_00001117	exon_number "1"
lincRNA_60	Chromosome16	6076819	6076890	-	"TCONS_LINC_00001117	exon_number "2"
lincRNA_60	Chromosome16	6077607	6077691	-	"TCONS_LINC_00001117	exon_number "3"
lincRNA_60	Chromosome16	6080200	6080256	-	"TCONS_LINC_00001117	exon_number "4"
lincRNA_60	Chromosome16	6080541	6080580	-	"TCONS_LINC_00001117	exon_number "5"
lincRNA_60	Chromosome16	6080699	6081408	-	"TCONS_LINC_00001117	exon_number "6"
lincRNA_60	Chromosome16	6076795	6076890	-	"TCONS_LINC_00001118	exon_number "1"
lincRNA_60	Chromosome16	6077607	6077691	-	"TCONS_LINC_00001118	exon_number "2"
lincRNA_60	Chromosome16	6077800	6077845	-	"TCONS_LINC_00001118	exon_number "3"
lincRNA_60	Chromosome16	6079848	6079941	-	"TCONS_LINC_00001118	exon_number "4"
lincRNA_60	Chromosome16	6080200	6080220	-	"TCONS_LINC_00001118	exon_number "5"
lincRNA_60	Chromosome16	6076795	6076895	-	"TCONS_LINC_00001119	exon_number "1"
lincRNA_60	Chromosome16	6077611	6077691	-	"TCONS_LINC_00001119	exon_number "2"
lincRNA_60	Chromosome16	6077800	6077845	-	"TCONS_LINC_00001119	exon_number "3"
lincRNA_60	Chromosome16	6080200	6080256	-	"TCONS_LINC_00001119	exon_number "4"
lincRNA_60	Chromosome16	6080541	6080580	-	"TCONS_LINC_00001119	exon_number "5"
lincRNA_60	Chromosome16	6080699	6080783	-	"TCONS_LINC_00001119	exon_number "6"
lincRNA_60	Chromosome16	6082243	6084114	-	"TCONS_LINC_00001119	exon_number "7"
lincRNA_61	Chromosome16	9632681	9632730	+	"TCONS_LINC_00001080	exon_number "1"
lincRNA_61	Chromosome16	9639438	9639616	+	"TCONS_LINC_00001080	exon_number "2"
lincRNA_62	Chromosome17	10026803	10028219	+	"TCONS_LINC_00001157	exon_number "1"
lincRNA_62	Chromosome17	10030388	10030452	+	"TCONS_LINC_00001157	exon_number "2"
lincRNA_63	Chromosome17	11713658	11714166	+	"TCONS_LINC_00001158	exon_number "1"
lincRNA_63	Chromosome17	11714524	11714955	+	"TCONS_LINC_00001158	exon_number "2"
lincRNA_63	Chromosome17	11713666	11714166	+	"TCONS_LINC_00001159	exon_number "1"
lincRNA_63	Chromosome17	11715993	11716044	+	"TCONS_LINC_00001159	exon_number "2"
lincRNA_64	Chromosome17	12673341	12673352	+	"TCONS_LINC_00001160	exon_number "1"
lincRNA_64	Chromosome17	12682812	12683007	+	"TCONS_LINC_00001160	exon_number "2"
lincRNA_64	Chromosome17	12724652	12724665	+	"TCONS_LINC_00001160	exon_number "3"
lincRNA_65	Chromosome17	13187604	13188600	-	"TCONS_LINC_00001182	exon_number "1"
lincRNA_65	Chromosome17	13188675	13189059	-	"TCONS_LINC_00001182	exon_number "2"
lincRNA_65	Chromosome17	13189179	13189304	-	"TCONS_LINC_00001182	exon_number "3"
lincRNA_65	Chromosome17	13189511	13189598	-	"TCONS_LINC_00001182	exon_number "4"
lincRNA_65	Chromosome17	13189699	13190157	-	"TCONS_LINC_00001182	exon_number "5"

lincRNA_65	Chromosome17	13190243	13190401	-	"TCONS_LINC_00001182	exon_number "6"
lincRNA_66	Chromosome17	14970962	14971017	-	"TCONS_LINC_00001183	exon_number "1"
lincRNA_66	Chromosome17	14971118	14971221	-	"TCONS_LINC_00001183	exon_number "2"
lincRNA_66	Chromosome17	14971800	14972383	-	"TCONS_LINC_00001183	exon_number "3"
lincRNA_66	Chromosome17	14973740	14973828	-	"TCONS_LINC_00001183	exon_number "4"
lincRNA_66	Chromosome17	14973963	14974608	-	"TCONS_LINC_00001183	exon_number "5"
lincRNA_67	Chromosome17	1542857	1544819	+	"TCONS_LINC_00001142	exon_number "1"
lincRNA_67	Chromosome17	1544885	1545388	+	"TCONS_LINC_00001142	exon_number "2"
lincRNA_68	Chromosome17	18194185	18194690	-	"TCONS_LINC_00001184	exon_number "1"
lincRNA_68	Chromosome17	18194794	18195199	-	"TCONS_LINC_00001184	exon_number "2"
lincRNA_68	Chromosome17	18196232	18196473	-	"TCONS_LINC_00001184	exon_number "3"
lincRNA_69	Chromosome17	18283410	18285404	-	"TCONS_LINC_00001187	exon_number "1"
lincRNA_69	Chromosome17	18285506	18285935	-	"TCONS_LINC_00001187	exon_number "2"
lincRNA_69	Chromosome17	18283410	18284805	-	"TCONS_LINC_00001188	exon_number "1"
lincRNA_69	Chromosome17	18285345	18285404	-	"TCONS_LINC_00001188	exon_number "2"
lincRNA_69	Chromosome17	18285575	18285958	-	"TCONS_LINC_00001188	exon_number "3"
lincRNA_7	Chromosome10	12927211	12928698	+	"TCONS_LINC_00000674	exon_number "1"
lincRNA_7	Chromosome10	12929520	12929808	+	"TCONS_LINC_00000674	exon_number "2"
lincRNA_70	Chromosome17	19616118	19618471	+	"TCONS_LINC_00001165	exon_number "1"
lincRNA_70	Chromosome17	19644692	19644894	+	"TCONS_LINC_00001165	exon_number "2"
lincRNA_71	Chromosome17	19772945	19773346	-	"TCONS_LINC_00001190	exon_number "1"
lincRNA_71	Chromosome17	19773518	19773639	-	"TCONS_LINC_00001190	exon_number "2"
lincRNA_71	Chromosome17	19773756	19773865	-	"TCONS_LINC_00001190	exon_number "3"
lincRNA_71	Chromosome17	19773955	19774041	-	"TCONS_LINC_00001190	exon_number "4"
lincRNA_71	Chromosome17	19774117	19774221	-	"TCONS_LINC_00001190	exon_number "5"
lincRNA_71	Chromosome17	19775450	19775586	-	"TCONS_LINC_00001190	exon_number "6"
lincRNA_71	Chromosome17	19773627	19773639	-	"TCONS_LINC_00001191	exon_number "1"
lincRNA_71	Chromosome17	19773756	19773867	-	"TCONS_LINC_00001191	exon_number "2"
lincRNA_71	Chromosome17	19773955	19774041	-	"TCONS_LINC_00001191	exon_number "3"
lincRNA_71	Chromosome17	19774117	19774634	-	"TCONS_LINC_00001191	exon_number "4"
lincRNA_72	Chromosome17	20779468	20779741	+	"TCONS_LINC_00001166	exon_number "1"
lincRNA_72	Chromosome17	20789680	20789732	+	"TCONS_LINC_00001166	exon_number "2"
lincRNA_73	Chromosome17	21409547	21409796	-	"TCONS_LINC_00001192	exon_number "1"
lincRNA_73	Chromosome17	21409819	21410444	-	"TCONS_LINC_00001192	exon_number "2"
lincRNA_73	Chromosome17	21412215	21412347	-	"TCONS_LINC_00001192	exon_number "3"
lincRNA_74	Chromosome17	2307656	2307769	+	"TCONS_LINC_00001144	exon_number "1"
lincRNA_74	Chromosome17	2307856	2308104	+	"TCONS_LINC_00001144	exon_number "2"
lincRNA_74	Chromosome17	2312391	2312595	+	"TCONS_LINC_00001144	exon_number "3"
lincRNA_74	Chromosome17	2312692	2313466	+	"TCONS_LINC_00001144	exon_number "4"
lincRNA_74	Chromosome17	2313550	2313636	+	"TCONS_LINC_00001144	exon_number "5"
lincRNA_74	Chromosome17	2313725	2313812	+	"TCONS_LINC_00001144	exon_number "6"
lincRNA_74	Chromosome17	2326092	2326208	+	"TCONS_LINC_00001144	exon_number "7"
lincRNA_74	Chromosome17	2326291	2326381	+	"TCONS_LINC_00001144	exon_number "8"
lincRNA_74	Chromosome17	2342832	2342875	+	"TCONS_LINC_00001144	exon_number "9"
lincRNA_74	Chromosome17	2343091	2343194	+	"TCONS_LINC_00001144	exon_number "10"
lincRNA_74	Chromosome17	2343613	2343859	+	"TCONS_LINC_00001144	exon_number "11"
lincRNA_75	Chromosome17	23722971	23727447	-	"TCONS_LINC_00001198	exon_number "1"
lincRNA_75	Chromosome17	23727539	23727799	-	"TCONS_LINC_00001198	exon_number "2"
lincRNA_75	Chromosome17	23728117	23728258	-	"TCONS_LINC_00001198	exon_number "3"
lincRNA_76	Chromosome17	24444802	24444888	-	"TCONS_LINC_00001201	exon_number "1"
lincRNA_76	Chromosome17	24445142	24445241	-	"TCONS_LINC_00001201	exon_number "2"
lincRNA_76	Chromosome17	24445320	24447456	-	"TCONS_LINC_00001201	exon_number "3"
lincRNA_77	Chromosome17	25284528	25285401	+	"TCONS_LINC_00001170	exon_number "1"
lincRNA_77	Chromosome17	25286618	25286964	+	"TCONS_LINC_00001170	exon_number "2"
lincRNA_78	Chromosome17	26678258	26678476	+	"TCONS_LINC_00001171	exon_number "1"
lincRNA_78	Chromosome17	26720028	26720037	+	"TCONS_LINC_00001171	exon_number "2"
lincRNA_79	Chromosome17	26779341	26779873	+	"TCONS_LINC_00001172	exon_number "1"

lincRNA_79	Chromosome17	26780363	26780548	+	"TCONS_LINC_00001172	exon_number "2"
lincRNA_79	Chromosome17	26780741	26780865	+	"TCONS_LINC_00001172	exon_number "3"
lincRNA_79	Chromosome17	26786146	26788279	+	"TCONS_LINC_00001172	exon_number "4"
lincRNA_79	Chromosome17	26780876	26782323	+	"TCONS_LINC_00001173	exon_number "1"
lincRNA_79	Chromosome17	26782701	26786841	+	"TCONS_LINC_00001173	exon_number "2"
lincRNA_79	Chromosome17	26788611	26788654	+	"TCONS_LINC_00001173	exon_number "3"
lincRNA_8	Chromosome10	13147147	13149519	-	"TCONS_LINC_00000716	exon_number "1"
lincRNA_80	Chromosome17	26952765	26952817	-	"TCONS_LINC_00001204	exon_number "1"
lincRNA_80	Chromosome17	26959938	26960022	-	"TCONS_LINC_00001204	exon_number "2"
lincRNA_80	Chromosome17	26960131	26960176	-	"TCONS_LINC_00001204	exon_number "3"
lincRNA_80	Chromosome17	26962573	26962629	-	"TCONS_LINC_00001204	exon_number "4"
lincRNA_80	Chromosome17	26962901	26962940	-	"TCONS_LINC_00001204	exon_number "5"
lincRNA_80	Chromosome17	26963059	26963182	-	"TCONS_LINC_00001204	exon_number "6"
lincRNA_80	Chromosome17	26958277	26958644	-	"TCONS_LINC_00001205	exon_number "1"
lincRNA_80	Chromosome17	26959207	26959338	-	"TCONS_LINC_00001205	exon_number "2"
lincRNA_80	Chromosome17	26959938	26960022	-	"TCONS_LINC_00001205	exon_number "3"
lincRNA_80	Chromosome17	26962573	26962629	-	"TCONS_LINC_00001205	exon_number "4"
lincRNA_80	Chromosome17	26962901	26962940	-	"TCONS_LINC_00001205	exon_number "5"
lincRNA_80	Chromosome17	26963059	26963261	-	"TCONS_LINC_00001205	exon_number "6"
lincRNA_81	Chromosome17	4624320	4624585	-	"TCONS_LINC_00001176	exon_number "1"
lincRNA_81	Chromosome17	4626150	4626221	-	"TCONS_LINC_00001176	exon_number "2"
lincRNA_81	Chromosome17	4626377	4626442	-	"TCONS_LINC_00001176	exon_number "3"
lincRNA_81	Chromosome17	4627072	4627168	-	"TCONS_LINC_00001176	exon_number "4"
lincRNA_82	Chromosome17	4764758	4764766	-	"TCONS_LINC_00001177	exon_number "1"
lincRNA_82	Chromosome17	4813605	4813850	-	"TCONS_LINC_00001177	exon_number "2"
lincRNA_83	Chromosome17	5569122	5569140	+	"TCONS_LINC_00001146	exon_number "1"
lincRNA_83	Chromosome17	5574074	5575076	+	"TCONS_LINC_00001146	exon_number "2"
lincRNA_84	Chromosome17	5857587	5857814	+	"TCONS_LINC_00001147	exon_number "1"
lincRNA_84	Chromosome17	5862608	5863420	+	"TCONS_LINC_00001147	exon_number "2"
lincRNA_85	Chromosome17	6262487	6262991	+	"TCONS_LINC_00001149	exon_number "1"
lincRNA_85	Chromosome17	6264369	6269807	+	"TCONS_LINC_00001149	exon_number "2"
lincRNA_85	Chromosome17	6273760	6273960	+	"TCONS_LINC_00001149	exon_number "3"
lincRNA_86	Chromosome17	7600471	7600564	+	"TCONS_LINC_00001153	exon_number "1"
lincRNA_86	Chromosome17	7614381	7614445	+	"TCONS_LINC_00001153	exon_number "2"
lincRNA_86	Chromosome17	7614534	7615419	+	"TCONS_LINC_00001153	exon_number "3"
lincRNA_86	Chromosome17	7600564	7600564	+	"TCONS_LINC_00001154	exon_number "1"
lincRNA_86	Chromosome17	7600647	7602159	+	"TCONS_LINC_00001154	exon_number "2"
lincRNA_87	Scaffold01443	1045	1904	-	"TCONS_LINC_00001341	exon_number "1"
lincRNA_87	Scaffold01443	4747	4923	-	"TCONS_LINC_00001341	exon_number "2"
lincRNA_87	Scaffold01443	5735	5862	-	"TCONS_LINC_00001341	exon_number "3"
lincRNA_87	Scaffold01443	5984	6111	-	"TCONS_LINC_00001341	exon_number "4"
lincRNA_87	Scaffold01443	4483	4923	-	"TCONS_LINC_00001342	exon_number "1"
lincRNA_87	Scaffold01443	5735	6374	-	"TCONS_LINC_00001342	exon_number "2"
lincRNA_87	Scaffold01443	5020	5866	-	"TCONS_LINC_00001343	exon_number "1"
lincRNA_87	Scaffold01443	5984	6101	-	"TCONS_LINC_00001343	exon_number "2"
lincRNA_88	Chromosome15	10327315	10327323	-	"TCONS_LINC_00001058	exon_number "1"
lincRNA_88	Chromosome15	10331301	10331788	-	"TCONS_LINC_00001058	exon_number "2"
lincRNA_89	Chromosome15	11832083	11832279	+	"TCONS_LINC_00001026	exon_number "1"
lincRNA_89	Chromosome15	11832388	11832422	+	"TCONS_LINC_00001026	exon_number "2"
lincRNA_89	Chromosome15	11833266	11833523	+	"TCONS_LINC_00001026	exon_number "3"
lincRNA_89	Chromosome15	11839759	11839791	+	"TCONS_LINC_00001026	exon_number "4"
lincRNA_9	Chromosome10	14167856	14167936	-	"TCONS_LINC_00000717	exon_number "1"
lincRNA_9	Chromosome10	14168377	14168446	-	"TCONS_LINC_00000717	exon_number "2"
lincRNA_9	Chromosome10	14194011	14194253	-	"TCONS_LINC_00000717	exon_number "3"
lincRNA_90	Chromosome15	18388935	18389412	+	"TCONS_LINC_00001029	exon_number "1"
lincRNA_90	Chromosome15	18393482	18393618	+	"TCONS_LINC_00001029	exon_number "2"
lincRNA_91	Chromosome15	20709750	20709758	-	"TCONS_LINC_00001067	exon_number "1"

lincRNA_91	Chromosome15	20759032	20760581	-	"TCONS_LINC_0000106[illegible]	exon_number "2"
lincRNA_92	Chromosome15	21151501	21151701	-	"TCONS_LINC_0000106[illegible]	exon_number "1"
lincRNA_92	Chromosome15	21159396	21159609	-	"TCONS_LINC_0000106[illegible]	exon_number "2"
lincRNA_93	Chromosome15	23076113	23077533	+	"TCONS_LINC_0000103[illegible]	exon_number "1"
lincRNA_93	Chromosome15	23079826	23079950	+	"TCONS_LINC_0000103[illegible]	exon_number "2"
lincRNA_94	Chromosome15	23156497	23156516	-	"TCONS_LINC_0000107[illegible]	exon_number "1"
lincRNA_94	Chromosome15	23187831	23188620	-	"TCONS_LINC_0000107[illegible]	exon_number "2"
lincRNA_95	Chromosome15	23335607	23335963	+	"TCONS_LINC_0000103[illegible]	exon_number "1"
lincRNA_95	Chromosome15	23344081	23344087	+	"TCONS_LINC_0000103[illegible]	exon_number "2"
lincRNA_96	Chromosome15	23906993	23907320	+	"TCONS_LINC_0000103[illegible]	exon_number "1"
lincRNA_96	Chromosome15	23909529	23909700	+	"TCONS_LINC_0000103[illegible]	exon_number "2"
lincRNA_96	Chromosome15	23909840	23909948	+	"TCONS_LINC_0000103[illegible]	exon_number "3"
lincRNA_97	Chromosome15	23913036	23913365	+	"TCONS_LINC_0000103[illegible]	exon_number "1"
lincRNA_97	Chromosome15	23919018	23919649	+	"TCONS_LINC_0000103[illegible]	exon_number "2"
lincRNA_97	Chromosome15	23926051	23926119	+	"TCONS_LINC_0000103[illegible]	exon_number "3"
lincRNA_98	Chromosome15	24484377	24485218	+	"TCONS_LINC_0000103[illegible]	exon_number "1"
lincRNA_98	Chromosome15	24489352	24490465	+	"TCONS_LINC_0000103[illegible]	exon_number "2"
lincRNA_99	Chromosome15	25949598	25949609	-	"TCONS_LINC_0000107[illegible]	exon_number "1"
lincRNA_99	Chromosome15	26013198	26014008	-	"TCONS_LINC_0000107[illegible]	exon_number "2"
lncNAT_1	Chromosome04	19402156	19403375	-	"TCONS_NAT_00000098	exon_number "1"
lncNAT_1	Chromosome04	19407467	19408711	-	"TCONS_NAT_00000098	exon_number "2"
lncNAT_10	Chromosome05	22580911	22585586	-	"TCONS_NAT_00000127	exon_number "1"
lncNAT_100	Chromosome13	5135947	5136098	+	"TCONS_NAT_00000303	exon_number "1"
lncNAT_100	Chromosome13	5136205	5138806	+	"TCONS_NAT_00000303	exon_number "2"
lncNAT_101	Chromosome13	939146	939492	+	"TCONS_NAT_00000299	exon_number "1"
lncNAT_101	Chromosome13	956348	956950	+	"TCONS_NAT_00000299	exon_number "2"
lncNAT_101	Chromosome13	955778	956950	+	"TCONS_NAT_00000300	exon_number "1"
lncNAT_102	Chromosome12	1074950	1076307	+	"TCONS_NAT_00000280	exon_number "1"
lncNAT_102	Chromosome12	1076368	1077932	+	"TCONS_NAT_00000280	exon_number "2"
lncNAT_102	Chromosome12	1082125	1082801	+	"TCONS_NAT_00000280	exon_number "3"
lncNAT_103	Chromosome12	15994211	15994454	+	"TCONS_NAT_00000285	exon_number "1"
lncNAT_103	Chromosome12	16163464	16164219	+	"TCONS_NAT_00000285	exon_number "2"
lncNAT_104	Chromosome12	21532778	21532792	-	"TCONS_NAT_00000294	exon_number "1"
lncNAT_104	Chromosome12	21557740	21558355	-	"TCONS_NAT_00000294	exon_number "2"
lncNAT_105	Chromosome12	30110663	30110719	-	"TCONS_NAT_00000296	exon_number "1"
lncNAT_105	Chromosome12	30110948	30111081	-	"TCONS_NAT_00000296	exon_number "2"
lncNAT_105	Chromosome12	30111208	30111293	-	"TCONS_NAT_00000296	exon_number "3"
lncNAT_105	Chromosome12	30113063	30113134	-	"TCONS_NAT_00000296	exon_number "4"
lncNAT_105	Chromosome12	30113367	30113423	-	"TCONS_NAT_00000296	exon_number "5"
lncNAT_105	Chromosome12	30113620	30113730	-	"TCONS_NAT_00000296	exon_number "6"
lncNAT_105	Chromosome12	30113846	30113957	-	"TCONS_NAT_00000296	exon_number "7"
lncNAT_105	Chromosome12	30119563	30119634	-	"TCONS_NAT_00000296	exon_number "8"
lncNAT_105	Chromosome12	30119742	30119798	-	"TCONS_NAT_00000296	exon_number "9"
lncNAT_105	Chromosome12	30119875	30119971	-	"TCONS_NAT_00000296	exon_number "10"
lncNAT_105	Chromosome12	30120224	30120308	-	"TCONS_NAT_00000296	exon_number "11"
lncNAT_105	Chromosome12	30128888	30128959	-	"TCONS_NAT_00000296	exon_number "12"
lncNAT_105	Chromosome12	30129067	30129123	-	"TCONS_NAT_00000296	exon_number "13"
lncNAT_105	Chromosome12	30129200	30129296	-	"TCONS_NAT_00000296	exon_number "14"
lncNAT_105	Chromosome12	30129388	30129427	-	"TCONS_NAT_00000296	exon_number "15"
lncNAT_105	Chromosome12	30129549	30129633	-	"TCONS_NAT_00000296	exon_number "16"
lncNAT_105	Chromosome12	30132111	30132204	-	"TCONS_NAT_00000296	exon_number "17"
lncNAT_105	Chromosome12	30132312	30132368	-	"TCONS_NAT_00000296	exon_number "18"
lncNAT_105	Chromosome12	30136074	30136170	-	"TCONS_NAT_00000296	exon_number "19"
lncNAT_105	Chromosome12	30136262	30136302	-	"TCONS_NAT_00000296	exon_number "20"
lncNAT_105	Chromosome12	30140946	30141003	-	"TCONS_NAT_00000296	exon_number "21"
lncNAT_106	Chromosome12	30663547	30663875	-	"TCONS_NAT_00000297	exon_number "1"
lncNAT_106	Chromosome12	30731477	30731512	-	"TCONS_NAT_00000297	exon_number "2"

lncNAT_107	Chromosome12	30872521	30872776	+	"TCONS_NAT_00000286	exon_number "1"
lncNAT_107	Chromosome12	30876984	30877245	+	"TCONS_NAT_00000286	exon_number "2"
lncNAT_107	Chromosome12	30877335	30879003	+	"TCONS_NAT_00000286	exon_number "3"
lncNAT_107	Chromosome12	30876539	30877695	+	"TCONS_NAT_00000287	exon_number "1"
lncNAT_107	Chromosome12	30877832	30879003	+	"TCONS_NAT_00000287	exon_number "2"
lncNAT_108	Chromosome12	3820523	3821324	-	"TCONS_NAT_00000290	exon_number "1"
lncNAT_108	Chromosome12	3823101	3823236	-	"TCONS_NAT_00000290	exon_number "2"
lncNAT_109	Chromosome12	4651677	4652036	+	"TCONS_NAT_00000281	exon_number "1"
lncNAT_109	Chromosome12	4652239	4659439	+	"TCONS_NAT_00000281	exon_number "2"
lncNAT_109	Chromosome12	4651681	4652036	+	"TCONS_NAT_00000282	exon_number "1"
lncNAT_109	Chromosome12	4652239	4657092	+	"TCONS_NAT_00000282	exon_number "2"
lncNAT_109	Chromosome12	4658095	4659439	+	"TCONS_NAT_00000282	exon_number "3"
lncNAT_11	Chromosome05	25497673	25501856	+	"TCONS_NAT_00000112	exon_number "1"
lncNAT_110	Chromosome12	5812321	5818860	+	"TCONS_NAT_00000284	exon_number "1"
lncNAT_110	Chromosome12	5837679	5837689	+	"TCONS_NAT_00000284	exon_number "2"
lncNAT_111	Chromosome12	6044139	6044382	-	"TCONS_NAT_00000291	exon_number "1"
lncNAT_111	Chromosome12	6045173	6048019	-	"TCONS_NAT_00000291	exon_number "2"
lncNAT_112	Chromosome11	1166998	1168011	-	"TCONS_NAT_00000266	exon_number "1"
lncNAT_112	Chromosome11	1166998	1167759	-	"TCONS_NAT_00000267	exon_number "1"
lncNAT_112	Chromosome11	1168387	1168494	-	"TCONS_NAT_00000267	exon_number "2"
lncNAT_112	Chromosome11	1168589	1169449	-	"TCONS_NAT_00000267	exon_number "3"
lncNAT_113	Chromosome11	11806992	11807000	+	"TCONS_NAT_00000264	exon_number "1"
lncNAT_113	Chromosome11	11835550	11835762	+	"TCONS_NAT_00000264	exon_number "2"
lncNAT_114	Chromosome11	1469224	1470013	-	"TCONS_NAT_00000268	exon_number "1"
lncNAT_114	Chromosome11	1470126	1470285	-	"TCONS_NAT_00000268	exon_number "2"
lncNAT_114	Chromosome11	1470993	1471032	-	"TCONS_NAT_00000268	exon_number "3"
lncNAT_114	Chromosome11	1471140	1473429	-	"TCONS_NAT_00000268	exon_number "4"
lncNAT_115	Chromosome11	19869429	19869437	-	"TCONS_NAT_00000275	exon_number "1"
lncNAT_115	Chromosome11	19876317	19877764	-	"TCONS_NAT_00000275	exon_number "2"
lncNAT_116	Chromosome11	24450681	24450801	-	"TCONS_NAT_00000276	exon_number "1"
lncNAT_116	Chromosome11	24451057	24451165	-	"TCONS_NAT_00000276	exon_number "2"
lncNAT_116	Chromosome11	24451237	24451347	-	"TCONS_NAT_00000276	exon_number "3"
lncNAT_116	Chromosome11	24451585	24451787	-	"TCONS_NAT_00000276	exon_number "4"
lncNAT_116	Chromosome11	24451929	24452130	-	"TCONS_NAT_00000276	exon_number "5"
lncNAT_116	Chromosome11	24452672	24453264	-	"TCONS_NAT_00000276	exon_number "6"
lncNAT_117	Chromosome11	24645975	24646230	-	"TCONS_NAT_00000277	exon_number "1"
lncNAT_117	Chromosome11	24663996	24664228	-	"TCONS_NAT_00000277	exon_number "2"
lncNAT_118	Chromosome11	254168	256751	+	"TCONS_NAT_00000257	exon_number "1"
lncNAT_119	Chromosome11	27080746	27082321	-	"TCONS_NAT_00000278	exon_number "1"
lncNAT_119	Chromosome11	27082845	27083558	-	"TCONS_NAT_00000278	exon_number "2"
lncNAT_12	Chromosome05	27125230	27125654	+	"TCONS_NAT_00000118	exon_number "1"
lncNAT_12	Chromosome05	27126523	27127328	+	"TCONS_NAT_00000118	exon_number "2"
lncNAT_12	Chromosome05	27127697	27129242	+	"TCONS_NAT_00000118	exon_number "3"
lncNAT_120	Chromosome11	27095392	27095745	-	"TCONS_NAT_00000279	exon_number "1"
lncNAT_120	Chromosome11	27095853	27097421	-	"TCONS_NAT_00000279	exon_number "2"
lncNAT_121	Chromosome11	300503	303941	+	"TCONS_NAT_00000258	exon_number "1"
lncNAT_121	Chromosome11	304015	304161	+	"TCONS_NAT_00000258	exon_number "2"
lncNAT_122	Chromosome11	5566422	5568462	+	"TCONS_NAT_00000261	exon_number "1"
lncNAT_122	Chromosome11	5568495	5568799	+	"TCONS_NAT_00000261	exon_number "2"
lncNAT_123	Chromosome11	6442261	6444403	+	"TCONS_NAT_00000262	exon_number "1"
lncNAT_123	Chromosome11	6461727	6461758	+	"TCONS_NAT_00000262	exon_number "2"
lncNAT_124	Chromosome11	7185434	7190494	-	"TCONS_NAT_00000272	exon_number "1"
lncNAT_124	Chromosome11	7190519	7191172	-	"TCONS_NAT_00000272	exon_number "2"
lncNAT_124	Chromosome11	7185434	7188913	-	"TCONS_NAT_00000273	exon_number "1"
lncNAT_124	Chromosome11	7192294	7192380	-	"TCONS_NAT_00000273	exon_number "2"
lncNAT_125	Chromosome11	8081783	8083228	+	"TCONS_NAT_00000263	exon_number "1"
lncNAT_125	Chromosome11	8083361	8083556	+	"TCONS_NAT_00000263	exon_number "2"

lncNAT_125	Chromosome11	8083649	8084436	+	"TCONS_NAT_00000263	exon_number "3"
lncNAT_126	Chromosome10	10129151	10129195	-	"TCONS_NAT_00000245	exon_number "1"
lncNAT_126	Chromosome10	10131640	10132373	-	"TCONS_NAT_00000245	exon_number "2"
lncNAT_126	Chromosome10	10132477	10132767	-	"TCONS_NAT_00000245	exon_number "3"
lncNAT_127	Chromosome10	1114750	1115035	+	"TCONS_NAT_00000229	exon_number "1"
lncNAT_127	Chromosome10	1115168	1117054	+	"TCONS_NAT_00000229	exon_number "2"
lncNAT_128	Chromosome10	134290	138475	-	"TCONS_NAT_00000241	exon_number "1"
lncNAT_128	Chromosome10	138505	139379	-	"TCONS_NAT_00000241	exon_number "2"
lncNAT_129	Chromosome10	13772455	13775323	-	"TCONS_NAT_00000246	exon_number "1"
lncNAT_129	Chromosome10	13772455	13774944	-	"TCONS_NAT_00000247	exon_number "1"
lncNAT_129	Chromosome10	13776420	13776420	-	"TCONS_NAT_00000247	exon_number "2"
lncNAT_13	Chromosome05	27571373	27575495	+	"TCONS_NAT_00000119	exon_number "1"
lncNAT_13	Chromosome05	27575613	27575776	+	"TCONS_NAT_00000119	exon_number "2"
lncNAT_13	Chromosome05	27587027	27588309	+	"TCONS_NAT_00000119	exon_number "3"
lncNAT_130	Chromosome10	1802636	1806615	+	"TCONS_NAT_00000230	exon_number "1"
lncNAT_130	Chromosome10	1806807	1807037	+	"TCONS_NAT_00000230	exon_number "2"
lncNAT_131	Chromosome10	18960616	18961777	-	"TCONS_NAT_00000248	exon_number "1"
lncNAT_131	Chromosome10	18961941	18964449	-	"TCONS_NAT_00000248	exon_number "2"
lncNAT_132	Chromosome10	19317679	19318266	-	"TCONS_NAT_00000249	exon_number "1"
lncNAT_132	Chromosome10	19319559	19320246	-	"TCONS_NAT_00000249	exon_number "2"
lncNAT_132	Chromosome10	19320357	19320815	-	"TCONS_NAT_00000249	exon_number "3"
lncNAT_132	Chromosome10	19319053	19320246	-	"TCONS_NAT_00000250	exon_number "1"
lncNAT_132	Chromosome10	19320357	19320793	-	"TCONS_NAT_00000250	exon_number "2"
lncNAT_133	Chromosome10	19668200	19668474	-	"TCONS_NAT_00000252	exon_number "1"
lncNAT_133	Chromosome10	19692128	19692280	-	"TCONS_NAT_00000252	exon_number "2"
lncNAT_134	Chromosome10	21050432	21051028	+	"TCONS_NAT_00000234	exon_number "1"
lncNAT_134	Chromosome10	21064705	21064715	+	"TCONS_NAT_00000234	exon_number "2"
lncNAT_135	Chromosome10	22134494	22134507	+	"TCONS_NAT_00000235	exon_number "1"
lncNAT_135	Chromosome10	22172417	22172603	+	"TCONS_NAT_00000235	exon_number "2"
lncNAT_136	Chromosome10	23047861	23048091	+	"TCONS_NAT_00000236	exon_number "1"
lncNAT_136	Chromosome10	23048221	23048307	+	"TCONS_NAT_00000236	exon_number "2"
lncNAT_136	Chromosome10	23050015	23050887	+	"TCONS_NAT_00000236	exon_number "3"
lncNAT_137	Chromosome10	24162122	24166754	-	"TCONS_NAT_00000255	exon_number "1"
lncNAT_138	Chromosome10	8230747	8231229	+	"TCONS_NAT_00000232	exon_number "1"
lncNAT_138	Chromosome10	8237690	8237765	+	"TCONS_NAT_00000232	exon_number "2"
lncNAT_139	Chromosome10	8641591	8642032	-	"TCONS_NAT_00000243	exon_number "1"
lncNAT_139	Chromosome10	8644636	8644854	-	"TCONS_NAT_00000243	exon_number "2"
lncNAT_14	Chromosome05	28252300	28252661	+	"TCONS_NAT_00000120	exon_number "1"
lncNAT_14	Chromosome05	28252766	28252875	+	"TCONS_NAT_00000120	exon_number "2"
lncNAT_14	Chromosome05	28253599	28254967	+	"TCONS_NAT_00000120	exon_number "3"
lncNAT_140	Chromosome08	25872731	25874156	+	"TCONS_NAT_00000183	exon_number "1"
lncNAT_140	Chromosome08	25882040	25882058	+	"TCONS_NAT_00000183	exon_number "2"
lncNAT_140	Chromosome08	25872734	25874156	+	"TCONS_NAT_00000184	exon_number "1"
lncNAT_140	Chromosome08	25875460	25875601	+	"TCONS_NAT_00000184	exon_number "2"
lncNAT_141	Chromosome08	28780826	28782264	+	"TCONS_NAT_00000186	exon_number "1"
lncNAT_141	Chromosome08	28782924	28783023	+	"TCONS_NAT_00000186	exon_number "2"
lncNAT_142	Chromosome08	32055139	32057255	-	"TCONS_NAT_00000201	exon_number "1"
lncNAT_142	Chromosome08	32057745	32057998	-	"TCONS_NAT_00000201	exon_number "2"
lncNAT_143	Chromosome08	32618062	32619146	-	"TCONS_NAT_00000202	exon_number "1"
lncNAT_143	Chromosome08	32619289	32621207	-	"TCONS_NAT_00000202	exon_number "2"
lncNAT_144	Chromosome08	33776257	33776680	+	"TCONS_NAT_00000188	exon_number "1"
lncNAT_144	Chromosome08	33793893	33794154	+	"TCONS_NAT_00000188	exon_number "2"
lncNAT_144	Chromosome08	33794297	33795526	+	"TCONS_NAT_00000188	exon_number "3"
lncNAT_144	Chromosome08	33795610	33798151	+	"TCONS_NAT_00000188	exon_number "4"
lncNAT_144	Chromosome08	33776263	33780103	+	"TCONS_NAT_00000189	exon_number "1"
lncNAT_144	Chromosome08	33780133	33780938	+	"TCONS_NAT_00000189	exon_number "2"
lncNAT_144	Chromosome08	33791493	33794154	+	"TCONS_NAT_00000190	exon_number "1"

lncNAT_144	Chromosome08	33794297	33795526	+	"TCONS_NAT_00000190	exon_number "2"
lncNAT_144	Chromosome08	33795610	33798151	+	"TCONS_NAT_00000190	exon_number "3"
lncNAT_145	Chromosome08	3643943	3647119	+	"TCONS_NAT_00000179	exon_number "1"
lncNAT_146	Chromosome08	416004	416240	-	"TCONS_NAT_00000191	exon_number "1"
lncNAT_146	Chromosome08	437012	437386	-	"TCONS_NAT_00000191	exon_number "2"
lncNAT_147	Chromosome08	4375303	4375566	+	"TCONS_NAT_00000180	exon_number "1"
lncNAT_147	Chromosome08	4375767	4375917	+	"TCONS_NAT_00000180	exon_number "2"
lncNAT_147	Chromosome08	4377941	4380719	+	"TCONS_NAT_00000180	exon_number "3"
lncNAT_148	Chromosome08	4501849	4501856	-	"TCONS_NAT_00000194	exon_number "1"
lncNAT_148	Chromosome08	4508309	4509311	-	"TCONS_NAT_00000194	exon_number "2"
lncNAT_148	Chromosome08	4507615	4509311	-	"TCONS_NAT_00000195	exon_number "1"
lncNAT_149	Chromosome08	5738681	5739111	+	"TCONS_NAT_00000181	exon_number "1"
lncNAT_149	Chromosome08	5741275	5743632	+	"TCONS_NAT_00000181	exon_number "2"
lncNAT_149	Chromosome08	5744443	5746320	+	"TCONS_NAT_00000181	exon_number "3"
lncNAT_149	Chromosome08	5746411	5746488	+	"TCONS_NAT_00000181	exon_number "4"
lncNAT_149	Chromosome08	5747649	5747822	+	"TCONS_NAT_00000181	exon_number "5"
lncNAT_149	Chromosome08	5741244	5746488	+	"TCONS_NAT_00000182	exon_number "1"
lncNAT_149	Chromosome08	5747649	5747822	+	"TCONS_NAT_00000182	exon_number "2"
lncNAT_15	Chromosome05	3279406	3282241	-	"TCONS_NAT_00000122	exon_number "1"
lncNAT_15	Chromosome05	3296735	3297835	-	"TCONS_NAT_00000122	exon_number "2"
lncNAT_150	Chromosome08	8043421	8043493	-	"TCONS_NAT_00000196	exon_number "1"
lncNAT_150	Chromosome08	8072009	8072462	-	"TCONS_NAT_00000196	exon_number "2"
lncNAT_151	Chromosome09	12462288	12462637	+	"TCONS_NAT_00000206	exon_number "1"
lncNAT_151	Chromosome09	12463286	12463400	+	"TCONS_NAT_00000206	exon_number "2"
lncNAT_151	Chromosome09	12475423	12475678	+	"TCONS_NAT_00000206	exon_number "3"
lncNAT_152	Chromosome09	12683518	12684064	-	"TCONS_NAT_00000219	exon_number "1"
lncNAT_152	Chromosome09	12684101	12684721	-	"TCONS_NAT_00000219	exon_number "2"
lncNAT_152	Chromosome09	12685495	12685538	-	"TCONS_NAT_00000219	exon_number "3"
lncNAT_153	Chromosome09	14207775	14210323	+	"TCONS_NAT_00000208	exon_number "1"
lncNAT_153	Chromosome09	14210488	14211468	+	"TCONS_NAT_00000208	exon_number "2"
lncNAT_153	Chromosome09	14211503	14211698	+	"TCONS_NAT_00000208	exon_number "3"
lncNAT_154	Chromosome09	14483054	14484152	-	"TCONS_NAT_00000220	exon_number "1"
lncNAT_154	Chromosome09	14486470	14486532	-	"TCONS_NAT_00000220	exon_number "2"
lncNAT_155	Chromosome09	1595018	1596990	+	"TCONS_NAT_00000204	exon_number "1"
lncNAT_155	Chromosome09	1597593	1601166	+	"TCONS_NAT_00000204	exon_number "2"
lncNAT_156	Chromosome09	1776902	1777047	-	"TCONS_NAT_00000212	exon_number "1"
lncNAT_156	Chromosome09	1780268	1781678	-	"TCONS_NAT_00000212	exon_number "2"
lncNAT_157	Chromosome09	215933	216473	+	"TCONS_NAT_00000203	exon_number "1"
lncNAT_157	Chromosome09	237024	237031	+	"TCONS_NAT_00000203	exon_number "2"
lncNAT_158	Chromosome09	26031057	26031073	-	"TCONS_NAT_00000226	exon_number "1"
lncNAT_158	Chromosome09	26033505	26034905	-	"TCONS_NAT_00000226	exon_number "2"
lncNAT_158	Chromosome09	26032148	26032177	-	"TCONS_NAT_00000227	exon_number "1"
lncNAT_158	Chromosome09	26033505	26036162	-	"TCONS_NAT_00000227	exon_number "2"
lncNAT_158	Chromosome09	26036185	26037975	-	"TCONS_NAT_00000227	exon_number "3"
lncNAT_159	Chromosome09	26507412	26508193	+	"TCONS_NAT_00000210	exon_number "1"
lncNAT_159	Chromosome09	26508588	26510475	+	"TCONS_NAT_00000210	exon_number "2"
lncNAT_16	Chromosome05	3612595	3614157	-	"TCONS_NAT_00000123	exon_number "1"
lncNAT_16	Chromosome05	3614713	3614831	-	"TCONS_NAT_00000123	exon_number "2"
lncNAT_16	Chromosome05	3614988	3615318	-	"TCONS_NAT_00000123	exon_number "3"
lncNAT_16	Chromosome05	3615685	3615900	-	"TCONS_NAT_00000123	exon_number "4"
lncNAT_16	Chromosome05	3616802	3617268	-	"TCONS_NAT_00000123	exon_number "5"
lncNAT_160	Chromosome09	28399682	28400517	+	"TCONS_NAT_00000211	exon_number "1"
lncNAT_160	Chromosome09	28401423	28402483	+	"TCONS_NAT_00000211	exon_number "2"
lncNAT_160	Chromosome09	28402640	28405084	+	"TCONS_NAT_00000211	exon_number "3"
lncNAT_161	Chromosome09	4525724	4526560	-	"TCONS_NAT_00000214	exon_number "1"
lncNAT_161	Chromosome09	4526638	4528735	-	"TCONS_NAT_00000214	exon_number "2"
lncNAT_162	Chromosome09	5533665	5538043	-	"TCONS_NAT_00000215	exon_number "1"

lncNAT_162	Chromosome09	5533665	5536888	-	"TCONS_NAT_00000216	exon_number "1"
lncNAT_162	Chromosome09	5537194	5538724	-	"TCONS_NAT_00000216	exon_number "2"
lncNAT_163	Chromosome09	7748687	7749425	-	"TCONS_NAT_00000218	exon_number "1"
lncNAT_163	Chromosome09	7753813	7754617	-	"TCONS_NAT_00000218	exon_number "2"
lncNAT_164	Chromosome09	9328679	9329746	+	"TCONS_NAT_00000205	exon_number "1"
lncNAT_164	Chromosome09	9330054	9333604	+	"TCONS_NAT_00000205	exon_number "2"
lncNAT_165	Chromosome15	12225459	12225999	+	"TCONS_NAT_00000365	exon_number "1"
lncNAT_165	Chromosome15	12229965	12232997	+	"TCONS_NAT_00000365	exon_number "2"
lncNAT_166	Chromosome15	12387781	12390311	-	"TCONS_NAT_00000377	exon_number "1"
lncNAT_167	Chromosome15	1240952	1243787	-	"TCONS_NAT_00000370	exon_number "1"
lncNAT_167	Chromosome15	1243813	1245909	-	"TCONS_NAT_00000370	exon_number "2"
lncNAT_168	Chromosome15	1692974	1697359	-	"TCONS_NAT_00000371	exon_number "1"
lncNAT_168	Chromosome15	1692974	1696111	-	"TCONS_NAT_00000372	exon_number "1"
lncNAT_168	Chromosome15	1699266	1701333	-	"TCONS_NAT_00000372	exon_number "2"
lncNAT_169	Chromosome15	19062963	19063138	+	"TCONS_NAT_00000367	exon_number "1"
lncNAT_169	Chromosome15	19063236	19063302	+	"TCONS_NAT_00000367	exon_number "2"
lncNAT_169	Chromosome15	19063996	19066708	+	"TCONS_NAT_00000367	exon_number "3"
lncNAT_169	Chromosome15	19063679	19066708	+	"TCONS_NAT_00000368	exon_number "1"
lncNAT_17	Chromosome05	4074477	4074494	-	"TCONS_NAT_00000124	exon_number "1"
lncNAT_17	Chromosome05	4076266	4077077	-	"TCONS_NAT_00000124	exon_number "2"
lncNAT_170	Chromosome15	2168771	2168810	-	"TCONS_NAT_00000373	exon_number "1"
lncNAT_170	Chromosome15	2168842	2170793	-	"TCONS_NAT_00000373	exon_number "2"
lncNAT_171	Chromosome15	2297390	2297859	+	"TCONS_NAT_00000359	exon_number "1"
lncNAT_171	Chromosome15	2297893	2299013	+	"TCONS_NAT_00000359	exon_number "2"
lncNAT_171	Chromosome15	2299121	2299594	+	"TCONS_NAT_00000359	exon_number "3"
lncNAT_172	Chromosome15	22975585	22975611	-	"TCONS_NAT_00000378	exon_number "1"
lncNAT_172	Chromosome15	23005562	23006049	-	"TCONS_NAT_00000378	exon_number "2"
lncNAT_173	Chromosome15	4259127	4259318	+	"TCONS_NAT_00000361	exon_number "1"
lncNAT_173	Chromosome15	4272660	4276282	+	"TCONS_NAT_00000361	exon_number "2"
lncNAT_174	Chromosome15	4616695	4616836	+	"TCONS_NAT_00000362	exon_number "1"
lncNAT_174	Chromosome15	4619949	4621298	+	"TCONS_NAT_00000362	exon_number "2"
lncNAT_175	Chromosome14	1114548	1115849	+	"TCONS_NAT_00000332	exon_number "1"
lncNAT_175	Chromosome14	1116257	1116749	+	"TCONS_NAT_00000332	exon_number "2"
lncNAT_176	Chromosome14	11974525	11974657	-	"TCONS_NAT_00000353	exon_number "1"
lncNAT_176	Chromosome14	11974826	11978251	-	"TCONS_NAT_00000353	exon_number "2"
lncNAT_177	Chromosome14	20171713	20171728	-	"TCONS_NAT_00000355	exon_number "1"
lncNAT_177	Chromosome14	20240321	20241270	-	"TCONS_NAT_00000355	exon_number "2"
lncNAT_178	Chromosome14	2688331	2690293	+	"TCONS_NAT_00000333	exon_number "1"
lncNAT_178	Chromosome14	2690928	2691907	+	"TCONS_NAT_00000333	exon_number "2"
lncNAT_179	Chromosome14	3161893	3165280	+	"TCONS_NAT_00000335	exon_number "1"
lncNAT_179	Chromosome14	3167387	3167401	+	"TCONS_NAT_00000335	exon_number "2"
lncNAT_18	Chromosome05	8609508	8609561	+	"TCONS_NAT_00000108	exon_number "1"
lncNAT_18	Chromosome05	8612060	8612119	+	"TCONS_NAT_00000108	exon_number "2"
lncNAT_18	Chromosome05	8612472	8612516	+	"TCONS_NAT_00000108	exon_number "3"
lncNAT_18	Chromosome05	8613556	8613638	+	"TCONS_NAT_00000108	exon_number "4"
lncNAT_18	Chromosome05	8609743	8612119	+	"TCONS_NAT_00000109	exon_number "1"
lncNAT_18	Chromosome05	8612472	8612629	+	"TCONS_NAT_00000109	exon_number "2"
lncNAT_180	Chromosome14	3606554	3607189	-	"TCONS_NAT_00000347	exon_number "1"
lncNAT_180	Chromosome14	3613309	3613366	-	"TCONS_NAT_00000347	exon_number "2"
lncNAT_181	Chromosome14	379961	383451	+	"TCONS_NAT_00000330	exon_number "1"
lncNAT_182	Chromosome14	473730	473911	-	"TCONS_NAT_00000342	exon_number "1"
lncNAT_182	Chromosome14	476821	477041	-	"TCONS_NAT_00000342	exon_number "2"
lncNAT_182	Chromosome14	477748	478160	-	"TCONS_NAT_00000342	exon_number "3"
lncNAT_182	Chromosome14	474031	477041	-	"TCONS_NAT_00000343	exon_number "1"
lncNAT_182	Chromosome14	477748	477869	-	"TCONS_NAT_00000343	exon_number "2"
lncNAT_182	Chromosome14	478083	478548	-	"TCONS_NAT_00000343	exon_number "3"
lncNAT_183	Chromosome14	6132365	6133119	-	"TCONS_NAT_00000349	exon_number "1"

lncNAT_183	Chromosome14	6133268	6133557	-	"TCONS_NAT_00000349	exon_number "2"
lncNAT_183	Chromosome14	6133645	6133755	-	"TCONS_NAT_00000349	exon_number "3"
lncNAT_183	Chromosome14	6133863	6133948	-	"TCONS_NAT_00000349	exon_number "4"
lncNAT_183	Chromosome14	6136008	6136041	-	"TCONS_NAT_00000349	exon_number "5"
lncNAT_184	Chromosome14	6713639	6715190	+	"TCONS_NAT_00000338	exon_number "1"
lncNAT_184	Chromosome14	6715284	6717158	+	"TCONS_NAT_00000338	exon_number "2"
lncNAT_185	Chromosome14	8259796	8260395	-	"TCONS_NAT_00000352	exon_number "1"
lncNAT_185	Chromosome14	8260892	8262900	-	"TCONS_NAT_00000352	exon_number "2"
lncNAT_186	Chromosome14	8880068	8880132	+	"TCONS_NAT_00000339	exon_number "1"
lncNAT_186	Chromosome14	8881584	8882571	+	"TCONS_NAT_00000339	exon_number "2"
lncNAT_187	Chromosome14	965936	965986	-	"TCONS_NAT_00000344	exon_number "1"
lncNAT_187	Chromosome14	966023	967918	-	"TCONS_NAT_00000344	exon_number "2"
lncNAT_187	Chromosome14	968015	968199	-	"TCONS_NAT_00000344	exon_number "3"
lncNAT_188	Chromosome18	10476574	10476625	+	"TCONS_NAT_00000430	exon_number "1"
lncNAT_188	Chromosome18	10476741	10477101	+	"TCONS_NAT_00000430	exon_number "2"
lncNAT_188	Chromosome18	10477287	10478686	+	"TCONS_NAT_00000430	exon_number "3"
lncNAT_189	Chromosome18	12159322	12159343	-	"TCONS_NAT_00000441	exon_number "1"
lncNAT_189	Chromosome18	12174223	12175143	-	"TCONS_NAT_00000441	exon_number "2"
lncNAT_19	Chromosome05	955338	959796	-	"TCONS_NAT_00000121	exon_number "1"
lncNAT_190	Chromosome18	13994835	13994845	-	"TCONS_NAT_00000442	exon_number "1"
lncNAT_190	Chromosome18	14048218	14049028	-	"TCONS_NAT_00000442	exon_number "2"
lncNAT_191	Chromosome18	14160111	14160387	-	"TCONS_NAT_00000443	exon_number "1"
lncNAT_191	Chromosome18	14173359	14173410	-	"TCONS_NAT_00000443	exon_number "2"
lncNAT_192	Chromosome18	25222432	25222940	+	"TCONS_NAT_00000431	exon_number "1"
lncNAT_192	Chromosome18	25225030	25225045	+	"TCONS_NAT_00000431	exon_number "2"
lncNAT_193	Chromosome18	2554488	2556206	-	"TCONS_NAT_00000432	exon_number "1"
lncNAT_193	Chromosome18	2554488	2555872	-	"TCONS_NAT_00000433	exon_number "1"
lncNAT_193	Chromosome18	2557350	2557580	-	"TCONS_NAT_00000433	exon_number "2"
lncNAT_193	Chromosome18	2557722	2557753	-	"TCONS_NAT_00000433	exon_number "3"
lncNAT_193	Chromosome18	2557846	2557926	-	"TCONS_NAT_00000433	exon_number "4"
lncNAT_193	Chromosome18	2559396	2559409	-	"TCONS_NAT_00000433	exon_number "5"
lncNAT_194	Chromosome18	2786836	2787955	-	"TCONS_NAT_00000434	exon_number "1"
lncNAT_194	Chromosome18	2788011	2788417	-	"TCONS_NAT_00000434	exon_number "2"
lncNAT_194	Chromosome18	2788587	2788678	-	"TCONS_NAT_00000434	exon_number "3"
lncNAT_194	Chromosome18	2788852	2790487	-	"TCONS_NAT_00000434	exon_number "4"
lncNAT_195	Chromosome18	3256436	3262294	-	"TCONS_NAT_00000435	exon_number "1"
lncNAT_196	Chromosome18	4314373	4316241	-	"TCONS_NAT_00000436	exon_number "1"
lncNAT_196	Chromosome18	4316262	4316892	-	"TCONS_NAT_00000436	exon_number "2"
lncNAT_197	Chromosome18	4720836	4721463	-	"TCONS_NAT_00000437	exon_number "1"
lncNAT_197	Chromosome18	4722368	4724674	-	"TCONS_NAT_00000437	exon_number "2"
lncNAT_197	Chromosome18	4720836	4721707	-	"TCONS_NAT_00000438	exon_number "1"
lncNAT_197	Chromosome18	4722612	4732159	-	"TCONS_NAT_00000438	exon_number "2"
lncNAT_198	Chromosome18	5163717	5164000	+	"TCONS_NAT_00000425	exon_number "1"
lncNAT_198	Chromosome18	5168628	5168662	+	"TCONS_NAT_00000425	exon_number "2"
lncNAT_199	Chromosome18	5822912	5823896	-	"TCONS_NAT_00000439	exon_number "1"
lncNAT_199	Chromosome18	5823923	5827833	-	"TCONS_NAT_00000439	exon_number "2"
lncNAT_2	Chromosome04	22598891	22602258	-	"TCONS_NAT_00000101	exon_number "1"
lncNAT_2	Chromosome04	22602333	22604560	-	"TCONS_NAT_00000101	exon_number "2"
lncNAT_20	Chromosome06	10897444	10897741	-	"TCONS_NAT_00000141	exon_number "1"
lncNAT_20	Chromosome06	10958636	10958942	-	"TCONS_NAT_00000141	exon_number "2"
lncNAT_200	Chromosome18	6050559	6056829	+	"TCONS_NAT_00000427	exon_number "1"
lncNAT_201	Chromosome18	7206826	7207341	+	"TCONS_NAT_00000428	exon_number "1"
lncNAT_201	Chromosome18	7210907	7211006	+	"TCONS_NAT_00000428	exon_number "2"
lncNAT_202	Chromosome18	9044130	9044892	+	"TCONS_NAT_00000429	exon_number "1"
lncNAT_202	Chromosome18	9045158	9048347	+	"TCONS_NAT_00000429	exon_number "2"
lncNAT_203	Chromosome17	12129437	12129680	+	"TCONS_NAT_00000406	exon_number "1"
lncNAT_203	Chromosome17	12145395	12145421	+	"TCONS_NAT_00000406	exon_number "2"

lncNAT_204	Chromosome17	17566712	17567115	-	"TCONS_NAT_00000416	exon_number "1"
lncNAT_204	Chromosome17	17567292	17567450	-	"TCONS_NAT_00000416	exon_number "2"
lncNAT_204	Chromosome17	17567868	17569443	-	"TCONS_NAT_00000416	exon_number "3"
lncNAT_204	Chromosome17	17580071	17580126	-	"TCONS_NAT_00000416	exon_number "4"
lncNAT_204	Chromosome17	17567818	17568103	-	"TCONS_NAT_00000417	exon_number "1"
lncNAT_204	Chromosome17	17568489	17568864	-	"TCONS_NAT_00000417	exon_number "2"
lncNAT_204	Chromosome17	17570321	17571059	-	"TCONS_NAT_00000417	exon_number "3"
lncNAT_205	Chromosome17	17891909	17893767	+	"TCONS_NAT_00000407	exon_number "1"
lncNAT_205	Chromosome17	17894179	17894339	+	"TCONS_NAT_00000407	exon_number "2"
lncNAT_205	Chromosome17	17894529	17894597	+	"TCONS_NAT_00000407	exon_number "3"
lncNAT_206	Chromosome17	18052393	18052418	+	"TCONS_NAT_00000408	exon_number "1"
lncNAT_206	Chromosome17	18053911	18054911	+	"TCONS_NAT_00000408	exon_number "2"
lncNAT_206	Chromosome17	18055323	18055481	+	"TCONS_NAT_00000408	exon_number "3"
lncNAT_206	Chromosome17	18055657	18056263	+	"TCONS_NAT_00000408	exon_number "4"
lncNAT_206	Chromosome17	18053156	18054286	+	"TCONS_NAT_00000409	exon_number "1"
lncNAT_206	Chromosome17	18054676	18054911	+	"TCONS_NAT_00000409	exon_number "2"
lncNAT_206	Chromosome17	18055323	18055481	+	"TCONS_NAT_00000409	exon_number "3"
lncNAT_206	Chromosome17	18055657	18056263	+	"TCONS_NAT_00000409	exon_number "4"
lncNAT_206	Chromosome17	18055022	18055481	+	"TCONS_NAT_00000410	exon_number "1"
lncNAT_206	Chromosome17	18055657	18056263	+	"TCONS_NAT_00000410	exon_number "2"
lncNAT_207	Chromosome17	18484712	18486234	-	"TCONS_NAT_00000418	exon_number "1"
lncNAT_207	Chromosome17	18486291	18486712	-	"TCONS_NAT_00000418	exon_number "2"
lncNAT_208	Chromosome17	20716773	20716787	-	"TCONS_NAT_00000419	exon_number "1"
lncNAT_208	Chromosome17	20716876	20720089	-	"TCONS_NAT_00000419	exon_number "2"
lncNAT_209	Chromosome17	21211945	21213782	+	"TCONS_NAT_00000411	exon_number "1"
lncNAT_209	Chromosome17	21213970	21214457	+	"TCONS_NAT_00000411	exon_number "2"
lncNAT_21	Chromosome06	16352033	16354123	-	"TCONS_NAT_00000145	exon_number "1"
lncNAT_21	Chromosome06	16354416	16355447	-	"TCONS_NAT_00000145	exon_number "2"
lncNAT_21	Chromosome06	16355565	16356924	-	"TCONS_NAT_00000145	exon_number "3"
lncNAT_21	Chromosome06	16354346	16355438	-	"TCONS_NAT_00000146	exon_number "1"
lncNAT_21	Chromosome06	16355565	16356035	-	"TCONS_NAT_00000146	exon_number "2"
lncNAT_21	Chromosome06	16356125	16356398	-	"TCONS_NAT_00000146	exon_number "3"
lncNAT_210	Chromosome17	23140014	23140053	+	"TCONS_NAT_00000412	exon_number "1"
lncNAT_210	Chromosome17	23162544	23163348	+	"TCONS_NAT_00000412	exon_number "2"
lncNAT_210	Chromosome17	23163428	23163454	+	"TCONS_NAT_00000412	exon_number "3"
lncNAT_211	Chromosome17	24412969	24413030	-	"TCONS_NAT_00000421	exon_number "1"
lncNAT_211	Chromosome17	24415760	24416129	-	"TCONS_NAT_00000421	exon_number "2"
lncNAT_211	Chromosome17	24413215	24413342	-	"TCONS_NAT_00000422	exon_number "1"
lncNAT_211	Chromosome17	24414438	24416129	-	"TCONS_NAT_00000422	exon_number "2"
lncNAT_212	Chromosome17	8482008	8484236	+	"TCONS_NAT_00000405	exon_number "1"
lncNAT_213	Scaffold01115	42626	43293	-	"TCONS_NAT_00000450	exon_number "1"
lncNAT_213	Scaffold01115	56818	56827	-	"TCONS_NAT_00000450	exon_number "2"
lncNAT_213	Scaffold01115	56274	56827	-	"TCONS_NAT_00000451	exon_number "1"
lncNAT_214	Scaffold01105	12808	12819	-	"TCONS_NAT_00000449	exon_number "1"
lncNAT_214	Scaffold01105	34460	35288	-	"TCONS_NAT_00000449	exon_number "2"
lncNAT_215	Chromosome16	14334523	14334815	-	"TCONS_NAT_00000399	exon_number "1"
lncNAT_215	Chromosome16	14334853	14337098	-	"TCONS_NAT_00000399	exon_number "2"
lncNAT_216	Chromosome16	16339092	16339706	+	"TCONS_NAT_00000384	exon_number "1"
lncNAT_216	Chromosome16	16339767	16342434	+	"TCONS_NAT_00000384	exon_number "2"
lncNAT_216	Chromosome16	16435007	16435019	+	"TCONS_NAT_00000384	exon_number "3"
lncNAT_217	Chromosome16	1740919	1741206	-	"TCONS_NAT_00000393	exon_number "1"
lncNAT_217	Chromosome16	1752391	1753199	-	"TCONS_NAT_00000393	exon_number "2"
lncNAT_217	Chromosome16	1752159	1753245	-	"TCONS_NAT_00000394	exon_number "1"
lncNAT_218	Chromosome16	1854440	1856241	+	"TCONS_NAT_00000381	exon_number "1"
lncNAT_218	Chromosome16	1856263	1856554	+	"TCONS_NAT_00000381	exon_number "2"
lncNAT_219	Chromosome16	26904678	26905312	-	"TCONS_NAT_00000401	exon_number "1"
lncNAT_219	Chromosome16	26905393	26905507	-	"TCONS_NAT_00000401	exon_number "2"

lncNAT_219	Chromosome16	26906012	26906100	-	"TCONS_NAT_00000401	exon_number "3"
lncNAT_219	Chromosome16	26914592	26914630	-	"TCONS_NAT_00000401	exon_number "4"
lncNAT_22	Chromosome06	17667573	17669582	+	"TCONS_NAT_00000130	exon_number "1"
lncNAT_220	Chromosome16	2709426	2710385	-	"TCONS_NAT_00000396	exon_number "1"
lncNAT_220	Chromosome16	2711573	2711989	-	"TCONS_NAT_00000396	exon_number "2"
lncNAT_221	Chromosome16	27334117	27334590	-	"TCONS_NAT_00000402	exon_number "1"
lncNAT_221	Chromosome16	27334682	27335081	-	"TCONS_NAT_00000402	exon_number "2"
lncNAT_221	Chromosome16	27335166	27338399	-	"TCONS_NAT_00000402	exon_number "3"
lncNAT_222	Chromosome16	27935064	27935445	+	"TCONS_NAT_00000387	exon_number "1"
lncNAT_222	Chromosome16	27936627	27936706	+	"TCONS_NAT_00000387	exon_number "2"
lncNAT_222	Chromosome16	27937227	27937288	+	"TCONS_NAT_00000387	exon_number "3"
lncNAT_222	Chromosome16	27943467	27943631	+	"TCONS_NAT_00000387	exon_number "4"
lncNAT_222	Chromosome16	27944010	27944698	+	"TCONS_NAT_00000387	exon_number "5"
lncNAT_222	Chromosome16	27935251	27936706	+	"TCONS_NAT_00000388	exon_number "1"
lncNAT_222	Chromosome16	27937227	27937288	+	"TCONS_NAT_00000388	exon_number "2"
lncNAT_222	Chromosome16	27944010	27944698	+	"TCONS_NAT_00000388	exon_number "3"
lncNAT_222	Chromosome16	27938739	27943631	+	"TCONS_NAT_00000389	exon_number "1"
lncNAT_222	Chromosome16	27944010	27944698	+	"TCONS_NAT_00000389	exon_number "2"
lncNAT_223	Chromosome16	28018359	28019289	-	"TCONS_NAT_00000403	exon_number "1"
lncNAT_223	Chromosome16	28019365	28019759	-	"TCONS_NAT_00000403	exon_number "2"
lncNAT_223	Chromosome16	28019827	28021458	-	"TCONS_NAT_00000403	exon_number "3"
lncNAT_224	Chromosome16	28278034	28279638	-	"TCONS_NAT_00000404	exon_number "1"
lncNAT_224	Chromosome16	28279823	28280258	-	"TCONS_NAT_00000404	exon_number "2"
lncNAT_225	Chromosome16	3374286	3374792	+	"TCONS_NAT_00000382	exon_number "1"
lncNAT_225	Chromosome16	3375521	3376328	+	"TCONS_NAT_00000382	exon_number "2"
lncNAT_226	Chromosome16	4348052	4348749	+	"TCONS_NAT_00000383	exon_number "1"
lncNAT_226	Chromosome16	4380767	4380888	+	"TCONS_NAT_00000383	exon_number "2"
lncNAT_227	Chromosome16	542845	543138	+	"TCONS_NAT_00000380	exon_number "1"
lncNAT_227	Chromosome16	543239	543538	+	"TCONS_NAT_00000380	exon_number "2"
lncNAT_227	Chromosome16	543660	544162	+	"TCONS_NAT_00000380	exon_number "3"
lncNAT_227	Chromosome16	544439	544495	+	"TCONS_NAT_00000380	exon_number "4"
lncNAT_227	Chromosome16	546778	547506	+	"TCONS_NAT_00000380	exon_number "5"
lncNAT_228	Chromosome16	6925106	6925800	-	"TCONS_NAT_00000398	exon_number "1"
lncNAT_228	Chromosome16	6927072	6927456	-	"TCONS_NAT_00000398	exon_number "2"
lncNAT_229	Scaffold00550	204963	204993	-	"TCONS_NAT_00000446	exon_number "1"
lncNAT_229	Scaffold00550	227363	227780	-	"TCONS_NAT_00000446	exon_number "2"
lncNAT_23	Chromosome06	18405021	18405027	-	"TCONS_NAT_00000147	exon_number "1"
lncNAT_23	Chromosome06	18460500	18460942	-	"TCONS_NAT_00000147	exon_number "2"
lncNAT_24	Chromosome06	18819081	18819774	-	"TCONS_NAT_00000148	exon_number "1"
lncNAT_24	Chromosome06	18820920	18822428	-	"TCONS_NAT_00000148	exon_number "2"
lncNAT_25	Chromosome06	19785006	19786245	+	"TCONS_NAT_00000133	exon_number "1"
lncNAT_25	Chromosome06	19786345	19789628	+	"TCONS_NAT_00000133	exon_number "2"
lncNAT_26	Chromosome06	20523482	20524015	-	"TCONS_NAT_00000149	exon_number "1"
lncNAT_26	Chromosome06	20524126	20524726	-	"TCONS_NAT_00000149	exon_number "2"
lncNAT_26	Chromosome06	20525549	20527242	-	"TCONS_NAT_00000149	exon_number "3"
lncNAT_27	Chromosome06	22878444	22878940	+	"TCONS_NAT_00000134	exon_number "1"
lncNAT_27	Chromosome06	22880506	22880687	+	"TCONS_NAT_00000134	exon_number "2"
lncNAT_27	Chromosome06	22881492	22881700	+	"TCONS_NAT_00000134	exon_number "3"
lncNAT_28	Chromosome06	23595376	23595876	+	"TCONS_NAT_00000135	exon_number "1"
lncNAT_28	Chromosome06	23596774	23596847	+	"TCONS_NAT_00000135	exon_number "2"
lncNAT_28	Chromosome06	23597738	23599225	+	"TCONS_NAT_00000135	exon_number "3"
lncNAT_28	Chromosome06	23597040	23599225	+	"TCONS_NAT_00000136	exon_number "1"
lncNAT_29	Chromosome06	24663166	24663636	+	"TCONS_NAT_00000137	exon_number "1"
lncNAT_29	Chromosome06	24663725	24663802	+	"TCONS_NAT_00000137	exon_number "2"
lncNAT_29	Chromosome06	24664287	24670483	+	"TCONS_NAT_00000137	exon_number "3"
lncNAT_3	Chromosome04	23348274	23350949	+	"TCONS_NAT_00000091	exon_number "1"
lncNAT_30	Chromosome06	24767166	24769746	-	"TCONS_NAT_00000150	exon_number "1"

lncNAT_30	Chromosome06	24769872	24770178	-	"TCONS_NAT_00000150	exon_number "2"
lncNAT_31	Chromosome06	25752846	25755310	-	"TCONS_NAT_00000151	exon_number "1"
lncNAT_32	Chromosome06	3235115	3237203	+	"TCONS_NAT_00000129	exon_number "1"
lncNAT_32	Chromosome06	3237294	3238313	+	"TCONS_NAT_00000129	exon_number "2"
lncNAT_33	Chromosome07	12452208	12452221	-	"TCONS_NAT_00000168	exon_number "1"
lncNAT_33	Chromosome07	12470451	12470660	-	"TCONS_NAT_00000168	exon_number "2"
lncNAT_34	Chromosome07	17793820	17793897	-	"TCONS_NAT_00000171	exon_number "1"
lncNAT_34	Chromosome07	17799339	17799479	-	"TCONS_NAT_00000171	exon_number "2"
lncNAT_35	Chromosome07	21954676	21956042	-	"TCONS_NAT_00000173	exon_number "1"
lncNAT_35	Chromosome07	21979861	21979919	-	"TCONS_NAT_00000173	exon_number "2"
lncNAT_36	Chromosome07	23447479	23447789	+	"TCONS_NAT_00000156	exon_number "1"
lncNAT_36	Chromosome07	23451450	23451548	+	"TCONS_NAT_00000156	exon_number "2"
lncNAT_36	Chromosome07	23447479	23449354	+	"TCONS_NAT_00000155	exon_number "1"
lncNAT_36	Chromosome07	23451450	23451548	+	"TCONS_NAT_00000155	exon_number "2"
lncNAT_37	Chromosome07	26168512	26168811	+	"TCONS_NAT_00000158	exon_number "1"
lncNAT_37	Chromosome07	26168886	26169009	+	"TCONS_NAT_00000158	exon_number "2"
lncNAT_37	Chromosome07	26169396	26169544	+	"TCONS_NAT_00000158	exon_number "3"
lncNAT_37	Chromosome07	26169697	26173146	+	"TCONS_NAT_00000158	exon_number "4"
lncNAT_38	Chromosome07	26586457	26586557	-	"TCONS_NAT_00000176	exon_number "1"
lncNAT_38	Chromosome07	26590265	26590696	-	"TCONS_NAT_00000176	exon_number "2"
lncNAT_38	Chromosome07	26586457	26586758	-	"TCONS_NAT_00000177	exon_number "1"
lncNAT_38	Chromosome07	26587199	26587400	-	"TCONS_NAT_00000177	exon_number "2"
lncNAT_38	Chromosome07	26593133	26593386	-	"TCONS_NAT_00000177	exon_number "3"
lncNAT_38	Chromosome07	26593534	26593594	-	"TCONS_NAT_00000177	exon_number "4"
lncNAT_38	Chromosome07	26593702	26593809	-	"TCONS_NAT_00000177	exon_number "5"
lncNAT_38	Chromosome07	26593902	26594297	-	"TCONS_NAT_00000177	exon_number "6"
lncNAT_39	Chromosome07	4538196	4539140	-	"TCONS_NAT_00000161	exon_number "1"
lncNAT_39	Chromosome07	4539183	4541165	-	"TCONS_NAT_00000161	exon_number "2"
lncNAT_4	Chromosome04	26012670	26013112	+	"TCONS_NAT_00000092	exon_number "1"
lncNAT_4	Chromosome04	26019851	26020142	+	"TCONS_NAT_00000092	exon_number "2"
lncNAT_4	Chromosome04	26012795	26013166	+	"TCONS_NAT_00000093	exon_number "1"
lncNAT_4	Chromosome04	26019905	26020142	+	"TCONS_NAT_00000093	exon_number "2"
lncNAT_40	Chromosome07	8458425	8459587	-	"TCONS_NAT_00000163	exon_number "1"
lncNAT_40	Chromosome07	8530250	8530369	-	"TCONS_NAT_00000163	exon_number "2"
lncNAT_40	Chromosome07	8532446	8532972	-	"TCONS_NAT_00000163	exon_number "3"
lncNAT_41	Chromosome07	8613919	8614908	-	"TCONS_NAT_00000165	exon_number "1"
lncNAT_41	Chromosome07	8619635	8619754	-	"TCONS_NAT_00000165	exon_number "2"
lncNAT_41	Chromosome07	8621804	8622979	-	"TCONS_NAT_00000165	exon_number "3"
lncNAT_42	Chromosome07	9051920	9052743	-	"TCONS_NAT_00000166	exon_number "1"
lncNAT_42	Chromosome07	9052766	9054171	-	"TCONS_NAT_00000166	exon_number "2"
lncNAT_43	Scaffold01150	39090	40240	+	"TCONS_NAT_00000454	exon_number "1"
lncNAT_43	Scaffold01150	43222	44252	+	"TCONS_NAT_00000454	exon_number "2"
lncNAT_43	Scaffold01150	39108	40433	+	"TCONS_NAT_00000455	exon_number "1"
lncNAT_43	Scaffold01150	43412	44252	+	"TCONS_NAT_00000455	exon_number "2"
lncNAT_44	Chromosome01	16406762	16406770	-	"TCONS_NAT_00000017	exon_number "1"
lncNAT_44	Chromosome01	16497487	16498352	-	"TCONS_NAT_00000017	exon_number "2"
lncNAT_45	Chromosome01	19519296	19519327	+	"TCONS_NAT_00000004	exon_number "1"
lncNAT_45	Chromosome01	19520788	19522478	+	"TCONS_NAT_00000004	exon_number "2"
lncNAT_45	Chromosome01	19520531	19522478	+	"TCONS_NAT_00000005	exon_number "1"
lncNAT_46	Chromosome01	27639790	27640207	+	"TCONS_NAT_00000007	exon_number "1"
lncNAT_46	Chromosome01	27641745	27643763	+	"TCONS_NAT_00000007	exon_number "2"
lncNAT_47	Chromosome01	28019078	28022150	+	"TCONS_NAT_00000008	exon_number "1"
lncNAT_48	Chromosome01	28238856	28239144	+	"TCONS_NAT_00000009	exon_number "1"
lncNAT_48	Chromosome01	28252672	28252682	+	"TCONS_NAT_00000009	exon_number "2"
lncNAT_49	Chromosome01	29419782	29421001	+	"TCONS_NAT_00000010	exon_number "1"
lncNAT_49	Chromosome01	29421887	29421928	+	"TCONS_NAT_00000010	exon_number "2"
lncNAT_5	Chromosome04	26361972	26364005	+	"TCONS_NAT_00000094	exon_number "1"

lncNAT_5	Chromosome04	26365772	26365774	+	"TCONS_NAT_00000094	exon_number "2"
lncNAT_50	Chromosome01	29840090	29840226	+	"TCONS_NAT_00000011	exon_number "1"
lncNAT_50	Chromosome01	29840335	29840508	+	"TCONS_NAT_00000011	exon_number "2"
lncNAT_50	Chromosome01	29841606	29842120	+	"TCONS_NAT_00000011	exon_number "3"
lncNAT_51	Chromosome01	30430186	30431750	+	"TCONS_NAT_00000012	exon_number "1"
lncNAT_51	Chromosome01	30431827	30431896	+	"TCONS_NAT_00000012	exon_number "2"
lncNAT_51	Chromosome01	30433789	30434305	+	"TCONS_NAT_00000012	exon_number "3"
lncNAT_52	Chromosome01	31255714	31258358	+	"TCONS_NAT_00000014	exon_number "1"
lncNAT_53	Chromosome01	31620195	31622995	-	"TCONS_NAT_00000022	exon_number "1"
lncNAT_54	Chromosome01	32413115	32414300	-	"TCONS_NAT_00000023	exon_number "1"
lncNAT_54	Chromosome01	32415150	32415381	-	"TCONS_NAT_00000023	exon_number "2"
lncNAT_54	Chromosome01	32413115	32413283	-	"TCONS_NAT_00000024	exon_number "1"
lncNAT_54	Chromosome01	32414774	32415411	-	"TCONS_NAT_00000024	exon_number "2"
lncNAT_55	Chromosome01	32605209	32605642	+	"TCONS_NAT_00000015	exon_number "1"
lncNAT_55	Chromosome01	32605724	32606072	+	"TCONS_NAT_00000015	exon_number "2"
lncNAT_55	Chromosome01	32606250	32607737	+	"TCONS_NAT_00000015	exon_number "3"
lncNAT_56	Chromosome01	3666824	3668995	+	"TCONS_NAT_00000002	exon_number "1"
lncNAT_56	Chromosome01	3670238	3670880	+	"TCONS_NAT_00000002	exon_number "2"
lncNAT_57	Chromosome01	6396168	6396264	+	"TCONS_NAT_00000003	exon_number "1"
lncNAT_57	Chromosome01	6400166	6401091	+	"TCONS_NAT_00000003	exon_number "2"
lncNAT_58	Chromosome02	10544946	10546698	-	"TCONS_NAT_00000048	exon_number "1"
lncNAT_58	Chromosome02	10548302	10548337	-	"TCONS_NAT_00000048	exon_number "2"
lncNAT_59	Chromosome02	11882313	11884502	-	"TCONS_NAT_00000049	exon_number "1"
lncNAT_59	Chromosome02	11882313	11883282	-	"TCONS_NAT_00000050	exon_number "1"
lncNAT_59	Chromosome02	11886723	11888203	-	"TCONS_NAT_00000050	exon_number "2"
lncNAT_6	Chromosome04	26418797	26419895	-	"TCONS_NAT_00000103	exon_number "1"
lncNAT_6	Chromosome04	26420119	26421119	-	"TCONS_NAT_00000103	exon_number "2"
lncNAT_60	Chromosome02	1223212	1224211	+	"TCONS_NAT_00000027	exon_number "1"
lncNAT_60	Chromosome02	1224375	1229208	+	"TCONS_NAT_00000027	exon_number "2"
lncNAT_61	Chromosome02	15032431	15032937	+	"TCONS_NAT_00000036	exon_number "1"
lncNAT_61	Chromosome02	15033012	15034430	+	"TCONS_NAT_00000036	exon_number "2"
lncNAT_61	Chromosome02	15034691	15035017	+	"TCONS_NAT_00000036	exon_number "3"
lncNAT_61	Chromosome02	15035107	15036174	+	"TCONS_NAT_00000036	exon_number "4"
lncNAT_62	Chromosome02	18074463	18075805	-	"TCONS_NAT_00000053	exon_number "1"
lncNAT_62	Chromosome02	18076560	18076588	-	"TCONS_NAT_00000053	exon_number "2"
lncNAT_63	Chromosome02	18803284	18803559	+	"TCONS_NAT_00000037	exon_number "1"
lncNAT_63	Chromosome02	18810283	18810602	+	"TCONS_NAT_00000037	exon_number "2"
lncNAT_63	Chromosome02	18812897	18813458	+	"TCONS_NAT_00000037	exon_number "3"
lncNAT_63	Chromosome02	18808449	18810299	+	"TCONS_NAT_00000038	exon_number "1"
lncNAT_64	Chromosome02	2193194	2193258	-	"TCONS_NAT_00000041	exon_number "1"
lncNAT_64	Chromosome02	2194339	2194408	-	"TCONS_NAT_00000041	exon_number "2"
lncNAT_64	Chromosome02	2194533	2195639	-	"TCONS_NAT_00000041	exon_number "3"
lncNAT_65	Chromosome02	25909299	25909642	+	"TCONS_NAT_00000039	exon_number "1"
lncNAT_65	Chromosome02	25917595	25917622	+	"TCONS_NAT_00000039	exon_number "2"
lncNAT_66	Chromosome02	3467220	3468590	-	"TCONS_NAT_00000042	exon_number "1"
lncNAT_66	Chromosome02	3468865	3469223	-	"TCONS_NAT_00000042	exon_number "2"
lncNAT_67	Chromosome02	3532635	3535867	-	"TCONS_NAT_00000043	exon_number "1"
lncNAT_67	Chromosome02	3536098	3536410	-	"TCONS_NAT_00000043	exon_number "2"
lncNAT_68	Chromosome02	4364743	4370052	+	"TCONS_NAT_00000028	exon_number "1"
lncNAT_69	Chromosome02	4878840	4879534	-	"TCONS_NAT_00000045	exon_number "1"
lncNAT_69	Chromosome02	4922533	4922543	-	"TCONS_NAT_00000045	exon_number "2"
lncNAT_7	Chromosome04	27389899	27390393	-	"TCONS_NAT_00000104	exon_number "1"
lncNAT_7	Chromosome04	27390642	27390687	-	"TCONS_NAT_00000104	exon_number "2"
lncNAT_7	Chromosome04	27390829	27391610	-	"TCONS_NAT_00000104	exon_number "3"
lncNAT_7	Chromosome04	27391706	27391854	-	"TCONS_NAT_00000104	exon_number "4"
lncNAT_7	Chromosome04	27391898	27391943	-	"TCONS_NAT_00000104	exon_number "5"
lncNAT_70	Chromosome02	5635531	5635573	+	"TCONS_NAT_00000030	exon_number "1"

lncNAT_70	Chromosome02	5635601	5637552	+	"TCONS_NAT_00000030	exon_number "2"
lncNAT_71	Chromosome02	8329413	8329646	+	"TCONS_NAT_00000032	exon_number "1"
lncNAT_71	Chromosome02	8329806	8329963	+	"TCONS_NAT_00000032	exon_number "2"
lncNAT_71	Chromosome02	8331661	8333404	+	"TCONS_NAT_00000032	exon_number "3"
lncNAT_71	Chromosome02	8329413	8332075	+	"TCONS_NAT_00000031	exon_number "1"
lncNAT_71	Chromosome02	8332301	8333404	+	"TCONS_NAT_00000031	exon_number "2"
lncNAT_72	Chromosome03	1244980	1246142	-	"TCONS_NAT_00000078	exon_number "1"
lncNAT_72	Chromosome03	1244980	1245420	-	"TCONS_NAT_00000079	exon_number "1"
lncNAT_72	Chromosome03	1247345	1247991	-	"TCONS_NAT_00000079	exon_number "2"
lncNAT_72	Chromosome03	1247341	1247710	-	"TCONS_NAT_00000080	exon_number "1"
lncNAT_72	Chromosome03	1248472	1248704	-	"TCONS_NAT_00000080	exon_number "2"
lncNAT_73	Chromosome03	1384922	1386956	+	"TCONS_NAT_00000057	exon_number "1"
lncNAT_73	Chromosome03	1387080	1387149	+	"TCONS_NAT_00000057	exon_number "2"
lncNAT_74	Chromosome03	1484903	1486264	+	"TCONS_NAT_00000058	exon_number "1"
lncNAT_74	Chromosome03	1486352	1488926	+	"TCONS_NAT_00000058	exon_number "2"
lncNAT_75	Chromosome03	15714319	15714457	-	"TCONS_NAT_00000083	exon_number "1"
lncNAT_75	Chromosome03	15719066	15719407	-	"TCONS_NAT_00000083	exon_number "2"
lncNAT_76	Chromosome03	18161672	18163376	-	"TCONS_NAT_00000084	exon_number "1"
lncNAT_76	Chromosome03	18163736	18165529	-	"TCONS_NAT_00000084	exon_number "2"
lncNAT_77	Chromosome03	2533883	2534067	+	"TCONS_NAT_00000059	exon_number "1"
lncNAT_77	Chromosome03	2534195	2534292	+	"TCONS_NAT_00000059	exon_number "2"
lncNAT_77	Chromosome03	2534363	2534487	+	"TCONS_NAT_00000059	exon_number "3"
lncNAT_77	Chromosome03	2540167	2540259	+	"TCONS_NAT_00000059	exon_number "4"
lncNAT_77	Chromosome03	2540361	2540477	+	"TCONS_NAT_00000059	exon_number "5"
lncNAT_77	Chromosome03	2540562	2540568	+	"TCONS_NAT_00000059	exon_number "6"
lncNAT_77	Chromosome03	2533912	2534067	+	"TCONS_NAT_00000060	exon_number "1"
lncNAT_77	Chromosome03	2534195	2534292	+	"TCONS_NAT_00000060	exon_number "2"
lncNAT_77	Chromosome03	2534363	2534487	+	"TCONS_NAT_00000060	exon_number "3"
lncNAT_77	Chromosome03	2534589	2534701	+	"TCONS_NAT_00000060	exon_number "4"
lncNAT_77	Chromosome03	2540171	2540259	+	"TCONS_NAT_00000060	exon_number "5"
lncNAT_77	Chromosome03	2540361	2540645	+	"TCONS_NAT_00000060	exon_number "6"
lncNAT_77	Chromosome03	2551901	2552583	+	"TCONS_NAT_00000060	exon_number "7"
lncNAT_78	Chromosome03	25404963	25406105	+	"TCONS_NAT_00000066	exon_number "1"
lncNAT_78	Chromosome03	25406825	25407941	+	"TCONS_NAT_00000066	exon_number "2"
lncNAT_78	Chromosome03	25405077	25407003	+	"TCONS_NAT_00000067	exon_number "1"
lncNAT_78	Chromosome03	25407780	25407906	+	"TCONS_NAT_00000067	exon_number "2"
lncNAT_78	Chromosome03	25408238	25409051	+	"TCONS_NAT_00000067	exon_number "3"
lncNAT_79	Chromosome03	26572380	26573187	-	"TCONS_NAT_00000085	exon_number "1"
lncNAT_79	Chromosome03	26573344	26573728	-	"TCONS_NAT_00000085	exon_number "2"
lncNAT_79	Chromosome03	26575382	26575412	-	"TCONS_NAT_00000085	exon_number "3"
lncNAT_8	Chromosome04	947390	947885	-	"TCONS_NAT_00000095	exon_number "1"
lncNAT_8	Chromosome04	957236	958734	-	"TCONS_NAT_00000095	exon_number "2"
lncNAT_80	Chromosome03	26901226	26903120	+	"TCONS_NAT_00000071	exon_number "1"
lncNAT_80	Chromosome03	26906601	26906758	+	"TCONS_NAT_00000071	exon_number "2"
lncNAT_80	Chromosome03	26907582	26907602	+	"TCONS_NAT_00000071	exon_number "3"
lncNAT_80	Chromosome03	26901227	26903996	+	"TCONS_NAT_00000072	exon_number "1"
lncNAT_81	Chromosome03	27372692	27373924	+	"TCONS_NAT_00000074	exon_number "1"
lncNAT_81	Chromosome03	27374313	27376011	+	"TCONS_NAT_00000074	exon_number "2"
lncNAT_81	Chromosome03	27372868	27376011	+	"TCONS_NAT_00000075	exon_number "1"
lncNAT_82	Chromosome03	28374411	28375441	+	"TCONS_NAT_00000076	exon_number "1"
lncNAT_82	Chromosome03	28375655	28375805	+	"TCONS_NAT_00000076	exon_number "2"
lncNAT_82	Chromosome03	28376388	28377095	+	"TCONS_NAT_00000076	exon_number "3"
lncNAT_83	Chromosome03	28584953	28584961	-	"TCONS_NAT_00000088	exon_number "1"
lncNAT_83	Chromosome03	28596177	28596660	-	"TCONS_NAT_00000088	exon_number "2"
lncNAT_84	Chromosome03	3057472	3057538	+	"TCONS_NAT_00000062	exon_number "1"
lncNAT_84	Chromosome03	3060442	3062279	+	"TCONS_NAT_00000062	exon_number "2"
lncNAT_84	Chromosome03	3059587	3062279	+	"TCONS_NAT_00000063	exon_number "1"

lncNAT_85	Chromosome03	551023	551868	+	"TCONS_NAT_00000055	exon_number "1"
lncNAT_85	Chromosome03	552025	552263	+	"TCONS_NAT_00000055	exon_number "2"
lncNAT_85	Chromosome03	552347	553397	+	"TCONS_NAT_00000055	exon_number "3"
lncNAT_85	Chromosome03	551044	552263	+	"TCONS_NAT_00000056	exon_number "1"
lncNAT_85	Chromosome03	552347	552452	+	"TCONS_NAT_00000056	exon_number "2"
lncNAT_85	Chromosome03	552837	553397	+	"TCONS_NAT_00000056	exon_number "3"
lncNAT_86	Chromosome03	8745956	8746956	+	"TCONS_NAT_00000064	exon_number "1"
lncNAT_86	Chromosome03	8761521	8761531	+	"TCONS_NAT_00000064	exon_number "2"
lncNAT_87	Chromosome13	10945817	10946613	-	"TCONS_NAT_00000318	exon_number "1"
lncNAT_87	Chromosome13	10956454	10956911	-	"TCONS_NAT_00000318	exon_number "2"
lncNAT_88	Chromosome13	135124	135554	+	"TCONS_NAT_00000298	exon_number "1"
lncNAT_88	Chromosome13	203785	203819	+	"TCONS_NAT_00000298	exon_number "2"
lncNAT_89	Chromosome13	2187429	2188841	+	"TCONS_NAT_00000301	exon_number "1"
lncNAT_89	Chromosome13	2189149	2189232	+	"TCONS_NAT_00000301	exon_number "2"
lncNAT_89	Chromosome13	2189345	2189733	+	"TCONS_NAT_00000301	exon_number "3"
lncNAT_9	Chromosome05	21641619	21642035	+	"TCONS_NAT_00000110	exon_number "1"
lncNAT_9	Chromosome05	21642125	21643654	+	"TCONS_NAT_00000110	exon_number "2"
lncNAT_90	Chromosome13	22049817	22050385	-	"TCONS_NAT_00000320	exon_number "1"
lncNAT_90	Chromosome13	22103986	22104002	-	"TCONS_NAT_00000320	exon_number "2"
lncNAT_91	Chromosome13	2418293	2418958	-	"TCONS_NAT_00000314	exon_number "1"
lncNAT_91	Chromosome13	2420087	2422337	-	"TCONS_NAT_00000314	exon_number "2"
lncNAT_91	Chromosome13	2420038	2420479	-	"TCONS_NAT_00000315	exon_number "1"
lncNAT_91	Chromosome13	2422236	2422364	-	"TCONS_NAT_00000315	exon_number "2"
lncNAT_92	Chromosome13	24316576	24317480	-	"TCONS_NAT_00000321	exon_number "1"
lncNAT_92	Chromosome13	24316576	24317075	-	"TCONS_NAT_00000322	exon_number "1"
lncNAT_92	Chromosome13	24317965	24318062	-	"TCONS_NAT_00000322	exon_number "2"
lncNAT_92	Chromosome13	24318146	24318247	-	"TCONS_NAT_00000322	exon_number "3"
lncNAT_92	Chromosome13	24323548	24324239	-	"TCONS_NAT_00000322	exon_number "4"
lncNAT_93	Chromosome13	24697205	24697291	-	"TCONS_NAT_00000323	exon_number "1"
lncNAT_93	Chromosome13	24698493	24699710	-	"TCONS_NAT_00000323	exon_number "2"
lncNAT_94	Chromosome13	24961601	24961867	+	"TCONS_NAT_00000305	exon_number "1"
lncNAT_94	Chromosome13	24962234	24963151	+	"TCONS_NAT_00000305	exon_number "2"
lncNAT_94	Chromosome13	24963535	24963704	+	"TCONS_NAT_00000305	exon_number "3"
lncNAT_94	Chromosome13	24964479	24965495	+	"TCONS_NAT_00000305	exon_number "4"
lncNAT_94	Chromosome13	24965727	24965861	+	"TCONS_NAT_00000305	exon_number "5"
lncNAT_94	Chromosome13	24965950	24966055	+	"TCONS_NAT_00000305	exon_number "6"
lncNAT_94	Chromosome13	24973009	24973186	+	"TCONS_NAT_00000305	exon_number "7"
lncNAT_94	Chromosome13	24974027	24974995	+	"TCONS_NAT_00000305	exon_number "8"
lncNAT_94	Chromosome13	24977484	24978661	+	"TCONS_NAT_00000305	exon_number "9"
lncNAT_94	Chromosome13	24961602	24963151	+	"TCONS_NAT_00000306	exon_number "1"
lncNAT_94	Chromosome13	24963535	24964932	+	"TCONS_NAT_00000306	exon_number "2"
lncNAT_94	Chromosome13	24965516	24965861	+	"TCONS_NAT_00000307	exon_number "1"
lncNAT_94	Chromosome13	24965950	24966055	+	"TCONS_NAT_00000307	exon_number "2"
lncNAT_94	Chromosome13	24973009	24973186	+	"TCONS_NAT_00000307	exon_number "3"
lncNAT_94	Chromosome13	24974027	24974995	+	"TCONS_NAT_00000307	exon_number "4"
lncNAT_94	Chromosome13	24977484	24978661	+	"TCONS_NAT_00000307	exon_number "5"
lncNAT_94	Chromosome13	24973863	24975507	+	"TCONS_NAT_00000308	exon_number "1"
lncNAT_94	Chromosome13	24975652	24978661	+	"TCONS_NAT_00000309	exon_number "1"
lncNAT_95	Chromosome13	24983330	24983864	-	"TCONS_NAT_00000324	exon_number "1"
lncNAT_95	Chromosome13	24985806	24985971	-	"TCONS_NAT_00000324	exon_number "2"
lncNAT_95	Chromosome13	24986304	24987314	-	"TCONS_NAT_00000324	exon_number "3"
lncNAT_95	Chromosome13	24984141	24984648	-	"TCONS_NAT_00000325	exon_number "1"
lncNAT_95	Chromosome13	24984728	24984956	-	"TCONS_NAT_00000325	exon_number "2"
lncNAT_95	Chromosome13	24985806	24985971	-	"TCONS_NAT_00000325	exon_number "3"
lncNAT_95	Chromosome13	24986304	24987309	-	"TCONS_NAT_00000325	exon_number "4"
lncNAT_95	Chromosome13	24985068	24985855	-	"TCONS_NAT_00000326	exon_number "1"
lncNAT_96	Chromosome13	26649344	26649914	-	"TCONS_NAT_00000327	exon_number "1"

lncNAT_96	Chromosome13	26650806	26651651	-	"TCONS_NAT_00000327	exon_number "2"
lncNAT_97	Chromosome13	28022154	28022629	-	"TCONS_NAT_00000328	exon_number "1"
lncNAT_97	Chromosome13	28027423	28027791	-	"TCONS_NAT_00000328	exon_number "2"
lncNAT_98	Chromosome13	28032949	28033165	-	"TCONS_NAT_00000329	exon_number "1"
lncNAT_98	Chromosome13	28035213	28035510	-	"TCONS_NAT_00000329	exon_number "2"
lncNAT_99	Chromosome13	4650494	4650520	-	"TCONS_NAT_00000316	exon_number "1"
lncNAT_99	Chromosome13	4652175	4652952	-	"TCONS_NAT_00000316	exon_number "2"
lncNAT_99	Chromosome13	4651115	4652878	-	"TCONS_NAT_00000317	exon_number "1"

Supplemental Data S2. Expression levels of lncRNAs under cold conditions

Gene	Cold_norma	ck_normali	FoldChange	Log2FoldCha	pval	padj	Up/Down	Significar
**Novel lncRNAs**								
lincRNA_10	134.77959	146.5936	0.9194098	-0.12121998	0.7122	0.79	down	no
lincRNA_100	11.635149	9.297433	1.2514367	0.323585318	0.5907	0.6938	up	no
lincRNA_101	0.4775083	0.290121	1.6458917	0.718869409	1	1	up	no
lincRNA_102	86.598276	78.73174	1.0999157	0.137393004	0.596	0.6954	up	no
lincRNA_103	189.59882	99.24207	1.9104681	0.933926202	######	######	up	no
lincRNA_106	9.0608211	12.74231	0.7110813	-0.49191361	0.5436	0.6499	down	no
lincRNA_108	139.02235	142.7416	0.973944	-0.03808922	0.9542	0.9816	down	no
lincRNA_11	9.5321655	10.02616	0.9507293	-0.07289347	0.9807	0.9947	down	no
lincRNA_111	24.620463	33.48987	0.7351615	-0.4438668	0.2828	0.3904	down	no
lincRNA_113	749.67265	514.1475	1.4580888	0.544078581	0.0012	0.0034	up	no
lincRNA_114	4.3835089	0.507092	8.6444025	3.111766253	0.1322	0.2052	up	no
lincRNA_115	115.17353	174.6676	0.6593868	-0.60080316	0.0158	0.0333	down	no
lincRNA_116	39.086924	46.35878	0.8431396	-0.24615657	0.6371	0.7221	down	no
lincRNA_117	83.536538	36.94959	2.2608248	1.17684917	0.0254	0.0508	up	no
lincRNA_118	268.87424	276.1957	0.9734918	-0.03875923	0.9374	0.9717	down	no
lincRNA_12	41055.792	25090.08	1.6363354	0.710468457	0.1014	0.1698	up	no
lincRNA_120	51.935908	107.4608	0.483301	-1.04900613	0.3886	0.4985	down	no
lincRNA_122	284.81329	159.1434	1.789665	0.839689543	0.0043	0.0103	up	no
lincRNA_125	14.215478	13.52146	1.0513268	0.072211176	1	1	up	no
lincRNA_126	228.61797	146.852	1.5567921	0.638576295	0.0418	0.08	up	no
lincRNA_127	12.410034	16.92977	0.7330303	-0.44805528	0.6159	0.7098	down	no
lincRNA_129	43.173548	25.65629	1.6827665	0.750835044	0.0187	0.039	up	no
lincRNA_130	267.07742	310.3134	0.8606699	-0.21646815	0.333	0.4432	down	no
lincRNA_131	0	0	NA	NA	NA	NA	--	no
lincRNA_132	198.74124	142.7214	1.3925116	0.477689388	0.2247	0.327	up	no
lincRNA_133	15.467871	22.53344	0.6864407	-0.54279305	0.2848	0.3904	down	no
lincRNA_137	54.040631	87.58258	0.6170249	-0.69659934	0.022	0.0453	down	no
lincRNA_14	200.98082	154.7956	1.298363	0.376693761	0.0812	0.142	up	no
lincRNA_143	11.268231	5.950616	1.8936243	0.921150131	0.4186	0.5291	up	no
lincRNA_144	28.786693	36.61009	0.7863049	-0.34683919	0.378	0.4887	down	no
lincRNA_146	75.060897	111.3442	0.6741336	-0.56889352	0.0411	0.0792	down	no
lincRNA_147	126.737	66.01523	1.9198147	0.940967065	0.0002	0.0005	up	no
lincRNA_148	27.816267	30.06772	0.9251207	-0.11228643	0.9019	0.945	down	no
lincRNA_15	38.181219	45.82767	0.8331477	-0.26335578	0.4722	0.581	down	no
lincRNA_151	1830.3873	1636.808	1.1182664	0.161263925	0.3672	0.4783	up	no
lincRNA_152	0	1.886881	#NAME?	#NAME?	0.1268	0.1991	down	no
lincRNA_153	8.9568865	5.784215	1.548505	0.630876064	0.3044	0.4139	up	no
lincRNA_154	7.1507879	13.96255	0.5121405	-0.96538852	0.159	0.2398	down	no
lincRNA_155	2210.7659	2742.558	0.8060964	-0.31097575	0.2438	0.3476	down	no
lincRNA_156	10.59128	13.6427	0.7763329	-0.36525269	0.5129	0.6206	down	no
lincRNA_158	30.898431	27.2669	1.1331847	0.180383042	0.65	0.7355	up	no
lincRNA_159	77.399946	97.45633	0.7942014	-0.33242328	0.4589	0.5684	down	no
lincRNA_16	133.71071	157.2455	0.8503309	-0.2339038	0.3311	0.4414	down	no
lincRNA_160	74.794503	108.813	0.6873673	-0.5408468	0.0571	0.1053	down	no
lincRNA_161	435.8061	231.5004	1.8825284	0.912671644	######	######	up	no

lincRNA_162	40.204236	29.25869	1.3740955	0.458482301	0.1652	0.2479	up	no
lincRNA_163	116.15012	185.0735	0.627589	-0.67210807	0.007	0.0159	down	no
lincRNA_168	323.08808	397.7499	0.8122895	-0.29993408	0.1115	0.1827	down	no
lincRNA_169	8.874198	4.219366	2.1032065	1.072590492	0.1157	0.1877	up	no
lincRNA_17	29.493971	19.33526	1.5253984	0.609186108	0.1127	0.1838	up	no
lincRNA_170	13.713805	17.50053	0.7836221	-0.35177002	0.6339	0.7201	down	no
lincRNA_171	155.74484	154.7157	1.0066518	0.009564752	0.939	0.9719	up	no
lincRNA_172	303.72709	451.9947	0.6719705	-0.57353027	0.1169	0.1878	down	no
lincRNA_173	52.127353	47.7485	1.0917067	0.126585268	0.6548	0.7379	up	no
lincRNA_175	65.988076	95.6162	0.6901349	-0.53504969	0.068	0.1236	down	no
lincRNA_176	16.59751	21.45995	0.7734178	-0.37068011	0.5235	0.6311	down	no
lincRNA_178	21.094036	21.99211	0.9591639	-0.06015067	0.9481	0.9785	down	no
lincRNA_179	48.977254	77.76231	0.6298328	-0.66695926	0.1071	0.1779	down	no
lincRNA_180	326.30598	361.846	0.9017813	-0.1491505	0.5887	0.6926	down	no
lincRNA_181	62.590239	118.5828	0.5278187	-0.92188562	0.0021	0.0053	down	no
lincRNA_182	51.399024	39.81963	1.290796	0.368261009	0.2848	0.3904	up	no
lincRNA_183	262.36817	227.6095	1.152712	0.205032063	0.3087	0.4157	up	no
lincRNA_184	34.814169	21.10383	1.6496614	0.722169892	0.0968	0.1649	up	no
lincRNA_186	119.22985	159.9301	0.7455125	-0.42369552	0.0977	0.1652	down	no
lincRNA_188	130.60117	198.27	0.6587035	-0.60229893	0.0131	0.0282	down	no
lincRNA_189	28.40371	50.71194	0.560099	-0.8362462	0.0169	0.0354	down	no
lincRNA_19	22.462168	19.24667	1.1670676	0.222888119	0.6343	0.7201	up	no
lincRNA_190	135.46592	244.6275	0.553764	-0.85265686	0.0003	0.0009	down	no
lincRNA_191	93.381654	140.7475	0.6634694	-0.59189819	0.0287	0.0568	down	no
lincRNA_192	11.727084	17.58302	0.6669552	-0.58433818	0.3202	0.4287	down	no
lincRNA_194	31.409809	34.84226	0.9014859	-0.14962318	0.7778	0.8476	down	no
lincRNA_195	35.585317	65.56029	0.5427876	-0.88154024	0.0066	0.0153	down	no
lincRNA_196	80.332275	44.78391	1.7937755	0.84299932	0.0014	0.0038	up	no
lincRNA_198	26.034496	21.16983	1.2297922	0.298414619	0.5921	0.6942	up	no
lincRNA_199	73.879684	56.48916	1.3078559	0.38720361	0.1419	0.2163	up	no
lincRNA_201	291.0605	190.1489	1.5306979	0.614189615	0.0017	0.0044	up	no
lincRNA_202	23.358987	21.73871	1.0745343	0.103711492	0.7866	0.8531	up	no
lincRNA_205	18.038954	12.78093	1.4113965	0.497123317	0.5716	0.6759	up	no
lincRNA_208	136.11159	105.9991	1.2840828	0.360738251	0.1101	0.1818	up	no
lincRNA_209	104.9591	209.6219	0.5007068	-0.9979619	######	0.0003	down	no
lincRNA_21	5.4457057	1.631002	3.3388707	1.739360235	0.1029	0.1719	up	no
lincRNA_210	39.782366	51.58697	0.7711708	-0.37487769	0.2842	0.3904	down	no
lincRNA_211	14.715051	1.279393	11.501587	3.523761074	0.1171	0.1878	up	no
lincRNA_212	5.3476075	10.47643	0.5104417	-0.97018182	0.2251	0.327	down	no
lincRNA_213	56.520435	30.29387	1.8657385	0.89974677	0.0021	0.0054	up	no
lincRNA_214	21.134756	22.50868	0.9389604	-0.09086382	0.9691	0.9883	down	no
lincRNA_215	71.06329	132.4639	0.5364727	-0.89842339	0.0014	0.0038	down	no
lincRNA_216	132.84146	135.1931	0.9826053	-0.0253161	0.9351	0.9708	down	no
lincRNA_217	2.2959346	0.290121	7.913705	2.984353278	0.0659	0.1204	up	no
lincRNA_218	65.372404	87.29186	0.7488946	-0.41716532	0.1623	0.2442	down	no
lincRNA_219	3.9183283	2.754912	1.422306	0.508231863	0.6607	0.7408	up	no
lincRNA_22	7.1384602	6.327883	1.1280962	0.173890042	0.7914	0.8531	up	no
lincRNA_220	16.695281	13.18777	1.2659669	0.340239718	0.491	0.5986	up	no

lincRNA_222	10.383248	15.80819	0.656827	-0.60641467	0.3489	0.4607	down	no
lincRNA_223	48.900729	40.78618	1.1989535	0.261775678	0.3532	0.4645	up	no
lincRNA_225	127.57415	144.1169	0.885213	-0.17590345	0.4944	0.6014	down	no
lincRNA_226	20.784987	12.6116	1.6480854	0.720790995	0.4024	0.5126	up	no
lincRNA_227	52.816303	69.22101	0.7630098	-0.3902266	0.3265	0.4362	down	no
lincRNA_228	22.22043	24.64633	0.9015716	-0.14948596	0.7821	0.8509	down	no
lincRNA_23	22.970332	27.77399	0.8270447	-0.27396274	0.5438	0.6499	down	no
lincRNA_230	120.10648	140.3816	0.8555711	-0.22504031	0.5846	0.6889	down	no
lincRNA_231	13.254297	5.263128	2.5183308	1.332467795	0.0546	0.1012	up	no
lincRNA_233	124.74949	93.47676	1.3345509	0.416354305	0.2301	0.3322	up	no
lincRNA_235	1.4325249	2.074274	0.6906151	-0.53404631	0.9524	0.9812	down	no
lincRNA_238	46.278105	27.79672	1.6648766	0.735415243	0.0157	0.0333	up	no
lincRNA_239	38.03381	36.20791	1.0504282	0.070977607	0.8985	0.9444	up	no
lincRNA_240	169.72491	260.0889	0.6525649	-0.6158067	0.0056	0.0133	down	no
lincRNA_241	37.578039	55.185	0.6809466	-0.55438651	0.0991	0.1668	down	no
lincRNA_242	15.225773	9.694948	1.5704853	0.651210431	0.1847	0.2736	up	no
lincRNA_243	27.528463	34.49472	0.7980486	-0.32545154	0.4558	0.5655	down	no
lincRNA_244	31.20489	25.07823	1.2443018	0.31533644	0.4025	0.5126	up	no
lincRNA_246	24.427512	22.0125	1.1097108	0.15018376	0.6832	0.7616	up	no
lincRNA_247	13.257543	12.0391	1.1012075	0.139086401	0.7941	0.8531	up	no
lincRNA_249	20.206298	8.385829	2.4095767	1.268779705	0.0604	0.1108	up	no
lincRNA_251	66.701682	36.56026	1.8244311	0.867446693	0.0276	0.0549	up	no
lincRNA_254	38.624498	33.7456	1.1445789	0.194816864	0.6034	0.699	up	no
lincRNA_255	8.2162304	3.127366	2.6272045	1.393528502	0.0594	0.1092	up	no
lincRNA_256	4.8731811	3.593366	1.3561605	0.439527901	0.7593	0.836	up	no
lincRNA_26	27.837513	37.85697	0.7353338	-0.44352879	0.2429	0.3476	down	no
lincRNA_260	10.95269	8.150943	1.3437329	0.426246344	0.4345	0.545	up	no
lincRNA_261	2.8682954	4.65564	0.6160904	-0.69878596	0.5457	0.6502	down	no
lincRNA_262	27.283643	36.40637	0.7494196	-0.41615448	0.2778	0.386	down	no
lincRNA_263	108.46152	104.6857	1.0360678	0.051118367	0.7938	0.8531	up	no
lincRNA_264	26.729938	40.24281	0.6642165	-0.59027459	0.3687	0.4793	down	no
lincRNA_265	426.23561	416.6225	1.023074	0.032910448	1	1	up	no
lincRNA_267	312.89359	232.9999	1.3428918	0.425343054	0.0208	0.0428	up	no
lincRNA_268	1717.2104	968.379	1.7732834	0.826423129	######	######	up	no
lincRNA_27	199.77387	115.0658	1.73617	0.795908193	0.0002	0.0006	up	no
lincRNA_270	10.591444	10.31862	1.0264404	0.037649927	0.9607	0.9868	up	no
lincRNA_271	47.587876	48.1211	0.9889191	-0.01607552	1	1	down	no
lincRNA_273	273.44204	504.9662	0.5415056	-0.88495176	######	######	down	no
lincRNA_274	2.9569841	8.57337	0.3449033	-1.53573599	0.0733	0.1319	down	no
lincRNA_275	74.567815	101.4759	0.7348328	-0.4445121	0.1112	0.1826	down	no
lincRNA_276	6.7619683	8.963887	0.7543567	-0.40668127	0.739	0.8172	down	no
lincRNA_278	8.7639358	15.08646	0.5809139	-0.78360368	0.2393	0.344	down	no
lincRNA_28	120.92258	83.60593	1.4463396	0.532406363	0.1378	0.2111	up	no
lincRNA_280	365.0096	412.1286	0.8856692	-0.17516014	0.3556	0.4669	down	no
lincRNA_284	7.4293454	14.11277	0.5264271	-0.92569446	0.3073	0.4147	down	no
lincRNA_287	347.27743	317.33	1.0943732	0.130104744	0.463	0.5713	up	no
lincRNA_289	118.91346	110.0704	1.08034	0.111485404	0.6177	0.7108	up	no
lincRNA_290	89.328735	79.56627	1.122696	0.166967367	0.5056	0.6129	up	no

lincRNA_292	5.2282633	2.503699	2.0882158	1.062270806	0.3739	0.4853	up	no
lincRNA_294	17.160625	22.54684	0.76111	-0.3938231	0.5536	0.6559	down	no
lincRNA_295	30.635775	38.00402	0.8061193	-0.3109347	0.4453	0.5535	down	no
lincRNA_296	23.059184	11.74431	1.9634347	0.973379588	0.0276	0.0549	up	no
lincRNA_299	6.954919	6.725397	1.0341276	0.048414219	0.862	0.9122	up	no
lincRNA_30	552.63596	408.6373	1.3523876	0.435508684	0.0112	0.0244	up	no
lincRNA_300	38.618826	25.07357	1.5402206	0.62313701	0.0686	0.1243	up	no
lincRNA_301	2.1947545	0.870364	2.5216513	1.334368784	0.7598	0.836	up	no
lincRNA_304	21.164233	10.21837	2.0711951	1.050463436	0.0252	0.0506	up	no
lincRNA_305	14.124035	7.808067	1.8089028	0.855114918	0.1032	0.172	up	no
lincRNA_309	25.759184	32.74074	0.7867623	-0.3460002	0.443	0.5527	down	no
lincRNA_31	1.1418035	5.021245	0.2273945	-2.13673066	0.088	0.1515	down	no
lincRNA_313	111.6231	89.98558	1.2404554	0.31086984	0.2648	0.3705	up	no
lincRNA_314	119.68543	137.963	0.8675183	-0.20503392	0.4787	0.5876	down	no
lincRNA_315	240.29771	155.855	1.5418035	0.624618897	0.0025	0.0062	up	no
lincRNA_316	50.911943	54.02829	0.9423201	-0.08571091	0.8193	0.8732	down	no
lincRNA_320	26.218037	11.76441	2.2285894	1.15613085	0.0916	0.1566	up	no
lincRNA_321	10.597444	13.45079	0.7878675	-0.34397503	0.5997	0.6959	down	no
lincRNA_322	309.01057	202.3714	1.5269479	0.610650807	0.0012	0.0033	up	no
lincRNA_323	152.98133	125.5924	1.218078	0.284606493	0.1959	0.2882	up	no
lincRNA_325	53.0305	64.17584	0.8263312	-0.27520793	0.3881	0.4985	down	no
lincRNA_326	0.7653115	0	Inf	Inf	0.2898	0.3965	up	no
lincRNA_327	72.352142	48.13976	1.5029602	0.587806813	0.0302	0.0596	up	no
lincRNA_328	36.821809	28.31532	1.3004198	0.378977443	0.2763	0.385	up	no
lincRNA_329	0.8601641	4.94591	0.1739142	-2.52355213	0.0849	0.1476	down	no
lincRNA_33	8.8495426	5.272458	1.6784472	0.747127148	0.258	0.3624	up	no
lincRNA_330	794.58186	635.6134	1.2501025	0.322046345	0.0495	0.093	up	no
lincRNA_331	113.38584	145.2633	0.7805542	-0.35742936	0.1639	0.2444	down	no
lincRNA_332	46.350893	38.35082	1.2086025	0.273339844	0.3818	0.4927	up	no
lincRNA_333	172.61134	138.8781	1.2428983	0.313708295	0.149	0.2256	up	no
lincRNA_335	29.380627	34.59278	0.8493282	-0.23560597	0.608	0.7019	down	no
lincRNA_336	9.6364276	6.873883	1.40189	0.487373172	0.4241	0.533	up	no
lincRNA_337	698.59781	714.7708	0.9773732	-0.03301862	0.888	0.9348	down	no
lincRNA_338	24.80384	39.36967	0.6300242	-0.66652096	0.0701	0.1268	down	no
lincRNA_343	281.82369	223.2469	1.2623857	0.336152807	0.0749	0.1333	up	no
lincRNA_344	134.1834	173.448	0.7736232	-0.37029697	0.1277	0.1997	down	no
lincRNA_345	39.74181	40.32003	0.9856593	-0.02083911	0.9641	0.9874	down	no
lincRNA_348	19.046167	20.71738	0.9193329	-0.12134079	0.9227	0.9609	down	no
lincRNA_349	10.762821	7.232489	1.4881213	0.573492095	0.3033	0.4134	up	no
lincRNA_350	188.11682	134.2378	1.4013702	0.4868381	0.0723	0.1304	up	no
lincRNA_351	35.193415	51.72772	0.680359	-0.55563194	0.0981	0.1655	down	no
lincRNA_352	44.820433	88.49472	0.5064758	-0.9814348	0.0254	0.0508	down	no
lincRNA_353	22.033479	31.26358	0.704765	-0.50478575	0.399	0.51	down	no
lincRNA_355	134.40749	159.8483	0.840844	-0.25008994	0.344	0.456	down	no
lincRNA_356	28.826267	48.70119	0.5919006	-0.75657313	0.0249	0.0503	down	no
lincRNA_357	45.389712	44.99358	1.0088041	0.012646026	1	1	up	no
lincRNA_359	43.176957	71.94123	0.6001699	-0.73655719	0.0233	0.0474	down	no
lincRNA_36	28.149644	24.34747	1.1561631	0.209344905	0.7086	0.7873	up	no

lincRNA_361	40.886695	58.92437	0.6938842	-0.52723307	0.0976	0.1652	down	no
lincRNA_362	56.85673	60.58292	0.9384944	-0.09157994	0.8725	0.9213	down	no
lincRNA_365	76.42952	42.66591	1.7913485	0.841046065	0.0018	0.0046	up	no
lincRNA_366	25.082889	11.60674	2.1610621	1.111740514	0.1124	0.1838	up	no
lincRNA_367	21.859675	21.67474	1.0085323	0.012257242	0.9209	0.9604	up	no
lincRNA_368	181.06386	290.2396	0.6238428	-0.68074561	0.0021	0.0053	down	no
lincRNA_369	169.03678	139.1722	1.2145875	0.280466383	0.1846	0.2736	up	no
lincRNA_37	336.8474	413.551	0.8145245	-0.29596995	0.1277	0.1997	down	no
lincRNA_370	9.7158705	6.142822	1.5816623	0.661441647	0.2809	0.389	up	no
lincRNA_372	17.729741	17.04401	1.0402328	0.056906402	0.9017	0.945	up	no
lincRNA_373	83.912998	72.24882	1.1614446	0.215920286	0.4352	0.545	up	no
lincRNA_374	26.401742	33.25657	0.7938805	-0.33300621	0.4213	0.5315	down	no
lincRNA_375	61.078239	67.27407	0.9079016	-0.13939213	0.8071	0.8629	down	no
lincRNA_379	92.227686	111.3367	0.8283674	-0.2716574	0.3519	0.4637	down	no
lincRNA_38	7.8270833	8.46831	0.9242793	-0.11359924	0.914	0.9547	down	no
lincRNA_380	267.09585	251.5239	1.0619104	0.086662051	0.6539	0.7379	up	no
lincRNA_381	39.751547	33.95587	1.1706827	0.227350114	0.4143	0.5262	up	no
lincRNA_382	0.3795738	0.580243	0.654164	-0.61227579	1	1	down	no
lincRNA_386	4.4876072	3.344337	1.3418526	0.424226165	0.734	0.8129	up	no
lincRNA_39	30.752037	37.85553	0.8123525	-0.29982227	0.4912	0.5986	down	no
lincRNA_390	94.874311	167.6188	0.5660124	-0.82109434	0.0537	0.1	down	no
lincRNA_392	15.149235	20.81512	0.7277994	-0.45838721	0.3846	0.4952	down	no
lincRNA_394	68.5545	25.44925	2.6937732	1.429628402	0.0361	0.0704	up	no
lincRNA_396	51.107975	63.48384	0.8050549	-0.31284085	0.3461	0.4579	down	no
lincRNA_398	85.691555	61.80053	1.3865829	0.471533895	0.0796	0.1399	up	no
lincRNA_399	315.33369	412.6932	0.7640875	-0.38819021	0.0461	0.0877	down	no
lincRNA_40	76.395487	70.47866	1.083952	0.116300888	0.694	0.7723	up	no
lincRNA_404	48.138663	38.71176	1.2435154	0.314424359	0.2847	0.3904	up	no
lincRNA_405	44.777286	29.87551	1.4987958	0.583803816	0.0677	0.1235	up	no
lincRNA_407	12.382133	7.300827	1.6959905	0.762128112	0.2498	0.3534	up	no
lincRNA_408	6.8754761	4.244279	1.6199399	0.695940246	0.3605	0.4723	up	no
lincRNA_409	47.158991	36.89802	1.2780899	0.353989269	0.2287	0.3316	up	no
lincRNA_41	22.866561	37.55856	0.6088242	-0.7159025	0.1561	0.2359	down	no
lincRNA_410	5.8129518	4.943577	1.1758594	0.233715607	0.8626	0.9122	up	no
lincRNA_413	165.74301	139.6552	1.1868016	0.247078754	0.2367	0.341	up	no
lincRNA_416	71.234831	83.8107	0.8499491	-0.23455156	0.4623	0.5713	down	no
lincRNA_417	12.11574	15.6004	0.7766299	-0.36470087	0.5195	0.6275	down	no
lincRNA_42	26.998595	39.87239	0.6771251	-0.56250573	0.1346	0.2076	down	no
lincRNA_421	44.814597	47.65525	0.9403917	-0.08866635	0.8476	0.9006	down	no
lincRNA_423	78.171094	114.1639	0.6847271	-0.54639899	0.0551	0.1018	down	no
lincRNA_424	22.13125	10.75737	2.0573104	1.040759495	0.0298	0.0589	up	no
lincRNA_425	158.68321	230.9332	0.6871391	-0.54132601	0.0152	0.0324	down	no
lincRNA_426	39.013973	32.07524	1.2163268	0.282530894	0.3337	0.4432	up	no
lincRNA_427	4.1049514	9.760953	0.4205482	-1.24965684	0.1667	0.2492	down	no
lincRNA_428	1215.3055	1003.445	1.2111335	0.27635785	0.1011	0.1697	up	no
lincRNA_429	15.933215	9.338674	1.706154	0.770747852	0.1174	0.1878	up	no
lincRNA_43	3.1499348	5.176728	0.6084799	-0.71671841	0.5982	0.6954	down	no
lincRNA_430	199.36803	297.5095	0.6701232	-0.57750183	0.0066	0.0153	down	no

lincRNA_432	45.661778	72.03071	0.633921	-0.65762499	0.0462	0.0877	down	no
lincRNA_433	15.15233	16.47762	0.9195705	-0.12096793	0.8522	0.9041	down	no
lincRNA_434	68.145715	122.0856	0.5581799	-0.84119802	0.0023	0.0059	down	no
lincRNA_435	228.83748	249.9307	0.9156039	-0.12720454	0.68	0.7606	down	no
lincRNA_436	8.384362	15.87479	0.5281556	-0.92096499	0.2416	0.3466	down	no
lincRNA_437	166.86003	131.8658	1.2653777	0.339568031	0.1196	0.1896	up	no
lincRNA_438	28.217414	30.10414	0.9373266	-0.09337623	0.9067	0.9486	down	no
lincRNA_44	3.0458365	5.848036	0.5208307	-0.94111362	0.4148	0.5262	down	no
lincRNA_440	188.87311	283.0428	0.6672952	-0.58360298	0.0228	0.0466	down	no
lincRNA_441	216.95636	154.6504	1.4028822	0.488393908	0.02	0.0416	up	no
lincRNA_442	118.36661	108.1129	1.094843	0.130724046	0.5469	0.6502	up	no
lincRNA_443	13.342986	24.95684	0.5346424	-0.90335395	0.1166	0.1878	down	no
lincRNA_444	318.84175	477.9982	0.6670355	-0.58416449	0.0036	0.0088	down	no
lincRNA_446	45.750467	79.97996	0.5720241	-0.80585217	0.0076	0.0174	down	no
lincRNA_447	369.13301	250.8532	1.4715102	0.557297496	0.0087	0.0197	up	no
lincRNA_448	107.70205	58.77122	1.8325643	0.873863842	0.0006	0.0016	up	no
lincRNA_450	231.58925	298.3765	0.7761644	-0.36556575	0.0794	0.1399	down	no
lincRNA_451	4.4783614	6.366791	0.7033939	-0.50759535	0.6243	0.715	down	no
lincRNA_452	470.4128	249.7118	1.8838232	0.91366356	######	######	up	no
lincRNA_46	10.401739	8.566373	1.2142524	0.280068383	0.5938	0.695	up	no
lincRNA_47	8.5893129	7.054278	1.2176033	0.284044229	0.7675	0.841	up	no
lincRNA_5	15.063478	20.91817	0.7201145	-0.47370174	0.3656	0.4772	down	no
lincRNA_50	13.640199	19.18444	0.7110032	-0.49207195	0.3646	0.4768	down	no
lincRNA_51	97.961523	67.57104	1.449756	0.535810066	0.0324	0.0633	up	no
lincRNA_52	114.20635	107.4137	1.0632381	0.088464716	0.6554	0.7379	up	no
lincRNA_55	46.139614	36.41803	1.2669443	0.341353059	0.2782	0.386	up	no
lincRNA_58	25.455808	23.39448	1.0881119	0.121826945	0.777	0.8476	up	no
lincRNA_59	261.24093	376.9124	0.6931077	-0.52884847	0.0102	0.0226	down	no
lincRNA_60	473.0088	324.6476	1.4569917	0.542992693	0.0023	0.0059	up	no
lincRNA_61	12.375969	7.013186	1.7646714	0.819399603	0.131	0.2038	up	no
lincRNA_64	46107.378	32667.45	1.4114163	0.497143578	0.1969	0.2885	up	no
lincRNA_65	180.46698	143.4813	1.2577738	0.330872507	0.109	0.1804	up	no
lincRNA_66	61.923353	45.18286	1.3705053	0.454707954	0.1345	0.2076	up	no
lincRNA_67	90.221225	128.5219	0.701991	-0.51047547	0.0802	0.1406	down	no
lincRNA_68	40.237646	57.04712	0.7053405	-0.50360814	0.2125	0.3108	down	no
lincRNA_69	40.014367	48.46571	0.8256222	-0.2764463	0.4166	0.5275	down	no
lincRNA_72	4.8702629	12.99323	0.3748308	-1.41568872	0.1171	0.1878	down	no
lincRNA_73	92.310539	75.81727	1.2175398	0.283968885	0.3876	0.4985	up	no
lincRNA_74	703.41333	609.9614	1.1532096	0.205654799	0.2292	0.3316	up	no
lincRNA_76	81.826079	71.4385	1.1454059	0.195858942	0.552	0.6551	up	no
lincRNA_78	36.154432	59.97672	0.6028077	-0.73023015	0.0231	0.0472	down	no
lincRNA_79	983.09813	799.3067	1.2299385	0.298586204	0.0747	0.1333	up	no
lincRNA_8	18.403445	23.99105	0.7670964	-0.38252017	0.4806	0.5888	down	no
lincRNA_80	726.023	481.9237	1.5065104	0.591210588	0.0005	0.0014	up	no
lincRNA_81	68.978438	38.05851	1.8124314	0.857926359	0.0017	0.0044	up	no
lincRNA_82	15968.004	11999.28	1.3307465	0.412235812	0.1253	0.1976	up	no
lincRNA_83	14.004363	10.18398	1.375137	0.459575306	0.4241	0.533	up	no
lincRNA_84	30.611119	46.75937	0.654652	-0.6111998	0.0905	0.1551	down	no

lincRNA_86	146.81878	116.0646	1.2649747	0.339108529	0.1367	0.2099	up	no
lincRNA_87	240.18843	284.8224	0.8432919	-0.24589597	0.253	0.3562	down	no
lincRNA_88	66.110338	48.42288	1.3652706	0.449186918	0.1106	0.1821	up	no
lincRNA_89	59.400567	69.43286	0.8555108	-0.22514198	0.4991	0.606	down	no
lincRNA_9	27.258824	27.65275	0.9857546	-0.02069959	1	1	down	no
lincRNA_90	28.700922	24.72211	1.1609416	0.215295429	0.5357	0.6425	up	no
lincRNA_92	8.6810835	5.528337	1.5702885	0.651029618	0.3936	0.5041	up	no
lincRNA_93	39.944334	29.83908	1.3386583	0.420787742	0.1757	0.2614	up	no
lincRNA_94	48.530237	36.99842	1.311684	0.391420232	0.1901	0.2803	up	no
lincRNA_96	14.987444	33.60585	0.4459773	-1.1649578	0.025	0.0505	down	no
lincRNA_98	161.50544	175.1579	0.9220565	-0.11707297	0.5804	0.6852	down	no
lncNAT_1	72.691815	69.93748	1.0393829	0.055727214	0.7921	0.8531	up	no
lncNAT_101	13.074166	23.04535	0.5673234	-0.81775661	0.1438	0.2183	down	no
lncNAT_103	30.148693	28.87443	1.0441313	0.062303181	0.9622	0.9869	up	no
lncNAT_104	109.68277	150.4672	0.728948	-0.45611222	0.0748	0.1333	down	no
lncNAT_105	3005.1045	1850.238	1.6241715	0.699703995	######	0.0003	up	no
lncNAT_106	35.070989	24.11462	1.4543456	0.540370126	0.1188	0.1888	up	no
lncNAT_108	25.199151	27.826	0.9055973	-0.14305837	0.8062	0.8629	down	no
lncNAT_112	15.792134	26.11996	0.6046003	-0.72594647	0.2606	0.3653	down	no
lncNAT_114	202.11016	155.3449	1.3010413	0.379666708	0.077	0.1363	up	no
lncNAT_115	49.969745	83.09522	0.6013552	-0.73371062	0.0205	0.0425	down	no
lncNAT_116	18.779773	31.54542	0.5953249	-0.74825084	0.0873	0.1507	down	no
lncNAT_117	7.0557716	14.05595	0.5019776	-0.99430518	0.1425	0.2168	down	no
lncNAT_118	64.310207	35.3696	1.8182338	0.862537725	0.0024	0.0059	up	no
lncNAT_12	530.81527	951.2679	0.5580082	-0.84164189	######	######	down	no
lncNAT_120	33.543284	16.74238	2.003496	1.002519598	0.0311	0.0612	up	no
lncNAT_124	113.18661	127.2151	0.8897261	-0.16856683	0.5273	0.6347	down	no
lncNAT_127	21.186134	32.42745	0.6533396	-0.61409502	0.1656	0.2481	down	no
lncNAT_128	507.48713	775.5796	0.6543327	-0.61190371	0.0011	0.003	down	no
lncNAT_129	87.519555	118.0803	0.7411867	-0.43209113	0.1204	0.1903	down	no
lncNAT_130	85.851456	167.3807	0.5129113	-0.96321872	0.0003	0.0008	down	no
lncNAT_131	132.48687	253.4434	0.5227473	-0.93581438	######	0.0003	down	no
lncNAT_133	124.95952	137.5967	0.9081578	-0.13898502	0.9688	0.9883	down	no
lncNAT_134	22.716593	11.9588	1.8995714	0.925673916	0.1181	0.1881	up	no
lncNAT_135	5.5160666	3.682696	1.4978337	0.582877485	0.6612	0.7408	up	no
lncNAT_136	26.619184	14.85316	1.7921559	0.841696142	0.0857	0.1487	up	no
lncNAT_138	69.768241	103.7805	0.6722671	-0.57289358	0.0478	0.09	down	no
lncNAT_139	46.13086	38.33012	1.2035146	0.267253677	0.3103	0.417	up	no
lncNAT_14	198.84495	102.6606	1.9369159	0.953761324	0.0003	0.0008	up	no
lncNAT_140	65.754765	59.45886	1.1058867	0.145203636	0.5981	0.6954	up	no
lncNAT_141	31.18348	18.33959	1.7003373	0.765820929	0.1341	0.2076	up	no
lncNAT_143	404.82288	310.2205	1.3049519	0.383996631	0.0324	0.0633	up	no
lncNAT_146	20.579708	21.87613	0.9407381	-0.08813492	0.9348	0.9708	down	no
lncNAT_150	0	0	NA	NA	NA	NA	--	no
lncNAT_151	4.8610172	2.430548	1.9999673	0.999976436	0.2504	0.3534	up	no
lncNAT_152	165.13612	179.8572	0.9181513	-0.12319626	0.6263	0.715	down	no
lncNAT_154	36.806563	55.42937	0.6640264	-0.59068751	0.0859	0.1487	down	no
lncNAT_157	28.241578	24.61705	1.1472367	0.19816307	0.6261	0.715	up	no

lncNAT_158	74.172668	127.6354	0.5811295	-0.78306849	0.0044	0.0106	down	no
lncNAT_160	59.795386	92.31325	0.6477444	-0.62650355	0.0377	0.073	down	no
lncNAT_17	32.068136	26.6479	1.2034022	0.26711891	0.4416	0.5521	up	no
lncNAT_170	15.847772	7.80107	2.0314871	1.022536219	0.1181	0.1881	up	no
lncNAT_171	127.40569	96.76343	1.3166719	0.396895868	0.0975	0.1652	up	no
lncNAT_172	43.097023	58.3485	0.7386141	-0.43710736	0.1962	0.2882	down	no
lncNAT_174	9.918067	15.59807	0.6358521	-0.65323676	0.2952	0.4031	down	no
lncNAT_176	65.388828	71.2849	0.9172886	-0.12455243	0.7892	0.8531	down	no
lncNAT_177	7.7383946	6.693487	1.1561081	0.209276287	0.7911	0.8531	up	no
lncNAT_178	39.026628	35.67213	1.0940369	0.129661379	0.768	0.841	up	no
lncNAT_18	98.585753	161.3579	0.6109756	-0.71081339	0.0068	0.0156	down	no
lncNAT_180	3.5293449	0.509425	6.9280993	2.792459616	0.0463	0.0877	up	no
lncNAT_182	104.14391	188.7446	0.5517715	-0.85785704	0.0025	0.0061	down	no
lncNAT_183	103.58084	128.8691	0.8037676	-0.31514975	0.2505	0.3534	down	no
lncNAT_185	43.192727	58.18369	0.7423511	-0.42982634	0.2435	0.3476	down	no
lncNAT_187	47.098204	32.56487	1.4462889	0.532355787	0.2447	0.3481	up	no
lncNAT_189	9.8261326	11.73731	0.8371706	-0.25640641	0.7612	0.8362	down	no
lncNAT_19	2513.2172	2735.433	0.9187638	-0.12223406	0.6817	0.7612	down	no
lncNAT_190	28.584333	27.61151	1.0352325	0.04995487	0.8743	0.9217	up	no
lncNAT_191	2.6785904	1.343213	1.9941658	0.995785399	0.3199	0.4287	up	no
lncNAT_192	79.894341	74.25272	1.0759786	0.105649439	0.6585	0.7402	up	no
lncNAT_194	92.526241	94.28758	0.9813195	-0.02720511	0.9814	0.9947	down	no
lncNAT_196	122.12179	161.7819	0.7548543	-0.40572995	0.1073	0.1779	down	no
lncNAT_197	770.91793	921.3749	0.8367038	-0.25721107	0.1735	0.2587	down	no
lncNAT_20	197.68823	162.9465	1.2132091	0.278828182	0.2471	0.3508	up	no
lncNAT_203	26.307053	50.81874	0.5176644	-0.94991093	0.0113	0.0246	down	no
lncNAT_205	15.737183	30.17263	0.5215715	-0.93906306	0.0423	0.0809	down	no
lncNAT_206	34.155546	49.52828	0.689617	-0.53613268	0.1139	0.1854	down	no
lncNAT_207	153.2117	77.82946	1.9685565	0.977138141	######	######	up	no
lncNAT_208	48.404566	32.14199	1.5059605	0.590683921	0.0542	0.1006	up	no
lncNAT_209	35.245972	57.5826	0.6120941	-0.70817456	0.0752	0.1336	down	no
lncNAT_211	119.70703	143.9936	0.8313357	-0.26649695	0.3065	0.4147	down	no
lncNAT_213	56.084404	28.86524	1.9429735	0.958266195	0.0512	0.0959	up	no
lncNAT_214	13.631117	23.15711	0.5886364	-0.76455139	0.1307	0.2038	down	no
lncNAT_215	77.619652	53.88988	1.4403382	0.526407593	0.0532	0.0993	up	no
lncNAT_217	24.996955	20.43425	1.2232868	0.290762692	0.4725	0.581	up	no
lncNAT_219	193.54928	187.7483	1.0308976	0.043901016	0.8363	0.89	up	no
lncNAT_22	92.317194	75.41321	1.2241516	0.291782203	0.2228	0.3251	up	no
lncNAT_223	75.915061	89.22375	0.8508392	-0.23304165	0.445	0.5535	down	no
lncNAT_225	21.856429	13.58295	1.6091074	0.686260627	0.1165	0.1878	up	no
lncNAT_226	11.050625	16.75841	0.6594078	-0.60075721	0.3072	0.4147	down	no
lncNAT_228	37.051743	43.57285	0.8503401	-0.23388814	0.5462	0.6502	down	no
lncNAT_23	1.2428198	0	Inf	Inf	0.136	0.2093	up	no
lncNAT_26	18.712822	33.56287	0.5575453	-0.84283894	0.0744	0.1333	down	no
lncNAT_28	1224.6789	1522.724	0.8042682	-0.31425142	0.0794	0.1399	down	no
lncNAT_30	9.7097066	8.507217	1.1413493	0.190740368	0.7425	0.8196	up	no
lncNAT_32	202.27361	141.1829	1.4327064	0.518742975	0.0128	0.0278	up	no
lncNAT_34	5.3539351	1.557852	3.4367423	1.78104168	0.3053	0.4144	up	no

lncNAT_35	12.19551	25.65848	0.4753014	-1.07308535	0.0374	0.0727	down	no
lncNAT_37	17.368658	14.75044	1.1775014	0.235728788	0.6049	0.6996	up	no
lncNAT_38	133.28251	188.409	0.7074106	-0.49938032	0.0417	0.08	down	no
lncNAT_40	61.096076	108.252	0.5643874	-0.82524222	0.0052	0.0124	down	no
lncNAT_42	45.062171	45.73646	0.9852572	-0.02142774	1	1	down	no
lncNAT_44	83.956505	56.3178	1.4907632	0.576051142	0.0379	0.0733	up	no
lncNAT_47	191.95836	154.0871	1.245778	0.317047017	0.1408	0.2152	up	no
lncNAT_48	497.62713	356.9058	1.3942816	0.479521959	0.0844	0.1471	up	no
lncNAT_54	5453.5833	3559.204	1.5322481	0.615649885	0.0141	0.0301	up	no
lncNAT_55	579.21275	327.5953	1.7680742	0.822178844	######	######	up	no
lncNAT_58	50.076761	77.69527	0.6445278	-0.63368547	0.047	0.0889	down	no
lncNAT_59	135.30248	197.8686	0.6837996	-0.54835448	0.0207	0.0427	down	no
lncNAT_62	1.5244592	0.546	2.7920502	1.481324864	0.3758	0.4868	up	no
lncNAT_65	25.177578	34.5243	0.7292712	-0.45547266	0.27	0.3769	down	no
lncNAT_67	53.89391	60.93964	0.8843818	-0.17725876	0.6278	0.715	down	no
lncNAT_69	9.5415751	11.50635	0.8292446	-0.2701304	0.8049	0.8629	down	no
lncNAT_7	216.8959	138.234	1.5690486	0.649890085	0.0021	0.0053	up	no
lncNAT_70	5.3571808	3.897334	1.3745756	0.458986216	0.5343	0.6419	up	no
lncNAT_71	11.699346	14.41907	0.8113799	-0.30155054	0.7696	0.8414	down	no
lncNAT_72	30.917414	61.06292	0.5063206	-0.98187687	0.0117	0.0254	down	no
lncNAT_74	18.608396	33.96927	0.547801	-0.86827631	0.0902	0.1549	down	no
lncNAT_75	0	0.253546	#NAME?	#NAME?	1	1	down	no
lncNAT_77	220.41308	213.2889	1.0334015	0.047400911	0.8091	0.8637	up	no
lncNAT_78	28.009217	28.26317	0.9910148	-0.01302144	0.9694	0.9883	down	no
lncNAT_8	34.330005	64.53459	0.5319628	-0.91060266	0.0056	0.0133	down	no
lncNAT_82	7.9190176	15.42641	0.5133416	-0.96200885	0.1884	0.2784	down	no
lncNAT_83	112.01126	108.6832	1.030622	0.043515234	0.7899	0.8531	up	no
lncNAT_86	17.925937	10.87395	1.6485219	0.721173048	0.1258	0.198	up	no
lncNAT_87	5.3506894	7.771492	0.6885022	-0.53846679	0.6247	0.715	down	no
lncNAT_88	51.631353	44.87904	1.1504558	0.202205543	0.5981	0.6954	up	no
lncNAT_89	43.792465	87.21101	0.5021437	-0.99382769	0.0068	0.0156	down	no
lncNAT_90	42.684203	44.74966	0.9538441	-0.06817463	0.9779	0.9947	down	no
lncNAT_92	150.22314	152.2534	0.9866653	-0.01936737	0.9815	0.9947	down	no
lncNAT_97	36.607612	43.84461	0.8349399	-0.26025579	0.6274	0.715	down	no
lncNAT_98	37.283744	35.33055	1.0552835	0.077630654	0.9483	0.9785	up	no
lincRNA_1	71.541944	32.57826	2.1960024	1.134879626	######	0.0001	up	yes
lincRNA_104	27.179545	81.68908	0.3327194	-1.58762198	0.0003	0.001	down	yes
lincRNA_105	241.30456	27.34079	8.825807	3.141728196	######	######	up	yes
lincRNA_107	11.552788	0	Inf	Inf	######	######	up	yes
lincRNA_109	65.645812	151.4457	0.433461	-1.20602585	######	######	down	yes
lincRNA_110	119.69894	37.42536	3.1983372	1.677322039	######	######	up	yes
lincRNA_112	40.734137	8.249006	4.9380659	2.303946096	######	######	up	yes
lincRNA_119	41.214564	19.13154	2.1542736	1.10720146	0.0233	0.0474	up	yes
lincRNA_121	46.562335	123.5466	0.3768809	-1.40781958	######	######	down	yes
lincRNA_123	92.803129	14.78701	6.2759899	2.649843038	######	######	up	yes
lincRNA_124	14.674822	2.837245	5.1722086	2.370780467	0.0002	0.0007	up	yes
lincRNA_128	285.23667	132.8056	2.1477754	1.102843146	######	######	up	yes
lincRNA_13	215.28351	44.83592	4.801586	2.263511019	######	######	up	yes

lincRNA_134	286.83149	106.1412	2.7023586	1.434219144	######	######	up	yes
lincRNA_135	122.97385	60.38416	2.036525	1.02610949	######	0.0001	up	yes
lincRNA_136	14.971707	0.762971	19.62291	4.294467129	######	######	up	yes
lincRNA_138	20.641674	0.511757	40.334896	5.333956634	0.0089	0.02	up	yes
lincRNA_139	65.840994	27.35811	2.4066352	1.267017499	######	######	up	yes
lincRNA_140	196.13741	522.6946	0.3752428	-1.41410363	######	######	down	yes
lincRNA_141	256.69529	73.95094	3.4711567	1.795416501	######	######	up	yes
lincRNA_142	17.347249	55.80227	0.3108699	-1.68561696	0.0002	0.0007	down	yes
lincRNA_145	9.3517063	28.4319	0.328916	-1.60420898	0.0018	0.0046	down	yes
lincRNA_149	48.980859	24.12102	2.0306298	1.021927218	0.0012	0.0031	up	yes
lincRNA_150	49.773876	3.093123	16.091785	4.008252432	######	######	up	yes
lincRNA_157	30.34148	3.890337	7.7991908	2.96332445	######	######	up	yes
lincRNA_164	7.0374439	28.16292	0.2498833	-2.00067347	0.013	0.028	down	yes
lincRNA_165	44.604333	0.255879	174.31833	7.445580508	######	######	up	yes
lincRNA_166	35.949809	108.9479	0.3299724	-1.59958288	######	######	down	yes
lincRNA_167	115.75353	22.48347	5.148384	2.364119651	######	######	up	yes
lincRNA_174	67.150798	1.089667	61.625043	5.945444839	######	######	up	yes
lincRNA_177	107.78989	279.1742	0.3861026	-1.37294367	######	######	down	yes
lincRNA_18	59.957354	26.52184	2.2606784	1.176755757	######	0.0002	up	yes
lincRNA_185	65.721159	0.253546	259.20794	8.017966098	######	######	up	yes
lincRNA_187	87.190798	0.253546	343.8854	8.425784058	######	######	up	yes
lincRNA_193	122.04189	43.173	2.8268101	1.499174992	######	######	up	yes
lincRNA_197	19.481215	2.181667	8.9295082	3.15858072	######	######	up	yes
lincRNA_2	123.60543	48.17648	2.5656798	1.359341124	######	######	up	yes
lincRNA_20	35.184497	3.122701	11.267328	3.494073498	######	######	up	yes
lincRNA_200	381.62535	4.573308	83.446242	6.382775177	######	######	up	yes
lincRNA_203	318.43815	112.9237	2.8199413	1.495665144	######	######	up	yes
lincRNA_204	350.48343	121.5896	2.8825127	1.527326949	######	######	up	yes
lincRNA_206	97.826637	44.95279	2.1762084	1.121816734	######	######	up	yes
lincRNA_207	2060.7388	454.8351	4.5307381	2.179746094	######	######	up	yes
lincRNA_221	114.67235	24.36132	4.7071492	2.234853577	######	######	up	yes
lincRNA_224	18.314593	40.71695	0.4498027	-1.15263576	0.0054	0.0129	down	yes
lincRNA_229	81.213849	205.001	0.3961632	-1.33583329	######	######	down	yes
lincRNA_232	163.73281	39.27334	4.1690577	2.059721336	######	######	up	yes
lincRNA_234	185.93071	29.68826	6.262768	2.646800441	######	######	up	yes
lincRNA_236	80.029717	0	Inf	Inf	######	######	up	yes
lincRNA_237	243.56865	1.567182	155.41824	7.280012049	######	######	up	yes
lincRNA_24	17.356658	5.343276	3.2483177	1.699692725	0.0014	0.0038	up	yes
lincRNA_245	28.302693	2.439878	11.600043	3.536058208	0.0002	0.0005	up	yes
lincRNA_248	37.944957	98.3663	0.3857516	-1.37425593	######	######	down	yes
lincRNA_25	24.960299	1.567182	15.926868	3.993390709	######	######	up	yes
lincRNA_250	176.51885	383.0142	0.4608677	-1.11757551	######	######	down	yes
lincRNA_252	79.540048	0.799546	99.481512	6.636356529	######	######	up	yes
lincRNA_253	44.857744	96.96626	0.4626119	-1.11212579	0.0004	0.001	down	yes
lincRNA_257	358.02418	11.51086	31.103158	4.958989147	######	######	up	yes
lincRNA_258	86.674441	27.3533	3.1687017	1.663891838	######	######	up	yes
lincRNA_259	41.478039	106.1314	0.3908178	-1.35543174	######	0.0001	down	yes
lincRNA_266	366.60144	173.6616	2.11101	1.077933397	0.0002	0.0006	up	yes

lincRNA_269	537.64759	262.3674	2.0492167	1.035072541	######	######	up	yes
lincRNA_272	44.321352	91.32909	0.4852928	-1.04307261	0.0007	0.002	down	yes
lincRNA_277	46.158761	5.679155	8.127752	3.022856379	######	0.0001	up	yes
lincRNA_279	11.840592	0.765303	15.471763	3.951565721	######	######	up	yes
lincRNA_281	100.8533	334.2066	0.3017693	-1.72848209	######	######	down	yes
lincRNA_282	3.3458037	15.05674	0.2222131	-2.16998436	0.0049	0.0119	down	yes
lincRNA_283	5.730427	21.24675	0.2697083	-1.89052796	0.0157	0.0333	down	yes
lincRNA_285	25.56607	74.73316	0.3420981	-1.54751821	######	######	down	yes
lincRNA_286	28.171545	2.654517	10.612683	3.407717556	######	######	up	yes
lincRNA_288	79.852012	4.918516	16.234979	4.021033645	######	######	up	yes
lincRNA_29	19.371117	1.599092	12.113822	3.598582257	######	######	up	yes
lincRNA_291	377.92852	173.2444	2.1814759	1.125304516	######	######	up	yes
lincRNA_293	113.3614	3.707609	30.575339	4.934296581	######	######	up	yes
lincRNA_297	14.402592	0.801879	17.961064	4.166800938	######	######	up	yes
lincRNA_298	144.93956	70.56596	2.0539588	1.038407224	######	######	up	yes
lincRNA_3	24.960299	9.007312	2.7711151	1.470466658	0.0011	0.003	up	yes
lincRNA_302	46.234303	115.5105	0.4002606	-1.32098848	0.0105	0.0231	down	yes
lincRNA_303	110.80867	24.90295	4.4496202	2.153682187	0.0016	0.0041	up	yes
lincRNA_306	130.9692	2.145092	61.055281	5.932044192	######	######	up	yes
lincRNA_307	401.33928	949.5054	0.4226824	-1.24235389	######	######	down	yes
lincRNA_308	21.0942	44.86535	0.4701669	-1.08875522	0.0088	0.0197	down	yes
lincRNA_310	263.74024	564.0229	0.4676055	-1.09663614	######	######	down	yes
lincRNA_311	68.333485	13.55337	5.0418059	2.333940586	######	######	up	yes
lincRNA_312	2.09682	20.14428	0.1040901	-3.26409542	######	######	down	yes
lincRNA_317	878.21759	408.7358	2.1486193	1.103409914	######	######	up	yes
lincRNA_318	92.678636	25.27203	3.667242	1.874695462	######	######	up	yes
lincRNA_319	15.936133	1.016517	15.677195	3.970595569	0.0007	0.002	up	yes
lincRNA_32	20.66033	0.290121	71.212722	6.154063087	######	######	up	yes
lincRNA_324	35.848465	3.456395	10.371633	3.374571225	######	######	up	yes
lincRNA_334	87.43778	0.253546	344.85951	8.429864948	######	######	up	yes
lincRNA_339	287.78726	123.2876	2.3342757	1.222974993	######	######	up	yes
lincRNA_34	66.236337	0.507092	130.61992	7.029231078	######	0.0002	up	yes
lincRNA_340	124.20353	305.0002	0.4072244	-1.29610398	######	######	down	yes
lincRNA_341	170.4438	529.9103	0.3216465	-1.63645206	######	######	down	yes
lincRNA_342	119.98769	488.0833	0.2458344	-2.02424105	######	######	down	yes
lincRNA_346	11.249739	0.546	20.60392	4.364846957	######	######	up	yes
lincRNA_347	3.143771	14.68413	0.214093	-2.22369019	0.0029	0.0071	down	yes
lincRNA_35	3.0518366	11.55458	0.2641235	-1.92071565	0.0137	0.0294	down	yes
lincRNA_354	77.763619	184.4716	0.4215479	-1.24623155	######	######	down	yes
lincRNA_358	7.6406239	0	Inf	Inf	######	######	up	yes
lincRNA_360	144.60261	68.70384	2.1047239	1.073630999	######	######	up	yes
lincRNA_363	7.0526897	43.653	0.1615625	-2.62983533	######	######	down	yes
lincRNA_364	73.964565	1.057757	69.925847	6.127753917	######	######	up	yes
lincRNA_371	238.73266	116.5621	2.0481164	1.03429772	######	######	up	yes
lincRNA_376	97.899688	2.110849	46.379289	5.535408797	######	######	up	yes
lincRNA_377	19.006462	41.12439	0.4621701	-1.11350426	0.0088	0.0198	down	yes
lincRNA_378	76.561356	173.5868	0.4410553	-1.18096858	######	######	down	yes
lincRNA_383	63.864369	233.0422	0.2740463	-1.86750829	######	######	down	yes

lincRNA_384	69.559225	280.5781	0.2479139	-2.01208877	######	######	down	yes
lincRNA_385	50.903024	120.882	0.4210969	-1.24777582	######	0.0001	down	yes
lincRNA_387	18.109314	1.126243	16.079408	4.0071424	0.0014	0.0037	up	yes
lincRNA_388	81.066402	0	Inf	Inf	######	######	up	yes
lincRNA_389	93.32877	30.67754	3.0422512	1.605139294	######	######	up	yes
lincRNA_391	77.269289	26.17271	2.9522843	1.561831643	######	######	up	yes
lincRNA_393	0	5.007249	#NAME?	#NAME?	0.0048	0.0115	down	yes
lincRNA_395	60.28555	143.2952	0.4207089	-1.24910578	0.0003	0.0008	down	yes
lincRNA_397	27.056955	2.90573	9.3115853	3.219026813	######	######	up	yes
lincRNA_4	249.5963	107.2081	2.3281477	1.219182604	######	######	up	yes
lincRNA_400	44.492565	0.801879	55.485416	5.794036716	######	######	up	yes
lincRNA_401	8.0169521	54.81925	0.1462434	-2.77355687	######	######	down	yes
lincRNA_402	47.287418	106.3915	0.4444662	-1.1698544	0.0001	0.0004	down	yes
lincRNA_403	2.2928527	0	Inf	Inf	0.017	0.0355	up	yes
lincRNA_406	141.19589	354.3443	0.3984709	-1.32745377	######	######	down	yes
lincRNA_411	139.41701	0	Inf	Inf	######	######	up	yes
lincRNA_412	12.204592	0	Inf	Inf	######	######	up	yes
lincRNA_414	95.352606	222.3768	0.4287884	-1.22166217	######	######	down	yes
lincRNA_415	187.35413	1.308971	143.13088	7.161191122	######	######	up	yes
lincRNA_418	161.7196	358.1505	0.4515409	-1.14707129	######	######	down	yes
lincRNA_419	418.69893	26.25663	15.946406	3.995159385	######	######	up	yes
lincRNA_420	11.932198	30.66354	0.3891331	-1.3616644	0.0052	0.0124	down	yes
lincRNA_422	15.158494	34.67745	0.4371283	-1.19387136	0.0073	0.0166	down	yes
lincRNA_431	30.467807	0	Inf	Inf	######	######	up	yes
lincRNA_439	39.492823	4.509487	8.757719	3.130555167	######	######	up	yes
lincRNA_445	23.650364	2.069609	11.427455	3.514432256	######	######	up	yes
lincRNA_449	210.48013	46.40776	4.5354508	2.181245956	######	######	up	yes
lincRNA_45	11.34751	0.253546	44.755216	5.483983929	######	######	up	yes
lincRNA_453	299.04683	131.3609	2.2765288	1.186835694	######	######	up	yes
lincRNA_48	30.332071	117.0917	0.2590454	-1.94872335	######	######	down	yes
lincRNA_49	37.409907	95.77213	0.3906137	-1.35618544	######	0.0001	down	yes
lincRNA_53	304.91061	112.6295	2.7072004	1.436801663	######	######	up	yes
lincRNA_54	7.7323945	22.57701	0.3424897	-1.54586757	0.0064	0.0148	down	yes
lincRNA_56	61.51296	0.546	112.66111	6.815845797	######	######	up	yes
lincRNA_57	7.7233125	40.9762	0.1884829	-2.40749453	0.0015	0.0039	down	yes
lincRNA_6	54.555287	160.9355	0.3389885	-1.56069174	######	######	down	yes
lincRNA_62	34.652529	1.021182	33.933747	5.084648834	######	0.0001	up	yes
lincRNA_63	50.887615	134.7648	0.3776031	-1.40505746	######	######	down	yes
lincRNA_7	36.050497	77.9577	0.4624366	-1.11267236	0.0005	0.0014	down	yes
lincRNA_70	33.448104	0.255879	130.71864	7.030321083	######	######	up	yes
lincRNA_71	12.204756	32.01142	0.3812626	-1.39114319	0.005	0.0119	down	yes
lincRNA_75	66.431683	485.9583	0.1367024	-2.87088915	######	######	down	yes
lincRNA_77	12.608985	1.057757	11.920491	3.575371796	0.0023	0.0057	up	yes
lincRNA_85	286.65945	134.1339	2.1371148	1.095664406	######	######	up	yes
lincRNA_91	1184.4281	486.4185	2.4349978	1.283920489	######	######	up	yes
lincRNA_95	59.348501	183.2177	0.3239234	-1.62627557	######	######	down	yes
lincRNA_97	8.2129847	21.85514	0.375792	-1.41199383	0.0106	0.0232	down	yes
lincRNA_99	115.28618	49.63334	2.3227568	1.215838128	######	######	up	yes

lncNAT_10	89.77562	253.2938	0.3544328	-1.49641596	######	######	down	yes
lncNAT_100	25.572889	0.509425	50.199546	5.649602414	######	######	up	yes
lncNAT_102	1132.954	47.11639	24.045856	4.587716391	######	######	up	yes
lncNAT_107	21.464364	81.533	0.2632598	-1.92544065	######	######	down	yes
lncNAT_109	209.53416	963.6469	0.2174387	-2.20131909	######	######	down	yes
lncNAT_11	333.72821	44.55542	7.4901817	2.905000713	######	######	up	yes
lncNAT_110	501.54437	9.069397	55.300741	5.789226903	######	######	up	yes
lncNAT_111	35.190333	215.376	0.1633902	-2.61360669	######	######	down	yes
lncNAT_113	52.139025	23.85174	2.1859629	1.128268947	0.0004	0.001	up	yes
lncNAT_119	286.86407	47.54627	6.0333669	2.592963327	######	######	up	yes
lncNAT_121	140.44937	41.43679	3.3894844	1.761065827	######	######	up	yes
lncNAT_122	95.741224	2.142759	44.681274	5.481598431	######	######	up	yes
lncNAT_123	25.037675	0	Inf	Inf	######	######	up	yes
lncNAT_125	26.809381	75.37633	0.3556737	-1.49137373	######	######	down	yes
lncNAT_126	21.286823	68.15665	0.312322	-1.67889369	0.0004	0.0012	down	yes
lncNAT_13	439.40158	196.7981	2.2327537	1.158824102	######	######	up	yes
lncNAT_132	41.793088	88.31289	0.4732388	-1.07935967	0.0005	0.0014	down	yes
lncNAT_137	81.694275	212.7488	0.3839941	-1.3808439	######	######	down	yes
lncNAT_142	69.382503	9.635793	7.2004978	2.84809664	######	######	up	yes
lncNAT_144	150.6953	301.5109	0.4998005	-1.00057567	######	######	down	yes
lncNAT_145	155.97864	34.92271	4.466396	2.159111182	0.0002	0.0005	up	yes
lncNAT_147	25.452562	94.02733	0.2706932	-1.88526927	######	######	down	yes
lncNAT_148	579.12963	221.3044	2.6168919	1.387854325	######	######	up	yes
lncNAT_149	143.72071	317.2556	0.4530124	-1.14237766	0.0005	0.0014	down	yes
lncNAT_15	162.84973	10.46958	15.554559	3.959265628	######	######	up	yes
lncNAT_153	93.690998	46.69163	2.0065907	1.004746394	0.0001	0.0004	up	yes
lncNAT_155	221.87807	19.48434	11.387507	3.509380093	######	######	up	yes
lncNAT_156	21.063544	124.8083	0.1687672	-2.56689362	######	######	down	yes
lncNAT_159	36.25594	122.8691	0.2950778	-1.76083288	######	######	down	yes
lncNAT_16	12.69151	61.7501	0.2055302	-2.28257787	######	######	down	yes
lncNAT_161	10.95869	86.20527	0.1271232	-2.97570063	######	######	down	yes
lncNAT_162	212.80577	20.57589	10.342481	3.370510455	######	######	up	yes
lncNAT_163	70.0485	3.812669	18.37256	4.19948078	######	######	up	yes
lncNAT_164	8.2160666	162.0885	0.0506888	-4.30218995	######	######	down	yes
lncNAT_165	52.784759	1.377456	38.320464	5.260043108	######	######	up	yes
lncNAT_166	55.326598	1.272395	43.482234	5.442354163	######	######	up	yes
lncNAT_167	19.273183	6.613339	2.9142892	1.543144055	0.0031	0.0076	up	yes
lncNAT_168	881.56217	350.6235	2.5142702	1.330139689	######	######	up	yes
lncNAT_169	22.349347	52.10761	0.4289075	-1.22126142	0.0103	0.0227	down	yes
lncNAT_173	316.99515	2.508364	126.37527	6.981570364	######	######	up	yes
lncNAT_175	239.8344	10.05589	23.850148	4.575926326	######	######	up	yes
lncNAT_179	99.334836	31.97018	3.1071092	1.635572951	######	######	up	yes
lncNAT_181	116.72641	23.28271	5.0134367	2.325799904	######	######	up	yes
lncNAT_184	83.005225	356.0635	0.2331192	-2.10086048	######	######	down	yes
lncNAT_186	40.030268	3.705276	10.803585	3.433438258	######	######	up	yes
lncNAT_188	121.11605	3.232427	37.469077	5.227628544	######	######	up	yes
lncNAT_193	10.86692	32.4072	0.3353243	-1.57637122	0.0015	0.0038	down	yes
lncNAT_195	264.23249	4.255941	62.085559	5.95618583	######	######	up	yes

lncNAT_198	38.998563	18.99065	2.053567	1.038131998	0.0062	0.0146	up	yes
lncNAT_199	107.03975	0.507092	211.08539	7.721682935	0.0002	0.0005	up	yes
lncNAT_2	155.88799	315.6015	0.4939393	-1.01759441	######	######	down	yes
lncNAT_200	225.96472	1354.417	0.1668354	-2.58350241	######	######	down	yes
lncNAT_201	18.700167	1.092	17.124697	4.098006537	######	######	up	yes
lncNAT_202	62.769777	1.087335	57.728103	5.851201909	######	######	up	yes
lncNAT_204	28.223087	59.35553	0.4754921	-1.07250679	0.003	0.0073	down	yes
lncNAT_21	745.24049	159.0024	4.6869773	2.228657814	######	######	up	yes
lncNAT_210	27.663217	177.1757	0.1561343	-2.67914028	######	######	down	yes
lncNAT_212	47.407089	116.05	0.4085057	-1.29157185	######	0.0001	down	yes
lncNAT_216	1.1418035	8.032036	0.1421562	-2.81445131	0.0101	0.0226	down	yes
lncNAT_218	10.322296	48.71866	0.2118756	-2.23871051	0.0096	0.0215	down	yes
lncNAT_220	471.36919	116.7927	4.0359465	2.012907035	######	######	up	yes
lncNAT_221	55.728073	118.5824	0.4699521	-1.08941425	0.0016	0.0043	down	yes
lncNAT_222	287.62366	129.0331	2.2290694	1.156441547	######	######	up	yes
lncNAT_224	41.798401	1.055425	39.603397	5.30755229	######	######	up	yes
lncNAT_227	350.83373	723.3397	0.4850193	-1.04388593	######	######	down	yes
lncNAT_229	26.313053	12.71259	2.0698423	1.049520867	0.0156	0.0331	up	yes
lncNAT_24	22.698429	0.580243	39.118856	5.289792287	0.0029	0.0072	up	yes
lncNAT_25	61.710499	0.546	113.0229	6.820471347	######	######	up	yes
lncNAT_27	1.6193117	13.90732	0.116436	-3.1023914	0.0004	0.0011	down	yes
lncNAT_29	86.702538	467.6243	0.1854107	-2.43120367	######	######	down	yes
lncNAT_3	74.818799	23.40366	3.1968849	1.676666799	######	######	up	yes
lncNAT_31	9.2568537	56.71953	0.163204	-2.61525187	######	######	down	yes
lncNAT_33	234.85627	492.6917	0.47668	-1.06890701	######	0.0002	down	yes
lncNAT_36	18.580495	65.58521	0.2833031	-1.81958148	######	######	down	yes
lncNAT_39	92.244804	13.59228	6.7865573	2.762679904	######	######	up	yes
lncNAT_4	86.514502	24.77908	3.4914336	1.80381952	######	######	up	yes
lncNAT_41	140.75792	319.0964	0.4411141	-1.18077612	######	######	down	yes
lncNAT_43	607.69678	240.4833	2.5269816	1.337415181	######	######	up	yes
lncNAT_45	90.725783	8.577887	10.576705	3.402818328	######	######	up	yes
lncNAT_46	44.418037	0.799546	55.554072	5.795820754	######	######	up	yes
lncNAT_49	22.731839	0	Inf	Inf	######	######	up	yes
lncNAT_5	104.14893	0.797214	130.6412	7.029466171	######	######	up	yes
lncNAT_50	12.890624	2.181667	5.9086116	2.562819178	0.0002	0.0006	up	yes
lncNAT_51	9.914985	40.9029	0.242403	-2.04452066	######	######	down	yes
lncNAT_52	82.001061	5.816126	14.098915	3.817512265	######	######	up	yes
lncNAT_53	365.16341	146.0464	2.5003244	1.322115292	0.0078	0.0178	up	yes
lncNAT_56	24.289676	75.2076	0.3229684	-1.63053522	######	######	down	yes
lncNAT_57	24.139872	4.294849	5.6206568	2.490738724	######	0.0003	up	yes
lncNAT_6	129.58939	0.255879	506.44871	8.984272348	######	######	up	yes
lncNAT_60	77.854141	12.32688	6.3158003	2.658965562	######	######	up	yes
lncNAT_61	7896.6645	2787.474	2.8329109	1.502285207	######	######	up	yes
lncNAT_63	143.25897	71.52098	2.0030341	1.002187001	######	######	up	yes
lncNAT_64	245.25771	101.675	2.4121744	1.270334208	######	######	up	yes
lncNAT_66	21.520494	0	Inf	Inf	######	######	up	yes
lncNAT_68	269.66921	1.306638	206.38399	7.68918727	######	######	up	yes
lncNAT_73	223.85663	5.24288	42.697259	5.416071565	######	######	up	yes

lncNAT_76	37.9622	0.801879	47.341583	5.565036053	######	######	up	yes
lncNAT_79	208.6258	20.20547	10.325212	3.36809951	######	######	up	yes
lncNAT_80	29.181349	183.0873	0.1593849	-2.6494133	######	######	down	yes
lncNAT_81	177.92642	34.94266	5.0919543	2.348219463	######	######	up	yes
lncNAT_84	3.1499348	41.04017	0.0767525	-3.70364291	######	######	down	yes
lncNAT_85	9.1589193	42.44924	0.2157617	-2.21248938	######	######	down	yes
lncNAT_9	0.7591477	12.99819	0.0584041	-4.09778666	0.0064	0.015	down	yes
lncNAT_91	25.091807	58.23833	0.430847	-1.21475251	0.0005	0.0014	down	yes
lncNAT_93	7.9253452	0.836121	9.478703	3.244689671	0.0005	0.0014	up	yes
lncNAT_94	131.47176	275.4603	0.4772803	-1.06709128	######	######	down	yes
lncNAT_95	30.37194	135.6086	0.2239676	-2.15863807	######	######	down	yes
lncNAT_96	38.252234	113.3375	0.3375072	-1.56700969	######	######	down	yes
lncNAT_99	2.2928527	25.14905	0.0911705	-3.45528837	0.0002	0.0007	down	yes
**Known lncRNAs**								
Manes.01G00010	1.0855284	0	Inf	Inf	0.2231	0.3682	up	no
Manes.01G00070	14.867452	9.671412	1.5372576	0.620358983	0.3146	0.478	up	no
Manes.01G00080	353.55308	333.9463	1.0587123	0.082310555	0.6327	0.7778	up	no
Manes.01G00090	3.236327	4.304917	0.7517745	-0.41162808	0.8068	0.9182	down	no
Manes.01G00130	12.269358	31.85346	0.3851813	-1.37639047	0.0051	0.0161	down	yes
Manes.01G00230	342.31821	899.7074	0.3804773	-1.39411787	######	1E-05	down	yes
Manes.01G00360	223.2714	156.7977	1.4239456	0.509894026	0.0179	0.0475	up	no
Manes.01G00430	6.07024	4.217163	1.4394132	0.525480827	0.5318	0.6923	up	no
Manes.01G00590	154.66119	195.6659	0.7904349	-0.33928144	0.1556	0.2793	down	no
Manes.01G00670	1.3691932	3.960668	0.3456975	-1.53241786	0.265	0.4194	down	no
Manes.01G00720	1.0753993	1.490747	0.721383	-0.47116276	0.9245	0.9966	down	no
Manes.01G00980	7.1370251	6.638559	1.0750866	0.10445284	0.9491	1	up	no
Manes.01G01040	5.676555	0.307855	18.439083	4.204694999	0.0015	0.0056	up	yes
Manes.01G01160	99.18858	184.2356	0.538379	-0.89330603	0.0014	0.0054	down	no
Manes.01G01190	13.767315	24.36192	0.5651162	-0.82338055	0.1194	0.2264	down	no
Manes.01G01340	3.9484287	4.82074	0.8190503	-0.28797611	0.8663	0.962	down	no
Manes.01G01500	10.869778	7.918502	1.3727063	0.457022946	0.4678	0.6306	up	no
Manes.01G01560	3.6502703	6.996356	0.5217388	-0.9386004	0.3027	0.4643	down	no
Manes.01G01590	1.4039451	1.195024	1.1748261	0.232447254	1	1	up	no
Manes.01G01980	0	1.83783	#NAME?	#NAME?	0.1187	0.2255	down	no
Manes.01G02020	157.7691	153.2548	1.0294559	0.041882055	0.8537	0.9525	up	no
Manes.01G02110	1.7324908	1.527141	1.1344667	0.182014323	1	1	up	no
Manes.01G02480	6.68833	3.363554	1.9884712	0.991659673	0.4927	0.6558	up	no
Manes.01G02580	12.039189	40.08753	0.3003225	-1.7354153	0.0003	0.0013	down	yes
Manes.01G03000	16.915395	13.77766	1.227741	0.296006226	0.632	0.7773	up	no
Manes.01G03220	7.598699	5.749972	1.3215192	0.402197425	0.5993	0.751	up	no
Manes.01G03610	45.241875	18.91192	2.3922411	1.258362799	0.0011	0.0042	up	yes
Manes.01G03960	206.49552	176.3406	1.171004	0.227746013	0.3148	0.4783	up	no
Manes.01G03980	0.7468535	4.861386	0.1536298	-2.70247043	0.1181	0.2246	down	no
Manes.01G03990	0.7468535	1.232835	0.6058016	-0.72308264	0.9924	1	down	no
Manes.01G04030	338.70492	521.9824	0.6488819	-0.62397215	0.1754	0.3068	down	no
Manes.01G04150	268.08919	243.7337	1.0999266	0.137407252	0.483	0.6459	up	no
Manes.01G04160	1.5240944	2.110707	0.7220777	-0.46977405	0.8593	0.9569	down	no
Manes.01G04200	29.766577	30.21682	0.9850995	-0.02165868	1	1	down	no

Manes.01G04210	30.355679	57.90874	0.5241986	-0.93181454	0.0138	0.038	down	no
Manes.01G04290	50.448487	24.96629	2.020664	1.014829454	0.0605	0.1308	up	no
Manes.01G04610	73.354079	119.1832	0.6154733	-0.70023191	0.0154	0.0417	down	no
Manes.01G04690	1507.0469	1084.073	1.3901706	0.475261977	0.0216	0.0556	up	no
Manes.01G04770	24.961389	11.79646	2.1160063	1.081343938	0.0588	0.1279	up	no
Manes.01G04900	29.609171	18.49347	1.6010612	0.679028418	0.144	0.2626	up	no
Manes.01G04920	38.766486	62.88905	0.6164266	-0.69799891	0.0738	0.154	down	no
Manes.01G05010	1.1506677	0	Inf	Inf	0.1951	0.3333	up	no
Manes.01G05200	85.5384	18.6077	4.5969368	2.200672831	######	7E-06	up	yes
Manes.01G05230	57.906433	64.81229	0.8934483	-0.1625438	0.6398	0.784	down	no
Manes.01G05340	396.22082	390.0718	1.0157638	0.022565035	0.9029	0.9887	up	no
Manes.01G05350	17.258435	19.18701	0.8994853	-0.15282831	0.8318	0.9376	down	no
Manes.01G05400	19.239723	16.96207	1.1342789	0.181775471	0.6783	0.8175	up	no
Manes.01G05470	54.276011	28.90411	1.8777954	0.909039848	0.0123	0.0345	up	no
Manes.01G05500	1221.1751	82.81451	14.745907	3.882242641	######	2E-50	up	yes
Manes.01G05510	539.61121	46.82819	11.523214	3.52647129	######	1E-67	up	yes
Manes.01G05520	897.30962	575.7492	1.5585079	0.640165448	######	0.0001	up	no
Manes.01G06490	9.128558	5.116463	1.7841538	0.835240019	0.3374	0.5026	up	no
Manes.01G06630	35.774757	55.24029	0.6476208	-0.62677886	0.1922	0.3297	down	no
Manes.01G07130	8.5944654	24.63559	0.3488638	-1.51926428	0.0063	0.0194	down	yes
Manes.01G07250	5.9299473	0	Inf	Inf	0.0003	0.0013	up	yes
Manes.01G07280	1.8975212	0	Inf	Inf	0.0648	0.1384	up	no
Manes.01G07760	13.677553	7.897074	1.7319774	0.792420105	0.158	0.2827	up	no
Manes.01G08040	38.064514	76.91882	0.4948661	-1.01488999	0.0029	0.0099	down	yes
Manes.01G08120	296.67834	138.6271	2.1401185	1.097690691	######	4E-06	up	yes
Manes.01G08210	14.844229	36.97985	0.401414	-1.31683707	0.0042	0.0136	down	yes
Manes.01G08250	3.2117043	8.443623	0.3803704	-1.39452311	0.1314	0.2439	down	no
Manes.01G08440	17.377184	7.312091	2.3765	1.248838427	0.0261	0.0651	up	no
Manes.01G08530	2.2058087	7.284994	0.302788	-1.72362001	0.071	0.149	down	no
Manes.01G08650	34.487997	80.16593	0.4302076	-1.21689496	0.0005	0.0019	down	yes
Manes.01G08710	53.033027	35.2783	1.5032762	0.588110151	0.09	0.1811	up	no
Manes.01G08990	58.544667	24.69687	2.3705298	1.245209501	0.0004	0.0018	up	yes
Manes.01G09190	2.5792355	0.914266	2.8210989	1.496257242	0.2974	0.4581	up	no
Manes.01G09220	10.690254	21.56918	0.4956264	-1.01267504	0.0676	0.1431	down	no
Manes.01G09350	3.0017929	6.657153	0.4509124	-1.14908091	0.4128	0.5784	down	no
Manes.01G09400	2.270948	5.464964	0.4155468	-1.26691723	0.2177	0.3621	down	no
Manes.01G09440	114.04439	47.69897	2.3909192	1.257565389	######	0.0001	up	yes
Manes.01G09490	12.598019	19.11059	0.6592166	-0.6011756	0.3714	0.5398	down	no
Manes.01G09600	131.97418	232.3581	0.5679776	-0.81609399	0.0065	0.0199	down	no
Manes.01G09650	6.4379022	5.783532	1.1131436	0.154639721	0.8527	0.952	up	no
Manes.01G10110	8.7233438	4.271357	2.0422886	1.030186783	0.16	0.2856	up	no
Manes.01G10140	6.5725452	10.94994	0.6002358	-0.73639875	0.3427	0.5085	down	no
Manes.01G10200	105.85072	312.361	0.3388731	-1.56118304	######	9E-07	down	yes
Manes.01G10220	74.594804	120.3888	0.6196158	-0.69055418	0.0141	0.0387	down	no
Manes.01G10230	29.170375	120.0381	0.2430093	-2.04091645	######	2E-08	down	yes
Manes.01G10580	273.72778	54.98083	4.9786044	2.315741383	######	8E-07	up	yes
Manes.01G10610	1.8873921	1.81215	1.041521	0.058691924	1	1	up	no
Manes.01G10720	379.57878	630.7075	0.6018301	-0.73257185	0.0004	0.0015	down	no

Manes.01G10820	10.422598	2.456373	4.2430843	2.08511335	0.0063	0.0194	up	yes
Manes.01G10840	0.3285458	2.379333	0.1380831	-2.85639096	0.2143	0.3576	down	no
Manes.01G10990	2.3997115	4.577794	0.5242069	-0.93179171	0.4712	0.6339	down	no
Manes.01G11020	79.033713	40.71758	1.9410218	0.956816324	0.0028	0.0095	up	no
Manes.01G11030	698.41116	201.3562	3.4685359	1.794326796	######	5E-26	up	yes
Manes.01G11070	9.9984105	6.091387	1.6414013	0.714927987	0.2694	0.4249	up	no
Manes.01G11340	216.73923	66.90534	3.2394906	1.695766951	0.0002	0.0007	up	yes
Manes.01G11450	521.62952	541.915	0.9625671	-0.05504101	0.7568	0.8822	down	no
Manes.01G11770	6.1296147	17.29136	0.3544901	-1.49618283	0.0183	0.0483	down	yes
Manes.01G11910	55.282432	104.2408	0.5303339	-0.91502714	0.0027	0.0092	down	no
Manes.01G11920	7.1253811	38.28689	0.186105	-2.42581133	######	3E-05	down	yes
Manes.01G12250	3.2869726	1.502878	2.1871184	1.129031323	0.3616	0.5296	up	no
Manes.01G12390	6.8011998	13.33211	0.5101369	-0.97104351	0.1499	0.2711	down	no
Manes.01G12410	21.35012	17.5885	1.2138684	0.279611968	0.5375	0.6976	up	no
Manes.01G12560	5.5810284	6.65857	0.8381722	-0.25468144	0.8798	0.9719	down	no
Manes.01G12740	55.919035	48.06465	1.1634129	0.21836326	0.4948	0.6578	up	no
Manes.01G13180	56.736907	55.59542	1.020532	0.029321397	0.935	0.997	up	no
Manes.01G13470	112.35301	121.0275	0.9283263	-0.10729615	0.7702	0.8913	down	no
Manes.01G13510	10053.084	5351.68	1.8784911	0.909574289	0.0002	0.0009	up	no
Manes.01G13600	278.75925	264.8657	1.0524551	0.073758693	0.7021	0.8379	up	no
Manes.01G13650	13.324499	25.86055	0.5152443	-0.95667139	0.06	0.13	down	no
Manes.01G13790	33.977012	2.738548	12.406946	3.633076159	######	8E-10	up	yes
Manes.01G14100	2.2405606	0	Inf	Inf	0.0349	0.0831	up	no
Manes.01G14400	1.8671339	2.427859	0.7690455	-0.3788592	1	1	down	no
Manes.01G14710	8.8376137	48.64317	0.1816825	-2.46050845	######	4E-06	down	yes
Manes.01G14960	74.06944	135.2887	0.5474917	-0.86909096	0.0035	0.0116	down	no
Manes.01G15080	16.63033	4.54928	3.6555959	1.870106601	0.002	0.0071	up	yes
Manes.01G15400	4.9992052	7.906371	0.6323009	-0.6613169	0.4554	0.6194	down	no
Manes.01G15830	1.3590641	3.639265	0.3734446	-1.42103373	0.3241	0.4886	down	no
Manes.01G15840	9.6511214	19.65714	0.4909728	-1.02628507	0.0614	0.1325	down	no
Manes.01G15930	0.7121017	1.519261	0.4687158	-1.09321454	0.7275	0.8583	down	no
Manes.01G15940	1.4937071	0.604995	2.4689591	1.303902957	0.4754	0.6381	up	no
Manes.01G16030	64.704603	24.05911	2.6894013	1.42728504	######	0.0002	up	yes
Manes.01G16200	17.995159	14.89706	1.2079673	0.272581343	0.5843	0.7368	up	no
Manes.01G16370	1119.0461	718.4327	1.5576212	0.639344445	0.0747	0.1555	up	no
Manes.01G16380	14.823971	0.900718	16.457954	4.040713127	######	9E-06	up	yes
Manes.01G16750	5.9704638	9.736321	0.6132156	-0.70553366	0.4481	0.6124	down	no
Manes.01G16840	21.065006	7.609231	2.7683489	1.469025799	0.0064	0.0196	up	yes
Manes.01G16960	6.68693	9.730652	0.6872026	-0.5411925	0.6822	0.8208	down	no
Manes.01G17070	9.9796672	44.20843	0.2257412	-2.14725805	######	8E-05	down	yes
Manes.01G17420	4444.1264	4648.042	0.9561287	-0.06472328	0.6622	0.8035	down	no
Manes.01G17440	4202.3445	3742.424	1.1228936	0.167221254	0.2569	0.4101	up	no
Manes.01G17450	17.343947	9.114943	1.9028036	0.928126674	0.0931	0.1861	up	no
Manes.01G17760	15492.512	8915.123	1.7377788	0.797244472	######	2E-05	up	no
Manes.01G17880	131.28131	50.72204	2.5882499	1.371976926	0.0011	0.0043	up	yes
Manes.01G17960	17.53785	41.64194	0.4211583	-1.2475654	0.0042	0.0136	down	yes
Manes.01G18050	247.1136	189.0496	1.3071365	0.386409821	0.0546	0.1202	up	no
Manes.01G18130	432.41914	435.6786	0.9925186	-0.01083389	0.9711	1	down	no

Manes.01G18340	1.1202803	0.591446	1.8941375	0.921541095	0.8608	0.9578	up	no
Manes.01G18550	38.112129	30.0213	1.2695028	0.344263624	0.3683	0.5367	up	no
Manes.01G18860	11.638405	7.622779	1.5267928	0.610504285	0.3479	0.5143	up	no
Manes.01G18880	6.643449	52.69221	0.1260803	-2.98758547	######	1E-06	down	yes
Manes.01G19060	144.77579	557.3517	0.2597566	-1.94476767	######	5E-08	down	yes
Manes.01G19110	2.4894735	8.238488	0.302176	-1.726539	0.0615	0.1327	down	no
Manes.01G19360	249.48542	272.3776	0.9159543	-0.12665244	0.6501	0.793	down	no
Manes.01G19390	0.7671118	3.932154	0.1950869	-2.35781117	0.1947	0.333	down	no
Manes.01G19410	196.06949	209.7882	0.9346069	-0.09756847	0.7604	0.8847	down	no
Manes.01G19630	534.87457	260.1124	2.0563206	1.040065241	0.0314	0.0762	up	no
Manes.01G19830	921.06316	89.54052	10.286551	3.362687465	######	2E-10	up	yes
Manes.01G19880	185.75771	17.03407	10.905071	3.446927205	######	3E-09	up	yes
Manes.01G20290	1.4937071	1.492164	1.0010342	0.001491303	1	1	up	no
Manes.01G20300	11.059431	11.1672	0.9903491	-0.01399097	1	1	down	no
Manes.01G20500	19.857928	24.90342	0.7973976	-0.32662889	0.5132	0.6768	down	no
Manes.01G20590	14.514284	3.342125	4.3428307	2.11863572	0.0014	0.0051	up	yes
Manes.01G20880	355.52236	149.4947	2.3781599	1.24984569	######	9E-06	up	yes
Manes.01G20920	9.5352217	8.150734	1.1698605	0.226336482	0.7253	0.8564	up	no
Manes.01G20960	1922.1472	806.3643	2.3837207	1.25321519	######	9E-10	up	yes
Manes.01G21290	64.094137	18.1571	3.529977	1.819658787	######	6E-05	up	yes
Manes.01G21350	41.490428	93.56343	0.4434471	-1.17316613	0.0038	0.0124	down	yes
Manes.01G21520	99.883224	162.5399	0.614515	-0.70247989	0.0063	0.0194	down	no
Manes.01G21650	196.54344	304.9719	0.644464	-0.63382831	0.0064	0.0198	down	no
Manes.01G21870	141.96921	138.6239	1.0241325	0.034402312	0.8364	0.9404	up	no
Manes.01G21890	16.311799	8.185711	1.9927161	0.994736179	0.0679	0.1437	up	no
Manes.01G21950	30.645224	21.31127	1.4379823	0.524045953	0.2173	0.3617	up	no
Manes.01G22090	21.58029	39.71728	0.5433476	-0.88005268	0.0429	0.0988	down	no
Manes.01G22100	33.903373	19.74348	1.7171935	0.780052598	0.0755	0.157	up	no
Manes.01G22200	431.85588	154.5956	2.7934557	1.482050918	######	2E-14	up	yes
Manes.01G22510	2.8629003	6.104936	0.4689485	-1.09249865	0.2833	0.4411	down	no
Manes.01G22540	2.9425332	8.564143	0.3435876	-1.54125016	0.218	0.3623	down	no
Manes.01G22610	21.651194	29.52266	0.7333755	-0.447376	0.3369	0.5022	down	no
Manes.01G22780	1.0305183	0.307855	3.3474198	1.74304948	0.527	0.6881	up	no
Manes.01G23030	8.0111272	6.994939	1.1452748	0.195693807	0.7502	0.8765	up	no
Manes.01G23090	1.1202803	0	Inf	Inf	0.1992	0.3379	up	no
Manes.01G23210	7.6985901	11.185	0.6882957	-0.53889969	0.4309	0.5966	down	no
Manes.01G23380	51.726288	79.9255	0.6471813	-0.62775828	0.0534	0.1178	down	no
Manes.01G23730	40.930263	9.067834	4.5137859	2.174337991	######	0.0003	up	yes
Manes.01G23810	128.08997	34.29487	3.7349599	1.901092738	######	3E-09	up	yes
Manes.01G23870	53.074878	60.50959	0.8771317	-0.18913456	0.6238	0.7696	down	no
Manes.01G24030	164.54977	116.4984	1.4124637	0.498213754	0.0325	0.0783	up	no
Manes.01G24120	12.679052	2.452122	5.1706454	2.370344359	0.0564	0.1235	up	no
Manes.01G24220	84.084619	240.8159	0.3491655	-1.51801693	######	2E-07	down	yes
Manes.01G24350	171.62066	181.4005	0.9460871	-0.07995511	0.7335	0.8627	down	no
Manes.01G24430	135.4929	196.9588	0.6879252	-0.53967645	0.022	0.0566	down	no
Manes.01G24480	530.77455	367.8279	1.4429969	0.529068223	0.003	0.0101	up	no
Manes.01G24510	0.7569827	2.122838	0.3565899	-1.48766246	0.44	0.6054	down	no
Manes.01G24590	52.65377	73.19463	0.7193665	-0.47520107	0.1601	0.2857	down	no

Manes.01G24770	6.3380111	12.21554	0.5188482	-0.94661548	0.1915	0.3286	down	no
Manes.01G24890	127.73802	287.4129	0.4444407	-1.16993704	######	6E-06	down	yes
Manes.01G25390	113.21774	120.4198	0.9401917	-0.08897318	0.749	0.8757	down	no
Manes.01G25650	5.3074928	4.591343	1.1559783	0.209114306	0.7984	0.9122	up	no
Manes.01G25790	8.5944654	16.11918	0.5331826	-0.9072985	0.1426	0.2606	down	no
Manes.01G25840	48.015013	84.77791	0.5663623	-0.82020283	0.0124	0.0348	down	no
Manes.01G25870	141.90001	162.1911	0.8748938	-0.19282023	0.4439	0.6081	down	no
Manes.01G26020	412.81787	480.7397	0.8587139	-0.21975054	0.216	0.36	down	no
Manes.01G26490	13.833969	3.019305	4.581839	2.195926766	0.0013	0.0048	up	yes
Manes.01G26700	4.1337173	2.402179	1.7208199	0.783096143	0.6147	0.7612	up	no
Manes.01G26860	11.063681	1.219287	9.0738966	3.181722213	0.0084	0.0248	up	yes
Manes.01G26880	47.278354	8.505697	5.5584336	2.474678388	######	2E-06	up	yes
Manes.01G27060	16.35668	0	Inf	Inf	######	7E-09	up	yes
Manes.01G27530	1.1304094	0.309272	3.6550703	1.869899173	0.5176	0.6806	up	no
Manes.02G00250	1.0305183	6.970676	0.1478362	-2.75792844	0.0162	0.0436	down	yes
Manes.02G00440	249.20695	199.4479	1.2494838	0.321332161	0.1727	0.3035	up	no
Manes.02G00560	157.05293	179.9687	0.8726681	-0.19649497	0.3945	0.5616	down	no
Manes.02G00630	2.9975433	3.912142	0.7662153	-0.38417833	0.9316	0.9966	down	no
Manes.02G00640	288.0934	275.1043	1.0472154	0.066558156	0.8247	0.9325	up	no
Manes.02G00830	172.21528	279.6733	0.6157731	-0.69952915	0.0014	0.0051	down	no
Manes.02G00900	41.556853	181.6707	0.2287483	-2.12816729	######	3E-11	down	yes
Manes.02G00970	186.68009	137.1815	1.3608253	0.444481868	0.0477	0.1078	up	no
Manes.02G00990	11.961071	20.43402	0.585351	-0.77262622	0.1591	0.2843	down	no
Manes.02G01000	2.1261758	1.839247	1.1560035	0.209145776	0.9321	0.9966	up	no
Manes.02G01020	3.2667144	5.115046	0.638648	-0.64690701	0.5746	0.7281	down	no
Manes.02G01110	13.324499	67.64112	0.1969881	-2.34381925	######	4E-07	down	yes
Manes.02G01140	85.529261	129.0566	0.6627268	-0.59351374	0.217	0.3613	down	no
Manes.02G01330	337.00383	780.7516	0.4316403	-1.21209861	######	1E-09	down	yes
Manes.02G01340	632.25269	73.08133	8.6513568	3.112926415	######	4E-57	up	yes
Manes.02G01410	45.855715	53.94364	0.8500671	-0.23435142	0.5191	0.6815	down	no
Manes.02G01790	395.79606	269.7515	1.4672616	0.55312614	0.0049	0.0155	up	no
Manes.02G01840	59.760703	84.95421	0.703446	-0.50748837	0.0973	0.1927	down	no
Manes.02G01880	0.7468535	6.160547	0.1212317	-3.04416113	0.113	0.2171	down	no
Manes.02G02340	4.3623719	10.05772	0.4337335	-1.20511913	0.122	0.2303	down	no
Manes.02G02470	66.101564	7.316342	9.0347833	3.175490001	######	2E-13	up	yes
Manes.02G02620	1.0753993	0	Inf	Inf	0.2236	0.3685	up	no
Manes.02G02770	10.068029	12.79202	0.7870555	-0.34546279	0.7927	0.9076	down	no
Manes.02G02910	49.517515	33.30277	1.4868887	0.572296698	0.1462	0.2657	up	no
Manes.02G02960	1.0855284	2.415727	0.4493588	-1.15406016	0.5252	0.6867	down	no
Manes.02G03190	160.52839	139.5049	1.1507011	0.202513156	0.6798	0.819	up	no
Manes.02G03460	3.6502703	3.956417	0.9226202	-0.11619123	1	1	down	no
Manes.02G03560	2.2810771	12.15205	0.1877113	-2.41341236	0.0083	0.0245	down	yes
Manes.02G03680	406.04243	17.94771	22.623632	4.499758645	######	2E-75	up	yes
Manes.02G03710	18.970782	90.85198	0.2088098	-2.25973872	######	4E-05	down	yes
Manes.02G03850	26.211833	29.83113	0.8786737	-0.18660057	0.7177	0.8507	down	no
Manes.02G03860	332.5604	30.16467	11.024832	3.462684759	######	3E-44	up	yes
Manes.02G03880	14.036716	0	Inf	Inf	######	2E-07	up	yes
Manes.02G03910	232.9065	308.5404	0.7548656	-0.40570832	0.0464	0.1054	down	no

Manes.02G03930	3.2913372	0.887169	3.7099318	1.891392653	0.4653	0.6282	up	no
Manes.02G03970	4.4811212	0.617126	7.2612731	2.860222519	0.0228	0.0582	up	no
Manes.02G04030	227.2502	151.8225	1.4968148	0.581895696	0.0059	0.0183	up	no
Manes.02G04100	95.899863	96.96365	0.989029	-0.01591527	0.9988	1	down	no
Manes.02G04360	213.18881	72.72955	2.9312544	1.551518197	######	4E-06	up	yes
Manes.02G04390	11.024564	4.203614	2.6226393	1.39101942	0.1309	0.2432	up	no
Manes.02G04520	1.0956575	7.633494	0.1435329	-2.80054657	0.0104	0.0297	down	yes
Manes.02G04740	1.4140742	3.357091	0.4212201	-1.24735371	0.4014	0.567	down	no
Manes.02G04760	8.8391286	6.415624	1.3777504	0.462314517	0.517	0.6801	up	no
Manes.02G04800	2.5546127	1.825698	1.3992524	0.484656188	0.752	0.8781	up	no
Manes.02G05070	16.717243	11.87634	1.4076094	0.493247032	0.3751	0.5438	up	no
Manes.02G05080	42.620953	0.899301	47.393442	5.566615527	######	3E-15	up	yes
Manes.02G05140	406.50073	175.6567	2.3141775	1.210499514	######	8E-10	up	yes
Manes.02G05270	1.1202803	2.76281	0.4054858	-1.30227682	0.6211	0.7671	down	no
Manes.02G05340	104.09919	149.7844	0.6949933	-0.52492895	0.0562	0.1232	down	no
Manes.02G05420	278.6027	129.972	2.1435597	1.10000858	######	6E-06	up	yes
Manes.02G05510	26.229176	58.55815	0.4479168	-1.15869746	0.0068	0.0207	down	yes
Manes.02G05560	1.0956575	0.926398	1.1827075	0.242093356	1	1	up	no
Manes.02G05680	1920.608	1606.047	1.1958607	0.258049368	0.1848	0.3194	up	no
Manes.02G05690	8.7927327	17.31562	0.507792	-0.97769055	0.1113	0.2144	down	no
Manes.02G05710	135.71988	119.2499	1.1381133	0.186644201	0.45	0.6141	up	no
Manes.02G05760	342.84902	303.309	1.130362	0.176784889	0.3381	0.5034	up	no
Manes.02G05830	1.832382	0.309272	5.9248311	2.566774038	0.2008	0.3399	up	no
Manes.02G05900	642.26939	699.2203	0.9185508	-0.12256854	0.455	0.6191	down	no
Manes.02G06030	4.3319846	9.751286	0.4442475	-1.17056441	0.1399	0.2566	down	no
Manes.02G06040	7.0212403	8.577691	0.8185466	-0.28886347	0.7562	0.8816	down	no
Manes.02G06120	67.253632	62.35246	1.0786043	0.109165647	0.7381	0.8664	up	no
Manes.02G06210	33.811932	10.96916	3.0824553	1.624079971	0.0003	0.0012	up	yes
Manes.02G06240	43.301283	29.49335	1.4681712	0.554020159	0.1688	0.2983	up	no
Manes.02G06260	6.0499818	2.107873	2.8701834	1.521142928	0.0901	0.1812	up	no
Manes.02G06310	5.4870168	0.604995	9.0695293	3.181027683	0.0075	0.0226	up	yes
Manes.02G06330	16.611587	12.77201	1.3006245	0.379204527	0.4872	0.6501	up	no
Manes.02G06420	16.377053	1.839247	8.9042168	3.154488715	######	5E-05	up	yes
Manes.02G06480	246.30831	296.0325	0.8320313	-0.26529035	0.1811	0.3144	down	no
Manes.02G06790	53.475958	132.4799	0.4036534	-1.30881105	######	7E-05	down	yes
Manes.02G06820	38.579797	61.54295	0.6268759	-0.67374815	0.0851	0.1729	down	no
Manes.02G07100	11.104197	6.689919	1.6598404	0.731044541	0.2421	0.3919	up	no
Manes.02G07220	182.02121	68.75595	2.6473519	1.404549973	######	1E-07	up	yes
Manes.02G07230	26.281517	5.819927	4.5157814	2.174975634	0.0089	0.0261	up	yes
Manes.02G07280	1.4589552	0.295723	4.9335185	2.30261691	0.3237	0.4881	up	no
Manes.02G07320	685.92769	629.5942	1.0894758	0.123634207	0.4368	0.6028	up	no
Manes.02G07460	1.1202803	0.295723	3.7882751	1.921541095	0.4588	0.6218	up	no
Manes.02G07890	108.68545	0	Inf	Inf	######	1E-42	up	yes
Manes.02G08100	97.773931	41.16313	2.3752791	1.248097068	######	0.0001	up	yes
Manes.02G09030	790.11167	2346.75	0.3366834	-1.5705355	######	5E-10	down	yes
Manes.02G09300	22.457422	51.03148	0.44007	-1.18419523	0.0095	0.0276	down	yes
Manes.02G09350	420.21532	207.417	2.0259443	1.018594527	######	0.0002	up	yes
Manes.02G09450	42.383339	58.93738	0.719125	-0.47568557	0.1852	0.3201	down	no

Manes.02G09480	1.1202803	0.899301	1.2457239	0.316984354	0.9225	0.9966	up	no
Manes.02G09610	2.5893646	8.1479	0.3177953	-1.65383013	0.0713	0.1494	down	no
Manes.02G09630	29.914083	6.125569	4.883478	2.287908993	0.0023	0.008	up	yes
Manes.02G09910	6.7316961	9.087846	0.7407361	-0.43296837	0.6186	0.7646	down	no
Manes.02G10100	6.2888804	3.963502	1.5866978	0.666027344	0.4162	0.5812	up	no
Manes.02G10150	143.00457	402.6615	0.3551483	-1.4935064	######	1E-10	down	yes
Manes.02G10420	4.4072529	8.799208	0.5008692	-0.99749406	0.2123	0.355	down	no
Manes.02G10600	699.23815	601.6178	1.1622631	0.216936721	0.1706	0.3007	up	no
Manes.02G10860	2.4445925	2.121421	1.1523371	0.204562757	0.9133	0.9962	up	no
Manes.02G12130	1465.7335	1488.84	0.98448	-0.02256625	0.9098	0.9937	down	no
Manes.02G12140	41.256009	13.09987	3.1493439	1.655051288	0.0002	0.0008	up	yes
Manes.02G12640	7.2151431	32.63176	0.221108	-2.1771767	######	0.0003	down	yes
Manes.02G12820	14.301523	34.99834	0.4086344	-1.29111754	0.0056	0.0175	down	yes
Manes.02G13020	5.462394	43.76823	0.1248027	-3.00227891	######	3E-07	down	yes
Manes.02G13030	21.490528	29.27608	0.7340643	-0.44602157	0.3536	0.5207	down	no
Manes.02G13070	9.1357721	17.38257	0.5255709	-0.92804266	0.2747	0.4311	down	no
Manes.02G13200	21.934973	30.57777	0.7173503	-0.47925031	0.2789	0.4359	down	no
Manes.02G13260	2.664633	1.517844	1.7555383	0.811913471	0.5733	0.727	up	no
Manes.02G13800	22.412541	47.06183	0.476236	-1.07025132	0.0091	0.0267	down	yes
Manes.02G13810	18.149945	13.90779	1.3050199	0.384071833	0.5921	0.7442	up	no
Manes.02G14010	14.381041	8.538463	1.6842658	0.752119825	0.1807	0.3138	up	no
Manes.02G14060	10.879907	57.78096	0.1882957	-2.4089279	######	1E-06	down	yes
Manes.02G14360	0.3285458	5.185001	0.0633646	-3.98017801	0.0286	0.0703	down	no
Manes.02G14920	66.981316	81.83786	0.8184637	-0.28900965	0.3711	0.5395	down	no
Manes.02G14950	4.1134591	4.291369	0.9585425	-0.06108576	1	1	down	no
Manes.02G15000	1.1202803	0	Inf	Inf	0.1992	0.3379	up	no
Manes.02G15260	48.699527	72.89086	0.6681157	-0.58183013	0.0842	0.1714	down	no
Manes.02G15290	617.80407	27.68828	22.312834	4.479801841	######	4E-98	up	yes
Manes.02G15360	14.544556	20.73541	0.7014357	-0.51161714	0.37	0.5383	down	no
Manes.02G15620	295.50879	272.178	1.0857191	0.118650842	0.4981	0.6615	up	no
Manes.02G15630	3.1769524	2.71145	1.17168	0.228578652	0.8746	0.968	up	no
Manes.02G15710	16.817019	10.62995	1.5820408	0.661786787	0.2227	0.3677	up	no
Manes.02G15720	71.797803	2.162067	33.207945	5.053456553	######	0.0002	up	yes
Manes.02G15760	4.1178236	0	Inf	Inf	0.0029	0.0098	up	yes
Manes.02G16050	22.508068	6.145581	3.6624799	1.872820858	0.0074	0.0222	up	yes
Manes.02G16080	185.09115	222.1542	0.8331654	-0.26332523	0.2319	0.3791	down	no
Manes.02G16140	72.110914	63.75718	1.1310243	0.177629886	0.5626	0.7175	up	no
Manes.02G16270	9.2878238	7.544322	1.2311011	0.299949279	0.7432	0.8709	up	no
Manes.02G16300	18.723154	0.926398	20.210709	4.33704802	######	2E-07	up	yes
Manes.02G16360	22.764195	134.3596	0.1694274	-2.56126117	######	7E-12	down	yes
Manes.02G16380	11.532749	36.1505	0.3190204	-1.64827922	0.0055	0.0173	down	yes
Manes.02G16390	579.77699	457.0929	1.2684008	0.343010712	0.1971	0.336	up	no
Manes.02G16490	16.999393	9.719938	1.7489199	0.806464197	0.1331	0.2463	up	no
Manes.02G16750	19.757922	54.05553	0.3655116	-1.45201093	0.0028	0.0095	down	yes
Manes.02G16980	2.9077813	0.912849	3.1853907	1.671470336	0.2133	0.3563	up	no
Manes.02G17200	1.1405385	0.911432	1.2513697	0.323508117	1	1	up	no
Manes.02G17380	141.56989	261.0136	0.5423852	-0.88261039	0.0001	0.0006	down	no
Manes.02G17700	4.1279527	0.618543	6.6736694	2.738480212	0.2262	0.3718	up	no

Manes.02G17960	30.154497	47.35251	0.6368088	-0.65106775	0.0978	0.1935	down	no
Manes.02G17980	124.49615	71.7286	1.7356557	0.795480779	0.0172	0.0458	up	no
Manes.02G18300	1.877263	0	Inf	Inf	0.065	0.1386	up	no
Manes.02G18420	0	1.813567	#NAME?	#NAME?	0.1181	0.2246	down	no
Manes.02G18820	4.0540844	6.071375	0.6677374	-0.58264724	0.6819	0.8206	down	no
Manes.02G18890	561.90265	378.8734	1.4830883	0.568604462	0.0005	0.0021	up	no
Manes.02G18900	0.7019725	1.517844	0.4624801	-1.11253687	0.7265	0.8574	down	no
Manes.02G18910	5.3913753	8.157819	0.6608844	-0.59753024	0.7611	0.8851	down	no
Manes.02G18930	8.1457703	3.384188	2.407009	1.267241508	0.2002	0.3393	up	no
Manes.02G19010	201.59208	238.8568	0.8439873	-0.24470676	0.2793	0.4364	down	no
Manes.02G19440	1368.847	1114.384	1.2283442	0.296714915	0.0388	0.0907	up	no
Manes.02G19550	33.851048	12.4684	2.7149462	1.440923607	0.0165	0.0443	up	yes
Manes.02G19670	1.0406474	2.417144	0.4305276	-1.21582235	0.522	0.6841	down	no
Manes.02G19720	0.7569827	2.441407	0.31006	-1.6893809	0.3406	0.5061	down	no
Manes.02G20230	53.337114	102.3025	0.5213668	-0.93962924	0.004	0.013	down	no
Manes.02G20600	83.888031	174.1782	0.4816218	-1.05402746	0.0001	0.0005	down	yes
Manes.02G20620	3.9136768	6.649273	0.5885872	-0.76467197	0.424	0.5894	down	no
Manes.02G20940	45.41127	104.0319	0.4365131	-1.19590325	0.0002	0.0009	down	yes
Manes.02G20950	66.305481	51.59048	1.2852271	0.362023255	0.2543	0.4073	up	no
Manes.02G21060	198.70182	167.0483	1.1894875	0.250340151	0.2407	0.3901	up	no
Manes.02G21440	8.1516498	5.452832	1.4949387	0.580086323	0.4504	0.6144	up	no
Manes.02G21850	36.068436	51.61412	0.6988095	-0.51702896	0.1932	0.331	down	no
Manes.02G21920	52.532286	84.04894	0.6250202	-0.67802524	0.0343	0.082	down	no
Manes.02G21930	175.67905	146.2931	1.20087	0.264079943	0.2469	0.3982	up	no
Manes.02G22100	83.514785	175.5061	0.4758513	-1.07141726	######	0.0003	down	yes
Manes.02G22190	262.65618	99.91839	2.628707	1.394353336	######	4E-09	up	yes
Manes.02G22200	2.7731383	1.517844	1.8270248	0.869496185	0.4743	0.637	up	no
Manes.02G22320	242.42291	217.0994	1.1166446	0.159170138	0.4318	0.5978	up	no
Manes.02G22400	29.073333	15.52552	1.8726156	0.905054772	0.0379	0.089	up	no
Manes.02G22470	178.36755	104.5255	1.7064501	0.770998253	0.0041	0.0133	up	no
Manes.02G22480	6.4480313	11.18784	0.5763429	-0.79500072	0.2669	0.4219	down	no
Manes.02G22560	1.1304094	0.603578	1.8728486	0.905234277	0.8067	0.9182	up	no
Manes.03G00210	70.242446	71.24509	0.9859268	-0.02044757	0.9674	1	down	no
Manes.03G00500	50.450991	46.49119	1.0851731	0.117925135	0.7285	0.859	up	no
Manes.03G00630	198.55303	150.058	1.3231754	0.404004368	0.0631	0.1353	up	no
Manes.03G00680	123.02952	148.2887	0.8296623	-0.26940394	0.2848	0.443	down	no
Manes.03G00840	3.2261979	2.121421	1.5207718	0.604803713	0.5977	0.7498	up	no
Manes.03G01070	32.701715	57.84728	0.5653112	-0.8228829	0.0437	0.1003	down	no
Manes.03G01470	17.989394	6.43059	2.7974719	1.484123647	0.0074	0.0223	up	yes
Manes.03G01670	17.24982	10.09695	1.7084186	0.772661476	0.1711	0.3014	up	no
Manes.03G01950	594.52963	316.7678	1.8768625	0.908322944	######	4E-06	up	no
Manes.03G01970	1818.5695	1883.729	0.9654091	-0.05078767	0.7334	0.8626	down	no
Manes.03G02010	2.2361961	0	Inf	Inf	0.0371	0.0874	up	no
Manes.03G02120	18.60312	16.71913	1.1126848	0.15404499	0.7766	0.8952	up	no
Manes.03G02280	38.462333	34.68543	1.1088901	0.149116329	0.6812	0.82	up	no
Manes.03G02660	175.72365	128.324	1.3693747	0.453517271	0.0489	0.11	up	no
Manes.03G02690	3.3115954	2.459207	1.346611	0.429333159	0.8867	0.9772	up	no
Manes.03G02740	0.3285458	2.417144	0.1359231	-2.87913745	0.1737	0.3047	down	no

Manes.03G02750	0.7019725	8.499234	0.0825924	-3.59784636	0.002	0.0073	down	yes
Manes.03G02810	1359.4688	1639.608	0.8291426	-0.27030779	0.2113	0.3535	down	no
Manes.03G02880	19.942041	140.4206	0.1420165	-2.8158698	######	3E-07	down	yes
Manes.03G03090	1.0855284	2.106456	0.5153341	-0.95642015	0.6534	0.7956	down	no
Manes.03G03120	5.0295926	15.58113	0.3228002	-1.63128666	0.0377	0.0885	down	no
Manes.03G03190	81.944639	100.0689	0.8188826	-0.28827155	0.3775	0.5465	down	no
Manes.03G03350	6.160002	4.873517	1.2639745	0.337967406	0.6883	0.8258	up	no
Manes.03G03400	293.09017	279.1166	1.0500635	0.070476545	0.7259	0.857	up	no
Manes.03G03490	213.17717	234.94	0.9073685	-0.14023957	0.5289	0.6898	down	no
Manes.03G04270	67.697897	96.15972	0.7040151	-0.50632167	0.1106	0.2132	down	no
Manes.03G04310	1.3590641	0.309272	4.3944032	2.135667239	0.33	0.4947	up	no
Manes.03G04340	49.151187	97.6243	0.5034729	-0.99001399	0.0019	0.007	down	no
Manes.03G04430	2.5691064	10.04922	0.2556523	-1.96774515	0.0956	0.1902	down	no
Manes.03G04490	68.711072	53.25452	1.2902392	0.367638509	0.2394	0.3885	up	no
Manes.03G04630	2.9627914	20.24292	0.1463619	-2.77238843	0.0004	0.0015	down	yes
Manes.03G04740	10.481857	6.712764	1.5614815	0.642915495	0.3149	0.4784	up	no
Manes.03G05010	2.270948	0.604995	3.7536662	1.908300351	0.2533	0.4062	up	no
Manes.03G05140	17.30908	21.1857	0.8170171	-0.29156174	0.5843	0.7368	down	no
Manes.03G05170	1.3590641	0	Inf	Inf	0.1228	0.2312	up	no
Manes.03G05480	6.2237412	8.789289	0.7081052	-0.49796438	0.6835	0.8219	down	no
Manes.03G05590	91.347788	94.44303	0.9672263	-0.04807455	0.8948	0.983	down	no
Manes.03G05620	32.370435	28.90899	1.119736	0.163158679	0.6745	0.8145	up	no
Manes.03G05970	0.3285458	1.223538	0.2685211	-1.89689247	0.5983	0.7502	down	no
Manes.03G05990	30.901695	67.19981	0.459848	-1.12077116	0.0437	0.1002	down	no
Manes.03G06020	1.1202803	2.147101	0.521764	-0.93853056	0.8353	0.9401	down	no
Manes.03G06040	27.58964	25.00251	1.1034747	0.142053534	0.6924	0.8294	up	no
Manes.03G06360	284.39359	308.9981	0.9203734	-0.11970882	0.5883	0.7403	down	no
Manes.03G06380	350.84305	293.0725	1.1971203	0.259568133	0.3616	0.5296	up	no
Manes.03G06390	200.35199	462.9333	0.432788	-1.20826759	######	3E-08	down	yes
Manes.03G06450	6.6579427	7.237885	0.9198741	-0.12049172	0.9303	0.9966	down	no
Manes.03G06490	7.5929344	0.591446	12.837914	3.682338874	0.0597	0.1294	up	no
Manes.03G06520	103.93026	168.8738	0.6154314	-0.70032999	0.0393	0.0918	down	no
Manes.03G06530	79.698084	140.6591	0.5666044	-0.81958632	0.0028	0.0097	down	no
Manes.03G06580	183.69582	143.8409	1.2770769	0.352845351	0.1143	0.2193	up	no
Manes.03G06710	64.842046	75.19505	0.862318	-0.21370802	0.5314	0.692	down	no
Manes.03G06750	65.684656	100.5563	0.6532128	-0.61437512	0.0385	0.0901	down	no
Manes.03G06880	106.12961	52.73445	2.0125291	1.009009634	0.0003	0.0013	up	yes
Manes.03G06900	98.644243	108.6319	0.9080598	-0.13914086	0.6472	0.7904	down	no
Manes.03G07060	38.26703	58.5287	0.6538166	-0.61304219	0.0931	0.1861	down	no
Manes.03G07180	422.57035	313.9566	1.3459515	0.428626474	0.0136	0.0374	up	no
Manes.03G07200	18.653765	45.78898	0.4073855	-1.2955334	0.0055	0.0172	down	yes
Manes.03G07210	620.41528	534.7395	1.1602197	0.214398074	0.1791	0.3115	up	no
Manes.03G07310	1.4488261	0	Inf	Inf	0.1199	0.227	up	no
Manes.03G07390	3.7400323	7.037002	0.531481	-0.91191011	0.3088	0.4716	down	no
Manes.03G07510	170.96985	81.27177	2.1036805	1.072915638	0.0021	0.0076	up	yes
Manes.03G07530	1.0753993	4.900615	0.2194417	-2.18809022	0.0846	0.1721	down	no
Manes.03G07670	168.13518	246.349	0.682508	-0.55108206	0.0967	0.1919	down	no
Manes.03G07910	1.8222528	0.926398	1.9670308	0.976019572	0.5533	0.7122	up	no

Manes.03G07940	38.866262	12.84258	3.0263582	1.59758275	0.0201	0.0523	up		no
Manes.03G08440	58.455644	0	Inf	Inf	######	8E-24	up		yes
Manes.03G08720	9.4904556	0.591446	16.046188	4.004158678	0.0328	0.0791	up		no
Manes.03G08740	55.347456	97.44232	0.5680022	-0.81603155	0.0081	0.0241	down		no
Manes.03G09430	8.8145059	5.514906	1.5983056	0.676543273	0.3373	0.5026	up		no
Manes.03G09460	2.0711657	2.110707	0.9812663	-0.02728335	1	1	down		no
Manes.03G09550	3.8832895	1.196441	3.2457013	1.698530255	0.219	0.3636	up		no
Manes.03G09830	1.8222528	0	Inf	Inf	0.0665	0.141	up		no
Manes.03G09890	66.800457	40.11622	1.6651734	0.735672458	0.0238	0.0603	up		no
Manes.03G09910	2587.6941	4896.189	0.5285119	-0.91999211	0.0011	0.0042	down		no
Manes.03G09930	201.34754	376.0961	0.5353619	-0.90141357	0.09	0.1811	down		no
Manes.03G09940	423.68009	620.137	0.683204	-0.54961173	0.0424	0.0978	down		no
Manes.03G09950	22.19553	32.17912	0.6897495	-0.53585562	0.227	0.373	down		no
Manes.03G10070	7.0457482	11.57273	0.6088232	-0.71590483	0.3327	0.4979	down		no
Manes.03G10080	148.81168	218.2116	0.6819604	-0.55224005	0.0232	0.059	down		no
Manes.03G10160	54.204117	117.712	0.4604807	-1.11878735	0.0004	0.0018	down		yes
Manes.03G10280	1.8425111	0	Inf	Inf	0.066	0.1403	up		no
Manes.03G10330	0.3734268	3.962085	0.0942501	-3.4073627	0.0398	0.0926	down		no
Manes.03G10780	2.9830496	0.307855	9.689803	3.27646733	0.0491	0.1103	up		no
Manes.03G11090	85.967877	78.4387	1.095988	0.132231983	0.6228	0.7687	up		no
Manes.03G11170	156.30893	221.4849	0.7057318	-0.50280803	0.0258	0.0644	down		no
Manes.03G11230	96.090801	180.1828	0.5332962	-0.90699103	0.0005	0.002	down		no
Manes.03G11250	120.20994	168.9022	0.7117135	-0.49063153	0.0516	0.1147	down		no
Manes.03G11270	4.3319846	4.546446	0.9528288	-0.06971115	1	1	down		no
Manes.03G11440	81.879221	25.56341	3.2029856	1.679417306	######	3E-06	up		yes
Manes.03G11610	370.87978	355.1424	1.0443128	0.06255389	0.7039	0.8394	up		no
Manes.03G11730	1488.3731	313.3907	4.7492576	2.247702023	######	4E-30	up		yes
Manes.03G12050	19.484501	56.62375	0.3441048	-1.53908017	0.0002	0.0009	down		yes
Manes.03G12180	4.1076945	0.295723	13.890342	3.796010213	0.0128	0.0357	up		yes
Manes.03G12260	29.45241	16.45776	1.7895758	0.839617687	0.1427	0.2607	up		no
Manes.03G12910	6428.3515	549.634	11.695696	3.547905757	######	3E-135	up		yes
Manes.03G13070	101.54872	160.799	0.6315257	-0.66308662	0.0111	0.0316	down		no
Manes.03G13430	81.667794	59.74923	1.3668427	0.450847211	0.1216	0.2296	up		no
Manes.03G13700	118.50311	103.2553	1.147671	0.198709127	0.4073	0.5726	up		no
Manes.03G13720	1.832382	0.615709	2.9760518	1.57339962	0.6124	0.7594	up		no
Manes.03G13820	1.4937071	0.307855	4.8519901	2.278576613	0.2798	0.4369	up		no
Manes.03G14050	53.743958	141.9427	0.3786314	-1.40113385	######	4E-05	down		yes
Manes.03G14060	11.538513	22.31249	0.5171325	-0.95139424	0.0811	0.1661	down		no
Manes.03G14180	8.8983884	74.72364	0.119084	-3.06994881	######	5E-09	down		yes
Manes.03G14200	901.73683	689.9953	1.3068739	0.386119923	0.0104	0.0298	up		no
Manes.03G14410	69.45781	54.32207	1.2786296	0.354598345	0.2813	0.4386	up		no
Manes.03G14560	22.108618	16.20693	1.3641456	0.447997681	0.4019	0.5673	up		no
Manes.03G14570	1.1202803	1.529975	0.7322212	-0.44964859	1	1	down		no
Manes.03G14980	10.645373	13.78395	0.7723021	-0.37276271	0.6604	0.802	down		no
Manes.03G15110	0.3734268	1.234252	0.302553	-1.72473998	0.6096	0.7568	down		no
Manes.03G15590	4.2567162	3.640682	1.1692084	0.225532058	0.8143	0.9242	up		no
Manes.03G15630	1436.2867	1505.419	0.9540774	-0.06782177	0.7722	0.8919	down		no
Manes.03G16040	133.83125	137.7403	0.9716199	-0.04153613	0.8837	0.9749	down		no

Manes.03G16250	6.203368	1.231418	5.0375808	2.332731075	0.2317	0.3789	up	no
Manes.03G16830	5.0484508	0.295723	17.071549	4.093522065	0.0041	0.0134	up	yes
Manes.03G17050	173.26332	177.8918	0.9739813	-0.03803401	0.8999	0.9867	down	no
Manes.03G17090	1.8671339	0.295723	6.3137918	2.658506689	0.1767	0.3085	up	no
Manes.03G17210	65.972685	76.83815	0.8585928	-0.21995398	0.4812	0.6438	down	no
Manes.03G17260	1.4589552	9.811943	0.1486918	-2.74960334	0.0054	0.0171	down	yes
Manes.03G17460	3.0033079	6.759873	0.4442847	-1.17044379	0.3745	0.5432	down	no
Manes.03G17570	5.0440862	2.723582	1.8520046	0.889087679	0.3389	0.5042	up	no
Manes.03G17700	172.9929	214.5932	0.8061436	-0.31089131	0.487	0.65	down	no
Manes.03G17800	4.2320935	5.155692	0.8208585	-0.28479459	0.8868	0.9772	down	no
Manes.03G17840	53.513674	42.97356	1.2452697	0.316458267	0.4538	0.6178	up	no
Manes.03G17950	8.9881504	11.50924	0.7809507	-0.35669663	0.63	0.7754	down	no
Manes.03G18270	2317.2682	900.6532	2.572875	1.36338139	######	4E-19	up	yes
Manes.03G18800	7.125496	0	Inf	Inf	######	0.0004	up	yes
Manes.03G18810	120.67883	104.7881	1.1516462	0.203697595	0.4182	0.5833	up	no
Manes.03G18920	112.28862	95.68467	1.1735278	0.230852015	0.3815	0.5509	up	no
Manes.03G19000	96.613545	119.7728	0.8066402	-0.31000281	0.2471	0.3985	down	no
Manes.03G19110	12.990189	4.550697	2.8545491	1.513262895	0.0215	0.0555	up	no
Manes.03G19140	128.28095	197.1646	0.6506288	-0.6200934	0.0201	0.0523	down	no
Manes.03G19640	22.172242	1.195024	18.55381	4.213643534	0.0001	0.0006	up	yes
Manes.03G19900	80.340977	53.67723	1.4967422	0.581825797	0.0615	0.1327	up	no
Manes.03G20330	1.8222528	0	Inf	Inf	0.0665	0.141	up	no
Manes.03G20530	4.0888363	5.169241	0.7909936	-0.33826203	0.797	0.9111	down	no
Manes.03G20800	5.4421358	4.1929	1.2979408	0.376224552	0.865	0.9609	up	no
Manes.03G20820	54.148877	123.5522	0.4382671	-1.19011783	######	0.0005	down	yes
Manes.03G20940	116.67096	126.506	0.9222562	-0.11676046	0.6842	0.8224	down	no
Manes.03G21000	400.17006	366.3864	1.0922077	0.127247243	0.456	0.6195	up	no
Manes.03G21030	3.5199918	1.787887	1.9688	0.977316585	0.6166	0.7628	up	no
Manes.03G21300	39.450935	235.4578	0.1675499	-2.577337	######	2E-14	down	yes
Manes.04G00030	19.10531	27.62275	0.6916512	-0.53188343	0.2625	0.4166	down	no
Manes.04G00050	2413.6889	1121.451	2.1522918	1.105873687	######	2E-14	up	yes
Manes.04G00360	42.121448	76.61677	0.549768	-0.86310513	0.016	0.043	down	no
Manes.04G00370	0	1.813567	#NAME?	#NAME?	0.1181	0.2246	down	no
Manes.04G00510	24.430212	37.196	0.6567967	-0.60648119	0.1517	0.2737	down	no
Manes.04G00710	1445.9264	1154.193	1.2527597	0.325109675	0.1013	0.1993	up	no
Manes.04G00810	149.67113	166.8376	0.8971068	-0.15664841	0.5306	0.6912	down	no
Manes.04G00970	82.093267	18.78919	4.3691755	2.127361068	######	6E-09	up	yes
Manes.04G01200	3.450488	2.392882	1.4419803	0.528051434	0.7709	0.8919	up	no
Manes.04G01240	78.705233	143.3811	0.5489232	-0.86532369	0.0015	0.0056	down	no
Manes.04G01410	1.0855284	0.307855	3.5261085	1.818076854	0.5219	0.6841	up	no
Manes.04G01520	0	2.715702	#NAME?	#NAME?	0.046	0.1046	down	no
Manes.04G01590	190.57387	117.3433	1.6240706	0.699614364	0.0023	0.0081	up	no
Manes.04G01840	10.376317	0.307855	33.70526	5.074901862	######	9E-05	up	yes
Manes.04G02110	11.830792	28.54694	0.414433	-1.27078934	0.0124	0.0347	down	yes
Manes.04G02140	25.682105	54.2608	0.4733087	-1.07914674	0.0069	0.0209	down	yes
Manes.04G02400	0.3835559	1.852795	0.2070147	-2.27219487	0.3345	0.4999	down	no
Manes.04G02420	13.82384	8.788494	1.5729475	0.653470562	0.3973	0.5622	up	no
Manes.04G02480	300.9811	239.0644	1.2589958	0.332273421	0.0919	0.1843	up	no

Manes.04G02500	16.75188	0.923564	18.138307	4.180967934	0.023	0.0585	up	no
Manes.04G02610	67.285698	294.1399	0.228754	-2.12813085	######	9E-05	down	yes
Manes.04G02720	1565.1119	70.01416	22.354219	4.482475214	######	3E-36	up	yes
Manes.04G02960	10.780016	16.42624	0.6562681	-0.6076429	0.3138	0.4772	down	no
Manes.04G03070	20.415129	32.21976	0.6336213	-0.65830727	0.15	0.2713	down	no
Manes.04G03240	1.7773718	0	Inf	Inf	0.0674	0.1427	up	no
Manes.04G03270	729.09449	625.7203	1.1652082	0.220587815	0.1613	0.2874	up	no
Manes.04G03280	1.0753993	0.307855	3.4932062	1.804551799	0.5228	0.6847	up	no
Manes.04G03380	283.16813	254.5894	1.1122541	0.15348646	0.4266	0.5921	up	no
Manes.04G03600	357.00961	174.0082	2.0516824	1.036807451	######	5E-07	up	yes
Manes.04G03890	16.999278	2.752096	6.1768475	2.626870713	######	0.0003	up	yes
Manes.04G03910	77.334344	63.24609	1.2227529	0.290132866	0.3595	0.527	up	no
Manes.04G03920	10.092537	2.133553	4.7303899	2.241959111	0.0039	0.0129	up	yes
Manes.04G04250	19.10531	44.03119	0.433904	-1.20455223	0.005	0.0158	down	yes
Manes.04G05020	595.10554	492.8777	1.2074101	0.271915779	0.0922	0.1847	up	no
Manes.04G05540	2.6342456	1.798601	1.4646079	0.550514466	0.8034	0.9164	up	no
Manes.04G05630	44.266252	64.29284	0.6885098	-0.53845084	0.1324	0.2454	down	no
Manes.04G05730	174.04342	198.4431	0.8770446	-0.18927789	0.4169	0.5819	down	no
Manes.04G05740	4.1785983	33.76408	0.1237587	-3.01439839	######	0.0004	down	yes
Manes.04G05800	195.46851	220.465	0.886619	-0.17361376	0.4642	0.6272	down	no
Manes.04G05900	6.4726541	3.945703	1.6404313	0.714075151	0.3755	0.5442	up	no
Manes.04G05970	103.90378	97.74531	1.0630053	0.088148757	0.737	0.8655	up	no
Manes.04G06950	4.6055202	0.603578	7.6303699	2.931753001	0.0169	0.0453	up	yes
Manes.04G07090	23.292408	7.602768	3.0636748	1.615263163	0.0014	0.0051	up	yes
Manes.04G07290	9.8609179	4.229294	2.3315752	1.221304987	0.0945	0.1884	up	no
Manes.04G07710	83.496271	80.12812	1.0420346	0.059403117	0.8564	0.9548	up	no
Manes.04G07960	34.496956	73.02085	0.4724261	-1.08183939	0.0191	0.0502	down	no
Manes.04G08230	407.87152	410.4103	0.9938141	-0.00895215	1	1	down	no
Manes.04G08250	10.943531	11.88484	0.9207976	-0.11904402	0.9658	1	down	no
Manes.04G08320	80.86878	62.19285	1.3002907	0.378834202	0.1846	0.3193	up	no
Manes.04G08330	3.1566941	1.532809	2.0594172	1.042236138	0.5601	0.716	up	no
Manes.04G08860	2202.7597	2006.231	1.0979593	0.134824544	0.3357	0.5011	up	no
Manes.04G08960	4.8356898	3.339291	1.4481187	0.534179906	0.6314	0.7767	up	no
Manes.04G09090	319.74238	182.8525	1.7486356	0.806229652	######	0.0002	up	no
Manes.04G09120	370.90755	265.4634	1.3972077	0.482546516	0.0082	0.0243	up	no
Manes.04G09650	38.391429	20.129	1.90727	0.931509083	0.0183	0.0483	up	no
Manes.04G09790	216.22198	291.07	0.7428522	-0.42885292	0.0558	0.1224	down	no
Manes.04G09900	236.18784	230.1432	1.0262645	0.037402604	0.8689	0.9635	up	no
Manes.04G10140	22.975621	27.9577	0.8217993	-0.28314206	0.5399	0.7001	down	no
Manes.04G11540	24.179784	18.77218	1.2880645	0.365204795	0.5567	0.714	up	no
Manes.04G11580	16.287176	5.146395	3.164774	1.662102467	0.0095	0.0276	up	yes
Manes.04G11880	18.620529	5.749972	3.2383684	1.695267119	0.0039	0.0128	up	yes
Manes.04G12150	3.8051715	8.460006	0.4497836	-1.15269714	0.2376	0.3865	down	no
Manes.04G12220	1.1506677	0.615709	1.8688497	0.902150563	0.8638	0.9599	up	no
Manes.04G12230	1.8873921	1.208572	1.5616709	0.643090452	0.7686	0.89	up	no
Manes.04G12290	5.1279687	0.307855	16.657117	4.05806682	0.0029	0.0099	up	yes
Manes.04G12370	61.174777	119.8211	0.5105508	-0.96987363	0.0009	0.0034	down	no
Manes.04G12590	11.363354	13.14557	0.8644249	-0.21018754	0.7831	0.9003	down	no

Manes.04G12670	99.03177	19.86116	4.9862015	2.3179412	######	1E-10	up	yes
Manes.04G12970	6.6521781	0.591446	11.24731	3.491508126	0.0994	0.1963	up	no
Manes.04G13180	61.605834	67.03643	0.9189903	-0.1218784	0.7454	0.8726	down	no
Manes.04G13350	13.48365	9.677875	1.3932449	0.478448882	0.3937	0.5611	up	no
Manes.04G13950	6.9213492	12.74349	0.5431281	-0.8806357	0.1876	0.3233	down	no
Manes.04G14140	718.63483	362.1583	1.9843111	0.988638222	######	4E-08	up	no
Manes.04G14230	81.462477	180.2622	0.4519111	-1.14588923	######	9E-05	down	yes
Manes.04G14400	8.6697337	19.1399	0.4529665	-1.14252381	0.0498	0.1116	down	no
Manes.04G14480	23.885875	28.00606	0.8528824	-0.22958119	0.702	0.8379	down	no
Manes.04G14530	0	4.24426	#NAME?	#NAME?	0.0074	0.0224	down	yes
Manes.04G14720	8.2109095	18.19854	0.4511851	-1.14820863	0.0555	0.1218	down	no
Manes.04G14750	87.16779	13.28641	6.5606707	2.713843313	######	1E-05	up	yes
Manes.04G14880	9.0373959	5.211303	1.7341912	0.794262936	0.3606	0.5283	up	no
Manes.04G14910	1.8627693	0.309272	6.0230857	2.590502792	0.1944	0.3326	up	no
Manes.04G15120	6.0195944	7.327056	0.821557	-0.28356742	0.8341	0.9392	down	no
Manes.04G15250	95.668555	46.68703	2.0491462	1.035022942	0.0015	0.0055	up	yes
Manes.04G15350	2.1913151	14.86775	0.1473871	-2.76231746	0.003	0.0103	down	yes
Manes.04G15500	1.4937071	0.591446	2.5255167	1.336578594	0.6689	0.8095	up	no
Manes.04G15750	10.701898	33.46331	0.3198099	-1.64471364	0.0009	0.0035	down	yes
Manes.04G15790	162.7228	9.976432	16.310721	4.027748631	######	1E-25	up	yes
Manes.04G15850	4.7256696	8.256288	0.5723722	-0.80497456	0.3418	0.5074	down	no
Manes.04G16190	0.7468535	2.456373	0.3040473	-1.71763238	0.468	0.6308	down	no
Manes.05G00160	27.282983	42.8398	0.6368607	-0.65095029	0.1041	0.2037	down	no
Manes.05G00340	2.1956796	0.307855	7.1321988	2.834346911	0.1234	0.2323	up	no
Manes.05G00560	333.98333	1.813567	184.15827	7.524802347	######	2E-92	up	yes
Manes.05G00570	7.2657887	3.6243	2.0047428	1.003417169	0.208	0.3492	up	no
Manes.05G00750	10.406589	39.79685	0.2614928	-1.93515703	######	0.0004	down	yes
Manes.05G00860	7.6537091	4.926295	1.5536442	0.635656187	0.5385	0.6987	up	no
Manes.05G00980	3791.1438	1897.457	1.998013	0.99856597	0.0098	0.0282	up	no
Manes.05G01020	3.2565853	2.441407	1.3338967	0.415646953	0.7366	0.8653	up	no
Manes.05G01390	1.8222528	0	Inf	Inf	0.0665	0.141	up	no
Manes.05G01400	1.1202803	0.309272	3.6223188	1.856913532	0.4558	0.6194	up	no
Manes.05G01500	156.18901	42.16689	3.7040678	1.88911049	######	3E-11	up	yes
Manes.05G01800	2.619752	0.927815	2.8235722	1.497521499	0.6377	0.7823	up	no
Manes.05G02120	779.1325	757.8825	1.0280386	0.039894449	0.7616	0.8855	up	no
Manes.05G02150	750.54386	530.133	1.4157653	0.501582135	0.0011	0.0043	up	no
Manes.05G02310	13263.494	9153.484	1.4490105	0.535068028	0.0012	0.0047	up	no
Manes.05G02360	0.3734268	3.957834	0.0943513	-3.40581389	0.0397	0.0926	down	no
Manes.05G02450	6.3278819	57.23316	0.1105632	-3.17705678	######	2E-06	down	yes
Manes.05G02580	3582.6148	4655.384	0.7695638	-0.37788713	0.0661	0.1404	down	no
Manes.05G02600	7.3786585	3.352839	2.2007193	1.137975126	0.1358	0.2503	up	no
Manes.05G02960	85.286277	45.27412	1.8837755	0.91362704	0.0147	0.0399	up	no
Manes.05G03220	3.9730515	11.81426	0.3362928	-1.57221015	0.1024	0.201	down	no
Manes.05G03420	77.497876	70.28576	1.1026114	0.140924389	0.6256	0.7713	up	no
Manes.05G03640	188.57214	195.099	0.9665457	-0.04909012	0.8441	0.946	down	no
Manes.05G03690	16.540568	16.63642	0.9942385	-0.00833618	1	1	down	no
Manes.05G03890	15.074399	18.54058	0.8130492	-0.29858551	0.587	0.7391	down	no
Manes.05G04060	6.5334288	0.911432	7.168311	2.841633237	0.0059	0.0183	up	yes

Manes.05G04120	16.640345	2.43211	6.8419372	2.77440487	######	0.0002	up	yes
Manes.05G04500	6.85621	0.309272	22.168897	4.470465061	0.0004	0.0017	up	yes
Manes.05G04900	4.630143	0.926398	4.9980078	2.321353166	0.0391	0.0914	up	no
Manes.05G05210	2.9874142	5.742887	0.5201938	-0.94287889	0.6237	0.7695	down	no
Manes.05G05710	75.64115	90.20648	0.8385335	-0.25405976	0.3913	0.5595	down	no
Manes.05G05780	510.42153	908.9982	0.5615209	-0.83258825	######	5E-05	down	no
Manes.05G05930	1.4242033	0.924981	1.5397115	0.622660048	0.7915	0.9068	up	no
Manes.05G06040	13.760216	2.970779	4.6318542	2.211589841	0.1949	0.3333	up	no
Manes.05G06090	64.027549	55.8823	1.1457573	0.196301417	0.7222	0.8543	up	no
Manes.05G06150	26.608368	5.780698	4.6029677	2.202564312	0.0004	0.0016	up	yes
Manes.05G06420	27.964697	66.88882	0.4180773	-1.25815831	0.0007	0.0029	down	yes
Manes.05G06450	112.09303	113.158	0.9905886	-0.01364209	1	1	down	no
Manes.05G06710	1.4937071	3.043568	0.490775	-1.02686629	0.7316	0.8613	down	no
Manes.05G06730	7.8622204	4.83854	1.6249158	0.700364969	0.4314	0.5973	up	no
Manes.05G06770	2.2506897	0	Inf	Inf	0.0369	0.087	up	no
Manes.05G06820	20.665671	69.70631	0.2964677	-1.75405302	######	0.0004	down	yes
Manes.05G06840	0.3734268	5.444952	0.0685822	-3.86602204	0.0181	0.0478	down	yes
Manes.05G06900	22.809076	16.38418	1.3921405	0.477304826	0.2935	0.4536	up	no
Manes.05G07130	3.2667144	0	Inf	Inf	0.0076	0.0228	up	yes
Manes.05G07390	7262.614	734.2363	9.8913851	3.306172558	######	4E-117	up	yes
Manes.05G07510	64.63388	47.40325	1.3634905	0.447304662	0.1701	0.3	up	no
Manes.05G07770	2.4098406	1.81215	1.3298242	0.41123549	0.7673	0.8892	up	no
Manes.05G08110	30.324072	40.07668	0.7566513	-0.40229955	0.5729	0.7267	down	no
Manes.05G08200	1.1202803	0.604995	1.8517194	0.888865458	0.7049	0.84	up	no
Manes.05G08610	426.76733	75.92198	5.6211302	2.490860232	######	2E-34	up	yes
Manes.05G08650	2.1160467	0.604995	3.4976288	1.806377182	0.263	0.4171	up	no
Manes.05G08870	44.689219	114.0669	0.3917807	-1.35188184	0.0038	0.0124	down	yes
Manes.05G08890	4.7054113	0.307855	15.284529	3.934000201	0.0054	0.017	up	yes
Manes.05G09020	43.017323	136.4747	0.3152036	-1.66564429	######	7E-07	down	yes
Manes.05G09190	1.5038362	0	Inf	Inf	0.1181	0.2246	up	no
Manes.05G09230	2.574871	4.576377	0.5626439	-0.82970587	0.562	0.7171	down	no
Manes.05G09250	1.6427288	10.37913	0.1582724	-2.6595188	0.063	0.1351	down	no
Manes.05G09410	3.5301209	4.822157	0.7320626	-0.44996117	0.7443	0.8717	down	no
Manes.05G09420	27.303241	70.94574	0.3848468	-1.37764387	0.0002	0.0009	down	yes
Manes.05G09660	18.089171	23.42543	0.7722023	-0.37294922	0.522	0.6841	down	no
Manes.05G09850	6.6130617	1.516427	4.3609503	2.124642538	0.024	0.0607	up	no
Manes.05G09920	4.9094432	8.474971	0.5792873	-0.78764906	0.357	0.5241	down	no
Manes.05G10140	19.032957	31.0854	0.6122797	-0.70773733	0.1191	0.2259	down	no
Manes.05G10170	64.671021	50.09123	1.2910647	0.368561332	0.2552	0.4083	up	no
Manes.05G10300	9.0099236	6.411373	1.4053033	0.490881529	0.467	0.63	up	no
Manes.05G10340	13.334629	42.27545	0.3154225	-1.66464241	0.0003	0.0014	down	yes
Manes.05G10360	17.364205	30.74761	0.5647335	-0.824358	0.0772	0.1598	down	no
Manes.05G10460	7.4598063	12.5141	0.5961123	-0.74634406	0.261	0.4149	down	no
Manes.05G10490	21.890092	22.51275	0.9723419	-0.04046441	0.9654	1	down	no
Manes.05G10790	0.7569827	1.208572	0.6263446	-0.67497155	0.9358	0.9976	down	no
Manes.05G10830	7.4437977	12.68363	0.5868822	-0.76885713	0.316	0.4797	down	no
Manes.05G10940	18.254316	3.337874	5.4688453	2.451236251	0.0205	0.0532	up	no
Manes.05G10960	1.1202803	0.591446	1.8941375	0.921541095	0.8608	0.9578	up	no

Manes.05G11290	47.775999	19.13769	2.4964351	1.319869391	0.0005	0.002	up	yes
Manes.05G11350	13.130597	10.65705	1.2321043	0.301124352	0.6125	0.7595	up	no
Manes.05G11550	9.3630921	4.59276	2.0386635	1.027623654	0.1566	0.2808	up	no
Manes.05G11690	1.5342235	21.37697	0.0717699	-3.8004767	0.0338	0.0809	down	no
Manes.05G11920	14.676349	17.94063	0.8180511	-0.28973711	0.682	0.8207	down	no
Manes.05G11980	42.885414	15.47921	2.7705175	1.470155458	0.0002	0.001	up	yes
Manes.05G12050	1.1202803	0	Inf	Inf	0.1992	0.3379	up	no
Manes.05G12240	0.6570915	1.207155	0.5443306	-0.87744487	0.9796	1	down	no
Manes.05G12300	23.875746	13.70629	1.7419559	0.800708089	0.081	0.166	up	no
Manes.05G12470	15.440431	13.66989	1.1295211	0.175711232	0.7055	0.8404	up	no
Manes.05G12720	14.77909	40.70341	0.3630922	-1.46159223	0.0013	0.005	down	yes
Manes.05G12730	4.5808975	22.79413	0.2009683	-2.31496032	0.0003	0.0013	down	yes
Manes.05G12750	6.1353792	12.81124	0.4789061	-1.06218533	0.2077	0.3489	down	no
Manes.05G12770	12.338862	13.06631	0.9443262	-0.08264287	0.9589	1	down	no
Manes.05G12810	134.79908	127.61	1.0563364	0.079069291	0.7297	0.8598	up	no
Manes.05G12870	112.18285	102.7789	1.0914971	0.126308243	0.6604	0.802	up	no
Manes.05G12890	0	2.132136	#NAME?	#NAME?	0.0818	0.1673	down	no
Manes.05G12910	42.323964	71.11607	0.5951392	-0.74870085	0.0303	0.0739	down	no
Manes.05G13060	27.912651	107.0151	0.260829	-1.93882362	######	0.0001	down	yes
Manes.05G13290	82.665305	108.4537	0.7622176	-0.39172509	0.1804	0.3134	down	no
Manes.05G13460	1.8222528	7.262148	0.2509248	-1.99467314	0.1025	0.2013	down	no
Manes.05G13510	41.004247	16.3314	2.5107614	1.328124919	0.0077	0.0229	up	yes
Manes.05G13690	443.64018	294.0079	1.5089398	0.593535279	0.0007	0.0027	up	no
Manes.05G13910	71.044424	9.397118	7.5602356	2.9184312	######	2E-12	up	yes
Manes.05G14030	239.44808	244.0335	0.9812097	-0.02736655	0.9447	1	down	no
Manes.05G14130	141.33787	331.6548	0.4261596	-1.2305344	######	3E-05	down	yes
Manes.05G14260	112.59411	99.35294	1.1332741	0.180496779	0.5051	0.6685	up	no
Manes.05G14480	0.7121017	7.020619	0.10143	-3.3014431	0.0072	0.0218	down	yes
Manes.05G15080	23.998925	60.0894	0.399387	-1.32414067	0.0059	0.0183	down	yes
Manes.05G15340	1.1202803	0.604995	1.8517194	0.888865458	0.7049	0.84	up	no
Manes.05G15450	36.418985	0	Inf	Inf	######	2E-16	up	yes
Manes.05G15810	22.08836	4.576377	4.8266037	2.271008379	######	0.0003	up	yes
Manes.05G16010	402.7239	100.0389	4.0256721	2.009229663	######	3E-13	up	yes
Manes.05G16040	965.32638	625.7426	1.5426893	0.625447557	0.0006	0.0023	up	no
Manes.05G16200	208.80343	206.6803	1.0102725	0.01474451	0.9243	0.9966	up	no
Manes.05G16530	455.24619	119.0928	3.8226175	1.934560834	######	1E-23	up	yes
Manes.05G16540	495.10153	138.9172	3.5640033	1.83349869	######	1E-22	up	yes
Manes.05G16550	695.72211	181.6039	3.8309865	1.937715951	######	1E-29	up	yes
Manes.05G16560	338.12364	109.154	3.0976741	1.631185351	######	1E-07	up	yes
Manes.05G16650	0.7019725	10.04134	0.0699082	-3.83839354	0.0006	0.0024	down	yes
Manes.05G16980	1.832382	0.900718	2.0343576	1.02457332	0.578	0.7312	up	no
Manes.05G17010	0	14.84853	#NAME?	#NAME?	######	3E-06	down	yes
Manes.05G17260	27.193221	25.07751	1.0843667	0.116852703	0.8262	0.9338	up	no
Manes.05G17270	15.803729	14.99411	1.0539958	0.075869092	0.9016	0.9878	up	no
Manes.05G17300	502.15206	472.105	1.0636449	0.089016573	0.6972	0.8335	up	no
Manes.05G17310	4729.1717	2899.419	1.6310757	0.705823701	0.0017	0.0063	up	no
Manes.05G17320	351.39729	174.393	2.0149741	1.010761279	0.0084	0.0249	up	yes
Manes.05G17330	5.0788381	13.41136	0.3786968	-1.40088493	0.0796	0.1639	down	no

Manes.05G17340	5.1686001	13.19409	0.3917359	-1.35204656	0.1742	0.3053	down	no
Manes.05G17500	1.0305183	0.911432	1.1306583	0.177163041	1	1	up	no
Manes.05G17800	1.3691932	0	Inf	Inf	0.1225	0.2309	up	no
Manes.05G17880	7.4539268	10.34353	0.7206369	-0.47265566	0.5566	0.714	down	no
Manes.05G18050	43.10837	31.69969	1.3598988	0.443499293	0.2193	0.364	up	no
Manes.05G18210	420.89431	243.2808	1.7300762	0.79083559	######	7E-05	up	no
Manes.05G18300	225.88381	343.8293	0.6569649	-0.60611176	0.0028	0.0096	down	no
Manes.05G18770	310.80091	58.44599	5.3177459	2.41081485	######	8E-25	up	yes
Manes.05G18790	40.79824	20.99633	1.9431133	0.958370028	0.0348	0.083	up	no
Manes.05G19030	796.6622	437.6482	1.8203257	0.864196644	######	2E-07	up	no
Manes.05G19080	136.14696	133.6007	1.0190587	0.027237159	0.9183	0.9966	up	no
Manes.05G19090	9.2719301	4.869266	1.904174	0.929165298	0.1988	0.3379	up	no
Manes.05G19140	31.989729	84.82328	0.3771338	-1.40685159	0.0004	0.0017	down	yes
Manes.05G19290	2.1956796	0	Inf	Inf	0.038	0.0892	up	no
Manes.05G19310	32.794392	27.33837	1.199574	0.262522105	0.4943	0.6574	up	no
Manes.05G19420	61.455297	101.9297	0.6029187	-0.72996468	0.0166	0.0444	down	no
Manes.05G19540	9.7350039	12.80699	0.7601323	-0.39567747	0.5759	0.7293	down	no
Manes.05G19570	39.151327	24.84135	1.5760549	0.656317754	0.1065	0.2075	up	no
Manes.05G19590	215.62751	207.2177	1.0405843	0.057393806	0.7522	0.8781	up	no
Manes.05G19630	32.75684	26.49184	1.2364878	0.306248003	0.4212	0.5866	up	no
Manes.05G19780	117.04276	32.59032	3.5913355	1.844520443	######	3E-09	up	yes
Manes.05G19790	4.8154316	2.714285	1.7741071	0.827093102	0.4082	0.5735	up	no
Manes.05G19900	4.4217466	11.82781	0.3738432	-1.41949481	0.0512	0.1139	down	no
Manes.05G19940	73.482793	26.24686	2.7996796	1.485261751	######	8E-05	up	yes
Manes.05G20130	7.115252	2.688605	2.6464479	1.404057229	0.3207	0.485	up	no
Manes.05G20150	35.896306	20.65349	1.7380258	0.797449529	0.0503	0.1124	up	no
Manes.05G20160	29.493156	4.280654	6.8898711	2.784476987	######	1E-06	up	yes
Manes.05G20630	161.23863	107.9968	1.4929947	0.578209032	0.0156	0.0422	up	no
Manes.05G20720	27.183207	9.630766	2.8225382	1.496993122	0.0482	0.1085	up	no
Manes.06G00280	2.2810771	7.910622	0.2883562	-1.79407597	0.0666	0.1414	down	no
Manes.06G00300	39.163021	29.1406	1.3439334	0.426461623	0.271	0.4267	up	no
Manes.06G00340	36.680531	39.99662	0.9170907	-0.12486367	0.8482	0.9491	down	no
Manes.06G00350	510.32185	198.1053	2.5760133	1.365140017	######	3E-06	up	yes
Manes.06G01540	1960.0015	559.9598	3.500254	1.807459623	######	2E-15	up	yes
Manes.06G01640	7.6131926	2.403596	3.1674178	1.663307187	0.0627	0.1347	up	no
Manes.06G01670	535.47136	374.2805	1.4306686	0.516689509	0.0053	0.0168	up	no
Manes.06G01790	0.7671118	1.208572	0.6347256	-0.655795	1	1	down	no
Manes.06G01810	17.170187	6.992105	2.4556536	1.296107094	0.0217	0.0559	up	no
Manes.06G02000	2078.7692	1318.336	1.5768134	0.657011956	######	2E-05	up	no
Manes.06G02410	84.662193	66.93987	1.2647499	0.338852091	0.2948	0.4551	up	no
Manes.06G02470	1.0753993	0.924981	1.1626182	0.217377388	1	1	up	no
Manes.06G02620	149.47612	144.2122	1.036501	0.051721446	0.8666	0.9621	up	no
Manes.06G02980	429.44657	341.315	1.2582118	0.331374761	0.0604	0.1306	up	no
Manes.06G02990	213.53195	182.541	1.1697752	0.226231303	0.2836	0.4415	up	no
Manes.06G03000	209.20166	173.7624	1.2039521	0.267778044	0.2186	0.363	up	no
Manes.06G03190	94.948813	118.5702	0.8007814	-0.32051962	0.242	0.3918	down	no
Manes.06G03270	1.1405385	4.547863	0.2507856	-1.99547365	0.1071	0.2084	down	no
Manes.06G03340	52.199556	35.66762	1.4635	0.549422753	0.3537	0.5207	up	no

Manes.06G03350	0.7671118	1.237086	0.6200956	-0.68943752	0.8619	0.9586	down	no
Manes.06G03360	6.0746045	8.1479	0.7455424	-0.42363777	0.6521	0.7946	down	no
Manes.06G03500	25.689385	43.74822	0.5872098	-0.76805209	0.0585	0.1273	down	no
Manes.06G03700	77.849579	133.3166	0.583945	-0.77609556	0.006	0.0187	down	no
Manes.06G03710	298.81388	209.6087	1.4255795	0.511548453	0.0089	0.0261	up	no
Manes.06G03870	9.1430516	10.3577	0.8827301	-0.17995571	0.848	0.9488	down	no
Manes.06G03930	145.52704	244.9006	0.5942291	-0.75090888	0.0032	0.0108	down	no
Manes.06G04110	9.9695381	14.34263	0.6950984	-0.52471094	0.3952	0.5616	down	no
Manes.06G04150	1.5342235	0.617126	2.4860778	1.313871468	0.4624	0.6255	up	no
Manes.06G04280	2.181186	1.223538	1.7826878	0.834054055	0.6856	0.8236	up	no
Manes.06G04300	133.97719	56.30818	2.3793556	1.250570882	######	4E-05	up	yes
Manes.06G04370	23.019102	50.20954	0.4584607	-1.12512993	0.0214	0.0552	down	no
Manes.06G04750	3.0033079	3.924274	0.7653156	-0.38587338	0.8633	0.9597	down	no
Manes.06G04760	1.3590641	26.07073	0.0521299	-4.26174542	######	9E-05	down	yes
Manes.06G04820	1.1304094	2.441407	0.4630155	-1.11086765	0.5304	0.6912	down	no
Manes.06G05140	10.177934	40.8942	0.2488845	-2.00645149	######	0.0002	down	yes
Manes.06G05240	3.5301209	5.205635	0.6781346	-0.56035653	0.6648	0.8058	down	no
Manes.06G05250	65.92344	101.5999	0.6488532	-0.62403598	0.0359	0.0851	down	no
Manes.06G05340	41.183426	53.4532	0.7704577	-0.37621234	0.314	0.4773	down	no
Manes.06G05770	110.77319	9.102812	12.169118	3.605152752	######	1E-22	up	yes
Manes.06G05810	0.7121017	2.722165	0.2615939	-1.93459933	0.2803	0.4375	down	no
Manes.06G05980	9877.5766	16351.24	0.6040873	-0.72717106	0.0089	0.0261	down	no
Manes.06G06190	4.4318757	0	Inf	Inf	0.0016	0.0058	up	yes
Manes.06G06280	10.216936	0.912849	11.192359	3.484442282	0.0001	0.0006	up	yes
Manes.06G06390	55.195174	369.2423	0.1494823	-2.74195385	######	1E-14	down	yes
Manes.06G06500	289.88548	292.3775	0.9914766	-0.0123494	0.9738	1	down	no
Manes.06G06730	191.76749	222.8187	0.8606436	-0.21651213	0.3445	0.5104	down	no
Manes.06G06750	141.26632	135.1253	1.0454467	0.064119495	0.7845	0.9017	up	no
Manes.06G06770	138.40266	10.34069	13.384273	3.742466857	######	8E-07	up	yes
Manes.06G06840	133.36422	106.2069	1.2557025	0.32849469	0.219	0.3636	up	no
Manes.06G06900	277.11244	160.4763	1.7268119	0.788110919	0.0001	0.0005	up	no
Manes.06G07000	44.327256	34.11967	1.2991702	0.377590444	0.3026	0.4642	up	no
Manes.06G07010	17.834378	19.80618	0.9004453	-0.15128942	0.8868	0.9772	down	no
Manes.06G07300	3.8485376	2.427859	1.585157	0.664625777	0.5079	0.6714	up	no
Manes.06G07420	48.528437	0.900718	53.87752	5.75161155	######	1E-17	up	yes
Manes.06G07470	3364.7417	2648.056	1.2706461	0.345562289	0.2067	0.3476	up	no
Manes.06G07480	482.07038	497.403	0.9691746	-0.04517147	0.8412	0.9439	down	no
Manes.06G07550	11.034808	31.47959	0.3505385	-1.51235517	0.0041	0.0134	down	yes
Manes.06G07790	41.085165	57.9141	0.7094156	-0.49529706	0.1571	0.2815	down	no
Manes.06G07810	0	1.490747	#NAME?	#NAME?	0.1864	0.3217	down	no
Manes.06G07830	23.393814	24.63984	0.9494303	-0.07486595	0.9332	0.9966	down	no
Manes.06G08740	281.58745	248.444	1.133404	0.180662201	0.3562	0.5234	up	no
Manes.06G08770	327.7876	306.8879	1.068102	0.095049386	0.6054	0.7541	up	no
Manes.06G08790	1.4792134	0	Inf	Inf	0.1189	0.2257	up	no
Manes.06G08890	3.3303387	4.832872	0.6891014	-0.53721185	0.8509	0.9511	down	no
Manes.06G09020	1.8671339	7.289245	0.2561491	-1.96494401	0.1905	0.3274	down	no
Manes.06G09120	8.3803044	4.280654	1.9577158	0.969171329	0.1859	0.3209	up	no
Manes.06G09170	54.11971	26.73337	2.0244251	1.017512288	0.0063	0.0193	up	yes

Manes.06G09230	28.665155	39.85246	0.7192819	-0.47537083	0.2577	0.411	down	no
Manes.06G09280	0.7569827	1.529975	0.4947679	-1.01517619	0.7331	0.8624	down	no
Manes.06G09590	47.841483	45.24543	1.0573771	0.080489957	0.8315	0.9374	up	no
Manes.06G09810	43.391969	41.74386	1.0394814	0.055863887	0.8182	0.9274	up	no
Manes.06G09900	69.893806	181.7703	0.3845172	-1.37887987	######	0.0002	down	yes
Manes.06G09990	840.06348	744.8234	1.1278693	0.173599912	0.2524	0.405	up	no
Manes.06G10030	99.298321	39.46615	2.5160376	1.33115348	######	5E-05	up	yes
Manes.06G10080	177.51853	145.7203	1.2182142	0.284767784	0.2047	0.345	up	no
Manes.06G10340	460.25099	643.9769	0.7147011	-0.48458801	0.0044	0.0141	down	no
Manes.06G10490	5.9863575	0.591446	10.12156	3.339359767	0.103	0.2021	up	no
Manes.06G10750	1.4937071	0	Inf	Inf	0.1074	0.2085	up	no
Manes.06G10930	5.1338482	1.801435	2.8498652	1.510893702	0.2296	0.3761	up	no
Manes.06G11020	0.3734268	1.54069	0.2423764	-2.04467904	0.4422	0.6067	down	no
Manes.06G11230	62.600381	107.032	0.5848756	-0.77379826	0.0085	0.0249	down	no
Manes.06G11270	116.90386	74.23447	1.5747923	0.655161531	0.0126	0.0352	up	no
Manes.06G11290	167.34613	171.4887	0.9758437	-0.03527804	0.921	0.9966	down	no
Manes.06G11430	5.6114158	24.08258	0.2330073	-2.10155316	0.0006	0.0025	down	yes
Manes.06G11440	24.621199	22.83761	1.0780987	0.108489232	0.7828	0.9001	up	no
Manes.06G11630	1078.089	910.1885	1.1844678	0.244239023	0.1058	0.2065	up	no
Manes.06G11750	4.1178236	7.349902	0.5602556	-0.83584302	0.6293	0.7748	down	no
Manes.06G11910	2.9830496	5.216349	0.5718654	-0.80625249	0.4775	0.6401	down	no
Manes.06G12210	2.619752	6.032147	0.4342984	-1.20324131	0.2761	0.4325	down	no
Manes.06G12660	30.952111	9.098561	3.401869	1.766327594	0.0002	0.0008	up	yes
Manes.06G12680	18.37435	15.57121	1.1800205	0.238811904	0.6188	0.7649	up	no
Manes.06G13430	1.9076503	2.122838	0.8986319	-0.15419787	1	1	down	no
Manes.06G13500	108.30666	67.60552	1.6020387	0.679909008	0.0161	0.0433	up	no
Manes.06G13530	19.940296	14.27205	1.3971569	0.482494008	0.3005	0.4618	up	no
Manes.06G13750	0.3835559	1.81215	0.2116579	-2.24019352	0.3247	0.489	down	no
Manes.06G14010	11.139064	29.63357	0.3758934	-1.41160464	0.0451	0.103	down	no
Manes.06G14020	66.917806	100.8281	0.6636821	-0.59143574	0.0457	0.104	down	no
Manes.06G14210	21.520915	8.864117	2.4278691	1.279690618	0.0292	0.0715	up	no
Manes.06G14260	12.893213	25.28044	0.5100075	-0.97140954	0.0573	0.125	down	no
Manes.06G14610	6.4075148	1.824281	3.5123503	1.812436728	0.048	0.1082	up	no
Manes.06G14620	7.8491268	30.11331	0.2606531	-1.93979715	0.0045	0.0144	down	yes
Manes.06G14730	66.063782	46.18963	1.430273	0.516290569	0.1042	0.2039	up	no
Manes.06G14750	248.066	309.2953	0.8020361	-0.31826097	0.1229	0.2313	down	no
Manes.06G14960	3.54025	8.601159	0.4116015	-1.28067986	0.2292	0.3758	down	no
Manes.06G15580	121.77711	136.4886	0.8922145	-0.16453745	0.5593	0.7154	down	no
Manes.06G15690	3.101684	7.277113	0.4262245	-1.23031457	0.1967	0.3356	down	no
Manes.06G15930	394.46653	405.9237	0.971775	-0.04130573	0.833	0.9384	down	no
Manes.06G16210	112.93577	126.0293	0.8961073	-0.15825655	0.5404	0.7004	down	no
Manes.06G16870	7.7723435	2.147101	3.6199238	1.855959336	0.0232	0.0591	up	no
Manes.06G17000	368.53554	362.7994	1.0158109	0.022631831	0.9213	0.9966	up	no
Manes.06G17050	0	1.517844	#NAME?	#NAME?	0.1732	0.3041	down	no
Manes.06G17060	508.18772	545.5889	0.9314481	-0.10245273	0.694	0.8309	down	no
Manes.06G17070	3.4114865	14.63268	0.2331415	-2.10072199	0.0091	0.0265	down	yes
Manes.06G17080	8292.493	8394.805	0.9878124	-0.01769098	1	1	down	no
Manes.06G17100	43.989981	56.94201	0.77254	-0.37231841	0.3012	0.4626	down	no

Manes.06G17110	35.508271	123.5196	0.2874706	-1.79851352	######	4E-07	down	yes
Manes.06G17230	0.7468535	2.14285	0.3485328	-1.52063372	0.5639	0.7187	down	no
Manes.06G17410	46.066846	53.35361	0.863425	-0.21185718	0.6021	0.753	down	no
Manes.06G17450	31.588715	37.34915	0.8457679	-0.24166633	0.5996	0.7512	down	no
Manes.06G17620	5.5766639	17.91132	0.3113486	-1.68339724	0.0202	0.0525	down	no
Manes.06G17720	1.4488261	2.122838	0.6824947	-0.55111019	0.872	0.9658	down	no
Manes.06G17830	6.1932389	13.46476	0.4599592	-1.1204222	0.1767	0.3085	down	no
Manes.07G00020	9.4209518	5.224852	1.8031041	0.85048273	0.417	0.582	up	no
Manes.07G00280	19.542246	41.04057	0.4761689	-1.0704546	0.086	0.1744	down	no
Manes.07G00400	0.7121017	2.726416	0.261186	-1.93685064	0.2601	0.4137	down	no
Manes.07G00470	319.75828	98.69545	3.2398483	1.695926248	######	9E-05	up	yes
Manes.07G00520	487.3928	34.39963	14.168548	3.824619962	######	6E-68	up	yes
Manes.07G00560	74.610517	117.4365	0.6353267	-0.65442944	0.0213	0.055	down	no
Manes.07G00570	41.334078	209.6045	0.1972003	-2.34226605	######	2E-10	down	yes
Manes.07G00790	41.441018	14.90273	2.7807675	1.475483114	0.0004	0.0016	up	yes
Manes.07G00840	2.9526623	1.209989	2.4402383	1.287022047	0.3224	0.487	up	no
Manes.07G00880	1.0305183	0.900718	1.1441079	0.194223181	1	1	up	no
Manes.07G00910	106.39605	118.0793	0.9010562	-0.15031105	0.5884	0.7403	down	no
Manes.07G01580	17.825764	106.1223	0.1679738	-2.57369184	######	1E-09	down	yes
Manes.07G01590	639.79358	392.83	1.6286779	0.703701294	######	0.0003	up	no
Manes.07G01620	156.95013	236.5645	0.6634559	-0.59192761	0.0084	0.0248	down	no
Manes.07G02170	0	1.539273	#NAME?	#NAME?	0.1849	0.3196	down	no
Manes.07G02210	13.503909	21.50285	0.6280055	-0.67115096	0.2126	0.3553	down	no
Manes.07G02260	20.924599	31.6447	0.6612355	-0.59676398	0.1894	0.3259	down	no
Manes.07G02590	199.25989	107.2216	1.8583926	0.894055303	######	0.0005	up	no
Manes.07G02800	175.7194	85.78751	2.0483099	1.03443401	0.0002	0.0007	up	yes
Manes.07G03010	0	3.344959	#NAME?	#NAME?	0.0201	0.0523	down	no
Manes.07G03070	125.18887	276.072	0.4534646	-1.14093815	######	7E-06	down	yes
Manes.07G03910	10.830661	15.53624	0.6971226	-0.52051564	0.4005	0.5658	down	no
Manes.07G04350	376.31064	419.2678	0.8975424	-0.15594806	0.3846	0.5543	down	no
Manes.07G04400	69.51707	27.10613	2.564625	1.358747873	######	0.0003	up	yes
Manes.07G04420	160.09169	53.30384	3.0033797	1.586586901	######	6E-09	up	yes
Manes.07G04450	96.936031	18.23777	5.3151262	2.410103935	######	4E-12	up	yes
Manes.07G04680	63.088307	50.44115	1.250731	0.322771528	0.353	0.5199	up	no
Manes.07G05390	12.537015	10.26002	1.2219284	0.289159732	0.6343	0.7794	up	no
Manes.07G05730	20.549772	31.21159	0.658402	-0.60295929	0.2575	0.4109	down	no
Manes.07G05760	97.022713	84.65044	1.1461573	0.196805	0.4534	0.6173	up	no
Manes.07G05830	7676.679	2315.416	3.3154636	1.72921063	######	1E-29	up	yes
Manes.07G05900	22.198265	7.016368	3.1637831	1.66165068	0.0014	0.0053	up	yes
Manes.07G06120	22.843827	43.82464	0.5212553	-0.93993787	0.0259	0.0649	down	no
Manes.07G06250	0.6570915	8.549177	0.0768602	-3.7016193	0.0177	0.0469	down	yes
Manes.07G06260	29.763547	17.56849	1.6941441	0.760556611	0.0877	0.1773	up	no
Manes.07G06460	22.057858	36.5083	0.6041875	-0.72693177	0.1016	0.1998	down	no
Manes.07G06490	4.3117263	5.443535	0.7920821	-0.33627817	0.8009	0.9143	down	no
Manes.07G06640	12.938094	7.328473	1.7654556	0.820040502	0.1806	0.3137	up	no
Manes.07G06690	0.7019725	1.51501	0.4633452	-1.10984052	0.7262	0.8572	down	no
Manes.07G06940	14.399899	12.22767	1.1776485	0.235909053	0.654	0.7961	up	no
Manes.07G07000	3.0481889	5.730755	0.5319	-0.91077304	0.4779	0.6405	down	no

Manes.07G07330	3.4114865	0.899301	3.7934883	1.923525107	0.146	0.2655	up	no
Manes.07G07560	60.032773	44.84637	1.3386318	0.420759186	0.2861	0.4446	up	no
Manes.07G08200	0	1.234252	#NAME?	#NAME?	0.2581	0.4117	down	no
Manes.07G08330	41.885398	44.71101	0.9368027	-0.0941829	0.8554	0.954	down	no
Manes.07G08360	4.4318757	1.234252	3.5907375	1.844280177	0.0925	0.1853	up	no
Manes.07G08440	18.558239	3.351422	5.5374216	2.469214369	0.0078	0.0233	up	yes
Manes.07G08680	549.6703	449.8772	1.2218229	0.289035233	0.0752	0.1564	up	no
Manes.07G08870	13.15377	14.87926	0.8840339	-0.17782632	0.9276	0.9966	down	no
Manes.07G08970	1.1202803	0.307855	3.6389926	1.863539113	0.4561	0.6195	up	no
Manes.07G09070	8.1574143	3.932154	2.0745408	1.052792049	0.1746	0.3058	up	no
Manes.07G09240	11.316958	15.5334	0.7285563	-0.45688757	0.4874	0.6503	down	no
Manes.07G09350	913.74708	180.6884	5.0570337	2.338291382	######	3E-43	up	yes
Manes.07G09420	5.8358208	1.182892	4.9335185	2.30261691	0.2961	0.4566	up	no
Manes.07G09530	214.52658	36.45269	5.885069	2.557059324	######	5E-22	up	yes
Manes.07G09640	73.210707	45.06031	1.6247271	0.700197413	0.0232	0.0589	up	no
Manes.07G09670	40.558237	34.70102	1.1687908	0.225016766	0.5459	0.7055	up	no
Manes.07G09770	7.1051228	30.37343	0.2339256	-2.09587843	0.0002	0.0007	down	yes
Manes.07G09840	348.42143	521.397	0.6682459	-0.58154895	0.1149	0.2202	down	no
Manes.07G10190	21.257624	24.67969	0.8613407	-0.21534412	0.6654	0.8064	down	no
Manes.07G10240	12.583525	7.35557	1.7107477	0.774627036	0.21	0.3518	up	no
Manes.07G10370	23.066783	29.80545	0.7739115	-0.36975953	0.4059	0.5712	down	no
Manes.07G10450	10.745379	23.63561	0.4546267	-1.13724573	0.0967	0.1919	down	no
Manes.07G10640	275.6558	385.6762	0.7147337	-0.48452231	0.0125	0.035	down	no
Manes.07G10660	8.1558994	3.692042	2.2090482	1.143424899	0.2068	0.3477	up	no
Manes.07G10750	20.215461	68.00427	0.2972675	-1.75016623	######	0.0001	down	yes
Manes.07G11180	304.78362	242.7376	1.2556096	0.328387974	0.1225	0.2309	up	no
Manes.07G11370	1.0753993	2.722165	0.395053	-1.33988199	0.4377	0.6036	down	no
Manes.07G11630	15.388451	27.94132	0.5507417	-0.86055225	0.126	0.2359	down	no
Manes.07G11780	67.198392	61.55257	1.0917236	0.126607628	0.6834	0.8218	up	no
Manes.07G11930	7.6825815	2.41431	3.1821019	1.669980036	0.093	0.186	up	no
Manes.07G11970	1.5240944	0.900718	1.6920889	0.758805346	0.7834	0.9006	up	no
Manes.07G12260	486.2336	438.6196	1.1085541	0.14867917	0.6015	0.7527	up	no
Manes.07G12370	16.531839	86.10213	0.1920027	-2.38080166	######	3E-08	down	yes
Manes.07G12430	164.21417	208.3711	0.788085	-0.34357686	0.1363	0.2511	down	no
Manes.07G12530	2.6732471	8.772906	0.3047163	-1.71446164	0.2793	0.4364	down	no
Manes.07G13350	14.10314	10.70053	1.3179853	0.398334238	0.4648	0.6278	up	no
Manes.07G13380	1.1202803	0.603578	1.8560668	0.892248635	0.7052	0.8402	up	no
Manes.07G13640	200.72432	187.9908	1.0677346	0.094553119	0.6445	0.7878	up	no
Manes.07G13660	2.4344634	25.55411	0.095267	-3.39187956	######	6E-05	down	yes
Manes.07G13970	67.598071	163.5546	0.4133059	-1.2747181	######	3E-05	down	yes
Manes.07G13980	1.9076503	3.073499	0.620677	-0.68808532	0.7497	0.8762	down	no
Manes.07G13990	133.85838	428.5699	0.3123374	-1.67882296	######	2E-09	down	yes
Manes.07G14120	9.7103812	0.309272	31.397585	4.972581696	######	8E-05	up	yes
Manes.07G14280	55.716518	29.21401	1.907185	0.931444817	0.0081	0.024	up	no
Manes.07G14370	18.48722	27.80425	0.6649063	-0.58877695	0.307	0.4695	down	no
Manes.07G14380	86.965043	27.61425	3.1492813	1.655022632	0.0002	0.0007	up	yes
Manes.08G00120	15.664951	2.750679	5.6949398	2.509680597	0.0002	0.0008	up	yes
Manes.08G00200	4.023697	15.46424	0.2601936	-1.94234251	0.0055	0.0172	down	yes

Manes.08G00420	95.481325	177.4632	0.5380346	-0.89422906	0.0006	0.0025	down	no
Manes.08G00440	7.8534913	17.3142	0.4535866	-1.14054992	0.0641	0.1372	down	no
Manes.08G00450	21.367644	34.92634	0.6117917	-0.70888767	0.1449	0.2639	down	no
Manes.08G00470	763.89213	724.6071	1.0542156	0.076169996	0.6995	0.8355	up	no
Manes.08G00530	72.357967	209.0959	0.3460516	-1.53094099	######	7E-06	down	yes
Manes.08G00660	5.9762284	41.76875	0.1430789	-2.80511678	######	3E-05	down	yes
Manes.08G00690	0.7569827	3.67566	0.2059447	-2.27967102	0.1209	0.2287	down	no
Manes.08G00880	5.9154537	0.604995	9.7776957	3.289494512	0.0051	0.0162	up	yes
Manes.08G01000	956.42613	938.0941	1.0195418	0.027920953	0.8362	0.9403	up	no
Manes.08G01090	355.51241	360.9874	0.9848334	-0.02204846	0.9173	0.9966	down	no
Manes.08G01100	6.0992273	26.11199	0.2335795	-2.09801427	0.0005	0.002	down	yes
Manes.08G01250	1.4937071	1.81215	0.8242735	-0.27880497	1	1	down	no
Manes.08G01450	14.609695	13.95003	1.047288	0.066658169	0.9612	1	up	no
Manes.08G02040	1.5240944	0.615709	2.4753485	1.307631646	0.773	0.8926	up	no
Manes.08G02110	61.349642	55.00694	1.1153072	0.157441171	0.6905	0.8277	up	no
Manes.08G02270	555.65999	586.4952	0.9474246	-0.07791695	0.642	0.7853	down	no
Manes.08G02310	35.603912	56.03939	0.6353373	-0.65440534	0.0749	0.1559	down	no
Manes.08G02390	6.7274464	23.97277	0.2806286	-1.83326591	0.0109	0.031	down	yes
Manes.08G02410	13.100094	17.9285	0.7306857	-0.45267718	0.4538	0.6178	down	no
Manes.08G02440	30.086508	34.02483	0.8842516	-0.17747121	0.6909	0.828	down	no
Manes.08G02680	2.2810771	0.307855	7.4095943	2.889394555	0.1228	0.2312	up	no
Manes.08G02900	365.45931	402.6949	0.907534	-0.13997642	0.4552	0.6192	down	no
Manes.08G03060	491.37617	647.009	0.759458	-0.39695799	0.0194	0.0507	down	no
Manes.08G03100	3.6995158	9.0828	0.4073101	-1.29580066	0.2627	0.4168	down	no
Manes.08G03200	1.8077592	0.900718	2.0070208	1.005055566	0.5804	0.7332	up	no
Manes.08G03260	53.72347	104.845	0.5124085	-0.96463371	0.0018	0.0065	down	no
Manes.08G03740	1.5139653	0.911432	1.661084	0.732125067	0.7834	0.9006	up	no
Manes.08G04110	8.127027	5.164989	1.5734838	0.653962323	0.387	0.5562	up	no
Manes.08G04120	34.064039	21.12363	1.6126038	0.689392039	0.2821	0.4396	up	no
Manes.08G04150	3.6647639	5.478512	0.6689342	-0.58006386	0.5931	0.7452	down	no
Manes.08G04570	2.9381686	1.219287	2.409744	1.268879894	0.4463	0.6105	up	no
Manes.08G04600	7.8519764	5.155692	1.5229724	0.606889753	0.4005	0.5659	up	no
Manes.08G04760	340.11769	247.8341	1.3723601	0.456659082	0.0144	0.0394	up	no
Manes.08G04790	1.3691932	0	Inf	Inf	0.1225	0.2309	up	no
Manes.08G04800	25.713892	16.10909	1.5962352	0.67467326	0.2517	0.4041	up	no
Manes.08G04810	313.83655	141.5327	2.2174132	1.148877651	0.0034	0.0112	up	yes
Manes.08G04860	5.1034609	17.08622	0.2986887	-1.74328565	0.0386	0.0904	down	no
Manes.08G04990	5.2337394	25.89756	0.2020939	-2.30690234	0.0006	0.0026	down	yes
Manes.08G05010	26.877719	12.77705	2.1035928	1.072855446	0.0274	0.0679	up	no
Manes.08G05020	934.56566	829.6323	1.1264817	0.171823888	0.2544	0.4074	up	no
Manes.08G05080	564.30624	145.9473	3.8665064	1.951030609	######	9E-08	up	yes
Manes.08G05150	13.608164	11.81426	1.1518421	0.203942922	0.719	0.8516	up	no
Manes.08G05210	217.98974	639.4276	0.3409139	-1.55252073	######	5E-14	down	yes
Manes.08G05530	1139.1267	35.10389	32.450156	5.020153527	######	8E-47	up	yes
Manes.08G05660	397.69459	119.8444	3.3184234	1.730497986	######	6E-09	up	yes
Manes.08G05750	81.954538	72.32022	1.1332175	0.180424791	0.5359	0.6962	up	no
Manes.08G05890	18.019667	5.184206	3.4758778	1.797377348	0.0016	0.0059	up	yes
Manes.08G06010	76.82232	76.08191	1.0097317	0.013972053	1	1	up	no

Manes.08G06300	74.917404	100.2351	0.7474171	-0.42001449	0.1521	0.2742	down	no
Manes.08G06440	165.01504	144.308	1.143492	0.193446232	0.4106	0.5761	up	no
Manes.08G06600	167.88429	208.7186	0.8043572	-0.31409174	0.2235	0.3685	down	no
Manes.08G06700	4.6359076	6.094221	0.7607055	-0.39459005	0.7493	0.8758	down	no
Manes.08G06710	156.53333	182.3833	0.8582657	-0.22050369	0.3477	0.5141	down	no
Manes.08G06900	270.66463	451.371	0.59965	-0.73780732	0.0003	0.0012	down	no
Manes.08G06960	232.62801	223.9304	1.0388408	0.054974624	0.7497	0.8762	up	no
Manes.08G07010	18.988076	14.37619	1.3208003	0.401412403	0.4196	0.5849	up	no
Manes.08G07030	16.401561	6.40491	2.5607793	1.356582943	0.0164	0.044	up	yes
Manes.08G07060	3.7443968	1.502878	2.4914838	1.317005219	0.2528	0.4056	up	no
Manes.08G07140	137.54223	85.16218	1.6150623	0.691589795	0.0061	0.019	up	no
Manes.08G07230	208.40545	146.7026	1.420598	0.506498361	0.0209	0.0542	up	no
Manes.08G07240	19.872307	25.21553	0.788098	-0.34355307	0.5138	0.6775	down	no
Manes.08G07350	98.839857	3.302897	29.925205	4.903289223	######	2E-06	up	yes
Manes.08G07530	428.99723	410.3547	1.0454304	0.06409701	0.7235	0.8554	up	no
Manes.08G07860	341.32359	274.3	1.2443443	0.315385736	0.0901	0.1812	up	no
Manes.08G07900	3367.1996	5270.876	0.6388312	-0.6464934	0.0015	0.0054	down	no
Manes.08G07910	82.471567	37.1689	2.2188323	1.149800654	0.0006	0.0025	up	yes
Manes.08G07920	1.4937071	1.798601	0.8304826	-0.26797815	1	1	down	no
Manes.08G07980	37.828464	35.89401	1.0538936	0.075729241	0.8363	0.9403	up	no
Manes.08G08060	1.8077592	4.253557	0.4249994	-1.23446734	0.3181	0.4821	down	no
Manes.08G08230	0.3835559	1.196441	0.3205808	-1.64124028	0.6013	0.7525	down	no
Manes.08G08400	305.38799	321.6985	0.9492988	-0.07506591	0.7129	0.8464	down	no
Manes.08G08500	312.58986	333.1506	0.938284	-0.09190346	0.6459	0.7891	down	no
Manes.08G08640	1.0406474	0.307855	3.3803221	1.757160705	0.5261	0.6874	up	no
Manes.08G08750	138.15781	280.3332	0.4928342	-1.0208257	0.0001	0.0005	down	yes
Manes.08G08800	1.4937071	2.16065	0.691323	-0.53256808	1	1	down	no
Manes.08G08830	150.9278	166.9198	0.9041932	-0.14529697	0.5435	0.7031	down	no
Manes.08G08870	43.21844	55.43235	0.779661	-0.35908121	0.3421	0.5077	down	no
Manes.08G08930	11.347345	54.11822	0.209677	-2.25375968	######	8E-06	down	yes
Manes.08G08960	0	2.467087	#NAME?	#NAME?	0.0656	0.1397	down	no
Manes.08G09190	77.897375	64.42961	1.2090307	0.27385087	0.3484	0.5148	up	no
Manes.08G09380	129.30403	248.5478	0.520238	-0.94275637	######	0.0002	down	no
Manes.08G09510	43.89457	181.0377	0.242461	-2.04417559	######	2E-07	down	yes
Manes.08G09650	1.3141831	4.545029	0.2891473	-1.79012326	0.4676	0.6304	down	no
Manes.08G09710	8.7812689	2.122838	4.1365695	2.048434822	0.016	0.043	up	yes
Manes.08G09830	7.464056	6.057827	1.2321343	0.301159473	0.6804	0.8195	up	no
Manes.08G09850	106.93451	67.59702	1.5819411	0.661695914	0.0446	0.1021	up	no
Manes.08G10270	0.3734268	2.147101	0.1739213	-2.52349306	0.2392	0.3883	down	no
Manes.08G10390	13.191371	2.14285	6.1559934	2.621991676	0.0821	0.1677	up	no
Manes.08G10420	55.431338	72.35047	0.7661504	-0.38430049	0.3428	0.5086	down	no
Manes.08G10590	246.37211	116.7656	2.1099714	1.077223461	######	6E-06	up	yes
Manes.08G10610	15.68661	10.69486	1.4667427	0.552615779	0.3088	0.4716	up	no
Manes.08G10740	17.925655	15.29187	1.1722341	0.229260763	0.6405	0.7843	up	no
Manes.08G10860	19.451265	16.12485	1.2062914	0.270578418	0.6028	0.753	up	no
Manes.08G11030	56.922196	76.97284	0.7395101	-0.43535819	0.174	0.3051	down	no
Manes.08G11090	132.85452	228.3144	0.5818929	-0.78117448	0.0008	0.0032	down	no
Manes.08G11290	2.9179104	0.603578	4.8343585	2.273324451	0.1178	0.2242	up	no

Manes.08G11320	122.09401	19.05923	6.4060296	2.679430467	######	7E-09	up	yes
Manes.08G11390	7.1066378	18.15851	0.3913667	-1.35340713	0.0303	0.074	down	no
Manes.08G11670	15.038132	16.64855	0.9032697	-0.14677125	0.8471	0.9481	down	no
Manes.08G11690	1660.2707	1377.356	1.2054044	0.269517253	0.056	0.1226	up	no
Manes.08G11990	63.284649	99.69764	0.6347658	-0.65570378	0.0377	0.0887	down	no
Manes.08G12150	0.7569827	1.83783	0.4118894	-1.27967102	0.57	0.7241	down	no
Manes.08G12340	0.7569827	1.516427	0.4991884	-1.00234366	0.7318	0.8615	down	no
Manes.08G12590	7.1992494	4.51572	1.5942639	0.672890445	0.5187	0.6813	up	no
Manes.08G12660	32.803417	20.09906	1.6320867	0.706717738	0.11	0.2124	up	no
Manes.08G12850	9.6148546	10.65342	0.9025133	-0.14797983	0.921	0.9966	down	no
Manes.08G12930	13.687797	26.83972	0.5099828	-0.97147938	0.0543	0.1195	down	no
Manes.08G13220	26.41598	16.71125	1.5807305	0.660591468	0.1999	0.3389	up	no
Manes.08G14380	55.193955	41.57199	1.3276719	0.408898621	0.2296	0.3761	up	no
Manes.08G14420	2.9874142	0.295723	10.102067	3.336578594	0.0464	0.1053	up	no
Manes.08G14440	88.694964	72.20112	1.2284431	0.296831034	0.3067	0.4692	up	no
Manes.08G14460	35.249229	21.3048	1.6545203	0.726413016	0.0703	0.1479	up	no
Manes.08G14490	9773.2212	2204.887	4.4325277	2.148129636	######	5E-10	up	yes
Manes.08G14760	17.199175	4.232128	4.0639539	2.022884029	0.001	0.0037	up	yes
Manes.08G14770	33.574713	12.50985	2.6838631	1.4243111	0.0011	0.0042	up	yes
Manes.08G14840	152.3098	23.67767	6.4326342	2.68540964	######	4E-19	up	yes
Manes.08G14860	1161.7525	910.1668	1.2764172	0.352099988	0.4362	0.6021	up	no
Manes.08G14950	4.3913592	24.71467	0.1776823	-2.4926282	0.0062	0.019	down	yes
Manes.08G14970	9.171924	15.84914	0.5787018	-0.78910792	0.2147	0.3582	down	no
Manes.08G15100	2.4894735	3.918606	0.6352957	-0.65449973	0.6492	0.7921	down	no
Manes.08G15220	183.96226	180.9632	1.016573	0.023713748	0.9283	0.9966	up	no
Manes.08G15350	50.061671	90.78659	0.5514214	-0.85877275	0.0072	0.0218	down	no
Manes.08G15360	44.33587	98.48153	0.4501948	-1.15137884	0.0004	0.0016	down	yes
Manes.08G15440	0	1.532809	#NAME?	#NAME?	0.1863	0.3215	down	no
Manes.08G15610	18.888185	21.30622	0.8865104	-0.17379059	0.7656	0.8881	down	no
Manes.08G15690	225.02251	293.22	0.7674187	-0.38191414	0.0623	0.1339	down	no
Manes.08G15750	366.69817	277.4438	1.3217027	0.402397665	0.027	0.0672	up	no
Manes.08G15910	45.456381	1.813567	25.064629	4.64758095	######	3E-14	up	yes
Manes.08G16000	2.5343545	2.145684	1.1811404	0.240180501	0.9024	0.9883	up	no
Manes.08G16120	36.316129	21.09258	1.7217488	0.783874678	0.092	0.1845	up	no
Manes.08G16200	53.720211	48.22693	1.1139049	0.155626097	0.6233	0.7692	up	no
Manes.08G16850	9.9434003	29.29672	0.3394032	-1.55892776	0.0032	0.0108	down	yes
Manes.08G16860	29.920078	22.90898	1.3060413	0.385200475	0.3437	0.5095	up	no
Manes.08G16970	36.016226	19.38852	1.8576061	0.893444641	0.0271	0.0673	up	no
Manes.08G17160	131.32845	4.265689	30.787163	4.944257016	######	1E-35	up	yes
Manes.08G17360	10.23283	0.618543	16.543436	4.048187012	0.0309	0.0751	up	no
Manes.09G00330	15.601212	21.81133	0.7152802	-0.48341953	0.3448	0.5107	down	no
Manes.09G00570	18.933066	7.340605	2.5792242	1.366937189	0.0116	0.0328	up	yes
Manes.09G00750	73.766507	58.56399	1.2595881	0.332952065	0.2609	0.4148	up	no
Manes.09G00940	6.3727629	1.834996	3.4729036	1.79614235	0.249	0.4008	up	no
Manes.09G00950	30.052976	0.295723	101.62541	6.667117296	######	2E-13	up	yes
Manes.09G00970	91.194106	14.35304	6.3536448	2.667584443	0.0011	0.0043	up	yes
Manes.09G01140	23.139136	30.39769	0.7612135	-0.39362691	0.3879	0.5565	down	no
Manes.09G01190	0	1.490747	#NAME?	#NAME?	0.1864	0.3217	down	no

Manes.09G01400	34.915034	48.65672	0.7175789	-0.47879064	0.2295	0.3761	down	no
Manes.09G01490	45.839707	37.37909	1.2263464	0.29436655	0.4108	0.5763	up	no
Manes.09G01500	5.9645843	14.00422	0.4259133	-1.23136825	0.0999	0.1971	down	no
Manes.09G01670	2.6545039	7.904954	0.3358026	-1.57431481	0.0774	0.1601	down	no
Manes.09G01730	1.4937071	0	Inf	Inf	0.1074	0.2085	up	no
Manes.09G01850	29.458174	13.9371	2.1136515	1.079737546	0.0152	0.0411	up	yes
Manes.09G02000	44.076599	14.25567	3.0918645	1.628477089	######	0.0003	up	yes
Manes.09G02020	37.583801	36.3528	1.0338626	0.048044432	0.8971	0.9847	up	no
Manes.09G02160	40.112046	16.10988	2.4899032	1.316089635	0.0022	0.0076	up	yes
Manes.09G02340	919.45193	654.5383	1.4047336	0.490296603	0.0011	0.0041	up	no
Manes.09G02360	3.4852399	2.759976	1.2627789	0.336602099	0.8977	0.9851	up	no
Manes.09G02510	1.74262	1.543524	1.1289881	0.175030227	0.9694	1	up	no
Manes.09G02670	296.33521	309.4535	0.9576082	-0.06249257	0.7876	0.9038	down	no
Manes.09G02710	2.9034167	3.959251	0.7333247	-0.44747595	0.8095	0.9202	down	no
Manes.09G02910	22.670183	18.62187	1.2173959	0.283798368	0.5217	0.684	up	no
Manes.09G02960	151.05198	89.0866	1.6955634	0.761764762	0.1069	0.2081	up	no
Manes.09G02970	7.4540418	3.331411	2.2375031	1.161889668	0.1526	0.2749	up	no
Manes.09G02980	3.7240237	2.767062	1.3458405	0.428507452	0.6781	0.8174	up	no
Manes.09G03050	895.70828	663.4014	1.3501754	0.433146857	0.0039	0.0128	up	no
Manes.09G03390	1.1304094	2.739965	0.4125635	-1.27731185	0.4245	0.59	down	no
Manes.09G03420	1.1202803	0	Inf	Inf	0.1992	0.3379	up	no
Manes.09G03460	8.127027	1.232835	6.5921441	2.720747786	0.0308	0.075	up	no
Manes.09G03480	100.40269	58.90762	1.7044093	0.769271849	0.0102	0.0293	up	no
Manes.09G03840	72.365592	246.1906	0.2939414	-1.76639964	######	3E-10	down	yes
Manes.09G03900	17.08479	42.80907	0.3990927	-1.32520403	0.0024	0.0084	down	yes
Manes.09G04090	17.796892	22.50346	0.7908515	-0.33852125	0.5121	0.6758	down	no
Manes.09G04220	20.100962	10.05631	1.9988414	0.999163994	0.0437	0.1003	up	no
Manes.09G04290	4.0830717	0	Inf	Inf	0.0028	0.0095	up	yes
Manes.09G04940	61.225243	44.8043	1.3665036	0.450489232	0.2946	0.455	up	no
Manes.09G04950	306.64316	182.8216	1.6772808	0.746124257	0.0003	0.0011	up	no
Manes.09G05130	7.5828053	6.722062	1.1280476	0.1738279	0.7672	0.8892	up	no
Manes.09G05640	3.7545259	0.295723	12.696088	3.666312091	0.0204	0.053	up	no
Manes.09G05660	86.729683	23.42197	3.7029197	1.888663247	######	4E-07	up	yes
Manes.09G05750	0	1.182892	#NAME?	#NAME?	0.2611	0.4149	down	no
Manes.09G05810	7.6783319	14.947	0.5137038	-0.96099128	0.1283	0.2394	down	no
Manes.09G05840	2.9381686	1.232835	2.3832616	1.252937296	0.4391	0.6044	up	no
Manes.09G06340	1.4792134	2.130719	0.6942321	-0.52650993	0.8334	0.9386	down	no
Manes.09G06470	217.91242	298.5142	0.7299902	-0.45405097	0.0454	0.1036	down	no
Manes.09G06590	47.25663	75.76064	0.6237623	-0.68093177	0.0474	0.1072	down	no
Manes.09G06650	1.0956575	1.220704	0.8975622	-0.15591611	1	1	down	no
Manes.09G06710	46.19439	55.38507	0.8340585	-0.26177946	0.4908	0.654	down	no
Manes.09G06850	22.282377	4.24426	5.2500031	2.392318264	######	0.0002	up	yes
Manes.09G06970	18.947559	21.21563	0.8930943	-0.16311558	0.7646	0.8876	down	no
Manes.09G06990	6.5131705	11.52279	0.5652425	-0.82305822	0.2415	0.3911	down	no
Manes.09G07020	1.8671339	0	Inf	Inf	0.0604	0.1306	up	no
Manes.09G07070	7.9358589	2.122838	3.7383244	1.902391763	0.0205	0.0532	up	no
Manes.09G07240	12.583411	15.22634	0.8264237	-0.27504638	0.7002	0.8362	down	no
Manes.09G07330	0.3734268	5.828429	0.0640699	-3.96420988	0.0101	0.0292	down	yes

Manes.09G07350	234.6629	89.21818	2.630214	1.395180164	######	3E-08	up	yes
Manes.09G07490	1907.8598	383.7434	4.971707	2.31374128	######	4E-56	up	yes
Manes.09G07830	479.74366	306.8188	1.5636057	0.644876727	0.0186	0.0491	up	no
Manes.09G07860	163.95536	141.4423	1.1591678	0.213089395	0.3566	0.5237	up	no
Manes.09G07880	20.23002	4.602057	4.3958645	2.136146921	0.0006	0.0024	up	yes
Manes.09G08120	96.630773	101.4487	0.9525089	-0.07019555	0.8207	0.9295	down	no
Manes.09G08180	39.62173	32.75055	1.2098034	0.274772606	0.4679	0.6307	up	no
Manes.09G08340	25.030778	34.8934	0.7173499	-0.47925118	0.2749	0.4312	down	no
Manes.09G08410	4.0135679	5.435032	0.7384626	-0.43740332	0.7024	0.8382	down	no
Manes.09G08480	46.344517	66.49746	0.6969367	-0.52090055	0.1826	0.3164	down	no
Manes.09G08530	8.5539489	25.30833	0.3379895	-1.56494973	0.005	0.0159	down	yes
Manes.09G08700	464.34268	747.1425	0.6214915	-0.68619353	0.0003	0.0012	down	no
Manes.09G09240	18.359857	15.96935	1.1496934	0.201249139	0.8167	0.9261	up	no
Manes.09G09580	184.32735	54.07174	3.4089407	1.769323511	######	2E-11	up	yes
Manes.09G09650	0	2.13497	#NAME?	#NAME?	0.0818	0.1674	down	no
Manes.09G09660	44.037712	15.5512	2.8317884	1.501713484	0.0002	0.0008	up	yes
Manes.09G09740	307.42286	14.03982	21.896494	4.452628016	######	2E-60	up	yes
Manes.09G09840	5.5462765	1.234252	4.493633	2.167882303	0.0275	0.0681	up	no
Manes.09G09860	1505.9581	1323.867	1.1375452	0.185923933	0.2313	0.3783	up	no
Manes.09G10110	6.6072971	0	Inf	Inf	0.0001	0.0005	up	yes
Manes.09G10200	390.23768	708.7728	0.5505822	-0.86097008	######	6E-06	down	no
Manes.09G10230	25.205937	29.75126	0.8472225	-0.23918714	0.6149	0.7613	down	no
Manes.09G10420	1.4937071	6.394195	0.2336036	-2.09786558	0.1912	0.3283	down	no
Manes.09G10510	4.5461456	6.979973	0.6513127	-0.61857763	0.5809	0.7336	down	no
Manes.09G10700	68.873187	119.9626	0.574122	-0.80057084	0.0059	0.0183	down	no
Manes.09G10730	75.800186	93.5045	0.8106581	-0.30283444	0.3123	0.4756	down	no
Manes.09G10890	8.0212564	3.674243	2.1831048	1.126381362	0.1576	0.2822	up	no
Manes.09G10920	542.73542	256.662	2.1145917	1.080379149	######	3E-09	up	yes
Manes.09G11000	70.621457	139.3024	0.506965	-0.98004181	0.0039	0.0129	down	no
Manes.09G11010	65.240211	108.9868	0.5986063	-0.74032072	0.0128	0.0357	down	no
Manes.09G11050	0.7671118	2.738548	0.2801163	-1.83590221	0.3928	0.5601	down	no
Manes.09G11160	58.731635	41.7815	1.405685	0.491273379	0.3143	0.4777	up	no
Manes.09G11170	42.617939	28.19024	1.5117976	0.59626498	0.307	0.4695	up	no
Manes.09G11210	14.789219	21.55705	0.6860504	-0.5436136	0.3018	0.4632	down	no
Manes.09G11290	1.1202803	0	Inf	Inf	0.1992	0.3379	up	no
Manes.09G11350	7.3744089	1.235669	5.967947	2.577234721	0.0481	0.1083	up	no
Manes.09G11400	110.10296	437.4434	0.2516965	-1.9902432	######	1E-12	down	yes
Manes.09G11480	246.16306	147.1896	1.6724219	0.741938811	0.0048	0.0153	up	no
Manes.09G11790	10.34145	21.87482	0.4727559	-1.08083268	0.0513	0.114	down	no
Manes.09G11890	7.126896	2.147101	3.3193104	1.730883554	0.0492	0.1104	up	no
Manes.09G12630	140.28789	147.8132	0.9490891	-0.07538461	0.7809	0.8986	down	no
Manes.09G12640	97.513899	133.6101	0.7298391	-0.45434968	0.0849	0.1726	down	no
Manes.09G12850	242.69984	145.5443	1.6675325	0.737714846	0.0005	0.0021	up	no
Manes.09G13050	14.803828	26.3792	0.5611931	-0.83343074	0.1672	0.2959	down	no
Manes.09G13310	142.12622	230.403	0.6168591	-0.69698706	0.0023	0.0082	down	no
Manes.09G13640	64.128545	63.79862	1.0051714	0.007441508	0.9745	1	up	no
Manes.09G13810	9357.7538	6869.017	1.3623134	0.446058585	0.0529	0.117	up	no
Manes.09G13830	23.896119	23.63136	1.0112038	0.016073752	0.9961	1	up	no

Manes.09G13920	64.439747	211.2491	0.3050415	-1.71292278	######	3E-08	down	yes
Manes.09G14070	5774.7446	3429.779	1.6837074	0.751641447	######	0.0001	up	no
Manes.09G14100	106.10563	119.4559	0.8882409	-0.17097707	0.5794	0.7326	down	no
Manes.09G14130	539.40406	210.1993	2.566155	1.359608325	######	8E-10	up	yes
Manes.09G14890	3.9933097	3.046402	1.3108282	0.390478612	0.7217	0.854	up	no
Manes.09G15200	1.4488261	0	Inf	Inf	0.1199	0.227	up	no
Manes.09G15440	1.8222528	0.295723	6.1620248	2.623404488	0.199	0.3379	up	no
Manes.09G15950	3.2464562	1.827115	1.7768205	0.82929795	0.4789	0.6415	up	no
Manes.09G16140	2.1507986	2.712868	0.7928137	-0.33494616	0.9485	1	down	no
Manes.09G16630	0.7121017	5.130012	0.1388109	-2.8488071	0.0327	0.0788	down	no
Manes.09G16950	2.0812948	3.936405	0.5287298	-0.91939745	0.505	0.6685	down	no
Manes.09G17040	26.685151	28.00765	0.9527809	-0.06978363	0.9317	0.9966	down	no
Manes.09G17050	26.546258	63.79893	0.4160925	-1.2650238	0.0008	0.0032	down	yes
Manes.09G17060	14.632918	5.117881	2.8591754	1.515599107	0.049	0.1102	up	no
Manes.09G17110	2556.2212	191.7815	13.328819	3.736477037	######	1E-140	up	yes
Manes.09G17520	6.1049919	1.840664	3.3167335	1.729763117	0.0901	0.1813	up	no
Manes.09G17580	91.349188	11.25416	8.1169232	3.020932966	######	9E-16	up	yes
Manes.09G17780	171.37855	197.0442	0.8697468	-0.20133262	0.3946	0.5616	down	no
Manes.09G17790	16.203293	58.84001	0.2753789	-1.86051033	######	7E-05	down	yes
Manes.09G18110	141.17474	96.5687	1.4619099	0.547854399	0.0252	0.0633	up	no
Manes.09G18140	3.6603994	0.603578	6.0645052	2.600389945	0.053	0.1171	up	no
Manes.09G18380	818.00573	833.7656	0.9810979	-0.02753097	0.8738	0.9673	down	no
Manes.09G18480	12.637021	0.924981	13.66193	3.772089401	######	7E-05	up	yes
Manes.09G18500	46.226112	43.74318	1.0567617	0.07965003	0.8165	0.9261	up	no
Manes.09G18510	46.916736	51.30861	0.9144028	-0.12909833	0.7862	0.9028	down	no
Manes.10G00280	242.48794	262.9671	0.9221228	-0.11696919	0.58	0.733	down	no
Manes.10G00420	70.158268	106.7288	0.6573507	-0.60526491	0.0436	0.1002	down	no
Manes.10G00460	21.177991	12.14133	1.7442887	0.802638835	0.1081	0.2094	up	no
Manes.10G00510	3.2464562	21.28054	0.1525552	-2.71259711	######	0.0004	down	yes
Manes.10G01150	2.619752	3.369222	0.7775539	-0.36298533	0.895	0.9831	down	no
Manes.10G01470	3.3303387	4.876352	0.682957	-0.55013326	0.8597	0.9572	down	no
Manes.10G01500	11.645504	0	Inf	Inf	######	1E-06	up	yes
Manes.10G01540	1.4937071	0	Inf	Inf	0.1074	0.2085	up	no
Manes.10G01900	34.211497	65.01743	0.5261896	-0.92634523	0.0175	0.0466	down	no
Manes.10G02130	8.0777814	0.617126	13.089353	3.710321869	0.0004	0.0018	up	yes
Manes.10G02280	1.4690843	2.417144	0.6077768	-0.71838651	0.7229	0.8548	down	no
Manes.10G02380	2.4445925	7.662008	0.3190538	-1.64812852	0.088	0.1778	down	no
Manes.10G02530	33.38506	27.71113	1.2047528	0.2687371	0.4861	0.6491	up	no
Manes.10G02560	1.8671339	0	Inf	Inf	0.0604	0.1306	up	no
Manes.10G02870	38.471292	18.62612	2.0654488	1.046455304	0.0341	0.0815	up	no
Manes.10G02880	4.023697	3.669196	1.0966153	0.133057536	1	1	up	no
Manes.10G02890	289.35851	50.48729	5.731314	2.51886595	######	9E-11	up	yes
Manes.10G03310	296.23295	237.9138	1.2451272	0.316293105	0.096	0.1908	up	no
Manes.10G03730	32.137646	174.8655	0.183785	-2.44390919	######	6E-08	down	yes
Manes.10G03740	31.960971	73.12322	0.4370837	-1.19401842	0.0823	0.168	down	no
Manes.10G03790	29.611675	67.69815	0.4374075	-1.19295029	0.0013	0.005	down	yes
Manes.10G03890	3.9585578	9.414917	0.4204559	-1.24997353	0.1227	0.2311	down	no
Manes.10G04070	1.877263	0.295723	6.3480438	2.666312091	0.1964	0.3351	up	no

Manes.10G04100	28.718535	22.10422	1.2992333	0.377660488	0.3572	0.5244	up	no
Manes.10G04230	33.10431	10.07774	3.2848958	1.715847588	0.0002	0.0007	up	yes
Manes.10G04240	34.485147	4.855718	7.101967	2.828218663	######	3E-05	up	yes
Manes.10G04260	91.747402	114.1626	0.8036558	-0.31535029	0.2654	0.42	down	no
Manes.10G04590	201.05537	120.2226	1.6723593	0.741884826	0.0009	0.0034	up	no
Manes.10G04600	10.17072	6.415624	1.5853049	0.66476032	0.305	0.4672	up	no
Manes.10G04700	147.22043	122.6253	1.2005718	0.263721668	0.3128	0.4761	up	no
Manes.10G04710	117.37718	83.42189	1.4070309	0.492654014	0.0822	0.168	up	no
Manes.10G04720	32.803351	13.285	2.4692027	1.30404528	0.0401	0.0933	up	no
Manes.10G04770	10.00266	2.430693	4.1151474	2.040944118	0.0082	0.0242	up	yes
Manes.10G04950	47.77053	2.442824	19.555449	4.289498788	######	1E-14	up	yes
Manes.10G04960	128.85609	50.03938	2.5750936	1.364624875	######	3E-06	up	yes
Manes.10G04980	89.728118	7.856428	11.420981	3.513614698	######	2E-16	up	yes
Manes.10G04990	99.106163	96.71853	1.0246864	0.035182417	0.9259	0.9966	up	no
Manes.10G05110	78.018859	115.0433	0.6781698	-0.56028165	0.0547	0.1204	down	no
Manes.10G05180	12.318604	9.375067	1.3139751	0.393937898	0.7247	0.8563	up	no
Manes.10G05320	13.577662	21.90838	0.6197474	-0.69024775	0.2661	0.4209	down	no
Manes.10G05330	18.597125	9.434929	1.9710933	0.978996047	0.0566	0.1239	up	no
Manes.10G05360	26.122071	8.457172	3.0887479	1.62702214	0.0026	0.0088	up	yes
Manes.10G05810	13.269489	35.5641	0.3731147	-1.42230885	0.0029	0.01	down	yes
Manes.10G05840	21.871349	1.220704	17.917001	4.163257266	######	8E-08	up	yes
Manes.10G06060	22.471801	11.5428	1.9468237	0.96112225	0.0456	0.1039	up	no
Manes.10G06250	33.929281	54.62364	0.6211465	-0.68699456	0.0692	0.1458	down	no
Manes.10G06290	21.584655	33.06266	0.6528409	-0.61519674	0.1669	0.2954	down	no
Manes.10G06480	130.3761	112.4021	1.1599083	0.214010697	0.3781	0.5473	up	no
Manes.10G06500	2.8629003	0	Inf	Inf	0.0131	0.0362	up	yes
Manes.10G06690	3.585131	2.124256	1.6877117	0.755068509	0.5662	0.721	up	no
Manes.10G06720	12.289617	9.063583	1.3559336	0.439286485	0.4731	0.6356	up	no
Manes.10G07140	52.48589	48.97954	1.0715881	0.09975048	0.7397	0.8678	up	no
Manes.10G07460	5.8705727	9.718521	0.6040603	-0.7272355	0.3726	0.5411	down	no
Manes.10G07520	430.02222	260.6963	1.6495143	0.722041327	######	0.0004	up	no
Manes.10G07860	400.36317	355.6739	1.1256467	0.170754068	0.3986	0.5638	up	no
Manes.10G07940	3.058318	2.13497	1.4324876	0.518522657	0.629	0.7745	up	no
Manes.10G08080	4.5317668	3.047819	1.4868884	0.572296349	0.585	0.7373	up	no
Manes.10G08100	96.222824	126.9792	0.7577844	-0.40014061	0.131	0.2433	down	no
Manes.10G08230	9.7046166	28.30195	0.3428956	-1.54415851	0.0035	0.0116	down	yes
Manes.10G08350	78.110201	108.1622	0.7221577	-0.46961411	0.0994	0.1963	down	no
Manes.10G08380	27.281468	37.6277	0.7250368	-0.46387382	0.2724	0.4284	down	no
Manes.10G08490	64.116966	46.7948	1.370173	0.454358011	0.169	0.2985	up	no
Manes.10G08640	50.572246	78.0163	0.6482266	-0.62542979	0.1005	0.1979	down	no
Manes.10G08680	75.167602	33.16334	2.2665873	1.1805217	0.0002	0.0011	up	yes
Manes.10G09110	107.45904	71.13874	1.5105558	0.595079504	0.0399	0.0929	up	no
Manes.10G09380	12.328618	3.358508	3.6708619	1.87611883	0.0052	0.0164	up	yes
Manes.10G09430	180.07953	14.93912	12.054225	3.59146698	######	5E-30	up	yes
Manes.10G09840	2.619752	4.241426	0.6176583	-0.69511908	0.6014	0.7527	down	no
Manes.10G09930	1.7527491	3.030019	0.5784613	-0.78970754	0.6572	0.7991	down	no
Manes.10G09970	1.4937071	0	Inf	Inf	0.1074	0.2085	up	no
Manes.10G10020	1.1202803	0	Inf	Inf	0.1992	0.3379	up	no

Manes.10G10100	0	1.528558	#NAME?	#NAME?	0.1736	0.3047	down	no
Manes.10G10130	39.015399	152.4503	0.2559221	-1.96622336	######	3E-05	down	yes
Manes.10G10200	428.06295	325.9842	1.3131401	0.393020888	0.025	0.0629	up	no
Manes.10G10760	58.407223	139.8771	0.4175611	-1.25994079	######	0.0004	down	yes
Manes.10G10780	5.5029105	0.295723	18.608324	4.217876191	0.0022	0.0077	up	yes
Manes.10G10830	1.0956575	0.307855	3.5590108	1.831476291	0.521	0.6832	up	no
Manes.10G10870	1.787501	1.814984	0.9848577	-0.02201279	1	1	down	no
Manes.10G10900	6.8764682	8.535629	0.8056194	-0.31182962	0.7465	0.8735	down	no
Manes.10G11070	100.68224	120.8913	0.8328331	-0.26390065	0.3892	0.5575	down	no
Manes.10G11100	10.133053	5.736424	1.7664409	0.820845474	0.2414	0.3911	up	no
Manes.10G11620	273.52587	109.8969	2.4889324	1.315527073	######	6E-09	up	yes
Manes.10G11650	21.725177	3.020722	7.1920477	2.846402585	######	4E-05	up	yes
Manes.10G12030	29.22102	27.33412	1.0690311	0.096303878	0.8455	0.947	up	no
Manes.10G12080	56.916661	28.94317	1.9664972	0.975628104	0.0277	0.0685	up	no
Manes.10G12090	29.464284	13.3863	2.2010775	1.138209919	0.0133	0.0367	up	yes
Manes.10G12270	12.214348	0.912849	13.380467	3.74205657	######	0.0001	up	yes
Manes.10G12650	1.1202803	0	Inf	Inf	0.1992	0.3379	up	no
Manes.10G12910	19.016948	9.707806	1.9589336	0.970068499	0.061	0.1317	up	no
Manes.10G13250	13.066858	4.245677	3.0776853	1.621845715	0.0128	0.0356	up	yes
Manes.10G13370	1.8627693	2.430693	0.7663532	-0.38391867	0.9872	1	down	no
Manes.10G13620	418.00568	289.3579	1.4445975	0.53066761	0.0024	0.0085	up	no
Manes.10G13690	1.4039451	0.307855	4.5604173	2.189165845	0.3268	0.4912	up	no
Manes.10G13930	108.70152	45.30025	2.3995788	1.262781217	######	0.0005	up	yes
Manes.10G13970	8.6335818	12.70789	0.6793873	-0.55769391	0.4627	0.6259	down	no
Manes.10G13980	22.182486	39.18335	0.5661201	-0.82081984	0.0851	0.1729	down	no
Manes.10G14250	1.3590641	11.57636	0.1173999	-3.0904966	0.0021	0.0074	down	yes
Manes.10G14360	571.2873	143.594	3.9784911	1.992221388	######	4E-21	up	yes
Manes.10G14450	29.862218	13.35212	2.2365155	1.161252754	0.01	0.0288	up	yes
Manes.10G14480	5.7953043	0.604995	9.5791	3.259890115	0.006	0.0185	up	yes
Manes.10G14490	243.67938	38.88259	6.267057	2.647788123	######	2E-24	up	yes
Manes.10G14770	1.877263	1.219287	1.5396404	0.622593458	0.8189	0.9279	up	no
Manes.10G14840	148.552	0	Inf	Inf	######	8E-55	up	yes
Manes.10G14850	5635.1546	13.30926	423.40104	8.725881	######	4E-83	up	yes
Manes.10G14860	2630.1333	24.22785	108.55826	6.762325652	######	6E-67	up	yes
Manes.10G15090	2.5893646	17.04983	0.1518704	-2.71908717	0.0003	0.0013	down	yes
Manes.10G15250	21.380508	55.45852	0.3855225	-1.37511302	0.0022	0.0077	down	yes
Manes.11G00080	26.038189	35.004	0.7438632	-0.42689085	0.3243	0.4887	down	no
Manes.11G00110	12.987225	19.50478	0.6658482	-0.58673488	0.2969	0.4575	down	no
Manes.11G00170	7.3786585	1.220704	6.0445942	2.595645492	0.0051	0.016	up	yes
Manes.11G00350	66.244772	73.23368	0.904567	-0.14470068	0.7114	0.8452	down	no
Manes.11G00540	62.476906	9.449895	6.6113864	2.724952844	######	9E-11	up	yes
Manes.11G00640	9.6249837	40.59768	0.2370821	-2.07654142	######	0.0002	down	yes
Manes.11G00650	13.975826	0.923564	15.132501	3.919578544	0.0399	0.0929	up	no
Manes.11G00680	436.48996	20.02486	21.797405	4.446084511	######	6E-57	up	yes
Manes.11G00760	21.994233	9.744201	2.2571613	1.174509541	0.0186	0.0491	up	yes
Manes.11G01070	1.8526402	1.851378	1.0006816	0.000982979	1	1	up	no
Manes.11G01160	2.9280395	3.882211	0.7542195	-0.40694361	0.7431	0.8708	down	no
Manes.11G01180	1.0753993	0.309272	3.4772004	1.797926217	0.5227	0.6847	up	no

Manes.11G01240	5.6983282	2.444242	2.3313278	1.221151858	0.1694	0.2992	up	no
Manes.11G01330	4.7502924	7.054179	0.6734012	-0.57046189	0.5731	0.7268	down	no
Manes.11G01530	45.376583	43.86514	1.0344565	0.048873046	1	1	up	no
Manes.11G01750	29.319397	48.30397	0.606977	-0.72028624	0.0644	0.1376	down	no
Manes.11G01800	0.3835559	1.787887	0.2145303	-2.22074677	0.3403	0.5057	down	no
Manes.11G02140	335.18729	492.3502	0.6807905	-0.55471728	0.0024	0.0084	down	no
Manes.11G02170	10.069314	13.74126	0.7327794	-0.44854922	0.5955	0.7476	down	no
Manes.11G02300	167.03724	307.9379	0.5424382	-0.88246941	0.0004	0.0017	down	no
Manes.11G02380	250.42618	289.5255	0.8649537	-0.20930523	0.3287	0.4931	down	no
Manes.11G02440	347.08284	665.3498	0.5216547	-0.93883299	######	1E-05	down	no
Manes.11G02780	22.855472	53.20679	0.4295593	-1.21907086	0.0024	0.0083	down	yes
Manes.11G02910	14.654691	0.307855	47.602651	5.572970013	######	6E-07	up	yes
Manes.11G03060	13.676038	3.085631	4.4321697	2.148013111	0.0337	0.0808	up	no
Manes.11G03300	19.928947	27.36626	0.7282306	-0.45753271	0.3137	0.4771	down	no
Manes.11G03600	13.463392	25.9839	0.5181436	-0.94857603	0.0693	0.1461	down	no
Manes.11G04700	7.6131926	10.83367	0.7027344	-0.50894862	0.5314	0.692	down	no
Manes.11G05390	10.486222	15.83134	0.6623712	-0.59428807	0.3287	0.4931	down	no
Manes.11G05400	8.0618877	5.490644	1.4682956	0.554142433	0.453	0.6169	up	no
Manes.11G05590	18.184812	35.03331	0.519072	-0.94599338	0.0406	0.0942	down	no
Manes.11G05760	112.99356	2.13497	52.92513	5.725881008	######	2E-35	up	yes
Manes.11G05770	10.133053	14.35981	0.7056539	-0.50296734	0.4387	0.6042	down	no
Manes.11G05910	44.070834	75.35732	0.5848248	-0.77392354	0.0243	0.0613	down	no
Manes.11G06170	1.0956575	0	Inf	Inf	0.2227	0.3677	up	no
Manes.11G06870	96.331444	182.3782	0.5281959	-0.92085498	0.0006	0.0025	down	no
Manes.11G06930	21.590649	0.295723	73.009689	6.190016035	######	2E-09	up	yes
Manes.11G07130	4.2523517	16.15416	0.2632358	-1.92557261	0.006	0.0185	down	yes
Manes.11G07140	74.829042	78.02104	0.9590879	-0.06026498	0.8716	0.9655	down	no
Manes.11G07210	37.760591	8.524914	4.4294394	2.147124116	######	2E-05	up	yes
Manes.11G07280	307.3985	250.62	1.2265521	0.2946085	0.3002	0.4614	up	no
Manes.11G07290	10.078043	68.04904	0.1480997	-2.75535914	######	8E-08	down	yes
Manes.11G07370	5.8950805	1.502878	3.9225271	1.971783402	0.0477	0.1078	up	no
Manes.11G07540	1.1202803	0	Inf	Inf	0.1992	0.3379	up	no
Manes.11G07760	46.515837	134.6653	0.3454182	-1.53358417	######	0.0001	down	yes
Manes.11G07900	3.5301209	6.094221	0.5792571	-0.78772426	0.4516	0.6155	down	no
Manes.11G07960	15.058505	98.69831	0.1525711	-2.71244682	######	1E-10	down	yes
Manes.11G08000	55.042018	58.0874	0.9475725	-0.07769178	0.8592	0.9568	down	no
Manes.11G08020	9.9796672	20.6078	0.4842664	-1.04612706	0.0543	0.1196	down	no
Manes.11G08170	320.3945	209.3037	1.5307637	0.61425159	0.0014	0.0053	up	no
Manes.11G08180	8.7986122	5.739258	1.5330575	0.616411778	0.5556	0.714	up	no
Manes.11G08700	559.0037	478.5113	1.1682142	0.224304882	0.1555	0.2791	up	no
Manes.11G08820	40.218047	35.85257	1.121762	0.165766632	0.647	0.7901	up	no
Manes.11G09100	2721.9276	1054.115	2.582193	1.368596863	######	1E-06	up	yes
Manes.11G09220	1433.7238	1562.38	0.9176537	-0.12397826	0.396	0.5616	down	no
Manes.11G09250	1.0406474	4.26994	0.2437148	-2.0367344	0.1336	0.2471	down	no
Manes.11G09380	22.14762	16.08925	1.3765478	0.461054729	0.3163	0.48	up	no
Manes.11G09460	39.579813	142.3694	0.2780079	-1.84680198	######	4E-08	down	yes
Manes.11G09880	84.02129	125.9554	0.6670718	-0.58408597	0.034	0.0813	down	no
Manes.11G09910	40.876653	10.31989	3.9609596	1.985849989	0.0017	0.0063	up	yes

Manes.11G10250	8.4150563	4.835706	1.7401919	0.799246379	0.2819	0.4394	up	no
Manes.11G10790	8.4353145	5.755018	1.465732	0.551621331	0.5196	0.6819	up	no
Manes.11G10920	26.398637	55.35673	0.4768822	-1.06829526	0.0111	0.0315	down	yes
Manes.11G10990	15.728461	30.56391	0.514609	-0.95845146	0.1116	0.2148	down	no
Manes.11G11030	85.703645	3.364971	25.469357	4.670690605	######	4E-06	up	yes
Manes.11G11210	2.2506897	10.66555	0.2110242	-2.24451959	0.0472	0.1068	down	no
Manes.11G11460	9310.2258	3109.844	2.9937916	1.581973787	######	7E-09	up	yes
Manes.11G11470	6248.2946	2256.592	2.768908	1.469317101	######	1E-05	up	yes
Manes.11G11490	53.970868	29.05568	1.8574982	0.893360849	0.0182	0.0482	up	no
Manes.11G11670	2.8629003	0.309272	9.2569131	3.210531183	0.0512	0.1139	up	no
Manes.11G11850	4.5953911	28.75429	0.1598159	-2.64551742	######	9E-05	down	yes
Manes.11G11870	2.5647419	9.748452	0.2630922	-1.92635952	0.0249	0.0627	down	no
Manes.11G12310	1.1202803	4.538566	0.2468357	-2.01837674	0.2608	0.4147	down	no
Manes.11G12530	18.193426	34.25706	0.5310854	-0.91298409	0.0454	0.1035	down	no
Manes.11G12770	6.1991184	20.58717	0.3011156	-1.73161045	0.0121	0.034	down	yes
Manes.11G12890	1.0406474	0	Inf	Inf	0.225	0.3703	up	no
Manes.11G12940	565.87908	307.3651	1.8410646	0.880540211	######	2E-06	up	no
Manes.11G12960	7.6189572	2.145684	3.5508286	1.828155711	0.029	0.0712	up	no
Manes.11G13200	14.968743	39.35665	0.3803358	-1.39465438	0.0022	0.0079	down	yes
Manes.11G13260	5.4522649	0.615709	8.8552621	3.146535006	0.1182	0.2248	up	no
Manes.11G13270	2543.9714	1916.042	1.3277219	0.40895298	0.0037	0.0123	up	no
Manes.11G13310	6.1542374	0.914266	6.7313405	2.750893836	0.0086	0.0252	up	yes
Manes.11G13390	1.8873921	0.307855	6.1307922	2.61607351	0.198	0.3372	up	no
Manes.11G13450	4.6257785	52.67016	0.0878254	-3.50921785	######	2E-09	down	yes
Manes.11G13760	1.4792134	0.307855	4.8049106	2.264509578	0.3213	0.4857	up	no
Manes.11G13880	3889.5861	56.8837	68.377872	6.095457612	######	2E-131	up	yes
Manes.11G13890	335.98385	18.42856	18.231695	4.188376822	######	2E-15	up	yes
Manes.11G13900	1422.1952	16.08199	88.434031	6.466529744	######	1E-22	up	yes
Manes.11G13910	155.55148	9.066417	17.156884	4.100715687	######	1E-15	up	yes
Manes.11G13920	413.31304	39.60638	10.435517	3.383430214	######	9E-10	up	yes
Manes.11G14190	187.73133	277.9394	0.6754397	-0.56610113	0.008	0.0237	down	no
Manes.11G14510	589.88303	495.7615	1.1898524	0.25078263	0.1732	0.3041	up	no
Manes.11G14520	339.38798	250.7241	1.3536313	0.436834867	0.062	0.1335	up	no
Manes.11G14610	6.7173173	0	Inf	Inf	0.0001	0.0005	up	yes
Manes.11G14680	5.4073839	33.71476	0.1603862	-2.64037773	######	2E-05	down	yes
Manes.11G14760	25.547642	56.33936	0.4534599	-1.140953	0.0103	0.0296	down	yes
Manes.11G14960	32.75824	104.1466	0.3145397	-1.66868578	######	8E-06	down	yes
Manes.11G15020	45.727942	9.739155	4.695268	2.231207514	######	2E-05	up	yes
Manes.11G15280	8.170393	8.152151	1.0022377	0.003224679	1	1	up	no
Manes.11G15310	0.3835559	1.543524	0.2484937	-2.00871903	0.4462	0.6105	down	no
Manes.11G15590	132.78769	149.3952	0.8888353	-0.17001204	0.5069	0.6703	down	no
Manes.11G15600	5.1092255	6.765541	0.7551835	-0.40510078	0.7103	0.8444	down	no
Manes.11G15640	68.336196	425.0722	0.1607637	-2.63698618	######	1E-18	down	yes
Manes.11G15780	518.46461	367.4393	1.4110213	0.496739734	0.0027	0.0092	up	no
Manes.11G15920	2.6545039	2.133553	1.2441707	0.31518438	0.8923	0.9813	up	no
Manes.11G16560	0.3734268	1.531392	0.2438479	-2.0359467	0.4445	0.6086	down	no
Manes.12G00170	1.0305183	0	Inf	Inf	0.2255	0.3707	up	no
Manes.12G00200	99.597994	78.23185	1.2731132	0.348360674	0.2203	0.3651	up	no

Manes.12G00540	123.7301	167.5329	0.7385421	-0.43724803	0.0833	0.1701	down	no
Manes.12G00590	296.09829	348.2113	0.8503408	-0.233887	0.2889	0.4481	down	no
Manes.12G00650	258.22234	61.72462	4.1834577	2.064695838	######	4E-18	up	yes
Manes.12G00940	1.4140742	2.750679	0.5140819	-0.95992996	0.5958	0.748	down	no
Manes.12G00950	8.2659196	9.656446	0.8560002	-0.22431704	0.814	0.9239	down	no
Manes.12G00990	257.99863	270.4083	0.9541076	-0.06777615	0.7345	0.8634	down	no
Manes.12G01110	307.87944	341.7004	0.9010216	-0.15036636	0.4181	0.5831	down	no
Manes.12G01140	311.96934	300.0365	1.0397712	0.056266105	0.7516	0.8777	up	no
Manes.12G01540	19.210966	8.843483	2.1723303	1.119243454	0.0329	0.0793	up	no
Manes.12G01560	140.80278	72.63454	1.9385101	0.954948248	0.0002	0.0008	up	no
Manes.12G01590	26.776248	23.98128	1.1165481	0.159045384	0.6785	0.8178	up	no
Manes.12G01770	8.6943565	47.07555	0.1846894	-2.43682695	######	8E-06	down	yes
Manes.12G01990	595.18783	477.2833	1.2470325	0.31849902	0.0493	0.1107	up	no
Manes.12G02180	6.0746045	1.83783	3.305314	1.724787321	0.0576	0.1255	up	no
Manes.12G02210	830.95233	98.20091	8.461758	3.080957425	######	5E-46	up	yes
Manes.12G02230	2.2405606	0.307855	7.2779852	2.863539113	0.1112	0.2142	up	no
Manes.12G02250	124.6988	252.8173	0.4932368	-1.01964777	0.0001	0.0006	down	yes
Manes.12G02400	140.13871	55.04967	2.5456776	1.34804971	######	3E-06	up	yes
Manes.12G02660	149.19752	211.1817	0.7064887	-0.50126154	0.104	0.2036	down	no
Manes.12G02930	85.717679	57.73748	1.4846107	0.570084677	0.0522	0.1158	up	no
Manes.12G03580	87.809168	106.3087	0.8259833	-0.27581556	0.3199	0.4842	down	no
Manes.12G03920	259.25478	57.69604	4.493459	2.167826429	######	1E-17	up	yes
Manes.12G04250	59.589793	43.41407	1.3725919	0.456902696	0.1753	0.3067	up	no
Manes.12G04390	575.87524	493.0566	1.1679698	0.224002959	0.2225	0.3676	up	no
Manes.12G04410	0	2.430693	#NAME?	#NAME?	0.0569	0.1244	down	no
Manes.12G04530	1.877263	0	Inf	Inf	0.065	0.1386	up	no
Manes.12G04610	3.2768435	10.68698	0.3066201	-1.7054757	0.0341	0.0816	down	no
Manes.12G04760	691.80337	497.8571	1.3895621	0.474630301	0.0024	0.0083	up	no
Manes.12G04990	11.642769	3.959251	2.9406493	1.556134721	0.028	0.0692	up	no
Manes.12G05280	1.4937071	0	Inf	Inf	0.1074	0.2085	up	no
Manes.12G05380	1800.1613	1569.582	1.1469053	0.197746235	0.3892	0.5575	up	no
Manes.12G05400	364.61213	1406.96	0.259149	-1.94814646	######	2E-13	down	yes
Manes.12G05660	77.965478	72.4557	1.0760433	0.105736186	0.6999	0.8359	up	no
Manes.12G05860	236.33556	273.1365	0.8652654	-0.20878539	0.31	0.473	down	no
Manes.12G06200	5.5766639	5.466381	1.0201748	0.028816387	0.9657	1	up	no
Manes.12G06270	156.112	166.2711	0.9389005	-0.09095576	0.7173	0.8504	down	no
Manes.12G06360	202.25859	163.4665	1.2373091	0.307205964	0.153	0.2755	up	no
Manes.12G06590	16.052987	7.292079	2.201428	1.138439631	0.0568	0.1242	up	no
Manes.12G07030	221.60362	192.1108	1.1535198	0.206042792	0.3104	0.4736	up	no
Manes.12G07130	9.2906733	7.25285	1.2809685	0.357235048	0.596	0.7482	up	no
Manes.12G07170	2.1160467	1.529975	1.3830594	0.467863137	0.7687	0.8901	up	no
Manes.12G07200	90.773064	217.3334	0.4176674	-1.25957362	######	7E-06	down	yes
Manes.12G07310	10.968154	18.49284	0.5931026	-0.75364638	0.2451	0.3959	down	no
Manes.12G07340	10.357344	18.83772	0.5498195	-0.86296996	0.1349	0.249	down	no
Manes.12G07380	1019.3451	992.2315	1.0273259	0.03889391	0.8486	0.9493	up	no
Manes.12G07700	9.5569949	8.564143	1.1159313	0.158248213	0.7904	0.906	up	no
Manes.12G07710	54.764348	98.14721	0.5579817	-0.8417102	0.0072	0.0217	down	no
Manes.12G07750	2.3895824	50.06396	0.0477306	-4.38894204	######	1E-06	down	yes

Manes.12G07770	36.336092	17.98269	2.020615	1.014794482	0.0119	0.0336	up	yes
Manes.12G07790	28.737458	18.47	1.5558994	0.637748742	0.1407	0.2578	up	no
Manes.12G07940	54.23154	53.1162	1.020998	0.02998009	0.9399	1	up	no
Manes.12G08220	102.56414	26.70265	3.8409731	1.941471866	######	3E-05	up	yes
Manes.12G08230	21.551533	10.71124	2.0120476	1.008664409	0.0535	0.118	up	no
Manes.12G08240	1.1202803	0.309272	3.6223188	1.856913532	0.4558	0.6194	up	no
Manes.12G08270	58.488831	11.58628	5.0481105	2.335743494	######	2E-07	up	yes
Manes.12G08730	554.48841	124.118	4.4674293	2.159444888	######	9E-20	up	yes
Manes.12G08770	26.80792	3.022139	8.8705115	3.149017303	######	1E-06	up	yes
Manes.12G09010	16.746115	9.744201	1.7185725	0.781210684	0.1487	0.2695	up	no
Manes.12G09200	150.24661	182.8856	0.8215335	-0.28360878	0.2682	0.4235	down	no
Manes.12G09250	22.745451	10.58001	2.1498516	1.10423707	0.0257	0.0644	up	no
Manes.12G09490	13.395518	4.538566	2.951487	1.56144199	0.018	0.0476	up	yes
Manes.12G09760	21.270487	43.79976	0.4856303	-1.04206972	0.0152	0.0411	down	yes
Manes.12G09800	24.514209	20.1112	1.2189334	0.285619333	0.537	0.6972	up	no
Manes.12G09840	175.74797	175.0405	1.0040419	0.005819477	0.9412	1	up	no
Manes.12G10070	8.3108006	32.92261	0.2524345	-1.98601914	0.0006	0.0024	down	yes
Manes.12G10180	9.3659417	14.30482	0.6547404	-0.61100514	0.3412	0.5067	down	no
Manes.12G10210	56.82102	270.4997	0.2100594	-2.25113058	######	2E-15	down	yes
Manes.12G10240	401.54685	549.8716	0.7302556	-0.4535265	0.0098	0.0284	down	no
Manes.12G10750	15.256658	9.419964	1.619609	0.695645553	0.1966	0.3354	up	no
Manes.12G11030	73.055871	137.5147	0.5312585	-0.91251419	0.0023	0.008	down	no
Manes.12G11370	29.701667	84.27753	0.3524269	-1.50460412	0.0039	0.0127	down	yes
Manes.12G11550	181.87793	138.6196	1.3120648	0.391838962	0.0832	0.1697	up	no
Manes.12G11700	99.499372	130.8773	0.7602495	-0.39545509	0.1492	0.2702	down	no
Manes.12G11710	310.65646	318.4714	0.975461	-0.03584389	0.9147	0.9966	down	no
Manes.12G12050	3.4852399	8.820637	0.3951234	-1.33962491	0.1226	0.231	down	no
Manes.12G12060	51.668198	50.23868	1.0284546	0.040478162	0.9075	0.9921	up	no
Manes.12G12190	267.36625	273.5138	0.9775237	-0.03279642	0.9001	0.9868	down	no
Manes.12G12250	2.2361961	4.262855	0.5245772	-0.93077312	0.4642	0.6272	down	no
Manes.12G12580	101.37636	68.5015	1.4799144	0.565513738	0.0406	0.0942	up	no
Manes.12G13130	0.3285458	1.528558	0.2149383	-2.21800524	0.4409	0.6064	down	no
Manes.12G13150	3.54025	7.289245	0.4856813	-1.04191811	0.2589	0.4126	down	no
Manes.12G13240	14.606961	54.19999	0.2695011	-1.89163668	0.022	0.0565	down	no
Manes.12G13360	3.6893867	0.295723	12.475817	3.641062345	0.021	0.0544	up	no
Manes.12G13800	10.895801	8.511366	1.2801472	0.356309687	0.5777	0.731	up	no
Manes.12G13940	163.8568	156.5583	1.0466181	0.065735117	0.7476	0.8744	up	no
Manes.12G13970	681.4019	220.7553	3.0866846	1.626058054	######	3E-19	up	yes
Manes.12G14200	11.736781	25.55694	0.4592404	-1.12267851	0.0283	0.0697	down	no
Manes.12G14230	6.2295058	6.418458	0.9705611	-0.04310911	1	1	down	no
Manes.12G14300	2433.6109	1134.958	2.1442307	1.100460109	######	3E-14	up	yes
Manes.12G14490	6.8663391	7.008487	0.9797177	-0.02956201	1	1	down	no
Manes.12G14510	71.918297	54.97308	1.3082458	0.387633587	0.2867	0.4454	up	no
Manes.12G14520	60.705709	52.57736	1.1545978	0.207390432	0.5349	0.6952	up	no
Manes.12G14550	782.48448	413.0994	1.8941795	0.921573064	0.0098	0.0284	up	no
Manes.12G14650	70.8621	4.280654	16.554035	4.049111004	######	5E-18	up	yes
Manes.12G14940	3.5098627	0	Inf	Inf	0.0048	0.0153	up	yes
Manes.12G15100	5.856079	3.652814	1.6031693	0.680926815	0.4322	0.598	up	no

Manes.12G15210	173.5829	105.5921	1.6439007	0.717123174	0.0137	0.0378	up	no
Manes.12G15460	1.1202803	1.237086	0.9055797	-0.14308647	1	1	down	no
Manes.13G00060	27.87057	30.66615	0.9088383	-0.13790444	0.7912	0.9065	down	no
Manes.13G00560	414.92856	385.1034	1.0774472	0.10761717	0.5455	0.7051	up	no
Manes.13G00810	21.172111	57.95222	0.3653374	-1.45269867	0.0004	0.0015	down	yes
Manes.13G00970	4.5707683	3.370639	1.3560539	0.439414477	0.6172	0.7633	up	no
Manes.13G00980	4.3667365	8.515617	0.5127916	-0.96355557	0.2423	0.3921	down	no
Manes.13G01010	311.13381	357.4763	0.8703621	-0.20031239	0.3971	0.562	down	no
Manes.13G01060	452.55265	474.6912	0.9533622	-0.06890362	0.689	0.8265	down	no
Manes.13G01090	75.116957	114.9628	0.6534025	-0.61395611	0.0345	0.0823	down	no
Manes.13G01230	1237.7574	1224.955	1.0104513	0.014999817	0.9212	0.9966	up	no
Manes.13G01450	4.4014884	0.309272	14.23179	3.83104522	0.009	0.0262	up	yes
Manes.13G01510	0.3734268	1.235669	0.3022061	-1.72639542	0.5999	0.7515	down	no
Manes.13G01700	230.99564	465.2432	0.4965051	-1.01011953	######	3E-06	down	yes
Manes.13G01790	1.1202803	0.295723	3.7882751	1.921541095	0.4588	0.6218	up	no
Manes.13G02060	3.3608409	6.374979	0.5271925	-0.92359824	0.5825	0.7352	down	no
Manes.13G02160	165.38556	226.9138	0.7288476	-0.45631083	0.0402	0.0935	down	no
Manes.13G02320	480.05379	143.275	3.3505772	1.744409661	######	6E-19	up	yes
Manes.13G02360	1.0753993	3.949954	0.2722562	-1.87696337	0.1689	0.2984	down	no
Manes.13G02500	20.654027	14.3127	1.4430562	0.529127484	0.2749	0.4313	up	no
Manes.13G02550	38.391544	13.69982	2.8023387	1.486631344	0.0004	0.0017	up	yes
Manes.13G02560	379.85897	192.0325	1.9780975	0.984113535	######	9E-07	up	no
Manes.13G02870	2.0610366	22.56899	0.0913216	-3.45289957	######	0.0002	down	yes
Manes.13G03070	257.82388	156.4767	1.6476818	0.720437683	0.0015	0.0054	up	no
Manes.13G03380	19.293563	1.207155	15.982671	3.998436582	######	2E-06	up	yes
Manes.13G03710	145.61885	233.2652	0.6242632	-0.67977368	0.0205	0.0532	down	no
Manes.13G03840	600.87521	89.6749	6.7005956	2.74428935	######	1E-34	up	yes
Manes.13G04070	50.908301	38.85407	1.3102437	0.389835125	0.2563	0.4096	up	no
Manes.13G04170	74.536583	5.787784	12.87826	3.686865772	######	1E-17	up	yes
Manes.13G04310	74.174866	62.51867	1.1864434	0.246643249	0.4622	0.6254	up	no
Manes.13G04330	105.47939	21.49355	4.9074894	2.294985149	######	1E-09	up	yes
Manes.13G04340	100.35002	213.9225	0.4690953	-1.09204698	######	0.0004	down	yes
Manes.13G04580	6.6318049	0.923564	7.1806698	2.844118425	0.1453	0.2644	up	no
Manes.13G04620	20.250098	24.13757	0.8389453	-0.25335139	0.6906	0.8278	down	no
Manes.13G04770	44.667331	32.6097	1.3697558	0.45391868	0.2149	0.3584	up	no
Manes.13G04830	55.636705	67.77377	0.8209179	-0.28469011	0.4276	0.5931	down	no
Manes.13G04860	74.044358	108.6971	0.6811989	-0.55385189	0.0582	0.1267	down	no
Manes.13G04870	56.823639	50.30487	1.1295853	0.17579322	0.5848	0.7372	up	no
Manes.13G04970	1093.2093	972.7397	1.1238457	0.168443958	0.223	0.3682	up	no
Manes.13G05710	5.507275	2.727833	2.0189193	1.013583253	0.2691	0.4245	up	no
Manes.13G05780	122.75447	124.758	0.9839406	-0.02335693	0.9326	0.9966	down	no
Manes.13G05930	635.29874	70.18604	9.0516391	3.178179058	######	4E-60	up	yes
Manes.13G06100	1521.2826	1453.059	1.0469517	0.066194827	0.6815	0.8203	up	no
Manes.13G06110	12.75554	4.230711	3.0149871	1.592151818	0.0162	0.0435	up	yes
Manes.13G06710	241.45806	288.2432	0.8376886	-0.25551405	0.1996	0.3385	down	no
Manes.13G06780	159.84453	80.21446	1.9927147	0.994735201	######	0.0003	up	no
Manes.13G06790	37.713965	21.27487	1.7727	0.825948414	0.0349	0.0832	up	no
Manes.13G06940	5.6114158	3.962085	1.4162784	0.502104844	0.5305	0.6912	up	no

Manes.13G07030	1.6876098	15.51702	0.1087586	-3.20079811	0.0024	0.0083	down	yes
Manes.13G07080	314.81044	331.2201	0.950457	-0.07330679	0.719	0.8516	down	no
Manes.13G07160	0.3835559	1.81215	0.2116579	-2.24019352	0.3247	0.489	down	no
Manes.13G07190	1.1202803	0.912849	1.2272348	0.295411346	0.9186	0.9966	up	no
Manes.13G07450	12.414131	9.478409	1.3097273	0.389266414	0.5131	0.6768	up	no
Manes.13G07590	16.825748	20.61993	0.8159943	-0.29336906	0.5714	0.7253	down	no
Manes.13G07740	1.4792134	0.615709	2.4024553	1.264509578	0.7668	0.8892	up	no
Manes.13G07880	123.99775	210.6273	0.588707	-0.76437841	0.0014	0.0051	down	no
Manes.13G08080	64.031568	36.59543	1.7497148	0.80711982	0.0896	0.1805	up	no
Manes.13G08160	3.8137857	0	Inf	Inf	0.003	0.0101	up	yes
Manes.13G08310	142.19689	161.0215	0.8830928	-0.17936305	0.4674	0.6302	down	no
Manes.13G08320	24.55321	27.18317	0.9032503	-0.14680228	0.8019	0.9151	down	no
Manes.13G08470	1.8526402	2.726416	0.6795148	-0.55742302	0.7793	0.8973	down	no
Manes.13G08620	6.3727629	2.121421	3.0040061	1.586887762	0.0695	0.1464	up	no
Manes.13G08840	1353.8047	477.9123	2.8327471	1.502201786	######	3E-10	up	yes
Manes.13G08980	15.601212	22.19339	0.7029666	-0.50847186	0.3245	0.4889	down	no
Manes.13G09070	30.95944	0	Inf	Inf	######	3E-14	up	yes
Manes.13G09090	9.2660506	16.69991	0.5548563	-0.84981397	0.1621	0.2885	down	no
Manes.13G09400	21.889978	23.84388	0.9180542	-0.12334877	0.8744	0.9678	down	no
Manes.13G09920	16.462335	4.584258	3.5910581	1.844409014	0.0541	0.1193	up	no
Manes.13G10090	218.86122	326.8268	0.6696551	-0.57850994	0.0046	0.0149	down	no
Manes.13G10490	18.43221	127.4069	0.144672	-2.7891425	######	9E-13	down	yes
Manes.13G10560	241.25044	327.6587	0.7362859	-0.4416621	0.0274	0.0678	down	no
Manes.13G10730	48.235513	3.064202	15.741624	3.976512503	0.0011	0.0043	up	yes
Manes.13G10950	2.5546127	2.14285	1.1921565	0.253573622	1	1	up	no
Manes.13G10960	76.403782	38.69463	1.974532	0.981510751	0.0018	0.0066	up	no
Manes.13G11070	389.74068	261.1377	1.4924716	0.577703509	0.0015	0.0056	up	no
Manes.13G11170	1.4937071	3.328577	0.4487525	-1.1560081	0.6263	0.7719	down	no
Manes.13G11270	165.42003	163.7435	1.010239	0.014696667	0.9471	1	up	no
Manes.13G11480	5.2279748	0.924981	5.651983	2.498757124	0.0242	0.0611	up	no
Manes.13G11510	349.85402	234.6072	1.4912333	0.576506025	0.0281	0.0694	up	no
Manes.13G11800	29.038581	1.237086	23.473366	4.552952839	0.0073	0.022	up	yes
Manes.13G11830	10.366073	5.834892	1.7765662	0.829091438	0.2964	0.4568	up	no
Manes.13G12010	1.8671339	0.914266	2.0422211	1.030139089	0.481	0.6437	up	no
Manes.13G12210	1.0753993	0.309272	3.4772004	1.797926217	0.5227	0.6847	up	no
Manes.13G12520	274.50358	184.0443	1.4915082	0.576771888	0.1487	0.2695	up	no
Manes.13G12630	1.1506677	1.51501	0.7595118	-0.3968558	1	1	down	no
Manes.13G12720	2.9975433	0	Inf	Inf	0.0127	0.0354	up	yes
Manes.13G12750	33.357587	32.32611	1.0319084	0.045314894	0.909	0.9933	up	no
Manes.13G12800	7608.4167	5357.153	1.4202351	0.506129721	0.0017	0.0061	up	no
Manes.13G12850	68.741344	9.742784	7.0556164	2.818772123	######	7E-12	up	yes
Manes.13G13180	0.7468535	1.542107	0.4843073	-1.04600537	0.8389	0.9424	down	no
Manes.13G13230	7.5090519	9.089263	0.8261453	-0.2755326	0.8855	0.9762	down	no
Manes.13G13310	3.3564764	13.04851	0.2572305	-1.95886616	0.0088	0.0258	down	yes
Manes.13G13840	1.0406474	13.62925	0.076354	-3.71115251	0.0001	0.0005	down	yes
Manes.13G13890	456.56342	223.9622	2.0385736	1.027560066	######	6E-08	up	yes
Manes.13G14270	150.45087	82.98577	1.8129719	0.858356588	0.0005	0.0021	up	no
Manes.13G14300	2.7282573	0.911432	2.9933741	1.581772599	0.2232	0.3683	up	no

Manes.13G14550	1.5240944	0.617126	2.4696645	1.304315051	0.5505	0.7097	up	no
Manes.13G14690	2.4648507	6.399242	0.3851786	-1.37640064	0.1767	0.3085	down	no
Manes.13G14700	196.48949	431.3753	0.4554955	-1.13449138	######	2E-07	down	yes
Manes.13G14940	262.51293	334.8962	0.7838635	-0.35132566	0.0687	0.145	down	no
Manes.13G15040	57.927566	42.42763	1.3653264	0.44924585	0.2815	0.4389	up	no
Manes.13G15110	530.22667	407.5927	1.3008739	0.379481069	0.0506	0.1129	up	no
Manes.13G15210	31.315524	35.91606	0.8719087	-0.19775099	0.7378	0.8662	down	no
Manes.13G15220	32.709455	26.71336	1.2244605	0.292146249	0.4794	0.6419	up	no
Manes.13G15230	439.03868	37.11029	11.830647	3.564457012	######	1E-16	up	yes
Manes.13G15450	1.4937071	0	Inf	Inf	0.1074	0.2085	up	no
Manes.14G00120	19.78266	52.7124	0.3752942	-1.41390591	0.0007	0.0028	down	yes
Manes.14G00130	4.9297015	15.80707	0.3118668	-1.68099812	0.0329	0.0792	down	no
Manes.14G00450	36.479759	9.131326	3.9950123	1.998199949	######	0.0001	up	yes
Manes.14G00480	3.1364359	2.767062	1.1334897	0.180771293	0.93	0.9966	up	no
Manes.14G00600	4.7806797	1.219287	3.9208826	1.97117845	0.1619	0.2882	up	no
Manes.14G00610	3.3014663	0.309272	10.674974	3.416160679	0.0316	0.0766	up	no
Manes.14G00840	56.408066	82.94118	0.6800972	-0.55618709	0.1159	0.2216	down	no
Manes.14G01050	8.4049272	3.987765	2.1076785	1.075654797	0.1527	0.275	up	no
Manes.14G01140	129.25745	238.6627	0.5415905	-0.88472553	0.0097	0.0281	down	no
Manes.14G01150	6.7765771	8.185711	0.8278544	-0.27255106	0.8025	0.9157	down	no
Manes.14G01270	155.19227	86.03376	1.8038531	0.851081879	0.0011	0.0041	up	no
Manes.14G01400	8.2920573	5.142143	1.6125683	0.689360231	0.3741	0.5427	up	no
Manes.14G01510	13.777559	6.967842	1.9773065	0.983536545	0.1001	0.1974	up	no
Manes.14G01660	1.5139653	4.5414	0.3333697	-1.58480497	0.1776	0.3095	down	no
Manes.14G01720	30.468779	5.440701	5.6001572	2.485467326	######	2E-05	up	yes
Manes.14G01940	1.8222528	0.914266	1.9931315	0.995036888	0.5551	0.714	up	no
Manes.14G02200	5.5130396	1.83783	2.9997552	1.584844793	0.0973	0.1928	up	no
Manes.14G02490	15.081613	23.75046	0.635003	-0.65516478	0.2048	0.345	down	no
Manes.14G02530	10.367473	27.07165	0.3829642	-1.38471842	0.0405	0.0941	down	no
Manes.14G02690	9.4599533	13.30501	0.7110069	-0.49206452	0.4806	0.6432	down	no
Manes.14G02750	4.7749151	0.309272	15.43923	3.948528859	0.0059	0.0184	up	yes
Manes.14G02980	0.3835559	2.741382	0.1399133	-2.83739449	0.13	0.2419	down	no
Manes.14G03010	17.752011	6.426339	2.7623833	1.46591354	0.0088	0.0258	up	yes
Manes.14G03030	4.4521339	6.121318	0.7273162	-0.45934537	0.6595	0.8011	down	no
Manes.14G03710	4.0280616	1.517844	2.653805	1.408062379	0.2014	0.3408	up	no
Manes.14G04460	157.4508	136.7405	1.1514567	0.203460171	0.3842	0.5538	up	no
Manes.14G04790	1.0753993	1.542107	0.6973572	-0.52003018	0.9929	1	down	no
Manes.14G04890	16.770738	12.78777	1.3114671	0.391181617	0.4619	0.6251	up	no
Manes.14G04960	11.377848	42.82749	0.2656669	-1.91230957	0.0006	0.0023	down	yes
Manes.14G04980	1430.5232	12.14275	117.80882	6.880303778	######	1E-84	up	yes
Manes.14G05000	88.077054	113.912	0.7732023	-0.37108217	0.1726	0.3035	down	no
Manes.14G05400	12.752805	0.924981	13.787105	3.785247691	######	7E-05	up	yes
Manes.14G05470	346.86831	407.7921	0.850601	-0.23344561	0.6657	0.8065	down	no
Manes.14G05570	369.1706	274.2271	1.3462223	0.428916619	0.0198	0.0518	up	no
Manes.14G05680	58.271131	64.06614	0.9095464	-0.13678083	0.6892	0.8266	down	no
Manes.14G05880	30.935873	126.0778	0.2453713	-2.02696177	######	6E-06	down	yes
Manes.14G06100	276.09811	256.7637	1.0753006	0.104739982	0.6086	0.756	up	no
Manes.14G06200	13.449013	16.75411	0.8027294	-0.3170143	0.6014	0.7526	down	no

Manes.14G06310	54.185144	15.87686	3.4128385	1.770972151	0.0015	0.0056	up	yes
Manes.14G06730	987.89899	896.7071	1.1016964	0.139726771	0.3379	0.5032	up	no
Manes.14G06950	64.12161	66.03694	0.9709961	-0.04246265	0.9627	1	down	no
Manes.14G07000	62.524932	76.54619	0.8168262	-0.29189898	0.3812	0.5505	down	no
Manes.14G07040	437.46287	148.1084	2.9536667	1.56250704	######	2E-06	up	yes
Manes.14G07110	2841.4334	2785.311	1.0201493	0.028780345	0.8385	0.9419	up	no
Manes.14G07120	6.0195944	1.824281	3.2997074	1.722338098	0.0586	0.1276	up	no
Manes.14G07180	360.0227	296.73	1.2133006	0.278937083	0.1175	0.2238	up	no
Manes.14G07460	2581.0122	1662.683	1.5523174	0.634423606	0.0007	0.0028	up	no
Manes.14G07490	26.659128	9.6514	2.7622031	1.465819409	0.0799	0.1643	up	no
Manes.14G07530	20.468624	36.70555	0.5576438	-0.84258423	0.0601	0.1303	down	no
Manes.14G07650	4.8009379	6.751993	0.7110401	-0.49199709	0.6405	0.7843	down	no
Manes.14G07810	8.8376137	10.03709	0.8804956	-0.18361224	0.8938	0.9824	down	no
Manes.14G07890	11.010185	37.0413	0.2972408	-1.75029594	0.0003	0.0013	down	yes
Manes.14G08330	12.473505	80.5951	0.1547675	-2.69182523	######	2E-07	down	yes
Manes.14G08360	140.33714	86.35862	1.6250507	0.700484714	0.0054	0.017	up	no
Manes.14G08370	6.6926946	0.900718	7.4304018	2.893440227	0.0056	0.0175	up	yes
Manes.14G08470	27.407382	78.35617	0.3497795	-1.51548233	0.0002	0.0009	down	yes
Manes.14G08550	22.955248	32.6461	0.7031544	-0.50808661	0.2758	0.4322	down	no
Manes.14G08720	0.3734268	2.759976	0.1353007	-2.88575861	0.159	0.2842	down	no
Manes.14G08830	8.9825007	0	Inf	Inf	######	5E-05	up	yes
Manes.14G08970	5.2539976	4.537149	1.1579954	0.211629562	0.8852	0.976	up	no
Manes.14G09270	14.744338	0.604995	24.371022	4.607094866	######	2E-06	up	yes
Manes.14G09320	3.9382996	0.603578	6.5249269	2.70596173	0.0367	0.0866	up	no
Manes.14G09560	3518.425	3229.17	1.0895758	0.123766544	0.5681	0.7227	up	no
Manes.14G09570	3014.7092	2547.748	1.183284	0.242796424	0.41	0.5755	up	no
Manes.14G09610	2.3895824	3.339291	0.7155957	-0.4827833	0.8586	0.9563	down	no
Manes.14G09690	4.6504012	9.671412	0.4808399	-1.05637135	0.1768	0.3086	down	no
Manes.14G09750	2.2405606	0	Inf	Inf	0.0349	0.0831	up	no
Manes.14G09760	3824.8756	845.101	4.5259392	2.178217189	######	2E-51	up	yes
Manes.14G10040	894.73614	586.4921	1.5255723	0.609350511	0.0014	0.0052	up	no
Manes.14G10050	46.912536	13.377	3.5069541	1.810218568	######	0.0002	up	yes
Manes.14G10060	168.21993	353.4216	0.4759753	-1.07104147	######	0.0001	down	yes
Manes.14G10080	16.450806	12.43564	1.3228758	0.40367759	0.4353	0.6011	up	no
Manes.14G10120	4.8053025	8.829935	0.5442059	-0.8777755	0.2674	0.4225	down	no
Manes.14G10180	384.12351	152.4129	2.5202826	1.3335855	######	3E-11	up	yes
Manes.14G10200	111.90803	20.41905	5.4805699	2.454325922	######	1E-13	up	yes
Manes.14G10260	18.807152	15.2027	1.2370928	0.306953691	0.538	0.6982	up	no
Manes.14G10680	4.2972327	2.73713	1.5699773	0.650743706	0.5075	0.671	up	no
Manes.14G10740	1.8671339	0.617126	3.0255305	1.597188113	0.3211	0.4855	up	no
Manes.14G10840	24.16977	15.2213	1.5878917	0.667112533	0.1657	0.2936	up	no
Manes.14G10910	56.049428	26.23473	2.1364594	1.095221934	0.002	0.0071	up	yes
Manes.14G11210	15007.521	11918.5	1.2591788	0.332483117	0.0578	0.126	up	no
Manes.14G11230	101.70518	109.3503	0.9300859	-0.1045642	0.7506	0.8768	down	no
Manes.14G11250	212.93598	339.9476	0.6263788	-0.67489268	0.0012	0.0046	down	no
Manes.14G11560	17.262864	60.64127	0.2846719	-1.81262809	0.0281	0.0693	down	no
Manes.14G11570	81.398738	924.0675	0.0880874	-3.50491997	######	1E-48	down	yes
Manes.14G11590	12.979945	49.04414	0.2646584	-1.91779651	0.0002	0.0007	down	yes

Manes.14G11660	18.446704	17.31704	1.0652344	0.091170914	0.8285	0.9356	up	no
Manes.14G11730	1.8671339	5.207052	0.3585779	-1.47964145	0.4096	0.5751	down	no
Manes.14G12080	13.450413	49.32662	0.2726806	-1.87471594	######	0.0002	down	yes
Manes.14G12100	20.376242	2.095741	9.722689	3.281355373	0.003	0.0102	up	yes
Manes.14G12150	103.84557	99.85065	1.040009	0.056595969	0.7918	0.9069	up	no
Manes.14G12190	10.655617	2.106456	5.0585523	2.338724566	0.0234	0.0594	up	no
Manes.14G12800	5945.7254	20507.8	0.289925	-1.78624829	######	6E-11	down	yes
Manes.14G12840	41.396187	24.55776	1.6856665	0.753319126	0.0474	0.1072	up	no
Manes.14G12890	165.95429	0.307855	539.06724	9.074321424	######	4E-54	up	yes
Manes.14G12920	1.3691932	8.197843	0.1670187	-2.58191831	0.0131	0.0362	down	yes
Manes.14G12960	1.787501	1.517844	1.177658	0.235920656	0.9628	1	up	no
Manes.14G13120	56.837143	132.4696	0.4290579	-1.22075563	0.0005	0.0021	down	yes
Manes.14G13590	0.3734268	2.110707	0.1769202	-2.49882908	0.2447	0.3954	down	no
Manes.14G13660	5.2177307	5.799915	0.8996219	-0.1526093	1	1	down	no
Manes.14G13750	12.18984	8.766443	1.3905116	0.475615736	0.4583	0.6217	up	no
Manes.14G13800	2.1160467	0.900718	2.3492896	1.232224538	0.4248	0.5902	up	no
Manes.14G13880	5.4218775	0.295723	18.334307	4.196473861	0.0028	0.0095	up	yes
Manes.14G13900	0.3835559	1.209989	0.3169911	-1.65748559	0.6082	0.7558	down	no
Manes.14G13910	38.526072	29.76197	1.294473	0.372364895	0.328	0.4924	up	no
Manes.14G13990	507.92549	422.44	1.2023613	0.265870534	0.1123	0.2159	up	no
Manes.14G14020	30.261783	30.73123	0.9847241	-0.0222085	0.9807	1	down	no
Manes.14G14040	9.9028839	5.824178	1.7003058	0.765794253	0.2285	0.3749	up	no
Manes.14G14050	613.39771	484.961	1.2648392	0.338953935	0.0462	0.1051	up	no
Manes.14G14240	1.0753993	0.617126	1.7425925	0.801235204	0.8113	0.9216	up	no
Manes.14G14270	672.54445	454.3909	1.480101	0.565695589	0.0004	0.0017	up	no
Manes.14G14530	7.8621055	7.624196	1.0312045	0.04433043	0.9567	1	up	no
Manes.14G14550	5.2684913	9.132121	0.5769187	-0.79356006	0.5201	0.6824	down	no
Manes.14G14790	2.5647419	1.505712	1.7033412	0.768367413	0.6023	0.753	up	no
Manes.14G14820	26.774978	0.591446	45.270359	5.500494837	0.0074	0.0224	up	yes
Manes.14G15170	10.62523	2.738548	3.8798777	1.956011189	0.0109	0.0311	up	yes
Manes.14G15290	18.021182	13.37275	1.3476046	0.43039727	0.3945	0.5616	up	no
Manes.14G15360	1.3590641	0.591446	2.2978662	1.200294802	0.7398	0.8678	up	no
Manes.14G15480	3.8485376	2.426442	1.5860828	0.665468084	0.622	0.768	up	no
Manes.14G15600	1.0305183	4.564246	0.2257806	-2.14700638	0.1056	0.206	down	no
Manes.14G15760	5.1946229	2.735713	1.8988184	0.925101949	0.3186	0.4828	up	no
Manes.14G15920	1.1202803	0.924981	1.2111392	0.276364703	0.9152	0.9966	up	no
Manes.14G15970	65.878264	44.56703	1.4781839	0.563825802	0.073	0.1525	up	no
Manes.14G15990	9.4150723	20.91282	0.4502057	-1.15134372	0.0501	0.112	down	no
Manes.14G16000	11.043422	8.192797	1.3479429	0.430759413	0.4777	0.6404	up	no
Manes.14G16240	46.223148	53.89812	0.8576022	-0.22161957	0.5699	0.7241	down	no
Manes.14G16280	249.9553	310.542	0.8049001	-0.31311835	0.1269	0.2373	down	no
Manes.14G16290	66.608004	76.10409	0.8752223	-0.19227853	0.602	0.753	down	no
Manes.14G16310	0.7468535	7.926383	0.0942238	-3.40776536	0.0378	0.0888	down	no
Manes.14G16410	7.2686382	9.767047	0.7442002	-0.42623728	0.6962	0.8328	down	no
Manes.14G16460	2.0711657	8.823471	0.2347337	-2.09090337	0.0268	0.0666	down	no
Manes.14G16570	49.894136	80.88497	0.616853	-0.69700143	0.3516	0.5184	down	no
Manes.14G16770	0.3285458	5.155692	0.0637249	-3.97199982	0.0125	0.035	down	yes
Manes.14G16790	56.922016	147.8792	0.3849223	-1.3773607	######	2E-05	down	yes

Manes.14G16840	0.7468535	1.220704	0.6118221	-0.70881579	1	1	down	no
Manes.14G16900	0	1.220704	#NAME?	#NAME?	0.2572	0.4105	down	no
Manes.14G17430	70.767809	139.4693	0.5074077	-0.97878275	0.0022	0.0078	down	no
Manes.15G00100	31.561472	25.22057	1.2514177	0.323563384	0.4535	0.6175	up	no
Manes.15G00160	41.564132	36.55257	1.1371055	0.185366117	0.6092	0.7565	up	no
Manes.15G00240	85.213809	42.61465	1.9996364	0.999737711	0.001	0.0038	up	no
Manes.15G00280	76.703111	89.52254	0.8568022	-0.22296584	0.5049	0.6684	down	no
Manes.15G00580	3.6097538	3.304314	1.0924368	0.127549804	0.9956	1	up	no
Manes.15G00640	385.73756	504.5441	0.764527	-0.38736067	0.0775	0.1603	down	no
Manes.15G00690	0.3734268	10.58851	0.0352672	-4.82553076	0.0002	0.0011	down	yes
Manes.15G01050	168.82749	119.8446	1.4087201	0.494384995	0.1707	0.3008	up	no
Manes.15G01060	16.352315	11.95904	1.3673596	0.451392746	0.4514	0.6154	up	no
Manes.15G01270	33.865657	28.39458	1.1926803	0.254207354	0.5229	0.6847	up	no
Manes.15G01430	18.776879	23.72983	0.7912775	-0.33774429	0.6241	0.7699	down	no
Manes.15G01680	1.1202803	0.295723	3.7882751	1.921541095	0.4588	0.6218	up	no
Manes.15G01710	6.5435579	4.831455	1.354366	0.437617613	0.6033	0.753	up	no
Manes.15G01730	3.3014663	2.427859	1.3598263	0.443422356	0.7338	0.863	up	no
Manes.15G02130	2.9381686	4.5414	0.6469742	-0.62821993	0.5994	0.7511	down	no
Manes.15G02150	11.532749	15.28258	0.7546339	-0.40615125	0.6396	0.7839	down	no
Manes.15G02320	19.290599	1.195024	16.14244	4.012786779	0.0004	0.0016	up	yes
Manes.15G02470	40774.753	6701.781	6.0841666	2.605059664	######	1E-40	up	yes
Manes.15G02850	2.5893646	1.517844	1.7059493	0.770574772	0.5943	0.7465	up	no
Manes.15G02860	4.3522428	3.396319	1.2814587	0.357786981	0.854	0.9527	up	no
Manes.15G02910	39.382946	7.260731	5.4241023	2.439384388	######	2E-06	up	yes
Manes.15G02990	0.6570915	8.809923	0.0745854	-3.74496313	0.0152	0.0411	down	yes
Manes.15G03340	27.018176	209.6127	0.1288957	-2.95572368	######	3E-13	down	yes
Manes.15G03510	57.036515	63.59442	0.8968792	-0.15701438	0.6742	0.8144	down	no
Manes.15G03740	74.882882	1.836413	40.776717	5.349673725	######	8E-09	up	yes
Manes.15G03820	1977.7882	762.738	2.5930111	1.374628364	######	5E-09	up	yes
Manes.15G03920	12.557503	6.402076	1.9614736	0.971937943	0.1273	0.2378	up	no
Manes.15G03980	1886.848	2042.414	0.9238322	-0.11429732	0.6972	0.8335	down	no
Manes.15G04430	1301.8209	238.5574	5.4570549	2.448122556	######	9E-59	up	yes
Manes.15G04670	26.884818	26.49122	1.0148577	0.021277397	0.9467	1	up	no
Manes.15G05030	3.5750019	2.442824	1.4634707	0.549393821	0.6114	0.7585	up	no
Manes.15G05100	114.81278	92.83065	1.2367982	0.306610128	0.2379	0.3869	up	no
Manes.15G05110	10.800274	0.603578	17.893763	4.161384898	######	0.0001	up	yes
Manes.15G05230	63.487462	113.9138	0.5573291	-0.8433985	0.0045	0.0145	down	no
Manes.15G05250	93.770837	120.6477	0.7772289	-0.36358864	0.1838	0.3181	down	no
Manes.15G05400	1014.7923	1132.491	0.8960709	-0.15831528	0.2947	0.4551	down	no
Manes.15G05670	8.1805221	10.3399	0.7911608	-0.33795714	0.6957	0.8324	down	no
Manes.15G05750	1.4140742	7.937719	0.1781462	-2.48886672	0.0165	0.0442	down	yes
Manes.15G05880	113.89922	62.77154	1.8145042	0.859575405	0.0015	0.0055	up	no
Manes.15G05910	0	1.505712	#NAME?	#NAME?	0.1727	0.3035	down	no
Manes.15G06090	136.88194	239.1997	0.5722495	-0.80528368	0.0005	0.0022	down	no
Manes.15G06260	18.468477	30.45897	0.6063394	-0.72180247	0.1203	0.2278	down	no
Manes.15G06540	4.4708772	9.051452	0.4939403	-1.01759128	0.2771	0.4337	down	no
Manes.15G06550	14.989002	0.615709	24.344294	4.60551173	0.0106	0.0302	up	yes
Manes.15G07030	4.9558392	0.295723	16.758379	4.066810706	0.0053	0.0168	up	yes

Manes.15G07210	746.24881	66.19903	11.272805	3.49477466	######	6E-23	up	yes
Manes.15G07220	6.3583842	0	Inf	Inf	0.0002	0.0008	up	yes
Manes.15G07580	14.874617	3.369222	4.4148519	2.14236503	0.001	0.0039	up	yes
Manes.15G07670	2.226067	0	Inf	Inf	0.0372	0.0876	up	no
Manes.15G07810	57.188567	103.1375	0.5544887	-0.85077005	0.005	0.0159	down	no
Manes.15G07860	1.7773718	0	Inf	Inf	0.0674	0.1427	up	no
Manes.15G07970	68.182744	127.5314	0.5346351	-0.9033736	0.0016	0.0059	down	no
Manes.15G08000	21.827688	50.74254	0.4301655	-1.2170364	0.0039	0.0128	down	yes
Manes.15G08010	23.871496	74.93037	0.3185824	-1.65026152	######	7E-05	down	yes
Manes.15G08230	7.4437977	7.664842	0.9711613	-0.04221721	1	1	down	no
Manes.15G08260	8.2991565	11.15144	0.7442226	-0.42619397	0.7285	0.859	down	no
Manes.15G08280	1.4937071	0.899301	1.6609652	0.732021853	0.6681	0.8088	up	no
Manes.15G08320	128.8693	292.0906	0.4411963	-1.18050744	######	2E-06	down	yes
Manes.15G08620	117.98223	150.3792	0.784565	-0.35003516	0.3439	0.5096	down	no
Manes.15G09110	550.44571	1489.294	0.3696017	-1.4359568	######	3E-15	down	yes
Manes.15G09130	79.194559	7.577088	10.451847	3.385685968	######	3E-12	up	yes
Manes.15G09140	155.10801	93.24541	1.6634385	0.734168562	0.395	0.5616	up	no
Manes.15G09150	3.2261979	0	Inf	Inf	0.0079	0.0236	up	yes
Manes.15G09380	1.8873921	0.295723	6.3822959	2.674075492	0.1986	0.3379	up	no
Manes.15G09460	4.6055202	10.88786	0.4229957	-1.24128499	0.1023	0.2009	down	no
Manes.15G09550	164.6543	201.6694	0.8164565	-0.29255206	0.2025	0.3424	down	no
Manes.15G09630	1.1405385	0	Inf	Inf	0.2206	0.3653	up	no
Manes.15G09690	374.31612	435.9962	0.8585306	-0.22005854	0.5837	0.7363	down	no
Manes.15G09710	10.585998	11.51571	0.9192662	-0.12144542	0.9517	1	down	no
Manes.15G09730	294.60646	173.7012	1.6960535	0.762181662	0.0001	0.0006	up	no
Manes.15G09750	1.0305183	0	Inf	Inf	0.2255	0.3707	up	no
Manes.15G09850	531.99719	138.2325	3.8485675	1.944321552	######	6E-25	up	yes
Manes.15G10440	300.10148	210.1132	1.4282848	0.514283702	0.012	0.0336	up	no
Manes.15G10450	262.98568	184.0236	1.429087	0.515093712	0.0109	0.031	up	no
Manes.15G10600	6.5420429	3.94712	1.657422	0.728940934	0.3357	0.5011	up	no
Manes.15G10620	65.921925	72.11021	0.9141829	-0.1294452	0.7121	0.8458	down	no
Manes.15G10940	13.773195	14.02627	0.9819569	-0.0262684	1	1	down	no
Manes.15G10950	391.09958	179.4417	2.1795362	1.124021162	######	2E-08	up	yes
Manes.15G10990	26.086034	0	Inf	Inf	######	5E-13	up	yes
Manes.15G11120	69.521615	11.83569	5.8738958	2.554317664	######	2E-10	up	yes
Manes.15G11360	173.60485	237.1519	0.7320407	-0.45000418	0.0423	0.0976	down	no
Manes.15G11970	40.836022	9.791932	4.1703744	2.060176926	0.0018	0.0066	up	yes
Manes.15G12140	3.7443968	13.04851	0.2869596	-1.80108033	0.0888	0.1791	down	no
Manes.15G12420	14.181373	21.86065	0.6487169	-0.624339	0.2277	0.3739	down	no
Manes.15G12440	14.583672	3.289348	4.4336057	2.148480484	0.063	0.1351	up	no
Manes.15G12540	111.82218	186.3848	0.5999533	-0.73707783	0.0032	0.0106	down	no
Manes.15G12620	1.9712746	0.914266	2.1561275	1.10844249	0.4048	0.5702	up	no
Manes.15G12730	59.977419	66.28161	0.9048878	-0.1441892	0.7197	0.8523	down	no
Manes.15G12860	16.611357	85.48676	0.194315	-2.36353112	0.0005	0.0022	down	yes
Manes.15G13200	1.5240944	10.89212	0.1399264	-2.83726002	0.0058	0.0181	down	yes
Manes.15G13370	51.508933	27.03335	1.905385	0.930082548	0.0099	0.0287	up	no
Manes.15G13860	5.2076016	16.12626	0.3229267	-1.63072132	0.0241	0.0609	down	no
Manes.15G14000	44.441411	54.12673	0.8210622	-0.28443653	0.4505	0.6145	down	no

Manes.15G14010	18.358457	8.518451	2.1551402	1.107781758	0.0921	0.1846	up	no
Manes.15G14420	1092.5792	887.1005	1.2316295	0.300568334	0.06	0.1301	up	no
Manes.15G14590	1688.3971	376.027	4.4900955	2.166746132	######	0.0002	up	yes
Manes.15G14890	649.79415	363.6251	1.7869895	0.83753112	0.0151	0.041	up	no
Manes.15G15060	197.58538	168.5659	1.1721552	0.229163636	0.5483	0.7074	up	no
Manes.15G15080	6.6868151	10.69628	0.6251534	-0.67771781	0.3867	0.5561	down	no
Manes.15G15100	382.62443	342.632	1.1167212	0.159269094	0.3854	0.555	up	no
Manes.15G15390	7.947503	4.546446	1.7480693	0.805762395	0.3962	0.5616	up	no
Manes.15G15480	34.690514	31.92625	1.0865827	0.119797956	0.7407	0.8686	up	no
Manes.15G15500	15.67956	6.67637	2.3485157	1.231749248	0.0438	0.1005	up	no
Manes.15G15610	4.6605303	15.5419	0.2998687	-1.73759722	0.0185	0.0489	down	yes
Manes.15G15620	130.63218	117.7519	1.1093851	0.149760276	0.5206	0.6829	up	no
Manes.15G16210	106.34866	75.63145	1.4061435	0.491743801	0.1889	0.3252	up	no
Manes.15G16220	5.8604435	17.03345	0.3440551	-1.53928854	0.0504	0.1125	down	no
Manes.15G16230	33.392339	21.8734	1.5266184	0.610339477	0.1394	0.2558	up	no
Manes.15G16240	7.4539268	11.56848	0.6443306	-0.63412701	0.3871	0.5563	down	no
Manes.15G16250	265.84362	199.4378	1.3329649	0.414638814	0.0336	0.0805	up	no
Manes.15G16280	34.145072	53.35237	0.6399917	-0.64387498	0.0977	0.1935	down	no
Manes.15G16300	4.4911354	12.41217	0.3618332	-1.4666034	0.1313	0.2438	down	no
Manes.15G16640	3.2913372	10.05064	0.3274754	-1.61054139	0.0476	0.1075	down	no
Manes.15G16910	3.1813169	10.89433	0.2920159	-1.77588116	0.0701	0.1474	down	no
Manes.15G16990	1641.3979	1803.736	0.9099991	-0.13606302	0.3472	0.5136	down	no
Manes.15G17110	249.09495	160.2641	1.5542778	0.636244398	0.0019	0.0069	up	no
Manes.15G17130	74.991338	180.9705	0.4143842	-1.27095898	######	0.0004	down	yes
Manes.15G17250	197.54422	172.5849	1.1446205	0.194869388	0.3537	0.5207	up	no
Manes.15G17300	351.75876	425.9478	0.825826	-0.27609033	0.1344	0.2483	down	no
Manes.15G17310	150.59174	81.08037	1.8573144	0.893218047	0.0004	0.0019	up	no
Manes.15G17370	137.884	156.0003	0.8838699	-0.17809404	0.4578	0.6211	down	no
Manes.15G17430	85.19477	179.6608	0.4741978	-1.07643908	######	0.0003	down	yes
Manes.15G17600	37.132371	76.91661	0.4827614	-1.05061769	0.0023	0.008	down	yes
Manes.15G17650	14.27102	1.543524	9.2457403	3.208788843	0.0003	0.0015	up	yes
Manes.15G17790	83.927213	64.49877	1.3012219	0.379867059	0.1894	0.3259	up	no
Manes.15G17830	1.1506677	0.914266	1.2585696	0.331784944	0.9075	0.9921	up	no
Manes.15G17860	1.8671339	0	Inf	Inf	0.0604	0.1306	up	no
Manes.15G17960	45.092853	46.172	0.9766276	-0.03411953	0.9555	1	down	no
Manes.15G18090	5.5825434	15.80203	0.3532802	-1.50111523	0.0203	0.0528	down	no
Manes.15G18460	2.5994937	1.209989	2.1483609	1.103236377	0.4403	0.6058	up	no
Manes.15G18670	8.03575	10.53936	0.7624511	-0.39128328	0.6826	0.8211	down	no
Manes.15G18690	279.8332	274.4832	1.019491	0.027849055	0.8957	0.9836	up	no
Manes.15G18730	467.09353	625.6791	0.7465385	-0.42171139	0.0364	0.0862	down	no
Manes.15G18850	16.366809	24.35404	0.6720367	-0.57338799	0.2692	0.4246	down	no
Manes.15G19070	3.191446	5.167823	0.617561	-0.69534653	0.552	0.711	down	no
Manes.15G19140	0.3835559	1.798601	0.2132523	-2.2293667	0.3328	0.498	down	no
Manes.15G19150	4.2320935	1.854212	2.282421	1.190564907	0.4921	0.6553	up	no
Manes.15G19210	4.4275112	10.95986	0.4039752	-1.30766131	0.1377	0.2532	down	no
Manes.15G19280	161.82139	114.3149	1.4155756	0.50138876	0.0322	0.0778	up	no
Manes.16G00040	59.721407	73.82814	0.8089247	-0.30592274	0.3499	0.5165	down	no
Manes.16G00520	156.00152	80.31372	1.9424018	0.957841634	######	0.0005	up	no

Manes.16G00640	240.6763	168.9222	1.4247762	0.510735349	0.0442	0.1013	up	no
Manes.16G00670	31.146129	31.5998	0.9856431	-0.02086274	1	1	down	no
Manes.16G00810	114.1241	7.035584	16.220984	4.019789425	######	1E-22	up	yes
Manes.16G00910	0.3734268	1.504295	0.2482403	-2.01019054	0.4425	0.6068	down	no
Manes.16G01000	40.711738	65.53954	0.6211783	-0.68692073	0.0627	0.1347	down	no
Manes.16G01230	4.0830717	8.144271	0.5013428	-0.99613065	0.4108	0.5763	down	no
Manes.16G01980	26.440603	4.254974	6.2140452	2.635532741	######	1E-05	up	yes
Manes.16G02240	266.78777	150.2707	1.7753815	0.82812904	0.0005	0.0019	up	no
Manes.16G02250	11.024679	1.182892	9.3201043	3.220346096	0.1012	0.1991	up	no
Manes.16G02350	106.98382	144.3783	0.7409966	-0.43246109	0.1034	0.2026	down	no
Manes.16G02410	1.1304094	6.068541	0.1862737	-2.42450434	0.0333	0.0799	down	no
Manes.16G02760	3.2261979	0	Inf	Inf	0.0079	0.0236	up	yes
Manes.16G02900	7.4785496	33.26606	0.2248102	-2.15322057	######	0.0004	down	yes
Manes.16G02960	1.5139653	0.309272	4.8952615	2.291385923	0.3187	0.4828	up	no
Manes.16G03100	43.885775	28.8989	1.518597	0.602739054	0.3157	0.4793	up	no
Manes.16G03120	154.18817	116.6681	1.321597	0.402282279	0.0872	0.1765	up	no
Manes.16G03360	42.072087	37.96549	1.1081667	0.148174941	0.6806	0.8196	up	no
Manes.16G03470	0.3835559	1.208572	0.3173628	-1.655795	0.608	0.7557	down	no
Manes.16G03580	3.7747842	3.034271	1.24405	0.315044413	0.8285	0.9356	up	no
Manes.16G03590	54.080063	2.433527	22.222913	4.473976058	0.0001	0.0005	up	yes
Manes.16G03630	17.711494	12.72003	1.3924102	0.477584305	0.4572	0.6206	up	no
Manes.16G03880	84.346986	65.83495	1.2811887	0.357482938	0.2269	0.3728	up	no
Manes.16G04280	2.7383864	2.687188	1.0190529	0.027228999	1	1	up	no
Manes.16G04300	5.8950805	25.53693	0.2308453	-2.11500173	0.0018	0.0065	down	yes
Manes.16G04340	5.1642356	17.36052	0.2974701	-1.74918319	0.0071	0.0215	down	yes
Manes.16G04370	173.42638	126.7041	1.3687515	0.452860578	0.047	0.1064	up	no
Manes.16G04390	1.4488261	0	Inf	Inf	0.1199	0.227	up	no
Manes.16G04660	434.78923	264.5443	1.6435405	0.716806988	######	0.0002	up	no
Manes.16G04710	14.130613	48.12309	0.2936347	-1.76790539	######	0.0005	down	yes
Manes.16G04730	12.483519	35.80484	0.3486546	-1.52012971	0.0017	0.0061	down	yes
Manes.16G05120	103.11379	160.6437	0.641879	-0.63962677	0.0124	0.0346	down	no
Manes.16G05390	3.674893	0.591446	6.2134028	2.635383595	0.2353	0.3835	up	no
Manes.16G05640	1.8671339	5.170658	0.3611018	-1.46952242	0.4037	0.5691	down	no
Manes.16G05650	3.3666055	8.79354	0.3828498	-1.38514941	0.0921	0.1846	down	no
Manes.16G05670	302.29522	278.1862	1.0866652	0.119907521	0.5473	0.7066	up	no
Manes.16G05740	202.05228	395.0767	0.5114255	-0.96740412	######	0.0003	down	no
Manes.16G06160	12.469026	12.73278	0.9792854	-0.03019866	1	1	down	no
Manes.16G06290	2.5198608	1.208572	2.0849898	1.06004036	0.4469	0.6112	up	no
Manes.16G06410	1.4589552	1.528558	0.9544649	-0.06723592	1	1	down	no
Manes.16G06470	1.7527491	4.576377	0.3829992	-1.38458654	0.262	0.4158	down	no
Manes.16G06520	1.8526402	2.10929	0.8783241	-0.18717471	1	1	down	no
Manes.16G06680	2.4547216	0.295723	8.3007446	3.05324075	0.0825	0.1685	up	no
Manes.16G06690	11.830792	14.66908	0.8065123	-0.31023151	0.6631	0.8042	down	no
Manes.16G06920	44.256352	88.44064	0.5004074	-0.99882497	0.0022	0.0079	down	no
Manes.16G07070	0	2.121421	#NAME?	#NAME?	0.0816	0.167	down	no
Manes.16G07130	6.3236323	0	Inf	Inf	0.0002	0.0008	up	yes
Manes.16G07500	109.83501	76.03086	1.444611	0.530681021	0.0468	0.106	up	no
Manes.16G07700	340.58639	275.6491	1.2355795	0.305187896	0.0958	0.1905	up	no

Manes.16G07760	885.09964	830.6042	1.0656094	0.09167874	0.5802	0.7331	up	no
Manes.16G07840	0.3734268	4.259226	0.0876748	-3.51169385	0.0297	0.0727	down	no
Manes.16G07880	12.150724	48.15414	0.2523298	-1.98661748	######	9E-05	down	yes
Manes.16G07940	6.7723274	2.427859	2.7894239	1.479967179	0.0949	0.189	up	no
Manes.16G08180	1.0406474	1.505712	0.6911329	-0.53296486	0.9859	1	down	no
Manes.16G08240	15.395665	27.75115	0.5547757	-0.8500235	0.0866	0.1755	down	no
Manes.16G09200	139.53114	155.2954	0.8984883	-0.15442841	0.5448	0.7044	down	no
Manes.16G09260	695.59739	261.2294	2.6627836	1.412935165	0.0001	0.0006	up	yes
Manes.16G09550	92.117864	235.5494	0.3910766	-1.35447705	######	7E-07	down	yes
Manes.16G10180	431.02363	364.6907	1.1818883	0.241093667	0.1634	0.2903	up	no
Manes.16G10220	41.263059	68.08588	0.6060443	-0.72250484	0.0389	0.0911	down	no
Manes.16G10250	4.8255607	4.26994	1.1301238	0.176480828	0.9017	0.9878	up	no
Manes.16G10390	1.4937071	0.615709	2.4259951	1.278576613	0.6755	0.8153	up	no
Manes.16G10450	33.985806	60.83099	0.5586923	-0.83987411	0.0201	0.0525	down	no
Manes.16G10970	79.337357	18.42572	4.3057932	2.106279048	######	8E-05	up	yes
Manes.16G11090	5.068709	3.343542	1.5159698	0.600241023	0.5136	0.6773	up	no
Manes.16G11180	3.1219423	0.295723	10.556979	3.400125187	0.034	0.0814	up	no
Manes.16G11360	1.832382	0.295723	6.1962768	2.631401601	0.2015	0.341	up	no
Manes.16G11680	0.7468535	1.529975	0.4881475	-1.03461109	0.8361	0.9403	down	no
Manes.16G11690	59.141213	7.331308	8.0669394	3.012021411	######	1E-11	up	yes
Manes.16G11710	133.87799	14.06187	9.520638	3.25105825	######	5E-07	up	yes
Manes.16G11750	5.0194635	6.057827	0.8285914	-0.27126717	0.8504	0.9508	down	no
Manes.16G11890	0	1.83783	#NAME?	#NAME?	0.1187	0.2255	down	no
Manes.16G12090	1.0305183	2.456373	0.4195284	-1.25315952	0.5242	0.6858	down	no
Manes.16G12210	6.5377933	6.364264	1.0272662	0.038810033	1	1	up	no
Manes.16G12260	0.6570915	4.859969	0.1352049	-2.88678084	0.0996	0.1967	down	no
Manes.16G12450	4.0280616	0.615709	6.5421511	2.709765092	0.2192	0.3638	up	no
Manes.16G12790	212.52098	67.90612	3.1296293	1.645991792	######	3E-07	up	yes
Manes.16G12860	2.9179104	4.547863	0.6416003	-0.64025328	0.5985	0.7504	down	no
Manes.16G13150	10.651252	36.77755	0.2896129	-1.78780218	0.0003	0.0011	down	yes
Manes.16G13390	92.33377	107.8257	0.8563243	-0.22377082	0.411	0.5765	down	no
Manes.16G13700	17.207904	166.2562	0.1035023	-3.27226483	######	3E-05	down	yes
Manes.16G13820	76.156614	136.6277	0.5574024	-0.84320883	0.0062	0.0191	down	no
Manes.16G13850	39.551056	60.34179	0.6554505	-0.60944116	0.1901	0.3269	down	no
Manes.17G00050	57.738553	45.64104	1.2650577	0.339203242	0.3118	0.475	up	no
Manes.17G00180	2.5647419	6.389944	0.4013716	-1.31698972	0.1788	0.3112	down	no
Manes.17G00280	2.9179104	20.70973	0.1408956	-2.82730109	######	0.0003	down	yes
Manes.17G00480	375.40737	454.4167	0.8261301	-0.27555904	0.1273	0.2378	down	no
Manes.17G00740	383.41816	463.0306	0.8280622	-0.27218888	0.3061	0.4684	down	no
Manes.17G00770	3.933935	11.87209	0.3313601	-1.59352833	0.0388	0.0907	down	no
Manes.17G01180	7.4989228	0	Inf	Inf	######	0.0002	up	yes
Manes.17G01720	13.234737	22.71709	0.5825894	-0.77944861	0.1376	0.2531	down	no
Manes.17G02050	16.562341	10.37913	1.5957356	0.674221595	0.2162	0.3603	up	no
Manes.17G02420	1076.2349	891.6828	1.2069706	0.271390573	0.1975	0.3366	up	no
Manes.17G02650	46.960102	15.51135	3.027467	1.598111229	######	0.0004	up	yes
Manes.17G02860	0.3835559	1.505712	0.2547338	-1.9729375	0.4434	0.6075	down	no
Manes.17G03340	20.84368	0.887169	23.494595	4.554256972	0.0123	0.0345	up	yes
Manes.17G03560	137.04475	239.2175	0.5728875	-0.80367614	0.0029	0.0098	down	no

Manes.17G03850	0	2.756347	#NAME?	#NAME?	0.0562	0.1231	down	no
Manes.17G03860	2.8932876	0.307855	9.3982302	3.232389098	0.0507	0.113	up	no
Manes.17G04020	14.207396	24.97054	0.5689663	-0.81358501	0.1052	0.2054	down	no
Manes.17G04060	396.26272	32.22968	12.294961	3.6199953	######	1E-53	up	yes
Manes.17G04140	103.37143	207.9199	0.4971695	-1.00819031	0.002	0.0071	down	yes
Manes.17G04440	1.4690843	3.034271	0.4841639	-1.04643255	0.4924	0.6555	down	no
Manes.17G04450	1.1304094	3.007174	0.3759043	-1.41156272	0.3415	0.5071	down	no
Manes.17G04680	235.85597	90.92681	2.5939099	1.375128379	######	3E-06	up	yes
Manes.17G04690	50.753629	12.51126	4.0566353	2.02028362	######	5E-06	up	yes
Manes.17G04880	302.90486	259.8661	1.1656189	0.221096181	0.2241	0.3692	up	no
Manes.17G05320	21.004231	31.72174	0.6621399	-0.59479194	0.1913	0.3283	down	no
Manes.17G05330	22.971371	17.07693	1.3451702	0.427788734	0.378	0.5472	up	no
Manes.17G05350	1.4792134	2.147101	0.6889351	-0.5375601	0.8842	0.9752	down	no
Manes.17G05460	459.36626	249.0755	1.8442851	0.883061656	######	3E-05	up	no
Manes.17G05730	73.265946	78.11951	0.93787	-0.09254014	0.7854	0.9023	down	no
Manes.17G05860	28.815691	5.190669	5.5514404	2.472862154	######	5E-05	up	yes
Manes.17G05870	2.9280395	7.944182	0.3685766	-1.43996373	0.1076	0.2088	down	no
Manes.17G05910	12.977096	13.38063	0.9698418	-0.04417872	1	1	down	no
Manes.17G05930	11.269227	0.924981	12.183204	3.606821656	######	0.0003	up	yes
Manes.17G06070	109.87541	48.24331	2.2775264	1.187467776	######	0.0002	up	yes
Manes.17G07060	109.28525	8.449291	12.934251	3.693124575	######	9E-10	up	yes
Manes.17G07080	149.34968	181.0309	0.8249956	-0.27754163	0.2456	0.3966	down	no
Manes.17G07100	23.635447	16.07774	1.4700728	0.555887625	0.2108	0.3528	up	no
Manes.17G07140	141.51249	91.68793	1.5434147	0.626125734	0.0133	0.0368	up	no
Manes.17G07220	161.3834	145.9018	1.1061098	0.145494644	0.537	0.6972	up	no
Manes.17G07320	279.73179	278.154	1.0056722	0.008160168	0.9663	1	up	no
Manes.17G07370	3.6995158	11.94771	0.3096423	-1.69132554	0.2174	0.3618	down	no
Manes.17G07550	6.8909618	11.88563	0.5797723	-0.78644157	0.3381	0.5034	down	no
Manes.17G07760	3.1668233	10.35628	0.3057877	-1.70939773	0.0397	0.0924	down	no
Manes.17G07860	73.222466	175.0967	0.418183	-1.25779373	######	3E-05	down	yes
Manes.17G08650	37.02968	39.59313	0.9352551	-0.09656819	0.9168	0.9966	down	no
Manes.17G08780	45.185465	128.9449	0.3504245	-1.51282442	######	3E-05	down	yes
Manes.17G08820	8.9476339	15.82062	0.5655678	-0.8222282	0.1858	0.3208	down	no
Manes.17G08890	48.395834	115.1967	0.4201147	-1.25114482	######	0.0003	down	yes
Manes.17G09400	0.7468535	4.014862	0.1860222	-2.42645329	0.2654	0.42	down	no
Manes.17G09670	36.872044	1.517844	24.292383	4.602432141	######	6E-12	up	yes
Manes.17G09680	11.75116	0.591446	19.868521	4.312412547	0.0162	0.0436	up	yes
Manes.17G09690	330.24023	34.49889	9.5724872	3.258893831	######	1E-16	up	yes
Manes.17G09770	14.524298	32.81059	0.4426711	-1.17569304	0.0148	0.0402	down	yes
Manes.17G09980	50.172976	37.81596	1.3267672	0.407915209	0.2282	0.3745	up	no
Manes.17G10010	2.9729205	1.223538	2.4297741	1.280822167	0.4485	0.6126	up	no
Manes.17G10100	467.80931	265.716	1.7605615	0.816035597	######	2E-05	up	no
Manes.17G10150	12613.983	215.2372	58.605024	5.872952438	######	3E-266	up	yes
Manes.17G10170	317.82663	173.7627	1.8290843	0.87112155	0.0035	0.0116	up	no
Manes.17G10220	42.319945	44.21348	0.9571729	-0.06314858	0.9384	0.9995	down	no
Manes.17G10230	16.675096	9.46486	1.76179	0.81704197	0.1179	0.2244	up	no
Manes.17G10240	107.00576	139.9147	0.764793	-0.38685885	0.1538	0.2766	down	no
Manes.17G10640	3.281208	6.46415	0.5076008	-0.97823361	0.4046	0.5701	down	no

Manes.17G11070	2.2159379	7.675556	0.2887006	-1.79235392	0.0709	0.1489	down	no
Manes.17G11230	64.57421	73.13916	0.8828952	-0.1796859	0.6354	0.7803	down	no
Manes.17G11310	19.076323	38.67178	0.4932879	-1.01949817	0.021	0.0544	down	no
Manes.17G11850	1.1506677	0	Inf	Inf	0.1951	0.3333	up	no
Manes.17G11890	138.23577	367.0471	0.3766158	-1.40883446	######	2E-09	down	yes
Manes.17G11900	163.15838	102.573	1.5906569	0.669622676	0.0089	0.0262	up	no
Manes.17G11910	1186.3674	737.045	1.6096267	0.686726143	0.0105	0.03	up	no
Manes.17G12000	41.92015	40.63266	1.0316861	0.045004039	0.8974	0.9849	up	no
Manes.17G12040	31.861146	48.10025	0.6623905	-0.59424622	0.1498	0.271	down	no
Manes.17G12140	14.177009	5.152858	2.7512905	1.460108467	0.0185	0.0489	up	yes
Manes.17G12180	63.069154	206.2392	0.3058059	-1.70931201	######	2E-08	down	yes
Manes.17G12200	34.680384	97.82563	0.3545123	-1.4960926	######	7E-05	down	yes
Manes.17G12400	4.068578	2.464253	1.6510389	0.723374155	0.8168	0.9261	up	no
Manes.18G00010	16.212023	24.59698	0.6591061	-0.60141739	0.2295	0.3761	down	no
Manes.18G00160	551.86313	438.2798	1.2591571	0.332458327	0.0408	0.0946	up	no
Manes.18G00220	15.515815	26.7285	0.5804971	-0.78463935	0.132	0.2448	down	no
Manes.18G00260	1223.0673	935.4991	1.3073955	0.386695653	0.0075	0.0226	up	no
Manes.18G00530	29.252857	12.17348	2.4029994	1.264836291	0.0053	0.0167	up	yes
Manes.18G00720	194.7194	195.3617	0.9967123	-0.00475093	0.989	1	down	no
Manes.18G00810	19.170334	76.52414	0.2505136	-1.9970394	######	4E-06	down	yes
Manes.18G00980	1.877263	1.505712	1.2467607	0.318184549	0.9152	0.9966	up	no
Manes.18G01160	1.7527491	0	Inf	Inf	0.068	0.1437	up	no
Manes.18G01240	92.834101	37.83172	2.4538693	1.29505839	######	9E-05	up	yes
Manes.18G01420	1.4937071	0	Inf	Inf	0.1074	0.2085	up	no
Manes.18G01560	252.8312	198.9532	1.2708073	0.345745262	0.0894	0.18	up	no
Manes.18G01690	73.976418	101.3943	0.7295914	-0.45483945	0.126	0.236	down	no
Manes.18G01740	167.834	643.6927	0.2607362	-1.93933697	######	2E-16	down	yes
Manes.18G01760	151.95506	474.2106	0.3204379	-1.64188316	######	5E-10	down	yes
Manes.18G01790	2.5691064	0.603578	4.2564642	2.089655508	0.1669	0.2954	up	no
Manes.18G02020	59.038522	108.0127	0.5465886	-0.87147272	0.0183	0.0483	down	no
Manes.18G02190	6.1788602	3.047819	2.0273054	1.019563448	0.2303	0.3771	up	no
Manes.18G02290	48.317601	11.91982	4.0535525	2.019186836	######	5E-06	up	yes
Manes.18G02410	7.7622144	21.24415	0.3653813	-1.45252512	0.0137	0.0376	down	yes
Manes.18G02520	65.911911	54.32115	1.2133748	0.27902524	0.3866	0.5561	up	no
Manes.18G02760	327.57204	2071.131	0.1581609	-2.66053479	######	3E-34	down	yes
Manes.18G02860	3.5605083	1.209989	2.9425948	1.557088899	0.1828	0.3167	up	no
Manes.18G03110	12357.231	3299.209	3.7455132	1.905163418	######	3E-15	up	yes
Manes.18G03120	2165.5171	517.8359	4.18186	2.064144777	######	3E-48	up	yes
Manes.18G03380	6.068725	10.90062	0.5567322	-0.84494459	0.6155	0.7619	down	no
Manes.18G03430	288.83288	53.12958	5.4363855	2.442647761	######	4E-15	up	yes
Manes.18G03460	3.3463473	11.19855	0.2988196	-1.74265327	0.0243	0.0613	down	no
Manes.18G03580	10.671511	17.10119	0.6240216	-0.68033217	0.2388	0.388	down	no
Manes.18G03590	8.0705019	15.5698	0.5183435	-0.94801973	0.1518	0.2739	down	no
Manes.18G03600	609.60315	624.5863	0.976011	-0.03503066	0.889	0.9789	down	no
Manes.18G03610	868.38264	654.971	1.3258336	0.406899747	0.0254	0.0638	up	no
Manes.18G03620	164.01182	943.8135	0.1737757	-2.52470208	######	6E-18	down	yes
Manes.18G03630	121.12052	929.1747	0.1303528	-2.93950659	######	1E-11	down	yes
Manes.18G03640	29.355778	33.11544	0.8864681	-0.17385934	0.7364	0.8651	down	no

Manes.18G03650	4.0584489	15.30055	0.2652486	-1.91458293	0.0205	0.0532	down	no
Manes.18G03980	0.9856373	1.234252	0.7985704	-0.32450849	1	1	down	no
Manes.18G04180	5.0585799	12.2398	0.4132893	-1.27477592	0.1314	0.244	down	no
Manes.18G04210	356.18831	10.57213	33.691254	5.074302207	######	2E-15	up	yes
Manes.18G04310	4.1438464	0.604995	6.8493934	2.775976225	0.0351	0.0834	up	no
Manes.18G04390	262.882	263.8596	0.996295	-0.00535509	0.974	1	down	no
Manes.18G04440	4.0483198	12.48133	0.32435	-1.62437664	0.0272	0.0674	down	no
Manes.18G04480	193.9971	281.5918	0.6889301	-0.53757039	0.081	0.166	down	no
Manes.18G04500	732.82957	388.7729	1.884981	0.914549991	0.0003	0.0015	up	no
Manes.18G04880	1.4792134	7.000607	0.2112979	-2.24264983	0.0676	0.143	down	no
Manes.18G04900	106.29639	134.148	0.7923814	-0.33573308	0.2019	0.3414	down	no
Manes.18G04980	559.76766	747.8195	0.7485331	-0.41786189	0.0107	0.0305	down	no
Manes.18G05050	2.5242254	7.87644	0.320478	-1.64170293	0.1294	0.2411	down	no
Manes.18G05290	202.4702	57.06	3.5483736	1.8271579	######	1E-12	up	yes
Manes.18G05380	4.4361253	6.749159	0.6572857	-0.60540756	0.7078	0.842	down	no
Manes.18G05710	14.236383	0.309272	46.031979	5.524564557	######	9E-07	up	yes
Manes.18G05780	11.312708	29.00905	0.3899718	-1.35855847	0.1121	0.2157	down	no
Manes.18G05860	43.467418	12.1207	3.5862136	1.84246142	######	6E-05	up	yes
Manes.18G06320	43.590417	48.66035	0.8958098	-0.15873559	0.6948	0.8316	down	no
Manes.18G06340	41.944888	4.550697	9.2172441	3.204335451	######	7E-10	up	yes
Manes.18G06390	154.8531	199.1892	0.777417	-0.3632395	0.1754	0.3068	down	no
Manes.18G06480	0.3734268	1.51501	0.2464847	-2.02042975	0.4433	0.6075	down	no
Manes.18G06490	590.87145	342.0922	1.7272285	0.788458951	######	3E-05	up	no
Manes.18G07120	1.4242033	0	Inf	Inf	0.1207	0.2284	up	no
Manes.18G07220	197.26655	182.2469	1.0824138	0.114252204	0.5996	0.7512	up	no
Manes.18G07320	160.93762	177.0951	0.9087637	-0.13802287	0.557	0.714	down	no
Manes.18G07330	1.0753993	4.561412	0.2357602	-2.08460795	0.1063	0.2071	down	no
Manes.18G07510	132.35215	153.6345	0.8614739	-0.21512102	0.3836	0.5533	down	no
Manes.18G07700	360.51017	339.1731	1.062909	0.088018105	0.6405	0.7843	up	no
Manes.18G07770	35.449126	156.4323	0.22661	-2.1417165	######	1E-09	down	yes
Manes.18G08160	0.7569827	3.94712	0.191781	-2.38246814	0.0928	0.1857	down	no
Manes.18G08610	9.3703062	42.27324	0.2216605	-2.17357647	0.0006	0.0023	down	yes
Manes.18G08980	0.7468535	1.517844	0.492049	-1.0231261	0.8333	0.9386	down	no
Manes.18G09220	26.266843	35.60758	0.7376756	-0.43894161	0.3132	0.4765	down	no
Manes.18G09320	10.098302	14.9826	0.6740019	-0.56917543	0.373	0.5415	down	no
Manes.18G09360	10.590478	5.462129	1.9388917	0.955232253	0.162	0.2884	up	no
Manes.18G09370	2.9627914	0.912849	3.2456527	1.698508636	0.2105	0.3524	up	no
Manes.18G09420	154.61369	169.4936	0.9122096	-0.1325627	0.608	0.7556	down	no
Manes.18G09430	389.13299	339.3258	1.1467828	0.197592207	0.2727	0.4288	up	no
Manes.18G09680	0	1.544941	#NAME?	#NAME?	0.1858	0.3208	down	no
Manes.18G09730	62.400533	43.77532	1.4254729	0.511440634	0.1171	0.2233	up	no
Manes.18G09790	10.049056	12.72428	0.7897546	-0.34052372	0.6321	0.7774	down	no
Manes.18G09810	396.47867	279.4279	1.4188942	0.504767001	0.0043	0.014	up	no
Manes.18G09840	7.9112361	2.13497	3.7055492	1.889687373	0.0219	0.0562	up	no
Manes.18G09890	5.1439774	7.313508	0.703353	-0.50767924	0.5757	0.7291	down	no
Manes.18G10380	4.3971238	10.36274	0.4243204	-1.236774	0.1063	0.2071	down	no
Manes.18G10390	604.44231	881.4449	0.6857403	-0.54426578	0.0021	0.0075	down	no
Manes.18G10400	180.54117	155.9745	1.1575046	0.211017979	0.4759	0.6385	up	no

Manes.18G10630	3.0380597	6.382859	0.4759716	-1.0710525	0.3303	0.495	down	no
Manes.18G10650	65.75841	123.231	0.533619	-0.90611803	0.0018	0.0066	down	no
Manes.18G10920	98.167731	118.4175	0.8289966	-0.27056185	0.3409	0.5064	down	no
Manes.18G11000	1.877263	0	Inf	Inf	0.065	0.1386	up	no
Manes.18G11140	1.4589552	1.528558	0.9544649	-0.06723592	1	1	down	no
Manes.18G11670	64.520895	36.4812	1.7686066	0.8226132	0.0135	0.0372	up	no
Manes.18G11690	22.183886	28.27769	0.7845013	-0.35015217	0.45	0.6141	down	no
Manes.18G11730	10.054936	29.82325	0.3371509	-1.56853383	0.0064	0.0196	down	yes
Manes.18G11910	25.79364	33.44835	0.7711484	-0.37491957	0.387	0.5563	down	no
Manes.18G11930	9.834895	18.80858	0.5228941	-0.93540928	0.1542	0.2772	down	no
Manes.18G13740	75.671308	47.31075	1.5994526	0.67757824	0.031	0.0753	up	no
Manes.18G13830	10858.114	12251.7	0.8862534	-0.17420889	0.3085	0.4713	down	no
Manes.18G13850	690.19022	478.1783	1.4433742	0.529445329	0.002	0.0071	up	no
Manes.18G14020	38.570953	40.2038	0.9593858	-0.05981696	0.9286	0.9966	down	no
Manes.18G14060	47.486915	35.14565	1.3511464	0.434184037	0.2494	0.4013	up	no
Manes.18G14130	44.823747	80.14923	0.5592536	-0.83842545	0.0165	0.0443	down	no
Manes.18G14210	425.46683	352.0891	1.2084066	0.273105974	0.1277	0.2385	up	no
Manes.18G14610	6.5826743	5.131429	1.282815	0.359313153	0.6825	0.8211	up	no
Manes.S000700.v	35.434747	33.71556	1.050991	0.071750331	0.8629	0.9593	up	no
Manes.S002500.v	101.58806	73.81097	1.3763275	0.460823792	0.0942	0.188	up	no
Manes.S002600.v	16.520195	39.4441	0.4188255	-1.25557881	0.006	0.0187	down	yes
Manes.S002800.v	6.3481402	0.923564	6.8735282	2.781050837	0.1633	0.2902	up	no
Manes.S003200.v	3.6445057	5.821344	0.6260592	-0.6756291	0.6948	0.8317	down	no
Manes.S004000.v	14.524298	10.92568	1.3293729	0.410745874	0.4386	0.6042	up	no
Manes.S004800.v	54.246034	46.18051	1.1746522	0.232233692	0.4946	0.6576	up	no
Manes.S005400.v	1.74262	4.893529	0.356107	-1.48961732	0.255	0.4081	down	no
Manes.S006000.v	53.623579	72.10517	0.7436857	-0.42723509	0.2001	0.3391	down	no
Manes.S007000.v	0.3835559	1.827115	0.2099243	-2.25205906	0.3255	0.4899	down	no
Manes.S007200.v	2.4894735	6.05641	0.4110477	-1.28262215	0.2093	0.3508	down	no
Manes.S008700.v	1.4937071	0.591446	2.5255167	1.336578594	0.6689	0.8095	up	no
Manes.S009700.v	1.1304094	0.295723	3.8225271	1.934526736	0.5189	0.6813	up	no
Manes.S011000.v	6.4277731	10.37346	0.6196365	-0.69050604	0.3696	0.5381	down	no
Manes.S012700.v	3.6097538	5.400055	0.6684661	-0.58107368	0.6138	0.7604	down	no
Manes.S015900.v	1.0855284	0	Inf	Inf	0.2231	0.3682	up	no
Manes.S017500.v	5.1728497	19.74773	0.2619465	-1.93265565	0.0078	0.0232	down	yes
Manes.S019100.v	6.642049	0.295723	22.460369	4.489309694	0.0008	0.0031	up	yes
Manes.S019600.v	579.63588	310.2807	1.8681019	0.901573127	######	5E-07	up	no
Manes.S020300.v	11.426978	13.06994	0.8742944	-0.19380892	0.8278	0.9351	down	no
Manes.S020600.v	0	8.776363	#NAME?	#NAME?	0.0002	0.0011	down	yes
Manes.S023200.v	5.1974725	6.979973	0.7446264	-0.42541129	0.7545	0.8801	down	no
Manes.S023800.v	203.27297	118.6639	1.7130141	0.776537044	0.0823	0.1682	up	no
Manes.S026700.v	169.56829	197.4218	0.8589136	-0.21941509	0.3371	0.5023	down	no
Manes.S028000.v	317.43574	384.1578	0.826316	-0.27523449	0.1443	0.2629	down	no
Manes.S028600.v	1.7527491	5.207052	0.3366106	-1.57084733	0.1787	0.3111	down	no
Manes.S029100.v	2.181186	0.615709	3.5425596	1.824792129	0.4958	0.6588	up	no
Manes.S030600.v	13.906323	68.63181	0.2026221	-2.30313635	######	8E-07	down	yes
Manes.S030700.v	3.2117043	28.90615	0.111108	-3.16996567	######	8E-06	down	yes
Manes.S033800.v	14.972993	18.30268	0.8180767	-0.2896919	0.6529	0.7952	down	no

Manes.S037300.v	8.9968795	2.145684	4.1930117	2.067986854	0.0107	0.0305	up	yes
Manes.S037500.v	31.41792	29.49051	1.0653568	0.091336692	0.7788	0.897	up	no
Manes.S041300.v	107.13557	57.52021	1.8625726	0.897296692	0.0014	0.0052	up	no
Manes.S041700.v	218.69235	90.78043	2.4090252	1.268449503	######	2E-07	up	yes
Manes.S044900.v	6.9068555	33.40234	0.2067776	-2.2738482	0.0005	0.0021	down	yes
Manes.S045000.v	0	1.827115	#NAME?	#NAME?	0.1185	0.2252	down	no
Manes.S045300.v	3.3014663	1.519261	2.1730739	1.119737262	0.3808	0.5501	up	no
Manes.S045500.v	7.1500038	1.222121	5.8504888	2.548557161	0.0068	0.0207	up	yes
Manes.S046200.v	5.5275332	2.68577	2.058081	1.041299777	0.5476	0.7069	up	no
Manes.S046300.v	599.70956	472.0056	1.270556	0.345460011	0.0347	0.0828	up	no
Manes.S047000.v	147.48558	138.0622	1.0682543	0.095255177	0.6966	0.833	up	no
Manes.S049700.v	16.850256	26.12979	0.6448675	-0.63292526	0.2003	0.3393	down	no
Manes.S049800.v	18.058898	53.94555	0.3347616	-1.57879409	0.0021	0.0075	down	yes
Manes.S056200.v	101.97068	316.3906	0.3222936	-1.63355252	######	5E-07	down	yes
Manes.S056300.v	1.5342235	5.224852	0.2936396	-1.76788138	0.424	0.5894	down	no
Manes.S058300.v	3.2464562	6.739861	0.48168	-1.0538532	0.2736	0.4298	down	no
Manes.S059700.v	0.7019725	5.753601	0.1220058	-3.03497875	0.031	0.0753	down	no
Manes.S060100.v	26.311839	18.85206	1.3957011	0.480990017	0.2871	0.4458	up	no
Manes.S061600.v	74.047437	43.53963	1.7006905	0.76612059	0.014	0.0384	up	no
Manes.S061700.v	0.9856373	1.504295	0.6552153	-0.60995905	1	1	down	no
Manes.S065600.v	28.656426	30.97701	0.9250869	-0.11233921	0.8458	0.9471	down	no
Manes.S068200.v	17.471081	17.93558	0.9741018	-0.0378556	1	1	down	no
Manes.S068900.v	8.3990477	28.32684	0.2965049	-1.75387194	0.0034	0.0114	down	yes
Manes.S069800.v	218.46071	264.4416	0.8261208	-0.27557534	0.2325	0.3798	down	no
Manes.S071500.v	3569.9948	434.8607	8.2095129	3.037296621	######	2E-100	up	yes
Manes.S073800.v	51.165828	55.93967	0.9146608	-0.1286912	0.7714	0.8919	down	no
Manes.S074000.v	1.4488261	1.504295	0.9631261	-0.05420338	1	1	down	no
Manes.S075900.v	0.7121017	3.046402	0.2337517	-2.09695124	0.201	0.3403	down	no
Manes.S077200.v	130.19635	77.55406	1.6787818	0.747414693	0.0138	0.038	up	no
Manes.S077800.v	405.53494	260.8481	1.5546784	0.636616134	0.0006	0.0026	up	no
Manes.S077900.v	14.77769	5.745721	2.571947	1.362860923	0.0267	0.0664	up	no
Manes.S079300.v	1.4242033	10.10736	0.1409075	-2.82717938	0.0165	0.0443	down	yes
Manes.S079500.v	29.068969	49.19994	0.5908334	-0.75917676	0.0823	0.1681	down	no
Manes.S085300.v	23.576187	62.90592	0.3747849	-1.41586546	0.0003	0.0014	down	yes
Manes.S085500.v	57.55035	185.2307	0.3106955	-1.68642679	######	3E-08	down	yes
Manes.S087400.v	220.92255	347.6718	0.6354341	-0.65418553	0.0138	0.038	down	no
Manes.S087800.v	128.64512	117.194	1.0977109	0.134498203	0.5704	0.7245	up	no
Manes.S087900.v	102.22891	177.7444	0.5751456	-0.79800074	0.0021	0.0074	down	no
Manes.S088500.v	3.9730515	3.008591	1.320569	0.401159677	0.6833	0.8217	up	no
Manes.S089800.v	403.2944	167.7215	2.4045484	1.265765969	######	2E-10	up	yes
Manes.S090500.v	89.262063	118.5611	0.7528784	-0.40951126	0.2054	0.3459	down	no
Manes.S090700.v	2.6747621	5.116463	0.5227756	-0.93573638	0.5562	0.714	down	no
Manes.S091400.v	70.239416	181.5637	0.3868583	-1.37012269	0.0001	0.0006	down	yes
Manes.S092300.v	166.33476	189.0554	0.8798201	-0.18471946	0.4295	0.5951	down	no
Manes.S093200.v	8.9027529	32.68219	0.2724038	-1.87618116	0.0013	0.0048	down	yes
Manes.S093300.v	14.517199	45.11309	0.3217957	-1.63578293	0.0021	0.0075	down	yes
Manes.S093500.v	7.4480473	0.309272	24.082546	4.589916012	0.0003	0.0012	up	yes
Manes.S094600.v	1.8222528	1.209989	1.5060074	0.590728851	0.7396	0.8677	up	no

Manes.S094700.v	0.3734268	2.456373	0.1520236	-2.71763238	0.1763	0.308	down	no
Manes.S095500.v	125.69864	124.3285	1.0110201	0.015811647	0.8943	0.9827	up	no
Manes.S096600.v	89.266608	168.6839	0.5291945	-0.91812995	0.0005	0.0021	down	no
Manes.S096900.v	12.629806	10.33423	1.2221333	0.289401662	0.6396	0.7839	up	no
Manes.S097000.v	10.477608	5.801332	1.8060693	0.852853221	0.213	0.3559	up	no
Manes.S099300.v	45.728171	58.71098	0.7788691	-0.36054723	0.3256	0.49	down	no
Manes.S100700.v	152.73556	69.97994	2.1825619	1.126022578	0.0001	0.0007	up	yes
Manes.S102000.v	26.395722	59.27879	0.445281	-1.1672119	0.0024	0.0084	down	yes
Manes.S102100.v	112.9024	387.6974	0.2912127	-1.77985495	######	6E-09	down	yes
Manes.S104000.v	4.8053025	3.934988	1.2211733	0.288267898	0.8058	0.9177	up	no
Manes.S105300.v	71.165564	144.2447	0.493367	-1.01926696	0.0003	0.0014	down	yes
Manes.S105700.v	124.07105	5.725709	21.669114	4.437568237	######	2E-07	up	yes
Manes.S106300.v	121.73211	31.15456	3.9073612	1.966194638	######	8E-10	up	yes
Manes.S108900.v	77.590602	50.72049	1.5297684	0.613313279	0.0453	0.1034	up	no

Supplemental Data S2. Expression levels of lncRNAs under drought conditions

Gene	PEG_normaliz	ck_normaliz	FoldChang	Log2FoldCha	pval	padj	Up/Down	Significar
**Novel lncRNAs**								
lincRNA_10	114.7460898	173.81284	0.66017	-0.59908969	0.008	0.03	down	no
lincRNA_100	8.180856737	11.022015	0.742229	-0.43006417	0.522	0.68	down	no
lincRNA_102	100.4272982	93.401548	1.075221	0.1046331	0.648	0.79	up	no
lincRNA_103	86.94751642	117.84487	0.737813	-0.43867218	0.043	0.12	down	no
lincRNA_104	92.79012562	97.116973	0.955447	-0.06575217	0.764	0.86	down	no
lincRNA_105	36.65114875	32.53337	1.126571	0.17193815	0.775	0.87	up	no
lincRNA_106	16.42627749	15.079594	1.089305	0.12340797	0.827	0.9	up	no
lincRNA_107	0.68687455	0	Inf	Inf	0.412	0.58	up	no
lincRNA_108	184.4823407	169.33152	1.089474	0.12363218	0.475	0.64	up	no
lincRNA_109	158.4214151	179.83246	0.880939	-0.18288608	0.383	0.56	down	no
lincRNA_11	15.54186847	11.894615	1.306631	0.38585138	0.465	0.64	up	no
lincRNA_111	29.37986319	39.775039	0.738651	-0.43703566	0.181	0.32	down	no
lincRNA_112	8.708135598	9.7869275	0.889772	-0.16849213	0.839	0.91	down	no
lincRNA_113	718.5361894	610.30344	1.177343	0.23553409	0.11	0.23	up	no
lincRNA_114	6.127157394	0.6002376	10.20789	3.35161235	0.11	0.23	up	no
lincRNA_115	231.4670753	207.24922	1.116854	0.15944033	0.363	0.55	up	no
lincRNA_116	78.11209614	54.930822	1.422008	0.50793008	0.17	0.31	up	no
lincRNA_117	75.72967723	43.744155	1.731195	0.79176851	0.116	0.24	up	no
lincRNA_118	396.7248197	327.57224	1.211106	0.27632555	0.086	0.2	up	no
lincRNA_119	39.32715862	22.665168	1.735136	0.79504907	0.054	0.14	up	no
lincRNA_12	34150.09301	29775.783	1.146908	0.19775006	0.549	0.71	up	no
lincRNA_120	21.20068324	126.8603	0.167118	-2.58105803	0.043	0.12	down	no
lincRNA_121	130.5933011	146.72915	0.89003	-0.16807463	0.495	0.66	down	no
lincRNA_122	130.6137517	188.91831	0.691377	-0.53245576	0.122	0.25	down	no
lincRNA_123	9.934518812	17.528536	0.566763	-0.81918355	0.124	0.25	down	no
lincRNA_124	8.033490852	3.3770551	2.378845	1.25026129	0.102	0.22	up	no
lincRNA_125	7.231883701	16.051494	0.450543	-1.15026417	0.09	0.2	down	no
lincRNA_126	203.2831169	174.26168	1.166539	0.22223504	0.411	0.58	up	no
lincRNA_128	181.8294939	157.45493	1.154803	0.2076473	0.284	0.46	up	no
lincRNA_129	39.34593247	30.433869	1.292834	0.37053676	0.248	0.41	up	no
lincRNA_130	375.3710343	368.02128	1.019971	0.02852814	0.832	0.91	up	no
lincRNA_132	166.1717157	169.51917	0.980253	-0.02877362	0.987	1	down	no
lincRNA_134	166.4424516	125.98745	1.321103	0.40174344	0.036	0.1	up	no
lincRNA_135	50.8822025	71.705296	0.709602	-0.49491856	0.089	0.2	down	no
lincRNA_137	113.3845706	104.01828	1.090045	0.12438729	0.556	0.71	up	no
lincRNA_138	0	0.6139496	#NAME?	#NAME?	0.495	0.66	down	no
lincRNA_139	47.21782856	32.435068	1.455765	0.54177723	0.059	0.15	up	no
lincRNA_14	239.4407137	183.69387	1.303477	0.38236503	0.033	0.1	up	no
lincRNA_140	810.6890731	620.23068	1.307077	0.3863438	0.043	0.12	up	no
lincRNA_141	46.34965832	87.74076	0.528257	-0.9206885	2E-04	0	down	no
lincRNA_142	48.08784215	66.333233	0.724943	-0.46405964	0.214	0.37	down	no
lincRNA_143	6.481908214	7.0545617	0.918825	-0.12213786	0.877	0.94	down	no
lincRNA_144	32.71574211	43.489808	0.752262	-0.41069236	0.17	0.31	down	no
lincRNA_145	24.9748174	33.778962	0.73936	-0.43565087	0.202	0.35	down	no
lincRNA_146	170.7913316	132.13126	1.292588	0.37026296	0.056	0.14	up	no

lincRNA_147	40.86742772	78.380675	0.521397	-0.93954655	2E-04	0	down	no
lincRNA_148	47.53701994	35.701097	1.331528	0.41308306	0.14	0.27	up	no
lincRNA_15	65.45795373	54.374928	1.203826	0.26762691	0.315	0.5	up	no
lincRNA_151	2666.043528	1942.9062	1.372194	0.45648411	0.003	0.01	up	no
lincRNA_152	4.016745426	2.2320931	1.799542	0.84762986	0.37	0.56	up	no
lincRNA_153	12.35481851	6.8785158	1.796146	0.84490461	0.148	0.28	up	no
lincRNA_154	30.366775	16.572667	1.832341	0.87368791	0.021	0.07	up	no
lincRNA_155	3316.640021	3252.6814	1.019663	0.02809293	0.849	0.92	up	no
lincRNA_156	10.3937715	16.226874	0.640528	-0.64266589	0.204	0.36	down	no
lincRNA_157	5.881297685	4.6258548	1.271397	0.34641453	0.734	0.84	up	no
lincRNA_158	23.14674084	32.382206	0.714798	-0.48439219	0.182	0.32	down	no
lincRNA_159	110.2771826	115.62628	0.953738	-0.068335	0.79	0.88	down	no
lincRNA_16	161.7545344	186.69074	0.86643	-0.20684422	0.262	0.43	down	no
lincRNA_160	109.2719244	129.19387	0.845798	-0.24161481	0.394	0.57	down	no
lincRNA_161	394.4116475	274.67922	1.435899	0.52195441	0.003	0.01	up	no
lincRNA_162	20.52236263	34.741687	0.590713	-0.759471	0.072	0.17	down	no
lincRNA_163	217.7561624	219.59966	0.991605	-0.01216224	0.942	0.98	down	no
lincRNA_164	44.40284018	33.482383	1.326155	0.40724973	0.298	0.47	up	no
lincRNA_165	1.053474908	0.3069748	3.431796	1.77896393	0.584	0.74	up	no
lincRNA_166	161.2912727	129.29207	1.247495	0.31903458	0.099	0.21	up	no
lincRNA_167	27.54193616	26.747952	1.029684	0.04220157	0.997	1	up	no
lincRNA_168	432.5489349	472.34152	0.915755	-0.12696701	0.387	0.56	down	no
lincRNA_17	28.83088432	22.95091	1.256198	0.32906373	0.379	0.56	up	no
lincRNA_170	17.47482717	20.74005	0.842564	-0.2471412	0.616	0.76	down	no
lincRNA_171	202.4565249	183.55697	1.102963	0.14138424	0.449	0.62	up	no
lincRNA_172	601.1522893	535.84284	1.121882	0.16592058	0.427	0.59	up	no
lincRNA_173	78.01923009	56.675024	1.376607	0.46111666	0.06	0.15	up	no
lincRNA_175	125.3333868	113.48518	1.104403	0.14326683	0.481	0.65	up	no
lincRNA_176	20.20170817	25.520052	0.791601	-0.33715401	0.394	0.57	down	no
lincRNA_177	327.0263991	331.48438	0.986551	-0.01953378	0.882	0.94	down	no
lincRNA_178	38.36379016	26.110783	1.46927	0.55509951	0.089	0.2	up	no
lincRNA_179	48.08253665	92.281251	0.521043	-0.94052456	9E-04	0	down	no
lincRNA_18	38.60142903	31.461614	1.226937	0.29506158	0.412	0.58	up	no
lincRNA_180	621.7443294	430.05682	1.445726	0.53179417	0.029	0.09	up	no
lincRNA_181	71.76732308	140.67989	0.510146	-0.97101709	#####	####	down	no
lincRNA_182	35.31279254	47.200499	0.748144	-0.4186112	0.229	0.39	down	no
lincRNA_183	351.1983237	270.12014	1.300156	0.37868474	0.024	0.08	up	no
lincRNA_184	27.76902203	25.099401	1.106362	0.14582344	0.752	0.86	up	no
lincRNA_185	3.105711171	0.3001188	10.34827	3.3713181	0.034	0.1	up	no
lincRNA_186	132.9358799	189.81457	0.700346	-0.51386021	0.008	0.03	down	no
lincRNA_187	2.720872962	0.3001188	9.065987	3.18046403	0.054	0.14	up	no
lincRNA_188	372.3039644	235.19003	1.582992	0.6626541	#####	0	up	no
lincRNA_189	80.97432694	60.207782	1.344915	0.42751461	0.093	0.21	up	no
lincRNA_19	27.05605824	22.871513	1.182959	0.24239985	0.592	0.74	up	no
lincRNA_190	257.3419937	290.30053	0.886468	-0.17386032	0.297	0.47	down	no
lincRNA_191	116.7959468	166.96518	0.699523	-0.51555707	0.01	0.04	down	no
lincRNA_192	13.96219752	20.850411	0.669637	-0.57854981	0.202	0.35	down	no
lincRNA_195	66.02154174	77.80167	0.848588	-0.2368643	0.329	0.51	down	no

lincRNA_196	77.12096163	53.153552	1.450909	0.53695692	0.028	0.09	up	no
lincRNA_198	15.56010632	25.178465	0.617993	-0.69433842	0.117	0.24	down	no
lincRNA_199	43.27148864	67.065729	0.64521	-0.63215898	0.015	0.05	down	no
lincRNA_2	60.87544384	57.206594	1.064133	0.08967894	0.712	0.83	up	no
lincRNA_20	5.033744633	3.7049305	1.358661	0.44218556	0.632	0.77	up	no
lincRNA_200	2.762273896	5.412867	0.510316	-0.97053655	0.306	0.48	down	no
lincRNA_201	297.3250232	225.83026	1.316586	0.39680205	0.017	0.06	up	no
lincRNA_202	19.22010186	25.793969	0.745139	-0.42441782	0.332	0.51	down	no
lincRNA_203	134.2830037	133.96836	1.002349	0.00338438	0.94	0.98	up	no
lincRNA_204	74.4008492	144.15637	0.516112	-0.9542436	#####	0	down	no
lincRNA_205	13.10971923	15.161309	0.864683	-0.20975755	0.704	0.82	down	no
lincRNA_206	28.54453994	53.319759	0.535346	-0.90145545	0.007	0.03	down	no
lincRNA_207	336.5428127	539.09716	0.624271	-0.67975525	0.041	0.11	down	no
lincRNA_208	145.8165142	125.73367	1.159725	0.2137831	0.262	0.43	up	no
lincRNA_209	243.4408401	248.83615	0.978318	-0.03162486	0.887	0.94	down	no
lincRNA_21	7.168782763	1.9251183	3.723814	1.89678109	0.041	0.11	up	no
lincRNA_210	53.50366538	61.246255	0.873583	-0.19498391	0.459	0.63	down	no
lincRNA_211	5.519622559	1.5348739	3.596141	1.8464495	0.341	0.52	up	no
lincRNA_212	8.172469352	12.459907	0.655901	-0.60844939	0.39	0.57	down	no
lincRNA_213	32.14706232	36.003867	0.892878	-0.16346494	0.609	0.76	down	no
lincRNA_214	26.99204104	26.72175	1.010115	0.01451959	0.932	0.97	up	no
lincRNA_215	142.3768359	157.17703	0.905837	-0.14267597	0.471	0.64	down	no
lincRNA_216	119.5485839	160.70114	0.743919	-0.42678316	0.122	0.24	down	no
lincRNA_217	2.404060923	0.3415873	7.037911	2.81514722	0.094	0.21	up	no
lincRNA_218	97.75822342	103.59177	0.943687	-0.08361938	0.695	0.81	down	no
lincRNA_219	7.575320975	3.249999	2.330869	1.22086776	0.285	0.46	up	no
lincRNA_22	5.015506782	7.5202219	0.666936	-0.58437975	0.56	0.72	down	no
lincRNA_220	21.00677748	15.64787	1.342469	0.42488858	0.345	0.53	up	no
lincRNA_221	57.32217379	28.91923	1.982147	0.98706417	9E-04	0	up	no
lincRNA_222	13.85877846	18.74958	0.739151	-0.43605815	0.534	0.69	down	no
lincRNA_223	25.71385961	48.410148	0.531167	-0.91276334	0.006	0.02	down	no
lincRNA_224	59.53470829	48.272696	1.2333	0.30252359	0.237	0.39	up	no
lincRNA_225	131.5994756	171.08467	0.769207	-0.37855677	0.074	0.17	down	no
lincRNA_226	24.38042863	14.893485	1.636986	0.71104213	0.359	0.54	up	no
lincRNA_227	54.81393328	82.280889	0.666181	-0.58601471	0.031	0.09	down	no
lincRNA_228	18.70229319	29.297191	0.638365	-0.64754718	0.152	0.28	down	no
lincRNA_229	147.3824922	243.23463	0.605927	-0.72278348	#####	0	down	no
lincRNA_23	30.6845033	32.982443	0.930328	-0.10418804	0.81	0.89	down	no
lincRNA_231	13.5832008	6.2371423	2.177792	1.1228664	0.099	0.21	up	no
lincRNA_232	25.74687316	46.585327	0.552682	-0.8554784	0.008	0.03	down	no
lincRNA_233	70.20535398	110.80668	0.633584	-0.65839186	0.013	0.04	down	no
lincRNA_235	2.354272604	2.4698428	0.953207	-0.06913783	1	1	down	no
lincRNA_237	5.263912385	1.8696052	2.815521	1.49340183	0.137	0.26	up	no
lincRNA_238	63.54468514	32.966081	1.927578	0.94678909	8E-04	0	up	no
lincRNA_239	39.46104534	43.01696	0.917337	-0.12447639	0.734	0.84	down	no
lincRNA_24	14.51648188	6.3406473	2.289432	1.19498982	0.032	0.09	up	no
lincRNA_240	225.2424961	308.65688	0.72975	-0.45452493	0.007	0.03	down	no
lincRNA_241	33.31418701	65.506972	0.508559	-0.97551177	0.002	0.01	down	no

lincRNA_242	11.27179221	11.481152	0.981765	-0.02655048	1	1	down	no
lincRNA_243	32.7633648	40.94809	0.800119	-0.32171263	0.331	0.51	down	no
lincRNA_244	26.62143171	29.774245	0.894109	-0.16147678	0.69	0.81	down	no
lincRNA_245	5.483146858	2.9012231	1.889943	0.91834289	0.386	0.56	up	no
lincRNA_246	32.66833313	26.087565	1.252257	0.32453056	0.33	0.51	up	no
lincRNA_247	17.39695053	14.299105	1.216646	0.28290955	0.52	0.68	up	no
lincRNA_248	126.9443946	116.73816	1.087428	0.1209204	0.592	0.74	up	no
lincRNA_249	20.84571871	9.9420728	2.096718	1.06813254	0.106	0.22	up	no
lincRNA_25	2.770661281	1.8696052	1.48195	0.5674967	0.632	0.77	up	no
lincRNA_250	460.5308394	454.47928	1.013315	0.01908324	0.852	0.92	up	no
lincRNA_251	62.88990788	43.475431	1.446562	0.53262818	0.121	0.24	up	no
lincRNA_252	0	0.9486809	#NAME?	#NAME?	0.292	0.47	down	no
lincRNA_253	158.8864513	115.11283	1.380267	0.46494743	0.021	0.06	up	no
lincRNA_255	3.082548088	3.7186424	0.828944	-0.27065263	0.876	0.94	down	no
lincRNA_256	13.23538511	4.2470043	3.116405	1.63988269	0.071	0.17	up	no
lincRNA_257	15.12894195	13.653859	1.108034	0.14800231	0.876	0.94	up	no
lincRNA_258	49.29885569	32.438051	1.519785	0.603867	0.063	0.15	up	no
lincRNA_26	66.39268706	44.912003	1.478284	0.56392326	0.028	0.09	up	no
lincRNA_260	6.573097467	9.6968019	0.677862	-0.56093563	0.397	0.57	down	no
lincRNA_261	9.39993538	5.5399231	1.696763	0.76278488	0.3	0.48	up	no
lincRNA_262	46.51141963	43.204067	1.076552	0.10641785	0.78	0.87	up	no
lincRNA_263	177.3531155	124.28249	1.427016	0.51300157	0.008	0.03	up	no
lincRNA_264	35.55134767	47.886755	0.742405	-0.42972243	0.232	0.39	down	no
lincRNA_265	410.4752033	494.16952	0.830636	-0.26771097	0.108	0.23	down	no
lincRNA_266	154.9346501	205.93575	0.752345	-0.41053447	0.065	0.16	down	no
lincRNA_267	263.5686914	276.54782	0.953067	-0.06934998	0.716	0.83	down	no
lincRNA_268	1885.305782	1149.5297	1.640067	0.71375485	#####	####	up	no
lincRNA_269	260.0505789	311.21068	0.835609	-0.25909932	0.125	0.25	down	no
lincRNA_27	269.4294225	136.57421	1.972769	0.98022232	#####	####	up	no
lincRNA_271	68.02415624	57.126973	1.190754	0.25187508	0.378	0.56	up	no
lincRNA_272	59.04298887	108.46842	0.544333	-0.87743739	2E-04	0	down	no
lincRNA_273	870.1949433	599.52261	1.45148	0.53752447	3E-04	0	up	no
lincRNA_274	11.69803135	10.163127	1.151027	0.20292136	0.722	0.84	up	no
lincRNA_275	101.9440347	120.45349	0.846335	-0.24069885	0.243	0.4	down	no
lincRNA_276	5.62558751	10.601696	0.530631	-0.91421942	0.203	0.35	down	no
lincRNA_277	21.57199512	6.7678222	3.187435	1.67239603	0.018	0.06	up	no
lincRNA_278	21.59862036	17.897548	1.206792	0.27117724	0.529	0.69	up	no
lincRNA_279	2.816987448	0.9140683	3.081813	1.62377919	0.246	0.41	up	no
lincRNA_28	120.3001311	99.138751	1.213452	0.27911723	0.424	0.59	up	no
lincRNA_280	518.257458	489.09534	1.059625	0.08355325	0.583	0.74	up	no
lincRNA_281	285.0055504	396.72143	0.718402	-0.47713632	0.004	0.02	down	no
lincRNA_282	12.70664317	17.893342	0.710132	-0.49383995	0.319	0.5	down	no
lincRNA_283	22.94574422	25.223582	0.909694	-0.13654658	0.713	0.83	down	no
lincRNA_284	25.05908235	16.684026	1.501981	0.58686614	0.407	0.58	up	no
lincRNA_286	1.053474908	3.1530174	0.334116	-1.58157717	0.219	0.38	down	no
lincRNA_287	395.8494501	376.52877	1.051313	0.07219176	0.624	0.76	up	no
lincRNA_289	217.952223	130.66163	1.668066	0.7381764	#####	0	up	no
lincRNA_291	281.226491	205.53863	1.368242	0.45232292	0.008	0.03	up	no

lincRNA_292	2.372510455	2.9567362	0.802409	-0.31759107	0.909	0.96	down		no
lincRNA_294	16.13262854	26.701847	0.604177	-0.72695802	0.109	0.23	down		no
lincRNA_295	24.19382744	45.146105	0.535901	-0.89996249	0.012	0.04	down		no
lincRNA_296	21.07972888	13.943806	1.511763	0.59623189	0.175	0.32	up		no
lincRNA_297	3.737872168	0.9555369	3.911803	1.96783381	0.085	0.2	up		no
lincRNA_298	107.9424444	83.793209	1.2882	0.36535703	0.095	0.21	up		no
lincRNA_299	9.33044613	7.9793587	1.169323	0.22567326	0.725	0.84	up		no
lincRNA_3	13.54580884	10.680428	1.268283	0.3428771	0.55	0.71	up		no
lincRNA_30	535.5033452	485.21678	1.103637	0.14226614	0.356	0.54	up		no
lincRNA_300	31.86910532	29.760533	1.070851	0.09875813	0.802	0.89	up		no
lincRNA_301	6.5267713	1.0247619	6.369061	2.67108075	0.136	0.26	up		no
lincRNA_302	175.5589108	136.74142	1.283875	0.36050486	0.435	0.6	up		no
lincRNA_303	37.56672797	29.672393	1.26605	0.34033417	0.574	0.73	up		no
lincRNA_304	12.35828067	12.129381	1.018871	0.02697206	0.962	0.98	up		no
lincRNA_305	7.749692362	9.2490589	0.83789	-0.25516754	0.812	0.89	down		no
lincRNA_306	3.384584428	2.5459238	1.329413	0.41078946	0.719	0.83	up		no
lincRNA_308	45.70548123	53.229077	0.858656	-0.21984735	0.508	0.67	down		no
lincRNA_309	23.69117474	38.925658	0.608626	-0.71637167	0.05	0.13	down		no
lincRNA_310	498.5040584	669.52504	0.744564	-0.42553276	0.004	0.02	down		no
lincRNA_311	8.653422046	16.07925	0.538173	-0.89385747	0.096	0.21	down		no
lincRNA_313	77.98037505	106.91142	0.729392	-0.45523299	0.051	0.14	down		no
lincRNA_314	127.8884424	163.69428	0.781264	-0.35611804	0.074	0.17	down		no
lincRNA_315	192.9351464	185.07614	1.042464	0.05999709	0.74	0.85	up		no
lincRNA_316	52.84087018	64.102803	0.824315	-0.27873321	0.277	0.45	down		no
lincRNA_317	566.2535169	485.40853	1.16655	0.22224866	0.147	0.28	up		no
lincRNA_318	40.48258951	29.947641	1.351779	0.43485923	0.216	0.37	up		no
lincRNA_319	3.100785938	1.2073312	2.568298	1.36081249	0.379	0.56	up		no
lincRNA_32	0.993836123	0.3415873	2.909465	1.54075365	0.592	0.74	up		no
lincRNA_320	27.47244692	13.953978	1.96879	0.97730895	0.1	0.22	up		no
lincRNA_322	390.4872213	240.0888	1.626428	0.70170723	#####	0	up		no
lincRNA_323	142.085244	149.0151	0.953496	-0.06870184	0.814	0.89	down		no
lincRNA_325	57.81605884	76.156103	0.759178	-0.39748939	0.102	0.22	down		no
lincRNA_326	0.68687455	0	Inf	Inf	0.412	0.58	up		no
lincRNA_327	64.89272526	57.18182	1.134849	0.18250021	0.519	0.68	up		no
lincRNA_328	26.53387116	33.617293	0.789292	-0.3413684	0.387	0.56	down		no
lincRNA_329	15.79866146	5.8648152	2.693804	1.42964477	0.129	0.25	up		no
lincRNA_33	12.65539177	6.2645662	2.020155	1.01446564	0.081	0.19	up		no
lincRNA_330	888.4178087	754.09645	1.178122	0.23648926	0.097	0.21	up		no
lincRNA_331	172.5802184	173.52065	0.99458	-0.00784029	1	1	down		no
lincRNA_332	46.44068102	45.539332	1.019793	0.02827599	0.924	0.97	up		no
lincRNA_333	247.2635132	164.7698	1.50066	0.58559757	1E-03	0	up		no
lincRNA_334	2.717180714	0.3012768	9.018883	3.17294884	0.055	0.14	up		no
lincRNA_336	6.183334026	8.168784	0.756947	-0.40173639	0.606	0.75	down		no
lincRNA_337	1057.988062	848.05214	1.247551	0.31909848	0.042	0.12	up		no
lincRNA_339	180.330948	146.19539	1.233493	0.30274919	0.149	0.28	up		no
lincRNA_34	2.093637197	0.6002376	3.488014	1.8024059	0.512	0.68	up		no
lincRNA_340	328.2077635	361.95485	0.906764	-0.1412004	0.367	0.55	down		no
lincRNA_341	569.0890139	629.19133	0.904477	-0.14484446	0.33	0.51	down		no

lincRNA_343	312.1269224	264.86188	1.178452	0.23689258	0.135	0.26	up	no
lincRNA_344	195.4635658	205.84107	0.949585	-0.07463114	0.671	0.8	down	no
lincRNA_345	63.03364506	47.898482	1.315984	0.39614217	0.129	0.25	up	no
lincRNA_346	4.14587346	0.6485621	6.392408	2.67635952	0.026	0.08	up	no
lincRNA_347	29.13746563	17.441394	1.670593	0.74036004	0.065	0.16	up	no
lincRNA_348	29.32168748	24.589622	1.192442	0.2539186	0.498	0.66	up	no
lincRNA_349	3.430910595	8.5795963	0.399892	-1.32231824	0.086	0.2	down	no
lincRNA_35	10.93328017	13.6992	0.798096	-0.3253654	0.62	0.76	down	no
lincRNA_351	43.62585919	61.346886	0.711134	-0.49180656	0.059	0.15	down	no
lincRNA_352	97.15945624	104.85031	0.926649	-0.10990478	0.695	0.81	down	no
lincRNA_353	41.50451395	36.93907	1.123594	0.16812071	0.752	0.86	up	no
lincRNA_354	251.6919466	218.96768	1.149448	0.20094112	0.233	0.39	up	no
lincRNA_355	246.8294443	189.77244	1.30066	0.37924402	0.036	0.1	up	no
lincRNA_358	0	0	NA	NA	NA	NA	--	no
lincRNA_359	50.41364617	85.394657	0.590361	-0.76033151	0.002	0.01	down	no
lincRNA_36	38.39750625	28.861399	1.33041	0.41187138	0.361	0.55	up	no
lincRNA_360	104.8575169	81.585	1.285255	0.36205446	0.111	0.23	up	no
lincRNA_361	123.4128245	69.953241	1.764219	0.81902952	2E-04	0	up	no
lincRNA_362	114.710697	71.859013	1.59633	0.67475891	0.003	0.01	up	no
lincRNA_364	2.400598771	1.2625116	1.901447	0.92709762	0.595	0.74	up	no
lincRNA_365	85.28304453	50.638368	1.684159	0.75202802	0.002	0.01	up	no
lincRNA_366	4.740095677	13.720434	0.345477	-1.53333799	0.088	0.2	down	no
lincRNA_367	30.86104032	25.755152	1.198247	0.26092567	0.496	0.66	up	no
lincRNA_368	308.4545885	344.41826	0.895581	-0.15910351	0.385	0.56	down	no
lincRNA_369	101.2743152	165.238	0.6129	-0.70627715	5E-04	0	down	no
lincRNA_37	876.1393748	490.93553	1.784632	0.83562681	#####	####	up	no
lincRNA_371	144.4363768	138.23273	1.044878	0.06333492	0.713	0.83	up	no
lincRNA_372	12.58244037	20.222417	0.622203	-0.68454368	0.162	0.3	down	no
lincRNA_374	34.81906322	39.468397	0.882201	-0.18082052	0.654	0.79	down	no
lincRNA_375	102.3972794	79.803759	1.283113	0.35964877	0.112	0.23	up	no
lincRNA_379	109.5206522	132.2069	0.828403	-0.2715945	0.218	0.38	down	no
lincRNA_38	9.688659103	10.052434	0.963812	-0.05317591	0.995	1	down	no
lincRNA_380	327.9509706	298.51577	1.098605	0.13567299	0.379	0.56	up	no
lincRNA_383	283.0230063	276.59172	1.023252	0.03316138	0.841	0.91	up	no
lincRNA_384	251.5201211	332.90349	0.755535	-0.40443018	0.014	0.05	down	no
lincRNA_385	145.3898948	143.48048	1.013308	0.0190725	0.946	0.98	up	no
lincRNA_386	13.1560454	3.9772927	3.307789	1.72586724	0.022	0.07	up	no
lincRNA_387	7.462431719	1.3317367	5.603534	2.48633698	0.057	0.15	up	no
lincRNA_388	0.343437275	0	Inf	Inf	0.956	0.98	up	no
lincRNA_389	52.1760759	36.405172	1.433205	0.51924501	0.053	0.14	up	no
lincRNA_39	42.019563	44.956679	0.934668	-0.09747432	0.76	0.86	down	no
lincRNA_390	202.7811667	199.2486	1.017729	0.02535409	0.983	1	up	no
lincRNA_393	1.016999206	5.9301675	0.171496	-2.54375431	0.019	0.06	down	no
lincRNA_394	33.10333992	30.287565	1.092968	0.12825119	0.81	0.89	up	no
lincRNA_395	152.6190768	169.90444	0.898264	-0.15478826	0.485	0.65	down	no
lincRNA_396	76.50011418	75.308276	1.015826	0.02265348	0.918	0.96	up	no
lincRNA_398	62.6575745	73.37862	0.853894	-0.22787085	0.368	0.55	down	no
lincRNA_399	465.7738629	490.14828	0.950271	-0.07358858	0.604	0.75	down	no

lincRNA_40	128.4996059	83.685831	1.5355	0.61870864	0.005	0.02	up	no
lincRNA_400	2.455312322	0.9555369	2.569563	1.36152313	0.342	0.52	up	no
lincRNA_402	101.0992413	126.22918	0.800918	-0.32027327	0.134	0.26	down	no
lincRNA_404	64.02401903	45.957001	1.393129	0.4783286	0.06	0.15	up	no
lincRNA_405	38.4575253	35.466331	1.084339	0.11681586	0.756	0.86	up	no
lincRNA_406	383.8461448	420.68337	0.912435	-0.13220664	0.408	0.58	down	no
lincRNA_407	12.48887178	8.6655166	1.441215	0.52728549	0.466	0.64	up	no
lincRNA_409	41.33014256	43.807845	0.943442	-0.08399489	0.805	0.89	down	no
lincRNA_41	24.76743246	44.577829	0.5556	-0.84788204	0.061	0.15	down	no
lincRNA_410	15.74779033	5.8579592	2.688272	1.42667935	0.053	0.14	up	no
lincRNA_411	0.68687455	0	Inf	Inf	0.412	0.58	up	no
lincRNA_412	0	0	NA	NA	NA	NA	--	no
lincRNA_413	178.4662292	165.70145	1.077035	0.10706489	0.565	0.72	up	no
lincRNA_415	6.898296891	1.5557744	4.433996	2.14860732	0.015	0.05	up	no
lincRNA_416	116.8925973	99.459329	1.17528	0.23300496	0.272	0.45	up	no
lincRNA_418	395.4555219	425.15466	0.930145	-0.1044723	0.538	0.7	down	no
lincRNA_419	19.28812803	31.127215	0.619655	-0.69046338	0.071	0.17	down	no
lincRNA_42	48.12648347	47.306655	1.01733	0.02478787	0.928	0.97	up	no
lincRNA_420	35.19659685	36.364036	0.967896	-0.0470764	0.93	0.97	down	no
lincRNA_421	50.74377083	56.581915	0.89682	-0.15711028	0.57	0.72	down	no
lincRNA_422	40.31314336	41.172128	0.979137	-0.03041773	0.943	0.98	down	no
lincRNA_423	110.9297942	135.4883	0.818741	-0.28852138	0.178	0.32	down	no
lincRNA_424	14.62136402	12.757376	1.146111	0.19674633	0.662	0.79	up	no
lincRNA_425	236.8384157	274.33517	0.863318	-0.2120365	0.229	0.39	down	no
lincRNA_426	34.25985362	38.023648	0.901014	-0.15037798	0.77	0.87	down	no
lincRNA_428	1231.159006	1191.3735	1.033395	0.04739135	0.747	0.85	up	no
lincRNA_429	9.408322765	11.077196	0.849342	-0.23558323	0.76	0.86	down	no
lincRNA_43	5.780257966	6.1812966	0.935121	-0.09677562	0.918	0.96	down	no
lincRNA_430	340.8598489	353.24256	0.964946	-0.0514805	0.744	0.85	down	no
lincRNA_431	0	0	NA	NA	NA	NA	--	no
lincRNA_433	30.5697707	19.597074	1.559915	0.6414675	0.128	0.25	up	no
lincRNA_434	81.45944433	144.95895	0.561948	-0.83149057	#####	0	down	no
lincRNA_436	22.40731837	18.913566	1.184722	0.24454858	0.798	0.89	up	no
lincRNA_437	181.8924283	156.50724	1.162198	0.21685612	0.228	0.39	up	no
lincRNA_438	48.47614251	35.759261	1.355625	0.43895799	0.123	0.25	up	no
lincRNA_439	2.135038131	5.3573539	0.398525	-1.32725876	0.183	0.32	down	no
lincRNA_440	281.7300708	335.85802	0.838837	-0.25353794	0.239	0.4	down	no
lincRNA_441	236.8259722	183.54613	1.29028	0.36768458	0.039	0.11	up	no
lincRNA_442	188.1886624	128.33676	1.466366	0.55224527	0.003	0.01	up	no
lincRNA_443	27.97348081	29.61556	0.944553	-0.08229558	0.858	0.92	down	no
lincRNA_444	291.5533663	567.42449	0.513819	-0.96066844	#####	####	down	no
lincRNA_445	4.471072884	2.4561308	1.820372	0.86423366	0.4	0.57	up	no
lincRNA_446	101.929259	94.976893	1.073201	0.10191977	0.676	0.8	up	no
lincRNA_447	315.0425702	297.69626	1.058268	0.0817057	0.62	0.76	up	no
lincRNA_448	75.39447161	69.750103	1.080923	0.11226339	0.653	0.79	up	no
lincRNA_449	89.01361212	55.074798	1.616231	0.69263367	0.003	0.01	up	no
lincRNA_45	1.680710673	0.3001188	5.600151	2.48546583	0.223	0.38	up	no
lincRNA_450	323.4882271	354.07392	0.913618	-0.13033739	0.412	0.58	down	no

lincRNA_451	9.366921831	7.5685464	1.237612	0.30755878	0.655	0.79	up	no
lincRNA_452	311.1825523	296.30524	1.050209	0.07067701	0.628	0.77	up	no
lincRNA_453	165.4289145	155.93055	1.060914	0.0853078	0.657	0.79	up	no
lincRNA_46	7.767930213	10.142559	0.765875	-0.3848196	0.618	0.76	down	no
lincRNA_47	12.31796255	8.3859657	1.468878	0.5547148	0.387	0.56	up	no
lincRNA_48	168.3060984	138.985	1.210966	0.27615827	0.199	0.35	up	no
lincRNA_49	109.0851094	113.64509	0.959875	-0.05908118	0.823	0.9	down	no
lincRNA_50	20.81801066	22.754629	0.914891	-0.12832784	0.828	0.9	down	no
lincRNA_51	78.24377006	80.18261	0.97582	-0.03531349	0.949	0.98	down	no
lincRNA_52	97.26487437	127.48506	0.762951	-0.39033738	0.071	0.17	down	no
lincRNA_53	174.3778289	133.84969	1.302788	0.38160284	0.088	0.2	up	no
lincRNA_54	14.52486927	26.80767	0.541818	-0.88412065	0.036	0.1	down	no
lincRNA_56	3.142186872	0.6485621	4.844851	2.27645241	0.087	0.2	up	no
lincRNA_57	34.06324624	48.621363	0.700582	-0.51337442	0.173	0.31	down	no
lincRNA_58	40.37624429	27.742972	1.455368	0.5413844	0.11	0.23	up	no
lincRNA_59	294.1621503	447.41353	0.657473	-0.60499727	1E-04	0	down	no
lincRNA_6	99.63116307	191.22425	0.521017	-0.94059652	#####	0	down	no
lincRNA_60	473.550941	385.16276	1.229483	0.2980514	0.05	0.13	up	no
lincRNA_61	8.666734664	8.31409	1.042415	0.05993016	0.953	0.98	up	no
lincRNA_62	4.740095677	1.2210431	3.882005	1.95680203	0.138	0.26	up	no
lincRNA_64	38510.27916	38743.032	0.993992	-0.00869327	1	1	down	no
lincRNA_65	211.7907049	170.17017	1.244582	0.31566109	0.12	0.24	up	no
lincRNA_66	64.12635527	53.568013	1.197102	0.25954562	0.308	0.49	up	no
lincRNA_67	133.7962096	152.63156	0.876596	-0.19001608	0.379	0.56	down	no
lincRNA_68	39.91991776	67.715181	0.589527	-0.76237056	0.012	0.04	down	no
lincRNA_69	28.85258433	57.496649	0.501813	-0.99477733	7E-04	0	down	no
lincRNA_7	46.28401149	92.544979	0.500125	-0.9996408	#####	0	down	no
lincRNA_70	2.047311031	0.3069748	6.669314	2.73753828	0.138	0.26	up	no
lincRNA_71	28.73438957	37.968135	0.756803	-0.40201074	0.232	0.39	down	no
lincRNA_72	10.6627943	15.406248	0.692108	-0.53092995	0.392	0.57	down	no
lincRNA_73	81.13132957	89.861063	0.902853	-0.147437	0.6	0.75	down	no
lincRNA_74	385.8472382	723.90751	0.533006	-0.9077756	#####	####	down	no
lincRNA_75	315.4619322	577.11583	0.546618	-0.87139499	#####	####	down	no
lincRNA_76	98.46241956	84.689819	1.162624	0.21738465	0.514	0.68	up	no
lincRNA_77	0.325199424	1.2625116	0.257581	-1.95690006	0.471	0.64	down	no
lincRNA_79	1115.732488	948.35907	1.176487	0.23448586	0.095	0.21	up	no
lincRNA_8	28.12923408	28.474138	0.987887	-0.0175819	1	1	down	no
lincRNA_80	630.8792765	571.60583	1.103696	0.14234332	0.306	0.48	up	no
lincRNA_81	46.83553624	45.174194	1.036776	0.05210473	0.881	0.94	up	no
lincRNA_82	13895.86049	14239.205	0.975887	-0.03521342	0.878	0.94	down	no
lincRNA_83	12.64554131	12.111464	1.044097	0.06225551	0.951	0.98	up	no
lincRNA_84	90.39774768	55.465042	1.629815	0.70470805	0.003	0.01	up	no
lincRNA_85	115.7059853	159.15048	0.727023	-0.45992797	0.039	0.11	down	no
lincRNA_86	146.5925789	137.77823	1.063975	0.08946417	0.65	0.79	up	no
lincRNA_87	204.7085085	337.92645	0.605778	-0.72313819	#####	0	down	no
lincRNA_88	35.36258086	57.50284	0.614971	-0.70140963	0.012	0.04	down	no
lincRNA_89	66.6730234	82.424209	0.808901	-0.30596499	0.198	0.35	down	no
lincRNA_9	33.3320446	32.807063	1.016002	0.02290339	0.958	0.98	up	no

lincRNA_90	23.10533991	29.353594	0.787138	-0.34531083	0.372	0.56	down	no
lincRNA_91	495.745627	577.51949	0.858405	-0.22026959	0.145	0.27	down	no
lincRNA_92	16.20904209	6.571541	2.466551	1.30249521	0.025	0.08	up	no
lincRNA_93	38.37071446	35.408167	1.083668	0.11592347	0.694	0.81	up	no
lincRNA_94	63.55399961	43.904826	1.44754	0.53360339	0.037	0.11	up	no
lincRNA_95	185.2346483	217.37321	0.85215	-0.23082019	0.223	0.38	down	no
lincRNA_96	37.36356572	39.851785	0.937563	-0.0930122	0.825	0.9	down	no
lincRNA_97	26.11839875	25.972333	1.005624	0.00809082	1	1	up	no
lincRNA_98	166.830752	208.04938	0.801881	-0.31854072	0.077	0.18	down	no
lincRNA_99	56.7186371	58.866871	0.963507	-0.05363308	0.92	0.96	down	no
lncNAT_1	118.9621551	83.034286	1.432687	0.51872361	0.018	0.06	up	no
lncNAT_10	317.0353814	300.78799	1.054016	0.07589691	0.662	0.79	up	no
lncNAT_100	1.355511248	0.6070936	2.232788	1.15884629	0.618	0.76	up	no
lncNAT_102	102.4827937	55.937226	1.832104	0.87350109	8E-04	0	up	no
lncNAT_103	29.64964651	34.255459	0.865545	-0.20831903	0.773	0.87	down	no
lncNAT_104	232.3215418	178.62192	1.300633	0.37921379	0.064	0.16	up	no
lncNAT_105	2448.479199	2194.7334	1.115616	0.15784021	0.284	0.46	up	no
lncNAT_106	48.59558663	28.656375	1.695804	0.76196919	0.017	0.06	up	no
lncNAT_107	76.27249232	96.670354	0.788996	-0.34191068	0.172	0.31	down	no
lncNAT_108	47.95302835	33.020372	1.452226	0.53826557	0.163	0.3	up	no
lncNAT_109	1525.010099	1144.0462	1.332997	0.41467344	0.004	0.02	up	no
lncNAT_110	23.95781819	10.814007	2.215443	1.14759528	0.101	0.22	up	no
lncNAT_112	29.30344963	30.955375	0.946635	-0.07911941	0.901	0.95	down	no
lncNAT_113	15.0742284	28.328832	0.532116	-0.91018696	0.029	0.09	down	no
lncNAT_114	182.0521433	184.38412	0.987353	-0.01836269	0.915	0.96	down	no
lncNAT_115	121.9679094	98.559638	1.237504	0.30743275	0.151	0.28	up	no
lncNAT_116	42.88427109	37.439773	1.14542	0.1958769	0.533	0.69	up	no
lncNAT_117	19.36708747	16.649081	1.163253	0.2181645	0.625	0.77	up	no
lncNAT_119	58.53978935	56.525737	1.035631	0.05050965	0.884	0.94	up	no
lncNAT_12	1689.548365	1129.6017	1.495703	0.58082346	#####	0	up	no
lncNAT_120	8.848030356	19.829855	0.446197	-1.16424586	0.124	0.25	down	no
lncNAT_122	0.733200716	2.5390678	0.288768	-1.79201883	0.427	0.59	down	no
lncNAT_123	2.088711964	0	Inf	Inf	0.039	0.11	up	no
lncNAT_124	88.35357652	151.09934	0.584738	-0.77413691	9E-04	0	down	no
lncNAT_125	83.64227171	89.474898	0.934813	-0.09725075	0.666	0.79	down	no
lncNAT_126	69.4840028	80.766593	0.860306	-0.21707782	0.452	0.62	down	no
lncNAT_127	19.31513353	38.498815	0.501707	-0.9950824	0.013	0.04	down	no
lncNAT_128	1034.318012	920.02006	1.124234	0.16894261	0.225	0.38	up	no
lncNAT_129	86.51343671	140.29119	0.61667	-0.69742828	0.001	0.01	down	no
lncNAT_13	343.1387908	233.6516	1.468592	0.5544333	7E-04	0	up	no
lncNAT_130	255.2857013	198.82266	1.283987	0.36063051	0.053	0.14	up	no
lncNAT_131	249.7533129	300.84385	0.830176	-0.26851104	0.108	0.23	down	no
lncNAT_132	119.0675732	104.82951	1.135821	0.18373573	0.41	0.58	up	no
lncNAT_133	116.1285486	163.2241	0.711467	-0.49113142	0.124	0.25	down	no
lncNAT_134	16.01681312	14.212296	1.126969	0.17244751	0.809	0.89	up	no
lncNAT_135	1.401837415	4.3946284	0.318989	-1.64842216	0.159	0.3	down	no
lncNAT_137	231.7828045	252.45317	0.918122	-0.12324227	0.553	0.71	down	no
lncNAT_138	118.8808163	123.1382	0.965426	-0.05076243	0.797	0.89	down	no

lncNAT_139	59.19327009	45.444238	1.302547	0.38133578	0.128	0.25	up	no
lncNAT_14	86.89059008	121.93993	0.712569	-0.4888988	0.064	0.16	down	no
lncNAT_140	44.88071099	70.550827	0.636147	-0.65256747	0.013	0.04	down	no
lncNAT_141	15.08900409	21.821978	0.691459	-0.53228429	0.273	0.45	down	no
lncNAT_143	330.1849913	368.37932	0.896318	-0.15791752	0.304	0.48	down	no
lncNAT_144	299.4089657	357.81313	0.836775	-0.25708892	0.113	0.23	down	no
lncNAT_146	53.00301176	26.034037	2.035912	1.02567529	0.017	0.06	up	no
lncNAT_149	264.7836525	376.50324	0.703271	-0.50784821	0.036	0.1	down	no
lncNAT_15	23.28171037	12.422644	1.874135	0.90622475	0.033	0.1	up	no
lncNAT_150	0	0	NA	NA	NA	NA	--	no
lncNAT_151	3.412672744	2.8737991	1.187513	0.24794284	0.857	0.92	up	no
lncNAT_152	194.450945	213.48294	0.91085	-0.13471452	0.478	0.65	down	no
lncNAT_154	110.3277314	65.762971	1.677657	0.74644807	7E-04	0	up	no
lncNAT_156	150.7822326	148.06288	1.018366	0.02625641	0.891	0.94	up	no
lncNAT_157	30.71221135	29.2429	1.050245	0.07072599	0.874	0.94	up	no
lncNAT_158	111.2787036	151.54211	0.734309	-0.44554125	0.028	0.09	down	no
lncNAT_159	198.806427	145.82625	1.36331	0.44711393	0.015	0.05	up	no
lncNAT_16	37.11278078	73.279321	0.506456	-0.98149002	2E-04	0	down	no
lncNAT_160	112.1595924	109.54638	1.023855	0.03401126	0.903	0.95	up	no
lncNAT_165	6.518383915	1.6249995	4.011314	2.00407507	0.046	0.12	up	no
lncNAT_166	3.095860705	1.5143059	2.044409	1.03168386	0.427	0.59	up	no
lncNAT_168	298.4865201	416.03467	0.717456	-0.47903797	0.058	0.15	down	no
lncNAT_17	27.97418335	31.634011	0.884307	-0.17738049	0.725	0.84	down	no
lncNAT_170	9.825091708	9.228491	1.064648	0.09037611	0.902	0.95	up	no
lncNAT_171	139.4181684	114.87862	1.213613	0.27930821	0.19	0.34	up	no
lncNAT_172	70.22104594	69.317168	1.01304	0.01869076	0.981	1	up	no
lncNAT_173	2.367585222	2.9704481	0.797046	-0.32726424	0.969	0.99	down	no
lncNAT_174	9.422015649	18.528192	0.508523	-0.97561451	0.115	0.24	down	no
lncNAT_175	8.973696237	11.89882	0.754167	-0.40704429	0.57	0.72	down	no
lncNAT_176	92.14578198	84.536767	1.090008	0.12433918	0.545	0.71	up	no
lncNAT_177	4.164111311	7.9516022	0.523682	-0.93323695	0.225	0.38	down	no
lncNAT_18	129.627173	191.53887	0.676767	-0.56326903	0.007	0.02	down	no
lncNAT_181	12.97821186	27.57832	0.470595	-1.08744293	0.042	0.12	down	no
lncNAT_182	254.1789759	224.14113	1.134013	0.18143727	0.296	0.47	up	no
lncNAT_183	198.5446616	152.9747	1.297892	0.37617046	0.04	0.11	up	no
lncNAT_187	46.0071373	38.738883	1.187622	0.24807534	0.535	0.7	up	no
lncNAT_19	1954.09381	3246.6029	0.601889	-0.7324312	0.024	0.07	down	no
lncNAT_190	21.18953625	32.751882	0.646972	-0.6282258	0.096	0.21	down	no
lncNAT_191	3.105711171	1.590387	1.952802	0.96554583	0.415	0.58	up	no
lncNAT_192	101.9385627	88.116627	1.15686	0.21021374	0.349	0.53	up	no
lncNAT_193	28.40426491	38.505339	0.737671	-0.43895091	0.184	0.33	down	no
lncNAT_196	126.830589	191.94383	0.660769	-0.59778142	0.004	0.02	down	no
lncNAT_198	29.48474532	22.581234	1.305719	0.38484443	0.313	0.49	up	no
lncNAT_199	2.052236263	0.6002376	3.41904	1.77359127	0.505	0.67	up	no
lncNAT_2	365.5780399	374.75015	0.975525	-0.03574964	0.789	0.88	down	no
lncNAT_20	134.7263979	193.42009	0.696548	-0.52170513	0.014	0.05	down	no
lncNAT_200	1000.473037	1607.5255	0.622368	-0.68415932	#####	####	down	no
lncNAT_201	1.337273398	1.2971241	1.030953	0.04397788	1	1	up	no

lncNAT_202	2.070474114	1.2834122	1.613257	0.68997658	0.681	0.8	up	no
lncNAT_203	68.48887015	60.240409	1.136926	0.185138	0.47	0.64	up	no
lncNAT_204	49.84496637	70.362067	0.708407	-0.49735005	0.062	0.15	down	no
lncNAT_205	23.69609997	35.828486	0.661376	-0.59645744	0.091	0.2	down	no
lncNAT_206	44.84678118	58.756177	0.763269	-0.3897361	0.149	0.28	down	no
lncNAT_207	47.06424092	92.34903	0.509634	-0.97246548	4E-04	0	down	no
lncNAT_208	27.94993746	38.17094	0.732231	-0.44962965	0.18	0.32	down	no
lncNAT_21	278.4236273	188.62382	1.476079	0.56176976	0.053	0.14	up	no
lncNAT_210	208.2579361	210.28248	0.990372	-0.01395721	0.985	1	down	no
lncNAT_211	95.69635041	170.86904	0.560057	-0.83635524	#####	0	down	no
lncNAT_212	122.3201143	137.65217	0.888617	-0.17036566	0.399	0.57	down	no
lncNAT_214	24.60074592	27.50754	0.894327	-0.16112506	0.693	0.81	down	no
lncNAT_215	68.84691657	64.009028	1.075581	0.10511663	0.669	0.8	up	no
lncNAT_216	4.535636901	9.5282772	0.476019	-1.07091023	0.171	0.31	down	no
lncNAT_217	16.77956523	24.268602	0.69141	-0.53238568	0.22	0.38	down	no
lncNAT_219	222.3601949	222.74615	0.998267	-0.00250194	0.981	1	down	no
lncNAT_22	123.7427246	89.482976	1.382863	0.46765856	0.045	0.12	up	no
lncNAT_220	212.6161197	138.5659	1.534404	0.61767868	8E-04	0	up	no
lncNAT_221	159.459734	140.91111	1.131634	0.17840685	0.424	0.59	up	no
lncNAT_223	186.3895216	105.84122	1.76103	0.81641915	9E-04	0	up	no
lncNAT_224	6.553396536	1.2556556	5.219103	2.38380196	0.017	0.05	up	no
lncNAT_225	27.97239799	16.100151	1.7374	0.79692973	0.062	0.15	up	no
lncNAT_226	14.92102102	19.911237	0.749377	-0.41623659	0.411	0.58	down	no
lncNAT_227	1672.843016	858.36599	1.948869	0.96263725	#####	####	up	no
lncNAT_228	56.08810574	51.704599	1.08478	0.11740225	0.676	0.8	up	no
lncNAT_229	24.36473667	15.075389	1.616193	0.69259941	0.098	0.21	up	no
lncNAT_24	5.534398257	0.6831746	8.101001	3.01810023	0.131	0.26	up	no
lncNAT_25	1.685635905	0.6485621	2.599036	1.37797638	0.433	0.6	up	no
lncNAT_26	54.68626832	39.874671	1.371454	0.455706	0.101	0.22	up	no
lncNAT_27	8.051728703	16.476351	0.488684	-1.03302627	0.118	0.24	down	no
lncNAT_28	2926.21333	1807.6103	1.61883	0.69495131	#####	####	up	no
lncNAT_29	438.5589224	555.20739	0.789901	-0.34025609	0.02	0.06	down	no
lncNAT_3	24.98466787	27.787091	0.899147	-0.15337175	0.664	0.79	down	no
lncNAT_32	166.2440009	167.43781	0.99287	-0.01032304	0.976	1	down	no
lncNAT_33	563.6044654	585.07444	0.963304	-0.05393715	0.754	0.86	down	no
lncNAT_34	11.56890332	1.8421813	6.280003	2.65076518	0.056	0.15	up	no
lncNAT_35	18.67220581	30.45742	0.613059	-0.70590137	0.066	0.16	down	no
lncNAT_37	19.97992781	17.487068	1.142554	0.19226296	0.651	0.79	up	no
lncNAT_38	185.2210026	223.49944	0.828731	-0.27102351	0.142	0.27	down	no
lncNAT_39	18.53815254	16.127575	1.149469	0.20096797	0.657	0.79	up	no
lncNAT_4	24.0942508	29.371844	0.820318	-0.28574489	0.505	0.67	down	no
lncNAT_40	100.9199447	128.49876	0.785377	-0.34854311	0.104	0.22	down	no
lncNAT_41	306.0428427	378.65371	0.808239	-0.30714542	0.06	0.15	down	no
lncNAT_42	38.91276902	54.322066	0.716334	-0.4812947	0.082	0.19	down	no
lncNAT_43	335.7166772	285.45407	1.176079	0.23398556	0.143	0.27	up	no
lncNAT_44	130.7128901	66.909362	1.953581	0.96612142	1E-04	0	up	no
lncNAT_46	3.064310237	0.9486809	3.230075	1.69156761	0.172	0.31	up	no
lncNAT_48	494.3398965	423.45261	1.167403	0.22330284	0.29	0.47	up	no

lncNAT_49	2.005910097	0	Inf	Inf	0.039	0.11	up	no
lncNAT_51	33.62823945	48.555122	0.692579	-0.52995023	0.078	0.18	down	no
lncNAT_52	13.66054144	6.9062723	1.977991	0.98403554	0.096	0.21	up	no
lncNAT_53	144.2176783	173.29784	0.832195	-0.26500567	0.287	0.46	down	no
lncNAT_54	4676.865809	4221.0963	1.107974	0.1479243	0.464	0.64	up	no
lncNAT_55	228.9641403	388.73245	0.589002	-0.76365588	#####	####	down	no
lncNAT_56	157.1362622	89.291996	1.759802	0.81541338	#####	0	up	no
lncNAT_58	86.76914689	92.16735	0.94143	-0.08707363	0.742	0.85	down	no
lncNAT_6	1.34219863	0.3069748	4.372342	2.12840617	0.359	0.54	up	no
lncNAT_63	135.211135	84.951883	1.59162	0.67049613	0.002	0.01	up	no
lncNAT_64	215.3939298	120.72552	1.784162	0.8352469	#####	0	up	no
lncNAT_65	29.06835665	40.968991	0.709521	-0.49508283	0.134	0.26	down	no
lncNAT_68	1.076637991	1.5489185	0.69509	-0.52472795	0.863	0.93	down	no
lncNAT_69	16.74162645	13.623452	1.228883	0.29734738	0.548	0.71	up	no
lncNAT_7	252.6746937	164.03111	1.540407	0.62331174	5E-04	0	up	no
lncNAT_72	56.37265393	72.580214	0.776695	-0.36458082	0.33	0.51	down	no
lncNAT_73	10.29273178	6.2436657	1.648508	0.72116074	0.291	0.47	up	no
lncNAT_75	0.343437275	0.3001188	1.144338	0.19451297	1	1	up	no
lncNAT_77	325.3861731	252.91818	1.286527	0.36348222	0.059	0.15	up	no
lncNAT_78	39.45758318	33.59606	1.174471	0.23201053	0.497	0.66	up	no
lncNAT_79	30.30074791	23.992367	1.262933	0.3367779	0.384	0.56	up	no
lncNAT_8	48.18049448	76.55022	0.629397	-0.66795732	0.028	0.09	down	no
lncNAT_81	53.95198479	41.506526	1.299843	0.37833783	0.163	0.3	up	no
lncNAT_82	22.6909503	18.253512	1.243101	0.31394295	0.552	0.71	up	no
lncNAT_83	96.78630101	128.90759	0.750819	-0.4134624	0.06	0.15	down	no
lncNAT_85	64.79114955	50.346328	1.286909	0.3639102	0.178	0.32	up	no
lncNAT_86	14.43860524	12.919044	1.117622	0.16043204	0.772	0.87	up	no
lncNAT_87	4.117785144	9.2075904	0.447216	-1.16095511	0.114	0.23	down	no
lncNAT_88	60.77455985	53.1519	1.143413	0.19334628	0.622	0.76	up	no
lncNAT_90	46.65953522	53.11894	0.878397	-0.18705442	0.592	0.74	down	no
lncNAT_91	106.4466581	69.091145	1.54067	0.62355793	0.006	0.02	up	no
lncNAT_92	137.2825363	180.63872	0.759984	-0.39595905	0.036	0.1	down	no
lncNAT_94	364.3616628	326.79739	1.114947	0.15697473	0.331	0.51	up	no
lncNAT_95	94.6490501	160.79737	0.588623	-0.76458391	2E-04	0	down	no
lncNAT_96	123.047843	134.62719	0.91399	-0.12975049	0.516	0.68	down	no
lncNAT_97	31.00823965	51.957948	0.596795	-0.74469283	0.062	0.15	down	no
lncNAT_98	40.12583962	41.97163	0.956023	-0.06488291	0.843	0.91	down	no
lincRNA_1	17.4797524	38.695096	0.45173	-1.146466	0.002	0.01	down	yes
lincRNA_101	174.586532	0.3415873	511.1037	8.99747222	#####	####	up	yes
lincRNA_110	108.4175556	44.401559	2.441751	1.28791615	#####	####	up	yes
lincRNA_127	43.30704808	20.067604	2.158058	1.10973344	8E-04	0	up	yes
lincRNA_13	171.4014015	53.191481	3.222347	1.6881118	#####	####	up	yes
lincRNA_131	77.6017586	0	Inf	Inf	#####	####	up	yes
lincRNA_133	12.46424562	26.745634	0.466029	-1.10150781	0.011	0.04	down	yes
lincRNA_136	7.401329855	0.9072124	8.158321	3.02827231	0.002	0.01	up	yes
lincRNA_149	326.1182694	28.59202	11.40592	3.5117108	#####	####	up	yes
lincRNA_150	14.45191786	3.6840299	3.922856	1.97190428	0.004	0.02	up	yes
lincRNA_169	17.01519421	5.0157666	3.392342	1.7622815	0.004	0.02	up	yes

lincRNA_174	15.06584101	1.2902682	11.67652	3.54553836	#####	####	up		yes
lincRNA_193	18.5115273	51.238606	0.361281	-1.4688073	2E-04	0	down		yes
lincRNA_194	18.41594881	41.409545	0.444727	-1.16900762	0.013	0.04	down		yes
lincRNA_197	10.20262535	2.5873923	3.943208	1.97936971	0.007	0.03	up		yes
lincRNA_230	50.17893345	166.74832	0.300926	-1.73251854	#####	0	down		yes
lincRNA_234	12.82245859	35.211886	0.364151	-1.45738958	4E-04	0	down		yes
lincRNA_236	7.804405914	0	Inf	Inf	#####	####	up		yes
lincRNA_254	10.86141158	40.098709	0.270867	-1.88434417	#####	####	down		yes
lincRNA_259	43.4679403	125.85841	0.345372	-1.53377794	#####	####	down		yes
lincRNA_270	3.852224505	12.243058	0.314646	-1.66820029	0.014	0.05	down		yes
lincRNA_285	36.19335914	88.787743	0.407639	-1.29463551	#####	####	down		yes
lincRNA_288	23.08325964	5.8674659	3.934111	1.97603752	0.012	0.04	up		yes
lincRNA_29	19.5733896	1.8973617	10.31611	3.36682695	#####	####	up		yes
lincRNA_290	44.14258504	94.453069	0.467349	-1.09742656	#####	####	down		yes
lincRNA_293	30.56777163	4.401817	6.944353	2.7958403	#####	####	up		yes
lincRNA_307	505.9815773	1127.2411	0.448867	-1.1556394	#####	####	down		yes
lincRNA_31	29.70890503	5.9713034	4.97528	2.31477765	#####	####	up		yes
lincRNA_312	10.63616906	23.91032	0.444836	-1.1686548	0.015	0.05	down		yes
lincRNA_321	4.12763561	15.958717	0.258645	-1.95095719	0.003	0.01	down		yes
lincRNA_324	32.1337497	4.1085542	7.821182	2.96738663	#####	####	up		yes
lincRNA_335	123.3150224	41.038216	3.004883	1.58730865	#####	####	up		yes
lincRNA_338	101.8840737	46.753519	2.179174	1.12378168	#####	####	up		yes
lincRNA_342	45.2655492	579.3776	0.078128	-3.67801855	#####	####	down		yes
lincRNA_350	56.16235367	159.27245	0.352618	-1.50382148	#####	####	down		yes
lincRNA_356	270.9700104	57.826842	4.685886	2.22832196	#####	####	up		yes
lincRNA_357	180.3725626	53.425025	3.376181	1.75539231	#####	####	up		yes
lincRNA_363	18.0754377	51.808104	0.348892	-1.5191472	#####	0	down		yes
lincRNA_370	21.7157323	7.2893281	2.979113	1.57488285	0.001	0	up		yes
lincRNA_373	270.8675656	85.772951	3.15796	1.65899299	#####	####	up		yes
lincRNA_376	28.45205416	2.5113113	11.32956	3.50202004	#####	####	up		yes
lincRNA_377	17.53446595	48.844179	0.358988	-1.47799315	#####	0	down		yes
lincRNA_378	101.2584567	206.23429	0.490988	-1.0262418	#####	####	down		yes
lincRNA_381	17.428501	40.303401	0.432433	-1.20945308	0.001	0.01	down		yes
lincRNA_382	56.99091916	0.6831746	83.42072	6.38233392	6E-04	0	up		yes
lincRNA_391	65.41016448	31.06153	2.105826	1.07438593	#####	0	up		yes
lincRNA_392	6.075419392	24.835811	0.244623	-2.03136595	0.002	0.01	down		yes
lincRNA_397	16.98164467	3.4462802	4.927529	2.30086429	1E-04	0	up		yes
lincRNA_4	4625.360994	127.29408	36.33603	5.18332875	#####	####	up		yes
lincRNA_401	27.94793838	65.092287	0.429359	-1.21974473	#####	0	down		yes
lincRNA_403	9.686279764	0	Inf	Inf	#####	####	up		yes
lincRNA_408	20.65695189	5.0229552	4.11251	2.04001908	0.003	0.01	up		yes
lincRNA_414	119.8004517	263.95543	0.453866	-1.13966101	#####	####	down		yes
lincRNA_417	39.86282487	18.535048	2.150673	1.10478806	0.002	0.01	up		yes
lincRNA_427	49.18233772	11.560216	4.254448	2.08897192	1E-04	0	up		yes
lincRNA_432	41.26704162	85.525586	0.482511	-1.05136607	#####	0	down		yes
lincRNA_435	142.3768359	296.65026	0.479948	-1.05904862	#####	####	down		yes
lincRNA_44	25.00420224	6.9340289	3.606014	1.85040483	2E-04	0	up		yes
lincRNA_5	6.077369075	24.783585	0.245218	-2.02786606	0.002	0.01	down		yes

lincRNA_55	14.52633235	43.238347	0.335959	-1.57364087	#####	0	down		yes
lincRNA_63	64.3944618	159.89956	0.402718	-1.31215744	#####	####	down		yes
lincRNA_78	29.3497758	71.133813	0.4126	-1.27718602	#####	####	down		yes
lncNAT_101	56.39506731	27.342888	2.062513	1.04440336	3E-04	0	up		yes
lncNAT_11	26.21835565	52.843927	0.496147	-1.01116048	0.002	0.01	down		yes
lncNAT_111	118.295148	255.59215	0.462828	-1.1114526	#####	0	down		yes
lncNAT_118	16.1937304	42.003259	0.385535	-1.37506591	2E-04	0	down		yes
lncNAT_121	144.4156038	49.219489	2.934114	1.55292503	#####	####	up		yes
lncNAT_136	2.336034754	17.590906	0.132798	-2.91269611	7E-04	0	down		yes
lncNAT_142	39.67405805	11.439351	3.468209	1.79419078	#####	####	up		yes
lncNAT_145	123.2494336	41.479659	2.971322	1.57110506	3E-04	0	up		yes
lncNAT_147	25.88438858	111.66678	0.2318	-2.10904587	#####	####	down		yes
lncNAT_148	1014.832595	262.76469	3.862135	1.94939845	#####	####	up		yes
lncNAT_153	118.6309476	55.464045	2.13888	1.09685569	#####	####	up		yes
lncNAT_155	114.5381689	23.225258	4.931621	2.30206193	#####	####	up		yes
lncNAT_161	35.54187748	102.37658	0.347168	-1.52629395	#####	####	down		yes
lncNAT_162	49.7647104	24.420764	2.037803	1.0270147	7E-04	0	up		yes
lncNAT_163	25.13933832	4.5125106	5.571031	2.47794445	#####	####	up		yes
lncNAT_164	541.0750086	192.13558	2.81611	1.49370391	#####	####	up		yes
lncNAT_167	295.1565441	7.8480972	37.60868	5.2329936	#####	####	up		yes
lncNAT_169	30.12036848	61.786011	0.487495	-1.03654082	9E-04	0	down		yes
lncNAT_178	104.15771	42.447354	2.453809	1.29502312	4E-04	0	up		yes
lncNAT_179	99.13581494	37.912955	2.614827	1.38671546	#####	####	up		yes
lncNAT_180	10.99676137	0.6070936	18.11378	4.179016	#####	0	up		yes
lncNAT_184	89.0668626	422.7674	0.210676	-2.24690346	#####	####	down		yes
lncNAT_185	4.879074176	69.079751	0.07063	-3.82358357	#####	####	down		yes
lncNAT_186	16.8381212	4.3949611	3.831233	1.93780889	0.001	0.01	up		yes
lncNAT_188	36.9743491	3.829336	9.655551	3.27135859	#####	####	up		yes
lncNAT_189	2.070474114	13.923238	0.148706	-2.74946172	2E-04	0	down		yes
lncNAT_194	42.39125515	112.02085	0.378423	-1.40192875	#####	0	down		yes
lncNAT_195	378.6719006	5.0572351	74.87726	6.22645571	#####	####	up		yes
lncNAT_197	3648.835772	1093.0835	3.338113	1.73903262	#####	####	up		yes
lncNAT_209	160.4621241	68.318178	2.348747	1.2318914	#####	####	up		yes
lncNAT_213	7.14561968	34.21134	0.208867	-2.25934359	#####	####	down		yes
lncNAT_218	19.32990923	57.711845	0.334938	-1.5780326	0.002	0.01	down		yes
lncNAT_222	37.69677226	153.16059	0.246126	-2.02253221	#####	####	down		yes
lncNAT_23	4.107934679	0	Inf	Inf	0.002	0.01	up		yes
lncNAT_30	316.1094771	10.100758	31.29562	4.96788879	#####	####	up		yes
lncNAT_31	10.60353578	67.432297	0.157247	-2.66889432	0.006	0.02	down		yes
lncNAT_36	31.38322739	77.808859	0.403337	-1.3099407	#####	0	down		yes
lncNAT_45	29.32915861	10.193534	2.877232	1.52468131	0.008	0.03	up		yes
lncNAT_47	84.0137973	182.99587	0.459102	-1.12311293	#####	####	down		yes
lncNAT_5	29.67480867	0.9418249	31.50778	4.97763608	#####	####	up		yes
lncNAT_50	14.21444554	2.5873923	5.493734	2.4577871	2E-04	0	up		yes
lncNAT_57	16.89391757	5.1055596	3.308926	1.72636292	0.005	0.02	up		yes
lncNAT_59	510.5707109	234.7854	2.174627	1.12076805	#####	####	up		yes
lncNAT_60	42.55252763	14.633837	2.907818	1.53993676	#####	0	up		yes
lncNAT_61	7553.278668	3305.0419	2.285381	1.19243446	#####	####	up		yes

lncNAT_62	1736.099204	0.6485621	2676.844	11.3863171	#####	####	up	yes
lncNAT_66	7.274747714	0	Inf	Inf	#####	0	up	yes
lncNAT_67	29.40540561	72.364587	0.406351	-1.29920247	#####	0	down	yes
lncNAT_70	63.4134023	4.6464227	13.64779	3.77059546	#####	####	up	yes
lncNAT_71	270.4984773	17.0903	15.8276	3.98437083	#####	####	up	yes
lncNAT_74	12.90271456	40.259614	0.320488	-1.64165872	1E-04	0	down	yes
lncNAT_76	5.8216589	0.9555369	6.092553	2.6070469	0.009	0.03	up	yes
lncNAT_80	85.6994332	217.30542	0.394373	-1.34236657	#####	####	down	yes
lncNAT_84	11.78575845	48.811885	0.241453	-2.05018785	#####	####	down	yes
lncNAT_89	21.9641379	103.60117	0.212007	-2.23781845	#####	####	down	yes
lncNAT_9	3.430910595	15.386569	0.222981	-2.16500813	0.002	0.01	down	yes
lncNAT_93	46.06823917	0.9901494	46.52655	5.53998246	#####	####	up	yes
lncNAT_99	3.797510953	29.850326	0.127218	-2.97462068	#####	####	down	yes
**Known lncRNAs**								
Manes.01G000	3.84752304	9.9057331	0.388414	-1.36433379	0.063	0.48	up	no
Manes.01G000	178.2145205	341.56411	0.52176	-0.9385415	#####	0.78	up	no
Manes.01G000	3.086350073	4.3895572	0.703112	-0.50817371	0.757	0.92	down	no
Manes.01G001	53.54704818	32.567959	1.644163	0.71735372	0.029	0.02	down	yes
Manes.01G002	391.9332927	919.56075	0.426218	-1.23033676	#####	0	down	yes
Manes.01G003	382.9451563	160.2722	2.389342	1.25661362	#####	0.05	up	no
Manes.01G004	0	4.3184089	#NAME?	#NAME?	0.004	0.69	up	no
Manes.01G005	194.3916453	200.06123	0.971661	-0.04147538	0.811	0.28	down	no
Manes.01G006	7.284056382	4.0506768	1.798232	0.84657913	0.293	0.42	down	no
Manes.01G007	7.437108085	1.5271269	4.87	2.28392177	0.092	1	down	no
Manes.01G009	18.5501785	6.8067429	2.725265	1.44639655	0.061	1	up	no
Manes.01G011	108.4463752	188.44919	0.575467	-0.79719373	2E-04	0.01	down	no
Manes.01G011	19.30391622	24.901153	0.775222	-0.36731896	0.425	0.23	down	no
Manes.01G013	6.655570035	4.934542	1.348772	0.43164604	0.607	0.96	down	no
Manes.01G015	3.947203562	8.091833	0.487801	-1.03563561	0.194	0.63	up	no
Manes.01G015	1.366317941	7.1591876	0.190848	-2.38950264	0.016	0.46	down	no
Manes.01G015	4.352059455	1.2234623	3.557167	1.83072859	0.202	1	up	no
Manes.01G019	0	1.8755279	#NAME?	#NAME?	0.094	0.23	down	no
Manes.01G020	127.2129301	156.78867	0.811366	-0.30157598	0.17	0.95	up	no
Manes.01G021	2.479903656	1.5535388	1.596293	0.67472579	0.815	1	up	no
Manes.01G024	1.441729054	3.4243065	0.421028	-1.24801176	0.355	0.66	up	no
Manes.01G025	31.74806899	41.030101	0.773775	-0.37001385	0.309	0	down	yes
Manes.01G030	47.7786493	14.062057	3.3977	1.76455837	0.001	0.78	up	no
Manes.01G032	5.999283358	5.8909888	1.018383	0.02628036	0.98	0.75	up	no
Manes.01G036	62.25042028	19.328109	3.22072	1.68738307	#####	0	up	yes
Manes.01G039	247.7836781	180.40424	1.373491	0.45784791	0.014	0.48	up	no
Manes.01G039	3.163062627	4.9752348	0.635761	-0.65344248	0.538	0.22	down	no
Manes.01G039	0.658889561	1.2546345	0.525165	-0.92915857	0.939	1	down	no
Manes.01G040	101.6693735	534.46709	0.190226	-2.39421596	#####	0.31	down	no
Manes.01G041	231.707801	249.35857	0.929215	-0.1059152	0.62	0.65	up	no
Manes.01G041	2.27571025	2.1663449	1.050484	0.07105395	1	0.96	down	no
Manes.01G042	30.78009973	30.857096	0.997505	-0.0036044	1	1	down	no
Manes.01G042	65.44723582	59.165664	1.106169	0.14557209	0.606	0.04	down	no
Manes.01G042	1.367619383	0.3124686	4.376822	2.12988386	0.353	1	down	no

Manes.01G042	9.790458926	25.488007	0.38412	-1.3803702	0.008	0.13	up	no
Manes.01G045	6.522883414	0.6249371	10.43766	3.38372692	0.074	0.71	down	no
Manes.01G046	59.65084442	121.93584	0.489199	-1.03150775	#####	0.04	down	no
Manes.01G046	868.2158521	1109.2213	0.782726	-0.35342154	0.127	0.06	up	no
Manes.01G047	11.63574243	12.056364	0.965112	-0.05123161	0.976	0.13	up	no
Manes.01G049	65.71885046	18.931645	3.471376	1.7955075	#####	0.26	up	no
Manes.01G049	83.90944677	64.316773	1.304628	0.38363822	0.221	0.15	down	no
Manes.01G052	14.29464205	18.995883	0.752513	-0.41021227	0.46	0	up	yes
Manes.01G052	17.60462257	66.268701	0.265655	-1.91237333	#####	0.78	down	no
Manes.01G053	499.4958418	398.90401	1.252171	0.32443105	0.076	0.99	up	no
Manes.01G053	30.96560291	19.594408	1.580329	0.66022454	0.104	0.94	down	no
Manes.01G054	6.046520735	17.363825	0.348225	-1.52190765	0.008	0.82	up	no
Manes.01G054	37.85223864	29.525837	1.282004	0.35840069	0.328	0.03	up	no
Manes.01G055	254.9463983	84.751057	3.00818	1.58889069	#####	0	up	yes
Manes.01G055	98.96714087	47.892278	2.066453	1.04715654	1E-04	0	up	yes
Manes.01G055	1853.273123	588.74775	3.147822	1.65435396	#####	0	up	no
Manes.01G064	28.81254115	5.2382066	5.500459	2.45955203	2E-04	0.5	up	no
Manes.01G066	25.71065835	56.550287	0.454651	-1.13716778	0.006	0.33	down	no
Manes.01G071	14.9470244	25.162691	0.594015	-0.75142792	0.093	0.02	down	yes
Manes.01G077	16.80234604	8.0837456	2.078535	1.05556686	0.044	0.28	up	no
Manes.01G080	147.3717582	78.613015	1.874648	0.90661999	3E-04	0.01	down	yes
Manes.01G081	134.3652279	141.67135	0.948429	-0.07638815	0.741	0	up	yes
Manes.01G082	45.24303739	37.839488	1.195657	0.25780322	0.438	0.01	down	yes
Manes.01G082	15.33281665	8.6361012	1.775433	0.82817069	0.241	0.24	down	no
Manes.01G084	23.88313705	7.4661792	3.198843	1.6775503	6E-04	0.07	up	no
Manes.01G085	11.69301794	7.4390508	1.571843	0.65245689	0.267	0.15	down	no
Manes.01G086	169.9770995	81.997601	2.072952	1.05168678	#####	0	down	yes
Manes.01G087	15.96966628	36.070324	0.442737	-1.17547822	0.007	0.18	up	no
Manes.01G089	112.9599572	25.24075	4.475301	2.16198479	#####	0	up	yes
Manes.01G091	2.477300773	0.9381223	2.640701	1.40092118	0.33	0.46	up	no
Manes.01G092	8.227382825	22.04681	0.373178	-1.42206442	0.027	0.14	down	no
Manes.01G093	5.009647574	6.8053097	0.736138	-0.4419517	0.598	0.58	down	no
Manes.01G094	3.844920156	5.5913679	0.687653	-0.54024764	0.597	0.36	down	no
Manes.01G094	103.8431508	48.780187	2.128798	1.09003887	#####	0	up	yes
Manes.01G094	16.9210902	19.561342	0.865027	-0.20918283	0.691	0.54	down	no
Manes.01G096	129.622713	237.81556	0.545056	-0.87552459	2E-04	0.02	down	no
Manes.01G096	8.827695436	5.9078802	1.494224	0.5793963	0.397	0.95	up	no
Manes.01G101	8.291454365	4.3726659	1.896201	0.92311207	0.218	0.29	up	no
Manes.01G101	7.25848553	11.186063	0.648887	-0.62396188	0.355	0.51	down	no
Manes.01G102	76.09790902	319.10404	0.238474	-2.06809815	#####	0	down	yes
Manes.01G102	40.36581062	123.02194	0.328119	-1.60770985	#####	0.04	down	no
Manes.01G102	14.31500713	122.6933	0.116673	-3.09945613	#####	0	down	yes
Manes.01G105	6.42840866	56.253767	0.114275	-3.12941616	5E-04	0	up	yes
Manes.01G106	1.721333573	1.8531597	0.928864	-0.10646052	1	1	up	no
Manes.01G107	1334.37354	644.92433	2.069039	1.04896078	#####	0	down	no
Manes.01G108	3.821952188	2.5099856	1.522699	0.60663065	0.554	0.02	up	yes
Manes.01G108	1.088014864	2.442881	0.445382	-1.1668853	0.45	0.36	down	no
Manes.01G109	0.329444781	4.6803741	0.070389	-3.82851529	0.012	0.63	down	no

Manes.01G110	43.48832431	41.639324	1.044405	0.06268148	0.848	0.01	up	no
Manes.01G110	512.4198312	205.89818	2.488705	1.3153952	#####	0	up	yes
Manes.01G110	8.624803471	6.2203487	1.386547	0.47149612	0.521	0.42	up	no
Manes.01G113	115.4790974	68.548606	1.684631	0.75243251	0.099	0	up	yes
Manes.01G114	335.5621562	553.94459	0.605768	-0.72316166	#####	0.88	down	no
Manes.01G117	20.44344619	17.684381	1.156017	0.2091627	0.675	0.05	down	yes
Manes.01G119	113.288921	106.56033	1.063143	0.08833627	0.705	0.01	down	no
Manes.01G119	39.01398977	39.151706	0.996482	-0.00508365	1	0	down	yes
Manes.01G122	1.012603751	1.5359309	0.659277	-0.60104356	0.922	0.53	up	no
Manes.01G123	12.04189977	13.638464	0.882937	-0.17961821	0.78	0.27	down	no
Manes.01G124	6.876597604	17.992806	0.382186	-1.38765335	0.021	0.7	up	no
Manes.01G125	5.487311696	6.81007	0.805764	-0.31157009	0.761	0.97	down	no
Manes.01G127	66.719079	49.127155	1.35809	0.44157869	0.129	0.66	up	no
Manes.01G131	52.41216935	56.829894	0.922264	-0.1167482	0.709	1	up	no
Manes.01G134	108.074152	123.80113	0.872966	-0.19600296	0.409	0.89	down	no
Manes.01G135	6919.954302	5467.2735	1.265705	0.33994096	0.065	0	up	no
Manes.01G136	236.0586666	270.86895	0.871487	-0.19844956	0.273	0.84	up	no
Manes.01G136	26.68635168	26.422803	1.009974	0.01431859	1	0.13	down	no
Manes.01G137	11.12507221	2.8000859	3.973118	1.99027169	0.005	0	up	yes
Manes.01G144	7.660738539	2.4780968	3.09138	1.62825092	0.053	1	down	no
Manes.01G147	13.76843911	49.754958	0.276725	-1.85347529	5E-04	0	down	yes
Manes.01G149	140.0497558	138.25464	1.012984	0.01861162	0.94	0.01	down	no
Manes.01G150	4.375027422	4.6484854	0.941173	-0.08746866	1	0.01	up	yes
Manes.01G154	12.19272199	8.083029	1.508435	0.59305231	0.331	0.62	down	no
Manes.01G158	3.796381337	3.724644	1.01926	0.02752239	1	0.49	down	no
Manes.01G158	11.89498187	20.113698	0.591387	-0.75782525	0.135	0.13	down	no
Manes.01G159	3.92163271	1.5590157	2.515454	1.33081898	0.256	0.86	down	no
Manes.01G159	4.654631942	0.6208934	7.496668	2.90624953	0.018	0.64	up	no
Manes.01G160	99.35125828	24.573687	4.042994	2.01542393	#####	0	up	yes
Manes.01G162	17.82657818	15.223175	1.171016	0.22776048	0.671	0.74	up	no
Manes.01G163	252.784131	734.12253	0.344335	-1.53811497	#####	0.16	up	no
Manes.01G163	4.150095527	0.924558	4.488735	2.16630879	0.06	0	up	yes
Manes.01G167	1.417459644	9.9640338	0.142258	-2.81342225	0.002	0.61	down	no
Manes.01G168	17.05117394	7.7746041	2.193189	1.13302995	0.03	0.02	up	yes
Manes.01G169	5.667235693	9.9449927	0.569858	-0.8113251	0.241	0.82	down	no
Manes.01G170	21.96281583	45.2488	0.485379	-1.04281649	0.01	0	down	yes
Manes.01G174	8958.902455	4751.8124	1.885365	0.91484413	#####	0.8	down	no
Manes.01G174	4879.992838	3826.2356	1.275403	0.35095331	0.01	0.41	up	no
Manes.01G174	9.865870039	9.3200556	1.058563	0.08210773	0.933	0.19	up	no
Manes.01G177	8887.753343	9112.0184	0.975388	-0.03595188	0.808	0	up	no
Manes.01G178	8.425442427	51.865122	0.162449	-2.62194034	#####	0	up	yes
Manes.01G179	23.1527407	42.547451	0.544163	-0.87788973	0.02	0.01	down	yes
Manes.01G180	212.818055	193.31588	1.100882	0.13866042	0.45	0.12	up	no
Manes.01G181	388.7587323	445.599	0.872441	-0.19687092	0.195	1	down	no
Manes.01G185	28.69770131	30.711473	0.934429	-0.0978425	0.895	0.54	up	no
Manes.01G188	16.82140968	7.7881684	2.159867	1.11094263	0.035	0.51	up	no
Manes.01G188	10.35487373	53.910822	0.192074	-2.38026494	#####	0	down	yes
Manes.01G190	397.6846256	569.42828	0.698393	-0.51788936	0.005	0	down	yes

Manes.01G191	3.491205966	8.4131055	0.414972	-1.26891296	0.112	0.13	down		no
Manes.01G193	137.8865713	278.29031	0.495477	-1.01310871	#####	0.79	down		no
Manes.01G193	2.076349205	4.018788	0.516661	-0.95271137	0.442	0.33	down		no
Manes.01G194	121.6382055	214.78453	0.566327	-0.82029365	0.021	0.88	down		no
Manes.01G196	325.4327074	265.51469	1.225667	0.29356755	0.554	0.08	up		no
Manes.01G198	19.75731093	91.672998	0.215519	-2.21411026	#####	0	up		yes
Manes.01G198	18.06898345	17.445927	1.035714	0.05062508	0.928	0	up		yes
Manes.01G199	1.721333573	0	Inf	Inf	0.074	0.58	up		no
Manes.01G202	0	1.5318872	#NAME?	#NAME?	0.156	1	up		no
Manes.01G203	7.336126121	11.417863	0.642513	-0.63820226	0.369	1	down		no
Manes.01G205	66.13039479	25.468506	2.596556	1.3765992	#####	0.68	down		no
Manes.01G205	6.877899046	3.4162191	2.013307	1.00956744	0.22	0.01	up		yes
Manes.01G208	187.6962731	152.74648	1.228809	0.29726088	0.206	0	up		yes
Manes.01G209	3.821952188	8.3419572	0.45816	-1.12607617	0.151	0.86	up		no
Manes.01G209	817.2627217	824.82229	0.990835	-0.01328339	0.971	0	up		yes
Manes.01G212	15.88942281	18.587287	0.854854	-0.22624952	0.663	0	up		yes
Manes.01G213	25.78309318	95.587	0.269734	-1.89038906	#####	0.01	down		yes
Manes.01G215	146.6751038	166.18793	0.882586	-0.18019158	0.416	0.02	down		no
Manes.01G216	126.6912937	311.91688	0.40617	-1.29984425	#####	0.02	down		no
Manes.01G218	64.24047679	141.78846	0.453073	-1.14218559	#####	0.94	up		no
Manes.01G218	12.11600944	8.3636088	1.448658	0.53471712	0.38	0.14	up		no
Manes.01G219	2.478602214	21.774317	0.113831	-3.13502882	#####	0.36	up		no
Manes.01G220	37.08585991	40.590764	0.913653	-0.13028226	0.749	0.1	down		no
Manes.01G221	17.35634931	20.180086	0.860073	-0.21746877	0.706	0.16	up		no
Manes.01G222	163.0242594	158.13704	1.030905	0.0439113	0.831	0	up		yes
Manes.01G225	5.337790913	6.233913	0.85625	-0.22389522	0.856	0.44	down		no
Manes.01G225	7.234216121	8.7534191	0.826445	-0.27500995	0.749	0.36	down		no
Manes.01G226	57.37588099	30.160294	1.902365	0.9277939	0.005	0.5	down		no
Manes.01G228	1.41485676	0.6073292	2.329637	1.22010541	0.695	1	down		no
Manes.01G230	7.666872344	7.1544273	1.071626	0.09980188	0.91	0.88	up		no
Manes.01G230	1.366317941	0	Inf	Inf	0.131	0.34	up		no
Manes.01G232	20.66410035	11.445708	1.805402	0.85231989	0.081	0.6	down		no
Manes.01G233	92.58764444	81.674865	1.133612	0.18092751	0.526	0.12	down		no
Manes.01G237	13.1590164	9.2896	1.416532	0.50236328	0.394	0	up		yes
Manes.01G238	20.64373527	35.05962	0.588818	-0.76410634	0.051	0	up		yes
Manes.01G238	116.270577	61.854625	1.879739	0.91053267	6E-04	0.77	down		no
Manes.01G240	183.1602777	119.09265	1.537965	0.62102223	0.003	0.08	up		no
Manes.01G241	5.667235693	2.4957048	2.270796	1.18319793	0.376	0.12	up		no
Manes.01G241	1.012603751	0.6208934	1.630882	0.70565217	0.868	1	down		no
Manes.01G242	368.3515343	246.21196	1.496075	0.5811824	5E-04	0	down		yes
Manes.01G243	200.9746729	185.4713	1.083589	0.11581773	0.537	0.86	down		no
Manes.01G244	127.1356629	201.40843	0.631233	-0.66375527	0.001	0.06	down		no
Manes.01G244	552.9071966	376.1094	1.47007	0.555885	0.002	0.01	up		no
Manes.01G245	0.35371419	2.1751488	0.162616	-2.62045812	0.193	0.61	down		no
Manes.01G245	125.5875649	74.826743	1.678378	0.74706773	0.003	0.29	down		no
Manes.01G247	23.56309118	12.486867	1.88703	0.91611727	0.067	0.33	down		no
Manes.01G248	95.43668741	293.86337	0.324766	-1.62252966	#####	0	down		yes
Manes.01G253	114.2404295	123.1074	0.927974	-0.10784416	0.671	0.88	down		no

Manes.01G256	3.995742381	4.6939384	0.851256	-0.23233534	0.879	0.91	up	no
Manes.01G257	16.97613623	16.473766	1.030495	0.04333772	0.965	0.26	down	no
Manes.01G258	78.473939	86.728905	0.904819	-0.14429928	0.592	0.03	down	no
Manes.01G258	126.9045972	165.87882	0.765044	-0.38638534	0.07	0.61	down	no
Manes.01G260	549.4854843	491.50764	1.117959	0.16086754	0.245	0.36	down	no
Manes.01G264	29.75624099	3.085426	9.644127	3.26965069	#####	0	up	yes
Manes.01G267	4.251077491	2.4557286	1.731086	0.79167742	0.565	0.76	up	no
Manes.01G268	0.708729822	1.2410703	0.571063	-0.80827713	0.844	0.02	up	yes
Manes.01G268	1.697064164	8.6848813	0.195404	-2.35546502	0.039	0	up	yes
Manes.01G275	2.478602214	0.3172288	7.813294	2.96593094	0.088	0.68	up	no
Manes.02G002	3.693169894	7.1368194	0.517481	-0.95042163	0.443	0.04	down	yes
Manes.02G004	293.9819591	204.08672	1.440476	0.52654531	0.01	0.3	up	no
Manes.02G005	211.6182842	184.02748	1.149928	0.20154303	0.316	0.56	down	no
Manes.02G006	1.088014864	4.0154609	0.270956	-1.88386732	0.152	1	down	no
Manes.02G006	156.1240316	281.56912	0.554479	-0.85079652	0.006	0.93	up	no
Manes.02G008	200.1887604	285.93813	0.700112	-0.51434202	0.011	0.01	down	no
Manes.02G009	112.1479084	185.894	0.60329	-0.72907752	1E-03	0	down	yes
Manes.02G009	213.8239704	140.27728	1.524295	0.60814223	0.002	0.11	up	no
Manes.02G009	23.14270257	20.896645	1.107484	0.14728599	0.76	0.28	down	no
Manes.02G010	0	1.8802882	#NAME?	#NAME?	0.094	1	up	no
Manes.02G010	9.108601398	5.2334463	1.740459	0.79946824	0.345	0.73	down	no
Manes.02G011	29.65395759	69.193505	0.428566	-1.22241197	2E-04	0	down	yes
Manes.02G011	131.4429803	131.73268	0.997801	-0.0031762	0.995	0.36	down	no
Manes.02G013	723.1517078	798.64213	0.905477	-0.14325084	0.489	0	down	yes
Manes.02G013	1542.841876	74.752494	20.63934	4.36732469	#####	0	up	yes
Manes.02G014	63.21783491	55.164023	1.145998	0.19660395	0.503	0.68	down	no
Manes.02G017	358.9388162	275.92061	1.300877	0.37948471	0.058	0.02	up	no
Manes.02G018	246.3533852	86.841924	2.836802	1.50426571	#####	0.19	down	no
Manes.02G018	1.342048532	6.2929302	0.213263	-2.2292951	0.08	0.22	down	no
Manes.02G023	6.350394664	10.290067	0.617138	-0.69633416	0.311	0.23	down	no
Manes.02G024	51.62319605	7.4804601	6.901072	2.78682055	#####	0	up	yes
Manes.02G027	18.56478318	13.075872	1.419774	0.50566134	0.311	0.91	down	no
Manes.02G029	32.54745383	34.083672	0.954928	-0.06653608	0.941	0.27	up	no
Manes.02G029	3.917728384	2.4692929	1.586579	0.66591943	0.531	0.69	down	no
Manes.02G031	155.040379	142.74822	1.086111	0.11917122	0.678	0.82	up	no
Manes.02G034	2.857887256	4.0363959	0.708029	-0.49811869	0.693	1	down	no
Manes.02G035	2.403191102	12.433327	0.193286	-2.37118907	0.006	0.02	down	yes
Manes.02G036	5.870127658	18.350011	0.319898	-1.64431713	0.005	0	up	yes
Manes.02G037	58.29169592	92.814042	0.628048	-0.67105272	0.03	0	down	yes
Manes.02G038	13.76676426	30.506801	0.451269	-1.14794141	0.029	0.85	down	no
Manes.02G038	55.52669315	30.841638	1.800381	0.84830209	0.01	0	up	yes
Manes.02G038	1.086713422	0	Inf	Inf	0.238	0	up	yes
Manes.02G039	262.7052162	315.40351	0.832918	-0.26375388	0.13	0.11	down	no
Manes.02G040	116.9818251	155.20794	0.75371	-0.40791796	0.072	0.02	up	no
Manes.02G041	108.1333912	99.19839	1.090072	0.12442348	0.716	1	down	no
Manes.02G043	180.4060829	74.292456	2.428323	1.27996035	#####	0	up	yes
Manes.02G043	2.756905292	4.3048446	0.640419	-0.64291147	0.662	0.24	up	no
Manes.02G045	2.857887256	7.792212	0.366762	-1.44708393	0.081	0.03	down	yes

Manes.02G047	2.453031363	3.4345436	0.714223	-0.48555272	0.771	0.57	down	no
Manes.02G047	8.87660766	6.5559021	1.353987	0.43721413	0.512	0.68	up	no
Manes.02G048	0.329444781	1.866724	0.176483	-2.50240003	0.273	0.88	up	no
Manes.02G050	11.46325368	12.132989	0.9448	-0.08191843	0.986	0.54	up	no
Manes.02G050	1.038174603	0.9197977	1.128699	0.17466055	1	0	up	yes
Manes.02G051	277.0805718	179.61413	1.542644	0.62540474	6E-04	0	up	yes
Manes.02G052	2.174728285	2.8176939	0.771811	-0.37367972	0.872	0.77	down	no
Manes.02G053	504.1993181	153.27987	3.289403	1.71782588	#####	0.12	down	no
Manes.02G054	13.61631544	132.96389	0.102406	-3.28762623	#####	0	up	yes
Manes.02G055	43.215227	59.948898	0.720868	-0.47219349	0.174	0.02	down	yes
Manes.02G056	1224.434062	1641.5005	0.745924	-0.42290008	0.009	0.32	up	no
Manes.02G056	15.86162248	17.701989	0.896036	-0.15837111	0.839	0.21	down	no
Manes.02G057	128.421364	121.87227	1.053737	0.07551537	0.755	0.61	up	no
Manes.02G057	218.6227481	310.06703	0.705082	-0.5041366	0.028	0.5	up	no
Manes.02G058	1.870854356	0.3172288	5.897491	2.56010143	0.209	0.34	up	no
Manes.02G059	355.8168847	715.13816	0.49755	-1.00708702	#####	0.62	down	no
Manes.02G060	13.83864445	9.9823583	1.38631	0.47125004	0.395	0.26	down	no
Manes.02G060	7.535487166	8.7669834	0.85953	-0.21837973	0.801	0.88	down	no
Manes.02G061	159.8915033	63.715156	2.509474	1.32738478	#####	0.87	up	no
Manes.02G062	7.662039981	11.218668	0.682972	-0.55010097	0.459	0	up	yes
Manes.02G062	17.22756701	30.157684	0.57125	-0.80780666	0.059	0.3	up	no
Manes.02G062	1.745602983	2.1568243	0.809339	-0.30518319	0.991	0.18	up	no
Manes.02G063	12.01632891	13.072545	0.919204	-0.12154378	0.886	0.65	up	no
Manes.02G064	8.705046947	1.8802882	4.629634	2.21089827	0.006	0	up	yes
Manes.02G064	377.5341608	302.73912	1.247061	0.31853206	0.047	0.31	down	no
Manes.02G067	239.0403962	135.4553	1.764718	0.81943764	#####	0	down	yes
Manes.02G068	69.61676839	62.905305	1.106692	0.14625315	0.61	0.17	down	no
Manes.02G071	6.802487934	6.8514793	0.99285	-0.01035302	1	0.39	up	no
Manes.02G072	40.15566464	70.262254	0.571511	-0.80714634	0.034	0	up	yes
Manes.02G072	7.085996779	5.9342921	1.194076	0.25589488	0.822	0.03	up	yes
Manes.02G072	2.098015731	0.3036646	6.90899	2.7884749	0.142	0.49	up	no
Manes.02G073	727.6794814	643.81731	1.130258	0.17665175	0.205	0.6	up	no
Manes.02G074	1926.987804	0.3036646	6345.777	12.6315811	0	0.62	up	no
Manes.02G078	6.829360227	0	Inf	Inf	#####	0	up	yes
Manes.02G081	72.88992308	42.062917	1.732878	0.79317048	0.007	0	up	yes
Manes.02G090	2814.258154	2398.2168	1.173479	0.23079259	0.468	0	down	yes
Manes.02G093	82.57326623	52.119034	1.584321	0.66386443	0.054	0.03	down	yes
Manes.02G093	426.7876298	211.92571	2.013855	1.00995976	#####	0	up	yes
Manes.02G094	50.08661884	60.30943	0.830494	-0.26795837	0.392	0.32	down	no
Manes.02G094	1.012603751	0.9197977	1.100898	0.13868118	1	1	up	no
Manes.02G096	5.23811039	8.3324366	0.628641	-0.66969197	0.401	0.15	down	no
Manes.02G096	21.3494888	6.2712786	3.404328	1.76737001	0.014	0.01	up	yes
Manes.02G099	5.664632809	9.2929271	0.609564	-0.71415064	0.313	0.76	down	no
Manes.02G101	8.753585766	4.0601973	2.155951	1.10832427	0.117	0.58	up	no
Manes.02G101	384.6846025	411.56168	0.934695	-0.09743258	0.527	0	down	yes
Manes.02G104	6.373362631	9.0130639	0.707125	-0.49996287	0.495	0.35	down	no
Manes.02G106	622.8295489	615.10176	1.012563	0.01801229	0.889	0.3	up	no
Manes.02G107	3.311281968	0	Inf	Inf	0.008	1	up	no

Manes.02G108	1.440427612	2.1703886	0.663673	-0.59145618	0.807	1	up	no
Manes.02G121	384.5477227	1521.8794	0.252679	-1.98461951	#####	0.99	down	no
Manes.02G121	11.81213551	13.38834	0.88227	-0.18070731	0.772	0	up	yes
Manes.02G126	11.41341342	33.355667	0.342173	-1.54720158	8E-04	0	down	yes
Manes.02G128	40.56702774	35.755706	1.134561	0.18213412	0.63	0.02	down	yes
Manes.02G130	27.04880256	44.766159	0.604224	-0.72684382	0.046	0	down	yes
Manes.02G130	277.9285921	29.925884	9.287231	3.21524849	#####	0.52	down	no
Manes.02G130	20.36896311	17.799088	1.144382	0.19456919	0.75	0.43	down	no
Manes.02G132	1.697064164	31.29715	0.054224	-4.20491827	#####	0.44	down	no
Manes.02G132	17.45863271	1.5542554	11.2328	3.48964508	#####	0.73	up	no
Manes.02G138	95.95776916	48.147162	1.99301	0.99494884	3E-04	0.03	down	yes
Manes.02G138	8.606667867	14.256747	0.603691	-0.72811817	0.369	0.74	up	no
Manes.02G140	9.861037676	8.7310509	1.129422	0.17558416	0.811	0.31	up	no
Manes.02G140	70.9944259	59.087861	1.201506	0.26484397	0.343	0	down	yes
Manes.02G143	3.844920156	5.2767496	0.728653	-0.45669588	0.656	0.07	down	no
Manes.02G149	35.71954196	83.686066	0.426828	-1.22827385	#####	0.54	down	no
Manes.02G149	5.056884951	4.375993	1.155597	0.20863854	0.912	1	down	no
Manes.02G149	1.517140166	0	Inf	Inf	0.113	1	up	no
Manes.02G152	68.44784782	74.591873	0.917631	-0.12401326	0.713	0.17	down	no
Manes.02G152	41.88350542	28.328325	1.478503	0.56413687	0.115	0	up	yes
Manes.02G153	34.30598665	21.219351	1.616731	0.69307982	0.091	0.54	down	no
Manes.02G156	377.0058129	278.27181	1.354811	0.438092	0.007	0.66	up	no
Manes.02G156	12.7298911	2.7729574	4.590727	2.19872269	0.001	0.97	up	no
Manes.02G157	6.045219293	10.86479	0.556405	-0.84579376	0.199	0.37	up	no
Manes.02G157	17.29647091	2.2110813	7.82263	2.96765374	0.009	0	up	yes
Manes.02G160	1.695762722	6.2746057	0.270258	-1.88759048	0.073	0.02	up	yes
Manes.02G160	335.9665311	227.18109	1.478849	0.56447478	0.001	0.38	down	no
Manes.02G161	28.77013613	65.131845	0.441721	-1.17879104	4E-04	0.72	up	no
Manes.02G162	4.806382204	7.7163035	0.622887	-0.6829585	0.524	0.87	up	no
Manes.02G163	12.42118481	0.9469262	13.11737	3.71340695	#####	0	up	yes
Manes.02G163	30.95388993	137.43959	0.225218	-2.15060505	#####	0	down	yes
Manes.02G163	44.97338641	36.962993	1.216714	0.28298992	0.396	0.02	down	yes
Manes.02G163	1435.363006	467.7456	3.068683	1.61761965	#####	0.34	up	no
Manes.02G164	15.35931554	9.940949	1.545055	0.62765844	0.226	0.25	up	no
Manes.02G167	68.69808476	55.233513	1.243775	0.314726	0.377	0.01	down	yes
Manes.02G169	5.413202025	0.933362	5.799681	2.53597362	0.019	0.36	up	no
Manes.02G170	1.038174603	0.3036646	3.41882	1.77349851	0.561	1	down	no
Manes.02G173	340.1821578	266.86347	1.274742	0.35020565	0.036	0	down	no
Manes.02G179	41.00683039	48.465824	0.846098	-0.24110356	0.63	0.19	down	no
Manes.02G179	113.7626808	73.41458	1.549592	0.63188886	0.023	0.05	up	no
Manes.02G183	1.896425208	0	Inf	Inf	0.064	0.14	up	no
Manes.02G184	0.329444781	1.85792	0.177319	-2.4955798	0.273	0.22	down	no
Manes.02G188	4.603490239	6.2170216	0.740466	-0.43349551	0.649	0.82	down	no
Manes.02G188	274.5457912	387.464	0.708571	-0.49701549	0.003	0	up	no
Manes.02G189	2.859188698	1.5542554	1.839587	0.87938224	0.509	0.86	down	no
Manes.02G189	12.52699913	8.3657586	1.497413	0.58247259	0.449	0.89	down	no
Manes.02G189	1.769872392	3.4616721	0.511277	-0.96782373	0.48	0.34	up	no
Manes.02G190	201.3628022	244.12625	0.824831	-0.27783026	0.132	0.44	down	no

Manes.02G194	1179.651568	1139.7977	1.034966	0.04958305	0.686	0.09	up	no
Manes.02G195	21.83496158	12.774357	1.709281	0.77338933	0.185	0.04	up	yes
Manes.02G196	3.163062627	2.4740531	1.278494	0.35444563	0.811	0.68	down	no
Manes.02G197	3.21420433	2.4916611	1.289985	0.36735381	0.79	0.51	down	no
Manes.02G202	81.02506063	104.57082	0.774834	-0.36804017	0.192	0.01	down	no
Manes.02G206	131.7627834	178.03249	0.740105	-0.43419758	0.073	0	down	yes
Manes.02G206	3.184729153	6.8107866	0.467601	-1.09665074	0.348	0.59	down	no
Manes.02G209	52.22284643	106.42041	0.490722	-1.02702191	2E-04	0	down	yes
Manes.02G209	48.35952486	52.758282	0.916624	-0.12559752	0.686	0.41	up	no
Manes.02G210	169.2215903	170.83977	0.990528	-0.01373023	0.933	0.39	up	no
Manes.02G213	1.062444012	0.3124686	3.400163	1.7656039	0.559	1	down	no
Manes.02G214	10.3448356	5.582564	1.853062	0.88991094	0.177	0.61	up	no
Manes.02G218	55.61084096	52.741852	1.054397	0.07641794	0.826	0.33	down	no
Manes.02G219	42.10424417	85.947135	0.489885	-1.02948388	5E-04	0.08	down	no
Manes.02G219	173.3594918	149.57304	1.159029	0.21291664	0.281	0.4	up	no
Manes.02G221	144.621168	179.52348	0.805584	-0.31189378	0.132	0	down	yes
Manes.02G221	1.038174603	0.9109938	1.139607	0.18853601	1	0.54	down	no
Manes.02G221	242.7696791	102.09888	2.37779	1.24962118	#####	0	up	yes
Manes.02G222	4.349456571	1.5542554	2.798418	1.48461156	0.168	0.64	up	no
Manes.02G223	186.3288349	222.11829	0.838872	-0.25347727	0.203	0.6	up	no
Manes.02G224	28.05880342	15.890955	1.765709	0.82024765	0.048	0.09	up	no
Manes.02G224	65.25281473	106.78187	0.611085	-0.71055463	0.022	0.01	up	no
Manes.02G224	6.372061189	11.455228	0.556258	-0.84617418	0.187	0.42	down	no
Manes.02G225	1.467299905	0.6161331	2.381466	1.25184972	0.589	0.92	up	no
Manes.03G002	269.6794691	72.818409	3.703452	1.88887056	#####	1	down	no
Manes.03G005	53.36851021	47.513882	1.123219	0.16763965	0.6	0.86	up	no
Manes.03G006	192.6174105	153.40001	1.255655	0.32843955	0.142	0.14	up	no
Manes.03G006	120.0526425	151.56302	0.792097	-0.33625066	0.124	0.44	down	no
Manes.03G008	3.566617078	2.1703886	1.643308	0.71660299	0.54	0.75	up	no
Manes.03G010	28.93649108	59.150922	0.489198	-1.03151068	0.002	0.1	down	no
Manes.03G014	0.70742838	6.5742266	0.107606	-3.21616518	0.051	0.02	up	yes
Manes.03G016	13.74026537	10.325999	1.330648	0.4121285	0.46	0.3	up	no
Manes.03G019	61.73306163	323.73449	0.19069	-2.39069581	#####	0	up	no
Manes.03G019	6664.193724	1926.3736	3.45945	1.7905428	#####	0.86	down	no
Manes.03G020	1.491569315	0	Inf	Inf	0.128	0.09	up	no
Manes.03G021	8.477885572	17.109657	0.495503	-1.01303445	0.076	0.9	up	no
Manes.03G022	19.60165634	35.458235	0.55281	-0.85514515	0.03	0.82	up	no
Manes.03G026	1.036873161	0	Inf	Inf	0.24	1	up	no
Manes.03G026	154.3925632	131.21495	1.176639	0.23467121	0.28	0.11	up	no
Manes.03G026	5.00704469	2.5195062	1.987312	0.99081835	0.501	0.98	up	no
Manes.03G027	1.671493312	2.4740531	0.675609	-0.56573891	0.889	0.3	down	no
Manes.03G027	1.695762722	8.6951184	0.195025	-2.35827136	0.009	0.01	down	yes
Manes.03G028	4251.853628	1677.6496	2.534411	1.34165054	#####	0.35	down	no
Manes.03G028	185.6352524	143.52594	1.293392	0.37115922	0.077	0	down	yes
Manes.03G030	0	2.152064	#NAME?	#NAME?	0.064	0.8	down	no
Manes.03G031	5.740043922	15.949972	0.359878	-1.47442024	0.025	0.09	down	no
Manes.03G031	155.4746485	102.37499	1.518678	0.60281608	0.025	0.55	down	no
Manes.03G033	6.400234925	4.9840387	1.284146	0.36080958	0.674	0.83	up	no

Manes.03G034	368.2688691	285.41577	1.290289	0.36769442	0.022	0.86	up	no
Manes.03G034	113.6181	240.22315	0.472969	-1.08018249	#####	0.69	down	no
Manes.03G040	1.41485676	0.6208934	2.278743	1.18823844	0.517	1	up	no
Manes.03G042	90.83710149	98.30573	0.924027	-0.11399384	0.68	0.21	down	no
Manes.03G043	86.90904616	99.795717	0.870869	-0.19947155	0.46	0.01	down	no
Manes.03G044	1.441729054	10.261505	0.140499	-2.83137036	0.001	0.19	down	no
Manes.03G044	127.676363	54.452224	2.344741	1.22942859	#####	0.39	up	no
Manes.03G046	4.480841749	20.701986	0.216445	-2.20792747	0.002	0	down	yes
Manes.03G047	2.455634247	6.8643269	0.357739	-1.48302257	0.115	0.48	up	no
Manes.03G049	9.436744736	0.9374057	10.06687	3.33154386	0.057	0.53	down	no
Manes.03G050	36.06414606	0.6208934	58.08428	5.8600758	#####	0.41	up	no
Manes.03G051	22.84235956	21.671997	1.054003	0.0758796	0.884	0.74	down	no
Manes.03G051	2.95886922	0	Inf	Inf	0.015	0.23	up	no
Manes.03G054	3.542347669	8.9797419	0.394482	-1.34196817	0.078	0.82	down	no
Manes.03G055	37.8925954	96.581559	0.392338	-1.34983179	#####	0.98	down	no
Manes.03G056	17.56221756	29.574156	0.593837	-0.75186197	0.08	0.81	up	no
Manes.03G059	0.35371419	1.2553511	0.281765	-1.82743491	0.544	0.75	down	no
Manes.03G059	18.37705053	68.662055	0.267645	-1.90160779	5E-04	0.1	down	no
Manes.03G060	0	2.1927568	#NAME?	#NAME?	0.062	0.94	down	no
Manes.03G060	33.1707344	25.577736	1.29686	0.37502237	0.319	0.83	up	no
Manes.03G063	319.4641546	315.95805	1.011097	0.01592105	0.941	0.74	down	no
Manes.03G063	51.21703871	299.85465	0.170806	-2.5495676	#####	0.53	up	no
Manes.03G063	212.2334673	473.49327	0.448229	-1.15769175	#####	0	down	yes
Manes.03G064	3.897363301	7.4085952	0.52606	-0.92670158	0.254	1	down	no
Manes.03G064	2.100618615	0.6073292	3.458781	1.79026366	0.5	0.13	up	no
Manes.03G065	167.3208994	172.68723	0.968925	-0.04554373	0.913	0.09	down	no
Manes.03G065	165.8510812	143.82914	1.153112	0.2055324	0.371	0.01	down	no
Manes.03G065	162.0673409	147.14066	1.101445	0.13939747	0.516	0.22	up	no
Manes.03G067	92.60511781	76.865042	1.204775	0.26876431	0.31	0.69	down	no
Manes.03G067	75.87361634	102.90735	0.7373	-0.43967582	0.1	0.09	down	no
Manes.03G068	73.9555246	53.892958	1.372267	0.45656115	0.1	0	up	yes
Manes.03G069	104.894544	111.0796	0.944319	-0.0826542	0.732	0.79	down	no
Manes.03G070	61.98420359	59.804882	1.036441	0.05163734	0.856	0.19	down	no
Manes.03G071	249.9075535	321.10556	0.778272	-0.36165316	0.037	0.04	up	no
Manes.03G072	18.9796772	46.885874	0.404806	-1.30469786	0.003	0.02	down	yes
Manes.03G072	518.8375165	546.86759	0.948744	-0.07590876	0.63	0.31	up	no
Manes.03G073	1.491569315	0	Inf	Inf	0.128	0.23	up	no
Manes.03G073	2.380223134	7.1998803	0.330592	-1.5968761	0.112	0.47	down	no
Manes.03G075	50.89428237	83.023016	0.613014	-0.70600776	0.131	0.01	up	yes
Manes.03G075	3.664996158	5.0111672	0.731366	-0.451335	0.719	0.17	down	no
Manes.03G076	109.6625101	252.25581	0.434727	-1.2018171	#####	0.19	down	no
Manes.03G079	1.137855125	0.9469262	1.20163	0.26499294	0.933	0.71	up	no
Manes.03G079	42.13586424	13.149886	3.204276	1.67999838	0.016	0.05	up	no
Manes.03G087	3.617758781	0.6073292	5.956833	2.57454563	0.238	0.08	up	no
Manes.03G087	109.6909727	99.663658	1.100612	0.13830537	0.585	0.02	down	no
Manes.03G088	2.579584179	0.3036646	8.494847	3.08658793	0.087	1	up	no
Manes.03G094	4.90866561	5.6313441	0.871669	-0.19814841	0.927	0.5	up	no
Manes.03G094	3.642028191	2.1663449	1.681186	0.74947916	0.605	1	down	no

Manes.03G095	7.006681341	1.2282226	5.704732	2.51215922	0.035	0.36	up	no
Manes.03G098	2.050778354	0	Inf	Inf	0.042	0.14	up	no
Manes.03G098	36.73697808	40.998673	0.896053	-0.15834425	0.694	0.06	up	no
Manes.03G099	2688.095065	5003.3291	0.537261	-0.89630419	#####	0	down	no
Manes.03G099	206.2441485	383.54004	0.537738	-0.89502399	0.065	0.18	down	no
Manes.03G099	390.3102755	633.38176	0.616232	-0.69845388	0.043	0.1	down	no
Manes.03G099	34.2985514	32.908273	1.042247	0.05969733	0.929	0.37	down	no
Manes.03G100	1.036873161	11.834801	0.087612	-3.51272417	1E-04	0.5	down	no
Manes.03G100	183.6975544	223.23634	0.822884	-0.28123951	0.144	0.06	down	no
Manes.03G101	94.46304233	120.31468	0.785133	-0.34899082	0.145	0	down	yes
Manes.03G103	0	4.055437	#NAME?	#NAME?	0.006	0.09	down	no
Manes.03G110	85.57740916	80.206069	1.066969	0.09351859	0.706	0.77	up	no
Manes.03G111	201.6830293	226.53593	0.890292	-0.16765021	0.396	0.06	down	no
Manes.03G112	139.0845706	184.17168	0.75519	-0.40508881	0.058	0	down	no
Manes.03G112	117.5775712	172.6603	0.680976	-0.55432349	0.011	0.11	down	no
Manes.03G112	1.744301541	4.6389648	0.376011	-1.41115344	0.359	1	down	no
Manes.03G114	134.744513	26.114378	5.159783	2.36731029	#####	0	up	yes
Manes.03G115	1.76857095	0.3036646	5.824093	2.54203351	0.202	1	down	no
Manes.03G116	407.1651504	363.16206	1.121167	0.16500059	0.288	0.84	up	no
Manes.03G117	694.110628	320.3792	2.166528	1.11538514	#####	0	up	yes
Manes.03G120	208.8832037	57.895571	3.607931	1.85117159	#####	0	down	yes
Manes.03G122	39.54502507	16.793606	2.354767	1.2355842	0.014	0.26	up	no
Manes.03G129	325.9039995	562.24191	0.579651	-0.78674395	#####	0	up	yes
Manes.03G130	371.0188202	164.36357	2.257306	1.17460183	#####	0.03	down	no
Manes.03G134	36.6055929	61.114029	0.598972	-0.73943951	0.023	0.23	up	no
Manes.03G137	107.8253241	105.59342	1.021137	0.03017618	0.906	0.57	up	no
Manes.03G137	2.025207502	0.6249371	3.240658	1.69628682	0.498	0.76	up	no
Manes.03G140	158.4154667	145.17706	1.091188	0.12589971	0.572	0	down	yes
Manes.03G140	23.09769467	22.812866	1.012485	0.01790116	0.959	0.17	down	no
Manes.03G141	118.9182107	76.482398	1.554844	0.63677	0.027	0	down	yes
Manes.03G142	998.7431428	705.81118	1.415029	0.50083142	1E-04	0.03	up	no
Manes.03G144	77.98883964	55.553833	1.403843	0.48938122	0.109	0.44	up	no
Manes.03G145	14.38651392	16.544915	0.869543	-0.2016708	0.754	0.57	up	no
Manes.03G145	2.60515503	1.5630594	1.666703	0.73699665	0.558	1	down	no
Manes.03G149	16.85478918	14.115137	1.194093	0.25591544	0.775	0.8	down	no
Manes.03G151	1.06114257	1.2593948	0.842581	-0.24711209	1	0.76	down	no
Manes.03G155	19.33339139	3.7294042	5.184043	2.37407765	#####	0.92	up	no
Manes.03G156	1694.471572	1540.2386	1.100136	0.13768158	0.488	0.89	down	no
Manes.03G160	147.3571535	140.85771	1.046142	0.0650786	0.796	0.97	down	no
Manes.03G162	10.32056619	1.2498742	8.257284	3.0456673	0.08	0.38	up	no
Manes.03G168	2.453031363	0.3036646	8.078095	3.01401509	0.089	0.01	up	yes
Manes.03G170	301.4678718	181.83398	1.657929	0.72938242	#####	0.99	down	no
Manes.03G172	55.43835221	78.565668	0.705631	-0.50301464	0.083	0.64	down	no
Manes.03G172	14.26516687	10.026378	1.422764	0.50869607	0.421	0.02	down	yes
Manes.03G174	7.105060421	6.8947825	1.030498	0.04334187	1	0.54	down	no
Manes.03G175	3.062080663	2.7817614	1.10077	0.1385136	0.944	0.5	up	no
Manes.03G177	28.51219814	219.15889	0.130098	-2.94232602	#####	0.65	down	no
Manes.03G178	7.437108085	5.2741391	1.410108	0.49580612	0.515	0.98	down	no

Manes.03G178	50.79144433	43.955366	1.155523	0.20854619	0.595	0.62	up	no
Manes.03G179	13.30723574	11.781261	1.129526	0.17571695	0.761	0.78	down	no
Manes.03G182	984.0087212	921.02536	1.068384	0.09543022	0.455	0	up	yes
Manes.03G188	71.38013357	107.166	0.666071	-0.58625269	0.024	0.58	up	no
Manes.03G189	109.7753017	97.820705	1.122209	0.16634173	0.461	0.55	up	no
Manes.03G190	105.1786921	122.44105	0.859015	-0.21924488	0.376	0.4	down	no
Manes.03G191	8.678174653	4.6532457	1.864972	0.89915427	0.189	0.06	up	no
Manes.03G191	77.81114512	201.58288	0.386001	-1.37332438	#####	0.05	down	no
Manes.03G196	15.61074632	1.2234623	12.75948	3.67349793	6E-04	0	up	yes
Manes.03G199	74.93541108	54.862969	1.365865	0.44981493	0.13	0.13	up	no
Manes.03G205	1.038174603	5.2877033	0.196338	-2.34859213	0.041	0.91	down	no
Manes.03G208	3.970171529	4.300801	0.923124	-0.11540403	0.894	0.96	up	no
Manes.03G208	102.2626013	126.41297	0.808957	-0.30586592	0.215	0	down	yes
Manes.03G209	196.2617528	129.40606	1.516635	0.60087385	0.004	0.82	down	no
Manes.03G210	451.8239625	374.6085	1.206123	0.27037717	0.077	0.62	up	no
Manes.03G210	9.786928006	1.8355518	5.331872	2.41464227	0.086	0.76	up	no
Manes.03G213	270.0824459	240.58294	1.122617	0.16686552	0.432	0	down	yes
Manes.04G000	22.71618015	28.235986	0.804512	-0.31381476	0.445	0.42	down	no
Manes.04G000	2048.68553	1146.7909	1.786451	0.83709614	#####	0	up	yes
Manes.04G003	45.95428551	78.338854	0.586609	-0.76952862	0.023	0.04	down	no
Manes.04G003	0	1.85792	#NAME?	#NAME?	0.094	0.22	down	no
Manes.04G005	54.28748266	38.022477	1.427773	0.51376706	0.1	0.27	down	no
Manes.04G007	1373.837179	1180.6821	1.163596	0.21859052	0.146	0.2	up	no
Manes.04G008	65.84856079	170.617	0.385944	-1.3735376	#####	0.69	down	no
Manes.04G009	38.08582264	19.235309	1.979995	0.98549703	0.011	0	up	yes
Manes.04G012	3.518078259	2.4564452	1.432183	0.51821552	0.831	0.89	up	no
Manes.04G012	132.7634699	146.66946	0.905188	-0.14371026	0.507	0.01	down	no
Manes.04G014	1.391888793	0.3124686	4.454492	2.15526103	0.352	0.68	up	no
Manes.04G015	1.417459644	2.7872383	0.508553	-0.97552869	0.525	0.1	down	no
Manes.04G015	21.62295952	120.02151	0.180159	-2.4726571	#####	0.01	up	no
Manes.04G016	1.845283505	0.3172288	5.816885	2.54024667	0.218	1	down	no
Manes.04G017	1.088014864	0.6073292	1.791475	0.84114767	1	0.72	down	no
Manes.04G021	19.25277451	29.20743	0.659174	-0.60126907	0.161	0.03	down	yes
Manes.04G021	58.73439027	55.475775	1.058739	0.08234756	0.793	0.02	down	yes
Manes.04G024	1.821014095	1.8938525	0.96154	-0.05658185	1	0.5	down	no
Manes.04G024	11.01376329	9.0090202	1.222526	0.28986539	0.69	0.56	up	no
Manes.04G024	302.6979722	244.40282	1.238521	0.30861807	0.074	0.18	up	no
Manes.04G026	96.2623899	300.93699	0.319876	-1.64441732	#####	0	down	yes
Manes.04G027	205.2069865	71.583307	2.866688	1.51938476	#####	0	up	yes
Manes.04G028	1.695762722	0.3036646	5.584328	2.48138372	0.224	1	down	no
Manes.04G029	9.892742332	16.815513	0.58831	-0.76535039	0.158	0.48	down	no
Manes.04G030	23.70191161	32.948966	0.719352	-0.47522976	0.23	0.27	down	no
Manes.04G032	801.1834904	639.93038	1.251985	0.32421773	0.018	0.29	up	no
Manes.04G032	2.150458876	0.3124686	6.882161	2.78286162	0.139	0.68	up	no
Manes.04G033	1.467299905	0.3172288	4.625367	2.20956796	0.343	1	up	no
Manes.04G033	198.9798686	260.36159	0.764244	-0.38789414	0.041	0.59	up	no
Manes.04G035	1.06114257	0	Inf	Inf	0.218	NA	--	no
Manes.04G036	372.4742149	177.97255	2.092875	1.06548584	#####	0	up	yes

Manes.04G038	2.806745553	2.8136502	0.997546	-0.00354469	1	0	up		yes
Manes.04G039	71.2189844	64.615912	1.10219	0.14037237	0.62	0.53	up		no
Manes.04G039	6.34779178	2.1791925	2.91291	1.54246115	0.075	0.01	up		yes
Manes.04G042	59.01789912	45.023654	1.31082	0.39046941	0.209	0.02	down		yes
Manes.04G045	2.806745553	0.3172288	8.8477	3.14530246	0.056	0.95	up		no
Manes.04G048	1.012603751	0	Inf	Inf	0.24	NA	--		no
Manes.04G050	568.6941283	504.03477	1.128284	0.17412966	0.233	0.18	up		no
Manes.04G051	1.06114257	0.3124686	3.395998	1.76383558	0.491	1	down		no
Manes.04G055	0.329444781	1.8395955	0.179085	-2.48127997	0.282	0.92	up		no
Manes.04G056	60.09810533	65.743473	0.91413	-0.12952817	0.678	0.25	down		no
Manes.04G057	222.6207079	202.94579	1.096947	0.13349338	0.5	0.58	down		no
Manes.04G057	13.78620131	34.496311	0.399643	-1.3232171	0.002	0	down		yes
Manes.04G058	446.8812891	225.37668	1.98282	0.98755341	#####	0.63	down		no
Manes.04G059	4.552348536	4.0323522	1.128956	0.17498935	0.899	0.54	up		no
Manes.04G059	117.4028529	99.927807	1.174877	0.23250937	0.348	0.87	up		no
Manes.04G060	1.416158202	0	Inf	Inf	0.13	NA	--		no
Manes.04G070	9.487886439	7.7848413	1.218764	0.28541912	0.666	0.01	up		yes
Manes.04G072	0.683158971	4.3272129	0.157875	-2.66314485	0.046	0.19	up		no
Manes.04G074	1.744301541	0.6161331	2.831046	1.50133541	0.405	1	down		no
Manes.04G077	78.07912124	81.966429	0.952574	-0.07009633	0.819	0.95	up		no
Manes.04G079	60.69367016	74.645157	0.813096	-0.2985026	0.292	0.05	down		no
Manes.04G082	319.540663	419.63211	0.761478	-0.39312555	0.015	1	down		no
Manes.04G082	5.617395432	12.161551	0.461898	-1.11435394	0.089	1	down		no
Manes.04G083	66.84982497	63.581172	1.051409	0.07232417	0.827	0.32	up		no
Manes.04G083	0	1.5725799	#NAME?	#NAME?	0.155	0.72	up		no
Manes.04G086	1.036873161	0	Inf	Inf	0.24	1	up		no
Manes.04G088	1805.31977	2052.0136	0.87978	-0.18478591	0.137	0.5	up		no
Manes.04G089	0	3.4066985	#NAME?	#NAME?	0.012	0.78	up		no
Manes.04G090	276.2705096	186.97727	1.477562	0.56321869	0.004	0	up		no
Manes.04G091	442.854578	271.52936	1.630964	0.70572485	#####	0.02	up		no
Manes.04G096	13.71209164	20.593697	0.665839	-0.58675422	0.239	0.05	up		no
Manes.04G097	692.6339774	297.61586	2.327275	1.21864176	#####	0.12	down		no
Manes.04G099	150.0887538	235.35026	0.637725	-0.64899358	0.001	0.96	up		no
Manes.04G101	21.71063824	28.575583	0.759762	-0.39638081	0.47	0.7	down		no
Manes.04G115	10.47622078	19.178186	0.546257	-0.87234789	0.133	0.71	up		no
Manes.04G115	7.643904376	5.2748557	1.449121	0.53517811	0.465	0.03	up		yes
Manes.04G118	1.417459644	0.3124686	4.536327	2.18152474	0.351	0.89	up		no
Manes.04G118	5.114160459	5.8909888	0.868133	-0.20401239	0.968	0.01	up		yes
Manes.04G121	11.08136576	8.659186	1.279724	0.35583239	0.545	0.39	down		no
Manes.04G122	0	1.2370266	#NAME?	#NAME?	0.226	0.89	up		no
Manes.04G123	45.56932465	122.53958	0.371874	-1.42711297	#####	0	down		no
Manes.04G125	18.2864801	13.414036	1.363235	0.44703407	0.473	0.9	down		no
Manes.04G126	32.37655534	20.287883	1.595857	0.67433117	0.089	0	up		yes
Manes.04G129	1.771173834	0.6073292	2.916333	1.54415522	0.627	0.2	up		no
Manes.04G131	65.12559969	68.528562	0.950342	-0.07348066	0.836	0.87	down		no
Manes.04G133	11.61407591	9.895496	1.173673	0.23103045	0.718	0.56	up		no
Manes.04G137	1.491569315	0	Inf	Inf	0.128	NA	--		no
Manes.04G139	12.39301107	13.040656	0.950336	-0.07348967	0.95	0.32	down		no

Manes.04G141	1183.926106	370.22722	3.197836	1.67709616	#####	0	up	no
Manes.04G142	199.5059869	184.35567	1.08218	0.11394027	0.556	0	down	yes
Manes.04G144	29.22706176	19.563953	1.493924	0.57910693	0.141	0.11	down	no
Manes.04G144	8.249049351	28.674116	0.287683	-1.79744923	0.006	0.84	down	no
Manes.04G145	8.200510531	4.3455374	1.887111	0.91617913	0.199	0.02	down	yes
Manes.04G147	26.02839015	18.598702	1.399473	0.48488416	0.25	0.12	down	no
Manes.04G147	13.05803444	13.612769	0.959249	-0.06002282	1	0	up	yes
Manes.04G148	3.643329633	5.3331563	0.683147	-0.54973209	0.745	0.53	up	no
Manes.04G149	3.693169894	0.3172288	11.64197	3.54126381	0.024	0.33	up	no
Manes.04G151	5.54198432	7.4845038	0.740461	-0.43350404	0.638	0.94	down	no
Manes.04G152	5.567555171	47.737594	0.116628	-3.10001001	#####	0.01	up	yes
Manes.04G153	4.502508275	15.220565	0.295817	-1.75722108	0.012	0.01	down	yes
Manes.04G155	2.708366473	0.6073292	4.45947	2.15687237	0.323	0.81	up	no
Manes.04G157	34.55221166	34.207644	1.010073	0.01445932	0.985	0	down	yes
Manes.04G157	20.83016648	10.208681	2.040437	1.02887788	0.053	0	up	yes
Manes.04G158	10.30112915	8.4409506	1.220375	0.28732511	0.609	0.51	down	no
Manes.04G161	0.758570083	2.5099856	0.302221	-1.72632471	0.456	0.63	down	no
Manes.05G001	31.71246001	43.780434	0.724352	-0.46523647	0.183	0.2	down	no
Manes.05G005	26.96335331	1.85792	14.51266	3.85923965	#####	0	up	yes
Manes.05G005	56.28749272	3.7063194	15.1869	3.92475525	#####	0.35	up	no
Manes.05G007	72.74515007	40.711439	1.786848	0.83741686	0.005	0	down	yes
Manes.05G008	0.683158971	5.0335354	0.135721	-2.88127883	0.034	0.7	up	no
Manes.05G009	1657.678526	1936.9437	0.855822	-0.22461776	0.221	0.03	up	no
Manes.05G010	1.721333573	2.4916611	0.690838	-0.53358114	0.832	0.87	up	no
Manes.05G013	5.055583509	0	Inf	Inf	7E-04	0.14	up	no
Manes.05G014	3.995742381	0.3172288	12.59577	3.65486775	0.014	0.62	up	no
Manes.05G015	32.10178318	43.155036	0.743871	-0.4268755	0.23	0	up	yes
Manes.05G018	1.317779122	0.9516865	1.384678	0.46955025	0.92	0.78	up	no
Manes.05G021	1272.884396	775.0196	1.64239	0.7157967	#####	0.89	up	no
Manes.05G021	488.3485421	542.0965	0.900852	-0.15063851	0.378	0	up	no
Manes.05G023	12513.88078	9361.0246	1.336807	0.41879092	0.015	0	up	no
Manes.05G023	0	4.0411562	#NAME?	#NAME?	0.006	0.09	down	no
Manes.05G024	32.5435495	58.467429	0.55661	-0.84526153	0.015	0	down	yes
Manes.05G025	2207.742872	4757.7917	0.464027	-1.10771996	#####	0.14	down	no
Manes.05G026	1.491569315	3.4202628	0.436098	-1.19727615	0.396	0.25	up	no
Manes.05G029	70.37403703	46.248294	1.521657	0.60564313	0.079	0.04	up	no
Manes.05G032	9.794363252	12.084209	0.810509	-0.30309944	0.654	0.2	down	no
Manes.05G034	82.31179732	71.88863	1.14499	0.19533561	0.495	0.77	up	no
Manes.05G036	102.5084529	199.63218	0.513487	-0.9616014	#####	0.95	down	no
Manes.05G036	28.58798265	17.023511	1.679323	0.74788017	0.095	1	down	no
Manes.05G038	13.94315734	18.9621	0.735317	-0.44356148	0.399	0.74	down	no
Manes.05G040	4.528079126	0.9286017	4.876234	2.28576734	0.041	0.02	up	yes
Manes.05G041	7.204740943	2.4923777	2.89071	1.53142386	0.068	0	up	yes
Manes.05G045	1.086713422	0.3172288	3.425645	1.77637572	0.557	0	up	yes
Manes.05G049	2.758206734	0.9469262	2.9128	1.54240666	0.239	0.09	up	no
Manes.05G052	6.700204528	5.8671874	1.141979	0.19153606	0.845	0.77	down	no
Manes.05G057	107.2130097	92.293861	1.161648	0.21617338	0.375	0.56	down	no
Manes.05G057	3715.355231	929.52745	3.997037	1.99893078	#####	0	down	no

Manes.05G059	3.767279564	0.9421659	3.998531	1.99947001	0.105	0.91	up		no
Manes.05G060	4.858825349	3.0502101	1.592948	0.67169894	0.766	0.33	up		no
Manes.05G060	56.86223448	57.23163	0.993546	-0.00934192	0.971	0.85	up		no
Manes.05G061	2.076349205	5.8983596	0.352021	-1.50626468	0.15	0	up		yes
Manes.05G064	7.79342516	0	Inf	Inf	#####	NA	--		no
Manes.05G064	215.4907159	68.384116	3.151181	1.65589256	#####	0	down		yes
Manes.05G064	418.6945359	115.63715	3.620762	1.85629319	#####	1	down		no
Manes.05G067	0.988334342	3.1030339	0.318506	-1.6506084	0.49	0.86	down		no
Manes.05G067	6.678538002	4.9623871	1.345832	0.42849803	0.674	0.6	up		no
Manes.05G068	40.07866326	71.270838	0.562343	-0.8304775	0.013	0	down		yes
Manes.05G068	1.062444012	5.5880408	0.190128	-2.39495574	0.05	0.05	down		yes
Manes.05G069	20.69357553	16.77006	1.23396	0.30329509	0.536	0.45	up		no
Manes.05G073	143.4622855	750.78031	0.191084	-2.38771929	#####	0	up		yes
Manes.05G075	85.43300956	48.476522	1.762358	0.8175074	0.003	0.3	up		no
Manes.05G077	6.119328963	1.8531597	3.302106	1.72338623	0.062	0.89	up		no
Manes.05G081	113.6065165	41.00679	2.770432	1.47011086	0.064	0.73	down		no
Manes.05G082	1.417459644	0.6208934	2.282936	1.1908901	0.603	0.84	up		no
Manes.05G083	1.06114257	0	Inf	Inf	0.218	NA	--		no
Manes.05G085	2.150458876	0	Inf	Inf	0.041	NA	--		no
Manes.05G086	64.09318549	77.608014	0.825858	-0.27603466	0.396	0	up		yes
Manes.05G086	1.416158202	0.3172288	4.464153	2.15838662	0.35	1	down		no
Manes.05G086	1.088014864	0	Inf	Inf	0.238	NA	--		no
Manes.05G088	29.59761012	116.75645	0.253499	-1.9799497	#####	0.01	down		yes
Manes.05G088	2.931996927	0.3124686	9.383334	3.23010067	0.055	0.02	up		yes
Manes.05G090	133.0250355	139.5569	0.953196	-0.0691557	0.83	0	down		yes
Manes.05G091	2.907727517	0	Inf	Inf	0.014	0.22	up		no
Manes.05G092	2.428761953	4.6756139	0.519453	-0.94493469	0.391	0.72	down		no
Manes.05G092	4.62906109	10.616099	0.436042	-1.19746228	0.085	0.14	down		no
Manes.05G094	2.504173066	4.9393023	0.506989	-0.979973	0.326	0.87	down		no
Manes.05G094	34.68657313	72.534502	0.478208	-1.06429009	7E-04	0	down		yes
Manes.05G096	37.56965783	23.984221	1.566432	0.64748238	0.119	0.68	down		no
Manes.05G098	0.379285042	1.5494951	0.24478	-2.03044384	0.389	0.06	up		no
Manes.05G099	3.541046227	8.6775105	0.408072	-1.29310553	0.093	0.52	down		no
Manes.05G101	35.66877367	31.769523	1.122736	0.16701818	0.679	0.23	down		no
Manes.05G101	21.78121699	51.202594	0.425393	-1.23313232	7E-04	0.41	up		no
Manes.05G103	22.39900298	6.5416212	3.424075	1.77571438	0.002	0.63	up		no
Manes.05G103	19.75600949	43.200233	0.457313	-1.12874754	0.003	0	down		yes
Manes.05G103	19.52754667	31.420406	0.621493	-0.68619109	0.093	0.16	down		no
Manes.05G104	13.96872819	12.800052	1.091302	0.12605098	0.807	0.41	down		no
Manes.05G104	34.80754677	22.987542	1.514192	0.5985479	0.16	1	down		no
Manes.05G107	4.778581874	1.2370266	3.862958	1.94970605	0.077	1	down		no
Manes.05G108	1.896425208	0	Inf	Inf	0.064	NA	--		no
Manes.05G108	2.428761953	12.967358	0.187298	-2.41659154	0.002	0.48	down		no
Manes.05G109	3.897363301	3.4019382	1.14563	0.19614147	1	0.05	up		no
Manes.05G112	22.31522858	19.588471	1.139202	0.18802383	0.689	0	up		yes
Manes.05G113	25.27632728	10.891919	2.32065	1.21452871	0.007	0.76	up		no
Manes.05G115	6.171772108	4.6986987	1.313507	0.39342354	0.655	0.28	up		no
Manes.05G116	122.205065	21.80334	5.604878	2.48668305	#####	0.08	down		no

Manes.05G119	29.70602733	18.326209	1.620959	0.69684728	0.082	0.82	down	no
Manes.05G119	17.43826763	15.831221	1.101511	0.13948418	0.772	0	up	yes
Manes.05G122	0	1.2322663	#NAME?	#NAME?	0.226	1	down	no
Manes.05G123	27.52516524	14.013994	1.96412	0.97388305	0.023	0.17	up	no
Manes.05G124	8.47658413	13.987582	0.606008	-0.72259168	0.217	0.84	up	no
Manes.05G127	59.0828987	41.591721	1.420545	0.50644422	0.107	0.01	down	yes
Manes.05G127	35.27135302	23.306921	1.513342	0.59773849	0.114	0	down	yes
Manes.05G127	17.48680645	13.108477	1.334007	0.41576676	0.382	0.35	down	no
Manes.05G127	30.13645407	13.371449	2.253791	1.17235387	0.005	1	down	no
Manes.05G128	132.561506	130.4515	1.016175	0.02314837	0.938	0.86	up	no
Manes.05G128	85.16633488	105.14318	0.810003	-0.30400017	0.231	0.8	up	no
Manes.05G128	0.329444781	2.1744322	0.151508	-2.72253017	0.192	0.17	down	no
Manes.05G129	92.21383097	72.67253	1.268895	0.34357303	0.185	0.07	down	no
Manes.05G130	53.44941591	109.35279	0.48878	-1.03274399	1E-04	0	down	yes
Manes.05G132	35.23890155	110.94705	0.317619	-1.65463039	#####	0.31	down	no
Manes.05G134	7.239048483	7.4262031	0.974798	-0.03682469	1	0.2	down	no
Manes.05G135	560.3462344	16.720563	33.5124	5.06662317	#####	0.02	up	yes
Manes.05G136	308.9076674	300.47849	1.028053	0.03991394	0.779	0	up	no
Manes.05G139	12.17235691	9.6101559	1.266614	0.3409768	0.572	0	up	yes
Manes.05G140	427.0632224	249.53511	1.711435	0.77520682	#####	1	down	no
Manes.05G141	159.7624322	339.23193	0.470953	-1.08634378	#####	0	down	yes
Manes.05G142	118.8141788	101.59201	1.169523	0.22592008	0.365	0.67	up	no
Manes.05G144	6.628697741	7.1767955	0.923629	-0.11461434	0.991	0.02	down	yes
Manes.05G150	53.8749027	61.375312	0.877794	-0.18804509	0.66	0.02	down	yes
Manes.05G154	2.834919289	0	Inf	Inf	0.014	0	up	yes
Manes.05G158	4.6533305	4.6756139	0.995234	-0.00689213	1	0	up	yes
Manes.05G160	165.227704	102.33834	1.614524	0.69110889	0.004	0	up	yes
Manes.05G160	1003.404763	639.46019	1.569143	0.64997724	#####	0	up	no
Manes.05G162	450.0881209	211.29082	2.130183	1.09097741	#####	1	up	no
Manes.05G165	176.2877766	121.79185	1.447451	0.53351482	0.012	0	up	yes
Manes.05G165	285.2114195	141.97524	2.008881	1.00639244	#####	0	up	yes
Manes.05G165	335.8129247	185.73359	1.808036	0.85442303	#####	0	up	yes
Manes.05G165	162.6406846	111.59508	1.457418	0.54341481	0.042	0	up	yes
Manes.05G166	30.96783238	10.266982	3.016255	1.59275827	3E-04	0	down	yes
Manes.05G170	0.379285042	15.18796	0.024973	-5.32350178	#####	0	down	yes
Manes.05G172	44.75366029	25.606042	1.747777	0.80552142	0.049	0.93	up	no
Manes.05G172	15.30817384	15.293607	1.000952	0.00137347	1	0.99	up	no
Manes.05G173	400.8656327	482.22788	0.831278	-0.26659632	0.504	0.83	up	no
Manes.05G173	3034.080364	2963.5717	1.023792	0.03392233	0.842	0.01	up	no
Manes.05G173	206.5813064	178.08858	1.159992	0.21411473	0.542	0.02	up	yes
Manes.05G173	3.716137861	13.681051	0.271627	-1.88030314	0.086	0.16	down	no
Manes.05G173	7.691141753	13.449251	0.571864	-0.80625619	0.266	0.31	down	no
Manes.05G175	2.200299137	0.9286017	2.369476	1.24456785	0.455	1	up	no
Manes.05G178	9.886235122	0	Inf	Inf	#####	0.23	up	no
Manes.05G178	4.298314868	0.6249371	6.877996	2.78198825	0.173	1	down	no
Manes.05G178	7.892732276	10.560409	0.747389	-0.42006902	0.693	0.71	down	no
Manes.05G180	980.3593009	32.3897	30.26763	4.91970358	#####	0.36	up	no
Manes.05G182	367.48457	248.75051	1.477322	0.5629842	9E-04	0	up	no

Manes.05G183	297.9620721	351.56046	0.847541	-0.23864413	0.15	0.01	down	no
Manes.05G187	454.5686848	59.718736	7.611827	2.92824276	#####	0	up	yes
Manes.05G187	93.10806396	21.438047	4.343122	2.11873262	#####	0.08	up	no
Manes.05G190	355.081679	447.48852	0.793499	-0.33369976	0.036	0	up	no
Manes.05G190	108.3368378	136.62117	0.792973	-0.33465721	0.145	1	up	no
Manes.05G190	4.881793316	4.9697579	0.9823	-0.02576436	1	0.34	up	no
Manes.05G191	19.90813315	86.676512	0.229683	-2.12228316	#####	0	down	yes
Manes.05G193	11.09689848	27.970404	0.396737	-1.33374458	0.034	0.66	up	no
Manes.05G194	37.51666006	104.28263	0.359759	-1.47489557	#####	0.04	down	no
Manes.05G195	9.033190285	13.094196	0.689862	-0.53562	0.399	0.73	down	no
Manes.05G195	29.81993913	25.419725	1.173102	0.23032886	0.586	0.21	up	no
Manes.05G195	223.7199548	211.78057	1.056376	0.07912368	0.657	0.88	up	no
Manes.05G196	45.9126273	27.100103	1.694186	0.76059267	0.031	0.59	up	no
Manes.05G197	475.0175916	33.344253	14.24586	3.83247091	#####	0	up	yes
Manes.05G197	7.561058017	2.782478	2.717383	1.44221784	0.075	0.57	up	no
Manes.05G199	15.30687239	12.097773	1.265264	0.339438	0.501	0.11	down	no
Manes.05G199	17.51367874	26.807136	0.653322	-0.61413494	0.214	0	up	yes
Manes.05G201	2.126189466	2.7601098	0.770328	-0.3764555	0.799	0.49	up	no
Manes.05G201	12.46972363	21.103927	0.590872	-0.759082	0.113	0.11	up	no
Manes.05G201	5.967205296	4.3719493	1.364884	0.44877875	0.575	0	up	yes
Manes.05G204	1.391888793	0.6344577	2.193825	1.13344813	0.797	0.71	down	no
Manes.05G206	173.0987697	110.48537	1.566712	0.64774012	0.003	0.04	up	no
Manes.05G207	12.54513474	9.8650404	1.271676	0.3467311	0.728	0.11	up	no
Manes.06G002	5.665934251	8.0973098	0.69973	-0.51512882	0.512	0.14	down	no
Manes.06G003	8.680777537	29.790242	0.291397	-1.77894365	2E-04	0.43	up	no
Manes.06G003	46.07404229	40.871343	1.127295	0.17286452	0.566	0.95	down	no
Manes.06G003	576.0398961	202.71881	2.841571	1.50668876	#####	0	up	yes
Manes.06G011	1.062444012	0.3172288	3.349141	1.743791	0.558	1	up	no
Manes.06G015	301.0654025	572.73145	0.525666	-0.9277819	9E-04	0	up	yes
Manes.06G016	3.063382105	2.4604889	1.24503	0.31618032	0.822	0.13	up	no
Manes.06G016	125.0165353	382.54132	0.326805	-1.61349665	#####	0.02	up	no
Manes.06G017	1.870854356	1.2370266	1.51238	0.59682076	0.766	1	down	no
Manes.06G018	1.06114257	0.3172288	3.345038	1.74202268	0.49	1	up	no
Manes.06G018	8.775252291	7.1449067	1.228183	0.29652543	0.667	0.06	up	no
Manes.06G020	1452.042345	1348.3217	1.076926	0.10691875	0.384	0	up	no
Manes.06G024	32.04711056	68.472902	0.468026	-1.09533887	1E-03	0.46	up	no
Manes.06G026	81.06310333	147.42408	0.549863	-0.86285489	0.002	0.96	up	no
Manes.06G029	249.3784588	349.11885	0.714308	-0.4853814	0.005	0.13	up	no
Manes.06G029	176.0627601	186.68456	0.943103	-0.0845128	0.67	0.44	up	no
Manes.06G030	141.8201828	177.70886	0.798048	-0.32545274	0.126	0.36	up	no
Manes.06G031	66.21650626	121.30116	0.545885	-0.87333049	5E-04	0.39	down	no
Manes.06G032	3.617758781	4.6437251	0.779064	-0.36018635	0.776	0.21	down	no
Manes.06G033	76.28070171	36.400861	2.095574	1.06734553	0.01	0.52	up	no
Manes.06G033	1.416158202	1.2689153	1.116038	0.15838662	1	0.96	down	no
Manes.06G033	7.738379131	8.3324366	0.928705	-0.10670702	0.938	0.79	down	no
Manes.06G035	32.43903662	44.762832	0.724687	-0.46457034	0.189	0.13	down	no
Manes.06G037	133.9958844	136.31154	0.983012	-0.02471903	0.943	0.02	down	no
Manes.06G037	299.4990118	214.33963	1.397311	0.48265265	0.006	0.03	up	no

Manes.06G038	3.565315636	10.608012	0.336096	-1.57305261	0.037	0.95	down	no
Manes.06G039	188.6287179	250.42351	0.753239	-0.4088207	0.029	0.01	down	no
Manes.06G039	1.036873161	0	Inf	Inf	0.24	1	up	no
Manes.06G041	28.48532584	14.676297	1.940907	0.95673093	0.028	0.56	down	no
Manes.06G041	1.41485676	0.6296974	2.246884	1.16792541	0.515	0.63	up	no
Manes.06G042	0.379285042	1.2553511	0.302135	-1.72673653	0.543	0.82	up	no
Manes.06G043	285.1648564	57.525263	4.957211	2.30952862	#####	0	up	yes
Manes.06G043	28.72541705	51.344429	0.559465	-0.83787992	0.053	0.06	down	no
Manes.06G047	0.683158971	4.0242649	0.16976	-2.55843202	0.067	0.96	down	no
Manes.06G047	1.417459644	26.630801	0.053226	-4.23171625	#####	0	down	yes
Manes.06G048	0.35371419	2.4916611	0.141959	-2.81645184	0.136	0.69	down	no
Manes.06G051	29.62968818	41.830431	0.708329	-0.49750941	0.166	0	down	yes
Manes.06G052	5.562722808	5.3141152	1.046782	0.06596171	0.914	0.81	down	no
Manes.06G052	85.72944825	103.86472	0.825395	-0.27684291	0.277	0.09	down	no
Manes.06G053	15.68392796	54.685457	0.286803	-1.80187028	#####	0.48	down	no
Manes.06G057	12.65225051	9.3112516	1.358813	0.44234702	0.439	0	up	yes
Manes.06G058	1.467299905	2.7770011	0.528376	-0.92036399	0.589	0.44	down	no
Manes.06G059	24636.57708	16715.65	1.473863	0.55960238	0.018	0.03	down	no
Manes.06G061	1.036873161	0	Inf	Inf	0.24	0.01	up	yes
Manes.06G062	3.943299236	0.933362	4.224834	2.07889459	0.083	0	up	yes
Manes.06G063	220.9810039	377.31615	0.585665	-0.7718515	#####	0	down	yes
Manes.06G065	256.0727108	299.06499	0.856244	-0.22390551	0.224	1	down	no
Manes.06G067	358.1325156	227.79719	1.572155	0.65274356	2E-04	0.51	down	no
Manes.06G067	91.75178415	138.12114	0.664285	-0.59012606	0.025	0.9	up	no
Manes.06G067	9.565900436	10.550889	0.906644	-0.14139184	0.881	0	up	yes
Manes.06G068	16.4473304	108.61102	0.151433	-2.72324515	#####	0.36	up	no
Manes.06G069	57.69528764	164.05204	0.351689	-1.50762814	#####	0	up	no
Manes.06G070	102.3354095	34.873274	2.934494	1.55311172	#####	0.46	up	no
Manes.06G070	20.44567567	20.262904	1.00902	0.01295477	0.996	0.98	down	no
Manes.06G073	3.995742381	2.4780968	1.612424	0.68923101	0.547	0.67	up	no
Manes.06G074	54.7781612	0.924558	59.24794	5.88869318	#####	0	up	yes
Manes.06G074	2893.890861	2706.0135	1.06943	0.0968415	0.549	0.35	up	no
Manes.06G074	353.2892966	508.82632	0.694322	-0.52632325	0.001	0.94	down	no
Manes.06G075	38.73308381	32.14838	1.204822	0.26882034	0.494	0.01	down	yes
Manes.06G077	88.04400958	59.228755	1.486508	0.57192709	0.054	0.28	down	no
Manes.06G078	0.658889561	1.5271269	0.431457	-1.21271139	0.75	0.32	down	no
Manes.06G078	45.26377587	25.176972	1.797824	0.84625215	0.017	1	down	no
Manes.06G087	267.6675418	254.1458	1.053205	0.07478579	0.682	0.52	up	no
Manes.06G087	386.59755	313.90832	1.231562	0.30048921	0.056	0.75	up	no
Manes.06G088	2.527141034	4.943346	0.511221	-0.96798171	0.422	0.95	down	no
Manes.06G090	5.010949016	7.4533316	0.67231	-0.57280159	0.589	0.33	down	no
Manes.06G091	4.958505871	4.3719493	1.134164	0.1816288	0.886	0.32	up	no
Manes.06G091	5.667235693	27.34951	0.207215	-2.2707979	#####	0.02	up	yes
Manes.06G092	27.14810967	40.770456	0.665877	-0.58667235	0.125	0.41	down	no
Manes.06G092	2.049476912	1.5630594	1.311196	0.39088316	0.834	0.86	down	no
Manes.06G095	36.68490834	46.279722	0.792678	-0.33519351	0.332	0.94	up	no
Manes.06G098	46.96437096	42.666202	1.100739	0.13847298	0.655	0.93	up	no
Manes.06G099	142.9997807	185.81328	0.769589	-0.37784071	0.077	0	down	yes

Manes.06G099	1223.88092	761.56908	1.607052	0.68441638	#####	0.41	up	no
Manes.06G100	75.15001602	40.386123	1.860788	0.89591377	0.003	0	up	yes
Manes.06G100	168.3991529	148.96428	1.130467	0.17691844	0.432	0.34	up	no
Manes.06G102	1.06114257	0.6249371	1.697999	0.76383558	0.891	0.71	down	no
Manes.06G103	1522.063257	658.37813	2.311838	1.20904001	#####	0.01	down	no
Manes.06G104	1.391888793	0.6073292	2.291819	1.19649336	0.785	0.2	up	no
Manes.06G107	1.517140166	0	Inf	Inf	0.113	0.21	up	no
Manes.06G109	4.503809717	1.849116	2.435656	1.28431012	0.288	0.38	up	no
Manes.06G110	0	1.5671031	#NAME?	#NAME?	0.155	0.61	down	no
Manes.06G112	97.25937635	109.47323	0.888431	-0.17066893	0.487	0.02	down	no
Manes.06G112	77.69425703	75.867186	1.024082	0.03433193	0.876	0.04	up	no
Manes.06G112	191.6491525	175.36497	1.092859	0.12810701	0.531	1	down	no
Manes.06G114	14.97612617	24.620573	0.608277	-0.71719983	0.13	0	down	yes
Manes.06G114	18.90036177	23.357134	0.80919	-0.30544943	0.532	0.9	up	no
Manes.06G116	529.8250226	930.76141	0.569238	-0.81289542	#####	0.21	up	no
Manes.06G117	3.161761185	0	Inf	Inf	0.008	NA	--	no
Manes.06G117	11.7609938	7.4973514	1.568686	0.64955704	0.37	0.77	down	no
Manes.06G119	3.59218793	5.3181589	0.675457	-0.56606406	0.579	0.64	down	no
Manes.06G122	26.35653349	6.1810892	4.26406	2.09222765	7E-04	0.43	down	no
Manes.06G126	5.23811039	9.2969708	0.563421	-0.82771425	0.255	0	up	yes
Manes.06G126	33.14256067	15.91665	2.082257	1.05814832	0.008	0.76	up	no
Manes.06G134	1.038174603	0	Inf	Inf	0.24	NA	--	no
Manes.06G134	7.96591391	2.1751488	3.662239	1.87272577	0.024	1	down	no
Manes.06G135	86.58677081	69.137815	1.252379	0.3246716	0.244	0.04	up	no
Manes.06G135	4.935537903	14.598955	0.338075	-1.56458586	0.021	0.46	up	no
Manes.06G137	2.758206734	1.8531597	1.48838	0.57374337	0.668	0.49	down	no
Manes.06G140	41.47055205	30.322379	1.367655	0.45170432	0.259	0.1	down	no
Manes.06G140	101.000157	103.06677	0.979949	-0.02922177	0.884	0.1	down	no
Manes.06G142	184.4232996	9.0713645	20.33027	4.34555754	#####	0.07	up	no
Manes.06G142	15.51143921	25.853556	0.599973	-0.73703016	0.102	0.13	down	no
Manes.06G146	1.038174603	1.8619637	0.55757	-0.84277583	0.736	0.11	up	no
Manes.06G146	32.12567919	30.796902	1.043146	0.06094174	0.855	0.01	down	yes
Manes.06G147	60.79427872	47.254493	1.286529	0.36348405	0.235	0.2	up	no
Manes.06G147	365.7788537	316.1636	1.156929	0.21030039	0.203	0.23	down	no
Manes.06G149	4.8843962	8.8138696	0.554172	-0.85159532	0.336	0.38	down	no
Manes.06G155	133.6552813	139.52642	0.957921	-0.06202145	0.819	0.72	down	no
Manes.06G156	0.329444781	7.4445276	0.044253	-4.49807173	6E-04	0.34	down	no
Manes.06G158	1.06114257	0.3172288	3.345038	1.74202268	0.49	1	up	no
Manes.06G159	525.0963281	415.06644	1.26509	0.33923981	0.02	0.94	down	no
Manes.06G162	106.3893555	128.87774	0.825506	-0.27664931	0.247	0.7	down	no
Manes.06G166	1.137855125	0	Inf	Inf	0.207	1	up	no
Manes.06G168	2.477300773	2.1927568	1.129765	0.17602325	0.955	0.06	up	no
Manes.06G170	280.6589018	371.20588	0.756073	-0.40340175	0.035	1	up	no
Manes.06G170	671.2021434	1.5542554	431.848	8.75437992	#####	0.3	down	no
Manes.06G170	586.987386	558.53354	1.050944	0.07168559	0.801	0.83	down	no
Manes.06G170	19.80845263	14.96092	1.324013	0.40491728	0.388	0.03	down	yes
Manes.06G170	4830.640972	8586.9424	0.562557	-0.82992988	#####	1	down	no
Manes.06G170	24.33132599	0	Inf	Inf	#####	NA	--	no

Manes.06G171	62.75793299	58.256134	1.077276	0.10738788	0.755	0.46	down	no
Manes.06G171	147.6955162	126.30349	1.16937	0.22573155	0.291	0	down	yes
Manes.06G172	5.741345364	2.1784759	2.635487	1.39806968	0.413	0.72	down	no
Manes.06G174	130.3005817	54.561454	2.388143	1.25588952	#####	0.75	down	no
Manes.06G174	13.15678692	38.166698	0.344719	-1.53650717	3E-04	0.75	down	no
Manes.06G176	67.24305247	18.323599	3.669751	1.87568233	#####	0.05	down	no
Manes.06G177	2.149157434	2.1751488	0.988051	-0.01734295	1	0.97	down	no
Manes.06G178	11.68948702	13.764586	0.849244	-0.23574963	0.721	0.31	down	no
Manes.07G000	7.715411163	5.3467206	1.443017	0.52908876	0.621	0.58	up	no
Manes.07G002	37.13830305	41.9068	0.886212	-0.17427647	0.66	0.17	down	no
Manes.07G004	0.658889561	2.791282	0.236053	-2.0828193	0.336	0.41	down	no
Manes.07G004	303.6654605	101.08229	3.004141	1.58695255	#####	0	up	yes
Manes.07G005	76.88677472	35.187892	2.185035	1.12765637	4E-04	0	up	yes
Manes.07G005	137.7597297	120.15575	1.14651	0.19724851	0.387	0.05	down	no
Manes.07G005	300.4485796	214.12062	1.403174	0.48869435	0.038	0	down	yes
Manes.07G007	25.65077996	15.242217	1.682877	0.75092998	0.088	0	up	yes
Manes.07G008	0.708729822	1.2417869	0.570734	-0.8091099	0.872	0.49	up	no
Manes.07G009	63.53788077	120.80782	0.525942	-0.92702493	6E-04	0.74	down	no
Manes.07G015	156.2146866	108.42276	1.440792	0.52686248	0.048	0	down	yes
Manes.07G015	312.287214	401.62933	0.777551	-0.36299114	0.025	0	up	no
Manes.07G016	88.54270101	241.92521	0.365992	-1.4501158	#####	0.02	down	no
Manes.07G021	0	1.5623428	#NAME?	#NAME?	0.155	0.32	down	no
Manes.07G022	21.18090438	21.983749	0.96348	-0.05367323	0.979	0.36	down	no
Manes.07G022	123.2184892	32.364721	3.807185	1.92872477	#####	0.33	down	no
Manes.07G025	151.4549871	109.66313	1.381093	0.46581049	0.033	0	up	no
Manes.07G028	70.6730786	87.776749	0.805146	-0.31267808	0.244	0	up	yes
Manes.07G029	1.441729054	0.3124686	4.613997	2.20601714	0.349	1	down	no
Manes.07G029	1.112284273	0	Inf	Inf	0.238	NA	--	no
Manes.07G030	2.883458108	3.4257396	0.841704	-0.24861545	0.925	0.05	down	no
Manes.07G030	347.3292838	282.18134	1.230873	0.29968148	0.077	0	down	yes
Manes.07G039	22.00614889	15.894999	1.38447	0.46933379	0.302	0.57	down	no
Manes.07G043	744.0068399	428.81658	1.735023	0.79495518	#####	0.55	down	no
Manes.07G044	38.1327712	27.72028	1.375627	0.46008957	0.192	0	up	yes
Manes.07G044	75.15299231	54.458161	1.380013	0.46468228	0.11	0	up	yes
Manes.07G044	12.04189977	18.634634	0.646211	-0.62992349	0.214	0	up	yes
Manes.07G046	57.40015039	51.560515	1.113258	0.15478784	0.634	0.52	up	no
Manes.07G053	14.47456605	10.503542	1.378065	0.46264422	0.462	0.78	up	no
Manes.07G057	10.21698135	31.905882	0.320222	-1.64285342	0.002	0.41	down	no
Manes.07G057	112.0856083	86.607053	1.294186	0.37204463	0.139	0.62	up	no
Manes.07G058	8939.285717	2368.1826	3.774745	1.91637922	#####	0	up	yes
Manes.07G059	6.853629637	7.1625147	0.956875	-0.06359795	0.998	0.01	up	yes
Manes.07G061	30.74810626	44.795898	0.686405	-0.54286908	0.163	0.06	down	no
Manes.07G062	8.529955312	8.7350946	0.976516	-0.03428514	0.998	0.05	down	yes
Manes.07G062	14.0111332	17.989479	0.778852	-0.36057974	0.456	0.18	up	no
Manes.07G064	40.70845105	37.315438	1.090928	0.1255557	0.71	0.2	down	no
Manes.07G064	4.323885719	5.5832805	0.774435	-0.36878466	0.703	0.91	down	no
Manes.07G066	9.461014146	7.4892641	1.263277	0.33717088	0.599	0.31	up	no
Manes.07G066	0.658889561	1.5447348	0.426539	-1.22925064	0.769	0.86	down	no

Manes.07G069	16.60038211	12.495671	1.328491	0.40978808	0.382	0.8	up	no
Manes.07G070	4.575316503	5.8583835	0.780986	-0.35663108	0.88	0.64	down	no
Manes.07G075	1.390587351	0.3172288	4.383547	2.13209855	0.351	1	down	no
Manes.07G075	70.99052158	45.852546	1.548235	0.63062457	0.05	0.44	up	no
Manes.07G082	0	1.2593948	#NAME?	#NAME?	0.227	0.41	down	no
Manes.07G083	38.30563335	45.73617	0.837535	-0.25577899	0.46	0.95	down	no
Manes.07G083	4.754312464	1.2593948	3.775077	1.91650613	0.076	0.19	up	no
Manes.07G084	1.416158202	0.3172288	4.464153	2.15838662	0.35	1	up	no
Manes.07G084	0.758570083	3.4155025	0.222096	-2.17074346	0.297	0.02	up	yes
Manes.07G086	799.5422927	460.19368	1.737404	0.79693319	#####	0.16	up	no
Manes.07G088	3.516776817	15.19533	0.231438	-2.11130235	0.002	1	down	no
Manes.07G090	7.083393895	4.018788	1.76257	0.8176803	0.296	0.31	up	no
Manes.07G092	12.46842219	15.885478	0.784894	-0.34942961	0.52	0.65	down	no
Manes.07G093	1271.189517	184.80997	6.87836	2.78206463	#####	0	up	yes
Manes.07G094	14.3441089	1.2146584	11.80917	3.56183585	0.027	0.46	up	no
Manes.07G095	394.1510387	37.256421	10.57941	3.40318759	#####	0	up	yes
Manes.07G096	65.78541729	46.060053	1.428253	0.51425174	0.078	0.06	up	no
Manes.07G096	45.55723827	35.510598	1.282919	0.35943065	0.285	0.71	up	no
Manes.07G097	14.82177303	31.044876	0.477431	-1.06663714	0.013	0	down	yes
Manes.07G098	133.8713689	533.0386	0.251148	-1.99339257	#####	0.22	down	no
Manes.07G101	10.40211111	25.246943	0.412015	-1.27923236	0.009	0.81	down	no
Manes.07G102	4.603490239	7.5163925	0.61246	-0.70731233	0.388	0.35	up	no
Manes.07G103	10.02282607	30.484433	0.328785	-1.60478336	0.001	0.57	down	no
Manes.07G104	16.33891319	24.192219	0.675379	-0.56623111	0.203	0.19	down	no
Manes.07G106	145.7539019	394.51637	0.36945	-1.43655064	#####	0.03	down	no
Manes.07G106	2.859188698	3.7741407	0.757573	-0.40054235	0.789	0.35	up	no
Manes.07G107	85.86436434	69.486903	1.235691	0.30531843	0.233	0	down	yes
Manes.07G111	302.0876554	248.22244	1.217004	0.28333366	0.238	0.23	up	no
Manes.07G113	1.745602983	2.7770011	0.628593	-0.66980229	0.688	0.6	down	no
Manes.07G114	1.137855125	0.6073292	1.873539	0.90576629	0.88	0.72	down	no
Manes.07G116	9.182711068	28.552498	0.321608	-1.63662493	6E-04	0.24	down	no
Manes.07G117	14.77323421	0.6296974	23.46085	4.5521832	#####	1	down	no
Manes.07G117	111.8021841	62.982677	1.775126	0.82792138	0.001	0.82	up	no
Manes.07G119	396.2536826	2.4645326	160.7825	7.32896648	#####	0.19	up	no
Manes.07G119	2.934599811	0.924558	3.174057	1.66632802	0.227	0.9	up	no
Manes.07G122	584.0891604	448.81626	1.301399	0.38006366	0.084	0.75	up	no
Manes.07G123	17.57737688	88.03493	0.199664	-2.32435628	#####	0	down	yes
Manes.07G124	165.0700011	213.05594	0.774773	-0.36815431	0.067	0.25	down	no
Manes.07G125	2.477300773	8.9566571	0.276588	-1.85419134	0.093	0.44	down	no
Manes.07G133	16.37099125	10.942132	1.496143	0.58124779	0.272	0.63	up	no
Manes.07G136	366.7030935	192.19878	1.907937	0.93201327	#####	0.79	up	no
Manes.07G136	20.31038616	26.115094	0.777726	-0.36266624	0.392	0	down	yes
Manes.07G137	1.416158202	0.6296974	2.24895	1.16925185	0.603	1	up	no
Manes.07G137	1.086713422	0.3036646	3.578664	1.83942094	0.558	1	down	no
Manes.07G139	79.33282882	167.22078	0.47442	-1.0757642	1E-04	0	down	yes
Manes.07G139	1.390587351	3.139683	0.442907	-1.17492453	0.462	0.88	down	no
Manes.07G139	208.5825949	437.9664	0.476253	-1.0702014	#####	0	down	yes
Manes.07G142	73.5202655	29.877104	2.460756	1.29910167	#####	0.02	up	no

Manes.07G143	32.33415033	28.475413	1.135511	0.18334196	0.728	0.47	down	no
Manes.07G143	69.48966094	28.207424	2.463524	1.3007234	0.001	0	up	yes
Manes.08G001	1.342048532	2.8088899	0.477786	-1.06556324	0.527	0	up	yes
Manes.08G002	10.80306268	15.812897	0.683181	-0.5496613	0.328	0.02	down	yes
Manes.08G004	168.0014434	181.44824	0.925892	-0.11108447	0.58	0	down	no
Manes.08G004	4.957204429	17.697229	0.280112	-1.83592479	0.002	0.14	down	no
Manes.08G004	20.97448149	35.673604	0.587955	-0.76622182	0.078	0.26	down	no
Manes.08G004	1021.936795	741.20631	1.378748	0.4633589	0.006	0.84	up	no
Manes.08G005	654.0270719	213.74085	3.059907	1.61348773	#####	0	down	yes
Manes.08G006	128.8429465	42.717849	3.016138	1.59270265	#####	0	down	yes
Manes.08G006	11.0496611	3.7510559	2.945747	1.55863347	0.021	0.23	down	no
Manes.08G008	2.859188698	0.6208934	4.604959	2.20318828	0.13	0.02	up	yes
Manes.08G010	967.587747	959.1994	1.008745	0.01256174	0.886	0.94	up	no
Manes.08G010	417.2542545	369.16727	1.130258	0.17665212	0.247	1	down	no
Manes.08G011	24.73265096	26.705532	0.926125	-0.11072175	0.781	0	down	yes
Manes.08G012	12.44415278	1.8531597	6.7151	2.74740888	2E-04	1	down	no
Manes.08G014	3.870491007	14.238884	0.271825	-1.87924753	0.011	1	up	no
Manes.08G020	1.062444012	0.6249371	1.700081	0.7656039	1	0.89	up	no
Manes.08G021	61.89615146	56.251352	1.10035	0.13796193	0.727	0.83	up	no
Manes.08G022	395.7609316	599.65676	0.659979	-0.59950774	#####	0.79	down	no
Manes.08G023	30.30894282	57.312043	0.528841	-0.91909481	0.007	0.16	down	no
Manes.08G023	11.94129121	24.507299	0.487254	-1.03725262	0.099	0.03	down	yes
Manes.08G024	6.374664073	18.317405	0.348011	-1.52279394	0.007	0.62	down	no
Manes.08G024	1.416158202	0.6073292	2.33178	1.22143185	0.789	1	down	no
Manes.08G024	25.4779178	34.778324	0.73258	-0.44894103	0.263	0.83	down	no
Manes.08G029	580.5632396	411.84661	1.409659	0.4953461	6E-04	0.62	down	no
Manes.08G030	830.16152	661.52566	1.25492	0.32759494	0.015	0.05	down	no
Manes.08G031	7.518653003	9.3079245	0.807769	-0.30798529	0.807	0.42	down	no
Manes.08G032	2.380223134	0.924558	2.574444	1.36426107	0.336	0.73	up	no
Manes.08G032	50.46330099	107.21051	0.470694	-1.0871398	#####	0.01	down	no
Manes.08G037	1.036873161	0.9286017	1.116596	0.15910759	1	0.9	up	no
Manes.08G041	6.066885818	5.2734225	1.150465	0.20221657	0.821	0.56	up	no
Manes.08G041	29.2044672	21.623217	1.350607	0.43360791	0.469	0.44	up	no
Manes.08G041	5.641664842	5.6049322	1.006554	0.00942405	1	0.75	down	no
Manes.08G045	1.062444012	1.2410703	0.856071	-0.22419797	1	0.61	up	no
Manes.08G046	2.806745553	5.2741391	0.532171	-0.91003733	0.36	0.57	up	no
Manes.08G047	303.9253731	253.35445	1.199605	0.26255994	0.13	0.04	up	no
Manes.08G047	1.467299905	0	Inf	Inf	0.128	0.23	up	no
Manes.08G048	23.51938473	16.503761	1.425092	0.51105547	0.387	0.4	up	no
Manes.08G048	129.2537549	144.93129	0.891828	-0.16516294	0.562	0.01	up	yes
Manes.08G048	11.9409178	17.461385	0.683847	-0.54825428	0.307	0.09	down	no
Manes.08G049	15.91759654	26.483253	0.601044	-0.73445783	0.151	0	down	yes
Manes.08G050	17.3324533	13.057547	1.32739	0.40859196	0.416	0.07	up	no
Manes.08G050	877.4198464	848.44329	1.034153	0.04844911	0.739	0.41	up	no
Manes.08G050	147.7450907	149.41486	0.988825	-0.01621343	0.895	0	up	yes
Manes.08G051	25.39934918	12.084209	2.101863	1.07166848	0.023	0.85	up	no
Manes.08G052	741.6698411	653.6075	1.134733	0.18235256	0.174	0	down	yes
Manes.08G054	1.137855125	0	Inf	Inf	0.207	NA	--	no

Manes.08G055	108.6142128	35.854699	3.029288	1.59897878	#####	0	up	yes
Manes.08G055	2.173426843	0	Inf	Inf	0.041	1	up	no
Manes.08G056	675.9220315	122.64979	5.510992	2.4623121	#####	0	up	yes
Manes.08G057	102.5295648	73.958592	1.38631	0.47125031	0.065	0.7	up	no
Manes.08G058	8.858098651	5.3060279	1.669441	0.73936482	0.273	0.01	up	yes
Manes.08G060	400.7539033	77.820086	5.149749	2.36450209	#####	1	up	no
Manes.08G063	115.1357718	102.518	1.123079	0.16745889	0.495	0.27	down	no
Manes.08G064	180.3740048	147.50902	1.2228	0.29018827	0.164	0.58	up	no
Manes.08G066	214.4749546	213.27771	1.005614	0.00807602	1	0.37	down	no
Manes.08G067	7.816393127	6.2298693	1.254664	0.32730114	0.67	0.88	down	no
Manes.08G067	113.6105572	186.44797	0.609342	-0.71467617	0.001	0.51	down	no
Manes.08G069	233.3568074	461.46229	0.50569	-0.98367519	#####	0	down	no
Manes.08G069	257.3141387	229.10519	1.123127	0.16752058	0.358	0.88	up	no
Manes.08G070	9.20828192	14.693188	0.626704	-0.67414355	0.242	0.58	up	no
Manes.08G070	27.47179406	6.5518584	4.192977	2.06797505	#####	0.04	up	yes
Manes.08G070	217.5955892	1.5359309	141.6702	7.14639221	#####	0.41	up	no
Manes.08G071	101.118239	87.07444	1.161285	0.21572205	0.383	0.02	up	no
Manes.08G072	118.9448171	150.04831	0.79271	-0.33513469	0.133	0.05	up	no
Manes.08G072	15.78230704	25.795255	0.61183	-0.7087976	0.103	0.68	down	no
Manes.08G073	18.20716466	3.3802866	5.386278	2.42928878	0.004	0	up	yes
Manes.08G075	501.2543551	419.77782	1.194094	0.25591694	0.167	0.86	up	no
Manes.08G078	420.1204325	280.55882	1.497442	0.58249968	0.001	0.18	up	no
Manes.08G079	19013.22336	5389.637	3.527737	1.81874313	#####	0.01	down	no
Manes.08G079	405.1119009	37.995349	10.66214	3.41442574	#####	0	up	yes
Manes.08G079	1.467299905	1.8395955	0.797621	-0.32622477	0.968	1	down	no
Manes.08G079	38.32209411	36.695261	1.044334	0.06258263	0.89	0.94	up	no
Manes.08G080	12.99852945	4.3448208	2.99173	1.58097984	0.014	0.48	down	no
Manes.08G082	3.338154262	0.9381223	3.558336	1.83120277	0.16	0.56	down	no
Manes.08G082	0.329444781	1.2282226	0.268229	-1.89846349	0.547	0.75	down	no
Manes.08G084	366.5408241	328.92371	1.114364	0.1562209	0.307	0.85	down	no
Manes.08G085	489.6876374	340.62192	1.437628	0.52369048	6E-04	0.79	down	no
Manes.08G086	3.188633479	0.3036646	10.50051	3.39238768	0.036	1	down	no
Manes.08G087	215.8532744	286.584	0.753194	-0.40890708	0.028	0	down	yes
Manes.08G088	2.198997695	2.206321	0.996681	-0.00479662	1	1	down	no
Manes.08G088	168.2855069	170.60579	0.9864	-0.01975569	0.959	0.7	down	no
Manes.08G088	68.04941456	56.652351	1.201175	0.26444692	0.344	0.51	down	no
Manes.08G089	28.54167331	55.316331	0.515972	-0.95463556	0.005	0	down	yes
Manes.08G089	1.088014864	2.5140293	0.432777	-1.20830319	0.46	0.14	down	no
Manes.08G091	64.47275935	65.864609	0.978868	-0.03081375	0.95	0.51	up	no
Manes.08G093	328.5277481	254.16195	1.292592	0.37026717	0.029	0	down	no
Manes.08G095	103.9020165	184.92913	0.561848	-0.83174886	1E-03	0	down	yes
Manes.08G096	0.758570083	4.6342045	0.163689	-2.61096734	0.197	0.63	down	no
Manes.08G097	44.361553	2.1751488	20.39472	4.35012385	#####	0.04	up	yes
Manes.08G098	5.995379032	6.2034574	0.966458	-0.04922153	1	0.82	up	no
Manes.08G098	107.2792261	69.109254	1.552313	0.63441993	0.03	0.1	up	no
Manes.08G102	1.112284273	2.1927568	0.507254	-0.97922023	0.584	0.39	down	no
Manes.08G102	76.93886747	0.3124686	246.2292	7.94385777	#####	1	up	no
Manes.08G103	81.06961054	2.1784759	37.21391	5.21777014	#####	0.17	up	no

Manes.08G104	47.1064565	74.07333	0.635944	-0.65302938	0.057	0.51	down	no
Manes.08G105	125.9116964	119.36443	1.054851	0.07703932	0.727	0	up	yes
Manes.08G106	6.297951519	10.923091	0.576572	-0.79442662	0.243	0.47	up	no
Manes.08G107	9.767490959	15.636071	0.624677	-0.67881808	0.243	0.78	up	no
Manes.08G108	27.47272209	16.492807	1.66574	0.73616287	0.079	0.75	up	no
Manes.08G110	42.81037111	78.730589	0.543758	-0.87896393	0.004	0.31	down	no
Manes.08G110	451.0021753	233.39593	1.932348	0.95035499	#####	0	down	no
Manes.08G112	3.039112696	0.6161331	4.932558	2.30233612	0.099	0.22	up	no
Manes.08G113	41.43335281	19.516606	2.122979	1.08609041	0.026	0	up	yes
Manes.08G113	4.53068201	18.592048	0.243689	-2.03688552	8E-04	0.07	down	no
Manes.08G116	5.945538771	17.032315	0.349074	-1.51839511	0.01	0.95	down	no
Manes.08G116	2731.599929	1408.4097	1.939492	0.95567913	#####	0.12	up	no
Manes.08G119	43.26376582	102.02825	0.424037	-1.23773745	#####	0.09	down	no
Manes.08G121	1.845283505	1.8755279	0.983874	-0.02345426	1	0.72	down	no
Manes.08G123	0.758570083	1.5494951	0.48956	-1.03044384	0.815	0.86	down	no
Manes.08G125	16.85991037	4.631594	3.640196	1.86401615	0.055	0.68	up	no
Manes.08G126	9.382072112	20.557048	0.456392	-1.13165464	0.025	0.21	up	no
Manes.08G128	12.97909241	10.911677	1.189468	0.25031671	0.696	1	down	no
Manes.08G129	23.77862416	27.419226	0.867225	-0.20552258	0.636	0.12	down	no
Manes.08G132	21.20034143	17.115134	1.23869	0.30881491	0.553	0.34	up	no
Manes.08G143	50.00738798	42.504148	1.17653	0.23453761	0.488	0.38	up	no
Manes.08G144	78.62438782	73.846035	1.064707	0.09045643	0.75	0.47	up	no
Manes.08G144	30.47752725	21.784554	1.399043	0.48444025	0.226	0.15	up	no
Manes.08G144	237.1769771	2256.8088	0.105094	-3.25024831	#####	0	up	yes
Manes.08G147	17.27740727	4.3367334	3.983968	1.99420607	7E-04	0	up	yes
Manes.08G147	33.05971431	12.785771	2.585664	1.37053506	0.001	0	up	yes
Manes.08G148	46.34751301	24.237673	1.91221	0.93524076	0.008	0	up	yes
Manes.08G148	1904.573596	929.2432	2.049596	1.0353399	0.051	0.6	up	no
Manes.08G149	26.42682342	25.268595	1.045837	0.06465759	0.83	0.02	down	yes
Manes.08G149	23.65988	16.19247	1.461166	0.5471197	0.217	0.36	down	no
Manes.08G151	0.658889561	4.0052237	0.164508	-2.60377426	0.169	0.79	down	no
Manes.08G152	569.9286526	184.9156	3.082102	1.6239144	#####	1	up	no
Manes.08G153	96.39926967	92.863108	1.038079	0.05391664	0.875	0.02	down	no
Manes.08G153	66.73200884	100.67006	0.662878	-0.59318387	0.024	0	down	yes
Manes.08G154	1.391888793	1.5725799	0.885099	-0.1760894	1	0.32	down	no
Manes.08G156	24.56629602	21.789315	1.127447	0.17305953	0.677	0.89	down	no
Manes.08G156	404.5296152	299.85769	1.349072	0.43196735	0.01	0.13	down	no
Manes.08G157	292.7081292	283.73731	1.031617	0.04490693	0.77	0.07	up	no
Manes.08G159	1.720032131	1.85792	0.925784	-0.11125286	1	0	up	yes
Manes.08G160	2.049476912	2.1879965	0.936691	-0.09435468	1	0.99	up	no
Manes.08G161	32.17849574	21.537757	1.49405	0.57922886	0.175	0.18	up	no
Manes.08G162	35.52668813	49.333976	0.720126	-0.47367835	0.165	0.77	up	no
Manes.08G168	28.96336337	29.96325	0.96663	-0.04896496	0.917	0.01	down	yes
Manes.08G168	32.58688255	23.405198	1.392293	0.47746238	0.202	0.51	up	no
Manes.08G169	19.01817789	19.837162	0.958715	-0.06082658	0.898	0.07	up	no
Manes.08G171	26.66301031	4.3536248	6.124324	2.6145507	#####	0	up	yes
Manes.08G173	6.350394664	0.6344577	10.00917	3.32325044	0.072	0.08	up	no
Manes.09G003	8.399871576	22.330256	0.376165	-1.4105606	0.006	0.51	down	no

Manes.09G004	3.008709481	0	Inf	Inf	0.014	NA	--	no
Manes.09G005	10.47752222	7.498068	1.397363	0.48270676	0.421	0.03	up	yes
Manes.09G007	57.7236426	59.904622	0.963592	-0.05350498	0.931	0.41	up	no
Manes.09G009	10.54772757	1.8660074	5.652565	2.49890563	0.062	0.4	up	no
Manes.09G009	158.7629516	0.3036646	522.8234	9.03017989	#####	0	up	yes
Manes.09G009	100.7472326	14.72439	6.842201	2.7744604	6E-04	0	up	yes
Manes.09G011	44.41882851	31.062484	1.429983	0.51599814	0.171	0.56	down	no
Manes.09G011	1.491569315	1.5271269	0.976716	-0.03398894	1	0.32	down	no
Manes.09G014	53.45870723	49.768522	1.074147	0.10319139	0.715	0.38	down	no
Manes.09G014	43.35181795	38.203347	1.134765	0.18239345	0.597	0.58	up	no
Manes.09G015	9.968153445	14.293141	0.697408	-0.51992476	0.386	0.2	down	no
Manes.09G016	9.413776769	8.0782687	1.165321	0.22072751	0.737	0.16	down	no
Manes.09G018	14.31630857	14.259358	1.003994	0.00575053	1	0.04	up	yes
Manes.09G020	16.97093046	14.57587	1.164317	0.21948366	0.68	0	up	yes
Manes.09G020	34.16493674	37.176469	0.918994	-0.12187328	0.818	0.98	up	no
Manes.09G021	17.27294832	16.474483	1.048467	0.06828118	0.86	0.01	up	yes
Manes.09G023	892.5430717	669.20437	1.333738	0.41547492	0.002	0	up	no
Manes.09G023	0.329444781	2.8081733	0.117316	-3.0915234	0.121	0.99	up	no
Manes.09G024	1.366317941	0	Inf	Inf	0.131	1	up	no
Manes.09G025	2.809348437	1.5766236	1.781876	0.83339728	0.53	1	up	no
Manes.09G026	164.3408216	316.61968	0.519048	-0.94606007	#####	0.9	down	no
Manes.09G027	4.984076722	4.0459165	1.231878	0.30085974	0.781	0.92	down	no
Manes.09G029	22.15659771	19.043486	1.163474	0.21843879	0.612	0.68	up	no
Manes.09G029	171.7086524	91.24011	1.881943	0.91222264	0.005	0.21	up	no
Manes.09G029	2.428761953	3.4121754	0.711793	-0.49047071	0.769	0.27	up	no
Manes.09G029	6.018346999	2.8319747	2.125142	1.08755891	0.323	0.82	up	no
Manes.09G030	697.038033	678.2462	1.027707	0.03942833	0.78	0.01	up	no
Manes.09G033	1.417459644	2.8048462	0.505361	-0.98461401	0.519	0.59	down	no
Manes.09G034	1.06114257	1.2546345	0.845778	-0.24164864	1	0.08	up	no
Manes.09G034	38895.98062	60.209464	646.0111	9.33541509	#####	0.03	up	no
Manes.09G038	330.9211669	251.76119	1.314425	0.39443165	0.018	0	down	yes
Manes.09G039	43.39581322	43.773063	0.991382	-0.01248749	0.974	0.01	down	yes
Manes.09G040	24.06436249	22.988259	1.046811	0.066001	0.869	0.68	down	no
Manes.09G042	12.72896306	10.285306	1.237587	0.30753014	0.595	0.1	up	no
Manes.09G042	1.137855125	0	Inf	Inf	0.207	0.01	up	yes
Manes.09G047	1.417459644	0.933362	1.51866	0.60279905	0.85	0.94	down	no
Manes.09G049	56.4881552	45.807093	1.233175	0.30237738	0.445	0.45	up	no
Manes.09G049	90.40704815	187.03321	0.483374	-1.04878734	#####	0	up	no
Manes.09G051	5.617395432	6.8636103	0.818432	-0.28906629	0.789	0.89	up	no
Manes.09G056	8.420236659	0.3036646	27.72874	4.79331019	#####	0.05	up	no
Manes.09G056	12.98039385	23.940662	0.54219	-0.88312889	0.062	0	up	yes
Manes.09G057	0.708729822	1.2146584	0.583481	-0.77724293	0.745	0.41	down	no
Manes.09G058	16.64669145	15.263152	1.090646	0.12518258	0.825	0.24	down	no
Manes.09G058	2.025207502	1.2546345	1.614181	0.69080259	0.72	0.6	up	no
Manes.09G063	4.400598274	2.169672	2.028232	1.02022275	0.535	0.94	down	no
Manes.09G064	390.2071498	305.21368	1.278472	0.3544206	0.029	0.1	down	no
Manes.09G065	22.36209255	77.51332	0.288494	-1.79338905	#####	0.11	down	no
Manes.09G066	3.543649111	1.2458305	2.844407	1.50812792	0.201	1	down	no

Manes.09G067	51.0966197	56.685212	0.90141	-0.14974457	0.675	0.65	down	no
Manes.09G068	3.919029826	4.3455374	0.901852	-0.14903804	0.98	0	up	yes
Manes.09G068	1226.492142	0	Inf	Inf	#####	0.56	up	no
Manes.09G069	22.31392714	21.708646	1.027882	0.03967471	0.969	0.89	down	no
Manes.09G069	15.00207043	11.794825	1.27192	0.34700757	0.54	0.39	down	no
Manes.09G070	1.137855125	0	Inf	Inf	0.207	NA	--	no
Manes.09G070	2.025207502	2.1751488	0.931066	-0.10304438	1	0.05	up	no
Manes.09G072	20.50677092	15.543731	1.319295	0.39976746	0.46	0.84	down	no
Manes.09G073	1.491569315	5.9628537	0.250144	-1.99917193	0.113	0.03	down	yes
Manes.09G073	1300.945034	91.234838	14.2593	3.83383137	#####	0	up	yes
Manes.09G074	2.478602214	0.3124686	7.932325	2.98774384	0.088	1	down	no
Manes.09G074	691.9153786	392.28234	1.76382	0.8187032	#####	0	up	yes
Manes.09G078	741.3919476	313.4263	2.365443	1.24211021	#####	0.05	up	no
Manes.09G078	163.2478668	144.66517	1.128453	0.17434653	0.431	0.52	up	no
Manes.09G078	23.98932478	4.6979821	5.106304	2.35227944	#####	0	up	yes
Manes.09G078	1.441729054	0	Inf	Inf	0.129	1	up	no
Manes.09G081	161.1152887	103.74003	1.553068	0.63512071	0.013	0.93	down	no
Manes.09G081	46.02876858	33.512275	1.37349	0.45784622	0.195	0.63	up	no
Manes.09G083	43.42974736	35.690751	1.216835	0.2831333	0.416	0.43	down	no
Manes.09G084	2.529743918	5.5547189	0.455422	-1.13472255	0.229	0.84	down	no
Manes.09G084	47.04918099	68.01478	0.691749	-0.53167867	0.071	0.32	down	no
Manes.09G085	46.05850957	25.851406	1.781664	0.83322499	0.024	0.02	down	yes
Manes.09G087	640.5485522	764.32986	0.838053	-0.25488747	0.127	0	down	no
Manes.09G092	105.5103664	16.353838	6.451719	2.68968361	#####	0.93	up	no
Manes.09G095	72.94543915	55.319914	1.318611	0.39901886	0.174	0	up	yes
Manes.09G096	0	2.1839528	#NAME?	#NAME?	0.062	0.17	down	no
Manes.09G096	30.33060935	15.913323	1.905988	0.93053933	0.023	0	up	yes
Manes.09G097	15.60591396	14.348831	1.087609	0.12115967	0.837	0	up	yes
Manes.09G098	50.69251062	1.2593948	40.25149	5.33097012	#####	0.07	up	no
Manes.09G098	930.0707768	1353.182	0.687321	-0.54094353	0.001	0.38	up	no
Manes.09G102	2442.701989	724.5016	3.371562	1.75341708	#####	0	down	no
Manes.09G102	16.69523027	30.430176	0.548641	-0.86606669	0.039	0.76	down	no
Manes.09G104	6.576254596	6.5478147	1.004343	0.00625266	0.976	0.33	down	no
Manes.09G105	9.539028142	7.1361028	1.336728	0.4187059	0.532	0.73	down	no
Manes.09G107	114.9931546	122.56763	0.938202	-0.09203009	0.686	0.02	down	no
Manes.09G107	114.0840281	95.625829	1.193025	0.25462458	0.272	0.48	down	no
Manes.09G108	0.758570083	3.7462956	0.202485	-2.30411035	0.222	0.28	up	no
Manes.09G109	241.9343119	262.33609	0.92223	-0.11680089	0.576	0	up	yes
Manes.09G110	143.8787698	142.51548	1.009566	0.01373509	0.954	0.01	down	no
Manes.09G110	125.4556988	111.42252	1.125946	0.17113715	0.44	0.04	down	no
Manes.09G110	1.038174603	2.8000859	0.370765	-1.43142201	0.352	0.56	down	no
Manes.09G111	101.9803093	42.760691	2.384908	1.25393353	8E-04	0.48	up	no
Manes.09G111	42.61687806	28.781657	1.480696	0.56627524	0.258	0.47	up	no
Manes.09G112	42.09484525	22.038006	1.910102	0.9336499	0.021	0.46	down	no
Manes.09G112	1.112284273	0	Inf	Inf	0.238	0.34	up	no
Manes.09G113	15.98259612	1.2641551	12.64291	3.66025643	9E-04	0.11	up	no
Manes.09G114	106.7687251	447.30059	0.238696	-2.06675554	#####	0	down	yes
Manes.09G114	203.1788763	150.5659	1.349435	0.43235531	0.063	0.02	up	no

Manes.09G117	14.97872906	22.383796	0.669177	-0.57953952	0.232	0.11	down	no
Manes.09G118	2.579584179	2.1927568	1.176411	0.23439274	0.925	0.11	up	no
Manes.09G126	159.8015721	151.10278	1.057569	0.08075139	0.693	0.9	down	no
Manes.09G126	119.4742622	136.63972	0.874374	-0.19367711	0.427	0.17	down	no
Manes.09G128	169.3744498	148.80721	1.138214	0.1867718	0.351	0	up	no
Manes.09G130	19.85438857	26.977308	0.735966	-0.44228846	0.295	0.3	down	no
Manes.09G133	125.9524265	235.52015	0.534784	-0.90297157	#####	0.01	down	no
Manes.09G136	1.795443244	0.6296974	2.851279	1.51160945	0.414	1	down	no
Manes.09G136	331.0215943	65.143259	5.08144	2.34523753	#####	1	up	no
Manes.09G138	34462.0071	7018.2003	4.910377	2.2958337	#####	0.12	up	no
Manes.09G138	14.46936028	24.177939	0.598453	-0.74069011	0.106	1	up	no
Manes.09G139	226.4539997	215.84528	1.04915	0.0692205	0.767	0	down	yes
Manes.09G140	638.2886295	3508.8261	0.181909	-2.45870761	#####	0	up	no
Manes.09G141	107.5625537	122.28997	0.87957	-0.1851302	0.492	0.73	down	no
Manes.09G141	245.1691241	215.05882	1.14001	0.18904602	0.328	0	up	yes
Manes.09G148	11.35613791	3.1125545	3.648494	1.86730127	0.007	0.85	up	no
Manes.09G150	4.930705541	0	Inf	Inf	0.001	NA	--	no
Manes.09G159	1.088014864	1.8714842	0.581365	-0.78248463	0.739	0.64	up	no
Manes.09G161	1.41485676	2.7777177	0.509359	-0.973244	0.758	1	down	no
Manes.09G166	0.329444781	5.2517709	0.06273	-3.9946954	0.006	0.08	down	no
Manes.09G169	1.012603751	4.0330688	0.251075	-1.99380829	0.122	0.67	down	no
Manes.09G170	42.45795836	28.615559	1.483737	0.56923519	0.101	1	down	no
Manes.09G170	64.47247053	65.221348	0.988518	-0.01666099	1	0	down	yes
Manes.09G170	8.655206686	5.2429669	1.650822	0.72318483	0.358	0.11	up	no
Manes.09G171	6603.284946	196.10693	33.67186	5.07347145	#####	0	up	yes
Manes.09G175	1.086713422	1.8850485	0.576491	-0.7946301	0.736	0.18	up	no
Manes.09G175	15.17939154	11.518289	1.317851	0.39818751	0.45	0	up	yes
Manes.09G177	214.5561261	201.48483	1.064875	0.0906839	0.643	0.56	down	no
Manes.09G177	65.06497449	60.16091	1.081516	0.11305475	0.672	0	down	yes
Manes.09G181	54.24192013	98.766228	0.549195	-0.86460957	0.002	0.06	up	no
Manes.09G181	2.124888024	0.6161331	3.448748	1.78607276	0.284	0.12	up	no
Manes.09G183	1225.776519	852.58719	1.437714	0.5237767	#####	0.97	down	no
Manes.09G184	1.467299905	0.9421659	1.557369	0.63911069	0.823	0	up	yes
Manes.09G185	17.63725527	44.77783	0.393884	-1.34415854	0.002	0.93	up	no
Manes.09G185	79.63736498	52.424132	1.519097	0.60321442	0.044	0.9	down	no
Manes.10G002	248.6495682	268.93013	0.924588	-0.11311749	0.565	0.73	down	no
Manes.10G004	95.83233656	109.06768	0.87865	-0.18663919	0.454	0.1	down	no
Manes.10G004	31.53447667	12.429283	2.537111	1.34318692	0.002	0.21	up	no
Manes.10G005	11.9677901	21.766946	0.549815	-0.86298226	0.078	0	down	yes
Manes.10G011	3.843618714	3.4433476	1.116245	0.15865341	0.901	0.98	down	no
Manes.10G014	1.647223903	4.9935593	0.32987	-1.60003183	0.393	0.96	down	no
Manes.10G019	75.36528319	66.491696	1.133454	0.18072592	0.579	0.05	down	no
Manes.10G021	1.794141802	0.6296974	2.849213	1.51056333	0.401	0	up	yes
Manes.10G022	1.342048532	2.4740531	0.542449	-0.88243964	0.646	0.85	down	no
Manes.10G023	1.062444012	0	Inf	Inf	0.239	1	up	no
Manes.10G023	7.840662537	7.8241008	1.002117	0.00305061	1	0.18	down	no
Manes.10G025	25.65077996	28.341173	0.905071	-0.14389678	0.734	0.65	up	no
Manes.10G028	1.088014864	0	Inf	Inf	0.238	1	up	no

Manes.10G028	17.02727793	19.057767	0.893456	-0.16253124	0.777	0.08	up	no
Manes.10G028	1.366317941	3.761293	0.363258	-1.46093546	0.292	1	up	no
Manes.10G028	116.5658955	51.683566	2.255376	1.17336824	3E-04	0	up	yes
Manes.10G033	256.1557493	243.21956	1.053187	0.07476201	0.682	0.19	up	no
Manes.10G037	42.73886432	178.84689	0.238969	-2.06510457	#####	0	down	yes
Manes.10G037	19.97154247	74.861264	0.266781	-1.90627364	0.003	0.17	down	no
Manes.10G037	69.60606803	69.257283	1.005036	0.00724729	0.963	0.01	down	yes
Manes.10G038	6.936475996	9.638001	0.719701	-0.47453105	0.587	0.23	down	no
Manes.10G041	13.38264686	22.6244	0.591514	-0.75751604	0.109	0.52	up	no
Manes.10G042	12.64964762	10.293394	1.228909	0.29737849	0.596	0	up	yes
Manes.10G042	7.313158154	4.9561937	1.475559	0.561262	0.457	0	up	yes
Manes.10G042	91.61184351	116.69999	0.78502	-0.34919837	0.17	0.42	down	no
Manes.10G045	178.3466524	122.87893	1.451401	0.53744663	0.012	0	up	no
Manes.10G046	27.25849055	6.5559021	4.157855	2.05583947	#####	0.47	up	no
Manes.10G047	136.3006118	125.34943	1.087365	0.12083665	0.641	0.48	up	no
Manes.10G047	103.9214536	85.284116	1.218532	0.28514455	0.29	0.17	up	no
Manes.10G047	14.74636191	13.608009	1.083653	0.11590308	0.894	0.09	up	no
Manes.10G047	6.326125254	2.4876174	2.543046	1.34655752	0.112	0.02	up	yes
Manes.10G049	8.222550462	2.4964214	3.293735	1.7197245	0.03	0	up	yes
Manes.10G049	52.10606594	51.143085	1.018829	0.02691214	0.972	0	up	yes
Manes.10G049	881.9119673	8.0430528	109.6489	6.77674764	#####	0	up	yes
Manes.10G049	37.84573143	98.886157	0.38272	-1.38563798	#####	1	up	no
Manes.10G051	178.1799241	117.62122	1.514862	0.59918647	0.005	0.12	down	no
Manes.10G051	11.25775883	9.56803	1.176602	0.23462583	0.854	0.86	up	no
Manes.10G053	20.74434383	22.400688	0.926058	-0.11082499	0.854	0.42	down	no
Manes.10G053	13.81437504	9.6413281	1.432829	0.5188665	0.352	0.12	up	no
Manes.10G053	5.484708812	8.6496654	0.634095	-0.6572293	0.455	0.01	up	yes
Manes.10G058	41.54633657	36.340667	1.143246	0.19313638	0.579	0.01	down	yes
Manes.10G058	0.658889561	1.2458305	0.528876	-0.91899926	0.958	0	up	yes
Manes.10G059	3.037811254	0.933362	3.254698	1.70252363	0.177	0.56	down	no
Manes.10G060	12.54903907	11.798152	1.063644	0.08901595	0.905	0.1	up	no
Manes.10G062	82.33717599	55.813222	1.475227	0.56093703	0.053	0.15	down	no
Manes.10G062	35.39846047	33.839024	1.046084	0.06499864	0.835	0.3	down	no
Manes.10G064	191.658613	114.96965	1.667037	0.73728575	4E-04	0.55	up	no
Manes.10G066	1.062444012	2.1799091	0.48738	-1.03688117	0.581	0.72	up	no
Manes.10G067	6.171772108	9.2753191	0.665397	-0.58771213	0.45	0.64	up	no
Manes.10G068	2.907727517	0.6208934	4.683135	2.22747452	0.129	1	down	no
Manes.10G071	52.60733724	50.099316	1.050061	0.07047314	0.809	0.87	up	no
Manes.10G074	5.543285762	9.9361887	0.557889	-0.84195119	0.24	0.54	down	no
Manes.10G075	126.4397554	266.61503	0.474241	-1.07630798	#####	0	up	no
Manes.10G078	276.4293447	363.59424	0.760269	-0.39541858	0.087	0.56	up	no
Manes.10G079	2.907727517	2.1839528	1.331406	0.41295041	0.811	0.77	up	no
Manes.10G080	3.972774413	3.1173148	1.274422	0.34984303	0.743	0.74	up	no
Manes.10G081	65.32811825	129.87155	0.503021	-0.99130949	1E-04	0.24	down	no
Manes.10G082	21.30615575	28.914464	0.736868	-0.44052103	0.31	0.01	down	yes
Manes.10G083	120.7082669	110.57508	1.091641	0.12649822	0.583	0.2	down	no
Manes.10G083	16.80104459	38.476556	0.436657	-1.19542874	0.003	0.43	down	no
Manes.10G084	26.66951752	47.81207	0.557799	-0.84218314	0.019	0.3	up	no

Manes.10G086	69.87470638	79.751274	0.876158	-0.19073725	0.505	0.2	down	no
Manes.10G086	43.80000689	33.889698	1.292428	0.3700843	0.269	0	up	yes
Manes.10G091	49.76973049	72.748694	0.684132	-0.54765278	0.136	0.09	up	no
Manes.10G093	16.94275672	3.4393039	4.926217	2.30048013	2E-04	0.02	up	yes
Manes.10G094	39.52335855	15.268628	2.588534	1.37213507	4E-04	0	up	yes
Manes.10G098	5.816756476	4.3360168	1.341498	0.42384455	0.67	0.75	down	no
Manes.10G099	2.833617847	3.0894697	0.917186	-0.12471402	0.989	0.8	down	no
Manes.10G099	1.821014095	0	Inf	Inf	0.072	0.21	up	no
Manes.10G099	6.167867782	0.6073292	10.15572	3.34422125	0.087	0.72	down	no
Manes.10G099	9.174902416	0	Inf	Inf	#####	NA	--	no
Manes.10G100	6.826757343	0	Inf	Inf	#####	NA	--	no
Manes.10G100	1.06114257	0	Inf	Inf	0.218	0.34	up	no
Manes.10G100	2.477300773	0.9381223	2.640701	1.40092118	0.33	0.56	down	no
Manes.10G101	1.41485676	1.5582991	0.907949	-0.13931616	1	0.3	down	no
Manes.10G101	47.77065942	155.41297	0.307379	-1.70191021	0.109	0	down	yes
Manes.10G102	542.3318114	333.28066	1.627253	0.7024382	#####	0.06	up	no
Manes.10G107	186.3600926	142.91979	1.303949	0.38288729	0.134	0	down	yes
Manes.10G107	1.745602983	0.3036646	5.748457	2.52317488	0.222	0.01	up	yes
Manes.10G108	2.578282737	0.3124686	8.251335	3.04462756	0.086	0.68	up	no
Manes.10G108	2.225869988	0.6249371	3.56175	1.83258641	0.503	1	up	no
Manes.10G108	1.367619383	1.8626803	0.734221	-0.44571329	0.958	1	down	no
Manes.10G109	10.49565783	8.7215303	1.203419	0.26713938	0.714	0.87	down	no
Manes.10G109	1.012603751	0	Inf	Inf	0.24	1	up	no
Manes.10G110	1.417459644	0	Inf	Inf	0.13	NA	--	no
Manes.10G110	103.9479525	123.49004	0.841752	-0.24853338	0.357	0.56	down	no
Manes.10G111	9.41154729	5.8774246	1.601305	0.67924781	0.361	0.39	up	no
Manes.10G116	119.565206	112.34636	1.064255	0.08984422	0.684	0	up	yes
Manes.10G116	4.151396969	3.0901863	1.343413	0.42590308	0.681	0	up	yes
Manes.10G118	1.088014864	0	Inf	Inf	0.238	NA	--	no
Manes.10G120	58.1172435	27.956123	2.078874	1.05580198	0.001	0.95	up	no
Manes.10G120	21.55182614	29.625086	0.727486	-0.45900922	0.353	0.07	up	no
Manes.10G120	21.21550074	13.692721	1.5494	0.63170952	0.199	0.04	up	yes
Manes.10G122	1.317779122	0.933362	1.411863	0.49759997	0.745	0	up	yes
Manes.10G129	43.44759414	9.932145	4.374442	2.12909906	#####	0.13	up	no
Manes.10G132	7.917375091	4.3502977	1.819962	0.86390808	0.253	0.04	up	yes
Manes.10G133	0.379285042	2.4876174	0.152469	-2.71341022	0.137	1	down	no
Manes.10G136	267.1854924	295.98406	0.902702	-0.14767779	0.47	0.01	up	no
Manes.10G137	1.112284273	0.6161331	1.805266	0.8522115	0.862	0.75	down	no
Manes.10G139	63.27919597	46.368017	1.364716	0.44860123	0.212	0	up	yes
Manes.10G139	21.62203148	12.984966	1.665159	0.73565988	0.232	0.63	down	no
Manes.10G139	63.9020065	40.061984	1.595078	0.67362737	0.042	0.17	down	no
Manes.10G142	15.6340877	11.815044	1.323236	0.40407007	0.498	0.01	down	yes
Manes.10G143	200.053013	146.82816	1.362498	0.4462537	0.026	0	up	yes
Manes.10G144	8.120267056	13.641792	0.595249	-0.74843404	0.205	0.03	up	yes
Manes.10G144	4.833254497	0.6208934	7.784354	2.9605774	0.012	0.02	up	yes
Manes.10G144	17.27387635	39.773316	0.434308	-1.203209	0.004	0	up	yes
Manes.10G147	0	1.2410703	#NAME?	#NAME?	0.23	0.93	up	no
Manes.10G148	13.63184816	0	Inf	Inf	#####	0	up	yes

Manes.10G148	73.90420167	13.625617	5.423916	2.43933485	#####	0	up	yes
Manes.10G148	116.2003716	24.77027	4.691122	2.22993316	#####	0	up	yes
Manes.10G149	1.088014864	0.6073292	1.791475	0.84114767	1	1	down	no
Manes.10G150	27.9707513	17.434973	1.60429	0.68193485	0.102	0	down	yes
Manes.10G152	114.8707719	56.689491	2.026315	1.01885854	0.001	0.01	down	yes
Manes.11G000	22.7925193	35.774747	0.637112	-0.65038116	0.101	0.49	down	no
Manes.11G001	19.30521766	19.940199	0.968156	-0.04668896	0.991	0.46	down	no
Manes.11G001	1.137855125	1.2458305	0.913331	-0.13079096	1	0.02	up	yes
Manes.11G003	158.722764	74.803854	2.121853	1.08532455	0.002	0.85	down	no
Manes.11G005	36.24444346	9.6596526	3.752148	1.90771663	#####	0	up	yes
Manes.11G006	16.73306873	41.556044	0.402663	-1.31235629	0.02	0	down	yes
Manes.11G006	17.10882285	0.9374057	18.25125	4.18992318	0.009	0.09	up	no
Manes.11G006	96.02357712	20.499464	4.684199	2.22780247	#####	0	up	yes
Manes.11G007	30.75713177	9.9585569	3.088513	1.62691237	2E-04	0.05	up	yes
Manes.11G010	3.870491007	1.8890922	2.048863	1.0348235	0.356	1	up	no
Manes.11G011	4.02001179	3.9788118	1.010355	0.01486206	0.951	0.87	down	no
Manes.11G011	25.92610675	0.3172288	81.72683	6.35273785	#####	0.68	up	no
Manes.11G012	0.329444781	2.5011816	0.131716	-2.92450125	0.136	0.3	up	no
Manes.11G013	23.24591401	7.1936868	3.231433	1.69217389	0.013	0.73	down	no
Manes.11G015	12.0370674	44.695185	0.269315	-1.89263548	0.031	1	up	no
Manes.11G017	25.49055882	49.40108	0.515992	-0.95457959	0.044	0.14	down	no
Manes.11G018	1.416158202	1.8355518	0.771516	-0.37423138	0.869	0.51	down	no
Manes.11G021	405.9348688	503.3939	0.806396	-0.31043946	0.038	0.01	down	no
Manes.11G021	9.896646658	14.035646	0.705108	-0.50408375	0.52	0.75	down	no
Manes.11G023	321.5073902	314.91877	1.020922	0.02987214	0.833	0	down	no
Manes.11G023	260.2700546	296.04925	0.879144	-0.18582785	0.27	0.49	down	no
Manes.11G024	419.8362724	680.41044	0.617034	-0.69657847	#####	0	down	no
Manes.11G027	255.2019037	54.38773	4.69227	2.23028599	#####	0.01	down	yes
Manes.11G028	1.794141802	0.9421659	1.904274	0.92924083	0.598	1	down	no
Manes.11G029	5.617395432	0.3124686	17.97747	4.16811845	0.002	0	up	yes
Manes.11G030	8.727086877	3.148487	2.771835	1.47084147	0.176	0.08	up	no
Manes.11G033	12.69725841	27.968254	0.453988	-1.13927317	0.045	0.48	down	no
Manes.11G036	18.09195142	26.549641	0.681439	-0.55334433	0.229	0.15	down	no
Manes.11G045	1.036873161	0.6344577	1.634267	0.7086436	1	1	up	no
Manes.11G047	3.113222366	11.083026	0.2809	-1.83187128	0.029	0.69	down	no
Manes.11G053	14.60074546	16.164625	0.903253	-0.14679799	0.852	0.49	down	no
Manes.11G054	82.61808195	5.6137361	14.71713	3.87942441	#####	0.62	up	no
Manes.11G055	22.05821863	35.777357	0.616541	-0.69773054	0.086	0.09	down	no
Manes.11G057	4.55495142	2.1839528	2.085646	1.06049399	0.272	0	up	yes
Manes.11G057	26.03229448	14.670103	1.774513	0.82742345	0.053	0.6	down	no
Manes.11G059	70.61747794	77.071863	0.916255	-0.12617897	0.678	0.06	down	no
Manes.11G068	210.8288299	186.46296	1.130674	0.17718305	0.343	0	down	no
Manes.11G069	4.753011022	0.3036646	15.65217	3.96829116	0.006	0	up	yes
Manes.11G071	19.25928172	16.495418	1.167553	0.22348857	0.627	0.02	down	yes
Manes.11G071	802.1959866	79.780327	10.05506	3.32984981	#####	0.97	down	no
Manes.11G072	123.397774	8.7174867	14.1552	3.82326031	#####	0	up	yes
Manes.11G072	325.048498	256.03909	1.269527	0.34429088	0.205	0.46	up	no
Manes.11G072	89.52917928	69.522609	1.287771	0.36487573	0.26	0	down	yes

Manes.11G073	0.732999232	1.5359309	0.477235	-1.0672297	0.663	0.11	up	no
Manes.11G074	1.417459644	0	Inf	Inf	0.13	NA	--	no
Manes.11G077	31.97820666	137.694	0.232241	-2.10630472	#####	0	down	yes
Manes.11G079	8.40247446	6.2298693	1.34874	0.43161236	0.527	0.62	down	no
Manes.11G079	134.5187376	100.88709	1.333359	0.41506558	0.067	0	down	yes
Manes.11G080	29.54646841	59.39557	0.497452	-1.00736962	0.003	0.96	down	no
Manes.11G080	33.51794139	21.078232	1.590169	0.6691797	0.087	0.12	down	no
Manes.11G081	169.8631877	214.03668	0.793617	-0.33348481	0.087	0.01	up	no
Manes.11G081	5.591824581	5.8869451	0.949869	-0.07420008	0.994	0.71	up	no
Manes.11G087	265.6631574	489.43822	0.542792	-0.88152859	#####	0.28	up	no
Manes.11G088	21.06505192	36.683847	0.574232	-0.80029346	0.038	0.79	up	no
Manes.11G091	386.063623	1078.3256	0.358021	-1.48188236	#####	0	up	yes
Manes.11G092	1227.209324	1597.6527	0.768133	-0.38057246	0.004	0.56	down	no
Manes.11G092	6.372061189	4.3679056	1.458837	0.54481845	0.501	0.25	down	no
Manes.11G093	14.85347768	16.437117	0.903655	-0.14615654	0.87	0.48	up	no
Manes.11G094	78.9179348	145.56355	0.542155	-0.88322404	3E-04	0	down	yes
Manes.11G098	161.598979	128.77611	1.254883	0.32755313	0.132	0.08	down	no
Manes.11G099	36.17033379	10.57684	3.419768	1.77389827	0.002	0.01	up	yes
Manes.11G102	4.833254497	4.9528666	0.97585	-0.03526879	1	0.44	up	no
Manes.11G107	6.096360996	5.8759914	1.037503	0.05311605	1	0.68	up	no
Manes.11G109	52.61896564	56.590007	0.929828	-0.10496442	0.753	0.03	down	yes
Manes.11G109	31.18700388	31.205497	0.999407	-0.00085524	1	0.21	down	no
Manes.11G110	13.43992236	3.4290667	3.919411	1.97063692	0.019	0	up	yes
Manes.11G112	4.860126791	10.920481	0.445047	-1.16797049	0.099	0.11	down	no
Manes.11G114	3988.575881	3180.4273	1.254101	0.32665312	0.257	0	up	yes
Manes.11G114	3037.923973	2306.9279	1.31687	0.39711284	0.243	0	up	yes
Manes.11G114	33.70660207	29.728614	1.13381	0.181179	0.624	0.05	up	no
Manes.11G118	26.18349011	29.405908	0.890416	-0.16744863	0.747	0	down	yes
Manes.11G118	6.427107218	9.9728378	0.644461	-0.63383454	0.38	0.06	down	no
Manes.11G123	4.62906109	4.6444417	0.996688	-0.00478558	1	0.41	down	no
Manes.11G125	33.54351224	35.028448	0.957608	-0.06249332	0.888	0.1	down	no
Manes.11G127	8.979819103	21.040866	0.42678	-1.22843581	0.021	0.03	down	yes
Manes.11G129	523.0593231	314.45142	1.663403	0.73413745	#####	0	up	no
Manes.11G129	2.529743918	2.1879965	1.156192	0.20938093	0.978	0.07	up	no
Manes.11G132	39.48198917	40.228798	0.981436	-0.02703397	0.963	0.01	down	yes
Manes.11G132	1590.698102	1959.8855	0.811628	-0.30110935	0.015	0.01	up	no
Manes.11G133	7.509916314	0.9381223	8.005264	3.00094897	0.002	0.03	up	yes
Manes.11G134	18.9793038	53.868696	0.352325	-1.50502007	9E-04	0	down	yes
Manes.11G137	1.746904425	0.3124686	5.590657	2.48301776	0.222	0.49	up	no
Manes.11G138	81.64101355	58.206863	1.402601	0.48810484	0.129	0	up	yes
Manes.11G138	653.1302073	18.873344	34.60596	5.11294867	#####	0	up	yes
Manes.11G139	63.85152702	16.476633	3.875278	1.9542997	#####	0	up	yes
Manes.11G139	10.75322242	9.2848397	1.158148	0.21182015	0.727	0	up	yes
Manes.11G139	110.0850436	40.550818	2.714743	1.44081557	2E-04	0	up	yes
Manes.11G141	240.6664346	284.13756	0.847007	-0.23955463	0.175	0.02	down	no
Manes.11G145	577.5529398	507.18927	1.138733	0.18742896	0.209	0.3	up	no
Manes.11G145	278.5799727	256.54277	1.085901	0.11889217	0.591	0.13	up	no
Manes.11G146	14.59331021	34.490373	0.423113	-1.24088658	0.003	0	down	yes

Manes.11G147	19.65763041	57.629989	0.341101	-1.55173032	#####	0.03	down	yes
Manes.11G149	33.87778938	106.49942	0.318103	-1.65243397	#####	0	down	yes
Manes.11G150	1.137855125	9.9735543	0.114087	-3.13179086	0.035	0	up	yes
Manes.11G152	5.413202025	8.3467174	0.648543	-0.6247267	0.383	1	up	no
Manes.11G153	0	1.5766236	#NAME?	#NAME?	0.145	0.61	down	no
Manes.11G155	158.4727422	152.74462	1.037501	0.05311319	0.824	0.67	down	no
Manes.11G156	4.274045458	6.9138236	0.618188	-0.69388155	0.446	0.84	down	no
Manes.11G156	719.8672475	434.41121	1.65711	0.72866956	#####	0	down	yes
Manes.11G157	133.4353739	375.73031	0.355136	-1.49355634	#####	0.01	up	no
Manes.11G159	1.794141802	2.1791925	0.823306	-0.28049973	1	0.98	up	no
Manes.11G165	1.367619383	1.5678197	0.872307	-0.19709284	1	0.61	down	no
Manes.12G002	99.08206529	80.022363	1.23818	0.3082207	0.242	0.37	up	no
Manes.12G005	105.0368954	171.28003	0.613247	-0.70546078	0.002	0.17	down	no
Manes.12G005	477.9138093	355.93454	1.342701	0.42513851	0.005	0.45	down	no
Manes.12G006	200.2477229	63.081415	3.174433	1.66649891	#####	0	up	yes
Manes.12G009	717.4172816	2.8088899	255.4095	7.99666861	#####	0.75	down	no
Manes.12G009	23.1077328	9.8874086	2.337087	1.22471136	0.036	0.92	down	no
Manes.12G009	934.2655126	276.56146	3.378148	1.7562325	#####	0.86	down	no
Manes.12G011	409.5001965	349.39106	1.17204	0.22902146	0.146	0.58	down	no
Manes.12G011	264.205811	306.82075	0.861108	-0.21573384	0.215	0.88	up	no
Manes.12G015	5.239411832	9.0339989	0.579966	-0.78595987	0.293	0.08	up	no
Manes.12G015	662.9866346	74.260824	8.927812	3.15830657	#####	0	up	no
Manes.12G015	7.388569266	24.53586	0.301133	-1.73152492	8E-04	0.82	up	no
Manes.12G017	89.3255405	48.09741	1.85718	0.89311354	0.005	0	down	yes
Manes.12G019	353.7510776	488.02718	0.724859	-0.46422696	0.003	0.11	up	no
Manes.12G021	2.528442476	1.8755279	1.348123	0.43095221	0.819	0.13	up	no
Manes.12G022	464.1290906	100.40399	4.622616	2.20870954	#####	0	up	yes
Manes.12G022	139.5093446	258.4325	0.539829	-0.88942575	2E-04	0	down	yes
Manes.12G024	32.05361777	56.24826	0.56986	-0.81132127	0.018	0	up	yes
Manes.12G026	95.68345499	215.97768	0.443025	-1.17454085	0.001	0.2	down	no
Manes.12G029	400.5234382	59.037648	6.784204	2.76217952	#####	0.12	up	no
Manes.12G035	152.5523779	108.7105	1.40329	0.48881332	0.034	0.48	down	no
Manes.12G039	129.9068841	59.026234	2.200833	1.13804969	#####	0	up	yes
Manes.12G042	61.8129317	44.393957	1.392373	0.47754539	0.134	0.31	up	no
Manes.12G043	509.257153	503.91405	1.010603	0.01521666	0.968	0.37	up	no
Manes.12G044	2.099317173	2.4876174	0.843907	-0.24484445	1	0.12	down	no
Manes.12G045	1.317779122	0	Inf	Inf	0.132	0.14	up	no
Manes.12G046	78.9143193	10.928568	7.22092	2.85218274	#####	0.08	down	no
Manes.12G047	671.0743656	509.19589	1.31791	0.39825188	0.012	0.01	up	no
Manes.12G049	7.742283457	4.0459165	1.913604	0.93629259	0.204	0.07	up	no
Manes.12G053	1408.194647	1604.4889	0.877659	-0.18826706	0.44	0.56	up	no
Manes.12G054	703.4225815	1439.3254	0.488717	-1.03292923	#####	0	down	yes
Manes.12G056	106.9891134	74.094235	1.44396	0.53003081	0.036	0.84	up	no
Manes.12G058	348.1378524	279.23087	1.246774	0.31820025	0.052	0.47	down	no
Manes.12G062	1.746904425	5.5961282	0.312163	-1.67962833	0.1	1	up	no
Manes.12G062	188.0142937	169.97874	1.106105	0.14548807	0.522	0.85	down	no
Manes.12G063	135.7432589	167.07154	0.812486	-0.29958543	0.167	0.28	up	no
Manes.12G065	9.540329584	7.4628522	1.278376	0.354312	0.597	0.12	up	no

Manes.12G070	160.9664808	196.42749	0.81947	-0.28723655	0.173	0.47	up	no
Manes.12G071	15.66579235	7.4269197	2.109326	1.07678186	0.046	0.75	up	no
Manes.12G071	2.173426843	0.3036646	7.157327	2.83942094	0.138	1	down	no
Manes.12G071	1.846584947	1.5630594	1.181391	0.24048705	0.934	0.89	up	no
Manes.12G072	189.1600421	222.32913	0.850811	-0.23308961	0.234	0	down	yes
Manes.12G073	5.89569851	18.897606	0.311981	-1.68046883	0.004	0.4	down	no
Manes.12G073	8.302793938	19.270525	0.430855	-1.21472709	0.044	0.25	down	no
Manes.12G073	222.6395443	1013.4061	0.219694	-2.1864306	#####	0.95	up	no
Manes.12G077	3.466936556	8.7534191	0.396067	-1.33618521	0.093	0.91	up	no
Manes.12G077	102.7644502	100.3645	1.023912	0.03409216	0.918	0.02	down	no
Manes.12G077	21.25110972	51.238782	0.414747	-1.269698	8E-04	0	down	yes
Manes.12G077	15.27646918	18.371662	0.831524	-0.26617104	0.607	0.03	up	yes
Manes.12G077	34.71633713	18.884758	1.838326	0.87839241	0.047	0.26	up	no
Manes.12G079	52.59469623	54.307061	0.968469	-0.04622246	0.901	1	up	no
Manes.12G082	30.260404	27.34214	1.106731	0.14630511	0.736	0	up	yes
Manes.12G082	3.264044591	10.946176	0.29819	-1.74569427	0.069	0.12	up	no
Manes.12G082	15.83307534	11.848366	1.336309	0.41825343	0.411	0	up	yes
Manes.12G087	414.3779779	126.99794	3.262872	1.70614229	#####	0	up	yes
Manes.12G087	59.91937518	3.0949466	19.36039	4.27503617	#####	0	up	yes
Manes.12G090	12.27166402	9.9585569	1.232273	0.30132229	0.598	0.27	up	no
Manes.12G092	106.8614404	186.97939	0.571515	-0.80713787	3E-04	0.42	down	no
Manes.12G092	14.28943628	10.824814	1.320063	0.40060673	0.491	0.06	up	no
Manes.12G094	22.8589049	4.6444417	4.921777	2.2991792	#####	0.05	up	yes
Manes.12G097	18.49067351	44.744252	0.413252	-1.27490458	9E-04	0.04	down	yes
Manes.12G098	37.04318908	20.565852	1.801199	0.84895744	0.048	0.7	up	no
Manes.12G098	309.1430338	178.97442	1.727303	0.78852112	#####	1	up	no
Manes.12G099	1.440427612	0.3124686	4.609832	2.20471424	0.35	1	down	no
Manes.12G100	9.312794805	33.611013	0.277076	-1.85164791	6E-04	0	down	yes
Manes.12G101	12.11470799	14.645125	0.827218	-0.27366083	0.636	0.51	down	no
Manes.12G102	152.4709406	276.53027	0.551372	-0.85890308	#####	0	down	yes
Manes.12G102	598.9098748	562.14569	1.0654	0.09139485	0.512	0.03	down	no
Manes.12G107	5.114160459	9.6230036	0.531452	-0.91198983	0.298	0.34	up	no
Manes.12G110	77.32827523	140.53403	0.550246	-0.86185156	4E-04	0.01	down	no
Manes.12G112	2.149157434	0	Inf	Inf	0.041	NA	--	no
Manes.12G113	59.13022066	86.094878	0.686803	-0.54203175	0.08	0.01	down	yes
Manes.12G115	81.4053813	141.77418	0.57419	-0.80039871	0.005	0.17	up	no
Manes.12G117	179.8057703	133.85868	1.343251	0.42572863	0.042	0.27	down	no
Manes.12G117	401.3580091	325.78672	1.231966	0.30096187	0.236	1	down	no
Manes.12G119	1.06114257	0	Inf	Inf	0.218	NA	--	no
Manes.12G120	11.18364916	9.0211512	1.239714	0.31000755	0.6	0.23	down	no
Manes.12G120	77.99552807	51.410357	1.517117	0.60133238	0.043	0.99	up	no
Manes.12G121	128.5072712	279.69401	0.459457	-1.12199934	#####	0.99	down	no
Manes.12G122	0.379285042	4.3441042	0.08731	-3.51770433	0.026	0.63	down	no
Manes.12G125	61.62918796	70.033321	0.879998	-0.18442771	0.544	0.09	up	no
Manes.12G128	3.896061859	0	Inf	Inf	0.003	1	up	no
Manes.12G131	1.870854356	1.5582991	1.200575	0.26372509	0.937	0.61	down	no
Manes.12G131	41.48868766	7.4533316	5.566462	2.47676067	#####	0.41	down	no
Manes.12G132	46.47602949	55.29035	0.840581	-0.25054088	0.631	0.06	down	no

Manes.12G138	3.088952957	8.7039224	0.354892	-1.4945478	0.085	0.73	up	no
Manes.12G139	60.27096749	160.08085	0.376503	-1.40926565	#####	0.87	up	no
Manes.12G139	752.7989539	225.59798	3.336905	1.73851044	#####	0	up	yes
Manes.12G142	12.59720448	26.124615	0.482197	-1.05230616	0.024	0.07	down	no
Manes.12G142	3.134888892	6.5654226	0.477485	-1.06647357	0.412	1	down	no
Manes.12G143	1502.562056	1160.9268	1.294278	0.37214755	0.004	0	up	yes
Manes.12G144	5.945538771	7.1679915	0.829457	-0.26976138	0.805	1	down	no
Manes.12G145	38.25579309	56.278511	0.679758	-0.55690592	0.114	0.45	up	no
Manes.12G145	34.9133611	53.812545	0.648796	-0.62416329	0.068	0.7	up	no
Manes.12G145	371.7023791	421.87541	0.881071	-0.18266907	0.709	0.03	up	no
Manes.12G146	12.74412238	4.3719493	2.914975	1.54348346	0.017	0	up	yes
Manes.12G151	25.41971426	3.7382082	6.799973	2.76552899	#####	0.6	up	no
Manes.12G152	78.9597006	107.97607	0.73127	-0.45152321	0.129	0.04	up	no
Manes.12G154	2105.202025	1.2689153	1659.056	10.6961472	#####	1	down	no
Manes.13G000	45.44751961	31.402337	1.447266	0.53332963	0.194	0.91	down	no
Manes.13G005	485.1915517	393.78546	1.232122	0.30114457	0.042	0.71	up	no
Manes.13G007	1.088014864	0.9469262	1.148996	0.20037433	1	0.94	down	no
Manes.13G008	21.721712	59.215877	0.366822	-1.44684624	0.001	0	down	yes
Manes.13G009	5.411900583	3.4481079	1.569528	0.65033043	0.476	0.76	up	no
Manes.13G009	16.01690366	8.7182033	1.837179	0.87749253	0.091	0.39	down	no
Manes.13G010	121.5509848	365.55549	0.33251	-1.58852882	#####	0.56	down	no
Manes.13G010	847.3901963	485.4256	1.745664	0.80377624	#####	0.83	down	no
Manes.13G010	154.4698304	117.51055	1.314519	0.39453476	0.069	0.08	down	no
Manes.13G012	1399.389276	1252.7346	1.117068	0.1597165	0.197	1	up	no
Manes.13G014	1.342048532	0.3172288	4.230538	2.08084102	0.354	0.03	up	yes
Manes.13G015	2.783777586	1.2641551	2.202086	1.13887051	0.471	0.75	down	no
Manes.13G017	370.7462775	475.58076	0.779565	-0.35925814	0.019	0	down	yes
Manes.13G017	3.262743149	0.3036646	10.74456	3.42553483	0.035	0.62	up	no
Manes.13G020	2.478602214	6.5152093	0.380433	-1.39428478	0.122	0.74	down	no
Manes.13G021	232.8903137	232.05684	1.003592	0.00517243	0.986	0.09	down	no
Manes.13G023	218.5029333	146.53643	1.491117	0.57639329	0.007	0	up	yes
Manes.13G023	2.126189466	4.0466331	0.525422	-0.92845188	0.427	0.3	down	no
Manes.13G025	155.6164222	14.639648	10.62979	3.41004158	#####	0.43	up	no
Manes.13G025	8.168805875	14.024231	0.582478	-0.77972457	0.183	0	up	yes
Manes.13G025	246.6593074	196.37489	1.256063	0.32890925	0.072	0	up	no
Manes.13G028	13.64058485	23.080598	0.590998	-0.75877511	0.115	0	down	yes
Manes.13G030	106.5136788	159.92138	0.666038	-0.58632412	0.01	0.01	up	no
Manes.13G033	9.941281151	1.2322663	8.067478	3.01211773	4E-04	0	up	yes
Manes.13G037	124.9557289	238.30117	0.524361	-0.931369	1E-04	0.05	down	no
Manes.13G038	105.0835781	91.75983	1.145202	0.1956026	0.434	0	up	yes
Manes.13G040	20.65117052	39.741428	0.519638	-0.94442015	0.025	0.41	up	no
Manes.13G041	17.09878472	5.922161	2.887254	1.52969817	0.006	0	up	yes
Manes.13G043	77.35329145	63.858169	1.21133	0.27659149	0.38	0.63	up	no
Manes.13G043	2.428761953	21.984465	0.110476	-3.17819144	#####	0	up	yes
Manes.13G043	18.32431856	218.83583	0.083735	-3.57801754	#####	0	down	yes
Manes.13G045	2.050778354	0.9374057	2.187717	1.12942616	0.736	0.26	up	no
Manes.13G046	15.2561041	24.645551	0.619021	-0.69194067	0.144	0.83	down	no
Manes.13G047	11.38523968	33.313541	0.34176	-1.54894407	0.005	0.36	up	no

Manes.13G048	37.74251999	69.319627	0.544471	-0.87707315	0.004	0.59	down	no
Manes.13G048	46.3743853	111.21599	0.416976	-1.2619641	#####	0.13	down	no
Manes.13G048	33.830552	51.454151	0.657489	-0.60496075	0.079	0.74	up	no
Manes.13G049	593.0561113	994.85468	0.596123	-0.7463172	#####	0.37	up	no
Manes.13G057	2.758206734	2.7960423	0.986468	-0.01965557	1	0.42	up	no
Manes.13G057	165.8611423	127.5579	1.300281	0.37882372	0.134	1	down	no
Manes.13G059	48.83235662	71.745361	0.680634	-0.55504815	0.138	0	up	yes
Manes.13G061	1878.036181	1486.4349	1.26345	0.3373686	0.009	0.82	up	no
Manes.13G061	3.944600678	4.3319731	0.910578	-0.13514504	0.981	0.04	up	yes
Manes.13G067	256.5226822	294.85199	0.870005	-0.20090454	0.252	0.34	down	no
Manes.13G067	78.54087924	82.032817	0.957432	-0.06275743	0.876	0	up	no
Manes.13G067	5.287950652	21.747905	0.243148	-2.04009583	3E-04	0.08	up	no
Manes.13G069	1.769872392	4.055437	0.43642	-1.19621205	0.309	0.69	up	no
Manes.13G070	3.114523808	15.862393	0.196346	-2.34852696	0.007	0.01	down	yes
Manes.13G070	343.160017	338.70951	1.01314	0.01883297	0.917	0.85	down	no
Manes.13G071	0.379285042	1.8531597	0.204669	-2.28863285	0.274	0.49	down	no
Manes.13G074	7.140669403	9.6915414	0.736794	-0.44066681	0.717	0.68	up	no
Manes.13G075	7.157503566	21.087036	0.339427	-1.55882792	0.003	0.73	down	no
Manes.13G078	260.5681682	215.30866	1.210208	0.27525447	0.163	0.01	down	no
Manes.13G080	13.1858887	37.352548	0.353012	-1.50221185	0.003	0.18	up	no
Manes.13G083	207.0555011	164.59964	1.257934	0.33105639	0.086	0.63	down	no
Manes.13G083	26.42934172	27.787384	0.951127	-0.07228954	0.928	0.92	down	no
Manes.13G084	0.329444781	2.791282	0.118026	-3.0828193	0.096	0.9	down	no
Manes.13G086	1.441729054	2.1703886	0.664272	-0.59015328	0.793	0.15	up	no
Manes.13G088	255.2978491	488.95401	0.522131	-0.93751739	#####	0	up	yes
Manes.13G089	36.73958096	22.700308	1.618462	0.69462327	0.06	0.49	down	no
Manes.13G090	15.60851684	17.077052	0.914005	-0.12972547	0.827	0.29	down	no
Manes.13G094	7.287587302	24.380686	0.298908	-1.74222554	0.001	0.97	down	no
Manes.13G098	1.517140166	0.3172288	4.782479	2.25775856	0.29	1	down	no
Manes.13G099	2.856585814	4.670137	0.611671	-0.709173	0.52	0.12	up	no
Manes.13G100	421.3712823	334.21477	1.26078	0.33431651	0.034	0.01	down	no
Manes.13G103	1.744301541	0.6344577	2.74928	1.45905364	0.622	0.71	down	no
Manes.13G104	159.3085795	130.2673	1.222936	0.29034895	0.178	0	down	yes
Manes.13G105	293.8030477	335.02269	0.876965	-0.18940946	0.264	0.07	down	no
Manes.13G107	5.568856613	3.1403996	1.773296	0.82643301	0.498	0	up	yes
Manes.13G109	5.999283358	2.1784759	2.75389	1.46147101	0.414	1	up	no
Manes.13G109	57.33049968	39.544127	1.449785	0.5358394	0.08	0.01	up	no
Manes.13G110	176.693476	267.08317	0.661567	-0.59604028	0.001	0.01	up	no
Manes.13G111	1.647223903	3.4026548	0.4841	-1.04662414	0.699	0.77	down	no
Manes.13G112	192.9100065	167.43995	1.152115	0.20428417	0.282	1	up	no
Manes.13G114	3.062080663	0.9421659	3.250044	1.7004592	0.171	0.06	up	no
Manes.13G115	77.34779686	239.9619	0.322334	-1.63337328	#####	0.07	up	no
Manes.13G118	2.708366473	1.2689153	2.134395	1.09382715	0.695	0.02	up	yes
Manes.13G118	7.485646905	5.9526166	1.257539	0.33060303	0.742	0.46	up	no
Manes.13G122	2.073746321	0.3172288	6.537068	2.7086436	0.141	0.68	up	no
Manes.13G125	136.8723773	188.40043	0.726497	-0.46097092	0.078	0.27	up	no
Manes.13G126	0	1.5447348	#NAME?	#NAME?	0.145	1	down	no
Manes.13G127	35.94019613	33.018681	1.088481	0.1223158	0.738	0.99	up	no

Manes.13G128	7456.66203	5476.8598	1.361485	0.445181	0.005	0.01	up		no
Manes.13G128	12.94999063	9.9537966	1.30101	0.37963224	0.536	0	up		yes
Manes.13G131	3.892157533	1.5718633	2.476142	1.30809431	0.248	0.94	down		no
Manes.13G132	0.329444781	9.2976873	0.035433	-4.81876334	#####	0.98	down		no
Manes.13G133	4.2753469	13.343604	0.320404	-1.64203497	0.012	0.03	down		yes
Manes.13G138	3.743010155	13.946889	0.268376	-1.89767252	0.072	0	down		yes
Manes.13G138	720.8982636	229.03923	3.147488	1.65420093	#####	0	up		yes
Manes.13G142	40.03599244	84.879073	0.471683	-1.08411133	2E-04	0	up		no
Manes.13G143	5.999283358	0.9286017	6.460556	2.69165835	0.012	0.37	up		no
Manes.13G145	4.854921023	0.6296974	7.709927	2.94671723	0.016	0.71	up		no
Manes.13G146	4.62906109	6.5328173	0.708586	-0.49698568	0.575	0.31	down		no
Manes.13G147	478.631961	441.15089	1.084962	0.11764455	0.461	0	down		yes
Manes.13G149	210.4071629	342.47534	0.614372	-0.70281629	3E-04	0.15	down		no
Manes.13G150	109.1384521	43.437049	2.512566	1.32916148	#####	0.44	up		no
Manes.13G151	435.0215549	416.9704	1.043291	0.06114192	0.772	0.11	up		no
Manes.13G152	37.86702455	36.737387	1.030749	0.04369304	0.893	0.87	down		no
Manes.13G152	30.16807415	27.346183	1.103191	0.14168311	0.71	0.64	up		no
Manes.13G152	58.93802905	37.990128	1.551404	0.63357425	0.095	0	up		yes
Manes.14G001	49.88986068	53.850832	0.926445	-0.110222	0.767	0	down		yes
Manes.14G001	14.57164368	16.147017	0.902436	-0.14810403	0.801	0.08	down		no
Manes.14G004	5.948141655	9.3431404	0.636632	-0.65146854	0.375	0	up		yes
Manes.14G004	2.782476144	2.8319747	0.982522	-0.02543906	1	1	up		no
Manes.14G006	2.808046995	1.2410703	2.262601	1.17798229	0.471	0.29	up		no
Manes.14G006	2.404492544	0.3172288	7.579678	2.92213663	0.09	0.08	up		no
Manes.14G008	45.0036084	84.780049	0.530828	-0.91368412	0.005	0.22	down		no
Manes.14G010	4.505111159	4.0778052	1.104788	0.14376983	0.899	0.28	up		no
Manes.14G011	1413.636906	243.65853	5.801713	2.53647897	#####	0.03	down		no
Manes.14G011	6.019648441	8.3636088	0.719743	-0.47444634	0.532	0.92	down		no
Manes.14G012	142.8280388	87.933071	1.624281	0.69980147	0.002	0	up		no
Manes.14G014	12.77620044	5.2605748	2.42867	1.28016649	0.038	0.54	up		no
Manes.14G015	12.27036258	7.1272988	1.721601	0.78375057	0.183	0.2	up		no
Manes.14G016	3.745239634	4.6539623	0.804742	-0.31340148	0.901	0.31	down		no
Manes.14G017	10.97685287	5.57376	1.96938	0.9777417	0.126	0	up		yes
Manes.14G022	6.377266957	1.8755279	3.400252	1.76564153	0.047	0.19	up		no
Manes.14G024	19.70877211	24.290496	0.811378	-0.30155407	0.519	0.35	down		no
Manes.14G025	1.491569315	27.7134	0.053821	-4.21568078	6E-04	0.09	down		no
Manes.14G026	3.164364069	13.611336	0.23248	-2.10482117	0.01	0.64	down		no
Manes.14G029	2.405793986	2.8096065	0.856274	-0.22385498	1	0.24	down		no
Manes.14G030	92.31119743	6.5599458	14.07195	3.81474987	#####	0.03	up		yes
Manes.14G030	4.857523907	6.2569978	0.776335	-0.36524949	0.75	0.8	down		no
Manes.14G037	3.488603082	1.5542554	2.244549	1.16642587	0.298	0.34	up		no
Manes.14G044	1.062444012	0.6296974	1.687229	0.75465622	0.864	1	down		no
Manes.14G044	190.1715256	139.82392	1.360079	0.44369008	0.03	0.55	up		no
Manes.14G047	5.263681242	1.5718633	3.348689	1.74359634	0.089	1	down		no
Manes.14G048	9.159743101	13.061591	0.701273	-0.51195158	0.4	0.63	up		no
Manes.14G049	21.52067611	43.834947	0.490948	-1.0263581	0.023	0	down		yes
Manes.14G049	3.188633479	12.434043	0.256444	-1.96328532	0.006	0	up		yes
Manes.14G050	116.1875384	116.52938	0.997066	-0.00423845	1	0.3	down		no

Manes.14G054	4.857523907	0.9421659	5.155699	2.366168	0.029	0	up	yes
Manes.14G054	334.0426594	416.50136	0.802021	-0.31828887	0.543	0.81	down	no
Manes.14G055	479.3235569	280.40415	1.709403	0.77349216	#####	0.05	up	no
Manes.14G056	36.92630099	65.493124	0.56382	-0.82669469	0.008	0.83	down	no
Manes.14G058	133.6398561	128.91296	1.036667	0.05195305	0.859	0	down	yes
Manes.14G061	154.2163514	262.49889	0.587493	-0.76735558	2E-04	0.76	up	no
Manes.14G062	14.75417057	17.131309	0.86124	-0.21551255	0.72	0.75	down	no
Manes.14G063	24.20477318	16.253637	1.489191	0.574529	0.328	0.01	up	yes
Manes.14G067	1045.709886	916.89565	1.14049	0.18965319	0.137	0.5	up	no
Manes.14G069	111.5944597	67.572862	1.651469	0.72374954	0.013	1	down	no
Manes.14G070	63.71288782	78.261513	0.814102	-0.29671777	0.366	0.55	down	no
Manes.14G070	442.3959895	151.5964	2.918249	1.54510285	#####	0	up	yes
Manes.14G071	3256.407319	2847.7132	1.143517	0.19347733	0.107	0.94	up	no
Manes.14G071	1.416158202	1.8619637	0.760572	-0.39484249	0.964	0.13	up	no
Manes.14G071	246.6964946	303.46022	0.812945	-0.29877018	0.088	0.22	up	no
Manes.14G074	1669.53332	1699.1936	0.982545	-0.02540534	0.821	0	up	no
Manes.14G074	6.045219293	9.902406	0.61048	-0.71198443	0.357	0.16	up	no
Manes.14G075	76.96107657	37.543911	2.049895	1.03555005	6E-04	0.13	down	no
Manes.14G076	3.466936556	6.9002594	0.502436	-0.99298916	0.278	0.78	down	no
Manes.14G078	8.603136946	10.252701	0.839109	-0.25306931	0.799	0.98	down	no
Manes.14G078	19.05378688	37.854229	0.503346	-0.99037674	0.012	0	down	yes
Manes.14G079	1.745602983	0	Inf	Inf	0.073	1	up	no
Manes.14G083	17.50847297	82.39811	0.212486	-2.23455798	#####	0	down	yes
Manes.14G083	73.45778422	88.302663	0.831886	-0.26554156	0.323	0.02	up	no
Manes.14G083	2.124888024	0.924558	2.298274	1.20055106	0.459	0.02	up	yes
Manes.14G084	84.66737764	80.056607	1.057594	0.08078574	0.837	0	down	yes
Manes.14G085	44.66048697	33.339953	1.339549	0.4217469	0.215	0.43	down	no
Manes.14G087	2.149157434	2.8081733	0.765322	-0.38586081	0.793	0.28	down	no
Manes.14G088	1.794141802	0.3172288	5.655671	2.4996981	0.217	1	up	no
Manes.14G089	2.075047763	4.6396814	0.447239	-1.1608812	0.287	0.98	up	no
Manes.14G093	3.917728384	0.6161331	6.358574	2.66870333	0.044	0.09	up	no
Manes.14G095	2523.867552	3304.238	0.763827	-0.38868139	0.217	0.72	up	no
Manes.14G095	2343.227154	2605.0997	0.899477	-0.15284176	0.484	0.58	up	no
Manes.14G096	31.76695141	3.4066985	9.32485	3.22108046	#####	0.96	down	no
Manes.14G096	13.81177216	9.9057331	1.394321	0.47956278	0.424	0.31	down	no
Manes.14G097	3.920331268	0	Inf	Inf	0.003	0.08	up	no
Manes.14G097	822.4981896	863.84073	0.952141	-0.07075284	0.577	0	up	yes
Manes.14G100	1058.919931	599.40398	1.766621	0.82099294	#####	0.01	up	no
Manes.14G100	10.04709548	13.693438	0.733716	-0.44670622	0.48	0	up	yes
Manes.14G100	250.0129823	361.25844	0.692061	-0.53102829	0.003	0	down	yes
Manes.14G100	4.53068201	12.728187	0.355957	-1.49022682	0.026	0.6	up	no
Manes.14G101	9.760055712	9.0204347	1.081994	0.11369243	0.934	0.42	down	no
Manes.14G101	160.5999443	155.8886	1.030223	0.04295598	0.839	0	up	yes
Manes.14G102	24.64300857	20.878321	1.180316	0.23917271	0.568	0	up	yes
Manes.14G102	1.366317941	0	Inf	Inf	0.131	NA	--	no
Manes.14G102	16.71949968	15.560162	1.074507	0.1036746	0.872	0.7	up	no
Manes.14G106	2.049476912	2.7953257	0.73318	-0.44776063	0.865	0.67	up	no
Manes.14G108	20.87480097	15.558729	1.341678	0.42403825	0.359	0.29	up	no

Manes.14G109	40.56023171	26.798332	1.513536	0.59792268	0.087	0.01	up		yes
Manes.14G112	15338.04427	12186.886	1.25857	0.33178505	0.089	0.13	up		no
Manes.14G112	139.7593663	111.79401	1.250151	0.32210213	0.149	0.88	down		no
Manes.14G112	327.5289793	347.59642	0.942268	-0.08579068	0.649	0	down		no
Manes.14G115	16.34021463	62.073342	0.26324	-1.9255469	#####	0.07	down		no
Manes.14G115	55.40088715	944.49874	0.058656	-4.09156789	#####	0	down		yes
Manes.14G115	35.40664252	50.201666	0.705288	-0.5037152	0.293	0	down		yes
Manes.14G116	36.09622412	17.706749	2.038557	1.02754857	0.009	0.94	up		no
Manes.14G117	3.492507408	5.3188755	0.656625	-0.60685809	0.569	0.58	down		no
Manes.14G120	20.93757107	50.447717	0.415035	-1.26869488	0.003	0	down		yes
Manes.14G121	0	2.1480203	#NAME?	#NAME?	0.068	0.01	up		yes
Manes.14G121	116.7037878	102.03106	1.143806	0.19384298	0.409	0.91	up		no
Manes.14G121	0	2.152064	#NAME?	#NAME?	0.064	0.06	up		no
Manes.14G125	1.137855125	0	Inf	Inf	0.207	NA	--		no
Manes.14G128	15420.8173	20977.856	0.7351	-0.443988	0.103	0	down		yes
Manes.14G128	19.47287405	25.124865	0.775044	-0.36765001	0.417	0.11	up		no
Manes.14G128	1.036873161	0.3124686	3.318328	1.7304565	0.56	0	up		yes
Manes.14G129	4.37372598	8.3724127	0.522397	-0.9367806	0.209	0.04	down		yes
Manes.14G129	3.21420433	1.5542554	2.068003	1.04823805	0.391	1	up		no
Manes.14G131	41.36603917	135.54861	0.305175	-1.71229156	#####	0	down		yes
Manes.14G135	3.465635114	2.1663449	1.599762	0.67785684	0.603	0.4	down		no
Manes.14G136	10.12120515	5.930965	1.706502	0.77104233	0.219	1	down		no
Manes.14G137	11.10563516	8.9668943	1.238515	0.30861162	0.605	0.62	up		no
Manes.14G138	1.062444012	0.924558	1.149137	0.20055106	1	0.59	up		no
Manes.14G139	0.683158971	1.2417869	0.550142	-0.86212432	0.87	0.76	down		no
Manes.14G139	25.09640328	30.43422	0.824611	-0.27821376	0.495	0.49	up		no
Manes.14G139	322.7585794	431.96814	0.747181	-0.42046947	0.01	0.22	up		no
Manes.14G140	11.05839779	31.397321	0.352208	-1.50549908	0.002	1	down		no
Manes.14G140	7.562359459	5.9485729	1.27129	0.34629283	0.667	0.37	up		no
Manes.14G140	620.8456106	495.86061	1.252057	0.32429991	0.067	0.11	up		no
Manes.14G142	704.8311463	464.70389	1.516732	0.60096596	#####	0	up		no
Manes.14G145	6.32352237	7.7929286	0.811444	-0.3014372	0.748	1	up		no
Manes.14G145	7.716712605	9.3138621	0.828519	-0.27139314	0.745	0.68	down		no
Manes.14G147	2.176029727	1.5454514	1.408022	0.49366994	0.808	0.75	up		no
Manes.14G148	2.685398505	0.6073292	4.421652	2.1445856	0.348	0.02	up		yes
Manes.14G151	4.783414236	2.8000859	1.70831	0.77256962	0.383	0.03	up		yes
Manes.14G152	13.83734301	13.679157	1.011564	0.01658761	1	0.56	up		no
Manes.14G154	1.366317941	2.4733365	0.552419	-0.85616532	0.64	0.77	up		no
Manes.14G156	1.845283505	4.6668099	0.395406	-1.33859422	0.2	0.21	down		no
Manes.14G157	3.211601446	2.7905654	1.150878	0.20273542	0.945	0.48	up		no
Manes.14G159	42.40105626	45.571966	0.93042	-0.10404641	0.771	0.15	up		no
Manes.14G159	19.68320126	21.38118	0.920585	-0.11937658	0.832	0.11	down		no
Manes.14G160	14.87681905	8.3874102	1.773708	0.82676877	0.133	0.64	up		no
Manes.14G162	34.91596398	55.075011	0.633971	-0.65751107	0.053	0.72	down		no
Manes.14G162	542.6202493	317.50989	1.708987	0.77314137	#####	0.24	down		no
Manes.14G162	80.44149759	77.881479	1.032871	0.04665966	0.887	0.75	down		no
Manes.14G163	32.49399806	8.0863561	4.018373	2.00661163	8E-04	0.09	down		no
Manes.14G164	15.10435383	9.9714046	1.514767	0.59909583	0.295	0.83	down		no

Manes.14G164	11.96648865	9.0306718	1.325094	0.40609466	0.485	0.07	down	no
Manes.14G165	46.05758153	82.623911	0.557436	-0.8431207	0.056	0.52	down	no
Manes.14G167	10.82510261	5.2741391	2.052487	1.03737318	0.117	0.04	down	yes
Manes.14G167	179.4829173	151.20989	1.186979	0.24729402	0.24	0	down	yes
Manes.14G168	1.896425208	1.2458305	1.522218	0.60617464	0.656	1	down	no
Manes.14G169	0.35371419	1.2458305	0.283918	-1.81645184	0.552	0.41	down	no
Manes.14G174	156.6032739	142.52737	1.098759	0.13587543	0.58	0.01	down	no
Manes.15G001	20.69710645	25.780258	0.802828	-0.31683759	0.484	0.62	up	no
Manes.15G001	31.69432441	37.336373	0.848886	-0.23635727	0.543	0.76	up	no
Manes.15G002	141.8366436	43.554111	3.256562	1.70334949	#####	0	up	no
Manes.15G002	33.87165558	91.586331	0.369833	-1.4350538	#####	0.67	down	no
Manes.15G005	2.758206734	3.3850469	0.814821	-0.29544523	0.905	1	up	no
Manes.15G006	284.522331	516.14072	0.55125	-0.85922256	#####	0.16	down	no
Manes.15G006	15.93926307	10.853376	1.4686	0.55444107	0.421	0	down	yes
Manes.15G010	32.15905869	122.46433	0.262599	-1.92906454	#####	0.3	up	no
Manes.15G010	32.57395271	12.219135	2.665815	1.41457665	0.013	0.62	up	no
Manes.15G012	15.07450525	29.033931	0.519203	-0.94562928	0.043	0.68	up	no
Manes.15G014	275.8479761	24.253131	11.37371	3.5076305	#####	0.77	down	no
Manes.15G017	4.930332135	4.9385857	0.998329	-0.00241311	1	0.75	up	no
Manes.15G017	2.554013327	2.4780968	1.030635	0.04353349	1	0.86	up	no
Manes.15G021	4.478238865	4.6539623	0.962242	-0.05552803	1	0.75	down	no
Manes.15G021	6.273682109	15.636787	0.401213	-1.31755978	0.04	0.78	down	no
Manes.15G023	7.592762673	1.2234623	6.205964	2.63365524	0.024	0	up	yes
Manes.15G024	1788.38675	6854.5946	0.260903	-1.93841258	#####	0	up	yes
Manes.15G028	4.224205197	1.5542554	2.717832	1.44245632	0.171	0.75	up	no
Manes.15G028	2.427460511	3.4704761	0.69946	-0.51568575	0.825	0.95	up	no
Manes.15G029	15.28650731	7.4214428	2.059776	1.04248722	0.055	0	up	yes
Manes.15G029	3.161761185	9.0171076	0.35064	-1.51193633	0.054	0.04	down	yes
Manes.15G033	188.5253373	214.39796	0.879324	-0.18553273	0.385	0	down	yes
Manes.15G035	34.93577443	65.032391	0.537206	-0.89645334	0.005	0.81	down	no
Manes.15G036	1.038174603	0.9109938	1.139607	0.18853601	1	0.86	down	no
Manes.15G037	10.97685287	1.8707676	5.867566	2.5527622	0.004	0	up	yes
Manes.15G038	682.9588163	780.09895	0.875477	-0.19185855	0.237	0	up	yes
Manes.15G039	11.15937975	6.5423378	1.705717	0.77037868	0.2	0.24	up	no
Manes.15G039	3512.565563	2086.845	1.683194	0.75120167	0.111	0.83	down	no
Manes.15G044	819.0085399	243.84235	3.358763	1.7479298	#####	0	up	yes
Manes.15G046	25.44751459	27.066064	0.9402	-0.08896036	0.864	1	up	no
Manes.15G050	2.907727517	2.4964214	1.164758	0.22003063	0.958	0.76	up	no
Manes.15G051	90.00134881	94.888304	0.948498	-0.07628364	0.809	0.39	up	no
Manes.15G052	47.59415875	116.49009	0.408568	-1.29135085	#####	0.01	down	no
Manes.15G052	17.91240083	123.40656	0.14515	-2.78438845	#####	0.32	down	no
Manes.15G054	1101.078472	1158.0374	0.950814	-0.07276453	0.696	0.46	down	no
Manes.15G056	3.516776817	10.580167	0.332393	-1.5890367	0.03	0.83	down	no
Manes.15G057	5.184739209	8.1244383	0.638166	-0.64799665	0.603	0.04	down	yes
Manes.15G058	76.43962141	64.145659	1.191657	0.252969	0.365	0.01	up	no
Manes.15G059	3.264044591	1.5454514	2.112033	1.07863244	0.391	0.3	down	no
Manes.15G060	245.2029846	244.60178	1.002458	0.00354164	0.995	0	down	no
Manes.15G062	34.06711229	31.140542	1.093979	0.12958559	0.748	0.23	down	no

Manes.15G065	25.04173066	9.2665152	2.702389	1.43423548	0.011	0.43	down	no
Manes.15G065	1.137855125	0.6249371	1.820751	0.86453396	0.884	0.03	up	yes
Manes.15G070	1.76857095	0.3036646	5.824093	2.54203351	0.202	0.02	up	yes
Manes.15G072	109.4435429	67.720861	1.616098	0.69251461	0.05	0	up	yes
Manes.15G072	1.038174603	0	Inf	Inf	0.24	0	up	yes
Manes.15G075	2.198997695	3.4433476	0.638622	-0.64696572	0.621	0	up	yes
Manes.15G078	25.34690603	105.46635	0.240332	-2.05690122	#####	0.02	down	no
Manes.15G079	80.6729367	130.44295	0.618454	-0.69326231	0.004	0.01	down	no
Manes.15G080	24.50511618	51.883221	0.472313	-1.08218506	0.003	0.01	down	yes
Manes.15G080	53.54872303	76.646837	0.698642	-0.51737409	0.069	0	down	yes
Manes.15G082	4.955902987	7.8336214	0.632645	-0.66053145	0.416	1	down	no
Manes.15G082	1431.944196	11.428816	125.2924	6.96915546	#####	0.86	down	no
Manes.15G082	4.984076722	0.9197977	5.418666	2.43793773	0.028	0.81	up	no
Manes.15G083	270.721067	298.74583	0.906192	-0.14211144	0.426	0	down	yes
Manes.15G084	76.62391978	0.9286017	82.51538	6.3665911	#####	0.56	down	no
Manes.15G086	0	153.87194	#NAME?	#NAME?	#####	0.51	down	no
Manes.15G091	504.569939	1523.4199	0.331209	-1.59418753	#####	0	down	yes
Manes.15G091	3.772111927	7.7624731	0.485942	-1.04114387	0.214	0	up	yes
Manes.15G091	106.1596758	95.591832	1.110552	0.15127662	0.817	0.56	up	no
Manes.15G094	6.018346999	11.137283	0.540378	-0.8879581	0.173	0.2	down	no
Manes.15G095	280.4177134	206.27374	1.359445	0.44301728	0.014	0.34	down	no
Manes.15G096	328.249456	445.35439	0.737052	-0.44016118	0.082	0.74	down	no
Manes.15G097	11.68558269	11.771024	0.992741	-0.01051014	1	1	down	no
Manes.15G097	306.1662102	177.6308	1.72361	0.78543331	#####	0	up	no
Manes.15G098	1224.588887	141.41912	8.659288	3.11424838	#####	0	up	yes
Manes.15G104	196.9957756	214.72439	0.917435	-0.12432138	0.618	0.03	up	no
Manes.15G104	181.9634832	188.13907	0.967175	-0.04815054	0.802	0.03	up	no
Manes.15G106	4.957204429	4.0371125	1.227908	0.29620296	0.783	0.5	up	no
Manes.15G106	42.36990623	73.687277	0.574996	-0.79837561	0.008	0.85	down	no
Manes.15G106	1.088014864	0	Inf	Inf	0.238	0.6	up	no
Manes.15G109	20.15696105	14.335266	1.40611	0.49170943	0.293	1	down	no
Manes.15G109	260.7971856	183.46368	1.421519	0.50743378	0.006	0	up	yes
Manes.15G111	9.107299956	12.092296	0.753149	-0.40899294	0.527	0	up	yes
Manes.15G113	189.7932532	242.45494	0.782798	-0.35328792	0.066	0.1	down	no
Manes.15G118	1.137855125	0	Inf	Inf	0.207	NA	--	no
Manes.15G119	9.34096854	10.023051	0.931949	-0.10167768	0.988	0.01	up	yes
Manes.15G121	16.90072512	13.343604	1.266579	0.34093679	0.5	0.18	down	no
Manes.15G124	16.44602896	22.336193	0.736295	-0.44164406	0.355	0.37	down	no
Manes.15G124	6.835494032	3.3667224	2.030311	1.02170085	0.435	0.14	up	no
Manes.15G125	106.2190962	190.59816	0.557293	-0.84349102	3E-04	0.01	down	no
Manes.15G126	12.44712906	0.9381223	13.26813	3.72989326	#####	0.57	up	no
Manes.15G127	78.5697152	67.78774	1.159055	0.21294895	0.446	0.85	down	no
Manes.15G128	78.82218162	87.28336	0.903061	-0.14710495	0.73	0	down	yes
Manes.15G132	21.42657476	11.151564	1.921396	0.94215522	0.052	0.02	down	yes
Manes.15G133	578.4418209	27.667456	20.90694	4.38590997	#####	0.03	up	no
Manes.15G138	2.95886922	16.497568	0.179352	-2.4791355	0.009	0.06	down	no
Manes.15G140	57.44868921	55.344893	1.038012	0.05382378	0.887	0.61	down	no
Manes.15G140	7.691141753	8.7277238	0.881231	-0.18240767	0.87	0.18	up	no

Manes.15G144	665.086614	907.45767	0.732912	-0.44828811	0.004	0.13	up	no
Manes.15G145	775.0294283	383.83387	2.019179	1.01376907	2E-04	0	up	yes
Manes.15G148	143.8125534	371.26398	0.387359	-1.36825573	8E-04	0.04	up	no
Manes.15G150	58.21990031	172.41779	0.337668	-1.56632436	#####	0.71	up	no
Manes.15G150	9.63610578	10.927851	0.881793	-0.18148763	0.815	0.56	down	no
Manes.15G151	222.6799362	350.25967	0.635757	-0.6534533	0.003	0.55	up	no
Manes.15G153	0.683158971	4.6389648	0.147265	-2.76350967	0.042	0.56	up	no
Manes.15G154	23.87830468	32.620783	0.731997	-0.450091	0.245	0.87	up	no
Manes.15G155	10.57199698	6.837915	1.546085	0.62861951	0.315	0.1	up	no
Manes.15G156	15.17028145	15.91404	0.953264	-0.06905227	1	0.05	down	yes
Manes.15G156	115.4118454	120.38465	0.958692	-0.0608602	0.879	0.68	up	no
Manes.15G162	63.34531576	77.430757	0.81809	-0.28966881	0.381	0.33	up	no
Manes.15G162	4.654631942	17.411889	0.267325	-1.90333369	0.002	0.11	down	no
Manes.15G162	25.75008708	22.379036	1.150634	0.20242942	0.635	0.26	up	no
Manes.15G162	24.83456096	11.820521	2.10097	1.07105567	0.019	0.56	down	no
Manes.15G162	91.79986498	203.99458	0.450011	-1.15196686	#####	0.08	up	no
Manes.15G162	88.4970539	54.493377	1.623997	0.69954853	0.02	0.19	down	no
Manes.15G163	13.69135315	12.681301	1.079649	0.11056227	0.836	0.24	down	no
Manes.15G166	27.18605573	10.266265	2.648096	1.40495542	0.005	0.11	down	no
Manes.15G169	3.110619482	11.127046	0.279555	-1.83879677	0.089	0.15	down	no
Manes.15G169	3221.435487	1844.9227	1.746109	0.8041433	#####	0.51	down	no
Manes.15G171	125.3172517	163.80524	0.765038	-0.3863965	0.076	0.01	up	no
Manes.15G171	109.6187191	185.22221	0.591823	-0.75676293	0.003	0	down	yes
Manes.15G172	207.6374123	176.43515	1.176848	0.23492838	0.231	0.52	up	no
Manes.15G173	346.0380772	435.5126	0.794554	-0.33178367	0.038	0.25	down	no
Manes.15G173	113.1627416	82.872407	1.365506	0.44943531	0.067	0	up	no
Manes.15G173	203.3018982	159.47115	1.274851	0.35032824	0.077	0.62	down	no
Manes.15G174	245.5293455	183.71502	1.336469	0.41842591	0.024	0	down	yes
Manes.15G176	26.78826168	78.637533	0.340655	-1.5536171	#####	0.01	down	yes
Manes.15G176	2.529743918	1.5766236	1.604533	0.68215306	0.668	0	up	yes
Manes.15G177	63.41207477	65.937191	0.961704	-0.05633485	0.899	0.33	up	no
Manes.15G178	3.235870856	0.9381223	3.449306	1.78630617	0.166	0.99	up	no
Manes.15G179	35.13959444	47.163331	0.745062	-0.42456808	0.271	1	down	no
Manes.15G180	25.06209574	16.162014	1.550679	0.63290005	0.157	0.05	down	no
Manes.15G184	5.89569851	1.2417869	4.747754	2.24724519	0.021	0.61	up	no
Manes.15G186	8.374300724	10.784122	0.77654	-0.36486803	0.63	0.82	down	no
Manes.15G186	389.4186235	280.47165	1.388442	0.47346692	0.008	0.98	up	no
Manes.15G187	379.7091888	639.97779	0.593316	-0.75312692	1E-04	0.09	down	no
Manes.15G188	19.27964681	24.906629	0.774077	-0.36945118	0.425	0.42	down	no
Manes.15G190	3.871792449	5.282943	0.732886	-0.44834024	0.682	0.71	down	no
Manes.15G191	0	1.8395955	#NAME?	#NAME?	0.1	0.5	down	no
Manes.15G191	1.012603751	1.8986127	0.533339	-0.90687593	0.684	0.66	up	no
Manes.15G192	15.15121781	11.219385	1.35045	0.43344018	0.406	0.25	down	no
Manes.15G192	148.5382341	116.87349	1.270932	0.34588666	0.111	0.08	up	no
Manes.16G000	86.55041502	75.479526	1.146674	0.19745538	0.478	0.52	down	no
Manes.16G005	48.77534692	82.078731	0.594251	-0.75085629	0.009	0	up	no
Manes.16G006	138.5828293	172.78576	0.80205	-0.31823582	0.144	0.1	up	no
Manes.16G006	13.05580496	32.309748	0.404083	-1.30727808	0.003	1	down	no

Manes.16G008	7.669475228	7.19512	1.065927	0.09210911	0.909	0	up		yes
Manes.16G009	2.124888024	1.5406912	1.379178	0.46380912	0.831	0.61	down		no
Manes.16G010	32.97360284	67.089761	0.491485	-1.02478107	0.009	0.13	down		no
Manes.16G012	6.196041518	8.3521943	0.741846	-0.43080846	0.625	0.58	down		no
Manes.16G019	17.04634157	4.3495811	3.919077	1.97051379	8E-04	0	up		yes
Manes.16G022	102.2997775	153.53944	0.666277	-0.58580627	0.059	0	up		no
Manes.16G022	2.856585814	1.2146584	2.351761	1.23374128	0.707	0.2	up		no
Manes.16G023	46.72196569	147.63041	0.316479	-1.65981706	#####	0.2	down		no
Manes.16G024	2.378921692	6.2075011	0.383233	-1.38370482	0.163	0.08	down		no
Manes.16G028	6.729679705	0.9197977	7.316478	2.87114929	0.005	1	down		no
Manes.16G029	21.83366013	33.979171	0.64256	-0.63809667	0.113	0	down		yes
Manes.16G029	2.126189466	0.3172288	6.702384	2.74467434	0.139	0.48	up		no
Manes.16G031	51.54712271	29.604151	1.741213	0.80009243	0.091	0.48	up		no
Manes.16G031	52.40696358	119.27923	0.439364	-1.18651238	#####	0.18	up		no
Manes.16G033	61.8854511	38.825674	1.593931	0.67258932	0.027	0.82	up		no
Manes.16G034	3.21420433	1.2370266	2.598331	1.37758516	0.271	0.76	down		no
Manes.16G035	5.796017988	3.1037505	1.867424	0.90104947	0.276	0.94	up		no
Manes.16G035	1.038174603	2.4971379	0.415746	-1.26622642	0.463	0	up		yes
Manes.16G036	12.42118481	12.99377	0.955934	-0.06501724	0.934	0.62	up		no
Manes.16G038	201.4816925	67.315337	2.993102	1.58164161	#####	0.37	up		no
Manes.16G042	59.80687242	2.7553495	21.70573	4.44000403	#####	1	up		no
Manes.16G043	7.082092453	26.121288	0.271123	-1.88297844	2E-04	0.01	down		yes
Manes.16G043	4.528079126	17.756962	0.255003	-1.97141374	0.001	0.02	down		yes
Manes.16G043	121.2069583	129.60526	0.935201	-0.0966517	0.652	0.11	up		no
Manes.16G043	1.366317941	0	Inf	Inf	0.131	0.23	up		no
Manes.16G046	2.856585814	0.9109938	3.135681	1.64877878	0.544	0.86	down		no
Manes.16G046	289.9849999	270.42078	1.072347	0.10077228	0.546	0	up		no
Manes.16G047	35.81754764	49.195693	0.728063	-0.45786544	0.187	0	down		yes
Manes.16G047	53.79624949	36.619353	1.469066	0.55489931	0.09	0.01	down		yes
Manes.16G051	75.93192748	164.24386	0.462312	-1.11306093	#####	0.03	down		no
Manes.16G053	3.691868452	0.6073292	6.078859	2.60380056	0.258	0.38	up		no
Manes.16G056	4.958505871	5.2924636	0.936899	-0.09403398	1	0.57	down		no
Manes.16G056	5.792487067	8.9940228	0.644037	-0.63478362	0.39	0.18	down		no
Manes.16G056	154.4532851	284.51338	0.542868	-0.88132594	#####	0.71	up		no
Manes.16G057	407.3438696	403.82395	1.008716	0.01252071	0.918	0	down		no
Manes.16G061	20.01097119	13.036612	1.534982	0.61822219	0.218	1	down		no
Manes.16G062	1.795443244	1.2370266	1.451418	0.53746356	0.838	0.61	up		no
Manes.16G064	4.580522271	1.5582991	2.939437	1.55553994	0.127	1	down		no
Manes.16G064	1.390587351	4.6756139	0.297413	-1.74946142	0.174	0.42	down		no
Manes.16G065	2.478602214	2.1615846	1.14666	0.19743746	0.982	1	down		no
Manes.16G066	3.237172298	0.3036646	10.66035	3.41418356	0.035	0.17	up		no
Manes.16G066	8.85456773	14.987332	0.590803	-0.75924981	0.184	0.8	down		no
Manes.16G069	129.1058244	90.500436	1.426577	0.51255745	0.03	0.01	down		no
Manes.16G070	2.123586582	2.1703886	0.978436	-0.03145041	1	0.17	down		no
Manes.16G075	37.4907158	77.7313	0.482312	-1.05196227	6E-04	0.11	up		no
Manes.16G077	287.9516949	281.83391	1.021707	0.03098161	0.841	0.19	up		no
Manes.16G077	1690.966395	849.64439	1.990205	0.99291694	#####	0.73	up		no
Manes.16G078	2.781174702	4.3638619	0.63732	-0.64991108	0.722	0.07	down		no

Manes.16G078	23.19254283	49.281152	0.470617	-1.08737497	0.003	0	down	yes
Manes.16G079	1.038174603	2.4780968	0.41894	-1.25518346	0.453	0.19	up	no
Manes.16G081	0	1.5454514	#NAME?	#NAME?	0.145	1	down	no
Manes.16G082	19.52661863	28.347827	0.688822	-0.53779602	0.301	0.18	down	no
Manes.16G086	2.176029727	0.6249371	3.481998	1.79991535	0.519	1	up	no
Manes.16G092	134.8853817	158.7703	0.849563	-0.2352071	0.277	0.7	down	no
Manes.16G092	136.3992677	267.41737	0.510061	-0.97125727	0.037	0	up	yes
Manes.16G095	163.1052267	240.795	0.677361	-0.56200245	0.007	0	down	yes
Manes.16G101	467.2222258	372.99978	1.252607	0.32493413	0.043	0.29	up	no
Manes.16G102	28.20702277	69.646377	0.405003	-1.3039939	#####	0.09	down	no
Manes.16G102	5.490842617	4.3679056	1.257088	0.33008589	0.682	0.99	up	no
Manes.16G104	90.4144834	62.180658	1.454061	0.54008801	0.041	0.05	down	no
Manes.16G109	1.012603751	0.6208934	1.630882	0.70565217	0.868	0.75	down	no
Manes.16G109	21.53238909	18.863823	1.141465	0.19088628	0.753	0	up	yes
Manes.16G110	1.41485676	0.6249371	2.263999	1.17887308	0.699	0.71	down	no
Manes.16G110	8.117664172	3.4209794	2.372906	1.24665525	0.095	0.68	up	no
Manes.16G113	1.062444012	0.3036646	3.498742	1.80683623	0.559	0.34	up	no
Manes.16G116	5.815455034	1.5630594	3.720559	1.8955195	0.05	0.94	down	no
Manes.16G116	59.46516003	7.4987846	7.929973	2.98731597	#####	0	up	yes
Manes.16G117	28.71118578	14.390957	1.995085	0.99645042	0.062	0	up	yes
Manes.16G117	2.100618615	6.2034574	0.338621	-1.56225825	0.101	0.95	down	no
Manes.16G118	1.038174603	1.8755279	0.553537	-0.85324764	0.737	0.23	down	no
Manes.16G120	0.658889561	2.5099856	0.262507	-1.92957051	0.414	0.69	down	no
Manes.16G122	4.479540307	6.5111657	0.687978	-0.53956515	0.571	1	up	no
Manes.16G122	5.160096394	4.9704745	1.03815	0.05401444	0.929	0.2	down	no
Manes.16G127	128.726166	69.489799	1.852447	0.88943222	8E-04	0	up	yes
Manes.16G128	2.478602214	4.6437251	0.533753	-0.90575581	0.391	0.75	down	no
Manes.16G131	25.06822955	37.626013	0.666247	-0.58587031	0.124	0	down	yes
Manes.16G133	143.5813185	110.29404	1.301805	0.38051324	0.09	0.58	down	no
Manes.16G137	48.64173227	170.06226	0.286023	-1.80579651	#####	0	down	yes
Manes.16G138	193.286134	139.6423	1.384152	0.4690021	0.019	0.02	down	no
Manes.16G138	39.8757713	61.770169	0.645551	-0.63139785	0.068	0.33	down	no
Manes.17G000	69.2964337	46.663339	1.485029	0.57049157	0.054	0.48	up	no
Manes.17G001	8.522520065	6.5335339	1.304427	0.38341656	0.595	0.31	down	no
Manes.17G002	8.14713935	21.196983	0.384354	-1.37949344	0.008	0	down	yes
Manes.17G003	1.391888793	0.6296974	2.210409	1.14431336	0.604	1	down	no
Manes.17G004	492.2844907	464.74552	1.059256	0.08305132	0.558	0.24	down	no
Manes.17G007	722.5354954	472.9688	1.52766	0.61132344	0.002	0.47	down	no
Manes.17G007	4.325187161	12.118708	0.356902	-1.48640147	0.027	0.09	down	no
Manes.17G012	1.416158202	0.3036646	4.663561	2.22143185	0.351	0.89	up	no
Manes.17G017	16.82791689	23.239816	0.724099	-0.46574207	0.316	0.25	down	no
Manes.17G020	16.74376909	10.616099	1.577205	0.65737054	0.203	0.36	up	no
Manes.17G024	946.4135034	911.37544	1.038445	0.05442517	0.752	0.34	up	no
Manes.17G024	1.896425208	0	Inf	Inf	0.064	1	up	no
Manes.17G024	1.467299905	0	Inf	Inf	0.128	NA	--	no
Manes.17G026	12.19755435	15.843352	0.769885	-0.37728571	0.516	0	up	yes
Manes.17G028	1.745602983	1.5454514	1.12951	0.17569715	1	0.61	down	no
Manes.17G030	4.0442812	0	Inf	Inf	0.003	1	up	no

Manes.17G033	2.455634247	0.9109938	2.695555	1.4305826	0.627	0.03	up	yes
Manes.17G035	165.502765	244.62963	0.676544	-0.56374381	0.003	0.01	down	no
Manes.17G038	4.577919387	2.827931	1.618823	0.69494513	0.707	0.12	down	no
Manes.17G040	31.77624273	25.502288	1.246015	0.31732185	0.413	0.21	down	no
Manes.17G040	28.64786105	32.982288	0.868583	-0.20326404	0.721	0	up	yes
Manes.17G041	55.12927251	212.81803	0.259044	-1.94872989	#####	0.01	down	yes
Manes.17G044	5.942935887	3.1037505	1.91476	0.93716321	0.267	0.66	down	no
Manes.17G044	3.136190334	3.076622	1.019362	0.0276659	1	0.51	down	no
Manes.17G044	1.720032131	0.6161331	2.791657	1.48112146	0.419	1	down	no
Manes.17G045	1.137855125	0	Inf	Inf	0.207	NA	--	no
Manes.17G046	134.9669266	93.027803	1.450824	0.53687205	0.037	0	up	yes
Manes.17G046	8.85456773	12.790532	0.692275	-0.53058245	0.387	0	up	yes
Manes.17G048	124.0758118	265.76782	0.466858	-1.09894452	#####	0.37	up	no
Manes.17G053	1.137855125	32.431826	0.035085	-4.83302147	1E-04	0.33	down	no
Manes.17G053	5.313521503	17.462102	0.304289	-1.716487	0.004	0.55	up	no
Manes.17G053	1.012603751	2.1927568	0.461795	-1.11467605	0.579	0.98	down	no
Manes.17G054	340.6008825	254.55507	1.338024	0.4201044	0.014	0	up	no
Manes.17G057	61.50784091	79.855519	0.770239	-0.37662178	0.186	0.9	down	no
Manes.17G058	0.732999232	5.2957907	0.138412	-2.85296251	0.019	0	up	yes
Manes.17G058	1.671493312	8.1142012	0.205996	-2.27931149	0.056	0.21	down	no
Manes.17G059	78.65646589	13.67368	5.752399	2.52416377	#####	1	down	no
Manes.17G059	1.441729054	0.9421659	1.530228	0.61374697	0.847	0	up	yes
Manes.17G060	81.49584413	49.35706	1.651149	0.72347002	0.009	0	up	yes
Manes.17G070	13.15418404	8.6551423	1.519811	0.60389231	0.374	0	up	yes
Manes.17G070	229.0338497	185.18814	1.236763	0.30656911	0.104	0.4	down	no
Manes.17G071	44.85464225	16.462352	2.72468	1.44608685	1E-04	0.35	up	no
Manes.17G071	63.2958489	93.821705	0.67464	-0.56781083	0.034	0.04	up	no
Manes.17G072	173.8744628	149.20371	1.165349	0.22076267	0.304	0.7	up	no
Manes.17G073	435.151084	284.29653	1.530624	0.61411992	3E-04	1	up	no
Manes.17G073	1.038174603	0	Inf	Inf	0.24	0.58	up	no
Manes.17G073	105.2777334	12.181053	8.642745	3.11148962	#####	0.36	down	no
Manes.17G075	2.176029727	12.132273	0.179359	-2.47907964	0.01	0.5	down	no
Manes.17G077	11.86810957	10.603252	1.11929	0.16258338	0.775	0.09	down	no
Manes.17G078	26.71675489	179.06747	0.149199	-2.74468661	#####	0	down	yes
Manes.17G086	18.89162508	40.493203	0.466538	-1.09993297	0.005	1	down	no
Manes.17G087	186.1082653	131.76157	1.412462	0.4982125	0.021	0	down	yes
Manes.17G088	24.58573306	16.160581	1.52134	0.6053423	0.174	0.32	down	no
Manes.17G088	117.0601049	117.72139	0.994383	-0.00812698	1	0	down	yes
Manes.17G094	7.485646905	4.1049337	1.823573	0.86676806	0.494	0.42	down	no
Manes.17G096	0.758570083	1.5542554	0.48806	-1.03486922	0.817	0	up	yes
Manes.17G096	7.208645269	35.233806	0.204595	-2.28916025	#####	0	up	yes
Manes.17G097	20.86829376	33.522256	0.622521	-0.68380661	0.086	0.04	down	yes
Manes.17G099	69.37928006	38.661695	1.794522	0.84359997	0.005	0.37	up	no
Manes.17G100	1.391888793	1.2553511	1.108765	0.14895304	1	0.61	up	no
Manes.17G101	118.028363	271.73876	0.434345	-1.20308676	#####	0	up	no
Manes.17G101	537.0996426	220.00117	2.441349	1.28767857	#####	0	up	yes
Manes.17G101	134.1812184	177.74739	0.754898	-0.40564565	0.091	0.01	up	no
Manes.17G102	14.70693319	45.233802	0.325131	-1.62090483	0.003	1	down	no

Manes.17G102	9.587566961	9.6779771	0.990658	-0.01354078	1	0.22	up	no
Manes.17G102	96.19860718	143.09686	0.672262	-0.57290409	0.017	0.28	down	no
Manes.17G106	6.453606107	6.5911179	0.979137	-0.03041766	1	0.57	down	no
Manes.17G106	3.541046227	0	Inf	Inf	0.005	NA	--	no
Manes.17G110	5.691505103	7.8376651	0.726174	-0.4616137	0.586	0.15	down	no
Manes.17G112	16.29650818	74.786993	0.217906	-2.1982245	#####	0.78	down	no
Manes.17G113	36.9539201	39.53128	0.934802	-0.09726724	0.888	0.05	down	no
Manes.17G118	1.465998463	0	Inf	Inf	0.129	0.33	up	no
Manes.17G118	207.4766366	375.30767	0.552817	-0.8551249	#####	0	down	yes
Manes.17G119	136.3601168	104.94947	1.299293	0.3777269	0.097	0.03	up	no
Manes.17G119	1132.606029	753.84421	1.50244	0.5873078	0.089	0.03	up	no
Manes.17G120	25.09510184	41.577696	0.603571	-0.72840402	0.048	0.98	up	no
Manes.17G120	28.95043353	49.182845	0.588629	-0.76457023	0.074	0.27	down	no
Manes.17G121	18.03337447	5.2646185	3.425391	1.77626848	0.002	0.05	up	yes
Manes.17G121	75.56713952	210.94033	0.358239	-1.48100403	#####	0	down	yes
Manes.17G122	42.45145115	100.10179	0.424083	-1.23758196	#####	0	down	yes
Manes.17G124	3.6662976	2.5045087	1.463879	0.54979626	0.942	0.93	up	no
Manes.18G000	38.68101407	25.160797	1.537352	0.62044798	0.095	0.38	down	no
Manes.18G001	605.8264558	448.03919	1.352173	0.43527965	0.002	0.09	up	no
Manes.18G002	169.1060036	27.301191	6.194089	2.63089208	#####	0.24	down	no
Manes.18G002	1062.010718	956.5883	1.110207	0.15082827	0.279	0.02	up	no
Manes.18G005	16.36578549	12.441414	1.315428	0.39553238	0.452	0.02	up	yes
Manes.18G007	254.3491357	199.81158	1.272945	0.34816996	0.056	1	down	no
Manes.18G008	59.52596646	78.219387	0.761013	-0.39400709	0.173	0	down	yes
Manes.18G009	448.0389098	1.5454514	289.9081	8.17945189	#####	1	up	no
Manes.18G012	37.01602797	38.650741	0.957706	-0.062346	0.928	0	up	yes
Manes.18G015	203.5180088	203.47173	1.000227	0.00032807	0.995	0.18	up	no
Manes.18G016	131.4008641	103.74909	1.266525	0.34087609	0.129	0.24	down	no
Manes.18G017	647.9957335	658.31625	0.984323	-0.02279649	0.855	0	down	yes
Manes.18G017	480.0625317	485.14552	0.989523	-0.01519522	0.903	0	down	yes
Manes.18G017	1.366317941	0.6161331	2.217569	1.14897918	0.607	0.3	up	no
Manes.18G020	93.63696643	110.4111	0.848076	-0.23773512	0.457	0.05	down	no
Manes.18G021	0.379285042	3.1173148	0.12167	-3.03894946	0.068	0.38	up	no
Manes.18G022	9.081729104	12.183202	0.74543	-0.4238545	0.528	0	up	yes
Manes.18G024	12.47455599	21.740535	0.573793	-0.80139895	0.108	0.04	down	yes
Manes.18G025	58.23896395	55.563845	1.048145	0.06783826	0.84	0.56	up	no
Manes.18G027	3320.165729	2116.5901	1.568639	0.64951336	#####	0	down	yes
Manes.18G028	3.31258341	1.2417869	2.667594	1.41553922	0.265	0.32	up	no
Manes.18G031	6191.225652	3373.9969	1.834983	0.87576641	#####	0	up	yes
Manes.18G031	1032.285362	529.48972	1.949585	0.96316727	#####	0	up	yes
Manes.18G033	0	11.180125	#NAME?	#NAME?	#####	0.76	down	no
Manes.18G034	30.41290108	54.383942	0.559226	-0.83849728	0.065	0	up	yes
Manes.18G034	14.83088312	11.459272	1.294226	0.37208912	0.507	0.06	down	no
Manes.18G035	21.24274644	17.47971	1.21528	0.28128907	0.571	0.39	down	no
Manes.18G035	17.50233917	15.91189	1.099953	0.13744253	0.814	0.27	down	no
Manes.18G036	498.3855379	637.99332	0.781177	-0.35627912	0.078	0.98	down	no
Manes.18G036	685.4188709	669.34085	1.024021	0.03424485	0.843	0.06	up	no
Manes.18G036	867.8497529	964.633	0.899668	-0.15253487	0.329	0	down	yes

Manes.18G036	500.8343255	949.22882	0.527622	-0.92242246	#####	0	down	yes
Manes.18G036	29.38922356	33.888521	0.867232	-0.20550944	0.74	0.87	down	no
Manes.18G036	35.10239519	15.601315	2.249964	1.1699018	0.067	0.05	down	no
Manes.18G039	1.086713422	1.2593948	0.862885	-0.21275906	1	1	down	no
Manes.18G041	23.69930872	12.504475	1.895266	0.92240049	0.041	0.24	down	no
Manes.18G042	19.33078851	10.830291	1.784882	0.83582846	0.147	0	up	yes
Manes.18G042	1.467299905	0	Inf	Inf	0.128	NA	--	no
Manes.18G042	1.695762722	0	Inf	Inf	0.074	NA	--	no
Manes.18G043	3.261441707	0.6208934	5.25282	2.39309228	0.087	0.08	up	no
Manes.18G043	379.6618329	269.78208	1.407291	0.49292045	0.002	1	down	no
Manes.18G044	6.319991449	12.753883	0.495535	-1.01294199	0.248	0.07	down	no
Manes.18G044	2557.696541	287.57369	8.894056	3.15284149	#####	0.17	down	no
Manes.18G045	109.7678665	397.86912	0.275889	-1.85783816	#####	0	up	no
Manes.18G048	4.429700046	7.1734684	0.617512	-0.69546183	0.509	0.14	down	no
Manes.18G049	151.3080692	137.22684	1.102613	0.1409263	0.5	0.34	down	no
Manes.18G049	420.6787474	764.91072	0.549971	-0.86257243	#####	0.03	down	no
Manes.18G050	5.945538771	8.0463799	0.738909	-0.43653231	0.592	0.24	down	no
Manes.18G052	66.28549474	58.319881	1.136585	0.18470543	0.531	0	up	yes
Manes.18G053	3.717439303	6.8907388	0.539483	-0.89034949	0.495	0.84	down	no
Manes.18G057	131.0919766	0.3172288	413.241	8.69083976	#####	0	up	yes
Manes.18G057	15.40785436	29.590791	0.520698	-0.94148231	0.075	0.22	down	no
Manes.18G058	16.39656211	12.391917	1.323166	0.40399393	0.462	0	up	yes
Manes.18G063	34.47717395	49.748765	0.693026	-0.52901918	0.114	0.83	down	no
Manes.18G063	30.03807499	4.6532457	6.455295	2.6904831	#####	0	up	yes
Manes.18G063	176.951414	203.63878	0.868948	-0.20265907	0.306	0.31	down	no
Manes.18G064	0.683158971	1.5447348	0.44225	-1.17706598	0.661	0.61	down	no
Manes.18G064	471.308465	349.77516	1.347461	0.43024374	0.005	0	up	no
Manes.18G072	403.9212787	186.32234	2.167863	1.11627352	#####	0.75	up	no
Manes.18G073	302.2587858	180.98439	1.670082	0.73991901	#####	0.71	down	no
Manes.18G073	11.58497413	4.6572893	2.487493	1.3146924	0.054	0.21	down	no
Manes.18G075	157.391127	157.04183	1.002224	0.00320529	0.959	0.55	down	no
Manes.18G077	206.3326476	346.72564	0.595089	-0.74882241	#####	0.78	up	no
Manes.18G077	152.1786951	160.0859	0.950606	-0.07307986	0.731	0	down	yes
Manes.18G081	2.808046995	4.0371125	0.695558	-0.52375671	0.691	0.19	down	no
Manes.18G084	1.845283505	0	Inf	Inf	0.072	0.56	up	no
Manes.18G086	64.06928948	43.224751	1.482236	0.56777532	0.056	0	down	yes
Manes.18G089	0.758570083	1.5542554	0.48806	-1.03486922	0.817	0.94	down	no
Manes.18G092	29.82384345	36.39088	0.819542	-0.28711075	0.442	0.48	down	no
Manes.18G093	971.7658493	15.318842	63.43599	5.98722961	#####	0.54	down	no
Manes.18G093	1.416158202	0.9469262	1.495532	0.58065851	0.849	0.56	down	no
Manes.18G093	4.298314868	5.5818474	0.770052	-0.3769715	0.709	0.29	up	no
Manes.18G094	234.7657116	173.26763	1.354931	0.4382196	0.02	0.76	down	no
Manes.18G094	450.8335799	346.97723	1.299317	0.37775399	0.014	0.43	up	no
Manes.18G096	2.123586582	1.5813839	1.342866	0.42531529	0.961	0.32	down	no
Manes.18G097	29.04137737	44.789961	0.64839	-0.62506552	0.136	0.22	up	no
Manes.18G097	151.8007115	13.00805	11.66975	3.54470189	#####	0.78	down	no
Manes.18G098	196.1738698	285.77179	0.68647	-0.54273063	0.003	0.01	up	no
Manes.18G098	0.758570083	2.1839528	0.347338	-1.52558729	0.55	0.06	up	no

Manes.18G098	6.852328195	7.4709395	0.917198	-0.12469543	0.918	0.73	down	no
Manes.18G101	2.174728285	0	Inf	Inf	0.041	NA	--	no
Manes.18G103	5.944237329	10.593015	0.561147	-0.83354959	0.226	0.21	down	no
Manes.18G103	571.5336987	901.11527	0.634251	-0.65687311	#####	0.01	down	no
Manes.18G104	28.78436741	159.38996	0.180591	-2.46920335	#####	0.64	up	no
Manes.18G106	8.597931178	6.5097325	1.320781	0.40139131	0.72	0.49	down	no
Manes.18G106	127.0862807	126.02363	1.008432	0.01211408	0.962	0.01	down	no
Manes.18G109	93.57048419	121.04957	0.772993	-0.3714725	0.12	0.51	down	no
Manes.18G111	1.038174603	1.5582991	0.666223	-0.58592306	0.925	1	down	no
Manes.18G115	1.137855125	0	Inf	Inf	0.207	NA	--	no
Manes.18G116	37.69918694	37.28831	1.011019	0.01581001	0.978	0.04	up	no
Manes.18G116	18.7754838	28.896856	0.649741	-0.62206244	0.167	0.61	down	no
Manes.18G117	15.23146128	30.512278	0.499191	-1.00233554	0.04	0.02	down	yes
Manes.18G119	37.89649973	34.189319	1.108431	0.1485189	0.671	0.56	down	no
Manes.18G119	1.317779122	0.9421659	1.39867	0.48405548	0.742	0.56	down	no
Manes.18G119	13.99169616	19.204598	0.72856	-0.45688088	0.43	0.28	down	no
Manes.18G120	1.745602983	0	Inf	Inf	0.073	NA	--	no
Manes.18G137	39.78511628	48.376321	0.822409	-0.28207225	0.458	0.08	up	no
Manes.18G138	12106.62711	12532.6	0.966011	-0.0498887	0.832	0.47	down	no
Manes.18G138	551.6744669	488.95298	1.128277	0.17412146	0.264	0.01	up	no
Manes.18G140	32.77490402	41.133138	0.7968	-0.32770958	0.354	1	down	no
Manes.18G140	55.12351211	35.944202	1.533586	0.61690873	0.062	0.4	up	no
Manes.18G141	107.0874079	81.896428	1.307596	0.38691642	0.127	0.04	down	no
Manes.18G142	456.8010971	360.08453	1.268594	0.3432305	0.04	0.24	up	no
Manes.18G145	2.60515503	0.9469262	2.75117	1.46004529	0.291	0.56	down	no
Manes.18G146	2.732635882	5.2565312	0.519855	-0.94381782	0.381	0.82	up	no
Manes.S00070(	9.767490959	34.461095	0.283435	-1.81890863	#####	0.96	up	no
Manes.S00250(	61.90303207	75.485719	0.820063	-0.28619365	0.297	0.19	up	no
Manes.S00260(	28.69342358	40.343997	0.711219	-0.49163389	0.189	0.02	down	yes
Manes.S00280(	2.330382873	0.9374057	2.485992	1.31382158	0.622	0.29	up	no
Manes.S00320(	6.678538002	5.9390523	1.124512	0.16929957	0.863	0.83	down	no
Manes.S00400(	10.27072593	11.168455	0.919619	-0.12089147	0.894	0.6	up	no
Manes.S00480(	64.15502755	47.191893	1.35945	0.44302329	0.14	0.66	up	no
Manes.S00540(	4.021313232	4.9873658	0.8063	-0.31061131	0.809	0.41	down	no
Manes.S00600(	80.83278444	73.702274	1.096747	0.1332314	0.656	0.34	down	no
Manes.S00700(	0.329444781	1.8714842	0.176034	-2.50607432	0.273	0.49	down	no
Manes.S00720(	5.642966284	6.1986971	0.910347	-0.13551128	0.955	0.35	down	no
Manes.S01100(	11.71468446	10.597058	1.105466	0.14465427	0.774	0.54	down	no
Manes.S01270(	6.227746174	5.5330672	1.12555	0.17063069	0.937	0.76	down	no
Manes.S01750(	3.717439303	20.194367	0.184083	-2.4415718	0.009	0.02	down	yes
Manes.S01960(	267.3396643	317.31598	0.842503	-0.24724625	0.16	0	up	no
Manes.S02030(	20.7945575	13.351691	1.557447	0.63918349	0.2	0.94	down	no
Manes.S02060(	4.175666378	9.0002162	0.463952	-1.10795321	0.192	0	down	yes
Manes.S02320(	5.944237329	7.1361028	0.832981	-0.26364467	0.805	0.88	down	no
Manes.S02380(	57.83986846	121.22516	0.477128	-1.06755293	0.003	0.17	up	no
Manes.S02670(	191.8130737	201.90391	0.950022	-0.07396783	0.714	0.5	down	no
Manes.S02720(	1.695762722	0.3172288	5.34555	2.4183385	0.224	1	down	no
Manes.S02800(	355.6022907	392.72335	0.905478	-0.14324876	0.383	0.26	down	no

Manes.S02860(	2.478602214	5.3188755	0.466001	-1.10159451	0.27	0.31	down	no
Manes.S02910(	5.764313331	0.6249371	9.223829	3.20536584	0.096	0.66	up	no
Manes.S03060(	61.89429539	70.164694	0.882129	-0.18093882	0.537	0	down	yes
Manes.S03070(	18.66966947	29.564635	0.631487	-0.6631761	0.174	0	down	yes
Manes.S03380(	14.02209937	18.692935	0.750128	-0.41479073	0.566	0.8	down	no
Manes.S03730(	5.766916215	2.1879965	2.635706	1.39818964	0.125	0.03	up	yes
Manes.S03750(	23.32132512	30.148163	0.773557	-0.37042034	0.367	0.9	up	no
Manes.S04130(	48.57078011	58.805848	0.825952	-0.27587098	0.39	0.01	up	no
Manes.S04170(	121.2199728	92.829295	1.305837	0.38497535	0.111	0	up	yes
Manes.S04490(	4.251077491	34.085535	0.124718	-3.00325918	#####	0	down	yes
Manes.S04500(	2.578282737	1.8714842	1.377667	0.46222758	0.815	0.23	down	no
Manes.S04530(	9.411173885	1.5590157	6.036613	2.59373924	0.005	0.55	up	no
Manes.S04550(	16.67319034	1.2505908	13.33225	3.73684845	#####	0.02	up	yes
Manes.S04620(	4.122295196	2.7505892	1.498695	0.58370711	0.748	0.71	up	no
Manes.S04630(	398.8734072	482.73383	0.82628	-0.275297	0.073	0.08	up	no
Manes.S04700(	123.255773	141.19851	0.872925	-0.19606966	0.387	0.83	up	no
Manes.S04940(	1.036873161	0.3172288	3.268534	1.7086436	0.56	1	down	no
Manes.S04950(	1.744301541	0.6344577	2.74928	1.45905364	0.622	0.71	down	no
Manes.S04970(	27.08860469	26.733377	1.013288	0.01904398	1	0.34	down	no
Manes.S04980(	29.57724503	55.183555	0.535979	-0.89975073	0.008	0.01	down	yes
Manes.S05620(	205.4766495	323.41275	0.635339	-0.65440211	0.001	0	down	yes
Manes.S05630(	3.767279564	5.3467206	0.704596	-0.50513119	0.89	0.59	down	no
Manes.S05700(	1.845283505	0	Inf	Inf	0.072	0.56	up	no
Manes.S05830(	1.517140166	6.8914554	0.220148	-2.18345432	0.169	0.43	down	no
Manes.S05970(	1.062444012	5.8712311	0.180958	-2.46627623	0.035	0.08	down	no
Manes.S06010(	29.82421686	19.254811	1.548923	0.63126529	0.157	0.45	up	no
Manes.S06160(	46.98083172	44.496277	1.055837	0.07838761	0.798	0.04	up	no
Manes.S06170(	1.086713422	1.5406912	0.705342	-0.50360616	0.927	1	down	no
Manes.S06560(	31.19108943	31.661009	0.985158	-0.0215733	1	0.95	down	no
Manes.S06820(	16.6450166	18.341207	0.90752	-0.13999826	0.9	1	down	no
Manes.S06890(	24.63036756	28.96611	0.850317	-0.2339278	0.573	0.01	down	yes
Manes.S06980(	422.9846106	270.37086	1.564461	0.64566551	6E-04	0.38	down	no
Manes.S07150(	269.1167291	444.77986	0.605056	-0.72485937	#####	0	up	yes
Manes.S07380(	68.23752061	57.168774	1.193615	0.25533787	0.372	0.89	down	no
Manes.S07400(	0.658889561	1.5406912	0.427658	-1.22546912	0.768	1	down	no
Manes.S07590(	2.075047763	3.1125545	0.66667	-0.58495455	0.734	0.34	down	no
Manes.S07720(	96.53325773	79.348646	1.216571	0.28282046	0.353	0.04	up	no
Manes.S07780(	264.3877833	266.77376	0.991056	-0.01296126	0.945	0	up	no
Manes.S07790(	12.13804937	5.876708	2.06545	1.04645648	0.121	0.07	up	no
Manes.S07930(	0	10.374093	#NAME?	#NAME?	#####	0.04	down	yes
Manes.S07950(	80.02371186	50.296586	1.591037	0.66996708	0.04	0.17	down	no
Manes.S08530(	49.75531798	64.35463	0.773143	-0.37119321	0.258	0	down	yes
Manes.S08550(	139.6063992	189.37134	0.73721	-0.43985292	0.036	0	down	yes
Manes.S08740(	166.6555752	355.73285	0.468485	-1.09392462	#####	0.04	down	no
Manes.S08780(	162.8739797	119.79422	1.359615	0.44319788	0.039	0.72	up	no
Manes.S08790(	274.4025119	181.62622	1.510809	0.59532122	0.002	0.01	down	no
Manes.S08850(	2.149157434	3.0813823	0.697465	-0.51980653	0.736	0.82	up	no
Manes.S08980(	123.057821	171.42112	0.717868	-0.47820851	0.036	0	up	yes

Manes.S09050(	97.55124848	121.11642	0.805434	-0.31216218	0.246	0.35	down	no
Manes.S09060(	1.746904425	0	Inf	Inf	0.073	NA	--	no
Manes.S09070(	4.503809717	5.2382066	0.8598	-0.21792709	0.898	0.71	down	no
Manes.S09140(	165.8111824	185.7507	0.892654	-0.1638263	0.534	0	down	yes
Manes.S09230(	181.8662134	193.2716	0.940988	-0.08775212	0.68	0.6	down	no
Manes.S09320(	31.08955284	33.410415	0.930535	-0.10386805	0.775	0	down	yes
Manes.S09330(	52.23577627	46.10955	1.132862	0.17997266	0.568	0.01	down	yes
Manes.S09350(	26.33746985	0.3172288	83.02357	6.37544903	#####	0	up	yes
Manes.S09460(	0	1.2417869	#NAME?	#NAME?	0.226	0.87	up	no
Manes.S09470(	0.379285042	2.5099856	0.15111	-2.72632471	0.137	0.31	down	no
Manes.S09550(	99.60537653	127.0793	0.783805	-0.35143353	0.136	0.98	up	no
Manes.S09660(	78.67330005	172.52109	0.456021	-1.13282674	#####	0	down	no
Manes.S09690(	2.554013327	10.561126	0.241832	-2.04792568	0.007	0.78	up	no
Manes.S09700(	8.041325023	5.9357252	1.354733	0.43800893	0.564	0.36	up	no
Manes.S09930(	39.56446212	60.01503	0.659243	-0.6011187	0.06	0.49	down	no
Manes.S10070(	24.66634994	71.61496	0.34443	-1.53771675	2E-04	0	up	yes
Manes.S10200(	45.48275519	60.63879	0.75006	-0.41492131	0.234	0.01	down	yes
Manes.S10210(	329.9623078	396.48916	0.83221	-0.26498019	0.166	0	down	yes
Manes.S10400(	5.965903854	4.0283085	1.480995	0.56656654	0.48	0.92	up	no
Manes.S10530(	73.83688804	147.39216	0.500955	-0.99724615	0.001	0	down	yes
Manes.S10570(	19.98224283	5.8733809	3.40217	1.76645542	0.028	0	up	yes
Manes.S10630(	45.88445357	31.842105	1.440999	0.52706973	0.126	0	up	yes
Manes.S10890(	53.07942219	51.841095	1.023887	0.03405648	0.896	0.1	up	no

Supplemental Data S3. lncRNAs as potential miRNA precursors

miRNA precursor	Mature miRNA sequence (5'-3')	miRNA	Cold/CK	Drought/CK
lincRNA_151	UUGACGACGAGAGAGAGCACG	miR535c	1.118266	1.372193666
lincRNA_230	UGCCUGGCUCCCUGAAUGCCA	miR160d	0.855571	0.300926166
lincRNA_268	UGACAGAAGAGAGUGAGCAC	miR156g	1.773283	1.64006711
lincRNA_315	CAGCCAAGGAUGACUUGCCGG	miR169d	1.541803	1.042463656
lincRNA_342	UGAAGCUGCCAGCAUGAUCUGA	miR167f	0.245834	0.078127889
lincRNA_37	UGACAACGAGAGAGAGCACGG	miR535a	0.814525	1.784632248
lincRNA_384	UCGGACCAGGCUUCAUUCCCC	miR166h	0.247914	0.755534647
lincRNA_55	UUGACAGAAGAGAGAGAGCAC	miR156h	1.266944	0.335959476
lincRNA_63	UUGGAAGAGCUUACUUUAAAU	novel-28	0.377603	0.402718195
lincRNA_79	UUGCAAGGAUGCAUGGUACUA	novel-19	1.229939	1.176487395
lncNAT_181	ACUUCUGUCUCAGAUGGCUCAC	novel-4	5.013437	0.470594731
lncNAT_53	ACUGGGAACAGGCAGAGCAUG	miR408b	2.500324	0.832195466

Supplemental Data S4. lncRNAs as potential miRNA target mimics

<table>
<tr><th>lncRNA ID</th><th>ıcRNA-miRNA interaction regic</th><th>location</th><th>miRNA ID</th><th>miRNA sequence</th></tr>
<tr><td>lincRNA_290</td><td>UUCUGAACUC---UCUCCCUCAU
||||*||||* |||||||||*
AAGAGUUGAUUAGAGAGGGAGUU</td><td>223-246</td><td>mes-miR1446</td><td>UUCUGAACUCUCUCCCUCAU</td></tr>
<tr><td>lincRNA_271</td><td>UUGACAGAAG---AUAGAGAGCAC
|||||||||: |*|||**||||
AACUGUCUUUUUAUUUCUAGCGUG</td><td>774-798</td><td>mes-miR156h</td><td>UGACAGAAGAUAGAGAGCA</td></tr>
<tr><td>lncNAT_7</td><td>AUUGGAGUGA---AGGGAGCUCUG
|||:||:*:| ||:|||||||:
UAAUCUUCUUUCAUCUCUCGAGAU</td><td>383-407</td><td>mes-miR159c</td><td>UUGGAGUGAAGGGAGCUCU</td></tr>
<tr><td>lncNAT_159</td><td>UGGAGAAGCA---GGGCACGUGCA
:||||||| ||||||||||*
CUCUCGUCGUUUACCCGUGCACGC</td><td>2603-262</td><td>mes-miR164a</td><td>GGAGAAGCAGGGCACGUGC</td></tr>
<tr><td>lincRNA_161</td><td>UUGGACCAGG---CUUCAUUCCCC
|||*|**||| ||||||||||*
AACAUCUUCCGUCGAAGUAAGGGU</td><td>509-533</td><td>mes-miR166i</td><td>UGGACCAGGCUUCAUUCCC</td></tr>
<tr><td>lincRNA_340</td><td>UGAGCCAAGG---AUGACUUGCCG
|:|||||||* ||||*:||||*
AUUCGGUUCAGUUUACUCGACGGG</td><td>464-488</td><td>mes-miR169h</td><td>GAGCCAAGGAUGACUUGCC</td></tr>
<tr><td>lincRNA_13</td><td>AGAUUGAGCC---GCGCCAAUAUC
||||||*||| ||:|*||*|||
UCUAACACGGAUACGUGAUUCUAG</td><td>1985-200</td><td>mes-miR171e</td><td>GAUUGAGCCGCGCCAAUAU</td></tr>
<tr><td>lncNAT_114</td><td>AGAAUCUUGA---UGAUGCUGCAU
|||||||* ||*||||||||
ACCUAGAACGGCUACAACGACGUA</td><td>2732-275</td><td>mes-miR172a</td><td>GAAUCUUGAUGAUGCUGCA</td></tr>
<tr><td>lincRNA_119</td><td>UUUGGUUUCC---UCCAAUAUCUUA
|||**||||* ||||||||||*|
AAAAAAAAGAAGAAGGUUAUAGAUU</td><td>716-741</td><td>mes-miR2275</td><td>UUGGUUUCCUCCAAUAUCUU</td></tr>
<tr><td>lncNAT_78</td><td>UUGGACUGAA---GGGAGCUCCCU
|||||||||| ||*|:||**||
AACCUGACUUUGACCGUUGAAUGA</td><td>1783-180</td><td>mes-miR319a</td><td>UGGACUGAAGGGAGCUCCC</td></tr>
<tr><td>lncNAT_142</td><td>UCCAAAGGGA---UCGCAUUGAUCU
||*||||::| ||*|*|||||||
AGAUUUCUUUCGAAGAGAAACUAGA</td><td>2217-224</td><td>mes-miR393c</td><td>CCAAAGGGAUCGCAUUGAU</td></tr>
<tr><td>lncNAT_60</td><td>UUGGCAUUCU---GUCCACCUCCAU
|||:*||||| |||**|||||||
AACUUUAAGAAUUCAGUAGGAGGUA</td><td>605-630</td><td>mes-miR394c</td><td>UGGCAUUCUGUCCACCUCCA</td></tr>
<tr><td>lncNAT_182</td><td>CUGAAGGGUU---UGGAGGAACUC
|||||*||:| ||||*|||*:|
GACUUACCGAAGUACCUACUUCGG</td><td>1425-144</td><td>mes-miR395e</td><td>UGAAGGGUUUGGAGGAACU</td></tr>
<tr><td>lincRNA_74</td><td>UUCCACAGCU---UUCUUGAACUG
||||||*||| ||*:||||*||
AAGGUGACGAGUCAAAGACUUUAC</td><td>155-179</td><td>mes-miR396a</td><td>UCCACAGCUUUCUUGAACU</td></tr>
<tr><td>lncNAT_124</td><td>ACUCUCCCUC---AAGGGCUUCAG
|||||*|||* |||:||||||:
UGAGAAGGAACGAUUCUCGAAGUU</td><td>3958-398</td><td>mes-miR477h</td><td>CUCUCCCUCAAGGGCUUCA</td></tr>
<tr><td>lncNAT_169</td><td>UGCAUUUGCA---CCUGCACCUU
|||||||||| |*|||***||
ACGUAAACGUUCUGAACGCUUAA</td><td>209-232</td><td>mes-miR530a</td><td>UGCAUUUGCACCUGCACCUU</td></tr>
</table>

Supplemental Data S5. lncNATs associated with small RNAs and expresion information.

lncNAT	siRNA reads				siRNA Fold Change			lncNAT Fold Change	
	CA	CCA	CS	NC	CCA/N(	CA/NC	CS/NC	Cold/CK	Drought/CK
lncNAT_229	15	146	13	12	11.23	1.15	1.00	2.07	1.62
lncNAT_76	558	318	120	44	7.07	12.40	2.67	47.34	6.09
lncNAT_43	258	794	181	141	5.59	1.82	1.27	2.53	1.18
lncNAT_222	129	219	67	63	3.42	2.02	1.05	2.23	0.25
lncNAT_36	58	215	68	88	2.42	0.65	0.76	0.28	0.40
lncNAT_95	35	58	34	24	2.32	1.40	1.36	0.22	0.59
lncNAT_110	33	54	35	26	2.00	1.22	1.30	55.30	2.22
lncNAT_149	56	75	29	41	1.79	1.33	0.69	0.45	0.70
lncNAT_94	268	382	226	267	1.43	1.00	0.84	0.48	1.11
lncNAT_179	701	1152	761	876	1.31	0.80	0.87	3.11	2.61
lncNAT_73	47	47	44	36	1.27	1.27	1.19	42.70	1.65
lncNAT_61	13	24	16	22	1.04	0.57	0.70	2.83	2.29
lncNAT_195	37	30	36	29	1.00	1.23	1.20	62.09	74.88
lncNAT_212	18	24	24	26	0.89	0.67	0.89	0.41	0.89
lncNAT_185	17	16	24	23	0.67	0.71	1.00	0.74	0.07
lncNAT_156	168	139	190	213	0.65	0.79	0.89	0.17	1.02
lncNAT_109	29	10	19	15	0.63	1.81	1.19	0.22	1.33
lncNAT_64	15	7	7	14	0.47	1.00	0.47	2.41	1.78
lncNAT_33	15	14	30	41	0.33	0.36	0.71	0.48	0.96
lncNAT_104	36	93	29	17	5.17	2.00	1.61	0.73	1.30
lncNAT_12	31	40	18	27	1.43	1.11	0.64	0.56	1.50
lncNAT_128	1035	1034	648	552	1.87	1.87	1.17	0.65	1.12
lncNAT_134	90	73	72	82	0.88	1.08	0.87	1.90	1.13
lncNAT_135	311	305	229	305	1.00	1.02	0.75	1.50	0.32
lncNAT_14	13	76	15	23	3.17	0.54	0.63	1.94	0.71
lncNAT_157	6	115	1	2	38.33	2.00	0.33	1.15	1.05
lncNAT_172	11	24	10	6	3.43	1.57	1.43	0.74	1.01
lncNAT_187	50	52	23	24	2.08	2.00	0.92	1.45	1.19
lncNAT_191	42	35	30	20	1.67	2.00	1.43	1.99	1.95
lncNAT_192	235	249	154	528	0.47	0.44	0.29	1.08	1.16
lncNAT_22	254	463	158	147	3.13	1.72	1.07	1.22	1.38
lncNAT_223	1432	2365	1233	1950	1.21	0.73	0.63	0.85	1.76
lncNAT_34	31	38	29	27	1.36	1.11	1.04	3.44	6.28
lncNAT_40	16	43	20	28	1.48	0.55	0.69	0.56	0.79
lncNAT_48	1458	3155	1528	1626	1.94	0.90	0.94	1.39	1.17
lncNAT_58	1602	1289	1149	1128	1.14	1.42	1.02	0.64	0.94
lncNAT_69	1271	1047	805	675	1.55	1.88	1.19	0.83	1.23
lncNAT_77	355	352	314	336	1.04	1.05	0.93	1.03	1.29
lncNAT_8	100	92	72	62	1.46	1.59	1.14	0.53	0.63
lncNAT_83	123	106	172	164	0.64	0.75	1.04	1.03	0.75
lncNAT_88	830	581	511	477	1.22	1.74	1.07	1.15	1.14
lncNAT_90	15	203	12	9	20.30	1.50	1.20	0.95	0.88

Supplemental Data S6. Expression information of lincRNAs and nearby protein-coding genes under cold stress

	lincRNA ID	Cold/CK	strand	Left gene ID	Cold/CK	Left g	Left gene	lincRNA ID	Cold/CK	strand	Right gene ID	Cold/CK	Right g	Right gene
	lincRNA_147	1.92	-	Manes.13G141400.v6.1	22.72	+	-634	lincRNA_114	8.64	-	Manes.12G092700.v6.1	Inf	+	-2808
	lincRNA_91	2.43	-	Manes.15G183200.v6.1	17.79	-	-7594	lincRNA_143	1.89	+	Manes.13G114800.v6.1	5.65	+	-2193
	lincRNA_245	11.60	-	Manes.02G213200.v6.1	15.51	-	-5968	lincRNA_147	1.92	-	Manes.13G141500.v6.1	5.54	+	-6208
	lincRNA_234	6.26	+	Manes.07G130600.v6.1	14.35	+	-1353	lincRNA_419	15.95	-	Manes.04G126100.v6.1	4.32	+	-7589
	lincRNA_70	130.72	+	Manes.17G057700.v6.1	11.07	+	-4016	lincRNA_257	31.10	-	Manes.02G122700.v6.1	3.89	-	-208
	lincRNA_2	2.57	+	Manes.S033700.v6.1	8.36	+	-1913	lincRNA_445	11.43	+	Manes.08G166600.v6.1	3.27	+	-3667
	lincRNA_397	9.31	+	Manes.05G187700.v6.1	5.32	+	-8828	lincRNA_318	3.67	-	Manes.01G250600.v6.1	2.99	-	-1329
	lincRNA_419	15.95	-	Manes.04G126000.v6.1	5.24	-	-5905	lincRNA_306	61.06	+	Manes.01G123100.v6.1	2.98	+	-2772
	lincRNA_21	3.34	+	Manes.10G127100.v6.1	4.89	-	-8831	lincRNA_53	2.71	+	Manes.16G108100.v6.1	2.93	+	-2508
	lincRNA_315	1.54	-	Manes.01G208000.v6.1	4.89	+	-93	lincRNA_237	155.42	-	Manes.07G050000.v6.1	2.74	-	-726
	lincRNA_376	46.38	+	Manes.09G062400.v6.1	3.67	+	-3405	lincRNA_1	2.20	+	Manes.S034000.v6.1	2.70	+	-4307
	lincRNA_122	1.79	-	Manes.12G120700.v6.1	3.56	-	-9178	lincRNA_20	11.27	+	Manes.10G123500.v6.1	2.67	+	-678
	lincRNA_403	Inf	+	Manes.05G063900.v6.1	3.56	-	-501	lincRNA_286	10.61	-	Manes.06G161400.v6.1	2.44	-	-679
	lincRNA_110	3.20	+	Manes.15G103800.v6.1	3.50	+	-116	lincRNA_141	3.47	-	Manes.13G089600.v6.1	2.33	-	-13
	lincRNA_453	2.28	+	Manes.S085700.v6.1	3.36	-	-6710	lincRNA_105	8.83	-	Manes.15G060000.v6.1	2.22	+	-7884
	lincRNA_3	2.77	+	Manes.S034000.v6.1	2.70	+	-9777	lincRNA_123	6.28	+	Manes.12G123100.v6.1	2.16	+	-25
	lincRNA_327	1.50	-	Manes.03G092100.v6.1	2.67	+	-2060	lincRNA_366	2.16	-	Manes.09G157700.v6.1	2.13	-	-445
	lincRNA_232	4.17	+	Manes.07G112600.v6.1	2.54	+	-841	lincRNA_167	5.15	+	Manes.11G153800.v6.1	1.99	+	-2493
	lincRNA_366	2.16	-	Manes.09G157600.v6.1	2.51	+	-2645	lincRNA_453	2.28	+	Manes.S085800.v6.1	1.93	+	-5086
	lincRNA_258	3.17	+	Manes.02G132100.v6.1	2.38	-	-1914	lincRNA_56	112.66	-	Manes.16G130200.v6.1	1.91	-	-516
	lincRNA_318	3.67	-	Manes.01G250500.v6.1	2.32	-	-29	lincRNA_448	1.83	+	Manes.08G053800.v6.1	1.91	+	-151
	lincRNA_204	2.88	+	Manes.18G048200.v6.1	2.27	+	-1749	lincRNA_203	2.82	+	Manes.18G045000.v6.1	1.88	+	-2248
	lincRNA_18	2.26	-	Manes.10G110100.v6.1	2.26	-	-5307	lincRNA_169	2.10	+	Manes.11G072300.v6.1	1.81	+	-9451
	lincRNA_33	1.68	-	Manes.10G011800.v6.1	2.22	+	-5425	lincRNA_236	Inf	-	Manes.07G046900.v6.1	1.79	-	-1700
Concordant	lincRNA_365	1.79	-	Manes.09G148600.v6.1	2.01	+	-7529	lincRNA_234	6.26	+	Manes.07G130700.v6.1	1.75	-	-433
	lincRNA_138	40.33	+	Manes.13G015400.v6.1	1.95	+	-838	lincRNA_32	71.21	-	Manes.10G062400.v6.1	1.68	-	-899
	lincRNA_101	1.65	+	Manes.15G003200.v6.1	1.93	+	-478	lincRNA_206	2.18	+	Manes.18G054800.v6.1	1.68	-	-3028
	lincRNA_174	61.63	+	Manes.14G142200.v6.1	1.78	+	-278	lincRNA_431	Inf	+	Manes.08G080800.v6.1	1.61	-	-6232
	lincRNA_203	2.82	+	Manes.18G044900.v6.1	1.71	+	-1282	lincRNA_45	44.76	-	Manes.16G060700.v6.1	1.59	-	-1419
	lincRNA_237	155.42	-	Manes.07G049900.v6.1	1.65	+	-1420	lincRNA_204	2.88	+	Manes.18G048300.v6.1	1.55	-	-974
	lincRNA_411	Inf	+	Manes.04G057600.v6.1	1.63	+	-1794	lincRNA_161	1.88	-	Manes.11G101400.v6.1	1.51	-	-7393
	lincRNA_139	2.41	+	Manes.13G087300.v6.1	1.56	+	-1493	lincRNA_121	0.38	+	Manes.12G119600.v6.1	0.64	-	-4878
	lincRNA_311	5.04	-	Manes.01G166700.v6.1	1.53	-	-1575	lincRNA_401	0.15	-	Manes.05G048800.v6.1	0.64	+	-806
	lincRNA_401	0.15	-	Manes.05G048700.v6.1	0.67	+	-5684	lincRNA_430	0.67	+	Manes.08G015000.v6.1	0.63	+	-1917
	lincRNA_378	0.44	-	Manes.S087000.v6.1	0.63	-	-1656	lincRNA_209	0.50	+	Manes.18G082600.v6.1	0.62	+	-5231
	lincRNA_395	0.42	-	Manes.05G183400.v6.1	0.57	+	-121	lincRNA_75	0.14	-	Manes.17G094600.v6.1	0.62	+	-363
	lincRNA_248	0.39	+	Manes.02G035500.v6.1	0.57	-	-2780	lincRNA_177	0.39	+	Manes.14G148500.v6.1	0.58	+	-6213
	lincRNA_54	0.34	+	Manes.16G127200.v6.1	0.55	+	-1998	lincRNA_189	0.56	-	Manes.14G075300.v6.1	0.56	-	-5165
	lincRNA_163	0.63	-	Manes.11G105400.v6.1	0.54	+	-6799	lincRNA_146	0.67	-	Manes.13G130000.v6.1	0.55	-	-209
	lincRNA_341	0.32	-	Manes.03G009700.v6.1	0.49	+	-581	lincRNA_340	0.41	+	Manes.03G009700.v6.1	0.49	+	-124
	lincRNA_120	0.48	+	Manes.12G102500.v6.1	0.48	-	-9283	lincRNA_368	0.62	+	Manes.09G170500.v6.1	0.42	+	-1530
	lincRNA_189	0.56	-	Manes.14G075200.v6.1	0.46	-	-3387	lincRNA_172	0.67	-	Manes.14G134600.v6.1	0.38	-	-6157
	lincRNA_209	0.50	+	Manes.18G082500.v6.1	0.46	+	-4939	lincRNA_378	0.44	-	Manes.S087100.v6.1	0.35	-	-490
	lincRNA_177	0.39	+	Manes.14G148400.v6.1	0.45	+	-312	lincRNA_272	0.49	-	Manes.06G057300.v6.1	0.33	-	-666
	lincRNA_109	0.43	+	Manes.15G083200.v6.1	0.44	+	-420	lincRNA_341	0.32	-	Manes.03G009800.v6.1	0.29	-	-3291
	lincRNA_71	0.38	-	Manes.17G059200.v6.1	0.44	-	-1575	lincRNA_191	0.66	+	Manes.14G085200.v6.1	0.27	+	-5595
	lincRNA_6	0.34	-	Manes.10G074200.v6.1	0.34	-	-4107	lincRNA_307	0.42	+	Manes.01G130000.v6.1	0.19	-	-1307
	lincRNA_382	0.65	+	Manes.05G118400.v6.1	0.23	+	-1156	lincRNA_248	0.39	+	Manes.02G035600.v6.1	0.19	-	-3502
	lincRNA_140	0.38	-	Manes.13G088300.v6.1	0.22	-	-57	lincRNA_107	Inf	+	Manes.15G076000.v6.1	#NAME?	+	-4202
	lincRNA_187	343.89	-	Manes.14G068700.v6.1	0.67	+	-3041	lincRNA_365	1.79	-	Manes.09G148700.v6.1	0.67	-	-113
	lincRNA_136	19.62	+	Manes.13G082600.v6.1	0.66	+	-1576	lincRNA_279	15.47	+	Manes.06G125600.v6.1	0.66	+	-1476
	lincRNA_277	8.13	+	Manes.06G118600.v6.1	0.63	+	-1865	lincRNA_136	19.62	+	Manes.13G082700.v6.1	0.60	-	-2339
	lincRNA_317	2.15	-	Manes.01G209800.v6.1	0.60	-	-83	lincRNA_311	5.04	-	Manes.01G166800.v6.1	0.55	-	-2580
	lincRNA_24	3.25	+	Manes.10G047400.v6.1	0.57	-	-8247	lincRNA_317	2.15	-	Manes.01G209900.v6.1	0.54	+	-3847
	lincRNA_56	112.66	-	Manes.16G130100.v6.1	0.57	+	-3307	lincRNA_334	344.86	+	Manes.03G032300.v6.1	0.46	+	-5201
	lincRNA_324	10.37	+	Manes.03G086800.v6.1	0.54	+	-1813	lincRNA_33	1.68	-	Manes.10G011900.v6.1	0.42	+	-2769
	lincRNA_303	4.45	+	Manes.01G059700.v6.1	0.49	+	-3240	lincRNA_277	8.13	+	Manes.06G118700.v6.1	0.33	-	-2449
	lincRNA_286	10.61	-	Manes.06G161300.v6.1	0.36	+	-78	lincRNA_188	0.66	+	Manes.14G072400.v6.1	Inf	-	-137
	lincRNA_326	Inf	+	Manes.03G018200.v6.1	0.19	-	-666	lincRNA_352	0.51	+	Manes.09G076700.v6.1	Inf	-	-652
Discordant l	lincRNA_179	0.63	-	Manes.14G151500.v6.1	Inf	+	-9117	lincRNA_285	0.34	+	Manes.06G146600.v6.1	Inf	+	-7837
	lincRNA_359	0.60	-	Manes.09G090500.v6.1	Inf	+	-7580	lincRNA_71	0.38	-	Manes.17G059300.v6.1	12.18	+	-3448
	lincRNA_340	0.41	+	Manes.03G009600.v6.1	13.02	-	-1755	lincRNA_181	0.53	-	Manes.14G161600.v6.1	12.03	-	-2628
	lincRNA_307	0.42	+	Manes.01G129900.v6.1	7.58	+	-6196	lincRNA_440	0.67	-	Manes.08G034000.v6.1	8.72	+	-9242
	lincRNA_264	0.66	-	Manes.S002400.v6.1	5.64	+	-2223	lincRNA_420	0.39	-	Manes.04G152700.v6.1	2.37	-	-1974
	lincRNA_414	0.43	-	Manes.04G012800.v6.1	2.51	-	-4102	lincRNA_308	0.47	+	Manes.01G132000.v6.1	2.30	+	-5404
	lincRNA_310	0.47	-	Manes.01G145500.v6.1	2.18	+	-5937	lincRNA_195	0.54	+	Manes.18G021600.v6.1	2.25	-	-271
	lincRNA_278	0.58	-	Manes.06G120300.v6.1	1.93	-	-3130	lincRNA_354	0.42	+	Manes.09G079500.v6.1	2.10	-	-2464
	lincRNA_440	0.67	-	Manes.08G033900.v6.1	1.82	+	-796	lincRNA_115	0.66	-	Manes.12G094100.v6.1	1.50	-	-4288
	lincRNA_434	0.56	-	Manes.08G092200.v6.1	1.64	+	-1306							
	lincRNA_284	0.53	+	Manes.06G141400.v6.1	1.62	+	-2942							
	lincRNA_330	1.25	+	Manes.03G026300.v6.1	#######	-	-613	lincRNA_207	4.53	+	Manes.18G005200.v6.1	#NAME?	+	-80
	lincRNA_76	1.15	-	Manes.17G102900.v6.1	#######	+	-1016	lincRNA_110	3.20	+	Manes.15G103900.v6.1	#NAME?	+	-2461
	lincRNA_314	0.87	+	Manes.01G204000.v6.1	#######	-	-4626	lincRNA_246	1.11	-	Manes.02G213400.v6.1	#NAME?	+	-778
	lincRNA_399	0.76	+	Manes.05G200400.v6.1	#######	+	-3565	lincRNA_355	0.84	-	Manes.09G081100.v6.1	#NAME?	+	-3732
	lincRNA_107	Inf	+	Manes.15G075900.v6.1	NA	+	-1594	lincRNA_37	0.81	+	Manes.S087700.v6.1	#NAME?	-	-2274
	lincRNA_141	3.47	-	Manes.13G089500.v6.1	NA	+	-5727	lincRNA_403	Inf	+	Manes.05G064000.v6.1	NA	-	-663
	lincRNA_269	2.05	-	Manes.06G039000.v6.1	NA	+	-9817	lincRNA_245	11.60	-	Manes.02G213300.v6.1	NA	+	-527
	lincRNA_370	1.58	+	Manes.09G001700.v6.1	NA	-	-1066	lincRNA_266	2.11	+	Manes.S011500.v6.1	NA	+	-7009
	lincRNA_126	1.56	-	Manes.12G146900.v6.1	NA	+	-1741	lincRNA_269	2.05	-	Manes.06G039100.v6.1	NA	+	-416
	lincRNA_183	1.15	-	Manes.14G166200.v6.1	NA	+	-210	lincRNA_88	1.37	-	Manes.15G134800.v6.1	NA	+	-301
	lincRNA_158	1.13	+	Manes.11G088100.v6.1	NA	-	-2556	lincRNA_413	1.19	-	Manes.04G058200.v6.1	NA	+	-3910
	lincRNA_246	1.11	-	Manes.02G213300.v6.1	NA	+	-8098	lincRNA_133	0.69	-	Manes.13G076400.v6.1	NA	+	-5067
	lincRNA_433	0.92	-	Manes.08G085400.v6.1	NA	+	-4918	lincRNA_423	0.68	-	Manes.04G049300.v6.1	NA	+	-2985
	lincRNA_348	0.92	-	Manes.09G073200.v6.1	NA	+	-5661	lincRNA_162	1.37	+	Manes.11G101500.v6.1	Inf	-	-3117
	lincRNA_335	0.85	-	Manes.03G195000.v6.1	NA	-	-736	lincRNA_8	0.77	-	Manes.10G075900.v6.1	Inf	+	-5959
	lincRNA_144	0.79	-	Manes.13G115600.v6.1	NA	+	-2645	lincRNA_216	0.98	+	Manes.S050000.v6.1	28.81	-	-5097

	lincRNA_68	0.71	-	Manes.17G045100.v6.1	NA	+	-2236	lincRNA_66	1.37	-	Manes.17G033400.v6.1	23.49	-	-1736
	lincRNA_333	1.24	+	Manes.03G032200.v6.1	Inf	-	-6101	lincRNA_176	0.77	-	Manes.14G145700.v6.1	14.48	-	-965
	lincRNA_373	1.16	-	Manes.09G048900.v6.1	Inf	+	-6677	lincRNA_108	0.97	-	Manes.15G005500.v6.1	9.33	-	-841
	lincRNA_239	1.05	+	Manes.07G064500.v6.1	Inf	+	-7569	lincRNA_398	1.39	-	Manes.05G195500.v6.1	3.42	+	-4313
	lincRNA_66	1.37	-	Manes.17G033300.v6.1	14.39	+	-6932	lincRNA_254	1.14	+	Manes.02G043900.v6.1	2.62	+	-5260
	lincRNA_374	0.79	-	Manes.09G055000.v6.1	11.40	+	-3021	lincRNA_287	1.09	+	Manes.06G164800.v6.1	2.41	-	-3203
	lincRNA_435	0.92	+	Manes.08G002500.v6.1	9.53	-	-1309	lincRNA_39	0.81	+	Manes.16G011900.v6.1	2.28	-	-1831
	lincRNA_73	1.22	-	Manes.17G074700.v6.1	6.02	-	-332	lincRNA_168	0.81	-	Manes.11G064300.v6.1	2.04	+	-2299
	lincRNA_100	1.25	-	Manes.15G035000.v6.1	2.46	-	-3385	lincRNA_392	0.73	-	Manes.05G029600.v6.1	1.88	-	-3058
	lincRNA_404	1.24	-	Manes.05G070700.v6.1	2.34	-	-445	lincRNA_332	1.21	-	Manes.03G152400.v6.1	1.82	-	-1330
	lincRNA_413	1.19	-	Manes.04G058100.v6.1	2.08	+	-769	lincRNA_111	0.74	+	Manes.15G127400.v6.1	1.75	+	-3602
	lincRNA_254	1.14	+	Manes.02G043800.v6.1	1.89	+	-3263	lincRNA_428	1.21	+	Manes.S050600.v6.1	1.67	+	-5243
	lincRNA_151	1.12	-	Manes.13G056000.v6.1	1.81	-	-720	lincRNA_404	1.24	-	Manes.05G070800.v6.1	1.64	+	-7034
	lincRNA_287	1.09	+	Manes.06G164600.v6.1	1.75	-	-7505	lincRNA_69	0.83	-	Manes.17G045800.v6.1	1.64	+	-2674
	lincRNA_131	NA	-	Manes.13G010500.v6.1	1.66	-	-4616	lincRNA_375	0.91	-	Manes.09G000500.v6.1	1.62	-	-1282
	lincRNA_133	0.69	-	Manes.13G076300.v6.1	1.62	-	-4308	lincRNA_102	1.10	-	Manes.15G047200.v6.1	1.60	-	-406
	lincRNA_89	0.86	+	Manes.15G150500.v6.1	1.61	+	-5940	lincRNA_208	1.28	+	Manes.18G076800.v6.1	1.53	+	-3885
	lincRNA_328	1.30	+	Manes.03G107900.v6.1	1.57	+	-1358	lincRNA_391	2.95	+	Manes.05G136900.v6.1	1.51	-	-3798
	lincRNA_50	0.71	+	Manes.16G081300.v6.1	1.56	-	-4661	lincRNA_193	2.83	-	Manes.18G020700.v6.1	1.50	-	-3317
	lincRNA_267	1.34	-	Manes.S077800.v6.1	1.55	-	-1631	lincRNA_156	0.78	-	Manes.11G081100.v6.1	1.49	-	-1292
	lincRNA_331	0.78	-	Manes.03G142900.v6.1	1.49	+	-299	lincRNA_331	0.78	-	Manes.03G143000.v6.1	1.48	-	-1261
	lincRNA_392	0.73	-	Manes.05G029500.v6.1	1.47	-	-6243	lincRNA_271	0.99	-	Manes.06G055600.v6.1	1.48	-	-1650
	lincRNA_196	1.79	-	Manes.18G138500.v6.1	1.44	-	-1708	lincRNA_364	69.93	-	Manes.09G106700.v6.1	1.44	-	-500
	lincRNA_102	1.10	-	Manes.15G047100.v6.1	1.44	-	-1144	lincRNA_367	1.01	+	Manes.09G018800.v6.1	1.42	-	-6078
	lincRNA_225	0.89	+	Manes.07G017600.v6.1	1.43	-	-7187	lincRNA_99	2.32	-	Manes.15G192800.v6.1	1.42	-	-1332
	lincRNA_364	69.93	-	Manes.09G106600.v6.1	1.42	-	-5764	lincRNA_23	0.83	+	Manes.10G047100.v6.1	1.41	+	-1928
	lincRNA_67	0.70	+	Manes.17G006500.v6.1	1.39	+	-123	lincRNA_112	4.94	+	Manes.12G084900.v6.1	1.40	+	-9002
	lincRNA_53	2.71	+	Manes.16G108000.v6.1	1.38	+	-1736	lincRNA_76	1.15	-	Manes.17G103000.v6.1	1.40	-	-2337
	lincRNA_168	0.81	-	Manes.11G064200.v6.1	1.37	-	-9843	lincRNA_256	1.36	-	Manes.02G115900.v6.1	1.39	-	-570
	lincRNA_127	0.73	+	Manes.12G042500.v6.1	1.37	-	-9393	lincRNA_10	0.92	-	Manes.10G084900.v6.1	1.37	-	-177
	lincRNA_103	1.91	-	Manes.15G048000.v6.1	1.36	+	-1322	lincRNA_214	0.94	-	Manes.S044100.v6.1	1.35	-	-359
	lincRNA_332	1.21	-	Manes.03G152300.v6.1	1.36	-	-1101	lincRNA_210	0.77	-	Manes.18G099000.v6.1	1.33	-	-1744
	lincRNA_337	0.98	+	Manes.03G053100.v6.1	1.35	+	-737	lincRNA_441	1.40	-	Manes.08G143800.v6.1	1.33	-	-267
	lincRNA_167	5.15	+	Manes.11G153700.v6.1	1.35	+	-3854	lincRNA_325	0.83	-	Manes.03G088000.v6.1	1.30	+	-1840
	lincRNA_394	2.69	-	Manes.05G173900.v6.1	1.35	-	-1592	lincRNA_324	10.37	+	Manes.03G086900.v6.1	1.29	+	-5371
	lincRNA_257	31.10	-	Manes.02G122600.v6.1	1.34	-	-1812	lincRNA_17	1.53	-	Manes.10G110000.v6.1	1.27	-	-95
	lincRNA_251	1.82	+	Manes.02G220600.v6.1	1.32	+	-5399	lincRNA_376	46.38	+	Manes.09G062500.v6.1	1.26	-	-5614
	lincRNA_130	0.86	-	Manes.12G080600.v6.1	1.31	-	-6546	lincRNA_371	2.05	+	Manes.09G041200.v6.1	1.24	+	-3624
	lincRNA_417	0.78	+	Manes.04G066000.v6.1	1.29	+	-9163	lincRNA_410	1.18	-	Manes.04G057100.v6.1	1.24	-	-431
	lincRNA_112	4.94	+	Manes.12G084800.v6.1	1.28	+	-5819	lincRNA_205	1.41	+	Manes.18G052100.v6.1	1.24	+	-1550
	lincRNA_143	1.89	+	Manes.13G114700.v6.1	1.28	+	-4549	lincRNA_11	0.95	+	Manes.10G089300.v6.1	1.24	-	-4074
	lincRNA_355	0.84	-	Manes.09G081000.v6.1	1.27	-	-2035	lincRNA_165	174.32	-	Manes.11G113100.v6.1	1.22	-	-1503
	lincRNA_439	8.76	+	Manes.08G134600.v6.1	1.27	+	-1417	lincRNA_339	2.33	+	Manes.03G064800.v6.1	1.22	-	-2612
	lincRNA_62	33.93	+	Manes.17G021900.v6.1	1.26	+	-1569	lincRNA_449	4.54	+	Manes.08G008700.v6.1	1.21	-	-1677
	lincRNA_344	0.77	-	Manes.03G070300.v6.1	1.26	-	-9370	lincRNA_313	1.24	+	Manes.01G175900.v6.1	1.21	+	-3431
	lincRNA_236	Inf	-	Manes.07G046800.v6.1	1.25	-	-7180	lincRNA_293	30.58	+	Manes.06G036000.v6.1	1.20	-	-4870
	lincRNA_13	4.80	+	Manes.10G093600.v6.1	1.24	+	-6503	lincRNA_394	2.69	-	Manes.05G174000.v6.1	1.19	-	-1228
	lincRNA_211	11.50	+	Manes.S016700.v6.1	1.24	+	-4244	lincRNA_280	0.89	+	Manes.06G126800.v6.1	1.18	+	-2999
	lincRNA_409	1.28	-	Manes.04G010400.v6.1	1.23	-	-1226	lincRNA_89	0.86	+	Manes.15G150600.v6.1	1.17	+	-7693
	lincRNA_448	1.83	+	Manes.08G053700.v6.1	1.23	+	-1741	lincRNA_314	0.87	+	Manes.01G204100.v6.1	1.16	-	-2967
	lincRNA_30	1.35	+	Manes.10G058600.v6.1	1.22	+	-3881	lincRNA_187	343.89	-	Manes.14G068800.v6.1	1.16	-	-5365
	lincRNA_117	2.26	+	Manes.12G098000.v6.1	1.22	+	-927	lincRNA_231	2.52	+	Manes.07G112500.v6.1	1.15	+	-6567
	lincRNA_88	1.37	-	Manes.15G134700.v6.1	1.21	+	-5355	lincRNA_22	1.13	-	Manes.10G129200.v6.1	1.13	-	-687
	lincRNA_268	1.77	-	Manes.06G036900.v6.1	1.21	-	-5113	lincRNA_178	0.96	+	Manes.14G148900.v6.1	1.12	-	-5683
	lincRNA_449	4.54	+	Manes.08G008600.v6.1	1.21	+	-368	lincRNA_369	1.21	-	Manes.09G173400.v6.1	1.11	-	-4479
	lincRNA_125	1.05	-	Manes.12G140200.v6.1	1.20	-	-4108	lincRNA_322	1.53	-	Manes.03G078500.v6.1	1.11	-	-786
	lincRNA_23	0.83	+	Manes.10G047000.v6.1	1.20	+	-60	lincRNA_34	130.62	-	Manes.10G068000.v6.1	1.09	-	-4811
	lincRNA_193	2.83	-	Manes.18G020600.v6.1	1.20	-	-3441	lincRNA_150	16.09	-	Manes.13G040900.v6.1	1.08	-	-3131
	lincRNA_271	0.99	-	Manes.06G055500.v6.1	1.18	+	-4594	lincRNA_235	0.69	+	Manes.07G136000.v6.1	1.06	-	-476
	lincRNA_221	4.71	+	Manes.07G071100.v6.1	1.16	+	-2389	lincRNA_319	15.68	+	Manes.01G040600.v6.1	1.06	+	-2587
	lincRNA_184	1.65	+	Manes.14G167200.v6.1	1.14	+	-2091	lincRNA_173	1.09	+	Manes.14G140300.v6.1	1.05	+	-1840
	lincRNA_442	1.09	-	Manes.08G146000.v6.1	1.13	-	-7413	lincRNA_288	16.23	+	Manes.06G179100.v6.1	1.05	+	-714
	lincRNA_17	1.53	-	Manes.10G109900.v6.1	1.10	-	-1645	lincRNA_184	1.65	+	Manes.14G167300.v6.1	1.05	+	-9830
	lincRNA_59	0.69	+	Manes.16G043100.v6.1	1.10	+	-9691	lincRNA_345	0.99	-	Manes.03G070400.v6.1	1.05	-	-2416
	lincRNA_40	1.08	-	Manes.16G057100.v6.1	1.09	+	-8731	lincRNA_442	1.09	-	Manes.08G146100.v6.1	1.05	-	-981
	lincRNA_51	1.45	+	Manes.16G088300.v6.1	1.07	-	-3145	lincRNA_447	1.47	-	Manes.08G048300.v6.1	1.05	-	-175
	lincRNA_52	1.06	+	Manes.16G094100.v6.1	1.07	+	-9274	lincRNA_70	130.72	+	Manes.17G057800.v6.1	1.04	+	-5660
	lincRNA_288	16.23	+	Manes.06G179000.v6.1	1.06	+	-1781	lincRNA_122	1.79	-	Manes.12G120800.v6.1	1.03	-	-1503
	lincRNA_118	0.97	+	Manes.12G098500.v6.1	1.06	+	-1218	lincRNA_16	0.85	-	Manes.10G109600.v6.1	1.02	-	-709
	lincRNA_25	15.93	-	Manes.10G047500.v6.1	1.06	+	-5093	lincRNA_330	1.25	+	Manes.03G026400.v6.1	1.00	-	-2203
	lincRNA_372	1.04	-	Manes.09G045800.v6.1	1.05	-	-1026	lincRNA_451	0.70	-	Manes.08G064000.v6.1	1.00	+	-3693
	lincRNA_171	1.01	-	Manes.14G121500.v6.1	1.04	-	-750	lincRNA_232	4.17	+	Manes.07G112700.v6.1	0.99	+	-1451
	lincRNA_306	61.06	+	Manes.01G123000.v6.1	1.04	-	-2262	lincRNA_381	1.17	-	Manes.05G117700.v6.1	0.99	-	-2460
	lincRNA_289	1.08	-	Manes.06G029500.v6.1	1.04	-	-5703	lincRNA_415	143.13	-	Manes.04G062000.v6.1	0.98	-	-1018
	lincRNA_304	2.07	+	Manes.01G107400.v6.1	1.04	+	-3120	lincRNA_90	1.16	+	Manes.15G179600.v6.1	0.98	+	-522
	lincRNA_305	1.81	+	Manes.01G107400.v6.1	1.04	+	-4423	lincRNA_397	9.31	+	Manes.05G187800.v6.1	0.97	-	-459
	lincRNA_280	0.89	+	Manes.06G126700.v6.1	1.04	+	-2741	lincRNA_289	1.08	-	Manes.06G029600.v6.1	0.96	-	-971
	lincRNA_45	44.76	-	Manes.16G060600.v6.1	1.04	+	-2758	lincRNA_387	16.08	-	Manes.05G129500.v6.1	0.96	-	-6642
	lincRNA_20	11.27	+	Manes.10G123400.v6.1	1.02	+	-7856	lincRNA_131	NA	-	Manes.13G010600.v6.1	0.95	+	-3458
	lincRNA_321	0.79	+	Manes.03G076400.v6.1	0.99	+	-1795	lincRNA_138	40.33	+	Manes.13G015500.v6.1	0.94	-	-660
	lincRNA_208	1.28	+	Manes.18G076700.v6.1	0.99	+	-5159	lincRNA_400	55.49	-	Manes.05G203200.v6.1	0.94	-	-9238
Uncorrelate	lincRNA_339	2.33	+	Manes.03G064700.v6.1	0.99	+	-7823	lincRNA_106	0.71	-	Manes.15G074600.v6.1	0.93	+	-501
	lincRNA_431	Inf	+	Manes.08G080700.v6.1	0.98	+	-1138	lincRNA_225	0.89	+	Manes.07G017700.v6.1	0.91	+	-1569
	lincRNA_11	0.95	+	Manes.10G089200.v6.1	0.98	+	-7806	lincRNA_438	0.94	-	Manes.08G033200.v6.1	0.90	+	-252
	lincRNA_255	2.63	+	Manes.02G081900.v6.1	0.97	+	-397	lincRNA_100	1.25	-	Manes.15G035100.v6.1	0.90	-	-1904
	lincRNA_393	#NAME?	-	Manes.05G158200.v6.1	0.96	-	-7020	lincRNA_218	0.75	+	Manes.S004600.v6.1	0.89	+	-9487
	lincRNA_77	11.92	+	Manes.17G112400.v6.1	0.96	+	-1025	lincRNA_13	4.80	+	Manes.10G093700.v6.1	0.89	-	-934
	lincRNA_207	4.53	+	Manes.18G005100.v6.1	0.96	-	-3615	lincRNA_437	1.27	+	Manes.08G128900.v6.1	0.88	+	-63

lincRNA_214	0.94	-	Manes.S044000.v6.1	0.95	+	-5865	lincRNA_170	0.78	-	Manes.11G079700.v6.1	0.88	-	-610
lincRNA_1	2.20	+	Manes.S033900.v6.1	0.95	+	-3638	lincRNA_227	0.76	-	Manes.07G082200.v6.1	0.88	-	-2015
lincRNA_319	15.68	+	Manes.01G040500.v6.1	0.93	+	-379	lincRNA_14	1.30	+	Manes.10G096700.v6.1	0.88	+	-4471
lincRNA_323	1.22	+	Manes.03G000500.v6.1	0.91	+	-1119	lincRNA_360	2.10	-	Manes.09G091700.v6.1	0.87	-	-8775
lincRNA_243	0.80	+	Manes.02G211600.v6.1	0.91	+	-4938	lincRNA_67	0.70	+	Manes.17G006600.v6.1	0.84	-	-428
lincRNA_111	0.74	+	Manes.15G127300.v6.1	0.90	+	-1296	lincRNA_290	1.12	+	Manes.06G031200.v6.1	0.83	+	-8278
lincRNA_357	1.01	-	Manes.09G081500.v6.1	0.90	-	-8118	lincRNA_343	1.26	+	Manes.03G069600.v6.1	0.83	-	-7563
lincRNA_235	0.69	+	Manes.07G135900.v6.1	0.90	-	-3187	lincRNA_439	8.76	+	Manes.08G134700.v6.1	0.83	+	-8741
lincRNA_343	1.26	+	Manes.03G069500.v6.1	0.90	+	-4453	lincRNA_2	2.57	+	Manes.S033800.v6.1	0.82	+	-8094
lincRNA_230	0.86	-	Manes.07G101400.v6.1	0.90	+	-1494	lincRNA_247	1.10	+	Manes.02G215900.v6.1	0.80	+	-5274
lincRNA_360	2.10	-	Manes.09G091600.v6.1	0.89	-	-2801	lincRNA_263	1.04	-	Manes.S003000.v6.1	0.78	-	-395
lincRNA_441	1.40	-	Manes.08G143700.v6.1	0.86	-	-446	lincRNA_262	0.75	-	Manes.S003000.v6.1	0.78	-	-6715
lincRNA_247	1.10	+	Manes.02G215800.v6.1	0.85	+	-8836	lincRNA_326	Inf	+	Manes.03G018300.v6.1	0.78	-	-62
lincRNA_325	0.83	-	Manes.03G087900.v6.1	0.84	-	-3710	lincRNA_301	2.52	+	Manes.01G054800.v6.1	0.78	+	-7881
lincRNA_55	1.27	+	Manes.16G128500.v6.1	0.82	+	-6654	lincRNA_362	0.94	-	Manes.09G011100.v6.1	0.77	+	-8701
lincRNA_113	1.46	+	Manes.12G092000.v6.1	0.82	+	-4953	lincRNA_316	0.94	-	Manes.01G208100.v6.1	0.76	+	-7996
lincRNA_182	1.29	-	Manes.14G163200.v6.1	0.82	-	-1972	lincRNA_87	0.84	-	Manes.S090500.v6.1	0.75	-	-558
lincRNA_5	0.72	+	Manes.10G072400.v6.1	0.82	+	-9201	lincRNA_144	0.79	-	Manes.13G115700.v6.1	0.75	-	-8360
lincRNA_185	259.21	-	Manes.14G168100.v6.1	0.81	-	-4677	lincRNA_125	1.05	-	Manes.12G140300.v6.1	0.75	-	-523
lincRNA_279	15.47	+	Manes.06G125500.v6.1	0.81	+	-923	lincRNA_450	0.78	+	Manes.08G063000.v6.1	0.75	+	-2259
lincRNA_19	1.17	+	Manes.10G114300.v6.1	0.81	+	-500	lincRNA_186	0.75	-	Manes.14G041500.v6.1	0.74	-	-4054
lincRNA_148	0.93	+	Manes.13G144400.v6.1	0.77	-	-5470	lincRNA_124	5.17	+	Manes.12G130600.v6.1	0.73	+	-1404
lincRNA_351	0.68	-	Manes.09G076200.v6.1	0.76	-	-2852	lincRNA_119	2.15	+	Manes.12G102400.v6.1	0.73	-	-8554
lincRNA_398	1.39	-	Manes.05G195400.v6.1	0.76	-	-221	lincRNA_405	1.50	-	Manes.05G085900.v6.1	0.72	-	-7107
lincRNA_99	2.32	-	Manes.15G192700.v6.1	0.75	-	-7383	lincRNA_197	8.93	-	Manes.18G138800.v6.1	0.72	-	-1259
lincRNA_293	30.58	+	Manes.06G035900.v6.1	0.75	-	-1002	lincRNA_65	1.26	-	Manes.17G026200.v6.1	0.71	-	-795
lincRNA_336	1.40	+	Manes.03G212600.v6.1	0.71	+	-2833	lincRNA_258	3.17	+	Manes.02G132200.v6.1	0.70	-	-2393
lincRNA_210	0.77	-	Manes.18G098900.v6.1	0.70	-	-318	lincRNA_351	0.68	-	Manes.09G076300.v6.1	0.70	-	-5681
lincRNA_276	0.75	-	Manes.06G108700.v6.1	0.68	-	-5270	lincRNA_309	0.79	+	Manes.01G143900.v6.1	0.70	+	-6526
lincRNA_105	8.83	-	Manes.15G059900.v6.1	0.68	-	-695	lincRNA_127	0.73	+	Manes.12G042600.v6.1	0.68	+	-1614
lincRNA_106	0.71	-	Manes.15G074500.v6.1	0.68	-	-767	lincRNA_98	0.92	+	Manes.15G189100.v6.1	0.67	+	-5112
lincRNA_369	1.21	-	Manes.09G173300.v6.1	0.67	-	-9804	lincRNA_55	1.27	+	Manes.16G128600.v6.1	0.64	-	-5127
lincRNA_199	1.31	+	Manes.18G145600.v6.1	0.65	+	-1471	lincRNA_421	0.94	+	Manes.04G163200.v6.1	0.63	+	-1313
lincRNA_362	0.94	-	Manes.09G011000.v6.1	0.61	-	-6862	lincRNA_241	0.68	-	Manes.02G190000.v6.1	0.62	+	-4316
lincRNA_361	0.69	-	Manes.09G011000.v6.1	0.61	-	-1210	lincRNA_199	1.31	+	Manes.18G145700.v6.1	0.61	+	-5112
lincRNA_160	0.69	+	Manes.11G017500.v6.1	0.61	+	-110	lincRNA_399	0.76	+	Manes.05G200500.v6.1	0.61	+	-8289
lincRNA_178	0.96	+	Manes.14G148800.v6.1	0.58	+	-2594	lincRNA_276	0.75	-	Manes.06G108800.v6.1	0.61	+	-999
lincRNA_218	0.75	+	Manes.S004500.v6.1	0.58	+	-848	lincRNA_171	1.01	-	Manes.14G121600.v6.1	0.61	-	-2213
lincRNA_367	1.01	+	Manes.09G018700.v6.1	0.52	+	-3739	lincRNA_239	1.05	+	Manes.07G064600.v6.1	0.60	+	-9713
lincRNA_205	1.41	+	Manes.18G052000.v6.1	0.50	+	-593	lincRNA_202	1.07	-	Manes.18G043000.v6.1	0.60	-	-165
lincRNA_275	0.73	+	Manes.06G013900.v6.1	0.46	+	-556	lincRNA_52	1.06	+	Manes.16G094200.v6.1	0.57	+	-88
lincRNA_428	1.21	+	Manes.S050500.v6.1	0.41	+	-3989	lincRNA_151	1.12	-	Manes.13G056100.v6.1	0.56	-	-1995
lincRNA_39	0.81	+	Manes.16G011800.v6.1	0.40	-	-511	lincRNA_28	1.45	-	Manes.10G058000.v6.1	0.56	-	-2950
lincRNA_15	0.83	+	Manes.10G105300.v6.1	0.38	+	-438	lincRNA_373	1.16	-	Manes.09G049000.v6.1	0.54	-	-133
lincRNA_438	0.94	-	Manes.08G033100.v6.1	0.36	-	-1030	lincRNA_51	1.45	+	Manes.16G088400.v6.1	0.53	+	-3483
lincRNA_256	1.36	-	Manes.02G115800.v6.1	0.33	+	-3964	lincRNA_295	0.81	-	Manes.06G036200.v6.1	0.50	-	-2344
lincRNA_58	1.09	-	Manes.16G136200.v6.1	0.28	+	-5209	lincRNA_294	0.76	-	Manes.06G036200.v6.1	0.50	-	-3864
lincRNA_241	0.68	-	Manes.02G189900.v6.1	0.23	-	-6743	lincRNA_333	1.24	+	Manes.03G032300.v6.1	0.46	+	-9200
lincRNA_384	0.25	-	Manes.05G018600.v6.1	#######	+	-2761	lincRNA_47	1.22	-	Manes.16G065600.v6.1	0.42	-	-4578
lincRNA_342	0.25	-	Manes.03G069400.v6.1	#######	+	-565	lincRNA_50	0.71	+	Manes.16G081400.v6.1	0.41	-	-4151
lincRNA_420	0.39	-	Manes.04G152600.v6.1	NA	+	-1447	lincRNA_130	0.86	-	Manes.12G080700.v6.1	0.35	-	-7066
lincRNA_285	0.34	+	Manes.06G146500.v6.1	1.35	+	-2415	lincRNA_393	#NAME?	-	Manes.05G158300.v6.1	0.34	+	-3929
lincRNA_78	0.60	+	Manes.17G120300.v6.1	1.34	+	-256	lincRNA_374	0.79	-	Manes.09G055100.v6.1	0.26	+	-768
lincRNA_146	0.67	-	Manes.13G129900.v6.1	1.31	-	-1121	lincRNA_275	0.73	+	Manes.06G014000.v6.1	0.21	+	-1892
lincRNA_166	0.33	+	Manes.11G137100.v6.1	1.31	-	-2797	lincRNA_222	0.66	-	Manes.07G071800.v6.1	#NAME?	+	-5869
lincRNA_253	0.46	-	Manes.02G226100.v6.1	1.28	+	-8606	lincRNA_278	0.58	-	Manes.06G120400.v6.1	#NAME?	+	-774
lincRNA_7	0.46	+	Manes.10G075000.v6.1	1.21	+	-7266	lincRNA_190	0.55	+	Manes.14G076600.v6.1	#NAME?	+	-5836
lincRNA_72	0.37	+	Manes.17G068300.v6.1	1.20	-	-1749	lincRNA_48	0.26	+	Manes.16G067700.v6.1	#NAME?	-	-1873
lincRNA_49	0.39	-	Manes.16G073700.v6.1	1.19	-	-4336	lincRNA_363	0.16	-	Manes.09G098200.v6.1	#NAME?	+	-4442
lincRNA_282	0.22	-	Manes.06G000500.v6.1	1.18	-	-6446	lincRNA_422	0.44	+	Manes.04G030600.v6.1	NA	+	-6702
lincRNA_312	0.10	-	Manes.01G167900.v6.1	1.18	+	-522	lincRNA_395	0.42	-	Manes.05G183500.v6.1	NA	-	-2155
lincRNA_436	0.53	+	Manes.08G113000.v6.1	1.11	+	-9861	lincRNA_229	0.40	+	Manes.07G100800.v6.1	NA	-	-1734
lincRNA_444	0.67	+	Manes.08G166500.v6.1	1.09	+	-306	lincRNA_282	0.22	-	Manes.06G000600.v6.1	NA	-	-4620
lincRNA_446	0.57	-	Manes.08G172300.v6.1	1.06	-	-3448	lincRNA_264	0.66	-	Manes.S002500.v6.1	1.38	+	-3503
lincRNA_443	0.53	-	Manes.08G155700.v6.1	0.98	-	-2532	lincRNA_402	0.44	+	Manes.05G057600.v6.1	1.37	+	-855
lincRNA_368	0.62	+	Manes.09G170400.v6.1	0.95	+	-200	lincRNA_283	0.27	+	Manes.06G136700.v6.1	1.36	-	-280
lincRNA_57	0.19	-	Manes.16G132400.v6.1	0.95	-	-1476	lincRNA_154	0.51	+	Manes.13G009700.v6.1	1.36	-	-4326
lincRNA_191	0.66	+	Manes.14G085100.v6.1	0.95	+	-2995	lincRNA_281	0.30	+	Manes.06G130200.v6.1	1.29	+	-2723
lincRNA_406	0.40	-	Manes.05G099000.v6.1	0.93	-	-870	lincRNA_44	0.52	-	Manes.16G019200.v6.1	1.25	-	-527
lincRNA_145	0.33	-	Manes.13G129100.v6.1	0.93	-	-6221	lincRNA_109	0.43	+	Manes.15G083300.v6.1	1.22	-	-9632
lincRNA_192	0.67	+	Manes.18G000300.v6.1	0.92	+	-7705	lincRNA_414	0.43	-	Manes.04G012900.v6.1	1.18	+	-6964
lincRNA_356	0.59	-	Manes.09G081500.v6.1	0.90	-	-2156	lincRNA_54	0.34	+	Manes.16G127300.v6.1	1.11	-	-652
lincRNA_281	0.30	+	Manes.06G130100.v6.1	0.89	-	-491	lincRNA_310	0.47	-	Manes.01G145600.v6.1	1.09	+	-5862
lincRNA_240	0.65	-	Manes.02G171200.v6.1	0.88	-	-9193	lincRNA_253	0.46	-	Manes.02G226200.v6.1	1.08	-	-7182
lincRNA_188	0.66	+	Manes.14G072300.v6.1	0.88	-	-1065	lincRNA_166	0.33	+	Manes.11G137200.v6.1	1.07	-	-5462
lincRNA_259	0.39	-	Manes.S033400.v6.1	0.85	+	-1369	lincRNA_192	0.67	+	Manes.18G000400.v6.1	1.02	+	-5192
lincRNA_75	0.14	-	Manes.17G094500.v6.1	0.85	-	-9574	lincRNA_163	0.63	-	Manes.11G105500.v6.1	1.02	-	-6044
lincRNA_41	0.61	+	Manes.16G057200.v6.1	0.80	+	-5911	lincRNA_179	0.63	-	Manes.14G151600.v6.1	0.98	+	-630
lincRNA_338	0.63	-	Manes.03G062100.v6.1	0.77	+	-9536	lincRNA_312	0.10	-	Manes.01G168000.v6.1	0.96	+	-1635
lincRNA_274	0.34	-	Manes.06G092300.v6.1	0.72	+	-1262	lincRNA_7	0.46	+	Manes.10G075100.v6.1	0.94	-	-1425
lincRNA_142	0.31	+	Manes.13G105300.v6.1	0.71	+	-7247	lincRNA_436	0.53	+	Manes.08G113100.v6.1	0.91	+	-2823
lincRNA_190	0.55	+	Manes.14G076500.v6.1	0.71	+	-1569	lincRNA_57	0.19	-	Manes.16G132500.v6.1	0.89	+	-158
lincRNA_164	0.25	+	Manes.11G108700.v6.1	0.68	+	-964	lincRNA_164	0.25	+	Manes.11G108800.v6.1	0.83	+	-2411
lincRNA_137	0.62	-	Manes.13G083500.v6.1	0.68	+	-5801	lincRNA_49	0.39	-	Manes.16G073800.v6.1	0.80	+	-2161
lincRNA_418	0.45	-	Manes.04G088700.v6.1	0.68	+	-2863	lincRNA_443	0.53	-	Manes.08G155800.v6.1	0.80	+	-1622
lincRNA_96	0.45	+	Manes.15G188500.v6.1	0.67	+	-8355	lincRNA_274	0.34	-	Manes.06G092400.v6.1	0.80	-	-401
							lincRNA_384	0.25	-	Manes.05G018700.v6.1	0.77	+	-6189
							lincRNA_145	0.33	-	Manes.13G129200.v6.1	0.76	+	-6569

lincRNA_104	0.33	+	Manes.15G000200.v6.1	0.75	+	-2988
lincRNA_31	0.23	-	Manes.10G060200.v6.1	0.75	-	-3092
lincRNA_383	0.27	+	Manes.05G118700.v6.1	0.74	+	-8803
lincRNA_137	0.62	-	Manes.13G083600.v6.1	0.69	-	-69

Supplemental Data S6. Expression information of lincRNAs and nearby protein-coding genes under drought stress

	lincRNA ID	Drought/CK	Stranc	Left gene ID	peg/ck	Left g	Left gene	lincRNA ID	PEG Fold(	lnc_st	Right gene ID	Drought/(	Right g	Right gene
	lincRNA_107	Inf	+	Manes.15G075900.v6.1	Inf	+	-1594	lincRNA_403	Inf	+	Manes.05G064000.v6.1	Inf	-	-663
	lincRNA_335	3.00	-	Manes.03G195000.v6.1	Inf	-	-736	lincRNA_114	10.21	-	Manes.12G092700.v6.1	Inf	+	-2808
	lincRNA_101	511.10	+	Manes.15G003200.v6.1	3199.81	+	-478	lincRNA_376	11.33	+	Manes.09G062500.v6.1	9.91	-	-5614
	lincRNA_403	Inf	+	Manes.05G063900.v6.1	284.81	-	-501	lincRNA_31	4.98	-	Manes.10G060200.v6.1	5.39	-	-3092
	lincRNA_365	1.68	-	Manes.09G148600.v6.1	31.31	+	-7529	lincRNA_44	3.61	-	Manes.16G019200.v6.1	4.14	-	-527
	lincRNA_376	11.33	+	Manes.09G062400.v6.1	12.06	+	-3405	lincRNA_13	3.22	+	Manes.10G093700.v6.1	3.24	-	-934
	lincRNA_397	4.93	+	Manes.05G187700.v6.1	7.61	+	-8828	lincRNA_277	3.19	+	Manes.06G118700.v6.1	3.14	-	-2449
	lincRNA_382	83.42	+	Manes.05G118400.v6.1	6.53	+	-1156	lincRNA_449	1.62	+	Manes.08G008700.v6.1	2.66	-	-1677
	lincRNA_131	Inf	-	Manes.13G010500.v6.1	4.90	-	-4616	lincRNA_373	3.16	-	Manes.09G049000.v6.1	2.64	-	-133
	lincRNA_245	1.89	-	Manes.02G213200.v6.1	3.75	-	-5968	lincRNA_231	2.18	+	Manes.07G112500.v6.1	2.30	+	-6567
	lincRNA_110	2.44	+	Manes.15G103800.v6.1	2.38	+	-116	lincRNA_110	2.44	+	Manes.15G103900.v6.1	2.27	+	-2461
	lincRNA_117	1.73	+	Manes.12G098000.v6.1	1.80	+	-927	lincRNA_124	2.38	+	Manes.12G130600.v6.1	2.19	+	-1404
	lincRNA_289	1.67	-	Manes.06G029500.v6.1	1.69	-	-5703	lincRNA_410	2.69	-	Manes.04G057100.v6.1	2.11	-	-431
	lincRNA_174	11.68	+	Manes.14G142200.v6.1	1.69	+	-278	lincRNA_107	Inf	+	Manes.15G076000.v6.1	1.90	+	-4202
	lincRNA_258	1.52	+	Manes.02G132100.v6.1	1.66	-	-1914	lincRNA_187	9.07	-	Manes.14G068800.v6.1	1.85	-	-5365
	lincRNA_268	1.64	-	Manes.06G036900.v6.1	1.65	-	-5113	lincRNA_32	2.91	-	Manes.10G062400.v6.1	1.77	-	-899
	lincRNA_361	1.76	-	Manes.09G011000.v6.1	1.51	-	-1210	lincRNA_131	Inf	-	Manes.13G010600.v6.1	1.75	+	-3458
	lincRNA_362	1.60	-	Manes.09G011000.v6.1	1.51	-	-6862	lincRNA_165	3.43	-	Manes.11G113100.v6.1	1.69	-	-1503
	lincRNA_276	0.53	-	Manes.06G108700.v6.1	0.65	-	-5270	lincRNA_236	Inf	-	Manes.07G046900.v6.1	1.66	-	-1700
	lincRNA_321	0.26	+	Manes.03G076400.v6.1	0.65	+	-1795	lincRNA_289	1.67	-	Manes.06G029600.v6.1	1.66	-	-971
	lincRNA_230	0.30	-	Manes.07G101400.v6.1	0.64	+	-1494	lincRNA_445	1.82	+	Manes.08G166600.v6.1	1.64	+	-3667
	lincRNA_435	0.48	+	Manes.08G002500.v6.1	0.60	-	-1309	lincRNA_33	2.02	-	Manes.10G011900.v6.1	1.60	+	-2769
	lincRNA_5	0.25	+	Manes.10G072400.v6.1	0.57	+	-9201	lincRNA_154	1.83	+	Manes.13G009700.v6.1	1.57	-	-4326
	lincRNA_286	0.33	-	Manes.06G161300.v6.1	0.56	+	-78	lincRNA_254	0.27	+	Manes.02G043900.v6.1	0.64	+	-5260
Concordar	lincRNA_59	0.66	+	Manes.16G043100.v6.1	0.55	+	-9691	lincRNA_294	0.60	-	Manes.06G036200.v6.1	0.64	-	-3864
	lincRNA_125	0.45	-	Manes.12G140200.v6.1	0.53	-	-4108	lincRNA_295	0.54	-	Manes.06G036200.v6.1	0.64	-	-2344
	lincRNA_54	0.54	+	Manes.16G127200.v6.1	0.50	+	-1998	lincRNA_75	0.55	-	Manes.17G094600.v6.1	0.62	+	-363
	lincRNA_204	0.52	+	Manes.18G048200.v6.1	0.49	+	-1749	lincRNA_311	0.54	-	Manes.01G166800.v6.1	0.60	-	-2580
	lincRNA_6	0.52	-	Manes.10G074200.v6.1	0.46	-	-4107	lincRNA_10	0.66	-	Manes.10G084900.v6.1	0.56	-	-177
	lincRNA_444	0.51	+	Manes.08G166500.v6.1	0.45	+	-306	lincRNA_401	0.43	-	Manes.05G048800.v6.1	0.56	+	-806
	lincRNA_232	0.55	+	Manes.07G112600.v6.1	0.42	+	-841	lincRNA_272	0.54	-	Manes.06G057300.v6.1	0.55	-	-666
	lincRNA_193	0.36	-	Manes.18G020600.v6.1	0.39	-	-3441	lincRNA_22	0.67	-	Manes.10G129200.v6.1	0.54	-	-687
	lincRNA_311	0.54	-	Manes.01G166700.v6.1	0.39	-	-1575	lincRNA_55	0.34	+	Manes.16G128600.v6.1	0.53	-	-5127
	lincRNA_285	0.41	+	Manes.06G146500.v6.1	0.36	+	-2415	lincRNA_7	0.50	+	Manes.10G075100.v6.1	0.47	-	-1425
	lincRNA_241	0.51	-	Manes.02G189900.v6.1	0.22	-	-6743	lincRNA_309	0.61	+	Manes.01G143900.v6.1	0.46	+	-6526
								lincRNA_381	0.43	-	Manes.05G117700.v6.1	0.44	-	-2460
								lincRNA_439	0.40	+	Manes.08G134700.v6.1	0.42	+	-8741
								lincRNA_125	0.45	-	Manes.12G140300.v6.1	0.42	-	-523
								lincRNA_363	0.35	-	Manes.09G098200.v6.1	0.40	+	-4442
								lincRNA_141	0.53	-	Manes.13G089600.v6.1	0.39	-	-13
								lincRNA_123	0.57	+	Manes.12G123100.v6.1	0.38	+	-25
								lincRNA_206	0.54	+	Manes.18G054800.v6.1	0.37	-	-3028
								lincRNA_54	0.54	+	Manes.16G127300.v6.1	0.34	-	-652
								lincRNA_179	0.52	-	Manes.14G151600.v6.1	0.26	+	-630
								lincRNA_378	0.49	-	Manes.S087100.v6.1	0.24	-	-490
								lincRNA_234	0.36	+	Manes.07G130700.v6.1	0.07	-	-433
	lincRNA_187	9.07	-	Manes.14G068700.v6.1	0.60	+	-3041	lincRNA_362	1.60	-	Manes.09G011100.v6.1	0.65	+	-8701
	lincRNA_40	1.54	-	Manes.16G057100.v6.1	0.57	+	-8731	lincRNA_326	Inf	+	Manes.03G018300.v6.1	0.60	-	-62
	lincRNA_256	3.12	-	Manes.02G115800.v6.1	0.56	+	-3964	lincRNA_301	6.37	+	Manes.01G054800.v6.1	0.56	+	-7881
	lincRNA_56	4.84	-	Manes.16G130100.v6.1	0.54	+	-3307	lincRNA_334	9.02	+	Manes.03G032300.v6.1	0.36	+	-5201
	lincRNA_417	2.15	+	Manes.04G066000.v6.1	0.51	+	-9163	lincRNA_333	1.50	+	Manes.03G032300.v6.1	0.36	+	-9200
	lincRNA_277	3.19	+	Manes.06G118600.v6.1	0.50	+	-1865	lincRNA_136	8.16	+	Manes.13G082700.v6.1	0.22	-	-2339
	lincRNA_33	2.02	-	Manes.10G011800.v6.1	0.47	+	-5425	lincRNA_279	3.08	+	Manes.06G125600.v6.1	0.16	+	-1476
	lincRNA_293	6.94	+	Manes.06G035900.v6.1	0.46	-	-1002	lincRNA_162	0.59	+	Manes.11G101500.v6.1	Inf	-	-3117
	lincRNA_326	Inf	+	Manes.03G018200.v6.1	0.14	-	-666	lincRNA_181	0.51	-	Manes.14G161600.v6.1	10.42	-	-2628
	lincRNA_411	Inf	+	Manes.04G057600.v6.1	0.09	+	-1794	lincRNA_307	0.45	+	Manes.01G130000.v6.1	2.26	-	-1307
	lincRNA_68	0.59	-	Manes.17G045100.v6.1	Inf	+	-2236	lincRNA_69	0.50	-	Manes.17G045800.v6.1	2.18	+	-2674
	lincRNA_141	0.53	-	Manes.13G089500.v6.1	Inf	+	-5727	lincRNA_204	0.52	+	Manes.18G048300.v6.1	1.60	-	-974
	lincRNA_179	0.52	-	Manes.14G151500.v6.1	Inf	+	-9117	lincRNA_241	0.51	-	Manes.02G190000.v6.1	1.57	+	-4316
	lincRNA_120	0.17	+	Manes.12G102500.v6.1	3.91	-	-9283	lincRNA_392	0.24	-	Manes.05G029600.v6.1	1.52	-	-3058
	lincRNA_392	0.24	-	Manes.05G029500.v6.1	3.36	-	-6243							
	lincRNA_307	0.45	+	Manes.01G129900.v6.1	3.21	+	-6196							
	lincRNA_366	0.35	-	Manes.09G157600.v6.1	2.87	+	-2645							
	lincRNA_259	0.35	-	Manes.S033400.v6.1	1.86	+	-1369							
	lincRNA_414	0.45	-	Manes.04G012800.v6.1	1.52	-	-4102							
	lincRNA_75	0.55	-	Manes.17G094500.v6.1	1.51	-	-9574							
	lincRNA_133	0.47	-	Manes.13G076300.v6.1	1.50	-	-4308							
	lincRNA_138	#NAME?	+	Manes.13G015400.v6.1	0.64	+	-838	lincRNA_245	1.89	-	Manes.02G213300.v6.1	NA	+	-527
	lincRNA_373	3.16	-	Manes.09G048900.v6.1	NA	+	-6677	lincRNA_188	1.58	+	Manes.14G072400.v6.1	NA	-	-137
	lincRNA_370	2.98	+	Manes.09G001700.v6.1	NA	-	-1066	lincRNA_397	4.93	+	Manes.05G187800.v6.1	1.46	-	-459
	lincRNA_433	1.56	-	Manes.08G085400.v6.1	NA	+	-4918	lincRNA_256	3.12	-	Manes.02G115900.v6.1	1.44	-	-570
	lincRNA_333	1.50	+	Manes.03G032200.v6.1	NA	-	-6101	lincRNA_34	3.49	-	Manes.10G068000.v6.1	1.37	-	-4811
	lincRNA_237	2.82	-	Manes.07G049900.v6.1	1.48	+	-1420	lincRNA_258	1.52	+	Manes.02G132200.v6.1	1.34	-	-2393
	lincRNA_21	3.72	+	Manes.10G127100.v6.1	1.47	-	-8831	lincRNA_365	1.68	-	Manes.09G148700.v6.1	1.33	-	-113
	lincRNA_221	1.98	+	Manes.07G071100.v6.1	1.46	+	-2389	lincRNA_322	1.63	-	Manes.03G078500.v6.1	1.31	-	-786
	lincRNA_127	2.16	+	Manes.12G042500.v6.1	1.39	-	-9393	lincRNA_45	5.60	-	Manes.16G060700.v6.1	1.22	-	-1419
	lincRNA_62	3.88	+	Manes.17G021900.v6.1	1.33	+	-1569	lincRNA_293	6.94	+	Manes.06G036000.v6.1	1.19	-	-4870
	lincRNA_45	5.60	-	Manes.16G060600.v6.1	1.25	+	-2758	lincRNA_70	6.67	+	Manes.17G057800.v6.1	1.17	+	-5660
	lincRNA_279	3.08	+	Manes.06G125500.v6.1	1.25	+	-923	lincRNA_400	2.57	-	Manes.05G203200.v6.1	1.13	-	-9238
	lincRNA_338	2.18	-	Manes.03G062100.v6.1	1.24	+	-9536	lincRNA_387	5.60	-	Manes.05G129500.v6.1	1.11	-	-6642
	lincRNA_449	1.62	+	Manes.08G008600.v6.1	1.22	+	-368	lincRNA_237	2.82	-	Manes.07G050000.v6.1	1.11	-	-726
	lincRNA_288	3.93	+	Manes.06G179000.v6.1	1.20	+	-1781	lincRNA_119	1.74	+	Manes.12G102400.v6.1	1.07	-	-8554
	lincRNA_236	Inf	-	Manes.07G046800.v6.1	1.11	-	-7180	lincRNA_319	2.57	+	Manes.01G040600.v6.1	1.06	+	-2587
	lincRNA_13	3.22	+	Manes.10G093600.v6.1	1.04	+	-6503	lincRNA_138	#NAME?	+	Manes.13G015500.v6.1	1.06	-	-660
	lincRNA_319	2.57	+	Manes.01G040500.v6.1	1.04	+	-379	lincRNA_37	1.78	+	Manes.S087700.v6.1	1.05	-	-2274
	lincRNA_211	3.60	+	Manes.S016700.v6.1	0.88	+	-4244	lincRNA_127	2.16	+	Manes.12G042600.v6.1	1.04	+	-1614

lincRNA_324	7.82	+	Manes.03G086800.v6.1	0.87	+	-1813	lincRNA_415	4.43	-	Manes.04G062000.v6.1	1.03	-	-1018
lincRNA_185	10.35	-	Manes.14G168100.v6.1	0.86	-	-4677	lincRNA_391	2.11	+	Manes.05G136900.v6.1	1.03	-	-3798
lincRNA_356	4.69	-	Manes.09G081500.v6.1	0.85	-	-2156	lincRNA_288	3.93	+	Manes.06G179100.v6.1	1.00	+	-714
lincRNA_357	3.38	-	Manes.09G081500.v6.1	0.85	-	-8118	lincRNA_56	4.84	-	Manes.16G130200.v6.1	0.93	-	-516
lincRNA_24	2.29	+	Manes.10G047400.v6.1	0.79	-	-8247	lincRNA_197	3.94	-	Manes.18G138800.v6.1	0.86	-	-1259
lincRNA_364	1.90	-	Manes.09G106600.v6.1	0.76	-	-5764	lincRNA_324	7.82	+	Manes.03G086900.v6.1	0.83	+	-5371
lincRNA_188	1.58	+	Manes.14G072300.v6.1	0.75	-	-1065	lincRNA_169	3.39	+	Manes.11G072300.v6.1	0.80	+	-9451
lincRNA_284	1.50	+	Manes.06G141400.v6.1	0.74	+	-2942	lincRNA_431	NA	+	Manes.08G080800.v6.1	0.76	-	-6232
lincRNA_431	NA	+	Manes.08G080700.v6.1	0.71	+	-1138	lincRNA_364	1.90	-	Manes.09G106700.v6.1	0.75	-	-500
lincRNA_136	8.16	+	Manes.13G082600.v6.1	0.68	+	-1576	lincRNA_150	3.92	-	Manes.13G040900.v6.1	0.73	-	-3131
lincRNA_70	6.67	+	Manes.17G057700.v6.1	0.67	+	-4016	lincRNA_178	1.47	+	Manes.14G148900.v6.1	2.13	-	-5683
lincRNA_25	1.48	-	Manes.10G047500.v6.1	1.04	+	-5093	lincRNA_47	1.47	-	Manes.16G065600.v6.1	0.76	-	-4578
lincRNA_178	1.47	+	Manes.14G148800.v6.1	10.08	+	-2594	lincRNA_442	1.47	-	Manes.08G146100.v6.1	0.77	-	-981
lincRNA_442	1.47	-	Manes.08G146000.v6.1	0.37	-	-7413	lincRNA_161	1.44	-	Manes.11G101400.v6.1	1.39	-	-7393
lincRNA_139	1.46	+	Manes.13G087300.v6.1	1.81	+	-1493	lincRNA_263	1.43	-	Manes.S003000.v6.1	1.14	-	-395
lincRNA_58	1.46	-	Manes.16G136200.v6.1	1.53	+	-5209	lincRNA_404	1.39	-	Manes.05G070800.v6.1	3.89	+	-7034
lincRNA_196	1.45	-	Manes.18G138500.v6.1	1.13	-	-1708	lincRNA_253	1.38	-	Manes.02G226200.v6.1	1.07	-	-7182
lincRNA_251	1.45	+	Manes.02G220600.v6.1	4.77	+	-5399	lincRNA_173	1.38	+	Manes.14G140300.v6.1	1.35	+	-1840
lincRNA_404	1.39	-	Manes.05G070700.v6.1	3.81	-	-445	lincRNA_151	1.37	-	Manes.13G056100.v6.1	0.90	-	-1995
lincRNA_253	1.38	-	Manes.02G226100.v6.1	1.18	+	-8606	lincRNA_20	1.36	+	Manes.10G123500.v6.1	0.79	+	-678
lincRNA_151	1.37	-	Manes.13G056000.v6.1	0.47	-	-720	lincRNA_438	1.36	-	Manes.08G033200.v6.1	1.13	+	-252
lincRNA_20	1.36	+	Manes.10G123400.v6.1	0.57	+	-7856	lincRNA_318	1.35	-	Manes.01G250600.v6.1	1.08	-	-1329
lincRNA_438	1.36	-	Manes.08G033100.v6.1	0.41	-	-1030	lincRNA_189	1.34	-	Manes.14G075300.v6.1	2.05	-	-5165
lincRNA_318	1.35	-	Manes.01G250500.v6.1	0.70	-	-29	lincRNA_306	1.33	+	Manes.01G123100.v6.1	0.85	+	-2772
lincRNA_189	1.34	-	Manes.14G075200.v6.1	0.49	-	-3387	lincRNA_164	1.33	+	Manes.11G108800.v6.1	1.32	+	-2411
lincRNA_148	1.33	+	Manes.13G144400.v6.1	4.70	-	-5470	lincRNA_345	1.32	-	Manes.03G070400.v6.1	1.54	-	-2416
lincRNA_306	1.33	+	Manes.01G123000.v6.1	0.78	-	-2262	lincRNA_11	1.31	+	Manes.10G089300.v6.1	1.15	-	-4074
lincRNA_164	1.33	+	Manes.11G108700.v6.1	1.38	+	-964	lincRNA_14	1.30	+	Manes.10G096700.v6.1	1.22	+	-4471
lincRNA_140	1.31	-	Manes.13G088300.v6.1	1.46	-	-57	lincRNA_53	1.30	+	Manes.16G108100.v6.1	0.65	+	-2508
lincRNA_11	1.31	+	Manes.10G089200.v6.1	1.13	+	-7806	lincRNA_355	1.30	-	Manes.09G081100.v6.1	2.23	+	-3732
lincRNA_53	1.30	+	Manes.16G108000.v6.1	1.57	+	-1736	lincRNA_146	1.29	-	Manes.13G130000.v6.1	1.51	-	-209
lincRNA_355	1.30	-	Manes.09G081000.v6.1	1.18	-	-2035	lincRNA_441	1.29	-	Manes.08G143800.v6.1	1.18	-	-267
lincRNA_183	1.30	-	Manes.14G166200.v6.1	NA	+	-210	lincRNA_360	1.29	-	Manes.09G091700.v6.1	1.54	-	-8775
lincRNA_146	1.29	-	Manes.13G129900.v6.1	2.14	-	-1121	lincRNA_375	1.28	-	Manes.09G000500.v6.1	1.24	-	-1282
lincRNA_441	1.29	-	Manes.08G143700.v6.1	0.62	-	-446	lincRNA_17	1.26	-	Manes.10G110000.v6.1	1.00	-	-95
lincRNA_360	1.29	-	Manes.09G091600.v6.1	1.62	-	-2801	lincRNA_246	1.25	-	Manes.02G213400.v6.1	3.40	+	-778
lincRNA_3	1.27	+	Manes.S034000.v6.1	1.06	+	-9777	lincRNA_166	1.25	+	Manes.11G137200.v6.1	0.50	-	-5462
lincRNA_303	1.27	+	Manes.01G059700.v6.1	0.47	+	-3240	lincRNA_65	1.24	-	Manes.17G026200.v6.1	1.17	-	-795
lincRNA_17	1.26	-	Manes.10G109900.v6.1	0.80	-	-1645	lincRNA_451	1.24	-	Manes.08G064000.v6.1	2.67	+	-3693
lincRNA_246	1.25	-	Manes.02G213300.v6.1	NA	+	-8098	lincRNA_339	1.23	+	Manes.03G064800.v6.1	1.78	-	-2612
lincRNA_337	1.25	+	Manes.03G053100.v6.1	0.37	+	-737	lincRNA_247	1.22	+	Manes.02G215900.v6.1	0.86	+	-5274
lincRNA_166	1.25	+	Manes.11G137100.v6.1	0.38	-	-2797	lincRNA_28	1.21	-	Manes.10G058000.v6.1	1.71	-	-2950
lincRNA_339	1.23	+	Manes.03G064700.v6.1	1.25	+	-7823	lincRNA_48	1.21	+	Manes.16G067700.v6.1	3.01	-	-1873
lincRNA_18	1.23	-	Manes.10G110100.v6.1	2.67	-	-5307	lincRNA_278	1.21	-	Manes.06G120400.v6.1	0.40	+	-774
lincRNA_247	1.22	+	Manes.02G215800.v6.1	0.94	+	-8836	lincRNA_367	1.20	+	Manes.09G018800.v6.1	0.50	-	-6078
lincRNA_118	1.21	+	Manes.12G098500.v6.1	1.30	+	-1218	lincRNA_66	1.20	-	Manes.17G033400.v6.1	2.70	-	-1736
lincRNA_278	1.21	-	Manes.06G120300.v6.1	0.67	-	-3130	lincRNA_271	1.19	-	Manes.06G055600.v6.1	1.19	-	-1650
lincRNA_15	1.20	+	Manes.10G105300.v6.1	1.25	+	-438	lincRNA_436	1.18	+	Manes.08G113100.v6.1	1.61	+	-2823
lincRNA_367	1.20	+	Manes.09G018700.v6.1	1.10	+	-3739	lincRNA_343	1.18	+	Manes.03G069600.v6.1	1.13	-	-7563
lincRNA_66	1.20	-	Manes.17G033300.v6.1	0.88	+	-6932	lincRNA_330	1.18	+	Manes.03G026400.v6.1	0.68	-	-2203
lincRNA_348	1.19	-	Manes.09G073200.v6.1	Inf	+	-5661	lincRNA_317	1.17	-	Manes.01G209900.v6.1	0.87	+	-3847
lincRNA_271	1.19	-	Manes.06G055500.v6.1	1.16	+	-4594	lincRNA_76	1.16	-	Manes.17G103000.v6.1	0.30	-	-2337
lincRNA_436	1.18	+	Manes.08G113000.v6.1	2.74	+	-9861	lincRNA_437	1.16	+	Manes.08G128900.v6.1	1.11	+	-63
lincRNA_19	1.18	+	Manes.10G114300.v6.1	1.14	+	-500	lincRNA_208	1.16	+	Manes.18G076800.v6.1	1.53	+	-3885
lincRNA_343	1.18	+	Manes.03G069500.v6.1	1.25	+	-4453	lincRNA_274	1.15	-	Manes.06G092400.v6.1	0.34	-	-401
lincRNA_330	1.18	+	Manes.03G026300.v6.1	#######	-	-613	lincRNA_354	1.15	+	Manes.09G079500.v6.1	0.94	-	-2464
lincRNA_113	1.18	+	Manes.12G092000.v6.1	0.57	+	-4953	lincRNA_105	1.13	-	Manes.15G060000.v6.1	13.16	+	-7884
lincRNA_317	1.17	-	Manes.01G209800.v6.1	1.28	-	-83	lincRNA_172	1.12	-	Manes.14G134600.v6.1	0.46	-	-6157
lincRNA_126	1.17	-	Manes.12G146900.v6.1	NA	+	-1741	lincRNA_115	1.12	-	Manes.12G094100.v6.1	2.58	-	-4288
lincRNA_76	1.16	-	Manes.17G102900.v6.1	1.20	+	-1016	lincRNA_257	1.11	-	Manes.02G122700.v6.1	0.77	-	-208
lincRNA_208	1.16	+	Manes.18G076700.v6.1	1.18	+	-5159	lincRNA_184	1.11	+	Manes.14G167300.v6.1	0.66	+	-9830
lincRNA_274	1.15	-	Manes.06G092300.v6.1	0.67	+	-1262	lincRNA_171	1.10	-	Manes.14G121600.v6.1	1.55	-	-2213
lincRNA_327	1.13	-	Manes.03G092100.v6.1	5.19	+	-2060	lincRNA_394	1.09	-	Manes.05G174000.v6.1	1.71	-	-1228
lincRNA_105	1.13	-	Manes.15G059900.v6.1	0.76	-	-695	lincRNA_137	1.09	-	Manes.13G083600.v6.1	1.13	-	-69
lincRNA_257	1.11	-	Manes.02G122600.v6.1	1.29	-	-1812	lincRNA_108	1.09	-	Manes.15G005500.v6.1	8.45	-	-841
lincRNA_184	1.11	+	Manes.14G167200.v6.1	0.62	+	-2091	lincRNA_106	1.09	-	Manes.15G074600.v6.1	1.09	+	-501
lincRNA_30	1.10	+	Manes.10G058600.v6.1	1.04	+	-3881	lincRNA_248	1.09	+	Manes.02G035600.v6.1	0.19	-	-3502
lincRNA_171	1.10	-	Manes.14G121500.v6.1	1.14	-	-750	lincRNA_405	1.08	-	Manes.05G085900.v6.1	1.13	-	-7107
lincRNA_394	1.09	-	Manes.05G173900.v6.1	0.84	-	-1592	lincRNA_448	1.08	+	Manes.08G053800.v6.1	1.97	+	-151
lincRNA_137	1.09	-	Manes.13G083500.v6.1	1.37	+	-5801	lincRNA_413	1.08	-	Manes.04G058200.v6.1	NA	+	-3910
lincRNA_106	1.09	-	Manes.15G074500.v6.1	1.01	-	-767	lincRNA_262	1.08	-	Manes.S003000.v6.1	1.14	-	-6715
lincRNA_248	1.09	+	Manes.02G035500.v6.1	0.51	-	-2780	lincRNA_102	1.08	-	Manes.15G047200.v6.1	1.02	-	-406
lincRNA_448	1.08	+	Manes.08G053700.v6.1	1.01	+	-1741	lincRNA_2	1.06	+	Manes.S033800.v6.1	0.75	+	-8094
lincRNA_413	1.08	-	Manes.04G058100.v6.1	3.31	+	-769	lincRNA_453	1.06	+	Manes.S085800.v6.1	0.94	+	-5086
lincRNA_102	1.08	-	Manes.15G047100.v6.1	1.04	-	-1144	lincRNA_280	1.06	+	Manes.06G126800.v6.1	2.08	+	-2999
lincRNA_446	1.07	-	Manes.08G172300.v6.1	4.54	-	-3448	lincRNA_447	1.06	-	Manes.08G048300.v6.1	1.11	-	-175
lincRNA_2	1.06	+	Manes.S033700.v6.1	2.70	+	-1913	lincRNA_287	1.05	+	Manes.06G164800.v6.1	1.52	-	-3203
lincRNA_453	1.06	+	Manes.S085700.v6.1	5.48	-	-6710	lincRNA_371	1.04	+	Manes.09G041200.v6.1	1.22	+	-3624
lincRNA_280	1.06	+	Manes.06G126700.v6.1	1.76	+	-2741	lincRNA_428	1.03	+	Manes.S050600.v6.1	1.20	+	-5243
lincRNA_287	1.05	+	Manes.06G164600.v6.1	0.61	-	-7505	lincRNA_167	1.03	+	Manes.11G153800.v6.1	0.10	+	-2493
lincRNA_315	1.04	-	Manes.01G208000.v6.1	0.41	+	-93	lincRNA_383	1.02	+	Manes.05G118700.v6.1	0.84	+	-8803
lincRNA_428	1.03	+	Manes.S050500.v6.1	0.58	+	-3989	lincRNA_130	1.02	-	Manes.12G080700.v6.1	1.03	-	-7066
lincRNA_167	1.03	+	Manes.11G153700.v6.1	0.48	+	-3854	lincRNA_332	1.02	-	Manes.03G152400.v6.1	0.69	-	-1330
lincRNA_130	1.02	-	Manes.12G080600.v6.1	1.80	-	-6546	lincRNA_214	1.01	-	Manes.S044100.v6.1	1.17	-	-359
lincRNA_332	1.02	-	Manes.03G152300.v6.1	0.62	-	-1101	lincRNA_203	1.00	+	Manes.18G045000.v6.1	0.28	+	-2248
lincRNA_304	1.02	+	Manes.01G107400.v6.1	0.69	+	-3120	lincRNA_331	0.99	-	Manes.03G143000.v6.1	2.48	-	-1261
lincRNA_214	1.01	-	Manes.S044000.v6.1	0.72	+	-5865	lincRNA_163	0.99	-	Manes.11G105500.v6.1	1.09	-	-6044
lincRNA_203	1.00	+	Manes.18G044900.v6.1	0.74	+	-1282	lincRNA_8	0.99	-	Manes.10G075900.v6.1	NA	+	-5959

lincRNA_331	0.99	-	Manes.03G142900.v6.1	1.53	+	-299	lincRNA_177	0.99	+	Manes.14G148500.v6.1	1.04	+	-6213
lincRNA_163	0.99	-	Manes.11G105400.v6.1	0.55	+	-6799	lincRNA_422	0.98	+	Manes.04G030600.v6.1	NA	+	-6702
lincRNA_177	0.99	+	Manes.14G148400.v6.1	1.15	+	-312	lincRNA_209	0.98	+	Manes.18G082600.v6.1	1.14	+	-5231
lincRNA_209	0.98	+	Manes.18G082500.v6.1	1.03	+	-4939	lincRNA_51	0.98	+	Manes.16G088400.v6.1	1.69	+	-3483
lincRNA_51	0.98	+	Manes.16G088300.v6.1	2.32	-	-3145	lincRNA_420	0.97	-	Manes.04G152700.v6.1	1.08	-	-1974
lincRNA_420	0.97	-	Manes.04G152600.v6.1	NA	+	-1447	lincRNA_430	0.96	+	Manes.08G015000.v6.1	1.04	+	-1917
lincRNA_99	0.96	-	Manes.15G192700.v6.1	1.80	-	-7383	lincRNA_99	0.96	-	Manes.15G192800.v6.1	1.27	-	-1332
lincRNA_49	0.96	-	Manes.16G073700.v6.1	165.60	-	-4336	lincRNA_49	0.96	-	Manes.16G073800.v6.1	1.42	+	-2161
lincRNA_323	0.95	+	Manes.03G000500.v6.1	0.35	+	-1119	lincRNA_104	0.96	+	Manes.15G000200.v6.1	0.85	+	-2988
lincRNA_235	0.95	+	Manes.07G135900.v6.1	0.85	-	-3187	lincRNA_235	0.95	+	Manes.07G136000.v6.1	2.84	-	-476
lincRNA_267	0.95	-	Manes.S077800.v6.1	0.99	-	-1631	lincRNA_399	0.95	+	Manes.05G200500.v6.1	0.96	+	-8289
lincRNA_399	0.95	+	Manes.05G200400.v6.1	#######	+	-3565	lincRNA_443	0.94	-	Manes.08G155800.v6.1	1.15	+	-1622
lincRNA_344	0.95	-	Manes.03G070300.v6.1	1.57	-	-9370	lincRNA_218	0.94	+	Manes.S004600.v6.1	1.27	+	-9487
lincRNA_443	0.94	-	Manes.08G155700.v6.1	1.03	-	-2532	lincRNA_39	0.93	+	Manes.16G011900.v6.1	1.08	-	-1831
lincRNA_218	0.94	+	Manes.S004500.v6.1	1.04	+	-848	lincRNA_23	0.93	+	Manes.10G047100.v6.1	1.22	+	-1928
lincRNA_409	0.94	-	Manes.04G010400.v6.1	1.21	-	-1226	lincRNA_352	0.93	+	Manes.09G076700.v6.1	Inf	-	-652
lincRNA_96	0.94	+	Manes.15G188500.v6.1	0.77	+	-8355	lincRNA_143	0.92	+	Manes.13G114800.v6.1	3.25	+	-2193
lincRNA_39	0.93	+	Manes.16G011800.v6.1	1.27	-	-511	lincRNA_239	0.92	+	Manes.07G064600.v6.1	1.09	+	-9713
lincRNA_23	0.93	+	Manes.10G047000.v6.1	1.09	+	-60	lincRNA_168	0.92	-	Manes.11G064300.v6.1	1.22	+	-2299
lincRNA_418	0.93	-	Manes.04G088700.v6.1	0.08	+	-2863	lincRNA_50	0.91	+	Manes.16G081400.v6.1	2.41	-	-4151
lincRNA_143	0.92	+	Manes.13G114700.v6.1	1.62	+	-4549	lincRNA_450	0.91	+	Manes.08G063000.v6.1	1.12	+	-2259
lincRNA_239	0.92	+	Manes.07G064500.v6.1	NA	+	-7569	lincRNA_283	0.91	+	Manes.06G136700.v6.1	0.31	-	-280
lincRNA_168	0.92	-	Manes.11G064200.v6.1	0.60	-	-9843	lincRNA_340	0.91	+	Manes.03G009700.v6.1	0.85	+	-124
lincRNA_50	0.91	+	Manes.16G081300.v6.1	1.43	-	-4661	lincRNA_341	0.90	-	Manes.03G009800.v6.1	1.17	-	-3291
lincRNA_406	0.91	-	Manes.05G099000.v6.1	1.36	-	-870	lincRNA_395	0.90	-	Manes.05G183500.v6.1	Inf	-	-2155
lincRNA_340	0.91	+	Manes.03G009600.v6.1	23.06	-	-1755	lincRNA_421	0.90	+	Manes.04G163200.v6.1	1.08	+	-1313
lincRNA_341	0.90	-	Manes.03G009700.v6.1	0.85	+	-581	lincRNA_368	0.90	+	Manes.09G170500.v6.1	0.99	+	-1530
lincRNA_73	0.90	-	Manes.17G074700.v6.1	2.41	-	-332	lincRNA_121	0.89	+	Manes.12G119600.v6.1	0.31	-	-4878
lincRNA_395	0.90	-	Manes.05G183400.v6.1	0.24	+	-121	lincRNA_112	0.89	+	Manes.12G084900.v6.1	1.07	+	-9002
lincRNA_368	0.90	+	Manes.09G170400.v6.1	1.48	+	-200	lincRNA_190	0.89	+	Manes.14G076600.v6.1	1.10	+	-5836
lincRNA_112	0.89	+	Manes.12G084800.v6.1	0.81	+	-5819	lincRNA_374	0.88	-	Manes.09G055100.v6.1	0.48	+	-768
lincRNA_190	0.89	+	Manes.14G076500.v6.1	0.50	+	-1569	lincRNA_109	0.88	+	Manes.15G083300.v6.1	1.89	-	-9632
lincRNA_374	0.88	-	Manes.09G055000.v6.1	1.85	+	-3021	lincRNA_67	0.88	+	Manes.17G006600.v6.1	1.25	-	-428
lincRNA_109	0.88	+	Manes.15G083200.v6.1	0.91	+	-420	lincRNA_210	0.87	-	Manes.18G099000.v6.1	0.75	-	-1744
lincRNA_67	0.88	+	Manes.17G006500.v6.1	0.85	+	-123	lincRNA_16	0.87	-	Manes.10G109600.v6.1	1.08	-	-709
lincRNA_210	0.87	-	Manes.18G098900.v6.1	0.92	-	-318	lincRNA_205	0.86	+	Manes.18G052100.v6.1	0.47	+	-1550
lincRNA_205	0.86	+	Manes.18G052000.v6.1	0.75	+	-593	lincRNA_308	0.86	+	Manes.01G132000.v6.1	1.67	+	-5404
lincRNA_91	0.86	-	Manes.15G183200.v6.1	1.41	-	-7594	lincRNA_398	0.85	-	Manes.05G195500.v6.1	1.20	+	-4313
lincRNA_398	0.85	-	Manes.05G195400.v6.1	0.69	-	-221	lincRNA_195	0.85	+	Manes.18G021600.v6.1	0.10	-	-271
lincRNA_275	0.85	+	Manes.06G013900.v6.1	0.87	+	-556	lincRNA_275	0.85	+	Manes.06G014000.v6.1	1.08	+	-1892
lincRNA_160	0.85	+	Manes.11G017500.v6.1	0.52	+	-110	lincRNA_170	0.84	-	Manes.11G079700.v6.1	0.75	-	-610
lincRNA_440	0.84	-	Manes.08G033900.v6.1	3.76	+	-796	lincRNA_440	0.84	-	Manes.08G034000.v6.1	4.72	+	-9242
lincRNA_305	0.84	+	Manes.01G107400.v6.1	0.69	+	-4423	lincRNA_269	0.84	-	Manes.06G039100.v6.1	NA	+	-416
lincRNA_269	0.84	-	Manes.06G039000.v6.1	Inf	+	-9817	lincRNA_316	0.82	-	Manes.01G208100.v6.1	1.15	+	-7996
lincRNA_255	0.83	+	Manes.02G081900.v6.1	1.37	+	-397	lincRNA_423	0.82	-	Manes.04G049300.v6.1	Inf	+	-2985
lincRNA_89	0.81	+	Manes.15G150500.v6.1	0.65	+	-5940	lincRNA_89	0.81	+	Manes.15G150600.v6.1	0.34	+	-7693
lincRNA_243	0.80	+	Manes.02G211600.v6.1	0.87	+	-4938	lincRNA_98	0.80	+	Manes.15G189100.v6.1	0.74	+	-5112
lincRNA_328	0.79	+	Manes.03G107900.v6.1	1.77	+	-1358	lincRNA_402	0.80	+	Manes.05G057600.v6.1	1.48	+	-855
lincRNA_314	0.78	+	Manes.01G204000.v6.1	#######	-	-4626	lincRNA_176	0.79	-	Manes.14G145700.v6.1	1.12	-	-965
lincRNA_225	0.77	+	Manes.07G017600.v6.1	0.84	-	-7187	lincRNA_90	0.79	+	Manes.15G179600.v6.1	0.75	+	-522
lincRNA_52	0.76	+	Manes.16G094100.v6.1	1.81	+	-9274	lincRNA_314	0.78	+	Manes.01G204100.v6.1	0.74	-	-2967
lincRNA_325	0.76	-	Manes.03G087900.v6.1	0.87	-	-3710	lincRNA_225	0.77	+	Manes.07G017700.v6.1	1.29	-	-1569
lincRNA_336	0.76	+	Manes.03G212600.v6.1	0.49	+	-2833	lincRNA_52	0.76	+	Manes.16G094200.v6.1	0.82	+	-88
lincRNA_71	0.76	-	Manes.17G059200.v6.1	0.99	-	-1575	lincRNA_325	0.76	-	Manes.03G088000.v6.1	8.49	+	-1840
lincRNA_384	0.76	-	Manes.05G018600.v6.1	9.66	+	-2761	lincRNA_71	0.76	-	Manes.17G059300.v6.1	1.53	+	-3448
lincRNA_144	0.75	-	Manes.13G115600.v6.1	NA	+	-2645	lincRNA_384	0.76	-	Manes.05G018700.v6.1	691.52	+	-6189
lincRNA_182	0.75	-	Manes.14G163200.v6.1	0.89	-	-1972	lincRNA_266	0.75	+	Manes.S011500.v6.1	NA	+	-7009
lincRNA_310	0.74	-	Manes.01G145500.v6.1	0.77	+	-5937	lincRNA_144	0.75	-	Manes.13G115700.v6.1	0.89	-	-8360
lincRNA_264	0.74	-	Manes.S002400.v6.1	1.67	+	-2223	lincRNA_202	0.75	-	Manes.18G043000.v6.1	0.93	-	-165
lincRNA_100	0.74	-	Manes.15G035000.v6.1	0.64	-	-3385	lincRNA_310	0.74	-	Manes.01G145600.v6.1	1.16	+	-5862
lincRNA_145	0.74	-	Manes.13G129100.v6.1	0.85	-	-6221	lincRNA_216	0.74	+	Manes.S050000.v6.1	13.10	-	-5097
lincRNA_111	0.74	+	Manes.15G127300.v6.1	1.16	+	-1296	lincRNA_264	0.74	-	Manes.S002500.v6.1	0.82	+	-3503
lincRNA_103	0.74	-	Manes.15G048000.v6.1	0.15	+	-1322	lincRNA_100	0.74	-	Manes.15G035100.v6.1	0.54	-	-1904
lincRNA_240	0.73	-	Manes.02G171200.v6.1	0.72	-	-9193	lincRNA_145	0.74	-	Manes.13G129200.v6.1	1.43	+	-6569
lincRNA_142	0.72	+	Manes.13G105300.v6.1	0.89	+	-7247	lincRNA_222	0.74	-	Manes.07G071800.v6.1	#NAME?	+	-5869
lincRNA_281	0.72	+	Manes.06G130100.v6.1	1.36	-	-491	lincRNA_111	0.74	+	Manes.15G127400.v6.1	5.50	+	-3602
lincRNA_158	0.71	+	Manes.11G088100.v6.1	Inf	-	-2556	lincRNA_313	0.73	+	Manes.01G175900.v6.1	0.63	+	-3431
lincRNA_351	0.71	-	Manes.09G076200.v6.1	1.12	-	-2852	lincRNA_281	0.72	+	Manes.06G130200.v6.1	1.21	+	-2723
lincRNA_282	0.71	-	Manes.06G000500.v6.1	0.98	-	-6446	lincRNA_351	0.71	-	Manes.09G076300.v6.1	1.06	-	-5681
lincRNA_57	0.70	-	Manes.16G132400.v6.1	0.60	-	-1476	lincRNA_282	0.71	-	Manes.06G000600.v6.1	NA	-	-4620
lincRNA_191	0.70	+	Manes.14G085100.v6.1	0.29	+	-2995	lincRNA_57	0.70	-	Manes.16G132500.v6.1	1.11	+	-158
lincRNA_72	0.69	+	Manes.17G068300.v6.1	1.28	-	-1749	lincRNA_186	0.70	-	Manes.14G041500.v6.1	1.10	-	-4054
lincRNA_122	0.69	-	Manes.12G120700.v6.1	1.05	-	-9178	lincRNA_191	0.70	+	Manes.14G085200.v6.1	0.71	+	-5595
lincRNA_192	0.67	+	Manes.18G000300.v6.1	0.96	+	-7705	lincRNA_122	0.69	-	Manes.12G120800.v6.1	0.48	-	-1503
lincRNA_88	0.61	-	Manes.15G134700.v6.1	#######	+	-5355	lincRNA_393	0.17	-	Manes.05G158300.v6.1	#NAME?	+	-3929
lincRNA_359	0.59	-	Manes.09G090500.v6.1	NA	+	-7580	lincRNA_88	0.61	-	Manes.15G134800.v6.1	NA	+	-301
lincRNA_312	0.44	-	Manes.01G167900.v6.1	1.45	+	-522	lincRNA_229	0.61	+	Manes.07G100800.v6.1	NA	-	-1734
lincRNA_147	0.52	-	Manes.13G141400.v6.1	1.39	+	-634	lincRNA_133	0.47	-	Manes.13G076400.v6.1	NA	+	-5067
lincRNA_207	0.62	+	Manes.18G005100.v6.1	1.35	-	-3615	lincRNA_285	0.41	+	Manes.06G146600.v6.1	NA	+	-7837
lincRNA_254	0.27	+	Manes.02G043800.v6.1	1.26	+	-3263	lincRNA_232	0.55	+	Manes.07G112700.v6.1	1.45	+	-1451
lincRNA_342	0.08	-	Manes.03G069400.v6.1	1.20	+	-565	lincRNA_193	0.36	-	Manes.18G020700.v6.1	1.43	-	-3317
lincRNA_372	0.62	-	Manes.09G045800.v6.1	1.15	-	-1026	lincRNA_276	0.53	-	Manes.06G108800.v6.1	1.36	+	-999
lincRNA_41	0.56	+	Manes.16G057200.v6.1	1.03	+	-5911	lincRNA_419	0.62	-	Manes.04G126100.v6.1	1.15	+	-7589
lincRNA_55	0.34	+	Manes.16G128500.v6.1	0.99	+	-6654	lincRNA_207	0.62	+	Manes.18G005200.v6.1	1.08	+	-80
lincRNA_378	0.49	-	Manes.S087000.v6.1	0.97	-	-1656	lincRNA_1	0.45	+	Manes.S034000.v6.1	1.06	+	-4307
lincRNA_234	0.36	+	Manes.07G130600.v6.1	0.95	+	-1353	lincRNA_147	0.52	-	Manes.13G141500.v6.1	1.03	+	-6208
lincRNA_1	0.45	+	Manes.S033900.v6.1	0.93	+	-3638	lincRNA_156	0.64	-	Manes.11G081100.v6.1	0.99	-	-1292
lincRNA_439	0.40	+	Manes.08G134600.v6.1	0.92	+	-1417	lincRNA_192	0.67	+	Manes.18G000400.v6.1	0.96	+	-5192

lincRNA_199	0.65	+	Manes.18G145600.v6.1	0.90	+	-1471	lincRNA_199	0.65	+	Manes.18G145700.v6.1	0.95	+	-5112
lincRNA_78	0.41	+	Manes.17G120300.v6.1	0.87	+	-256	lincRNA_312	0.44	-	Manes.01G168000.v6.1	0.92	+	-1635
lincRNA_369	0.61	-	Manes.09G173300.v6.1	0.86	-	-9804	lincRNA_369	0.61	-	Manes.09G173400.v6.1	0.92	-	-4479
lincRNA_419	0.62	-	Manes.04G126000.v6.1	0.85	-	-5905	lincRNA_366	0.35	-	Manes.09G157700.v6.1	0.90	-	-445
lincRNA_77	0.26	+	Manes.17G112400.v6.1	0.80	+	-1025	lincRNA_286	0.33	-	Manes.06G161400.v6.1	0.86	-	-679
lincRNA_401	0.43	-	Manes.05G048700.v6.1	0.80	+	-5684	lincRNA_290	0.47	+	Manes.06G031200.v6.1	0.83	+	-8278
lincRNA_434	0.56	-	Manes.08G092200.v6.1	0.74	+	-1306	lincRNA_87	0.61	-	Manes.S090500.v6.1	0.81	-	-558
lincRNA_393	0.17	-	Manes.05G158200.v6.1	0.73	-	-7020	lincRNA_414	0.45	-	Manes.04G012900.v6.1	0.78	+	-6964
lincRNA_7	0.5001245	+	Manes.10G075000.v6.1	0.69548	+	-7266	lincRNA_227	0.666181	-	Manes.07G082200.v6.1	0.76781	-	-2015

Supplemental Data S7. Expression information of protein-coding genes correlated with cold-responsive lncRNAs under cold stress

Gene ID	Cold/CK Fol	pval	Up/Dow	Signi	Uniprot:Desc	Pfam:pfam_	Pfam:pfam_Name	Pfam:pfam_	GO:biological	GO:cellular_c	GO:molecular_f	KEGG:KO
Manes.01G00080	1.1393313	0.24522	up	no	.	pfam08192	Peptidase_S64	Peptidase fa	.	.	.	.
Manes.01G00110	0.7598354	0.00813	down	no	Autophagy-r	pfam10033	ATG13	Autophagy-	GO:0006914|	GO:0034045|	.	K08331
Manes.01G00130	0.4145797	0.00659	down	yes	.	pfam14675	FANCI_S1	FANCI sole	.	.	.	.
Manes.01G00190	3.4617721	2.03E-42	up	yes	Trihelix trans	pfam13837	Myb_DNA-bind_4	Myb/SANT	GO:0042631|	GO:0005634|	GO:0003677|D	.
Manes.01G00210	1.9317219	4.43E-13	up	no	Nuclear pore	pfam04096	Nucleoporin2	Nucleoporin	GO:0051028|	GO:0005635|	.	K14297
Manes.01G00230	0.4103378	2.89E-05	down	yes	.	pfam05997	Nop52	Nucleolar p	.	.	.	.
Manes.01G00240	1.5713865	8.14E-07	up	no	Calmodulin	pfam13499	EF-hand_7	EF-hand do	.	.	GO:0005509|ca	.
Manes.01G00250	0.8570432	0.11063	down	no	Protein trans	pfam04811	Sec23_trunk	Sec23/Sec2	GO:0006888|	GO:0030127|	GO:0008270|zi	.
Manes.01G00280	0.2705773	1.23E-20	down	yes	Endoglucana	pfam00759	Glyco_hydro_9	Glycosyl hy	GO:0030245|	.	GO:0008810|ce	.
Manes.01G00290	1.1237895	0.33853	up	no	DNA topoiso	pfam01131	Topoisom_bac	DNA topois	GO:0006265|	.	GO:0003677|D	K03165
Manes.01G00330	0.5598302	0.00096	down	no	Xyloglucan g	pfam03016	Exostosin	Exostosin fa	GO:0010256|	GO:0005768|	GO:0016757|tra	.
Manes.01G00350	0.9938043	0.9931	down	no	.	pfam00397	WW	WW domai	.	.	.	.
Manes.01G00360	1.5346038	0.00173	up	no	.	pfam09971	DUF2206	Predicted m	.	.	.	.
Manes.01G00370	1.2377558	0.02648	up	no	Calcineurin B	pfam13499	EF-hand_7	EF-hand do	GO:0005513|	GO:0016020|	GO:0005509|ca	K06268
Manes.01G00380	0.708317	0.02555	down	no	Heat shock c	pfam00183	HSP90	Hsp90 prote	GO:0006457|	GO:0005737|	GO:0005524|A	K04079
Manes.01G00410	0.8650829	0.19732	down	no	Regulatory p	pfam02631	RecX	RecX family	GO:0006282|	GO:0005737|	.	K03565
Manes.01G00420	21.808695	1.65E-17	up	yes	E3 ubiquitin-	pfam13639	zf-RING_2	Ring finger	GO:0016567|	.	GO:0016874|lig	K11982
Manes.01G00460	0.1942446	2.04E-08	down	yes	Fructose-bisp	pfam00274	Glycolytic	Fructose-bis	GO:0006096|	GO:0005737|	GO:0004332|fru	.
Manes.01G00480	0.7675074	0.09101	down	no	Transcription	pfam06093	Spt4	Spt4/RpoE2	GO:0032786|	GO:0005634|	GO:0008270|zi	.
Manes.01G00510	0.3759726	0.02101	down	yes	Probable ami	pfam01490	Aa_trans	Transmemb	GO:0003333|	GO:0016021|	GO:0015293|sy	.
Manes.01G00540	Inf	0.35135	up	no	.	pfam14244	UBN2_3	gag-polypep	.	.	.	.
Manes.01G00560	0.6515738	0.00014	down	no	.	pfam02536	mTERF	mTERF. Th	.	.	.	.
Manes.01G00620	0.7003008	0.00212	down	no	Phosphatidyli	pfam00650	CRAL_TRIO	CRAL/TRI	GO:0015031|	GO:0000139|	.	.
Manes.01G00630	2.6064194	4.90E-11	up	yes	GPI-anchore	pfam15024	Glyco_transf_18	Glycosyltra	GO:0009567|	GO:0031225|	.	.
Manes.01G00650	30.858328	9.29E-24	up	yes	Zinc finger p	pfam13912	zf-C2H2_6	C2H2-type	GO:0006355|	GO:0005634|	GO:0046872|m	.
Manes.01G00730	0.6339051	2.30E-05	down	no	.	pfam15320	RAM	mRNA cap	.	.	.	.
Manes.01G00740	0.8446349	0.10586	down	no	.	pfam04006	Mpp10	Mpp10 prot	.	.	.	.
Manes.01G00750	0.5494933	0.00172	down	no	Chloride cha	pfam00654	Voltage_CLC	Voltage gat	.	GO:0034707|	GO:0005247|vo	.
Manes.01G00780	1.2068263	0.03669	up	no	Methionine a	pfam00557	Peptidase_M24	Metallopept	GO:0070084|	GO:0005737|	GO:0046872|m	K01265
Manes.01G00790	0.685	0.00147	down	no	Probable pro	pfam00481	PP2C	Protein pho	GO:0006470|	GO:0005886|	GO:0046872|m	.
Manes.01G00800	1.3787118	0.00041	up	no	Peroxisome b	pfam13414	TPR_11	TPR repeat.	GO:0016560|	GO:0005829|	GO:0005052|pe	K13342
Manes.01G00880	0.8736584	0.16855	down	no	Methylenetet	pfam02219	MTHFR	Methylenete	GO:0006555|	GO:0005829|	GO:0004489|m	K00297
Manes.01G00910	0.4789092	3.78E-05	down	yes	F-box/kelch-	pfam01344	Kelch_1	Kelch motif.	GO:2000762|	GO:0005829|	.	.
Manes.01G00950	8.2065892	2.62E-60	up	yes	.	pfam06378	DUF1071	Protein of u	.	.	.	.
Manes.01G00970	0.6632604	2.71E-05	down	no	.	pfam08373	RAP	RAP domai	.	.	.	.
Manes.01G00990	0.9621155	0.74253	down	no	Inositol 1,3,4	pfam05770	Ins134_P3_kin	Inositol 1, 3	GO:0032957|	GO:0005829|	GO:0005524|A	.
Manes.01G01040	20.178775	0.00053	up	yes	Cytochrome	pfam00067	p450	Cytochrome	GO:0098542|	GO:0016021|	GO:0020037|he	.
Manes.01G01080	1.1562211	0.18415	up	no	.	pfam02536	mTERF	mTERF. Th	.	.	.	.
Manes.01G01130	2.0015275	1.89E-11	up	yes	.	pfam07891	DUF1666	Protein of u	.	.	.	.
Manes.01G01160	0.575459	0.00177	down	no	Succinate de	pfam13232	Complex1_LYR_1	Complex1_	GO:0016226|	GO:0005759|	.	.
Manes.01G01200	0.188497	3.45E-13	down	yes	Zinc finger p	pfam06203	CCT	CCT motif.	GO:0006355|	GO:0016020|	GO:0003700|tra	.
Manes.01G01210	0.9581581	0.65924	down	no	Carbon catab	pfam03372	Exo_endo_phos	Endonuclea	GO:0006355|	GO:0005737|	GO:0046872|m	.
Manes.01G01220	2.8924539	8.72E-07	up	yes	Ethylene-res	pfam00847	AP2	AP2 domai	GO:0009736|	GO:0005737|	GO:0003677|D	.
Manes.01G01250	1.2152874	0.03218	up	no	.	pfam00766	ETF_alpha	Electron tra	.	.	.	.
Manes.01G01260	0.8988699	0.30142	down	no	Aluminum-ac	pfam11744	ALMT	Aluminium	GO:0098656|	GO:0016021|	GO:0005253|an	.
Manes.01G01280	0.963893	0.72501	down	no	Actin-related	pfam00022	Actin	Actin.	GO:0006325|	GO:0005737|	GO:0005200|st	K11340
Manes.01G01290	0.8875146	0.2345	down	no	Protein CAS	pfam08172	CASP_C	CASP C ter	GO:0006891|	GO:0005768|	.	K09313
Manes.01G01330	0.6579915	0.00075	down	no	Short-chain d	pfam00106	adh_short	short chain	GO:0015031|	GO:0009507|	GO:0016491|ox	.
Manes.01G01350	1.265069	0.01602	up	no	Zinc finger C	pfam00400	WD40	WD domain	.	.	GO:0003677|D	.
Manes.01G01400	0.2856685	2.07E-06	down	yes	.	pfam07797	DUF1639	Protein of u	.	.	.	.
Manes.01G01420	15.934021	0.00031	up	yes	.	pfam02485	Branch	Core-2/I-Br	.	.	.	.
Manes.01G01490	0.5321625	6.24E-07	down	no	L-Ala-D/L-a	pfam13378	MR_MLE_C	Enolase C-t	GO:0006518|	.	GO:0000287|ma	.
Manes.01G01520	0.8484239	0.27611	down	no	.	pfam07247	AATase	Alcohol ace	.	.	.	.
Manes.01G01530	1.2728084	0.01223	up	no	Probable E3	pfam13639	zf-RING_2	Ring finger	GO:0016567|	.	GO:0016874|lig	.
Manes.01G01540	0.4005798	5.57E-10	down	yes	UDP-N-acet	pfam04101	Glyco_tran_28_C	Glycosyltra	GO:0007049|	GO:0005886|	GO:0030246|ca	K02563
Manes.01G01600	0.8661131	0.17598	down	no	Characteri	pfam03171	2OG-FeII_Oxy	2OG-Fe(II)	.	.	GO:0005506|irc	.
Manes.01G01610	0.3880176	0.00099	down	yes	.	pfam07887	Calmodulin_bind	Calmodulin	.	.	.	.
Manes.01G01690	0.9349655	0.56191	down	no	U5 small nuc	pfam00400	WD40	WD domain	GO:0010467|	GO:0071013|	GO:0044822|po	K12857
Manes.01G01700	1.2242284	0.03324	up	no	60S ribosom	pfam14204	Ribosomal_L18_c	Ribosomal	GO:0006412|	GO:0005840|	GO:0008097|5S	K02932
Manes.01G01710	2.5459852	4.01E-14	up	yes	Putative dise	pfam00931	NB-ARC	NB-ARC do	GO:0006952|	.	GO:0043531|A	.
Manes.01G01740	Inf	0.00381	up	yes	Zinc finger A	pfam01428	zf-AN1	AN1-like Zi	.	GO:0009506|	GO:0003677|D	.
Manes.01G01770	0.8861128	0.29537	down	no	Acyl-coenzy	pfam03061	4HBT	Thioesteras	GO:0006637|	GO:0005739|	GO:0003986|ac	K17361
Manes.01G01820	25.710055	5.55E-30	up	yes	UDP-glycosy	pfam00201	UDPGT	UDP-glucor	GO:0071475|	GO:0043231|	GO:0052638|in	.
Manes.01G01830	1.9638288	2.20E-11	up	no	LETM1 and	pfam07766	LETM1	LETM1-like	.	GO:0016021|	GO:0005509|ca	K17800
Manes.01G01870	0.6008027	5.00E-06	down	no	NADP-deper	pfam00107	ADH_zinc_N	Zinc-binding	GO:0006979|	GO:0005829|	GO:0032440|2-	K08070
Manes.01G01880	0.8483435	0.22268	down	no	NADP-deper	pfam00107	ADH_zinc_N	Zinc-binding	GO:0006979|	GO:0005829|	GO:0032440|2-	K08070
Manes.01G01960	0.4122867	9.72E-10	down	yes	Ankyrin repe	pfam12796	Ank_2	Ankyrin rep	.	GO:0005783|	.	.
Manes.01G01980	#NAME?	0.14005	down	no	.	pfam07030	DUF1320	Protein of u	.	.	.	.
Manes.01G02010	1.2549782	0.01617	up	no	Putative lyso	pfam04083	Abhydro_lipase	Partial alpha	GO:0016042|	GO:0005576|	GO:0004771|ste	.
Manes.01G02120	0.9685596	0.77451	down	no	Suppressor o	pfam00400	WD40	WD domain	GO:0008380|	GO:0080008|	.	K13111
Manes.01G02130	6.2950615	1.85E-06	up	yes	.	pfam14220	DUF4329	Domain of u	.	.	.	.
Manes.01G02140	1.7606382	3.88E-06	up	no	Oleosin 1	pfam01277	Oleosin	Oleosin.	.	GO:0016021|	.	.
Manes.01G02150	2.0279574	4.57E-10	up	yes	.	pfam00086	Thyroglobulin_1	Thyroglobul	.	.	.	.
Manes.01G02160	0.672306	0.05712	down	no	.	pfam00582	Usp	Universal st	.	.	.	.
Manes.01G02180	2.5205942	1.15E-25	up	yes	Nuclear-inter	pfam07967	zf-C3HC	C3HC zinc	GO:0051301|	GO:0031965|	GO:0019901|pr	.
Manes.01G02200	2.9307893	1.19E-21	up	yes	Protein MIZ	pfam04759	DUF617	Protein of u	GO:0009658|	GO:0032541|	.	.
Manes.01G02300	#NAME?	0.64948	down	no	Tetraspanin-1	pfam00335	Tetraspannin	Tetraspanin	.	GO:0016021|	.	.
Manes.01G02330	0.7840769	0.06223	down	no	Probable 2-a	pfam07847	DUF1637	Protein of u	.	.	GO:0047800|cy	K10712
Manes.01G02360	10.641878	1.31E-30	up	yes	Protein STRI	pfam03088	Str_synth	Strictosidine	GO:0009820|	GO:0005618|	GO:0016844|st	K01757
Manes.01G02370	1.2283268	0.06199	up	no	H/ACA ribon	pfam01248	Ribosomal_L7Ae	Ribosomal	GO:0031118|	GO:0005730|	GO:0030515|sn	K11129
Manes.01G02390	1.3583952	0.00424	up	no	Agmatine dei	pfam04371	PAD_porph	Porphyrom	GO:0006596|	.	GO:0047632|ag	K10536
Manes.01G02400	0.7885218	0.09704	down	no	Serine/threon	pfam00069	Pkinase	Protein kina	GO:0071560|	GO:0005798|	GO:0005524|A	K08856
Manes.01G02410	0.1954282	7.98E-14	down	yes	Zinc finger p	pfam06203	CCT	CCT motif.	GO:0009909|	GO:0005634|	GO:0003677|D	.
Manes.01G02420	12.073532	3.25E-17	up	yes	.	pfam07926	TPR_MLP1_2	TPR/MLP1/	.	.	.	.
Manes.01G02430	1.0624603	0.49534	up	no	Serine protea	pfam01343	Peptidase_S49	Peptidase fa	GO:0006508|	GO:0009507|	GO:0004252|se	K04773
Manes.01G02440	0.9614591	0.7032	down	no	Translin-asso	pfam01997	Translin	Translin fam	GO:0030154|	GO:0005737|	GO:0031687|A	.

Manes.01G02460(	0.5847509	0.00012	down	no	.	pfam14432	DYW_deaminase	DYW family	.	.	.	.
Manes.01G02530(	2.0723243	1.28E-05	up	yes	Cytochrome	pfam00067	p450	Cytochrome	.	GO:0016021|	GO:0020037|he	.
Manes.01G02550(	0.7044453	0.07014	down	no	.	pfam00582	Usp	Universal st	.	.	.	.
Manes.01G02580(	0.3225566	0.00036	down	yes	.	pfam10602	RPN7	26S proteas	.	.	.	.
Manes.01G02590(	0.4523895	3.80E-07	down	yes	.	pfam15440	THRAP3_BCLAF	THRAP3/B(	.	.	.	.
Manes.01G02600(	0.3829722	3.31E-09	down	yes	Squamosa pr	pfam03110	SBP	SBP domair	GO:0042742|	GO:0005634|	GO:0003677|DI	.
Manes.01G02620(	0.4449082	6.42E-06	down	yes	.	pfam05965	FYRC	F/Y rich C-t	.	.	.	.
Manes.01G02650(	1.8761253	1.47E-06	up	no	Cytochrome	pfam00173	Cyt-b5	Cytochrome	.	GO:0009707|	GO:0020037|he	.
Manes.01G02670(	1.2108742	0.04848	up	no	Fructokinase-	pfam00294	PfkB	pfkB family	GO:0009658|	GO:0042644|	GO:0016301|kii	.
Manes.01G02680(	0.5481121	3.85E-06	down	no	Probable 6-pl	pfam01182	Glucosamine_iso	Glucosamine	GO:0005975|	GO:0005829|	GO:0017057|6-	K01057
Manes.01G02700(	1.179722	0.12217	up	no	ATP-depende	pfam07717	OB_NTP_bind	Oligonucleo	GO:0000398|	GO:0071013|	GO:0005524|A'	K12818
Manes.01G02720(	1.7333791	8.45E-05	up	no	Ninja-family	pfam07897	DUF1675	Protein of u	GO:0009737|	GO:0005634|	.	.
Manes.01G02740(	0.3573838	9.55E-17	down	yes	Magnesium t	pfam00038	Filament	Intermediate	GO:0098655|	GO:0016021|	GO:0015095|ma	.
Manes.01G02760(	1.5311877	0.00948	up	no	.	pfam13041	PPR_2	PPR repeat	.	.	.	.
Manes.01G02770(	2.8267191	6.17E-20	up	yes	Protein root	pfam04884	DUF647	Vitamin B6	.	GO:0016021|	.	.
Manes.01G02790(	0.3324103	3.59E-06	down	yes	Ureide perme	pfam07168	Ureide_permease	Ureide perm	GO:0015720|	GO:0016021|	GO:0005274|all	.
Manes.01G02870(	0.713352	0.00194	down	no	Probable met	pfam03141	Methyltransf_29	Putative S-a	GO:0032259|	GO:0005789|	GO:0008757|S-	.
Manes.01G02910(	2.0172234	2.03E-15	up	yes	AT-rich inter	pfam00249	Myb_DNA-bindin	Myb-like DI	GO:0006355|	GO:0005634|	GO:0003677|DI	.
Manes.01G03020(	0.4419244	1.39E-11	down	yes	.	pfam01661	Macro	Macro doma	.	.	.	.
Manes.01G03040(	0.6396189	3.48E-05	down	no	F-box only pi	pfam08755	YccV-like	Hemimethyl	.	.	GO:0003677|DI	K10301
Manes.01G03050(	1.2600296	0.1875	up	no	Putative pent	pfam13041	PPR_2	PPR repeat	.	GO:0005739|	.	.
Manes.01G03070(	0.7184614	0.00098	down	no	.	pfam05904	DUF863	Plant protei	.	.	.	.
Manes.01G03090(	0.70361	0.05358	down	no	Chorismate n	pfam01817	CM_2	Chorismate	GO:0009073|	GO:0009507|	GO:0004106|ch	K01850
Manes.01G03140(	0.7907126	0.06926	down	no	Isopentenyl p	pfam00696	AA_kinase	Amino acid	GO:0008299|	.	GO:0005524|A'	K06981
Manes.01G03150(	0.2301644	0.01304	down	yes	.	pfam10070	DUF2309	Uncharacter	.	.	.	.
Manes.01G03200(	0.709559	0.0475	down	no	.	pfam06012	DUF908	Domain of U	.	.	.	.
Manes.01G03230(	0.7320174	0.03726	down	no	Protein argor	pfam02171	Piwi	Piwi domair	GO:0035195|	GO:0005737|	GO:0003723|RI	K11593
Manes.01G03240(	0.4584998	1.45E-08	down	yes	CSC1-like pr	pfam02714	DUF221	Domain of u	GO:0006811|	GO:0016021|	.	.
Manes.01G03250(	1.6325476	4.53E-07	up	no	26S proteaso	pfam01399	PCI	PCI domain	GO:0030163|	GO:0005829|	.	K03036
Manes.01G03260(	0.5712139	1.60E-06	down	no	ARF guanine	pfam01369	Sec7	Sec7 domai	GO:0010540|	GO:0005829|	GO:0005086|AI	K18443
Manes.01G03280(	0.5202614	1.28E-09	down	no	ARF guanine	pfam01369	Sec7	Sec7 domai	GO:0010540|	GO:0005829|	GO:0005086|AI	K18443
Manes.01G03320(	0.4718133	3.60E-08	down	yes	Uncharacteri	pfam10250	O-FucT	GDP-fucose	.	GO:0005768|	GO:0016757|tra	.
Manes.01G03340(	1.9046241	0.00535	up	no	.	pfam07802	GCK	GCK domai	.	.	.	.
Manes.01G03350(	0.3778356	0.00843	down	yes	Phosphate tra	pfam03124	EXS	EXS family.	GO:0006817|	GO:0016021|	.	.
Manes.01G03370(	0.1645487	5.36E-15	down	yes	Galactoside 2	pfam03254	XG_FTase	Xyloglucan	GO:0042546|	GO:0005794|	GO:0008417|fu	K13681
Manes.01G03380(	0.9234348	0.51041	down	no	.	pfam13741	MRP-S25	Mitochondr	.	.	.	.
Manes.01G03430(	1.2804139	0.00862	up	no	.	pfam00013	KH_1	KH domain.	.	.	.	.
Manes.01G03440(	0.6413595	0.0011	down	no	.	pfam13639	zf-RING_2	Ring finger	.	.	.	.
Manes.01G03470(	2.1895547	5.05E-08	up	yes	.	pfam02536	mTERF	mTERF. Th	.	.	.	.
Manes.01G03480(	0.2773394	4.30E-07	down	yes	Transcription	pfam13921	Myb_DNA-bind_6	Myb-like DI	GO:0009738|	GO:0005634|	GO:0003677|DI	K09422
Manes.01G03490(	4.32333	0.02822	up	yes	Probable calc	pfam13499	EF-hand_7	EF-hand do	.	.	GO:0005509|ca	.
Manes.01G03500(	18.946286	#######	up	yes	Cyclic dof fa	pfam02701	zf-Dof	Dof domain	GO:0009908|	GO:0005634|	GO:0003677|DI	.
Manes.01G03540(	0.5922474	1.30E-05	down	no	Probable dise	pfam00931	NB-ARC	NB-ARC do	GO:0006952|	.	GO:0043531|AI	.
Manes.01G03550(	2.4522228	1.44E-06	up	yes	Disease resist	pfam00931	NB-ARC	NB-ARC do	GO:0006952|	.	GO:0043531|AI	.
Manes.01G03570(	0.681268	0.00079	down	no	Disease resist	pfam00931	NB-ARC	NB-ARC do	GO:0006952|	.	GO:0043531|AI	.
Manes.01G03620(	2.5060817	1.48E-07	up	yes	E3 ubiquitin-	pfam13639	zf-RING_2	Ring finger	GO:0016567|	.	GO:0016874|lig	.
Manes.01G03630(	0.5340527	1.67E-07	down	no	Beta-1,3-gala	pfam01762	Galactosyl_T	Galactosyltr	GO:0010493|	GO:0005794|	GO:0030246|ca	K14413
Manes.01G03690(	1.4669717	3.69E-05	up	no	Protein MAT	pfam00076	RRM_1	RNA recogi	GO:0000398|	.	GO:0000166|nu	.
Manes.01G03720(	0.8229824	0.0469	down	no	MAG2-intera	pfam08314	Sec39	Secretory pa	GO:0051604|	GO:0005789|	.	.
Manes.01G03740(	1.1766528	0.075	up	no	Molybdate-ai	pfam05631	DUF791	Protein of u	GO:0055085|	GO:0016021|	GO:0015098|mo	.
Manes.01G03750(	0.3869678	4.86E-17	down	yes	Rhodanese-lil	pfam00581	Rhodanese	Rhodanese-l	.	GO:0009507|	.	.
Manes.01G03760(	0.9890366	0.92384	down	no	Vacuolar pro	pfam03643	Vps26	Vacuolar pr	GO:0006886|	GO:0005794|	GO:0008565|pr	K18466
Manes.01G03770(	0.8885343	0.22955	down	no	Vacuolar pro	pfam04100	Vps53_N	Vps53-like,	GO:0010286|	GO:0010008|	.	.
Manes.01G03790(	2.9852953	3.34E-13	up	yes	Mitochondria	pfam14363	AAA_assoc	Domain ass	GO:0051131|	GO:0005829|	GO:0005524|A'	K08900
Manes.01G03800(	1.3209977	0.00387	up	no	ATPase famil	pfam00004	AAA	ATPase fam	GO:0031122|	GO:0030054|	GO:0005524|A'	.
Manes.01G03860(	1.1051519	0.28595	up	no	Probable 6-pl	pfam01182	Glucosamine_iso	Glucosamine	GO:0005975|	GO:0009507|	GO:0017057|6-	K01057
Manes.01G03880(	Inf	0.01334	up	yes	.	pfam10093	DUF2331	Uncharacter	.	.	.	.
Manes.01G03890(	2.2779505	0.00041	up	yes	Homeobox-le	pfam00046	Homeobox	Homeobox	GO:0045893|	GO:0005634|	GO:0043565|se	.
Manes.01G03900(	NA	NA	--	no	.	pfam00953	Glycos_transf_4	Glycosyl tra	.	.	.	.
Manes.01G03930(	0.1544592	1.19E-08	down	yes	Histone-lysin	pfam00856	SET	SET domair	GO:0009901|	GO:0005634|	GO:0046976|his	.
Manes.01G04010(	1.1951242	0.05485	up	no	Acyl-CoA-bi	pfam00887	ACBP	Acyl CoA b	GO:0006869|	GO:0005783|	GO:0000062|fa	.
Manes.01G04020(	0.7857571	0.01302	down	no	Importin sub	pfam13513	HEAT_EZ	HEAT-like	GO:0051028|	GO:0005737|	GO:0005525|G'	K14293
Manes.01G04040(	0.8394407	0.07035	down	no	Importin sub	pfam13513	HEAT_EZ	HEAT-like	GO:0006606|	GO:0005737|	GO:0019899|en	K14293
Manes.01G04090(	0.1841108	0.15267	down	no	Probable leuc	pfam13855	LRR_8	Leucine rich	.	GO:0016021|	GO:0005524|A'	.
Manes.01G04120(	4.1268115	4.22E-39	up	yes	Auxin-induce	pfam02519	Auxin_inducible	Auxin respo	GO:0009734|	.	.	.
Manes.01G04170(	0.5202282	0.00029	down	no	Leucine-rich	pfam00069	Pkinase	Protein kina	GO:0006182|	GO:0016021|	GO:0005524|A'	.
Manes.01G04180(	2.1849213	9.21E-08	up	yes	Growth-regu	pfam08879	WRC	WRC. The	GO:0048366|	GO:0005634|	GO:0005524|A'	.
Manes.01G04190(	0.378465	1.41E-11	down	yes	.	pfam13178	DUF4005	Protein of u	.	.	.	.
Manes.01G04210(	0.5654392	0.01997	down	no	.	pfam09446	VMA21	VMA21-like	.	.	.	.
Manes.01G04240(	0.5669536	1.35E-07	down	no	Polyadenylate	pfam00076	RRM_1	RNA recogi	.	GO:0005634|	GO:0000166|nu	.
Manes.01G04260(	0.6958444	0.0003	down	no	Polyadenylate	pfam00076	RRM_1	RNA recogi	.	GO:0005634|	GO:0000166|nu	.
Manes.01G04270(	0.8564863	0.15453	down	no	.	pfam12657	TFIIIC_delta	Transcriptio	.	.	.	.
Manes.01G04300(	1.3847017	0.19105	up	no	StAR-related	pfam14684	Tricorn_C1	Tricorn prot	GO:1903955|	GO:0005739|	GO:0008289|lip	.
Manes.01G04310(	2.0987431	2.59E-16	up	yes	.	pfam14953	DUF4504	Domain of u	.	.	.	.
Manes.01G04320(	23.684842	1.39E-27	up	yes	Two-compor	pfam00072	Response_reg	Response re	GO:0000160|	GO:0005634|	.	K12130
Manes.01G04330(	1.2065796	0.14464	up	no	Protein YLS	pfam03168	LEA_2	Late embryo	GO:0051607|	GO:0009507|	.	.
Manes.01G04340(	2.5660662	6.22E-24	up	yes	.	pfam07223	DUF1421	Protein of u	.	.	.	.
Manes.01G04410(	1.0885671	0.35435	up	no	.	pfam03251	Tymo_45kd_70kd	Tymovirus 4	.	.	.	.
Manes.01G04430(	2.5670589	4.11E-08	up	yes	Heavy metal-	pfam00403	HMA	Heavy-meta	GO:0010286|	GO:0031965|	GO:0046872|me	.
Manes.01G04460(	1.041409	0.67275	up	no	IAA-amino a	pfam01546	Peptidase_M20	Peptidase fa	GO:0009850|	.	GO:0010178|IA	K14664
Manes.01G04470(	2.6517352	5.48E-29	up	yes	NAC domain	pfam02365	NAM	No apical m	GO:0090603|	GO:0005634|	GO:0003677|DI	.
Manes.01G04550(	0.7639491	0.08602	down	no	Probable GA	pfam01490	Aa_trans	Transmemb	GO:0006865|	GO:0016021|	.	.
Manes.01G04590(	0.7425756	0.0151	down	no	.	pfam01576	Myosin_tail_1	Myosin tail.	.	.	.	.
Manes.01G04620(	0.3936259	0.00021	down	yes	Ethylene-resp	pfam00847	AP2	AP2 domair	GO:0048825|	GO:0005737|	GO:0003677|DI	.
Manes.01G04730(	NA	NA	--	no	.	pfam00215	OMPdecase	Orotidine 5'	.	.	.	.
Manes.01G04740(	2.1889924	1.71E-14	up	yes	Hydroquinon	pfam00201	UDPGT	UDP-glucor	.	.	GO:0050505|hy	.
Manes.01G04790(	1.0071655	0.90532	up	no	Protein SPA1	pfam00069	Pkinase	Protein kina	GO:0009585|	GO:0080008|	GO:0005524|A'	.
Manes.01G04810(	0.8750787	0.23764	down	no	Translation ir	pfam00009	GTP_EFTU	Elongation f	.	GO:0005737|	GO:0005525|G'	K02519

Manes.01G04890(	0.8546098	0.14047	down	no	Nucleoside d	pfam00334	NDK	Nucleoside (	GO:0006241|	GO:0009543|	GO:0005524|A'	K00940
Manes.01G04930(	1.0750364	0.55665	up	no	.	pfam00169	PH	PH domain.	.	.	.	.
Manes.01G04970(	1.817311	1.52E-11	up	no	Dynamin-rela	pfam01031	Dynamin_M	Dynamin cer	GO:0007049|	GO:0005874|	GO:0005525|G'	.
Manes.01G04980(	0.7351022	0.0041	down	no	GDSL estera	pfam01764	Lipase_3	Lipase (clas	GO:0016042|	.	GO:0016787|hy	.
Manes.01G04990(	0.4907453	0.00141	down	yes	Calcineurin E	pfam13499	EF-hand_7	EF-hand dor	.	GO:0032578|	GO:0005509|ca	K06268
Manes.01G05020(	0.6722529	4.79E-05	down	no	.	pfam13923	zf-C3HC4_2	Zinc finger,	.	.	.	.
Manes.01G05040(	14.230436	8.02E-11	up	yes	Pathogenesis	pfam00314	Thaumatin	Thaumatin f	GO:0031540|	GO:0048046|	.	.
Manes.01G05050(	2.0484093	7.31E-15	up	yes	Cation/H(+) a	pfam00999	Na_H_Exchanger	Sodium/hyd	GO:0006813|	GO:0016021|	GO:0015299|so	.
Manes.01G05060(	2.1495478	2.23E-05	up	yes	Cation/H(+) a	pfam00999	Na_H_Exchanger	Sodium/hyd	GO:0006813|	GO:0016021|	GO:0015299|so	.
Manes.01G05070(	0.3812546	3.06E-05	down	yes	Disease resist	pfam03018	Dirigent	Dirigent-like	GO:0006952|	GO:0048046|	.	.
Manes.01G05120(	28.142992	1.21E-13	up	yes	.	pfam02517	Abi	CAAX prote	.	.	.	.
Manes.01G05130(	Inf	9.53E-06	up	yes	.	pfam02517	Abi	CAAX prote	.	.	.	.
Manes.01G05140(	0.8145657	0.17288	down	no	Uncharacteriz	pfam00004	AAA	ATPase fam	GO:0031122|	GO:0005741|	GO:0005524|A'	.
Manes.01G05160(	0.839519	0.12129	down	no	GTPase-activ	pfam00566	RabGAP-TBC	Rab-GTPase	.	GO:0005829|	GO:0005096|G'	.
Manes.01G05170(	0.9480912	0.61916	down	no	.	pfam05616	Neisseria_TspB	Neisseria me	.	.	.	.
Manes.01G05190(	5.9885166	2.94E-49	up	yes	Early nodulin	pfam02298	Cu_bind_like	Plastocyanir	.	GO:0031225|	GO:0009055|ele	.
Manes.01G05200(	4.9478592	1.54E-07	up	yes	.	pfam03637	Mob1_phocein	Mob1/phoce	.	.	.	.
Manes.01G05210(	0.8658567	0.17921	down	no	Molybdate-an	pfam05631	DUF791	Protein of u	GO:0055085|	GO:0016021|	GO:0015098|mo	.
Manes.01G05220(	0.5781325	1.59E-07	down	no	.	pfam01500	Keratin_B2	Keratin, hig	.	.	.	.
Manes.01G05230(	0.9599101	0.88446	down	no	.	pfam03706	UPF0104	Uncharacter	.	.	.	.
Manes.01G05280(	3.9787251	1.47E-16	up	yes	Galactose ox	pfam07250	Glyoxal_oxid_N	Glyoxal oxid	.	GO:0005576|	GO:0045480|ga	.
Manes.01G05290(	1.2023933	0.71369	up	no	.	pfam15548	DUF4655	Domain of u	.	.	.	.
Manes.01G05310(	2.4010171	0.04235	up	no	Bidirectional	pfam03083	MtN3_slv	Sugar efflux	GO:0034219|	GO:0005887|	GO:0051119|su	K15382
Manes.01G05330(	0.6889928	0.00016	down	no	Luminal-bind	pfam00012	HSP70	Hsp70 prote	.	GO:0005788|	GO:0005524|A'	.
Manes.01G05360(	0.6791616	0.04747	down	no	Trehalase	pfam01204	Trehalase	Trehalase. T	GO:0005993|	GO:0016021|	GO:0004555|alp	K01194
Manes.01G05370(	0.2748316	1.34E-09	down	yes	Short-chain d	pfam00106	adh_short	short chain (	GO:0015031|	GO:0009507|	GO:0016491|ox	.
Manes.01G05380(	0.8343727	0.09364	down	no	Methyltransfe	pfam09243	Rsm22	Mitochondr	GO:0006412|	GO:0005739|	GO:0005507|co	.
Manes.01G05410(	0.3342981	0.11919	down	no	Transcription	pfam14215	bHLH-MYC_N	bHLH-MYC	GO:0006355|	GO:0005634|	GO:0003677|DI	.
Manes.01G05450(	0.5256645	5.76E-12	down	no	Exocyst com	pfam09749	HVSL	Uncharacter	GO:0048354|	GO:0005618|	.	K06111
Manes.01G05460(	0.5260418	1.96E-10	down	no	Exocyst com	pfam04048	Sec8_exocyst	Sec8 exocys	GO:0048354|	GO:0005618|	.	K06111
Manes.01G05500(	15.823322	1.30E-53	up	yes	.	pfam04490	Pox_T4_C	Poxvirus T4	.	.	.	.
Manes.01G05510(	12.372298	1.16E-87	up	yes	.	pfam03535	Paxillin	Paxillin fami	.	.	.	.
Manes.01G05520(	1.6805087	4.90E-08	up	no	.	pfam09057	Smac_DIABLO	Second Mito	.	.	.	.
Manes.01G05550(	0.8426582	0.086	down	no	Polyadenylate	pfam00076	RRM_1	RNA recogn	.	GO:0005634|	GO:0000166|nu	.
Manes.01G05610(	1.1553093	0.10935	up	no	Ubiquitin carl	pfam00443	UCH	Ubiquitin ca	GO:0043161|	.	GO:0004197|cy	K11835
Manes.01G05650(	0.9629753	0.82346	down	no	Long-chain-fa	pfam11710	Git3	G protein-co	GO:0030497|	GO:0009507|	GO:0008922|lo	K01897
Manes.01G05680(	1.0414223	0.65567	up	no	Receptor pro	pfam00069	Pkinase	Protein kina	GO:0009755|	GO:0005576|	GO:0005524|A'	K00924
Manes.01G05690(	0.8122597	0.07217	down	no	.	pfam10318	7TM_GPCR_Srh	Serpentine t	.	.	.	.
Manes.01G05780(	0.8351931	0.07823	down	no	1-aminocyclo	pfam00155	Aminotran_1_2	Aminotransf	GO:0009693|	.	GO:0016847|1-	K01762
Manes.01G05810(	0.4621501	7.16E-10	down	yes	Probable poly	pfam00295	Glyco_hydro_28	Glycosyl hyd	GO:0005975|	GO:0016021|	GO:0004650|po	.
Manes.01G05830(	0.7426486	0.07996	down	no	.	pfam12680	SnoaL_2	SnoaL-like (	.	.	.	.
Manes.01G05860(	0.6007956	1.95E-06	down	no	.	pfam04646	DUF604	Protein of u	.	.	.	.
Manes.01G05930(	0.346578	7.58E-06	down	yes	Transcription	pfam14215	bHLH-MYC_N	bHLH-MYC	GO:0001708|	GO:0005634|	GO:0003677|DI	.
Manes.01G05950(	0.9872606	0.91612	down	no	Signal peptid	pfam04258	Peptidase_A22B	Signal pepti	.	GO:0005768|	GO:0004190|as	.
Manes.01G05970(	0.5319336	5.61E-10	down	no	Short-chain d	pfam00106	adh_short	short chain (	GO:0015031|	GO:0009706|	GO:0016491|ox	.
Manes.01G05990(	6.3996823	7.58E-32	up	yes	Uncharacteriz	pfam05109	Herpes_BLLF1	Herpes virus	.	GO:0005886|	.	.
Manes.01G06010(	0.7582566	0.02972	down	no	AT-rich inter	pfam01388	ARID	ARID/BRIC	GO:0006355|	GO:0005634|	GO:0003677|DI	.
Manes.01G06020(	0.7504567	0.01551	down	no	E3 ubiquitin-	pfam13639	zf-RING_2	Ring finger (	GO:0016567|	GO:0005737|	GO:0016874|lig	.
Manes.01G06030(	1.7465282	3.25E-10	up	no	.	pfam13867	SAP30_Sin3_bdg	Sin3 binding	.	.	.	.
Manes.01G06050(	7.3503624	1.32E-05	up	yes	Zinc finger pr	pfam06203	CCT	CCT motif.	GO:0010161|	GO:0005634|	GO:0003700|tra	.
Manes.01G06080(	0.323831	0.01362	down	yes	.	pfam00153	Mito_carr	Mitochondri	.	.	.	.
Manes.01G06090(	0.2211861	0.00383	down	yes	.	pfam13650	Asp_protease_2	Aspartyl pro	.	.	.	.
Manes.01G06110(	1.2371219	0.01899	up	no	Lysine-specif	pfam02373	JmjC	JmjC domai	GO:0034720|	GO:0005634|	GO:0003677|DI	K11446
Manes.01G06140(	1.5417957	1.12E-05	up	no	Tubulin beta-	pfam03953	Tubulin_C	Tubulin C-te	GO:0007017|	GO:0005737|	GO:0005525|G'	.
Manes.01G06150(	7.0875065	9.68E-81	up	yes	Glutamate--c	pfam04107	GCS2	Glutamate-c	GO:0006750|	GO:0009507|	GO:0005524|A'	.
Manes.01G06180(	1.9474813	2.04E-10	up	no	.	pfam04032	Rpr2	RNAse P Rp	.	.	.	.
Manes.01G06190(	1.0694924	0.4637	up	no	.	pfam01426	BAH	BAH domai	.	.	.	.
Manes.01G06240(	1.3763845	0.00047	up	no	Probable pho	pfam00255	GSHPx	Glutathione	GO:0046686|	GO:0048046|	GO:0004602|glu	K00432
Manes.01G06260(	0.4632927	2.04E-11	down	yes	Glutathione S	pfam02798	GST_N	Glutathione	GO:0009636|	GO:0005737|	GO:0004364|glu	K00799
Manes.01G06270(	1.9922128	1.71E-12	up	no	Probable cop	pfam00122	E1-E2_ATPase	E1-E2 ATPa	GO:0006825|	GO:0016021|	GO:0005524|A'	K17686
Manes.01G06280(	23.736034	#######	up	yes	Oxalate--CoA	pfam00501	AMP-binding	AMP-bindin	GO:0050832|	GO:0048046|	GO:0005524|A'	.
Manes.01G06290(	Inf	0.04209	up	no	Actin-depoly	pfam00241	Cofilin_ADF	Cofilin/tropo	GO:0030042|	GO:0015629|	.	K05765
Manes.01G06310(	0.6079967	1.19E-05	down	no	Probable LRI	pfam07714	Pkinase_Tyr	Protein tyro	.	GO:0016021|	GO:0005524|A'	.
Manes.01G06330(	0.2906372	9.14E-09	down	yes	O-glucosyltra	pfam05686	Glyco_transf_90	Glycosyl tra	GO:0007030|	GO:0012505|	GO:0046527|glu	.
Manes.01G06360(	0.7424059	0.00276	down	no	.	pfam01576	Myosin_tail_1	Myosin tail.	.	.	.	.
Manes.01G06460(	0.8036036	0.0675	down	no	General trans	pfam02186	TFIIE_beta	TFIIE beta s	GO:0006355|	GO:0005673|	GO:0003677|DI	K03137
Manes.01G06500(	4.4100479	6.90E-48	up	yes	Myb-related p	pfam00249	Myb_DNA-bindin	Myb-like DI	.	GO:0005634|	GO:0003677|DI	.
Manes.01G06520(	0.2673518	1.19E-25	down	yes	Heat stress tr	pfam00447	HSF_DNA-bind	HSF-type D	GO:0045892|	GO:0005634|	GO:0043565|se	K09419
Manes.01G06530(	1.8600664	0.3466	up	no	BON1-associ	pfam00168	C2	C2 domain.	GO:0006952|	GO:0016020|	.	.
Manes.01G06540(	0.8709545	0.19333	down	no	.	pfam05664	DUF810	Protein of u	.	.	.	.
Manes.01G06570(	0.3974092	1.01E-06	down	yes	Pentatricope	pfam13041	PPR_2	PPR repeat	GO:0031425|	GO:0009507|	.	.
Manes.01G06590(	7.7060116	1.00E-71	up	yes	Serine/threon	pfam07714	Pkinase_Tyr	Protein tyro	GO:0071281|	GO:0005789|	GO:0005524|A'	K14510
Manes.01G06600(	0.5501541	0.00053	down	no	Protein HAP	pfam10699	HAP2-GCS1	Male gamete	GO:0048235|	GO:0005783|	.	.
Manes.01G06610(	0.6722289	0.00065	down	no	Phosphatidyli	pfam06728	PIG-U	GPI transam	GO:0006506|	GO:0042765|	.	K05293
Manes.01G06620(	1.2361823	0.01876	up	no	.	pfam00789	UBX	UBX domai	.	.	.	.
Manes.01G06680(	0.896254	0.32515	down	no	Lysophospho	pfam03062	MBOAT	MBOAT, m	GO:0019375|	GO:0005783|	GO:0047184|1-	K13519
Manes.01G06690(	0.9542208	0.73751	down	no	BAG family r	pfam00240	ubiquitin	Ubiquitin fa	.	GO:0005739|	.	.
Manes.01G06770(	0.4175611	0.00073	down	yes	.	pfam14309	DUF4378	Domain of u	.	.	.	.
Manes.01G06800(	0.8401174	0.19393	down	no	.	pfam00638	Ran_BP1	RanBP1 dor	.	.	.	.
Manes.01G06810(	0.2845167	6.83E-24	down	yes	Calcium-depe	pfam00069	Pkinase	Protein kina	GO:0009738|	GO:0005737|	GO:0005524|A'	K13412
Manes.01G06840(	1.516987	6.37E-06	up	no	SPX domain-	pfam03105	SPX	SPX domain	GO:0055085|	GO:0016021|	.	.
Manes.01G06880(	0.1737737	2.61E-13	down	yes	Molybdenum	pfam12252	SidE	Dot/Icm sub	GO:0006777|	.	GO:0016829|lya	K15631
Manes.01G06970(	2.3619779	0.07269	up	no	Protein STAY	pfam12638	Staygreen	Staygreen p	GO:0015996|	GO:0009535|	.	.
Manes.01G07050(	46.286709	4.99E-08	up	yes	Subtilisin-like	pfam00082	Peptidase_S8	Subtilase fa	GO:0042127|	GO:0048046|	GO:0004252|se	.
Manes.01G07080(	7.41476	6.48E-29	up	yes	Growth-regu	pfam08879	WRC	WRC. The V	GO:0032502|	GO:0005634|	GO:0005524|A'	.
Manes.01G07120(	0.8274999	0.14671	down	no	Probable sod	pfam01758	SBF	Sodium Bile	GO:0006810|	GO:0009941|	.	.
Manes.01G07140(	1.192843	0.06532	up	no	Zinc finger A	pfam01754	zf-A20	A20-like zin	.	GO:0009506|	GO:0003677|DI	.
Manes.01G07150(	1.0752165	0.46223	up	no	Armadillo rep	pfam00225	Kinesin	Kinesin mot	GO:0007018|	GO:0005737|	GO:0005524|A'	.

Manes.01G07170(	0.3497755	8.09E-09	down	yes	.	pfam01842	ACT	ACT domai	.	.	.	.
Manes.01G07210(	0.8783866	0.17948	down	no	Methylsterol	pfam04116	FA_hydroxylase	Fatty acid h	GO:0080064|	GO:0005789|	GO:0000254|C-	K14423
Manes.01G07250(	Inf	9.24E-05	up	yes	SNARE-inter	pfam04790	Sarcoglycan_1	Sarcoglycan	GO:0007049|	GO:0005856|	.	K15292
Manes.01G07260(	Inf	0.01269	up	yes	.	pfam11945	WASH_WAHD	WAHD don	.	.	.	.
Manes.01G07340(	0.9096088	0.3434	down	no	.	pfam10248	Mlf1IP	Myelodyspl	.	.	.	.
Manes.01G07370(	0.6342023	4.06E-05	down	no	Putative trans	pfam01571	GCV_T	Aminometh	GO:0016226|	GO:0005759|	GO:0016740|tra	.
Manes.01G07460(	1.0608794	0.85993	up	no	Lipid transfer	pfam14368	LTP_2	Probable lip	GO:0006810|	GO:0031225|	GO:0008289|lip	.
Manes.01G07470(	0.8741344	0.1513	down	no	.	pfam05536	Neurochondrin	Neurochond	.	.	.	.
Manes.01G07490(	0.5475358	0.00014	down	no	21 kDa prote	pfam04043	PMEI	Plant inverta	.	.	GO:0004857|en	.
Manes.01G07500(	0.8948436	0.25948	down	no	Elongation fa	pfam00009	GTP_EFTU	Elongation f	.	GO:0009507|	GO:0005525|G	K02355
Manes.01G07510(	0.5852632	6.40E-07	down	no	Hsp70-Hsp9(	pfam13414	TPR_11	TPR repeat.	GO:0051131|	GO:0005737|	GO:0051879|H	K09553
Manes.01G07560(	NA	NA	--	no	Probable pec	pfam01095	Pectinesterase	Pectinestera	GO:0042545|	GO:0005618|	GO:0045330|as	.
Manes.01G07580(	12.759453	0.01084	up	yes	.	pfam10104	Brr6_like_C_C	Di-sulfide br	.	.	.	.
Manes.01G07600(	2.8997973	6.73E-05	up	yes	NAC domain	pfam02365	NAM	No apical m	GO:0071555|	GO:0005634|	GO:0043565|se	.
Manes.01G07640(	1.0490161	0.65933	up	no	Monocopper	pfam00394	Cu-oxidase	Multicopper	GO:0009932|	GO:0031225|	GO:0005507|co	.
Manes.01G07660(	1.0522871	0.58204	up	no	Transaldolase	pfam00923	Transaldolase	Transaldola	GO:0005975|	GO:0005737|	GO:0004801|se	K00616
Manes.01G07700(	0.8168652	0.04525	down	no	Flavin-contai	pfam00743	FMO-like	Flavin-bindi	.	GO:0005773|	GO:0050660|fla	.
Manes.01G07730(	0.6238991	0.14298	down	no	Probable WR	pfam03106	WRKY	WRKY DN	GO:0042742|	GO:0005634|	GO:0005516|ca	.
Manes.01G07740(	0.3258614	2.01E-07	down	yes	Adenine phos	pfam00156	Pribosyltran	Phosphoribo	GO:0006168|	GO:0005737|	GO:0003999|ad	K00759
Manes.01G07750(	0.6338333	0.0149	down	no	Probable lipi	pfam01569	PAP2	PAP2 super	.	GO:0016021|	GO:0016787|hy	.
Manes.01G07860(	Inf	0.1623	up	no	14 kDa proli	pfam14547	Hydrophob_seed	Hydrophobi	.	GO:0016021|	.	.
Manes.01G07950(	0.8503064	0.12912	down	no	.	pfam03253	UT	Urea transp	.	.	.	.
Manes.01G07960(	1.8638125	3.24E-07	up	no	.	pfam10197	Cir_N	N-terminal c	.	.	.	.
Manes.01G07970(	0.8229836	0.08614	down	no	.	pfam13041	PPR_2	PPR repeat	.	.	.	.
Manes.01G08000(	0.5727837	0.00063	down	no	Phosphatidyli	pfam00650	CRAL_TRIO	CRAL/TRIC	GO:0015031|	GO:0000139|	.	.
Manes.01G08030(	0.6875898	0.00151	down	no	Chaperone p	pfam11875	DUF3395	Domain of u	GO:0055122|	GO:0016021|	.	K09531
Manes.01G08040(	0.5311834	0.00419	down	no	.	pfam15244	HSD3	Hydroxy-ste	.	.	.	.
Manes.01G08050(	Inf	0.35135	up	no	Protein EXO	pfam04674	Phi_1	Phosphate-i	.	GO:0048046|	.	.
Manes.01G08070(	0.8904664	0.3509	down	no	.	pfam12874	zf-met	Zinc-finger c	.	.	.	.
Manes.01G08080(	1.4590008	0.00021	up	no	Asparagine--	pfam00152	tRNA-synt_2	tRNA synth	GO:0006421|	GO:0009507|	GO:0004816|as	K01893
Manes.01G08100(	0.598107	0.00156	down	no	Zinc finger p	pfam13912	zf-C2H2_6	C2H2-type	GO:0006355|	GO:0005634|	GO:0046872|m	.
Manes.01G08110(	8.0929132	0.00027	up	yes	WAT1-relate	pfam00892	EamA	EamA-like t	.	GO:0016021|	GO:0022857|tra	.
Manes.01G08120(	2.3116124	4.77E-09	up	yes	.	pfam12225	MTHFR_C	Methylene-t	.	.	.	.
Manes.01G08140(	1.0210856	0.80739	up	no	.	pfam05641	Agenet	Agenet dom	.	.	.	.
Manes.01G08150(	0.7322516	0.00176	down	no	.	pfam04784	DUF547	Protein of u	.	.	.	.
Manes.01G08180(	0.6805943	0.00101	down	no	.	pfam05703	Auxin_canalis	Auxin canal	.	.	.	.
Manes.01G08200(	0.3632656	0.00042	down	yes	.	pfam13301	DUF4079	Protein of u	.	.	.	.
Manes.01G08220(	1.1188132	0.30863	up	no	Phosphatidyl	pfam02666	PS_Dcarbxylase	Phosphatidy	GO:0006646|	GO:0005743|	GO:0004609|ph	K01613
Manes.01G08240(	0.2903694	1.51E-05	down	yes	DNA mismat	pfam00488	MutS_V	MutS doma	GO:0051026|	GO:0000794|	GO:0005524|A	K08740
Manes.01G08290(	0.8724038	0.61311	down	no	Probable glyc	pfam03016	Exostosin	Exostosin fa	GO:0071555|	GO:0000139|	GO:0016757|tra	.
Manes.01G08350(	Inf	2.44E-10	up	yes	.	pfam09742	Dymeclin	Dyggve-Me	.	.	.	.
Manes.01G08360(	11.766235	2.70E-11	up	yes	Alcohol dehy	pfam08240	ADH_N	Alcohol deh	.	GO:0005737|	GO:0004022|alc	.
Manes.01G08380(	0.7882356	0.07302	down	no	.	pfam10250	O-FucT	GDP-fucose	.	.	.	.
Manes.01G08410(	0.8119476	0.14665	down	no	1-aminocyclo	pfam14226	DIOX_N	non-haem di	GO:0009693|	.	GO:0009815|1-	.
Manes.01G08430(	2.5286426	1.22E-21	up	yes	.	pfam07797	DUF1639	Protein of u	.	.	.	.
Manes.01G08460(	0.8790258	0.25284	down	no	Putative dual	pfam00782	DSPc	Dual specifi	.	.	GO:0004725|pr	.
Manes.01G08470(	0.682132	0.00013	down	no	Palmitoyl-pro	pfam02089	Palm_thioest	Palmitoyl pr	GO:0044257|	GO:0005764|	GO:0008474|pa	K01074
Manes.01G08490(	0.7178809	0.00617	down	no	DeSI-like pro	pfam05903	Peptidase_C97	PPPDE puta	.	.	GO:0008233|pe	.
Manes.01G08510(	0.1879246	0.0001	down	yes	Ethylene-resp	pfam00847	AP2	AP2 domair	GO:0051301|	GO:0005634|	GO:0003677|D	K09286
Manes.01G08520(	6.0098991	2.56E-29	up	yes	Ethylene-resp	pfam00847	AP2	AP2 domair	GO:0006952|	GO:0005634|	GO:0003677|D	K09286
Manes.01G08540(	148.1738	#######	up	yes	Ethylene-resp	pfam00847	AP2	AP2 domair	GO:0051301|	GO:0005634|	GO:0003677|D	K09286
Manes.01G08560(	47.982211	2.79E-09	up	yes	G-type lectin	pfam00069	Pkinase	Protein kina	.	GO:0016021|	GO:0005524|A	.
Manes.01G08570(	2.4193446	1.86E-08	up	yes	Ubiquitin car	pfam01088	Peptidase_C12	Ubiquitin ca	GO:0016579|	GO:0005737|	GO:0004843|ub	K05609
Manes.01G08580(	3.4606262	6.04E-06	up	yes	AP2/ERF anc	pfam02362	B3	B3 DNA bir	GO:0009873|	GO:0005634|	GO:0003677|D	K09287
Manes.01G08610(	3.0760513	5.95E-10	up	yes	Phospholipas	pfam01764	Lipase_3	Lipase (clas	GO:0016042|	GO:0009507|	GO:0047714|ga	.
Manes.01G08640(	1.8338304	7.12E-10	up	no	GTP-binding	pfam00071	Ras	Ras family.	GO:0015031|	GO:0005622|	GO:0005525|G	K07874
Manes.01G08650(	0.461632	0.00062	down	yes	.	pfam09480	PrgH	Type III sec	.	.	.	.
Manes.01G08660(	0.3972046	0.00244	down	yes	Hyoscyamine	pfam14226	DIOX_N	non-haem di	.	.	GO:0046872|m	K06892
Manes.01G08680(	1.3789258	0.04022	up	no	.	pfam10401	IRF-3	Interferon-r	.	.	.	.
Manes.01G08690(	0.9442326	0.89419	down	no	Putative glyc	pfam07690	MFS_1	Major Facili	GO:0006820|	GO:0016021|	GO:0015315|or	K13783
Manes.01G08700(	0.8642842	0.18216	down	no	50S ribosom	pfam01245	Ribosomal_L19	Ribosomal p	GO:0006412|	GO:0009507|	GO:0019843|r	K02884
Manes.01G08710(	1.6225991	0.02959	up	no	.	pfam06432	GPI2	Phosphatidy	.	.	.	.
Manes.01G08740(	2.3505533	6.10E-22	up	yes	Bax inhibitor	pfam01027	Bax1-I	Inhibitor of	GO:0006983|	GO:0005737|	.	.
Manes.01G08760(	0.8963874	0.53042	down	no	.	pfam06317	Arena_RNA_pol	Arenavirus	.	.	.	.
Manes.01G08770(	0.6893683	0.0003	down	no	Histone-lysin	pfam02182	SAD_SRA	SAD/SRA c	GO:0016571|	GO:0000775|	GO:0018024|his	K11420
Manes.01G08790(	0.8046201	0.02475	down	no	ATP-depend	pfam00574	CLP_protease	Clp protease	GO:0010468|	GO:0009507|	GO:0004252|se	K01358
Manes.01G08850(	0.7916019	0.06953	down	no	.	pfam00504	Chloroa_b-bind	Chlorophyll	.	.	.	.
Manes.01G08870(	6.0490563	2.30E-24	up	yes	Subtilisin-like	pfam00082	Peptidase_S8	Subtilase fa	.	GO:0005618|	GO:0004252|se	.
Manes.01G08920(	0.4118906	7.08E-05	down	yes	Putative gluc	pfam01263	Aldose_epim	Aldose 1-ep	GO:0005975|	.	GO:0030246|ca	.
Manes.01G08950(	Inf	0.08543	up	no	Germin-like	pfam00190	Cupin_1	Cupin. This	.	GO:0048046|	GO:0030145|m	.
Manes.01G08960(	0.6567027	8.34E-05	down	no	Phospho-2-d	pfam01474	DAHP_synth_2	Class-II DA	GO:0009073|	GO:0009507|	GO:0003849|3-	K01626
Manes.01G08980(	1.0640404	0.55686	up	no	.	pfam00160	Pro_isomerase	Cyclophilin	.	.	.	.
Manes.01G09010(	1.5776982	1.01E-05	up	no	ATP-depend	pfam00271	Helicase_C	Helicase co	GO:0070827|	GO:0045025|	GO:0034458|3'	K17675
Manes.01G09020(	0.6190011	8.78E-06	down	no	Protease Do-	pfam13365	Trypsin_2	Trypsin-like	GO:0010206|	GO:0009507|	GO:0008233|pe	.
Manes.01G09030(	1.0173455	0.99987	up	no	Protein FEZ	pfam02365	NAM	No apical m	GO:0006355|	GO:0005634|	GO:0003677|D	.
Manes.01G09040(	0.8502836	0.10356	down	no	60S ribosom	pfam01199	Ribosomal_L34e	Ribosomal p	GO:0006412|	GO:0005840|	GO:0003735|str	.
Manes.01G09070(	2.393756	9.01E-23	up	yes	Myb-related	pfam00249	Myb_DNA-binding	Myb-like D	GO:0006355|	GO:0005634|	GO:0003677|D	.
Manes.01G09110(	0.7374682	0.00489	down	no	Gibberellin-r	pfam02704	GASA	Gibberellin	GO:0045454|	GO:0005623|	.	.
Manes.01G09140(	0.8377383	0.4729	down	no	Vacuolar ami	pfam01490	Aa_trans	Transmemb	GO:0003333|	GO:0000324|	GO:0015186|L-	K15015
Manes.01G09150(	0.2030666	2.65E-06	down	yes	.	pfam13041	PPR_2	PPR repeat	.	.	.	.
Manes.01G09170(	0.4886706	0.04013	down	no	Granule-bour	pfam08323	Glyco_transf_5	Starch synth	GO:0010021|	GO:0009501|	GO:0004373|gl	K00703
Manes.01G09200(	0.9076708	0.62351	down	no	Uncharacteri	pfam08881	CVNH	CVNH dom	GO:0006412|	GO:0016021|	GO:0003735|str	.
Manes.01G09210(	7.7870018	#######	up	yes	Cyclic dof fa	pfam02701	zf-Dof	Dof domain	GO:0009908|	GO:0005634|	GO:0003677|D	.
Manes.01G09230(	5.0447955	3.31E-10	up	yes	Probable calc	pfam13499	EF-hand_7	EF-hand do	.	.	GO:0005509|ca	.
Manes.01G09260(	0.2512202	9.52E-16	down	yes	Expansin-A1	pfam01357	Pollen_allerg_1	Pollen allerg	GO:0009664|	GO:0005618|	.	.
Manes.01G09310(	0.3766373	0.00259	down	yes	.	pfam13041	PPR_2	PPR repeat	.	.	.	.
Manes.01G09320(	1.0262376	0.99145	up	no	NAC domain	pfam02365	NAM	No apical m	GO:0006351|	GO:0005634|	GO:0003677|D	.
Manes.01G09340(	0.9631261	0.71192	down	no	Cystathionine	pfam01053	Cys_Met_Meta_P	Cys/Met me	GO:0009086|	GO:0009507|	GO:0003962|cy	K01739

Manes.01G09420(	0.3687966	6.91E-11	down	yes	Transcription	pfam03634	TCP	TCP family	GO:0030154|(	GO:0009507|(	GO:0003677|D	.
Manes.01G09430(	1.3748263	0.00123	up	no	Transcription	pfam07524	Bromo_TP	Bromodoma	GO:0045893|(	GO:0005669|(	.	K14649
Manes.01G09440(	2.5558211	5.20E-07	up	yes	.	pfam00844	Gemini_coat	Geminivirus	.	.	.	.
Manes.01G09530(	0.2946648	2.29E-09	down	yes	Phenolic gluc	pfam02458	Transferase	Transferase	GO:0009636|r	GO:0005829|(	GO:0050736|O-	.
Manes.01G09540(	0.8005529	1	down	no	Phenolic gluc	pfam02458	Transferase	Transferase	GO:0009636|r	GO:0005829|(	GO:0050736|O-	.
Manes.01G09590(	1.1096054	0.23898	up	no	Serine carbox	pfam00450	Peptidase_S10	Serine carbo	.	GO:0005576|(	GO:0004185|se	K16297
Manes.01G09610(	1.820729	8.12E-11	up	no	.	pfam12095	DUF3571	Protein of u	.	.	.	.
Manes.01G09660(	0.2159073	5.37E-08	down	yes	UDP-glycosy	pfam00201	UDPGT	UDP-glucor	.	.	GO:0016758|tra	.
Manes.01G09720(	5.8041521	2.55E-57	up	yes	.	pfam12119	DUF3581	Protein of u	.	.	.	.
Manes.01G09730(	0.2803247	0.0003	down	yes	.	pfam12937	F-box-like	F-box-like.	.	.	.	.
Manes.01G09750(	1.973396	1.42E-06	up	no	Biotin carbox	pfam00364	Biotin_lipoyl	Biotin-requi	GO:0006633|f	GO:0009317|(	GO:0003989|ac	K02160
Manes.01G09790(	2.6108614	2.29E-16	up	yes	UDP-glucose	pfam03721	UDPG_MGDP_dl	UDP-glucos	GO:0006065|U	.	GO:0051287|N.	K00012
Manes.01G09850(	0.6108405	0.00111	down	no	Membrane-an	pfam13881	Rad60-SLD_2	Ubiquitin-2	.	GO:0005886|(	.	.
Manes.01G09860(	0.789996	0.01397	down	no	Chaperone pr	pfam07724	AAA_2	AAA domai	GO:0009658|(	GO:0009507|(	GO:0005524|A'	K03695
Manes.01G09870(	0.7226407	0.06774	down	no	.	pfam03055	RPE65	Retinal pign	.	.	.	.
Manes.01G09880(	0.6585336	0.00016	down	no	.	pfam12937	F-box-like	F-box-like.	.	.	.	.
Manes.01G09900(	2.5541104	1.94E-26	up	yes	.	pfam07839	CaM_binding	Plant calmo	.	.	.	.
Manes.01G09910(	0.9812136	0.84172	down	no	.	pfam14234	DUF4336	Domain of u	.	.	.	.
Manes.01G09920(	0.9062741	0.33395	down	no	.	pfam13445	zf-RING_UBOX	RING-type	.	.	.	.
Manes.01G09930(	0.3286948	4.46E-09	down	yes	Cyclic nucleo	pfam00027	cNMP_binding	Cyclic nucle	GO:0070588|(	GO:0005887|(	GO:0005262|ca	K05391
Manes.01G09950(	1.0016461	0.95329	up	no	Probable mito	pfam03435	Saccharop_dh	Saccharopin	.	GO:0009941|(	GO:0016491|ox	.
Manes.01G09980(	0.4625653	0.00022	down	yes	.	pfam13041	PPR_2	PPR repeat	.	.	.	.
Manes.01G10000(	1.7402634	6.47E-06	up	no	Lamin-like pr	pfam02298	Cu_bind_like	Plastocyanin	.	GO:0031225|(	GO:0009055|ele	.
Manes.01G10010(	1.8102512	1.09E-07	up	no	U3 small nuc	pfam01479	S4	S4 domain.	GO:0006364|r	GO:0034457|(	GO:0044822|po	K14560
Manes.01G10020(	7.7697788	8.36E-18	up	yes	.	pfam06836	DUF1240	Protein of u	.	.	.	.
Manes.01G10070(	1.2073471	0.24456	up	no	.	pfam07059	DUF1336	Protein of u	.	.	.	.
Manes.01G10100(	19.800997	0.00028	up	yes	Ethylene-resp	pfam04954	SIP	Siderophore	GO:0035865|(	GO:0005634|(	GO:0043565|se	K09286
Manes.01G10200(	0.364065	3.01E-07	down	yes	.	pfam05498	RALF	Rapid ALka	.	.	.	.
Manes.01G10230(	0.2606716	1.38E-09	down	yes	.	pfam12842	DUF3819	Domain of u	.	.	.	.
Manes.01G10260(	0.3677562	0.13709	down	no	PI-PLC X do	pfam01683	EB	EB module.	GO:0006629|l	GO:0046658|(	GO:0008081|ph	.
Manes.01G10280(	0.2623996	6.02E-07	down	yes	Cytochrome	pfam00067	p450	Cytochrome	.	GO:0016021|(	GO:0020037|he	.
Manes.01G10300(	1.1834694	0.08248	up	no	Probable tRN	pfam02005	TRM	N2,N2-dime	.	.	GO:0004809|tR	K00555
Manes.01G10330(	0.710902	0.0102	down	no	Ultraviolet-B	pfam00415	RCC1	Regulator o	GO:0009649|(	GO:0000785|(	GO:0003682|ch	.
Manes.01G10410(	0.7191605	0.02611	down	no	Uncharacteriz	pfam10250	O-FucT	GDP-fucose	.	GO:0005768|(	GO:0016757|tra	.
Manes.01G10430(	0.4256686	5.18E-14	down	yes	Phosphatidyli	pfam00454	PI3_PI4_kinase	Phosphatidy	GO:0046854|(	.	GO:0004430|1-	.
Manes.01G10440(	0.3994858	8.95E-13	down	yes	Probable leuc	pfam07714	Pkinase_Tyr	Protein tyro	GO:0009755|l	GO:0016021|(	GO:0005524|A'	.
Manes.01G10470(	1.1537009	0.1229	up	no	Alpha/beta hy	pfam12695	Abhydrolase_5	Alpha/beta l	.	GO:0005576|(	GO:0016787|hy	.
Manes.01G10500(	1.3438462	0.00184	up	no	Probable sod	pfam01490	Aa_trans	Transmemb	GO:0003333|a	GO:0005887|(	GO:0015171|an	K14993
Manes.01G10520(	21.21994	0.00036	up	yes	.	pfam00001	7tm_1	7 transmeml	.	.	.	.
Manes.01G10560(	0.692583	0.00047	down	no	6-phosphoglu	pfam00393	6PGD	6-phosphog	GO:0019521|(	GO:0009570|(	GO:0050661|N.	K00033
Manes.01G10580(	5.3578482	1.50E-08	up	yes	Protein GLU	pfam13877	RPAP3_C	Potential Mo	GO:0006865|a	GO:0016021|(	.	.
Manes.01G10590(	5.4159477	3.37E-22	up	yes	Protein GLU	pfam14221	DUF4330	Domain of u	GO:0006865|a	GO:0016021|(	.	.
Manes.01G10600(	0.8665271	0.14716	down	no	Biogenesis of	pfam10046	BLOC1_2	Biogenesis c	.	GO:0031083|(	.	K16750
Manes.01G10620(	1.966314	2.01E-14	up	no	Zinc finger pr	pfam06203	CCT	CCT motif.	GO:0009658|(	GO:0005634|(	GO:0003677|D	.
Manes.01G10630(	0.6057964	1.27E-06	down	no	Protein NSP-	pfam00069	Pkinase	Protein kina	GO:0051607|(	GO:0016021|(	GO:0005524|A'	.
Manes.01G10660(	13.381525	#######	up	yes	S-adenosylme	pfam01536	SAM_decarbox	Adenosylme	GO:0006557|S	.	GO:0004014|ad	.
Manes.01G10670(	16.494022	3.01E-66	up	yes	.	pfam08132	AdoMetDC_leade	S-adenosyl-	.	.	.	.
Manes.01G10680(	1.0288584	0.7805	up	no	EVI5-like pro	pfam00566	RabGAP-TBC	Rab-GTPase	GO:1902018|r	.	GO:0005096|G'	.
Manes.01G10690(	0.2289037	1.94E-10	down	yes	.	pfam14432	DYW_deaminase	DYW family	.	.	.	.
Manes.01G10720(	0.6424939	0.00054	down	no	.	pfam06162	DUF976	Caenorhabd	.	.	.	.
Manes.01G10750(	1.2710088	0.00929	up	no	Histone acety	pfam08214	KAT11	Histone ace	GO:0009908|(	GO:0005634|(	GO:0004402|his	.
Manes.01G10820(	4.5109069	0.00211	up	yes	.	pfam00421	PSII	Photosysten	.	.	.	.
Manes.01G10830(	0.2805202	1.33E-23	down	yes	.	pfam12780	AAA_8	P-loop conta	.	.	.	.
Manes.01G10850(	1.0375862	0.74564	up	no	Cell division	pfam10497	zf-4CXXC_R1	Zinc-finger	GO:0008284|(	GO:0005737|(	.	.
Manes.01G10970(	1.0118882	0.88464	up	no	Probable doli	pfam03155	Alg6_Alg8	ALG6, ALG	GO:0006486|(	GO:0005789|(	GO:0016758|tra	K03848
Manes.01G11010(	14.975364	0.00107	up	yes	.	pfam04388	Hamartin	Hamartin pr	.	.	.	.
Manes.01G11020(	2.0784966	0.0003	up	yes	.	pfam14727	PHTB1_N	PTHB1 N-te	.	.	.	.
Manes.01G11030(	3.7379792	6.08E-37	up	yes	.	pfam15255	CAP-ZIP_m	WASH com	.	.	.	.
Manes.01G11040(	1.1199245	0.2577	up	no	Caffeoylshiki	pfam12697	Abhydrolase_6	Alpha/beta l	GO:0009809|l	GO:0005886|(	GO:0003846|2-	K18368
Manes.01G11050(	0.3581071	1.28E-13	down	yes	Caffeoylshiki	pfam12697	Abhydrolase_6	Alpha/beta l	GO:0009809|l	GO:0005886|(	GO:0003846|2-	K18368
Manes.01G11080(	1.2888904	0.03694	up	no	Mannose-6-p	pfam01238	PMI_typeI	Phosphoma	GO:0009298|(	.	GO:0004476|ma	K01809
Manes.01G11090(	2.7504553	2.57E-20	up	yes	.	pfam13398	Peptidase_M50B	Peptidase M	.	.	.	.
Manes.01G11100(	3.3943537	4.08E-19	up	yes	.	pfam03125	Sre	C. elegans S	.	.	.	.
Manes.01G11120(	0.6209417	1.33E-06	down	no	.	pfam14309	DUF4378	Domain of u	.	.	.	.
Manes.01G11130(	2.8067772	2.58E-08	up	yes	.	pfam06391	MAT1	CDK-activa	.	.	.	.
Manes.01G11170(	0.5489222	8.23E-07	down	no	Plastidic gluc	pfam00083	Sugar_tr	Sugar (and	GO:0046323|g	GO:0009941|(	GO:0005355|glu	.
Manes.01G11180(	0.9072672	0.321	down	no	Delta(7)-ster	pfam04116	FA_hydroxylase	Fatty acid h	GO:0006633|f	GO:0005789|(	GO:0005506|iro	.
Manes.01G11250(	1.6786595	0.13495	up	no	Probable non	pfam14368	LTP_2	Probable lip	GO:0006810|t	.	GO:0008289|lip	.
Manes.01G11270(	0.5463286	4.44E-09	down	no	Probable non	pfam00234	Tryp_alpha_amyl	Protease inh	GO:0006810|t	.	GO:0008289|lip	.
Manes.01G11290(	0.701498	0.00048	down	no	.	pfam01576	Myosin_tail_1	Myosin tail.	.	.	.	.
Manes.01G11300(	3.4134318	0.0009	up	yes	Probable inac	pfam00069	Pkinase	Protein kina	.	GO:0016021|(	GO:0005524|A'	.
Manes.01G11310(	45.071374	#######	up	yes	.	pfam10344	Fmp27	Mitochondri	.	.	.	.
Manes.01G11340(	3.4705509	4.97E-05	up	yes	.	.	.	.	.	.	.	.
Manes.01G11370(	1.4021674	0.00024	up	no	2-hydroxyacy	pfam00205	TPP_enzyme_M	Thiamine py	.	GO:0005829|(	GO:0016831|ca	K12261
Manes.01G11380(	1.6430621	1.96E-05	up	no	.	pfam12874	zf-met	Zinc-finger	.	.	.	.
Manes.01G11410(	0.7832714	0.04564	down	no	Gamma-inter	pfam03227	GILT	Gamma inte	GO:0042590|a	GO:0005576|(	GO:0016667|ox	K08059
Manes.01G11420(	7.6683479	1.48E-08	up	yes	Probable S-a	pfam03492	Methyltransf_7	SAM depen	.	.	GO:0008168|me	.
Manes.01G11440(	Inf	0.08944	up	no	.	pfam07128	DUF1380	Protein of u	.	.	.	.
Manes.01G11570(	0.591414	0.00259	down	no	.	pfam05090	VKG_Carbox	Vitamin K-c	.	.	.	.
Manes.01G11610(	0.9215698	0.43959	down	no	Double-strand	pfam00035	dsrm	Double-stra	.	.	GO:0003725|do	.
Manes.01G11620(	1.9890258	1.57E-12	up	no	Pentatricope	pfam13041	PPR_2	PPR repeat	.	GO:0005739|(	.	.
Manes.01G11660(	1.3573864	0.00079	up	no	Probable fruc	pfam00294	PfkB	pfkB family	GO:0006014|(	GO:0009507|(	GO:0005524|A'	K00847
Manes.01G11670(	13.16998	#######	up	yes	Probable calc	pfam13499	EF-hand_7	EF-hand do	GO:0009909|r	.	GO:0005509|ca	K13448
Manes.01G11680(	9.8336814	0.00659	up	yes	GDP-L-galac	pfam14895	PPPI_inhib	Protein pho	GO:0052544|(	GO:0005737|(	GO:0010475|ga	K14190
Manes.01G11690(	2.5220334	4.63E-15	up	yes	Probable AD	pfam00168	C2	C2 domain.	.	.	GO:0005096|G'	K12486
Manes.01G11710(	0.1082566	0.001	down	yes	Transcription	pfam00249	Myb_DNA-binding	Myb-like DI	GO:0007275|(	GO:0005634|(	GO:0003677|D	K09422
Manes.01G11800(	1.9616946	2.13E-14	up	no	.	pfam04576	Zein-binding	Zein-binding	.	.	.	.
Manes.01G11810(	1.5042529	4.82E-05	up	no	Aldose 1-epi	pfam01263	Aldose_epim	Aldose 1-ep	GO:0006012|(	GO:0005737|(	GO:0004034|ald	K01785

Manes.01G11840(	0.408941	5.98E-11	down	yes	PsbQ-like pro	pfam05757	PsbQ	Oxygen evo	GO:0009767|	GO:0009535|	GO:0005509|ca	.
Manes.01G11870(	2.567638	1.07E-11	up	yes	Protein ODO	pfam00249	Myb_DNA-binding	Myb-like DI	GO:0006355|	GO:0005634|	GO:0003677|DI	.
Manes.01G11890(	0.9237302	0.4487	down	no	Probable inac	pfam00069	Pkinase	Protein kina	GO:0009755|	GO:0016021|	GO:0005524|A'	.
Manes.01G11910(	0.5690635	0.00422	down	no	.	pfam03193	DUF258	Protein of u	.	.	.	.
Manes.01G11920(	0.2008314	5.53E-06	down	yes	.	pfam14897	EpsG	EpsG family	.	.	.	.
Manes.01G11930(	0.2552414	4.75E-16	down	yes	Cysteine-rich	pfam01657	Stress-antifung	Salt stress re	GO:0006810|	GO:0016021|	.	.
Manes.01G11940(	2.0423114	3.55E-12	up	yes	Nudix hydrol	pfam00293	NUDIX	NUDIX dor	.	GO:0009507|	GO:0016787|hy	K07766
Manes.01G11960(	1.0778642	0.51824	up	no	Kinesin light	pfam13424	TPR_12	Tetratricope	GO:0060271|	GO:0005929|	.	K10407
Manes.01G12000(	1.8277082	1.54E-08	up	no	Aspartic prot	pfam14541	TAXi_C	Xylanase inl	GO:0012501|	GO:0005783|	GO:0004190|as	.
Manes.01G12040(	0.4982014	9.97E-05	down	yes	Bidirectional	pfam03083	MtN3_slv	Sugar efflux	GO:0034219|	GO:0005887|	GO:0051119|su	K15382
Manes.01G12080(	0.5321564	0.00064	down	no	Potassium ch	pfam12796	Ank_2	Ankyrin rep	GO:0071805|	GO:0005887|	GO:0015271|ou	.
Manes.01G12120(	Inf	0.08088	up	no	.	pfam00643	zf-B_box	B-box zinc f	.	.	.	.
Manes.01G12130(	72.910958	#######	up	yes	U-box domai	pfam04564	U-box	U-box doma	GO:0010200|	.	GO:0016874|lig	.
Manes.01G12140(	1.9817577	3.67E-09	up	no	Receptor pro	pfam00069	Pkinase	Protein kina	GO:0009755|	GO:0005576|	GO:0005524|A'	K00924
Manes.01G12150(	2.5010761	3.89E-20	up	yes	Zinc finger pr	pfam13894	zf-C2H2_4	C2H2-type	GO:0006355|	GO:0005737|	GO:0046872|m	.
Manes.01G12180(	0.5660897	0.00058	down	no	.	pfam12675	DUF3795	Protein of u	.	.	.	.
Manes.01G12210(	0.9483487	0.65244	down	no	Protein FAR1	pfam03101	FAR1	FAR1 DNA	GO:0006355|	GO:0005634|	GO:0008270|zi	.
Manes.01G12240(	1.2358375	0.03597	up	no	Glutamate de	pfam00282	Pyridoxal_deC	Pyridoxal-de	GO:0006536|	GO:0005829|	GO:0005516|ca	K01580
Manes.01G12260(	0.6031906	8.07E-05	down	no	Uncharacteriz	pfam03109	ABC1	ABC1 famil	.	GO:0005576|	GO:0005524|A'	K08869
Manes.01G12290(	NA	NA	--	no	Homeobox-le	pfam00046	Homeobox	Homeobox	GO:0006355|	GO:0005634|	GO:0008289|lip	.
Manes.01G12310(	3.210384	1.10E-34	up	yes	Protein REV	pfam00249	Myb_DNA-binding	Myb-like DI	GO:0009851|	GO:0005634|	GO:0003677|DI	.
Manes.01G12320(	1.5988902	3.68E-05	up	no	Autophagy p	pfam04106	APG5	Autophagy	GO:0000045|	GO:0034274|	.	K08339
Manes.01G12330(	6.166102	1.34E-11	up	yes	Probable glut	pfam13417	GST_N_3	Glutathione	GO:0009734|	.	GO:0004364|gl	.
Manes.01G12340(	0.9697265	0.75363	down	no	Coatomer sul	pfam00928	Adap_comp_sub	Adaptor cor	GO:0006886|	GO:0030131|	.	.
Manes.01G12380(	1.1565472	0.1847	up	no	Sucrose syntl	pfam00862	Sucrose_synth	Sucrose syn	GO:0009058|	GO:0005618|	GO:0016157|su	.
Manes.01G12400(	0.6095593	0.00111	down	no	Protein SET	pfam09273	Rubis-subs-bind	Rubisco LSI	.	.	GO:0008168|m	.
Manes.01G12430(	1.3484354	0.13959	up	no	7-deoxylogar	pfam00201	UDPGT	UDP-glucor	.	.	GO:0035251|U	.
Manes.01G12440(	3.2901264	0.0288	up	yes	Butyrate--Co	pfam00501	AMP-binding	AMP-bindir	GO:0019605|	GO:0005777|	GO:0047760|bu	.
Manes.01G12450(	12.312525	9.21E-82	up	yes	Probable poly	pfam01593	Amino_oxidase	Flavin conta	GO:0006598|	GO:0005777|	GO:0052895|N	K17839
Manes.01G12520(	2.2312539	1.28E-19	up	yes	Probable LRI	pfam11721	Malectin	Di-glucose l	.	GO:0016021|	GO:0005524|A'	.
Manes.01G12580(	0.8447731	0.07968	down	no	WAT1-relate	pfam00892	EamA	EamA-like t	.	GO:0009507|	.	.
Manes.01G12590(	46.16496	7.23E-08	up	yes	Protein YLS	pfam03168	LEA_2	Late embryo	GO:0051607|	GO:0009507|	.	.
Manes.01G12610(	4.4850381	1.30E-11	up	yes	Alpha-amylas	pfam00197	Kunitz_legume	Trypsin and	GO:0010951|	.	GO:0015066|al	.
Manes.01G12630(	Inf	0.04608	up	no	Alpha-amylas	pfam00197	Kunitz_legume	Trypsin and	GO:0010951|	.	GO:0015066|al	.
Manes.01G12640(	18.35735	0.00128	up	yes	Miraculin	pfam00197	Kunitz_legume	Trypsin and	.	.	GO:0004866|en	.
Manes.01G12670(	0.6902381	0.00119	down	no	Cell division	pfam00069	Pkinase	Protein kina	GO:0051301|	.	GO:0005524|A'	.
Manes.01G12730(	8.3450782	1.80E-10	up	yes	.	pfam00646	F-box	F-box doma	.	.	.	.
Manes.01G12780(	13.880001	1.62E-39	up	yes	Glycerol-3-pl	pfam01553	Acyltransferase	Acyltransfer	GO:0016024|	GO:0016021|	GO:0090447|gl	K13508
Manes.01G12800(	14.634132	2.13E-11	up	yes	Probable WR	pfam03106	WRKY	WRKY DN	GO:0006351|	GO:0005634|	GO:0043565|se	.
Manes.01G12840(	0.4312903	7.17E-09	down	yes	.	pfam06859	Bin3	Bicoid-inter	.	.	.	.
Manes.01G12880(	0.843955	0.09844	down	no	Serine/threon	pfam08321	PPP5	PPP5 TPR r	GO:0010019|	GO:0005737|	GO:0046872|m	K04460
Manes.01G12900(	1.2884546	0.00754	up	no	Citrate syntha	pfam00285	Citrate_synt	Citrate syntl	GO:0006097|	GO:0009514|	GO:0046912|tra	.
Manes.01G12910(	1.194183	0.05234	up	no	Glutamyl-tRI	pfam01425	Amidase	Amidase.	GO:0006655|	GO:0009570|	GO:0005524|A'	K02433
Manes.01G12920(	0.7659	0.02374	down	no	Actin-related	pfam00400	WD40	WD domain	GO:0007015|	GO:0005885|	GO:0051015|ac	K05757
Manes.01G12930(	3.5902207	2.41E-11	up	yes	Probable LRI	pfam00069	Pkinase	Protein kina	.	GO:0016021|	GO:0005524|A'	.
Manes.01G12950(	2.8871784	3.70E-08	up	yes	Probable LRI	pfam00069	Pkinase	Protein kina	.	GO:0016021|	GO:0005524|A'	.
Manes.01G12980(	4.4202295	0.00696	up	yes	Small heat sh	pfam00011	HSP20	Hsp20/alpha	.	.	.	K13993
Manes.01G12990(	8.1631057	1.14E-05	up	yes	Heat stress tr	pfam00447	HSF_DNA-bind	HSF-type D	GO:0009408|	GO:0005737|	GO:0043565|se	K09414
Manes.01G13010(	1.9976908	1.00E-14	up	no	.	pfam06972	DUF1296	Protein of u	.	.	.	.
Manes.01G13020(	6.9515946	7.31E-35	up	yes	AT-hook mo	pfam03479	DUF296	Domain of u	GO:0006325|	GO:0005634|	GO:0003680|A'	.
Manes.01G13060(	0.4548944	0.00388	down	yes	Cytochrome	pfam03188	Cytochrom_B561	Eukaryotic	GO:0055114|	GO:0016021|	GO:0046872|m	.
Manes.01G13070(	1.095	0.38074	up	no	Aspartate am	pfam00155	Aminotran_1_2	Aminotransl	GO:0006103|	GO:0005759|	GO:0005507|co	K14455
Manes.01G13080(	1.2925963	0.00539	up	no	.	pfam00169	PH	PH domain.	.	.	.	.
Manes.01G13090(	1.6076728	0.00124	up	no	Glutathione S	pfam00043	GST_C	Glutathione	GO:0006952|	GO:0048046|	GO:0005507|co	K00799
Manes.01G13110(	3.1667619	1.13E-25	up	yes	Protein ASPA	pfam14541	TAXi_C	Xylanase inl	GO:0030163|	GO:0005783|	GO:0004190|as	.
Manes.01G13120(	0.7826067	0.01183	down	no	WD repeat-co	pfam00400	WD40	WD domain	.	GO:0005737|	.	.
Manes.01G13130(	0.4753818	1.86E-11	down	yes	Oxygen-evolv	pfam01789	PsbP	PsbP. This f	GO:0015979|	GO:0009535|	GO:0005509|ca	K02717
Manes.01G13140(	1.2731884	0.01398	up	no	Reticulon-lik	pfam02453	Reticulon	Reticulon. F	.	GO:0005789|	.	.
Manes.01G13190(	0.4753621	1.30E-05	down	yes	N-acetyl-D-g	pfam01869	BcrAD_BadFG	BadF/BadG	.	.	GO:0005524|A'	.
Manes.01G13240(	0.3939079	2.41E-15	down	yes	Serine/threon	pfam07714	Pkinase_Tyr	Protein tyro	.	.	GO:0005524|A'	K14510
Manes.01G13250(	1.0727137	0.43691	up	no	ATP-depende	pfam04408	HA2	Helicase ass	GO:0001503|	GO:0000781|	GO:0005524|A'	K14442
Manes.01G13300(	2.2923925	0.00216	up	yes	Bifunctional	pfam13419	HAD_2	Haloacid de	GO:0009398|	.	GO:0005524|A'	K00861
Manes.01G13350(	0.1793843	0.00221	down	yes	Transcription	pfam04844	Ovate	Transcriptio	GO:0045892|	GO:0005634|	.	.
Manes.01G13370(	6.206695	0.14967	up	no	.	pfam04788	DUF620	Protein of u	.	.	.	.
Manes.01G13380(	0.3492526	3.14E-05	down	yes	.	pfam06708	DUF1195	Protein of u	.	.	.	.
Manes.01G13400(	0.8136339	0.03802	down	no	.	pfam09871	DUF2098	Uncharacter	.	.	.	.
Manes.01G13410(	2.3759311	0.0001	up	yes	F-box protein	pfam12937	F-box-like	F-box-like.	GO:0016567|	.	.	.
Manes.01G13440(	0.8986185	0.35695	down	no	U1 small nuc	pfam00076	RRM_1	RNA recogn	GO:0000398|	GO:0016607|	GO:0000166|nu	K11093
Manes.01G13490(	1.165211	0.09594	up	no	Scarecrow-lil	pfam03514	GRAS	GRAS doma	GO:0006355|	GO:0005634|	GO:0003700|tra	.
Manes.01G13500(	1.035899	0.9123	up	no	.	pfam04376	ATE_N	Arginine-tR	.	.	.	.
Manes.01G13530(	0.5728243	0.00081	down	no	Protein AE7	pfam09615	Cas_Csy3	CRISPR-ass	GO:0007059|	GO:0005737|	.	.
Manes.01G13540(	0.3004852	8.84E-05	down	yes	.	pfam06219	DUF1005	Protein of u	.	.	.	.
Manes.01G13550(	0.1997185	6.17E-06	down	yes	.	pfam10259	Rogdi_lz	Rogdi leucir	.	.	.	.
Manes.01G13570(	0.9301175	1	down	no	.	pfam00012	HSP70	Hsp70 prote	.	.	.	.
Manes.01G13620(	0.2506021	0.02543	down	yes	Transcription	pfam00249	Myb_DNA-binding	Myb-like DI	GO:0048262|	GO:0005634|	GO:0003677|DI	.
Manes.01G13650(	0.5531802	0.07964	down	no	.	pfam03276	Gag_spuma	Spumavirus	.	.	.	.
Manes.01G13670(	0.3804349	4.42E-05	down	yes	.	pfam13041	PPR_2	PPR repeat	.	.	.	.
Manes.01G13680(	0.8681137	0.38145	down	no	Syntaxin-112	pfam00804	Syntaxin	Syntaxin. Sy	GO:0006887|	GO:0012505|	GO:0000149|SI	K08486
Manes.01G13690(	0.6639686	0.00138	down	no	Transmembra	pfam05705	DUF829	Eukaryotic	.	GO:0016021|	.	.
Manes.01G13710(	1.1787983	0.09934	up	no	Protein RER	pfam03248	Rer1	Rer1 family.	GO:0006890|	GO:0005801|	.	.
Manes.01G13750(	3.5475135	5.98E-11	up	yes	Soluble inorg	pfam00719	Pyrophosphatase	Inorganic py	GO:0006796|	GO:0005737|	GO:0004427|in	K01507
Manes.01G13790(	13.290678	6.88E-13	up	yes	.	pfam04642	DUF601	Protein of u	.	.	.	.
Manes.01G13810(	0.1612886	6.20E-48	down	yes	Mannan endo	pfam00150	Cellulase	Cellulase (g	GO:0005975|	GO:0071944|	GO:0016985|m	K19355
Manes.01G13820(	0.3993315	8.06E-05	down	yes	.	pfam13041	PPR_2	PPR repeat	.	.	.	.
Manes.01G13830(	0.7018717	0.05237	down	no	Lipid phosph	pfam01569	PAP2	PAP2 super	GO:0016311|	GO:0009507|	GO:0008195|ph	.
Manes.01G13850(	24.508317	0.02055	up	yes	Salicylate car	pfam03492	Methyltransf_7	SAM depen	.	.	GO:0046872|m	.
Manes.01G13950(	12.38982	1.49E-91	up	yes	Probable mito	pfam14363	AAA_assoc	Domain ass	GO:0016226|	GO:0016021|	GO:0005524|A'	K08900
Manes.01G13960(	0.9417126	0.59468	down	no	Thioredoxin	pfam00085	Thioredoxin	Thioredoxin	GO:0051211|	GO:0005737|	GO:0048487|be	.

Manes.01G13970(	0.9830005	0.90133	down	no	Phosphatidyli	pfam00650	CRAL_TRIO	CRAL/TRI(	GO:0015031|	GO:0000139|	.	.
Manes.01G13990(	1.0012503	0.97442	up	no	Uncharacteriz	pfam00400	WD40	WD domain	GO:0006364|	GO:0005829|	.	K14791
Manes.01G14000(	0.7983728	0.03608	down	no	.	pfam03006	HlyIII	Haemolysin·	.	.	.	.
Manes.01G14010(	14.42223	0.00778	up	yes	Peroxidase 7.	pfam00141	peroxidase	Peroxidase.	GO:0042744|	GO:0005576|	GO:0020037|he	K00430
Manes.01G14020(	0.3633825	2.26E-12	down	yes	50S ribosoma	pfam01281	Ribosomal_L9_N	Ribosomal p	GO:0006412|	GO:0005840|	GO:0019843|rR	K02939
Manes.01G14030(	2.1178985	3.30E-07	up	yes	50S ribosoma	pfam00542	Ribosomal_L12	Ribosomal p	GO:0006412|	GO:0005840|	GO:0003735|stı	.
Manes.01G14060(	0.771432	0.00796	down	no	CDK5RAP1-	pfam00919	UPF0004	Uncharacter	GO:0000079|	.	GO:0051536|irc	.
Manes.01G14070(	0.5510841	2.92E-05	down	no	D-2-hydroxy	pfam02913	FAD-oxidase_C	FAD linked	GO:0006807|	GO:0005739|	GO:0051990|(R	K18204
Manes.01G14100(	Inf	0.02254	up	yes	.	pfam10060	DUF2298	Uncharacter	.	.	.	.
Manes.01G14120(	1.1264141	0.20745	up	no	Beta-galactos	pfam01301	Glyco_hydro_35	Glycosyl hy	GO:0005975|	GO:0048046|	GO:0004565|be	.
Manes.01G14140(	0.8214966	0.08629	down	no	Protein RTE1	pfam05608	DUF778	Protein of u	.	GO:0016021|	.	.
Manes.01G14210(	0.57155	3.86E-07	down	no	Receptor-like	pfam13855	LRR_8	Leucine rich	GO:0009755|	GO:0016021|	GO:0004672|pr	.
Manes.01G14220(	0.4349813	3.28E-06	down	yes	Probable leuc	pfam08263	LRRNT_2	Leucine rich	.	GO:0016021|	GO:0005524|A'	.
Manes.01G14240(	0.1146608	0.00239	down	yes	Cytochrome	pfam00067	p450	Cytochrome	GO:0009809|	GO:0005783|	GO:0020037|he	K09755
Manes.01G14250(	0.8821819	0.29122	down	no	Protein FMP.	pfam07798	DUF1640	Protein of u	GO:0033617|	GO:0016021|	.	.
Manes.01G14280(	1.2787673	0.00686	up	no	Zinc finger C	pfam03126	Plus-3	Plus-3 doma	GO:0006352|	GO:0005634|	GO:0003677|DI	.
Manes.01G14290(	1.1420593	0.15191	up	no	.	pfam11833	DUF3353	Protein of u	.	.	.	.
Manes.01G14340(	#NAME?	0.43419	down	no	NAC domain	pfam02365	NAM	No apical m	GO:0001666|	GO:0009507|	GO:0003700|tra	.
Manes.01G14410(	Inf	0.9009	up	no	Mitogen-activ	pfam00069	Pkinase	Protein kina	GO:0000186|	GO:0005938|	GO:0005524|A'	.
Manes.01G14440(	1.0803176	0.44686	up	no	V-type proto	pfam00137	ATP-synt_C	ATP syntha	GO:0015991|	GO:0016021|	GO:0015078|hy	K02155
Manes.01G14450(	0.7239955	0.16934	down	no	NAC domain	pfam02365	NAM	No apical m	GO:0071555|	GO:0005634|	GO:0043565|se	.
Manes.01G14460(	0.8509887	0.23534	down	no	Histone H1	pfam00538	Linker_histone	linker histon	GO:0006334|	GO:0000786|	GO:0003677|DI	.
Manes.01G14470(	1.9314054	3.17E-12	up	no	GDP-mannos	pfam01370	Epimerase	NAD depen	GO:0042351|	GO:0005829|	GO:0050662|co	K01711
Manes.01G14480(	2.3651867	6.83E-10	up	yes	Peptidyl-prol	pfam00639	Rotamase	PPIC-type F	.	.	GO:0003755|pe	.
Manes.01G14500(	0.5551603	0.00829	down	no	Transcription	pfam03366	YEATS	YEATS fam	GO:0030154|	GO:0005737|	.	K11341
Manes.01G14550(	2.3514472	0.20848	up	no	Protein WUS	pfam00046	Homeobox	Homeobox	GO:0030154|	GO:0005634|	GO:0003677|DI	.
Manes.01G14590(	2.9593261	3.37E-07	up	yes	Transcription	pfam00010	HLH	Helix-loop-l	GO:0006351|	GO:0005634|	GO:0003677|DI	.
Manes.01G14600(	0.5872461	6.35E-05	down	no	DNA-(apurin	pfam03372	Exo_endo_phos	Endonuclea	GO:0006284|	GO:0043231|	GO:0003677|DI	.
Manes.01G14610(	4.0386086	3.33E-46	up	yes	Chaperone pı	pfam00226	DnaJ	DnaJ domai	.	GO:0009507|	.	.
Manes.01G14620(	0.5760901	3.66E-06	down	no	.	pfam00514	Arm	Armadillo/b	.	.	.	.
Manes.01G14630(	1.8876088	6.20E-12	up	no	.	pfam00514	Arm	Armadillo/b	.	.	.	.
Manes.01G14660(	0.7688666	0.01697	down	no	Ferredoxin--l	pfam00175	NAD_binding_1	Oxidoreduc	GO:0015979|	GO:0009570|	GO:0004324|fe	.
Manes.01G14680(	1.7664141	0.00932	up	no	Thaumatin-lil	pfam00314	Thaumatin	Thaumatin f	GO:0006952|	GO:0005576|	.	.
Manes.01G14690(	0.3911649	2.51E-06	down	yes	Thaumatin-lil	pfam00314	Thaumatin	Thaumatin f	GO:0006952|	GO:0005576|	.	.
Manes.01G14700(	0.3397292	5.80E-07	down	yes	Calmodulin-l	pfam10294	Methyltransf_16	Putative me	.	GO:0005737|	GO:0018025|ca	K18826
Manes.01G14710(	0.1952638	5.53E-07	down	yes	.	pfam05602	CLPTM1	Cleft lip and	.	.	.	.
Manes.01G14750(	0.4115909	0.00039	down	yes	Myb-related	pfam00249	Myb_DNA-binding	Myb-like DI	GO:0006355|	GO:0005634|	GO:0003677|DI	.
Manes.01G14760(	0.4576802	3.64E-09	down	yes	Histidine kin	pfam00072	Response_reg	Response re	GO:0009738|	GO:0005783|	GO:0009927|his	.
Manes.01G14780(	2.1936195	2.19E-18	up	yes	Nucleolar GT	pfam08153	NGP1NT	NGP1NT (N	GO:0042254|	GO:0016020|	GO:0005525|G'	K14537
Manes.01G14820(	1.3284003	0.00158	up	no	V-type proto	pfam01991	vATP-synt_E	ATP syntha	GO:0015991|	GO:0033178|	GO:0046961|pr	.
Manes.01G14830(	Inf	0.08975	up	no	.	pfam06813	Nodulin-like	Nodulin-like	.	.	.	.
Manes.01G14850(	2.2838855	8.31E-15	up	yes	Cell cycle ch	pfam03215	Rad17	Rad17 cell	GO:0007049|	GO:0005634|	GO:0005524|A'	K06662
Manes.01G14870(	0.8026949	0.02782	down	no	Rac-like GTI	pfam00071	Ras	Ras family.	GO:0009738|	GO:0005737|	GO:0005525|G'	K04392
Manes.01G14880(	1.0525923	0.56866	up	no	Vacuolar pro	pfam03635	Vps35	Vacuolar pr	GO:0042147|	GO:0005794|	GO:0008565|pr	K18468
Manes.01G14920(	0.4581187	8.75E-10	down	yes	Protein MID	pfam04749	PLAC8	PLAC8 fam	GO:0006816|	GO:0005887|	.	.
Manes.01G14930(	Inf	0.38692	up	no	Senescence-s	pfam00112	Peptidase_C1	Papain famil	GO:0007568|	GO:0010282|	GO:0008234|cy	.
Manes.01G14960(	0.5858406	0.00462	down	no	Protein preY	pfam03966	Trm112p	Trm112p-lik	.	GO:0005739|	.	.
Manes.01G14970(	0.807453	0.54464	down	no	E3 ubiquitin-	pfam13639	zf-RING_2	Ring finger	.	GO:0016021|	GO:0016874|lig	.
Manes.01G14980(	0.8186091	0.18514	down	no	F-box protei	pfam00646	F-box	F-box doma	GO:0031348|	GO:0005737|	.	.
Manes.01G15010(	0.5815147	0.0022	down	no	Methyltransf	pfam10294	Methyltransf_16	Putative me	.	GO:0005737|	GO:0008168|me	.
Manes.01G15030(	0.5541171	4.10E-06	down	no	Aspartic prot	pfam14543	TAXi_N	Xylanase inl	.	GO:0031225|	GO:0004190|as	.
Manes.01G15040(	0.4442829	6.54E-13	down	yes	Probable prot	pfam00481	PP2C	Protein pho	GO:0006470|	GO:0016021|	GO:0046872|me	.
Manes.01G15080(	3.9115362	0.00048	up	yes	.	pfam02393	US22	US22 like.	.	.	.	.
Manes.01G15110(	1.0068744	0.92825	up	no	Phospholipas	pfam12357	PLD_C	Phospholipa	GO:0016042|	GO:0016020|	GO:0005509|ca	K01115
Manes.01G15210(	4.0850647	4.89E-23	up	yes	.	pfam13716	CRAL_TRIO_2	Divergent C	.	.	.	.
Manes.01G15220(	0.2687911	1.92E-09	down	yes	Putative dise	pfam12799	LRR_4	Leucine Ric	GO:0006952|	.	GO:0043531|Al	.
Manes.01G15230(	0.8347227	0.25222	down	no	ATP-depend	pfam00270	DEAD	DEAD/DEA	GO:0032508|	GO:0005634|	GO:0043138|3'-	K10901
Manes.01G15240(	0.9668614	0.77483	down	no	F-box protei	pfam03478	DUF295	Protein of u	GO:0016567|	GO:0005634|	.	.
Manes.01G15250(	0.5847514	0.00036	down	no	Putative F-bo	pfam03478	DUF295	Protein of u	GO:2000083|	.	.	.
Manes.01G15280(	Inf	4.47E-06	up	yes	GDSL estera	pfam00657	Lipase_GDSL	GDSL-like	GO:0016042|	GO:0005576|	GO:0016298|lip	.
Manes.01G15360(	2.0844178	7.69E-16	up	yes	Isocitrate del	pfam00180	Iso_dh	Isocitrate/is	GO:0006102|	GO:0005739|	GO:0004449|is	K00030
Manes.01G15370(	0.7889056	0.01728	down	no	Protein GRIF	pfam01576	Myosin_tail_1	Myosin tail.	GO:0048193|	GO:0000795|	.	.
Manes.01G15390(	0.4874733	3.47E-05	down	yes	G2/mitotic-sp	pfam00134	Cyclin_N	Cyclin, N-te	GO:0051301|	GO:0005634|	.	.
Manes.01G15410(	0.6797181	0.00097	down	no	Poly(A) poly	pfam01743	PolyA_pol	Poly A poly	GO:0006378|	.	GO:0005524|A'	K00970
Manes.01G15460(	42.570287	4.16E-13	up	yes	Dirigent prot	pfam03018	Dirigent	Dirigent-like	GO:1901599|	GO:0048046|	GO:0042349|gu	.
Manes.01G15480(	0.7651716	0.02455	down	no	Aspartic prot	pfam14543	TAXi_N	Xylanase inl	.	.	GO:0004190|as	.
Manes.01G15520(	1.0223651	0.82277	up	no	Heterogeneo	pfam14259	RRM_6	RNA recogı	GO:0006397|	GO:0005654|	GO:0000166|nu	K12898
Manes.01G15590(	0.7102747	0.00074	down	no	Uncharacteriz	pfam12763	EF-hand_4	Cytoskeleta	GO:0000147|	GO:0030479|	GO:0005509|ca	.
Manes.01G15610(	0.3685672	0.00144	down	yes	Pentatricope	pfam13041	PPR_2	PPR repeat	.	GO:0005739|	.	.
Manes.01G15640(	0.9993174	0.9743	down	no	Serine/threon	pfam06479	Ribonuc_2-5A	Ribonucleas	GO:0006987|	GO:0005783|	GO:0005524|A'	K08852
Manes.01G15660(	0.5256289	0.03762	down	no	.	pfam03547	Mem_trans	Membrane t	.	.	.	.
Manes.01G15680(	1.3985494	0.00036	up	no	MLO-like pro	pfam03094	Mlo	Mlo family.	GO:0006952|	GO:0016021|	.	K08472
Manes.01G15690(	2.8135565	3.92E-20	up	yes	RING-H2 fin	pfam13639	zf-RING_2	Ring finger	GO:0006952|	GO:0016021|	GO:0008270|zi	.
Manes.01G15700(	0.9011642	0.32468	down	no	.	pfam10199	Adaptin_binding	Alpha and g	.	.	.	.
Manes.01G15710(	1.6447116	2.54E-08	up	no	Eukaryotic tr	pfam01912	eIF-6	eIF-6 family	GO:0071215|	GO:0005829|	GO:0043023|ril	K03264
Manes.01G15730(	0.8758486	0.19727	down	no	RNA polyme	pfam05327	RRN3	RNA polym	GO:0006355|	GO:0005730|	.	K15216
Manes.01G15790(	0.8201755	0.0458	down	no	AT-rich inter	pfam01388	ARID	ARID/BRIC	GO:0006355|	GO:0005634|	GO:0003677|DI	.
Manes.01G15890(	1.0446606	0.73624	up	no	N-acylphospl	pfam01553	Acyltransferase	Acyltransfer	GO:0006650|	GO:0016021|	GO:0008374|O	K13511
Manes.01G15910(	0.7309714	0.02166	down	no	Histone deac	pfam00850	Hist_deacetyl	Histone dea	GO:0006355|	GO:0005634|	GO:0032041|N.	.
Manes.01G15920(	1.091411	0.55861	up	no	ABC transpo	pfam01061	ABC2_membrane	ABC-2 type	GO:0010588|	GO:0009897|	GO:0005524|A'	.
Manes.01G15950(	1.7819399	0.00027	up	no	.	pfam05623	DUF789	Protein of u	.	.	.	.
Manes.01G15960(	0.8360732	0.07494	down	no	Testis-expres	pfam10296	DUF2404	Putative int	.	GO:0016021|	.	.
Manes.01G15990(	0.4200514	4.94E-11	down	yes	.	pfam00149	Metallophos	Calcineurin-	.	.	.	.
Manes.01G16050(	0.4134607	0.00035	down	yes	U-box domai	pfam00514	Arm	Armadillo/b	.	GO:0005886|	GO:0016874|lig	.
Manes.01G16140(	0.8167717	0.07683	down	no	Protein ELF4	pfam07011	DUF1313	Protein of u	GO:0042753|	GO:0005634|	GO:0042803|pr	.
Manes.01G16170(	1.0106252	0.93999	up	no	NudC domai	pfam04969	CS	CS domain.	.	GO:0000777|	.	.
Manes.01G16190(	1.0234932	0.85599	up	no	Vacuolar cati	pfam01699	Na_Ca_ex	Sodium/calc	GO:0070588|	GO:0016021|	GO:0015369|ca	K07300
Manes.01G16220(	2.8334606	1.59E-05	up	yes	Ammonium t	pfam00909	Ammonium_trans	Ammonium	GO:0015696|	GO:0016021|	GO:0008519|an	K03320

Manes.01G16250(	0.6106452	0.00754	down	no	UDP-glycosy	pfam00201	UDPGT	UDP-glucor	GO:0009813|	GO:0043231|	GO:0047893|fla	.
Manes.01G16270(	1.0304471	0.78524	up	no	Probable NA	pfam03358	FMN_red	NADPH-dep	GO:0045892|	GO:0005886|	GO:0010181|FN	K03809
Manes.01G16280(	1.1524484	0.13803	up	no	Anthranilate :	pfam00425	Chorismate_bind	chorismate l	GO:0000162|	GO:0005950|	GO:0004049|an	K01657
Manes.01G16290(	Inf	0.35135	up	no	60S acidic rit	pfam00466	Ribosomal_L10	Ribosomal p	GO:0042254|	GO:0022626|	.	K02941
Manes.01G16350(	0.5998844	6.45E-06	down	no	.	pfam04158	Sof1	Sof1-like do	.	.	.	.
Manes.01G16380(	17.650961	1.20E-07	up	yes	.	pfam12835	Integrase_1	Integrase. T	.	.	.	.
Manes.01G16390(	0.7115745	0.10758	down	no	.	pfam14636	FNIP_N	Folliculin-in	.	.	.	.
Manes.01G16400(	0.8284678	0.08025	down	no	.	pfam00168	C2	C2 domain.	.	.	.	.
Manes.01G16420(	3.5885068	2.22E-11	up	yes	Phosphometh	pfam01964	ThiC	ThiC family.	GO:0010266|	GO:0009507|	GO:0051539|4	K03147
Manes.01G16430(	0.7831113	0.08504	down	no	ATP-depende	pfam00004	AAA	ATPase fam	.	GO:0009507|	GO:0005524|A'	K03798
Manes.01G16440(	Inf	0.9009	up	no	.	pfam10271	Tmp39	Putative trai	.	.	.	.
Manes.01G16460(	0.7898381	0.06404	down	no	Sugar transp	pfam00083	Sugar_tr	Sugar (and c	GO:0008643|	GO:0016021|	GO:0022891|su	.
Manes.01G16470(	0.8015731	0.0294	down	no	Beta-glucosic	pfam00933	Glyco_hydro_3	Glycosyl hy	GO:0085030|	GO:0042597|	GO:0008422|be	.
Manes.01G16530(	0.975638	0.8061	down	no	Helicase SKI	pfam08148	DSHCT	DSHCT (NI	GO:0006401|	GO:0005654|	GO:0005524|A'	K12599
Manes.01G16540(	0.6342404	0.00017	down	no	.	pfam04859	DUF641	Plant protei	.	.	.	.
Manes.01G16570(	0.9607007	0.71531	down	no	Cytosolic enc	pfam00113	Enolase_C	Enolase, C-t	GO:0006096|	GO:0005737|	GO:0000287|ma	K01689
Manes.01G16580(	0.9116444	0.57023	down	no	Neutral ceran	pfam04734	Ceramidase_alk	Neutral/alka	GO:0006665|	GO:0005576|	GO:0017040|ce	K12349
Manes.01G16610(	0.4562927	4.19E-05	down	yes	Tubulin beta-	pfam03953	Tubulin_C	Tubulin C-te	GO:0007017|	GO:0005794|	GO:0005525|G'	K07375
Manes.01G16630(	6.9071009	0.01968	up	yes	.	pfam06521	PAR1	PAR1 prote	.	.	.	.
Manes.01G16650(	0.7775388	0.08794	down	no	DNA-directe	pfam13656	RNA_pol_L_2	RNA polym	GO:0042254|	GO:0005736|	GO:0003677|DI	K03020
Manes.01G16670(	1.6524144	9.53E-08	up	no	Probable seri	pfam00069	Pkinase	Protein kina	GO:0009755|	GO:0005886|	GO:0005524|A'	K14500
Manes.01G16680(	0.5928062	1.93E-07	down	no	.	pfam12043	DUF3527	Domain of u	.	.	.	.
Manes.01G16690(	0.3423884	0.00024	down	yes	.	pfam00646	F-box	F-box doma	.	.	.	.
Manes.01G16740(	#NAME?	0.65369	down	no	Glutathione S	pfam00043	GST_C	Glutathione	GO:0009751|	GO:0005737|	GO:0004364|gl	K00799
Manes.01G16770(	Inf	0.00042	up	yes	Cucumisin	pfam00082	Peptidase_S8	Subtilase fai	.	GO:0005576|	GO:0004252|se	.
Manes.01G16780(	1.016651	0.86128	up	no	Ras-related p	pfam00071	Ras	Ras family.	GO:0015031|	GO:0005622|	GO:0005525|G'	.
Manes.01G16860(	1.7185446	0.05052	up	no	Probable prot	pfam00481	PP2C	Protein phos	GO:0006470|	.	GO:0046872|me	K14497
Manes.01G16870(	1.7561647	6.08E-05	up	no	.	pfam00106	adh_short	short chain c	.	.	.	.
Manes.01G16900(	NA	NA	--	no	Probable tran	pfam13912	zf-C2H2_6	C2H2-type	GO:0030154|	GO:0005634|	GO:0046872|me	.
Manes.01G16940(	1.8787316	1.17E-12	up	no	Transcription	pfam03153	TFIIA	Transcriptio	GO:0006355|	GO:0005672|	.	K03122
Manes.01G16950(	7.4577987	0.08789	up	no	.	pfam03124	EXS	EXS family.	.	.	.	.
Manes.01G16970(	15.374219	7.63E-13	up	yes	L-type lectin-	pfam00139	Lectin_legB	Legume lect	GO:0009755|	GO:0016021|	GO:0005524|A'	.
Manes.01G17000(	11.669564	4.65E-33	up	yes	Transcription	pfam00249	Myb_DNA-binding	Myb-like DI	GO:0007623|	GO:0005634|	GO:0003677|DI	.
Manes.01G17010(	1.9900301	8.92E-13	up	no	.	pfam14037	YoqO	YoqO-like p	.	.	.	.
Manes.01G17040(	0.6066784	1.81E-05	down	no	E3 ubiquitin-	pfam13639	zf-RING_2	Ring finger	GO:0009789|	GO:0005783|	GO:0016874|lig	K16283
Manes.01G17060(	54.162093	#######	up	yes	Laccase-2	pfam07732	Cu-oxidase_3	Multicopper	GO:0046274|	GO:0048046|	GO:0005507|co	K05909
Manes.01G17120(	1.3971868	0.0002	up	no	ATP-depende	pfam01434	Peptidase_M41	Peptidase fa	.	GO:0009535|	GO:0005524|A'	K08956
Manes.01G17160(	0.9177609	0.43912	down	no	.	pfam15395	DUF4617	Domain of u	.	.	.	.
Manes.01G17170(	6.289693	4.04E-95	up	yes	Scarecrow-lil	pfam03514	GRAS	GRAS doma	GO:0006355|	GO:0005634|	.	.
Manes.01G17180(	11.93595	0.01755	up	yes	Scarecrow-lil	pfam03514	GRAS	GRAS doma	GO:0045893|	GO:0005829|	GO:0003700|tra	.
Manes.01G17190(	25.450158	#######	up	yes	Scarecrow-lil	pfam03514	GRAS	GRAS doma	GO:0045893|	GO:0005829|	GO:0003700|tra	.
Manes.01G17200(	2.2945907	6.41E-20	up	yes	Scarecrow-lil	pfam03514	GRAS	GRAS doma	GO:0045893|	GO:0005829|	GO:0003700|tra	.
Manes.01G17230(	3.0600353	7.11E-30	up	yes	Oxysterol-bir	pfam01237	Oxysterol_BP	Oxysterol-b	GO:0006869|	GO:0005829|	GO:0008289|lip	.
Manes.01G17240(	2.4143925	2.87E-17	up	yes	Uncharacteriz	pfam07019	Rab5ip	Rab5-intera	.	GO:0005739|	.	.
Manes.01G17280(	1.9929492	5.25E-05	up	no	Cytochrome	pfam00067	p450	Cytochrome	GO:0016126|	GO:0016021|	GO:0000249|C-	K09832
Manes.01G17290(	1.6504421	0.00024	up	no	Pentatricope	pfam13041	PPR_2	PPR repeat	.	GO:0016020|	.	.
Manes.01G17310(	0.5422303	6.11E-06	down	no	Putative hydr	pfam12706	Lactamase_B_2	Beta-lactam	.	GO:0005829|	GO:0016787|hy	.
Manes.01G17330(	0.5379245	0.00017	down	no	Probable rece	pfam07714	Pkinase_Tyr	Protein tyro	.	GO:0005886|	GO:0005524|A'	K00924
Manes.01G17370(	1.0790264	0.5305	up	no	Glucan endo-	pfam00332	Glyco_hydro_17	Glycosyl hy	GO:0005975|	.	GO:0042973|gl	.
Manes.01G17390(	1.6168983	3.71E-06	up	no	Mitochondria	pfam02953	zf-Tim10_DDP	Tim10/DDP	GO:0007005|	GO:0005758|	GO:0046872|me	K17777
Manes.01G17450(	2.0305487	0.04361	up	no	.	pfam12691	Minor_capsid_3	Minor capsi	.	.	.	.
Manes.01G17480(	1.0122601	0.88465	up	no	.	pfam10058	DUF2296	Predicted in	.	.	.	.
Manes.01G17510(	2.3687608	4.62E-19	up	yes	CBL-interact	pfam00069	Pkinase	Protein kina	GO:0007165|	.	GO:0005524|A'	.
Manes.01G17520(	0.5647039	2.38E-05	down	no	.	pfam13041	PPR_2	PPR repeat	.	.	.	.
Manes.01G17530(	0.8755654	0.39977	down	no	RINT1-like p	pfam04437	RINT1_TIP1	RINT-1 / TI	GO:0048193|	GO:0005783|	.	.
Manes.01G17550(	4.5317495	1.03E-11	up	yes	Probable gala	pfam05691	Raffinose_syn	Raffinose sy	GO:0005975|	.	GO:0047274|ga	K06617
Manes.01G17570(	0.9222941	0.53248	down	no	Chlorophyll a	pfam00504	Chloroa_b-bind	Chlorophyll	GO:0009765|	GO:0009535|	GO:0016168|ch	.
Manes.01G17610(	5.1338328	0.22741	up	no	.	pfam10593	Z1	Z1 domain.	.	.	.	.
Manes.01G17630(	Inf	1.44E-06	up	yes	Proline-rich r	pfam00069	Pkinase	Protein kina	GO:0009755|	GO:0016021|	GO:0005524|A'	.
Manes.01G17650(	1.3457308	0.00091	up	no	.	pfam00627	UBA	UBA/TS-N	.	.	.	.
Manes.01G17660(	0.7130947	0.05939	down	no	Dr1-associate	pfam00808	CBFD_NFYB_HM	Histone-like	GO:0000122|	GO:0005634|	GO:0043565|se	.
Manes.01G17670(	13.337939	2.32E-05	up	yes	Indole-3-acet	pfam02519	Auxin_inducible	Auxin respo	GO:0009733|	.	.	.
Manes.01G17690(	14.19627	0.00023	up	yes	Indole-3-acet	pfam02519	Auxin_inducible	Auxin respo	GO:0009733|	.	.	.
Manes.01G17700(	5.4955875	7.58E-38	up	yes	Feruloyl CoA	pfam14226	DIOX_N	non-haem di	GO:0009805|	.	GO:0051213|di	K06892
Manes.01G17720(	0.5295292	4.77E-09	down	no	Molybdopter	pfam02391	MoaE	MoaE prote	GO:0006777|	GO:0005829|	GO:0030366|mo	K03635
Manes.01G17740(	1.005084	0.95473	up	no	.	pfam07810	TMC	TMC domai	.	.	.	.
Manes.01G17760(	1.8737461	9.48E-09	up	no	.	pfam00527	E7	E7 protein,	.	.	.	.
Manes.01G17780(	1.1541689	0.1403	up	no	Hydroxymeth	pfam00682	HMGL-like	HMGL-like.	.	GO:0005759|	GO:0004419|hy	K01640
Manes.01G17840(	2.6807226	5.40E-21	up	yes	.	pfam13041	PPR_2	PPR repeat	.	.	.	.
Manes.01G17850(	0.4681199	0.00061	down	yes	.	pfam00068	Phospholip_A2_1	Phospholipa	.	.	.	.
Manes.01G17880(	2.7766453	0.00064	up	yes	.	pfam14630	ORC5_C	Origin recog	.	.	.	.
Manes.01G17920(	128.82585	9.55E-20	up	yes	.	pfam14009	DUF4228	Domain of u	.	.	.	.
Manes.01G17940(	0.5195855	0.03707	down	no	.	pfam13041	PPR_2	PPR repeat	.	.	.	.
Manes.01G17960(	0.4513975	0.00548	down	yes	.	pfam02790	COX2_TM	Cytochrome	.	.	.	.
Manes.01G17970(	1.4960224	0.00265	up	no	.	pfam12796	Ank_2	Ankyrin rep	.	.	.	.
Manes.01G17980(	1.8886938	1.80E-12	up	no	Signal peptid	pfam04258	Peptidase_A22B	Signal pepti	GO:0009555|	GO:0005783|	GO:0004190|as	K09595
Manes.01G17990(	0.5194201	1.54E-08	down	no	Homeobox p	pfam00046	Homeobox	Homeobox	GO:0007275|	GO:0005634|	GO:0043565|se	.
Manes.01G18000(	99.478987	2.26E-27	up	yes	E3 ubiquitin	pfam13639	zf-RING_2	Ring finger	GO:0009790|	GO:0016021|	GO:0016874|lig	.
Manes.01G18010(	0.8552626	0.10453	down	no	Ultraviolet-B	pfam08381	BRX	Transcriptio	GO:0009649|	GO:0000785|	GO:0003682|ch	.
Manes.01G18020(	29.274481	3.42E-16	up	yes	Receptor-like	pfam13855	LRR_8	Leucine rich	GO:0009755|	GO:0016021|	GO:0004672|pr	.
Manes.01G18030(	4.0073472	2.42E-11	up	yes	Receptor-like	pfam13855	LRR_8	Leucine rich	GO:0009755|	GO:0016021|	GO:0004672|pr	.
Manes.01G18070(	Inf	0.00043	up	yes	.	pfam11050	Viral_env_E26	Virus envelo	.	.	.	.
Manes.01G18140(	0.8067377	0.06302	down	no	.	pfam05342	Peptidase_M26_N	M26 IgA1-s	.	.	.	.
Manes.01G18150(	0.9082208	0.40082	down	no	Probable mag	pfam05653	Mg_trans_NIPA	Magnesium	GO:0015693|	GO:0005769|	GO:0015095|ma	.
Manes.01G18190(	0.1840209	1.67E-09	down	yes	60S ribosoma	pfam00181	Ribosomal_L2	Ribosomal I	GO:0006412|	GO:0005739|	GO:0003735|str	.
Manes.01G18240(	0.504564	1.18E-07	down	no	Scarecrow-lil	pfam03514	GRAS	GRAS doma	GO:0006355|	GO:0005634|	GO:0003700|tra	.
Manes.01G18260(	2.213069	9.41E-09	up	yes	F-box protein	pfam00646	F-box	F-box doma	GO:0016567|	.	.	.
Manes.01G18270(	2.2769361	2.73E-19	up	yes	Probable pect	pfam01095	Pectinesterase	Pectinestera	GO:0042545|	GO:0005618|	GO:0045330|as	.

Manes.01G18290(	1.1979651	0.06542	up	no	Decapping nu	pfam08652	RAI1	RAI1 like P	.	GO:0009507|	GO:0046872|m	.
Manes.01G18300(	1.2063983	0.04385	up	no	Dipeptidyl pe	pfam00930	DPPIV_N	Dipeptidyl p	.	GO:0005737|	GO:0004177|an	K08655
Manes.01G18320(	2.2730481	5.49E-09	up	yes	Pentatricopep	pfam13041	PPR_2	PPR repeat	GO:0009793|	GO:0009507|	GO:0005524|A	.
Manes.01G18380(	0.4341475	3.20E-15	down	yes	.	pfam13178	DUF4005	Protein of u	.	.	.	.
Manes.01G18390(	0.4729259	2.48E-10	down	yes	Uncharacteriz	pfam02567	PhzC-PhzF	Phenazine b	GO:0009058|	.	GO:0016853|iso	.
Manes.01G18410(	0.7279268	0.01183	down	no	Probable VA	pfam13774	Longin	Regulated-S	GO:0006810|	GO:0016021|	.	.
Manes.01G18430(	0.5768726	1.95E-06	down	no	.	pfam05557	MAD	Mitotic chec	.	.	.	.
Manes.01G18480(	1.0398468	0.6936	up	no	Protein ABIL	pfam06728	PIG-U	GPI transam	.	GO:0005737|	.	.
Manes.01G18490(	18.99223	3.33E-08	up	yes	.	pfam04892	VanZ	VanZ like fa	.	.	.	.
Manes.01G18510(	0.7149688	0.00066	down	no	.	pfam00013	KH_1	KH domain.	.	.	.	.
Manes.01G18520(	0.7722478	0.00925	down	no	Probable apy	pfam01150	GDA1_CD39	GDA1/CD3	GO:0009901|	GO:0030659|	GO:0005524|A	K01510
Manes.01G18560(	0.6245113	7.06E-05	down	no	Zinc-finger h	pfam04770	ZF-HD_dimer	ZF-HD prot	GO:0045893|	GO:0005634|	GO:0003677|D	.
Manes.01G18580(	0.3235789	3.04E-14	down	yes	.	pfam07891	DUF1666	Protein of u	.	.	.	.
Manes.01G18620(	1.5298269	2.10E-06	up	no	Probable met	pfam03141	Methyltransf_29	Putative S-a	.	GO:0005768|	GO:0008168|m	.
Manes.01G18640(	0.823162	0.08128	down	no	Sphinganine	pfam04116	FA_hydroxylase	Fatty acid h	GO:0006633|	GO:0005783|	GO:0005506|iro	K04713
Manes.01G18680(	1.7760629	0.00013	up	no	Probable ran	pfam04603	Mog1	Ran-interact	GO:0015031|	GO:0005829|	GO:0005085|gu	.
Manes.01G18690(	1.7538328	0.00226	up	no	Pentatricopep	pfam13041	PPR_2	PPR repeat	GO:0080156|	GO:0005739|	.	.
Manes.01G18720(	1.0114121	0.91833	up	no	Ultraviolet-B	pfam08381	BRX	Transcriptio	GO:0009649|	GO:0000785|	GO:0003682|ch	.
Manes.01G18740(	1.3239157	0.0032	up	no	.	pfam08317	Spc7	Spc7 kineto	.	.	.	.
Manes.01G18760(	1.9738835	6.12E-12	up	no	F-box/kelch-r	pfam01344	Kelch_1	Kelch motif.	GO:0016567|	GO:0005634|	.	.
Manes.01G18780(	1.4791504	3.24E-05	up	no	.	pfam00076	RRM_1	RNA recogn	.	.	.	.
Manes.01G18790(	0.6365976	5.21E-06	down	no	Homeobox-le	pfam02183	HALZ	Homeobox	GO:0048826|	GO:0005634|	GO:0043565|se	K09338
Manes.01G18820(	2.7397872	7.78E-11	up	yes	Protein kinas	pfam00069	Pkinase	Protein kina	GO:0009755|	GO:0005737|	GO:0005524|A	K04733
Manes.01G18830(	4.0539465	4.63E-10	up	yes	.	pfam13090	PP_kinase_C	Polyphospha	.	.	.	.
Manes.01G18850(	0.6358525	0.01307	down	no	Vacuolar pro	pfam12850	Metallophos_2	Calcineurin-	GO:0006623|	GO:0005794|	GO:0008270|zi	K18467
Manes.01G18870(	0.9787184	0.99095	down	no	Pentatricopep	pfam13041	PPR_2	PPR repeat	GO:0080156|	GO:0005739|	.	.
Manes.01G18880(	0.1356196	7.38E-08	down	yes	.	pfam10328	7TM_GPCR_Srx	Serpentine t	.	.	.	.
Manes.01G18910(	4.0065861	0.46784	up	no	SNF1-related	pfam00571	CBS	CBS domain	GO:0005975|	.	.	.
Manes.01G18950(	1.1548142	0.1139	up	no	.	pfam07889	DUF1664	Protein of u	.	.	.	.
Manes.01G18980(	1.7799474	1.20E-09	up	no	Alpha-amylas	pfam00128	Alpha-amylase	Alpha amyla	GO:0005983|	GO:0009507|	GO:0004556|al	.
Manes.01G18990(	1.2110977	0.03548	up	no	Mitochondria	pfam01459	Porin_3	Eukaryotic	.	GO:0005741|	GO:0015288|po	K15040
Manes.01G19030(	0.8817143	0.35007	down	no	.	pfam04918	DltD_M	DltD central	.	.	.	.
Manes.01G19040(	0.3525545	3.76E-16	down	yes	Protein TWI	pfam13426	PAS_9	PAS domain	GO:0018298|	GO:0005622|	GO:0000155|ph	.
Manes.01G19050(	0.2373935	0.00045	down	yes	.	pfam02028	BCCT	BCCT famil	.	.	.	.
Manes.01G19060(	0.2809066	2.48E-08	down	yes	.	pfam03283	PAE	Pectinacetyl	.	.	.	.
Manes.01G19110(	0.3244061	0.07363	down	no	Protein NRT	pfam00858	ASC	Amiloride-se	GO:0080168|	GO:0016021|	GO:0090440|ab	.
Manes.01G19120(	0.2615263	6.52E-09	down	yes	Protein TWI	pfam13426	PAS_9	PAS domain	GO:0018298|	GO:0005622|	GO:0000155|ph	.
Manes.01G19160(	0.5223994	6.56E-05	down	no	Ultraviolet-B	pfam00415	RCC1	Regulator o	GO:0009649|	GO:0000785|	GO:0003682|ch	.
Manes.01G19170(	0.3762654	0.00024	down	yes	Outer envelo	pfam11854	DUF3374	Protein of u	GO:0034765|	GO:0009507|	GO:0015288|po	.
Manes.01G19180(	0.6392234	0.00193	down	no	Sucrose nonf	pfam02922	CBM_48	Carbohydra	GO:0032147|	GO:0005737|	GO:0030295|pr	.
Manes.01G19190(	11.738052	0.10742	up	no	Protein NRT	pfam00854	PTR2	POT family.	GO:0080055|	GO:0016021|	GO:0080054|lo	.
Manes.01G19210(	3.040329	1.46E-14	up	yes	Thioredoxin	pfam00085	Thioredoxin	Thioredoxin	GO:0045454|	GO:0005737|	GO:0016671|ox	K03671
Manes.01G19240(	3.1974938	1.34E-24	up	yes	.	pfam04601	DUF569	Protein of u	.	.	.	.
Manes.01G19250(	0.5171526	2.98E-05	down	no	Probable seri	pfam00069	Pkinase	Protein kina	GO:0071215|	GO:0005886|	GO:0005524|A	.
Manes.01G19260(	1.5337841	3.90E-06	up	no	Tetratricopep	pfam06386	GvpL_GvpF	Gas vesicle	.	GO:0070062|	.	.
Manes.01G19320(	0.5883668	0.0014	down	no	.	pfam07059	DUF1336	Protein of u	.	.	.	.
Manes.01G19340(	1.7843861	4.38E-07	up	no	Transcription	pfam00010	HLH	Helix-loop-l	GO:0048446|	GO:0005634|	GO:0003677|D	.
Manes.01G19370(	0.7440465	0.00939	down	no	Serine/threon	pfam00149	Metallophos	Calcineurin-	.	GO:0005737|	GO:0046872|m	.
Manes.01G19380(	0.8820186	0.35242	down	no	.	pfam14963	CAML	Calcium sign	.	.	.	.
Manes.01G19400(	1.1496276	0.13374	up	no	Transcription	pfam00249	Myb_DNA-binding	Myb-like D	GO:0006355|	GO:0005829|	GO:0003677|D	.
Manes.01G19420(	1.9240031	4.66E-09	up	no	UPF0392 pro	pfam01697	Glyco_transf_92	Glycosyltran	.	GO:0016021|	.	.
Manes.01G19460(	0.7619244	0.01797	down	no	Vacuolar pro	pfam05871	ESCRT-II	ESCRT-II c	GO:0043328|	GO:0000814|	GO:0042803|pr	K12189
Manes.01G19480(	4.3865234	2.98E-08	up	yes	Germin-like	pfam00190	Cupin_1	Cupin. This	.	GO:0048046|	GO:0030145|m	.
Manes.01G19490(	0.5014273	9.45E-10	down	no	Protein WAV	pfam06886	TPX2	Targeting pr	GO:0007163|	GO:0055028|	.	.
Manes.01G19510(	0.4653934	5.14E-06	down	yes	Two-compon	pfam00072	Response_reg	Response re	GO:0007623|	GO:0005737|	GO:0000156|ph	K14492
Manes.01G19530(	0.5087581	1.20E-05	down	no	Zinc finger M	pfam01753	zf-MYND	MYND fing	GO:0045892|	GO:0005737|	GO:0042826|his	.
Manes.01G19540(	0.8675073	0.1354	down	no	.	pfam05708	DUF830	Orthopoxvir	.	.	.	.
Manes.01G19560(	2.4668209	3.93E-05	up	yes	Metalloendop	pfam00413	Peptidase_M10	Matrixin. Th	.	GO:0031012|	GO:0004222|m	.
Manes.01G19590(	2.1077634	2.21E-07	up	yes	.	pfam14432	DYW_deaminase	DYW family	.	.	.	.
Manes.01G19600(	2.7954508	0.00249	up	yes	Zinc finger pr	pfam13912	zf-C2H2_6	C2H2-type	GO:0007275|	GO:0005737|	GO:0046872|m	.
Manes.01G19640(	4.0537383	0.0014	up	yes	Snakin-2	pfam02704	GASA	Gibberellin	GO:0006952|	GO:0005618|	.	.
Manes.01G19670(	0.8742406	0.35943	down	no	DNA polyme	pfam14792	DNA_pol_B_palm	DNA polym	GO:0006281|	GO:0005634|	GO:0003677|D	K03512
Manes.01G19690(	0.1543753	2.64E-14	down	yes	Cyclin-D1-1	pfam00134	Cyclin_N	Cyclin, N-te	GO:0007049|	GO:0005634|	.	K18810
Manes.01G19710(	0.6312097	6.86E-06	down	no	.	pfam14009	DUF4228	Domain of u	.	.	.	.
Manes.01G19730(	1.0034964	0.9523	up	no	.	pfam09793	AD	Anticodon-b	.	.	.	.
Manes.01G19740(	0.3885337	3.26E-09	down	yes	Protein ALT	pfam13839	PC-Esterase	GDSL/SGN	GO:0010411|	GO:0005794|	GO:0016413|O-	.
Manes.01G19750(	0.7855448	0.02793	down	no	.	pfam00583	Acetyltransf_1	Acetyltransf	.	.	.	.
Manes.01G19780(	0.0738276	1.64E-21	down	yes	WAT1-relate	pfam00892	EamA	EamA-like t	.	GO:0016021|	GO:0022857|tra	.
Manes.01G19790(	0.7891429	0.36185	down	no	WAT1-relate	pfam00892	EamA	EamA-like t	.	GO:0016021|	GO:0022857|tra	.
Manes.01G19800(	0.3718729	2.56E-05	down	yes	Transcription	pfam14215	bHLH-MYC_N	bHLH-MYC	GO:0006355|	GO:0005634|	GO:0003677|D	.
Manes.01G19830(	11.111388	3.30E-12	up	yes	.	pfam10080	DUF2318	Predicted m	.	.	.	.
Manes.01G19860(	0.660665	0.00161	down	no	30S ribosom	pfam00886	Ribosomal_S16	Ribosomal p	GO:0006412|	GO:0005840|	GO:0003735|st	K02959
Manes.01G19870(	1.1536125	0.11237	up	no	.	pfam01436	NHL	NHL repeat	.	.	.	.
Manes.01G19880(	11.601081	1.65E-10	up	yes	.	pfam08524	rRNA_processing	rRNA proce	.	.	.	.
Manes.01G19890(	6.3682884	6.61E-40	up	yes	Probable alph	pfam00982	Glyco_transf_20	Glycosyltran	GO:0005992|	.	GO:0016757|tra	K16055
Manes.01G19910(	0.7640883	0.01858	down	no	Bet1-like SN	pfam05739	SNARE	SNARE dom	GO:0015031|	GO:0005789|	.	K08504
Manes.01G19980(	NA	NA	--	no	.	pfam05095	DUF687	Protein of u	.	.	.	.
Manes.01G20000(	2.5033018	3.86E-25	up	yes	Cytochrome	pfam00067	p450	Cytochrome	.	GO:0005789|	GO:0020037|he	.
Manes.01G20010(	0.567968	8.56E-05	down	no	.	pfam04576	Zein-binding	Zein-binding	.	.	.	.
Manes.01G20040(	1.1844851	0.17926	up	no	.	pfam00013	KH_1	KH domain.	.	.	.	.
Manes.01G20080(	0.652018	8.75E-05	down	no	Probable WR	pfam03106	WRKY	WRKY DN	GO:0006351|	GO:0005634|	GO:0005516|ca	.
Manes.01G20110(	2.312937	1.08E-13	up	yes	PRA1 family	pfam03208	PRA1	PRA1 family	GO:0016192|	GO:0005783|	.	.
Manes.01G20130(	0.3070034	1.94E-14	down	yes	Magnesium t	pfam05856	ARPC4	ARP2/3 con	.	GO:0016021|	GO:0015095|m	.
Manes.01G20140(	0.8721281	0.29976	down	no	DNA-directe	pfam08292	RNA_pol_Rbc25	RNA polym	GO:0051607|	GO:0005813|	GO:0003677|D	K03022
Manes.01G20150(	0.4731499	5.46E-08	down	yes	F-box/LRR-r	pfam00859	CTF_NFI	CTF/NF-I f	GO:0009793|	GO:0001673|	.	.
Manes.01G20170(	4.093131	9.78E-56	up	yes	Auxin-induce	pfam02309	AUX_IAA	AUX/IAA f	GO:0009734|	GO:0005634|	.	.
Manes.01G20180(	0.8723501	0.18836	down	no	NADH dehyc	pfam01257	2Fe-2S_thioredx	Thioredoxin	GO:0006979|	GO:0005747|	GO:0051537|2	K03943
Manes.01G20190(	1.8102948	3.20E-10	up	no	Transcription	pfam11804	DUF3325	Protein of u	GO:0009567|	GO:0005634|	GO:0003677|D	.

Manes.01G20230(	0.9793821	0.83016	down	no	Histone acety	pfam00439	Bromodomain	Bromodoma	GO:0009908|]	GO:0000123|	GO:0003677|D]	K06062
Manes.01G20260(	0.9026505	0.3767	down	no	Guanylate-bi	pfam02263	GBP	Guanylate-b	.	GO:0005737|	GO:0005525|G'	.
Manes.01G20310(	0.6492321	0.00288	down	no	.	pfam14309	DUF4378	Domain of u	.	.	.	.
Manes.01G20320(	0.8412961	0.08498	down	no	.	pfam03735	ENT	ENT domai	.	.	.	.
Manes.01G20330(	0.7031769	0.00056	down	no	2-methyl-6-p	pfam08241	Methyltransf_11	Methyltrans	GO:0010189|	GO:0009706|	GO:0008168|m	.
Manes.01G20370(	2.4766952	1.13E-07	up	yes	Pentatricope	pfam14432	DYW_deaminase	DYW family	GO:0006397|	GO:0009507|	.	.
Manes.01G20390(	0.5024955	4.05E-05	down	no	.	pfam04762	IKI3	IKI3 family	.	.	.	.
Manes.01G20450(	1.6081287	0.93871	up	no	Chlorophylla	pfam07224	Chlorophyllase	Chlorophyll	GO:0015996|	.	GO:0047746|ch	.
Manes.01G20460(	0.8855047	0.59824	down	no	BI1-like prot	pfam01027	Bax1-I	Inhibitor of	.	GO:0016021|	.	K06890
Manes.01G20470(	1.258573	0.35788	up	no	.	pfam03171	2OG-FeII_Oxy	2OG-Fe(II)	.	.	.	.
Manes.01G20500(	0.8568365	0.66092	down	no	.	pfam06681	DUF1182	Protein of u	.	.	.	.
Manes.01G20520(	1.8944831	1.53E-11	up	no	TP53-regulat	pfam00069	Pkinase	Protein kina	GO:0006468|	GO:0005829|	GO:0005524|A'	K08851
Manes.01G20530(	4.00094	2.80E-07	up	yes	CST comple	pfam01336	tRNA_anti-codon	OB-fold nuc	GO:0016233|	GO:0000784|	GO:0003677|D]	.
Manes.01G20590(	4.6353273	0.00031	up	yes	.	pfam02724	CDC45	CDC45-like	.	.	.	.
Manes.01G20600(	4.0782789	5.42E-50	up	yes	.	pfam04570	DUF581	Protein of u	.	.	.	.
Manes.01G20630(	7.3856264	6.58E-13	up	yes	.	pfam12051	DUF3533	Protein of u	.	.	.	.
Manes.01G20650(	0.3486411	3.58E-06	down	yes	Transcription	pfam04844	Ovate	Transcriptio	GO:0045892|	GO:0005634|	.	.
Manes.01G20680(	0.4620642	0.00618	down	yes	.	pfam06047	SynMuv_product	Ras-induced	.	.	.	.
Manes.01G20790(	2.2391965	0.00024	up	yes	.	pfam11250	DUF3049	Protein of u	.	.	.	.
Manes.01G20800(	5.2584971	0.02763	up	yes	.	pfam05210	Sprouty	Sprouty pro	.	.	.	.
Manes.01G20810(	0.8212622	0.17491	down	no	Pentatricope	pfam13041	PPR_2	PPR repeat	.	GO:0005739|	.	.
Manes.01G20860(	2.1635312	2.08E-14	up	yes	Protein trich	pfam13839	PC-Esterase	GDSL/SGN	.	GO:0016021|	.	.
Manes.01G20870(	1.2783304	0.03143	up	no	.	pfam04114	Gaa1	Gaa1-like, C	.	.	.	.
Manes.01G20880(	2.5512045	8.32E-08	up	yes	.	pfam10136	SpecificRecomb	Site-specific	.	.	.	.
Manes.01G20910(	0.7775346	0.66124	down	no	Adenylate isc	pfam01715	IPPT	IPP transfer	GO:0009691|	GO:0009507|	GO:0052623|A]	K10760
Manes.01G20930(	0.4150453	0.00011	down	yes	.	pfam13340	DUF4096	Putative tra	.	.	.	.
Manes.01G20950(	1.4535211	3.05E-05	up	no	.	pfam00213	OSCP	ATP syntha	.	.	.	.
Manes.01G20960(	2.5522352	7.63E-13	up	yes	.	pfam08137	DVL	DVL family	.	.	.	.
Manes.01G20970(	1.2088978	0.04525	up	no	Probable glut	pfam00255	GSHPx	Glutathione	GO:0006979|	GO:0005829|	GO:0004602|gl	K00432
Manes.01G20980(	0.6445	9.64E-06	down	no	HUA2-like p	pfam09726	Macoilin	Transmemb	GO:0006397|	GO:0005634|	.	.
Manes.01G20990(	0.5821957	0.0194	down	no	CSC1-like pr	pfam02714	DUF221	Domain of u	GO:0006811|	GO:0016021|	.	.
Manes.01G21000(	0.4755044	9.74E-09	down	yes	Protein PALI	pfam09444	MRC1	MRC1-like	GO:0071482|	GO:0009501|	.	.
Manes.01G21010(	0.8513078	0.1086	down	no	Mediator of I	pfam03035	RNA_capsid	Calicivirus p	GO:0009698|	GO:0016592|	.	.
Manes.01G21020(	11.276726	7.52E-14	up	yes	.	pfam13321	DUF4084	Domain of u	.	.	.	.
Manes.01G21050(	0.567989	1.05E-05	down	no	.	pfam03143	GTP_EFTU_D3	Elongation f	.	.	.	.
Manes.01G21060(	1.1026584	0.41823	up	no	Elongation fa	pfam00009	GTP_EFTU	Elongation f	GO:0046686|	GO:0005618|	GO:0005524|A'	K02358
Manes.01G21100(	0.7941875	0.25757	down	no	.	pfam03171	2OG-FeII_Oxy	2OG-Fe(II)	.	.	.	.
Manes.01G21110(	0.6319774	0.01321	down	no	.	pfam03909	BSD	BSD domai	.	.	.	.
Manes.01G21130(	0.4764318	2.29E-08	down	yes	Auxin-bindin	pfam02041	Auxin_BP	Auxin bindi	GO:0009734|	GO:0005788|	GO:0010011|au	.
Manes.01G21160(	10.00883	0.02845	up	yes	Probable indc	pfam03321	GH3	GH3 auxin-	GO:0009733|	.	GO:0016874|lig	.
Manes.01G21190(	0.6084632	1.16E-05	down	no	Autophagy-r	pfam12490	BCAS3	Breast carci	GO:0006914|	GO:0005737|	.	.
Manes.01G21230(	2.6845914	1.13E-20	up	yes	Gibberellin r	pfam07859	Abhydrolase_3	alpha/beta h	GO:0048444|	GO:0005634|	GO:0016787|hy	K14493
Manes.01G21240(	1.0792718	0.39198	up	no	Serine/threon	pfam00149	Metallophos	Calcineurin-	GO:0009742|	GO:0005634|	GO:0046872|m	K01090
Manes.01G21250(	1.0208638	0.80429	up	no	Sister-chrom	pfam08514	STAG	STAG doma	GO:0051455|	GO:0005694|	.	K06671
Manes.01G21260(	0.1837068	8.36E-10	down	yes	Rho GTPase-	pfam00620	RhoGAP	RhoGAP do	GO:0007165|	.	GO:0005096|G'	.
Manes.01G21290(	3.7691737	2.44E-06	up	yes	.	pfam12036	DUF3522	Protein of u	.	.	.	.
Manes.01G21300(	1.3559098	0.01406	up	no	CUGBP Elav	pfam00076	RRM_1	RNA recog	GO:0006397|	GO:0005737|	GO:0000166|nu	K13207
Manes.01G21310(	1.4540295	9.14E-05	up	no	U1 small nuc	pfam06220	zf-U1	U1 zinc fing	GO:0000395|	GO:0000243|	GO:0003729|m	K11095
Manes.01G21330(	0.3800687	2.34E-13	down	yes	Secoisolarici	pfam00106	adh_short	short chain	.	.	GO:0016491|ox	.
Manes.01G21340(	0.5107216	2.31E-05	down	no	Secoisolarici	pfam00106	adh_short	short chain	.	.	GO:0016491|ox	.
Manes.01G21370(	1.3289375	0.00165	up	no	Protease Do-	pfam00089	Trypsin	Trypsin.	GO:0009658|	GO:0009507|	GO:0004252|se	.
Manes.01G21390(	0.6854431	0.04526	down	no	.	pfam07765	KIP1	KIP1-like p	.	.	.	.
Manes.01G21400(	2.0277022	4.64E-07	up	yes	Sodium/hydr	pfam00999	Na_H_Exchanger	Sodium/hyd	GO:0055075|	GO:0016021|	GO:0015386|pc	.
Manes.01G21410(	0.6865994	0.00036	down	no	Tubby-like F	pfam01167	Tub	Tub family.	.	GO:0005654|	.	.
Manes.01G21430(	1.2267921	0.02639	up	no	Protein TRA	pfam12937	F-box-like	F-box-like.	GO:0009734|	GO:0005634|	GO:0010011|au	K14485
Manes.01G21460(	0.0724739	1.09E-10	down	yes	Membrane-a	pfam13881	Rad60-SLD_2	Ubiquitin-2	.	GO:0005886|	.	.
Manes.01G21480(	5.0576852	4.66E-38	up	yes	Glutaredoxin	pfam00462	Glutaredoxin	Glutaredoxi	GO:0045454|	GO:0005737|	GO:0009055|el	.
Manes.01G21520(	0.6588428	0.01172	down	no	.	pfam15100	TMEM187	TMEM187	.	.	.	.
Manes.01G21530(	0.6193512	0.00067	down	no	BTB/POZ do	pfam03000	NPH3	NPH3 famil	GO:0009860|	.	.	.
Manes.01G21600(	0.9434106	0.63645	down	no	.	pfam15051	FAM198	FAM198 pr	.	.	.	.
Manes.01G21610(	2.4332934	1.81E-23	up	yes	Autophagy-r	pfam00400	WD40	WD domain	GO:0048102|	GO:0005829|	GO:0080025|ph	.
Manes.01G21620(	24.416736	0.00324	up	yes	Glutathione S	pfam00043	GST_C	Glutathione	GO:0009636|	GO:0005737|	GO:0004364|gl	K00799
Manes.01G21650(	0.6887647	0.01085	down	no	.	pfam11933	DUF3451	Domain of u	.	.	.	.
Manes.01G21660(	1.0604041	0.51803	up	no	Bifunctional	pfam02882	THF_DHG_CYH	Tetrahydrof	GO:0009396|	GO:0009507|	GO:0004477|m	.
Manes.01G21680(	0.3716183	8.80E-12	down	yes	Putative indo	pfam03321	GH3	GH3 auxin-	.	GO:0009941|	GO:0016874|lig	K14487
Manes.01G21700(	6.5089155	6.86E-08	up	yes	Protein RAL	pfam05498	RALF	Rapid ALka	GO:0019722|	GO:0048046|	GO:0005179|ho	.
Manes.01G21750(	0.7555123	0.00497	down	no	DNA mismat	pfam01119	DNA_mis_repair	DNA misma	GO:0006310|	GO:0032389|	GO:0005524|A'	K10858
Manes.01G21760(	0.5463558	0.00011	down	no	Probable U6	pfam01423	LSM	LSM domai	GO:0006397|	GO:0005681|	GO:0003723|R]	.
Manes.01G21770(	1.5046693	2.10E-05	up	no	.	pfam00400	WD40	WD domain	.	.	.	.
Manes.01G21810(	1.1156237	0.35652	up	no	Universal stre	pfam00582	Usp	Universal st	GO:0006950|	GO:0005886|	GO:0016208|A]	.
Manes.01G21820(	0.8737218	0.28256	down	no	Beta-glucosic	pfam00232	Glyco_hydro_1	Glycosyl hy	GO:0005975|	.	GO:0008422|be	K01188
Manes.01G21830(	0.7307923	0.00297	down	no	Serine/argini	pfam00076	RRM_1	RNA recog	GO:0006397|	GO:0035061|	GO:0000166|nu	K12890
Manes.01G21860(	1.9505612	3.97E-13	up	no	.	pfam07223	DUF1421	Protein of u	.	.	.	.
Manes.01G21890(	2.1552873	0.03021	up	no	.	pfam14897	EpsG	EpsG family	.	.	.	.
Manes.01G21960(	0.8188893	0.13856	down	no	Magnesium/p	pfam01699	Na_Ca_ex	Sodium/calc	GO:0006811|	GO:0016021|	.	K03452
Manes.01G21970(	0.6770349	7.55E-05	down	no	Blue-light ph	pfam00875	DNA_photolyase	DNA photo	GO:0006281|	.	GO:0003913|D]	.
Manes.01G22040(	183.34709	0	up	yes	Indole-3-acet	pfam03242	LEA_3	Late embryo	GO:0006950|	.	.	.
Manes.01G22050(	1.2772148	0.02651	up	no	.	pfam04398	DUF538	Protein of u	.	.	.	.
Manes.01G22080(	0.3939526	7.60E-07	down	yes	.	pfam04398	DUF538	Protein of u	.	.	.	.
Manes.01G22100(	1.8300545	0.0297	up	no	.	pfam04398	DUF538	Protein of u	.	.	.	.
Manes.01G22140(	0.3683617	1.64E-08	down	yes	Bifunctional	pfam12697	Abhydrolase_6	Alpha/beta l	GO:0019369|	GO:0005737|	GO:0033885|1(	K08726
Manes.01G22150(	9.3693945	1.99E-53	up	yes	RING-H2 fin	pfam13639	zf-RING_2	Ring finger	GO:0006461|	GO:0016021|	GO:0008270|zi	.
Manes.01G22160(	25.817656	1.57E-44	up	yes	Probable pec	pfam01095	Pectinesterase	Pectinestera	GO:0042545|	GO:0005618|	GO:0045330|as	K01051
Manes.01G22170(	0.2652657	2.78E-16	down	yes	Probable pec	pfam01095	Pectinesterase	Pectinestera	GO:0042545|	GO:0005618|	GO:0045330|as	K01051
Manes.01G22190(	1.0992208	0.33092	up	no	Sucrose synt	pfam00862	Sucrose_synth	Sucrose syn	GO:0009058|	.	GO:0016157|su	.
Manes.01G22200(	3.0005235	4.91E-20	up	yes	.	pfam00232	Glyco_hydro_1	Glycosyl hy	.	.	.	.
Manes.01G22230(	Inf	5.09E-06	up	yes	Proline-rich p	pfam01190	Pollen_Ole_e_I	Pollen prote	.	GO:0005618|	.	.
Manes.01G22260(	18.11566	7.58E-59	up	yes	Ethylene-res	pfam00847	AP2	AP2 domain	GO:0008219|	GO:0005737|	GO:0003677|D]	K09286

Manes.01G22350(	0.5683517	5.55E-07	down	no	Nicotinamide	pfam00153	Mito_carr	Mitochondr	GO:0043132|	GO:0031969|	GO:0015297|an	K15115
Manes.01G22360(	0.637157	0.00036	down	no	Cytidine dea	pfam08211	dCMP_cyt_deam_	Cytidine and	GO:0009972|	GO:0005829|	GO:0004126|cy	K01489
Manes.01G22410(	0.7886533	0.03196	down	no	.	pfam04941	LEF-8	Late express	.	.	.	.
Manes.01G22450(	0.4221723	4.34E-12	down	yes	.	pfam13555	AAA_29	P-loop cont	.	.	.	.
Manes.01G22460(	5.7875728	0.00739	up	yes	Importin subu	pfam01749	IBB	Importin bet	GO:0006606|	GO:0005737|	GO:0008565|pr	.
Manes.01G22500(	0.4763167	0.0001	down	yes	Kynurenine--	pfam00155	Aminotran_1_2	Aminotransf	GO:0009058|	GO:0005737|	GO:0047804|cy	K00816
Manes.01G22520(	0.5096212	3.79E-11	down	no	Protein FAR1	pfam03101	FAR1	FAR1 DNA	GO:0006355|	GO:0005634|	GO:0008270|zi	.
Manes.01G22530(	0.2551079	0.00061	down	yes	Zinc finger pr	pfam13912	zf-C2H2_6	C2H2-type	GO:0009736|	GO:0022626|	GO:0046872|m	.
Manes.01G22560(	11.50255	1.74E-15	up	yes	.	pfam05553	DUF761	Cotton fibre	.	.	.	.
Manes.01G22570(	0.7087943	0.00369	down	no	CTP synthase	pfam06418	CTP_synth_N	CTP syntha	GO:0044210|	.	GO:0005524|A	K01937
Manes.01G22580(	0.3139175	2.79E-05	down	yes	.	pfam06273	eIF-4B	Plant specifi	.	.	.	.
Manes.01G22600(	0.4679777	9.76E-05	down	yes	.	pfam15449	Retinal	Retinal prot	.	.	.	.
Manes.01G22630(	0.6923052	0.00237	down	no	DnaJ protein	pfam00226	DnaJ	DnaJ domai	GO:0052033|	GO:0005783|	.	K09517
Manes.01G22640(	0.9311283	0.67504	down	no	.	pfam01764	Lipase_3	Lipase (clas	.	.	.	.
Manes.01G22650(	1.1615109	0.11242	up	no	Transmembra	pfam13664	DUF4149	Domain of u	.	GO:0016021|	.	.
Manes.01G22660(	0.8283593	0.39825	down	no	BRCT domai	pfam12738	PTCB-BRCT	twin BRCT	.	.	.	K10728
Manes.01G22710(	5.7110804	8.78E-76	up	yes	Probable E3	pfam12428	DUF3675	Protein of u	GO:0042335|	GO:0016021|	GO:0016874|lig	K10661
Manes.01G22730(	0.581152	3.46E-05	down	no	Pentatricope	pfam13041	PPR_2	PPR repeat	.	GO:0005739|	.	.
Manes.01G22740(	1.8902579	4.05E-09	up	no	Probable dim	pfam00398	RrnaAD	Ribosomal	.	GO:0005737|	GO:0052909|18	K14191
Manes.01G22750(	1.0691668	0.46147	up	no	PH-interactin	pfam00439	Bromodomain	Bromodoma	GO:0007010|	GO:0070062|	GO:0005158|in	K11797
Manes.01G22760(	0.4432568	2.21E-07	down	yes	DNA replicat	pfam00493	MCM	MCM2/3/5	GO:0007049|	GO:0005737|	GO:0005524|A	K02210
Manes.01G22770(	0.8354401	0.06035	down	no	.	pfam15469	Sec5	Exocyst con	.	.	.	.
Manes.01G22790(	2.4742591	2.32E-19	up	yes	Ocs element-	pfam00170	bZIP_1	bZIP transc	GO:0006351|	GO:0005634|	GO:0043565|se	.
Manes.01G22830(	0.6594714	4.61E-05	down	no	Histone-lysin	pfam03638	TCR	Tesmin/TSC	GO:0006349|	GO:0005677|	GO:0018024|his	K11430
Manes.01G22870(	Inf	0.9009	up	no	.	pfam04043	PMEI	Plant inverta	.	.	.	.
Manes.01G22890(	0.9608732	0.71065	down	no	DEAD-box A	pfam00270	DEAD	DEAD/DEA	.	.	GO:0005524|A	K12835
Manes.01G22900(	0.5437772	2.16E-07	down	no	Pentatricope	pfam01535	PPR	PPR repeat.	.	GO:0005739|	.	.
Manes.01G22910(	3.5547571	1.07E-34	up	yes	Peptidyl-prol	pfam00160	Pro_isomerase	Cyclophilin	GO:0006457|	GO:0005768|	GO:0042277|pe	.
Manes.01G22980(	1.6240222	0.32822	up	no	Stachyose sy	pfam05691	Raffinose_syn	Raffinose sy	GO:0009312|	GO:0005737|	GO:0047268|ga	.
Manes.01G23000(	6.6083519	2.60E-16	up	yes	Probable WR	pfam03106	WRKY	WRKY DN	GO:0009733|	GO:0005634|	GO:0043565|se	.
Manes.01G23010(	0.8427165	0.10602	down	no	Flowering tin	pfam00076	RRM_1	RNA recogi	GO:0030154|	GO:0005634|	GO:0000166|nu	.
Manes.01G23020(	0.8820887	0.20853	down	no	.	pfam13868	Trichoplein	tumor suppr	.	.	.	.
Manes.01G23040(	Inf	#######	up	yes	Probable glyc	pfam01553	Acyltransferase	Acyltransfer	GO:0016024|	GO:0016021|	GO:0004366|gl	K13508
Manes.01G23050(	0.92274	0.42016	down	no	Probable pre-	pfam04408	HA2	Helicase ass	GO:0006397|	GO:0009941|	GO:0005524|A	K12820
Manes.01G23080(	0.7469849	0.00273	down	no	.	pfam05266	DUF724	Protein of u	.	.	.	.
Manes.01G23200(	0.5344723	0.00146	down	no	Probable bor	pfam00955	HCO3_cotransp	HCO3- tran	GO:0035445|	GO:0005887|	GO:0080139|bo	.
Manes.01G23240(	7.5680609	6.74E-16	up	yes	Polygalactur	pfam00295	Glyco_hydro_28	Glycosyl hy	GO:0005975|	GO:0005618|	GO:0004650|pc	.
Manes.01G23250(	2.1653403	3.91E-18	up	yes	Shaggy-relat	pfam00069	Pkinase	Protein kina	GO:0042538|	GO:0005829|	GO:0005524|A	K00924
Manes.01G23260(	6.3698794	9.78E-99	up	yes	Probable prot	pfam00481	PP2C	Protein phos	GO:0006470|	.	GO:0046872|m	K14803
Manes.01G23270(	9.0909871	0.00059	up	yes	Short-chain d	pfam00106	adh_short	short chain	.	.	GO:0016491|ox	.
Manes.01G23290(	1.3374596	0.00176	up	no	Ubiquitin-40S	pfam00240	ubiquitin	Ubiquitin fa	GO:0006412|	GO:0005634|	GO:0046872|m	.
Manes.01G23300(	4.3192245	2.29E-20	up	yes	Protein TolB	pfam00930	DPPIV_N	Dipeptidyl p	GO:0017038|	GO:0042597|	.	K03641
Manes.01G23310(	0.5804009	0.00175	down	no	Autophagy-r	pfam02991	Atg8	Autophagy	GO:0000045|	GO:0000421|	GO:0019779|A	K08341
Manes.01G23350(	1.4692507	2.04E-05	up	no	Pto-interacti	pfam07714	Pkinase_Tyr	Protein tyro	GO:0009626|	.	GO:0005524|A	K13436
Manes.01G23360(	2.4886835	5.57E-23	up	yes	Meiosis arres	pfam01936	NYN	NYN domai	GO:0051321|	GO:0005777|	GO:0000166|nu	.
Manes.01G23370(	0.4812145	9.37E-13	down	yes	.	pfam04937	DUF659	Protein of u	.	.	.	.
Manes.01G23390(	36.838122	#######	up	yes	.	pfam05678	VQ	VQ motif. T	.	.	.	.
Manes.01G23400(	0.3098519	0.02048	down	yes	Protein RAD	pfam00249	Myb_DNA-binding	Myb-like D	GO:0006355|	GO:0005634|	GO:0003677|D	.
Manes.01G23470(	0.2651907	1.36E-17	down	yes	.	pfam01257	2Fe-2S_thioredx	Thioredoxin	.	.	.	.
Manes.01G23480(	2.8965564	5.02E-29	up	yes	Probable leu	pfam11721	Malectin	Di-glucose	.	GO:0016021|	GO:0005524|A	.
Manes.01G23490(	0.8061268	0.0848	down	no	L-cysteine de	pfam00266	Aminotran_5	Aminotransf	GO:0019450|	GO:0005829|	GO:0080146|L-	.
Manes.01G23520(	1.3636155	0.00266	up	no	Probable poly	pfam00295	Glyco_hydro_28	Glycosyl hy	GO:0005975|	GO:0016021|	GO:0004650|pc	.
Manes.01G23530(	0.476116	0.00011	down	yes	Protein S-acy	pfam01529	zf-DHHC	DHHC palm	.	GO:0030659|	GO:0019706|pr	K18932
Manes.01G23550(	1.3107711	0.54444	up	no	Auxin-respor	pfam02309	AUX_IAA	AUX/IAA f	GO:0009734|	GO:0005634|	GO:0003700|tra	K14484
Manes.01G23560(	1.3510087	0.54952	up	no	Cytochrome	pfam00067	p450	Cytochrome	GO:0010268|	GO:0016021|	GO:0020037|he	.
Manes.01G23580(	0.3974454	1.49E-13	down	yes	Transcription	pfam00249	Myb_DNA-binding	Myb-like D	GO:0006355|	GO:0005634|	GO:0003677|D	K09422
Manes.01G23640(	0.5225398	3.43E-08	down	no	Protein phosp	pfam00481	PP2C	Protein phos	GO:0010074|	GO:0005634|	GO:0046872|m	.
Manes.01G23660(	NA	NA	--	no	Putative expa	pfam01357	Pollen_allerg_1	Pollen allerg	GO:0009664|	GO:0005618|	.	.
Manes.01G23670(	0.5051266	0.01317	down	no	Glucose-1-ph	pfam00483	NTP_transferase	Nucleotidyl	GO:0005978|	GO:0009507|	GO:0005524|A	K00975
Manes.01G23690(	1.1933944	0.05413	up	no	Cathepsin B	pfam00112	Peptidase_C1	Papain famil	GO:0097067|	GO:0070062|	GO:0004197|cy	K01363
Manes.01G23720(	1.6072846	1.60E-06	up	no	Tricin syntha	pfam01596	Methyltransf_3	O-methyltra	GO:0032259|	GO:0005634|	GO:0046872|m	K18883
Manes.01G23730(	4.8283382	3.12E-05	up	yes	.	pfam11971	CAMSAP_CH	CAMSAP C	.	.	.	.
Manes.01G23760(	27.098258	4.16E-89	up	yes	Uncharacteriz	pfam06966	DUF1295	Protein of u	GO:0055114|	GO:0005783|	GO:0016627|ox	.
Manes.01G23770(	0.8266782	0.27612	down	no	Probable inac	pfam00149	Metallophos	Calcineurin-	GO:0016311|	GO:0005576|	GO:0003993|ac	.
Manes.01G23780(	2.060804	5.08E-07	up	yes	.	pfam00050	Kazal_1	Kazal-type	.	.	.	.
Manes.01G23810(	4.0512959	2.00E-13	up	yes	NAC domain	pfam02365	NAM	No apical m	GO:0090603|	GO:0005634|	GO:0003700|tra	.
Manes.01G23850(	0.3562695	0.00053	down	yes	.	pfam05309	TraE	TraE protei	.	.	.	.
Manes.01G23890(	4.8750396	7.40E-18	up	yes	Soluble inorg	pfam00719	Pyrophosphatase	Inorganic py	GO:0006796|	GO:0005737|	GO:0004427|in	.
Manes.01G23900(	0.5821603	2.19E-05	down	no	Protein LHY	pfam00249	Myb_DNA-binding	Myb-like D	GO:0007623|	GO:0005634|	GO:0003700|tra	K12133
Manes.01G24000(	1.0047854	0.94231	up	no	Vacuolar pro	pfam12816	Vps8	Golgi COR	.	GO:0033263|	GO:0008270|zi	.
Manes.01G24020(	1.7284908	1.64E-08	up	no	.	pfam13041	PPR_2	PPR repeat	.	.	.	.
Manes.01G24040(	5.9898484	9.23E-68	up	yes	RNA-binding	pfam00076	RRM_1	RNA recogi	GO:0070935|	GO:0005829|	GO:0003730|m	.
Manes.01G24090(	0.9514662	0.63349	down	no	Heterogeneo	pfam00076	RRM_1	RNA recogi	GO:0007623|	GO:0070937|	GO:0000166|nu	K13160
Manes.01G24100(	3.9411483	0.00807	up	yes	3-ketoacyl-C	pfam08392	FAE1_CUT1_Rpp	FAE1/Type	GO:0006633|	GO:0022626|	GO:0009922|fa	K15397
Manes.01G24140(	0.7060737	0.00098	down	no	GRF1-intera	pfam05030	SSXT	SSXT prote	GO:0008283|	GO:0005634|	GO:0003713|tra	.
Manes.01G24150(	5.5867839	1.83E-14	up	yes	.	pfam13578	Methyltransf_24	Methyltrans	.	.	.	.
Manes.01G24170(	4.5509808	3.26E-07	up	yes	Transcription	pfam14215	bHLH-MYC_N	bHLH-MY	GO:0042538|	GO:0005634|	GO:0005509|ca	.
Manes.01G24180(	0.7144409	0.00252	down	no	CDPK-relate	pfam00069	Pkinase	Protein kina	GO:0009738|	GO:0005737|	GO:0005524|A	.
Manes.01G24220(	0.377404	3.76E-08	down	yes	.	pfam10325	7TM_GPCR_Srz	Serpentine t	.	.	.	.
Manes.01G24230(	4.9752983	1.35E-37	up	yes	Homeobox-le	pfam00046	Homeobox	Homeobox	GO:0045893|	GO:0005634|	GO:0043565|se	.
Manes.01G24240(	0.4571239	0.006	down	yes	Cytochrome	pfam00067	p450	Cytochrome	.	.	GO:0020037|he	.
Manes.01G24300(	1.9038119	1.83E-06	up	no	Ras-related p	pfam00071	Ras	Ras family.	GO:0006886|	GO:0009504|	GO:0005525|G	.
Manes.01G24320(	1.4450633	0.0002	up	no	Serine/argini	pfam00076	RRM_1	RNA recogi	GO:0000398|	GO:0016607|	GO:0000166|nu	K12893
Manes.01G24350(	1.0167594	0.90771	up	no	DNA-directe	pfam01096	TFIIS_C	Transcriptio	GO:0051607|	GO:0005829|	GO:0003677|D	K03019
Manes.01G24360(	0.8484534	0.11447	down	no	Bifunctional	pfam07959	Fucokinase	L-fucokinas	GO:0005975|	GO:0005737|	GO:0005524|A	K05305
Manes.01G24390(	2.2375412	2.20E-15	up	yes	ORM1-like p	pfam04061	ORMDL	ORMDL fa	GO:0006672|	GO:0005789|	.	.
Manes.01G24430(	0.738086	0.04527	down	no	.	pfam07817	GLE1	GLE1-like	.	.	.	.
Manes.01G24440(	0.6549128	0.00299	down	no	Kinesin light	pfam13424	TPR_12	Tetratricope	GO:0019886|	GO:0030424|	GO:0003777|mi	K10407

Manes.01G24450(	1.055647	0.55236	up	no	.	pfam12234	Rav1p_C	RAVE prote	.	.	.	.
Manes.01G24470(	1.1167651	0.22843	up	no	.	pfam14897	EpsG	EpsG family	.	.	.	.
Manes.01G24490(	0.5337337	5.84E-07	down	no	Uncharacteri:	pfam05097	DUF688	Protein of u	GO:0009793|(	.	GO:0003700|tra	.
Manes.01G24550(	0.4718009	0.06785	down	no	.	pfam11412	DsbC	Disulphide b	.	.	.	.
Manes.01G24560(	3.6242397	1.67E-27	up	yes	Transcription	pfam14215	bHLH-MYC_N	bHLH-MYC	GO:0006355|r	GO:0005634|	GO:0003700|tra	.
Manes.01G24580(	1.5897425	2.38E-07	up	no	Protein ASPA	pfam14541	TAXi_C	Xylanase inl	GO:0030163|p	GO:0005783|	GO:0004190|as	.
Manes.01G24590(	0.7739214	0.24505	down	no	.	pfam00501	AMP-binding	AMP-bindin	.	.	.	.
Manes.01G24600(	0.6234328	0.0008	down	no	E3 ubiquitin-	pfam03145	Sina	Seven in abs	GO:0007275|r	GO:0005634|	GO:0016874|lig	K04506
Manes.01G24610(	0.407262	1.33E-11	down	yes	E3 ubiquitin-	pfam03145	Sina	Seven in abs	GO:0007275|r	GO:0005634|	GO:0016874|lig	K04506
Manes.01G24670(	1.1373046	0.16605	up	no	Beclin-1-like	pfam04111	APG6	Autophagy p	GO:0000045|a	GO:0016023|	.	.
Manes.01G24700(	0.5936509	6.78E-06	down	no	.	pfam09713	A_thal_3526	Plant protein	.	.	.	.
Manes.01G24730(	1.9105353	2.70E-13	up	no	Abscisic acid	pfam10604	Polyketide_cyc2	Polyketide c	GO:0009738|a	GO:0005737|	GO:0010427|ab	K14496
Manes.01G24740(	2.7322512	2.92E-23	up	yes	Protein ROO	pfam11955	PORR	Plant organe	GO:0010102|l	.	.	.
Manes.01G24760(	1.5704205	0.01128	up	no	Transcription	pfam00249	Myb_DNA-binding	Myb-like DI	GO:0007275|r	GO:0005634|	GO:0003677|DI	K09422
Manes.01G24800(	3.2235613	1.48E-06	up	yes	Protein tricho	pfam13839	PC-Esterase	GDSL/SGN	.	GO:0016021|	.	.
Manes.01G24810(	1.060802	0.53223	up	no	.	pfam11331	DUF3133	Protein of u	.	.	.	.
Manes.01G24850(	0.8410608	0.20383	down	no	Mitochondria	pfam00153	Mito_carr	Mitochondri	GO:0055085|t	GO:0016021|	.	.
Manes.01G24880(	0.8402576	0.37708	down	no	Light-sensor	pfam06760	DUF1221	Protein of u	GO:0009584|(	GO:0005622|	GO:0005524|A	.
Manes.01G24890(	0.4758558	5.57E-07	down	yes	.	pfam03542	Tuberin	Tuberin. Tu	.	.	.	.
Manes.01G24960(	1.5370735	0.01652	up	no	Ethylene-resp	pfam00847	AP2	AP2 domain	GO:0009736|(	GO:0005737|	GO:0003677|DI	.
Manes.01G24970(	1.6374157	7.58E-06	up	no	Exosome con	pfam01138	RNase_PH	3' exoribonu	GO:0051607|(	GO:0005737|	GO:0000175|3'-	K11600
Manes.01G24980(	0.7837516	0.14921	down	no	Putative gluc	pfam01263	Aldose_epim	Aldose 1-ep	GO:0005975|(	.	GO:0030246|ca	.
Manes.01G24990(	0.6933324	0.00069	down	no	Common plai	pfam07777	MFMR	G-box bindi	GO:0006351|t	GO:0005634|	GO:0043565|se	.
Manes.01G25000(	0.6883606	0.0039	down	no	GDSL estera	pfam00657	Lipase_GDSL	GDSL-like l	GO:0016042|l	GO:0005576|	GO:0016788|hy	.
Manes.01G25010(	1.3187408	0.01079	up	no	Protein CUR	pfam14159	CAAD	CAAD dom	.	GO:0009507|	.	.
Manes.01G25020(	0.9168518	0.40777	down	no	BTB/POZ do	pfam07707	BACK	BTB And C	GO:0016567|p	GO:0005634|	GO:0046982|pr	.
Manes.01G25040(	0.546549	2.77E-06	down	no	Uncharacteri:	pfam13851	GAS	Growth-arre	.	GO:0009507|	.	.
Manes.01G25060(	3.2314525	3.94E-26	up	yes	F-box/kelch-r	pfam00646	F-box	F-box doma	GO:0031571|r	GO:0019005|	GO:0004842|ub	.
Manes.01G25100(	0.6837823	0.0002	down	no	.	pfam00534	Glycos_transf_1	Glycosyl tra	.	.	.	.
Manes.01G25140(	4.0759164	4.71E-28	up	yes	Delta(8)-fatty	pfam00487	FA_desaturase	Fatty acid de	GO:0070417|(	GO:0005783|	GO:0046872|me	K13076
Manes.01G25150(	0.3966665	2.33E-09	down	yes	Probable mer	pfam03805	CLAG	Cytoadherer	GO:0009738|a	GO:0005886|	.	.
Manes.01G25160(	0.7407073	0.0254	down	no	U11/U12 sma	pfam13853	7tm_4	Olfactory re	GO:0006397|r	GO:0005681|	.	.
Manes.01G25180(	3.2639679	1.68E-14	up	yes	RING-H2 fin	pfam13639	zf-RING_2	Ring finger (	GO:0016567|p	GO:0016021|	GO:0008270|zir	.
Manes.01G25240(	5.0965928	1.42E-42	up	yes	WRKY trans	pfam03106	WRKY	WRKY DN.	GO:0006952|(	GO:0005634|	GO:0043565|se	K13425
Manes.01G25260(	0.4338369	9.88E-05	down	yes	.	pfam13639	zf-RING_2	Ring finger (	.	.	.	.
Manes.01G25270(	#NAME?	0.60004	down	no	1-aminocyclo	pfam00155	Aminotran_1_2	Aminotransf	GO:0009693|(	.	GO:0016847|1-	.
Manes.01G25280(	0.7075815	0.04123	down	no	.	pfam00403	HMA	Heavy-meta	.	.	.	.
Manes.01G25300(	1.4380438	0.00015	up	no	Protein REVI	pfam00249	Myb_DNA-binding	Myb-like DI	GO:0009737|r	GO:0005634|	GO:0003677|DI	.
Manes.01G25310(	0.9175639	0.37064	down	no	.	pfam09770	PAT1	Topoisomer	.	.	.	.
Manes.01G25340(	23.029027	#######	up	yes	Probable WR	pfam03106	WRKY	WRKY DN.	GO:0006351|t	GO:0005634|	GO:0043565|se	.
Manes.01G25350(	2.4227805	4.84E-13	up	yes	Probable seri	pfam00069	Pkinase	Protein kina	GO:0046777|p	GO:0016021|	GO:0005524|A	.
Manes.01G25380(	2.5593202	2.51E-05	up	yes	Putative UDI	pfam00201	UDPGT	UDP-glucor	.	GO:0016021|	GO:0016758|tra	.
Manes.01G25400(	0.8072393	0.06543	down	no	Mechanosens	pfam00924	MS_channel	Mechanoser	GO:0006811|i	GO:0009507|	.	.
Manes.01G25440(	1.0446237	0.6287	up	no	RAB6A-GEF	pfam07064	RIC1	RIC1. RIC1	GO:1903363|r	GO:0005829|	GO:0017137|Ra	.
Manes.01G25450(	8.2285097	1.43E-37	up	yes	E3 ubiquitin-	pfam13639	zf-RING_2	Ring finger (	GO:0016567|p	.	GO:0016874|lig	K16281
Manes.01G25470(	2.622179	7.14E-06	up	yes	.	pfam08879	WRC	WRC. The V	.	.	.	.
Manes.01G25480(	0.6773636	0.00013	down	no	.	pfam12036	DUF3522	Protein of u	.	.	.	.
Manes.01G25490(	3.3661733	4.93E-11	up	yes	Calmodulin-b	pfam07714	Pkinase_Tyr	Protein tyro	GO:0009755|l	GO:0005737|	GO:0005524|A	.
Manes.01G25510(	0.7888029	0.09936	down	no	AT-rich inter	pfam01388	ARID	ARID/BRIC	GO:0016575|l	GO:0048555|	GO:0003677|DI	.
Manes.01G25520(	1.5863435	9.37E-07	up	no	Ubiquitin-cor	pfam00179	UQ_con	Ubiquitin-cc	GO:0016567|p	GO:0005737|	GO:0005524|A	K10576
Manes.01G25530(	1.2899554	0.01202	up	no	ATP-depende	pfam00176	SNF2_N	SNF2 family	GO:0043044|.	GO:0005829|	GO:0005524|A	K11647
Manes.01G25540(	0.886038	0.19514	down	no	Bifunctional l	pfam00291	PALP	Pyridoxal-pl	GO:0019499|(	GO:0005739|	GO:0004124|cy	.
Manes.01G25560(	0.8113308	0.05695	down	no	.	pfam08264	Anticodon_1	Anticodon-b	.	.	.	.
Manes.01G25570(	2.9208578	6.21E-30	up	yes	.	pfam00303	Thymidylat_synt	Thymidylate	.	.	.	.
Manes.01G25580(	0.7597478	0.00467	down	no	Ascorbate tra	pfam07690	MFS_1	Major Facili	GO:0098656|a	GO:0009507|	GO:0005315|ino	.
Manes.01G25590(	0.6983635	0.00321	down	no	Glycerol-3-pl	pfam01553	Acyltransferase	Acyltransfer	GO:0010143|(	GO:0016021|	GO:0090447|gly	K13508
Manes.01G25620(	2.1132445	2.48E-15	up	yes	U-box domai	pfam07714	Pkinase_Tyr	Protein tyro	GO:0006950|r	.	GO:0005524|A	.
Manes.01G25630(	22.161302	0.00023	up	yes	.	pfam03554	Herpes_UL73	UL73 viral e	.	.	.	.
Manes.01G25660(	1.8408791	6.39E-10	up	no	.	pfam14309	DUF4378	Domain of u	.	.	.	.
Manes.01G25670(	0.6561728	1.86E-05	down	no	Mediator of I	pfam09497	Med12	Transcriptio	GO:0007275|r	GO:0016592|	GO:0001104|RI	.
Manes.01G25700(	0.9489879	0.70807	down	no	Rop guanine	pfam03759	PRONE	PRONE (Pl:	GO:0043547|p	.	GO:0005089|RI	.
Manes.01G25730(	27.947308	4.33E-12	up	yes	Poly(A)-spec	pfam04857	CAF1	CAF1 family	GO:0006952|(	GO:0005737|	GO:0046872|me	K01148
Manes.01G25760(	0.7619351	0.00756	down	no	60S ribosoma	pfam01158	Ribosomal_L36e	Ribosomal p	GO:0006412|t	GO:0022625|	GO:0003735|str	K02920
Manes.01G25780(	0.483388	2.53E-10	down	yes	Acyl-coenzyr	pfam02551	Acyl_CoA_thio	Acyl-CoA tl	GO:0006637|a	GO:0005739|	GO:0047617|ac	K11992
Manes.01G25790(	0.5756059	0.18187	down	no	.	pfam04030	ALO	D-arabinonc	.	.	.	.
Manes.01G25810(	0.7021379	0.06686	down	no	.	pfam01524	Gemini_V1	Geminivirus	.	.	.	.
Manes.01G25840(	0.6084595	0.01957	down	no	.	pfam13766	ECH_C	2-enoyl-CoA	.	.	.	.
Manes.01G25880(	1.3349229	0.00172	up	no	.	pfam03479	DUF296	Domain of u	.	.	.	.
Manes.01G25900(	Inf	0.00074	up	yes	Kiwellin	pfam03330	DPBB_1	Rare lipopro	.	GO:0005576|	.	.
Manes.01G25940(	0.5004507	1.40E-06	down	no	tRNA-specifi	pfam02137	A_deamin	Adenosine-c	GO:0008033|t	.	GO:0046872|me	K15440
Manes.01G25960(	1.8511664	2.87E-11	up	no	DEAD-box A	pfam00270	DEAD	DEAD/DEA	GO:0033962|(	GO:0000932|	GO:0005524|A	K12614
Manes.01G25970(	7.3140874	1.29E-13	up	yes	.	pfam06160	EzrA	Septation rir	.	.	.	.
Manes.01G25980(	1.7986883	1.34E-08	up	no	.	pfam07287	DUF1446	Protein of u	.	.	.	.
Manes.01G26040(	0.2745146	1.32E-25	down	yes	Pentatricopep	pfam13041	PPR_2	PPR repeat	.	GO:0009507|	.	.
Manes.01G26060(	0.3850904	4.42E-06	down	yes	Cell division p	pfam00448	SRP54	SRP54-type	GO:0006614|S	GO:0009570|	GO:0005525|G	K03110
Manes.01G26070(	8.3699539	1.70E-26	up	yes	BON1-associ	pfam00168	C2	C2 domain.	GO:0006952|(	GO:0016020|	.	.
Manes.01G26140(	1.4622243	2.40E-05	up	no	Probable met	pfam03141	Methyltransf_29	Putative S-a	GO:0052546|(	GO:0005768|	GO:0008757|S-	.
Manes.01G26150(	0.8119094	0.07478	down	no	FAS-associat	pfam00789	UBX	UBX domai	GO:0006915|a	GO:0031265|	GO:0031072|he	.
Manes.01G26160(	5.8247674	5.49E-18	up	yes	Probable met	pfam03141	Methyltransf_29	Putative S-a	.	GO:0005768|	GO:0008168|me	.
Manes.01G26170(	38.288698	2.13E-14	up	yes	F-box protein	pfam14299	PP2	Phloem prot	GO:0016567|p	GO:0005634|	.	.
Manes.01G26180(	2.2719386	7.49E-21	up	yes	.	pfam10395	Utp8	Utp8 family.	.	.	.	.
Manes.01G26200(	0.8694735	0.1855	down	no	Armadillo rep	pfam00514	Arm	Armadillo/b	GO:0003341|(	GO:0005930|	.	.
Manes.01G26210(	1.1015224	0.86054	up	no	Ethylene-resp	pfam00847	AP2	AP2 domain	GO:0035865|(	GO:0005634|	GO:0043565|se	K09286
Manes.01G26220(	0.4524269	7.08E-07	down	yes	RING-H2 fin	pfam13639	zf-RING_2	Ring finger (	GO:0016567|p	GO:0016021|	GO:0008270|zir	K19039
Manes.01G26230(	0.7260085	0.01484	down	no	Xyloglucan e	pfam00722	Glyco_hydro_16	Glycosyl hy	GO:0042546|(	GO:0048046|	GO:0004553|hy	K08235
Manes.01G26250(	1.9582596	0.00157	up	no	.	pfam09948	DUF2182	Predicted m	.	.	.	.
Manes.01G26260(	0.1408773	3.03E-12	down	yes	Two-compon	pfam00072	Response_reg	Response re	GO:0071368|(	GO:0005634|	GO:0003677|DI	K14491
Manes.01G26270(	0.4571713	1.33E-08	down	yes	Flap endonuc	pfam00867	XPG_I	XPG I-regic	GO:0006281|l	GO:0005634|	GO:0003677|DI	.

Manes.01G26390(	0.8591589	0.16436	down	no	Histone deac	pfam06487	SAP18	Sin3 associa	GO:0006355|	GO:0005730|	.	.
Manes.01G26420(	0.2585939	0.00251	down	yes	Probable carl	pfam07859	Abhydrolase_3	alpha/beta h	.	.	GO:0052689|ca	.
Manes.01G26470(	0.7620702	0.05352	down	no	Growth-regu	pfam08879	WRC	WRC. The	GO:0032502|	GO:0005634|	GO:0005524|A	.
Manes.01G26550(	0.2404655	4.48E-13	down	yes	AT-hook mo	pfam03479	DUF296	Domain of u	GO:0006325|	GO:0005634|	GO:0003680|A	.
Manes.01G26580(	1.6483793	1.99E-08	up	no	Proteasome s	pfam00227	Proteasome	Proteasome	GO:0051603|	GO:0005737|	GO:0004298|th	.
Manes.01G26600(	0.3555801	4.30E-16	down	yes	Glutathione S	pfam13417	GST_N_3	Glutathione	GO:0009072|	GO:0005737|	GO:0004364|gl	.
Manes.01G26610(	Inf	0.02401	up	yes	Protein trans	pfam00995	Sec1	Sec1 family.	GO:0015031|	GO:0005623|	.	.
Manes.01G26630(	1.017739	0.82814	up	no	Probable prot	pfam01529	zf-DHHC	DHHC palm	.	GO:0005794|	GO:0019706|pr	.
Manes.01G26660(	0.5705196	0.00013	down	no	.	pfam01501	Glyco_transf_8	Glycosyl tra	.	.	.	.
Manes.01G26670(	1.1413042	0.20504	up	no	UDP-glucuro	pfam01370	Epimerase	NAD depen	GO:0005975|	GO:0032580|	GO:0050662|co	K08679
Manes.01G26720(	1.1794146	0.08782	up	no	Subtilisin-like	pfam00082	Peptidase_S8	Subtilase fa	GO:0010102|	GO:0005618|	GO:0004252|se	.
Manes.01G26740(	0.8948447	0.27786	down	no	.	pfam00881	Nitroreductase	Nitroreducta	.	.	.	.
Manes.01G26750(	0.5048834	8.12E-08	down	no	.	pfam04859	DUF641	Plant protein	.	.	.	.
Manes.01G26770(	1.3343681	0.00152	up	no	Methionine a	pfam00557	Peptidase_M24	Metallopept	GO:0031365|	GO:0005737|	GO:0046872|m	K01265
Manes.01G26790(	7.0438929	7.56E-34	up	yes	Auxin-induce	pfam02519	Auxin_inducible	Auxin respo	GO:0009734|	.	.	K14488
Manes.01G26800(	0.8205555	0.118	down	no	Golgi SNAP	pfam12352	V-SNARE_C	Snare regio	GO:0006888|	GO:0005801|	.	K08495
Manes.01G26820(	2.025761	3.49E-13	up	yes	.	pfam04859	DUF641	Plant protein	.	.	.	.
Manes.01G26830(	0.208328	0.00096	down	yes	.	pfam04640	PLATZ	PLATZ tran	.	.	.	.
Manes.01G26850(	1.619961	0.00068	up	no	Vesicle-assoc	pfam00635	Motile_Sperm	MSP (Majo	.	GO:0005783|	.	.
Manes.01G26860(	9.8274639	0.00689	up	yes	.	pfam13966	zf-RVT	zinc-binding	.	.	.	.
Manes.01G26870(	0.4926557	6.22E-05	down	yes	Putative lipid	pfam14547	Hydrophob_seed	Hydrophobi	.	GO:0005576|	.	.
Manes.01G26880(	6.0158377	4.08E-08	up	yes	14 kDa prolin	pfam14547	Hydrophob_seed	Hydrophobi	.	GO:0016021|	.	.
Manes.01G26920(	NA	NA	--	no	.	pfam04478	Mid2	Mid2 like c	.	.	.	.
Manes.01G26930(	1.9575263	1.77E-10	up	no	Zinc finger p	pfam13912	zf-C2H2_6	C2H2-type	GO:0006355|	GO:0005634|	GO:0046872|m	.
Manes.01G26940(	#NAME?	0.30365	down	no	Zinc finger p	pfam13912	zf-C2H2_6	C2H2-type	GO:0048235|	GO:0005634|	GO:0046872|m	.
Manes.01G26970(	1.3308239	0.00299	up	no	Transcription	pfam00010	HLH	Helix-loop-l	GO:0009567|	GO:0005634|	GO:0003677|D	.
Manes.01G26980(	0.6400524	0.03277	down	no	.	pfam04924	Pox_A6	Poxvirus A6	.	.	.	.
Manes.01G26990(	0.8694091	0.2548	down	no	F-box protein	pfam12937	F-box-like	F-box-like.	.	.	.	K10268
Manes.01G27010(	1.0447116	0.65328	up	no	CCR4-NOT	pfam04054	Not1	CCR4-Not	GO:0035195|	GO:0030014|	GO:0070016|ar	K12604
Manes.01G27020(	0.983574	0.88355	down	no	.	pfam13851	GAS	Growth-arre	.	.	.	.
Manes.01G27050(	1.9650857	0.00132	up	no	.	pfam00628	PHD	PHD-finger.	.	.	.	.
Manes.01G27060(	Inf	2.67E-11	up	yes	.	pfam15461	BCD	Beta-carote	.	.	.	.
Manes.01G27070(	0.8201689	0.44288	down	no	Squamosa pr	pfam03110	SBP	SBP domai	GO:0048653|	GO:0005737|	GO:0003677|D	.
Manes.01G27080(	0.5809192	1.58E-07	down	no	.	pfam01960	ArgJ	ArgJ family.	.	.	.	.
Manes.01G27130(	1.042187	0.90338	up	no	Ethylene-resp	pfam00847	AP2	AP2 domai	GO:0009873|	GO:0005634|	GO:0003700|tr	.
Manes.01G27150(	1.6908073	0.00018	up	no	.	pfam00300	His_Phos_1	Histidine ph	.	.	.	.
Manes.01G27170(	2.2307446	3.69E-07	up	yes	.	pfam07797	DUF1639	Protein of u	.	.	.	.
Manes.01G27180(	0.5472547	0.00017	down	no	.	pfam02536	mTERF	mTERF. Th	.	.	.	.
Manes.01G27190(	1.0132038	0.87484	up	no	KH domain-c	pfam00013	KH_1	KH domain.	.	GO:0005634|	GO:0003723|R	K13162
Manes.01G27220(	0.9977661	1	down	no	Actin-related	pfam04699	P16-Arc	ARP2/3 con	GO:0034314|	GO:0005885|	.	K05754
Manes.01G27240(	0.9930323	0.96757	down	no	Peptidyl-prol	pfam00254	FKBP_C	FKBP-type	GO:0055114|	GO:0009507|	GO:0016491|ox	.
Manes.01G27260(	0.2661863	9.27E-08	down	yes	.	pfam13041	PPR_2	PPR repeat	.	.	.	.
Manes.01G27270(	0.4505487	5.22E-05	down	yes	UPF0565 pro	pfam10561	UPF0565	Uncharacter	.	GO:0005576|	.	.
Manes.01G27290(	0.9650391	0.76719	down	no	COP9 signal	pfam01399	PCI	PCI domain	GO:0010387|	GO:0008180|	.	K12180
Manes.01G27310(	1.0621874	0.59908	up	no	Metacaspase-	pfam00656	Peptidase_C14	Caspase do	GO:0006952|	.	GO:0004197|cy	.
Manes.01G27360(	1.0140608	0.86804	up	no	Pentatricope	pfam13041	PPR_2	PPR repeat	.	GO:0009507|	.	.
Manes.01G27420(	0.481838	1.48E-11	down	yes	.	pfam04774	HABP4_PAI-RBP	Hyaluronan	.	.	.	.
Manes.01G27430(	0.8237591	0.08719	down	no	Ras-related p	pfam00071	Ras	Ras family.	GO:0006888|	GO:0005789|	GO:0005525|G	K07874
Manes.01G27460(	1.1356862	0.1708	up	no	GRAM doma	pfam02893	GRAM	GRAM dom	.	GO:0016021|	.	.
Manes.01G27490(	0.8747949	0.27179	down	no	Protein ECE	pfam12076	Wax2_C	WAX2 C-te	GO:0006633|	GO:0005789|	GO:0005506|ir	K15404
Manes.02G00010(	1.1162814	0.67586	up	no	Heavy metal-	pfam00403	HMA	Heavy-meta	GO:0010286|	GO:0031965|	GO:0046872|m	.
Manes.02G00060(	0.7482655	0.01519	down	no	Metal toleran	pfam01545	Cation_efflux	Cation efflu	GO:0006829|	GO:0016021|	GO:0008324|ca	.
Manes.02G00090(	0.8369518	0.07508	down	no	StAR-related	pfam03901	Glyco_transf_22	Alg9-like m	GO:1903955|	GO:0005739|	GO:0008289|lip	.
Manes.02G00130(	0.4519117	0.01159	down	yes	.	pfam04968	CHORD	CHORD. C	.	.	.	.
Manes.02G00140(	0.4785114	1.47E-05	down	yes	Polyadenylat	pfam00076	RRM_1	RNA recogn	.	GO:0005634|	GO:0000166|nu	.
Manes.02G00150(	0.3907059	3.02E-10	down	yes	Alcohol dehy	pfam08240	ADH_N	Alcohol deh	.	GO:0005737|	GO:0004022|al	K00001
Manes.02G00180(	0.4004367	0.00696	down	yes	.	pfam00063	Myosin_head	Myosin hea	.	.	.	.
Manes.02G00190(	0.660348	2.48E-05	down	no	Protein GPR	pfam06814	Lung_7-TM_R	Lung seven	GO:0072583|	GO:0030136|	GO:0032050|cl	.
Manes.02G00210(	0.3934823	6.83E-13	down	yes	Beta-carote	pfam13225	DUF4033	Domain of u	GO:0010223|	GO:0009507|	GO:0016853|is	K17911
Manes.02G00260(	0.5388263	0.0378	down	no	Cytochrome	pfam00067	p450	Cytochrome	.	.	GO:0020037|he	.
Manes.02G00290(	0.4452968	0.00107	down	yes	Methionine g	pfam01053	Cys_Met_Meta_P	Cys/Met me	GO:0009970|	GO:0005829|	GO:0018826|m	K01761
Manes.02G00330(	8.9656132	4.64E-10	up	yes	.	pfam10101	DUF2339	Predicted m	.	.	.	.
Manes.02G00340(	1.296043	0.00403	up	no	Serine/threon	pfam00069	Pkinase	Protein kina	.	.	GO:0005524|A	K08835
Manes.02G00350(	1.0255519	0.84956	up	no	.	pfam02262	Cbl_N	CBL proto-	.	.	.	.
Manes.02G00370(	0.9734232	0.82102	down	no	UPF0160 pro	pfam03690	UPF0160	Uncharacter	GO:0035641|	GO:0070062|	.	.
Manes.02G00380(	0.7583299	0.01578	down	no	Molybdate-a	pfam05631	DUF791	Protein of u	GO:0055085|	GO:0016021|	GO:0015098|m	.
Manes.02G00390(	6.5541442	1.74E-14	up	yes	Early nodulin	pfam02298	Cu_bind_like	Plastocyani	.	GO:0031225|	GO:0009055|el	.
Manes.02G00400(	1.6821157	4.93E-08	up	no	Probable N6-	pfam05063	MT-A70	MT-A70. M	.	GO:0009507|	GO:0016422|m	K05925
Manes.02G00420(	0.4261031	1.12E-09	down	yes	.	pfam03226	Yippee-Mis18	Yippee zinc	.	.	.	.
Manes.02G00460(	1.5340518	0.00026	up	no	Zinc finger H	pfam04438	zf-HIT	HIT zinc fin	.	.	GO:0046872|m	.
Manes.02G00470(	4.9004384	0.05284	up	no	Aluminum-ac	pfam11744	ALMT	Aluminium	GO:0015743|	GO:0016021|	.	.
Manes.02G00500(	3.1410875	6.59E-15	up	yes	Dof zinc fing	pfam02701	zf-Dof	Dof domain	GO:0006355|	GO:0005634|	GO:0003677|D	.
Manes.02G00510(	1.8590425	5.15E-10	up	no	WD repeat-c	pfam00400	WD40	WD domain	.	GO:0005829|	.	.
Manes.02G00520(	2.2323856	0.00545	up	yes	Transcription	pfam00249	Myb_DNA-binding	Myb-like D	GO:0009813|	GO:0005634|	GO:0003677|D	K09422
Manes.02G00560(	0.9367147	0.66667	down	no	.	pfam09762	KOG2701	Coiled-coil	.	.	.	.
Manes.02G00590(	0.7386294	0.01017	down	no	Glutamine-de	pfam02540	NAD_synthase	NAD synth	GO:0009435|	GO:0005829|	GO:0005524|A	K01950
Manes.02G00600(	0.8653904	0.15811	down	no	Luminal-bind	pfam00012	HSP70	Hsp70 prot	.	GO:0005788|	GO:0005524|A	.
Manes.02G00620(	0.7454066	0.01982	down	no	Beta-amylase	pfam01373	Glyco_hydro_14	Glycosyl hy	GO:0000024|	GO:0009570|	GO:0016161|be	K01177
Manes.02G00670(	0.8854388	0.20744	down	no	Protein NLP	pfam02042	RWP-RK	RWP-RK d	GO:0042128|	GO:0005634|	GO:0003677|D	.
Manes.02G00680(	0.4336137	0.00106	down	yes	Protein ABI	pfam05770	Ins134_P3_kin	Inositol 1, 3	.	GO:0005737|	.	.
Manes.02G00690(	0.8285035	0.08942	down	no	26S proteaso	pfam03399	SAC3_GANP	SAC3/GAN	GO:0048825|	GO:0009941|	.	K03031
Manes.02G00730(	3.639533	0.03502	up	no	Cellulose syn	pfam03552	Cellulose_synt	Cellulose sy	GO:0071555|	GO:0000139|	GO:0016760|ce	.
Manes.02G00760(	1.7428287	6.10E-10	up	no	Auxin respon	pfam06507	Auxin_resp	Auxin respo	GO:0009734|	GO:0005634|	GO:0003677|D	.
Manes.02G00770(	1.6745528	8.02E-08	up	no	3-oxoacyl-[a	pfam00109	ketoacyl-synt	Beta-ketoac	GO:0006633|	GO:0009570|	GO:0004315|3-	K09458
Manes.02G00790(	0.4137446	6.46E-14	down	yes	Uncharacteri	pfam00462	Glutaredoxin	Glutaredoxi	GO:0045454|	GO:0005886|	GO:0009055|el	.
Manes.02G00830(	0.6618036	0.00308	down	no	.	pfam08766	DEK_C	DEK C term	.	.	.	.
Manes.02G00840(	0.9145693	0.47032	down	no	F-box protein	pfam13474	SnoaL_3	SnoaL-like	GO:0016567|	.	.	.
Manes.02G00860(	0.6983335	0.00023	down	no	ATP-depend	pfam00004	AAA	ATPase fam	GO:0030163|	GO:0016021|	GO:0005524|A	K03798

Manes.02G00900(	0.2456555	1.04E-11	down	yes	.	pfam03168	LEA_2	Late embryo	.	.	.	.
Manes.02G00930(	1.5300698	2.08E-05	up	no	UDP-glucose	pfam01370	Epimerase	NAD depen	GO:0006012|	.	GO:0050662|co	K01784
Manes.02G00940(	1.0600202	0.52545	up	no	.	pfam07078	FYTT	Forty-two-t	.	.	.	.
Manes.02G00950(	1.1883746	0.06429	up	no	Nucleoside d	pfam00334	NDK	Nucleoside	GO:0006241|	GO:0009543|	GO:0005524|A	K00940
Manes.02G00960(	0.5595504	1.20E-07	down	no	.	pfam11623	DUF3252	Protein of u	.	.	.	.
Manes.02G00980(	0.5826211	1	down	no	.	pfam06500	DUF1100	Alpha/beta l	.	.	.	.
Manes.02G01010(	0.8242371	1	down	no	RING-H2 fin	pfam13639	zf-RING_2	Ring finger	GO:0016567|	GO:0016021|	GO:0008270|zi	.
Manes.02G01030(	1.2977213	0.00474	up	no	Long chain a	pfam00501	AMP-binding	AMP-bindin	GO:0006631|	GO:0005794|	GO:0005524|A	K01897
Manes.02G01040(	0.4975825	5.76E-08	down	yes	.	pfam13855	LRR_8	Leucine rich	.	.	.	.
Manes.02G01060(	0.4291853	4.19E-10	down	yes	Endoglucana	pfam00759	Glyco_hydro_9	Glycosyl hy	GO:0071555|	GO:0005576|	GO:0030246|ca	.
Manes.02G01070(	1.9768837	0.0003	up	no	Ethylene-resp	pfam00847	AP2	AP2 domain	GO:0009873|	GO:0005634|	GO:0003677|D	.
Manes.02G01080(	0.7022561	0.00062	down	no	Abscisic acid	pfam10604	Polyketide_cyc2	Polyketide c	GO:0009738|	GO:0005737|	GO:0010427|ab	K14496
Manes.02G01090(	1.0117294	0.90094	up	no	Probable poly	pfam00295	Glyco_hydro_28	Glycosyl hy	GO:0005975|	GO:0016021|	GO:0004650|pc	.
Manes.02G01100(	1.6282675	4.71E-06	up	no	.	pfam05340	DUF740	Protein of u	.	.	.	.
Manes.02G01110(	0.211252	3.65E-08	down	yes	.	pfam05922	Inhibitor_I9	Peptidase in	.	.	.	.
Manes.02G01150(	0.6796339	0.25838	down	no	Probable WR	pfam03106	WRKY	WRKY DN	GO:0006351|	GO:0005634|	GO:0043565|se	.
Manes.02G01160(	0.8022792	0.03397	down	no	.	pfam12159	DUF3593	Protein of u	.	.	.	.
Manes.02G01180(	0.6285366	7.43E-06	down	no	Protein SEH1	pfam00400	WD40	WD domain	GO:0051028|	GO:0005737|	.	K14299
Manes.02G01190(	1.2717491	0.06906	up	no	.	pfam10328	7TM_GPCR_Srx	Serpentine t	.	.	.	.
Manes.02G01260(	0.5035954	1.05E-06	down	no	.	pfam01576	Myosin_tail_1	Myosin tail.	.	.	.	.
Manes.02G01290(	0.6407235	0.00036	down	no	Probable beta	pfam01762	Galactosyl_T	Galactosyltr	GO:0006486|	GO:0005768|	GO:1990714|hy	.
Manes.02G01300(	2.6512184	0.11454	up	no	Putative gluc	pfam01263	Aldose_epim	Aldose 1-ep	GO:0005975|	.	GO:0030246|ca	.
Manes.02G01310(	1.2906685	0.00996	up	no	Ubiquitin-cor	pfam00179	UQ_con	Ubiquitin-cc	GO:0016567|	.	GO:0005524|A	K06689
Manes.02G01320(	0.7254775	0.09088	down	no	Ethylene-resp	pfam00847	AP2	AP2 domain	GO:0048825|	GO:0005737|	GO:0003677|D	.
Manes.02G01330(	0.4642484	1.16E-08	down	yes	Arabinogalac	pfam06376	DUF1070	Protein of u	.	GO:0031225|	.	.
Manes.02G01350(	0.5157757	1.23E-05	down	no	CMP-sialic a	pfam04142	Nuc_sug_transp	Nucleotide-	GO:0015780|	GO:0000139|	GO:0005338|nu	K15272
Manes.02G01480(	NA	NA	--	no	.	pfam07571	DUF1546	Protein of u	.	.	.	.
Manes.02G01490(	0.6796952	0.0003	down	no	Probable WR	pfam03154	Atrophin-1	Atrophin-1	GO:0006952|	GO:0009941|	GO:0043531|A	.
Manes.02G01520(	0.9807127	0.84888	down	no	Potassium tra	pfam02705	K_trans	K+ potassiu	GO:0009932|	GO:0016021|	GO:0015079|pc	K03549
Manes.02G01530(	0.2367648	2.03E-16	down	yes	.	pfam02517	Abi	CAAX prot	.	.	.	.
Manes.02G01540(	0.6688575	3.50E-05	down	no	Uncharacteri	pfam00004	AAA	ATPase fam	GO:0031122|	GO:0005741|	GO:0005524|A	.
Manes.02G01570(	0.2556954	0.50461	down	no	.	pfam00150	Cellulase	Cellulase (g	.	.	.	.
Manes.02G01580(	0.2024537	0.11036	down	no	LRR recepto	pfam13855	LRR_8	Leucine rich	GO:0009793|	GO:0016021|	GO:0005524|A	.
Manes.02G01600(	11.287247	0.01788	up	yes	.	pfam03168	LEA_2	Late embryo	.	.	.	.
Manes.02G01620(	1.5114421	0.07901	up	no	Respiratory b	pfam08030	NAD_binding_6	Ferric reduc	.	GO:0016021|	GO:0005509|ca	K13447
Manes.02G01630(	1.2006189	0.87657	up	no	.	pfam04525	Tub_2	Tubby C 2.	.	.	.	.
Manes.02G01650(	1.1420862	0.14267	up	no	Serine/threon	pfam00149	Metallophos	Calcineurin-	.	.	GO:0046872|m	.
Manes.02G01660(	0.8493353	0.12497	down	no	E3 SUMO-p	pfam02891	zf-MIZ	MIZ/SP-RII	GO:0030999|	GO:0005634|	GO:0016874|lig	K04706
Manes.02G01670(	0.467296	2.95E-07	down	yes	Protein SGT1	pfam05002	SGS	SGS domain	GO:0071365|	GO:0005634|	.	K12795
Manes.02G01680(	0.7082931	0.0008	down	no	WD repeat-c	pfam00400	WD40	WD domain	GO:0002244|	GO:0008021|	.	.
Manes.02G01770(	8.1696268	8.52E-05	up	yes	1-aminocyclo	pfam00155	Aminotran_1_2	Aminotrans	GO:0009693|	.	GO:0016847|1-	K01762
Manes.02G01810(	0.4717516	7.13E-08	down	yes	Probable poly	pfam00295	Glyco_hydro_28	Glycosyl hy	GO:0005975|	GO:0016021|	GO:0004650|pc	.
Manes.02G01830(	2.083848	0.10045	up	no	Beta-carotene	pfam04116	FA_hydroxylase	Fatty acid h	GO:0006633|	GO:0031969|	GO:0016787|hy	K15746
Manes.02G01860(	0.5963454	0.00025	down	no	.	pfam04646	DUF604	Protein of u	.	.	.	.
Manes.02G01890(	0.8347442	0.32438	down	no	Phosphoglyc	pfam00300	His_Phos_1	Histidine ph	GO:0005975|	.	GO:0003824|ca	.
Manes.02G01940(	0.237027	6.01E-16	down	yes	Transcription	pfam14215	bHLH-MYC_N	bHLH-MYC	GO:0001708|	GO:0005634|	GO:0003677|D	.
Manes.02G01950(	5.9796078	8.14E-19	up	yes	Gibberellin 2	pfam03171	2OG-FeII_Oxy	2OG-Fe(II)	.	.	GO:0046872|m	.
Manes.02G01980(	1.6541903	1.64E-08	up	no	UDP-N-acety	pfam08245	Mur_ligase_M	Mur ligase r	GO:0007049|	GO:0005737|	GO:0005524|A	K01928
Manes.02G01990(	5.6071462	1.34E-06	up	yes	.	pfam11230	DUF3029	Protein of u	.	.	.	.
Manes.02G02000(	0.8847116	0.35914	down	no	Signal peptid	pfam04258	Peptidase_A22B	Signal pepti	.	GO:0005768|	GO:0004190|as	.
Manes.02G02010(	1.5878538	0.00181	up	no	Probable aqu	pfam00230	MIP	Major intrin	GO:0009992|	GO:0005887|	GO:0015254|gl	K09872
Manes.02G02060(	4.1306811	2.08E-57	up	yes	BTB/POZ do	pfam02214	BTB_2	BTB/POZ d	GO:0051260|	.	.	.
Manes.02G02080(	8.8798482	#######	up	yes	Uncharacteri	pfam15336	Auts2	Autism susc	.	GO:0005886|	.	.
Manes.02G02130(	1.3451007	0.0216	up	no	Ubiquitin-cor	pfam00179	UQ_con	Ubiquitin-cc	GO:0016567|	GO:0005737|	GO:0005524|A	K06689
Manes.02G02150(	0.8012874	0.08658	down	no	.	pfam13867	SAP30_Sin3_bdg	Sin3 binding	.	.	.	.
Manes.02G02200(	1.5118378	0.00051	up	no	.	pfam12043	DUF3527	Domain of u	.	.	.	.
Manes.02G02230(	0.4789635	3.61E-06	down	yes	.	pfam01426	BAH	BAH domai	.	.	.	.
Manes.02G02250(	0.5145905	4.31E-08	down	no	AT-hook mo	pfam03479	DUF296	Domain of u	GO:0006325|	GO:0005634|	GO:0003680|A	.
Manes.02G02260(	1.0576102	0.52655	up	no	Probable pho	pfam00255	GSHPx	Glutathione	GO:0046686|	GO:0048046|	GO:0004602|gl	K00432
Manes.02G02270(	5.3048666	0.22895	up	no	Xyloglucan g	pfam03016	Exostosin	Exostosin fa	GO:0010256|	GO:0005768|	GO:0016757|tra	.
Manes.02G02280(	1.7706163	0.0004	up	no	C2 and GRA	pfam08372	PRT_C	Plant phospl	.	GO:0016021|	.	.
Manes.02G02310(	0.4021252	6.75E-07	down	yes	Glutathione S	pfam02798	GST_N	Glutathione	GO:0009636|	GO:0005737|	GO:0004364|gl	K00799
Manes.02G02320(	0.44925	0.00032	down	yes	Probable cop	pfam00122	E1-E2_ATPase	E1-E2 ATP	GO:0006825|	GO:0016021|	GO:0005524|A	K17686
Manes.02G02330(	0.7248949	0.05854	down	no	Serine racem	pfam00291	PALP	Pyridoxal-pl	GO:0070179|	GO:0005737|	GO:0005524|A	K12235
Manes.02G02360(	1.9074248	0.00015	up	no	.	pfam04525	Tub_2	Tubby C 2.	.	.	.	.
Manes.02G02390(	0.2963103	2.91E-13	down	yes	.	pfam01576	Myosin_tail_1	Myosin tail.	.	.	.	.
Manes.02G02420(	0.6981637	0.00078	down	no	30S ribosoma	pfam00575	S1	S1 RNA bin	GO:0040007|	GO:0005618|	GO:0003723|R	K02945
Manes.02G02430(	2.2088229	2.76E-18	up	yes	Tetraspanin-1	pfam00335	Tetraspannin	Tetraspanin	.	GO:0016021|	.	.
Manes.02G02450(	2.9231648	2.87E-23	up	yes	Probable DE	pfam01271	Granin	Granin (chro	.	.	GO:0005524|A	.
Manes.02G02470(	9.6682348	8.23E-18	up	yes	.	pfam14358	DUF4405	Domain of u	.	.	.	.
Manes.02G02490(	0.6358403	1.31E-05	down	no	Methyl-CpG-	pfam01429	MBD	Methyl-CpC	GO:0006355|	GO:0005634|	GO:0008327|m	.
Manes.02G02500(	0.9811856	0.87826	down	no	.	pfam00168	C2	C2 domain.	.	.	.	.
Manes.02G02520(	0.0384569	1.56E-07	down	yes	Thaumatin-lil	pfam00314	Thaumatin	Thaumatin f	GO:0050832|	GO:0005576|	.	.
Manes.02G02530(	1.0090447	0.94986	up	no	Bifunctional	pfam01370	Epimerase	NAD depen	GO:0045227|	GO:0005829|	GO:0050662|co	K01784
Manes.02G02540(	1.2449785	0.33186	up	no	CASP-like pr	pfam04535	DUF588	Domain of u	GO:0055114|	GO:0016021|	GO:0051536|irc	.
Manes.02G02550(	0.4053085	2.38E-10	down	yes	DEAD-box A	pfam00270	DEAD	DEAD/DEA	GO:0009663|	GO:0005739|	GO:0005524|A	.
Manes.02G02560(	1.1260486	0.22743	up	no	E3 ubiquitin-	pfam13639	zf-RING_2	Ring finger	GO:0016567|	GO:0016021|	GO:0016874|lig	.
Manes.02G02620(	Inf	0.17988	up	no	.	pfam11176	DUF2962	Protein of u	.	.	.	.
Manes.02G02650(	3.5840571	2.86E-39	up	yes	.	pfam01694	Rhomboid	Rhomboid f	.	.	.	.
Manes.02G02660(	0.5901481	0.00108	down	no	.	pfam13178	DUF4005	Protein of u	.	.	.	.
Manes.02G02690(	0.3741824	4.98E-12	down	yes	Phosphatidyli	pfam06728	PIG-U	GPI transam	GO:0006506|	GO:0042765|	.	K05293
Manes.02G02740(	2.2341474	1.62E-16	up	yes	F-box protein	pfam14299	PP2	Phloem prot	.	.	GO:0030246|ca	.
Manes.02G02750(	0.9795773	0.83752	down	no	Lysophospho	pfam03062	MBOAT	MBOAT, m	GO:0019375|	GO:0005783|	GO:0047184|1-	K13519
Manes.02G02790(	0.1615111	1.08E-11	down	yes	.	pfam12730	ABC2_membrane_	ABC-2 fami	.	.	.	.
Manes.02G02800(	2.4002463	2.91E-06	up	yes	UDP-galacto	pfam08449	UAA	UAA transp	GO:0008643|	GO:0016021|	.	K15277
Manes.02G02810(	1.0697467	0.4582	up	no	.	pfam00638	Ran_BP1	RanBP1 do	.	.	.	.
Manes.02G02860(	29.205319	2.98E-05	up	yes	Geraniol 8-hy	pfam00067	p450	Cytochrome	GO:0016099|	GO:0005789|	GO:0020037|he	.
Manes.02G02940(	0.5811795	3.11E-06	down	no	Zinc finger C	pfam04146	YTH	YT521-B-lil	GO:0000278|	GO:0005847|	GO:0003677|D	K14404

Manes.02G02990(	0.6927866	0.00399	down	no	Protein FIZZ	pfam00400	WD40	WD domain	GO:0051301|	GO:0005634|	.	K03364
Manes.02G03000(	1.3335301	0.00955	up	no	Protein STAY	pfam12638	Staygreen	Staygreen p	GO:0015996|	GO:0009535|	.	.
Manes.02G03050(	0.6873952	0.00058	down	no	Homeobox p	pfam05920	Homeobox_KN	Homeobox	GO:0045892|	GO:0005634|	GO:0003677|D	.
Manes.02G03070(	1.066942	0.4728	up	no	Bifunctional	pfam01370	Epimerase	NAD depen	GO:0071555|	GO:0048046|	GO:0008830|d	K12451
Manes.02G03110(	1.8721585	2.44E-06	up	no	SPX domain-	pfam07690	MFS_1	Major Facili	GO:0055085|	GO:0016021|	.	.
Manes.02G03120(	6.0019911	1.26E-25	up	yes	Growth-regu	pfam08879	WRC	WRC. The	GO:0032502|	GO:0005634|	GO:0005524|A	.
Manes.02G03130(	3.2956593	1.06E-22	up	yes	Probable sug	pfam03151	TPT	Triose-phos	GO:0008643|	GO:0016021|	.	.
Manes.02G03150(	0.7871705	0.02762	down	no	Probable ami	pfam00155	Aminotran_1_2	Aminotransf	GO:0009058|	.	GO:0030170|py	K14270
Manes.02G03210(	0.7516076	0.00451	down	no	Armadillo rep	pfam00225	Kinesin	Kinesin mot	GO:0007018|	GO:0005737|	GO:0005524|A	.
Manes.02G03220(	4.9998574	1.40E-38	up	yes	AT-hook mo	pfam03479	DUF296	Domain of u	GO:0006325|	GO:0005634|	GO:0003680|A	.
Manes.02G03250(	4.5686904	2.97E-49	up	yes	Bifunctional	pfam01842	ACT	ACT domai	GO:0006807|	.	GO:0008773|[p	K00990
Manes.02G03270(	1.9306302	6.61E-07	up	no	.	pfam13041	PPR_2	PPR repeat	.	.	.	.
Manes.02G03290(	1.0583404	0.8483	up	no	.	pfam00455	DeoRC	DeoR C terr	.	.	.	.
Manes.02G03320(	0.9441494	0.62514	down	no	.	pfam03155	Alg6_Alg8	ALG6, ALG	.	.	.	.
Manes.02G03330(	0.5318017	4.02E-06	down	no	Probable prot	pfam01529	zf-DHHC	DHHC palm	.	GO:0005794|	GO:0019706|pr	.
Manes.02G03350(	10.008294	2.43E-25	up	yes	.	pfam07279	DUF1442	Protein of u	.	.	.	.
Manes.02G03360(	3.8595553	6.22E-29	up	yes	Protein NSP-	pfam07714	Pkinase_Tyr	Protein tyro	GO:0006952|	GO:0016021|	GO:0005524|A	.
Manes.02G03370(	0.5079163	7.29E-07	down	no	Probable calc	pfam13499	EF-hand_7	EF-hand do	.	GO:0005737|	GO:0005509|ca	K13448
Manes.02G03390(	0.9394391	0.55277	down	no	Maspardin	pfam12697	Abhydrolase_6	Alpha/beta	.	GO:0005829|	GO:0042609|C	K19367
Manes.02G03420(	0.6870105	0.00774	down	no	.	pfam01311	Bac_export_1	Bacterial ex	.	.	.	.
Manes.02G03430(	1.0948541	0.65833	up	no	Protein ODO	pfam00249	Myb_DNA-binding	Myb-like D	GO:0006355|	GO:0005634|	GO:0003677|D	.
Manes.02G03440(	2.2261751	2.86E-05	up	yes	FAM10 famil	pfam13414	TPR_11	TPR repeat.	GO:0046686|	GO:0005829|	.	K09560
Manes.02G03480(	0.6882552	0.00868	down	no	21 kDa prote	pfam04043	PMEI	Plant inverta	.	.	GO:0004857|en	.
Manes.02G03490(	2.6891419	9.16E-10	up	yes	21 kDa prote	pfam04043	PMEI	Plant inverta	.	.	GO:0004857|en	.
Manes.02G03500(	1.1813426	0.09574	up	no	Elongation fa	pfam00009	GTP_EFTU	Elongation f	.	GO:0009507|	GO:0005525|G	K02355
Manes.02G03510(	0.7736629	0.03704	down	no	Hsp70-Hsp90	pfam13414	TPR_11	TPR repeat.	GO:0051131|	GO:0005737|	GO:0051879|H	K09553
Manes.02G03520(	0.5259362	0.00077	down	no	NADH dehyd	pfam00494	SQS_PSY	Squalene/ph	GO:0009058|	GO:0005743|	GO:0016740|tra	K18163
Manes.02G03560(	0.20094	0.00782	down	yes	.	pfam09794	Avl9	Transport pr	.	.	.	.
Manes.02G03580(	1.0880457	0.50761	up	no	Monocopper	pfam00394	Cu-oxidase	Multicopper	GO:0009932|	GO:0031225|	GO:0005507|co	.
Manes.02G03590(	1.4618912	0.02024	up	no	Acid beta-fru	pfam00251	Glyco_hydro_32N	Glycosyl hyd	GO:0005985|	GO:0005773|	GO:0004575|su	.
Manes.02G03620(	1.4028634	0.00024	up	no	3-oxoacyl-[ac	pfam08541	ACP_syn_III_C	3-Oxoacyl-[	GO:0006633|	GO:0009507|	GO:0004315|3-	.
Manes.02G03660(	15.341854	1.09E-38	up	yes	Probable WR	pfam03106	WRKY	WRKY DN	GO:0010200|	GO:0005634|	GO:0005516|ca	.
Manes.02G03670(	1.3414364	0.02688	up	no	Adenine phos	pfam00156	Pribosyltran	Phosphoribo	GO:0006168|	GO:0005737|	GO:0003999|ad	K00759
Manes.02G03680(	24.362764	1.32E-95	up	yes	.	pfam04878	Baculo_p48	Baculovirus	.	.	.	.
Manes.02G03700(	0.3567134	6.90E-05	down	yes	.	pfam03999	MAP65_ASE1	Microtubule	.	.	.	.
Manes.02G03710(	0.22241	4.50E-06	down	yes	.	pfam10325	7TM_GPCR_Srz	Serpentine t	.	.	.	.
Manes.02G03720(	0.651497	0.00037	down	no	Oxysterol-bin	pfam01237	Oxysterol_BP	Oxysterol-b	GO:0006869|	.	GO:0008289|lip	.
Manes.02G03790(	1.6733887	0.00279	up	no	NADH dehyd	pfam05821	NDUF_B8	NADH-ubiq	GO:0055114|	GO:0016021|	.	.
Manes.02G03820(	5.9976346	2.88E-43	up	yes	L-lactate deh	pfam00056	Ldh_1_N	lactate/mala	GO:0005975|	GO:0005737|	GO:0004459|L-	.
Manes.02G03830(	0.7516834	0.05715	down	no	Autophagy-re	pfam02991	Atg8	Autophagy p	GO:0000045|	GO:0000421|	.	K08341
Manes.02G03860(	11.945758	8.88E-59	up	yes	.	pfam15054	DUF4535	Domain of u	.	.	.	.
Manes.02G03880(	Inf	1.65E-09	up	yes	.	pfam00403	HMA	Heavy-meta	.	.	.	.
Manes.02G03910(	0.8115119	0.10809	down	no	Histidine triad	pfam11969	DcpS_C	Scavenger m	.	GO:0070062|	GO:0016787|hy	.
Manes.02G03920(	1.3471748	0.00253	up	no	Chaperone pr	pfam11875	DUF3395	Domain of u	GO:0055122|	GO:0016021|	.	K09531
Manes.02G03940(	10.182815	6.01E-40	up	yes	Putative gluc	pfam01501	Glyco_transf_8	Glycosyl tra	.	GO:0016021|	GO:0046872|me	.
Manes.02G03970(	7.6024467	0.0121	up	yes	.	pfam12874	zf-met	Zinc-finger	.	.	.	.
Manes.02G04050(	0.5953865	3.07E-06	down	no	Uncharacteriz	pfam10250	O-FucT	GDP-fucose	.	GO:0005768|	GO:0016757|tra	.
Manes.02G04120(	0.8726076	0.20776	down	no	.	pfam01987	AIM24	Mitochondr	.	.	.	.
Manes.02G04130(	0.167121	4.37E-05	down	yes	Transcription	pfam00249	Myb_DNA-binding	Myb-like D	GO:0006355|	GO:0005634|	GO:0003677|D	K09422
Manes.02G04160(	1.0844737	0.36535	up	no	Peroxisomal	pfam01756	ACOX	Acyl-CoA o	GO:0006635|	GO:0005777|	GO:0003995|ac	.
Manes.02G04180(	3.3897338	2.57E-41	up	yes	Homeobox-le	pfam04618	HD-ZIP_N	HD-ZIP pro	GO:0008283|	GO:0005634|	GO:0042803|pr	K09338
Manes.02G04210(	1.3188349	0.00317	up	no	Putative dual	pfam00782	DSPc	Dual specific	.	.	GO:0004725|pr	.
Manes.02G04230(	0.9392692	0.57758	down	no	Nudix hydrol	pfam00293	NUDIX	NUDIX dor	GO:0009611|	.	GO:0016787|hy	.
Manes.02G04240(	8.6413762	5.21E-53	up	yes	Ethylene-resp	pfam00847	AP2	AP2 domain	GO:0051301|	GO:0005634|	GO:0003677|D	K09286
Manes.02G04250(	37.193308	3.05E-18	up	yes	Ethylene-resp	pfam00847	AP2	AP2 domain	GO:0051301|	GO:0005634|	GO:0003677|D	K09286
Manes.02G04270(	1.5046016	0.00835	up	no	Ubiquitin carl	pfam01088	Peptidase_C12	Ubiquitin ca	GO:0016579|	GO:0005737|	GO:0004843|ub	K05609
Manes.02G04310(	0.8521967	0.16161	down	no	.	pfam10333	Pga1	GPI-Manno	.	.	.	.
Manes.02G04320(	4.7568466	9.20E-16	up	yes	Phospholipas	pfam01764	Lipase_3	Lipase (clas	GO:0016042|	GO:0009507|	GO:0047714|ga	.
Manes.02G04350(	1.9777087	7.83E-10	up	no	GTP-binding	pfam00071	Ras	Ras family.	GO:0015031|	GO:0005622|	GO:0005525|G	K07874
Manes.02G04400(	1.4296302	0.0216	up	no	.	pfam15009	TMEM173	Transmemb	.	.	.	.
Manes.02G04410(	1.639247	1.87E-07	up	no	Vesicle-assoc	pfam00635	Motile_Sperm	MSP (Major	.	GO:0005783|	.	.
Manes.02G04450(	2.5479007	4.65E-05	up	yes	.	pfam07773	DUF1619	Protein of u	.	.	.	.
Manes.02G04460(	2.5194825	3.29E-05	up	yes	Histone-lysin	pfam02182	SAD_SRA	SAD/SRA d	GO:0031048|	GO:0000775|	GO:0042054|his	K11420
Manes.02G04470(	4.0045915	0.00019	up	yes	Expansin-like	pfam03330	DPBB_1	Rare lipopro	GO:0019953|	GO:0005576|	.	.
Manes.02G04480(	0.323032	8.24E-14	down	yes	ATP-depende	pfam00574	CLP_protease	Clp protease	GO:0010468|	GO:0009507|	GO:0004252|se	K01358
Manes.02G04500(	0.8928834	0.25024	down	no	.	pfam00504	Chloroa_b-bind	Chlorophyll	.	.	.	.
Manes.02G04520(	0.1562151	0.01243	down	yes	.	pfam02906	Fe_hyd_lg_C	Iron only hy	.	.	.	.
Manes.02G04590(	0.9671928	0.7544	down	no	60S ribosoma	pfam01199	Ribosomal_L34e	Ribosomal p	GO:0006412|	GO:0005840|	GO:0003735|str	.
Manes.02G04600(	13.254955	4.10E-07	up	yes	.	pfam07891	DUF1666	Protein of u	.	.	.	.
Manes.02G04610(	5.48863	2.18E-28	up	yes	Myb-related p	pfam00249	Myb_DNA-binding	Myb-like D	GO:0006355|	GO:0005634|	GO:0003677|D	.
Manes.02G04620(	Inf	0.9009	up	no	Mini zinc fing	pfam04770	ZF-HD_dimer	ZF-HD prot	GO:0007275|	GO:0005737|	GO:0003677|D	.
Manes.02G04690(	0.485474	4.02E-10	down	yes	Granule-bour	pfam08323	Glyco_transf_5	Starch synth	GO:0010021|	GO:0009501|	GO:0004373|gly	K00703
Manes.02G04710(	2.1165613	1.17E-10	up	yes	Uncharacteriz	pfam08881	CVNH	CVNH dom	GO:0006412|	GO:0016021|	GO:0003735|str	.
Manes.02G04770(	37.195245	1.49E-06	up	yes	Casparian str	pfam04535	DUF588	Domain of u	GO:0071555|	GO:0016021|	.	.
Manes.02G04810(	1.9365158	7.10E-13	up	no	Cystathionine	pfam01053	Cys_Met_Meta_P	Cys/Met me	GO:0009086|	GO:0009507|	GO:0003962|cy	K01739
Manes.02G04820(	1.9506656	4.77E-14	up	no	SPX domain-	pfam03105	SPX	SPX domain	GO:0016036|	GO:0005737|	GO:0015114|ph	.
Manes.02G04830(	0.4763298	9.39E-06	down	yes	Phosphate tra	pfam03124	EXS	EXS family.	GO:0006817|	GO:0016021|	.	.
Manes.02G04870(	0.4627115	4.35E-10	down	yes	.	pfam09366	DUF1997	Protein of u	.	.	.	.
Manes.02G04880(	2.5327644	2.30E-05	up	yes	Pentatricope	pfam14432	DYW_deaminase	DYW family	.	GO:0005739|	.	.
Manes.02G04890(	1.9738463	4.23E-11	up	no	Vesicle transp	pfam05008	V-SNARE	Vesicle tran	GO:0006886|	GO:0005794|	.	K08493
Manes.02G04900(	0.6382403	2.89E-06	down	no	ARF guanine	pfam01369	Sec7	Sec7 domai	GO:0010540|	GO:0005829|	GO:0005086|A	K18443
Manes.02G04910(	0.77157	0.01723	down	no	CSC1-like pr	pfam02714	DUF221	Domain of u	GO:0006811|	GO:0016021|	.	.
Manes.02G04970(	2.4876162	5.45E-24	up	yes	Probable WR	pfam03106	WRKY	WRKY DN	GO:0006952|	GO:0005634|	GO:0003677|D	.
Manes.02G04990(	1.2495973	0.08329	up	no	.	pfam12204	DUF3598	Domain of u	.	.	.	.
Manes.02G05020(	2.8675028	5.67E-11	up	yes	Chorismate n	pfam10265	DUF2217	Uncharacter	GO:0009073|	GO:0009507|	GO:0004106|ch	K01850
Manes.02G05040(	13.752478	7.28E-32	up	yes	UPF0301 pro	pfam02622	DUF179	Uncharacter	.	.	.	K07735
Manes.02G05050(	26.145467	4.23E-62	up	yes	Hydroquinon	pfam00201	UDPGT	UDP-glucor	.	.	GO:0050505|hy	.
Manes.02G05080(	50.578148	1.61E-19	up	yes	.	pfam03803	Scramblase	Scramblase.	.	.	.	.

Manes.02G05090(	10.073197	1.89E-14	up	yes	Phosphatidyli	pfam10409	PTEN_C2	C2 domain (	GO:0007015|	GO:0051285|	GO:0008289|lip	K01110
Manes.02G05100(	0.4755279	1.95E-05	down	yes	Cyclic nucleo	pfam00027	cNMP_binding	Cyclic nucle	GO:0070588|	GO:0005887|	GO:0005262|ca	K05391
Manes.02G05120(	0.83386	0.19024	down	no	.	pfam13445	zf-RING_UBOX	RING-type	.	.	.	.
Manes.02G05130(	2.2248768	1.77E-07	up	yes	.	pfam12937	F-box-like	F-box-like.	.	.	.	.
Manes.02G05150(	0.7675783	0.03975	down	no	.	pfam13041	PPR_2	PPR repeat	.	.	.	.
Manes.02G05180(	1.7685856	8.01E-09	up	no	Probable gala	pfam01501	Glyco_transf_8	Glycosyl tra	GO:0052325|	GO:0005794|	GO:0047262|pc	K13648
Manes.02G05200(	Inf	0.08088	up	no	E3 ubiquitin-	pfam13639	zf-RING_2	Ring finger	GO:0016567|	.	GO:0016874|lig	K11982
Manes.02G05210(	1.2674032	0.06996	up	no	BAG family	pfam05793	TFIIF_alpha	Transcriptio	.	GO:0009507|	.	.
Manes.02G05220(	0.6222852	0.20787	down	no	Alpha-1,4 glu	pfam00343	Phosphorylase	Carbohydrat	GO:0005975|	GO:0009501|	GO:0008184|gl	.
Manes.02G05260(	0.6696199	0.00539	down	no	Alpha-1,4 glu	pfam00343	Phosphorylase	Carbohydrat	GO:0005975|	GO:0009501|	GO:0008184|gl	.
Manes.02G05330(	0.7421285	0.0078	down	no	AT-rich inter	pfam00249	Myb_DNA-binding	Myb-like DI	GO:0006355|	GO:0005634|	GO:0003677|DI	.
Manes.02G05340(	0.7462884	0.13201	down	no	.	pfam07779	Cas1_AcylT	10 TM Acyl	.	.	.	.
Manes.02G05380(	7.5659347	1.52E-28	up	yes	Biotin carbox	pfam00364	Biotin_lipoyl	Biotin-requi	GO:0006633|	GO:0009317|	GO:0003989|ac	K02160
Manes.02G05400(	0.9987529	0.99131	down	no	.	pfam12937	F-box-like	F-box-like.	.	.	.	.
Manes.02G05430(	2.588171	9.97E-06	up	yes	UDP-glycosy	pfam00201	UDPGT	UDP-glucor	.	.	GO:0016758|tra	.
Manes.02G05470(	0.7864224	0.01247	down	no	Protein RIK	pfam02949	7tm_6	7tm Odoran	.	GO:0005634|	GO:0003723|RI	.
Manes.02G05540(	1.3117803	0.00459	up	no	Ninja-family	pfam07897	DUF1675	Protein of u	GO:0009737|	GO:0005634|	.	.
Manes.02G05590(	0.4209978	1.84E-09	down	yes	Transcription	pfam03634	TCP	TCP family	GO:0030154|	GO:0009507|	GO:0003677|DI	.
Manes.02G05600(	0.2240209	1.13E-10	down	yes	Transcription	pfam07524	Bromo_TP	Bromodoma	GO:0045893|	GO:0005669|	.	K14649
Manes.02G05610(	1.7689625	1.93E-09	up	no	Protein LON	pfam14309	DUF4378	Domain of u	GO:0051513|	GO:0005829|	.	.
Manes.02G05620(	0.9529462	0.77803	down	no	.	pfam03031	NIF	NLI interact	.	.	.	.
Manes.02G05630(	0.9102473	0.47721	down	no	Putative meth	pfam12697	Abhydrolase_6	Alpha/beta l	.	GO:0009507|	GO:0016787|hy	.
Manes.02G05650(	0.6663412	0.0152	down	no	60S ribosoma	pfam00832	Ribosomal_L39	Ribosomal I	GO:0006412|	GO:0005840|	GO:0003735|str	K02924
Manes.02G05700(	0.4867885	6.38E-11	down	yes	Receptor-like	pfam12819	Malectin_like	Carbohydrat	GO:0009738|	GO:0043680|	GO:0005524|A	.
Manes.02G05730(	0.471603	4.08E-05	down	yes	Receptor-like	pfam12819	Malectin_like	Carbohydrat	GO:0009738|	GO:0043680|	GO:0005524|A	.
Manes.02G05740(	0.31732	1.72E-06	down	yes	.	pfam06630	Exonuc_VIII	Enterobacte	.	.	.	.
Manes.02G05750(	0.3924904	3.33E-05	down	yes	Receptor-like	pfam12819	Malectin_like	Carbohydrat	GO:0009738|	GO:0043680|	GO:0005524|A	.
Manes.02G05770(	1.6255916	7.98E-05	up	no	Receptor-like	pfam12819	Malectin_like	Carbohydrat	GO:0009738|	GO:0043680|	GO:0005524|A	.
Manes.02G05780(	0.7073756	0.01321	down	no	.	pfam10217	DUF2039	Uncharacter	.	.	.	.
Manes.02G05790(	13.983967	0.00157	up	yes	Probable met	pfam03141	Methyltransf_29	Putative S-a	.	GO:0005789|	GO:0008168|me	.
Manes.02G05850(	0.4655989	0.00034	down	yes	Cytochrome	pfam00067	p450	Cytochrome	.	GO:0016021|	GO:0020037|he	.
Manes.02G05950(	1.1004733	0.39502	up	no	E3 ubiquitin-	pfam13639	zf-RING_2	Ring finger	GO:0016567|	GO:0005737|	GO:0016874|lig	.
Manes.02G05960(	0.8005206	0.04813	down	no	.	pfam14817	HAUS5	HAUS augn	.	.	.	.
Manes.02G05970(	0.3896244	0.00025	down	yes	.	pfam01190	Pollen_Ole_e_I	Pollen prote	.	.	.	.
Manes.02G05990(	0.9644596	0.77465	down	no	Auxin transp	pfam13764	E3_UbLigase_R4	E3 ubiquitin	GO:0009926|	GO:0005829|	GO:0008270|zi	K10691
Manes.02G06010(	0.5213017	1.44E-05	down	no	Transcription	pfam00010	HLH	Helix-loop-l	GO:0006351|	GO:0005634|	GO:0003677|DI	.
Manes.02G06020(	1.9209592	3.32E-13	up	no	Uncharacteriz	pfam10250	O-FucT	GDP-fucose	.	GO:0005768|	GO:0016757|tra	.
Manes.02G06030(	0.4747538	0.17151	down	no	.	pfam15395	DUF4617	Domain of u	.	.	.	.
Manes.02G06050(	0.3371454	7.81E-17	down	yes	Phosphatidyli	pfam00454	PI3_PI4_kinase	Phosphatidy	GO:0046854|	.	GO:0004430|1-	.
Manes.02G06060(	0.9074424	0.37274	down	no	Probable leuc	pfam00069	Pkinase	Protein kina	GO:0009755|	GO:0016021|	GO:0005524|A	.
Manes.02G06070(	NA	NA	--	no	.	pfam09532	FDF	FDF domair	.	.	.	.
Manes.02G06080(	0.754151	0.01248	down	no	.	pfam03350	UPF0114	Uncharacter	.	.	.	.
Manes.02G06110(	0.9068487	0.51249	down	no	Alpha/beta hy	pfam12695	Abhydrolase_5	Alpha/beta l	.	GO:0005576|	GO:0016787|hy	.
Manes.02G06120(	1.1589614	0.46554	up	no	.	pfam04925	SHQ1	SHQ1 prote	.	.	.	.
Manes.02G06130(	1.474493	0.00047	up	no	DNA-directe	pfam00940	RNA_pol	DNA-depen	GO:0006351|	GO:0009507|	GO:0003677|DI	.
Manes.02G06140(	2.198636	7.13E-19	up	yes	Sodium-coup	pfam01490	Aa_trans	Transmemb	GO:0003333|	GO:0005887|	GO:0015171|an	K14207
Manes.02G06160(	0.4031567	3.64E-05	down	yes	.	pfam13041	PPR_2	PPR repeat	.	.	.	.
Manes.02G06210(	3.3306417	2.88E-05	up	yes	.	pfam12222	PNGaseA	Peptide N-a	.	.	.	.
Manes.02G06270(	2.4099597	6.32E-22	up	yes	Zinc finger p	pfam06203	CCT	CCT motif.	GO:0009658|	GO:0005634|	GO:0003677|DI	.
Manes.02G06300(	1.6929879	7.59E-06	up	no	Pentatricope	pfam13041	PPR_2	PPR repeat	.	GO:0005739|	.	.
Manes.02G06310(	9.5798796	0.00309	up	yes	.	pfam02196	RBD	Raf-like Ras	.	.	.	.
Manes.02G06350(	7.8233458	#######	up	yes	S-adenosylme	pfam01536	SAM_decarbox	Adenosylme	GO:0006557|	.	GO:0004014|ad	.
Manes.02G06360(	9.1209575	#######	up	yes	.	pfam08132	AdoMetDC_leade	S-adenosyl-	.	.	.	.
Manes.02G06370(	0.4922989	7.01E-10	down	yes	EVI5-like pro	pfam00566	RabGAP-TBC	Rab-GTPas	GO:1902018|	.	GO:0005096|G	.
Manes.02G06430(	0.3672463	3.26E-13	down	yes	Uncharacteriz	pfam01370	Epimerase	NAD depen	GO:0006979|	GO:0009507|	.	.
Manes.02G06460(	0.2134555	2.88E-05	down	yes	.	pfam06880	DUF1262	Protein of u	.	.	.	.
Manes.02G06480(	0.8914912	0.37795	down	no	.	pfam05138	PaaA_PaaC	Phenylacetic	.	.	.	.
Manes.02G06540(	2.6570585	0.15806	up	no	Tetraketide a	pfam01073	3Beta_HSD	3-beta hydro	GO:0009555|	GO:0005783|	GO:0050662|co	.
Manes.02G06560(	1.350533	0.00094	up	no	Isoprenylcyst	pfam00135	COesterase	Carboxylest	.	GO:0005789|	GO:0010296|pr	K15889
Manes.02G06590(	2.5602866	0.0009	up	yes	Protein GAM	pfam13360	PQQ_2	PQQ-like do	GO:0009793|	GO:0016021|	.	.
Manes.02G06610(	1.603692	1.29E-07	up	no	Cyclin-B1-2	pfam05348	UMP1	Proteasome	GO:0007049|	.	.	.
Manes.02G06620(	0.9909498	0.941	down	no	Translation ir	pfam01008	IF-2B	Initiation fac	GO:0044267|	GO:0005737|	GO:0003743|tra	K03680
Manes.02G06630(	15.906836	4.24E-11	up	yes	DNA-directe	pfam03870	RNA_pol_Rpb8	RNA polym	GO:0006360|	GO:0005736|	.	K03016
Manes.02G06640(	#NAME?	0.14049	down	no	Transcription	pfam03634	TCP	TCP family	GO:0009908|	GO:0005634|	GO:0003677|DI	.
Manes.02G06650(	0.5436028	0.26976	down	no	.	pfam08137	DVL	DVL family	.	.	.	.
Manes.02G06690(	0.4577671	1.74E-07	down	yes	Rhomboid pr	pfam01694	Rhomboid	Rhomboid f	.	GO:0016021|	GO:0004252|se	K19225
Manes.02G06700(	1.4073701	0.02268	up	no	.	pfam05512	AWPM-19	AWPM-19-	.	.	.	.
Manes.02G06710(	1.9628283	4.43E-11	up	no	Ultraviolet-B	pfam00415	RCC1	Regulator o	GO:0009649|	GO:0000785|	GO:0003682|ch	.
Manes.02G06720(	1.3906809	0.00024	up	no	.	pfam00244	2014/3/3	14-3-3 prote	.	.	.	.
Manes.02G06750(	0.8370107	0.06506	down	no	.	pfam12717	Cnd1	non-SMC m	.	.	.	.
Manes.02G06760(	1.0969082	0.9039	up	no	.	pfam03398	Ist1	Regulator o	.	.	.	.
Manes.02G06780(	1.2824528	0.00682	up	no	Ubiquitin rec	pfam09280	XPC-binding	XPC-bindin	GO:0006289|	GO:0005737|	GO:0003684|da	.
Manes.02G06790(	0.4352823	1.58E-05	down	yes	.	pfam12004	DUF3498	Domain of u	.	.	.	.
Manes.02G06820(	0.6695566	0.10884	down	no	.	pfam15197	Leukemia_assc_2	Leukemia-a	.	.	.	.
Manes.02G06830(	0.5990228	6.47E-05	down	no	KH domain-c	pfam00013	KH_1	KH domain.	.	GO:0005634|	GO:0003723|RI	K13162
Manes.02G06850(	2.4431603	2.44E-22	up	yes	.	pfam04724	Glyco_transf_17	Glycosyltrar	.	.	.	.
Manes.02G06860(	0.5433482	1.46E-05	down	no	.	pfam14108	DUF4281	Domain of u	.	.	.	.
Manes.02G06870(	0.9018539	0.40012	down	no	.	pfam00892	EamA	EamA-like t	.	.	.	.
Manes.02G06890(	0.7457355	0.00557	down	no	Mannose-6-p	pfam12697	Abhydrolase_6	Alpha/beta l	GO:0009298|	.	GO:0004476|m	K01809
Manes.02G06970(	3.1421021	9.92E-25	up	yes	Chloroplastic	pfam01195	Pept_tRNA_hydro	Peptidyl-tRI	GO:0006397|	GO:0009570|	GO:0004045|an	K01056
Manes.02G07020(	6.7435677	0.00013	up	yes	Probable rece	pfam00069	Pkinase	Protein kina	.	GO:0016021|	GO:0005524|A	.
Manes.02G07030(	0.6818974	0.00061	down	no	Alpha/beta hy	pfam12695	Abhydrolase_5	Alpha/beta l	.	GO:0005576|	GO:0016787|hy	.
Manes.02G07040(	0.5038108	0.01578	down	no	Peptide meth	pfam01625	PMSR	Peptide met	GO:0006464|	.	GO:0008113|pe	K07304
Manes.02G07060(	4.3590882	1.25E-10	up	yes	Probable seri	pfam00069	Pkinase	Protein kina	GO:0071215|	GO:0005886|	GO:0005524|A	.
Manes.02G07110(	Inf	0.35135	up	no	Non-specific	pfam14368	LTP_2	Probable lip	GO:0006810|	.	GO:0008289|lip	.
Manes.02G07140(	1.0251694	0.76622	up	no	.	pfam05622	HOOK	HOOK prot	.	.	.	.
Manes.02G07150(	0.3422184	5.26E-23	down	yes	Protein PLAS	pfam05701	WEMBL	Weak chlor	GO:0009904|	GO:0005829|	.	.
Manes.02G07170(	4.249059	2.80E-54	up	yes	.	pfam00564	PB1	PB1 domain	.	.	.	.

Manes.02G07190(	0.5844299	0.00982	down	no	.	pfam11377	DUF3180	Protein of u	.	.	.	.
Manes.02G07220(	2.8570573	2.26E-11	up	yes	.	pfam10317	7TM_GPCR_Srd	Serpentine t	.	.	.	.
Manes.02G07240(	11.515657	0.01724	up	yes	MATE efflux	pfam01554	MatE	MatE. The I	.	GO:0016021|	GO:0015297|an	K03327
Manes.02G07250(	0.5734957	0.01752	down	no	Histone chap	pfam04729	ASF1_hist_chap	ASF1 like h	GO:0051301|	GO:0005737|	.	K10753
Manes.02G07260(	0.5319325	2.56E-09	down	no	DNA ligase 1	pfam04675	DNA_ligase_A_N	DNA ligase	GO:0007049|	GO:0005737|	GO:0005524|A'	K10747
Manes.02G07270(	8.0900441	0.00042	up	yes	.	pfam14009	DUF4228	Domain of u	.	.	.	.
Manes.02G07330(	0.4495627	8.49E-07	down	yes	E3 ubiquitin-	pfam12428	DUF3675	Protein of u	GO:0006897|	GO:0031410|	GO:0016874|lig	K10657
Manes.02G07360(	3.1506342	0.00631	up	yes	.	pfam05687	DUF822	Plant protei	.	.	.	.
Manes.02G07390(	0.3422656	3.51E-11	down	yes	Ell-associate	pfam09816	EAF	RNA polym	GO:0007275|	GO:0032783|	.	K15186
Manes.02G07410(	0.8783925	0.40841	down	no	Putative E3 u	pfam03145	Sina	Seven in abs	GO:0007275|	GO:0005634|	GO:0016874|lig	K04506
Manes.02G07420(	Inf	0.02254	up	yes	Heavy metal-	pfam00403	HMA	Heavy-meta	GO:0010286|	GO:0031965|	GO:0046872|m	.
Manes.02G07460(	3.97865	0.39698	up	no	.	pfam02993	MCPVI	Minor capsi	.	.	.	.
Manes.02G07480(	2.1363797	2.92E-17	up	yes	Protease Do-	pfam12812	PDZ_1	PDZ-like do	GO:0010205|	GO:0009507|	GO:0008233|pe	.
Manes.02G07500(	1.5347875	1.73E-05	up	no	U-box domai	pfam00514	Arm	Armadillo/b	.	.	GO:0016874|lig	.
Manes.02G07520(	0.7019971	0.00487	down	no	Serine/threon	pfam00069	Pkinase	Protein kina	GO:0009742|	GO:0005886|	GO:0005524|A'	.
Manes.02G07540(	6.5912112	4.52E-18	up	yes	Probable calc	pfam13499	EF-hand_7	EF-hand do	GO:0009909|	.	GO:0005509|ca	K13448
Manes.02G07560(	1.3511079	0.00992	up	no	Probable AD	pfam00168	C2	C2 domain.	.	.	GO:0005096|G'	K12486
Manes.02G07570(	0.5424893	2.05E-07	down	no	.	pfam15619	Lebercilin	Ciliary prot	.	.	.	.
Manes.02G07590(	0.3711027	2.61E-16	down	yes	DELLA prot	pfam03514	GRAS	GRAS doma	GO:0009740|	GO:0005634|	GO:0000989|tra	.
Manes.02G07630(	1.6594042	0.05679	up	no	40S ribosom	pfam01200	Ribosomal_S28e	Ribosomal p	GO:0006412|	GO:0005618|	GO:0003735|str	K02979
Manes.02G07640(	0.926931	0.46408	down	no	Gamma-tubu	pfam04130	Spc97_Spc98	Spc97 / Spc	GO:0007020|	GO:0008274|	GO:0008017|mi	.
Manes.02G07650(	1.0307028	0.80085	up	no	.	pfam10151	DUF2359	Uncharacter	.	.	.	.
Manes.02G07700(	0.8153092	0.09473	down	no	.	pfam01764	Lipase_3	Lipase (clas	.	.	.	.
Manes.02G07730(	0.8011536	0.05414	down	no	.	pfam06161	DUF975	Protein of u	.	.	.	.
Manes.02G07740(	0.354551	2.09E-05	down	yes	Protein ODO	pfam00249	Myb_DNA-binding	Myb-like DI	GO:0006355|	GO:0005634|	GO:0003677|DI	.
Manes.02G07760(	2.9102044	4.45E-14	up	yes	Probable inac	pfam00069	Pkinase	Protein kina	GO:0009755|	GO:0016021|	GO:0005524|A'	.
Manes.02G07800(	1.016582	0.87494	up	no	Eukaryotic tr	pfam01398	JAB	JAB1/Mov3	GO:0001731|	GO:0005829|	GO:0003743|tra	K03247
Manes.02G07840(	0.8276683	0.13281	down	no	Aspartic prot	pfam14541	TAXi_C	Xylanase inl	GO:0012501|	GO:0005783|	GO:0004190|as	.
Manes.02G07860(	1.0016565	0.98563	up	no	Nicastrin	pfam05450	Nicastrin	Nicastrin. N	GO:0007219|	GO:0016021|	.	.
Manes.02G07890(	Inf	1.14E-51	up	yes	.	pfam10395	Utp8	Utp8 family	.	.	.	.
Manes.02G07930(	5.1614569	6.21E-59	up	yes	Zinc finger p	pfam13912	zf-C2H2_6	C2H2-type	GO:0006355|	GO:0005737|	GO:0046872|m	.
Manes.02G07940(	3.4231478	0.01877	up	yes	Protein GAM	pfam05911	DUF869	Plant protei	GO:0009790|	GO:0005887|	GO:0042802|id	.
Manes.02G07950(	0.6750527	0.00101	down	no	Uncharacteri	pfam01408	GFO_IDH_MocA	Oxidoreduc	GO:0055114|	GO:0005829|	GO:0016491|ox	.
Manes.02G07960(	5.0656836	0.00033	up	yes	Importin-5	pfam04510	DUF577	Family of ur	GO:0071230|	GO:0005737|	GO:0044822|pc	.
Manes.02G07970(	0.6335088	0.00081	down	no	Transcription	pfam00249	Myb_DNA-binding	Myb-like DI	GO:0010158|	GO:0005634|	GO:0003677|DI	.
Manes.02G07980(	0.7910513	0.04048	down	no	.	pfam02134	UBACT	Repeat in ul	.	.	.	.
Manes.02G07990(	Inf	0.02272	up	yes	.	pfam06886	TPX2	Targeting pr	.	.	.	.
Manes.02G08020(	1.1721454	0.19263	up	no	Protein FAR1	pfam03101	FAR1	FAR1 DNA	GO:0006355|	GO:0005634|	GO:0008270|zi	.
Manes.02G08040(	1.1758262	0.1668	up	no	Glutamate de	pfam00282	Pyridoxal_deC	Pyridoxal-de	GO:0006536|	GO:0005829|	GO:0005516|ca	K01580
Manes.02G08050(	1.4032615	0.00043	up	no	Isocitrate del	pfam00180	Iso_dh	Isocitrate/is	GO:0006097|	GO:0005737|	GO:0004450|is	.
Manes.02G08070(	7.2734927	5.17E-46	up	yes	Protein REV	pfam00249	Myb_DNA-binding	Myb-like DI	GO:0009851|	GO:0005634|	GO:0003677|DI	.
Manes.02G08140(	0.4782791	7.77E-09	down	yes	ALA-interact	pfam03381	CDC50	LEM3 (ligar	GO:0015914|	GO:0005789|	.	.
Manes.02G08150(	0.8760062	0.23818	down	no	Sucrose syntl	pfam00862	Sucrose_synth	Sucrose syn	GO:0009058|	GO:0005618|	GO:0016157|su	.
Manes.02G08170(	1.1506376	0.17637	up	no	Phytoene syn	pfam00494	SQS_PSY	Squalene/ph	GO:0016117|	GO:0009507|	GO:0016767|ge	.
Manes.02G08210(	Inf	0.9009	up	no	Probable poly	pfam01593	Amino_oxidase	Flavin conta	GO:0006598|	GO:0005777|	GO:0052895|N	K17839
Manes.02G08250(	1.6404767	5.28E-08	up	no	Auxin respon	pfam06507	Auxin_resp	Auxin respo	GO:0009734|	GO:0005634|	GO:0003677|DI	.
Manes.02G08280(	0.896657	0.25774	down	no	Probable star	pfam08323	Glyco_transf_5	Starch synth	GO:0019252|	GO:0009501|	GO:0004373|gl	.
Manes.02G08290(	0.7872404	0.02076	down	no	Serine/threon	pfam07714	Pkinase_Tyr	Protein tyro	.	.	GO:0005524|A'	.
Manes.02G08310(	0.6151359	0.00666	down	no	Serine/threon	pfam07714	Pkinase_Tyr	Protein tyro	.	.	GO:0005524|A'	.
Manes.02G08350(	13.157838	6.50E-06	up	yes	Alpha-amylas	pfam00197	Kunitz_legume	Trypsin and	GO:0010951|	.	GO:0015066|al	.
Manes.02G08390(	Inf	0.00069	up	yes	Taxadiene 5-	pfam00067	p450	Cytochrome	GO:0042617|	GO:0016021|	GO:0020037|he	.
Manes.02G08420(	0.6250904	0.00053	down	no	.	pfam01576	Myosin_tail_1	Myosin tail.	.	.	.	.
Manes.02G08430(	0.4834754	5.91E-06	down	yes	Transcription	pfam14897	EpsG	EpsG family	GO:0009742|	GO:0005634|	GO:0042803|pr	.
Manes.02G08440(	0.3740668	4.70E-18	down	yes	Mitogen-acti	pfam00069	Pkinase	Protein kina	.	GO:0005622|	GO:0005524|A'	.
Manes.02G08460(	0.5811291	0.00132	down	no	Glucan endo-	pfam07983	X8	X8 domain.	GO:0005975|	GO:0031225|	GO:0042973|gl	.
Manes.02G08470(	0.3848532	1.19E-06	down	yes	.	pfam04450	BSP	Peptidase of	.	.	.	.
Manes.02G08490(	0.7407399	0.00997	down	no	Callose synth	pfam02364	Glucan_synthase	1,3-beta-glu	GO:0006075|	GO:0000148|	GO:0003843|1,	K11000
Manes.02G08520(	1.3753037	0.0004	up	no	tRNA uridine	pfam01134	GIDA	Glucose inh	GO:0002098|	GO:0005737|	GO:0050660|fla	K03495
Manes.02G08590(	0.3319988	3.40E-05	down	yes	ATP-depend	pfam05970	PIF1	PIF1-like he	GO:0006310|	GO:0005739|	GO:0005524|A'	K15255
Manes.02G08600(	0.783032	0.01934	down	no	Serine/threon	pfam00069	Pkinase	Protein kina	.	.	GO:0005524|A'	.
Manes.02G08670(	0.6363978	1.31E-05	down	no	Serine/threon	pfam08321	PPP5	PPP5 TPR r	GO:0010019|	GO:0005737|	GO:0046872|m	K04460
Manes.02G08680(	0.4013639	2.02E-05	down	yes	Protein TOO	pfam13855	LRR_8	Leucine rich	GO:0008356|	GO:0031225|	GO:0004672|pr	.
Manes.02G08690(	1.5971455	6.86E-07	up	no	Citrate synth	pfam00285	Citrate_synt	Citrate syntl	GO:0006097|	GO:0009514|	GO:0046912|tra	.
Manes.02G08710(	0.4507747	1.81E-09	down	yes	Actin-related	pfam00400	WD40	WD domain	GO:0007015|	GO:0005885|	GO:0051015|ac	K05757
Manes.02G08740(	26.891379	4.20E-31	up	yes	Heat stress tr	pfam00447	HSF_DNA-bind	HSF-type D	GO:0045893|	GO:0005737|	GO:0042803|pr	K09419
Manes.02G08760(	10.500358	2.14E-43	up	yes	G-type lectin	pfam00069	Pkinase	Protein kina	GO:0009755|	GO:0016021|	GO:0005524|A'	.
Manes.02G08770(	6.9911392	6.93E-51	up	yes	Pentatricope	pfam13041	PPR_2	PPR repeat	.	GO:0005739|	.	.
Manes.02G08780(	5.3508902	2.04E-42	up	yes	.	pfam06972	DUF1296	Protein of u	.	.	.	.
Manes.02G08790(	30.395342	2.80E-11	up	yes	AT-hook mo	pfam03479	DUF296	Domain of u	GO:0006325|	GO:0005634|	GO:0003680|A'	.
Manes.02G08840(	0.6954741	0.00146	down	no	Cytochrome	pfam03188	Cytochrom_B561	Eukaryotic	GO:0055114|	GO:0016021|	GO:0046872|m	.
Manes.02G08850(	0.9683145	0.74736	down	no	Shaggy-relat	pfam00069	Pkinase	Protein kina	GO:0009742|	.	GO:0005524|A'	K00924
Manes.02G08880(	1.5336117	0.00332	up	no	Transmembr	pfam03798	TRAM_LAG1_Cl	TLC domain	.	GO:0016021|	.	.
Manes.02G08890(	6.2672388	5.97E-96	up	yes	4-hydroxyph	pfam00903	Glyoxalase	Glyoxalase/I	GO:0006559|	GO:0005737|	GO:0003868|4-	.
Manes.02G08940(	1.0805114	0.4592	up	no	Persulfide dic	pfam00753	Lactamase_B	Metallo-beta	GO:0009793|	GO:0005739|	GO:0016788|hy	K17725
Manes.02G08950(	0.729159	0.57689	down	no	.	pfam03798	TRAM_LAG1_Cl	TLC domain	.	.	.	.
Manes.02G08980(	0.4773686	8.99E-13	down	yes	WD repeat-c	pfam00400	WD40	WD domain	.	GO:0005737|	.	.
Manes.02G09000(	0.7400789	0.00573	down	no	Reticulon-lik	pfam02453	Reticulon	Reticulon. F	.	GO:0005789|	.	.
Manes.02G09010(	0.354393	3.27E-05	down	yes	.	pfam10835	DUF2573	Protein of u	.	.	.	.
Manes.02G09020(	9.8077326	0.03169	up	no	.	pfam15084	DUF4550	Domain of u	.	.	.	.
Manes.02G09050(	0.9565289	0.69583	down	no	Probable poly	pfam01593	Amino_oxidase	Flavin conta	GO:0006598|	GO:0005777|	GO:0046592|pc	K17839
Manes.02G09060(	0.4641758	3.44E-07	down	yes	N-acetyl-D-g	pfam01869	BcrAD_BadFG	BadF/BadG	.	.	GO:0005524|A'	.
Manes.02G09080(	0.793334	0.01605	down	no	Serine/threon	pfam14381	EDR1	Ethylene-res	GO:0071281|	GO:0005789|	GO:0005524|A'	K14510
Manes.02G09110(	0.3343062	9.31E-09	down	yes	E3 ubiquitin-	pfam00415	RCC1	Regulator o	GO:0006974|	GO:0005814|	GO:0016874|lig	K10595
Manes.02G09200(	17.321639	5.48E-28	up	yes	E3 ubiquitin-	pfam08783	DWNN	DWNN don	GO:0048568|	GO:0005694|	GO:0016874|lig	K10624
Manes.02G09220(	2.3955269	3.61E-22	up	yes	Transcription	pfam00382	TFIIB	Transcriptio	GO:0051123|	GO:0005634|	GO:0001139|tra	K03124
Manes.02G09230(	2.5924695	2.62E-24	up	yes	.	pfam14580	LRR_9	Leucine-ricl	.	.	.	.
Manes.02G09240(	9.0550496	0.00494	up	yes	.	pfam00646	F-box	F-box doma	.	.	.	.
Manes.02G09260(	1.0149853	0.84559	up	no	U1 small nuc	pfam00076	RRM_1	RNA recogr	GO:0000398|	GO:0016607|	GO:0000166|nu	K11093

Manes.02G09270(	0.1634498	3.16E-08	down	yes	.	pfam01925	TauE	Sulfite expo	.	.	.	.
Manes.02G09300(	0.4727896	0.01766	down	yes	.	pfam09973	DUF2208	Predicted m	.	.	.	.
Manes.02G09330(	1.0809445	0.38748	up	no	Scarecrow-lil	pfam03514	GRAS	GRAS doma	GO:0006355|	GO:0005634|	GO:0003700|tra	.
Manes.02G09360(	1.1938084	0.11501	up	no	Protein AE7	pfam09615	Cas_Csy3	CRISPR-ass	GO:0007059|	GO:0005737|	.	.
Manes.02G09370(	0.6564993	1.87E-05	down	no	Vam6/Vps39	pfam10367	Vps39_2	Vacuolar so	GO:0006886|	GO:0030897|	.	.
Manes.02G09390(	Inf	0.35135	up	no	.	pfam07364	DUF1485	Protein of u	.	.	.	.
Manes.02G09470(	2.2512112	1.64E-13	up	yes	ATPase famil	pfam12037	DUF3523	Domain of u	GO:0007005|	GO:0016021|	GO:0005524|A'	K17681
Manes.02G09500(	2.431012	7.02E-21	up	yes	U-box domai	pfam04564	U-box	U-box doma	.	.	GO:0016874|lig	.
Manes.02G09510(	3.1759059	2.92E-07	up	yes	Transcription	pfam00010	HLH	Helix-loop-l	GO:0009742|	GO:0005634|	GO:0003677|D	.
Manes.02G09520(	0.4372828	6.02E-05	down	yes	Anaphase-pro	pfam03256	APC10	Anaphase-p	GO:0031145|	GO:0005680|	.	K03357
Manes.02G09530(	0.8800651	0.2333	down	no	Tubby-like F	pfam01167	Tub	Tub family.	GO:0006355|	GO:0005829|	GO:0035091|ph	.
Manes.02G09550(	1.1361111	0.19266	up	no	Putative gluc	pfam01263	Aldose_epim	Aldose 1-ep	GO:0005975|	.	GO:0030246|ca	.
Manes.02G09560(	0.510775	9.09E-07	down	no	Phosphopant	pfam01467	CTP_transf_2	Cytidylyltra	GO:0015937|	GO:0005829|	GO:0005524|A'	K02201
Manes.02G09570(	1.0899895	0.35699	up	no	Pheophytinas	pfam12697	Abhydrolase_6	Alpha/beta l	GO:0015996|	GO:0009507|	GO:0080124|ph	.
Manes.02G09580(	0.3663483	1.18E-07	down	yes	Protein RER	pfam03248	Rer1	Rer1 family.	GO:0006890|	GO:0005801|	.	.
Manes.02G09620(	2.9870512	8.95E-08	up	yes	.	pfam04006	Mpp10	Mpp10 prot	.	.	.	.
Manes.02G09650(	0.730598	0.00169	down	no	GTPase Era	pfam01926	MMR_HSR1	50S ribosom	GO:0042274|	GO:0005737|	GO:0005525|G'	K03595
Manes.02G09670(	0.6581485	0.05156	down	no	.	pfam04515	Choline_transpo	Plasma-men	.	.	.	.
Manes.02G09730(	8.7186286	4.55E-70	up	yes	Probable mit	pfam14363	AAA_assoc	Domain ass	GO:0051131|	GO:0016021|	GO:0005524|A'	K08900
Manes.02G09760(	0.5569016	0.00053	down	no	Phosphatidyli	pfam00650	CRAL_TRIO	CRAL/TRIC	GO:0015031|	GO:0000139|	.	.
Manes.02G09800(	0.6322324	9.50E-05	down	no	.	pfam09814	HECT_2	HECT-like l	.	.	.	.
Manes.02G09810(	14.767656	#######	up	yes	Probable mit	pfam00004	AAA	ATPase fam	GO:0016226|	GO:0016021|	GO:0005524|A'	K08900
Manes.02G09830(	0.7193035	0.00317	down	no	.	pfam00534	Glycos_transf_1	Glycosyl tra	.	.	.	.
Manes.02G09850(	0.8261607	0.14332	down	no	50S ribosoma	pfam00542	Ribosomal_L12	Ribosomal p	GO:0006412|	GO:0005840|	GO:0003735|str	.
Manes.02G09860(	Inf	0.02254	up	yes	.	pfam13947	GUB_WAK_bind	Wall-associa	.	.	.	.
Manes.02G09870(	4.6357311	1.26E-14	up	yes	Bifunctional	pfam12697	Abhydrolase_6	Alpha/beta l	GO:0019369|	GO:0005737|	GO:0033885|1(	K08726
Manes.02G09900(	0.6791328	0.00028	down	no	3-epi-6-deox	pfam00067	p450	Cytochrome	GO:0016132|	GO:0005789|	GO:0020037|he	K12637
Manes.02G09930(	0.3958311	0.0005	down	yes	Probable dim	pfam00398	RrnaAD	Ribosomal F	.	GO:0005730|	GO:0052909|18	K14191
Manes.02G09940(	0.3428003	7.76E-11	down	yes	ADP-ribosyla	pfam00025	Arf	ADP-ribosy	GO:0009793|	GO:0005737|	GO:0005525|G'	K07943
Manes.02G09960(	0.6184757	1.03E-06	down	no	Beta-galactos	pfam01301	Glyco_hydro_35	Glycosyl hy	GO:0005975|	GO:0048046|	GO:0004565|be	.
Manes.02G09990(	1.3589808	0.00433	up	no	MORC famil	pfam13589	HATPase_c_3	Histidine kin	GO:0007569|	GO:0045111|	GO:0008270|zi	.
Manes.02G10010(	0.5837367	0.00055	down	no	Protein SHI	pfam05142	DUF702	Domain of u	GO:0009851|	GO:0005634|	GO:0003677|D	.
Manes.02G10050(	0.7049135	0.00244	down	no	.	pfam01724	DUF29	Domain of u	.	.	.	.
Manes.02G10060(	0.4418976	1.44E-12	down	yes	Microspherul	pfam13325	MCRS_N	N-terminal	GO:0006464|	GO:0015629|	.	K11674
Manes.02G10070(	0.3660063	1.11E-14	down	yes	Receptor-like	pfam13855	LRR_8	Leucine rich	GO:0009755|	GO:0016021|	GO:0004672|pr	.
Manes.02G10080(	4.8696363	0.00117	up	yes	Cytochrome	pfam00067	p450	Cytochrome	GO:0009809|	GO:0005783|	GO:0020037|he	K09755
Manes.02G10110(	0.512128	3.90E-06	down	no	Transmembra	pfam05277	DUF726	Protein of u	.	GO:0016021|	.	.
Manes.02G10120(	0.7917982	0.01552	down	no	Zinc finger C	pfam03126	Plus-3	Plus-3 doma	GO:0006352|	GO:0005634|	GO:0003677|D	.
Manes.02G10130(	1.2387298	0.02174	up	no	.	pfam03676	UPF0183	Uncharacter	.	.	.	.
Manes.02G10150(	0.3819658	4.34E-12	down	yes	.	pfam00885	DMRL_synthase	6,7-dimethy	.	.	.	.
Manes.02G10170(	0.5483262	1.20E-07	down	no	Probable seri	pfam05577	Peptidase_S28	Serine carbo	GO:0009561|	GO:0005576|	GO:0008236|se	.
Manes.02G10190(	0.7784806	0.01508	down	no	Kinesin-like	pfam00225	Kinesin	Kinesin mot	GO:0000919|	GO:0005871|	GO:0005524|A'	.
Manes.02G10250(	1.0203113	0.80903	up	no	Heterogeneo	pfam00076	RRM_1	RNA recogi	GO:0007623|	GO:0070937|	GO:0000166|nu	K13160
Manes.02G10270(	1.0283876	0.82978	up	no	.	pfam10805	DUF2730	Protein of u	.	.	.	.
Manes.02G10290(	0.7105029	0.00151	down	no	Zinc finger C	pfam05109	Herpes_BLLF1	Herpes viru	.	.	GO:0003677|D	.
Manes.02G10340(	0.6785453	0.00084	down	no	Transcription	pfam03366	YEATS	YEATS fam	GO:0030154|	GO:0005737|	.	K11341
Manes.02G10360(	0.8693512	0.20661	down	no	.	pfam13851	GAS	Growth-arre	.	.	.	.
Manes.02G10380(	0.5013487	1.27E-11	down	no	Endoglucana	pfam00759	Glyco_hydro_9	Glycosyl hy	GO:0071555|	GO:0005576|	GO:0008810|ce	.
Manes.02G10390(	1.7985396	5.02E-11	up	no	RNA demeth	pfam13532	2OG-FeII_Oxy_2	2OG-Fe(II)	GO:0006406|	GO:0016607|	GO:0046872|m	K10767
Manes.02G10400(	Inf	5.51E-05	up	yes	Protein WUS	pfam00046	Homeobox	Homeobox	GO:0030154|	GO:0005634|	GO:0003677|D	.
Manes.02G10410(	0.8020296	0.04594	down	no	WD repeat-c	pfam00400	WD40	WD domain	.	GO:0005634|	.	K14962
Manes.02G10460(	3.8350071	0.00039	up	yes	Snakin-2	pfam02704	GASA	Gibberellin	GO:0006952|	GO:0005618|	.	.
Manes.02G10480(	1.3588224	0.00077	up	no	Calcium-dep	pfam00069	Pkinase	Protein kina	GO:0009738|	GO:0005737|	GO:0005524|A'	K13412
Manes.02G10490(	2.0184856	4.03E-07	up	yes	Chaperone p	pfam00226	DnaJ	DnaJ domai	.	GO:0009507|	.	.
Manes.02G10500(	2.6663938	2.29E-29	up	yes	.	pfam00514	Arm	Armadillo/b	.	.	.	.
Manes.02G10520(	3.6465225	2.64E-10	up	yes	Cold shock d	pfam00313	CSD	'Cold-shock	GO:0009631|	GO:0005737|	GO:0003677|D	K09250
Manes.02G10530(	2.5711176	5.38E-18	up	yes	Thaumatin-lil	pfam00314	Thaumatin	Thaumatin f	GO:0006952|	GO:0005576|	.	.
Manes.02G10540(	4.0986507	3.93E-48	up	yes	Thaumatin-lil	pfam00314	Thaumatin	Thaumatin f	GO:0006952|	GO:0005576|	.	.
Manes.02G10550(	6.8835076	3.42E-23	up	yes	.	pfam09223	YodA	YodA lipoca	.	.	.	.
Manes.02G10690(	0.567868	0.00517	down	no	Oligopeptide	pfam03169	OPT	OPT oligop	GO:0015833|	GO:0016021|	.	.
Manes.02G10720(	0.8790314	0.18567	down	no	3-dehydroqui	pfam01761	DHQ_synthase	3-dehydroq	GO:0009073|	GO:0009507|	GO:0003856|3-	.
Manes.02G10740(	0.6961205	0.00374	down	no	Rac-like GTI	pfam00071	Ras	Ras family.	GO:0009738|	GO:0005737|	GO:0005525|G'	K04392
Manes.02G10750(	1.3013779	0.00374	up	no	Vacuolar pro	pfam03635	Vps35	Vacuolar pr	GO:0042147|	GO:0005794|	GO:0008565|pr	K18468
Manes.02G10760(	0.5932406	0.00261	down	no	.	pfam02144	Rad1	Repair prote	.	.	.	.
Manes.02G10820(	0.7079271	0.00032	down	no	Protein MID	pfam04749	PLAC8	PLAC8 fam	GO:0006816|	GO:0005887|	.	.
Manes.02G10910(	0.9240071	0.43132	down	no	La-related p	pfam05383	La	La domain.	GO:0009738|	GO:0005737|	GO:0003723|R	K18757
Manes.02G10950(	1.7342626	3.36E-09	up	no	Aspartic prot	pfam14543	TAXi_N	Xylanase inl	.	GO:0031225|	GO:0004190|as	.
Manes.02G10980(	1.2401361	0.01618	up	no	Probable prot	pfam00481	PP2C	Protein phos	GO:0006470|	GO:0016021|	GO:0046872|m	.
Manes.02G11010(	1.949029	1.42E-10	up	no	NEP1-intera	pfam13639	zf-RING_2	Ring finger	.	GO:0009535|	GO:0008270|zi	.
Manes.02G11060(	0.9378834	0.61811	down	no	Phospholipas	pfam12357	PLD_C	Phospholipa	GO:0016042|	GO:0016020|	GO:0005509|ca	K01115
Manes.02G11090(	1.1150702	0.34093	up	no	Bifunctional	pfam02577	DNase-RNase	Bifunctional	.	GO:0005634|	GO:0004518|nu	.
Manes.02G11110(	0.3217845	0.11658	down	no	Protein IQ-D	pfam00104	Hormone_recep	Ligand-bind	.	GO:0005634|	.	.
Manes.02G11130(	2.8702439	3.96E-32	up	yes	.	pfam13716	CRAL_TRIO_2	Divergent C	.	.	.	.
Manes.02G11140(	0.6183875	5.85E-05	down	no	UPF0548 pro	pfam09348	DUF1990	Domain of u	.	GO:0009507|	.	.
Manes.02G11190(	0.8011452	0.80406	down	no	.	pfam03087	DUF241	Arabidopsis	.	.	.	.
Manes.02G11200(	0.3654743	0.49836	down	no	.	pfam03087	DUF241	Arabidopsis	.	.	.	.
Manes.02G11230(	7.2651515	6.84E-09	up	yes	Probable acyl	pfam00501	AMP-binding	AMP-bindin	GO:0006631|	GO:0005777|	GO:0016874|lig	.
Manes.02G11240(	1.0267893	0.77409	up	no	Protein STRI	pfam03088	Str_synth	Strictosidine	GO:0009058|	GO:0005783|	.	.
Manes.02G11260(	0.6884946	0.00166	down	no	Serine acetyl	pfam06426	SATase_N	Serine acety	GO:0006535|	GO:0005829|	GO:0009001|se	K00640
Manes.02G11270(	0.4331891	2.53E-13	down	yes	Protein GRIF	pfam10174	Cast	RIM-binding	GO:0048193|	GO:0000795|	.	.
Manes.02G11280(	18.153735	1.60E-56	up	yes	Phosphoserin	pfam00266	Aminotran_5	Aminotransf	GO:0006564|	GO:0009507|	GO:0004648|O	K00831
Manes.02G11340(	1.1690647	0.15115	up	no	Phosphoinosi	pfam02383	Syja_N	SacI homol	GO:0009651|	GO:0005783|	GO:0052866|ph	.
Manes.02G11350(	2.8647145	5.53E-09	up	yes	Dirigent prot	pfam03018	Dirigent	Dirigent-lik	GO:1901599|	GO:0048046|	GO:0042349|gu	.
Manes.02G11390(	2.6633351	5.79E-06	up	yes	Protein RKD	pfam02042	RWP-RK	RWP-RK d	GO:0006355|	GO:0005634|	GO:0003677|D	.
Manes.02G11400(	1.255134	0.01581	up	no	Syntaxin-bin	pfam08596	Lgl_C	Lethal giant	GO:0006887|	GO:0005892|	GO:0017075|sy	K08518
Manes.02G11410(	0.5981083	0.0006	down	no	.	pfam14309	DUF4378	Domain of u	.	.	.	.
Manes.02G11420(	0.5968998	7.44E-06	down	no	WUSCHEL-	pfam00046	Homeobox	Homeobox	GO:0007275|	GO:0005634|	GO:0003677|D	.
Manes.02G11430(	0.7677214	0.02894	down	no	DNA mismat	pfam08676	MutL_C	MutL C terr	GO:0006298|	GO:0032300|	GO:0005524|A'	K08739

Manes.02G11460(	1.6850424	6.81E-09	up	no	Serine/threon	pfam00069	Pkinase	Protein kina	.	GO:0005634|	GO:0005524|A'	.
Manes.02G11470(	0.3594441	1.92E-05	down	yes	Uncharacteriz	pfam12763	EF-hand_4	Cytoskeleta	GO:0000147|a	GO:0030479|	GO:0005509|ca	.
Manes.02G11480(	3.7987336	6.53E-50	up	yes	Proton-coupl	pfam01490	Aa_trans	Transmemb	GO:0006865|	GO:0005783|	GO:0015187|gl	K14209
Manes.02G11570(	NA	NA	--	no	Monosacchar	pfam00083	Sugar_tr	Sugar (and	GO:0008643|	GO:0016021|	GO:0022891|su	.
Manes.02G11580(	0.3485841	1.76E-05	down	yes	Mitochondria	pfam00153	Mito_carr	Mitochondr	GO:0009651|	GO:0016021|	.	.
Manes.02G11600(	Inf	0.9009	up	no	.	pfam01440	Gemini_AL2	Geminivirus	.	.	.	.
Manes.02G11690(	1.188471	0.07835	up	no	3-hydroxy-3-	pfam00368	HMG-CoA_red	Hydroxyme	GO:0015936|	GO:0005789|	GO:0004420|hy	.
Manes.02G11750(	16.144169	0.0025	up	yes	Vacuolar iron	pfam01988	VIT1	VIT family.	GO:0006811|	GO:0016021|	.	.
Manes.02G11760(	1.2945324	0.01941	up	no	.	pfam13632	Glyco_trans_2_3	Glycosyl tra	.	.	.	.
Manes.02G11780(	2.8563602	8.01E-14	up	yes	Probable L-ty	pfam00139	Lectin_legB	Legume lect	GO:0009755|	GO:0016021|	GO:0005524|A'	.
Manes.02G11810(	0.5336135	7.34E-09	down	no	.	pfam05623	DUF789	Protein of u	.	.	.	.
Manes.02G11820(	0.7143248	0.00072	down	no	Testis-expres	pfam10296	DUF2404	Putative inte	.	GO:0016021|	.	.
Manes.02G11840(	0.4882375	0.01823	down	yes	PHD finger-li	pfam03660	PHF5	PHF5-like p	.	.	.	K12834
Manes.02G11860(	3.3171274	0.0009	up	yes	.	pfam02118	Srg	Srg family c	.	.	.	.
Manes.02G11880(	5.6415007	2.32E-80	up	yes	Zinc finger C	pfam12796	Ank_2	Ankyrin rep	GO:0006355|	.	GO:0003677|D	.
Manes.02G11930(	1.1274364	0.31138	up	no	Peptidyl-prol	pfam00160	Pro_isomerase	Cyclophilin	GO:0006457|	GO:0009507|	GO:0042277|pe	K01802
Manes.02G11990(	0.8151185	0.09842	down	no	Protein DAM	pfam00400	WD40	WD domain	GO:0006281|	GO:0080008|	GO:0003677|D	K10140
Manes.02G12000(	2.8020747	5.43E-28	up	yes	RING finger	pfam13639	zf-RING_2	Ring finger	GO:0051865|	.	GO:0003677|D	.
Manes.02G12010(	1.3971158	0.00022	up	no	Non-specific	pfam04185	Phosphoesterase	Phosphoeste	.	GO:0005576|	GO:0016788|hy	K01114
Manes.02G12020(	1.1654376	0.09405	up	no	Villin-1	pfam00626	Gelsolin	Gelsolin rep	GO:0051017|	GO:0005737|	.	.
Manes.02G12050(	9.0594634	4.76E-07	up	yes	.	pfam00686	CBM_20	Starch bindi	.	.	.	.
Manes.02G12060(	1.6956355	2.54E-05	up	no	Pleckstrin ho	pfam00169	PH	PH domain.	.	GO:0005737|	GO:0008289|lip	.
Manes.02G12070(	9.9907494	1.04E-26	up	yes	.	pfam13414	TPR_11	TPR repeat.	.	.	.	.
Manes.02G12080(	Inf	0.18198	up	no	Transcription	pfam13921	Myb_DNA-bind_6	Myb-like D	GO:0009553|	GO:0005634|	GO:0003677|D	K09422
Manes.02G12100(	0.7120338	0.0008	down	no	.	pfam11380	DUF3184	Protein of u	.	.	.	.
Manes.02G12110(	2.1375124	7.49E-14	up	yes	Sodium-depe	pfam07690	MFS_1	Major Facili	GO:0009416|	GO:0009535|	GO:0005315|in	.
Manes.02G12140(	3.3521415	1.60E-05	up	yes	.	pfam01212	Beta_elim_lyase	Beta-elimin	.	.	.	.
Manes.02G12150(	2.9241126	5.03E-12	up	yes	Josephin-like	pfam02099	Josephin	Josephin.	.	.	GO:0000166|nu	K15235
Manes.02G12160(	4.3154127	2.95E-26	up	yes	.	pfam04602	Arabinose_trans	Mycobacter	.	.	.	.
Manes.02G12170(	2.2243232	1.28E-08	up	yes	Phosphometh	pfam01964	ThiC	ThiC family	GO:0010266|	GO:0009507|	GO:0051539|4	K03147
Manes.02G12180(	1.055097	0.55471	up	no	ATP-depende	pfam00004	AAA	ATPase fam	.	GO:0009507|	GO:0005524|A'	K03798
Manes.02G12190(	0.5317043	0.05425	down	no	.	pfam09528	Ehrlichia_rpt	Ehrlichia tar	.	.	.	.
Manes.02G12220(	0.355695	5.74E-06	down	yes	Transcription	pfam00249	Myb_DNA-binding	Myb-like D	GO:0048262|	GO:0005634|	GO:0003677|D	.
Manes.02G12230(	1.6599603	0.0389	up	no	Laccase-4	pfam07732	Cu-oxidase_3	Multicoppe	GO:0009809|	GO:0048046|	GO:0005507|co	K05909
Manes.02G12240(	0.9126054	0.53798	down	no	Phosphatidyli	pfam08510	PIG-P	PIG-P. PIG-	GO:0006506|	GO:0016021|	GO:0017176|ph	K03861
Manes.02G12260(	1.4445763	5.59E-05	up	no	Zinc finger C	pfam00076	RRM_1	RNA recogn	.	.	GO:0003677|D	K12872
Manes.02G12270(	4.2102225	1.75E-05	up	yes	.	pfam03172	Sp100	Sp100 doma	.	.	.	.
Manes.02G12280(	0.6426497	2.02E-05	down	no	.	pfam04859	DUF641	Plant protei	.	.	.	.
Manes.02G12390(	0.3216552	5.52E-12	down	yes	.	pfam12906	RINGv	RING-varia	.	.	.	.
Manes.02G12400(	1.7324458	1.91E-09	up	no	Mitochondria	pfam02953	zf-Tim10_DDP	Tim10/DDP	GO:0045039|	GO:0005758|	GO:0046872|m	K17778
Manes.02G12440(	0.5493605	4.73E-05	down	no	.	pfam12043	DUF3527	Domain of u	.	.	.	.
Manes.02G12510(	0.4684514	1.77E-08	down	yes	UDP-glucose	pfam00201	UDPGT	UDP-glucor	GO:1900994|	.	GO:0035251|U	.
Manes.02G12540(	2.6839922	1.56E-16	up	yes	Copper trans	pfam04145	Ctr	Ctr copper t	GO:0006825|	GO:0016021|	GO:0005375|co	K14686
Manes.02G12590(	0.1961501	4.18E-14	down	yes	Glutathione S	pfam13417	GST_N_3	Glutathione	GO:0009751|	GO:0005737|	GO:0004364|gl	K00799
Manes.02G12610(	0.3975084	6.70E-07	down	yes	Probable glut	pfam00043	GST_C	Glutathione	.	.	GO:0004364|gl	K00799
Manes.02G12630(	7.4666964	0.0132	up	yes	Glutathione S	pfam13417	GST_N_3	Glutathione	GO:0009751|	GO:0005737|	GO:0004364|gl	K00799
Manes.02G12640(	0.2374387	7.71E-05	down	yes	.	pfam09765	WD-3	WD-repeat	.	.	.	.
Manes.02G12660(	0.4469995	1.96E-11	down	yes	.	pfam09334	tRNA-synt_1g	tRNA synth	.	.	.	.
Manes.02G12670(	0.9845739	0.91756	down	no	Pentatricope	pfam13041	PPR_2	PPR repeat	.	GO:0009507|	GO:0008270|zi	.
Manes.02G12730(	1.6318	2.43E-07	up	no	DNA-directe	pfam03876	SHS2_Rpb7-N	SHS2 doma	GO:0006351|	GO:0005634|	GO:0003899|D	K03015
Manes.02G12760(	1.2865772	0.00587	up	no	.	pfam01576	Myosin_tail_1	Myosin tail.	.	.	.	.
Manes.02G12790(	0.8847502	0.23938	down	no	Methylsterol	pfam04116	FA_hydroxylase	Fatty acid h	GO:0080065|	GO:0009941|	GO:0000254|C	K14424
Manes.02G12800(	1.1387154	0.69438	up	no	Probable prot	pfam00481	PP2C	Protein pho	GO:0006470|	.	GO:0046872|m	K14497
Manes.02G12820(	0.4381865	0.00736	down	yes	.	pfam07120	DUF1376	Protein of u	.	.	.	.
Manes.02G12950(	19.609582	1.93E-10	up	yes	.	pfam05109	Herpes_BLLF1	Herpes viru	.	.	.	.
Manes.02G12980(	5.1236094	8.45E-08	up	yes	Transcription	pfam00249	Myb_DNA-binding	Myb-like D	GO:0007623|	GO:0005634|	GO:0003677|D	.
Manes.02G13000(	6.4585575	6.76E-90	up	yes	E3 ubiquitin-	pfam13639	zf-RING_2	Ring finger	GO:0016567|	.	GO:0016874|lig	.
Manes.02G13020(	0.1334011	3.03E-08	down	yes	.	pfam08137	DVL	DVL family	.	.	.	.
Manes.02G13030(	0.7891072	0.47049	down	no	.	pfam13315	DUF4085	Protein of u	.	.	.	.
Manes.02G13060(	0.6273564	1	down	no	Uncharacteriz	pfam13419	HAD_2	Haloacid de	GO:0016311|	GO:0005737|	GO:0008252|nu	K18551
Manes.02G13080(	29.771743	1.76E-16	up	yes	Scarecrow-lil	pfam03514	GRAS	GRAS doma	GO:0006355|	GO:0005634|	.	.
Manes.02G13090(	14.599183	#######	up	yes	Scarecrow-lil	pfam03514	GRAS	GRAS doma	GO:0006355|	GO:0005634|	.	.
Manes.02G13110(	Inf	0.01269	up	yes	Scarecrow-lil	pfam03514	GRAS	GRAS doma	GO:0006355|	GO:0005634|	GO:0003700|tra	.
Manes.02G13130(	1.6496056	7.23E-08	up	no	Oxysterol-bir	pfam01237	Oxysterol_BP	Oxysterol-b	GO:0006869|	GO:0005829|	GO:0008289|lip	.
Manes.02G13150(	3.277978	1.20E-14	up	yes	.	pfam14255	Cys_rich_CPXG	Cysteine-ric	.	.	.	.
Manes.02G13190(	0.2159671	1.37E-34	down	yes	Expansin-A4	pfam01357	Pollen_allerg_1	Pollen aller	GO:0009664|	GO:0005829|	.	.
Manes.02G13220(	0.7573279	0.00717	down	no	Factor of DN	pfam03469	XH	XH domain.	GO:0090502|	GO:0005655|	GO:0008168|m	.
Manes.02G13250(	1.8540332	5.24E-12	up	no	Protein kinas	pfam00069	Pkinase	Protein kina	.	GO:0009507|	GO:0005524|A'	.
Manes.02G13260(	1.8797445	0.46795	up	no	.	pfam14111	DUF4283	Domain of u	.	.	.	.
Manes.02G13270(	0.5769048	0.2252	down	no	.	pfam15326	TEX15	Testis expre	.	.	.	.
Manes.02G13280(	1.29331	0.00484	up	no	Triosephosph	pfam00121	TIM	Triosephos	GO:0006094|	GO:0005737|	GO:0004807|tri	.
Manes.02G13350(	1.1916223	0.06966	up	no	COP9 signalo	pfam01399	PCI	PCI domain	GO:0010388|	GO:0008180|	.	K12176
Manes.02G13390(	1.9067519	1.04E-07	up	no	Transcription	pfam00249	Myb_DNA-binding	Myb-like D	GO:0030154|	GO:0005634|	GO:0003677|D	K09422
Manes.02G13410(	0.6280318	0.00107	down	no	Tyrosine-spe	pfam03222	Trp_Tyr_perm	Tryptophan	.	GO:0005887|	GO:0015173|ar	K03834
Manes.02G13430(	7.8447665	2.76E-15	up	yes	Probably ina	pfam00069	Pkinase	Protein kina	GO:0042742|	GO:0016021|	GO:0005524|A'	.
Manes.02G13460(	0.5731884	0.00012	down	no	Armadillo re	pfam00514	Arm	Armadillo/b	.	GO:0005737|	.	.
Manes.02G13500(	2.1940866	3.65E-09	up	yes	Probable ribo	pfam06026	Rib_5-P_isom_A	Ribose 5-ph	GO:0008219|	GO:0005737|	GO:0004751|ri	K01807
Manes.02G13510(	2.8510081	3.98E-28	up	yes	Nuclear cont	pfam08637	NCA2	ATP syntha	.	GO:0016021|	.	K18158
Manes.02G13550(	0.4691371	1.49E-06	down	yes	.	pfam01344	Kelch_1	Kelch motif.	.	.	.	.
Manes.02G13580(	NA	NA	--	no	Cytochrome	pfam00067	p450	Cytochrome	GO:0098542|	GO:0016021|	GO:0020037|he	K00517
Manes.02G13590(	0.4492945	0.02695	down	yes	Cytochrome	pfam00067	p450	Cytochrome	GO:0098542|	GO:0016021|	GO:0020037|he	K00517
Manes.02G13650(	1.1180875	0.27182	up	no	Dihydropyrin	pfam13594	Amidohydro_5	Amidohydr	GO:0006208|	GO:0005737|	GO:0004157|di	K01464
Manes.02G13670(	0.2921936	1.38E-05	down	yes	Heat stress tr	pfam00447	HSF_DNA-bind	HSF-type D	GO:0009408|	GO:0005634|	GO:0042802|id	K09419
Manes.02G13730(	0.7275091	0.04516	down	no	Protein N-lys	pfam10294	Methyltransf_16	Putative me	GO:0018022|	GO:0005737|	GO:0051117|A'	.
Manes.02G13750(	1.2976034	0.00524	up	no	26S proteaso	pfam13519	VWA_2	von Willebr	GO:0006974|	GO:0005829|	GO:0001653|pe	K03029
Manes.02G13790(	1.0547901	0.61074	up	no	Hydroxymeth	pfam00682	HMGL-like	HMGL-like.	.	GO:0005759|	GO:0004419|hy	K01640
Manes.02G13800(	0.5115178	0.01253	down	no	.	pfam00146	NADHdh	NADH dehy	.	.	.	.
Manes.02G13850(	6.9020635	0.00038	up	yes	Calcium-depe	pfam00069	Pkinase	Protein kina	GO:0009738|	GO:0005737|	GO:0005524|A'	K13412

Manes.02G13860(	0.862039	0.11793	down	no	Proteinaceou	pfam13041	PPR_2	PPR repeat	GO:0090501|	GO:0009507|	GO:0004526|ri	K18213
Manes.02G13910(	0.6923587	0.00021	down	no	.	pfam00068	Phospholip_A2_1	Phospholipa	.	.	.	.
Manes.02G13920(	2.3073202	2.77E-19	up	yes	Protein SMG	pfam10373	EST1_DNA_bind	Est1 DNA/I	GO:0006952|	GO:0000932|	.	K14409
Manes.02G13940(	0.7447106	0.01246	down	no	.	pfam14594	Sipho_Gp37	Siphovirus I	.	.	.	.
Manes.02G13980(	1.6375406	5.14E-08	up	no	.	pfam13639	zf-RING_2	Ring finger	.	.	.	.
Manes.02G13990(	17.129205	0.00264	up	yes	.	pfam14009	DUF4228	Domain of u	.	.	.	.
Manes.02G14030(	Inf	0.35135	up	no	.	pfam06330	TRI5	Trichodiene	.	.	.	.
Manes.02G14040(	0.7451809	0.00539	down	no	Signal peptid	pfam04258	Peptidase_A22B	Signal pepti	GO:0009555|	GO:0005783|	GO:0004190|as	K09595
Manes.02G14050(	0.4799098	4.34E-11	down	yes	.	pfam00046	Homeobox	Homeobox	.	.	.	.
Manes.02G14060(	0.201583	1.13E-07	down	yes	.	pfam02660	G3P_acyltransf	Glycerol-3-p	.	.	.	.
Manes.02G14070(	0.3547561	6.08E-22	down	yes	Ultraviolet-B	pfam08381	BRX	Transcriptio	GO:0009649|	GO:0000785|	GO:0003682|ch	.
Manes.02G14120(	0.7506196	0.40983	down	no	UPF0481 prc	pfam03140	DUF247	Plant protei	.	GO:0016021|	.	.
Manes.02G14140(	5.786055	2.37E-27	up	yes	.	pfam05687	DUF822	Plant protei	.	.	.	.
Manes.02G14150(	2.6483747	2.09E-26	up	yes	Splicing facto	pfam00076	RRM_1	RNA recogi	GO:0010467|	GO:0005654|	GO:0000166|nu	K12831
Manes.02G14160(	2.0063893	1.11E-09	up	yes	Serine--glyox	pfam00266	Aminotran_5	Aminotransf	GO:0009853|	GO:0048046|	GO:0008453|ala	K00830
Manes.02G14170(	0.9352116	0.54667	down	no	Protein CHR	pfam00176	SNF2_N	SNF2 family	GO:0016568|	GO:0005634|	GO:0005524|A	K11367
Manes.02G14220(	0.9685431	0.81023	down	no	Phytochromo	pfam05996	Fe_bilin_red	Ferredoxin-	GO:0010019|	GO:0009507|	GO:0050897|co	K08101
Manes.02G14250(	0.4123298	2.23E-11	down	yes	Scarecrow-lil	pfam03514	GRAS	GRAS doma	GO:0006355|	GO:0005634|	GO:0003700|tra	.
Manes.02G14270(	2.6410869	1.55E-17	up	yes	.	pfam00646	F-box	F-box doma	.	.	.	.
Manes.02G14280(	1.3328587	0.00375	up	no	Probable pect	pfam01095	Pectinesterase	Pectinestera	GO:0042545|	GO:0005618|	GO:0045330|as	.
Manes.02G14290(	0.3476361	2.62E-20	down	yes	Phosphatidyli	pfam00454	PI3_PI4_kinase	Phosphatidy	.	GO:0005777|	GO:0004430|1-	.
Manes.02G14300(	0.6947578	0.00377	down	no	Photosystem	pfam02531	PsaD	PsaD. This f	GO:0015979|	GO:0009535|	.	K02692
Manes.02G14310(	0.5193898	2.16E-09	down	no	Dipeptidyl pe	pfam00930	DPPIV_N	Dipeptidyl p	.	GO:0005737|	GO:0004177|an	K08655
Manes.02G14320(	1.5186708	7.48E-05	up	no	Calcineurin B	pfam13499	EF-hand_7	EF-hand dor	GO:0030007|	GO:0005955|	GO:0005509|ca	K06268
Manes.02G14350(	0.3947501	8.26E-13	down	yes	.	pfam02463	SMC_N	RecF/RecN/	.	.	.	.
Manes.02G14370(	1.1292414	0.17857	up	no	.	pfam12795	MscS_porin	Mechanoser	.	.	.	.
Manes.02G14410(	1.7609651	1.69E-10	up	no	ATP-depende	pfam07724	AAA_2	AAA domai	GO:0006457|	.	GO:0005524|A	K03544
Manes.02G14430(	0.4281212	1.36E-05	down	yes	.	pfam05097	DUF688	Protein of u	.	.	.	.
Manes.02G14450(	0.6728644	0.00135	down	no	Protein ECEI	pfam02458	Transferase	Transferase	GO:0071555|	GO:0005783|	GO:0016747|tra	.
Manes.02G14460(	0.6631025	0.00041	down	no	Telomeric rep	pfam00249	Myb_DNA-binding	Myb-like DI	GO:0051301|	GO:0000781|	GO:0008301|DI	.
Manes.02G14470(	2.9373945	1.78E-24	up	yes	.	pfam07765	KIP1	KIP1-like pr	.	.	.	.
Manes.02G14480(	1.2392379	0.33616	up	no	.	pfam00308	Bac_DnaA	Bacterial dn	.	.	.	.
Manes.02G14490(	0.9389802	0.52767	down	no	Uncharacteri	pfam10250	O-FucT	GDP-fucose	.	GO:0005768|	GO:0016757|tra	.
Manes.02G14500(	0.7019344	0.00027	down	no	Probable ubic	pfam02902	Peptidase_C48	Ulp1 protea	GO:0016926|	.	GO:0016929|SU	K08596
Manes.02G14520(	1.9997371	1.48E-11	up	no	DnaJ homolo	pfam00226	DnaJ	DnaJ domai	.	GO:0016021|	.	K09518
Manes.02G14560(	8.2271114	2.61E-65	up	yes	AT-hook mo	pfam03479	DUF296	Domain of u	GO:0006325|	GO:0005634|	GO:0003680|A	.
Manes.02G14570(	0.4758381	4.03E-13	down	yes	Ubiquitin carl	pfam00443	UCH	Ubiquitin ca	GO:0008283|	GO:0016021|	GO:0004197|cy	K11855
Manes.02G14600(	36.93899	#######	up	yes	Mitochondria	pfam00153	Mito_carr	Mitochondr	GO:0006835|	GO:0016021|	GO:0005310|di	K15104
Manes.02G14620(	0.8724677	0.24247	down	no	.	pfam09709	Cas_Csd1	CRISPR-ass	.	.	.	.
Manes.02G14650(	1.8135744	7.73E-10	up	no	Chaperone pr	pfam00226	DnaJ	DnaJ domai	GO:0006260|	GO:0005737|	GO:0005524|A	K03686
Manes.02G14700(	1.8701713	3.86E-12	up	no	WD and tetra	pfam00400	WD40	WD domain	GO:0055082|	GO:0005829|	GO:0004857|en	K11807
Manes.02G14710(	2.7621649	6.94E-19	up	yes	.	pfam03290	Peptidase_C57	Vaccinia vir	.	.	.	.
Manes.02G14720(	0.3853573	1.18E-09	down	yes	Serine/threon	pfam00069	Pkinase	Protein kina	.	.	GO:0005524|A	.
Manes.02G14730(	1.0979186	0.36868	up	no	Zinc-finger h	pfam04770	ZF-HD_dimer	ZF-HD prot	GO:0009793|	GO:0005634|	GO:0003677|DI	.
Manes.02G14740(	0.8648307	0.13158	down	no	Transport inh	pfam00646	F-box	F-box doma	GO:0009734|	GO:0005634|	GO:0010011|au	.
Manes.02G14750(	0.379284	1.37E-08	down	yes	Pyridoxine/py	pfam03853	YjeF_N	YjeF-relatec	GO:0006734|	GO:0009507|	GO:0010181|FN	K00275
Manes.02G14770(	0.6990913	0.01103	down	no	Bet1-like pro	pfam09778	Guanylate_cyc_2	Guanylylate	GO:0015031|	GO:0005789|	.	K08505
Manes.02G14780(	5.6837685	1.49E-10	up	yes	.	pfam04577	DUF563	Protein of u	.	.	.	.
Manes.02G14790(	0.7101643	0.00257	down	no	Serine/threon	pfam00069	Pkinase	Protein kina	GO:0009738|	GO:0005737|	GO:0005524|A	.
Manes.02G14850(	0.1942271	2.98E-05	down	yes	Transcription	pfam00010	HLH	Helix-loop-l	GO:0006351|	GO:0005634|	GO:0016597|an	.
Manes.02G14880(	1.7937619	1.71E-10	up	no	.	pfam06224	HTH_42	Winged heli	.	.	.	.
Manes.02G14900(	1.5704704	6.16E-05	up	no	Queuine tRN	pfam01702	TGT	Queuine tRI	GO:0008616|	.	GO:0046872|m	K00773
Manes.02G14910(	0.3135099	4.51E-07	down	yes	Homeobox-le	pfam00046	Homeobox	Homeobox	GO:0030308|	GO:0005634|	GO:0043565|se	K09338
Manes.02G14930(	13.466445	3.60E-11	up	yes	.	pfam06312	Neurexophilin	Neurexophil	.	.	.	.
Manes.02G14940(	1.7239714	1.05E-05	up	no	Uncharacteri	pfam10250	O-FucT	GDP-fucose	.	GO:0005768|	GO:0016757|tra	.
Manes.02G14990(	1.3633638	0.00186	up	no	.	pfam00244	2014/3/3	14-3-3 prote	.	.	.	.
Manes.02G15000(	Inf	0.1623	up	no	Probable leuc	pfam01246	Ribosomal_L24e	Ribosomal p	.	GO:0016021|	GO:0005524|A	.
Manes.02G15030(	Inf	0.9009	up	no	Putative pum	pfam00806	PUF	Pumilio-fam	GO:0006417|	GO:0009507|	GO:0003723|RI	.
Manes.02G15070(	1.0302204	0.74831	up	no	F-box protein	pfam12937	F-box-like	F-box-like.	GO:0010629|	GO:0005634|	.	.
Manes.02G15080(	6.9341777	2.63E-26	up	yes	Protein EXO	pfam04674	Phi_1	Phosphate-i	.	GO:0048046|	.	.
Manes.02G15090(	27.411202	1.10E-45	up	yes	Protein EXO	pfam04674	Phi_1	Phosphate-i	GO:0009741|	GO:0048046|	.	.
Manes.02G15100(	47.828875	0	up	yes	Protein EXO	pfam04674	Phi_1	Phosphate-i	GO:0009741|	GO:0048046|	.	.
Manes.02G15110(	48.230592	0	up	yes	Protein EXO	pfam04674	Phi_1	Phosphate-i	GO:0009741|	GO:0048046|	.	.
Manes.02G15120(	8.374079	6.99E-17	up	yes	Protein EXO	pfam04674	Phi_1	Phosphate-i	GO:0009741|	GO:0048046|	.	.
Manes.02G15140(	5.5227656	3.12E-66	up	yes	7-deoxylogar	pfam00201	UDPGT	UDP-glucor	.	.	GO:0035251|UI	.
Manes.02G15150(	Inf	0.00022	up	yes	Protein TRAI	pfam00096	zf-C2H2	Zinc finger,	GO:0009813|	GO:0005634|	GO:0046872|m	.
Manes.02G15170(	1.8277793	1.93E-11	up	no	Probable pect	pfam03141	Methyltransf_29	Putative S-a	GO:0007155|	GO:0005768|	GO:0008168|m	.
Manes.02G15180(	1.9271376	4.11E-13	up	no	5'-adenylylsul	pfam00085	Thioredoxin	Thioredoxin	GO:0045454|	GO:0005623|	GO:0003756|pr	.
Manes.02G15200(	0.5212027	6.43E-08	down	no	Uncharacteri	pfam12697	Abhydrolase_6	Alpha/beta l	GO:0009636|	.	GO:0052689|ca	.
Manes.02G15210(	1.0977553	0.44756	up	no	Probable prot	pfam00481	PP2C	Protein phos	GO:0006470|	GO:0005886|	GO:0046872|m	.
Manes.02G15220(	1.8464953	2.18E-09	up	no	Probable prot	pfam00481	PP2C	Protein phos	GO:0006470|	GO:0005634|	GO:0046872|m	.
Manes.02G15240(	3.2396099	1.52E-34	up	yes	Cryptochrom	pfam03441	FAD_binding_7	FAD binding	GO:0046283|	GO:0005737|	GO:0005524|A	K12118
Manes.02G15270(	4.8108471	9.46E-33	up	yes	.	pfam02466	Tim17	Tim17/Tim2	.	.	.	.
Manes.02G15280(	3.0016194	7.31E-28	up	yes	Probable mito	pfam00153	Mito_carr	Mitochondr	GO:0055085|	GO:0016021|	.	.
Manes.02G15290(	23.875972	#######	up	yes	.	pfam04988	AKAP95	A-kinase an	.	.	.	.
Manes.02G15300(	9.0690741	#######	up	yes	.	pfam03314	DUF273	Protein of u	.	.	.	.
Manes.02G15340(	1.6895383	0.00124	up	no	.	pfam05678	VQ	VQ motif. T	.	.	.	.
Manes.02G15380(	3.0813902	2.27E-11	up	yes	.	pfam04921	XAP5	XAP5, circa	.	.	.	.
Manes.02G15410(	0.1904206	0.00075	down	yes	Probable treh	pfam02358	Trehalose_PPase	Trehalose-p	GO:0016311|	GO:0009507|	GO:0015927|tre	K01087
Manes.02G15430(	0.9516124	0.62168	down	no	Endoplasmic	pfam07970	COPIIcoated_ERV	Endoplasmi	GO:0016192|	GO:0005789|	.	.
Manes.02G15440(	1.3034786	0.00344	up	no	Ethylene-resp	pfam00847	AP2	AP2 domair	GO:0071472|	GO:0005634|	GO:0003677|DI	K09286
Manes.02G15460(	1.1598438	0.46904	up	no	Uncharacteri	pfam00400	WD40	WD domain	.	GO:0005829|	.	.
Manes.02G15480(	0.6003658	0.00552	down	no	Metallophosp	pfam02350	Epimerase_2	UDP-N-ace	GO:0006888|	GO:0005801|	GO:0034235|GI	.
Manes.02G15500(	1.6927692	6.61E-09	up	no	Protein DOW	pfam01190	Pollen_Ole_e_I	Pollen prote	.	GO:0005615|	.	.
Manes.02G15530(	0.7736513	0.01418	down	no	Acetyl-CoA	pfam08326	ACC_central	Acetyl-CoA	GO:0009793|	GO:0005829|	GO:0003989|ac	K11262
Manes.02G15540(	1.7041615	0.00043	up	no	Cysteine-rich	pfam07714	Pkinase_Tyr	Protein tyro	GO:0009755|	GO:0016021|	GO:0005524|A	.
Manes.02G15560(	2.3321605	4.92E-14	up	yes	Cysteine-rich	pfam07714	Pkinase_Tyr	Protein tyro	GO:0009755|	GO:0016021|	GO:0005524|A	.
Manes.02G15590(	0.7245094	0.58202	down	no	Cysteine-rich	pfam01657	Stress-antifung	Salt stress r	GO:0009755|	GO:0016021|	GO:0005524|A	.

Manes.02G15610(	0.5373623	0.0001	down	no	Low affinity s	pfam00916	Sulfate_transp	Sulfate trans	.	GO:0016021|	GO:0008271|se	.
Manes.02G15640(	Inf	0.01404	up	yes	Agamous-like	pfam00319	SRF-TF	SRF-type tr	GO:0071365|c	GO:0005737|	GO:0003677|DI	.
Manes.02G15660(	5.2359135	0.00509	up	yes	.	pfam11250	DUF3049	Protein of u	.	.	.	.
Manes.02G15680(	1.6591557	0.00034	up	no	.	pfam01344	Kelch_1	Kelch motif.	.	.	.	.
Manes.02G15690(	2.331648	4.20E-08	up	yes	Membrane-an	pfam13881	Rad60-SLD_2	Ubiquitin-2	.	GO:0005886|	.	.
Manes.02G15700(	0.8530386	0.12889	down	no	.	pfam10358	NT-C2	N-terminal (	.	.	.	.
Manes.02G15720(	35.8223	3.85E-05	up	yes	.	pfam04523	Herpes_U30	Herpes virus	.	.	.	.
Manes.02G15730(	0.3607035	4.13E-12	down	yes	Probable glyc	pfam01553	Acyltransferase	Acyltransfer	GO:0016024|(	GO:0016021|	GO:0004366|gl	K13508
Manes.02G15750(	1.4416837	5.78E-05	up	no	Uncharacteriz	pfam09728	Taxilin	Myosin-like	.	GO:0009507|	.	.
Manes.02G15760(	Inf	0.00126	up	yes	.	pfam04099	Sybindin	Sybindin-lik	.	.	.	.
Manes.02G15770(	0.8340989	0.09431	down	no	Phosphopante	pfam04127	DFP	DNA / pante	GO:0015937|c	.	GO:0004632|ph	K01922
Manes.02G15780(	0.5352445	0.00203	down	no	.	pfam05633	DUF793	Protein of u	.	.	.	.
Manes.02G15820(	0.5526372	4.01E-08	down	no	Eukaryotic tr	pfam01873	eIF-5_eIF-2B	Domain fou	.	.	GO:0005525|G	.
Manes.02G15830(	0.5167245	2.23E-05	down	no	Adrenodoxin	pfam00111	Fer2	2Fe-2S iron	GO:0055114|c	GO:0005739|	GO:0051537|2	.
Manes.02G15840(	4.7729	5.88E-40	up	yes	Beta-1,3-gala	pfam01762	Galactosyl_T	Galactosyltr	GO:0006486|p	GO:0005794|	GO:0048531|be	.
Manes.02G15860(	0.6334215	0.04653	down	no	.	pfam08082	PRO8NT	PRO8NT (N	.	.	.	.
Manes.02G15870(	1.5950706	0.00061	up	no	Protein Jade-	pfam13832	zf-HC5HC2H_2	PHD-zinc-fi	GO:0006915|a	GO:0005737|	GO:0008270|zi	K11347
Manes.02G15890(	0.9901078	0.94357	down	no	Mitogen-activ	pfam00069	Pkinase	Protein kina	GO:0000186|a	GO:0005634|	GO:0005524|A	K13414
Manes.02G15900(	0.726228	0.00434	down	no	.	pfam07800	DUF1644	Protein of u	.	.	.	.
Manes.02G15930(	0.3769667	0.01652	down	yes	.	pfam01692	Paramyxo_C	Paramyxovi	.	.	.	.
Manes.02G15940(	0.4880549	2.74E-05	down	yes	Small ribosor	pfam00416	Ribosomal_S13	Ribosomal p	GO:0006412|t	GO:0005763|	GO:0019843|rR	K02952
Manes.02G15970(	1.5121709	0.00053	up	no	Phosphatidyli	pfam01504	PIP5K	Phosphatidy	GO:0046854|p	GO:0005886|	GO:0016308|1-	K00889
Manes.02G15980(	1.9971562	1.76E-14	up	no	Oxysterol-bin	pfam01237	Oxysterol_BP	Oxysterol-b	GO:0006869|l	.	GO:0008289|lip	.
Manes.02G15990(	2.0402648	6.22E-14	up	yes	Biotin--prote	pfam03099	BPL_LplA_LipB	Biotin/lipoa	GO:0042966|l	GO:0009507|	GO:0005524|A	.
Manes.02G16000(	0.3701716	5.89E-06	down	yes	Probable seri	pfam00069	Pkinase	Protein kina	GO:0051304|c	GO:0005813|	GO:0005524|A	K08866
Manes.02G16010(	1.2387125	0.03286	up	no	.	pfam03552	Cellulose_synt	Cellulose sy	.	.	.	.
Manes.02G16040(	2.2195783	0.00012	up	yes	Auxin transpo	pfam01490	Aa_trans	Transmembr	GO:0006084|a	GO:0016021|	GO:0010328|au	K13946
Manes.02G16050(	3.9400551	0.00517	up	yes	.	pfam09484	Cas_TM1802	CRISPR-ass	.	.	.	.
Manes.02G16070(	0.4858882	4.28E-09	down	yes	Flavonol synt	pfam03171	2OG-FeII_Oxy	2OG-Fe(II)	.	GO:0005737|	GO:0045431|fla	.
Manes.02G16080(	0.8960156	0.43864	down	no	.	pfam10961	DUF2763	Protein of u	.	.	.	.
Manes.02G16100(	0.3331424	8.49E-06	down	yes	.	pfam03327	Herpes_VP19C	Herpesvirus	.	.	.	.
Manes.02G16110(	5.7018754	7.97E-05	up	yes	Nodulation-s	pfam03514	GRAS	GRAS doma	GO:0009877|r	GO:0005783|	.	.
Manes.02G16120(	0.7789464	0.01097	down	no	Probable N-s	pfam00155	Aminotran_1_2	Aminotransf	GO:0009089|l	GO:0005618|	GO:0030170|py	K14267
Manes.02G16170(	7.5536259	4.18E-13	up	yes	Ethylene-resp	pfam00847	AP2	AP2 domair	GO:0071497|c	GO:0005634|	GO:0003700|tra	.
Manes.02G16200(	0.7873973	0.13715	down	no	WAT1-relate	pfam00892	EamA	EamA-like t	.	GO:0016021|	GO:0022857|tra	.
Manes.02G16210(	2.0522868	4.03E-16	up	yes	.	pfam00999	Na_H_Exchanger	Sodium/hyd	.	.	.	.
Manes.02G16240(	0.584999	3.52E-07	down	no	ATPase famil	pfam00004	AAA	ATPase fam	GO:0006974|c	GO:0005634|	GO:0005524|A	.
Manes.02G16260(	0.7887765	0.01966	down	no	UDP-galacto	pfam03151	TPT	Triose-phos	GO:0008643|c	GO:0005794|	.	.
Manes.02G16280(	0.8728495	0.5259	down	no	Long chain ac	pfam00501	AMP-binding	AMP-bindin	GO:0006631|f	GO:0009507|	GO:0005524|A	K01897
Manes.02G16290(	0.8950734	0.40942	down	no	.	pfam05340	DUF740	Protein of u	.	.	.	.
Manes.02G16300(	21.705442	1.19E-09	up	yes	.	pfam02442	L1R_F9L	Lipid memb	.	.	.	.
Manes.02G16310(	0.3632373	4.18E-05	down	yes	Esterase	pfam00657	Lipase_GDSL	GDSL-like l	GO:0006952|c	.	GO:0016788|hy	.
Manes.02G16330(	1.0821108	0.50538	up	no	Esterase	pfam00657	Lipase_GDSL	GDSL-like l	GO:0006952|c	.	GO:0016788|hy	.
Manes.02G16340(	0.7997378	0.2559	down	no	Uncharacteriz	pfam12950	TaqI_C	TaqI-like C-	.	GO:0009507|	.	.
Manes.02G16360(	0.1819077	1.60E-13	down	yes	.	pfam13251	DUF4042	Domain of u	.	.	.	.
Manes.02G16390(	1.3545943	0.10264	up	no	Defensin-like	pfam00304	Gamma-thionin	Gamma-thic	GO:0050832|c	GO:0005576|	.	.
Manes.02G16410(	1.1149253	0.33483	up	no	BTB/POZ do	pfam00651	BTB	BTB/POZ d	GO:0016567|p	GO:0031463|	.	K10523
Manes.02G16420(	0.6571564	0.00689	down	no	V-type proto	pfam00137	ATP-synt_C	ATP syntha	GO:0015991|a	GO:0016021|	GO:0015078|hy	.
Manes.02G16430(	3.4368019	4.74E-34	up	yes	Acetyltransfe	pfam14542	Acetyltransf_CG	GCN5-relat	GO:0016573|h	GO:0005777|	GO:0004402|his	.
Manes.02G16440(	1.3566318	0.00116	up	no	F-box protein	pfam12937	F-box-like	F-box-like.	GO:0016567|p	GO:0005739|	.	K10295
Manes.02G16460(	0.7707349	0.00807	down	no	Delta-aminole	pfam00490	ALAD	Delta-amino	GO:0015995|c	GO:0009507|	GO:0004655|po	K01698
Manes.02G16500(	0.4872279	1.83E-10	down	yes	.	pfam04782	DUF632	Protein of u	.	.	.	.
Manes.02G16520(	1.7534881	0.00154	up	no	Aldehyde del	pfam00171	Aldedh	Aldehyde de	GO:0006081|c	GO:0005783|	GO:0004028|3-	K00128
Manes.02G16550(	0.5640986	1.75E-05	down	no	Guanine nucl	pfam08192	Peptidase_S64	Peptidase fa	GO:0010541|a	GO:0005737|	GO:0005525|G	.
Manes.02G16560(	0.8601609	0.22507	down	no	Ribosomal R	pfam01795	Methyltransf_5	MraW meth	GO:0070475|r	GO:0005737|	GO:0071424|rR	K03438
Manes.02G16570(	0.4765171	5.42E-09	down	yes	.	pfam09366	DUF1997	Protein of u	.	.	.	.
Manes.02G16590(	0.8071134	0.0255	down	no	U-box domai	pfam00168	C2	C2 domain.	.	.	GO:0016874|lig	.
Manes.02G16600(	10.874468	5.48E-61	up	yes	NAC domain	pfam02365	NAM	No apical m	GO:0009788|r	GO:0005634|	GO:0003677|DI	.
Manes.02G16660(	0.3144251	9.63E-05	down	yes	.	pfam05097	DUF688	Protein of u	.	.	.	.
Manes.02G16690(	0.7986031	0.14758	down	no	Chloroplast p	pfam00717	Peptidase_S24	Peptidase S	GO:0051604|p	GO:0009507|	GO:0008233|pe	K03100
Manes.02G16710(	4.7179922	6.98E-64	up	yes	Amino acid p	pfam01490	Aa_trans	Transmembr	GO:0003333|a	GO:0016021|	GO:0015171|an	.
Manes.02G16730(	8.97083	3.55E-29	up	yes	Probable rece	pfam00069	Pkinase	Protein kina	.	GO:0016021|	GO:0005524|A	.
Manes.02G16820(	0.6874019	0.00066	down	no	DNA topoiso	pfam12738	PTCB-BRCT	twin BRCT	GO:0006974|c	GO:0000785|	GO:0003682|ch	K10728
Manes.02G16840(	1.2293244	0.02072	up	no	.	pfam07879	PHB_acc_N	PHB/PHA a	.	.	.	.
Manes.02G16860(	0.9464871	0.59715	down	no	Histone-lysin	pfam00856	SET	SET domair	GO:0048653|a	GO:0000775|	GO:0042800|his	.
Manes.02G16880(	1.3579526	0.06083	up	no	Probable rece	pfam00069	Pkinase	Protein kina	.	.	GO:0005524|A	.
Manes.02G16900(	0.6629264	7.93E-05	down	no	NAD(P)H-qu	pfam10664	NdhM	Cyanobacter	GO:0006810|t	GO:0009535|	GO:0016655|ox	.
Manes.02G16930(	0.236566	2.54E-08	down	yes	UDP-sugar p	pfam09229	Aha1_N	Activator of	GO:0009226|r	GO:0005829|	GO:0047350|gl	K12447
Manes.02G16940(	0.8907277	0.9467	down	no	Probable calc	pfam13499	EF-hand_7	EF-hand do	.	.	GO:0005509|ca	K13448
Manes.02G16950(	1.2084247	0.41337	up	no	Probable met	pfam03141	Methyltransf_29	Putative S-a	.	GO:0000139|	GO:0008168|m	.
Manes.02G16970(	34.128868	0.00368	up	yes	Bidirectional	pfam03083	MtN3_slv	Sugar efflux	GO:0009901|a	GO:0005887|	GO:0051119|su	.
Manes.02G17130(	1.9067576	1.91E-11	up	no	Heat shock c	pfam00012	HSP70	Hsp70 prote	.	.	GO:0005524|A	.
Manes.02G17240(	1.595303	8.64E-07	up	no	2-oxoisovaler	pfam00676	E1_dh	Dehydrogen	GO:0043617|c	GO:0005759|	GO:0003863|3-	K00166
Manes.02G17250(	10.524516	1.70E-35	up	yes	Proline-rich r	pfam00069	Pkinase	Protein kina	GO:0009755|h	GO:0016021|	GO:0005524|A	.
Manes.02G17260(	0.7097826	0.00123	down	no	F-box protein	pfam12937	F-box-like	F-box-like.	GO:0009734|a	GO:0005634|	GO:0004842|ub	K03875
Manes.02G17340(	0.9829519	0.90913	down	no	Ribosome ma	pfam09377	SBDS_C	SBDS prote	GO:0048539|r	GO:0005737|	GO:0008017|mi	K14574
Manes.02G17370(	0.793896	0.02837	down	no	Transcription	pfam01803	LIM_bind	LIM-domain	GO:0030154|c	GO:0005654|	GO:0060090|bi	.
Manes.02G17380(	0.5849006	0.00024	down	no	.	pfam14750	INTS2	Integrator c	.	.	.	.
Manes.02G17410(	3.2564994	2.45E-10	up	yes	Bidirectional	pfam03083	MtN3_slv	Sugar efflux	GO:0034219|c	GO:0005783|	GO:0051119|su	K15382
Manes.02G17480(	1.631219	0.09958	up	no	Alcohol dehy	pfam08240	ADH_N	Alcohol deh	.	GO:0005737|	GO:0004022|alc	.
Manes.02G17510(	0.3128364	8.42E-15	down	yes	Protein tricho	pfam13839	PC-Esterase	GDSL/SGN	.	GO:0016021|	.	.
Manes.02G17550(	2.0403553	2.49E-08	up	yes	.	pfam09791	Oxidored-like	Oxidoreduct	.	.	.	.
Manes.02G17570(	6.9286052	0.023	up	yes	.	pfam04720	DUF506	Protein of u	.	.	.	.
Manes.02G17590(	0.3364168	4.89E-16	down	yes	Uncharacteriz	pfam02698	DUF218	DUF218 do	.	GO:0005737|	.	.
Manes.02G17600(	0.7845565	0.6503	down	no	.	pfam01139	RtcB	tRNA-splici	.	.	.	.
Manes.02G17610(	0.5490575	0.0013	down	no	Pleckstrin ho	pfam08718	GLTP	Glycolipid t	GO:0015031|p	GO:0005794|	GO:0051861|gl	K08051
Manes.02G17620(	2.0939006	7.78E-17	up	yes	.	pfam04658	TAFII55_N	TAFII55 pro	.	.	.	.
Manes.02G17690(	7.0927489	4.30E-12	up	yes	U-box domai	pfam04564	U-box	U-box doma	GO:0016567|p	GO:0009506|	GO:0016874|lig	.

Manes.02G17700(	6.9572073	0.20931	up	no	Protein IDA-	pfam13732	DUF4162	Domain of u	GO:0010227|r	GO:0005615|	.	.
Manes.02G17740(	40.271125	#######	up	yes	G-type lectin	pfam00069	Pkinase	Protein kina	.	GO:0016021|	GO:0005524|A'	.
Manes.02G17800(	25.250245	9.11E-14	up	yes	Abscisic acid	pfam00067	p450	Cytochrome	GO:0046345|a	GO:0016021|	GO:0010295|(+	K09843
Manes.02G17820(	0.9979313	0.9868	down	no	Probable GTl	pfam04607	RelA_SpoT	Region four	GO:0015969|g	GO:0009507|	GO:0005524|A'	.
Manes.02G17830(	1.585878	4.76E-06	up	no	50S ribosoma	pfam00297	Ribosomal_L3	Ribosomal p	GO:0006412|t	GO:0009507|	GO:0019843|rR	K02906
Manes.02G17880(	1.6510755	1.84E-08	up	no	Exocyst com	pfam15469	Sec5	Exocyst con	GO:0006887|e	GO:0005829|	.	K17637
Manes.02G17900(	2.2535043	5.55E-16	up	yes	.	pfam13837	Myb_DNA-bind_4	Myb/SANT-	.	.	.	.
Manes.02G17910(	0.5044258	1.93E-10	down	no	Trihelix trans	pfam13837	Myb_DNA-bind_4	Myb/SANT-	GO:0006351|t	GO:0005634|	GO:0003677|D	.
Manes.02G17920(	0.6037417	2.73E-05	down	no	Trihelix trans	pfam13837	Myb_DNA-bind_4	Myb/SANT-	GO:0006351|t	GO:0005634|	GO:0003677|D	.
Manes.02G17930(	13.101668	4.55E-34	up	yes	WAT1-relate	pfam00892	EamA	EamA-like t	.	GO:0016021|	GO:0022857|tra	.
Manes.02G17940(	0.9748039	0.83246	down	no	.	pfam01167	Tub	Tub family.	.	.	.	.
Manes.02G18000(	0.5909449	5.25E-05	down	no	.	pfam04441	Pox_VERT_large	Poxvirus ear	.	.	.	.
Manes.02G18020(	0.422966	7.22E-09	down	yes	G-box-bindin	pfam00170	bZIP_1	bZIP transc	GO:0006351|t	GO:0005634|	GO:0003677|D	K14432
Manes.02G18040(	0.9709535	0.81088	down	no	.	pfam14360	PAP2_C	PAP2 super	.	.	.	.
Manes.02G18080(	0.8860042	0.22037	down	no	Abscisic stre	pfam02496	ABA_WDS	ABA/WDS	GO:0006950|r	GO:0005634|	.	.
Manes.02G18100(	3.8317745	6.72E-47	up	yes	.	pfam02496	ABA_WDS	ABA/WDS	.	.	.	.
Manes.02G18180(	1.5653895	0.00196	up	no	U3 small nuc	pfam09384	UTP15_C	UTP15 C te	GO:0000462|r	GO:0030054|	GO:0044822|po	K14549
Manes.02G18200(	42.966978	3.90E-11	up	yes	Ethylene-resp	pfam14935	TMEM138	Transmemb	GO:0009873|e	GO:0005634|	GO:0003677|D	.
Manes.02G18210(	NA	NA	--	no	.	pfam04043	PMEI	Plant inverta	.	.	.	.
Manes.02G18230(	8.9480991	0.00466	up	yes	.	pfam04043	PMEI	Plant inverta	.	.	.	.
Manes.02G18250(	10.042287	3.82E-43	up	yes	Peroxidase 1	pfam00141	peroxidase	Peroxidase.	GO:0042744|l	GO:0005618|	GO:0020037|he	K00430
Manes.02G18280(	1.6066697	1.14E-07	up	no	.	pfam00504	Chloroa_b-bind	Chlorophyll	.	.	.	.
Manes.02G18290(	0.4076059	7.90E-14	down	yes	.	pfam08064	UME	UME (NUC	.	.	.	.
Manes.02G18320(	3.9119613	8.71E-07	up	yes	UDP-galacto	pfam03151	TPT	Triose-phos	GO:0015783|	GO:0005794|	GO:0015297|an	.
Manes.02G18340(	0.7150604	0.00867	down	no	Carboxypepti	pfam00246	Peptidase_M14	Zinc carbox	GO:0006508|p	GO:0016021|	GO:0004181|m	.
Manes.02G18350(	0.4003578	0.03482	down	no	Tryptophan a	pfam04864	Alliinase_C	Allinase. All	.	GO:0016021|	GO:0016846|ca	.
Manes.02G18380(	4.6254015	2.80E-52	up	yes	Probable cho	pfam01633	Choline_kinase	Choline/etha	GO:0006657|	.	GO:0005524|A'	K14156
Manes.02G18390(	0.8843234	0.27595	down	no	Type I inosit	pfam03372	Exo_endo_phos	Endonuclea	GO:0009738|a	.	GO:0046030|in	.
Manes.02G18440(	1.3102568	0.00283	up	no	DEAD-box A	pfam00270	DEAD	DEAD/DEA	GO:0080158|	GO:0009507|	GO:0005524|A'	.
Manes.02G18520(	2.0698312	2.87E-09	up	yes	Polyadenylat	pfam00658	PABP	Poly-adenyl	GO:0006417|r	GO:0005737|	GO:0000166|nu	K13126
Manes.02G18560(	0.9501539	0.70631	down	no	.	pfam15327	Tankyrase_bdg_C	Tankyrase b	.	.	.	.
Manes.02G18600(	0.848654	0.26271	down	no	tRNA pseud	pfam01416	PseudoU_synth_1	tRNA pseu	GO:0031119|	GO:0005634|	GO:0009982|ps	K01855
Manes.02G18640(	2.0073438	2.62E-13	up	yes	Sphinganine	pfam04116	FA_hydroxylase	Fatty acid h	GO:0006633|	GO:0005783|	GO:0005506|irc	K04713
Manes.02G18680(	NA	NA	--	no	17.9 kDa clas	pfam00011	HSP20	Hsp20/alpha	GO:0009408|	GO:0005634|	.	K13993
Manes.02G18700(	8.0873049	1.79E-39	up	yes	Cytochrome	pfam00067	p450	Cytochrome	GO:0007275|	GO:0016021|	GO:0020037|he	.
Manes.02G18710(	Inf	0.35135	up	no	Non-specific	pfam00234	Tryp_alpha_amyl	Protease inh	GO:0006869|l	.	GO:0008289|lip	.
Manes.02G18750(	1.4197422	0.00111	up	no	Somatic emb	pfam00069	Pkinase	Protein kina	GO:0009742|l	GO:0016021|	GO:0005524|A'	K13418
Manes.02G18760(	2.8873478	3.51E-16	up	yes	.	pfam03398	Ist1	Regulator o	.	.	.	.
Manes.02G18800(	1.9828207	4.27E-10	up	no	Angio-associ	pfam00400	WD40	WD domain	GO:0001525|a	GO:0009986|	.	.
Manes.02G18830(	0.5233481	1.24E-07	down	no	Transcription	pfam02536	mTERF	mTERF. Th	GO:0045892|r	GO:0005739|	GO:0003690|dc	K15032
Manes.02G18840(	0.8709095	0.1416	down	no	Putative 1-ph	pfam01504	PIP5K	Phosphatidy	.	.	GO:0000285|1-	K00921
Manes.02G18890(	1.5985417	6.02E-06	up	no	.	pfam12409	P5-ATPase	P5-type AT	.	.	.	.
Manes.02G18920(	6.5667845	1.95E-30	up	yes	GDSL estera	pfam00657	Lipase_GDSL	GDSL-like l	GO:0016042|l	GO:0048046|	GO:0016788|hy	.
Manes.02G18950(	8.635694	0.00069	up	yes	.	pfam12520	DUF3723	Protein of u	.	.	.	.
Manes.02G18960(	25.496418	#######	up	yes	Probable WR	pfam03106	WRKY	WRKY DN	GO:0010120|	GO:0005634|	GO:0043565|se	K13424
Manes.02G18980(	0.2764285	0.00068	down	yes	MATE efflux	pfam01554	MatE	MatE. The l	.	GO:0016021|	GO:0015297|an	K03327
Manes.02G19000(	0.6663558	0.00776	down	no	Protein-tyros	pfam00102	Y_phosphatase	Protein-tyro	GO:0006952|	GO:0005737|	GO:0033550|M	.
Manes.02G19030(	3.2606392	5.23E-14	up	yes	Sucrose trans	pfam00083	Sugar_tr	Sugar (and	GO:0005985|	GO:0005887|	GO:0008506|su	K15378
Manes.02G19040(	2.5223164	0.15281	up	no	.	pfam02770	Acyl-CoA_dh_M	Acyl-CoA d	.	.	.	.
Manes.02G19070(	1.0313841	0.76658	up	no	Protein SENS	pfam00096	zf-C2H2	Zinc finger,	GO:0006355|r	GO:0005634|	GO:0046872|m	.
Manes.02G19100(	0.5683888	0.01641	down	no	Probable mag	pfam05653	Mg_trans_NIPA	Magnesium	GO:0015693|r	GO:0005769|	GO:0015095|m	.
Manes.02G19120(	1.6096082	3.46E-07	up	no	Histidinol-ph	pfam00155	Aminotran_1_2	Aminotransl	GO:0000105|l	GO:0009507|	GO:0004400|his	.
Manes.02G19130(	9.1769313	0.05214	up	no	.	pfam03568	Peptidase_C50	Peptidase fa	.	.	.	.
Manes.02G19140(	0.8217095	0.15357	down	no	.	pfam04765	DUF616	Protein of u	.	.	.	.
Manes.02G19150(	0.8290851	0.11051	down	no	.	pfam02536	mTERF	mTERF. Th	.	.	.	.
Manes.02G19180(	1.3229459	0.00187	up	no	Chaperone p	pfam12572	DUF3752	Protein of u	GO:0006260|l	GO:0005737|	GO:0005524|A'	K03686
Manes.02G19220(	0.3398846	1.43E-15	down	yes	Calcium-dep	pfam00069	Pkinase	Protein kina	GO:0009738|a	GO:0005737|	GO:0005524|A'	K13412
Manes.02G19230(	1.3602544	0.00645	up	no	Subtilisin-lik	pfam05922	Inhibitor_I9	Peptidase in	GO:0009682|i	GO:0031012|	GO:0004252|se	.
Manes.02G19270(	0.7239024	0.00097	down	no	Protein disul	pfam00085	Thioredoxin	Thioredoxin	GO:0045454|	GO:0005783|	GO:0003756|pr	K09580
Manes.02G19280(	Inf	0.9009	up	no	Probable terp	pfam03936	Terpene_synth_C	Terpene syn	.	.	GO:0000287|m	.
Manes.02G19290(	0.6573011	0.00157	down	no	Alpha-(1,4)-f	pfam00852	Glyco_transf_10	Glycosyltran	GO:0071555|	GO:0032580|	GO:0008417|fu	K14412
Manes.02G19420(	0.4092717	4.35E-05	down	yes	Transcription	pfam03634	TCP	TCP family	GO:0048510|r	GO:0005634|	GO:0003677|D	.
Manes.02G19430(	0.4176902	0.07726	down	no	.	pfam03348	Serinc	Serine incor	.	.	.	.
Manes.02G19560(	0.1358436	1.47E-11	down	yes	Glucuronoxy	pfam04669	Polysacc_synt_4	Polysacchar	GO:0009808|l	GO:0005794|	GO:0030775|gl	K18801
Manes.02G19570(	NA	NA	--	no	.	pfam13456	RVT_3	Reverse trar	.	.	.	.
Manes.02G19600(	0.1498766	0.00018	down	yes	.	pfam15097	Ig_J_chain	Immunoglol	.	.	.	.
Manes.02G19610(	2.0836443	4.58E-07	up	yes	GDSL estera	pfam00657	Lipase_GDSL	GDSL-like l	GO:0016042|l	GO:0005576|	GO:0016788|hy	.
Manes.02G19620(	1.0759474	0.42308	up	no	.	pfam00622	SPRY	SPRY doma	.	.	.	.
Manes.02G19660(	1.2296965	0.0219	up	no	Phosphoinosi	pfam02383	Syja_N	SacI homol	GO:0007010|	GO:0005794|	GO:0043813|ph	.
Manes.02G19690(	0.6938786	0.00021	down	no	Multiple C2 a	pfam08372	PRT_C	Plant phospl	GO:0019722|	GO:0016021|	GO:0005509|ca	.
Manes.02G19700(	2.502935	5.36E-20	up	yes	.	pfam03231	Bunya_NS-S_2	Bunyavirus	.	.	.	.
Manes.02G19710(	1.1840867	1	up	no	Transmembra	pfam13664	DUF4149	Domain of u	.	GO:0070062|	.	.
Manes.02G19770(	0.2678539	9.87E-08	down	yes	Transcription	pfam00010	HLH	Helix-loop-l	GO:0048446|p	GO:0005634|	GO:0003677|D	.
Manes.02G19780(	0.2249353	8.49E-13	down	yes	.	pfam10322	7TM_GPCR_Sru	Serpentine t	.	.	.	.
Manes.02G19790(	1.8615664	1.26E-11	up	no	Transcription	pfam00010	HLH	Helix-loop-l	GO:0006355|r	GO:0005634|	GO:0003677|D	.
Manes.02G19800(	0.3634893	0.0043	down	yes	Protein NRT	pfam06691	DUF1189	Protein of u	GO:0006857|	GO:0016021|	GO:0005215|tra	.
Manes.02G19830(	3.3297758	1.50E-12	up	yes	Patellin-3	pfam00650	CRAL_TRIO	CRAL/TRIC	GO:0007049|	GO:0005737|	GO:0008289|lip	.
Manes.02G19860(	3.2268017	1.18E-06	up	yes	Carboxymeth	pfam01738	DLH	Dienelacton	.	GO:0005829|	GO:0016787|hy	K01061
Manes.02G19870(	7.9534657	1.23E-10	up	yes	.	pfam03916	NrfD	Polysulphide	.	.	.	.
Manes.02G19900(	0.7559062	0.2793	down	no	.	pfam00385	Chromo	Chromo (CI	.	.	.	.
Manes.02G19910(	0.8038185	0.13667	down	no	DNA mismat	pfam01119	DNA_mis_repair	DNA misma	GO:0010154|	GO:0005712|	GO:0005524|A'	K08734
Manes.02G19920(	0.6530781	1.55E-05	down	no	T-complex p	pfam05794	Tcp11	T-complex p	GO:0030154|	GO:0016021|	.	.
Manes.02G19930(	1.9528493	1.57E-05	up	no	Receptor-like	pfam00069	Pkinase	Protein kina	GO:0009755|l	GO:0016021|	GO:0005524|A'	K00924
Manes.02G19940(	16.15583	1.27E-24	up	yes	Serine/threon	pfam00069	Pkinase	Protein kina	GO:0009755|l	GO:0016021|	GO:0005524|A'	.
Manes.02G19970(	1.1721476	0.21808	up	no	Glyoxylate re	pfam02826	2-Hacid_dh_C	D-isomer sp	.	GO:0005737|	GO:0047964|gl	K00015
Manes.02G20010(	0.6710391	0.00909	down	no	Single-strand	pfam08536	Whirly	Whirly trans	GO:0006281|	GO:0005739|	GO:0003677|D	.
Manes.02G20060(	0.6030962	0.0003	down	no	Disease resist	pfam00931	NB-ARC	NB-ARC dc	GO:0006952|	.	GO:0043531|A	.
Manes.02G20090(	0.6131223	0.00015	down	no	Shikimate kir	pfam01202	SKI	Shikimate k	GO:0009073|a	GO:0009507|	GO:0005524|A'	.

Manes.02G20100(	Inf	0.38062	up	no	.	pfam07279	DUF1442	Protein of u	.	.	.	.
Manes.02G20120(	0.5964219	0.01845	down	no	Probable dise	pfam00931	NB-ARC	NB-ARC dc	GO:0006952|(	.	GO:0043531|Al	.
Manes.02G20130(	Inf	0.35135	up	no	.	pfam07279	DUF1442	Protein of u	.	.	.	.
Manes.02G20210(	2.9383953	1.43E-28	up	yes	TMV resistar	pfam01582	TIR	TIR domain	GO:0009626|p	GO:0005737|	GO:0043531|Al	.
Manes.02G20220(	0.3193657	8.93E-16	down	yes	T-complex pı	pfam05794	Tcp11	T-complex p	GO:0030154|c	GO:0016021|	.	.
Manes.02G20270(	0.6760352	0.06742	down	no	.	pfam09835	DUF2062	Uncharacter	.	.	.	.
Manes.02G20330(	21.164875	5.14E-64	up	yes	Probable LRI	pfam00069	Pkinase	Protein kina	.	GO:0016021|	GO:0005524|A'	.
Manes.02G20340(	18.347154	2.64E-15	up	yes	Putative rece	pfam00069	Pkinase	Protein kina	GO:0048544|ı	GO:0016021|	GO:0005524|A'	.
Manes.02G20410(	0.5764756	4.92E-05	down	no	CMP-sialic ac	pfam04142	Nuc_sug_transp	Nucleotide-s	GO:0015780|ı	GO:0005768|	GO:0005338|nu	K15272
Manes.02G20420(	0.4418537	5.34E-06	down	yes	Serpin-Z10	pfam00079	Serpin	Serpin (serir	GO:0010951|ı	GO:0005615|	GO:0004867|se	K13963
Manes.02G20450(	0.9827395	0.90321	down	no	Characteriz	pfam07738	Sad1_UNC	Sad1 / UNC	GO:0034975|ı	GO:0005789|	.	.
Manes.02G20490(	1.0411759	0.64112	up	no	Dynamin-2B	pfam00350	Dynamin_N	Dynamin far	GO:0072583|ı	GO:0009504|	GO:0030276|cla	K01528
Manes.02G20520(	12.959075	3.35E-10	up	yes	Cytochrome	pfam00067	p450	Cytochrome	.	GO:0016021|	GO:0020037|he	.
Manes.02G20550(	15.101583	0.00602	up	yes	Cytochrome	pfam00067	p450	Cytochrome	.	GO:0016021|	GO:0020037|he	.
Manes.02G20560(	5.6997237	1.87E-09	up	yes	Cytochrome	pfam00067	p450	Cytochrome	.	GO:0016021|	GO:0020037|he	.
Manes.02G20590(	0.7220928	0.00268	down	no	TMV resistar	pfam00931	NB-ARC	NB-ARC dc	GO:0009626|p	GO:0005737|	GO:0043531|Al	.
Manes.02G20610(	0.6643794	0.04034	down	no	TMV resistar	pfam01582	TIR	TIR domain	GO:0009626|p	GO:0005737|	GO:0043531|Al	.
Manes.02G20630(	11.79056	0.00061	up	yes	Probable mar	pfam08240	ADH_N	Alcohol deh	.	.	GO:0046029|ma	.
Manes.02G20640(	2.6325006	0.71586	up	no	Probable mar	pfam08240	ADH_N	Alcohol deh	.	.	GO:0046029|ma	.
Manes.02G20680(	1.2511037	1	up	no	Probable mar	pfam08240	ADH_N	Alcohol deh	.	.	GO:0046029|ma	.
Manes.02G20700(	0.7141689	0.20752	down	no	.	pfam07734	FBA_1	F-box assoc	.	.	.	.
Manes.02G20720(	0.6224338	0.04768	down	no	Probable prot	pfam01529	zf-DHHC	DHHC paln	.	GO:0016021|	GO:0019706|pr	.
Manes.02G20790(	0.551918	1.18E-05	down	no	Organic catio	pfam00083	Sugar_tr	Sugar (and c	GO:2001143|l	GO:0016021|	GO:0005524|A'	.
Manes.02G20840(	1.2327171	0.02239	up	no	.	pfam13806	Rieske_2	Rieske-like	.	.	.	.
Manes.02G20860(	0.8030156	0.0322	down	no	Protein ROO	pfam11955	PORR	Plant organ	GO:0010102|l	.	.	.
Manes.02G20870(	4.4134996	0.00021	up	yes	.	pfam09606	Med15	ARC105 or	.	.	.	.
Manes.02G20900(	0.8743467	0.2464	down	no	ABC transpo	pfam00664	ABC_membrane	ABC transp	GO:0006879|(	GO:0009507|	GO:0005524|A'	K05663
Manes.02G20940(	0.4679013	0.00026	down	yes	Uncharacteriz	pfam10674	Ycf54	Protein of u	.	GO:0009507|	.	.
Manes.02G21080(	1.0286277	0.75173	up	no	.	pfam07765	KIP1	KIP1-like pı	.	.	.	.
Manes.02G21120(	Inf	0.9009	up	no	.	pfam14223	UBN2	gag-polypep	.	.	.	.
Manes.02G21140(	0.4978319	3.51E-05	down	yes	Probable inac	pfam00069	Pkinase	Protein kina	.	GO:0016021|	GO:0005524|A'	.
Manes.02G21240(	0.7425934	0.03031	down	no	Alkylated DN	pfam08241	Methyltransf_11	Methyltrans	GO:0055114|(	GO:0005829|	GO:0005506|iro	K10770
Manes.02G21270(	Inf	0.1623	up	no	.	pfam10739	DUF2550	Protein of u	.	.	.	.
Manes.02G21280(	5.4743186	3.38E-21	up	yes	Asparagine sy	pfam00733	Asn_synthase	Asparagine	GO:0006529|a	.	GO:0004066|as	K01953
Manes.02G21300(	Inf	0.08088	up	no	Putative pect	pfam01095	Pectinesterase	Pectinestera	GO:0042545|c	GO:0005618|	GO:0045330|as	.
Manes.02G21320(	16.601305	0.00255	up	yes	.	pfam03732	Retrotrans_gag	Retrotransp	.	.	.	.
Manes.02G21340(	#NAME?	1	down	no	.	pfam07767	Nop53	Nop53 (60S	.	.	.	.
Manes.02G21350(	0.7063672	0.00965	down	no	Ion channel (	pfam06241	DUF1012	Protein of u	GO:0006811|i	GO:0016021|	.	.
Manes.02G21370(	1.5593813	3.53E-06	up	no	Peptidyl-prol	pfam03066	Nucleoplasmin	Nucleoplasn	GO:0006457|p	.	GO:0003755|pe	K14826
Manes.02G21400(	0.2018607	0.00012	down	yes	.	pfam07765	KIP1	KIP1-like pı	.	.	.	.
Manes.02G21480(	0.5318123	0.00244	down	no	.	pfam02438	Adeno_100	Late 100kD	.	.	.	.
Manes.02G21490(	0.5106805	2.21E-05	down	no	Disease resist	pfam00931	NB-ARC	NB-ARC dc	GO:0006952|(	.	GO:0043531|Al	.
Manes.02G21500(	0.4963932	1.73E-08	down	yes	Disease resist	pfam00931	NB-ARC	NB-ARC dc	GO:0006952|(	.	GO:0043531|Al	.
Manes.02G21510(	0.3214906	1.30E-07	down	yes	Disease resist	pfam00931	NB-ARC	NB-ARC dc	GO:0006952|(	.	GO:0043531|Al	.
Manes.02G21520(	0.3841875	0.00058	down	yes	.	pfam01633	Choline_kinase	Choline/etha	.	.	.	.
Manes.02G21530(	0.4696649	4.09E-06	down	yes	Disease resist	pfam00931	NB-ARC	NB-ARC dc	GO:0006952|(	.	GO:0043531|Al	.
Manes.02G21540(	0.4961233	2.02E-05	down	yes	Disease resist	pfam00931	NB-ARC	NB-ARC dc	GO:0006952|(	.	GO:0043531|Al	.
Manes.02G21560(	0.5427395	0.0004	down	no	Disease resist	pfam00931	NB-ARC	NB-ARC dc	GO:0006952|(	.	GO:0043531|Al	.
Manes.02G21580(	0.9128191	0.45655	down	no	Rab3 GTPase	pfam12326	EOS1	N-glycosyla	GO:0007420|l	GO:0005737|	GO:0005096|G'	K18270
Manes.02G21590(	0.8628771	0.28262	down	no	Rab3 GTPase	pfam13890	Rab3-GTPase_cat	Rab3 GTPas	GO:0043547|p	GO:0005737|	GO:0005096|G'	K18270
Manes.02G21630(	0.8807909	0.27373	down	no	Transcription	pfam00076	RRM_1	RNA recogı	GO:0006355|ı	GO:0009507|	GO:0000166|nu	K13098
Manes.02G21660(	8.5405275	0.0001	up	yes	Protein CHL	pfam06203	CCT	CCT motif.	GO:0045036|p	GO:0009507|	.	.
Manes.02G21680(	0.7872075	0.26371	down	no	UDP-glycosy	pfam00201	UDPGT	UDP-glucor	.	.	GO:0016758|tra	.
Manes.02G21710(	0.4194821	0.00016	down	yes	.	pfam14009	DUF4228	Domain of u	.	.	.	.
Manes.02G21760(	0.5946216	3.26E-06	down	no	Pentatricopep	pfam14010	PEPcase_2	Phosphoeno	.	GO:0009507|	.	.
Manes.02G21790(	0.9465858	0.58139	down	no	Probable LRI	pfam07714	Pkinase_Tyr	Protein tyro	.	GO:0016021|	GO:0005524|A'	.
Manes.02G21800(	1.2172521	0.02839	up	no	60S ribosoma	pfam00252	Ribosomal_L16	Ribosomal p	GO:0006412|t	GO:0005840|	GO:0003735|str	.
Manes.02G21830(	0.6150154	1.29E-06	down	no	Plastid divisic	pfam13355	DUF4101	Protein of u	GO:0010020|(	GO:0009507|	GO:0043621|pr	.
Manes.02G21910(	0.3629828	4.55E-07	down	yes	Ribonuclease	pfam09468	RNase_H2-Ydr279	Ydr279p prc	GO:0006401|l	GO:0005634|	.	K10744
Manes.02G21940(	0.7732859	0.03786	down	no	CDT1-like pı	pfam08839	CDT1	DNA replica	GO:0007049|(	GO:0009507|	GO:0004693|cy	K10727
Manes.02G21950(	1.0866254	0.36797	up	no	Ubiquitin-cor	pfam00179	UQ_con	Ubiquitin-cc	GO:0000209|p	GO:0005737|	GO:0005524|A'	K10583
Manes.02G21990(	1.1563283	0.1555	up	no	UPF0426 prc	pfam10317	7TM_GPCR_Srd	Serpentine t	.	GO:0010287|	.	.
Manes.02G22020(	0.9062977	0.30984	down	no	Dolichyl-diph	pfam02516	STT3	Oligosaccha	GO:0006486|p	GO:0005783|	GO:0004576|oli	K07151
Manes.02G22030(	0.7901003	0.04158	down	no	.	pfam01476	LysM	LysM doma	.	.	.	.
Manes.02G22060(	1.4260836	0.00011	up	no	.	pfam03097	BRO1	BRO1-like c	.	.	.	.
Manes.02G22090(	0.5819308	1.40E-06	down	no	Disease resist	pfam00931	NB-ARC	NB-ARC dc	GO:0006952|(	.	GO:0043531|Al	.
Manes.02G22100(	0.5108001	7.51E-05	down	no	.	pfam06086	Pox_A30L_A26L	Orthopoxvir	.	.	.	.
Manes.02G22120(	0.3724116	4.25E-07	down	yes	Disease resist	pfam00931	NB-ARC	NB-ARC dc	GO:0006952|(	.	GO:0043531|Al	.
Manes.02G22130(	0.6646038	0.00956	down	no	Disease resist	pfam00931	NB-ARC	NB-ARC dc	GO:0006952|(	.	GO:0043531|Al	.
Manes.02G22150(	Inf	0.09072	up	no	.	pfam07279	DUF1442	Protein of u	.	.	.	.
Manes.02G22210(	0.434911	1.78E-06	down	yes	Putative F-bc	pfam00646	F-box	F-box doma	GO:0016567|p	.	GO:0004842|ub	.
Manes.02G22270(	0.8482832	0.08642	down	no	Uncharacteriz	pfam03109	ABC1	ABC1 famil	GO:0050821|p	GO:0009507|	GO:0005524|A'	K08869
Manes.02G22290(	0.5662951	0.00441	down	no	.	pfam00665	rve	Integrase co	.	.	.	.
Manes.02G22310(	0.2169361	5.08E-14	down	yes	Microtubule-	pfam13513	HEAT_EZ	HEAT-like r	GO:0010031|(	GO:0010005|	GO:0008017|mi	.
Manes.02G22340(	1.0490077	0.72172	up	no	.	pfam08320	PIG-X	PIG-X / PB	.	.	.	.
Manes.02G22350(	1.6132762	1.51E-07	up	no	Ribosome bic	pfam08145	BOP1NT	BOP1NT (N	GO:0000466|ı	GO:0005730|	GO:0043021|rib	K14824
Manes.02G22360(	0.6308932	5.55E-05	down	no	NifU-like pro	pfam01106	NifU	NifU-like dc	GO:0009658|(	GO:0009507|	GO:0051537|2 i	.
Manes.02G22410(	0.7935085	0.02389	down	no	Probable RN	pfam03909	BSD	BSD domai	GO:0006289|ı	GO:0000439|	.	K03141
Manes.02G22420(	1.1273407	0.29824	up	no	Glycine-rich	pfam00076	RRM_1	RNA recogı	GO:0006397|ı	.	GO:0000166|nu	.
Manes.02G22460(	2.1042263	4.48E-12	up	yes	YTH domain	pfam04146	YTH	YT521-B-lil	.	.	GO:1990247|N(	.
Manes.02G22490(	0.5230727	1.17E-06	down	no	.	pfam04937	DUF659	Protein of u	.	.	.	.
Manes.02G22500(	0.5436567	3.87E-08	down	no	.	pfam04937	DUF659	Protein of u	.	.	.	.
Manes.02G22540(	0.7735355	0.06149	down	no	Organic catio	pfam00083	Sugar_tr	Sugar (and c	GO:2001143|l	GO:0016021|	GO:0005524|A'	.
Manes.02G22640(	0.7859383	0.04717	down	no	.	pfam07000	DUF1308	Protein of u	.	.	.	.
Manes.02G22650(	6.7770866	4.24E-05	up	yes	Protein YLS	pfam03168	LEA_2	Late embryc	GO:0051607|(	GO:0009507|	.	.
Manes.02G22670(	0.750659	0.04983	down	no	U11/U12 sm	pfam05031	NEAT	Iron Transp	GO:0006397|ı	GO:0005681|	.	K13153
Manes.03G00030(	1.1464366	0.13129	up	no	Calcium hom	pfam01805	Surp	Surp modul	GO:0006874|(	GO:0005737|	GO:0044325|io	K12841
Manes.03G00040(	1.0048214	0.9093	up	no	DNA topoiso	pfam01131	Topoisom_bac	DNA topois	GO:0006265|l	GO:0005694|	GO:0003677|D	K03168

Manes.03G00050(	0.9842029	0.92248	down	no	DNA topoiso	pfam01396	zf-C4_Topoisom	Topoisomer	GO:0006265|	GO:0005694|	GO:0003677|D	K03168
Manes.03G00070(	0.5509104	0.00068	down	no	U-box domai	pfam00069	Pkinase	Protein kina	.	.	GO:0005524|A	.
Manes.03G00080(	0.3805033	1.40E-16	down	yes	U-box domai	pfam00069	Pkinase	Protein kina	.	.	GO:0005524|A	.
Manes.03G00090(	0.4552725	4.75E-06	down	yes	.	pfam12056	DUF3537	Protein of u	.	.	.	.
Manes.03G00130(	0.5164188	9.93E-09	down	no	L-arabinokin	pfam13528	Glyco_trans_1_3	Glycosyl tra	GO:0046835|	GO:0005829|	GO:0005524|A	K12446
Manes.03G00170(	296.81291	6.38E-39	up	yes	Probable calc	pfam13499	EF-hand_7	EF-hand do	GO:0046686|	.	GO:0005509|ca	K13448
Manes.03G00180(	1.2445478	0.0167	up	no	Characteriz	pfam00144	Beta-lactamase	Beta-lactam	.	GO:0005576|	GO:0005524|A	K08869
Manes.03G00200(	1.0672977	0.47044	up	no	.	pfam08614	ATG16	Autophagy	.	.	.	.
Manes.03G00210(	1.0596233	0.773	up	no	.	pfam03731	Ku_N	Ku70/Ku80	.	.	.	.
Manes.03G00230(	1.2804204	0.00697	up	no	Poly(rC)-bin	pfam00013	KH_1	KH domain.	GO:0016071|	GO:0005829|	GO:0003677|D	K13162
Manes.03G00250(	1.0692941	0.51555	up	no	Replication p	pfam08784	RPA_C	Replication	GO:0006310|	GO:0005634|	GO:0003677|D	K10739
Manes.03G00260(	Inf	0.9009	up	no	.	pfam14907	NTP_transf_5	Uncharacter	.	.	.	.
Manes.03G00270(	0.9901855	0.94181	down	no	.	pfam10262	Rdx	Rdx family.	.	.	.	.
Manes.03G00280(	Inf	0.04432	up	no	.	pfam02485	Branch	Core-2/I-Br	.	.	.	.
Manes.03G00290(	1.1745632	0.10455	up	no	Uncharacteriz	pfam03109	ABC1	ABC1 famil	GO:0015996|	GO:0009507|	GO:0005524|A	.
Manes.03G00300(	0.5537859	1.15E-07	down	no	HBS1-like pr	pfam00009	GTP_EFTU	Elongation f	GO:0007165|	GO:0070062|	GO:0005525|G	K14416
Manes.03G00320(	0.8113713	0.07482	down	no	Zinc finger p	pfam12756	zf-C2H2_2	C2H2 type	GO:0008631|	GO:0005737|	GO:0044822|pc	K14816
Manes.03G00350(	3.8436377	1.58E-16	up	yes	Plasma memb	pfam00122	E1-E2_ATPase	E1-E2 ATP	GO:0006754|	GO:0016021|	GO:0005524|A	.
Manes.03G00390(	2.0497225	1.58E-15	up	yes	Probable E3	pfam13639	zf-RING_2	Ring finger	GO:0016567|	.	GO:0016874|lig	.
Manes.03G00420(	3.8563165	1.28E-06	up	yes	.	pfam02600	DsbB	Disulfide bo	.	.	.	.
Manes.03G00440(	2.8335368	0.00129	up	yes	.	pfam00719	Pyrophosphatase	Inorganic py	.	.	.	.
Manes.03G00460(	1.5340899	0.06656	up	no	Probable LRI	pfam00069	Pkinase	Protein kina	.	GO:0016021|	GO:0005524|A	.
Manes.03G00480(	0.3339667	0.00361	down	yes	Probable sarc	pfam01266	DAO	FAD depen	GO:0046653|	.	GO:0008115|sa	K00306
Manes.03G00540(	0.206393	1.84E-12	down	yes	Putative B3	pfam02362	B3	B3 DNA bin	GO:0009793|	GO:0005634|	GO:0003677|D	.
Manes.03G00550(	1.094202	0.40518	up	no	Ubiquitin car	pfam00443	UCH	Ubiquitin ca	GO:0043161|	GO:0016021|	GO:0004197|cy	.
Manes.03G00560(	0.3594909	2.33E-11	down	yes	Histidine kin	pfam02518	HATPase_c	Histidine kin	GO:0009738|	GO:0005737|	GO:0009927|his	.
Manes.03G00570(	1.0278755	0.77609	up	no	Transcription	pfam03540	TFIID_30kDa	Transcriptio	GO:0006352|	GO:0005634|	.	K03134
Manes.03G00590(	0.7975675	0.07657	down	no	ADP-ribosyl	pfam00025	Arf	ADP-ribosy	GO:0015031|	GO:0005794|	GO:0005525|G	K07942
Manes.03G00670(	0.8047248	0.06468	down	no	Lariat debran	pfam05011	DBR1	Lariat debra	GO:0009790|	GO:0005634|	GO:0008419|R	.
Manes.03G00700(	1.9166091	3.13E-11	up	no	.	pfam00684	DnaJ_CXXCXGX	DnaJ centra	.	.	.	.
Manes.03G00740(	44.302144	1.21E-07	up	yes	Uncharacteriz	pfam11884	DUF3404	Domain of u	.	GO:0016021|	.	.
Manes.03G00750(	0.8754446	0.17131	down	no	U2 snRNP-as	pfam01805	Surp	Surp modul	GO:0006396|	GO:0005634|	GO:0000166|nu	K12842
Manes.03G00760(	1.9260087	7.82E-13	up	no	Enhancer of	pfam01133	ER	Enhancer of	GO:0007049|	.	.	.
Manes.03G00830(	0.4629717	0.00041	down	yes	Early nodulin	pfam02298	Cu_bind_like	Plastocyanin	.	GO:0031225|	GO:0009055|el	.
Manes.03G00870(	0.8507827	0.22123	down	no	Phospholipas	pfam13091	PLDc_2	PLD-like do	GO:0016042|	.	GO:0070290|N	K16860
Manes.03G00880(	0.4584407	6.33E-06	down	yes	Protein POL	pfam02355	SecD_SecF	Protein expo	GO:0051301|	GO:0005634|	.	.
Manes.03G00920(	1.4548301	0.01614	up	no	.	pfam02181	FH2	Formin Hon	.	.	.	.
Manes.03G00930(	221.70755	#######	up	yes	Probable WR	pfam03106	WRKY	WRKY DN	GO:0042742|	GO:0005634|	GO:0043565|se	.
Manes.03G00950(	28.965181	#######	up	yes	Probable WR	pfam03106	WRKY	WRKY DN	GO:0042742|	GO:0005634|	GO:0043565|se	.
Manes.03G00970(	0.5238656	0.00033	down	no	Transcription	pfam00400	WD40	WD domain	GO:0006355|	GO:0005634|	.	K03130
Manes.03G00980(	0.311311	4.25E-09	down	yes	.	pfam07185	DUF1404	Protein of u	.	.	.	.
Manes.03G01010(	0.8202653	0.06827	down	no	Stromal cell-	pfam02815	MIR	MIR domai	GO:0042742|	GO:0005783|	.	.
Manes.03G01040(	1.0388992	0.66971	up	no	CHD3-type c	pfam00176	SNF2_N	SNF2 family	GO:0008283|	GO:0005634|	GO:0005524|A	K11643
Manes.03G01090(	0.7061115	0.0005	down	no	DeSI-like pro	pfam05903	Peptidase_C97	PPPDE puta	.	.	GO:0008233|pe	.
Manes.03G01120(	0.7515635	0.11773	down	no	Probable rec	pfam07714	Pkinase_Tyr	Protein tyro	.	GO:0016021|	GO:0005524|A	.
Manes.03G01160(	0.955987	0.70752	down	no	Aberrant roo	pfam08568	Kinetochor_Ybp2	Uncharacter	GO:0007275|	GO:0005634|	.	.
Manes.03G01170(	0.9496514	0.60778	down	no	Trafficking p	pfam08626	TRAPPC9-Trs120	Transport p	GO:0030154|	GO:0005783|	.	.
Manes.03G01180(	0.8985368	0.26572	down	no	Homeobox p	pfam03791	KNOX2	KNOX2 dor	GO:0006355|	GO:0005634|	GO:0003677|D	.
Manes.03G01250(	0.5485791	1.60E-07	down	no	.	pfam10324	7TM_GPCR_Srw	Serpentine t	.	.	.	.
Manes.03G01280(	1.060153	0.58661	up	no	Probable beta	pfam01762	Galactosyl_T	Galactosyltr	GO:0006486|	GO:0000139|	GO:1990714|hy	.
Manes.03G01290(	1.013567	0.89341	up	no	ER membran	pfam09430	DUF2012	Protein of u	.	GO:0005783|	GO:0030246|ca	.
Manes.03G01300(	0.7711305	0.07363	down	no	Uncharacteriz	pfam03151	TPT	Triose-phos	GO:0055085|	GO:0016021|	.	K15289
Manes.03G01320(	0.5155864	0.09048	down	no	Synaptotagm	pfam00168	C2	C2 domain.	.	GO:0005783|	GO:0046872|m	.
Manes.03G01330(	0.8236856	0.04471	down	no	Ubiquitin car	pfam00917	MATH	MATH dom	GO:0006511|	GO:0005829|	GO:0008234|cy	.
Manes.03G01370(	0.7212217	0.00083	down	no	.	pfam05483	SCP-1	Synaptonem	.	.	.	.
Manes.03G01380(	0.3789954	1.79E-09	down	yes	E3 ubiquitin-	pfam00069	Pkinase	Protein kina	GO:0009738|	GO:0005769|	GO:0005524|A	.
Manes.03G01400(	13.319162	3.83E-30	up	yes	U11/U12 sma	pfam00240	ubiquitin	Ubiquitin fa	GO:0006397|	GO:0005681|	.	K13153
Manes.03G01420(	0.5492425	5.98E-07	down	no	.	pfam10181	PIG-H	GPI-GlcNA	.	.	.	.
Manes.03G01430(	0.5904545	2.60E-05	down	no	.	pfam13310	Virulence_RhuM	Virulence p	.	.	.	.
Manes.03G01440(	0.5529044	0.00148	down	no	.	pfam02176	zf-TRAF	TRAF-type	.	.	.	.
Manes.03G01510(	1.8772384	1.52E-09	up	no	EIN3-binding	pfam00646	F-box	F-box doma	GO:0009873|	GO:0005634|	.	K14515
Manes.03G01540(	2.4126626	0.00014	up	yes	Ethylene-resp	pfam00847	AP2	AP2 domain	GO:0009873|	GO:0005634|	GO:0003677|D	.
Manes.03G01560(	1.9552197	2.86E-14	up	no	DEAD-box A	pfam00270	DEAD	DEAD/DEA	GO:0006397|	GO:0005634|	GO:0005524|A	.
Manes.03G01580(	Inf	1.09E-06	up	yes	Probable poly	pfam00295	Glyco_hydro_28	Glycosyl hy	GO:0005975|	GO:0031225|	GO:0004650|pc	.
Manes.03G01610(	0.4044144	3.58E-05	down	yes	.	pfam14432	DYW_deaminase	DYW family	.	.	.	.
Manes.03G01650(	1.1858303	0.06534	up	no	Cellulose syn	pfam03552	Cellulose_synt	Cellulose sy	GO:0071555|	GO:0005768|	GO:0016760|ce	K10999
Manes.03G01740(	1.4408357	0.00049	up	no	Pentatricope	pfam13041	PPR_2	PPR repeat	.	GO:0005739|	.	.
Manes.03G01790(	0.510227	7.32E-07	down	no	Probable 1-d	pfam13292	DXP_synthase_N	1-deoxy-D-	GO:0052865|	GO:0009507|	GO:0008661|1-	.
Manes.03G01820(	0.2091083	2.92E-22	down	yes	3-ketoacyl-C	pfam08392	FAE1_CUT1_Rpp	FAE1/Type	GO:0006633|	GO:0016021|	GO:0033807|ic	K15397
Manes.03G01850(	Inf	0.35135	up	no	Protein ECEI	pfam02458	Transferase	Transferase	GO:0071555|	GO:0009535|	GO:0016747|tra	.
Manes.03G01890(	1.300032	0.37549	up	no	.	pfam05311	Baculo_PP31	Baculovirus	.	.	.	.
Manes.03G01920(	1.2944025	0.32349	up	no	Multiprotein-	pfam08523	MBF1	Multiprotei	GO:0009873|	GO:0005737|	GO:0043565|se	.
Manes.03G01930(	0.9665791	0.78518	down	no	FRIGIDA-lik	pfam07899	Frigida	Frigida-like	GO:0030154|	.	.	.
Manes.03G01940(	6.2296778	7.79E-18	up	yes	Arabinogalac	pfam06376	DUF1070	Protein of u	.	GO:0031225|	.	.
Manes.03G01970(	1.0343468	0.73182	up	no	.	pfam00499	Oxidored_q3	NADH-ubiq	.	.	.	.
Manes.03G01980(	1.2136945	0.24513	up	no	Ras-related p	pfam00071	Ras	Ras family.	GO:0015031|	GO:0005622|	GO:0005525|G	.
Manes.03G01990(	3.0890675	7.01E-08	up	yes	.	pfam13136	DUF3984	Protein of u	.	.	.	.
Manes.03G02090(	0.0631197	0.00018	down	yes	Transcription	pfam04844	Ovate	Transcriptio	GO:0045892|	GO:0005634|	.	.
Manes.03G02100(	0.0584747	0.00379	down	yes	Probable poly	pfam00083	Sugar_tr	Sugar (and	GO:0046323|	GO:0005887|	GO:0005355|gl	.
Manes.03G02130(	0.952241	0.60878	down	no	.	pfam00118	Cpn60_TCP1	TCP-1/cpn6	.	.	.	.
Manes.03G02140(	1.7119563	3.17E-07	up	no	Protein cfxQ	pfam12796	Ank_2	Ankyrin rep	.	GO:0009507|	GO:0005524|A	.
Manes.03G02170(	3.3662887	1.89E-14	up	yes	Pentatricope	pfam13041	PPR_2	PPR repeat	.	.	GO:0005524|A	.
Manes.03G02240(	1.4516349	8.32E-05	up	no	Aspartate am	pfam00155	Aminotran_1_2	Aminotrans	GO:0006103|	GO:0005737|	GO:0004069|L-	.
Manes.03G02290(	1.0464941	0.63856	up	no	Peroxisomal	pfam02737	3HCDH_N	3-hydroxya	GO:0006635|	GO:0005618|	GO:0003857|3-	K10527
Manes.03G02300(	1.3848688	0.00076	up	no	Protein tesmi	pfam03638	TCR	Tesmin/TSC	GO:0007275|	GO:0005634|	GO:0046872|m	.
Manes.03G02310(	1.3253476	0.00236	up	no	.	pfam00641	zf-RanBP	Zn-finger in	.	.	.	.
Manes.03G02320(	0.8901798	0.47743	down	no	Probable plas	pfam00083	Sugar_tr	Sugar (and	GO:0008643|	GO:0031969|	GO:0022891|su	.
Manes.03G02340(	0.8736247	0.43737	down	no	Rac-like GTI	pfam00071	Ras	Ras family.	GO:0009738|	GO:0005622|	GO:0005525|G	K04392

Manes.03G02390(	0.243829	0.00678	down	yes	DNA-directe	pfam01191	RNA_pol_Rpb5_(	RNA polym	GO:0006360|	GO:0005736|	GO:0003677|DI	K03013
Manes.03G02410(	0.7606519	0.01466	down	no	Elongation fa	pfam00889	EF_TS	Elongation f	.	GO:0005737|	GO:0003746|tra	K02357
Manes.03G02440(	9.1496207	1.42E-20	up	yes	Probable xylo	pfam00722	Glyco_hydro_16	Glycosyl hyd	GO:0042546|	GO:0048046|	GO:0004553|hy	K08235
Manes.03G02460(	0.9810933	0.84892	down	no	Casein kinase	pfam00069	Pkinase	Protein kina	GO:0006897|	GO:0005737|	GO:0005524|AT	K02218
Manes.03G02500(	226.34865	5.98E-31	up	yes	Sugar transpo	pfam00083	Sugar_tr	Sugar (and c	GO:0046323|	GO:0005887|	GO:0005355|glu	.
Manes.03G02510(	0.4221671	1.01E-10	down	yes	Probable glyc	pfam03016	Exostosin	Exostosin fa	GO:0071555|	GO:0000139|	GO:0016757|tra	.
Manes.03G02550(	1.1921612	0.12761	up	no	Cell division	pfam12895	Apc3	Anaphase-pr	GO:0030154|	GO:0005680|	.	K03350
Manes.03G02570(	0.3282289	1.98E-12	down	yes	.	pfam13041	PPR_2	PPR repeat	.	.	.	.
Manes.03G02710(	2.4440561	3.51E-17	up	yes	Mitochondria	pfam02466	Tim17	Tim17/Tim2	GO:0006886|	GO:0016021|	GO:0015450|P-	K17795
Manes.03G02750(	0.0883765	0.00246	down	yes	.	pfam14956	DUF4505	Domain of u	.	.	.	.
Manes.03G02760(	0.4362776	3.29E-06	down	yes	Putative ABC	pfam00664	ABC_membrane	ABC transpo	.	GO:0016021|	GO:0005524|AT	.
Manes.03G02790(	5.9772393	1.92E-13	up	yes	.	pfam15402	Spc7_N	N-terminus	.	.	.	.
Manes.03G02820(	0.7719454	0.01597	down	no	Glucan endo-	pfam00332	Glyco_hydro_17	Glycosyl hyd	GO:0005975|	GO:0031225|	GO:0042973|glu	.
Manes.03G02860(	4.917379	5.45E-29	up	yes	.	pfam06219	DUF1005	Protein of u	.	.	.	.
Manes.03G02870(	0.2380087	1.32E-24	down	yes	Leucine-rich	pfam13855	LRR_8	Leucine rich	GO:0071555|	GO:0005618|	GO:0005199|str	.
Manes.03G02880(	0.1506179	3.33E-09	down	yes	.	pfam06125	DUF961	Bacterial pro	.	.	.	.
Manes.03G02930(	0.2826138	2.19E-05	down	yes	ATP-depende	pfam00365	PFK	Phosphofruc	GO:0046835|	GO:0005829|	GO:0003872|6-	K00850
Manes.03G02960(	1.7469532	9.58E-09	up	no	Zinc finger C	pfam00642	zf-CCCH	Zinc finger C	GO:0006355|	GO:0005886|	GO:0003677|DI	.
Manes.03G03000(	1.0662897	0.56071	up	no	IAA-amino a	pfam01546	Peptidase_M20	Peptidase fa	GO:0009611|	GO:0005783|	GO:0010179|IA	K14664
Manes.03G03040(	0.6700366	0.00269	down	no	.	pfam00646	F-box	F-box doma	.	.	.	.
Manes.03G03050(	4.0624253	5.45E-07	up	yes	Probable caff	pfam01596	Methyltransf_3	O-methyltra	GO:0009809|	GO:0005829|	GO:0042409|ca	K00588
Manes.03G03070(	1.5237059	4.78E-06	up	no	.	pfam04718	ATP-synt_G	Mitochondri	.	.	.	.
Manes.03G03150(	1.2136059	0.07882	up	no	Protein argini	pfam06325	PrmA	Ribosomal p	GO:0034969|	GO:0005737|	GO:0008469|his	K11434
Manes.03G03180(	0.7198632	0.00079	down	no	Serine acetylt	pfam06426	SATase_N	Serine acety	GO:0006535|	GO:0005829|	GO:0009001|se	K00640
Manes.03G03220(	Inf	0.9009	up	no	.	pfam03732	Retrotrans_gag	Retrotranspo	.	.	.	.
Manes.03G03230(	0.4937575	3.78E-08	down	yes	Transcription	pfam00249	Myb_DNA-binding	Myb-like DI	GO:0048262|	GO:0005634|	GO:0003677|DI	.
Manes.03G03270(	0.7247274	0.00204	down	no	Demethylmer	pfam08241	Methyltransf_11	Methyltrans	GO:0009234|	.	GO:0008168|me	K03183
Manes.03G03290(	0.9168465	0.39022	down	no	Thioredoxin-	pfam00085	Thioredoxin	Thioredoxin	GO:0045454|	GO:0009507|	GO:0016671|ox	.
Manes.03G03310(	0.4213269	2.12E-11	down	yes	.	pfam10325	7TM_GPCR_Srz	Serpentine t	.	.	.	.
Manes.03G03320(	0.3368237	6.93E-21	down	yes	Putative GAT	pfam00320	GATA	GATA zinc	GO:0007623|	GO:0005634|	GO:0043565|se	.
Manes.03G03360(	0.4633959	6.80E-07	down	yes	.	pfam04750	Far-17a_AIG1	FAR-17a/AI	.	.	.	.
Manes.03G03370(	0.3563286	3.35E-18	down	yes	Probable poly	pfam01593	Amino_oxidase	Flavin conta	GO:0048510|	GO:0005737|	GO:1990534|th	K12259
Manes.03G03380(	0.3649444	0.19644	down	no	.	pfam04780	DUF629	Protein of u	.	.	.	.
Manes.03G03390(	0.9010558	0.27127	down	no	WD-40 repea	pfam12265	CAF1C_H4-bd	Histone-bind	GO:0030154|	GO:0080008|	GO:0046872|me	K10752
Manes.03G03440(	1.2055564	0.04746	up	no	Translation in	pfam01253	SUI1	Translation	GO:0070124|	GO:0005739|	GO:0003743|tra	.
Manes.03G03550(	0.7727816	0.23515	down	no	Heat stress tr	pfam00447	HSF_DNA-bind	HSF-type D	GO:0034605|	GO:0005737|	GO:0043565|se	.
Manes.03G03570(	1.5392753	3.37E-06	up	no	Casein kinase	pfam00069	Pkinase	Protein kina	GO:0006897|	GO:0005737|	GO:0005524|AT	K02218
Manes.03G03590(	1.7354493	5.46E-06	up	no	.	pfam12023	DUF3511	Domain of u	.	.	.	.
Manes.03G03620(	0.8727883	0.23554	down	no	THO comple	pfam00400	WD40	WD domain	GO:0009738|	GO:0080008|	GO:0003723|RI	K13175
Manes.03G03630(	2.8906732	1.01E-17	up	yes	Disease resist	pfam00931	NB-ARC	NB-ARC do	GO:0006952|	GO:0005737|	GO:0043531|AI	K13459
Manes.03G03650(	0.9521304	0.64573	down	no	U-box domai	pfam00069	Pkinase	Protein kina	.	.	GO:0005524|AT	.
Manes.03G03670(	1.0341935	0.88458	up	no	.	pfam11884	DUF3404	Domain of u	.	.	.	.
Manes.03G03680(	0.6551103	2.17E-05	down	no	.	pfam05892	Tricho_coat	Trichovirus	.	.	.	.
Manes.03G03690(	2.536585	7.70E-12	up	yes	Protein phos	pfam00481	PP2C	Protein phos	GO:0009738|	GO:0005737|	GO:0046872|me	K14497
Manes.03G03710(	0.8092466	0.10225	down	no	Pre-mRNA c	pfam13869	NUDIX_2	Nucleotide I	GO:0006378|	GO:0005849|	GO:0016787|hy	K14397
Manes.03G03750(	1.1840221	0.07566	up	no	Myosin-17	pfam00063	Myosin_head	Myosin head	GO:0030048|	GO:0016459|	GO:0005524|AT	.
Manes.03G03770(	0.7476784	0.03698	down	no	Protein bem4	pfam12695	Abhydrolase_5	Alpha/beta I	.	GO:0005783|	.	K06889
Manes.03G03810(	3.0855341	1.33E-23	up	yes	Vacuolar pro	pfam03357	Snf7	Snf7. This fa	GO:0070676|	GO:0000815|	.	K12191
Manes.03G03830(	0.6146465	0.01211	down	no	Mechanosens	pfam00924	MS_channel	Mechanoser	GO:0006820|	GO:0016021|	GO:0008381|me	.
Manes.03G03870(	8.97917	6.18E-19	up	yes	.	pfam03478	DUF295	Protein of u	.	.	.	.
Manes.03G03950(	0.4487913	1.63E-05	down	yes	Growth-regu	pfam08879	WRC	WRC. The V	GO:0032502|	GO:0005634|	GO:0005524|AT	.
Manes.03G03990(	0.4544229	2.68E-07	down	yes	Auxin-binding	pfam00190	Cupin_1	Cupin. This	GO:0009734|	GO:0048046|	GO:0030145|ma	.
Manes.03G04000(	1.8965952	4.04E-08	up	no	MOB kinase	pfam03637	Mob1_phocein	Mob1/phoce	.	.	GO:0046872|me	K06685
Manes.03G04010(	0.9066557	0.32377	down	no	Uncharacteriz	pfam10250	O-FucT	GDP-fucose	.	GO:0005768|	GO:0016757|tra	.
Manes.03G04030(	0.7554372	0.03775	down	no	.	pfam02586	DUF159	Uncharacter	.	.	.	.
Manes.03G04040(	1.0249428	0.80278	up	no	Tubulin--tyro	pfam03133	TTL	Tubulin-tyro	GO:0006464|	.	.	K16609
Manes.03G04110(	0.5934566	3.42E-05	down	no	Copper transp	pfam04145	Ctr	Ctr copper t	GO:0015680|	GO:0005794|	GO:0005375|co	K14686
Manes.03G04120(	0.1707911	0.00073	down	yes	Cation/calciu	pfam01699	Na_Ca_ex	Sodium/calc	GO:0006812|	GO:0016021|	GO:0015297|an	K13754
Manes.03G04140(	0.5304795	0.00041	down	no	Adipocyte pla	pfam03088	Str_synth	Strictosidine	GO:0009058|	GO:0016021|	GO:0016844|str	.
Manes.03G04150(	1.3595654	0.00175	up	no	Proteasome s	pfam00227	Proteasome	Proteasome	GO:0006511|	GO:0005737|	GO:0004298|th	.
Manes.03G04160(	0.5042841	1.65E-05	down	no	Beta-galactos	pfam01301	Glyco_hydro_35	Glycosyl hyd	GO:0005975|	GO:0048046|	GO:0004565|be	.
Manes.03G04170(	4.1241178	2.54E-34	up	yes	.	pfam00092	VWA	von Willebra	.	.	.	.
Manes.03G04240(	0.7362757	0.015	down	no	Probable poly	pfam00295	Glyco_hydro_28	Glycosyl hyd	GO:0005975|	GO:0016021|	GO:0004650|po	.
Manes.03G04250(	9.3809302	2.54E-17	up	yes	Protein TIFY	pfam06200	tify	tify domain.	GO:0006952|	GO:0005634|	.	K13464
Manes.03G04260(	0.414977	3.73E-08	down	yes	.	pfam04570	DUF581	Protein of u	.	.	.	.
Manes.03G04330(	0.6004811	0.03495	down	no	Auxin-induce	pfam02519	Auxin_inducible	Auxin respo	GO:0009734|	.	.	.
Manes.03G04340(	0.5421459	0.00299	down	no	.	pfam01544	CorA	CorA-like M	.	.	.	.
Manes.03G04350(	21.968569	7.15E-18	up	yes	.	pfam01252	Peptidase_A8	Signal pepti	.	.	.	.
Manes.03G04380(	0.7197586	0.12601	down	no	.	pfam14372	DUF4413	Domain of u	.	.	.	.
Manes.03G04420(	3.0085274	6.72E-06	up	yes	Pectinesteras	pfam01095	Pectinesterase	Pectinestera	GO:0042545|	GO:0005618|	GO:0045330|as	.
Manes.03G04440(	1.2431	0.21541	up	no	Sucrose synth	pfam00862	Sucrose_synth	Sucrose syn	GO:0009058|	.	GO:0016157|su	K00695
Manes.03G04480(	0.8591925	0.13729	down	no	Phosphoinosi	pfam02383	Syja_N	SacI homolo	GO:0036092|	GO:0005774|	GO:0042578|ph	.
Manes.03G04490(	1.3849457	0.10111	up	no	.	pfam10292	7TM_GPCR_Srab	Serpentine t	.	.	.	.
Manes.03G04500(	0.9051532	0.39204	down	no	SH3 domain-	pfam04366	DUF500	Family of un	GO:0007015|	GO:0030479|	.	.
Manes.03G04510(	1.9795755	5.82E-05	up	no	Probable E3	pfam01485	IBR	IBR domain	.	GO:0016021|	GO:0016874|lig	K11977
Manes.03G04530(	0.5221919	1.35E-09	down	no	AT-rich inter	pfam01388	ARID	ARID/BRIG	GO:0006355|	GO:0005634|	GO:0003677|DI	.
Manes.03G04600(	1.1473046	0.14283	up	no	Exonuclease	pfam01612	DNA_pol_A_exo1	3'-5' exonuc	.	.	GO:0008408|3'-	K18740
Manes.03G04610(	1.7320719	6.54E-07	up	no	.	pfam05907	DUF866	Eukaryotic p	.	.	.	.
Manes.03G04620(	1.3316847	0.02103	up	no	.	pfam02485	Branch	Core-2/I-Br	.	.	.	.
Manes.03G04630(	0.1557931	0.00054	down	yes	.	pfam05721	PhyH	Phytanoyl-C	.	.	.	.
Manes.03G04640(	0.9301169	0.50359	down	no	.	pfam07797	DUF1639	Protein of u	.	.	.	.
Manes.03G04670(	2.175743	2.65E-14	up	yes	Uncharacteriz	pfam08576	DUF1764	Eukaryotic p	.	GO:0005829|	.	.
Manes.03G04680(	2.3192049	9.10E-21	up	yes	Ferrochelatas	pfam00762	Ferrochelatase	Ferrochelata	GO:0006783|	GO:0009507|	GO:0004325|fer	.
Manes.03G04700(	0.5434675	5.57E-05	down	no	.	pfam08568	Kinetochor_Ybp2	Uncharacter	.	.	.	.
Manes.03G04710(	0.5432326	2.60E-07	down	no	.	pfam03096	Ndr	Ndr family.	.	.	.	.
Manes.03G04750(	0.355801	2.18E-05	down	yes	Abscisic acid	pfam10604	Polyketide_cyc2	Polyketide c	GO:0009738|	GO:0005737|	GO:0010427|ab	K14496
Manes.03G04760(	0.9221902	0.61573	down	no	Histone deac	pfam00850	Hist_deacetyl	Histone dea	GO:0006355|	GO:0005634|	GO:0032041|N.	K11418
Manes.03G04770(	6.637281	3.12E-29	up	yes	Protein REV	pfam05608	DUF778	Protein of u	GO:0010105|	GO:0005783|	.	.

Manes.03G04800(	0.399903	2.72E-07	down	yes	.	pfam05875	Ceramidase	Ceramidase.	.	.	.	.
Manes.03G04830(	0.4387299	1.26E-05	down	yes	Probable leuc	pfam00069	Pkinase	Protein kina	.	GO:0016021|	GO:0005524|A'	.
Manes.03G04910(	0.2866123	1.14E-25	down	yes	Zinc finger pr	pfam14093	DUF4271	Domain of u	GO:0006355|r	GO:0005634|	GO:0046872|m	.
Manes.03G04920(	0.5432724	1.30E-06	down	no	Beta-fructofu	pfam00251	Glyco_hydro_32N	Glycosyl hyd	GO:0005975|c	GO:0048046|	GO:0004564|be	K01193
Manes.03G04940(	4.2305221	6.39E-17	up	yes	Glutaredoxin	pfam00462	Glutaredoxin	Glutaredoxi	GO:0045454|c	GO:0005737|	GO:0009055|ele	.
Manes.03G04950(	0.2103024	0.01002	down	yes	Heat shock fa	pfam00447	HSF_DNA-bind	HSF-type D	GO:0009408|r	GO:0005634|	GO:0043565|se	.
Manes.03G04970(	4.1507896	3.92E-35	up	yes	.	pfam03842	Silic_transp	Silicon trans	.	.	.	.
Manes.03G04990(	0.6909695	0.07434	down	no	Cytokinin rib	pfam03641	Lysine_decarbox	Possible lysi	GO:0009691|c	GO:0005829|	GO:0016799|hy	.
Manes.03G05000(	1.3486013	0.00879	up	no	Probable mag	pfam00892	EamA	EamA-like t	GO:0015693|r	GO:0005769|	GO:0015095|ma	.
Manes.03G05010(	3.9802306	0.18707	up	no	.	pfam03888	MucB_RseB	MucB/RseB	.	.	.	.
Manes.03G05020(	0.3479145	0.00077	down	yes	Pentatricopep	pfam13041	PPR_2	PPR repeat	GO:0006397|r	GO:0009507|	.	.
Manes.03G05070(	0.6757724	0.00032	down	no	Serine/threon	pfam00069	Pkinase	Protein kina	.	.	GO:0005524|A'	.
Manes.03G05080(	0.3241949	4.47E-15	down	yes	Protein phosp	pfam00481	PP2C	Protein phos	GO:0009738|a	GO:0005737|	GO:0046872|m	K14497
Manes.03G05110(	0.725353	0.01343	down	no	Aminopeptida	pfam01433	Peptidase_M1	Peptidase fa	GO:0009926|a	GO:0005829|	GO:0004177|an	K08776
Manes.03G05120(	1.029538	0.7353	up	no	.	pfam10358	NT-C2	N-terminal C	.	.	.	.
Manes.03G05130(	6.5312329	1.27E-05	up	yes	Probable WR	pfam03106	WRKY	WRKY DN	GO:0050832|c	GO:0005634|	GO:0043565|se	.
Manes.03G05160(	0.945848	1	down	no	18.8 kDa clas	pfam00011	HSP20	Hsp20/alpha	.	GO:0005737|	.	.
Manes.03G05180(	0.3460618	5.90E-14	down	yes	Solute carrier	pfam00153	Mito_carr	Mitochondr	GO:0055085|t	GO:0016021|	.	K15121
Manes.03G05200(	0.8321403	0.07285	down	no	PHD finger p	pfam12165	DUF3594	Domain of u	GO:0016568|c	GO:0005634|	GO:0003677|D	.
Manes.03G05210(	0.2514277	1.01E-06	down	yes	Pentatricopep	pfam13041	PPR_2	PPR repeat	.	GO:0005739|	.	.
Manes.03G05270(	0.8261728	0.0867	down	no	Peroxisome b	pfam04757	Pex2_Pex12	Pex2 / Pex1	GO:0010381|a	GO:0005829|	GO:0004842|ub	K13346
Manes.03G05290(	0.3518415	3.39E-10	down	yes	Cytokinin del	pfam09265	Cytokin-bind	Cytokinin de	GO:0009823|c	.	GO:0019139|cy	K00279
Manes.03G05340(	1.8708902	2.33E-12	up	no	Copper-trans	pfam00122	E1-E2_ATPase	E1-E2 ATP	GO:0035434|c	GO:0009535|	GO:0005524|A'	K01533
Manes.03G05350(	0.9450223	0.76285	down	no	.	pfam12714	TILa	TILa domai	.	.	.	.
Manes.03G05370(	3.4809908	1.90E-19	up	yes	.	pfam08507	COPI_assoc	COPI assoc	.	.	.	.
Manes.03G05380(	Inf	0.9009	up	no	.	pfam14227	UBN2_2	gag-polypep	.	.	.	.
Manes.03G05390(	Inf	0.9009	up	no	Endoglucana	pfam00759	Glyco_hydro_9	Glycosyl hyd	GO:0071555|c	GO:0005576|	GO:0008810|ce	K01179
Manes.03G05420(	0.6640666	0.0072	down	no	2-hydroxy-6-	pfam12697	Abhydrolase_6	Alpha/beta h	GO:0019380|3	GO:0005737|	GO:0052823|2-	K05714
Manes.03G05430(	0.5847415	9.78E-06	down	no	Pectinesteras	pfam01095	Pectinesterase	Pectinestera	GO:0042545|c	GO:0005618|	GO:0045330|as	.
Manes.03G05450(	1.0136124	0.95626	up	no	Glucan endo-	pfam00332	Glyco_hydro_17	Glycosyl hyd	GO:0005975|c	GO:0031225|	GO:0042973|glu	.
Manes.03G05460(	0.668303	0.00067	down	no	.	pfam04784	DUF547	Protein of u	.	.	.	.
Manes.03G05470(	0.8526758	0.0943	down	no	Transcription	pfam05236	TAF4	Transcriptio	GO:0006352|D	GO:0005669|	.	K03129
Manes.03G05490(	0.9218625	0.41873	down	no	Brefeldin A-i	pfam01369	Sec7	Sec7 domai	GO:0042742|c	GO:0005829|	GO:0005086|A	K13462
Manes.03G05500(	5.4449777	3.00E-52	up	yes	E3 ubiquitin-	pfam13639	zf-RING_2	Ring finger c	GO:0016567|p	GO:0016021|	GO:0016874|lig	K10663
Manes.03G05530(	0.8895912	0.4385	down	no	Protein FAT	pfam03647	Tmemb_14	Transmemb	.	GO:0016021|	.	.
Manes.03G05550(	0.8485663	0.15533	down	no	Acyl carrier p	pfam07082	DUF1350	Protein of u	GO:0006633|f	GO:0009507|	.	.
Manes.03G05560(	1.6275751	0.33364	up	no	Acyl carrier p	pfam00550	PP-binding	Phosphopan	GO:0006633|f	GO:0009507|	.	.
Manes.03G05570(	3.7606944	0.0023	up	yes	.	pfam02990	EMP70	Endomembr	.	.	.	.
Manes.03G05580(	1.4677096	4.65E-05	up	no	3-ketoacyl-C	pfam08392	FAE1_CUT1_Rpp	FAE1/Type	GO:0006633|f	GO:0005783|	GO:0016747|tra	K15397
Manes.03G05590(	1.0418384	0.81426	up	no	.	pfam13966	zf-RVT	zinc-binding	.	.	.	.
Manes.03G05650(	0.8918033	0.23651	down	no	Endoribonucl	pfam03368	Dicer_dimer	Dicer dimer	GO:0030422|p	GO:0005737|	GO:0005524|A'	K11592
Manes.03G05660(	0.7394037	0.0692	down	no	DNA topoiso	pfam12738	PTCB-BRCT	twin BRCT	GO:0006974|c	GO:0015629|	GO:0003677|D	K10728
Manes.03G05670(	0.618075	1.34E-06	down	no	.	pfam08590	DUF1771	Domain of u	.	.	.	.
Manes.03G05710(	6.419584	1.14E-78	up	yes	Zinc finger C	pfam00642	zf-CCCH	Zinc finger C	GO:0006355|r	GO:0005886|	GO:0003677|D	.
Manes.03G05740(	0.9556261	0.63975	down	no	.	pfam01311	Bac_export_1	Bacterial ex	.	.	.	.
Manes.03G05750(	0.9426024	0.54743	down	no	Glutamyl-tRN	pfam02686	Glu-tRNAGln	Glu-tRNAG	GO:0006450|r	GO:0009507|	GO:0005524|A'	K02435
Manes.03G05800(	NA	NA	--	no	Carotenoid 9	pfam03055	RPE65	Retinal pigm	.	GO:0005737|	GO:0051213|dio	.
Manes.03G05810(	6.6310833	0.13453	up	no	Beta-glucosid	pfam00232	Glyco_hydro_1	Glycosyl hyd	GO:0005975|c	.	GO:0008422|be	.
Manes.03G05840(	2.6059693	0.71404	up	no	Probable glyc	pfam03016	Exostosin	Exostosin fa	GO:0071555|c	GO:0000139|	GO:0016757|tra	.
Manes.03G05860(	0.4917365	1.81E-09	down	yes	Programmed	pfam04194	PDCD2_C	Programmed	GO:0006919|a	GO:0005737|	GO:0003677|D	K14801
Manes.03G05870(	0.6097115	5.29E-07	down	no	Fumarate hyd	pfam00206	Lyase_1	Lyase.	GO:0006106|f	GO:0009507|	GO:0004333|fu	.
Manes.03G05890(	0.6087214	1.26E-06	down	no	Probable E3 u	pfam00415	RCC1	Regulator o	GO:0009649|e	GO:0000785|	GO:0003682|ch	K10615
Manes.03G05900(	0.5107802	7.40E-06	down	no	Peptidyl-tRN	pfam01981	PTH2	Peptidyl-tRI	GO:2000811|n	GO:0005829|	GO:0004045|an	K04794
Manes.03G05920(	0.6613001	0.00045	down	no	Homeobox-le	pfam08670	MEKHLA	MEKHLA d	GO:0030154|c	GO:0005634|	GO:0003677|D	K09338
Manes.03G05940(	15.492958	1.05E-12	up	yes	.	pfam07911	DUF1677	Protein of u	.	.	.	.
Manes.03G05950(	1.279287	0.08177	up	no	.	pfam07911	DUF1677	Protein of u	.	.	.	.
Manes.03G05960(	0.6357926	0.05407	down	no	Lachrymatory	pfam10604	Polyketide_cyc2	Polyketide c	.	GO:0005773|	.	.
Manes.03G05980(	4.3573662	1.49E-05	up	yes	.	pfam05553	DUF761	Cotton fibre	.	.	.	.
Manes.03G06000(	1.0162507	0.83803	up	no	Casein kinase	pfam00069	Pkinase	Protein kina	GO:0006281|D	GO:0032153|	GO:0005524|A'	K14758
Manes.03G06060(	14.263708	5.05E-20	up	yes	Receptor-like	pfam00069	Pkinase	Protein kina	GO:0009755|h	GO:0016021|	GO:0005524|A'	K00924
Manes.03G06070(	1.0737537	0.48586	up	no	Endoribonucl	pfam08282	Hydrolase_3	haloacid del	GO:0006364|r	GO:0005737|	GO:0004521|en	K07042
Manes.03G06090(	0.5127184	0.00044	down	no	Putative dise	pfam00931	NB-ARC	NB-ARC do	GO:0006952|c	.	GO:0043531|A	.
Manes.03G06100(	1.3595101	0.00857	up	no	Aspartate am	pfam00155	Aminotran_1_2	Aminotransf	GO:0006103|2	GO:0009507|	GO:0004069|L-	K14454
Manes.03G06110(	0.7443425	0.04455	down	no	Aspartate am	pfam00155	Aminotran_1_2	Aminotransf	GO:0006103|2	GO:0005737|	GO:0004069|L-	K14454
Manes.03G06130(	1.4463397	0.00023	up	no	.	pfam04441	Pox_VERT_large	Poxvirus ear	.	.	.	.
Manes.03G06140(	0.5296547	0.0415	down	no	D-amino-acid	pfam01063	Aminotran_4	Aminotransf	GO:0008153|p	GO:0009507|	GO:0008696|4-	K18482
Manes.03G06150(	0.9867095	0.91736	down	no	D-amino-acid	pfam01063	Aminotran_4	Aminotransf	GO:0008153|p	GO:0009507|	GO:0008696|4-	K18482
Manes.03G06160(	1.2710234	0.00755	up	no	TOM1-like p	pfam00790	VHS	VHS domai	GO:0006886|i	GO:0070062|	GO:0030276|cla	.
Manes.03G06180(	0.867489	0.14544	down	no	ER membran	pfam07774	DUF1620	Protein of u	.	GO:0072546|	.	.
Manes.03G06190(	0.4150408	3.04E-05	down	yes	Putative D-ar	pfam01565	FAD_binding_4	FAD binding	.	GO:0031966|	GO:0003885|D-	K00107
Manes.03G06200(	0.5553265	0.00049	down	no	Protein tesmi	pfam03638	TCR	Tesmin/TSC	GO:0007275|r	GO:0005634|	GO:0046872|m	.
Manes.03G06220(	0.2675847	4.23E-05	down	yes	.	pfam08458	PH_2	Plant plecks	.	.	.	.
Manes.03G06240(	0.1818509	1.04E-05	down	yes	Myosin heavy	pfam00400	WD40	WD domain	GO:0000281|r	GO:0005826|	GO:0005524|A'	.
Manes.03G06250(	1.8792494	4.52E-06	up	no	.	pfam13812	PPR_3	Pentatricope	.	.	.	.
Manes.03G06310(	1.3359703	0.0043	up	no	Coiled-coil do	pfam05670	DUF814	Domain of u	.	GO:0070062|	.	.
Manes.03G06350(	0.3816769	2.02E-16	down	yes	L-type lectin-	pfam00069	Pkinase	Protein kina	GO:0009755|h	GO:0016021|	GO:0005524|A'	.
Manes.03G06370(	1.843532	2.17E-11	up	no	Cell number r	pfam04749	PLAC8	PLAC8 fam	.	GO:0016021|	.	.
Manes.03G06380(	1.2866028	0.18274	up	no	.	pfam11312	DUF3115	Protein of u	.	.	.	.
Manes.03G06390(	0.4654641	3.12E-09	down	yes	.	pfam10326	7TM_GPCR_Str	Serpentine t	.	.	.	.
Manes.03G06410(	4.6172071	0.01166	up	yes	Potassium ch	pfam00520	Ion_trans	Ion transpor	GO:0010163|h	GO:0005783|	GO:0005249|vo	.
Manes.03G06420(	1.0017207	0.96717	up	no	Protein DDB	pfam06814	Lung_7-TM_R	Lung seven	.	.	.	K19027
Manes.03G06460(	0.5920334	0.00017	down	no	RING finger	pfam14599	zinc_ribbon_6	Zinc-ribbon.	GO:0032436|p	GO:0005737|	GO:0016874|lig	K10144
Manes.03G06480(	1.3068479	0.00848	up	no	.	pfam02845	CUE	CUE domai	.	.	.	.
Manes.03G06490(	13.686227	0.05066	up	no	.	pfam02687	FtsX	FtsX-like pe	.	.	.	.
Manes.03G06540(	0.503793	9.67E-08	down	no	Pentatricopep	pfam13041	PPR_2	PPR repeat	GO:0009788|n	GO:0005829|	.	.
Manes.03G06550(	0.5826083	3.27E-07	down	no	Protein TIFY	pfam06200	tify	tify domain.	.	GO:0005634|	.	.
Manes.03G06570(	1.0537723	0.8477	up	no	.	pfam03181	BURP	BURP doma	.	.	.	.
Manes.03G06630(	1.0188992	0.91691	up	no	Dehydration-	pfam03181	BURP	BURP doma	GO:0009651|r	GO:0005768|	.	.

Manes.03G06500(	0.8076529	0.10534	down	no	Tudor domai	pfam08585	DUF1767	Domain of u	GO:0016568|c	GO:0005737|	GO:0003682|ch	K18404
Manes.03G06700(	1.4321822	0.00019	up	no	Probable AD	pfam01412	ArfGap	Putative GT	.	.	GO:0005096|G	.
Manes.03G06750(	0.7003893	0.08956	down	no	.	pfam05316	VAR1	Mitochondri	.	.	.	.
Manes.03G06780(	2.0573999	1.78E-09	up	yes	Leucine-rich	pfam13855	LRR_8	Leucine rich	GO:0071555|	GO:0005618|	GO:0005199|stı	.
Manes.03G06790(	0.5723572	1.24E-06	down	no	Serine/threon	pfam01603	B56	Protein phos	GO:0007165|	GO:0000159|	GO:0008601|pr	K11584
Manes.03G06810(	Inf	2.55E-06	up	yes	Protein YLS3	pfam14368	LTP_2	Probable lip	GO:0006869|	GO:0031225|	GO:0008289|lip	.
Manes.03G06820(	0.9272759	0.4529	down	no	DnaJ homolo	pfam00226	DnaJ	DnaJ domai	GO:0009660|	GO:0005783|	.	K09533
Manes.03G06930(	1.2441546	0.01588	up	no	Lecithin-chol	pfam02450	LCAT	Lecithin:cho	GO:0009395|	GO:0005829|	GO:0008374|O	.
Manes.03G07050(	0.3712011	9.49E-12	down	yes	Patatin-like p	pfam01734	Patatin	Patatin-like	GO:0019374|	GO:0005886|	GO:0047617|ac	.
Manes.03G07060(	0.7031516	0.13844	down	no	.	pfam08724	Rep_N	Rep protein	.	.	.	.
Manes.03G07100(	0.7690938	0.07037	down	no	Phosphatidat	pfam09139	Mmp37	Mitochondri	GO:0032049|	GO:0031314|	GO:0004605|ph	K17807
Manes.03G07110(	1.0254173	0.96909	up	no	Mitochondria	pfam03476	MOSC_N	MOSC N-te	GO:0042126|	GO:0070062|	GO:0030151|m	.
Manes.03G07120(	1.1870252	0.05804	up	no	.	pfam00856	SET	SET domair	.	.	.	.
Manes.03G07150(	0.3434833	1.02E-09	down	yes	Pentatricope	pfam14432	DYW_deaminase	DYW family	GO:0031425|	GO:0009507|	.	.
Manes.03G07160(	0.6017732	0.00014	down	no	.	pfam12854	PPR_1	PPR repeat.	.	.	.	.
Manes.03G07220(	3.3428426	5.01E-22	up	yes	LRR recepto	pfam00069	Pkinase	Protein kina	GO:0009793|	GO:0016021|	GO:0005524|A	.
Manes.03G07260(	1.0573304	0.63404	up	no	Probable GM	pfam03352	Adenine_glyco	Methyladeni	GO:0006529|	.	GO:0004066|as	K03790
Manes.03G07270(	0.9318684	0.48272	down	no	.	pfam01697	Glyco_transf_92	Glycosyltrar	.	.	.	.
Manes.03G07290(	2.0885777	0.1628	up	no	.	pfam02790	COX2_TM	Cytochrome	.	.	.	.
Manes.03G07300(	1.0874837	0.36885	up	no	DNA-directe	pfam04997	RNA_pol_Rpb1_1	RNA polym	GO:0006351|	GO:0009507|	GO:0003677|D	K03046
Manes.03G07330(	0.4276837	3.27E-10	down	yes	Potassium tra	pfam02705	K_trans	K+ potassiu	.	GO:0016021|	GO:0015079|pc	.
Manes.03G07370(	#NAME?	0.06869	down	no	.	pfam02724	CDC45	CDC45-like	.	.	.	.
Manes.03G07420(	0.605029	0.00019	down	no	50S ribosoma	pfam00542	Ribosomal_L12	Ribosomal p	GO:0006412|	GO:0005840|	GO:0003735|stı	.
Manes.03G07450(	25.807653	8.45E-15	up	yes	.	pfam05678	VQ	VQ motif. T	.	.	.	.
Manes.03G07460(	8.5793587	0.02062	up	yes	Fasciclin-like	pfam02469	Fasciclin	Fasciclin do	GO:0009834|	GO:0031225|	.	.
Manes.03G07490(	0.8399245	0.08787	down	no	DNA polyme	pfam00817	IMS	impB/mucB	GO:0006260|	GO:0005634|	GO:0003684|da	K03509
Manes.03G07500(	2.0996923	1.76E-10	up	yes	UDP-glucose	pfam00201	UDPGT	UDP-glucor	.	.	GO:0047893|fla	.
Manes.03G07520(	1.6475016	2.42E-06	up	no	GTP-binding	pfam01926	MMR_HSR1	50S riboson	.	GO:0005622|	GO:0005525|G	.
Manes.03G07590(	1.1896337	0.28457	up	no	Polygalacturc	pfam13229	Beta_helix	Right hande	GO:0071555|	GO:0005618|	GO:0004650|pc	.
Manes.03G07660(	2.7624886	5.89E-29	up	yes	Probable LRI	pfam11721	Malectin	Di-glucose l	.	GO:0016021|	GO:0005524|A	.
Manes.03G07710(	1.6497123	3.56E-08	up	no	Ras-related p	pfam00071	Ras	Ras family.	GO:0006886|	GO:0030139|	GO:0005525|G	.
Manes.03G07740(	0.5303925	5.52E-06	down	no	Putative dise	pfam00931	NB-ARC	NB-ARC dc	GO:0006952|	.	GO:0043531|A	.
Manes.03G07760(	0.1853163	1.31E-13	down	yes	.	pfam04788	DUF620	Protein of u	.	.	.	.
Manes.03G07770(	0.3833146	6.71E-07	down	yes	Myb family tı	pfam14379	Myb_CC_LHEQL	MYB-CC ty	GO:0010088|	GO:0005634|	GO:0003677|D	.
Manes.03G07800(	0.4844751	0.00052	down	yes	NuA3 HAT c	pfam13832	zf-HC5HC2H_2	PHD-zinc-fi	GO:0016573|	GO:0005737|	GO:0035064|m	K11380
Manes.03G07880(	2.6840239	8.02E-07	up	yes	Bidirectional	pfam03083	MtN3_slv	Sugar efflux	GO:0034219|	GO:0005887|	GO:0051119|su	K15382
Manes.03G07890(	2.2872002	3.02E-20	up	yes	Trifunctional	pfam01370	Epimerase	NAD depen	GO:0010315|	GO:0005829|	GO:0050662|co	K12450
Manes.03G07930(	0.198737	7.36E-07	down	yes	E3 ubiquitin-	pfam13923	zf-C3HC4_2	Zinc finger,	GO:0051026|	GO:0005712|	GO:0016874|lig	K10639
Manes.03G07970(	0.230111	0.47861	down	no	Probable xylo	pfam00722	Glyco_hydro_16	Glycosyl hy	GO:0042546|	GO:0048046|	GO:0004553|hy	K08235
Manes.03G08020(	0.2401871	6.86E-05	down	yes	.	pfam12937	F-box-like	F-box-like.	.	.	.	.
Manes.03G08100(	1.5317327	1.51E-05	up	no	Synaptotagm	pfam00168	C2	C2 domain.	GO:0009306|	GO:0005783|	GO:0046872|m	.
Manes.03G08120(	1.1633641	0.11943	up	no	Delta(24)-ste	pfam01565	FAD_binding_4	FAD binding	.	GO:0016021|	GO:0050614|de	.
Manes.03G08130(	0.8355617	0.05916	down	no	Calcium-tran	pfam00122	E1-E2_ATPase	E1-E2 ATP	GO:0009555|	GO:0016021|	GO:0005524|A	K01537
Manes.03G08140(	0.9287402	0.44429	down	no	Calcium-tran	pfam00689	Cation_ATPase_C	Cation trans	GO:0009555|	GO:0016021|	GO:0005524|A	K01537
Manes.03G08170(	0.7105652	0.00388	down	no	RNA-binding	pfam00076	RRM_1	RNA recogi	.	.	GO:0003676|nu	.
Manes.03G08180(	1.2477596	0.01509	up	no	ACT domain	pfam13291	ACT_4	ACT domai	.	GO:0009507|	.	.
Manes.03G08250(	0.5347066	4.21E-07	down	no	DNA repair p	pfam08423	Rad51	Rad51. Rad	GO:0000724|	GO:0033063|	GO:0005524|A	K10879
Manes.03G08280(	1.2910208	0.75207	up	no	NAC domain	pfam02365	NAM	No apical m	GO:0009734|	GO:0005634|	GO:0003677|D	.
Manes.03G08290(	0.9808344	0.84905	down	no	Photosystem	pfam04725	PsbR	Photosysten	GO:0015979|	GO:0009535|	.	.
Manes.03G08300(	0.6337735	0.00226	down	no	.	pfam13450	NAD_binding_8	NAD(P)-bin	.	.	.	.
Manes.03G08340(	0.4218589	2.76E-07	down	yes	.	pfam13041	PPR_2	PPR repeat	.	.	.	.
Manes.03G08350(	17.108167	3.27E-10	up	yes	9-cis-epoxyca	pfam03055	RPE65	Retinal pign	GO:0009688|	GO:0009570|	GO:0045549|9-	K09840
Manes.03G08390(	1.2399138	0.03465	up	no	Solanesyl dip	pfam00348	polyprenyl_synt	Polyprenyl s	GO:0009793|	GO:0009507|	GO:0052924|all	K14066
Manes.03G08400(	0.9197714	0.91712	down	no	Cytochrome	pfam00067	p450	Cytochrome	.	GO:0016021|	GO:0020037|he	.
Manes.03G08410(	1.1984203	0.04945	up	no	Cytochrome	pfam00067	p450	Cytochrome	.	GO:0016021|	GO:0020037|he	.
Manes.03G08430(	19.211688	1.01E-07	up	yes	Peroxidase N	pfam00141	peroxidase	Peroxidase.	GO:0042744|	GO:0005576|	GO:0020037|he	.
Manes.03G08440(	Inf	2.90E-29	up	yes	Solanesyl dip	pfam01824	MatK_N	MatK/TrnK	GO:0008299|	GO:0009507|	GO:0052924|all	K05356
Manes.03G08450(	0.8120632	0.04849	down	no	.	pfam13385	Laminin_G_3	Concanavali	.	.	.	.
Manes.03G08460(	0.7626841	0.04097	down	no	.	pfam12783	Sec7_N	Guanine nuc	.	.	.	.
Manes.03G08480(	1.4455918	3.83E-05	up	no	Serine/threon	pfam00149	Metallophos	Calcineurin-	.	GO:0005737|	GO:0046872|m	K04382
Manes.03G08550(	0.6467938	0.03539	down	no	Mechanosens	pfam00924	MS_channel	Mechanoser	GO:0006811|	GO:0016021|	.	.
Manes.03G08560(	7.9536431	3.48E-64	up	yes	B-box zinc fi	pfam00643	zf-B_box	B-box zinc f	GO:0006355|	GO:0005634|	GO:0008270|zi	.
Manes.03G08570(	Inf	0.9009	up	no	.	pfam01955	CbiZ	Adenosylcol	.	.	.	.
Manes.03G08670(	0.6438045	8.67E-06	down	no	Chaperonin 6	pfam00118	Cpn60_TCP1	TCP-1/cpn6	GO:0042026|	GO:0009507|	GO:0005524|A	K04077
Manes.03G08680(	0.5798127	0.0001	down	no	Putative RNA	pfam02590	SPOUT_MTase	Predicted SI	GO:0006364|	GO:0005737|	GO:0008168|m	K00783
Manes.03G08720(	17.12283	0.03079	up	no	.	pfam09772	Tmem26	Transmembi	.	.	.	.
Manes.03G08730(	1.2075663	0.03659	up	no	Lysine--tRNA	pfam00152	tRNA-synt_2	tRNA synth	GO:0006430|	GO:0005737|	GO:0005524|A	K04567
Manes.03G08740(	0.6110682	0.01324	down	no	.	pfam07263	DMP1	Dentin matr	.	.	.	.
Manes.03G08750(	0.6527428	2.00E-05	down	no	.	pfam03080	DUF239	Domain of u	.	.	.	.
Manes.03G08790(	0.9010187	0.3943	down	no	Probable acyl	pfam00501	AMP-binding	AMP-bindin	GO:0009850|	GO:0005777|	GO:0016874|lig	.
Manes.03G08810(	0.8589075	0.20322	down	no	Probable acyl	pfam00501	AMP-binding	AMP-bindin	GO:0009850|	GO:0005777|	GO:0016874|lig	.
Manes.03G08830(	0.9276865	0.50761	down	no	.	pfam04398	DUF538	Protein of u	.	.	.	.
Manes.03G08850(	84.439503	2.49E-13	up	yes	.	pfam04819	DUF716	Family of ur	.	.	.	.
Manes.03G08880(	1.6630587	3.91E-07	up	no	Dirigent prot	pfam03018	Dirigent	Dirigent-like	.	GO:0048046|	.	.
Manes.03G08900(	1.420765	0.18448	up	no	.	pfam14296	O-ag_pol_Wzy	O-antigen p	.	.	.	.
Manes.03G08920(	0.9221151	0.41091	down	no	.	pfam00684	DnaJ_CXXCXGX	DnaJ centra	.	.	.	.
Manes.03G08930(	1.7194605	1.65E-06	up	no	Uncharacteri	pfam01926	MMR_HSR1	50S riboson	GO:0032297|	.	GO:0005525|G	K06948
Manes.03G08950(	0.2868378	1.97E-16	down	yes	E3 ubiquitin-	pfam03145	Sina	Seven in abs	GO:0007275|	GO:0005634|	GO:0016874|lig	K04506
Manes.03G08960(	1.1291403	0.20843	up	no	Formate--teti	pfam01268	FTHFS	Formate--te	GO:0009396|	.	GO:0005524|A	.
Manes.03G09030(	0.4165821	1.78E-07	down	yes	Putative E3 u	pfam04564	U-box	U-box doma	GO:0009877|	.	GO:0016874|lig	.
Manes.03G09070(	0.7456709	0.02406	down	no	CASP-like pr	pfam04535	DUF588	Domain of u	.	GO:0016021|	.	.
Manes.03G09090(	2.897909	5.90E-12	up	yes	Kinesin-like p	pfam00225	Kinesin	Kinesin mot	GO:0090136|	GO:0005813|	GO:0005524|A	K10406
Manes.03G09100(	226.9765	3.68E-32	up	yes	Gibberellin 2	pfam03171	2OG-FeII_Oxy	2OG-Fe(II)	GO:0009686|	.	GO:0045543|gil	.
Manes.03G09110(	79.656941	0.00036	up	yes	Receptor-like	pfam13855	LRR_8	Leucine rich	GO:0009755|	GO:0016021|	GO:0004672|pr	.
Manes.03G09150(	0.1476507	2.19E-27	down	yes	BES1/BZR1	pfam05687	DUF822	Plant protei	GO:0006355|	.	GO:0003677|D	.
Manes.03G09170(	1.0595665	0.56867	up	no	.	pfam12864	DUF3822	Protein of u	.	.	.	.
Manes.03G09190(	2.8749295	3.28E-33	up	yes	Probable LRI	pfam11721	Malectin	Di-glucose l	.	GO:0016021|	GO:0005524|A	.
Manes.03G09200(	4.3243862	7.41E-62	up	yes	Probable leuc	pfam11721	Malectin	Di-glucose l	.	GO:0016021|	GO:0005524|A	.

Manes.03G09240(	1.366842	0.0138	up	no	UDP-glycosy	pfam00201	UDPGT	UDP-glucor	.	.	GO:0035251|U	.
Manes.03G09300(	0.6606781	0.01244	down	no	Putative pent	pfam13041	PPR_2	PPR repeat	.	GO:0005739|	.	.
Manes.03G09370(	0.6797048	0.00751	down	no	Pentatricopep	pfam13041	PPR_2	PPR repeat	.	GO:0005739|	.	.
Manes.03G09470(	0.7228005	0.02513	down	no	Peptide chain	pfam03462	PCRF	PCRF doma	GO:0009658|	GO:0009507|	GO:0003730|m	.
Manes.03G09530(	0.7914022	0.02165	down	no	.	pfam10513	EPL1	Enhancer of	.	.	.	.
Manes.03G09570(	1.7097998	2.49E-05	up	no	50S ribosoma	pfam00453	Ribosomal_L20	Ribosomal p	GO:0000027|	GO:0005840|	GO:0019843|rR	K02887
Manes.03G09610(	0.5457503	8.68E-06	down	no	Phototropin-2	pfam00069	Pkinase	Protein kina	GO:0009785|	GO:0005737|	GO:0005524|A	.
Manes.03G09640(	0.2213346	6.98E-07	down	yes	.	pfam13456	RVT_3	Reverse trar	.	.	.	.
Manes.03G09670(	0.9892814	0.93198	down	no	Brefeldin A-i	pfam01369	Sec7	Sec7 domai	GO:0015031|	GO:0005829|	GO:0005086|A	K18442
Manes.03G09680(	0.8701619	0.17789	down	no	Aminoacylase	pfam01546	Peptidase_M20	Peptidase fa	GO:0006520|	GO:0005737|	GO:0004046|an	K14677
Manes.03G09690(	0.8960061	0.25661	down	no	Aminoacylase	pfam01546	Peptidase_M20	Peptidase fa	GO:0006520|	GO:0005737|	GO:0004046|an	K14677
Manes.03G09700(	0.7423604	0.04091	down	no	.	pfam10601	zf-LITAF-like	LITAF-like	.	.	.	.
Manes.03G09720(	2.2664822	9.55E-05	up	yes	Xylem cystei	pfam00112	Peptidase_C1	Papain famil	GO:0010623|	GO:0005615|	GO:0004197|cy	K16290
Manes.03G09730(	3.3807496	1.83E-23	up	yes	Calcium-depe	pfam00069	Pkinase	Protein kina	.	GO:0016020|	GO:0005524|A	K13412
Manes.03G09750(	1.7684897	2.95E-06	up	no	Monosacchar	pfam00083	Sugar_tr	Sugar (and	GO:0008643|	GO:0005794|	GO:0005355|gl	.
Manes.03G09760(	0.3510916	9.09E-23	down	yes	Glutamate re	pfam01094	ANF_receptor	Receptor fa	GO:0070588|	GO:0016021|	GO:0005262|ca	.
Manes.03G09770(	4.0566307	3.30E-45	up	yes	Mitogen-acti	pfam00069	Pkinase	Protein kina	GO:0000186|	GO:0005737|	GO:0005524|A	K13413
Manes.03G09780(	0.7069964	0.00041	down	no	Retinoblastor	pfam01858	RB_A	Retinoblasto	GO:0007049|	GO:0005634|	.	K04681
Manes.03G09790(	0.8546562	0.12765	down	no	Mitochondria	pfam00153	Mito_carr	Mitochondr	GO:0006835|	GO:0005618|	GO:0005310|di	K15104
Manes.03G09810(	1.9477886	6.23E-13	up	no	Tubulin alpha	pfam03953	Tubulin_C	Tubulin C-te	GO:0007017|	GO:0005737|	GO:0005525|G	K07374
Manes.03G09840(	22.950665	2.69E-07	up	yes	.	pfam14111	DUF4283	Domain of u	.	.	.	.
Manes.03G09850(	1.6520213	3.70E-08	up	no	Importin-5	pfam13646	HEAT_2	HEAT repea	GO:0071230|	GO:0005737|	GO:0044822|pc	.
Manes.03G09860(	0.5125872	1.82E-06	down	no	Peptidyl-tRN	pfam01195	Pept_tRNA_hydro	Peptidyl-tRN	GO:0006397|	GO:0005739|	GO:0004045|an	K01056
Manes.03G09880(	1.3446388	0.00262	up	no	DNA polyme	pfam04042	DNA_pol_E_B	DNA polym	GO:0006261|	GO:0008622|	GO:0003677|D	K02325
Manes.03G09920(	NA	NA	--	no	ATP-depende	pfam03730	Ku_C	Ku70/Ku80	GO:0006310|	GO:0005737|	GO:0005524|A	K10884
Manes.03G09960(	0.5576755	1.29E-08	down	no	Gamma-gluta	pfam07722	Peptidase_C26	Peptidase C	GO:0006541|	GO:0005618|	GO:0034722|ga	.
Manes.03G09970(	1.11748	0.30678	up	no	DNA polyme	pfam09507	CDC27	DNA polym	GO:0006260|	GO:0005739|	GO:0003887|D	.
Manes.03G09980(	0.9274267	0.53072	down	no	Ubiquitin dor	pfam10011	DUF2254	Predicted m	.	GO:0005737|	.	.
Manes.03G09990(	0.7684594	0.01187	down	no	.	pfam01436	NHL	NHL repeat	.	.	.	.
Manes.03G10020(	2.0334727	0.00137	up	yes	Mitochondria	pfam02466	Tim17	Tim17/Tim2	GO:0009793|	GO:0016021|	.	K17790
Manes.03G10040(	1.0710324	0.49074	up	no	Alpha-1,3/1,6	pfam00534	Glycos_transf_1	Glycosyl tra	GO:0097502|	GO:0016021|	GO:0004378|G	K03843
Manes.03G10060(	1.1709711	0.19469	up	no	Tubby-like F	pfam01167	Tub	Tub family.	GO:0009555|	GO:0005829|	GO:0035091|ph	.
Manes.03G10070(	0.6604651	0.4105	down	no	.	pfam15245	VGLL4	Transcriptio	.	.	.	.
Manes.03G10080(	0.729889	0.05077	down	no	.	pfam02537	CRCB	CrcB-like p	.	.	.	.
Manes.03G10110(	Inf	0.1623	up	no	Olee1-like pr	pfam01190	Pollen_Ole_e_I	Pollen prote	.	GO:0005615|	.	.
Manes.03G10160(	0.4931581	0.00045	down	yes	.	pfam13595	DUF4138	Domain of u	.	.	.	.
Manes.03G10180(	0.4041368	5.20E-07	down	yes	.	pfam09753	Use1	Membrane f	.	.	.	.
Manes.03G10230(	102.3437	3.67E-16	up	yes	.	pfam04398	DUF538	Protein of u	.	.	.	.
Manes.03G10280(	Inf	0.04502	up	no	.	pfam02995	DUF229	Protein of u	.	.	.	.
Manes.03G10360(	1.8065049	9.27E-07	up	no	.	pfam08744	NOZZLE	Plant transc	.	.	.	.
Manes.03G10380(	137.14098	7.91E-20	up	yes	Basic leucine	pfam00170	bZIP_1	bZIP transc	GO:0045893|	GO:0005634|	GO:0003677|D	.
Manes.03G10390(	2.2520645	9.98E-16	up	yes	Homogentisa	pfam04209	HgmA	homogentisa	GO:1902000|	GO:0005829|	GO:0004411|ho	K00451
Manes.03G10420(	0.6392676	0.00043	down	no	RNA exonuc	pfam00929	RNase_T	Exonuclease	GO:0006355|	GO:0005730|	GO:0004527|ex	K18327
Manes.03G10440(	Inf	0.1623	up	no	UDP-glycosy	pfam00201	UDPGT	UDP-glucor	GO:0009813|	GO:0043231|	GO:0080043|qu	.
Manes.03G10500(	9.9125345	5.93E-12	up	yes	Homeobox-le	pfam00046	Homeobox	Homeobox	GO:0009646|	GO:0005634|	GO:0043565|se	.
Manes.03G10520(	Inf	0.38692	up	no	.	pfam13639	zf-RING_2	Ring finger	.	.	.	.
Manes.03G10550(	2.240937	1.14E-05	up	yes	Probable ami	pfam00155	Aminotran_1_2	Aminotransf	GO:0006520|	.	GO:0004838|L-	K00815
Manes.03G10610(	3.97865	0.39698	up	no	Putative RIN	pfam13639	zf-RING_2	Ring finger	GO:0016567|	GO:0016021|	GO:0008270|zi	.
Manes.03G10620(	0.7476117	0.01572	down	no	.	pfam13041	PPR_2	PPR repeat	.	.	.	.
Manes.03G10630(	6.3433022	9.90E-95	up	yes	Ethylene-resp	pfam00847	AP2	AP2 domain	GO:0009873|	GO:0016604|	GO:0003677|D	K09286
Manes.03G10650(	2.0631953	3.78E-08	up	yes	PRA1 family	pfam03208	PRA1	PRA1 family	GO:0016192|	GO:0005783|	.	.
Manes.03G10670(	0.3347118	0.00012	down	yes	.	pfam05424	Duffy_binding	Duffy bindir	.	.	.	.
Manes.03G10750(	1.2845499	0.00872	up	no	Probable E3	pfam01485	IBR	IBR domain	.	GO:0016021|	GO:0016874|lig	K11977
Manes.03G10800(	0.5699612	1.12E-07	down	no	Tetrapyrrole-	pfam05419	GUN4	GUN4-like.	GO:0015995|	GO:0009507|	GO:0019899|en	.
Manes.03G10820(	0.4591373	9.98E-14	down	yes	Exocyst comp	pfam03081	Exo70	Exo70 exoc	GO:0006887|	GO:0005829|	.	.
Manes.03G10910(	0.9958516	1	down	no	MATE efflux	pfam01554	MatE	MatE. The I	.	GO:0016021|	GO:0015297|an	K03327
Manes.03G10920(	4.1878306	2.29E-13	up	yes	Early nodulin	pfam02298	Cu_bind_like	Plastocyanir	.	GO:0032578|	GO:0009055|el	.
Manes.03G10930(	1.1735769	0.16625	up	no	Probable bifu	pfam00596	Aldolase_II	Class II Ald	GO:0019509|	GO:0005737|	GO:0043715|2,	K16054
Manes.03G10940(	1.7596086	5.72E-07	up	no	.	pfam05678	VQ	VQ motif. T	.	.	.	.
Manes.03G10960(	1.062624	0.49213	up	no	Proton pump	pfam05483	SCP-1	Synaptonem	GO:0010155|	GO:0005783|	.	.
Manes.03G10970(	0.6670243	0.35059	down	no	Xylosyltransf	pfam02485	Branch	Core-2/I-Br	GO:0030206|	GO:0005789|	GO:0008375|ac	K00771
Manes.03G10980(	0.3616806	5.15E-18	down	yes	Protein SPA1	pfam00400	WD40	WD domain	GO:0009585|	GO:0005634|	GO:0005524|A	.
Manes.03G11010(	0.7445763	0.02035	down	no	.	pfam04765	DUF616	Protein of u	.	.	.	.
Manes.03G11020(	NA	NA	--	no	.	pfam11957	efThoc1	THO compl	.	.	.	.
Manes.03G11060(	2.4063065	1.05E-19	up	yes	Probable ubi	pfam00179	UQ_con	Ubiquitin-co	GO:0000209|	GO:0005737|	GO:0005524|A	K10581
Manes.03G11140(	0.9739553	0.85731	down	no	E3 ubiquitin-	pfam13923	zf-C3HC4_2	Zinc finger,	.	GO:0005783|	GO:0016874|lig	K10666
Manes.03G11150(	9.1278974	0.12799	up	no	Expansin-A1	pfam01357	Pollen_allerg_1	Pollen allerg	GO:0009664|	GO:0005618|	.	.
Manes.03G11170(	0.7572483	0.05442	down	no	.	pfam07042	TrfA	TrfA proteir	.	.	.	.
Manes.03G11180(	1.4688862	0.00041	up	no	Pentatricopep	pfam13041	PPR_2	PPR repeat	.	GO:0005739|	.	.
Manes.03G11200(	0.3700191	4.29E-18	down	yes	Choline trans	pfam04515	Choline_transpo	Plasma-men	GO:0043123|	GO:0070062|	GO:0004871|sig	K15377
Manes.03G11220(	0.3154249	3.60E-11	down	yes	2-succinylber	pfam00501	AMP-binding	AMP-bindir	GO:0042372|	GO:0009507|	GO:0005524|A	K14760
Manes.03G11230(	0.573946	0.00083	down	no	.	pfam01638	HxlR	HxlR-like h	.	.	.	.
Manes.03G11290(	0.7502307	0.00813	down	no	.	pfam13855	LRR_8	Leucine rich	.	.	.	.
Manes.03G11300(	0.7507403	0.00424	down	no	Ultraviolet-B	pfam00415	RCC1	Regulator o	GO:0009649|	GO:0000785|	GO:0003682|ch	.
Manes.03G11330(	0.415676	1.50E-12	down	yes	Protochlorop	pfam00106	adh_short	short chain	GO:0015995|	GO:0009507|	GO:0016630|pr	K00218
Manes.03G11350(	3.5685574	2.27E-48	up	yes	.	pfam06749	DUF1218	Protein of u	.	.	.	.
Manes.03G11360(	1.3249311	0.00238	up	no	.	pfam13254	DUF4045	Domain of u	.	.	.	.
Manes.03G11420(	83.187641	8.12E-89	up	yes	NAC domain	pfam02365	NAM	No apical m	GO:0006355|	GO:0005634|	GO:0003677|D	.
Manes.03G11450(	1.9004203	7.36E-12	up	no	Peroxisomal	pfam04117	Mpv17_PMP22	Mpv17 / PM	.	GO:0016021|	.	K13347
Manes.03G11470(	0.7967588	0.01992	down	no	Trafficking p	pfam12584	TRAPPC10	Trafficking	GO:0034498|	GO:1990071|	.	.
Manes.03G11480(	0.3411087	9.57E-16	down	yes	Biotin biosyn	pfam08241	Methyltransf_11	Methyltrans	GO:0009102|	.	GO:0010340|ca	K02169
Manes.03G11490(	0.5008064	3.05E-05	down	no	Putative dise	pfam00931	NB-ARC	NB-ARC do	GO:0006952|	.	GO:0043531|A	.
Manes.03G11540(	Inf	0.9009	up	no	.	pfam06830	Root_cap	Root cap. T	.	.	.	.
Manes.03G11560(	0.5124723	6.29E-07	down	no	Lysine-specif	pfam01593	Amino_oxidase	Flavin conta	GO:0051568|	.	GO:0003677|D	K11450
Manes.03G11570(	0.7682578	0.02864	down	no	Uncharacteriz	pfam06838	Met_gamma_lyase	Methionine	.	.	GO:0003824|ca	.
Manes.03G11600(	0.7985502	0.10797	down	no	.	pfam05656	DUF805	Protein of u	.	.	.	.
Manes.03G11620(	0.8389915	0.18588	down	no	.	pfam08020	DUF1706	Protein of u	.	.	.	.
Manes.03G11630(	1.3117153	0.00314	up	no	Probable AD	pfam01412	ArfGap	Putative GT	GO:0090630|	GO:0005829|	GO:0005096|G	K12486

Manes.03G11640(	0.4269403	0.01437	down	yes	.	pfam05553	DUF761	Cotton fibre	.	.	.	.
Manes.03G11660(	0.6833798	0.00013	down	no	Myosin-2	pfam00063	Myosin_head	Myosin head	GO:0030048|	GO:0005768|	GO:0005524|A	K10357
Manes.03G11670(	0.6492991	0.00056	down	no	Chlorophyll a	pfam00504	Chloroa_b-bind	Chlorophyll	GO:0009765|	GO:0009535|	GO:0016168|ch	K08914
Manes.03G11680(	0.4327747	0.0012	down	yes	Cyclic nucleo	pfam00027	cNMP_binding	Cyclic nucle	GO:0009626|	GO:0005887|	GO:0030552|cA	.
Manes.03G11690(	0.7235672	0.07881	down	no	Dynein light	pfam01221	Dynein_light	Dynein light	GO:0007017|	GO:0005737|	GO:0003774|m	.
Manes.03G11720(	Inf	1.61E-07	up	yes	.	pfam12609	DUF3774	Wound-indu	.	.	.	.
Manes.03G11730(	5.0622322	2.33E-32	up	yes	.	pfam12609	DUF3774	Wound-indu	.	.	.	.
Manes.03G11750(	0.9387428	0.97825	down	no	Transcription	pfam00249	Myb_DNA-binding	Myb-like DI	GO:0006355|	GO:0005634|	GO:0003677|DI	K09422
Manes.03G11780(	6.4680247	0.01614	up	yes	.	pfam02867	Ribonuc_red_lgC	Ribonucleot	.	.	.	.
Manes.03G11790(	5.8222992	8.79E-84	up	yes	B-box zinc fi	pfam00643	zf-B_box	B-box zinc f	GO:0009785|	GO:0005737|	GO:0046872|m	.
Manes.03G11810(	2.7654147	9.67E-21	up	yes	G-type lectin	pfam07714	Pkinase_Tyr	Protein tyro	GO:0048544|	.	GO:0005524|A	.
Manes.03G11830(	23.866299	#######	up	yes	Calcium-bind	pfam13833	EF-hand_8	EF-hand do	GO:0009733|	.	GO:0005509|ca	.
Manes.03G11850(	0.4574391	0.05347	down	no	G-type lectin	pfam07714	Pkinase_Tyr	Protein tyro	GO:0048544|	.	GO:0005524|A	.
Manes.03G11860(	0.3912908	0.02759	down	yes	G-type lectin	pfam00954	S_locus_glycop	S-locus glyc	GO:0048544|	.	GO:0005524|A	.
Manes.03G11870(	NA	NA	--	no	G-type lectin	pfam07714	Pkinase_Tyr	Protein tyro	GO:0048544|	.	GO:0005524|A	.
Manes.03G11920(	Inf	0.35135	up	no	.	pfam02671	PAH	Paired amph	.	.	.	.
Manes.03G11940(	0.5752824	0.0012	down	no	G-type lectin	pfam07714	Pkinase_Tyr	Protein tyro	GO:0048544|	.	GO:0005524|A	.
Manes.03G11990(	0.3837179	5.34E-05	down	yes	.	pfam14656	RAB3GAP2_C	Rab3 GTPa	.	.	.	.
Manes.03G12060(	1.7615845	2.37E-10	up	no	Glycine-rich	pfam00076	RRM_1	RNA recogn	GO:0080156|	GO:0005739|	GO:0005524|A	K12741
Manes.03G12080(	4.6368743	2.92E-57	up	yes	Transcription	pfam00010	HLH	Helix-loop-l	GO:0006355|	GO:0005634|	GO:0003677|DI	.
Manes.03G12090(	Inf	0.00699	up	yes	.	pfam12717	Cnd1	non-SMC m	.	.	.	.
Manes.03G12100(	5.1517258	3.96E-36	up	yes	Chitin-induci	pfam03514	GRAS	GRAS doma	GO:0006355|	GO:0005634|	.	.
Manes.03G12150(	0.4035902	1.75E-10	down	yes	.	pfam02931	Neur_chan_LBD	Neurotransr	.	.	.	.
Manes.03G12170(	Inf	0.00421	up	yes	RING-H2 fin	pfam13639	zf-RING_2	Ring finger	GO:0016567|	GO:0016021|	GO:0008270|zi	.
Manes.03G12180(	14.588383	0.00654	up	yes	.	pfam00858	ASC	Amiloride-s	.	.	.	.
Manes.03G12190(	0.6244625	3.51E-05	down	no	Formin-like p	pfam02181	FH2	Formin Hon	GO:0030041|	GO:0005618|	GO:0003779|ac	.
Manes.03G12200(	0.7711726	0.32429	down	no	21.7 kDa clas	pfam00011	HSP20	Hsp20/alpha	.	GO:0005737|	.	.
Manes.03G12210(	3.3597389	3.38E-35	up	yes	Transcription	pfam00010	HLH	Helix-loop-l	GO:0006355|	GO:0005634|	GO:0003677|DI	.
Manes.03G12220(	1.603072	4.23E-05	up	no	.	pfam09353	DUF1995	Domain of u	.	.	.	.
Manes.03G12250(	0.7990262	0.0221	down	no	Helicase prot	pfam00176	SNF2_N	SNF2 family	GO:0006342|	GO:0005634|	GO:0051537|2	.
Manes.03G12280(	0.6176932	1.22E-06	down	no	.	pfam13306	LRR_5	Leucine rich	.	.	.	.
Manes.03G12340(	2.6982701	0.06135	up	no	2S albumin	pfam00234	Tryp_alpha_amyl	Protease inh	.	.	GO:0045735|nu	.
Manes.03G12370(	1.8544438	4.16E-10	up	no	Protein transl	pfam01253	SUI1	Translation	GO:0006417|	.	GO:0003743|tra	K03113
Manes.03G12380(	4.0151195	6.21E-05	up	yes	Thiamine thia	pfam01946	Thi4	Thi4 family.	GO:0006950|	GO:0009507|	GO:0046872|m	K03146
Manes.03G12390(	21.385908	#######	up	yes	.	pfam09756	DDRGK	DDRGK do	.	.	.	.
Manes.03G12420(	0.8169143	0.03471	down	no	Purple acid p	pfam00149	Metallophos	Calcineurin-	GO:0016311|	GO:0005576|	GO:0003993|ac	.
Manes.03G12430(	0.6920561	0.01911	down	no	CRS2-associa	pfam01985	CRS1_YhbY	CRS1 / Yhb	GO:0006397|	GO:0005739|	GO:0003723|RI	.
Manes.03G12440(	5.4998711	6.34E-05	up	yes	.	pfam03942	DTW	DTW doma	.	.	.	.
Manes.03G12500(	1.0407872	0.69408	up	no	.	pfam01190	Pollen_Ole_e_I	Pollen prote	.	.	.	.
Manes.03G12510(	0.4722422	1.41E-08	down	yes	Peptidyl-prol	pfam00160	Pro_isomerase	Cyclophilin	GO:0006457|	GO:0005737|	GO:0042277|pe	K12737
Manes.03G12520(	0.6572255	0.00454	down	no	Dehydration-	pfam03181	BURP	BURP doma	GO:0009651|	GO:0005768|	.	.
Manes.03G12540(	0.3340939	0.00157	down	yes	Probable LRI	pfam00069	Pkinase	Protein kina	.	GO:0016021|	GO:0005524|A	.
Manes.03G12570(	0.5122379	8.51E-07	down	no	Dehydration-	pfam03181	BURP	BURP doma	GO:0009651|	GO:0005768|	.	.
Manes.03G12580(	0.317542	4.64E-07	down	yes	Dehydration-	pfam03181	BURP	BURP doma	GO:0009651|	GO:0005768|	.	.
Manes.03G12610(	Inf	0.1623	up	no	Dehydration-	pfam03181	BURP	BURP doma	GO:0009651|	GO:0005768|	.	.
Manes.03G12670(	0.5268172	4.38E-06	down	no	.	pfam04824	Rad21_Rec8	Conserved r	.	.	.	.
Manes.03G12680(	0.1193042	2.87E-05	down	yes	.	pfam04824	Rad21_Rec8	Conserved r	.	.	.	.
Manes.03G12700(	0.6317145	4.32E-06	down	no	Protein LHCI	pfam13857	Ank_5	Ankyrin rep	GO:0090391|	GO:0009507|	.	.
Manes.03G12750(	0.8758283	0.19073	down	no	FHA domain-	pfam00498	FHA	FHA domai	GO:0008283|	GO:0009507|	GO:0003723|RI	K13108
Manes.03G12760(	1.4342383	5.31E-05	up	no	Tryptophan s	pfam00291	PALP	Pyridoxal-pl	GO:0000162|	GO:0009941|	GO:0030170|py	K01696
Manes.03G12790(	1.1242015	0.22645	up	no	Protein disulf	pfam07970	COPIIcoated_ERV	Endoplasmic	GO:0045454|	GO:0005623|	.	.
Manes.03G12840(	2.0939369	0.02113	up	yes	Probable dise	pfam00931	NB-ARC	NB-ARC do	GO:0006952|	.	GO:0043531|Al	.
Manes.03G12870(	1.9032522	2.40E-09	up	no	Polyadenylate	pfam14438	SM-ATX	Ataxin 2 SM	GO:0010603|	GO:0010494|	GO:0044822|pc	.
Manes.03G12890(	0.7949881	0.02113	down	no	Heat shock 7	pfam00012	HSP70	Hsp70 prote	.	.	GO:0005524|A	K09489
Manes.03G12900(	2.2846081	2.53E-08	up	yes	.	pfam11112	PyocinActivator	Pyocin activ	.	.	.	.
Manes.03G12910(	12.552401	#######	up	yes	.	pfam14009	DUF4228	Domain of u	.	.	.	.
Manes.03G12930(	0.3630039	1.19E-05	down	yes	NEP1-interac	pfam13639	zf-RING_2	Ring finger	.	GO:0009535|	GO:0008270|zi	.
Manes.03G12940(	1.0225995	0.81885	up	no	Serine/argini	pfam00076	RRM_1	RNA recogn	GO:0006397|	GO:0016607|	GO:0003676|nu	.
Manes.03G12960(	18.649318	#######	up	yes	Protein transl	pfam01253	SUI1	Translation	GO:0006417|	.	GO:0003743|tra	K03113
Manes.03G12980(	1.3374223	0.59737	up	no	Aconitate hyd	pfam00330	Aconitase	Aconitase fa	GO:0006097|	GO:0005737|	GO:0051539|4	.
Manes.03G12990(	1.11897	0.33145	up	no	.	pfam05071	NDUFA12	NADH ubiq	.	.	.	.
Manes.03G13010(	3.2865568	6.92E-26	up	yes	.	pfam04520	Senescence_reg	Senescence	.	.	.	.
Manes.03G13020(	Inf	0.1623	up	no	.	pfam00542	Ribosomal_L12	Ribosomal p	.	.	.	.
Manes.03G13030(	0.2959045	1.92E-13	down	yes	Geranylgeran	pfam00348	polyprenyl_synt	Polyprenyl s	GO:0016117|	GO:0009507|	GO:0004161|di	K13789
Manes.03G13080(	1.3046199	0.005	up	no	Autophagy-re	pfam02991	Atg8	Autophagy p	GO:0006914|	GO:0000421|	.	.
Manes.03G13140(	0.3109184	1.77E-21	down	yes	GDSL estera	pfam00657	Lipase_GDSL	GDSL-like l	GO:0016042|	GO:0005576|	GO:0016298|lip	.
Manes.03G13150(	0.550789	0.0012	down	no	DNA mismat	pfam00488	MutS_V	MutS doma	GO:0051026|	GO:0000794|	GO:0005524|A	K08741
Manes.03G13160(	0.7550535	0.02454	down	no	Rab3 GTPase	pfam13890	Rab3-GTPase_cat	Rab3 GTPa	GO:0007420|	GO:0005737|	GO:0005096|G	K18270
Manes.03G13210(	1.158122	0.10744	up	no	Aspartic prot	pfam00026	Asp	Eukaryotic a	GO:0006629|	GO:0005829|	GO:0004190|as	K08245
Manes.03G13220(	1.0976156	1	up	no	WRKY trans	pfam03106	WRKY	WRKY DN.	GO:0080169|	GO:0005634|	GO:0043565|se	.
Manes.03G13230(	5.0118876	1.10E-05	up	yes	Mitogen-acti	pfam00069	Pkinase	Protein kina	GO:0043622|	GO:0005737|	GO:0005524|A	.
Manes.03G13240(	1.0362322	0.74697	up	no	Small ubiquit	pfam11976	Rad60-SLD	Ubiquitin-2	.	GO:0005737|	.	K12160
Manes.03G13280(	9.63037	0.00988	up	yes	.	pfam04885	Stig1	Stigma-spec	.	.	.	.
Manes.03G13320(	1.0729123	0.50424	up	no	Cell different	pfam04078	Rcd1	Cell differen	GO:0031047|	GO:0030014|	.	K12606
Manes.03G13330(	1.7104251	2.34E-08	up	no	1,4-alpha-glu	pfam00128	Alpha-amylase	Alpha amyla	GO:0005975|	GO:0009501|	GO:0003844|1,	K00700
Manes.03G13380(	NA	NA	--	no	.	pfam04885	Stig1	Stigma-spec	.	.	.	.
Manes.03G13450(	0.9644063	0.87318	down	no	Pirin-like pro	pfam02678	Pirin	Pirin. This fa	.	GO:0005634|	.	K06911
Manes.03G13460(	2.6081986	2.34E-23	up	yes	Glycerophosp	pfam03009	GDPD	Glycerophos	GO:0006071|	GO:0031225|	GO:0008889|gl	.
Manes.03G13490(	0.3995079	7.00E-07	down	yes	Probable long	pfam13813	MBOAT_2	Membrane b	GO:0006629|	GO:0016021|	GO:0047196|lo	.
Manes.03G13530(	0.9921545	0.94655	down	no	DNA topoiso	pfam02919	Topoisom_I_N	Eukaryotic I	GO:0006265|	GO:0005694|	GO:0003677|DI	.
Manes.03G13550(	0.7895986	0.01806	down	no	Cell division	pfam12327	FtsZ_C	FtsZ family,	GO:0007049|	GO:0009507|	GO:0005525|G	K03531
Manes.03G13590(	NA	NA	--	no	.	pfam00223	PsaA_PsaB	Photosysten	.	.	.	.
Manes.03G13600(	1.3110758	0.03134	up	no	Putative BPI/	pfam01273	LBP_BPI_CETP	LBP / BPI /	.	GO:0005615|	GO:0008289|lip	.
Manes.03G13620(	4.2409403	0.00651	up	yes	.	pfam04054	Not1	CCR4-Not c	.	.	.	.
Manes.03G13630(	1.4311369	0.0001	up	no	Vacuolar pro	pfam04652	DUF605	Vta1 like. V	GO:0015031|	GO:0010008|	.	.
Manes.03G13650(	0.7690589	0.08603	down	no	Phosphatidat	pfam01148	CTP_transf_1	Cytidylyltran	GO:0016024|	GO:0016021|	GO:0004605|ph	K00981
Manes.03G13680(	1.6758165	2.67E-07	up	no	Protein GrpE	pfam01025	GrpE	GrpE.	GO:0006457|	GO:0005737|	GO:0000774|ad	K03687
Manes.03G13710(	0.9842385	0.90426	down	no	EH domain-c	pfam00350	Dynamin_N	Dynamin far	GO:1990090|	GO:0031901|	GO:0005524|A	K12483

Manes.03G13730(	34.025953	0.0042	up	yes	.	pfam00403	HMA	Heavy-meta	.	.	.	.
Manes.03G13740(	5.2332758	6.88E-14	up	yes	Probable LRI	pfam00069	Pkinase	Protein kina	.	GO:0016021|	GO:0005524|A'	.
Manes.03G13750(	3.4075017	3.09E-32	up	yes	Probable seri	pfam00069	Pkinase	Protein kina	.	.	GO:0005524|A'	K08867
Manes.03G13760(	1.9393419	7.81E-14	up	no	Probable WR	pfam03106	WRKY	WRKY DN.	GO:0045893||	GO:0005634|	GO:0043565|se	.
Manes.03G13770(	0.1871256	6.48E-11	down	yes	.	pfam01199	Ribosomal_L34e	Ribosomal p	.	.	.	.
Manes.03G13790(	0.5174447	7.66E-06	down	no	Zinc finger C	pfam14259	RRM_6	RNA recogı	.	.	GO:0003677|DI	.
Manes.03G13810(	2.0311924	2.14E-12	up	yes	U-box domai	pfam13646	HEAT_2	HEAT repea	GO:0046777||	.	GO:0016874|lig	.
Manes.03G13820(	5.1338328	0.22741	up	no	.	pfam03089	RAG2	Recombinat	.	.	.	.
Manes.03G13830(	0.0387782	0.00087	down	yes	.	pfam11981	DUF3482	Domain of u	.	.	.	.
Manes.03G13840(	1.1327849	0.17954	up	no	DUF21 doma	pfam01595	DUF21	Domain of u	GO:0006810|t	GO:0009941|	GO:0050660|fla	.
Manes.03G13850(	3.1670423	3.29E-13	up	yes	Putative glyc	pfam07690	MFS_1	Major Facili	GO:0006820|a	GO:0016021|	GO:0015315|or	.
Manes.03G13860(	25.252365	#######	up	yes	ABC transpo	pfam00664	ABC_membrane	ABC transpo	.	GO:0048046|	GO:0005524|A'	.
Manes.03G13870(	16.353177	1.89E-25	up	yes	ABC transpo	pfam00664	ABC_membrane	ABC transpo	.	GO:0048046|	GO:0005524|A'	.
Manes.03G13900(	0.7028933	0.00041	down	no	Serine acetylt	pfam06426	SATase_N	Serine acety	GO:0019344|c	GO:0005739|	GO:0009001|se	K00640
Manes.03G13910(	6.4404175	9.74E-14	up	yes	Cytochrome]	pfam00067	p450	Cytochrome	.	.	GO:0020037|he	.
Manes.03G13930(	2.1955923	3.99E-07	up	yes	Cytochrome]	pfam00067	p450	Cytochrome	.	.	GO:0020037|he	.
Manes.03G13940(	0.5005563	4.22E-06	down	no	Cytochrome]	pfam00067	p450	Cytochrome	.	.	GO:0020037|he	.
Manes.03G13970(	1.0120682	0.89995	up	no	30S ribosoma	pfam00338	Ribosomal_S10	Ribosomal p	GO:0006412|t	GO:0009507|	GO:0003723|RI	.
Manes.03G13980(	0.636249	6.78E-06	down	no	.	pfam05641	Agenet	Agenet dom	.	.	.	.
Manes.03G13990(	0.269992	8.56E-10	down	yes	DNA-depend	pfam08325	WLM	WLM doma	GO:0006974|c	GO:0005635|	GO:0046872|m	.
Manes.03G14020(	2.0263012	2.04E-14	up	yes	Mitochondria	pfam03031	NIF	NLI interact	GO:0015031||	GO:0016021|	.	.
Manes.03G14040(	0.6131273	8.58E-05	down	no	Two-pore po	pfam07885	Ion_trans_2	Ion channel.	GO:0030007|c	GO:0005887|	GO:0005216|ion	K05389
Manes.03G14050(	0.404677	4.20E-06	down	yes	.	pfam13520	AA_permease_2	Amino acid	.	.	.	.
Manes.03G14100(	1.0275657	0.78232	up	no	RNA-binding	pfam00076	RRM_1	RNA recogı	GO:0071347|c	GO:0071013|	GO:0003682|ch	K12885
Manes.03G14130(	0.8438799	0.17124	down	no	Nudix hydrol	pfam00293	NUDIX	NUDIX dor	GO:0006979|r	.	GO:0047631|Al	.
Manes.03G14160(	0.6974628	0.01074	down	no	Nicotinamide	pfam01467	CTP_transf_2	Cytidylyltra	GO:0009435||	GO:0005634|	GO:0005524|A'	.
Manes.03G14190(	0.6081529	2.34E-06	down	no	Poly(A)-spec	pfam04857	CAF1	CAF1 family	GO:0006952|c	GO:0005737|	GO:0046872|m	K01148
Manes.03G14240(	0.667398	3.38E-05	down	no	Cellulose syn	pfam03552	Cellulose_synt	Cellulose sy	GO:0071555|c	GO:0005783|	GO:0016760|ce	.
Manes.03G14250(	2.0279842	1.07E-14	up	yes	Cellulose syn	pfam03552	Cellulose_synt	Cellulose sy	GO:0071555|c	GO:0005783|	GO:0016760|ce	.
Manes.03G14280(	0.4323663	0.0015	down	yes	Serine/threon	pfam00069	Pkinase	Protein kina	GO:0009734|a	GO:0005737|	GO:0005524|A'	K08286
Manes.03G14300(	1.5917031	1.20E-06	up	no	Heterogeneo	pfam00076	RRM_1	RNA recogı	GO:0006397|r	GO:0005737|	GO:0000166|nu	K14411
Manes.03G14310(	2.0825628	6.66E-14	up	yes	RNA-binding	pfam00013	KH_1	KH domain.	GO:0022008|r	GO:0005730|	GO:0003723|RI	K11884
Manes.03G14320(	22.939814	4.25E-26	up	yes	ABC transpo	pfam00005	ABC_tran	ABC transpo	GO:0009555||	GO:0016021|	GO:0005524|A'	.
Manes.03G14350(	0.6533987	0.02593	down	no	Receptor-like	pfam13855	LRR_8	Leucine rich	GO:0009755|h	GO:0016021|	GO:0004672|pr	.
Manes.03G14360(	1.3209882	0.0327	up	no	E3 ubiquitin-	pfam13639	zf-RING_2	Ring finger	GO:0016567||	GO:0016021|	GO:0016874|lig	.
Manes.03G14370(	0.7345992	0.01111	down	no	Farnesyl pyro	pfam00348	polyprenyl_synt	Polyprenyl s	GO:0006695|c	GO:0005737|	GO:0004161|di	.
Manes.03G14390(	1.8333576	7.30E-10	up	no	Adenosylhon	pfam05221	AdoHcyase	S-adenosyl-	GO:0006730|c	.	GO:0004013|ad	.
Manes.03G14440(	1.1577224	0.13686	up	no	UDP-glucuro	pfam01370	Epimerase	NAD depen	GO:0005975|c	GO:0005768|	GO:0050662|co	K08679
Manes.03G14450(	1.9403605	8.10E-14	up	no	Glycine-rich]	pfam00076	RRM_1	RNA recogı	GO:0009631|c	GO:0005739|	GO:0005524|A'	.
Manes.03G14480(	1.6852579	9.89E-06	up	no	Chaperone pı	pfam00226	DnaJ	DnaJ domai	GO:0061077|c	GO:0009507|	.	.
Manes.03G14490(	0.8058704	0.02916	down	no	.	pfam00630	Filamin	Filamin/ABI	.	.	.	.
Manes.03G14510(	1.8553134	0.00032	up	no	.	pfam13837	Myb_DNA-bind_4	Myb/SANT-	.	.	.	.
Manes.03G14520(	9.7323523	0.00027	up	yes	.	pfam00646	F-box	F-box doma	.	.	.	.
Manes.03G14530(	2.8330262	1.95E-22	up	yes	.	pfam11600	CAF-1_p150	Chromatin a	.	.	.	.
Manes.03G14540(	1.0235407	0.80643	up	no	Beta-galactos	pfam01301	Glyco_hydro_35	Glycosyl hy	GO:0005975|c	GO:0048046|	GO:0004565|be	.
Manes.03G14550(	3.1592205	3.97E-05	up	yes	.	pfam01370	Epimerase	NAD depen	.	.	.	.
Manes.03G14590(	1.788936	5.10E-06	up	no	Alkaline cera	pfam05875	Ceramidase	Ceramidase.	GO:0006672|c	GO:0030176|	GO:0070774|ph	K04711
Manes.03G14640(	0.8801144	0.21003	down	no	Uncharacteri	pfam10250	O-FucT	GDP-fucose	.	GO:0005768|	GO:0016757|tra	.
Manes.03G14670(	0.7776147	0.07417	down	no	Elongation fa	pfam00009	GTP_EFTU	Elongation f	.	GO:0005737|	GO:0005525|G'	.
Manes.03G14700(	0.7947179	0.03239	down	no	Elongation fa	pfam00009	GTP_EFTU	Elongation f	.	GO:0005737|	GO:0005525|G'	.
Manes.03G14710(	0.9635366	0.74836	down	no	ER lumen pro	pfam00810	ER_lumen_recept	ER lumen p	GO:0006621||	GO:0005789|	GO:0046923|EI	.
Manes.03G14730(	0.7693936	0.03397	down	no	Ubiquitin car	pfam00443	UCH	Ubiquitin ca	GO:0043161||	GO:0009507|	GO:0004197|cy	K11844
Manes.03G14740(	1.4869136	6.15E-05	up	no	.	pfam01812	5-FTHF_cyc-lig	5-formyltetr	.	.	.	.
Manes.03G14750(	7.3794959	8.11E-12	up	yes	Ethylene-resp	pfam00847	AP2	AP2 domair	GO:0009873|c	GO:0005634|	GO:0003677|DI	.
Manes.03G14780(	0.6714212	0.01532	down	no	.	pfam04576	Zein-binding	Zein-binding	.	.	.	.
Manes.03G14800(	2.1300344	0.02701	up	yes	Receptor like	pfam00069	Pkinase	Protein kina	GO:0071395|c	GO:0005886|	GO:0005524|A'	.
Manes.03G14810(	0.7426503	0.00298	down	no	Myb family tı	pfam14379	Myb_CC_LHEQL	MYB-CC ty	GO:0010088||	GO:0005634|	GO:0003677|DI	.
Manes.03G14870(	9.1653987	#######	up	yes	Cyclin-depen	pfam02234	CDI	Cyclin-depe	GO:0007050|c	GO:0005654|	GO:0004861|cy	.
Manes.03G14880(	1.0763629	0.46654	up	no	Transcription	pfam00400	WD40	WD domain	GO:0030154|c	GO:0005634|	.	.
Manes.03G14900(	1.2571447	0.01116	up	no	.	pfam04093	MreD	rod shape-d	.	.	.	.
Manes.03G14960(	0.5138205	0.00026	down	no	Protein SULI	pfam00515	TPR_1	Tetratricope	GO:0010438|c	GO:0005634|	.	.
Manes.03G14970(	0.911015	0.36938	down	no	Ubiquinone b	pfam01494	FAD_binding_3	FAD binding	GO:0044550|s	GO:0042995|	GO:0050660|fla	K06126
Manes.03G15010(	0.5529593	0.00252	down	no	Dihydroxy-ac	pfam00920	ILVD_EDD	Dehydratase	GO:0009082||	GO:0009507|	GO:0051539|4	K01687
Manes.03G15020(	2.4921457	4.12E-24	up	yes	3-ketoacyl-C	pfam00108	Thiolase_N	Thiolase, N-	GO:0006635||	GO:0009507|	GO:0003988|ac	K07513
Manes.03G15040(	0.3483636	0.31037	down	no	9-cis-epoxyca	pfam03055	RPE65	Retinal pign	GO:0009688|a	GO:0009570|	GO:0045549|9-	K09840
Manes.03G15060(	NA	NA	--	no	UPF0481 pro	pfam03140	DUF247	Plant protei	.	GO:0016021|	.	.
Manes.03G15140(	Inf	0.9009	up	no	Probable volt	pfam13123	DUF3978	Protein of u	GO:0006813||	GO:0005886|	GO:0005244|vo	.
Manes.03G15230(	1.4616668	7.51E-05	up	no	Cytochrome l	pfam00173	Cyt-b5	Cytochrome	GO:0055114|c	GO:0005789|	GO:0020037|he	.
Manes.03G15240(	1.9572784	1.61E-06	up	no	Purple acid p	pfam00149	Metallophos	Calcineurin-	.	GO:0005576|	GO:0003993|ac	.
Manes.03G15250(	NA	NA	--	no	E3 ubiquitin-	pfam13639	zf-RING_2	Ring finger	GO:0016567||	.	GO:0016874|lig	K11982
Manes.03G15310(	0.1706866	0.00039	down	yes	.	pfam05938	Self-incomp_S1	Plant self-in	.	.	.	.
Manes.03G15350(	Inf	0.9009	up	no	.	pfam05938	Self-incomp_S1	Plant self-in	.	.	.	.
Manes.03G15380(	NA	NA	--	no	.	pfam05938	Self-incomp_S1	Plant self-in	.	.	.	.
Manes.03G15420(	NA	NA	--	no	.	pfam05938	Self-incomp_S1	Plant self-in	.	.	.	.
Manes.03G15440(	3.0096758	2.56E-29	up	yes	Probable leuc	pfam00069	Pkinase	Protein kina	.	GO:0016021|	GO:0005524|A'	.
Manes.03G15470(	6.4088811	9.58E-15	up	yes	Hydroxyethy	pfam02110	HK	Hydroxyeth	GO:0009228|t	.	GO:0005524|A'	K00878
Manes.03G15480(	Inf	0.35135	up	no	Dirigent prot	pfam03018	Dirigent	Dirigent-like	.	GO:0048046|	.	.
Manes.03G15530(	98.708012	2.29E-10	up	yes	Probable glut	pfam13417	GST_N_3	Glutathione	GO:0009734|a	.	GO:0004364|gl	.
Manes.03G15560(	0.0947439	8.48E-35	down	yes	.	pfam14694	LINES_N	Lines N-terr	.	.	.	.
Manes.03G15570(	0.5676113	1.01E-06	down	no	Protein kinas	pfam01764	Lipase_3	Lipase (clas	GO:0006915|a	GO:0005737|	GO:0050681|an	K02677
Manes.03G15580(	0.274089	1.02E-24	down	yes	Beta-amylase	pfam01373	Glyco_hydro_14	Glycosyl hy	GO:0009414|r	GO:0009507|	GO:0016161|be	K01177
Manes.03G15600(	0.8312607	0.05152	down	no	Activating m	pfam00400	WD40	WD domain	GO:0098779|r	GO:0005776|	GO:0031625|ub	K17985
Manes.03G15610(	0.627486	1.20E-05	down	no	Carboxyl-ter	pfam03572	Peptidase_S41	Peptidase fa	.	GO:0009543|	GO:0008236|se	.
Manes.03G15620(	0.4887412	4.71E-08	down	yes	.	pfam15395	DUF4617	Domain of u	.	.	.	.
Manes.03G15670(	0.2300144	0.00052	down	yes	Probable fola	pfam03092	BT1	BT1 family.	GO:0006810|t	GO:0031969|	.	.
Manes.03G15700(	NA	NA	--	no	.	pfam05512	AWPM-19	AWPM-19-	.	.	.	.
Manes.03G15730(	0.7462515	0.04662	down	no	.	pfam13904	DUF4207	Domain of u	.	.	.	.
Manes.03G15760(	4.186577	6.73E-09	up	yes	.	pfam00582	Usp	Universal st	.	.	.	.

Manes.03G15790(	0.772457	0.01268	down	no	DNA mismat	pfam00488	MutS_V	MutS doma	GO:0000710|ı	GO:0009507|	GO:0005524|A'	.
Manes.03G15800(	4.7206832	0.00319	up	yes	.	pfam13499	EF-hand_7	EF-hand do	.	.	.	.
Manes.03G15820(	3.3628578	6.69E-13	up	yes	3-isopropylm	pfam00330	Aconitase	Aconitase fa	GO:0019761|ı	GO:0009507|	GO:0003861|3-	K01703
Manes.03G15860(	0.6649339	0.0013	down	no	Putative G3B	pfam02136	NTF2	Nuclear tran	GO:0051028|ı	GO:0005737|	GO:0000166|nu	.
Manes.03G15930(	0.7535076	0.02269	down	no	Oxidation res	pfam07534	TLD	TLD. This c	GO:0007628|a	GO:0005739|	GO:0016491|ox	.
Manes.03G15940(	Inf	0.04685	up	no	.	pfam05109	Herpes_BLLF1	Herpes virus	.	.	.	.
Manes.03G15950(	1.011036	0.87255	up	no	Pre-mRNA-p	pfam08606	Prp19	Prp19/Pso4-	GO:0006281|l	GO:0071013|	GO:0016874|lig	K10599
Manes.03G15960(	4.9496611	1.89E-06	up	yes	Zinc finger pı	pfam06203	CCT	CCT motif.	.	GO:0005634|	GO:0008270|ziı	.
Manes.03G15970(	2.2682526	1.19E-20	up	yes	.	pfam12043	DUF3527	Domain of ı	.	.	.	.
Manes.03G15990(	0.8598875	0.49973	down	no	Peroxygenase	pfam05042	Caleosin	Caleosin rel	.	GO:0005783|	GO:0046872|me	.
Manes.03G16050(	0.5316358	0.04208	down	no	.	pfam00566	RabGAP-TBC	Rab-GTPase	.	.	.	.
Manes.03G16070(	0.2926166	0.02677	down	yes	Cyclopropane	pfam02353	CMAS	Mycolic acic	GO:0006633|ı	GO:0005829|	GO:0008825|cy	K00574
Manes.03G16080(	0.8043969	0.20633	down	no	Cyclopropane	pfam02353	CMAS	Mycolic acic	GO:0006633|ı	GO:0005829|	GO:0008825|cy	K00574
Manes.03G16140(	0.7391478	0.00508	down	no	.	pfam01593	Amino_oxidase	Flavin conta	.	.	.	.
Manes.03G16150(	0.2652662	5.25E-05	down	yes	Protein NRT	pfam05664	DUF810	Protein of u	GO:0042742|ı	GO:0016021|	GO:0042936|dip	.
Manes.03G16160(	0.5645735	0.01006	down	no	Protein NRT	pfam00854	PTR2	POT family.	GO:0042742|ı	GO:0016021|	GO:0042936|dip	.
Manes.03G16180(	0.398255	5.87E-18	down	yes	.	pfam13414	TPR_11	TPR repeat.	.	.	.	.
Manes.03G16190(	0.4927388	0.00046	down	yes	.	pfam09366	DUF1997	Protein of u	.	.	.	.
Manes.03G16240(	0.5401905	0.00017	down	no	Polyubiquitin	pfam00240	ubiquitin	Ubiquitin fa	.	GO:0005737|	.	.
Manes.03G16260(	1.1230472	0.44956	up	no	Polyubiquitin	pfam00240	ubiquitin	Ubiquitin fa	.	GO:0005737|	.	.
Manes.03G16270(	0.8966216	0.36802	down	no	Pleckstrin ho	pfam08718	GLTP	Glycolipid tı	GO:0015031|ı	GO:0005794|	GO:0051861|gl	K08051
Manes.03G16330(	1.345443	0.00115	up	no	Casein kinase	pfam00069	Pkinase	Protein kina	GO:0006897|ı	GO:0005737|	GO:0005524|A'	K02218
Manes.03G16340(	0.8111877	0.03115	down	no	Serine/threon	pfam00069	Pkinase	Protein kina	GO:0035556|ı	GO:0005737|	GO:0005524|A'	K08790
Manes.03G16360(	0.3696232	9.66E-12	down	yes	Protein LIGH	pfam04852	DUF640	Protein of u	GO:0010492|ı	GO:0005634|	GO:0003677|DI	.
Manes.03G16380(	1.1660766	0.16128	up	no	Protein tas	pfam00248	Aldo_ket_red	Aldo/keto re	.	.	GO:0016491|ox	.
Manes.03G16400(	1.6614119	0.00014	up	no	.	pfam05553	DUF761	Cotton fibre	.	.	.	.
Manes.03G16420(	210.03909	7.54E-26	up	yes	Myb-related p	pfam00249	Myb_DNA-binding	Myb-like DI	GO:0006355|ı	GO:0005634|	GO:0003677|DI	K09422
Manes.03G16430(	1.252203	0.09197	up	no	Chromatin m	pfam09340	NuA4	Histone acet	GO:0043968|l	GO:0070776|	.	K11344
Manes.03G16440(	1.2008273	0.05633	up	no	Actin-depoly	pfam00241	Cofilin_ADF	Cofilin/trop	GO:0030042|a	GO:0015629|	.	K05765
Manes.03G16450(	2.9362854	5.38E-10	up	yes	Phosphoenol	pfam00069	Pkinase	Protein kina	GO:0009738|a	GO:0005737|	GO:0005524|A'	.
Manes.03G16460(	1.1093623	0.5708	up	no	Inorganic pho	pfam00083	Sugar_tr	Sugar (and c	GO:0035435|ı	GO:0005794|	GO:0005315|in	K08176
Manes.03G16470(	4.3783863	3.47E-07	up	yes	Probable inor	pfam00083	Sugar_tr	Sugar (and c	GO:1901684|a	GO:0005887|	GO:1901683|ar	K08176
Manes.03G16510(	12.42063	3.03E-36	up	yes	Ethylene-resp	pfam00847	AP2	AP2 domair	GO:0006952|c	GO:0005634|	GO:0003677|DI	K14516
Manes.03G16530(	Inf	6.39E-64	up	yes	Ethylene-resp	pfam00847	AP2	AP2 domair	GO:0009873|c	GO:0005634|	GO:0003677|DI	.
Manes.03G16550(	1.4078288	0.00167	up	no	Electron tran	pfam01012	ETF	Electron tra	GO:0015996|c	GO:0005759|	GO:0009055|el	.
Manes.03G16590(	0.8366533	0.18532	down	no	1-aminocyclo	pfam03171	2OG-FeII_Oxy	2OG-Fe(II)	.	.	GO:0046872|me	.
Manes.03G16600(	21.904482	5.53E-07	up	yes	1-aminocyclo	pfam03171	2OG-FeII_Oxy	2OG-Fe(II)	.	.	GO:0046872|me	.
Manes.03G16610(	0.7068026	0.00136	down	no	1-aminocyclo	pfam03171	2OG-FeII_Oxy	2OG-Fe(II)	.	.	GO:0046872|me	.
Manes.03G16620(	4.4650991	0.08461	up	no	1-aminocyclo	pfam03171	2OG-FeII_Oxy	2OG-Fe(II)	.	.	GO:0046872|me	.
Manes.03G16630(	0.7654761	0.0068	down	no	1-aminocyclo	pfam03171	2OG-FeII_Oxy	2OG-Fe(II)	.	.	GO:0046872|me	.
Manes.03G16640(	7.1939474	1.83E-07	up	yes	1-aminocyclo	pfam03171	2OG-FeII_Oxy	2OG-Fe(II)	.	.	GO:0046872|me	.
Manes.03G16680(	1.499409	8.36E-06	up	no	.	pfam05514	HR_lesion	HR-like lesi	.	.	.	.
Manes.03G16720(	0.5652605	0.00248	down	no	Bet1-like SN	pfam05739	SNARE	SNARE dor	GO:0006888|l	GO:0005789|	GO:0005484|SN	K08504
Manes.03G16770(	0.4839595	7.49E-05	down	yes	Serine/threon	pfam00069	Pkinase	Protein kina	GO:0032147|a	GO:0005737|	GO:0005524|A'	.
Manes.03G16780(	0.6024257	0.09387	down	no	Cellulose syn	pfam03552	Cellulose_synt	Cellulose sy	GO:0071555|c	GO:0000139|	GO:0016760|ce	.
Manes.03G16810(	0.9082163	0.43517	down	no	.	pfam05641	Agenet	Agenet dom	.	.	.	.
Manes.03G16830(	18.140692	0.00166	up	yes	.	pfam03896	TRAP_alpha	Translocon-	.	.	.	.
Manes.03G16860(	1.6085454	1.50E-07	up	no	Protein WVD	pfam06886	TPX2	Targeting pı	.	GO:0005737|	.	.
Manes.03G16870(	0.6752826	0.0001	down	no	StAR-related	pfam01852	START	START dor	GO:1903955|ı	GO:0005739|	GO:0008289|lip	.
Manes.03G16880(	0.6276124	3.12E-06	down	no	.	pfam03126	Plus-3	Plus-3 doma	.	.	.	.
Manes.03G16890(	0.8838194	0.38029	down	no	.	pfam06414	Zeta_toxin	Zeta toxin. '	.	.	.	.
Manes.03G16900(	1.5363906	1.33E-06	up	no	40S ribosoma	pfam00203	Ribosomal_S19	Ribosomal p	GO:0006412|ı	GO:0022626|	GO:0003723|RI	.
Manes.03G16910(	6.337019	0.00263	up	yes	Transcription	pfam00010	HLH	Helix-loop-l	GO:0006355|ı	GO:0005634|	GO:0003677|DI	.
Manes.03G16920(	2.0450808	4.31E-15	up	yes	Chloroplastic	pfam01985	CRS1_YhbY	CRS1 / Yhb	GO:0000373|ı	GO:0009570|	GO:0003723|RI	.
Manes.03G16980(	3.066908	2.08E-34	up	yes	Auxin-respon	pfam02309	AUX_IAA	AUX/IAA f	GO:0009734|a	GO:0005634|	GO:0003677|DI	K14484
Manes.03G17010(	0.7897506	0.08355	down	no	Putative ion c	pfam06241	DUF1012	Protein of u	.	GO:0009507|	.	.
Manes.03G17030(	20.82845	7.89E-72	up	yes	CBL-interact	pfam00069	Pkinase	Protein kina	GO:0007165|s	.	GO:0005524|A'	.
Manes.03G17090(	6.6310833	0.13453	up	no	.	pfam07922	Glyco_transf_52	Glycosyltrar	.	.	.	.
Manes.03G17100(	0.4124308	1.16E-08	down	yes	.	pfam04720	DUF506	Protein of u	.	.	.	.
Manes.03G17120(	1.3972574	0.00022	up	no	Pyruvate kina	pfam00224	PK	Pyruvate kir	GO:0006096|g	GO:0009507|	GO:0005524|A'	K00873
Manes.03G17200(	4.8075176	6.07E-20	up	yes	Putative calci	pfam00689	Cation_ATPase_C	Cation trans	.	GO:0016021|	GO:0005524|A'	K01537
Manes.03G17220(	3.1595076	1.10E-06	up	yes	Calcium-tran	pfam00689	Cation_ATPase_C	Cation trans	.	GO:0016021|	GO:0005524|A'	K01537
Manes.03G17320(	49.968658	4.89E-17	up	yes	Heat stress tr	pfam00447	HSF_DNA-bind	HSF-type D	GO:0009408|ı	GO:0005737|	GO:0043565|se	K09419
Manes.03G17330(	1.3201732	0.00234	up	no	1,2-dihydrox	pfam03079	ARD	ARD/ARD'	GO:0019509|l	GO:0005737|	GO:0010309|ac	K08967
Manes.03G17340(	0.4794787	0.00199	down	yes	EPIDERMAL	pfam15621	PROL5-SMR	Proline-rich	GO:0010052|ı	GO:0005576|	.	.
Manes.03G17370(	0.4850359	5.31E-11	down	yes	Chlorophylla	pfam12740	Chlorophyllase2	Chlorophylla	GO:0015996|c	GO:0009507|	GO:0047746|ch	K08099
Manes.03G17430(	0.3014973	5.67E-07	down	yes	Leucine-rich	pfam13855	LRR_8	Leucine rich	GO:0071555|c	GO:0005618|	GO:0005199|stı	.
Manes.03G17440(	1.3977582	0.00021	up	no	40S ribosoma	pfam01015	Ribosomal_S3Ae	Ribosomal S	GO:0006412|ı	GO:0022627|	GO:0003735|stı	K02984
Manes.03G17470(	0.3858645	0.00066	down	yes	.	pfam01770	Folate_carrier	Reduced fol	.	.	.	.
Manes.03G17480(	0.326694	2.54E-17	down	yes	.	pfam07765	KIP1	KIP1-like pı	.	.	.	.
Manes.03G17510(	0.4428638	0.00028	down	yes	Pentatricope	pfam13041	PPR_2	PPR repeat	.	GO:0005739|	.	.
Manes.03G17550(	0.692481	0.0026	down	no	ATP-depende	pfam12513	SUV3_C	Mitochondr	GO:0070827|c	GO:0045025|	GO:0034458|3'-	K17675
Manes.03G17590(	0.6795756	0.00025	down	no	Probable RN	pfam13087	AAA_12	AAA domai	GO:0035194|l	GO:0005737|	GO:0005524|A'	K18422
Manes.03G17670(	10.604584	#######	up	yes	Uncharacteriz	pfam13302	Acetyltransf_3	Acetyltransf	.	.	GO:0008080|N-	.
Manes.03G17690(	0.2175729	4.24E-30	down	yes	.	pfam04570	DUF581	Protein of u	.	.	.	.
Manes.03G17730(	1.8404827	1.27E-10	up	no	.	pfam00011	HSP20	Hsp20/alpha	.	.	.	.
Manes.03G17760(	1.5915287	3.32E-07	up	no	.	pfam07926	TPR_MLP1_2	TPR/MLP1/	.	.	.	.
Manes.03G17810(	0.4898577	0.00457	down	yes	Transcription	pfam04844	Ovate	Transcriptio	GO:0045892|ı	GO:0005634|	.	.
Manes.03G17820(	0.5877397	1.21E-05	down	no	Preprotein tra	pfam01592	NifU_N	NifU-like N	GO:0009306|l	GO:0009507|	GO:0015450|P-	.
Manes.03G17830(	0.8161036	0.12928	down	no	Probable pref	pfam01920	Prefoldin_2	Prefoldin su	GO:0006457|l	GO:0016272|	.	K09549
Manes.03G17850(	1.2167668	0.036	up	no	FRIGIDA-lik	pfam07899	Frigida	Frigida-like	GO:0030154|c	.	.	.
Manes.03G17870(	0.2652299	6.47E-22	down	yes	Photosystem	pfam13326	PSII_Pbs27	Photosysten	GO:0010207|l	GO:0009507|	.	.
Manes.03G17900(	0.713748	0.00965	down	no	40S ribosoma	pfam01282	Ribosomal_S24e	Ribosomal p	GO:0006412|ı	GO:0005618|	GO:0000166|nu	K02974
Manes.03G17910(	6.3782216	7.61E-27	up	yes	.	pfam07466	DUF1517	Protein of u	.	.	.	.
Manes.03G17930(	0.2929338	0.00062	down	yes	5'-nucleotidas	pfam01975	SurE	Survival pro	GO:0016311|c	GO:0005737|	GO:0008253|5'-	K03787
Manes.03G17960(	0.4355914	6.47E-07	down	yes	.	pfam00650	CRAL_TRIO	CRAL/TRIC	.	.	.	.
Manes.03G18020(	4.562003	3.62E-34	up	yes	Uncharacteriz	pfam13419	HAD_2	Haloacid de	GO:0016311|c	GO:0005737|	GO:0008252|nu	K18551
Manes.03G18030(	0.7944644	0.03133	down	no	Vacuole men	pfam09335	SNARE_assoc	SNARE ass	GO:0010256|ı	GO:0012505|	.	.

Manes.03G18040(	1.3093892	0.46505	up	no	Sugar transp(	pfam00083	Sugar_tr	Sugar (and (	GO:0046323|	GO:0005887|	GO:0009679|he	.
Manes.03G18070(	1.1883606	0.05882	up	no	Long chain a(	pfam00501	AMP-binding	AMP-bindin	GO:0006631|	GO:0005777|	GO:0005524|A'	K01897
Manes.03G18090(	0.943742	0.54911	down	no	Cyclin-T1-4	pfam00134	Cyclin_N	Cyclin, N-te	GO:0007049|	GO:0005634|	.	K15188
Manes.03G18110(	2.3301084	2.36E-05	up	yes	Probable mer	pfam04925	SHQ1	SHQ1 prote	GO:0009742|	GO:0005886|	GO:0019210|ki	.
Manes.03G18120(	1.792582	3.66E-07	up	no	.	pfam01269	Fibrillarin	Fibrillarin.	.	.	.	.
Manes.03G18130(	3.1113641	8.57E-22	up	yes	Probable LRI	pfam12819	Malectin_like	Carbohydrat	.	GO:0016021|	GO:0005524|A'	.
Manes.03G18140(	0.7851382	0.01135	down	no	ARM REPEA	pfam00651	BTB	BTB/POZ d	GO:0010187|	GO:0005737|	.	.
Manes.03G18160(	2.6153449	4.89E-27	up	yes	Probable ATI	pfam00176	SNF2_N	SNF2 family	GO:0016568|	GO:0005634|	GO:0005524|A'	K11647
Manes.03G18230(	0.7762233	0.02658	down	no	E3 ubiquitin-	pfam13639	zf-RING_2	Ring finger	GO:0009561|	GO:0005886|	GO:0016874|lig	K19043
Manes.03G18270(	2.7458762	3.66E-28	up	yes	.	pfam03125	Sre	C. elegans S	.	.	.	.
Manes.03G18280(	2.1526719	4.45E-13	up	yes	NEDD8-activ	pfam00899	ThiF	ThiF family.	GO:0045116|	GO:0005829|	GO:0016881|ac	K10686
Manes.03G18290(	0.7739433	0.0319	down	no	Protein trich(	pfam13839	PC-Esterase	GDSL/SGN	.	GO:0016021|	.	.
Manes.03G18300(	0.7980549	0.15446	down	no	.	pfam00119	ATP-synt_A	ATP syntha:	.	.	.	.
Manes.03G18320(	2.4783086	1.03E-24	up	yes	Nuclear trans	pfam00808	CBFD_NFYB_HN	Histone-like	GO:0006351|	GO:0016602|	GO:0043565|se	.
Manes.03G18330(	0.9008391	0.29358	down	no	.	pfam12481	DUF3700	Aluminium i	.	.	.	.
Manes.03G18340(	2.4306838	0.00574	up	yes	Probable aqu	pfam00230	MIP	Major intrin	GO:0035445|	GO:0005887|	GO:0046715|bo	K09874
Manes.03G18350(	128.52892	#######	up	yes	Basic 7S glol	pfam14541	TAXi_C	Xylanase inl	.	.	GO:0004190|as	.
Manes.03G18370(	75.275214	1.51E-29	up	yes	.	pfam14541	TAXi_C	Xylanase inl	.	.	.	.
Manes.03G18380(	3.1156398	0.00588	up	yes	Basic 7S glol	pfam14541	TAXi_C	Xylanase inl	.	.	GO:0004190|as	.
Manes.03G18390(	3.1045101	0.17114	up	no	Basic 7S glol	pfam14541	TAXi_C	Xylanase inl	.	.	GO:0004190|as	.
Manes.03G18410(	3.1331932	3.69E-08	up	yes	Basic 7S glol	pfam14541	TAXi_C	Xylanase inl	.	.	GO:0004190|as	.
Manes.03G18440(	0.4276721	3.28E-16	down	yes	Copper trans|	pfam00403	HMA	Heavy-meta	GO:0006825|	GO:0000785|	GO:0046872|m	K07213
Manes.03G18460(	1.9980369	0.0058	up	no	.	pfam01763	Herpes_UL6	Herpesvirus	.	.	.	.
Manes.03G18480(	0.2883931	5.91E-20	down	yes	.	pfam00654	Voltage_CLC	Voltage gat	.	.	.	.
Manes.03G18530(	0.8183401	0.04868	down	no	Serine incorp	pfam03348	Serinc	Serine incor	GO:0006658|	GO:0000139|	GO:0015194|L-	.
Manes.03G18550(	1.7630191	2.35E-06	up	no	Pentatricope|	pfam13041	PPR_2	PPR repeat	.	GO:0005739|	.	.
Manes.03G18560(	0.4774568	1.06E-09	down	yes	Mediator of I	pfam08389	Xpo1	Exportin 1-l	GO:0009698|	GO:0016592|	.	.
Manes.03G18570(	1.2303027	0.10032	up	no	Ribonucleosi(	pfam00268	Ribonuc_red_sm	Ribonucleot	GO:0009186|	GO:0005829|	GO:0046872|m	K10808
Manes.03G18630(	Inf	0.00699	up	yes	.	pfam04020	DUF360	Membrane p	.	.	.	.
Manes.03G18670(	9.5166845	8.68E-06	up	yes	Protein trich(	pfam13839	PC-Esterase	GDSL/SGN	.	GO:0016021|	.	.
Manes.03G18680(	5.9614934	1.63E-05	up	yes	Protein FAR1	pfam13839	PC-Esterase	GDSL/SGN	GO:0006355|	GO:0005634|	GO:0008270|zi	.
Manes.03G18730(	1.2066544	0.14142	up	no	.	pfam03909	BSD	BSD domai	.	.	.	.
Manes.03G18740(	0.7811114	0.14622	down	no	Calcium-bind	pfam08569	Mo25	Mo25-like.	GO:0007050|	GO:0005829|	.	K08272
Manes.03G18750(	0.8570851	0.13895	down	no	F-box/LRR-r	pfam02948	Amelogenin	Amelogenin	.	.	.	K10268
Manes.03G18760(	1.9869316	3.68E-12	up	no	AP-5 comple	pfam00928	Adap_comp_sub	Adaptor cor	GO:0006886|	GO:0030131|	.	K19023
Manes.03G18780(	3.4203268	3.77E-45	up	yes	Probable prot	pfam00481	PP2C	Protein pho:	GO:0006470|	GO:0005886|	GO:0046872|m	.
Manes.03G18790(	0.9116101	0.34594	down	no	Germinal-cen	pfam03399	SAC3_GANP	SAC3/GAN	GO:0002376|	GO:0005737|	.	.
Manes.03G18800(	Inf	1.76E-05	up	yes	.	pfam05188	MutS_II	MutS doma	.	.	.	.
Manes.03G18840(	1.0796965	0.4858	up	no	Uncharacteriz	pfam00588	SpoU_methylase	SpoU rRNA	GO:0006396|	.	GO:0003723|RI	K03437
Manes.03G18850(	1.8649557	0.03901	up	no	.	pfam05078	DUF679	Protein of u	.	.	.	.
Manes.03G18870(	3.4564973	0.02766	up	yes	.	pfam15558	DUF4659	Domain of u	.	.	.	.
Manes.03G18880(	2.2583546	4.11E-20	up	yes	Uncharacteriz	pfam15449	Retinal	Retinal prot	.	GO:0016021|	GO:0022857|tra	.
Manes.03G18900(	0.7033793	0.00063	down	no	Histidine-con	pfam01627	Hpt	Hpt domain	GO:0009736|	GO:0005737|	GO:0009927|his	K14490
Manes.03G18930(	0.8581974	0.36188	down	no	.	pfam00400	WD40	WD domain	.	.	.	.
Manes.03G18950(	0.7666358	0.00707	down	no	Serine/argini	pfam00076	RRM_1	RNA recogi	GO:0000398|	GO:0016607|	GO:0000166|nu	K12900
Manes.03G19010(	0.933526	0.48005	down	no	Probable E3	pfam12906	RINGv	RING-varia	GO:0042335|	GO:0016021|	GO:0016874|lig	K10661
Manes.03G19020(	1.2169978	0.04711	up	no	PAX-interact	pfam04307	DUF457	Predicted m	GO:0060612|	GO:0035097|	.	K14972
Manes.03G19030(	0.7098861	0.00129	down	no	Chaperone pr	pfam11926	DUF3444	Domain of u	GO:0006260|	GO:0005737|	GO:0005524|A'	.
Manes.03G19050(	0.1414148	0.05677	down	no	.	pfam12301	CD99L2	CD99 antig	.	.	.	.
Manes.03G19070(	3.3724023	0.00049	up	yes	Sodium/hydr	pfam00999	Na_H_Exchanger	Sodium/hyd	GO:0055075|	GO:0016021|	GO:0015386|pc	.
Manes.03G19080(	0.7017671	0.0036	down	no	.	pfam08209	Sgf11	Sgf11 (trans	.	.	.	.
Manes.03G19100(	5.2086014	2.98E-15	up	yes	Inactive beta-	pfam01373	Glyco_hydro_14	Glycosyl hy	GO:0000272|	GO:0005737|	GO:0016161|be	.
Manes.03G19110(	3.0437484	0.00788	up	yes	.	pfam12734	CYSTM	Cysteine-ric	.	.	.	.
Manes.03G19120(	2.6168438	4.54E-28	up	yes	Probable seri	pfam00069	Pkinase	Protein kina	.	.	GO:0005524|A'	.
Manes.03G19130(	0.8933003	0.36784	down	no	.	pfam12077	DUF3556	Transmembi	.	.	.	.
Manes.03G19140(	0.6958299	0.02991	down	no	.	pfam06102	DUF947	Domain of u	.	.	.	.
Manes.03G19180(	1.2230637	0.0269	up	no	Lipase	pfam01764	Lipase_3	Lipase (clas	GO:0016042|	.	GO:0046872|m	.
Manes.03G19190(	0.9082385	0.3448	down	no	Putative chro	pfam00176	SNF2_N	SNF2 family	GO:0043044|	GO:0016589|	GO:0005524|A'	.
Manes.03G19200(	0.8691328	0.18128	down	no	Serine/threon	pfam07714	Pkinase_Tyr	Protein tyro	GO:0006952|	GO:0005886|	GO:0005524|A'	K13430
Manes.03G19230(	0.9717326	0.79314	down	no	Probable seri	pfam00036	EF-hand_1	EF hand. Th	GO:0030865|	GO:0005813|	GO:0005509|ca	K11583
Manes.03G19300(	0.6989027	0.00032	down	no	.	pfam07496	zf-CW	CW-type Zi	.	.	.	.
Manes.03G19310(	0.6583201	0.00255	down	no	Protein SRG1	pfam03171	2OG-FeII_Oxy	2OG-Fe(II)	GO:0010260|	.	GO:0046872|m	.
Manes.03G19340(	1.4511236	0.02296	up	no	Cullin-1	pfam00888	Cullin	Cullin family	GO:0009734|	GO:0000794|	.	K03347
Manes.03G19380(	0.4036589	0.0134	down	yes	.	pfam07780	Spb1_C	Spb1 C-tern	.	.	.	.
Manes.03G19390(	0.7862723	0.05121	down	no	.	pfam03772	Competence	Competence	.	.	.	.
Manes.03G19400(	1.1893736	0.39872	up	no	.	pfam11725	AvrE	Pathogenicit	.	.	.	.
Manes.03G19470(	0.7399991	0.07988	down	no	Alanyl-tRNA	pfam01411	tRNA-synt_2c	tRNA synth	GO:0006419|	GO:0005737|	GO:0004813|ala	K01872
Manes.03G19500(	NA	NA	--	no	.	pfam03956	DUF340	Membrane p	.	.	.	.
Manes.03G19520(	0.9239406	0.44541	down	no	Xyloglucan 6	pfam05637	Glyco_transf_34	galactosyl tr	GO:0000271|	GO:0005768|	GO:0035252|UI	K08238
Manes.03G19530(	1.6107796	8.97E-08	up	no	Probable hist	pfam05185	PRMT5	PRMT5 arg	GO:0034971|	GO:0005737|	GO:0016277|[n	K05931
Manes.03G19540(	0.7981231	0.0201	down	no	Isoleucine--tl	pfam00133	tRNA-synt_1	tRNA synth	GO:0006428|	GO:0005737|	GO:0002161|an	K01870
Manes.03G19550(	3.0862814	9.55E-07	up	yes	Translocase (	pfam11886	DUF3406	Domain of u	GO:0045036|	GO:0009507|	GO:0005525|G'	.
Manes.03G19560(	4.0815761	9.19E-50	up	yes	.	pfam14728	PHTB1_C	PTHB1 C-te	.	.	.	.
Manes.03G19570(	0.3014293	2.28E-05	down	yes	.	pfam13919	ASXH	Asx homolo	.	.	.	.
Manes.03G19580(	2.1596753	4.11E-09	up	yes	.	pfam13041	PPR_2	PPR repeat	.	.	.	.
Manes.03G19600(	0.5053363	1.39E-10	down	no	Ubiquitin-like	pfam02902	Peptidase_C48	Ulp1 protea	GO:0009911|	GO:0031965|	GO:0016929|SI	K08592
Manes.03G19620(	0.6993881	0.00282	down	no	.	pfam13041	PPR_2	PPR repeat	.	.	.	.
Manes.03G19630(	3.0942299	5.83E-25	up	yes	.	pfam13639	zf-RING_2	Ring finger	.	.	.	.
Manes.03G19640(	20.006052	0.00011	up	yes	.	pfam01677	Herpes_UL7	Herpesvirus	.	.	.	.
Manes.03G19660(	0.659989	0.00637	down	no	Chaperone pr	pfam00226	DnaJ	DnaJ domai	.	GO:0016021|	.	K09518
Manes.03G19680(	0.6791722	6.69E-05	down	no	Putative E3 u	pfam00400	WD40	WD domain	GO:0009877|	.	GO:0016874|lig	.
Manes.03G19690(	1.0654748	0.46602	up	no	Protein pleiot	pfam00400	WD40	WD domain	GO:0048825|	GO:0080008|	.	K12862
Manes.03G19700(	1.0837755	0.51075	up	no	60S ribosoma	pfam01776	Ribosomal_L22e	Ribosomal I	GO:0006412|	GO:0005829|	GO:0003735|stı	K02891
Manes.03G19710(	1.2528123	0.41954	up	no	Late embryog	pfam03242	LEA_3	Late embryo	GO:0006950|	.	.	.
Manes.03G19730(	0.9126131	0.93251	down	no	Bidirectional	pfam03083	MtN3_slv	Sugar efflux	GO:0034219|	GO:0005887|	GO:0051119|su	K15382
Manes.03G19740(	1.519287	3.34E-06	up	no	Glyoxysomal	pfam02737	3HCDH_N	3-hydroxyac	GO:0006635|	GO:0009514|	GO:0018812|3-	.
Manes.03G19760(	1.5183123	4.88E-06	up	no	Dynein 8 kDa	pfam01221	Dynein_light	Dynein light	GO:0009086|	GO:0005930|	GO:0003774|m	K10418
Manes.03G19790(	1.9521094	1.40E-05	up	no	.	pfam04570	DUF581	Protein of u	.	.	.	.

Manes.03G19810(	1.5825784	1.10E-06	up	no	Lipoamide ac	pfam00198	2-oxoacid_dh	2-oxoacid d	GO:0043617|	GO:0005759|	GO:0016407|ac	K09699
Manes.03G19820(	1.3816845	0.0011	up	no	Acylpyruvase	pfam01557	FAA_hydrolase	Fumarylacet	.	GO:0005829|	GO:0018773|ac	K01557
Manes.03G19840(	14.746281	#######	up	yes	RING-H2 fin	pfam13639	zf-RING_2	Ring finger	GO:0006952|	GO:0016021|	GO:0008270|zi	.
Manes.03G19860(	2.9613329	0.06397	up	no	Putative pect	pfam01095	Pectinesterase	Pectinestera	GO:0042545|	GO:0005618|	GO:0045330|as	K01051
Manes.03G19870(	3.0113505	0.02346	up	yes	.	pfam00646	F-box	F-box doma	.	.	.	.
Manes.03G19880(	1.1658369	0.10411	up	no	Acyl-CoA de	pfam01636	APH	Phosphotrar	GO:0033539|	GO:0005739|	GO:0003995|ac	K11729
Manes.03G19890(	0.399973	1.09E-06	down	yes	Sucrose synt	pfam00862	Sucrose_synth	Sucrose syn	GO:0009058|	GO:0005829|	GO:0016157|su	K00695
Manes.03G19930(	3.7436348	0.00899	up	yes	Ethylene-resp	pfam00847	AP2	AP2 domair	GO:0008219|	GO:0005737|	GO:0003677|DI	K09286
Manes.03G19970(	0.2552504	2.54E-08	down	yes	Probable prot	pfam00481	PP2C	Protein phos	GO:0006470|	.	GO:0046872|m	.
Manes.03G19980(	0.8842329	0.25712	down	no	Mediator of I	pfam08612	Med20	TATA-bindi	GO:0006351|	GO:0016592|	GO:0001104|RI	K13528
Manes.03G20000(	1.864797	1.02E-10	up	no	Pumilio hom	pfam08144	CPL	CPL (NUC1	GO:0006417|	GO:0005730|	GO:0003729|m	K14844
Manes.03G20040(	Inf	0.00044	up	yes	Major allerge	pfam00407	Bet_v_1	Pathogenesi	GO:0006952|	.	.	.
Manes.03G20050(	Inf	0.08849	up	no	Major allerge	pfam00407	Bet_v_1	Pathogenesi	GO:0006952|	.	.	.
Manes.03G20060(	3.1692946	6.15E-05	up	yes	Major allerge	pfam00407	Bet_v_1	Pathogenesi	GO:0006952|	.	.	.
Manes.03G20070(	2.2291536	1.22E-18	up	yes	.	pfam05623	DUF789	Protein of u	.	.	.	.
Manes.03G20080(	0.9738412	0.82014	down	no	Protein DEH	pfam14571	Di19_C	Stress-induc	GO:0009785|	GO:0005634|	.	.
Manes.03G20090(	1.1752914	0.48445	up	no	BTB/POZ an	pfam00917	MATH	MATH dom	GO:0071472|	GO:0005634|	.	K10523
Manes.03G20110(	2.9068129	1.94E-27	up	yes	COBRA-like	pfam04833	COBRA	COBRA-lik	GO:0016049|	GO:0031225|	.	.
Manes.03G20130(	0.5043908	1.93E-09	down	no	GATA transc	pfam00320	GATA	GATA zinc	GO:0006351|	GO:0005634|	GO:0043565|se	.
Manes.03G20140(	1.2529243	0.01339	up	no	Importin sub	pfam01749	IBB	Importin bet	GO:0006606|	GO:0005618|	GO:0008565|pr	.
Manes.03G20180(	1.472436	2.43E-05	up	no	AMSH-like u	pfam01398	JAB	JAB1/Mov3	GO:0006897|	GO:0016021|	GO:0046872|m	K11866
Manes.03G20190(	Inf	0.02411	up	yes	Protein tricho	pfam13839	PC-Esterase	GDSL/SGN	.	GO:0016021|	.	.
Manes.03G20220(	1.0289978	0.74482	up	no	Calmodulin-b	pfam03859	CG-1	CG-1 domai	GO:0006351|	GO:0005634|	GO:0003677|DI	.
Manes.03G20280(	23.914198	0.02642	up	yes	DNA-directe	pfam01096	TFIIS_C	Transcriptio	GO:0001193|	GO:0005665|	GO:0003677|DI	K03017
Manes.03G20310(	0.8174905	0.42342	down	no	.	pfam13041	PPR_2	PPR repeat	.	.	.	.
Manes.03G20330(	Inf	0.04608	up	no	.	pfam13961	DUF4219	Domain of u	.	.	.	.
Manes.03G20340(	1.2868539	0.00739	up	no	Ubiquitin-cor	pfam00179	UQ_con	Ubiquitin-co	GO:0016567|	GO:0005737|	GO:0005524|A	K10578
Manes.03G20410(	0.3713643	2.72E-10	down	yes	.	pfam04795	PAPA-1	PAPA-1-lik	.	.	.	.
Manes.03G20460(	0.6524937	1.23E-05	down	no	PsbB mRNA	pfam13432	TPR_16	Tetratricope	GO:0030154|	GO:0009941|	GO:0003727|sir	.
Manes.03G20500(	0.9835022	0.87061	down	no	ATP-citrate s	pfam00285	Citrate_synt	Citrate synt	GO:0044262|	GO:0005829|	GO:0005524|A	K01648
Manes.03G20510(	0.9826124	0.88615	down	no	Phytochrome	pfam00360	PHY	Phytochrom	GO:0009584|	GO:0005622|	GO:0000155|ph	.
Manes.03G20520(	0.6743296	0.00067	down	no	Serine/threon	pfam07714	Pkinase_Tyr	Protein tyro	GO:0009738|	GO:0005829|	GO:0005524|A	.
Manes.03G20570(	0.5266267	8.47E-05	down	no	Probable prot	pfam00481	PP2C	Protein phos	GO:0006470|	.	GO:0046872|m	K01102
Manes.03G20580(	1.4444351	3.79E-05	up	no	.	pfam12698	ABC2_membrane_	ABC-2 fami	.	.	.	.
Manes.03G20600(	0.5493395	0.00013	down	no	DNA replicat	pfam05916	Sld5	GINS comp	GO:0032508|	GO:0005737|	.	K10735
Manes.03G20630(	1.3931482	0.00164	up	no	Probable pref	pfam02996	Prefoldin	Prefoldin su	GO:0007017|	GO:0005829|	GO:0015631|tu	.
Manes.03G20640(	1.5497012	0.15522	up	no	Probable WR	pfam03106	WRKY	WRKY DN	GO:0042742|	GO:0005634|	GO:0003677|DI	.
Manes.03G20700(	17.46273	2.20E-13	up	yes	Cell wall / va	pfam04043	PMEI	Plant inverta	GO:0043086|	GO:0005618|	GO:0004857|en	.
Manes.03G20710(	2.0820604	2.34E-10	up	yes	Cell wall / va	pfam04043	PMEI	Plant inverta	GO:0043086|	GO:0005618|	GO:0004857|en	.
Manes.03G20750(	6.6091802	2.56E-10	up	yes	.	pfam13668	Ferritin_2	Ferritin-like	.	.	.	.
Manes.03G20760(	3.4800885	6.28E-12	up	yes	.	pfam13668	Ferritin_2	Ferritin-like	.	.	.	.
Manes.03G20770(	0.9058684	0.30462	down	no	30S ribosom	pfam01250	Ribosomal_S6	Ribosomal p	GO:0006412|	GO:0005840|	GO:0019843|rR	K02990
Manes.03G20780(	0.6934951	0.00018	down	no	DnaJ homolo	pfam14901	Jiv90	Cleavage in	GO:0015031|	GO:0005789|	.	K09534
Manes.03G20820(	0.4713414	0.00044	down	yes	.	pfam05405	Mt_ATP-synt_B	Mitochondr	.	.	.	.
Manes.03G20840(	8.6100965	6.23E-61	up	yes	Alkaline/neut	pfam12899	Glyco_hydro_100	Alkaline and	GO:0048364|	GO:0005829|	GO:0004564|be	.
Manes.03G20850(	14.923722	#######	up	yes	Transcription	pfam00249	Myb_DNA-binding	Myb-like DI	GO:0006952|	GO:0005634|	GO:0003677|DI	K09422
Manes.03G20860(	1.776176	1.85E-10	up	no	E3 ubiquitin-	pfam00632	HECT	HECT-dom	GO:0042787|	GO:0005737|	GO:0016874|lig	.
Manes.03G20870(	0.7838069	0.01596	down	no	Nuclear expo	pfam05833	FbpA	Fibronectin-	GO:0006611|	GO:0005634|	.	.
Manes.03G20880(	1.7910702	0.4079	up	no	Galactinol sy	pfam01501	Glyco_transf_8	Glycosyl tra	GO:0006012|	GO:0005737|	GO:0047216|in	K18819
Manes.03G20900(	0.9140678	0.35486	down	no	.	pfam07572	BCNT	Bucentaur o	.	.	.	.
Manes.03G20930(	0.8962652	0.2917	down	no	Probable beta	pfam01762	Galactosyl_T	Galactosyltr	GO:0006486|	GO:0000139|	GO:0030246|ca	.
Manes.03G20950(	1.0017876	0.96307	up	no	Putative vacu	pfam12624	Chorein_N	N-terminal r	GO:0045053|	GO:0019898|	.	.
Manes.03G20980(	0.9907841	0.9357	down	no	Vacuolar pro	pfam06650	DUF1162	Protein of u	GO:0045053|	GO:0005623|	.	.
Manes.03G20990(	0.1406989	4.45E-40	down	yes	Polygalacturo	pfam00295	Glyco_hydro_28	Glycosyl hy	GO:0005975|	GO:0005618|	GO:0004650|po	.
Manes.03G21010(	2.4932282	1.46E-17	up	yes	Probable boro	pfam00955	HCO3_cotransp	HCO3- tran	GO:0035445|	GO:0005887|	GO:0080139|bo	.
Manes.03G21020(	0.6048662	0.00012	down	no	DNA (cytosi	pfam00145	DNA_methylase	C-5 cytosin	GO:0050832|	GO:0005654|	GO:0003886|DI	.
Manes.03G21040(	Inf	0.1623	up	no	1-Cys peroxi	pfam00578	AhpC-TSA	AhpC/TSA	.	GO:0005634|	GO:0004601|pe	.
Manes.03G21060(	4.3990379	5.41E-65	up	yes	Calmodulin-b	pfam03859	CG-1	CG-1 domai	GO:0042742|	GO:0005634|	GO:0003677|DI	.
Manes.03G21070(	0.8751276	0.29603	down	no	Mediator of I	pfam11221	Med21	Subunit 21	GO:0050832|	GO:0016592|	.	K15152
Manes.03G21100(	2.8630859	0.0003	up	yes	.	pfam00917	MATH	MATH dom	.	.	.	.
Manes.03G21110(	0.8508929	0.10785	down	no	Pentatricope	pfam13041	PPR_2	PPR repeat	GO:0009793|	GO:0009507|	GO:0019843|rR	.
Manes.03G21130(	0.8184603	0.14674	down	no	Probable rece	pfam00069	Pkinase	Protein kina	.	GO:0016021|	GO:0005524|A	.
Manes.03G21140(	0.5712746	4.65E-07	down	no	Probable sug	pfam03151	TPT	Triose-phos	GO:0008643|	GO:0005794|	.	.
Manes.03G21150(	1.1233836	0.20578	up	no	Novel plant S	pfam07798	DUF1640	Protein of u	GO:0006888|	GO:0005789|	GO:0005484|SI	.
Manes.03G21160(	0.6700951	6.50E-05	down	no	[Pyruvate del	pfam10436	BCDHK_Adom3	Mitochondr	GO:0046777|	GO:0005739|	GO:0005524|A	K00898
Manes.03G21170(	0.760168	0.05918	down	no	rRNA-proces	pfam08698	Fcf2	Fcf2 pre-rR	GO:0006364|	GO:0005730|	.	.
Manes.03G21180(	1.0207921	0.82935	up	no	CBL-interact	pfam00069	Pkinase	Protein kina	GO:0035556|	GO:0005737|	GO:0005524|A	.
Manes.03G21190(	0.7118409	0.00324	down	no	.	pfam03108	DBD_Tnp_Mut	MuDR fami	.	.	.	.
Manes.03G21200(	0.7036554	0.01858	down	no	tRNA pseudo	pfam01416	PseudoU_synth_1	tRNA pseu	GO:0031119|	.	GO:0009982|ps	K06173
Manes.03G21210(	0.8469209	0.10831	down	no	Protein TRIC	pfam12600	DUF3769	Protein of u	GO:0034196|	GO:0009507|	GO:0070300|ph	.
Manes.03G21220(	0.8184547	0.10084	down	no	G-protein co	pfam05462	Dicty_CAR	Slime mold	GO:0009738|	GO:0005794|	GO:0004930|G	.
Manes.03G21230(	0.9211408	0.39863	down	no	Cytosolic pur	pfam05761	5_nucleotid	5' nucleotid	GO:0016311|	GO:0045335|	GO:0008253|5'	K01081
Manes.03G21260(	0.7674281	0.01136	down	no	Protein CHU	pfam04156	IncA	IncA protei	GO:0009902|	GO:0009507|	.	.
Manes.03G21280(	0.5703736	5.68E-06	down	no	SWI/SNF-rel	pfam00176	SNF2_N	SNF2 family	GO:0001525|	GO:0005634|	GO:0036310|an	K14440
Manes.03G21300(	0.1803719	7.66E-16	down	yes	.	pfam03772	Competence	Competence	.	.	.	.
Manes.03G21320(	0.7445743	0.01414	down	no	Putative este	pfam03061	4HBT	Thioesterase	.	.	GO:0016787|hy	.
Manes.04G00020(	0.7019554	0.00032	down	no	Serine/threon	pfam00069	Pkinase	Protein kina	GO:0043622|	GO:0055028|	GO:0005524|A	K08857
Manes.04G00050(	2.3127727	1.35E-21	up	yes	.	pfam15538	Toxin_61	Putative tox	.	.	.	.
Manes.04G00060(	1.968611	0.03098	up	no	NAC domain	pfam02365	NAM	No apical m	GO:0009910|	GO:0005783|	GO:0003677|DI	.
Manes.04G00080(	1.8351365	0.00013	up	no	Uncharacteriz	pfam01991	vATP-synt_E	ATP syntha	.	GO:0009507|	.	.
Manes.04G00130(	1.7143708	1.48E-08	up	no	RuvB-like pr	pfam06068	TIP49	TIP49 C-ter	GO:0030154|	GO:0009507|	GO:0005524|A	K04499
Manes.04G00140(	1.2304138	0.06477	up	no	.	pfam03899	ATP_synt_I	ATP syntha	.	.	.	.
Manes.04G00150(	0.6495147	1.33E-05	down	no	OTU domain	pfam02338	OTU	OTU-like cy	GO:0071108|	.	GO:0004843|ub	K12655
Manes.04G00170(	0.6593373	0.0653	down	no	Mitochondria	pfam06258	Mito_fiss_Elm1	Mitochondr	GO:0000266|	GO:0005741|	.	.
Manes.04G00180(	0.7699267	0.02601	down	no	Vesicle-asso	pfam00957	Synaptobrevin	Synaptobrev	GO:0006887|	GO:0005794|	GO:0005484|SI	K08515
Manes.04G00200(	3.8220659	0.00011	up	yes	NAC domain	pfam02365	NAM	No apical m	GO:0006351|	GO:0005634|	GO:0003677|DI	.
Manes.04G00230(	45.741813	7.07E-08	up	yes	Germin-like	pfam00190	Cupin_1	Cupin. This	.	GO:0048046|	GO:0030145|m	.
Manes.04G00250(	0.9916127	0.967	down	no	.	pfam05975	EcsB	Bacterial AI	.	.	.	.

Manes.04G00260(	6.5754206	3.45E-57	up	yes	Probable calc	pfam07223	DUF1421	Protein of u	.	.	GO:0005509|ca	K13448
Manes.04G00300(	0.5070817	2.23E-11	down	no	.	pfam11250	DUF3049	Protein of u	.	.	.	.
Manes.04G00390(	0.5780599	1.64E-05	down	no	Transmembra	pfam02990	EMP70	Endomembr	GO:0009624|	GO:0010008|	.	.
Manes.04G00410(	0.5662009	0.00141	down	no	TGACG-sequ	pfam14144	DOG1	Seed dorma	GO:0006351|	GO:0005634|	GO:0043565|se	.
Manes.04G00460(	0.5424607	4.54E-08	down	no	.	pfam12697	Abhydrolase_6	Alpha/beta l	.	.	.	.
Manes.04G00490(	0.4218491	2.18E-05	down	yes	CMP-N-acety	pfam00777	Glyco_transf_29	Glycosyltran	GO:0006054|	GO:0070062|	GO:0003836|be	K00780
Manes.04G00520(	Inf	4.07E-06	up	yes	Subtilisin-like	pfam00082	Peptidase_S8	Subtilase fa	GO:0010102|	GO:0005618|	GO:0004252|se	.
Manes.04G00570(	Inf	0.08088	up	no	Cucumisin	pfam00082	Peptidase_S8	Subtilase fa	.	GO:0005576|	GO:0004252|se	.
Manes.04G00590(	Inf	0.35135	up	no	.	pfam12409	P5-ATPase	P5-type AT	.	.	.	.
Manes.04G00630(	7.1440281	0.05166	up	no	.	pfam09810	Exo5	Exonuclease	.	.	.	.
Manes.04G00660(	0.7885347	0.10748	down	no	Serine/threon	pfam00069	Pkinase	Protein kina	GO:0009734|	GO:0005737|	GO:0005524|A	K08286
Manes.04G00670(	1.0242496	0.77667	up	no	Serine/threon	pfam00069	Pkinase	Protein kina	GO:0035556|	GO:0005737|	GO:0005524|A	.
Manes.04G00680(	1.9057013	2.48E-08	up	no	Putative low	pfam01451	LMWPc	Low molecu	.	.	GO:0004725|pr	K01104
Manes.04G00690(	0.7488856	0.00319	down	no	Alkaline/neut	pfam12899	Glyco_hydro_100	Alkaline and	GO:0048364|	GO:0005829|	GO:0004564|be	.
Manes.04G00720(	1.0904872	0.49002	up	no	.	pfam04949	Transcrip_act	Transcriptio	.	.	.	.
Manes.04G00730(	3.5611299	0.08133	up	no	UPF0481 pro	pfam03140	DUF247	Plant protein	.	GO:0016021|	.	.
Manes.04G00740(	1.6405388	0.00461	up	no	Ribosome-ina	pfam00161	RIP	Ribosome in	GO:0006952|	.	GO:0030598|rR	.
Manes.04G00800(	0.2291259	1.65E-05	down	yes	.	pfam05910	DUF868	Plant protein	.	.	.	.
Manes.04G00820(	2.8374426	6.81E-33	up	yes	Nucleobase-a	pfam00860	Xan_ur_permease	Permease fa	GO:0098702|	GO:0016021|	GO:0015207|ad	.
Manes.04G00830(	13.596115	#######	up	yes	Arogenate de	pfam00800	PDT	Prephenate	GO:0009094|	GO:0009507|	GO:0016597|an	K05359
Manes.04G00850(	3.8603421	9.92E-11	up	yes	.	pfam09191	CD4-extracel	CD4, extrac	.	.	.	.
Manes.04G00970(	4.7104049	5.50E-11	up	yes	.	pfam00860	Xan_ur_permease	Permease fa	.	.	.	.
Manes.04G00990(	0.3402023	9.48E-13	down	yes	.	pfam03479	DUF296	Domain of u	.	.	.	.
Manes.04G01000(	0.8462213	0.15016	down	no	.	pfam11326	DUF3128	Protein of u	.	.	.	.
Manes.04G01030(	0.4918286	5.60E-12	down	yes	Protein CHR	pfam02791	DDT	DDT domai	GO:0016568|	GO:0005634|	GO:0005524|A	.
Manes.04G01050(	0.5299299	3.35E-06	down	no	BTB/POZ do	pfam03000	NPH3	NPH3 famil	GO:0016567|	.	.	.
Manes.04G01080(	0.5699413	0.0038	down	no	.	pfam04768	DUF619	Protein of u	.	.	.	.
Manes.04G01090(	0.7847736	0.07357	down	no	Protein saal1	pfam04699	P16-Arc	ARP2/3 con	.	GO:0005615|	.	.
Manes.04G01190(	1.1732169	0.23979	up	no	Serine carbo	pfam00450	Peptidase_S10	Serine carbo	.	GO:0005576|	GO:0004185|se	K09646
Manes.04G01210(	9.4362052	6.37E-18	up	yes	RING-H2 fin	pfam13639	zf-RING_2	Ring finger	GO:0016567|	GO:0016021|	GO:0008270|zi	.
Manes.04G01240(	0.5885951	0.00257	down	no	.	pfam00227	Proteasome	Proteasome	.	.	.	.
Manes.04G01250(	0.7206238	0.00077	down	no	Pheophorbide	pfam08417	PaO	Pheophorbi	GO:0008219|	GO:0009507|	GO:0051537|2	K13071
Manes.04G01270(	45.933818	2.18E-17	up	yes	Zinc finger p	pfam00096	zf-C2H2	Zinc finger,	GO:0010447|	GO:0005634|	GO:0046872|m	.
Manes.04G01320(	0.58419	3.90E-05	down	no	Uncharacteri	pfam05495	zf-CHY	CHY zinc fi	GO:0016567|	.	GO:0061630|ub	.
Manes.04G01340(	0.6795187	0.01526	down	no	.	pfam06136	DUF966	Domain of u	.	.	.	.
Manes.04G01350(	0.2325501	1.57E-13	down	yes	.	pfam07795	DUF1635	Protein of u	.	.	.	.
Manes.04G01370(	0.8165146	0.12762	down	no	.	pfam01062	Bestrophin	Bestrophin,	.	.	.	.
Manes.04G01380(	1.3579433	0.00156	up	no	Probable gluc	pfam03016	Exostosin	Exostosin fa	GO:0071555|	GO:0005794|	GO:0015020|gl	.
Manes.04G01390(	0.2376427	1.05E-23	down	yes	Probable xylo	pfam00722	Glyco_hydro_16	Glycosyl hy	GO:0042546|	GO:0048046|	GO:0004553|hy	.
Manes.04G01400(	1.0603578	0.56346	up	no	Vacuolar pro	pfam03357	Snf7	Snf7. This f	GO:0070676|	GO:0005829|	.	K12193
Manes.04G01410(	3.8253315	0.43991	up	no	.	pfam01624	MutS_I	MutS domai	.	.	.	.
Manes.04G01490(	1.0344864	0.71218	up	no	Lactoylglutat	pfam00903	Glyoxalase	Glyoxalase/	.	.	GO:0004462|la	.
Manes.04G01510(	0.7588712	0.00448	down	no	4-coumarate-	pfam00501	AMP-binding	AMP-bindin	GO:0009695|	GO:0005777|	GO:0005524|A	K01904
Manes.04G01530(	0.5107255	0.04309	down	no	.	pfam00861	Ribosomal_L18p	Ribosomal l	.	.	.	.
Manes.04G01550(	1.5421126	3.84E-05	up	no	Dolichol kina	pfam01148	CTP_transf_1	Cytidylyltra	GO:0043048|	GO:0030176|	GO:0004168|do	K00902
Manes.04G01600(	1.9661645	8.90E-08	up	no	SNF1-related	pfam04739	AMPKBI	5'-AMP-acti	GO:0005975|	.	GO:0019887|pr	.
Manes.04G01660(	1.9052335	4.82E-06	up	no	Phosphatidyli	pfam08288	PIGA	PIGA (GPI	GO:0006501|	GO:0005789|	GO:0017176|ph	K03857
Manes.04G01670(	1.5318748	0.00173	up	no	Transcription	pfam03634	TCP	TCP family	GO:1900056|	GO:0005634|	GO:0003677|D	.
Manes.04G01690(	0.5170854	3.77E-06	down	no	OTU domain	pfam02338	OTU	OTU-like cy	.	.	GO:0008234|cy	.
Manes.04G01710(	4.2278766	1.59E-26	up	yes	Putative arse	pfam02374	ArsA_ATPase	Anion-trans	GO:0046685|	.	GO:0015446|ar	K01551
Manes.04G01740(	2.0313265	0.00079	up	yes	LysM domai	pfam00069	Pkinase	Protein kina	GO:0050832|	GO:0016021|	GO:0005524|A	.
Manes.04G01770(	NA	NA	--	no	.	pfam00981	Rota_NS53	Rotavirus R	.	.	.	.
Manes.04G01810(	NA	NA	--	no	.	pfam02326	YMF19	Plant ATP s	.	.	.	.
Manes.04G01820(	1.1000703	0.30201	up	no	Uncharacteri	pfam14624	Vwaint	VWA / Hh	.	.	.	K07114
Manes.04G01830(	8.2216177	3.28E-06	up	yes	.	pfam12734	CYSTM	Cysteine-ric	.	.	.	.
Manes.04G01840(	35.808638	2.40E-06	up	yes	Uncharacteri	pfam13456	RVT_3	Reverse tra	.	GO:0005739|	.	.
Manes.04G01940(	6.0355043	1.95E-10	up	yes	.	pfam13641	Glyco_tranf_2_3	Glycosyltra	.	.	.	.
Manes.04G01950(	0.4373934	8.63E-09	down	yes	.	pfam05212	DUF707	Protein of u	.	.	.	.
Manes.04G01970(	0.598982	2.40E-06	down	no	Protein-tyros	pfam00685	Sulfotransfer_1	Sulfotransfe	.	GO:0000139|	GO:0008476|pr	.
Manes.04G02020(	2.1560016	8.29E-18	up	yes	Double-stran	pfam00035	dsrm	Double-stra	GO:0035196|	GO:0005737|	GO:0003725|do	.
Manes.04G02110(	0.445367	0.01637	down	yes	.	pfam10271	Tmp39	Putative tra	.	.	.	.
Manes.04G02120(	1.4794245	1.13E-05	up	no	Probable aqu	pfam00230	MIP	Major intrin	.	GO:0016021|	GO:0005215|tra	K09872
Manes.04G02140(	0.5078237	0.00945	down	no	.	pfam00361	Oxidored_q1	NADH-Ubi	.	.	.	.
Manes.04G02150(	0.7633041	0.0323	down	no	Protein disul	pfam13848	Thioredoxin_6	Thioredoxin	GO:0045454|	GO:0005618|	GO:0003756|pr	.
Manes.04G02160(	0.6290916	0.00342	down	no	.	pfam11669	WBP-1	WW domain	.	.	.	.
Manes.04G02210(	0.6388903	7.50E-05	down	no	Serine/threon	pfam00069	Pkinase	Protein kina	GO:0006468|	.	GO:0005524|A	K07359
Manes.04G02230(	0.2203017	0.00263	down	yes	.	pfam05832	DUF846	Eukaryotic	.	.	.	.
Manes.04G02260(	2.0183101	4.62E-07	up	yes	Probable seri	pfam07727	RVT_2	Reverse tra	.	.	GO:0005524|A	.
Manes.04G02270(	3.5751361	4.57E-32	up	yes	Receptor-like	pfam13855	LRR_8	Leucine rich	GO:0009755|	GO:0016021|	GO:0004672|pr	.
Manes.04G02370(	2.3689292	1.31E-22	up	yes	Zinc finger p	pfam00096	zf-C2H2	Zinc finger,	GO:0051302|	GO:0005634|	GO:0003677|D	.
Manes.04G02410(	0.385368	0.25237	down	no	MLO-like pro	pfam03094	Mlo	Mlo family.	GO:0009817|	GO:0016021|	.	.
Manes.04G02440(	1.8735093	1.03E-11	up	no	Transmembra	pfam09786	CytochromB561_I	Cytochrome	.	GO:0016021|	.	.
Manes.04G02450(	0.7423457	0.0034	down	no	General trans	pfam04056	Ssl1	Ssl1-like. S	GO:0006370|	GO:0000439|	GO:0003676|nu	K03142
Manes.04G02460(	2.0057966	2.76E-15	up	yes	E3 SUMO-p	pfam02891	zf-MIZ	MIZ/SP-RII	GO:0051301|	GO:0016607|	GO:0016874|lig	.
Manes.04G02500(	19.719036	0.0232	up	yes	.	pfam06762	LMF1	Lipase matu	.	.	.	.
Manes.04G02530(	0.5297907	0.00032	down	no	LOB domain	pfam03195	DUF260	Protein of u	.	GO:0005739|	.	.
Manes.04G02540(	0.7139771	0.00086	down	no	Carbamoyl-p	pfam02786	CPSase_L_D2	Carbamoyl-	GO:0044205|	GO:0005951|	GO:0005524|A	K01955
Manes.04G02550(	3.6396098	2.12E-15	up	yes	Phosphatidyli	pfam02121	IP_trans	Phosphatidy	GO:0006810|	GO:0005794|	GO:0008289|lip	.
Manes.04G02560(	0.7592069	0.00522	down	no	Phosphoinosi	pfam00388	PI-PLC-X	Phosphatidy	GO:0035556|	GO:0005622|	GO:0004435|ph	K05857
Manes.04G02570(	0.2919072	1.05E-06	down	yes	tRNA-splicin	pfam01974	tRNA_int_endo	tRNA intron	GO:0006397|	GO:0005634|	GO:0016829|lya	K15322
Manes.04G02580(	2.2697899	1.51E-05	up	yes	Tetraspanin-	pfam00335	Tetraspannin	Tetraspanin	.	GO:0016021|	.	.
Manes.04G02610(	0.2438542	2.22E-05	down	yes	.	pfam00755	Carn_acyltransf	Choline/Car	.	.	.	.
Manes.04G02650(	0.9856429	0.90817	down	no	Arogenate de	pfam00800	PDT	Prephenate	GO:0009094|	GO:0009507|	GO:0016597|an	K05359
Manes.04G02660(	0.8610323	0.30469	down	no	NEDD8-spec	pfam02902	Peptidase_C48	Ulp1 protea	.	.	GO:0019784|N	K08597
Manes.04G02670(	18.122507	1.06E-33	up	yes	Mitogen-acti	pfam00069	Pkinase	Protein kina	GO:0009738|	GO:0005737|	GO:0005524|A	K04371
Manes.04G02680(	1.9564582	3.01E-06	up	no	L-ascorbate	pfam00141	peroxidase	Peroxidase.	GO:0042744|	GO:0005737|	GO:0020037|he	.
Manes.04G02720(	24.223853	1.32E-41	up	yes	.	pfam03202	Lipoprotein_10	Putative my	.	.	.	.
Manes.04G02750(	6.9539558	1.95E-71	up	yes	Probable seri	pfam07714	Pkinase_Tyr	Protein tyro	.	.	GO:0005524|A	.

Manes.04G02810(	Inf	0.08088	up	no	Putative resp	pfam08414	NADPH_Ox	Respiratory	.	GO:0016021|	GO:0005509|ca	.
Manes.04G02820(	0.7358517	0.00369	down	no	Nuclear facto	pfam09665	RE_Alw26IDE	Type II rest	GO:0006310|l	GO:0031011|	GO:0003677|Dl	K11671
Manes.04G02850(	Inf	0.9009	up	no	Chitinase 2	pfam00704	Glyco_hydro_18	Glycosyl hy	GO:0006032|	.	GO:0008061|ch	.
Manes.04G02860(	1.5873401	0.00025	up	no	Ribonuclease	pfam01876	RNase_P_p30	RNase P sul	GO:0090502|	GO:0005655|	GO:0044822|pc	K03539
Manes.04G02910(	1.1418099	0.14865	up	no	Probable NO	pfam04153	NOT2_3_5	NOT2 / NO	GO:0015074|	GO:0005634|	.	.
Manes.04G02930(	5.7288051	4.09E-13	up	yes	Ankyrin repe	pfam12796	Ank_2	Ankyrin rep	.	GO:0005783|	.	.
Manes.04G02940(	7.3270751	#######	up	yes	3-ketoacyl-C	pfam08392	FAE1_CUT1_Rpp	FAE1/Type	GO:0006633|	GO:0005783|	GO:0016747|tra	K15397
Manes.04G02950(	45.249932	4.72E-70	up	yes	Exocyst com	pfam03081	Exo70	Exo70 exoc	GO:0006887|	GO:0005618|	.	.
Manes.04G03030(	0.8406371	0.72107	down	no	.	pfam07795	DUF1635	Protein of u	.	.	.	.
Manes.04G03040(	1.2461397	0.22793	up	no	Aquaporin Tl	pfam00230	MIP	Major intrin	GO:0009992|	GO:0042807|	GO:0015254|gl	K09873
Manes.04G03070(	0.6817944	0.20525	down	no	.	pfam09451	ATG27	Autophagy-	.	.	.	.
Manes.04G03080(	#NAME?	0.43419	down	no	.	pfam02705	K_trans	K+ potassiu	.	.	.	.
Manes.04G03110(	207.45684	9.72E-71	up	yes	Zinc finger pi	pfam13912	zf-C2H2_6	C2H2-type	GO:0071289|	GO:0005634|	GO:0046872|m	.
Manes.04G03120(	1.1497891	0.13199	up	no	.	pfam07156	Prenylcys_lyase	Prenylcystei	.	.	.	.
Manes.04G03130(	3.3932912	5.90E-40	up	yes	Ras-related p	pfam00071	Ras	Ras family.	GO:0006886|	GO:0005829|	GO:0005525|G	K07901
Manes.04G03150(	1.7301929	7.27E-09	up	no	Histone H2B	pfam00125	Histone	Core histone	.	GO:0000786|	GO:0003677|Dl	K11252
Manes.04G03210(	0.252954	3.60E-06	down	yes	Cytochrome	pfam00067	p450	Cytochrome	.	GO:0016021|	GO:0020037|he	.
Manes.04G03230(	0.6536371	0.0096	down	no	.	pfam12491	ApoB100_C	Apolipoprot	.	.	.	.
Manes.04G03240(	Inf	0.04685	up	no	.	pfam14318	Mononeg_mRNA	Mononegav	.	.	.	.
Manes.04G03250(	1.5436154	1.33E-05	up	no	.	pfam01842	ACT	ACT domai	.	.	.	.
Manes.04G03290(	1.4471632	4.35E-05	up	no	UDP-glucuro	pfam01370	Epimerase	NAD depen	GO:0042732|	GO:0005829|	GO:0050662|co	K08678
Manes.04G03310(	0.5041432	1.08E-08	down	no	Phosphatidyl	pfam00650	CRAL_TRIO	CRAL/TRI	GO:0015031|	GO:0000139|	.	.
Manes.04G03360(	#NAME?	0.14049	down	no	WUSCHEL-r	pfam00046	Homeobox	Homeobox	GO:0010654|	GO:0005634|	GO:0003677|Dl	.
Manes.04G03370(	0.5760196	4.65E-06	down	no	.	pfam04238	DUF420	Protein of u	.	.	.	.
Manes.04G03390(	0.6812901	0.00358	down	no	Dof zinc fing	pfam02701	zf-Dof	Dof domain	GO:0006355|	GO:0005634|	GO:0046872|m	.
Manes.04G03400(	0.6278492	0.00046	down	no	Palmitoyltran	pfam01529	zf-DHHC	DHHC palm	GO:0042953|	GO:0005737|	GO:0042802|id	K18932
Manes.04G03420(	0.7391039	0.0025	down	no	Putative E3	pfam12796	Ank_2	Ankyrin rep	GO:0016567|	.	GO:0016874|lig	.
Manes.04G03430(	0.634433	0.00562	down	no	.	pfam00982	Glyco_transf_20	Glycosyltrar	.	.	.	.
Manes.04G03450(	1.7773765	6.44E-10	up	no	U-box domai	pfam00069	Pkinase	Protein kina	GO:0006950|	.	GO:0005524|A	.
Manes.04G03460(	2.0694462	2.49E-14	up	yes	Probable prot	pfam00481	PP2C	Protein phos	GO:0048366|	GO:0005634|	GO:0046872|m	.
Manes.04G03480(	2.8841766	4.14E-21	up	yes	.	pfam14309	DUF4378	Domain of u	.	.	.	.
Manes.04G03500(	0.9334256	0.51356	down	no	.	pfam13178	DUF4005	Protein of u	.	.	.	.
Manes.04G03520(	1.1704946	0.07916	up	no	Putative DEA	pfam00270	DEAD	DEAD/DEA	.	GO:0005634|	GO:0005524|A	K14808
Manes.04G03560(	0.3560331	3.18E-05	down	yes	F-box/kelch-r	pfam03154	Atrophin-1	Atrophin-1	GO:0016567|	GO:0005634|	.	.
Manes.04G03570(	NA	NA	--	no	.	pfam03101	FAR1	FAR1 DNA	.	.	.	.
Manes.04G03580(	2.5796602	1.75E-14	up	yes	Transcription	pfam00010	HLH	Helix-loop-l	GO:0006355|	GO:0005634|	GO:0003677|Dl	.
Manes.04G03590(	1.2707943	0.02154	up	no	Probable E3	pfam12428	DUF3675	Protein of u	GO:0042335|	GO:0016021|	GO:0016874|lig	K10661
Manes.04G03600(	2.205934	4.39E-11	up	yes	Transmembra	pfam05915	DUF872	Eukaryotic	.	GO:0016021|	.	.
Manes.04G03640(	5.5688097	9.07E-14	up	yes	.	pfam13405	EF-hand_6	EF-hand do	.	.	.	.
Manes.04G03660(	12.810398	2.76E-10	up	yes	.	pfam13597	NRDD	Anaerobic r	.	.	.	.
Manes.04G03680(	1.6181773	0.00026	up	no	Signal recogr	pfam02290	SRP14	Signal recog	GO:0006614|	GO:0005786|	GO:0008312|7S	K03104
Manes.04G03690(	0.956987	0.71865	down	no	Diphthamide	pfam01866	Diphthamide_syn	Putative dip	GO:0017183|	.	.	K17866
Manes.04G03700(	0.3282557	0.01411	down	yes	Cytochrome	pfam00067	p450	Cytochrome	.	GO:0016021|	GO:0020037|he	.
Manes.04G03720(	#NAME?	0.00658	down	yes	Cytochrome	pfam00067	p450	Cytochrome	.	GO:0016021|	GO:0020037|he	.
Manes.04G03750(	NA	NA	--	no	Cytochrome	pfam00067	p450	Cytochrome	.	GO:0016021|	GO:0020037|he	.
Manes.04G03790(	0.9191707	0.37109	down	no	Peptidyl-prol	pfam00254	FKBP_C	FKBP-type	GO:0006457|	GO:0009507|	GO:0003755|pe	K01802
Manes.04G03870(	0.5083293	0.00052	down	no	.	pfam02536	mTERF	mTERF. Th	.	.	.	.
Manes.04G03880(	0.389124	1.30E-08	down	yes	.	pfam02536	mTERF	mTERF. Th	.	.	.	.
Manes.04G03890(	6.6713426	5.99E-06	up	yes	.	pfam06257	DUF1021	Protein of u	.	.	.	.
Manes.04G03900(	1.7028631	2.11E-05	up	no	.	pfam02536	mTERF	mTERF. Th	.	.	.	.
Manes.04G03920(	5.0550596	0.00127	up	yes	.	pfam10421	OAS1_C	2'-5'-oligoa	.	.	.	.
Manes.04G03940(	13.112539	2.12E-05	up	yes	.	pfam01762	Galactosyl_T	Galactosyltr	.	.	.	.
Manes.04G03950(	2.6951345	1.28E-27	up	yes	Probable tran	pfam08614	ATG16	Autophagy	GO:0006351|	GO:0005737|	GO:0043565|se	.
Manes.04G03990(	0.4142007	6.68E-08	down	yes	Probable dise	pfam00931	NB-ARC	NB-ARC dc	GO:0006952|	.	GO:0043531|Al	.
Manes.04G04010(	0.4358362	1.74E-09	down	yes	Protein GAM	pfam00630	Filamin	Filamin/ABI	GO:0048235|	GO:0005887|	.	.
Manes.04G04090(	0.6066861	0.03632	down	no	.	pfam03372	Exo_endo_phos	Endonuclea	.	.	.	.
Manes.04G04150(	0.868274	0.17934	down	no	Probable E3	pfam01485	IBR	IBR domain	.	.	GO:0016874|lig	K11968
Manes.04G04230(	0.7030737	0.00325	down	no	Gamma-secre	pfam06105	Aph-1	Aph-1 prote	GO:0007219|	GO:0016021|	.	.
Manes.04G04250(	0.4649887	0.00653	down	yes	.	pfam13282	DUF4070	Domain of u	.	.	.	.
Manes.04G04260(	0.8278672	0.23101	down	no	.	pfam10567	Nab6_mRNP_bdg	RNA-recogi	.	.	.	.
Manes.04G04270(	1.0083316	0.88939	up	no	ATPase famil	pfam00004	AAA	ATPase fam	.	.	GO:0005524|A	.
Manes.04G04300(	2.8599624	3.66E-16	up	yes	Protein FAR1	pfam03101	FAR1	FAR1 DNA	GO:0006355|	GO:0005634|	GO:0008270|zi	.
Manes.04G04310(	0.7722954	0.0736	down	no	Protein FAR1	pfam03101	FAR1	FAR1 DNA	GO:0006355|	GO:0005634|	GO:0008270|zi	.
Manes.04G04330(	0.4415611	6.64E-07	down	yes	Protein FAR1	pfam03101	FAR1	FAR1 DNA	GO:0006355|	GO:0005634|	GO:0008270|zi	.
Manes.04G04360(	0.6996077	0.00282	down	no	Uncharacteri	pfam01985	CRS1_YhbY	CRS1 / Yhb	.	GO:0009507|	GO:0003723|Rl	.
Manes.04G04390(	1.7380798	2.45E-07	up	no	Protein YIF1	pfam03878	YIF1	YIF1. YIF1	.	GO:0016021|	.	.
Manes.04G04400(	1.3347307	0.00219	up	no	B-box zinc fi	pfam00643	zf-B_box	B-box zinc	GO:0080167|	GO:0005634|	GO:0003677|Dl	.
Manes.04G04480(	0.4677783	0.00212	down	yes	Glycerophos	pfam07714	Pkinase_Tyr	Protein tyro	GO:0006968|	GO:0016021|	GO:0005524|A	.
Manes.04G04510(	0.4318855	0.0691	down	no	.	pfam11557	DUF3233	Protein of u	.	.	.	.
Manes.04G04520(	0.3401184	0.00072	down	yes	Protein GAM	pfam00630	Filamin	Filamin/ABI	GO:0048235|	GO:0005887|	.	.
Manes.04G04530(	0.3341339	0.00207	down	yes	Protein GAM	pfam05086	Dicty_REP	Dictyosteliu	GO:0048235|	GO:0005887|	.	.
Manes.04G04600(	0.5018234	0.00025	down	no	Protein GAM	pfam00630	Filamin	Filamin/ABI	GO:0048235|	GO:0005887|	.	.
Manes.04G04650(	0.306179	1.34E-06	down	yes	Protein GAM	pfam00630	Filamin	Filamin/ABI	GO:0048235|	GO:0005887|	.	.
Manes.04G04710(	1.7843552	4.32E-10	up	no	.	pfam06075	DUF936	Plant protei	.	.	.	.
Manes.04G04770(	0.719918	0.04978	down	no	.	pfam04266	ASCH	ASCH doma	.	.	.	.
Manes.04G04780(	16.316073	0.00243	up	yes	.	pfam02351	GDNF	GDNF/GAS	.	.	.	.
Manes.04G04880(	1.1654836	0.17668	up	no	.	pfam07112	DUF1368	Protein of u	.	.	.	.
Manes.04G04890(	1.9265617	5.61E-08	up	no	Vesicle trans	pfam04178	Got1	Got1/Sft2-li	GO:0015031|	GO:0000139|	.	.
Manes.04G04960(	0.8833815	0.23928	down	no	Mitogen-acti	pfam00069	Pkinase	Protein kina	GO:0000187|	GO:0005622|	GO:0005524|A	.
Manes.04G05000(	2.8074128	2.84E-12	up	yes	Probable pre-	pfam07717	OB_NTP_bind	Oligonucleo	GO:0006397|	GO:0005829|	GO:0005524|A	K12818
Manes.04G05030(	2.3752634	9.56E-06	up	yes	.	pfam09605	Trep_Strep	Hypothetica	.	.	.	.
Manes.04G05060(	1.6529129	0.00025	up	no	.	pfam13041	PPR_2	PPR repeat	.	.	.	.
Manes.04G05080(	0.357075	5.74E-09	down	yes	.	pfam14897	EpsG	EpsG family	.	.	.	.
Manes.04G05100(	0.4721827	1.96E-08	down	yes	LysM domaiı	pfam01476	LysM	LysM doma	GO:0006952|	GO:0031225|	GO:0042834|pe	.
Manes.04G05110(	0.4598333	5.19E-14	down	yes	Abscisic-alde	pfam02738	Ald_Xan_dh_C2	Molybdopte	GO:0009688|	GO:0005829|	GO:0051537|2	K09842
Manes.04G05120(	0.9524628	0.71168	down	no	.	pfam14432	DYW_deaminase	DYW family	.	.	.	.
Manes.04G05150(	0.8474559	0.27055	down	no	.	pfam04084	ORC2	Origin recog	.	.	.	.
Manes.04G05160(	0.5495147	0.00606	down	no	Putative prot	pfam10536	PMD	Plant mobile	.	.	GO:0004252|se	.

Manes.04G05190(	Inf	0.09072	up	no	.	pfam01696	Adeno_E1B_55K	Adenovirus	.	.	.	.
Manes.04G05200(	1.7179709	3.04E-06	up	no	Protein ETH'	pfam04873	EIN3	Ethylene ins	GO:0071281|	GO:0005634|	GO:0003700|tr	K14514
Manes.04G05270(	0.3211649	1.67E-07	down	yes	Probable VA	pfam00957	Synaptobrevin	Synaptobrev	GO:0006810|	GO:0016021|	.	.
Manes.04G05370(	0.18867	4.98E-07	down	yes	.	pfam15272	BBP1_C	Spindle pole	.	.	.	.
Manes.04G05380(	0.7464439	0.01493	down	no	Non-structur	pfam07574	SMC_Nse1	Nse1 non-S	GO:0006310|	GO:0000781|	GO:0016874|lig	.
Manes.04G05440(	5.5452585	2.99E-17	up	yes	Pirin-like pro	pfam02678	Pirin	Pirin. This f	.	GO:0005634|	.	K06911
Manes.04G05450(	2.4755891	5.18E-21	up	yes	Transcription	pfam00010	HLH	Helix-loop-l	GO:0010119|	GO:0005829|	GO:0003700|tr	.
Manes.04G05460(	0.4732952	1.25E-05	down	yes	O-acyltransfe	pfam06974	DUF1298	Protein of u	GO:0019432|	GO:0005783|	GO:0004144|di	K15406
Manes.04G05490(	3.1121821	7.22E-23	up	yes	.	pfam00665	rve	Integrase co	.	.	.	.
Manes.04G05510(	0.6687946	3.87E-05	down	no	ENHANCER	pfam00855	PWWP	PWWP dom	GO:0030154|	GO:0005634|	GO:0003700|tr	.
Manes.04G05590(	1.6660377	1.36E-07	up	no	Succinyl-CoA	pfam02629	CoA_binding	CoA binding	GO:0006105|	GO:0005739|	GO:0005524|A	.
Manes.04G05660(	1.0390312	0.70521	up	no	BTB/POZ an	pfam00651	BTB	BTB/POZ d	GO:0071472|	GO:0005829|	.	K10523
Manes.04G05740(	0.1326445	4.49E-05	down	yes	.	pfam01107	MP	Viral mover	.	.	.	.
Manes.04G05760(	1.7586936	5.28E-05	up	no	Beta-xylosid	pfam00933	Glyco_hydro_3	Glycosyl hy	GO:0045493|	GO:0005578|	GO:0046556|al	.
Manes.04G05790(	1.6847842	1.16E-08	up	no	.	pfam00130	C1_1	Phorbol este	.	.	.	.
Manes.04G05800(	0.9532389	0.79129	down	no	.	pfam12726	SEN1_N	SEN1 N ter	.	.	.	.
Manes.04G05830(	0.8762506	0.27048	down	no	G-box-bindin	pfam00170	bZIP_1	bZIP transc	GO:0006351|	GO:0005634|	GO:0003677|D	K14432
Manes.04G06000(	8.8183188	3.74E-09	up	yes	Aluminum-ac	pfam11744	ALMT	Aluminium	GO:0015743|	GO:0016021|	.	.
Manes.04G06010(	0.409481	2.11E-17	down	yes	Transcription	pfam14215	bHLH-MYC_N	bHLH-MYC	GO:0010078|	GO:0005634|	GO:0003677|D	.
Manes.04G06020(	0.4283351	2.27E-08	down	yes	.	pfam00999	Na_H_Exchanger	Sodium/hyd	.	.	.	.
Manes.04G06030(	0.9420127	0.63451	down	no	Mitochondria	pfam11801	Tom37_C	Tom37 C-te	GO:0006626|	GO:0016021|	.	K17776
Manes.04G06040(	0.6845071	0.00012	down	no	Katanin p80	pfam13925	Katanin_con80	con80 doma	GO:0051013|	GO:0080008|	.	K18643
Manes.04G06050(	NA	NA	--	no	.	pfam06105	Aph-1	Aph-1 prote	.	.	.	.
Manes.04G06140(	1.0544312	0.71079	up	no	ATP-depend	pfam00574	CLP_protease	Clp protease	.	GO:0009535|	GO:0050897|co	K01358
Manes.04G06160(	1.1314303	0.17876	up	no	Probable imp	pfam03810	IBN_N	Importin-be	GO:0006886|	GO:0005737|	.	.
Manes.04G06220(	2.5637989	1.08E-23	up	yes	Protein FAF-	pfam11250	DUF3049	Protein of u	.	GO:0009507|	.	.
Manes.04G06240(	0.3906022	8.98E-12	down	yes	Probable WR	pfam05109	Herpes_BLLF1	Herpes viru	GO:0006952|	GO:0009941|	GO:0043531|A	.
Manes.04G06250(	1.0859207	0.47351	up	no	Large subuni	pfam11957	efThoc1	THO compl	GO:0051168|	GO:0015030|	GO:0005525|G	K14539
Manes.04G06270(	0.7850251	0.05232	down	no	Outer envelo	pfam01103	Bac_surface_Ag	Surface anti	GO:0009658|	GO:0009507|	.	.
Manes.04G06300(	0.3947415	5.96E-14	down	yes	DnaJ protein	pfam00226	DnaJ	DnaJ domai	GO:0006457|	GO:0005886|	GO:0005524|A	.
Manes.04G06330(	1.5007828	8.04E-06	up	no	50S ribosom	pfam00542	Ribosomal_L12	Ribosomal	GO:0006412|	GO:0009507|	GO:0003735|st	K02935
Manes.04G06340(	0.7414847	0.02386	down	no	.	pfam00684	DnaJ_CXXCXGX	DnaJ centra	.	.	.	.
Manes.04G06430(	0.88224	0.19711	down	no	Myosin-1	pfam00063	Myosin_head	Myosin hea	GO:0006897|	GO:0005783|	GO:0005524|A	.
Manes.04G06480(	0.8332866	0.4015	down	no	Metal-indepe	pfam00300	His_Phos_1	Histidine ph	GO:0070179|	.	GO:0004647|ph	K15634
Manes.04G06510(	0.3622618	1.34E-13	down	yes	Glycerol-3-p	pfam01553	Acyltransferase	Acyltransfer	GO:0016024|	GO:0005783|	GO:0003841|1-	K13506
Manes.04G06520(	0.6543503	0.06887	down	no	Uncharacteri	pfam10250	O-FucT	GDP-fucose	.	GO:0005768|	GO:0016757|tr	.
Manes.04G06540(	Inf	0.00042	up	yes	.	pfam14223	UBN2	gag-polypep	.	.	.	.
Manes.04G06610(	0.7398196	0.00378	down	no	Sacsin	pfam13920	zf-C3HC4_3	Zinc finger,	GO:0090084|	GO:0030424|	GO:0051087|ch	.
Manes.04G06630(	2.1863418	1.33E-05	up	yes	Probable leu	pfam00069	Pkinase	Protein kina	.	GO:0016021|	GO:0005524|A	.
Manes.04G06690(	4.1802648	1.17E-14	up	yes	Protein EXO	pfam04674	Phi_1	Phosphate-i	.	GO:0048046|	.	.
Manes.04G06700(	0.6919017	0.00487	down	no	Bromodomai	pfam00439	Bromodomain	Bromodoma	GO:0007596|	GO:0005829|	GO:0008270|zi	K11350
Manes.04G06710(	4.6948936	4.08E-49	up	yes	Probable seri	pfam07714	Pkinase_Tyr	Protein tyro	.	GO:0009507|	GO:0005524|A	.
Manes.04G06720(	0.8322779	0.07187	down	no	Probable gala	pfam01501	Glyco_transf_8	Glycosyl tra	GO:0071555|	GO:0005768|	GO:0047262|pc	.
Manes.04G06740(	0.7498084	0.00904	down	no	U-box domai	pfam04564	U-box	U-box doma	GO:0016567|	GO:0005737|	GO:0016874|lig	.
Manes.04G06760(	1.1717403	0.19271	up	no	Calcium-dep	pfam13833	EF-hand_8	EF-hand do	GO:0009738|	GO:0005737|	GO:0005524|A	K13412
Manes.04G06820(	3.674032	1.23E-44	up	yes	Expansin-like	pfam01357	Pollen_allerg_1	Pollen allerg	GO:0019953|	GO:0005576|	.	.
Manes.04G06870(	0.4082819	1.17E-08	down	yes	.	pfam07698	7TM-7TMR_HD	7TM recept	.	.	.	.
Manes.04G06880(	3.1757415	1.40E-09	up	yes	L-ascorbate	pfam00394	Cu-oxidase	Multicopper	.	GO:0005576|	GO:0005507|co	.
Manes.04G06890(	0.7808189	0.01195	down	no	Rop guanine	pfam03759	PRONE	PRONE (Pl	GO:0009860|	GO:0016324|	GO:0005089|R	.
Manes.04G06900(	0.7877568	0.04938	down	no	F-box protei	pfam03478	DUF295	Protein of u	GO:0016567|	GO:0005634|	.	.
Manes.04G06910(	4.5959434	0.04608	up	no	.	pfam05003	DUF668	Protein of u	.	.	.	.
Manes.04G06930(	2.1962473	5.96E-06	up	yes	Premnaspiro	pfam00067	p450	Cytochrome	.	GO:0016021|	GO:0020037|he	.
Manes.04G06950(	8.2440351	0.00827	up	yes	.	pfam05910	DUF868	Plant protei	.	.	.	.
Manes.04G06960(	4.1871743	2.32E-58	up	yes	Serine/threon	pfam07714	Pkinase_Tyr	Protein tyro	.	.	GO:0005524|A	.
Manes.04G06970(	3.8503746	0.39483	up	no	Mechanosens	pfam00924	MS_channel	Mechanoser	GO:0006811|	GO:0016021|	.	.
Manes.04G07000(	0.6154991	0.00059	down	no	.	pfam13041	PPR_2	PPR repeat	.	.	.	.
Manes.04G07080(	0.3915635	1.64E-06	down	yes	Probable poly	pfam13520	AA_permease_2	Amino acid	GO:1902047|	GO:0005887|	GO:0015297|an	.
Manes.04G07090(	3.3019119	0.00025	up	yes	.	pfam09852	DUF2079	Predicted m	.	.	.	.
Manes.04G07120(	1.5486904	1.10E-06	up	no	V-type proto	pfam00006	ATP-synt_ab	ATP syntha	GO:0015991|	GO:0033180|	GO:0005524|A	K02147
Manes.04G07130(	0.7328557	0.00485	down	no	.	pfam11833	DUF3353	Protein of u	.	.	.	.
Manes.04G07160(	0.8782391	0.26657	down	no	RNA-binding	pfam00076	RRM_1	RNA recog	GO:0006397|	GO:0005815|	GO:0000166|nu	K13091
Manes.04G07190(	1.473262	1.49E-05	up	no	Probable ubi	pfam00179	UQ_con	Ubiquitin-co	GO:0000209|	GO:0005737|	GO:0005524|A	K10581
Manes.04G07210(	1.0223314	0.77518	up	no	.	pfam05278	PEARLI-4	Arabidopsis	.	.	.	.
Manes.04G07280(	Inf	0.35135	up	no	.	pfam08863	YolD	YolD-like p	.	.	.	.
Manes.04G07300(	15.553181	3.90E-05	up	yes	.	pfam06813	Nodulin-like	Nodulin-like	.	.	.	.
Manes.04G07330(	0.9275089	0.51311	down	no	Protein PNS1	pfam04515	Choline_transpo	Plasma-men	GO:0006810|	GO:0016021|	.	.
Manes.04G07360(	3.0842293	0.01866	up	yes	Salicylate car	pfam03492	Methyltransf_7	SAM depen	.	.	GO:0046872|m	.
Manes.04G07370(	1.1862317	0.16986	up	no	Bifunctional	pfam00303	Thymidylat_synt	Thymidylate	GO:0006231|	.	GO:0004146|di	K13998
Manes.04G07420(	1.9327876	2.07E-06	up	no	.	pfam04825	Rad21_Rec8_N	N terminus	.	.	.	.
Manes.04G07430(	1.4855565	4.50E-05	up	no	Ocs element-	pfam00170	bZIP_1	bZIP transc	GO:0006351|	GO:0005634|	GO:0043565|se	.
Manes.04G07450(	1.3088539	0.00581	up	no	Subtilisin-like	pfam00082	Peptidase_S8	Subtilase fa	GO:0080001|	GO:0048046|	GO:0004252|se	.
Manes.04G07470(	1.2424339	0.25922	up	no	.	pfam06911	Senescence	Senescence-	.	.	.	.
Manes.04G07500(	1.1765262	0.08471	up	no	Synaptotagm	pfam00168	C2	C2 domain.	GO:0006897|	GO:0010008|	GO:0008289|lip	.
Manes.04G07510(	0.8904499	0.24953	down	no	Serine/threon	pfam07714	Pkinase_Tyr	Protein tyro	.	.	GO:0005524|A	.
Manes.04G07540(	4.4357518	7.70E-12	up	yes	Glycerophos	pfam00069	Pkinase	Protein kina	GO:0006968|	GO:0016021|	GO:0005524|A	.
Manes.04G07550(	8.2069325	6.03E-31	up	yes	Probable rec	pfam00069	Pkinase	Protein kina	.	GO:0016021|	GO:0005524|A	.
Manes.04G07560(	0.5948177	0.00037	down	no	Protein MCM	pfam09329	zf-primase	Primase zin	GO:0008283|	GO:0005737|	GO:0003688|D	K10736
Manes.04G07570(	1.9349252	1.48E-06	up	no	Rac-like GTI	pfam00071	Ras	Ras family.	GO:0007015|	GO:0005737|	GO:0005525|G	K04392
Manes.04G07610(	7.2799889	#######	up	yes	Probable BO	pfam13920	zf-C3HC4_3	Zinc finger,	GO:0006952|	GO:0005634|	GO:0016874|lig	K19042
Manes.04G07640(	0.532668	3.64E-08	down	no	CDT1-like pr	pfam08839	CDT1	DNA replica	GO:0007049|	GO:0009507|	GO:0004693|cy	K10727
Manes.04G07680(	2.2412633	1.38E-08	up	yes	Glycine-rich	pfam00313	CSD	'Cold-shock	GO:0006355|	.	GO:0003677|D	.
Manes.04G07690(	42.708505	8.70E-32	up	yes	.	pfam00514	Arm	Armadillo/b	.	.	.	.
Manes.04G07700(	3.3162341	1.64E-07	up	yes	Chaperone p	pfam00226	DnaJ	DnaJ domai	.	GO:0009507|	.	.
Manes.04G07730(	1.1234589	0.22407	up	no	ABC transpo	pfam00005	ABC_tran	ABC transp	.	GO:0016020|	GO:0051539|4	K06174
Manes.04G07770(	0.5160161	2.98E-10	down	no	Putative ami	pfam01425	Amidase	Amidase.	.	GO:0005829|	GO:0004040|an	K01426
Manes.04G07820(	1.6918595	9.50E-08	up	no	Protein XAP	pfam04921	XAP5	XAP5, circa	GO:0009793|	GO:0005634|	.	K13119
Manes.04G07830(	1.1886508	0.06651	up	no	Triosephosp	pfam00121	TIM	Triosephosp	GO:0006096|	GO:0009507|	GO:0004807|tri	.
Manes.04G07840(	1.9728584	0.01642	up	no	.	pfam13803	DUF4184	Domain of u	.	.	.	.

Manes.04G07860(	1.9023748	6.58E-10	up	no	ER lumen pro	pfam00810	ER_lumen_recept	ER lumen p	GO:0006621|p	GO:0005789|	GO:0046923|EI	K10949
Manes.04G07870(	6.147507	5.70E-09	up	yes	Chalcone syn	pfam00195	Chal_sti_synt_N	Chalcone an	GO:0009813|	.	GO:0016210|na	.
Manes.04G07900(	1.0083009	0.96818	up	no	.	pfam13499	EF-hand_7	EF-hand dor	.	.	.	.
Manes.04G07930(	2.1699801	6.70E-10	up	yes	Auxin-induce	pfam02519	Auxin_inducible	Auxin respo	GO:0009734|	.	.	.
Manes.04G08030(	0.3326777	0.01181	down	yes	Auxin-induce	pfam02519	Auxin_inducible	Auxin respo	GO:0009734|	.	.	.
Manes.04G08050(	0.2479692	7.01E-10	down	yes	Auxin-induce	pfam02519	Auxin_inducible	Auxin respo	GO:0009734|	.	.	.
Manes.04G08090(	3.1185626	2.27E-19	up	yes	Auxin-induce	pfam02519	Auxin_inducible	Auxin respo	GO:0009734|	.	.	.
Manes.04G08130(	0.4657159	0.02753	down	yes	Auxin-induce	pfam02519	Auxin_inducible	Auxin respo	GO:0009734|	.	.	.
Manes.04G08170(	8.5171124	9.30E-22	up	yes	.	pfam10327	7TM_GPCR_Sri	Serpentine t	.	.	.	.
Manes.04G08190(	1.2485086	0.32334	up	no	Auxin-induce	pfam02519	Auxin_inducible	Auxin respo	GO:0009734|	.	.	K14488
Manes.04G08200(	0.2728805	1.77E-05	down	yes	Auxin-induce	pfam02519	Auxin_inducible	Auxin respo	GO:0009734|	.	.	K14488
Manes.04G08210(	0.0301481	1.07E-05	down	yes	Auxin-induce	pfam02519	Auxin_inducible	Auxin respo	GO:0009734|	.	.	.
Manes.04G08240(	1.7111783	0.00039	up	no	Amidophospl	pfam00310	GATase_2	Glutamine a	GO:0006189|	GO:0009507|	GO:0004044|an	K00764
Manes.04G08260(	2.1627566	2.35E-17	up	yes	Transcription	pfam07716	bZIP_2	Basic regior	GO:0006351|	GO:0005634|	GO:0043565|se	.
Manes.04G08270(	1.6454448	6.85E-08	up	no	Protein BASI	pfam06217	GAGA_bind	GAGA bind	GO:0050793|	GO:0005634|	GO:0003677|DI	.
Manes.04G08300(	2.6825308	3.50E-24	up	yes	Arginine deca	pfam02784	Orn_Arg_deC_N	Pyridoxal-de	GO:0006527|	.	GO:0008792|ar	.
Manes.04G08340(	1.5206422	3.72E-06	up	no	NADH dehyo	pfam13232	Complex1_LYR_1	Complex1_I	GO:0005975|	GO:0031966|	GO:0008137|N.	K03965
Manes.04G08360(	0.6187077	6.29E-05	down	no	Kinesin-like p	pfam00225	Kinesin	Kinesin mot	GO:0000919|	GO:0005871|	GO:0005524|A'	.
Manes.04G08370(	0.5915454	2.66E-05	down	no	B-box zinc fi	pfam00643	zf-B_box	B-box zinc f	GO:0010100|	GO:0005634|	GO:0008270|zir	.
Manes.04G08390(	0.3917213	0.00378	down	yes	.	pfam01358	PARP_regulatory	Poly A poly	.	.	.	.
Manes.04G08410(	10.489255	#######	up	yes	Protein FMP	pfam07798	DUF1640	Protein of u	GO:0033617|	GO:0016021|	.	.
Manes.04G08430(	NA	NA	--	no	Cytochrome	pfam00067	p450	Cytochrome	GO:0009809|	GO:0005783|	GO:0020037|he	K09755
Manes.04G08440(	2.3903047	9.78E-21	up	yes	GATA transc	pfam00320	GATA	GATA zinc	GO:0045893|	GO:0005634|	GO:0043565|se	.
Manes.04G08470(	0.4630339	2.30E-05	down	yes	MATE efflux	pfam01554	MatE	MatE. The I	.	GO:0009507|	GO:0015297|an	.
Manes.04G08510(	0.7414638	0.00394	down	no	Gluconokinas	pfam02782	FGGY_C	FGGY famil	GO:0042732|	.	GO:0005524|A'	K00851
Manes.04G08520(	0.7768577	0.0095	down	no	Kinesin-relate	pfam00225	Kinesin	Kinesin mot	GO:0034613|	GO:0005737|	GO:0005524|A'	K11498
Manes.04G08530(	14.238698	1.92E-79	up	yes	B-box zinc fi	pfam00643	zf-B_box	B-box zinc f	GO:0006355|	GO:0005634|	GO:0003700|tra	.
Manes.04G08540(	1.0672187	0.47106	up	no	Coatomer sul	pfam06957	COPI_C	Coatomer (C	GO:0006886|	GO:0030126|	GO:0005198|stı	K05236
Manes.04G08550(	1.038372	0.67123	up	no	V-type proto	pfam01496	V_ATPase_I	V-type ATP	GO:0015991|	GO:0009507|	GO:0051117|A'	K02154
Manes.04G08560(	1.3882265	0.00127	up	no	Cysteine prot	pfam00112	Peptidase_C1	Papain famil	GO:0042742|	GO:0005615|	GO:0004197|cy	K01373
Manes.04G08570(	0.7830743	0.37304	down	no	.	pfam07734	FBA_1	F-box assoc	.	.	.	.
Manes.04G08580(	0.686043	0.02203	down	no	Probable prot	pfam01529	zf-DHHC	DHHC paln	.	GO:0016021|	GO:0019706|pr	.
Manes.04G08590(	Inf	0.35135	up	no	Cytochrome	pfam00067	p450	Cytochrome	.	GO:0016020|	GO:0020037|he	.
Manes.04G08610(	0.9274708	0.62594	down	no	Lysine-specif	pfam01426	BAH	BAH domai	.	.	GO:0003682|ch	K11445
Manes.04G08620(	Inf	0.35135	up	no	.	pfam15659	Toxin-JAB1	JAB-like to	.	.	.	.
Manes.04G08630(	1.7747051	1.36E-06	up	no	RNA-binding	pfam00076	RRM_1	RNA recogı	GO:0006397|	GO:0005730|	GO:0000166|nu	K14573
Manes.04G08640(	2.0349593	3.95E-10	up	yes	Serine/argini	pfam00076	RRM_1	RNA recogı	GO:0000398|	GO:0016607|	GO:0000166|nu	K12900
Manes.04G08650(	0.9768067	0.88189	down	no	SNF2 domair	pfam00176	SNF2_N	SNF2 family	.	GO:0005634|	GO:0005524|A'	K10875
Manes.04G08680(	0.8026662	0.04471	down	no	SUMO-activa	pfam00899	ThiF	ThiF family.	GO:0009793|	GO:0005829|	GO:0005524|A'	.
Manes.04G08740(	0.7001453	0.00522	down	no	Probable prot	pfam01529	zf-DHHC	DHHC paln	.	GO:0016021|	GO:0019706|pr	.
Manes.04G08750(	6.7914378	0.02038	up	yes	Serine/threon	pfam00069	Pkinase	Protein kina	GO:0006952|	GO:0005886|	GO:0005524|A'	K13430
Manes.04G08760(	0.5800163	4.95E-05	down	no	.	pfam07034	ORC3_N	Origin recog	.	.	.	.
Manes.04G08770(	#NAME?	0.60004	down	no	Exocyst com	pfam03081	Exo70	Exo70 exoc	GO:0006887|	GO:0005829|	.	.
Manes.04G08780(	1.0068711	0.9302	up	no	Regulation of	pfam04818	CTD_bind	RNA polym	GO:0070940|	GO:0016591|	GO:0000993|RI	K15559
Manes.04G08790(	0.5161714	2.44E-05	down	no	.	pfam01190	Pollen_Ole_e_I	Pollen prote	.	.	.	.
Manes.04G08800(	13.137914	8.89E-08	up	yes	Omega-hydro	pfam02458	Transferase	Transferase	GO:0052325|	.	GO:0050734|hy	K15400
Manes.04G08820(	1.0060015	0.92903	up	no	.	pfam02213	GYF	GYF domai	.	.	.	.
Manes.04G08880(	0.4659897	3.55E-10	down	yes	Transcription	pfam00010	HLH	Helix-loop-l	GO:0009911|	GO:0005634|	GO:0003677|DI	.
Manes.04G08900(	0.4723859	0.00026	down	yes	Laccase-11	pfam07732	Cu-oxidase_3	Multicopper	GO:0046274|	GO:0048046|	GO:0005507|co	K05909
Manes.04G08940(	1.9550102	9.83E-14	up	no	Protein RER	pfam03248	Rer1	Rer1 family.	GO:0006890|	GO:0005801|	.	.
Manes.04G08950(	7.6197156	#######	up	yes	F-box proteir	pfam06728	PIG-U	GPI transan	GO:0009658|	.	.	.
Manes.04G08970(	3.7533112	0.39312	up	no	.	pfam00892	EamA	EamA-like t	.	.	.	.
Manes.04G08980(	19.541252	4.14E-42	up	yes	Glycerophos	pfam03009	GDPD	Glycerophos	GO:0030643|	GO:0009507|	GO:0008889|gl	.
Manes.04G09000(	NA	NA	--	no	Putative ribo	pfam13966	zf-RVT	zinc-binding	.	.	GO:0046872|m	.
Manes.04G09020(	#NAME?	0.01741	down	yes	.	pfam14228	MOR2-PAG1_mic	Cell morphc	.	.	.	.
Manes.04G09040(	0.3884696	1.64E-16	down	yes	.	pfam13889	Chromosome_seg	Chromosom	.	.	.	.
Manes.04G09070(	#NAME?	1	down	no	CASP-like pr	pfam04535	DUF588	Domain of u	.	GO:0016021|	.	.
Manes.04G09130(	Inf	0.9009	up	no	.	pfam00915	Calici_coat	Calicivirus c	.	.	.	.
Manes.04G09150(	0.6854308	0.00041	down	no	Protein AUX	pfam12937	F-box-like	F-box-like.	GO:0009734|	GO:0005634|	GO:0010011|au	.
Manes.04G09160(	0.435949	3.29E-05	down	yes	.	pfam02138	Beach	Beige/BEAC	.	.	.	.
Manes.04G09190(	1.41821	0.00015	up	no	V-type proto	pfam03223	V-ATPase_C	V-ATPase s	GO:0015991|	GO:0009507|	GO:0015078|hy	K02148
Manes.04G09200(	1.8237099	1.36E-11	up	no	Mitochondria	pfam00153	Mito_carr	Mitochondr	GO:0009651|	GO:0005618|	.	K15102
Manes.04G09220(	1.427764	0.03365	up	no	Pentatricopeı	pfam14432	DYW_deaminase	DYW family	.	GO:0005739|	.	.
Manes.04G09230(	0.6286963	0.00308	down	no	.	pfam00300	His_Phos_1	Histidine ph	.	.	.	.
Manes.04G09250(	5.6435514	0.03943	up	no	Thioredoxin	pfam00085	Thioredoxin	Thioredoxin	GO:0045454|	GO:0005737|	GO:0015035|pr	.
Manes.04G09270(	32.880099	2.50E-54	up	yes	.	pfam13041	PPR_2	PPR repeat	.	.	.	.
Manes.04G09300(	2.0278345	5.20E-15	up	yes	Alpha-manno	pfam01074	Glyco_hydro_38	Glycosyl hy	GO:0006013|	GO:0000326|	GO:0004559|alı	.
Manes.04G09350(	Inf	0.1623	up	no	F-box proteir	pfam08268	FBA_3	F-box assoc	GO:0031348|	GO:0005737|	.	.
Manes.04G09360(	0.9548568	0.64399	down	no	Nuclear facto	pfam14897	EpsG	EpsG family	.	GO:0031011|	GO:0003677|DI	K11671
Manes.04G09380(	0.6064943	0.001	down	no	.	pfam12576	DUF3754	Protein of u	.	.	.	.
Manes.04G09430(	NA	NA	--	no	.	pfam04356	DUF489	Protein of u	.	.	.	.
Manes.04G09450(	0.5132211	4.18E-06	down	no	Nudix hydrol	pfam00293	NUDIX	NUDIX dor	.	GO:0005739|	GO:0016787|hy	.
Manes.04G09480(	Inf	1.63E-15	up	yes	Lipid transfer	pfam14368	LTP_2	Probable lip	GO:0006810|	GO:0031225|	GO:0008289|lip	.
Manes.04G09490(	0.9054322	0.41272	down	no	Syntaxin-22	pfam14523	Syntaxin_2	Syntaxin-lik	GO:0009660|	GO:0012505|	GO:0000149|SI	K08488
Manes.04G09580(	1.1738001	0.09362	up	no	Acetyl-coenz	pfam00501	AMP-binding	AMP-bindir	GO:0006083|	GO:0009507|	GO:0003987|ac	K01895
Manes.04G09590(	0.6727386	0.02419	down	no	Acetyl-coenz	pfam00501	AMP-binding	AMP-bindir	GO:0006083|	GO:0009507|	GO:0003987|ac	K01895
Manes.04G09600(	0.5526437	1.03E-06	down	no	WD repeat-c	pfam00400	WD40	WD domain	GO:0009718|	GO:0005634|	GO:0044212|tra	K11805
Manes.04G09630(	1.2255599	0.09761	up	no	Glyceraldehy	pfam00044	Gp_dh_N	Glyceraldeh	GO:0006006|	GO:0009507|	GO:0047100|gl	.
Manes.04G09650(	2.0518869	0.00658	up	yes	.	pfam12520	DUF3723	Protein of u	.	.	.	.
Manes.04G09670(	0.941861	0.632	down	no	DEAD-box A	pfam00270	DEAD	DEAD/DEA	.	.	GO:0005524|A'	.
Manes.04G09680(	1.1223123	0.49418	up	no	.	pfam05553	DUF761	Cotton fibre	.	.	.	.
Manes.04G09700(	0.6241412	0.00033	down	no	.	pfam00168	C2	C2 domain.	.	.	.	.
Manes.04G09710(	0.4358725	0.00148	down	yes	Pentatricopeı	pfam14432	DYW_deaminase	DYW family	.	GO:0005739|	.	.
Manes.04G09770(	0.7553207	0.45182	down	no	Putative unc	pfam01485	IBR	IBR domain	.	GO:0009507|	GO:0005524|A'	K12818
Manes.04G09800(	Inf	2.81E-09	up	yes	.	pfam03074	GCS	Glutamate-c	.	.	.	.
Manes.04G09830(	0.491513	2.77E-06	down	yes	.	pfam12767	SAGA-Tad1	Transcriptio	.	.	.	.
Manes.04G09840(	0.7617983	0.02591	down	no	Putative gluc	pfam01263	Aldose_epim	Aldose 1-ep	GO:0005975|	.	GO:0030246|ca	.
Manes.04G09890(	1.6605904	1.23E-08	up	no	Probable seri	pfam03348	Serinc	Serine incor	GO:0008654|	GO:0005789|	.	.

Manes.04G09910(	0.3975318	8.91E-05	down	yes	Receptor-like	pfam00069	Pkinase	Protein kina	GO:0006468|	GO:0005576|	GO:0005524|A'	.
Manes.04G09930(	0.7186025	0.0216	down	no	Receptor-like	pfam00069	Pkinase	Protein kina	GO:0006468|	GO:0005576|	GO:0005524|A'	.
Manes.04G09950(	0.332002	0.01723	down	yes	Wall-associat	pfam00069	Pkinase	Protein kina	GO:0009755|	GO:0016021|	GO:0005524|A'	.
Manes.04G09980(	0.2574782	0.00013	down	yes	.	pfam01697	Glyco_transf_92	Glycosyltrar	.	.	.	.
Manes.04G10020(	3.7905435	5.03E-11	up	yes	Wall-associat	pfam00069	Pkinase	Protein kina	.	GO:0016021|	GO:0005524|A'	.
Manes.04G10030(	Inf	6.08E-09	up	yes	Wall-associat	pfam00069	Pkinase	Protein kina	GO:0009755|	GO:0016021|	GO:0005524|A'	.
Manes.04G10070(	3.4359355	3.11E-26	up	yes	AP2-like ethy	pfam00847	AP2	AP2 domair	GO:0009873|	GO:0005634|	GO:0003677|DI	K09285
Manes.04G10130(	2.0332771	0.00215	up	yes	Trafficking pi	pfam04628	Sedlin_N	Sedlin, N-te	GO:0006888|	GO:0005783|	.	.
Manes.04G10150(	5.9385844	4.37E-84	up	yes	Magnesium tı	pfam01544	CorA	CorA-like M	GO:0098655|	GO:0016021|	GO:0015095|ma	K16075
Manes.04G10160(	17.667956	#######	up	yes	Chaperone pı	pfam00226	DnaJ	DnaJ domai	GO:0042026|	GO:0009507|	GO:0051087|ch	.
Manes.04G10170(	2.7723649	2.52E-18	up	yes	Shikimate O-	pfam02458	Transferase	Transferase	GO:0010252|	GO:0005737|	GO:0047205|qu	K13065
Manes.04G10210(	0.3020009	1.62E-09	down	yes	Putative rece	pfam00069	Pkinase	Protein kina	.	GO:0016021|	GO:0005524|A'	.
Manes.04G10230(	0.6932761	0.05283	down	no	Phosphatidyl	pfam04191	PEMT	Phospholipi	GO:0006656|	GO:0016021|	GO:0080101|ph	K00550
Manes.04G10260(	80.93864	7.30E-52	up	yes	Probable WR	pfam03106	WRKY	WRKY DN	GO:0042742|	GO:0005634|	GO:0043565|se	.
Manes.04G10330(	0.5585031	7.95E-08	down	no	Sister chroma	pfam02949	7tm_6	7tm Odoran	GO:0051301|	GO:0000794|	GO:0005198|stı	K11267
Manes.04G10340(	0.9163799	0.49154	down	no	Putative U-b	pfam04564	U-box	U-box doma	.	.	GO:0016874|lig	.
Manes.04G10350(	0.8777625	0.20765	down	no	.	pfam08737	Rgp1	Rgp1. Rgp1	.	.	.	.
Manes.04G10420(	0.7019874	0.00215	down	no	.	pfam12928	tRNA_int_end_N2	tRNA-splici	.	.	.	.
Manes.04G10470(	0.7534795	0.00367	down	no	Nuclear pore	pfam12110	Nup96	Nuclear pro	GO:0006952|	GO:0005635|	GO:0015288|pc	K14297
Manes.04G10480(	2.9406191	1.43E-29	up	yes	Protein RAE	pfam00400	WD40	WD domain	GO:0051028|	GO:0080008|	.	K14298
Manes.04G10500(	0.4271526	0.02624	down	yes	Ethylene-resp	pfam00847	AP2	AP2 domair	GO:0009873|	GO:0005634|	GO:0003677|DI	.
Manes.04G10570(	13.187594	1.14E-05	up	yes	.	pfam15046	DUF4532	Protein of u	.	.	.	.
Manes.04G10580(	14.689052	4.02E-12	up	yes	Pleiotropic dı	pfam01061	ABC2_membrane	ABC-2 type	GO:0006810|	GO:0016021|	GO:0005524|A'	.
Manes.04G10610(	1.9004528	6.39E-10	up	no	Pleiotropic dı	pfam01061	ABC2_membrane	ABC-2 type	GO:0006810|	GO:0016021|	GO:0005524|A'	.
Manes.04G10620(	0.6777482	0.00453	down	no	Lupeol synth	pfam13243	Prenyltrans_1	Prenyltransf	GO:0042335|	.	GO:0042299|luj	.
Manes.04G10630(	0.7350656	0.10392	down	no	Lupeol synth	pfam13249	Prenyltrans_2	Prenyltransf	GO:0042335|	.	GO:0042299|luj	.
Manes.04G10660(	0.7420309	0.00728	down	no	BRCA1-asso	pfam07576	BRAP2	BRCA1-ass	GO:0000165|	GO:0005737|	GO:0016874|lig	K10632
Manes.04G10760(	Inf	0.00115	up	yes	Peroxidase 5	pfam00141	peroxidase	Peroxidase.	GO:0042744|	GO:0005576|	GO:0020037|he	.
Manes.04G10800(	4.9191928	8.36E-15	up	yes	.	pfam07782	DC_STAMP	DC-STAMI	.	.	.	.
Manes.04G10820(	0.9185958	0.44005	down	no	.	pfam06813	Nodulin-like	Nodulin-like	.	.	.	.
Manes.04G10830(	0.4170239	0.05204	down	no	.	pfam07647	SAM_2	SAM domai	.	.	.	.
Manes.04G10860(	0.9719324	0.78572	down	no	Topless-relat	pfam00400	WD40	WD domain	GO:0010072|	GO:0005829|	.	.
Manes.04G10890(	16.204275	1.24E-08	up	yes	.	pfam10373	EST1_DNA_bind	Est1 DNA/I	.	.	.	.
Manes.04G10900(	2.2312455	3.57E-05	up	yes	.	pfam09762	KOG2701	Coiled-coil	.	.	.	.
Manes.04G10910(	0.67654	0.00654	down	no	Triphosphate	pfam01928	CYTH	CYTH dom	GO:0048364|	GO:0005634|	GO:0005524|A'	.
Manes.04G10920(	0.8859835	0.21715	down	no	DEAD-box A	pfam00270	DEAD	DEAD/DEA	.	.	GO:0005524|A'	K13116
Manes.04G10940(	9.6035618	1.35E-16	up	yes	.	pfam04706	Dickkopf_N	Dickkopf N	.	.	.	.
Manes.04G10960(	9.2921305	5.45E-28	up	yes	.	pfam13166	AAA_13	AAA domai	.	.	.	.
Manes.04G10990(	0.9787986	0.8429	down	no	Pyrophospha	pfam03030	H_PPase	Inorganic H	GO:0015992|	GO:0016021|	GO:0009678|hy	.
Manes.04G11060(	0.6320141	5.56E-05	down	no	Clustered mit	pfam12807	eIF3_p135	Translation	GO:0048312|	GO:0005737|	.	K03255
Manes.04G11090(	2.2090896	9.21E-09	up	yes	Haloalkane d	pfam12697	Abhydrolase_6	Alpha/beta l	GO:0019260|	.	GO:0018786|ha	.
Manes.04G11110(	0.9114751	0.42421	down	no	Protein FAR	pfam10551	MULE	MULE trans	GO:0006355|	GO:0005634|	GO:0008270|zi	.
Manes.04G11120(	0.5043745	7.95E-08	down	no	Protein FAR	pfam03101	FAR1	FAR1 DNA	GO:0006355|	GO:0005634|	GO:0008270|zi	.
Manes.04G11150(	3.4931346	1.85E-36	up	yes	Zinc finger B	pfam14372	DUF4413	Domain of u	GO:0009791|	GO:0005829|	GO:0003677|DI	.
Manes.04G11190(	0.2313339	1.17E-05	down	yes	.	pfam02845	CUE	CUE domai	.	.	.	.
Manes.04G11220(	1.6515172	2.61E-05	up	no	E3 ubiquitin-	pfam00069	Pkinase	Protein kina	GO:0009738|	GO:0005769|	GO:0005524|A'	.
Manes.04G11250(	Inf	1.99E-08	up	yes	.	pfam14609	GCP5-Mod21	gamma-Tub	.	.	.	.
Manes.04G11280(	0.5309993	1.70E-06	down	no	Palmitoyl-mo	pfam00487	FA_desaturase	Fatty acid de	GO:0031408|	GO:0031969|	GO:0016717|ox	K00507
Manes.04G11300(	Inf	0.00079	up	yes	Cucumber pe	pfam02298	Cu_bind_like	Plastocyanir	GO:0055114|	.	GO:0009055|ele	.
Manes.04G11310(	31.332459	5.60E-97	up	yes	Glucan endo-	pfam00332	Glyco_hydro_17	Glycosyl hy	GO:0005975|	GO:0046658|	GO:0042973|glı	.
Manes.04G11330(	0.6500608	0.00306	down	no	G-type lectin	pfam00069	Pkinase	Protein kina	GO:0009755|	GO:0016021|	GO:0005524|A'	.
Manes.04G11340(	1.3124802	0.07783	up	no	.	pfam13414	TPR_11	TPR repeat.	.	.	.	.
Manes.04G11360(	7.8248403	2.49E-25	up	yes	26.5 kDa hea	pfam00011	HSP20	Hsp20/alpha	GO:0009408|	GO:0005739|	.	K13993
Manes.04G11370(	1.7958483	1.10E-10	up	no	.	pfam13259	DUF4050	Protein of u	.	.	.	.
Manes.04G11380(	1.4886105	0.00382	up	no	.	pfam00519	PPV_E1_C	Papillomavi	.	.	.	.
Manes.04G11440(	1.0140762	0.94944	up	no	.	pfam06232	ATS3	Embryo-spe	.	.	.	.
Manes.04G11450(	0.4806582	2.94E-05	down	yes	DEAD-box A	pfam00270	DEAD	DEAD/DEA	GO:0006397|	GO:0005634|	GO:0005524|A'	.
Manes.04G11560(	4.9035736	1.45E-30	up	yes	Probable dise	pfam00931	NB-ARC	NB-ARC dc	GO:0006952|	.	GO:0043531|Al	.
Manes.04G11570(	45.047791	3.85E-08	up	yes	.	pfam12681	Glyoxalase_2	Glyoxalase-	.	.	.	.
Manes.04G11600(	0.4688409	4.74E-11	down	yes	Putative UDI	pfam00201	UDPGT	UDP-glucor	.	GO:0016021|	GO:0016758|tra	.
Manes.04G11620(	3.7533112	0.39312	up	no	.	pfam03181	BURP	BURP doma	.	.	.	.
Manes.04G11630(	0.8047475	0.04211	down	no	Biotin carbox	pfam00364	Biotin_lipoyl	Biotin-requi	GO:0006633|	GO:0009317|	GO:0003989|ac	K02160
Manes.04G11670(	2.0911376	3.11E-15	up	yes	.	pfam04003	Utp12	Dip2/Utp12	.	.	.	.
Manes.04G11710(	2.3174088	5.35E-14	up	yes	Periodic tryp	pfam04003	Utp12	Dip2/Utp12	GO:0000462|	GO:0005737|	GO:0044822|pc	K14558
Manes.04G11730(	0.7661881	0.06101	down	no	Acyl-protein	pfam02230	Abhydrolase_2	Phospholipa	GO:0006631|	GO:0005737|	GO:0016787|hy	K06130
Manes.04G11750(	0.6572011	8.21E-05	down	no	(6-4)DNA pł	pfam03441	FAD_binding_7	FAD binding	GO:0006281|	.	GO:0003914|DI	K02295
Manes.04G11850(	0.8163308	0.05943	down	no	Serine-threoı	pfam00400	WD40	WD domain	GO:0030512|	.	.	K13137
Manes.04G11890(	Inf	0.08088	up	no	.	pfam09815	XK-related	XK-related	.	.	.	.
Manes.04G11930(	1.720001	3.22E-05	up	no	Pentatricopej	pfam14432	DYW_deaminase	DYW family	GO:0009658|	GO:0009507|	.	.
Manes.04G11970(	NA	NA	--	no	Probable 2-o:	pfam03171	2OG-FeII_Oxy	2OG-Fe(II)	.	.	GO:0046872|me	.
Manes.04G12040(	0.4989541	8.69E-11	down	yes	Katanin p60	pfam00004	AAA	ATPase fam	GO:0043622|	GO:0005737|	GO:0005524|A'	K07767
Manes.04G12050(	0.5781108	8.36E-05	down	no	Aspartate am	pfam00155	Aminotran_1_2	Aminotransf	GO:0009058|	GO:0005737|	GO:0004069|L-	K00812
Manes.04G12080(	5.0675048	2.40E-22	up	yes	Auxin-respor	pfam02309	AUX_IAA	AUX/IAA f	GO:0009734|	GO:0005634|	GO:0003700|tra	K14484
Manes.04G12100(	2.3159468	1.98E-20	up	yes	ABC transpo	pfam00005	ABC_tran	ABC transp	.	.	GO:0005524|A'	K06184
Manes.04G12120(	1.2977146	0.00402	up	no	N-alpha-acet	pfam12569	NARP1	NMDA rece	.	GO:0005737|	.	K00670
Manes.04G12160(	15.177662	4.74E-31	up	yes	F-box/kelch-	pfam01344	Kelch_1	Kelch motif	GO:0080037|	GO:0005829|	.	.
Manes.04G12240(	1.5063856	0.00023	up	no	Thionin-like	pfam05522	Metallothio_6	Metallothior	GO:0006952|	GO:0005576|	.	.
Manes.04G12260(	1.520529	3.41E-06	up	no	Coatomer sul	pfam04053	Coatomer_WDAD	Coatomer W	GO:0006886|	GO:0030663|	GO:0005198|stı	.
Manes.04G12270(	1.7271647	0.04072	up	no	Coatomer sul	pfam00400	WD40	WD domain	GO:0006886|	GO:0030663|	GO:0005198|stı	.
Manes.04G12280(	3.7740858	0.00024	up	yes	Probable sulf	pfam00916	Sulfate_transp	Sulfate trans	.	GO:0016021|	GO:0008271|se	K17471
Manes.04G12310(	0.3046785	1.86E-23	down	yes	.	pfam04570	DUF581	Protein of u	.	.	.	.
Manes.04G12330(	1.8057022	3.28E-09	up	no	.	pfam13041	PPR_2	PPR repeat	.	.	.	.
Manes.04G12390(	0.7515352	0.04209	down	no	Trihelix trans	pfam13837	Myb_DNA-bind_4	Myb/SANT	GO:0009793|	GO:0005730|	GO:0003700|tra	.
Manes.04G12400(	2.3309874	5.81E-13	up	yes	Ras-related p	pfam00071	Ras	Ras family.	GO:0015031|	GO:0005622|	GO:0005525|G'	.
Manes.04G12410(	0.8076711	0.02465	down	no	Probable glut	pfam02798	GST_N	Glutathione	GO:0009734|	.	GO:0004364|glı	.
Manes.04G12420(	0.3447888	3.12E-07	down	yes	Probable glut	pfam13417	GST_N_3	Glutathione	GO:0009734|	.	GO:0004364|glı	.
Manes.04G12460(	0.8024143	0.02822	down	no	.	pfam09072	TMA7	Translation	.	.	.	.
Manes.04G12470(	Inf	3.97E-09	up	yes	Mitochondria	pfam00153	Mito_carr	Mitochondr	GO:1903826|	GO:0016021|	GO:0015181|ar	K15109

Manes.04G12490(	0.3703132	6.80E-10	down	yes	Kinesin-13A	pfam00225	Kinesin	Kinesin mot	GO:0007018|ı	GO:0005795|	GO:0005524|A	K10393
Manes.04G12540(	0.7366316	0.00907	down	no	25.3 kDa ves	pfam13774	Longin	Regulated-S	GO:0006887|(	GO:0005783|	GO:0005484|SI	K08517
Manes.04G12600(	5.660566	3.23E-49	up	yes	Proline-rich r	pfam00069	Pkinase	Protein kina	.	GO:0016021|	GO:0005524|A	.
Manes.04G12610(	4.6867888	0.00122	up	yes	Proline-rich r	pfam00069	Pkinase	Protein kina	GO:0009755|l	GO:0016021|	GO:0005524|A	.
Manes.04G12630(	5.6186706	9.46E-43	up	yes	Proline-rich r	pfam00069	Pkinase	Protein kina	GO:0009755|l	GO:0016021|	GO:0005524|A	.
Manes.04G12640(	3.2450937	9.61E-14	up	yes	Proline-rich r	pfam00069	Pkinase	Protein kina	.	GO:0016021|	GO:0005524|A	.
Manes.04G12650(	8.5300341	3.70E-05	up	yes	Proline-rich r	pfam07714	Pkinase_Tyr	Protein tyro	.	GO:0016021|	GO:0005524|A	.
Manes.04G12660(	5.16085	3.88E-11	up	yes	Proline-rich r	pfam07714	Pkinase_Tyr	Protein tyro	.	GO:0016021|	GO:0005524|A	.
Manes.04G12670(	5.3805834	5.07E-13	up	yes	.	pfam06208	BDV_G	Borna disea	.	.	.	.
Manes.04G12680(	6.2946723	0.00076	up	yes	Proline-rich r	pfam00069	Pkinase	Protein kina	.	GO:0016021|	GO:0005524|A	.
Manes.04G12690(	23.056331	5.74E-36	up	yes	Proline-rich r	pfam00069	Pkinase	Protein kina	.	GO:0016021|	GO:0005524|A	.
Manes.04G12840(	2.3640262	3.42E-19	up	yes	Ras-related p	pfam00071	Ras	Ras family.	GO:0015031|ı	GO:0005622|	GO:0005525|G	.
Manes.04G12860(	2.3020071	3.45E-06	up	yes	.	pfam01925	TauE	Sulfite expo	.	.	.	.
Manes.04G12880(	1.2381923	0.21937	up	no	.	pfam01925	TauE	Sulfite expo	.	.	.	.
Manes.04G12900(	Inf	0.9009	up	no	SKP1-like pr	pfam03931	Skp1_POZ	Skp1 family	GO:0009734|a	GO:0005829|	.	K03094
Manes.04G12910(	0.4097914	5.58E-13	down	yes	.	pfam03168	LEA_2	Late embryc	.	.	.	.
Manes.04G12920(	0.9915708	0.94867	down	no	La protein 1	pfam08777	RRM_3	RNA bindin	GO:0034470|ı	GO:0005618|	GO:0000166|nu	K11090
Manes.04G12970(	11.910072	0.08851	up	no	.	pfam01733	Nucleoside_tran	Nucleoside	.	.	.	.
Manes.04G13020(	8.0716136	3.34E-17	up	yes	.	pfam14432	DYW_deaminase	DYW family	.	.	.	.
Manes.04G13060(	1.0467995	0.69584	up	no	.	pfam03061	4HBT	Thioesterase	.	.	.	.
Manes.04G13100(	1.867999	8.79E-05	up	no	Transcription	pfam00010	HLH	Helix-loop-l	GO:0006355|ı	GO:0005634|	GO:0003677|DI	.
Manes.04G13140(	0.426556	0.0003	down	yes	.	pfam03012	PP_M1	Phosphopro	.	.	.	.
Manes.04G13200(	2.441964	2.65E-08	up	yes	Ankyrin repe	pfam13962	PGG	Domain of u	.	GO:0005783|	.	.
Manes.04G13240(	0.3186274	0.00235	down	yes	CASP-like pr	pfam04535	DUF588	Domain of u	.	GO:0016021|	.	.
Manes.04G13280(	0.4408055	0.0004	down	yes	GATA transc	pfam00320	GATA	GATA zinc	GO:0007623|(	GO:0005634|	GO:0043565|se	.
Manes.04G13310(	0.7744202	0.01384	down	no	Homeobox-le	pfam08670	MEKHLA	MEKHLA c	GO:0030154|(	GO:0005634|	GO:0003677|DI	.
Manes.04G13370(	1.150326	0.68734	up	no	ABC transpo	pfam01061	ABC2_membrane	ABC-2 type	GO:0070574|(	GO:0009507|	GO:0005524|A	.
Manes.04G13390(	1.8865084	3.94E-12	up	no	NADP-deper	pfam03949	Malic_M	Malic enzyn	GO:0006108|ı	GO:0005737|	GO:0004471|ma	K00029
Manes.04G13400(	1.9686942	4.24E-14	up	no	Transcription	pfam00249	Myb_DNA-binding	Myb-like DI	GO:0048262|(	GO:0005634|	GO:0003677|DI	.
Manes.04G13420(	1.2528432	0.03282	up	no	RNA polyme	pfam03985	Paf1	Paf1. Memb	GO:0007507|l	GO:0016593|	GO:0000993|RI	K15174
Manes.04G13430(	1.5181756	1.84E-05	up	no	LysM domair	pfam07714	Pkinase_Tyr	Protein tyro	GO:0050832|(	GO:0016021|	GO:0005524|A	.
Manes.04G13470(	1.4244664	0.00374	up	no	Hexokinase-2	pfam03727	Hexokinase_2	Hexokinase.	GO:0001678|(	GO:0009707|	GO:0005524|A	K00844
Manes.04G13480(	1.5560654	1.11E-06	up	no	Nuclear poly(	pfam04928	PAP_central	Poly(A) pol	GO:0006397|ı	GO:0005634|	GO:0005524|A	K14376
Manes.04G13500(	18.591955	1.59E-56	up	yes	.	pfam15385	SARG	Specifically	.	.	.	.
Manes.04G13550(	Inf	0.9009	up	no	.	pfam00234	Tryp_alpha_amyl	Protease inh	.	.	.	.
Manes.04G13570(	0.4560228	1.69E-08	down	yes	Protein CHU	pfam05557	MAD	Mitotic chec	GO:0009902|(	GO:0009507|	.	.
Manes.04G13620(	19.703598	6.63E-88	up	yes	Probable 2-o:	pfam03171	2OG-FeII_Oxy	2OG-Fe(II)	.	.	GO:0046872|m	.
Manes.04G13630(	7.3626697	1.17E-23	up	yes	Inositol oxyg	pfam05153	DUF706	Family of ur	GO:0019310|i	GO:0005737|	GO:0050113|in	.
Manes.04G13660(	0.7278511	0.00113	down	no	Protein EARI	pfam03594	BenE	Benzoate m	GO:0007623|(	GO:0005634|	GO:0003700|tra	K12125
Manes.04G13690(	1.4664524	0.01204	up	no	Jacalin-relate	pfam01419	Jacalin	Jacalin-like	GO:0019762|ı	.	GO:0030246|ca	.
Manes.04G13700(	1.8847033	3.40E-11	up	no	Charged mult	pfam03357	Snf7	Snf7. This fa	GO:0045324|l	GO:0005737|	.	K12197
Manes.04G13740(	0.9709058	0.82287	down	no	.	pfam05773	RWD	RWD doma	.	.	.	.
Manes.04G13750(	0.5636633	2.09E-08	down	no	NADP-specif	pfam00208	ELFV_dehydrog	Glutamate/I	GO:0006520|(	.	GO:0004354|glı	.
Manes.04G13770(	1.3140137	0.00271	up	no	Ubiquitin car	pfam00443	UCH	Ubiquitin ca	GO:0006511|ı	.	GO:0008234|cy	K11843
Manes.04G13790(	0.3012083	9.47E-10	down	yes	Eukaryotic tr	pfam05091	eIF-3_zeta	Eukaryotic t	GO:0001731|(	GO:0016282|	GO:0003743|tra	K03251
Manes.04G13800(	0.8580867	0.11367	down	no	Serine/threon	pfam15563	Imm11	Immunity pr	GO:0006470|ı	GO:0005829|	GO:0008601|pr	K04354
Manes.04G13820(	1.4904226	0.02303	up	no	ABC transpo	pfam00005	ABC_tran	ABC transp	.	GO:0016021|	GO:0005524|A	.
Manes.04G13860(	0.0993177	0.04692	down	no	18.1 kDa clas	pfam00011	HSP20	Hsp20/alpha	GO:0010286|l	GO:0005737|	.	K13993
Manes.04G13880(	0.6233086	3.21E-05	down	no	RNA-binding	pfam00076	RRM_1	RNA recogı	GO:0010862|ı	GO:0005737|	GO:0000166|nu	.
Manes.04G13920(	3.3011132	1.94E-07	up	yes	Beta-galacto:	pfam01301	Glyco_hydro_35	Glycosyl hy	GO:0005975|(	GO:0048046|	GO:0004565|be	.
Manes.04G13930(	3.6500897	7.97E-42	up	yes	.	pfam04633	Herpes_BMRF2	Herpesvirus	.	.	.	.
Manes.04G13940(	0.7611803	0.00473	down	no	Transmembra	pfam02990	EMP70	Endomembr	GO:0006878|(	GO:0005737|	.	K17086
Manes.04G13980(	0.9967654	1	down	no	Receptor-like	pfam07714	Pkinase_Tyr	Protein tyro	GO:0030154|(	GO:0016021|	GO:0005524|A	.
Manes.04G13990(	2.0129719	2.71E-12	up	yes	.	pfam03168	LEA_2	Late embryc	.	.	.	.
Manes.04G14000(	0.578189	0.00075	down	no	.	pfam13041	PPR_2	PPR repeat	.	.	.	.
Manes.04G14040(	0.7640585	0.02188	down	no	Putative hydr	pfam12706	Lactamase_B_2	Beta-lactam	.	GO:0005829|	GO:0016787|hy	.
Manes.04G14060(	1.174059	0.12447	up	no	Nucleosome	pfam00956	NAP	Nucleosome	GO:0009294|l	GO:0005737|	GO:0042393|his	.
Manes.04G14090(	0.2983335	5.40E-10	down	yes	Protein IQ-D	pfam00513	Late_protein_L2	Late Protein	.	GO:0005634|	.	.
Manes.04G14100(	0.6430916	0.08172	down	no	EPIDERMAI	pfam13210	DUF4018	Domain of u	GO:0010052|ı	GO:0005576|	.	.
Manes.04G14130(	1.3576558	0.00068	up	no	Casein kinase	pfam00069	Pkinase	Protein kina	GO:0006897|(	GO:0005737|	GO:0005524|A	K02218
Manes.04G14150(	0.9233203	0.47063	down	no	Eukaryotic in	pfam00270	DEAD	DEAD/DEA	.	.	GO:0005524|A	.
Manes.04G14180(	1.4694641	2.11E-05	up	no	Dihydrolipoy	pfam00198	2-oxoacid_dh	2-oxoacid d	GO:0006096|ı	GO:0009507|	GO:0005507|co	K00627
Manes.04G14200(	0.2746606	2.90E-07	down	yes	U-box domai	pfam00069	Pkinase	Protein kina	.	.	GO:0005524|A	.
Manes.04G14210(	0.4675379	2.22E-13	down	yes	Protein phosı	pfam00481	PP2C	Protein phos	GO:0009738|a	GO:0005737|	GO:0046872|m	K14497
Manes.04G14230(	0.484258	2.13E-05	down	yes	.	pfam10319	7TM_GPCR_Srj	Serpentine t	.	.	.	.
Manes.04G14250(	1.3659489	0.00086	up	no	Midasin	pfam07728	AAA_5	AAA domai	GO:0022618|ı	GO:0005634|	GO:0005524|A	K14572
Manes.04G14260(	2.454102	4.56E-15	up	yes	Nuclear trans	pfam02045	CBFB_NFYA	CCAAT-bin	GO:0006351|ı	GO:0016602|	GO:0003677|DI	.
Manes.04G14270(	1.0735142	0.43743	up	no	Polyadenylat	pfam06741	LsmAD	LsmAD dor	GO:0010603|ı	GO:0010494|	GO:0044822|pc	.
Manes.04G14300(	0.3967321	1.20E-11	down	yes	Protein Brevi	pfam08381	BRX	Transcriptio	.	GO:0005634|	.	.
Manes.04G14320(	0.6398632	0.00035	down	no	.	pfam14897	EpsG	EpsG family	.	.	.	.
Manes.04G14350(	17.378286	0.04264	up	no	F-box proteir	pfam08268	FBA_3	F-box assoc	GO:0016567|ı	.	.	.
Manes.04G14370(	0.8832673	0.21417	down	no	Telomere rep	pfam00249	Myb_DNA-binding	Myb-like DI	GO:0006355|ı	GO:0005634|	GO:0042162|tel	.
Manes.04G14430(	0.5555236	0.00216	down	no	.	pfam04110	APG12	Ubiquitin-lil	.	.	.	.
Manes.04G14460(	2.088163	9.12E-08	up	yes	.	pfam02336	Denso_VP4	Capsid prot	.	.	.	.
Manes.04G14540(	0.7741715	0.0358	down	no	Transcription	pfam02291	TFIID-31kDa	Transcriptio	GO:0006352|l	GO:0005634|	.	K03133
Manes.04G14550(	0.6882753	0.0357	down	no	Probable GTI	pfam04607	RelA_SpoT	Region four	GO:0015969|ı	GO:0009507|	GO:0005524|A	.
Manes.04G14570(	Inf	1.50E-05	up	yes	Cytochrome	pfam00067	p450	Cytochrome	GO:0009835|l	GO:0005789|	GO:0020037|he	.
Manes.04G14590(	0.2873396	0.00029	down	yes	.	pfam11808	DUF3329	Domain of u	.	.	.	.
Manes.04G14600(	0.7987059	0.07138	down	no	RING-box pr	pfam12678	zf-rbx1	RING-H2 z	GO:0016567|ı	GO:0005737|	GO:0008270|zi	.
Manes.04G14610(	0.8432434	0.07696	down	no	Subtilisin-like	pfam00082	Peptidase_S8	Subtilase fa	GO:0080001|ı	GO:0048046|	GO:0004252|se	.
Manes.04G14630(	0.9893262	0.93309	down	no	Aldehyde del	pfam00171	Aldedh	Aldehyde de	GO:0006081|(	GO:0005829|	GO:0004029|alı	K14085
Manes.04G14660(	0.5229682	2.21E-05	down	no	Inter-alpha-tr	pfam00092	VWA	von Willebr	GO:0030212|l	GO:0070062|	GO:0004867|se	.
Manes.04G14710(	0.793358	0.26539	down	no	Probable nuc	pfam00334	NDK	Nucleoside	GO:0006241|(	.	GO:0005524|A	K00940
Manes.04G14750(	7.0469693	7.67E-07	up	yes	.	pfam02461	AMO	Ammonia m	.	.	.	.
Manes.04G14800(	0.9034383	0.32079	down	no	Oligouridylat	pfam00076	RRM_1	RNA recogı	GO:0006397|ı	GO:0005634|	GO:0000166|nu	.
Manes.04G14810(	0.7799407	0.01816	down	no	Pentatricope	pfam13041	PPR_2	PPR repeat	.	GO:0005739|	.	.
Manes.04G14840(	0.9597518	0.69041	down	no	25.3 kDa ves	pfam13774	Longin	Regulated-S	GO:0006887|(	GO:0005783|	GO:0005484|SI	K08517
Manes.04G14870(	0.2824032	2.31E-11	down	yes	Trihelix trans	pfam13837	Myb_DNA-bind_4	Myb/SANT-	GO:0009793|(	GO:0005634|	GO:0003677|DI	.

Manes.04G14890(	6.1930956	5.22E-87	up	yes	DnaJ homolo	pfam00226	DnaJ	DnaJ domai	GO:0061077|	GO:0005829|	GO:0051087|ch	K09514
Manes.04G14920(	3.3453833	3.91E-07	up	yes	.	pfam02519	Auxin_inducible	Auxin respo	.	.	.	.
Manes.04G14950(	0.7974469	0.12546	down	no	Phosphoinosi	pfam02383	Syja_N	SacI homolo	GO:0036092|	GO:0005774|	GO:0042578|ph	.
Manes.04G14960(	0.2222592	0.00052	down	yes	GDSL estera	pfam00657	Lipase_GDSL	GDSL-like l	GO:0016042|	GO:0005576|	GO:0016298|lip	.
Manes.04G14990(	13.03707	2.44E-11	up	yes	GDSL estera	pfam00657	Lipase_GDSL	GDSL-like l	GO:0042742|	GO:0005615|	GO:0016298|lip	.
Manes.04G15010(	3.5230251	3.16E-44	up	yes	Ethylene-resp	pfam00847	AP2	AP2 domair	GO:0070483|	GO:0005634|	GO:0003677|DI	K09286
Manes.04G15030(	5.6835205	1.07E-10	up	yes	.	pfam04570	DUF581	Protein of u	.	.	.	.
Manes.04G15050(	0.7160579	0.0007	down	no	1-phosphatid	pfam01504	PIP5K	Phosphatidy	GO:0010256|	GO:0005768|	GO:0000285|1-	K00921
Manes.04G15060(	1.306862	0.18473	up	no	.	pfam12044	Metallopep	Putative pep	.	.	.	.
Manes.04G15070(	0.4973394	0.01115	down	yes	Uncharacteriz	pfam03763	Remorin_C	Remorin, C-	.	GO:0005886|	.	.
Manes.04G15090(	1.0308056	0.80292	up	no	Alanine amin	pfam00155	Aminotran_1_2	Aminotransf	GO:0009058|	GO:0005739|	GO:0004021|L-	K00814
Manes.04G15100(	0.5665237	1.75E-07	down	no	Pectinesteras	pfam01095	Pectinesterase	Pectinestera	GO:0042545|	GO:0005618|	GO:0045330|as	.
Manes.04G15110(	1.7196933	1.13E-09	up	no	Pumilio homo	pfam14910	MMS22L_N	S-phase gen	GO:0010252|	GO:0005730|	GO:0003723|RI	K14790
Manes.04G15130(	1.0368793	0.70305	up	no	Ubiquitin-cor	pfam00179	UQ_con	Ubiquitin-co	GO:0030433|	GO:0005737|	GO:0005524|A'	K04554
Manes.04G15140(	2.3255294	1.92E-06	up	yes	.	pfam12726	SEN1_N	SEN1 N ter	.	.	.	.
Manes.04G15150(	1.5309386	0.00534	up	no	RING-H2 fin	pfam13639	zf-RING_2	Ring finger	GO:0016567|	GO:0016021|	GO:0008270|zi	.
Manes.04G15170(	1.2980023	0.07121	up	no	Bifunctional	pfam02882	THF_DHG_CYH_	Tetrahydrof	GO:0009396|	GO:0009507|	GO:0004477|m	.
Manes.04G15200(	0.9577333	0.67545	down	no	26S protease	pfam00004	AAA	ATPase fam	GO:0030163|	GO:0005737|	GO:0005524|A'	K03061
Manes.04G15270(	2.5603962	5.08E-14	up	yes	Tyrosine-sulf	pfam00069	Pkinase	Protein kina	GO:0009755|	GO:0016021|	GO:0005524|A'	.
Manes.04G15280(	0.5530632	0.00013	down	no	.	pfam09164	VitD-bind_III	Vitamin D b	.	.	.	.
Manes.04G15310(	8.0380078	0.08571	up	no	CO(2)-respor	pfam00082	Peptidase_S8	Subtilase fa	GO:2000122|	GO:0048046|	GO:0004252|se	.
Manes.04G15340(	Inf	0.01269	up	yes	.	pfam04079	DUF387	Putative tra	.	.	.	.
Manes.04G15370(	1.5080598	1.85E-05	up	no	Transcription	pfam00249	Myb_DNA-binding	Myb-like DI	GO:0030154|	GO:0005634|	GO:0003677|DI	K09422
Manes.04G15450(	0.4774392	1.03E-05	down	yes	.	pfam12776	Myb_DNA-bind_3	Myb/SANT-	.	.	.	.
Manes.04G15460(	3.3484905	1.81E-21	up	yes	Protein ELC	pfam05743	UEV	UEV domai	GO:0051301|	GO:0005769|	GO:0043130|ub	K12183
Manes.04G15470(	0.4184809	8.45E-11	down	yes	Galactoside 2	pfam03254	XG_FTase	Xyloglucan	GO:0042546|	GO:0032580|	GO:0008107|ga	.
Manes.04G15530(	0.7856187	0.01776	down	no	Dr1-associate	pfam00808	CBFD_NFYB_HN	Histone-like	GO:0000122|	GO:0005634|	GO:0043565|se	.
Manes.04G15540(	2.6997049	1.29E-29	up	yes	Cysteine prot	pfam00031	Cystatin	Cystatin dor	GO:0006952|	GO:0005576|	GO:0004869|cy	.
Manes.04G15580(	0.558344	0.00112	down	no	Putative RIN	pfam13639	zf-RING_2	Ring finger	GO:0016567|	GO:0016021|	GO:0008270|zi	.
Manes.04G15590(	0.8091031	0.02754	down	no	DNA-directe	pfam00562	RNA_pol_Rpb2_6	RNA polym	GO:0051607|	GO:0005666|	GO:0003677|DI	K03021
Manes.04G15600(	0.51828	6.64E-09	down	no	Cytosolic sul	pfam00685	Sulfotransfer_1	Sulfotransfe	.	GO:0005737|	GO:0008146|su	.
Manes.04G15610(	1.2750038	0.04245	up	no	Pentatricope	pfam13812	PPR_3	Pentatricope	.	GO:0009941|	.	.
Manes.04G15620(	Inf	8.80E-11	up	yes	Protein MIZU	pfam04759	DUF617	Protein of u	GO:0009658|	GO:0032541|	.	.
Manes.04G15630(	1.6564389	1.54E-05	up	no	.	pfam01625	PMSR	Peptide met	.	.	.	.
Manes.04G15640(	1.0847639	0.36576	up	no	THO comple	pfam11957	efThoc1	THO compl	GO:0050832|	GO:0005634|	GO:0003723|RI	.
Manes.04G15650(	0.8184947	0.04213	down	no	ERI1 exoribo	pfam00929	RNase_T	Exonuclease	GO:0000738|	GO:0005737|	GO:0000175|3'-	K18417
Manes.04G15660(	1.7553812	9.90E-10	up	no	DNA-directe	pfam00562	RNA_pol_Rpb2_6	RNA polym	GO:0006360|	GO:0005736|	GO:0003677|DI	K03002
Manes.04G15670(	Inf	0.9009	up	no	.	pfam03134	TB2_DP1_HVA22	TB2/DP1, F	.	.	.	.
Manes.04G15710(	1.8121984	4.57E-09	up	no	NADH dehyc	pfam13233	Complex1_LYR_2	Complex1_I	GO:0055114|	GO:0016020|	.	K03950
Manes.04G15720(	0.6668479	6.77E-05	down	no	TGACG-sequ	pfam14144	DOG1	Seed dorma	GO:0006351|	GO:0005634|	GO:0043565|se	.
Manes.04G15740(	3.3178318	7.53E-06	up	yes	Sigma factor	pfam05678	VQ	VQ motif. T	GO:0071482|	GO:0009507|	.	.
Manes.04G15750(	0.3436606	0.00114	down	yes	.	pfam05539	Pneumo_att_G	Pneumovirir	.	.	.	.
Manes.04G15790(	17.660929	1.72E-29	up	yes	.	pfam14380	WAK_assoc	Wall-associ	.	.	.	.
Manes.04G15800(	3.8405701	7.60E-06	up	yes	Glutamate re	pfam00060	Lig_chan	Ligand-gate	GO:0070588|	GO:0016021|	GO:0005262|ca	K05387
Manes.04G15810(	1.5662406	0.00012	up	no	Thioredoxin-	pfam00085	Thioredoxin	Thioredoxin	GO:0008219|	GO:0009507|	GO:0015035|pr	.
Manes.04G15860(	0.6121272	0.00046	down	no	.	pfam03419	Peptidase_U4	Sporulation	.	.	.	.
Manes.04G15900(	NA	NA	--	no	NADPH:quir	pfam03358	FMN_red	NADPH-dep	GO:0042742|	GO:0009507|	GO:0003955|N.	.
Manes.04G15930(	0.4535374	4.38E-08	down	yes	Putative amic	pfam07969	Amidohydro_3	Amidohydrc	.	.	GO:0016810|hy	K07047
Manes.04G15950(	0.3689054	5.61E-07	down	yes	Protein NRT	pfam00854	PTR2	POT family.	GO:0006857|	GO:0016021|	GO:0005215|tra	.
Manes.04G15980(	5.2516779	0.00237	up	yes	Two-compor	pfam00072	Response_reg	Response re	GO:0007623|	GO:0005634|	GO:0000156|ph	K14492
Manes.04G16000(	1.0661625	0.46979	up	no	F-box protei	pfam00176	SNF2_N	SNF2 family	.	GO:0009506|	GO:0005524|A'	.
Manes.04G16030(	1.7766189	5.79E-10	up	no	Alpha-galact	pfam02065	Melibiase	Melibiase. C	GO:0005975|	.	GO:0052692|ra	.
Manes.04G16040(	1.5855244	2.87E-07	up	no	Pyruvate kin	pfam00224	PK	Pyruvate kir	GO:0006096|	GO:0005737|	GO:0005524|A'	K00873
Manes.04G16050(	2.025466	8.90E-15	up	yes	PAX3- and P	pfam07842	GCFC	GC-rich seq	GO:0007517|	GO:0005829|	GO:0000978|RI	K13211
Manes.04G16060(	1.3414183	0.0027	up	no	WD repeat-c	pfam00400	WD40	WD domain	.	GO:0005737|	.	.
Manes.04G16090(	1.0951092	0.65964	up	no	Mitochondria	pfam06747	CHCH	CHCH dom	GO:0006457|	GO:0031305|	GO:0051537|2	.
Manes.04G16110(	1.0196656	0.84638	up	no	G patch dom	pfam01585	G-patch	G-patch dor	.	.	GO:0003676|nu	.
Manes.04G16120(	0.9784308	0.83724	down	no	Phytochrome	pfam00149	Metallophos	Calcineurin-	.	GO:0005737|	GO:0046872|m	.
Manes.04G16130(	0.8578851	0.11644	down	no	Protease 2	pfam02897	Peptidase_S9_N	Prolyl oligo	.	.	GO:0004252|se	.
Manes.04G16140(	17.633925	0.00145	up	yes	DNA-directe	pfam14763	HPS3_C	Hermansky-	GO:0006351|	GO:0009507|	GO:0003677|DI	.
Manes.04G16150(	0.7294771	0.00314	down	no	.	pfam03097	BRO1	BRO1-like	.	.	.	.
Manes.04G16160(	2.9201402	1.02E-07	up	yes	.	pfam14559	TPR_19	Tetratricope	.	.	.	.
Manes.04G16210(	0.9539676	0.76983	down	no	Protein ASP	pfam14543	TAXi_N	Xylanase inl	.	.	GO:0004190|as	.
Manes.04G16230(	1.6211071	1.66E-07	up	no	Mitochondria	pfam01459	Porin_3	Eukaryotic	GO:0006811|	GO:0005743|	GO:0015450|P-	K11518
Manes.04G16250(	4.5912258	3.79E-26	up	yes	.	pfam02893	GRAM	GRAM dom	.	.	.	.
Manes.04G16270(	1.7880031	1.08E-09	up	no	F-box/LRR-r	pfam13855	LRR_8	Leucine rich	.	.	.	K10268
Manes.04G16290(	2.7628519	9.24E-14	up	yes	Transmembra	pfam05705	DUF829	Eukaryotic	.	GO:0016021|	.	.
Manes.04G16330(	1.7572206	0.00277	up	no	Cytokinin del	pfam09265	Cytokin-bind	Cytokinin d	GO:0009690|	GO:0005615|	GO:0019139|cy	K00279
Manes.04G16340(	1.0360226	0.71448	up	no	.	pfam13178	DUF4005	Protein of u	.	.	.	.
Manes.04G16370(	0.5214452	1.31E-09	down	no	Ubiquitin car	pfam00443	UCH	Ubiquitin ca	GO:0043161|	GO:0005634|	GO:0004197|cy	K11841
Manes.04G16420(	2.40275	4.97E-12	up	yes	Scarecrow-lil	pfam03514	GRAS	GRAS doma	GO:0006355|	GO:0005634|	GO:0003700|tra	.
Manes.04G16500(	0.9525915	0.65989	down	yes	CSC1-like pr	pfam02714	DUF221	Domain of u	GO:0006811|	GO:0016021|	.	.
Manes.04G16510(	0.7222484	0.00224	down	no	Annexin D3	pfam00191	Annexin	Annexin. Th	GO:0009409|	GO:0005773|	GO:0005509|ca	.
Manes.04G16520(	0.8331079	0.05557	down	no	Annexin D4	pfam00191	Annexin	Annexin. Th	GO:0009737|	GO:0009506|	GO:0005509|ca	.
Manes.04G16540(	0.527146	1.72E-09	down	no	.	pfam00003	7tm_3	7 transmeml	.	.	.	.
Manes.04G16560(	1.9439536	1.15E-12	up	no	Putative dise	pfam00931	NB-ARC	NB-ARC do	GO:0006952|	.	GO:0043531|Al	.
Manes.04G16570(	9.7817594	0.00187	up	yes	DNA-directe	pfam01191	RNA_pol_Rpb5_C	RNA polym	GO:0006351|	GO:0000419|	GO:0003677|DI	K03013
Manes.04G16590(	1.1395157	0.6008	up	no	Probable AD	pfam01412	ArfGap	Putative GT	.	.	GO:0005096|G'	K12486
Manes.04G16600(	1.915011	9.25E-06	up	no	Uncharacteriz	pfam00462	Glutaredoxin	Glutaredoxi	GO:0045454|	GO:0005886|	GO:0009055|el	.
Manes.05G00020(	40.114592	2.47E-05	up	yes	Protein EXO	pfam04674	Phi_1	Phosphate-i	.	GO:0048046|	.	.
Manes.05G00040(	0.7186699	0.00836	down	no	Programmed	pfam04194	PDCD2_C	Programme	GO:0006919|	GO:0005737|	GO:0003677|DI	K14801
Manes.05G00050(	0.5165196	4.02E-06	down	no	Nicotinamide	pfam00153	Mito_carr	Mitochondr	GO:0043132|	GO:0031969|	GO:0015297|an	K15115
Manes.05G00070(	0.4634228	2.42E-09	down	yes	.	pfam12776	Myb_DNA-bind_3	Myb/SANT-	.	.	.	.
Manes.05G00080(	0.5042178	1.14E-06	down	no	Cytidine dear	pfam08211	dCMP_cyt_deam_	Cytidine and	GO:0009972|	GO:0005829|	GO:0004126|cy	K01489
Manes.05G00100(	1.0259802	0.79346	up	no	Probable gala	pfam01501	Glyco_transf_8	Glycosyl tra	GO:0071555|	GO:0005794|	GO:0047262|pc	.
Manes.05G00140(	0.6848223	0.03777	down	no	Heparan-alph	pfam07786	DUF1624	Protein of u	GO:0007041|	GO:0016021|	GO:0015019|he	K10532
Manes.05G00180(	0.7477395	0.00969	down	no	Phosphoribos	pfam02769	AIRS_C	AIR synthas	GO:0006189|	GO:0009507|	GO:0005524|A'	.
Manes.05G00210(	1.0178093	0.90913	up	no	DnaJ protein	pfam00226	DnaJ	DnaJ domai	GO:0052033|	GO:0005783|	.	K09517

Manes.05G00220(	23.051827	#######	up	yes	.	pfam01764	Lipase_3	Lipase (clas:	.	.	.	.
Manes.05G00240(	2.3907475	7.24E-22	up	yes	.	pfam00226	DnaJ	DnaJ domai	.	.	.	.
Manes.05G00290(	9.2698343	2.81E-46	up	yes	Probable E3 ı	pfam12428	DUF3675	Protein of u	GO:0042335|ı	GO:0016021|	GO:0016874|lig	K10661
Manes.05G00320(	0.9061702	0.35242	down	no	Vacuolar pro	pfam15469	Sec5	Exocyst con	GO:0006914|a	GO:1990745|	.	.
Manes.05G00330(	3.0265195	1.79E-33	up	yes	Ocs element-	pfam00170	bZIP_1	bZIP transcı	GO:0006351|ı	GO:0005634|	GO:0043565|se	.
Manes.05G00380(	0.7262023	0.04491	down	no	.	pfam08450	SGL	SMP-30/Glı	.	.	.	.
Manes.05G00390(	0.5227269	2.19E-07	down	no	Protein trichc	pfam13839	PC-Esterase	GDSL/SGN	.	GO:0016021|	.	.
Manes.05G00400(	0.2096642	1.28E-05	down	yes	Histone-lysin	pfam00856	SET	SET domair	GO:0006355|ı	GO:0031519|	GO:0018024|his	K11430
Manes.05G00410(	20.873833	0.00058	up	yes	DELLA prot	pfam03514	GRAS	GRAS doma	GO:0030154|ı	GO:0005634|	GO:0003700|tra	K14494
Manes.05G00430(	2.2672686	1.03E-18	up	yes	Probable WR	pfam03106	WRKY	WRKY DN.	GO:0009793|ı	GO:0005634|	GO:0043565|se	.
Manes.05G00450(	0.8829603	0.33286	down	no	.	pfam04795	PAPA-1	PAPA-1-like	.	.	.	.
Manes.05G00460(	Inf	0.08088	up	no	.	pfam14244	UBN2_3	gag-polyper	.	.	.	.
Manes.05G00490(	1.3381859	0.00138	up	no	DEAD-box A	pfam00270	DEAD	DEAD/DEA	.	.	GO:0005524|A'	K12835
Manes.05G00520(	28.697062	1.87E-96	up	yes	.	pfam01104	Bunya_NS-S	Bunyavirus	.	.	.	.
Manes.05G00530(	3.2790302	1.31E-10	up	yes	NAC domain	pfam02365	NAM	No apical m	GO:0006351|ı	GO:0005634|	GO:0003677|DI	.
Manes.05G00560(	198.45925	#######	up	yes	.	pfam00050	Kazal_1	Kazal-type s	.	.	.	.
Manes.05G00570(	2.1762257	0.12947	up	no	.	pfam00050	Kazal_1	Kazal-type s	.	.	.	.
Manes.05G00610(	0.3921467	2.36E-11	down	yes	.	pfam13919	ASXH	Asx homolo	.	.	.	.
Manes.05G00620(	0.7374934	0.00312	down	no	.	pfam02893	GRAM	GRAM dom	.	.	.	.
Manes.05G00680(	0.6074714	9.86E-06	down	no	Protein phos	pfam00481	PP2C	Protein phos	GO:0010074|ı	GO:0005634|	GO:0046872|me	.
Manes.05G00710(	0.5233957	4.63E-07	down	no	AarF domain	pfam03109	ABC1	ABC1 famil	GO:0032836|ı	GO:0005829|	GO:0004674|pr	K08869
Manes.05G00720(	0.3372561	1.55E-05	down	yes	Palmitoleoyl-	pfam03283	PAE	Pectinacetyl	GO:0030178|ı	GO:0005576|	GO:1990699|pa	.
Manes.05G00730(	1.1382151	0.16326	up	no	.	pfam03222	Trp_Tyr_perm	Tryptophan/	.	.	.	.
Manes.05G00740(	4.5032126	2.04E-35	up	yes	Transcription	pfam00249	Myb_DNA-binding	Myb-like DI	GO:0006355|ı	GO:0005634|	GO:0003677|DI	K09422
Manes.05G00750(	0.2805831	9.18E-05	down	yes	.	.	.	.	.	.	.	.
Manes.05G00770(	0.6846053	0.00078	down	no	Cytochrome	pfam00067	p450	Cytochrome	GO:0010268|ı	GO:0016021|	GO:0020037|he	.
Manes.05G00820(	0.8006398	0.01992	down	no	Protoporphyı	pfam01593	Amino_oxidase	Flavin conta	GO:0015995|ı	GO:0009507|	GO:0004729|ox	K00231
Manes.05G00880(	0.5698005	4.63E-06	down	no	Probable poly	pfam00295	Glyco_hydro_28	Glycosyl hy	GO:0005975|ı	GO:0016021|	GO:0004650|pc	.
Manes.05G00890(	0.5784661	0.00067	down	no	.	pfam01248	Ribosomal_L7Ae	Ribosomal p	.	.	.	.
Manes.05G00930(	53.045944	1.24E-13	up	yes	ABC transpo	pfam00664	ABC_membrane	ABC transp	GO:0010541|ı	GO:0016021|	GO:0005524|A'	K05658
Manes.05G00940(	63.357766	#######	up	yes	ABC transpo	pfam00664	ABC_membrane	ABC transp	GO:0010541|ı	GO:0016021|	GO:0005524|A'	K05658
Manes.05G00950(	0.8569689	0.46748	down	no	Two-pore po	pfam07885	Ion_trans_2	Ion channel.	GO:0071805|ı	GO:0005887|	GO:0046872|me	K05389
Manes.05G00960(	1.448164	0.00076	up	no	.	pfam02028	BCCT	BCCT famil	.	.	.	.
Manes.05G00980(	2.1568087	0.00504	up	yes	.	pfam06501	Herpes_U55	Human herp	.	.	.	.
Manes.05G00990(	1.9517643	1.65E-13	up	no	.	pfam05678	VQ	VQ motif. T	.	.	.	.
Manes.05G01010(	0.7346775	0.05229	down	no	.	pfam00226	DnaJ	DnaJ domai	.	.	.	.
Manes.05G01040(	0.764611	0.00713	down	no	Squamosa pr	pfam03110	SBP	SBP domair	GO:0006355|ı	GO:0005634|	GO:0003677|DI	.
Manes.05G01070(	1.2399519	0.15995	up	no	Autophagy-r	pfam02991	Atg8	Autophagy p	GO:0000045|a	GO:0000421|	GO:0019779|At	K08341
Manes.05G01080(	0.9577425	0.66197	down	no	E3 ubiquitin-	pfam13920	zf-C3HC4_3	Zinc finger,	.	GO:0005737|	GO:0016874|lig	.
Manes.05G01130(	8.3207501	2.26E-99	up	yes	Probable prot	pfam00481	PP2C	Protein phos	GO:0006470|ı	.	GO:0046872|me	K14803
Manes.05G01140(	1.4105661	0.00023	up	no	Glycogen syı	pfam00069	Pkinase	Protein kina	.	.	GO:0005524|A'	.
Manes.05G01170(	0.2837545	0.00023	down	yes	GDSL estera	pfam00657	Lipase_GDSL	GDSL-like l	GO:0016042|l	GO:0009570|	GO:0016788|hy	.
Manes.05G01190(	0.2615753	7.48E-10	down	yes	.	pfam13041	PPR_2	PPR repeat	.	.	.	.
Manes.05G01210(	5.0684394	0.01624	up	yes	Transcription	pfam00249	Myb_DNA-binding	Myb-like DI	GO:0006952|ı	GO:0005634|	GO:0003677|DI	K09422
Manes.05G01230(	0.7535682	0.00376	down	no	SWR1-comp	pfam05499	DMAP1	DNA methy	GO:0006338|ı	GO:0035267|	GO:0003677|DI	K11324
Manes.05G01290(	17.805906	1.04E-07	up	yes	Probable glyc	pfam01553	Acyltransferase	Acyltransfer	GO:0016024|ı	GO:0016021|	GO:0004366|gl	K13508
Manes.05G01300(	0.4829936	4.21E-08	down	yes	.	pfam05602	CLPTM1	Cleft lip and	.	.	.	.
Manes.05G01320(	Inf	0.35135	up	no	.	pfam14291	DUF4371	Domain of u	.	.	.	.
Manes.05G01340(	0.2737629	1.06E-15	down	yes	.	pfam10327	7TM_GPCR_Sri	Serpentine t	.	.	.	.
Manes.05G01350(	3.8353048	0.00039	up	yes	Probable WR	pfam03106	WRKY	WRKY DN.	GO:0009733|ı	GO:0005634|	GO:0043565|se	.
Manes.05G01410(	0.7206203	0.00123	down	no	Pentatricopeı	pfam13041	PPR_2	PPR repeat	GO:0010019|ı	GO:0009507|	GO:0003677|DI	.
Manes.05G01420(	1.3733718	0.00044	up	no	Nodal modul	pfam13620	CarboxypepD_reg	Carboxypep	.	GO:0005789|	GO:0030246|ca	.
Manes.05G01430(	1.0050976	1	up	no	.	pfam09459	EB_dh	Ethylbenzen	.	.	.	.
Manes.05G01480(	1.0134829	0.91772	up	no	NAC domain	pfam02365	NAM	No apical m	GO:0090603|s	GO:0005634|	GO:0003700|tra	.
Manes.05G01490(	0.4133869	5.28E-09	down	yes	Protein arv1	pfam04161	Arv1	Arv1-like fa	GO:0006696|ı	GO:0032541|	.	.
Manes.05G01500(	3.9633852	1.05E-13	up	yes	.	pfam01529	zf-DHHC	DHHC palm	.	.	.	.
Manes.05G01510(	3.0316041	1.87E-34	up	yes	B3 domain-c	pfam02362	B3	B3 DNA bir	GO:0009908|ı	GO:0005634|	GO:0003677|DI	.
Manes.05G01550(	0.5183609	1.19E-07	down	no	Serine/threon	pfam00069	Pkinase	Protein kina	GO:0006914|a	GO:0034045|	GO:0005524|A'	.
Manes.05G01580(	0.6355454	4.85E-06	down	no	Transcription	pfam00010	HLH	Helix-loop-l	GO:0006355|ı	GO:0005634|	GO:0003677|DI	.
Manes.05G01600(	0.9504843	0.61345	down	no	Metal toleran	pfam01545	Cation_efflux	Cation efflu:	GO:0006882|ı	GO:0016021|	GO:0046872|me	K14689
Manes.05G01610(	42.893799	1.99E-07	up	yes	.	pfam03168	LEA_2	Late embryc	.	.	.	.
Manes.05G01620(	6.1206117	5.51E-93	up	yes	Two-compor	pfam00072	Response_reg	Response re	GO:0000160|ı	GO:0005634|	.	K12130
Manes.05G01650(	0.9574229	0.7118	down	no	Pyruvate deh	pfam00676	E1_dh	Dehydrogen	GO:0006096|ı	GO:0009507|	GO:0004739|py	K00161
Manes.05G01660(	1.1751887	0.14274	up	no	60S acidic rit	pfam00428	Ribosomal_60s	60s Acidic r	GO:0006414|ı	GO:0005840|	GO:0003735|stı	K02942
Manes.05G01690(	2.3991895	2.43E-11	up	yes	RNA-binding	pfam00076	RRM_1	RNA recogı	GO:0070935|3	GO:0005829|	GO:0003730|ml	.
Manes.05G01740(	0.981313	0.86468	down	no	Heterogeneoı	pfam00076	RRM_1	RNA recogı	GO:0007623|ı	GO:0070937|	GO:0000166|nu	K13160
Manes.05G01750(	0.9813849	0.87033	down	no	.	pfam06160	EzrA	Septation rii	.	.	.	.
Manes.05G01760(	9.2052381	1.41E-69	up	yes	3-ketoacyl-C	pfam08392	FAE1_CUT1_Rpp	FAE1/Type	GO:0006633|ı	GO:0022626|	GO:0009922|fa	K15397
Manes.05G01810(	0.5146289	1.09E-09	down	no	GRF1-interac	pfam05030	SSXT	SSXT prote	GO:0008283|ı	GO:0005634|	GO:0003713|tra	.
Manes.05G01820(	6.9921867	0.01993	up	yes	Transcription	pfam14215	bHLH-MYC_N	bHLH-MYC	GO:0006355|ı	GO:0005634|	GO:0003677|DI	.
Manes.05G01840(	0.6865342	0.00487	down	no	CDPK-relate	pfam00069	Pkinase	Protein kina	GO:0009738|a	GO:0005737|	GO:0005524|A'	.
Manes.05G01850(	0.3206522	1.46E-12	down	yes	Indole-3-acet	pfam02519	Auxin_inducible	Auxin respo	GO:0009733|ı	.	.	.
Manes.05G01870(	0.818289	0.73079	down	no	Homeobox-le	pfam00046	Homeobox	Homeobox (	GO:0045893|ı	GO:0005634|	GO:0043565|se	.
Manes.05G01880(	0.6290253	0.01062	down	no	Uncharacteriz	pfam04220	YihI	Der GTPase	.	GO:0016021|	.	.
Manes.05G01890(	0.4052349	6.64E-12	down	yes	Cytochrome	pfam00067	p450	Cytochrome	GO:0040008|ı	GO:0016021|	GO:0020037|he	.
Manes.05G01900(	1.0877412	0.39417	up	no	.	pfam03641	Lysine_decarbox	Possible lysi	.	.	.	.
Manes.05G01990(	Inf	0.08088	up	no	.	pfam04547	Anoctamin	Calcium-act	.	.	.	.
Manes.05G02000(	2.2954644	0.01118	up	yes	Calcium-bind	pfam13833	EF-hand_8	EF-hand doı	GO:0010091|t	.	GO:0005509|ca	.
Manes.05G02020(	0.6302372	3.73E-05	down	no	Probable seri	pfam00069	Pkinase	Protein kina	.	.	GO:0005524|A'	K08867
Manes.05G02050(	1.4070202	0.00089	up	no	ORM1-like p	pfam04061	ORMDL	ORMDL fai	GO:0006672|ı	GO:0005789|	.	.
Manes.05G02060(	3.662441	2.75E-17	up	yes	Dof zinc fing	pfam02701	zf-Dof	Dof domain	GO:0006355|ı	GO:0005634|	GO:0003677|DI	.
Manes.05G02140(	0.3911687	5.76E-14	down	yes	.	pfam11800	RP-C_C	Replication	.	.	.	.
Manes.05G02190(	NA	NA	--	no	Ethylene-resp	pfam00847	AP2	AP2 domair	GO:0009873|ı	GO:0005634|	GO:0003677|DI	K09286
Manes.05G02200(	1.6497636	1.70E-07	up	no	Transcription	pfam14215	bHLH-MYC_N	bHLH-MYC	GO:0006355|ı	GO:0005634|	GO:0003700|tra	.
Manes.05G02210(	0.6089997	6.10E-05	down	no	RNA polyme	pfam14227	UBN2_2	gag-polyper	GO:0006355|ı	GO:0005634|	GO:0003677|DI	.
Manes.05G02230(	0.5933723	0.00126	down	no	Cyclic pyranc	pfam01967	MoaC	MoaC famil	GO:0006777|ı	GO:0009507|	.	K03639
Manes.05G02250(	0.5954234	8.27E-05	down	no	Protein ASP/	pfam14541	TAXi_C	Xylanase inl	GO:0030163|ı	GO:0005783|	GO:0004190|as	.
Manes.05G02260(	0.8217565	0.08502	down	no	E3 ubiquitin-	pfam03145	Sina	Seven in abs	GO:0007275|ı	GO:0005634|	GO:0016874|lig	K04506

Manes.05G02270(	3.7329763	9.74E-50	up	yes	Phosphatidyli	pfam00454	PI3_PI4_kinase	Phosphatidy	GO:0046854|	GO:0005829|	GO:0004430|1-	.
Manes.05G02290(	1.2392132	0.69837	up	no	O-acyltransfe	pfam06974	DUF1298	Protein of u	GO:0019432|	GO:0005783|	GO:0004144|di	K15406
Manes.05G02320(	1.7219585	1.77E-09	up	no	Histone-lysin	pfam13832	zf-HC5HC2H_2	PHD-zinc-fi	.	GO:0005634|	GO:0018024|his	.
Manes.05G02330(	2.2270938	7.05E-08	up	yes	Histone-lysin	pfam00856	SET	SET domain	.	GO:0005634|	GO:0018024|his	.
Manes.05G02340(	9.9755951	0.02851	up	yes	Putative RIN	pfam13639	zf-RING_2	Ring finger	GO:0016567|	GO:0016021|	GO:0030247|pc	.
Manes.05G02360(	0.0991193	0.04704	down	no	.	pfam04197	Birna_RdRp	Birnavirus R	.	.	.	.
Manes.05G02380(	Inf	0.35135	up	no	.	pfam11155	DUF2935	Domain of u	.	.	.	.
Manes.05G02390(	0.5232532	0.00015	down	no	.	pfam09713	A_thal_3526	Plant protein	.	.	.	.
Manes.05G02400(	1.4907379	4.42E-05	up	no	DEAD-box A	pfam00270	DEAD	DEAD/DEA	.	.	GO:0005524|A	.
Manes.05G02450(	0.1187302	6.78E-08	down	yes	.	pfam06432	GPI2	Phosphatidy	.	.	.	.
Manes.05G02460(	0.4893191	2.55E-08	down	yes	Chitinase dor	pfam00704	Glyco_hydro_18	Glycosyl hy	GO:0005975|	GO:0005576|	GO:0004568|ch	K17525
Manes.05G02510(	0.8312542	0.23275	down	no	Protein tricho	pfam13839	PC-Esterase	GDSL/SGN	.	GO:0016021|	.	.
Manes.05G02520(	0.519963	4.81E-06	down	no	Protein tricho	pfam13839	PC-Esterase	GDSL/SGN	.	GO:0016021|	.	.
Manes.05G02530(	0.7082619	0.00347	down	no	.	pfam11331	DUF3133	Protein of u	.	.	.	.
Manes.05G02590(	4.5290184	8.13E-48	up	yes	Tubulin gamr	pfam00091	Tubulin	Tubulin/Fts	GO:0051641|	GO:0005938|	GO:0005525|G	K10389
Manes.05G02620(	10.867769	9.89E-17	up	yes	Ethylene-resp	pfam00847	AP2	AP2 domain	GO:0009736|	GO:0005737|	GO:0003677|D	.
Manes.05G02640(	0.2596825	6.23E-05	down	yes	.	pfam15279	SOBP	Sine oculis-l	.	.	.	.
Manes.05G02650(	0.5126779	5.45E-06	down	no	Putative gluc	pfam01263	Aldose_epim	Aldose 1-ep	GO:0005975|	.	GO:0030246|ca	.
Manes.05G02670(	0.6243042	0.0301	down	no	Common plan	pfam07777	MFMR	G-box bindi	GO:0006351|	GO:0005634|	GO:0043565|se	.
Manes.05G02790(	0.9352256	0.6521	down	no	UDP-D-xylos	pfam03407	Nucleotid_trans	Nucleotide-	GO:0042546|	GO:0005794|	GO:0035252|U	K11714
Manes.05G02810(	3.1233231	1.35E-28	up	yes	Delta(8)-fatty	pfam00487	FA_desaturase	Fatty acid d	GO:0070417|	GO:0005783|	GO:0046872|m	K13076
Manes.05G02820(	0.3327741	0.00092	down	yes	.	pfam05615	THOC7	Tho comple	.	.	.	.
Manes.05G02830(	0.4246223	1.62E-15	down	yes	Reticulon-lik	pfam02453	Reticulon	Reticulon. R	GO:0080167|	GO:0005783|	.	.
Manes.05G02850(	0.5665456	0.00283	down	no	RING-H2 fin	pfam13639	zf-RING_2	Ring finger	GO:0016567|	GO:0016021|	GO:0008270|zi	.
Manes.05G02860(	0.6212547	0.39918	down	no	.	pfam03168	LEA_2	Late embryo	.	.	.	.
Manes.05G02930(	0.692363	0.00906	down	no	3-methyl-2-o	pfam02548	Pantoate_transf	Ketopantoat	GO:0015940|	GO:0005739|	GO:0003864|3-	K00606
Manes.05G02940(	0.5648809	0.01198	down	no	Pentatricope	pfam13041	PPR_2	PPR repeat	.	GO:0005739|	.	.
Manes.05G02950(	1.5890222	0.0025	up	no	WRKY trans	pfam03106	WRKY	WRKY DN	GO:0006952|	GO:0005634|	GO:0043565|se	K13425
Manes.05G02990(	10.295685	0.03033	up	no	1-aminocyclo	pfam00155	Aminotran_1_2	Aminotrans	GO:0009693|	.	GO:0016847|1-	.
Manes.05G03000(	0.8656134	0.40022	down	no	.	pfam00403	HMA	Heavy-meta	.	.	.	.
Manes.05G03010(	1.0731098	0.46281	up	no	C-terminal bi	pfam02826	2-Hacid_dh_C	D-isomer sp	GO:0048444|	GO:0005829|	GO:0042802|id	.
Manes.05G03020(	1.5995422	1.30E-07	up	no	Protein REV	pfam00249	Myb_DNA-binding	Myb-like D	GO:0009737|	GO:0005634|	GO:0003677|D	.
Manes.05G03030(	0.7848085	0.02093	down	no	.	pfam03154	Atrophin-1	Atrophin-1	.	.	.	.
Manes.05G03050(	NA	NA	--	no	Dihydroflavo	pfam01370	Epimerase	NAD depen	GO:0009718|	.	GO:0050662|co	.
Manes.05G03060(	3.2990946	0.06944	up	no	UPF0481 pro	pfam03140	DUF247	Plant protein	.	GO:0016021|	.	.
Manes.05G03090(	16.996837	1.26E-49	up	yes	Probable WR	pfam03106	WRKY	WRKY DN	GO:0006351|	GO:0005634|	GO:0043565|se	.
Manes.05G03110(	2.3571313	1.57E-20	up	yes	Protein BPS1	pfam05633	DUF793	Protein of u	GO:0009793|	GO:0009507|	.	.
Manes.05G03120(	1.9446996	1.41E-12	up	no	Mitogen-acti	pfam00069	Pkinase	Protein kina	.	GO:0005622|	GO:0005524|A	.
Manes.05G03130(	0.3947339	1.91E-06	down	yes	Probable poly	pfam00295	Glyco_hydro_28	Glycosyl hy	GO:0005975|	GO:0016021|	GO:0004650|pc	.
Manes.05G03140(	24.247015	1.58E-66	up	yes	Putative UDI	pfam00201	UDPGT	UDP-glucor	.	GO:0016021|	GO:0016758|tra	.
Manes.05G03170(	0.2712146	1.88E-19	down	yes	E3 ubiquitin-	pfam13639	zf-RING_2	Ring finger	GO:0016567|	.	GO:0016874|lig	K16281
Manes.05G03180(	0.7804121	0.1915	down	no	.	pfam04749	PLAC8	PLAC8 fam	.	.	.	.
Manes.05G03200(	1.111475	0.39841	up	no	Syntaxin-71	pfam05739	SNARE	SNARE dor	GO:0006886|	GO:0005783|	GO:0008565|pr	K08506
Manes.05G03210(	0.5575261	7.96E-05	down	no	Calmodulin-b	pfam07714	Pkinase_Tyr	Protein tyro	GO:0009755|	GO:0005737|	GO:0005524|A	.
Manes.05G03230(	0.7100642	0.00716	down	no	Pentatricope	pfam13041	PPR_2	PPR repeat	.	GO:0005739|	.	.
Manes.05G03240(	0.8920854	0.29855	down	no	E3 ubiquitin-	pfam13639	zf-RING_2	Ring finger	GO:0016567|	GO:0005737|	GO:0016874|lig	.
Manes.05G03250(	0.5879389	9.68E-05	down	no	G-type lectin	pfam00069	Pkinase	Protein kina	GO:0048544|	GO:0016021|	GO:0005524|A	.
Manes.05G03260(	0.5284751	0.0002	down	no	AT-rich inter	pfam01448	ELM2	ELM2 doma	GO:0016575|	GO:0048555|	GO:0003677|D	.
Manes.05G03300(	0.8588521	0.1903	down	no	L-3-cyanoala	pfam00291	PALP	Pyridoxal-pl	GO:0019499|	GO:0005739|	GO:0004124|cy	K13034
Manes.05G03330(	0.4443997	0.00012	down	yes	Origin recogr	pfam14630	ORC5_C	Origin recog	GO:0006260|	GO:0005737|	GO:0005524|A	K02607
Manes.05G03350(	0.6100738	1.16E-06	down	no	Ascorbate tra	pfam07690	MFS_1	Major Facili	GO:0098656|	GO:0009507|	GO:0005315|in	.
Manes.05G03370(	1.0942885	0.32947	up	no	Signal peptid	pfam04258	Peptidase_A22B	Signal pepti	.	GO:0010008|	GO:0004190|as	.
Manes.05G03380(	1.6880557	0.47858	up	no	U-box domai	pfam00069	Pkinase	Protein kina	GO:0006950|	.	GO:0005524|A	.
Manes.05G03390(	6.1928387	2.38E-23	up	yes	U-box domai	pfam00069	Pkinase	Protein kina	GO:0006950|	.	GO:0005524|A	.
Manes.05G03400(	0.9799063	0.99862	down	no	Protein PAIR	pfam03124	EXS	EXS family.	GO:0051026|	GO:0005634|	.	.
Manes.05G03430(	1.0601124	0.52886	up	no	Mediator of I	pfam09497	Med12	Transcriptio	GO:0007275|	GO:0016592|	GO:0001104|R	.
Manes.05G03450(	#NAME?	0.00018	down	yes	.	pfam00168	C2	C2 domain.	.	.	.	.
Manes.05G03460(	Inf	3.32E-08	up	yes	Gibberellin 3-	pfam03171	2OG-FeII_Oxy	2OG-Fe(II)	GO:0009686|	.	GO:0016707|gil	K04124
Manes.05G03470(	1.2142117	0.03812	up	no	Transcription	pfam14215	bHLH-MYC_N	bHLH-MYC	GO:0009957|	GO:0005634|	GO:0003677|D	.
Manes.05G03480(	0.8075643	0.08498	down	no	Beta-amylase	pfam01373	Glyco_hydro_14	Glycosyl hy	GO:0000272|	GO:0009507|	GO:0016161|be	K01177
Manes.05G03490(	0.6123311	1.12E-05	down	no	Beta-amylase	pfam01373	Glyco_hydro_14	Glycosyl hy	GO:0000272|	GO:0005737|	GO:0016161|be	.
Manes.05G03510(	0.228948	1.98E-05	down	yes	.	pfam00336	DNA_pol_viral_C	DNA polym	.	.	.	.
Manes.05G03600(	1.320388	0.00557	up	no	UPF0187 pro	pfam01062	Bestrophin	Bestrophin,	.	GO:0031969|	.	.
Manes.05G03620(	1.5522789	6.86E-06	up	no	.	pfam07152	YaeQ	YaeQ protei	.	.	.	.
Manes.05G03670(	Inf	0.04761	up	no	Actin-depoly	pfam00241	Cofilin_ADF	Cofilin/trop	GO:0030042|	GO:0015629|	.	.
Manes.05G03700(	#NAME?	0.64948	down	no	.	pfam14906	DUF4495	Domain of u	.	.	.	.
Manes.05G03710(	5.4951521	1.55E-73	up	yes	Transcription	pfam00249	Myb_DNA-binding	Myb-like D	GO:0006355|	GO:0005634|	GO:0003677|D	K09422
Manes.05G03730(	0.6970277	0.04891	down	no	LIM domain-	pfam00412	LIM	LIM domain	GO:0051017|	GO:0005737|	GO:0051015|ac	.
Manes.05G03750(	0.8693276	0.23423	down	no	Phosphomann	pfam03332	PMM	Eukaryotic	GO:0009298|	GO:0005737|	GO:0004615|ph	K17497
Manes.05G03810(	0.9518097	0.71438	down	no	Digalactosyld	pfam00534	Glycos_transf_1	Glycosyl tra	GO:0009058|	GO:0009707|	GO:0046481|di	K09480
Manes.05G03830(	0.6044276	0.00597	down	no	.	pfam06218	NPR2	Nitrogen pe	.	.	.	.
Manes.05G03860(	0.3494735	9.69E-07	down	yes	.	pfam03061	4HBT	Thioesterase	.	.	.	.
Manes.05G03910(	2.4800563	7.71E-23	up	yes	Shaggy-relate	pfam00069	Pkinase	Protein kina	GO:0046777|	GO:0005829|	GO:0005524|A	K00924
Manes.05G03930(	20.265783	0.00139	up	yes	.	pfam02118	Srg	Srg family c	.	.	.	.
Manes.05G03940(	1.0930705	0.34706	up	no	Homeobox-le	pfam01852	START	START dom	GO:0043481|	GO:0005634|	GO:0008289|lip	.
Manes.05G03960(	0.6387886	0.05229	down	no	Probable met	pfam03141	Methyltransf_29	Putative S-a	.	GO:0005768|	GO:0008168|m	.
Manes.05G04090(	0.4519501	6.55E-11	down	yes	Peroxisome b	pfam08610	Pex16	Peroxisomal	GO:0006633|	GO:0005789|	.	K13335
Manes.05G04110(	0.5780066	8.02E-05	down	no	Ataxin-10 ho	pfam09759	Atx10homo_assoc	Spinocerebe	.	GO:0005737|	.	K19323
Manes.05G04170(	1.5995884	3.45E-06	up	no	CRS2-associ	pfam01985	CRS1_YhbY	CRS1 / Yhb	GO:0009793|	GO:0005739|	GO:0003723|R	.
Manes.05G04180(	0.5492475	4.62E-07	down	no	Lysophospho	pfam01553	Acyltransferase	Acyltransfer	GO:0008654|	GO:0016021|	GO:0005509|ca	K13510
Manes.05G04210(	1.7517247	1.07E-05	up	no	Histone deac	pfam06487	SAP18	Sin3 associa	GO:0006355|	GO:0005730|	.	.
Manes.05G04220(	1.2179966	0.03495	up	no	Polygalacturo	pfam01501	Glyco_transf_8	Glycosyl tra	GO:0010289|	GO:0005768|	GO:0047262|pc	K13648
Manes.05G04240(	1.2549458	0.10139	up	no	Glyoxylate/hy	pfam00389	2-Hacid_dh	D-isomer sp	GO:0009854|	.	GO:0030267|gl	K15919
Manes.05G04270(	0.9696499	0.77035	down	no	Poly(A) RNA	pfam03828	PAP_assoc	Cid1 family	GO:0006397|	GO:0005829|	GO:0005524|A	.
Manes.05G04370(	0.8048525	0.24675	down	no	Growth-regu	pfam08879	WRC	WRC. The	GO:0032502|	GO:0005634|	GO:0005524|A	.
Manes.05G04430(	0.5887858	2.66E-07	down	no	Brefeldin A-i	pfam01369	Sec7	Sec7 domain	GO:0015031|	GO:0005829|	GO:0005086|A	K18442
Manes.05G04470(	2.200492	9.21E-18	up	yes	.	pfam14897	EpsG	EpsG family	.	.	.	.
Manes.05G04500(	23.39609	0.00012	up	yes	.	pfam05553	DUF761	Cotton fibre	.	.	.	.

Manes.05G04520(	2.1501393	9.08E-10	up	yes	.	pfam07386	DUF1499	Protein of u	.	.	.	.
Manes.05G04560(	0.5177927	0.00449	down	no	Vestitone red	pfam01370	Epimerase	NAD depen	GO:0006952|	.	GO:0050662|co	.
Manes.05G04590(	4.553247	3.66E-20	up	yes	.	pfam14309	DUF4378	Domain of u	.	.	.	.
Manes.05G04610(	0.8793696	0.3131	down	no	Probable prot	pfam01529	zf-DHHC	DHHC paln	.	GO:0005794|	GO:0019706|pr	.
Manes.05G04620(	0.8277729	0.3692	down	no	.	pfam10248	Mlf1IP	Myelodyspla	.	.	.	.
Manes.05G04630(	12.246306	3.02E-06	up	yes	.	pfam07279	DUF1442	Protein of u	.	.	.	.
Manes.05G04650(	0.3225495	3.76E-09	down	yes	Protein STRU	pfam07714	Pkinase_Tyr	Protein tyro	GO:0009755|	GO:0016021|	GO:0005524|A	.
Manes.05G04670(	0.5389516	3.12E-05	down	no	.	pfam01501	Glyco_transf_8	Glycosyl tra	.	.	.	.
Manes.05G04680(	0.691468	0.0072	down	no	UDP-glucuro	pfam01370	Epimerase	NAD depen	GO:0005975|	GO:0032580|	GO:0050662|co	K08679
Manes.05G04690(	0.9992224	1	down	no	3-phosphoshi	pfam00275	EPSP_synthase	EPSP syntha	GO:0009073|	GO:0009507|	GO:0003866|3-	.
Manes.05G04700(	1.1586163	0.24555	up	no	Transketolase	pfam00456	Transketolase_N	Transketola	GO:0019253|	GO:0009535|	GO:0046872|m	K00615
Manes.05G04720(	0.8615762	0.12533	down	no	.	pfam04859	DUF641	Plant protein	.	.	.	.
Manes.05G04740(	0.7605097	0.00444	down	no	Tubulin-foldi	pfam12612	TFCD_C	Tubulin fold	GO:0009793|	GO:0005829|	GO:0048487|be	.
Manes.05G04810(	0.887701	0.25478	down	no	.	pfam04859	DUF641	Plant protein	.	.	.	.
Manes.05G04820(	0.4163324	0.00034	down	yes	.	pfam04640	PLATZ	PLATZ tran	.	.	.	.
Manes.05G04840(	9.50752	8.28E-07	up	yes	Lipid transfer	pfam14547	Hydrophob_seed	Hydrophobi	GO:0050832|	GO:0005576|	.	.
Manes.05G04870(	0.7210871	0.00179	down	no	Scarecrow-lil	pfam03514	GRAS	GRAS doma	GO:0030154|	GO:0005634|	GO:0003700|tra	.
Manes.05G04880(	0.6874638	0.0022	down	no	.	pfam13499	EF-hand_7	EF-hand do	.	.	.	.
Manes.05G04890(	0.9385663	0.55909	down	no	Vesicle-assoc	pfam00635	Motile_Sperm	MSP (Major	GO:0006605|	GO:0005829|	.	.
Manes.05G04910(	0.9448257	0.58939	down	no	.	pfam14093	DUF4271	Domain of u	.	.	.	.
Manes.05G04920(	18.843144	7.02E-60	up	yes	SPX domain-	pfam03105	SPX	SPX domain	GO:0016036|	GO:0005737|	GO:0015114|ph	.
Manes.05G04980(	10.350093	5.19E-34	up	yes	Cyclin-U2-1	pfam08613	Cyclin	Cyclin. This	GO:0007049|	.	.	.
Manes.05G05000(	0.4998528	3.75E-05	down	yes	F-box protein	pfam12799	LRR_4	Leucine Ric	.	.	.	K10268
Manes.05G05110(	1.8056464	1.79E-07	up	no	Malate dehyd	pfam02866	Ldh_1_C	lactate/mala	GO:0005975|	GO:0005737|	GO:0030060|L-	K00025
Manes.05G05130(	11.936097	1.67E-31	up	yes	Cell number	pfam04749	PLAC8	PLAC8 fam	.	GO:0016021|	.	.
Manes.05G05140(	0.6729364	0.00446	down	no	.	pfam07318	DUF1464	Protein of u	.	.	.	.
Manes.05G05180(	0.9654928	0.73155	down	no	.	pfam07819	PGAP1	PGAP1-like	.	.	.	.
Manes.05G05190(	0.84974	0.08905	down	no	E3 ubiquitin-	pfam02463	SMC_N	RecF/RecN	GO:0051301|	GO:0005739|	GO:0016874|lig	K10696
Manes.05G05200(	0.6998528	0.21966	down	no	Ethylene-resp	pfam00847	AP2	AP2 domain	GO:0009873|	GO:0005634|	GO:0003700|tra	.
Manes.05G05220(	3.7043154	3.32E-22	up	yes	DnaJ protein	pfam00226	DnaJ	DnaJ domai	GO:0006457|	GO:0005886|	GO:0005524|A	.
Manes.05G05240(	2.4039192	7.22E-23	up	yes	.	pfam09726	Macoilin	Transmembr	.	.	.	.
Manes.05G05270(	1.5703841	0.00251	up	no	UPF0481 pro	pfam03140	DUF247	Plant protein	.	GO:0016021|	.	.
Manes.05G05280(	2.9492632	8.90E-16	up	yes	.	pfam07797	DUF1639	Protein of u	.	.	.	.
Manes.05G05320(	0.1226708	0.0011	down	yes	Putative dual	pfam01184	Grp1_Fun34_Yaal	GPR1/FUN	.	.	GO:0004725|pr	.
Manes.05G05330(	0.8091128	0.04343	down	no	Cationic amir	pfam13520	AA_permease_2	Amino acid	GO:0003333|	GO:0031969|	GO:0015297|an	.
Manes.05G05340(	0.737166	0.00431	down	no	Protein ARA	pfam12937	F-box-like	F-box-like.	GO:0048527|	GO:0005737|	GO:0070696|tra	.
Manes.05G05350(	0.8152338	0.04	down	no	Palmitoyl-pro	pfam02089	Palm_thioest	Palmitoyl pr	GO:0044257|	GO:0005764|	GO:0008474|pa	K01074
Manes.05G05450(	1.3495129	0.01852	up	no	COP9 signalo	pfam01399	PCI	PCI domain	GO:0010387|	GO:0008180|	.	K12180
Manes.05G05470(	1.7505039	3.28E-10	up	no	.	pfam04782	DUF632	Protein of u	.	.	.	.
Manes.05G05480(	0.6812617	8.94E-05	down	no	Serine/threon	pfam00069	Pkinase	Protein kina	GO:0009734|	GO:0005737|	GO:0005524|A	K08286
Manes.05G05490(	Inf	0.9009	up	no	Phospholipas	pfam01764	Lipase_3	Lipase (clas	GO:0016042|	GO:0009507|	GO:0052740|1-	K16818
Manes.05G05510(	Inf	3.69E-11	up	yes	Phospholipas	pfam01764	Lipase_3	Lipase (clas	GO:0016042|	GO:0009507|	GO:0052740|1-	K16818
Manes.05G05550(	1.0254317	0.81941	up	no	Ras-related p	pfam00071	Ras	Ras family.	GO:0006888|	GO:0005789|	GO:0005525|G	K07874
Manes.05G05580(	0.918566	0.37307	down	no	.	pfam12204	DUF3598	Domain of u	.	.	.	.
Manes.05G05590(	0.5134039	2.67E-09	down	no	ELMO doma	pfam04727	ELMO_CED12	ELMO/CEI	GO:0048870|	GO:0005737|	GO:0045159|m	.
Manes.05G05610(	0.4858374	2.59E-05	down	yes	Probable WR	pfam03106	WRKY	WRKY DN	GO:0006351|	GO:0005634|	GO:0043565|se	.
Manes.05G05640(	1.4222082	0.00017	up	no	Pentatricoper	pfam13041	PPR_2	PPR repeat	.	GO:0009507|	.	.
Manes.05G05690(	1.934719	2.08E-13	up	no	Heterogeneo	pfam00076	RRM_1	RNA recogn	GO:0007623|	GO:0070937|	GO:0000166|nu	K13160
Manes.05G05770(	0.3972683	3.96E-15	down	yes	Probable doli	pfam03155	Alg6_Alg8	ALG6, ALC	GO:0006486|	GO:0005789|	GO:0016758|tra	K03849
Manes.05G05780(	0.6046352	1.82E-05	down	no	.	pfam04570	DUF581	Protein of u	.	.	.	.
Manes.05G05820(	1.4545782	0.02729	up	no	E3 ubiquitin-	pfam13639	zf-RING_2	Ring finger	GO:0016567|	GO:0016021|	GO:0016874|lig	K10663
Manes.05G05890(	Inf	0.02254	up	yes	.	pfam01504	PIP5K	Phosphatidy	.	.	.	.
Manes.05G05900(	1.0447498	0.6941	up	no	Probable puri	pfam03151	TPT	Triose-phos	GO:0006863|	GO:0016021|	GO:0005345|pu	.
Manes.05G05910(	0.917619	0.47593	down	no	.	pfam05340	DUF740	Protein of u	.	.	.	.
Manes.05G05950(	0.9906539	0.94946	down	no	Nucleolar GT	pfam06858	NOG1	Nucleolar G	GO:0042254|	GO:0005730|	GO:0005525|G	K06943
Manes.05G05960(	1.0037417	1	up	no	UMP-CMP k	pfam00406	ADK	Adenylate k	GO:0006207|	GO:0005737|	GO:0005524|A	K13800
Manes.05G05980(	1.1566169	0.62408	up	no	Protein ECEI	pfam12076	Wax2_C	WAX2 C-te	GO:0006633|	GO:0005789|	GO:0005506|irc	K15404
Manes.05G05990(	1.1995781	0.15088	up	no	Protein ECEI	pfam12076	Wax2_C	WAX2 C-te	GO:0006633|	GO:0005789|	GO:0005506|irc	K15404
Manes.05G06060(	0.496666	1.21E-09	down	yes	WD-40 repea	pfam12265	CAF1C_H4-bd	Histone-bin	.	GO:0005634|	.	K10752
Manes.05G06070(	0.9502409	0.59609	down	no	Ribose-phosp	pfam13793	Pribosyltran_N	N-terminal	GO:0009165|	.	GO:0005524|A	.
Manes.05G06100(	0.956621	0.65396	down	no	Protoheme IX	pfam01040	UbiA	UbiA prenyl	GO:0048034|	GO:0016021|	GO:0008495|pr	K02257
Manes.05G06110(	5.5444343	6.43E-10	up	yes	Protein NRT	pfam00854	PTR2	POT family.	GO:0015706|	GO:0016021|	GO:0015112|nit	.
Manes.05G06120(	0.8468359	0.1863	down	no	Probable seri	pfam00069	Pkinase	Protein kina	GO:0009742|	GO:0016020|	GO:0005524|A	K14500
Manes.05G06130(	7.7726355	2.78E-31	up	yes	Indole-3-acet	pfam03242	LEA_3	Late embryo	GO:0006950|	.	.	.
Manes.05G06150(	4.9765024	0.00016	up	yes	.	pfam03242	LEA_3	Late embryo	.	.	.	.
Manes.05G06160(	0.7209869	0.03909	down	no	Mediator of I	pfam05766	NinG	Bacteriopha	GO:0006355|	GO:0016592|	.	K15141
Manes.05G06180(	0.3886517	1.38E-15	down	yes	Gamma-tubu	pfam04130	Spc97_Spc98	Spc97 / Spc	GO:0043622|	GO:0005737|	.	.
Manes.05G06190(	1.1327624	0.16648	up	no	Blue-light ph	pfam00875	DNA_photolyase	DNA photo	GO:0006281|	.	GO:0003913|D	.
Manes.05G06200(	1.6432383	6.41E-05	up	no	Probable sma	pfam01423	LSM	LSM domai	GO:0000398|	GO:0005829|	.	K11096
Manes.05G06220(	0.3457968	2.42E-15	down	yes	21 kDa prote	pfam04043	PMEI	Plant inverta	.	.	GO:0004857|en	.
Manes.05G06240(	0.9190466	0.37445	down	no	Serine/argini	pfam00076	RRM_1	RNA recogn	GO:0006397|	GO:0035061|	GO:0000166|nu	K12890
Manes.05G06260(	0.4384474	2.40E-07	down	yes	Protein OPI1	pfam05603	DUF775	Protein of u	GO:0006606|	GO:0005829|	.	.
Manes.05G06270(	2.1131831	0.00139	up	yes	Beta-glucosic	pfam00232	Glyco_hydro_1	Glycosyl hy	GO:0005975|	.	GO:0008422|be	K01188
Manes.05G06290(	1.4666691	5.89E-05	up	no	Universal stre	pfam00582	Usp	Universal st	GO:0006950|	GO:0005886|	GO:0016208|A	.
Manes.05G06300(	1.6473098	3.21E-07	up	no	Probable E3	pfam03105	SPX	SPX domain	GO:0016036|	.	GO:0016874|lig	.
Manes.05G06320(	1.1966488	0.40257	up	no	ABC transpo	pfam00664	ABC_membrane	ABC transp	.	GO:0005794|	GO:0005524|A	.
Manes.05G06330(	0.9028532	0.4587	down	no	Uncharacteri:	pfam04969	CS	CS domain.	.	GO:0005829|	.	.
Manes.05G06340(	0.8952038	0.34808	down	no	Probable U6	pfam01423	LSM	LSM domai	GO:0006397|	GO:0005681|	GO:0003723|R	.
Manes.05G06370(	1.0087435	1	up	no	.	pfam05755	REF	Rubber elon	.	.	.	.
Manes.05G06380(	1.3471885	0.03306	up	no	Uncharacteri:	pfam13532	2OG-FeII_Oxy_2	2OG-Fe(II)	.	.	GO:0016706|ox	.
Manes.05G06390(	3.8098893	9.03E-10	up	yes	Translocator	pfam03073	TspO_MBR	TspO/MBR	GO:0006778|	GO:0031969|	GO:0020037|he	.
Manes.05G06400(	NA	NA	--	no	.	pfam03053	Corona_NS3b	ORF3b cor	.	.	.	.
Manes.05G06410(	0.4830227	9.52E-09	down	yes	Dol-P-Man:M	pfam05208	ALG3	ALG3 prote	GO:0097502|	GO:0005783|	GO:0000033|al	K03845
Manes.05G06420(	0.4484759	0.00094	down	yes	.	pfam06825	HSBP1	Heat shock	.	.	.	.
Manes.05G06440(	0.8143359	0.40894	down	no	Bifunctional	pfam02882	THF_DHG_CYH	Tetrahydrof	GO:0009396|	GO:0009507|	GO:0004477|m	.
Manes.05G06450(	1.0634581	0.68657	up	no	.	pfam05088	Bac_GDH	Bacterial N	.	.	.	.
Manes.05G06460(	1.1549221	0.32269	up	no	Heavy metal-	pfam00403	HMA	Heavy-meta	GO:0010286|	GO:0031965|	GO:0046872|m	.
Manes.05G06480(	0.5763122	0.00229	down	no	Glutathione S	pfam02798	GST_N	Glutathione	GO:0009734|	.	GO:0004364|gl	.

Manes.05G06520(	2.8423437	2.44E-14	up	yes	Potassium tra	pfam02705	K_trans	K+ potassiu	.	GO:0016021|	GO:0015079|pc	K03549
Manes.05G06560(	0.428855	0.00138	down	yes	.	pfam07516	SecA_SW	SecA Wing	.	.	.	.
Manes.05G06580(	0.8937526	0.39476	down	no	Metal toleran	pfam01545	Cation_efflux	Cation efflu	.	GO:0016021|	GO:0008324|ca	.
Manes.05G06610(	0.9078017	0.31466	down	no	Peroxisome b	pfam00004	AAA	ATPase fam	GO:0006635|t	GO:0005737|	GO:0005524|A	K13339
Manes.05G06620(	0.4881625	2.87E-06	down	yes	.	pfam02536	mTERF	mTERF. Th	.	.	.	.
Manes.05G06630(	1.1015702	0.3132	up	no	Spermatogen	pfam03190	Thioredox_DsbH	Protein of u	GO:0030154|c	GO:0005576|	GO:0003824|ca	.
Manes.05G06640(	0.9445986	0.54108	down	no	Zinc finger C	pfam00642	zf-CCCH	Zinc finger (	.	GO:0005634|	GO:0003677|D	.
Manes.05G06680(	1.7543451	6.08E-08	up	no	OTU domain	pfam02338	OTU	OTU-like cy	.	.	GO:0008234|cy	K18342
Manes.05G06700(	6.9298276	8.01E-08	up	yes	Monothiol gl	pfam00462	Glutaredoxin	Glutaredoxi	GO:0045454|c	GO:0005737|	GO:0051537|2	.
Manes.05G06720(	0.7057739	0.00117	down	no	Membrane-ar	pfam13881	Rad60-SLD_2	Ubiquitin-2	.	GO:0005886|	.	.
Manes.05G06740(	5.3855745	0.31072	up	no	Bidirectional	pfam03083	MtN3_slv	Sugar efflux	GO:0034219|c	GO:0005887|	GO:0051119|su	K15382
Manes.05G06770(	Inf	0.02355	up	yes	.	pfam03321	GH3	GH3 auxin-r	.	.	.	.
Manes.05G06780(	0.6652567	3.08E-05	down	no	Protein TRA	pfam12937	F-box-like	F-box-like.	GO:0009734|a	GO:0005634|	GO:0010011|au	K14485
Manes.05G06790(	0.4638692	7.53E-14	down	yes	Tubby-like F	pfam01167	Tub	Tub family.	.	GO:0005654|	.	.
Manes.05G06800(	0.4895673	4.55E-09	down	yes	.	pfam07765	KIP1	KIP1-like pr	.	.	.	.
Manes.05G06820(	0.3171883	0.00011	down	yes	.	pfam09878	DUF2105	Predicted m	.	.	.	.
Manes.05G06870(	0.4339975	2.52E-06	down	yes	PAX-interact	pfam00533	BRCT	BRCA1 C T	GO:0060612|a	GO:0035097|	.	K14972
Manes.05G06910(	2.6497368	3.73E-28	up	yes	CUGBP Elav	pfam00076	RRM_1	RNA recogn	GO:0006397|r	GO:0005737|	GO:0000166|nu	K13207
Manes.05G06920(	4.8068345	2.95E-10	up	yes	.	pfam03155	Alg6_Alg8	ALG6, ALG	.	.	.	.
Manes.05G06980(	1.607219	9.78E-07	up	no	Cytochrome	pfam09592	DUF2031	Protein of u	.	GO:0016021|	.	.
Manes.05G07020(	3.3111016	3.36E-22	up	yes	Histone H2A	pfam00125	Histone	Core histone	.	GO:0000786|	GO:0003677|D	.
Manes.05G07050(	2.5858575	2.57E-11	up	yes	Gibberellin re	pfam07859	Abhydrolase_3	alpha/beta h	GO:0048444|f	GO:0005634|	GO:0016787|hy	K14493
Manes.05G07080(	1.756665	2.59E-10	up	no	.	pfam00400	WD40	WD domain	.	.	.	.
Manes.05G07130(	Inf	0.00382	up	yes	.	pfam10325	7TM_GPCR_Srz	Serpentine t	.	.	.	.
Manes.05G07140(	0.7937018	0.01671	down	no	Sugar transpo	pfam00083	Sugar_tr	Sugar (and c	GO:0046323|g	GO:0009507|	GO:0005355|gl	.
Manes.05G07150(	3.669828	2.70E-30	up	yes	.	pfam03372	Exo_endo_phos	Endonucleas	.	.	.	.
Manes.05G07170(	1.0154137	0.84807	up	no	Piezo-type m	pfam12166	DUF3595	Protein of u	.	GO:0016021|	GO:0008381|m	.
Manes.05G07180(	0.62117	4.27E-06	down	no	.	pfam04526	DUF568	Protein of u	.	.	.	.
Manes.05G07190(	1.1541226	0.14858	up	no	.	pfam02536	mTERF	mTERF. Th	.	.	.	.
Manes.05G07210(	0.296786	4.71E-05	down	yes	.	pfam12031	DUF3518	Domain of u	.	.	.	.
Manes.05G07220(	0.7782074	0.03714	down	no	.	pfam03909	BSD	BSD domain	.	.	.	.
Manes.05G07230(	0.8838525	0.46738	down	no	.	pfam03171	2OG-FeII_Oxy	2OG-Fe(II)	.	.	.	.
Manes.05G07250(	Inf	1.57E-07	up	yes	.	pfam03231	Bunya_NS-S_2	Bunyavirus	.	.	.	.
Manes.05G07270(	1.7083846	7.03E-09	up	no	Mitochondria	pfam12483	GIDE	E3 Ubiquitin	GO:0006919|a	GO:0030424|	GO:0042802|id	K15688
Manes.05G07300(	1.9233328	6.84E-13	up	no	BTB/POZ an	pfam00651	BTB	BTB/POZ d	GO:0071472|c	GO:0005634|	.	K10523
Manes.05G07360(	182.17326	5.69E-27	up	yes	EID1-like F-l	.	.	.	GO:0009738|a	GO:0005634|	.	.
Manes.05G07390(	10.629389	#######	up	yes	.	pfam08137	DVL	DVL family	.	.	.	.
Manes.05G07400(	10.382522	1.29E-59	up	yes	.	pfam14897	EpsG	EpsG family	.	.	.	.
Manes.05G07410(	3.9087973	6.52E-26	up	yes	Protein ROO	pfam11955	PORR	Plant organ	GO:0010102|l	.	.	.
Manes.05G07420(	Inf	9.03E-10	up	yes	.	pfam09765	WD-3	WD-repeat	.	.	.	.
Manes.05G07440(	1.4833347	1.15E-05	up	no	.	pfam13902	R3H-assoc	R3H-associ	.	.	.	.
Manes.05G07450(	0.8512813	0.20709	down	no	Uncharacteriz	pfam02567	PhzC-PhzF	Phenazine b	GO:0009058|l	.	GO:0016853|iso	.
Manes.05G07460(	0.7008266	0.00777	down	no	Uncharacteriz	pfam02567	PhzC-PhzF	Phenazine b	GO:0009058|l	.	GO:0016853|iso	.
Manes.05G07470(	0.721969	0.11625	down	no	Uncharacteriz	pfam02567	PhzC-PhzF	Phenazine b	GO:0009058|l	.	GO:0016853|iso	.
Manes.05G07480(	0.5723948	0.00979	down	no	Basic 7S glot	pfam14541	TAXi_C	Xylanase inl	.	.	GO:0004190|as	.
Manes.05G07510(	1.463144	0.06559	up	no	.	pfam12339	DNAJ_related	DNA-J relat	.	.	.	.
Manes.05G07530(	0.5316903	0.00078	down	no	Antifungal pr	pfam01657	Stress-antifung	Salt stress re	GO:0042742|c	.	GO:0019828|as	.
Manes.05G07550(	0.6658804	0.00044	down	no	DNA repair h	pfam06733	DEAD_2	DEAD_2. T	GO:0006281|l	GO:0005634|	GO:0051539|4	K10844
Manes.05G07570(	3.0339867	0.00526	up	yes	.	pfam07699	GCC2_GCC3	GCC2 and C	.	.	.	.
Manes.05G07590(	0.6299792	0.02256	down	no	Mitochondria	pfam08356	EF_assoc_2	EF hand ass	GO:0007005|r	GO:0031307|	GO:0005509|ca	K07870
Manes.05G07600(	2.0720552	1.64E-05	up	yes	.	pfam11250	DUF3049	Protein of u	.	.	.	.
Manes.05G07620(	Inf	0.9009	up	no	.	pfam13102	Phage_int_SAM_5	Phage integr	.	.	.	.
Manes.05G07670(	0.7286525	0.00214	down	no	Cyclin-P3-1	pfam08613	Cyclin	Cyclin. This	GO:0007049|c	.	.	.
Manes.05G07690(	0.4057413	3.29E-13	down	yes	Probable inac	pfam01734	Patatin	Patatin-like	GO:0016042|l	.	GO:0016787|hy	.
Manes.05G07730(	0.5156691	3.07E-08	down	no	F-box protein	pfam12937	F-box-like	F-box-like.	GO:0016567|j	GO:0005634|	.	K10268
Manes.05G07750(	0.4848598	1.79E-09	down	yes	.	pfam05910	DUF868	Plant protein	.	.	.	.
Manes.05G07780(	0.6735666	0.00466	down	no	.	pfam10243	MIP-T3	Microtubule	.	.	.	.
Manes.05G07800(	0.5343154	0.00311	down	no	Transcription	pfam04844	Ovate	Transcriptio	GO:0045892|r	GO:0005634|	.	.
Manes.05G07830(	2.2610535	1.02E-17	up	yes	Methylcrotor	pfam02786	CPSase_L_D2	Carbamoyl-p	GO:0006552|l	GO:0005759|	GO:0005524|A	.
Manes.05G07840(	1.5156176	1.92E-05	up	no	Serine/threon	pfam07714	Pkinase_Tyr	Protein tyro	.	.	GO:0005524|A	.
Manes.05G07870(	0.5078206	0.00306	down	no	TP53-regulat	pfam00069	Pkinase	Protein kina	GO:0006468|p	GO:0005829|	GO:0005524|A	K08851
Manes.05G07900(	21.380882	0.00057	up	yes	Vicilin-like ar	pfam00190	Cupin_1	Cupin. This	GO:0042742|c	GO:0005576|	GO:0045735|nu	.
Manes.05G07920(	0.9749721	0.81746	down	no	50S ribosoma	pfam00467	KOW	KOW motif.	GO:0006412|t	GO:0005840|	GO:0019843|rR	K02895
Manes.05G07960(	1.7481032	2.07E-05	up	no	F-box/kelch-r	pfam01344	Kelch_1	Kelch motif.	GO:0016567|p	.	.	.
Manes.05G07970(	1.1831842	0.15453	up	no	tRNA (guani	pfam00400	WD40	WD domain	GO:0036265|l	GO:0005634|	.	.
Manes.05G07980(	0.7261599	0.128	down	no	.	pfam04570	DUF581	Protein of u	.	.	.	.
Manes.05G07990(	1.1638891	0.09227	up	no	.	pfam15246	NCKAP5	Nck-associa	.	.	.	.
Manes.05G08010(	0.5702476	0.23637	down	no	.	pfam14505	DUF4438	Domain of u	.	.	.	.
Manes.05G08020(	0.6381125	0.9721	down	no	.	pfam12718	Tropomyosin_1	Tropomyosi	.	.	.	.
Manes.05G08040(	0.5957769	7.69E-06	down	no	Sulfate transp	pfam00916	Sulfate_transp	Sulfate trans	GO:1902358|s	GO:0009507|	GO:0008271|se	K17471
Manes.05G08050(	0.3640602	0.04316	down	no	Serine/threon	pfam00069	Pkinase	Protein kina	GO:0006468|j	.	GO:0005524|A	K08857
Manes.05G08140(	0.6656233	0.00335	down	no	Protein TIFY	pfam06200	tify	tify domain.	GO:0048366|l	GO:0005634|	.	.
Manes.05G08160(	0.8074842	0.08322	down	no	Probable inac	pfam00481	PP2C	Protein phos	GO:0006470|j	.	GO:0005524|A	.
Manes.05G08170(	0.1157222	1.39E-06	down	yes	Heat stress tr	pfam00447	HSF_DNA-bind	HSF-type D	GO:0009408|r	GO:0005737|	GO:0043565|se	K09419
Manes.05G08180(	0.8745193	0.22623	down	no	Zinc finger pr	pfam01753	zf-MYND	MYND fing	GO:0006355|r	GO:0005634|	GO:0046872|m	.
Manes.05G08190(	0.9453106	0.62307	down	no	Putative lysin	pfam13621	Cupin_8	Cupin-like d	GO:0007623|c	GO:0005737|	GO:0051213|di	.
Manes.05G08220(	0.6782014	0.01527	down	no	.	pfam09897	DUF2124	Uncharacter	.	.	.	.
Manes.05G08230(	14.03077	0.0474	up	no	Calmodulin-li	pfam13499	EF-hand_7	EF-hand do	GO:0019722|c	GO:0005622|	GO:0005509|ca	K13448
Manes.05G08270(	1.3278444	0.0022	up	no	Protein LON	pfam14309	DUF4378	Domain of u	GO:0051513|r	GO:0005829|	.	.
Manes.05G08280(	2.2077636	0.05987	up	no	.	pfam02153	PDH	Prephenate	.	.	.	.
Manes.05G08310(	0.9883315	0.92026	down	no	.	pfam04720	DUF506	Protein of u	.	.	.	.
Manes.05G08330(	NA	NA	--	no	.	pfam00131	Metallothio	Metallothior	.	.	.	.
Manes.05G08370(	0.7402879	0.02341	down	no	Guanylate-bi	pfam02263	GBP	Guanylate-b	.	GO:0005737|	GO:0005525|G	.
Manes.05G08390(	0.9036281	0.30342	down	no	Light-inducib	pfam12498	bZIP_C	Basic leucin	GO:0006351|t	GO:0005634|	GO:0043565|se	.
Manes.05G08450(	0.6938932	0.00732	down	no	Putative uridi	pfam00485	PRK	Phosphoribu	GO:0044211|t	GO:0005829|	GO:0005524|A	.
Manes.05G08460(	3.5563693	1.15E-13	up	yes	Phospholipas	pfam01764	Lipase_3	Lipase (clas	GO:0016042|l	GO:0009507|	GO:0047714|ga	.
Manes.05G08480(	0.5828377	5.26E-07	down	no	Photosystem	pfam07123	PsbW	Photosysten	GO:0015979|p	GO:0009535|	.	.
Manes.05G08490(	0.9761807	0.81817	down	no	Probable gala	pfam01501	Glyco_transf_8	Glycosyl tra	GO:0071555|c	GO:0000139|	GO:0047262|pc	.

Manes.05G08550(	NA	NA	--	no	.	pfam04976	DmsC	DMSO redu	.	.	.	.
Manes.05G08580(	0.8487282	0.23615	down	no	Probable flav	pfam00743	FMO-like	Flavin-bindi	GO:0071456|(	.	GO:0050660|fla	K00485
Manes.05G08590(	0.7759851	0.09987	down	no	40S ribosoma	pfam00203	Ribosomal_S19	Ribosomal p	GO:0006412|t	GO:0015935|s	GO:0003723|RI	.
Manes.05G08610(	6.036699	7.74E-47	up	yes	.	pfam08016	PKD_channel	Polycystin c	.	.	.	.
Manes.05G08700(	0.9770771	0.90937	down	no	.	pfam01344	Kelch_1	Kelch motif.	.	.	.	.
Manes.05G08740(	0.9681012	0.73713	down	no	Bet1-like SN	pfam05739	SNARE	SNARE dor	GO:0015031|p	GO:0005789|e	.	K08504
Manes.05G08750(	1.2432537	0.02184	up	no	Deoxyhypusi	pfam13646	HEAT_2	HEAT repea	GO:0008612|p	GO:0005829|e	GO:0019135|de	K06072
Manes.05G08790(	7.8817421	2.61E-62	up	yes	Probable alph	pfam00982	Glyco_transf_20	Glycosyltran	GO:0005992|t	.	GO:0016757|tra	K16055
Manes.05G08800(	2.2854643	1.08E-20	up	yes	.	pfam01436	NHL	NHL repeat	.	.	.	.
Manes.05G08820(	0.7053813	0.00441	down	no	.	pfam01436	NHL	NHL repeat	.	.	.	.
Manes.05G08930(	0.4978352	0.02166	down	yes	Probable carl	pfam07859	Abhydrolase_3	alpha/beta h	.	.	GO:0052689|ca	.
Manes.05G09000(	0.7735673	0.02764	down	no	MLP-like prc	pfam00407	Bet_v_1	Pathogenesi	GO:0006952|c	GO:0016020|r	.	.
Manes.05G09020(	0.3385514	7.82E-08	down	yes	.	pfam00583	Acetyltransf_1	Acetyltransf	.	.	.	.
Manes.05G09030(	1.2067811	0.1086	up	no	Protein ALTI	pfam13839	PC-Esterase	GDSL/SGN	GO:0010411|:	GO:0005794|(	GO:0016413|O-	.
Manes.05G09040(	0.8007211	0.08433	down	no	Nucleobase-a	pfam00860	Xan_ur_permease	Permease fa	GO:0055085|t	GO:0016021|i	GO:0005215|tra	.
Manes.05G09050(	1.1412902	0.17894	up	no	.	pfam14009	DUF4228	Domain of u	.	.	.	.
Manes.05G09060(	0.1815223	4.87E-23	down	yes	Cyclin-D1-1	pfam00134	Cyclin_N	Cyclin, N-te	GO:0007049|c	GO:0005634|r	.	K18810
Manes.05G09090(	2.0127047	9.54E-12	up	yes	DEAD-box A	pfam00270	DEAD	DEAD/DEA	GO:0010501|I	GO:0009507|c	GO:0005524|A'	.
Manes.05G09100(	6.9805536	0.00029	up	yes	.	pfam15001	AP-5_subunit_s1	AP-5 compl	.	.	.	.
Manes.05G09130(	0.8408243	0.12162	down	no	Protein NLR(	pfam13855	LRR_8	Leucine rich	GO:0007249|I	GO:0005737|(	GO:0005524|A'	.
Manes.05G09140(	0.7837359	0.02736	down	no	.	pfam13516	LRR_6	Leucine Ric	.	.	.	.
Manes.05G09160(	0.7057966	0.0014	down	no	Chaperone pi	pfam00226	DnaJ	DnaJ domai	GO:0009737|r	GO:0016020|r	.	.
Manes.05G09200(	1.8623925	1.68E-07	up	no	Probable gala	pfam01501	Glyco_transf_8	Glycosyl tra	GO:0071555|c	GO:0005794|(	GO:0047262|pc	.
Manes.05G09210(	1.1750185	0.07655	up	no	.	pfam06886	TPX2	Targeting pi	.	.	.	.
Manes.05G09220(	0.3886116	9.77E-06	down	yes	Two-compor	pfam00072	Response_reg	Response re	GO:0007623|c	GO:0005737|(	GO:0000156|ph	K14492
Manes.05G09240(	0.4301211	2.12E-06	down	yes	Non-function	pfam00248	Aldo_ket_red	Aldo/keto re	.	GO:0005829|(	GO:0016491|ox	.
Manes.05G09250(	0.1730773	0.0496	down	no	.	pfam00999	Na_H_Exchanger	Sodium/hyd	.	.	.	.
Manes.05G09270(	0.0561512	2.47E-05	down	yes	Uncharacteri:	pfam00400	WD40	WD domain	.	.	GO:0004197|cy	.
Manes.05G09280(	1.5454732	0.00017	up	no	.	pfam11808	DUF3329	Domain of u	.	.	.	.
Manes.05G09290(	0.719035	0.00513	down	no	.	pfam00536	SAM_1	SAM domai	.	.	.	.
Manes.05G09310(	3.2196907	2.36E-05	up	yes	Zinc finger pi	pfam13912	zf-C2H2_6	C2H2-type	GO:0009736|(	GO:0005737|(	GO:0046872|m(	.
Manes.05G09340(	1.5197535	5.37E-06	up	no	Paired amphi	pfam08295	Sin3_corepress	Sin3 family	GO:0006355|r	GO:0005634|r	.	K11644
Manes.05G09350(	0.5979317	1.19E-06	down	no	Microtubule-	pfam03251	Tymo_45kd_70kd	Tymovirus 4	GO:0010031|c	GO:0010005|(	GO:0008017|mi	.
Manes.05G09360(	3.4615234	1.53E-12	up	yes	UPF0392 prc	pfam01697	Glyco_transf_92	Glycosyltran	.	GO:0016021|i	.	.
Manes.05G09400(	0.5295245	4.70E-08	down	no	Serine/threon	pfam00149	Metallophos	Calcineurin-	.	GO:0005737|(	GO:0046872|m(	.
Manes.05G09420(	0.4133393	0.00026	down	yes	CLAVATA3	pfam00509	Hemagglutinin	Haemagglut	GO:0045168|(	GO:0005615|(	GO:0033612|re	.
Manes.05G09430(	2.4548527	1.72E-21	up	yes	TMV resistar	pfam00931	NB-ARC	NB-ARC dc	GO:0009626|p	GO:0005737|(	GO:0043531|Al	.
Manes.05G09440(	2.1916289	5.88E-19	up	yes	Phospholipid	pfam13246	Hydrolase_like2	Putative hyc	GO:0048194|(	GO:0005789|(	GO:0005524|A'	K14802
Manes.05G09470(	1.4765458	6.24E-05	up	no	Transcription	pfam00010	HLH	Helix-loop-l	GO:0048446|p	GO:0005634|r	GO:0003677|DI	.
Manes.05G09500(	1.3573282	0.03835	up	no	Probable beta	pfam00337	Gal-bind_lectin	Galactoside-	GO:0006486|p	GO:0009507|c	GO:0030246|ca	.
Manes.05G09510(	0.6188336	0.00329	down	no	Pentatricope	pfam13041	PPR_2	PPR repeat	.	GO:0005739|r	.	.
Manes.05G09560(	1.2388439	0.01769	up	no	AMP deamin	pfam00962	A_deaminase	Adenosine/A	GO:0009793|(	GO:0005829|(	GO:0003876|Al	K01490
Manes.05G09570(	0.822929	0.20662	down	no	Protein NRT	pfam00854	PTR2	POT family.	GO:0080055|l	GO:0016021|i	GO:0080054|lo	.
Manes.05G09580(	0.898369	0.53136	down	no	.	pfam13041	PPR_2	PPR repeat	.	.	.	.
Manes.05G09590(	2.1855938	1.02E-13	up	yes	Uncharacteri:	pfam14259	RRM_6	RNA recogi	GO:0006355|r	.	GO:0000166|nu	.
Manes.05G09610(	0.3721382	1.13E-06	down	yes	Homeobox-le	pfam00046	Homeobox	Homeobox c	GO:0009965|l	GO:0005634|r	GO:0042803|pr	K09338
Manes.05G09630(	17.579713	4.54E-14	up	yes	Galactose ox	pfam07250	Glyoxal_oxid_N	Glyoxal oxic	.	GO:0005576|(	GO:0045480|ga	.
Manes.05G09670(	0.7133672	0.00436	down	no	Probable rece	pfam07714	Pkinase_Tyr	Protein tyro	.	GO:0016021|i	GO:0005524|A'	.
Manes.05G09680(	2.9703945	5.86E-26	up	yes	.	pfam07063	DUF1338	Domain of u	.	.	.	.
Manes.05G09710(	0.7572214	0.07146	down	no	Alpha-amylas	pfam00128	Alpha-amylase	Alpha amyla	GO:0005983|s	GO:0009507|c	GO:0004556|alp	.
Manes.05G09730(	3.7533112	0.39312	up	no	Probable pect	pfam00544	Pec_lyase_C	Pectate lyas(	GO:0045490|p	.	GO:0046872|m(	K01728
Manes.05G09800(	0.9632263	0.76991	down	no	Disease resist	pfam00931	NB-ARC	NB-ARC dc	GO:0006952|c	.	GO:0043531|Al	.
Manes.05G09830(	3.2007035	8.42E-39	up	yes	Vacuolar pro	pfam12850	Metallophos_2	Calcineurin-	GO:0006623|p	GO:0005794|(	GO:0008270|zin	K18467
Manes.05G09840(	6.4080275	2.36E-99	up	yes	Protein kinas	pfam07714	Pkinase_Tyr	Protein tyro	GO:0009755|l	GO:0005737|(	GO:0005524|A'	K04733
Manes.05G09850(	4.6834545	0.01104	up	yes	.	pfam08303	tRNA_lig_kinase	tRNA ligase	.	.	.	.
Manes.05G09870(	0.5273878	2.64E-08	down	no	Myb-related p	pfam13921	Myb_DNA-bind_6	Myb-like DI	GO:0000086|(	GO:0031523|	GO:0000978|RI	K09421
Manes.05G09900(	1.0049524	0.95341	up	no	Sucrose trans	pfam07690	MFS_1	Major Facili	GO:0009611|r	GO:0005794|(	GO:0008515|su	K15378
Manes.05G09950(	1.2442767	0.09574	up	no	F-box/kelch-i	pfam01344	Kelch_1	Kelch motif.	GO:0016567|p	GO:0005634|r	.	.
Manes.05G09990(	0.4357133	4.18E-10	down	yes	Ultraviolet-B	pfam08381	BRX	Transcriptio	GO:0009649|(	GO:0000785|(	GO:0003682|ch	.
Manes.05G10000(	0.4807386	1.30E-08	down	yes	HVA22-like p	pfam03134	TB2_DP1_HVA22	TB2/DP1, H	GO:0042538|l	GO:0016021|i	.	.
Manes.05G10010(	2.8996539	6.69E-31	up	yes	Transcription	pfam03634	TCP	TCP family	GO:0008283|(	GO:0005634|r	GO:0003677|DI	.
Manes.05G10030(	0.9516639	0.6964	down	no	Haloacid deh	pfam13419	HAD_2	Haloacid de	.	GO:0005739|r	GO:0016787|hy	.
Manes.05G10060(	2.3267898	4.57E-17	up	yes	Sphinganine (	pfam04116	FA_hydroxylase	Fatty acid h	GO:0006633|f	GO:0005783|(	GO:0005506|irc	K04713
Manes.05G10120(	0.7237288	0.13158	down	no	.	pfam07891	DUF1666	Protein of u	.	.	.	.
Manes.05G10160(	0.5146765	5.17E-07	down	no	UDP-galacto	pfam08449	UAA	UAA transp	GO:0008643|(	GO:0030176|i	GO:0005460|UI	K15275
Manes.05G10190(	2.2377366	5.41E-06	up	yes	Protein HOT	pfam05199	GMC_oxred_C	GMC oxido	GO:0007267|(	GO:0005576|(	GO:0050660|fla	.
Manes.05G10230(	0.4541743	1.90E-10	down	yes	Beta-1,3-gala	pfam01762	Galactosyl_T	Galactosyltr	GO:0010493|l	GO:0005794|(	GO:0030246|ca	K14413
Manes.05G10240(	6.1840093	3.53E-10	up	yes	Ribonuclease	pfam00445	Ribonuclease_T2	Ribonucleas	GO:0006401|I	GO:0005576|(	GO:0004521|en	K01166
Manes.05G10250(	0.5047338	0.29354	down	no	Extracellular	pfam00445	Ribonuclease_T2	Ribonucleas	.	GO:0005618|(	GO:0033897|rib	K01166
Manes.05G10260(	0.3926887	8.90E-11	down	yes	.	pfam14818	DUF4482	Domain of u	.	.	.	.
Manes.05G10270(	Inf	0.00042	up	yes	Vacuolar ami	pfam01490	Aa_trans	Transmembi	GO:0003333|a	GO:0000324|r	GO:0015186|L-	K15015
Manes.05G10310(	1.5026019	1.13E-05	up	no	Cyclic dof fac	pfam02701	zf-Dof	Dof domain	GO:0009908|t	GO:0005634|r	GO:0003677|DI	.
Manes.05G10340(	0.3389107	0.00039	down	yes	.	.	.	.	.	.	.	.
Manes.05G10410(	3.0761411	3.17E-27	up	yes	Methyltransfe	pfam08241	Methyltransf_11	Methyltrans	GO:0032259|r	GO:0005783|(	GO:0008757|S-	.
Manes.05G10440(	0.7118877	0.02305	down	no	Photosynthet	pfam05757	PsbQ	Oxygen evo	GO:0009767|p	GO:0009507|c	GO:0005509|ca	K08901
Manes.05G10470(	309.68023	2.09E-38	up	yes	Cytochrome I	pfam00067	p450	Cytochrome	GO:0010584|p	GO:0016021|i	GO:0018685|all	.
Manes.05G10520(	0.6550549	0.00095	down	no	.	pfam04041	DUF377	Domain of u	.	.	.	.
Manes.05G10550(	11.262456	4.32E-05	up	yes	Phosphate tra	pfam03124	EXS	EXS family.	GO:0016036|(	GO:0005737|(	GO:0015114|ph	.
Manes.05G10590(	3.4486477	6.84E-10	up	yes	.	pfam11360	DUF3110	Protein of u	.	.	.	.
Manes.05G10630(	0.5698523	1.33E-06	down	no	CSC1-like pr	pfam02714	DUF221	Domain of u	GO:0006811|i	GO:0016021|i	.	.
Manes.05G10680(	5.5869952	6.28E-14	up	yes	.	pfam07059	DUF1336	Protein of u	.	.	.	.
Manes.05G10690(	2.5624615	5.08E-26	up	yes	Probable WR	pfam03106	WRKY	WRKY DN	GO:0006351|t	GO:0005634|r	GO:0043565|se	.
Manes.05G10700(	1.9631674	8.54E-12	up	no	Eukaryotic tr	pfam01287	eIF-5a	Eukaryotic (	GO:0045901|p	.	GO:0043022|rib	.
Manes.05G10720(	0.2301413	1.34E-10	down	yes	Peroxidase N	pfam00141	peroxidase	Peroxidase.	GO:0042744|l	GO:0005576|(	GO:0020037|he	.
Manes.05G10750(	0.5189484	7.26E-10	down	no	.	pfam05904	DUF863	Plant protei	.	.	.	.
Manes.05G10760(	1.1204495	0.23107	up	no	.	pfam13716	CRAL_TRIO_2	Divergent C	.	.	.	.
Manes.05G10770(	0.7409372	0.00984	down	no	.	pfam11708	Slu7	Pre-mRNA	.	.	.	.
Manes.05G10810(	7.0301454	3.24E-15	up	yes	Probable xylc	pfam00722	Glyco_hydro_16	Glycosyl hy(	GO:0042546|(	GO:0048046|a	GO:0004553|hy	K08235

Manes.05G10820(	1.3324426	0.00624	up	no	Probable leuc	pfam13855	LRR_8	Leucine rich	.	GO:0016021|	GO:0005524|A'	.
Manes.05G10840(	0.3730783	0.48262	down	no	Putative SNA	pfam05739	SNARE	SNARE dor	GO:0061025|	GO:0005737|	GO:0005484|SN	K18211
Manes.05G10850(	0.7244675	0.00235	down	no	Alpha-1,4 glu	pfam00343	Phosphorylase	Carbohydra	GO:0005975|	GO:0009501|	GO:0008184|gly	K00688
Manes.05G10870(	0.5555025	0.00415	down	no	Zinc finger pr	pfam13912	zf-C2H2_6	C2H2-type	GO:0006355|	GO:0005634|	GO:0046872|me	.
Manes.05G10880(	0.7085945	0.00081	down	no	Probable met	pfam03141	Methyltransf_29	Putative S-a	GO:0032259|	GO:0005789|	GO:0008757|S-	.
Manes.05G10890(	0.310575	7.24E-07	down	yes	14-3-3-like p	pfam00244	2014/3/3	14-3-3 prote	.	.	.	.
Manes.05G10900(	0.9808349	0.9329	down	no	Triose phosp	pfam03151	TPT	Triose-phos	.	GO:0031969|	GO:0005215|tra	.
Manes.05G10940(	5.8754649	0.01706	up	yes	.	pfam03907	Spo7	Spo7-like pr	.	.	.	.
Manes.05G10970(	1.3539581	0.05634	up	no	Putative met	pfam12697	Abhydrolase_6	Alpha/beta h	.	GO:0009507|	GO:0016787|hy	.
Manes.05G10980(	0.9279119	0.5271	down	no	Asparagine sy	pfam00733	Asn_synthase	Asparagine	GO:0006529|	.	GO:0004066|as	.
Manes.05G11000(	2.3148808	3.37E-09	up	yes	Ninja-family	pfam07897	DUF1675	Protein of u	GO:0009737|	GO:0005634|	.	.
Manes.05G11070(	1.206074	0.04227	up	no	PLASMODE	pfam07983	X8	X8 domain.	.	GO:0031225|	GO:0001872|(1	.
Manes.05G11120(	#NAME?	1	down	no	Cytochrome	pfam00067	p450	Cytochrome	.	GO:0016021|	GO:0020037|he	.
Manes.05G11130(	0.420474	5.90E-05	down	yes	Cytochrome	pfam00067	p450	Cytochrome	.	GO:0016021|	GO:0020037|he	.
Manes.05G11150(	2.2067788	0.01337	up	yes	Receptor-like	pfam07714	Pkinase_Tyr	Protein tyro	GO:0009738|	GO:0043680|	GO:0005524|A'	.
Manes.05G11160(	0.520951	7.28E-07	down	no	Receptor-like	pfam12819	Malectin_like	Carbohydra	GO:0009738|	GO:0043680|	GO:0005524|A'	.
Manes.05G11210(	2.3170128	1.27E-05	up	yes	.	pfam04633	Herpes_BMRF2	Herpesvirus	.	.	.	.
Manes.05G11250(	0.6114874	4.71E-05	down	no	Probable 6-pl	pfam01182	Glucosamine_iso	Glucosamin	GO:0005975|	GO:0005829|	GO:0017057|6-	K01057
Manes.05G11290(	2.6963165	5.01E-05	up	yes	.	pfam07230	Peptidase_S80	Bacteriopha	.	.	.	.
Manes.05G11320(	84.813357	#######	up	yes	Calcium-bind	pfam13833	EF-hand_8	EF-hand do	GO:0010193|	GO:0005737|	GO:0005509|ca	K13448
Manes.05G11380(	2.4362955	4.09E-24	up	yes	UDP-galacto	pfam03151	TPT	Triose-phos	GO:0015783|	GO:0005794|	GO:0015297|an	.
Manes.05G11390(	1.5189781	2.75E-06	up	no	.	pfam14308	DnaJ-X	X-domain o	.	.	.	.
Manes.05G11410(	3.1648154	0.00025	up	yes	.	pfam03028	Dynein_heavy	Dynein heav	.	.	.	.
Manes.05G11420(	1.2578869	0.01437	up	no	Putative met	pfam12697	Abhydrolase_6	Alpha/beta h	.	GO:0009507|	GO:0016787|hy	.
Manes.05G11430(	1.2826705	0.00601	up	no	Protein AIG1	pfam04548	AIG1	AIG1 family	GO:0009617|	.	GO:0005525|G'	.
Manes.05G11440(	3.9426341	0.00244	up	yes	Transcription	pfam00249	Myb_DNA-binding	Myb-like DI	GO:0006952|	GO:0005634|	GO:0003677|DI	K09422
Manes.05G11450(	0.3815566	0.00077	down	yes	.	pfam13398	Peptidase_M50B	Peptidase M	.	.	.	.
Manes.05G11460(	1.9863335	2.51E-08	up	no	Cysteine-rich	pfam00069	Pkinase	Protein kina	GO:0009755|	GO:0016021|	GO:0005524|A'	.
Manes.05G11470(	1.1726838	0.0824	up	no	AMSH-like u	pfam01398	JAB	JAB1/Mov3	GO:0071108|	GO:0005737|	GO:0046872|me	K11866
Manes.05G11480(	1.5323115	0.00247	up	no	.	pfam02029	Caldesmon	Caldesmon.	.	.	.	.
Manes.05G11490(	0.4162982	2.46E-05	down	yes	.	pfam07797	DUF1639	Protein of u	.	.	.	.
Manes.05G11530(	Inf	0.00405	up	yes	.	pfam12822	DUF3816	Protein of u	.	.	.	.
Manes.05G11590(	1.0527054	0.58185	up	no	Serine/threon	pfam00069	Pkinase	Protein kina	GO:0071560|	GO:0005798|	GO:0005524|A'	K08856
Manes.05G11600(	1.7499354	3.86E-10	up	no	Uridine-cytid	pfam00485	PRK	Phosphoribu	GO:0044211|	.	GO:0005524|A'	.
Manes.05G11630(	0.5647671	2.30E-06	down	no	Scopoletin gl	pfam00201	UDPGT	UDP-glucor	.	.	GO:0042802|id	.
Manes.05G11690(	0.0779461	0.03568	down	no	.	pfam04656	Pox_E6	Pox virus E6	.	.	.	.
Manes.05G11720(	3.8837407	2.94E-12	up	yes	Putative phos	pfam13246	Hydrolase_like2	Putative hyc	.	GO:0016021|	GO:0005524|A'	K01530
Manes.05G11730(	0.8145269	0.05386	down	no	Retrovirus-re	pfam01805	Surp	Surp modul	GO:0015074|	.	GO:0004190|as	.
Manes.05G11740(	0.7683658	0.01492	down	no	Protein Dr1 h	pfam00808	CBFD_NFYB_HM	Histone-like	GO:0006355|	GO:0005634|	GO:0043565|se	.
Manes.05G11760(	0.2186962	6.29E-05	down	yes	Protein MIZU	pfam04759	DUF617	Protein of u	GO:0009658|	GO:0032541|	.	.
Manes.05G11780(	0.5985024	2.95E-07	down	no	Peptidyl-prol	pfam00160	Pro_isomerase	Cyclophilin	GO:0006457|	GO:0009507|	GO:0042277|pe	K03768
Manes.05G11790(	0.1075793	5.68E-14	down	yes	.	pfam00582	Usp	Universal st	.	.	.	.
Manes.05G11810(	0.9544027	0.65835	down	no	Zinc finger B	pfam05699	Dimer_Tnp_hAT	hAT family	GO:0009791|	GO:0005829|	GO:0003677|DI	.
Manes.05G11840(	0.2492623	0.00826	down	yes	Oleosin 1	pfam01277	Oleosin	Oleosin.	.	GO:0016021|	.	.
Manes.05G11850(	2.8414333	6.07E-07	up	yes	.	pfam14808	TMEM164	TMEM164	.	.	.	.
Manes.05G11890(	0.8337567	0.08446	down	no	.	pfam13041	PPR_2	PPR repeat	.	.	.	.
Manes.05G11930(	0.2705478	0.00295	down	yes	Transcription	pfam03634	TCP	TCP family	GO:2000032|	GO:0005634|	GO:0003677|DI	.
Manes.05G11970(	1.0453692	0.62198	up	no	Nuclear pore	pfam11894	DUF3414	Protein of u	GO:0051028|	GO:0009507|	.	K14310
Manes.05G12050(	Inf	0.1623	up	no	.	pfam04113	Gpi16	Gpi16 subu	.	.	.	.
Manes.05G12090(	7.1872814	7.60E-06	up	yes	UDP-glycosy	pfam00201	UDPGT	UDP-glucor	GO:0071475|	GO:0043231|	GO:0052638|in	.
Manes.05G12130(	0.9771466	0.81666	down	no	Serine/threon	pfam07714	Pkinase_Tyr	Protein tyro	GO:0071281|	GO:0005789|	GO:0005524|A'	K14510
Manes.05G12140(	Inf	0.02562	up	yes	.	pfam04484	DUF566	Family of ur	.	.	.	.
Manes.05G12160(	Inf	0.35135	up	no	2-alkenal red	pfam00107	ADH_zinc_N	Zinc-binding	.	.	GO:0047655|all	.
Manes.05G12170(	Inf	0.08088	up	no	2-alkenal red	pfam00107	ADH_zinc_N	Zinc-binding	.	.	GO:0047655|all	.
Manes.05G12190(	5.1566885	0.25011	up	no	Probably inac	pfam07714	Pkinase_Tyr	Protein tyro	GO:0042742|	GO:0016021|	GO:0005524|A'	.
Manes.05G12220(	NA	NA	--	no	Polygalacturo	pfam00295	Glyco_hydro_28	Glycosyl hy	GO:0005975|	GO:0005618|	GO:0004650|pc	.
Manes.05G12230(	Inf	0.9009	up	no	Polygalacturo	pfam00295	Glyco_hydro_28	Glycosyl hy	GO:0005975|	GO:0005618|	GO:0004650|pc	.
Manes.05G12250(	Inf	0.9009	up	no	Polygalacturo	pfam00295	Glyco_hydro_28	Glycosyl hy	GO:0005975|	GO:0005618|	GO:0004650|pc	.
Manes.05G12340(	0.9336309	0.46833	down	no	RNA-binding	pfam00076	RRM_1	RNA recog	GO:0030154|	GO:0005737|	GO:0003730|m	.
Manes.05G12390(	1.3252817	0.00293	up	no	Bromodomai	pfam00439	Bromodomain	Bromodoma	GO:0043966|	GO:0030425|	GO:0042393|his	.
Manes.05G12420(	0.5057783	0.06078	down	no	12-oxophyto	pfam00724	Oxidored_FMN	NADH:flavi	GO:0031408|	GO:0005737|	GO:0016629|12	K05894
Manes.05G12530(	1.2196848	0.0377	up	no	.	pfam05508	Ran-binding	RanGTP-bir	.	.	.	.
Manes.05G12550(	0.5566577	2.95E-06	down	no	Protein S-acy	pfam01529	zf-DHHC	DHHC palm	.	GO:0016021|	GO:0019706|pr	K18932
Manes.05G12560(	15.499232	2.50E-08	up	yes	MLO-like pro	pfam03094	Mlo	Mlo family.	GO:0006952|	GO:0016021|	.	K08472
Manes.05G12590(	0.2739346	2.12E-14	down	yes	Outer envelo	pfam06835	LptC	Lipopolysac	GO:1903959|	GO:0034426|	GO:0015288|pc	.
Manes.05G12610(	0.4335509	1.84E-11	down	yes	.	pfam02547	Queuosine_synth	Queuosine b	.	.	.	.
Manes.05G12630(	0.6773661	0.01132	down	no	.	pfam14620	YPEB	YpeB sporu	.	.	.	.
Manes.05G12650(	0.7314037	0.00228	down	no	.	pfam10358	NT-C2	N-terminal (	.	.	.	.
Manes.05G12720(	0.3915116	0.0017	down	yes	Tubulin-foldi	pfam03125	Sre	C. elegans S	GO:0009793|	GO:0005829|	GO:0048487|be	.
Manes.05G12730(	0.2178052	0.00037	down	yes	.	pfam08449	UAA	UAA transp	.	.	.	.
Manes.05G12790(	2.4425214	1.84E-20	up	yes	RNA-binding	pfam00076	RRM_1	RNA recog	GO:0030154|	GO:0005829|	GO:0000166|nu	.
Manes.05G12840(	0.665581	0.0014	down	no	.	pfam12274	DUF3615	Protein of u	.	.	.	.
Manes.05G12850(	0.8282487	0.18726	down	no	.	pfam12274	DUF3615	Protein of u	.	.	.	.
Manes.05G12890(	#NAME?	0.09698	down	no	.	pfam08454	RIH_assoc	RyR and IP3	.	.	.	.
Manes.05G12910(	0.6406965	0.04606	down	no	.	pfam05875	Ceramidase	Ceramidase.	.	.	.	.
Manes.05G12920(	0.5656986	1.09E-08	down	no	AT-rich inter	pfam01388	ARID	ARID/BRIC	GO:0006355|	GO:0005634|	GO:0003677|DI	.
Manes.05G12940(	0.5599813	2.17E-05	down	no	Protein FAR1	pfam03101	FAR1	FAR1 DNA	GO:0006355|	GO:0005634|	GO:0008270|zir	.
Manes.05G12950(	1.03461	0.69098	up	no	Pyrophospha	pfam00365	PFK	Phosphofruc	GO:0006096|	GO:0005737|	GO:0003872|6-	.
Manes.05G12990(	1.6530669	2.36E-08	up	no	.	pfam13514	AAA_27	AAA domai	.	.	.	.
Manes.05G13040(	3.2354162	1.25E-09	up	yes	.	pfam04640	PLATZ	PLATZ tran	.	.	.	.
Manes.05G13060(	0.2780493	7.28E-06	down	yes	.	pfam01755	Glyco_transf_25	Glycosyltrar	.	.	.	.
Manes.05G13070(	1.4336967	0.00077	up	no	Catalase isoz	pfam00199	Catalase	Catalase.	GO:0042744|	GO:0009514|	GO:0004096|ca	.
Manes.05G13100(	0.8463118	0.84166	down	no	Probable seri	pfam00069	Pkinase	Protein kina	GO:0009755|	GO:0005886|	GO:0005524|A'	.
Manes.05G13110(	0.6168499	1.08E-05	down	no	Serine/threon	pfam00069	Pkinase	Protein kina	GO:0009742|	GO:0005886|	GO:0005524|A'	.
Manes.05G13160(	1.3722263	0.00267	up	no	60S ribosoma	pfam00861	Ribosomal_L18p	Ribosomal I	GO:0006412|	GO:0005840|	GO:0008097|5S	K02932
Manes.05G13190(	0.4007612	9.73E-09	down	yes	.	pfam00836	Stathmin	Stathmin far	.	.	.	.
Manes.05G13200(	0.9318355	0.59166	down	no	.	pfam13344	Hydrolase_6	Haloacid de	.	.	.	.
Manes.05G13210(	1.5421361	0.00027	up	no	Mitogen-acti	pfam00069	Pkinase	Protein kina	.	GO:0005622|	GO:0005524|A'	.

Manes.05G13230(	5.0688121	2.19E-72	up	yes	Probable seri	pfam07714	Pkinase_Tyr	Protein tyro	GO:0071215|(	GO:0005886|	GO:0005524|A'	.
Manes.05G13240(	Inf	0.08849	up	no	Probable seri	pfam14380	WAK_assoc	Wall-associa	GO:0071215|(	GO:0005886|	GO:0005524|A'	.
Manes.05G13300(	1.2156651	0.0422	up	no	.	pfam10213	MRP-S28	Mitochondri	.	.	.	.
Manes.05G13350(	0.527728	0.00283	down	no	Cyclin-B2-4	pfam00134	Cyclin_N	Cyclin, N-te	GO:0007049|	GO:0005634|	.	K05868
Manes.05G13380(	0.9338318	0.48293	down	no	1,4-alpha-glu	pfam02922	CBM_48	Carbohydrat	GO:0005978|	GO:0009501|	GO:0003844|1,·	.
Manes.05G13420(	3.1352403	1.07E-05	up	yes	Probable flav	pfam00743	FMO-like	Flavin-bindi	GO:0071456|	.	GO:0050660|fla	K00485
Manes.05G13430(	Inf	0.35135	up	no	Vinorine synt	pfam02458	Transferase	Transferase	GO:0009820|	.	GO:0050636|vi	.
Manes.05G13440(	1.6188592	1.05E-05	up	no	Carboxypepti	pfam00246	Peptidase_M14	Zinc carbox	GO:0006508|	GO:0005615|	GO:0004181|m	K08782
Manes.05G13490(	1.6419841	0.08144	up	no	.	pfam14650	FAM75	FAM75 fam	.	.	.	.
Manes.05G13510(	2.6694122	0.00398	up	yes	.	pfam01925	TauE	Sulfite expo	.	.	.	.
Manes.05G13620(	0.4473185	1.13E-08	down	yes	Indole-3-acet	pfam02519	Auxin_inducible	Auxin respo	GO:0009733|	.	.	.
Manes.05G13650(	2.8330322	0.00296	up	yes	.	pfam14009	DUF4228	Domain of u	.	.	.	.
Manes.05G13670(	0.4387746	2.60E-06	down	yes	Ankyrin repe	pfam13962	PGG	Domain of u	.	GO:0005783|	.	.
Manes.05G13700(	3.9098118	6.86E-19	up	yes	Protein FAM	pfam05811	DUF842	Eukaryotic p	.	GO:0005737|	.	.
Manes.05G13710(	1.8740576	3.84E-05	up	no	Uncharacteri	pfam00149	Metallophos	Calcineurin-	.	GO:0005829|	GO:0016787|hy	.
Manes.05G13760(	0.793213	0.16087	down	no	Zinc finger pı	pfam06203	CCT	CCT motif.	GO:0006355|	GO:0016020|	GO:0003700|tra	.
Manes.05G13780(	5.5865813	1.12E-66	up	yes	.	pfam12517	DUF3720	Protein of u	.	.	.	.
Manes.05G13820(	0.9146722	0.48093	down	no	Aluminum-ac	pfam11744	ALMT	Aluminium a	GO:0098656|	GO:0016021|	GO:0005253|an	.
Manes.05G13840(	0.9287956	0.46265	down	no	Serine/threon	pfam13646	HEAT_2	HEAT repea	GO:0042325|	GO:0005829|	GO:0008601|pr	.
Manes.05G13850(	1.3781049	0.00148	up	no	Aspartic prot	pfam14543	TAXi_N	Xylanase inl	.	.	GO:0004190|as	.
Manes.05G13890(	1.0042507	0.95773	up	no	Lipase memb	pfam04083	Abhydro_lipase	Partial alpha	GO:0016042|	GO:0005576|	GO:0016788|hy	K19406
Manes.05G13910(	8.1074539	7.59E-17	up	yes	.	pfam08137	DVL	DVL family	.	.	.	.
Manes.05G13920(	0.2756856	8.65E-31	down	yes	Transcription	pfam00249	Myb_DNA-binding	Myb-like DI	GO:0048262|	GO:0005634|	GO:0003677|D	.
Manes.05G13930(	0.6712331	0.00047	down	no	Protein DEN	pfam02141	DENN	DENN (AE	GO:2000049|	GO:0005737|	GO:0017112|Ra	.
Manes.05G13940(	NA	NA	--	no	Cytochrome	pfam00067	p450	Cytochrome	GO:0048830|	GO:0005783|	GO:0020037|he	K11818
Manes.05G13950(	1.2834582	1	up	no	Cytochrome	pfam00067	p450	Cytochrome	GO:0048830|	GO:0005783|	GO:0020037|he	K11818
Manes.05G13990(	Inf	0.9009	up	no	.	pfam03372	Exo_endo_phos	Endonuclea	.	.	.	.
Manes.05G14040(	1.8728505	2.66E-05	up	no	Dehydrin ER	pfam00257	Dehydrin	Dehydrin.	GO:0009631|	GO:0005737|	GO:0003779|ac	.
Manes.05G14060(	0.3943341	0.00033	down	yes	Neutral ceram	pfam04734	Ceramidase_alk	Neutral/alka	GO:0006665|	GO:0005576|	GO:0017040|ce	K12349
Manes.05G14090(	1.2379956	0.10523	up	no	L-ascorbate c	pfam00394	Cu-oxidase	Multicopper	.	GO:0005576|	GO:0005507|co	.
Manes.05G14110(	0.4581446	3.93E-07	down	yes	Enoyl-CoA h	pfam01575	MaoC_dehydratas	MaoC like c	GO:0033542|	GO:0005777|	GO:0080023|3F	.
Manes.05G14120(	2.380747	1.19E-08	up	yes	Prolyl endop	pfam02897	Peptidase_S9_N	Prolyl oligo	GO:0019538|	GO:0005737|	GO:0004252|se	K01322
Manes.05G14130(	0.4613388	2.43E-05	down	yes	.	pfam04127	DFP	DNA / pant	.	.	.	.
Manes.05G14140(	1.1246084	0.31607	up	no	Probable alpł	pfam07821	Alpha-amyl_C2	Alpha-amyla	GO:0005975|	GO:0005829|	GO:0004556|al	K01176
Manes.05G14150(	4.901276	3.42E-17	up	yes	.	pfam13716	CRAL_TRIO_2	Divergent C	.	.	.	.
Manes.05G14160(	0.2732499	1.44E-05	down	yes	Pentatricoper	pfam14432	DYW_deaminase	DYW family	.	GO:0005739|	.	.
Manes.05G14170(	0.243784	2.81E-07	down	yes	Mechanosens	pfam00924	MS_channel	Mechanoser	GO:0006811|	GO:0016021|	.	.
Manes.05G14220(	0.6869238	0.05175	down	no	.	pfam07690	MFS_1	Major Facili	.	.	.	.
Manes.05G14230(	1.8625957	8.60E-12	up	no	24-methylene	pfam08241	Methyltransf_11	Methyltrans	GO:0032259|	GO:0005783|	GO:0030797|24	K08242
Manes.05G14270(	Inf	0.9009	up	no	.	pfam12756	zf-C2H2_2	C2H2 type z	.	.	.	.
Manes.05G14300(	1.5423177	1.15E-06	up	no	Transportin-1	pfam03810	IBN_N	Importin-be	GO:0006886|	GO:0005737|	.	.
Manes.05G14310(	0.6762673	0.00478	down	no	Probable prol	pfam03171	2OG-FeII_Oxy	2OG-Fe(II)	.	GO:0005788|	GO:0005506|irc	K00472
Manes.05G14320(	1.2578822	0.01159	up	no	V-type proto	pfam00006	ATP-synt_ab	ATP syntha	GO:0015991|	GO:0033180|	GO:0005524|A'	.
Manes.05G14340(	1.2716061	0.01394	up	no	Probable nuc	pfam13905	Thioredoxin_8	Thioredoxin	.	.	GO:0047134|pr	K17609
Manes.05G14360(	1.1502608	0.1268	up	no	.	pfam13462	Thioredoxin_4	Thioredoxin	.	.	.	.
Manes.05G14370(	0.8633608	0.31171	down	no	Ribonuclease	pfam01918	Alba	Alba. Alba i	.	GO:0005634|	GO:0044822|pc	K14525
Manes.05G14400(	0.8356149	1	down	no	UPF0496 prc	pfam05055	DUF677	Protein of u	.	GO:0016021|	.	.
Manes.05G14410(	22.288262	6.15E-15	up	yes	Aquaporin PI	pfam00230	MIP	Major intrin	GO:0009992|	GO:0046658|	GO:0015254|gl	K09872
Manes.05G14430(	0.4686234	2.55E-08	down	yes	La-related pr	pfam05383	La	La domain.	GO:0009738|	GO:0005737|	GO:0003723|RI	K18757
Manes.05G14440(	0.5285921	0.05735	down	no	Protein FD	pfam00170	bZIP_1	bZIP transc	GO:2000028|	GO:0005634|	GO:0043565|se	.
Manes.05G14450(	1.3664168	0.00073	up	no	SKP1-like pr	pfam01466	Skp1	Skp1 family	GO:0009734|	GO:0005634|	.	K03094
Manes.05G14480(	0.1103277	0.00864	down	yes	.	pfam05514	HR_lesion	HR-like lesi	.	.	.	.
Manes.05G14500(	0.35489	0.01393	down	yes	Probable BO	pfam13920	zf-C3HC4_3	Zinc finger,	GO:0006952|	GO:0005634|	GO:0016874|lig	K19042
Manes.05G14520(	0.5054995	0.00042	down	no	ABC transpo	pfam00005	ABC_tran	ABC transp	GO:0010541|	GO:0016021|	GO:0005524|A'	K05658
Manes.05G14530(	0.5357464	0.00027	down	no	.	pfam07420	DUF1509	Protein of u	.	.	.	.
Manes.05G14540(	0.8164258	0.03693	down	no	Synaptotagm	pfam00168	C2	C2 domain.	GO:0009306|	GO:0005783|	GO:0046872|m	.
Manes.05G14640(	11.20237	1.59E-46	up	yes	Chaperone pı	pfam00226	DnaJ	DnaJ domai	.	GO:0009507|	.	.
Manes.05G14650(	2.3648612	2.19E-22	up	yes	.	pfam05003	DUF668	Protein of u	.	.	.	.
Manes.05G14660(	1.0653545	0.4805	up	no	Peptidyl-prol	pfam00160	Pro_isomerase	Cyclophilin	GO:0006457|	GO:0005737|	GO:0042277|pe	.
Manes.05G14700(	6.1826765	7.18E-74	up	yes	Tubulin beta-	pfam03953	Tubulin_C	Tubulin C-t	GO:0007017|	GO:0005874|	GO:0005525|G'	K07375
Manes.05G14710(	13.630199	8.53E-20	up	yes	.	pfam06319	DUF1052	Protein of u	.	.	.	.
Manes.05G14740(	2.6653791	5.52E-12	up	yes	.	pfam01576	Myosin_tail_1	Myosin tail.	.	.	.	.
Manes.05G14770(	16.378079	0.00026	up	yes	Snakin-2	pfam02704	GASA	Gibberellin	GO:0006952|	GO:0005618|	.	.
Manes.05G14840(	1.3525656	0.01073	up	no	tRNA (adeni	pfam04189	Gcd10p	Gcd10p fam	GO:0030488|	GO:0005634|	.	K03256
Manes.05G14850(	1.417004	0.00033	up	no	.	pfam03134	TB2_DP1_HVA2	TB2/DP1, F	.	.	.	.
Manes.05G14860(	0.5210264	6.92E-08	down	no	Probable dipl	pfam00590	TP_methylase	Tetrapyrrol	GO:0017183|	.	GO:0004164|di	K00586
Manes.05G14890(	0.518911	0.16792	down	no	Cytokinin rib	pfam03641	Lysine_decarbox	Possible lysi	GO:0009691|	GO:0005829|	GO:0016799|hy	.
Manes.05G14910(	1.8457773	3.06E-11	up	no	5'-3' exoribor	pfam03159	XRN_N	XRN 5'-3' e	GO:0010587|	GO:0005634|	GO:0004534|5'-	K12619
Manes.05G14920(	0.4802068	1.20E-09	down	yes	ATP sulfuryla	pfam01747	ATP-sulfurylase	ATP-sulfury	GO:0009970|	GO:0009507|	GO:0004020|ad	K13811
Manes.05G14940(	0.4764165	2.86E-05	down	yes	Auxin-induce	pfam02519	Auxin_inducible	Auxin respo	GO:0009734|	.	.	.
Manes.05G14960(	1.3766915	0.00069	up	no	Filament-like	pfam05911	DUF869	Plant protei	.	GO:0005886|	.	.
Manes.05G15120(	0.4701668	1.37E-11	down	yes	Protein RCC	pfam00415	RCC1	Regulator o	GO:0090630|	GO:0034506|	GO:0008017|mi	.
Manes.05G15160(	0.3214612	4.41E-07	down	yes	Galactose ox	pfam07250	Glyoxal_oxid_N	Glyoxal oxi	.	GO:0005576|	GO:0045480|ga	.
Manes.05G15180(	1.0990173	0.40845	up	no	V-type proto	pfam00137	ATP-synt_C	ATP syntha	GO:0015991|	GO:0016021|	GO:0015078|hy	K02155
Manes.05G15210(	13.702923	3.04E-14	up	yes	Probable dise	pfam13855	LRR_8	Leucine rich	GO:0006952|	.	GO:0043531|Al	.
Manes.05G15220(	2.9235319	1.48E-08	up	yes	Probable dise	pfam12799	LRR_4	Leucine Ric	GO:0006952|	.	GO:0043531|Al	.
Manes.05G15230(	0.6011451	4.47E-06	down	no	Uncharacteri	pfam13414	TPR_11	TPR repeat.	.	GO:0005768|	GO:0005509|ca	.
Manes.05G15260(	1.807874	7.53E-07	up	no	DNA-binding	pfam00098	zf-CCHC	Zinc knuckl	.	GO:0005634|	GO:0003677|D	.
Manes.05G15330(	0.8759268	0.26509	down	no	Transcription	pfam02270	TFIIF_beta	Transcriptio	GO:0032508|	GO:0044732|	GO:0005524|A'	K03139
Manes.05G15370(	#NAME?	1	down	no	Dehydration-	pfam00847	AP2	AP2 domair	GO:0010200|	GO:0005634|	GO:0003677|D	.
Manes.05G15380(	2.184236	1.84E-18	up	yes	Thioredoxin	pfam00085	Thioredoxin	Thioredoxin	GO:0045454|	GO:0005737|	GO:0015035|pr	K03671
Manes.05G15420(	0.0264701	7.14E-31	down	yes	Cytokinin del	pfam09265	Cytokin-bind	Cytokinin d	GO:0009823|	GO:0005576|	GO:0019139|cy	.
Manes.05G15440(	Inf	9.74E-17	up	yes	.	pfam01735	PLA2_B	Lysophosph	.	.	.	.
Manes.05G15450(	Inf	1.54E-20	up	yes	.	pfam14111	DUF4283	Domain of u	.	.	.	.
Manes.05G15470(	0.7513383	0.00862	down	no	GTPase HflX	pfam01926	MMR_HSR1	50S ribosor	GO:0009408|	GO:0005737|	GO:0005524|A'	K03665
Manes.05G15480(	5.8477553	9.84E-05	up	yes	Peroxidase 9	pfam00141	peroxidase	Peroxidase.	GO:0042744|	GO:0005576|	GO:0020037|he	K00430
Manes.05G15490(	0.5464734	2.84E-06	down	no	Trehalose syr	pfam00534	Glycos_transf_1	Glycosyl tra	GO:0009058|	.	GO:0016757|tra	K13057
Manes.05G15510(	0.3745575	3.89E-09	down	yes	.	pfam01370	Epimerase	NAD depen	.	.	.	.

Manes.05G15520(	2.2115942	1.09E-15	up	yes	.	pfam13639	zf-RING_2	Ring finger	.	.	.	.
Manes.05G15530(	0.7038583	0.00129	down	no	Mitogen-acti	pfam00069	Pkinase	Protein kina	.	GO:0005622|	GO:0005524|A	.
Manes.05G15540(	0.3584882	0.00045	down	yes	Omega-hydrc	pfam02458	Transferase	Transferase	GO:0052325|	.	GO:0050734|hy	K15400
Manes.05G15580(	1.8570766	2.98E-07	up	no	Bifunctional	pfam02577	DNase-RNase	Bifunctional	.	GO:0005634|	GO:0004518|nu	.
Manes.05G15590(	1.8330856	5.79E-10	up	no	Phosphatidyli	pfam00650	CRAL_TRIO	CRAL/TRIC	GO:0015031|	GO:0000139|	GO:0005215|tra	.
Manes.05G15620(	0.7439462	0.04671	down	no	.	pfam00403	HMA	Heavy-meta	.	.	.	.
Manes.05G15660(	4.6939346	2.33E-30	up	yes	.	pfam11081	DUF2890	Protein of u	.	.	.	.
Manes.05G15690(	0.9351862	0.95858	down	no	Mannan endo	pfam00150	Cellulase	Cellulase (g	GO:0005975|	GO:0071944|	GO:0016985|ma	K19355
Manes.05G15700(	0.5613207	0.001	down	no	Bifunctional	pfam01370	Epimerase	NAD depen	GO:0009813|	.	GO:0050662|co	K13082
Manes.05G15710(	0.8682818	0.35239	down	no	Bifunctional	pfam01370	Epimerase	NAD depen	GO:0009813|	.	GO:0050662|co	.
Manes.05G15770(	1.4531529	0.00144	up	no	Protein RER	pfam03248	Rer1	Rer1 family.	GO:0006890|	GO:0005801|	.	.
Manes.05G15790(	1.0243732	0.79741	up	no	.	pfam01167	Tub	Tub family.	.	.	.	.
Manes.05G15810(	5.171937	6.18E-06	up	yes	.	pfam07733	DNA_pol3_alpha	Bacterial DI	.	.	.	.
Manes.05G15820(	1.0395475	0.65054	up	no	Protein NUC	pfam13041	PPR_2	PPR repeat	GO:0000741|	GO:0005739|	.	.
Manes.05G15860(	28.077493	1.47E-08	up	yes	Long-chain-f	pfam00501	AMP-binding	AMP-bindin	GO:0071766|	.	GO:0016874|lig	K12426
Manes.05G15920(	Inf	0.08543	up	no	Protein RAD	pfam00249	Myb_DNA-binding	Myb-like DI	GO:0006351|	GO:0005737|	GO:0003677|D	.
Manes.05G15940(	1.2436385	0.01721	up	no	Cytosolic pur	pfam05761	5_nucleotid	5' nucleotida	GO:0016311|	GO:0045335|	GO:0008253|5'-	K01081
Manes.05G15950(	0.7962467	0.03996	down	no	.	pfam04072	LCM	Leucine carl	.	.	.	.
Manes.05G15960(	1.2497059	0.01757	up	no	DUF21 doma	pfam01595	DUF21	Domain of u	.	GO:0016021|	.	K16302
Manes.05G15980(	0.7220734	0.00976	down	no	Uncharacteri	pfam05867	DUF851	Protein of u	.	GO:0016021|	.	.
Manes.05G15990(	0.7081625	0.00055	down	no	.	pfam00650	CRAL_TRIO	CRAL/TRIC	.	.	.	.
Manes.05G16010(	4.3298857	3.55E-16	up	yes	.	pfam08637	NCA2	ATP syntha	.	.	.	.
Manes.05G16020(	20.34193	5.43E-20	up	yes	.	pfam01148	CTP_transf_1	Cytidylyltra	.	.	.	.
Manes.05G16030(	3.094847	1.95E-18	up	yes	.	pfam11110	Phage_hub_GP28	Baseplate hu	.	.	.	.
Manes.05G16060(	1.280865	0.03642	up	no	Glucan endo-	pfam00332	Glyco_hydro_17	Glycosyl hy	GO:0005975|	GO:0005618|	GO:0042973|glu	.
Manes.05G16080(	1.1237352	0.2354	up	no	Glucose-6-ph	pfam00342	PGI	Phosphoglu	GO:0006094|	GO:0005737|	GO:0004347|glu	.
Manes.05G16110(	0.6550479	0.0001	down	no	.	pfam06708	DUF1195	Protein of u	.	.	.	.
Manes.05G16140(	0.5042112	1.66E-06	down	no	.	pfam05320	Pox_RNA_Pol_19	Poxvirus DI	.	.	.	.
Manes.05G16170(	0.8623327	0.14206	down	no	Uncharacteri	pfam14296	O-ag_pol_Wzy	O-antigen p	.	GO:0005783|	.	.
Manes.05G16200(	1.0851791	0.52023	up	no	.	pfam11779	DUF3317	Protein of u	.	.	.	.
Manes.05G16210(	1.4339822	0.00055	up	no	Zinc finger p	pfam00096	zf-C2H2	Zinc finger,	GO:0006355|	GO:0005634|	GO:0046872|me	.
Manes.05G16220(	0.52289	0.00533	down	no	Cytochrome	pfam00067	p450	Cytochrome	GO:0010268|	GO:0016021|	GO:0020037|he	K15639
Manes.05G16240(	0.5976263	5.89E-05	down	no	Probable prot	pfam13672	PP2C_2	Protein phos	.	.	GO:0046872|me	.
Manes.05G16250(	0.5254788	1.27E-08	down	no	J domain-con	pfam00226	DnaJ	DnaJ domai	GO:0007015|	GO:0005737|	.	.
Manes.05G16280(	0.6681606	0.04503	down	no	Peroxisomal	pfam05648	PEX11	Peroxisomal	GO:0016559|	GO:0005779|	GO:0042802|ide	.
Manes.05G16290(	0.6242478	2.80E-05	down	no	Probable GM	pfam03352	Adenine_glyco	Methyladeni	GO:0006529|	.	GO:0004066|as	K03790
Manes.05G16300(	1.8088615	1.36E-10	up	no	Protein BRA	pfam05687	DUF822	Plant protei	GO:0009742|	GO:0005829|	GO:0003677|D	K14503
Manes.05G16310(	0.5078021	9.50E-09	down	no	Methyltransfe	pfam05063	MT-A70	MT-A70. M	GO:0006139|	.	GO:0008168|me	.
Manes.05G16330(	0.5852389	7.89E-05	down	no	.	pfam13867	SAP30_Sin3_bdg	Sin3 binding	.	.	.	.
Manes.05G16340(	0.1239325	3.99E-12	down	yes	Calcium unip	pfam04678	DUF607	Protein of u	.	GO:0016021|	GO:0005262|ca	.
Manes.05G16350(	1.447068	4.93E-05	up	no	Leishmanolys	pfam01457	Peptidase_M8	Leishmanoly	GO:0007420|	GO:0005737|	GO:0046872|me	K13539
Manes.05G16360(	Inf	9.86E-17	up	yes	.	pfam14244	UBN2_3	gag-polyper	.	.	.	.
Manes.05G16380(	2.9238783	3.51E-07	up	yes	Elongation o	pfam01151	ELO	GNS1/SUR	GO:0071215|	GO:0016021|	GO:0009922|fat	.
Manes.05G16390(	0.3805402	1.84E-10	down	yes	E3 ubiquitin-	pfam13923	zf-C3HC4_2	Zinc finger,	GO:0043161|	GO:0005789|	GO:0016874|lig	.
Manes.05G16420(	1.5935117	0.0001	up	no	Protein DA1	pfam12315	DUF3633	Protein of u	GO:0046621|	.	GO:0043130|ub	.
Manes.05G16430(	#NAME?	1	down	no	Type IV inos	pfam03372	Exo_endo_phos	Endonuclea	GO:0046856|	GO:0005886|	GO:0034485|ph	.
Manes.05G16440(	0.4774064	7.10E-10	down	yes	.	pfam00646	F-box	F-box doma	.	.	.	.
Manes.05G16470(	0.9605494	0.70507	down	no	.	pfam04765	DUF616	Protein of u	.	.	.	.
Manes.05G16480(	Inf	0.1623	up	no	.	pfam06541	DUF1113	Protein of u	.	.	.	.
Manes.05G16530(	4.1131921	2.97E-33	up	yes	.	pfam09704	Cas_Cas5d	CRISPR-ass	.	.	.	.
Manes.05G16550(	4.1223223	1.23E-41	up	yes	.	pfam00146	NADHdh	NADH dehy	.	.	.	.
Manes.05G16560(	3.3408024	4.84E-10	up	yes	.	pfam13261	DUF4052	Protein of u	.	.	.	.
Manes.05G16570(	Inf	6.65E-10	up	yes	Dirigent prot	pfam03018	Dirigent	Dirigent-lik	.	GO:0048046|	.	.
Manes.05G16580(	32.18403	3.86E-06	up	yes	Probable E3	pfam01485	IBR	IBR domain	.	.	GO:0016874|lig	K11968
Manes.05G16600(	0.1393868	3.31E-07	down	yes	.	pfam04507	DUF576	Protein of u	.	.	.	.
Manes.05G16610(	0.3475452	2.97E-08	down	yes	Plastidial lipo	pfam03099	BPL_LplA_LipB	Biotin/lipoa	GO:0009107|	GO:0009507|	GO:0033819|lip	K03801
Manes.05G16620(	0.9516417	0.7059	down	no	Allene oxide	pfam00067	p450	Cytochrome	GO:0031408|	GO:0009507|	GO:0020037|he	.
Manes.05G16640(	0.4940361	0.36112	down	no	Wall-associat	pfam00069	Pkinase	Protein kina	.	GO:0016021|	GO:0005524|A	.
Manes.05G16680(	0.4039184	2.43E-14	down	yes	Cytochrome	pfam00067	p450	Cytochrome	.	GO:0016020|	GO:0020037|he	.
Manes.05G16700(	0.4682116	0.00017	down	yes	Cytochrome	pfam00067	p450	Cytochrome	.	GO:0016020|	GO:0020037|he	.
Manes.05G16710(	1.6456647	1.42E-07	up	no	Probably inac	pfam00069	Pkinase	Protein kina	.	GO:0016021|	GO:0005524|A	.
Manes.05G16720(	0.197616	0.00341	down	yes	Cytochrome	pfam00067	p450	Cytochrome	.	GO:0016020|	GO:0020037|he	.
Manes.05G16730(	0.8305883	0.65356	down	no	.	pfam11214	Med2	Mediator co	.	.	.	.
Manes.05G16770(	43.243665	1.09E-08	up	yes	Cytochrome	pfam00067	p450	Cytochrome	.	GO:0016020|	GO:0020037|he	.
Manes.05G16780(	0.9629215	0.76505	down	no	Transcription	pfam04433	SWIRM	SWIRM dor	GO:0016573|	GO:0005634|	GO:0003677|D	K11314
Manes.05G16830(	0.06168	0.01132	down	yes	Cytochrome	pfam00067	p450	Cytochrome	GO:0071281|	GO:0016021|	GO:0020037|he	K00517
Manes.05G16840(	0.3899238	0.01122	down	yes	Cytochrome	pfam00067	p450	Cytochrome	GO:0071281|	GO:0016021|	GO:0020037|he	K00517
Manes.05G16850(	0.3364773	2.27E-18	down	yes	Cytochrome	pfam00067	p450	Cytochrome	GO:0071281|	GO:0016021|	GO:0020037|he	K00517
Manes.05G16890(	0.6500815	1.91E-05	down	no	B3 domain-co	pfam02362	B3	B3 DNA bir	GO:0006355|	GO:0005634|	GO:0003677|D	.
Manes.05G16900(	0.9286966	0.56268	down	no	Ent-kaurene	pfam00067	p450	Cytochrome	GO:0010241|	GO:0009707|	GO:0052617|en	K04122
Manes.05G16920(	1.4908442	0.00212	up	no	Probable thyl	pfam10502	Peptidase_S26	Signal pepti	.	GO:0009535|	GO:0008236|se	K03100
Manes.05G16930(	0.8432741	0.17665	down	no	.	pfam13701	DDE_Tnp_1_4	Transposase	.	.	.	.
Manes.05G16960(	5.7437613	2.77E-42	up	yes	Probable dise	pfam00931	NB-ARC	NB-ARC dc	GO:0042742|	GO:0048046|	GO:0043531|A	.
Manes.05G17000(	0.6574899	0.00187	down	no	CST complex	pfam15491	CTC1_2	CST, telome	GO:0000723|	GO:0000781|	GO:0003677|D	.
Manes.05G17010(	#NAME?	5.00E-07	down	yes	.	pfam10214	Rrn6	RNA polym	.	.	.	.
Manes.05G17030(	Inf	0.35135	up	no	.	pfam14111	DUF4283	Domain of u	.	.	.	.
Manes.05G17040(	0.6482275	0.00664	down	no	Protein MIZU	pfam04759	DUF617	Protein of u	GO:0009658|	GO:0032541|	.	.
Manes.05G17050(	13.976611	1.57E-18	up	yes	.	pfam07816	DUF1645	Protein of u	.	.	.	.
Manes.05G17060(	1.3315483	0.00409	up	no	.	pfam04784	DUF547	Protein of u	.	.	.	.
Manes.05G17090(	2.3058765	5.56E-21	up	yes	Polyadenylate	pfam00076	RRM_1	RNA recog	GO:0034605|	GO:0010494|	GO:0000166|nu	.
Manes.05G17100(	1.02286	0.79641	up	no	.	pfam00149	Metallophos	Calcineurin-	.	.	.	.
Manes.05G17140(	28.627597	5.66E-09	up	yes	Patatin-like p	pfam01734	Patatin	Patatin-like	GO:0071456|	GO:0005737|	GO:0016298|lip	.
Manes.05G17200(	0.9490744	0.67001	down	no	.	pfam05296	TAS2R	Mammalian	.	.	.	.
Manes.05G17220(	2.8470132	2.20E-30	up	yes	Probable prot	pfam00481	PP2C	Protein phos	GO:0006470|	GO:0005829|	GO:0046872|me	.
Manes.05G17240(	1.5606176	1.28E-06	up	no	Phosphatidat	pfam08235	LNS2	LNS2 (Lipi	GO:0019375|	GO:0048046|	GO:0008195|ph	.
Manes.05G17250(	0.8232303	0.04751	down	no	Phosphatidat	pfam08235	LNS2	LNS2 (Lipi	GO:0019375|	GO:0048046|	GO:0008195|ph	.
Manes.05G17380(	1.0245862	0.77038	up	no	Protein WEA	pfam05701	WEMBL	Weak chlor	GO:0009904|	GO:0005829|	.	.
Manes.05G17400(	1.2822889	0.01817	up	no	Peroxisomal	pfam02036	SCP2	SCP-2 stero	GO:0008203|	GO:0005777|	GO:0033989|3a	K12405

Manes.05G17420(	4.6147204	1.27E-65	up	yes	Serine/threon	pfam00069	Pkinase	Protein kina	GO:0048800|;	GO:0030424|;	GO:0005524|A'	K08790
Manes.05G17450(	1.0509805	0.79365	up	no	ABSCISIC A	pfam00170	bZIP_1	bZIP transcr	GO:0009738|a	GO:0005634|	GO:0043565|se	.
Manes.05G17490(	0.7036659	0.05253	down	no	Heat stress tr	pfam00447	HSF_DNA-bind	HSF-type D	GO:0008356|a	GO:0005737|	GO:0043565|se	K09419
Manes.05G17510(	1.1646751	0.13152	up	no	Probable BO	pfam13920	zf-C3HC4_3	Zinc finger,	GO:0006952|c	GO:0005634|	GO:0016874|lig	K19042
Manes.05G17550(	0.5381853	2.00E-07	down	no	Probable seri	pfam00069	Pkinase	Protein kina	GO:0046777|p	GO:0016021|	GO:0005524|A'	.
Manes.05G17610(	2.4171059	1.72E-18	up	yes	.	pfam01936	NYN	NYN domai	.	.	.	.
Manes.05G17640(	1.8119184	2.34E-10	up	no	Chromosome	pfam00225	Kinesin	Kinesin mot	GO:0008089|a	GO:0005694|	GO:0005524|A'	K10395
Manes.05G17690(	1.1751181	0.21098	up	no	Cysteine prot	pfam00112	Peptidase_C1	Papain famil	GO:0050832|c	GO:0048046|	GO:0004197|cy	.
Manes.05G17700(	0.5123766	2.83E-07	down	no	Transcription	pfam14215	bHLH-MYC_N	bHLH-MYC	GO:0006355|r	GO:0005634|	GO:0003677|D!	.
Manes.05G17710(	0.4872476	1.96E-11	down	yes	Mannose-P-d	pfam04193	PQ-loop	PQ loop rep	GO:0006810|t	GO:0016021|	.	K09660
Manes.05G17720(	0.7182274	0.00116	down	no	F-box proteir	pfam12937	F-box-like	F-box-like.	GO:0016567|p	.	GO:0004842|ub	.
Manes.05G17740(	0.4453518	6.43E-14	down	yes	Cyclin-D3-1	pfam00134	Cyclin_N	Cyclin, N-te	GO:0051301|c	GO:0005634|	.	K14505
Manes.05G17790(	2.7058413	4.92E-19	up	yes	Transcription	pfam00249	Myb_DNA-binding	Myb-like DI	GO:0009738|a	GO:0005634|	GO:0003677|D!	K09422
Manes.05G17810(	0.6573697	0.01245	down	no	Mortality fact	pfam05712	MRG	MRG. This	GO:0008283|c	GO:0035267|	GO:0003682|ch	K11339
Manes.05G17820(	NA	NA	--	no	.	pfam05614	DUF782	Circovirus p	.	.	.	.
Manes.05G17910(	8.8505074	#######	up	yes	Cytochrome	pfam00067	p450	Cytochrome	.	GO:0016021|	GO:0020037|he	.
Manes.05G17930(	0.3523295	0.00132	down	yes	Cytochrome	pfam00067	p450	Cytochrome	GO:0098542|c	GO:0016021|	GO:0020037|he	K00517
Manes.05G17940(	0.6023008	0.00324	down	no	Isoflavone 2'-	pfam00067	p450	Cytochrome	.	GO:0016020|	GO:0020037|he	.
Manes.05G17960(	0.9061448	0.31625	down	no	Metal transpo	pfam01566	Nramp	Natural resi	GO:0006811|i	GO:0016021|	GO:0005215|tra	.
Manes.05G17970(	1.0014517	1	up	no	Aspartic prot	pfam00026	Asp	Eukaryotic a	.	GO:0031225|	GO:0004190|as	.
Manes.05G17980(	1.5365237	0.2294	up	no	Snakin-1	pfam02704	GASA	Gibberellin	GO:0006952|c	GO:0005618|	.	.
Manes.05G18000(	2.0447694	4.00E-09	up	yes	.	pfam04788	DUF620	Protein of u	.	.	.	.
Manes.05G18090(	66.258429	2.08E-44	up	yes	Zinc finger pr	pfam13912	zf-C2H2_6	C2H2-type	GO:0035264|r	GO:0005634|	GO:0046872|m	.
Manes.05G18100(	1.1991603	0.04576	up	no	Adenine phos	pfam00156	Pribosyltran	Phosphoribo	GO:0006168|a	GO:0009507|	GO:0003999|ad	.
Manes.05G18160(	3.1522331	2.31E-38	up	yes	Calcium-tran	pfam00122	E1-E2_ATPase	E1-E2 ATP	.	GO:0009706|	GO:0005524|A'	.
Manes.05G18170(	1.3560014	0.0051	up	no	U-box domai	pfam00514	Arm	Armadillo/b	.	.	GO:0016874|lig	.
Manes.05G18180(	4.6351424	2.18E-17	up	yes	Mitochondria	pfam01459	Porin_3	Eukaryotic p	GO:0006811|i	GO:0005741|	GO:0015288|pc	.
Manes.05G18220(	7.458586	1.62E-15	up	yes	.	pfam04511	DER1	Der1-like fa	.	.	.	.
Manes.05G18270(	2.2791584	2.30E-12	up	yes	.	pfam07600	DUF1564	Protein of u	.	.	.	.
Manes.05G18280(	0.4416489	7.86E-10	down	yes	Lecithin-chol	pfam02450	LCAT	Lecithin:cho	GO:0006629|l	GO:0016021|	GO:0008374|O-	.
Manes.05G18290(	0.6448223	0.002	down	no	Protein PHL	pfam08395	7tm_7	7tm Chemos	.	GO:0016021|	GO:0030246|ca	.
Manes.05G18300(	0.7064699	0.00717	down	no	.	pfam04156	IncA	IncA proteir	.	.	.	.
Manes.05G18380(	0.5545501	0.00444	down	no	.	pfam09897	DUF2124	Uncharacter	.	.	.	.
Manes.05G18400(	0.3094849	4.54E-17	down	yes	AP2-like ethy	pfam00847	AP2	AP2 domair	GO:0009908|f	GO:0005634|	GO:0003677|D!	K09285
Manes.05G18420(	0.989904	0.96008	down	no	Squalene mo	pfam08491	SE	Squalene ep	.	GO:0016021|	GO:0050660|fla	.
Manes.05G18460(	11.000266	1.17E-33	up	yes	Branched-cha	pfam01063	Aminotran_4	Aminotransf	GO:0009097|i	GO:0009507|	GO:0052656|L-	K00826
Manes.05G18470(	11.545959	2.16E-67	up	yes	Branched-cha	pfam01063	Aminotran_4	Aminotransf	GO:0009097|i	GO:0009507|	GO:0052656|L-	K00826
Manes.05G18540(	0.6040098	0.00892	down	no	Endo-1,4-bet	pfam00331	Glyco_hydro_10	Glycosyl hy	GO:0045493|	GO:0005576|	GO:0031176|en	K01181
Manes.05G18550(	0.5645349	0.00146	down	no	.	pfam04116	FA_hydroxylase	Fatty acid h	.	.	.	.
Manes.05G18570(	2.4273098	1.78E-05	up	yes	50S ribosoma	pfam06627	DUF1153	Protein of u	GO:0006412|t	GO:0009507|	GO:0019843|rR	K19035
Manes.05G18610(	0.6265167	0.00191	down	no	Amino acid p	pfam01490	Aa_trans	Transmembr	GO:0003333|a	GO:0016021|	GO:0015172|ac	.
Manes.05G18630(	0.6769319	0.03097	down	no	.	pfam05057	DUF676	Putative seri	.	.	.	.
Manes.05G18640(	0.8082664	0.05543	down	no	Xylulose kina	pfam02782	FGGY_C	FGGY famil	GO:0046835|c	GO:0005737|	GO:0005524|A'	K00854
Manes.05G18660(	0.6285209	0.00741	down	no	Sucrose trans	pfam07690	MFS_1	Major Facili	GO:0005985|s	GO:0005801|	GO:0008515|su	K15378
Manes.05G18680(	0.5943292	1.32E-05	down	no	Probable beta	pfam03360	Glyco_transf_43	Glycosyltrar	GO:0071555|c	GO:0005794|	GO:0015018|ga	.
Manes.05G18720(	0.816595	0.56979	down	no	Uncharacteriz	pfam05351	GMP_PDE_delta	GMP-PDE,	.	GO:0016021|	.	.
Manes.05G18760(	0.8088196	0.03417	down	no	Pentatricoper	pfam13041	PPR_2	PPR repeat	.	GO:0016020|	.	.
Manes.05G18770(	5.7579817	2.44E-34	up	yes	.	pfam11677	DUF3273	Protein of u	.	.	.	.
Manes.05G18900(	0.884269	0.2172	down	no	Alpha-glucan	pfam01326	PPDK_N	Pyruvate ph	GO:0005975|c	GO:0009941|	GO:0050521|al	K08244
Manes.05G18910(	0.8927362	0.49776	down	no	.	pfam04020	DUF360	Membrane p	.	.	.	.
Manes.05G18950(	0.5778206	1.39E-06	down	no	GATA transc	pfam06203	CCT	CCT motif.	GO:0006351|t	GO:0005634|	GO:0043565|se	.
Manes.05G18960(	0.5701863	4.88E-07	down	no	GATA transc	pfam06203	CCT	CCT motif.	GO:0006351|t	GO:0005634|	GO:0043565|se	.
Manes.05G18980(	3.7355883	3.73E-42	up	yes	.	pfam13414	TPR_11	TPR repeat.	.	.	.	.
Manes.05G19030(	1.9594206	4.89E-12	up	no	.	pfam09341	Pcc1	Transcriptio	.	.	.	.
Manes.05G19040(	2.0970966	3.10E-09	up	yes	Putative amir	pfam12697	Abhydrolase_6	Alpha/beta l	GO:0019740|r	.	GO:0016811|hy	K09023
Manes.05G19050(	0.3770853	4.15E-15	down	yes	.	pfam00013	KH_1	KH domain.	.	.	.	.
Manes.05G19060(	0.9984329	0.98334	down	no	Protein LST8	pfam00400	WD40	WD domain	GO:0007190|a	GO:0005737|	.	K08266
Manes.05G19110(	1.5201569	9.47E-05	up	no	CDPK-relate	pfam00069	Pkinase	Protein kina	GO:0046777|p	GO:0005886|	GO:0005524|A'	.
Manes.05G19120(	1.0268958	0.88604	up	no	Auxin-induce	pfam02519	Auxin_inducible	Auxin respo	GO:0009734|a	.	.	.
Manes.05G19190(	0.5425945	1.15E-06	down	no	Ankyrin repe	pfam13962	PGG	Domain of u	GO:0045842|p	GO:0005829|	.	K06694
Manes.05G19220(	0.7131921	0.00281	down	no	WD repeat-co	pfam00400	WD40	WD domain	.	GO:0005829|	.	.
Manes.05G19260(	9.5101776	4.21E-09	up	yes	.	pfam04639	Baculo_E56	Baculoviral	.	.	.	.
Manes.05G19270(	1.2916935	0.00483	up	no	ATPase famil	pfam00004	AAA	ATPase fam	GO:0031122|c	GO:0030054|	GO:0005524|A'	.
Manes.05G19290(	Inf	0.02442	up	yes	.	pfam02207	zf-UBR	Putative zinc	.	.	.	.
Manes.05G19300(	2.2090846	9.15E-17	up	yes	Cytochrome	pfam00173	Cyt-b5	Cytochrome	GO:0055114|c	GO:0005789|	GO:0020037|he	.
Manes.05G19320(	0.9543378	0.86587	down	no	GTPase-activ	pfam00566	RabGAP-TBC	Rab-GTPase	.	GO:0005829|	GO:0005096|G'	.
Manes.05G19350(	#NAME?	0.20083	down	no	.	pfam03125	Sre	C. elegans S	.	.	.	.
Manes.05G19430(	1.1528889	0.1334	up	no	Phosphogluca	pfam01326	PPDK_N	Pyruvate ph	GO:0046835|c	GO:0009507|	GO:0005524|A'	.
Manes.05G19450(	0.8942584	0.30307	down	no	Probable aldo	pfam00248	Aldo_ket_red	Aldo/keto re	.	.	GO:0016491|ox	.
Manes.05G19470(	0.5573794	0.00022	down	no	Fanconi-asso	pfam08774	VRR_NUC	VRR-NUC	GO:0036297|i	.	GO:0016818|hy	K15363
Manes.05G19480(	24.231032	2.75E-12	up	yes	Putative phos	pfam01504	PIP5K	Phosphatidy	GO:0046854|p	.	GO:0016308|1-	K00889
Manes.05G19490(	0.5617932	1.18E-07	down	no	Probably inac	pfam00069	Pkinase	Protein kina	.	GO:0016021|	GO:0005524|A'	.
Manes.05G19510(	1.1600102	0.1486	up	no	Chitinase-like	pfam00182	Glyco_hydro_19	Chitinase cl	GO:0016998|c	GO:0048046|	GO:0030247|pc	.
Manes.05G19520(	31.044558	1.61E-30	up	yes	.	pfam04884	DUF647	Vitamin B6	.	.	.	.
Manes.05G19550(	3.6790477	3.50E-50	up	yes	Uncharacteriz	pfam00400	WD40	WD domain	.	GO:0005829|	.	.
Manes.05G19580(	0.8828204	0.30967	down	no	.	pfam05935	Arylsulfotrans	Arylsulfotra	.	.	.	.
Manes.05G19610(	1.6603562	7.20E-08	up	no	Uncharacteriz	pfam01145	Band_7	SPFH doma	GO:0007006|r	GO:0016020|	.	.
Manes.05G19640(	1.6508143	2.28E-07	up	no	Calmodulin-r	pfam13499	EF-hand_7	EF-hand do	.	.	GO:0005509|ca	.
Manes.05G19700(	0.7764802	0.25547	down	no	.	pfam03455	dDENN	dDENN do	.	.	.	.
Manes.05G19710(	0.9316212	0.61802	down	no	.	pfam04547	Anoctamin	Calcium-act	.	.	.	.
Manes.05G19720(	0.6527762	0.00033	down	no	.	pfam12692	Methyltransf_17	S-adenosyl-	.	.	.	.
Manes.05G19740(	14.602621	2.35E-06	up	yes	.	pfam15290	Syntaphilin	Golgi-locali	.	.	.	.
Manes.05G19780(	3.8631015	2.60E-12	up	yes	.	pfam03492	Methyltransf_7	SAM depen	.	.	.	.
Manes.05G19820(	2.5386876	2.86E-16	up	yes	Transcription	pfam00010	HLH	Helix-loop-l	GO:0006355|r	GO:0005634|	GO:0003677|D!	.
Manes.05G19830(	7.3387563	0.00325	up	yes	Cysteine prot	pfam00031	Cystatin	Cystatin dor	GO:0006952|c	GO:0005576|	GO:0004869|cy	.
Manes.05G19850(	NA	NA	--	no	Cysteine prot	pfam00031	Cystatin	Cystatin dor	.	.	GO:0004869|cy	.
Manes.05G19890(	0.8147144	0.14259	down	no	Pentatricoper	pfam13041	PPR_2	PPR repeat	.	GO:0009507|	.	.
Manes.05G19910(	0.8214859	0.03954	down	no	.	pfam07263	DMP1	Dentin matr	.	.	.	.

Manes.05G19950(	2.1509106	8.77E-06	up	yes	Protein TIC 2	pfam04278	Tic22	Tic22-like fa	GO:0015031|p	GO:0031972|	.	.
Manes.05G19970(	0.2670436	6.32E-15	down	yes	Crocetin gluc	pfam00201	UDPGT	UDP-glucor	.	GO:0009507|	GO:0016758|tra	.
Manes.05G20020(	1.0390316	0.72864	up	no	Tubulin beta-	pfam03953	Tubulin_C	Tubulin C-te	GO:0007017|r	GO:0005737|	GO:0005525|G	K07375
Manes.05G20030(	1.211221	0.05069	up	no	.	pfam01576	Myosin_tail_1	Myosin tail.	.	.	.	.
Manes.05G20050(	0.6511885	0.00694	down	no	Transcription	pfam01496	V_ATPase_I	V-type ATP	GO:0006351|t	GO:0005634|	GO:0003677|DI	.
Manes.05G20120(	0.7657878	0.00758	down	no	Armadillo rep	pfam00225	Kinesin	Kinesin mot	GO:0007018|r	GO:0005737|	GO:0005524|A	.
Manes.05G20160(	7.3747853	6.78E-09	up	yes	.	pfam11262	Tho2	Transcriptio	.	.	.	.
Manes.05G20170(	0.3433605	1.42E-16	down	yes	Protein RALI	pfam05498	RALF	Rapid ALka	GO:0019722|	GO:0048046|	GO:0005179|ho	.
Manes.05G20200(	0.3605418	1.31E-11	down	yes	Xyloglucan g	pfam03016	Exostosin	Exostosin fa	GO:0010256|	GO:0005768|	GO:0016757|tra	.
Manes.05G20230(	0.3841531	1.69E-05	down	yes	.	pfam03168	LEA_2	Late embryo	.	.	.	.
Manes.05G20240(	0.7180937	0.07194	down	no	.	pfam14910	MMS22L_N	S-phase gen	.	.	.	.
Manes.05G20250(	1.5842351	7.47E-07	up	no	Uncharacteriz	pfam00076	RRM_1	RNA recogn	GO:0042254|r	GO:0005730|	GO:0000166|nu	K14838
Manes.05G20290(	1.342969	0.04609	up	no	.	pfam05777	Acp26Ab	Drosophila a	.	.	.	.
Manes.05G20300(	0.8713985	0.21342	down	no	Nodulation p	pfam09037	Sulphotransf	Stf0 sulphot	GO:0009877|r	.	GO:0008146|su	.
Manes.05G20310(	0.3115618	5.43E-18	down	yes	Probable LRI	pfam00069	Pkinase	Protein kina	.	GO:0016021|	GO:0005524|A	.
Manes.05G20330(	0.6907268	0.01139	down	no	Heat shock c	pfam00183	HSP90	Hsp90 prote	GO:0006457|p	GO:0005737|	GO:0005524|A	K04079
Manes.05G20340(	0.9645294	0.72316	down	no	Peroxisome b	pfam01653	DNA_ligase_aden	NAD-depen	GO:0016560|p	GO:0005829|	GO:0005052|pe	K13342
Manes.05G20370(	0.7923199	0.05015	down	no	Putative 4-hy	pfam03737	Methyltransf_6	Demethylme	GO:0051252|r	.	GO:0047443|4-	K02553
Manes.05G20380(	2.227655	8.50E-06	up	yes	Serine/threon	pfam00069	Pkinase	Protein kina	GO:0009755|l	GO:0016021|	GO:0005524|A	.
Manes.05G20400(	Inf	0.00066	up	yes	.	pfam10118	Metal_hydrol	Predicted m	.	.	.	.
Manes.05G20440(	1.0618957	0.50907	up	no	Chloride char	pfam00654	Voltage_CLC	Voltage gat	.	GO:0034707|	GO:0005247|vo	.
Manes.05G20450(	2.5202185	1.27E-23	up	yes	.	pfam01603	B56	Protein phos	.	.	.	.
Manes.05G20490(	1.3525617	0.00878	up	no	Actin-related	pfam00022	Actin	Actin.	GO:0030029|	GO:0005737|	GO:0005524|A	K16616
Manes.05G20500(	3.4328495	5.37E-10	up	yes	GPI-anchore	pfam01490	Aa_trans	Transmemb	GO:0009567|	GO:0031225|	.	.
Manes.05G20510(	0.4957778	0.00018	down	yes	Phosphatidyli	pfam00650	CRAL_TRIO	CRAL/TRIO	GO:0015031|p	GO:0000139|	.	.
Manes.05G20520(	1.8350085	1.74E-05	up	no	Ubiquitin-con	pfam00179	UQ_con	Ubiquitin-co	GO:0016567|p	.	GO:0005524|A	K06689
Manes.05G20530(	1.1562392	0.38107	up	no	Probable calc	pfam13499	EF-hand_7	EF-hand do	.	GO:0005737|	GO:0005509|ca	K13448
Manes.05G20550(	0.3883753	0.00141	down	yes	.	pfam08395	7tm_7	7tm Chemos	.	.	.	.
Manes.05G20560(	Inf	0.9009	up	no	.	pfam06728	PIG-U	GPI transam	.	.	.	.
Manes.05G20590(	0.4487599	1.20E-10	down	yes	Chloroplast e	pfam03040	CemA	CemA famil	.	GO:0009706|	GO:0015078|hy	.
Manes.05G20600(	7.5504428	7.08E-10	up	yes	Probable gala	pfam05691	Raffinose_syn	Raffinose sy	GO:0005975|	.	GO:0047274|ga	K06617
Manes.05G20610(	1.7886722	2.29E-10	up	no	Enhancer of r	pfam00400	WD40	WD domain	GO:0071365|	GO:0000932|	.	K12616
Manes.05G20640(	1.6061989	1.11E-07	up	no	DNA damage	pfam09668	Asp_protease	Aspartyl pro	GO:0015031|p	GO:0005737|	GO:0004190|as	K11885
Manes.05G20650(	0.7753247	0.1334	down	no	RNA-binding	pfam00013	KH_1	KH domain.	GO:0022008|r	GO:0005730|	GO:0003723|RI	K11884
Manes.05G20680(	0.2667085	4.22E-10	down	yes	Subtilisin-like	pfam00082	Peptidase_S8	Subtilase fa	GO:0010102|l	GO:0005618|	GO:0004252|se	.
Manes.05G20690(	0.6876932	0.00912	down	no	BTB/POZ do	pfam00651	BTB	BTB/POZ d	GO:0016567|p	.	.	K10523
Manes.05G20700(	0.7598688	0.03022	down	no	Probable urid	pfam01156	IU_nuc_hydro	Inosine-urid	GO:0006148|i	GO:0005829|	GO:0047724|in	.
Manes.05G20770(	0.6750135	0.00187	down	no	Poly(ADP-rib	pfam05028	PARG_cat	Poly (ADP-r	GO:0005975|	.	GO:0004649|po	.
Manes.05G20800(	0.6326859	0.11153	down	no	Glucose-1-ph	pfam00483	NTP_transferase	Nucleotidyl	GO:0005978|g	GO:0048046|	GO:0005524|A	K00975
Manes.05G20830(	NA	NA	--	no	.	pfam10598	RRM_4	RNA recogn	.	.	.	.
Manes.05G20840(	14.824926	2.01E-74	up	yes	.	pfam10588	NADH-G_4Fe-4S	NADH-ubiq	.	.	.	.
Manes.06G00070(	1.2533785	0.01206	up	no	Putative D-cy	pfam00291	PALP	Pyridoxal-pl	GO:0019447|l	GO:0009507|	GO:0008660|1-	.
Manes.06G00080(	1.3818033	0.03229	up	no	Glutamate de	pfam00282	Pyridoxal_deC	Pyridoxal-de	GO:0006536|g	.	GO:0004351|gl	K01580
Manes.06G00110(	0.6791525	0.52186	down	no	Ankyrin repe	pfam13962	PGG	Domain of u	.	.	GO:0005524|A	K16289
Manes.06G00130(	0.8138648	0.04399	down	no	Probable seri	pfam00069	Pkinase	Protein kina	.	GO:0005737|	GO:0005524|A	K18669
Manes.06G00140(	0.8906256	0.41525	down	no	Protein SHO	pfam05701	WEMBL	Weak chloro	GO:0006355|r	GO:0005634|	GO:0003677|DI	.
Manes.06G00150(	2.8681504	5.19E-05	up	yes	Zinc-finger h	pfam04770	ZF-HD_dimer	ZF-HD prot	GO:0006355|r	GO:0005634|	GO:0003677|DI	.
Manes.06G00180(	0.8121486	0.21817	down	no	Transcription	pfam00249	Myb_DNA-binding	Myb-like DI	GO:0010158|	GO:0005634|	GO:0003677|DI	.
Manes.06G00190(	1.1443714	0.18076	up	no	Annexin D5	pfam00191	Annexin	Annexin. Th	GO:0009555|p	.	GO:0051015|ac	.
Manes.06G00200(	1.6899616	2.12E-07	up	no	Monoglyceric	pfam12146	Hydrolase_4	Putative lys	GO:0036155|	GO:0005829|	GO:0047372|ac	K01054
Manes.06G00230(	0.9588072	0.75301	down	no	RNA polyme	pfam04722	Ssu72	Ssu72-like p	GO:0006397|r	GO:0005737|	GO:0004721|ph	K15544
Manes.06G00240(	Inf	0.01325	up	yes	U-box domai	pfam04564	U-box	U-box doma	GO:0010200|r	.	GO:0016874|lig	.
Manes.06G00250(	1.2442024	0.01695	up	no	.	pfam00769	ERM	Ezrin/radixi	.	.	.	.
Manes.06G00270(	12.835806	3.73E-50	up	yes	Mitogen-acti	pfam00069	Pkinase	Protein kina	GO:0000187|	GO:0005737|	GO:0005524|A	.
Manes.06G00280(	0.3082665	0.07156	down	no	.	pfam05216	UNC-50	UNC-50 far	.	.	.	.
Manes.06G00290(	0.7547113	0.00931	down	no	NAD-depend	pfam01180	DHO_dh	Dihydrooro	GO:0006208|p	GO:0005737|	GO:0051539|4	K17723
Manes.06G00320(	3.5466573	2.45E-44	up	yes	NF-X1-type z	pfam02705	K_trans	K+ potassiu	GO:0042742|	GO:0005634|	GO:0016874|lig	K12236
Manes.06G00350(	2.7497041	7.12E-08	up	yes	.	pfam14480	DNA_pol3_a_NI	DNA polym	.	.	.	.
Manes.06G00360(	3.2685895	4.54E-42	up	yes	Nudix hydrol	pfam00293	NUDIX	NUDIX dor	.	.	GO:0047631|Al	.
Manes.06G00390(	Inf	0.35135	up	no	CBL-interact	pfam00069	Pkinase	Protein kina	GO:0050832|	.	GO:0005524|A	.
Manes.06G00400(	1.4126058	0.00014	up	no	Protein TIFY	pfam06200	tify	tify domain.	GO:0006952|	GO:0005634|	GO:0042802|id	K13464
Manes.06G00430(	0.3631759	0.00011	down	yes	ABC transpo	pfam01061	ABC2_membrane	ABC-2 type	GO:0010208|p	GO:0016021|	GO:0005524|A	.
Manes.06G00490(	0.0978	0.00063	down	yes	.	pfam14009	DUF4228	Domain of u	.	.	.	.
Manes.06G00520(	1.2884075	0.09469	up	no	Fructose-bisp	pfam01116	F_bP_aldolase	Fructose-bis	GO:0030388|	.	GO:0004332|fru	.
Manes.06G00530(	66.650766	#######	up	yes	60S ribosoma	pfam01280	Ribosomal_L19e	Ribosomal p	GO:0006412|t	GO:0022625|	GO:0003735|str	K02885
Manes.06G00540(	0.7322128	0.01435	down	no	Down syndro	pfam03643	Vps26	Vacuolar pr	.	GO:0005634|	.	.
Manes.06G00570(	0.2911532	2.80E-05	down	yes	Boron transp	pfam03798	TRAM_LAG1_CI	TLC domain	GO:0010036|	GO:0005768|	GO:0005452|in	.
Manes.06G00580(	1.3634314	0.00083	up	no	ERAD-assoc	pfam13414	TPR_11	TPR repeat.	GO:0030433|	GO:0005783|	.	K14026
Manes.06G00610(	0.6944018	0.00017	down	no	Outer envelo	pfam01425	Amidase	Amidase.	GO:0015031|p	GO:0009507|	GO:0016884|ca	.
Manes.06G00620(	0.1912086	8.91E-46	down	yes	Cytochrome	pfam00067	p450	Cytochrome	GO:0071555|	GO:0005783|	GO:0020037|he	K15402
Manes.06G00650(	5.0417229	3.13E-77	up	yes	Cytochrome	pfam00067	p450	Cytochrome	GO:0071555|	GO:0005783|	GO:0020037|he	K15402
Manes.06G00660(	5.2197524	5.29E-37	up	yes	Cytochrome	pfam00067	p450	Cytochrome	GO:0071555|	GO:0005783|	GO:0020037|he	K15402
Manes.06G00670(	0.6115458	0.0026	down	no	Probable AD	pfam00168	C2	C2 domain.	GO:0009555|p	.	GO:0005096|G	K12486
Manes.06G00680(	0.6527413	0.00029	down	no	Auxin efflux	pfam03547	Mem_trans	Membrane t	GO:0048830|	GO:0009921|	GO:0005215|tra	K13947
Manes.06G00730(	0.4108998	0.00012	down	yes	WUSCHEL-r	pfam00046	Homeobox	Homeobox	GO:0048513|	GO:0005829|	GO:0003677|DI	.
Manes.06G00750(	0.4140914	5.90E-08	down	yes	.	pfam13041	PPR_2	PPR repeat	.	.	.	.
Manes.06G00760(	0.9953338	1	down	no	Probable calc	pfam13499	EF-hand_7	EF-hand do	.	GO:0005886|	GO:0005509|ca	K13448
Manes.06G00770(	1.2365913	0.30151	up	no	Ras-related p	pfam00071	Ras	Ras family.	GO:0006886|i	GO:0005622|	GO:0005525|G	K07976
Manes.06G00780(	0.8230017	0.08928	down	no	.	pfam06966	DUF1295	Protein of u	.	.	.	.
Manes.06G00790(	1.4698438	1.72E-05	up	no	Serine/threon	pfam14381	EDR1	Ethylene-res	GO:0009738|	GO:0005829|	GO:0005524|A	.
Manes.06G00800(	0.6221902	0.00033	down	no	Mitogen-acti	pfam00069	Pkinase	Protein kina	GO:0009738|	GO:0005829|	GO:0005524|A	.
Manes.06G00850(	1.8252481	1.39E-10	up	no	Peroxisome b	pfam04882	Peroxin-3	Peroxin-3. F	GO:0007031|p	GO:0005779|	.	.
Manes.06G00860(	1.4483988	4.52E-05	up	no	Chaperone pr	pfam00226	DnaJ	DnaJ domai	GO:0006260|	GO:0005737|	GO:0005524|A	K03686
Manes.06G00870(	0.441447	7.60E-08	down	yes	Ubiquinol ox	pfam01786	AOX	Alternative	GO:0010230|	GO:0016021|	GO:0009916|alt	K17893
Manes.06G00930(	3.9480157	0.01634	up	yes	.	pfam00146	NADHdh	NADH dehy	.	.	.	.
Manes.06G00950(	0.542945	1.42E-05	down	no	.	pfam01344	Kelch_1	Kelch motif.	.	.	.	.
Manes.06G00970(	1.1297952	0.31081	up	no	Copper trans	pfam00403	HMA	Heavy-meta	GO:0006825|	GO:0000785|	GO:0046872|m	K07213
Manes.06G01060(	1.3006117	0.00383	up	no	.	pfam02990	EMP70	Endomembr	.	.	.	.

Manes.06G01080(	0.4611049	7.50E-06	down	yes	G-type lectin	pfam07714	Pkinase_Tyr	Protein tyro	GO:0048544|	GO:0016021|	GO:0005524|A	.
Manes.06G01260(	0.7748467	0.00826	down	no	Copper-trans	pfam00122	E1-E2_ATPase	E1-E2 ATP	GO:0035434|	GO:0009507|	GO:0005524|A	.
Manes.06G01280(	1.9194376	3.26E-12	up	no	Calmodulin-b	pfam03859	CG-1	CG-1 domai	GO:0071275|	GO:0005634|	GO:0003677|D	.
Manes.06G01320(	0.4057494	1.48E-07	down	yes	Subtilisin-like	pfam00082	Peptidase_S8	Subtilase fa	GO:0080001|	GO:0048046|	GO:0004252|se	.
Manes.06G01350(	0.0691052	0.00036	down	yes	.	pfam09811	Yae1_N	Essential pro	.	.	.	.
Manes.06G01370(	1.507697	3.42E-05	up	no	NADH dehyc	pfam08122	NDUF_B12	NADH-ubiq	GO:0022900|	GO:0016021|	.	.
Manes.06G01390(	0.4960893	2.37E-08	down	yes	Putative disea	pfam00931	NB-ARC	NB-ARC do	GO:0006952|	.	GO:0043531|A	.
Manes.06G01400(	0.2250473	0.00551	down	yes	.	pfam10269	Tmemb_185A	Transmemb	.	.	.	.
Manes.06G01410(	0.4797458	1.21E-07	down	yes	RNA pseudo	pfam00849	PseudoU_synth_2	RNA pseud	GO:0001522|	GO:0009507|	GO:0009982|ps	K15452
Manes.06G01490(	0.5992992	0.00013	down	no	GTPase Der	pfam14714	KH_dom-like	KH-domain	GO:0042254|	.	GO:0005525|G	K03977
Manes.06G01530(	1.9322081	1.43E-07	up	no	PCTP-like pr	pfam01852	START	START dor	GO:0032782|	GO:0005829|	GO:0008289|lip	.
Manes.06G01540(	3.7645306	4.76E-19	up	yes	.	pfam05231	MASE1	MASE1. Pr	.	.	.	.
Manes.06G01550(	0.460279	0.00012	down	yes	.	pfam04615	Utp14	Utp14 prote	.	.	.	.
Manes.06G01600(	0.7052183	0.01535	down	no	Pentatricopep	pfam13041	PPR_2	PPR repeat	.	GO:0005739|	.	.
Manes.06G01620(	1.8744698	3.51E-07	up	no	Protein transp	pfam00400	WD40	WD domain	GO:0051028|	GO:0005783|	.	K14004
Manes.06G01660(	0.8584518	0.12623	down	no	Glutathione S	pfam13417	GST_N_3	Glutathione	GO:0009072|	GO:0005737|	GO:0004364|gl	.
Manes.06G01690(	1.6041403	5.30E-07	up	no	Endoglucanas	pfam00759	Glyco_hydro_9	Glycosyl hy	GO:0071555|	GO:0009504|	GO:0008810|ce	.
Manes.06G01710(	0.7018375	0.00388	down	no	.	pfam09335	SNARE_assoc	SNARE ass	.	.	.	.
Manes.06G01730(	0.3192493	1.54E-07	down	yes	Putative clath	pfam07651	ANTH	ANTH dom	GO:0048268|	GO:0030136|	GO:0005545|1-	.
Manes.06G01740(	0.5907648	0.00325	down	no	Putative olig	pfam04756	OST3_OST6	OST3 / OST	.	GO:0016021|	.	.
Manes.06G01770(	1.0947122	0.32497	up	no	Calcium-trans	pfam00122	E1-E2_ATPase	E1-E2 ATP	.	GO:0016021|	GO:0005524|A	K01537
Manes.06G01820(	0.9795146	0.82644	down	no	Protein CLE	pfam09758	FPL	Uncharacter	GO:0006914|	.	.	.
Manes.06G01840(	1.194787	0.1292	up	no	Mannosyl-oli	pfam01532	Glyco_hydro_47	Glycosyl hy	GO:0006491|	GO:0005783|	GO:0004559|al	K01230
Manes.06G01850(	1.1660039	0.13224	up	no	Mannosyl-oli	pfam01532	Glyco_hydro_47	Glycosyl hy	GO:0006491|	GO:0005783|	GO:0004559|al	K01230
Manes.06G01860(	1.6360093	2.89E-06	up	no	.	pfam04617	Hox9_act	Hox9 activa	.	.	.	.
Manes.06G01900(	0.8976268	0.33201	down	no	RNA polyme	pfam03031	NIF	NLI interact	GO:0006355|	GO:0005634|	GO:0046872|m	K18999
Manes.06G01930(	2.3990017	8.86E-22	up	yes	Protein CHL	pfam06203	CCT	CCT motif.	GO:0045036|	GO:0009507|	.	.
Manes.06G02000(	1.6944825	2.99E-09	up	no	.	pfam10206	WRW	Mitochondr	.	.	.	.
Manes.06G02020(	2.209807	1.66E-14	up	yes	.	pfam02694	UPF0060	Uncharacter	.	.	.	.
Manes.06G02030(	1.8999657	4.35E-13	up	no	TBC1 domai	pfam00566	RabGAP-TBC	Rab-GTPas	.	.	GO:0005096|G	.
Manes.06G02050(	0.3884854	0.00065	down	yes	Oxysterol-bin	pfam01237	Oxysterol_BP	Oxysterol-b	GO:0006869|	.	GO:0008289|lip	.
Manes.06G02070(	1.3216033	0.0045	up	no	.	pfam02485	Branch	Core-2/I-Br	.	.	.	.
Manes.06G02110(	1.6640946	8.50E-08	up	no	54S ribosom	pfam05047	L51_S25_CI-B8	Mitochondr	GO:0032543|	GO:0005762|	GO:0044822|pc	K17424
Manes.06G02140(	1.7218818	9.26E-10	up	no	Villin-4	pfam02209	VHP	Villin headp	GO:0051017|	GO:0005737|	.	.
Manes.06G02180(	Inf	0.9009	up	no	30S ribosom	pfam00164	Ribosom_S12_S2	Ribosomal p	GO:0006412|	GO:0009507|	GO:0019843|rR	.
Manes.06G02200(	1.1655227	0.33635	up	no	.	pfam14529	Exo_endo_phos_2	Endonuclea	.	.	.	.
Manes.06G02220(	7.1373536	2.31E-52	up	yes	.	pfam14915	CCDC144C	CCDC144C	.	.	.	.
Manes.06G02240(	0.5433765	1.23E-08	down	no	Homogentisa	pfam01040	UbiA	UbiA prenyl	GO:0071555|	GO:0009507|	GO:0010176|ho	K09833
Manes.06G02310(	1.1439152	0.13416	up	no	WD repeat-c	pfam00400	WD40	WD domain	.	GO:0005730|	.	K14552
Manes.06G02320(	0.7170942	0.14036	down	no	Probable rece	pfam00069	Pkinase	Protein kina	.	.	GO:0005524|A	.
Manes.06G02330(	0.7455166	0.00302	down	no	VIN3-like pr	pfam07227	DUF1423	Protein of u	GO:0032922|	GO:0005677|	GO:0003677|D	.
Manes.06G02370(	1.7845697	2.13E-10	up	no	.	pfam07887	Calmodulin_bind	Calmodulin	.	.	.	.
Manes.06G02380(	0.731104	0.00143	down	no	Bifunctional	pfam00202	Aminotran_3	Aminotrans	GO:0009102|	GO:0005739|	GO:0004015|ad	K00833
Manes.06G02420(	6.4399251	5.21E-92	up	yes	Tetraspanin-8	pfam00335	Tetraspannin	Tetraspanin	.	GO:0005794|	.	.
Manes.06G02430(	1.0511102	0.57631	up	no	Pheophytinas	pfam12697	Abhydrolase_6	Alpha/beta l	GO:0015996|	GO:0009507|	GO:0080124|ph	.
Manes.06G02600(	0.7454328	0.02076	down	no	.	pfam04111	APG6	Autophagy	.	.	.	.
Manes.06G02630(	0.5901166	9.03E-06	down	no	.	pfam07534	TLD	TLD. This c	.	.	.	.
Manes.06G02640(	1.7098716	6.74E-09	up	no	Chaperone pr	pfam07724	AAA_2	AAA domai	GO:0019538|	GO:0005737|	GO:0005524|A	K03695
Manes.06G02690(	0.1625583	6.88E-20	down	yes	Pathogenesis	pfam00188	CAP	Cysteine-ric	GO:0006952|	GO:0005576|	.	.
Manes.06G02700(	0.3810987	1.17E-07	down	yes	Pathogenesis	pfam00188	CAP	Cysteine-ric	GO:0006952|	GO:0005576|	.	.
Manes.06G02720(	0.8456379	0.11548	down	no	Putative lipas	pfam05057	DUF676	Putative ser	GO:0044255|	GO:0005737|	GO:0047372|ac	.
Manes.06G02790(	0.9412119	0.53234	down	no	ABC transpo	pfam06472	ABC_membrane_	ABC transp	GO:0016042|	GO:0046861|	GO:0005524|A	.
Manes.06G02870(	1.3725023	0.00045	up	no	Diacylglycerc	pfam00609	DAGK_acc	Diacylglyce	GO:0006952|	GO:0016020|	GO:0005524|A	K00901
Manes.06G02880(	0.610116	0.00022	down	no	.	pfam13088	BNR_2	BNR repeat	.	.	.	.
Manes.06G02900(	Inf	0.01359	up	yes	Putative rece	pfam00069	Pkinase	Protein kina	GO:0048544|	GO:0016021|	GO:0005524|A	.
Manes.06G03020(	0.5543109	0.02024	down	no	.	pfam14264	Glucos_trans_II	Glucosyl tra	.	.	.	.
Manes.06G03030(	5.0662983	2.64E-15	up	yes	18.5 kDa clas	pfam00011	HSP20	Hsp20/alpha	.	GO:0005737|	.	K13993
Manes.06G03040(	0.6403424	0.00312	down	no	Ribonuclease	pfam08615	RNase_H2_suC	Ribonucleas	GO:0006401|	GO:0005634|	.	K10745
Manes.06G03060(	1.6521878	2.43E-08	up	no	Outer envelo	pfam13414	TPR_11	TPR repeat.	GO:0061077|	GO:0009707|	GO:0005528|F	.
Manes.06G03120(	0.8950184	0.30773	down	no	Ubiquitin car	pfam00443	UCH	Ubiquitin ca	GO:0043161|	GO:0005634|	GO:0004197|cy	K11835
Manes.06G03140(	0.8520314	0.27535	down	no	.	pfam04576	Zein-binding	Zein-binding	.	.	.	.
Manes.06G03160(	0.664184	0.00218	down	no	mRNA cap g	pfam03291	Pox_MCEL	mRNA capp	.	GO:0005634|	GO:0004482|m	K00565
Manes.06G03180(	0.8367172	0.06949	down	no	.	pfam15279	SOBP	Sine oculis-l	.	.	.	.
Manes.06G03220(	0.9277313	0.55787	down	no	Clustered mit	pfam12807	eIF3_p135	Translation	GO:0048312|	GO:0005737|	.	K03255
Manes.06G03260(	3.5380296	1.16E-08	up	yes	Putative gluta	pfam00117	GATase	Glutamine a	GO:0006878|	GO:0005737|	GO:0016740|tra	.
Manes.06G03300(	1.6548615	4.45E-06	up	no	Transmembra	pfam05620	DUF788	Protein of u	GO:0006624|	GO:0005789|	.	.
Manes.06G03330(	0.6675271	0.00817	down	no	.	pfam01935	DUF87	Domain of u	.	.	.	.
Manes.06G03400(	0.6662799	0.00932	down	no	26S proteaso	pfam13180	PDZ_2	PDZ domai	GO:0046676|	GO:0005737|	GO:0043425|b	K06693
Manes.06G03410(	1.5346964	1.72E-06	up	no	Leucine amin	pfam00883	Peptidase_M17	Cytosol ami	GO:0010150|	GO:0009507|	GO:0004177|an	K01255
Manes.06G03430(	0.8838639	0.1934	down	no	Probable cycl	pfam00520	Ion_trans	Ion transpo	GO:0070588|	GO:0005887|	GO:0005262|ca	K05391
Manes.06G03480(	0.2284272	7.23E-26	down	yes	Shikimate O-	pfam02458	Transferase	Transferase	.	.	GO:0047172|sh	.
Manes.06G03490(	0.3991779	3.72E-10	down	yes	Shikimate O-	pfam02458	Transferase	Transferase	.	.	GO:0047172|sh	.
Manes.06G03530(	0.4764678	0.00536	down	yes	V-type proto	pfam00902	TatC	Sec-indepen	GO:0015991|	GO:0033180|	GO:0005524|A	.
Manes.06G03540(	0.6434235	0.00159	down	no	.	pfam12874	zf-met	Zinc-finger	.	.	.	.
Manes.06G03550(	2.8285488	1.76E-32	up	yes	.	pfam15390	DUF4613	Domain of u	.	.	.	.
Manes.06G03560(	0.6154967	3.37E-06	down	no	Protein PHR	pfam14379	Myb_CC_LHEQL	MYB-CC ty	GO:0006351|	GO:0005634|	GO:0003677|D	.
Manes.06G03630(	0.8767586	0.2107	down	no	Ubiquitin car	pfam00443	UCH	Ubiquitin ca	GO:0043161|	.	GO:0004197|cy	K11855
Manes.06G03640(	4.0702603	2.04E-41	up	yes	BTB/POZ do	pfam02214	BTB_2	BTB/POZ d	GO:0051260|	.	.	.
Manes.06G03660(	0.497737	1.40E-06	down	yes	Reticulon-like	pfam02453	Reticulon	Reticulon. F	.	GO:0005789|	.	.
Manes.06G03670(	3.4285818	4.46E-36	up	yes	CBL-interact	pfam00069	Pkinase	Protein kina	GO:0010540|	GO:0005783|	GO:0005524|A	.
Manes.06G03700(	0.6277264	0.01078	down	no	.	pfam02297	COX6B	Cytochrome	.	.	.	.
Manes.06G03750(	2.2952085	5.93E-06	up	yes	Small nuclear	pfam01423	LSM	LSM domai	GO:0000387|	GO:0005829|	GO:0003723|R	K11097
Manes.06G03790(	1.0576676	0.53389	up	no	FIP1[V]-like	pfam05182	Fip1	Fip1 motif.	GO:0006397|	GO:0005634|	GO:0042802|id	.
Manes.06G03810(	0.7217719	0.01939	down	no	.	pfam00149	Metallophos	Calcineurin-	.	.	.	.
Manes.06G03820(	1.1804606	0.19783	up	no	.	pfam06364	DUF1068	Protein of u	.	.	.	.
Manes.06G03840(	5.147676	5.71E-45	up	yes	.	pfam07887	Calmodulin_bind	Calmodulin	.	.	.	.
Manes.06G03850(	0.9064918	0.471	down	no	Two-compor	pfam00072	Response_reg	Response re	GO:0071368|	GO:0005634|	GO:0003677|D	K14491
Manes.06G03900(	NA	NA	--	no	.	pfam00728	Glyco_hydro_20	Glycosyl hy	.	.	.	.

Manes.06G03920(	0.8151482	0.08542	down	no	.	pfam06023	DUF911	Archaeal prc	.	.	.	.
Manes.06G03940(	0.994679	0.98356	down	no	VAMP-like p	pfam00957	Synaptobrevin	Synaptobrev	GO:0015031|p	GO:0016021|	.	.
Manes.06G03970(	0.4578521	7.52E-06	down	yes	.	pfam00400	WD40	WD domain	.	.	.	.
Manes.06G03980(	0.8723047	0.3321	down	no	.	pfam01266	DAO	FAD depend	.	.	.	.
Manes.06G04080(	4.5839729	3.75E-62	up	yes	Sugar carrier	pfam00083	Sugar_tr	Sugar (and c	GO:0008643|c	GO:0016021|	GO:0022891|su	.
Manes.06G04100(	1.5373452	1.61E-06	up	no	Transmembra	pfam02990	EMP70	Endomembr	.	GO:0010008|	.	.
Manes.06G04120(	1.5342777	2.78E-06	up	no	Homeobox p	pfam05920	Homeobox_KN	Homeobox 1	GO:0006355|r	GO:0005634|	GO:0003677|DI	.
Manes.06G04130(	0.5471446	1.47E-07	down	no	E3 ubiquitin-	pfam13639	zf-RING_2	Ring finger c	GO:0090090|n	GO:0005887|	GO:0016874|lig	K16273
Manes.06G04170(	0.3308464	0.06718	down	no	.	pfam13678	Peptidase_M85	NFkB-p65-c	.	.	.	.
Manes.06G04220(	10.204266	0.00013	up	yes	Probable LRI	pfam13855	LRR_8	Leucine rich	.	GO:0016021|	GO:0005524|A'	.
Manes.06G04260(	6.150623	0.00023	up	yes	LRR recepto	pfam13855	LRR_8	Leucine rich	.	GO:0016021|	GO:0005524|A'	.
Manes.06G04300(	2.5646451	3.03E-07	up	yes	.	pfam00055	Laminin_N	Laminin N-t	.	.	.	.
Manes.06G04310(	1.0038096	1	up	no	.	pfam05478	Prominin	Prominin. T	.	.	.	.
Manes.06G04320(	3.578409	1.09E-08	up	yes	.	pfam04640	PLATZ	PLATZ tran	.	.	.	.
Manes.06G04330(	0.5303145	0.00023	down	no	Probable flav	pfam00743	FMO-like	Flavin-bindi	GO:0071456|c	.	GO:0050660|fla	K00485
Manes.06G04360(	0.6457954	0.00033	down	no	.	pfam03251	Tymo_45kd_70kd	Tymovirus 4	.	.	.	.
Manes.06G04380(	1.6219492	4.91E-05	up	no	.	pfam06813	Nodulin-like	Nodulin-like	.	.	.	.
Manes.06G04390(	0.5005888	7.68E-06	down	no	.	pfam07647	SAM_2	SAM domai	.	.	.	.
Manes.06G04420(	1.0931105	0.32823	up	no	Protein S-acy	pfam01529	zf-DHHC	DHHC palm	GO:0009932|c	GO:0030659|	GO:0000035|ac	K18932
Manes.06G04460(	1.9844311	1.57E-12	up	no	Heparanase-l	pfam03662	Glyco_hydro_79n	Glycosyl hyd	.	GO:0005576|	GO:0004566|be	.
Manes.06G04500(	0.7105081	0.071	down	no	Protein SRG1	pfam03171	2OG-FeII_Oxy	2OG-Fe(II)	GO:0010260|c	.	GO:0046872|m	.
Manes.06G04510(	1.323903	0.01568	up	no	Pyrophospha	pfam03030	H_PPase	Inorganic H	GO:0015992|p	GO:0016021|	GO:0009678|hy	.
Manes.06G04520(	1.8534982	1.84E-07	up	no	.	pfam12678	zf-rbx1	RING-H2 zi	.	.	.	.
Manes.06G04530(	0.6833384	0.00055	down	no	.	pfam03108	DBD_Tnp_Mut	MuDR fami	.	.	.	.
Manes.06G04540(	0.8627953	0.36751	down	no	Translocase c	pfam11886	DUF3406	Domain of u	GO:0045036|p	GO:0009707|	GO:0005525|G'	.
Manes.06G04550(	7.0084243	1.44E-20	up	yes	.	pfam09989	DUF2229	CoA enzym	.	.	.	.
Manes.06G04570(	0.6620507	0.00082	down	no	Putative chlo	pfam00654	Voltage_CLC	Voltage gat	.	GO:0034707|	GO:0005247|vo	K05016
Manes.06G04590(	Inf	0.9009	up	no	Heptahelical	pfam03006	HlyIII	Haemolysin-	GO:0009788|n	GO:0016021|	GO:0004872|re	K07297
Manes.06G04640(	0.9946227	0.97207	down	no	Putative clath	pfam07651	ANTH	ANTH dom	GO:0048268|c	GO:0030136|	GO:0005545|1-	.
Manes.06G04650(	0.5378739	2.59E-08	down	no	.	pfam15279	SOBP	Sine oculis-l	.	.	.	.
Manes.06G04690(	0.4612193	1.29E-06	down	yes	.	pfam10327	7TM_GPCR_Sri	Serpentine t	.	.	.	.
Manes.06G04700(	1.6457745	2.71E-08	up	no	CCR4-NOT t	pfam13424	TPR_12	Tetratricope	GO:0031047|g	GO:0030014|	.	K12607
Manes.06G04720(	0.5655143	0.03374	down	no	Probable gala	pfam05691	Raffinose_syn	Raffinose sy	GO:0005975|c	.	GO:0047274|ga	K06617
Manes.06G04760(	0.0566019	3.38E-05	down	yes	.	pfam01034	Syndecan	Syndecan dc	.	.	.	.
Manes.06G04780(	1.8835536	7.85E-09	up	no	Calmodulin-li	pfam13499	EF-hand_7	EF-hand do	.	GO:0005777|	GO:0005509|ca	K13448
Manes.06G04790(	1.0423578	0.65958	up	no	.	pfam14817	HAUS5	HAUS augm	.	.	.	.
Manes.06G04800(	0.3779464	3.46E-09	down	yes	Probable rece	pfam07714	Pkinase_Tyr	Protein tyro	.	GO:0016021|	GO:0005524|A'	.
Manes.06G04830(	0.4891311	0.0084	down	yes	UDP-N-acety	pfam03151	TPT	Triose-phos	GO:0008643|c	GO:0000139|	.	K15281
Manes.06G04890(	1.6819546	0.00015	up	no	NHP2-like pr	pfam01248	Ribosomal_L7Ae	Ribosomal p	GO:0006397|n	GO:0005730|	GO:0003723|RI	K12845
Manes.06G04950(	0.5218839	1.50E-06	down	no	.	pfam01344	Kelch_1	Kelch motif.	.	.	.	.
Manes.06G04970(	1.6641171	7.99E-08	up	no	Mediator of I	pfam10156	Med17	Subunit 17 c	GO:0006351|t	GO:0016592|	GO:0001104|RI	K15133
Manes.06G05030(	4.5521611	2.92E-23	up	yes	.	pfam03798	TRAM_LAG1_CI	TLC domair	.	.	.	.
Manes.06G05060(	1.0323981	0.74215	up	no	.	pfam12906	RINGv	RING-varia	.	.	.	.
Manes.06G05070(	1.9582775	1.41E-06	up	no	EG45-like do	pfam03330	DPBB_1	Rare lipopro	.	GO:0005576|	.	.
Manes.06G05110(	6.9515663	#######	up	yes	3-ketoacyl-C	pfam08392	FAE1_CUT1_Rpp	FAE1/Type	GO:0006633|f	GO:0016021|	GO:0033807|ic	K15397
Manes.06G05140(	0.2666844	4.03E-05	down	yes	.	pfam09825	BPL_N	Biotin-prote	.	.	.	.
Manes.06G05160(	Inf	0.9009	up	no	Non-specific	pfam00234	Tryp_alpha_amyl	Protease inh	GO:0006869|l	.	GO:0008289|lip	.
Manes.06G05180(	1.8111797	0.00094	up	no	.	pfam13041	PPR_2	PPR repeat	.	.	.	.
Manes.06G05250(	0.6971702	0.06068	down	no	.	pfam08006	DUF1700	Protein of u	.	.	.	.
Manes.06G05260(	4.0413605	1.25E-54	up	yes	GTP-binding	pfam00071	Ras	Ras family.	GO:0006606|p	GO:0005737|	GO:0005525|G'	K07936
Manes.06G05270(	0.4417546	0.00045	down	yes	Cytochrome 1	pfam00067	p450	Cytochrome	GO:0010268|b	GO:0016021|	GO:0020037|he	K15639
Manes.06G05310(	0.7469019	0.004	down	no	Acyl-protein	pfam02230	Abhydrolase_2	Phospholipa	GO:0006631|f	GO:0005737|	GO:0016787|hy	K06130
Manes.06G05330(	0.9884827	0.93168	down	no	.	pfam07800	DUF1644	Protein of u	.	.	.	.
Manes.06G05380(	0.3365782	4.03E-07	down	yes	Pentatricopep	pfam14432	DYW_deaminase	DYW family	GO:0031425|c	GO:0009507|	.	.
Manes.06G05390(	0.8838371	0.32054	down	no	Coatomer sul	pfam04733	Coatomer_E	Coatomer ep	GO:0015031|p	GO:0030663|	GO:0005198|str	K17268
Manes.06G05400(	0.8795616	0.18597	down	no	Isoamylase 1	pfam00128	Alpha-amylase	Alpha amyla	GO:0010021|a	GO:0009507|	GO:0043169|ca	K01214
Manes.06G05410(	1.5457687	0.0002	up	no	UDP-arabino	pfam01370	Epimerase	NAD depen	GO:0019567|a	GO:0005794|	GO:0050662|co	K12448
Manes.06G05430(	0.2506373	2.82E-13	down	yes	Protein LIGH	pfam04852	DUF640	Protein of u	GO:0009299|n	GO:0005634|	GO:0003677|DI	.
Manes.06G05500(	1.8398807	7.42E-11	up	no	Homeobox-le	pfam08670	MEKHLA	MEKHLA d	GO:0009955|a	GO:0005634|	GO:0003677|DI	K09338
Manes.06G05530(	1.295127	0.00406	up	no	Phenylalanine	pfam03483	B3_4	B3/4 domai	GO:0006432|p	GO:0005829|	GO:0005524|A'	K01890
Manes.06G05570(	1.0208579	0.79527	up	no	187-kDa mic	pfam12799	LRR_4	Leucine Ric	GO:0051301|c	GO:0055028|	.	.
Manes.06G05580(	0.2049829	1.09E-06	down	yes	Secoisolaricir	pfam00106	adh_short	short chain c	.	.	GO:0016491|ox	.
Manes.06G05590(	0.589781	1.79E-06	down	no	.	pfam04724	Glyco_transf_17	Glycosyltrar	.	.	.	.
Manes.06G05630(	0.5205525	4.33E-07	down	no	.	pfam15331	TP53IP5	Cellular tum	.	.	.	.
Manes.06G05640(	1.772005	1.46E-10	up	no	Cucumber pe	pfam02298	Cu_bind_like	Plastocyanir	GO:0055114|c	.	GO:0009055|ele	.
Manes.06G05650(	88.179709	2.84E-05	up	yes	Cucumber pe	pfam02298	Cu_bind_like	Plastocyanir	GO:0055114|c	.	GO:0009055|ele	.
Manes.06G05700(	1.2323906	0.06257	up	no	Thylakoid lur	pfam00387	PI-PLC-Y	Phosphatidy	GO:0009735|r	GO:0009507|	.	.
Manes.06G05720(	0.8627583	0.12324	down	no	Axial regulato	pfam04690	YABBY	YABBY pro	GO:0009944|p	GO:0005634|	GO:0003677|DI	.
Manes.06G05730(	0.3560158	6.59E-05	down	yes	.	pfam01566	Nramp	Natural resi	.	.	.	.
Manes.06G05760(	1.0099072	0.91894	up	no	CBL-interact	pfam00069	Pkinase	Protein kina	GO:0035556|i	GO:0005737|	GO:0005524|A'	.
Manes.06G05770(	13.032559	2.22E-29	up	yes	.	pfam13090	PP_kinase_C	Polyphospha	.	.	.	.
Manes.06G05800(	1.1975294	0.38563	up	no	.	pfam13456	RVT_3	Reverse trar	.	.	.	.
Manes.06G05830(	1.0412837	0.63654	up	no	Histidinol del	pfam00815	Histidinol_dh	Histidinol de	GO:0000105|h	GO:0009507|	GO:0004399|his	.
Manes.06G05880(	1.3826952	0.00153	up	no	Fatty acid 2-h	pfam04116	FA_hydroxylase	Fatty acid hy	GO:0006633|f	GO:0005783|	GO:0005506|iro	.
Manes.06G05900(	3.9334416	1.90E-30	up	yes	Tetrahydroca	pfam08031	BBE	Berberine ar	.	GO:0048046|	GO:0050660|fla	.
Manes.06G05910(	7.0449657	#######	up	yes	Tetrahydroca	pfam01565	FAD_binding_4	FAD binding	.	GO:0048046|	GO:0050660|fla	.
Manes.06G05940(	0.8788286	0.56628	down	no	Reticuline ox	pfam01565	FAD_binding_4	FAD binding	GO:0006979|r	GO:0031225|	GO:0050660|fla	.
Manes.06G06020(	0.0369214	0.00012	down	yes	Probable 3-hy	pfam00378	ECH	Enoyl-CoA	GO:0006574|v	GO:0005777|	GO:0003860|3-	K05605
Manes.06G06030(	3.0258401	0.0261	up	yes	Cationic amir	pfam13520	AA_permease_2	Amino acid	.	GO:0005887|	GO:0015171|an	.
Manes.06G06050(	1.574631	3.65E-07	up	no	Cation-chlori	pfam00324	AA_permease	Amino acid	GO:1902476|c	GO:0005768|	GO:0008511|so	.
Manes.06G06060(	1.4941688	1.93E-05	up	no	BTB/POZ do	pfam03000	NPH3	NPH3 famil	GO:0016567|p	GO:0005886|	.	.
Manes.06G06070(	0.5715047	3.95E-08	down	no	.	pfam04782	DUF632	Protein of u	.	.	.	.
Manes.06G06160(	0.6115409	0.05359	down	no	.	pfam03547	Mem_trans	Membrane t	.	.	.	.
Manes.06G06170(	2.5331165	4.11E-24	up	yes	Probable ribo	pfam06026	Rib_5-P_isom_A	Ribose 5-ph	GO:0008219|c	GO:0005737|	GO:0004751|rib	K01807
Manes.06G06180(	0.5571751	3.26E-06	down	no	.	pfam00335	Tetraspannin	Tetraspanin	.	.	.	.
Manes.06G06220(	0.7246109	0.00338	down	no	Repressor of	pfam09174	Maf1	Maf1 regula	GO:0016480|n	GO:0005737|	.	.
Manes.06G06240(	6.4448232	1.10E-10	up	yes	.	pfam14009	DUF4228	Domain of u	.	.	.	.
Manes.06G06260(	1.5177287	3.79E-06	up	no	ALG-2 intera	pfam03097	BRO1	BRO1-like c	GO:0009653|a	GO:0005737|	.	K12200

Manes.06G06280(	12.072905	2.31E-05	up	yes	.	pfam09088	MIF4G_like	MIF4G like	.	.	.	.
Manes.06G06310(	0.6536636	0.00511	down	no	Sec-independ	pfam00902	TatC	Sec-indepen	GO:0045038|	GO:0009535|	.	.
Manes.06G06340(	2.0866112	5.25E-17	up	yes	CDP-diacylgl	pfam03034	PSS	Phosphatidy	GO:0009556|	GO:0005789|	GO:0003882|CI	.
Manes.06G06390(	0.1618223	1.34E-15	down	yes	.	pfam13456	RVT_3	Reverse trar	.	.	.	.
Manes.06G06400(	1.6321105	0.01568	up	no	Putative zinc	pfam00643	zf-B_box	B-box zinc f	GO:0006355|	GO:0005634|	GO:0003700|tra	.
Manes.06G06420(	Inf	0.35135	up	no	Two-compor	pfam00072	Response_reg	Response re	GO:0071368|	GO:0005634|	GO:0003677|DI	K14491
Manes.06G06450(	2.256979	5.03E-18	up	yes	.	pfam00564	PB1	PB1 domain	.	.	.	.
Manes.06G06460(	1.2640557	0.01483	up	no	Probable inac	pfam00069	Pkinase	Protein kina	.	GO:0016021|	GO:0005524|A'	.
Manes.06G06490(	1.0155819	0.90217	up	no	.	pfam01467	CTP_transf_2	Cytidylyltra	.	.	.	.
Manes.06G06540(	5.3405503	3.68E-78	up	yes	Probable seri	pfam00069	Pkinase	Protein kina	GO:0071215|	GO:0005886|	GO:0005524|A'	.
Manes.06G06550(	2.6216634	6.15E-15	up	yes	Probable seri	pfam07714	Pkinase_Tyr	Protein tyro	GO:0071215|	GO:0005886|	GO:0005524|A'	.
Manes.06G06560(	0.6178304	0.04967	down	no	.	pfam14009	DUF4228	Domain of u	.	.	.	.
Manes.06G06570(	0.6404798	2.83E-05	down	no	Probable prot	pfam00481	PP2C	Protein phos	GO:0006470|	.	GO:0046872|m	.
Manes.06G06600(	0.389267	0.03371	down	no	Zinc finger pr	pfam13894	zf-C2H2_4	C2H2-type	GO:0009736|	GO:0022626|	GO:0046872|m	.
Manes.06G06630(	0.8068995	0.19604	down	no	.	pfam07797	DUF1639	Protein of u	.	.	.	.
Manes.06G06650(	1.0727246	0.4373	up	no	Glutamine--tl	pfam00749	tRNA-synt_1c	tRNA synth	GO:0006425|	GO:0005737|	GO:0005524|A'	.
Manes.06G06690(	32.489235	4.64E-32	up	yes	.	pfam01673	Herpes_env	Herpesvirus	.	.	.	.
Manes.06G06730(	0.9280064	0.62408	down	no	.	pfam03398	Ist1	Regulator o	.	.	.	.
Manes.06G06760(	3.5748168	1.41E-45	up	yes	.	pfam06487	SAP18	Sin3 associa	.	.	.	.
Manes.06G06770(	14.348167	2.50E-08	up	yes	.	pfam00073	Rhv	picornavirus	.	.	.	.
Manes.06G06800(	0.9077373	0.3228	down	no	Small RNA 2	pfam13847	Methyltransf_31	Methyltrans	GO:0010589|	GO:0005737|	GO:0046872|m	.
Manes.06G06860(	3.1575756	9.96E-14	up	yes	Zinc finger pr	pfam06203	CCT	CCT motif.	GO:0006355|	GO:0016020|	GO:0003700|tra	.
Manes.06G06870(	0.4157168	1.43E-06	down	yes	Homocystein	pfam02574	S-methyl_trans	Homocystei	GO:0009086|	GO:0005737|	GO:0047150|be	.
Manes.06G06880(	0.5416462	0.00251	down	no	Tetraketide a	pfam01370	Epimerase	NAD depen	GO:0010584|	GO:0005829|	GO:0050662|co	.
Manes.06G06890(	1.9423194	2.27E-13	up	no	Ethylene-resp	pfam00847	AP2	AP2 domain	GO:0009873|	GO:0005634|	GO:0003677|DI	.
Manes.06G06900(	1.8518625	1.41E-06	up	no	.	pfam13261	DUF4052	Protein of u	.	.	.	.
Manes.06G06910(	3.1233248	1.15E-37	up	yes	Alpha-xylosic	pfam01055	Glyco_hydro_31	Glycosyl hy	GO:0071555|	GO:0048046|	GO:0061634|al	K15925
Manes.06G06920(	2.7287027	2.32E-24	up	yes	Alpha-xylosic	pfam01055	Glyco_hydro_31	Glycosyl hy	GO:0071555|	GO:0048046|	GO:0061634|al	K15925
Manes.06G06930(	1.5843433	3.29E-07	up	no	.	pfam01265	Cyto_heme_lyase	Cytochrome	.	.	.	.
Manes.06G06940(	3.2795685	5.92E-42	up	yes	Ubiquitin rec	pfam09280	XPC-binding	XPC-binding	GO:0006289|	GO:0005737|	GO:0003684|da	.
Manes.06G06950(	10.474118	1.06E-07	up	yes	Protein NRT	pfam00854	PTR2	POT family.	GO:0042128|	GO:0016021|	GO:0005215|tra	.
Manes.06G06960(	0.3896775	3.10E-09	down	yes	Protein NRT	pfam00854	PTR2	POT family.	GO:0042128|	GO:0016021|	GO:0005215|tra	.
Manes.06G07000(	1.3931311	0.1532	up	no	.	pfam14897	EpsG	EpsG family	.	.	.	.
Manes.06G07020(	1.3288001	0.03439	up	no	Serine/threon	pfam00069	Pkinase	Protein kina	GO:0006397|	GO:0071013|	GO:0005524|A'	K08827
Manes.06G07040(	0.6272918	0.002	down	no	30S ribosoma	pfam04839	PSRP-3_Ycf65	Plastid and	GO:0006412|	GO:0009507|	GO:0019843|rR	.
Manes.06G07090(	0.5108295	0.00398	down	no	Transcription	pfam14144	DOG1	Seed dorma	GO:0009909|	GO:0005634|	GO:0003677|DI	K14431
Manes.06G07140(	0.7653738	0.01837	down	no	BRO1 domai	pfam03097	BRO1	BRO1-like c	.	GO:0070062|	.	.
Manes.06G07150(	6.3265023	4.03E-88	up	yes	Transcription	pfam00249	Myb_DNA-binding	Myb-like DI	GO:0007623|	GO:0005634|	GO:0003677|DI	.
Manes.06G07170(	26.253164	#######	up	yes	AP2/ERF and	pfam02362	B3	B3 DNA bir	GO:0009873|	GO:0005634|	GO:0003677|DI	K09287
Manes.06G07190(	1.4675652	0.0005	up	no	Probable gala	pfam01501	Glyco_transf_8	Glycosyl tra	GO:0071555|	GO:0005794|	GO:0047262|pc	.
Manes.06G07210(	0.4157819	0.00076	down	yes	.	pfam09468	RNase_H2-Ydr27!	Ydr279p pro	.	.	.	.
Manes.06G07220(	7.7059096	4.54E-24	up	yes	.	pfam08137	DVL	DVL family	.	.	.	.
Manes.06G07230(	0.7965836	0.08215	down	no	NAC domain	pfam02365	NAM	No apical m	GO:0000077|	GO:0005634|	GO:0003677|DI	.
Manes.06G07270(	2.2734447	1.71E-11	up	yes	Aspartic prot	pfam14541	TAXi_C	Xylanase inl	.	GO:0005576|	GO:0004190|as	.
Manes.06G07280(	0.4596832	0.00214	down	yes	Transcription	pfam03634	TCP	TCP family	GO:0007275|	GO:0005634|	GO:0003677|DI	.
Manes.06G07290(	0.5153675	0.0018	down	no	Coiled-coil d	pfam04502	DUF572	Family of ur	.	.	.	K13115
Manes.06G07310(	0.6317817	0.00456	down	no	Uncharacteriz	pfam02374	ArsA_ATPase	Anion-trans	.	GO:0009507|	GO:0005524|A'	.
Manes.06G07320(	0.2241516	6.71E-28	down	yes	Protein CHU	pfam12325	TMF_TATA_bd	TATA elem	GO:0009902|	GO:0009507|	.	.
Manes.06G07330(	0.8396406	0.16131	down	no	.	pfam15558	DUF4659	Domain of u	.	.	.	.
Manes.06G07350(	0.5584247	7.23E-05	down	no	Probable tran	pfam03188	Cytochrom_B561	Eukaryotic	GO:0055114|	GO:0016021|	GO:0046872|m	K08360
Manes.06G07390(	0.8934399	0.25292	down	no	Protein decap	pfam12701	LSM14	Scd6-like Sr	GO:0033962|	GO:0000932|	GO:0042803|pr	K18749
Manes.06G07400(	0.8136273	0.04112	down	no	Protein CRO	pfam02463	SMC_N	RecF/RecN/	GO:0006997|	GO:0009507|	.	K00826
Manes.06G07530(	0.6357677	9.27E-05	down	no	Probable isop	pfam00135	COesterase	Carboxylest	.	GO:0005789|	GO:0052689|ca	.
Manes.06G07550(	0.3748019	0.00791	down	yes	.	pfam05180	zf-DNL	DNL zinc fi	.	.	.	.
Manes.06G07560(	3.8792341	0.0071	up	yes	Lysine-rich a	pfam09641	DUF2026	Protein of u	.	GO:0031225|	.	.
Manes.06G07570(	0.414968	8.04E-17	down	yes	tRNA(adenin	pfam00383	dCMP_cyt_deam_	Cytidine and	GO:0002100|	GO:0009507|	GO:0008251|tR	K11991
Manes.06G07580(	5.6786431	2.57E-10	up	yes	Probable calc	pfam13499	EF-hand_7	EF-hand do	.	.	GO:0005509|ca	K13448
Manes.06G07590(	6.0423284	7.44E-05	up	yes	.	pfam07314	DUF1461	Protein of u	.	.	.	.
Manes.06G07610(	1.5178646	6.83E-05	up	no	HD domain-c	pfam13023	HD_3	HD domain.	.	GO:0005829|	.	K07023
Manes.06G07660(	2.4021506	6.73E-06	up	yes	.	pfam05340	DUF740	Protein of u	.	.	.	.
Manes.06G07680(	0.556797	2.12E-05	down	no	Strictosidine	pfam03088	Str_synth	Strictosidine	GO:0009820|	GO:0005773|	GO:0016844|str	.
Manes.06G07720(	0.4210445	4.82E-07	down	yes	.	pfam00646	F-box	F-box doma	.	.	.	.
Manes.06G07750(	0.964009	0.80199	down	no	Uridine-cytid	pfam00485	PRK	Phosphoribu	GO:0044211|	.	GO:0005524|A'	.
Manes.06G07760(	Inf	8.73E-08	up	yes	Peroxidase 1	pfam00141	peroxidase	Peroxidase.	GO:0042744|	GO:0005576|	GO:0020037|he	K00430
Manes.06G07780(	0.9718031	0.83342	down	no	ASC1-like pr	pfam03798	TRAM_LAG1_CI	TLC domain	GO:0006629|	GO:0005789|	.	.
Manes.06G07800(	0.42022	0.00023	down	yes	Basic leucine	pfam00170	bZIP_1	bZIP transc	GO:0006351|	GO:0005634|	GO:0043565|se	.
Manes.06G07820(	0.8072585	0.09617	down	no	WPP domain	pfam05557	MAD	Mitotic chec	.	GO:0016021|	.	.
Manes.06G07900(	0.5460004	9.28E-08	down	no	Transcription	pfam00010	HLH	Helix-loop-l	GO:0006351|	GO:0005634|	GO:0003677|DI	.
Manes.06G07910(	7.5066224	0.07878	up	no	.	pfam03856	SUN	Beta-glucos	.	.	.	.
Manes.06G07930(	1.4692251	0.00029	up	no	.	pfam03909	BSD	BSD domain	.	.	.	.
Manes.06G07940(	10.108836	3.01E-31	up	yes	.	pfam03770	IPK	Inositol poly	.	.	.	.
Manes.06G07970(	34.715158	3.04E-18	up	yes	Basic leucine	pfam00170	bZIP_1	bZIP transc	GO:0006351|	GO:0005634|	GO:0043565|se	.
Manes.06G07990(	1.9799069	2.42E-14	up	no	E3 ubiquitin-	pfam13639	zf-RING_2	Ring finger	GO:0016567|	.	GO:0016874|lig	.
Manes.06G08040(	0.7595394	0.00612	down	no	Uncharacteriz	pfam10250	O-FucT	GDP-fucose	.	GO:0005768|	GO:0016757|tra	.
Manes.06G08050(	6.4538636	6.97E-82	up	yes	.	pfam00536	SAM_1	SAM domai	.	.	.	.
Manes.06G08060(	3.0378598	1.27E-29	up	yes	Dof zinc fing	pfam02701	zf-Dof	Dof domain	GO:0006355|	GO:0005634|	GO:0046872|m	.
Manes.06G08080(	0.7519659	0.01548	down	no	.	pfam12128	DUF3584	Protein of u	.	.	.	.
Manes.06G08120(	0.4787751	4.22E-06	down	yes	Putative ATP	pfam00270	DEAD	DEAD/DEA	GO:0032508|	GO:0005634|	GO:0005524|A'	K06877
Manes.06G08160(	1.2886903	0.00734	up	no	ADP,ATP ca	pfam00153	Mito_carr	Mitochondr	GO:0051503|	GO:0005789|	GO:0005471|A'	K05863
Manes.06G08210(	3.3323686	1.54E-31	up	yes	UDP-arabino	pfam03214	RGP	Reversibly g	GO:0071555|	GO:0005794|	GO:0016866|in	K13379
Manes.06G08250(	1.2379622	0.02194	up	no	Protein COB	pfam04833	COBRA	COBRA-lik	GO:0010215|	GO:0031225|	.	.
Manes.06G08280(	1.6026919	1.62E-07	up	no	RING finger	pfam13418	Kelch_4	Galactose o	.	GO:0005737|	GO:0008270|zi	.
Manes.06G08330(	1.2562458	0.01764	up	no	Protein LON	pfam14309	DUF4378	Domain of u	GO:0051513|	GO:0005737|	GO:0008017|mi	.
Manes.06G08370(	0.6511264	9.84E-06	down	no	.	pfam06549	DUF1118	Protein of u	.	.	.	.
Manes.06G08380(	3.8503746	0.39483	up	no	Putative ribo	pfam13456	RVT_3	Reverse trar	.	.	GO:0046872|m	.
Manes.06G08410(	1.7987912	3.31E-08	up	no	Glycerophos	pfam03009	GDPD	Glycerophos	GO:0006071|	GO:0005618|	GO:0008889|gl	K01126
Manes.06G08440(	2.2832925	1.61E-18	up	yes	Exocyst com	pfam03081	Exo70	Exo70 exoc	GO:0006887|	GO:0005829|	.	.
Manes.06G08560(	0.85572	0.11036	down	no	Chaperone pr	pfam07724	AAA_2	AAA domai	GO:0045727|	GO:0009941|	GO:0005524|A'	K03695

Manes.06G08590(	0.5981036	3.14E-07	down	no	.	pfam09606	Med15	ARC105 or	.	.	.	.
Manes.06G08600(	1.9216969	5.44E-07	up	no	.	pfam13042	DUF3902	Protein of u	.	.	.	.
Manes.06G08620(	16.576049	0.04597	up	no	UDP-glycosy	pfam00201	UDPGT	UDP-glucor	.	.	GO:0016758|tra	.
Manes.06G08640(	8.6666864	1.41E-07	up	yes	UDP-glycosy	pfam00201	UDPGT	UDP-glucor	.	.	GO:0016758|tra	.
Manes.06G08670(	1.451023	0.09082	up	no	Diacylglycerc	pfam00609	DAGK_acc	Diacylglycer	GO:0006952|	GO:0016021|	GO:0005524|A	K00901
Manes.06G08690(	9.3096268	2.37E-12	up	yes	UDP-glucose	pfam03721	UDPG_MGDP_dl	UDP-glucos	GO:0006065|	.	GO:0051287|N.	K00012
Manes.06G08700(	1.190148	0.12098	up	no	Plant intracel	pfam13855	LRR_8	Leucine rich	.	GO:0005886|	.	.
Manes.06G08730(	3.5760872	9.84E-21	up	yes	SNAP25 hon	pfam05739	SNARE	SNARE dor	GO:0000911|	GO:0009504|	GO:0005484|SN	.
Manes.06G08750(	0.8262383	0.0771	down	no	Heparanase-l	pfam03662	Glyco_hydro_79n	Glycosyl hy	GO:0009826|	GO:0005618|	GO:0004566|be	.
Manes.06G08760(	0.9173983	0.3781	down	no	S phase cycli	pfam13868	Trichoplein	tumor suppr	.	GO:0005737|	GO:0046872|m	.
Manes.06G08780(	0.7559441	0.00697	down	no	DNA anneali	pfam00176	SNF2_N	SNF2 family	GO:0006974|	GO:0043596|	GO:0036310|an	.
Manes.06G08790(	Inf	0.08663	up	no	.	pfam02250	Orthopox_35kD	35kD major	.	.	.	.
Manes.06G08830(	4.9206788	2.59E-64	up	yes	Flavin-contai	pfam05904	DUF863	Plant protei	.	.	GO:0050660|fla	.
Manes.06G08840(	0.5426935	0.00055	down	no	dCTP pyroph	pfam03819	MazG	MazG nucle	GO:0009143|	GO:0005829|	GO:0047840|dC	K16904
Manes.06G08870(	3.0789238	2.03E-14	up	yes	.	pfam01844	HNH	HNH endon	.	.	.	.
Manes.06G08900(	0.3360931	0.00056	down	yes	.	pfam12805	FUSC-like	FUSC-like i	.	.	.	.
Manes.06G08930(	0.8895315	0.22289	down	no	LON peptida	pfam02190	LON	ATP-depen	.	.	GO:0004176|A	.
Manes.06G08940(	3.2903516	1.59E-13	up	yes	Probable rece	pfam12819	Malectin_like	Carbohydra	GO:0097275|	GO:0016021|	GO:0005524|A	.
Manes.06G08950(	Inf	0.02355	up	yes	.	pfam08001	CMV_US	CMV US. T	.	.	.	.
Manes.06G08960(	1.7334536	8.22E-09	up	no	Putative glyc	pfam05637	Glyco_transf_34	galactosyl tr	GO:0010411|	GO:0000139|	GO:0016757|tra	K08238
Manes.06G08990(	43.682664	1.00E-05	up	yes	Protein NRT	pfam00854	PTR2	POT family.	GO:0009860|	GO:0016021|	GO:0005215|tra	.
Manes.06G09050(	6.1175165	1.96E-08	up	yes	NAC domain	pfam02365	NAM	No apical m	GO:0006351|	GO:0005634|	GO:0003677|D	.
Manes.06G09060(	0.1246167	5.49E-05	down	yes	.	pfam13041	PPR_2	PPR repeat	.	.	.	.
Manes.06G09080(	Inf	0.02254	up	yes	.	pfam11222	DUF3017	Protein of u	.	.	.	.
Manes.06G09110(	3.9046511	5.90E-14	up	yes	Thioredoxin-	pfam00085	Thioredoxin	Thioredoxin	GO:0045454|	GO:0009507|	GO:0016671|ox	.
Manes.06G09120(	2.092935	0.11367	up	no	.	pfam02516	STT3	Oligosaccha	.	.	.	.
Manes.06G09140(	0.3026912	1.98E-14	down	yes	Chlorophyll a	pfam00504	Chloroa_b-bind	Chlorophyll	GO:0009765|	GO:0009535|	GO:0016168|ch	.
Manes.06G09150(	0.5972034	4.48E-05	down	no	Floral homeo	pfam00319	SRF-TF	SRF-type tr	GO:0006351|	GO:0005634|	GO:0003677|D	.
Manes.06G09160(	1.95418	1.75E-13	up	no	Cyclic dof fa	pfam02701	zf-Dof	Dof domain	GO:0009908|	GO:0005634|	GO:0003677|D	.
Manes.06G09220(	2.1232289	8.31E-17	up	yes	Probable E3	pfam12428	DUF3675	Protein of u	GO:0042335|	GO:0016021|	GO:0016874|lig	K10661
Manes.06G09250(	0.329706	0.02573	down	yes	Mini zinc fing	pfam04770	ZF-HD_dimer	ZF-HD prot	GO:0007275|	GO:0005737|	GO:0003677|D	.
Manes.06G09260(	1.6154069	0.00054	up	no	Myb-related	pfam00249	Myb_DNA-binding	Myb-like D	GO:0006355|	GO:0005634|	GO:0003677|D	.
Manes.06G09290(	0.6767448	0.00176	down	no	.	pfam12697	Abhydrolase_6	Alpha/beta l	.	.	.	.
Manes.06G09310(	1.9024393	9.48E-06	up	no	60S ribosom	pfam01199	Ribosomal_L34e	Ribosomal	GO:0006412|	GO:0005840|	GO:0003735|str	.
Manes.06G09400(	0.6653793	0.00038	down	no	.	pfam12077	DUF3556	Transmemb	.	.	.	.
Manes.06G09460(	0.8501886	0.17415	down	no	RINT1-like p	pfam04437	RINT1_TIP1	RINT-1 / T	GO:0006888|	GO:0005829|	.	.
Manes.06G09500(	0.7946013	0.412	down	no	Aldose 1-epi	pfam01263	Aldose_epim	Aldose 1-ep	GO:0005975|	GO:0005737|	GO:0004034|al	K01785
Manes.06G09510(	1.0352242	0.75981	up	no	ABC transpo	pfam00005	ABC_tran	ABC transp	GO:0006869|	GO:0016021|	GO:0005524|A	.
Manes.06G09530(	0.6619606	2.59E-05	down	no	Protein IQ-D	pfam13178	DUF4005	Protein of u	.	GO:0005829|	.	.
Manes.06G09570(	0.3034694	3.73E-08	down	yes	Synaptotagm	pfam08372	PRT_C	Plant phosp	.	GO:0005783|	GO:0046872|m	.
Manes.06G09580(	0.8767777	0.18201	down	no	Hypersensitiv	pfam01145	Band_7	SPFH doma	.	GO:0005794|	GO:0043424|pr	.
Manes.06G09620(	NA	NA	--	no	.	pfam00646	F-box	F-box doma	.	.	.	.
Manes.06G09640(	0.7529267	0.06476	down	no	.	pfam06549	DUF1118	Protein of u	.	.	.	.
Manes.06G09660(	1.3778749	0.00038	up	no	Homeobox-le	pfam00046	Homeobox	Homeobox	GO:0009965|	GO:0005634|	GO:0042803|pr	K09338
Manes.06G09720(	1.9771928	1.08E-10	up	no	.	pfam13041	PPR_2	PPR repeat	.	.	.	.
Manes.06G09730(	0.8338131	0.07281	down	no	Inactive ubiq	pfam04780	DUF629	Protein of u	GO:0006511|	.	GO:0036459|ub	.
Manes.06G09740(	0.2793825	4.33E-24	down	yes	Uncharacteri	pfam14599	zinc_ribbon_6	Zinc-ribbon.	GO:0016567|	.	GO:0061630|ub	.
Manes.06G09760(	0.6622614	0.00025	down	no	Probable tran	pfam06813	Nodulin-like	Nodulin-like	GO:0055085|	GO:0016021|	.	.
Manes.06G09780(	8.7662254	3.71E-06	up	yes	HIPL1 protei	pfam07995	GSDH	Glucose / S	GO:0005975|	GO:0031225|	GO:0016901|ox	.
Manes.06G09800(	0.544969	0.00118	down	no	Probable mit	pfam14363	AAA_assoc	Domain ass	GO:0051131|	GO:0016021|	GO:0005524|A	K08900
Manes.06G09850(	0.7970679	0.05025	down	no	Transcription	pfam00010	HLH	Helix-loop-l	GO:0006355|	GO:0005634|	GO:0003677|D	.
Manes.06G09900(	0.4103423	1.94E-05	down	yes	.	pfam02108	FliH	Flagellar ass	.	.	.	.
Manes.06G09910(	0.5984568	3.09E-06	down	no	Transcription	pfam00249	Myb_DNA-binding	Myb-like D	GO:0006355|	GO:0005829|	GO:0003677|D	.
Manes.06G09930(	1.0443388	0.68053	up	no	.	pfam12072	DUF3552	Domain of u	.	.	.	.
Manes.06G09950(	0.9898417	0.9361	down	no	Putative gen	pfam14570	zf-RING_4	RING/Ubox	GO:0006402|	GO:0030014|	GO:0000166|nu	K10643
Manes.06G09970(	0.1207095	7.78E-19	down	yes	Two-compor	pfam00072	Response_reg	Response re	GO:0007623|	GO:0005737|	GO:0000156|ph	K14492
Manes.06G09980(	0.9132864	0.40542	down	no	.	pfam12455	Dynactin	Dynein asso	.	.	.	.
Manes.06G10010(	1.1523665	0.17587	up	no	Mannose-1-p	pfam00483	NTP_transferase	Nucleotidyl	GO:0009298|	.	GO:0004475|m	K00966
Manes.06G10030(	2.7060192	1.74E-07	up	yes	.	pfam15558	DUF4659	Domain of u	.	.	.	.
Manes.06G10070(	0.6020771	0.00422	down	no	Ribosomal R	pfam02527	GidB	rRNA small	.	GO:0005737|	GO:0070043|r	K03501
Manes.06G10090(	2.4783724	1.34E-22	up	yes	U-box domai	pfam04564	U-box	U-box doma	GO:0016567|	GO:0005737|	GO:0016874|lig	.
Manes.06G10100(	1.7963986	0.00557	up	no	G-type lectin	pfam00069	Pkinase	Protein kina	GO:0009755|	GO:0016021|	GO:0005524|A	.
Manes.06G10110(	0.5106598	0.00561	down	no	Protein ROS	pfam00730	HhH-GPD	HhH-GPD s	GO:0006284|	GO:0005730|	GO:0051539|4	.
Manes.06G10130(	0.6481665	0.00062	down	no	Probable alp	pfam01532	Glyco_hydro_47	Glycosyl hy	GO:0030968|	GO:0005783|	GO:0004559|al	K10084
Manes.06G10150(	0.5085617	0.00013	down	no	UDP-glycosy	pfam00201	UDPGT	UDP-glucor	GO:0052544|	GO:0043231|	GO:0080043|qu	K11820
Manes.06G10180(	1.6347871	4.84E-05	up	no	.	pfam08307	Glyco_hydro_98C	Glycosyl hy	.	.	.	.
Manes.06G10190(	1.1256675	0.51672	up	no	LIM domain-	pfam00412	LIM	LIM domair	GO:0051017|	GO:0005737|	GO:0051015|ac	.
Manes.06G10200(	1.1792614	0.07253	up	no	.	pfam14009	DUF4228	Domain of u	.	.	.	.
Manes.06G10210(	0.9185282	0.37751	down	no	Nucleobase-a	pfam00860	Xan_ur_permease	Permease fa	GO:0055085|	GO:0005618|	GO:0005215|tra	.
Manes.06G10220(	0.509248	7.87E-07	down	no	Rac-like GTF	pfam00071	Ras	Ras family.	GO:0007264|	GO:0005737|	GO:0005525|G	K04392
Manes.06G10230(	0.1975322	5.73E-08	down	yes	Protease Do-	pfam01843	DIL	DIL domain	.	GO:0009507|	GO:0004252|se	.
Manes.06G10240(	0.1800155	3.23E-25	down	yes	RING-H2 fin	pfam13639	zf-RING_2	Ring finger	GO:0016567|	GO:0016021|	GO:0008270|zi	.
Manes.06G10300(	1.1978084	0.1889	up	no	Probable alp	pfam00982	Glyco_transf_20	Glycosyltrar	GO:0005992|	.	GO:0016757|tra	K16055
Manes.06G10330(	2.812369	9.63E-10	up	yes	U-box domai	pfam04564	U-box	U-box doma	GO:0010029|	.	GO:0016874|lig	.
Manes.06G10340(	0.7673641	0.01454	down	no	.	pfam05160	DSS1_SEM1	DSS1/SEM	.	.	.	.
Manes.06G10360(	Inf	0.00126	up	yes	Bidirectional	pfam03083	MtN3_slv	Sugar efflux	GO:0034219|	GO:0005887|	GO:0051119|su	K15382
Manes.06G10380(	0.2052651	8.10E-07	down	yes	.	pfam00010	HLH	Helix-loop-l	.	.	.	.
Manes.06G10400(	1.2514698	0.07953	up	no	.	pfam00300	His_Phos_1	Histidine ph	.	.	.	.
Manes.06G10410(	0.7102822	0.0245	down	no	E3 ubiquitin-	pfam13639	zf-RING_2	Ring finger	GO:0016567|	GO:0016021|	GO:0016874|lig	K16284
Manes.06G10430(	2.175683	1.34E-10	up	yes	CASP-like pr	pfam04535	DUF588	Domain of u	GO:0055114|	GO:0016021|	GO:0051536|ir	.
Manes.06G10440(	0.4043169	0.00143	down	yes	Serine/threon	pfam07714	Pkinase_Tyr	Protein tyro	.	.	GO:0005524|A	.
Manes.06G10450(	0.8004992	0.02521	down	no	Nephrocystin	pfam13424	TPR_12	Tetratricope	GO:0003283|	GO:0005929|	.	K19360
Manes.06G10490(	10.906687	0.09606	up	no	.	pfam12174	RST	RCD1-SRO	.	.	.	.
Manes.06G10500(	0.7882928	0.0456	down	no	Serine/threon	pfam00454	PI3_PI4_kinase	Phosphatidy	GO:0006974|	GO:0005634|	GO:0005524|A	K04728
Manes.06G10520(	1.1260964	0.25586	up	no	50S ribosom	pfam00542	Ribosomal_L12	Ribosomal	GO:0006412|	GO:0005840|	GO:0003735|str	.
Manes.06G10630(	0.594773	2.10E-06	down	no	Anthranilate	pfam00591	Glycos_transf_3	Glycosyl tra	GO:0000162|	GO:0009507|	GO:0004048|an	K00766
Manes.06G10650(	0.6079646	1.61E-06	down	no	.	pfam13205	Big_5	Bacterial Ig	.	.	.	.
Manes.06G10690(	3.5372252	1.72E-05	up	yes	LRR recepto	pfam13855	LRR_8	Leucine rich	GO:0009793|	GO:0016021|	GO:0005524|A	.

Manes.06G10700(	1.3858162	0.0109	up	no	.	pfam03699	UPF0182	Uncharacter	.	.	.	.
Manes.06G10720(	1.1105642	0.24752	up	no	Protein MOD	pfam07001	BAT2_N	BAT2 N-ter	GO:0016568|(	.	.	.
Manes.06G10760(	0.2638885	4.30E-13	down	yes	FAS-associat	pfam00789	UBX	UBX domai	.	GO:0005783|	.	K18726
Manes.06G10770(	0.6720331	4.95E-05	down	no	.	pfam03080	DUF239	Domain of u	.	.	.	.
Manes.06G10780(	0.5862917	0.03196	down	no	Cell division	pfam00004	AAA	ATPase fam	GO:0007049|	GO:0005886|	GO:0005524|A	K13525
Manes.06G10790(	0.2605653	1.08E-26	down	yes	Probable LRI	pfam00069	Pkinase	Protein kina	.	GO:0016021|	GO:0005524|A	.
Manes.06G10820(	0.3499986	2.79E-10	down	yes	FKBP12-inte	pfam15397	DUF4618	Domain of u	GO:0080009|	GO:0016607|	.	.
Manes.06G10890(	0.2436223	0.00015	down	yes	.	pfam13041	PPR_2	PPR repeat	.	.	.	.
Manes.06G10950(	0.8802865	0.33218	down	no	DEAD-box A	pfam00270	DEAD	DEAD/DEA	GO:0000184|	GO:0005634|	GO:0005524|A	K12823
Manes.06G10960(	0.5686897	2.93E-06	down	no	Ferric reducti	pfam08022	FAD_binding_8	FAD-bindin	GO:0006811|	GO:0016021|	GO:0000293|fe	.
Manes.06G10970(	8.8305707	1.65E-29	up	yes	.	pfam00564	PB1	PB1 domain	.	.	.	.
Manes.06G10980(	0.9999647	1	down	no	C2 and GRA	pfam00168	C2	C2 domain.	.	GO:0016021|	.	.
Manes.06G11010(	1.5832108	6.54E-07	up	no	.	pfam00569	ZZ	Zinc finger,	.	.	.	.
Manes.06G11040(	0.2477136	2.24E-18	down	yes	.	pfam05462	Dicty_CAR	Slime mold	.	.	.	.
Manes.06G11060(	6.9032192	1.03E-44	up	yes	Pentatricoper	pfam13041	PPR_2	PPR repeat	GO:0008380|	GO:0009507|	.	.
Manes.06G11110(	0.8930063	0.33599	down	no	Probable seri	pfam00069	Pkinase	Protein kina	GO:0006468|	.	GO:0005524|A	K08867
Manes.06G11120(	0.2189017	1.96E-08	down	yes	.	pfam05910	DUF868	Plant protei	.	.	.	.
Manes.06G11130(	11.103918	3.63E-10	up	yes	.	pfam01645	Glu_synthase	Conserved r	.	.	.	.
Manes.06G11150(	2.0170049	3.27E-10	up	yes	Manganese-d	pfam00149	Metallophos	Calcineurin-	.	.	GO:0047631|Al	K01517
Manes.06G11170(	Inf	0.00027	up	yes	Cytochrome	pfam00067	p450	Cytochrome	GO:0009835|	GO:0005789|	GO:0020037|he	.
Manes.06G11180(	0.8956721	0.25251	down	no	Serine/threon	pfam00069	Pkinase	Protein kina	GO:0006397|	.	GO:0005524|A	K08287
Manes.06G11200(	1.1410156	0.15357	up	no	31 kDa ribon	pfam00076	RRM_1	RNA recogn	GO:0016553|	GO:0009507|	GO:0000166|nu	.
Manes.06G11230(	0.6279085	0.01433	down	no	.	pfam09415	CENP-X	CENP-S ass	.	.	.	.
Manes.06G11260(	0.7223583	0.00329	down	no	Alpha/beta hy	pfam12695	Abhydrolase_5	Alpha/beta l	.	GO:0005576|	GO:0016787|hy	.
Manes.06G11280(	0.7311334	0.0314	down	no	Protein tipD	pfam08614	ATG16	Autophagy	GO:0000045|	GO:0034274|	GO:0019776|At	K17890
Manes.06G11310(	20.090258	#######	up	yes	BTB/POZ an	pfam00651	BTB	BTB/POZ d	GO:0009553|	GO:0005737|	GO:0005516|ca	.
Manes.06G11320(	0.1973694	1.52E-40	down	yes	Probable pec	pfam00544	Pec_lyase_C	Pectate lyas	GO:0045490|	.	GO:0046872|m	K01728
Manes.06G11340(	0.8782204	0.20771	down	no	Probable Ufn	pfam07910	Peptidase_C78	Peptidase fa	.	.	GO:0008234|cy	.
Manes.06G11350(	0.7459889	0.00335	down	no	Pentatricoper	pfam13041	PPR_2	PPR repeat	.	GO:0009507|	.	.
Manes.06G11360(	1.6286248	6.33E-08	up	no	Programmed	pfam02847	MA3	MA3 domai	GO:0006915|	GO:0005737|	GO:0003723|RI	K16865
Manes.06G11370(	0.8253018	0.05746	down	no	Probable UD	pfam13414	TPR_11	TPR repeat.	GO:0007275|	.	GO:0016757|tra	K09667
Manes.06G11380(	0.4469384	4.08E-09	down	yes	.	pfam08241	Methyltransf_11	Methyltrans	.	.	.	.
Manes.06G11400(	0.5116746	5.40E-06	down	no	DNA-(apurin	pfam06638	Strabismus	Strabismus	GO:0006281|	GO:0009507|	GO:0003906|DI	.
Manes.06G11420(	0.9792854	0.85414	down	no	Zinc finger C	pfam00642	zf-CCCH	Zinc finger	.	GO:0005634|	GO:0003677|DI	.
Manes.06G11430(	0.2521176	0.00075	down	yes	RPM1-intera	pfam05627	AvrRpt-cleavage	Cleavage sit	GO:0010204|	GO:0012505|	.	K13456
Manes.06G11460(	0.5524394	1.21E-07	down	no	GDSL estera	pfam00657	Lipase_GDSL	GDSL-like l	GO:0016042|	GO:0005576|	GO:0016788|hy	.
Manes.06G11510(	0.7862882	0.04998	down	no	Oxygen-inde	pfam04055	Radical_SAM	Radical SAI	GO:0006779|	GO:0005737|	GO:0051537|2	K02495
Manes.06G11520(	1.0608417	0.56354	up	no	Elongator co	pfam04055	Radical_SAM	Radical SAI	GO:0009734|	GO:0005737|	GO:0004402|his	K07739
Manes.06G11530(	0.7932167	0.02566	down	no	Telomere len	pfam10193	Telomere_reg-2	Telomere le	GO:0032006|	GO:0000781|	GO:0032403|pr	K11137
Manes.06G11550(	1.4561754	3.45E-05	up	no	Nucleoside d	pfam00334	NDK	Nucleoside	GO:0006241|	GO:0009507|	GO:0005524|A	.
Manes.06G11660(	16.069311	4.51E-07	up	yes	Lysine histidi	pfam01490	Aa_trans	Transmemb	GO:0043090|	GO:0016021|	GO:0015171|an	.
Manes.06G11680(	4.3149014	1.23E-60	up	yes	Cyclin-depen	pfam00069	Pkinase	Protein kina	.	.	GO:0005524|A	K08818
Manes.06G11710(	NA	NA	--	no	.	pfam06783	UPF0239	Uncharacter	.	.	.	.
Manes.06G11740(	1.4481928	0.00057	up	no	4-coumarate-	pfam00501	AMP-binding	AMP-bindin	GO:0009695|	GO:0005777|	GO:0005524|A	.
Manes.06G11800(	0.7602634	0.00693	down	no	Probable inac	pfam00069	Pkinase	Protein kina	.	GO:0016021|	GO:0005524|A	.
Manes.06G11820(	21.846809	0.00033	up	yes	Calcium-bind	pfam13833	EF-hand_8	EF-hand do	GO:0010193|	GO:0005737|	GO:0005509|ca	K13448
Manes.06G11880(	0.7867463	0.05189	down	no	.	pfam10172	DDA1	Det1 compl	.	.	.	.
Manes.06G11890(	1.7404321	4.26E-09	up	no	SNF1-related	pfam00571	CBS	CBS domai	GO:0005975|	GO:0009505|	GO:0005524|A	.
Manes.06G11920(	0.6548477	0.00024	down	no	Pentatricoper	pfam14432	DYW_deaminase	DYW family	.	GO:0009507|	.	.
Manes.06G11930(	0.8085718	0.05902	down	no	Probable inac	pfam14008	Metallophos_C	Iron/zinc pu	GO:0016311|	GO:0005576|	GO:0003993|ac	.
Manes.06G11940(	0.8305454	0.06726	down	no	Serine/threon	pfam01163	RIO1	RIO1 family	GO:0006468|	GO:0005737|	GO:0005524|A	K07178
Manes.06G11950(	0.4260694	1.58E-11	down	yes	F-box protein	pfam01753	zf-MYND	MYND fing	GO:0016567|	GO:0005634|	.	.
Manes.06G11970(	1.1057872	0.41502	up	no	Deoxycytidyl	pfam00383	dCMP_cyt_deam_	Cytidine and	GO:0009165|	GO:0070062|	GO:0004132|dC	.
Manes.06G11980(	0.359709	2.70E-05	down	yes	.	pfam14346	DUF4398	Domain of u	.	.	.	.
Manes.06G12010(	1.033264	0.74098	up	no	Acetolactate	pfam02776	TPP_enzyme_N	Thiamine py	GO:0009097|	GO:0009507|	GO:0003984|ac	.
Manes.06G12050(	1.184044	0.2655	up	no	Non-specific	pfam04185	Phosphoesterase	Phosphoeste	.	GO:0005576|	GO:0016788|hy	K01114
Manes.06G12090(	0.53004	0.0003	down	no	.	pfam10442	FIST_C	FIST C dom	.	.	.	.
Manes.06G12100(	0.7284401	0.00432	down	no	SUMO-activa	pfam00899	ThiF	ThiF family.	GO:0016925|	GO:0005829|	GO:0019948|SU	K10684
Manes.06G12120(	2.1941003	0.00026	up	yes	Probable WR	pfam03106	WRKY	WRKY DN.	GO:0010055|	GO:0005634|	GO:0001046|co	.
Manes.06G12130(	0.5331609	0.00075	down	no	.	pfam12463	DUF3689	Protein of u	.	.	.	.
Manes.06G12140(	1.6145468	0.00028	up	no	Heavy metal-	pfam00403	HMA	Heavy-meta	GO:0010286|	GO:0031965|	GO:0046872|m	.
Manes.06G12150(	1.420764	0.01077	up	no	HVA22-like	pfam03134	TB2_DP1_HVA2	TB2/DP1, H	GO:0042538|	GO:0016021|	.	K17279
Manes.06G12160(	0.662312	1	down	no	11-beta-hydr	pfam00106	adh_short	short chain	GO:0006694|	GO:0016021|	GO:0070524|11	.
Manes.06G12170(	0.447956	9.71E-13	down	yes	11-beta-hydr	pfam00106	adh_short	short chain	GO:0006694|	GO:0016021|	GO:0070524|11	.
Manes.06G12180(	0.2248659	3.48E-10	down	yes	11-beta-hydr	pfam00106	adh_short	short chain	GO:0006694|	GO:0016021|	GO:0016491|ox	.
Manes.06G12230(	1.2754025	0.01217	up	no	SWI/SNF co	pfam04433	SWIRM	SWIRM do	GO:0006338|	GO:0016514|	GO:0003677|DI	K11649
Manes.06G12260(	Inf	0.02607	up	yes	Indole-3-acet	pfam02519	Auxin_inducible	Auxin respo	GO:0009733|	.	.	.
Manes.06G12280(	0.5947809	4.55E-07	down	no	Glutamate-1-	pfam00202	Aminotran_3	Aminotrans	GO:0015995|	GO:0009507|	GO:0042286|gl	K01845
Manes.06G12290(	1.0536812	0.6861	up	no	Membrane-ar	pfam13881	Rad60-SLD_2	Ubiquitin-2	.	GO:0005886|	.	.
Manes.06G12300(	0.6347679	0.002	down	no	RNA pseudo	pfam00849	PseudoU_synth_2	RNA pseud	GO:0001522|	.	GO:0009982|ps	.
Manes.06G12310(	0.6940975	0.02698	down	no	RNA pseudo	pfam10328	7TM_GPCR_Srx	Serpentine t	GO:0001522|	.	GO:0009982|ps	.
Manes.06G12370(	1.2474094	0.02049	up	no	Probable prot	pfam01529	zf-DHHC	DHHC palm	.	GO:0016021|	GO:0019706|pr	.
Manes.06G12380(	1.095816	0.50183	up	no	Mitochondria	pfam02953	zf-Tim10_DDP	Tim10/DDP	GO:0015031|	GO:0005758|	GO:0046872|m	K17780
Manes.06G12400(	0.3943206	4.81E-17	down	yes	Probable seri	pfam00069	Pkinase	Protein kina	.	.	GO:0005524|A	.
Manes.06G12420(	0.4131675	1.46E-08	down	yes	.	pfam12551	PHBC_N	Poly-beta-h	.	.	.	.
Manes.06G12430(	0.6901271	0.00201	down	no	Ubiquitin-co	pfam00179	UQ_con	Ubiquitin-co	GO:0016567|	.	GO:0005524|A	.
Manes.06G12440(	0.5093259	3.45E-08	down	no	Protein FAM	pfam13889	Chromosome_seg	Chromosom	.	GO:0005634|	.	.
Manes.06G12480(	0.6793093	0.00103	down	no	.	pfam00514	Arm	Armadillo/b	.	.	.	.
Manes.06G12490(	0.8315982	0.06154	down	no	Long chain b	pfam00155	Aminotran_1_2	Aminotrans	GO:0009640|	GO:0005783|	GO:0030170|py	K00654
Manes.06G12500(	0.2696089	2.59E-13	down	yes	Protein bicau	pfam00536	SAM_1	SAM domai	GO:0007275|	GO:0005737|	GO:0044822|po	K18756
Manes.06G12550(	0.8694735	0.18606	down	no	.	pfam13514	AAA_27	AAA domai	.	.	.	.
Manes.06G12560(	0.7094086	0.00254	down	no	Serine carbo	pfam00450	Peptidase_S10	Serine carbo	.	GO:0005576|	GO:0004185|se	K16297
Manes.06G12570(	0.4818626	5.24E-10	down	yes	Dual specific	pfam09192	Act-Frag_cataly	Actin-fragm	GO:0009738|	GO:0005737|	GO:0004721|ph	.
Manes.06G12580(	0.8023749	0.0567	down	no	Chaperone p	pfam07724	AAA_2	AAA domai	GO:0019538|	GO:0009507|	GO:0005524|A	.
Manes.06G12600(	1.6024092	5.61E-06	up	no	Auxin respon	pfam06507	Auxin_resp	Auxin respo	GO:0009734|	GO:0005634|	GO:0043565|se	.
Manes.06G12610(	3.2214997	1.14E-14	up	yes	ADP-ribosyla	pfam01412	ArfGap	Putative GT	GO:0006810|	GO:0005768|	GO:0005096|G	K12489
Manes.06G12630(	0.7068502	0.00068	down	no	Alpha-manno	pfam01074	Glyco_hydro_38	Glycosyl hy	GO:0006013|	GO:0000326|	GO:0004559|al	.
Manes.06G12640(	0.7452566	0.0035	down	no	Magnesium p	pfam07109	Mg-por_mtran_C	Magnesium-	GO:0015995|	GO:0009507|	GO:0046406|m	K03428

Manes.06G12660(	3.648047	2.00E-05	up	yes	.	pfam01062	Bestrophin	Bestrophin,	.	.	.	.
Manes.06G12690(	1.652362	5.12E-07	up	no	Protein BON	pfam07002	Copine	Copine. Thi	GO:0006952|	GO:0005886|	GO:0005544|ca	.
Manes.06G12720(	0.88622	0.29187	down	no	Probable beta	pfam03016	Exostosin	Exostosin fa	GO:0042546|	GO:0005768|	GO:0047517|1,4	.
Manes.06G12750(	0.6709612	0.00024	down	no	Uncharacteri	pfam06484	Ten_N	Teneurin Int	.	GO:0005773|	.	.
Manes.06G12770(	0.7779258	0.04013	down	no	.	pfam07712	SURNod19	Stress up-re	.	.	.	.
Manes.06G12800(	0.2613214	8.04E-05	down	yes	.	pfam13041	PPR_2	PPR repeat	.	.	.	.
Manes.06G12830(	1.5349037	1.62E-06	up	no	Staphylococc	pfam00567	TUDOR	Tudor doma	GO:0001649|	GO:0097433|	GO:0004518|nu	K15979
Manes.06G12840(	0.7339046	0.05266	down	no	Inositol polyp	pfam03770	IPK	Inositol poly	GO:0009793|	GO:0005634|	GO:0005524|A'	K00915
Manes.06G12900(	Inf	0.1623	up	no	Peroxidase 6(	pfam00141	peroxidase	Peroxidase.	GO:0042744|	GO:0005576|	GO:0020037|he	K00430
Manes.06G12960(	0.7026517	0.00354	down	no	Mediator of I	pfam05669	Med31	SOH1. The	GO:0006351|	GO:0016592|	GO:0001104|RI	K15153
Manes.06G13000(	0.8342243	0.18642	down	no	U6 snRNA pl	pfam09749	HVSL	Uncharacter	GO:0006397|	GO:0005634|	GO:0000175|3'-	.
Manes.06G13010(	0.9495709	0.58632	down	no	.	pfam14951	DUF4503	Domain of u	.	.	.	.
Manes.06G13040(	1.9798171	1.04E-14	up	no	Peptide meth	pfam01625	PMSR	Peptide met	GO:0030091|	.	GO:0008113|pe	.
Manes.06G13070(	1.2063816	0.03993	up	no	GTP-binding	pfam00071	Ras	Ras family.	GO:0015031|	GO:0005622|	GO:0005525|G'	K07874
Manes.06G13080(	0.6650227	0.00013	down	no	Polycomb gro	pfam09733	VEFS-Box	VEFS-Box	GO:0030154|	GO:0005634|	GO:0003677|DI	.
Manes.06G13100(	NA	NA	--	no	Oleosin 1	pfam01277	Oleosin	Oleosin.	.	GO:0016021|	.	.
Manes.06G13110(	4.8634444	6.71E-05	up	yes	Ethylene-resp	pfam00847	AP2	AP2 domain	GO:0006952|	GO:0005634|	GO:0003677|DI	.
Manes.06G13120(	7.1402859	8.28E-26	up	yes	Ethylene-resp	pfam00847	AP2	AP2 domain	GO:0009873|	GO:0005634|	GO:0003677|DI	.
Manes.06G13130(	31.908479	4.90E-07	up	yes	Ethylene-resp	pfam00847	AP2	AP2 domain	GO:0009873|	GO:0005634|	GO:0003677|DI	.
Manes.06G13190(	0.3403021	4.91E-13	down	yes	Putative kina	pfam00069	Pkinase	Protein kina	GO:0009755|	GO:0016021|	GO:0005524|A'	.
Manes.06G13200(	1.5706224	5.56E-06	up	no	Acid phospha	pfam03767	Acid_phosphat_B	HAD superf	.	.	GO:0003993|ac	.
Manes.06G13250(	0.7854664	0.01762	down	no	Uncharacteri	pfam00620	RhoGAP	RhoGAP do	GO:0007165|	.	GO:0005096|G'	.
Manes.06G13260(	17.269397	2.40E-23	up	yes	Hexose carrie	pfam00083	Sugar_tr	Sugar (and	GO:0008643|	GO:0016021|	GO:0022891|su	.
Manes.06G13270(	7.7501018	1.63E-17	up	yes	Hexose carrie	pfam00083	Sugar_tr	Sugar (and	GO:0008643|	GO:0016021|	GO:0022891|su	.
Manes.06G13280(	1.184804	0.07223	up	no	Autophagy-re	pfam03986	Autophagy_N	Autophagoc	GO:0000045|	GO:0000153|	GO:0019776|At	K08343
Manes.06G13300(	2.3668454	3.72E-22	up	yes	.	pfam11188	DUF2975	Protein of u	.	.	.	.
Manes.06G13320(	0.5100334	2.08E-06	down	no	Protein RFT1	pfam04506	Rft-1	Rft protein.	GO:0034203|	GO:0005789|	GO:0005319|lip	K06316
Manes.06G13370(	2.2534223	1.46E-19	up	yes	F-box protein	pfam07078	FYTT	Forty-two-tl	.	.	.	K10268
Manes.06G13380(	1.1348542	0.23382	up	no	2-phosphogly	pfam13207	AAA_17	AAA domai	.	.	GO:0005524|A'	K05715
Manes.06G13460(	0.9458021	0.56874	down	no	Conserved ol	pfam10191	COG7	Golgi compl	GO:0000916|	GO:0036063|	.	.
Manes.06G13490(	2.3710483	1.78E-07	up	yes	Putative glyc	pfam07690	MFS_1	Major Facili	GO:0006820|	GO:0016021|	GO:0015315|or	K13783
Manes.06G13510(	1.2376749	0.02176	up	no	E3 ubiquitin	pfam13639	zf-RING_2	Ring finger	GO:0009626|	GO:0016021|	GO:0016874|lig	K10636
Manes.06G13560(	1.6811325	7.18E-06	up	no	Monocopper	pfam00394	Cu-oxidase	Multicopper	.	GO:0031225|	GO:0005507|co	.
Manes.06G13590(	0.5495134	0.73792	down	no	Endoglucana	pfam00759	Glyco_hydro_9	Glycosyl hy	GO:0071555|	GO:0005576|	GO:0008810|ce	.
Manes.06G13610(	0.0704022	8.58E-33	down	yes	21 kDa prote	pfam04043	PMEI	Plant inverta	.	.	GO:0004857|en	.
Manes.06G13670(	1.4608497	4.29E-05	up	no	Protein EXO	pfam04674	Phi_1	Phosphate-i	GO:0080167|	GO:0048046|	.	.
Manes.06G13700(	1.1921137	0.05858	up	no	Chloride con	pfam03517	Voldacs	Regulator o	GO:0006884|	GO:0005829|	.	K05019
Manes.06G13730(	4.4366969	0.00028	up	yes	.	pfam07279	DUF1442	Protein of u	.	.	.	.
Manes.06G13740(	1.1911311	0.10947	up	no	Hypersensitiv	pfam01145	Band_7	SPFH doma	.	GO:0005794|	.	.
Manes.06G13760(	0.3488153	0.00171	down	yes	.	pfam03362	Herpes_UL47	Herpesvirus	.	.	.	.
Manes.06G13810(	1.8666713	3.98E-11	up	no	Protein tricho	pfam13839	PC-Esterase	GDSL/SGN	.	GO:0016021|	.	.
Manes.06G13820(	1.602219	2.53E-07	up	no	Clp protease-	pfam02861	Clp_N	Clp amino te	GO:0019538|	GO:0009507|	GO:0043424|pr	.
Manes.06G13870(	0.8002203	0.10951	down	no	Probable ami	pfam00155	Aminotran_1_2	Aminotransf	GO:0009058|	.	GO:0030170|py	K14270
Manes.06G13880(	1.8489839	0.06441	up	no	Protein TPX2	pfam12214	TPX2_importin	Cell cycle re	GO:0051301|	GO:0005874|	.	K16812
Manes.06G13990(	1.5300866	0.32947	up	no	Probable prot	pfam00481	PP2C	Protein phos	GO:0006470|	.	GO:0046872|me	K14497
Manes.06G14000(	0.3387413	0.68464	down	no	.	pfam14798	Ca_hom_mod	Calcium hor	.	.	.	.
Manes.06G14020(	0.7124678	0.0763	down	no	.	pfam03155	Alg6_Alg8	ALG6, ALG	.	.	.	.
Manes.06G14030(	0.6120412	0.00481	down	no	Glycosyltrans	pfam09258	Glyco_transf_64	Glycosyl tra	GO:0009738|	GO:0005768|	GO:0046872|me	.
Manes.06G14140(	1.7467464	1.39E-06	up	no	Probable fruc	pfam00294	PfkB	pfkB family	GO:0006014|	GO:0005829|	GO:0005524|A'	K00847
Manes.06G14150(	0.7815043	0.01803	down	no	.	pfam14309	DUF4378	Domain of u	.	.	.	.
Manes.06G14160(	0.2360334	2.76E-05	down	yes	MADS-box p	pfam01486	K-box	K-box regio	GO:0009908|	GO:0005737|	GO:0003677|DI	.
Manes.06G14180(	0.500221	0.00055	down	no	Transcription	pfam03634	TCP	TCP family	GO:0031347|	GO:0005634|	GO:0003677|DI	.
Manes.06G14210(	2.6066574	0.02153	up	yes	.	pfam01476	LysM	LysM doma	.	.	.	.
Manes.06G14250(	0.6592619	3.78E-05	down	no	Zinc finger C	pfam00400	WD40	WD domain	.	.	GO:0003677|DI	.
Manes.06G14290(	0.8183634	0.14028	down	no	ABC transpo	pfam00664	ABC_membrane	ABC transp	GO:0055085|	GO:0009507|	GO:0005524|A'	.
Manes.06G14300(	0.5414595	3.58E-09	down	no	Post-GPI atta	pfam04080	Per1	Per1-like. P	GO:0006506|	GO:0000139|	GO:0016788|hy	.
Manes.06G14310(	Inf	1.35E-07	up	yes	Dehydration-	pfam00847	AP2	AP2 domain	GO:0009631|	GO:0005634|	GO:0003677|DI	K09286
Manes.06G14330(	0.6384984	0.00072	down	no	.	pfam01963	TraB	TraB family	.	.	.	.
Manes.06G14350(	1.6754469	2.43E-08	up	no	Serine/argini	pfam00076	RRM_1	RNA recogi	GO:0000398|	GO:0016607|	GO:0000166|nu	K12893
Manes.06G14360(	4.9218891	1.01E-05	up	yes	MATE efflux	pfam01554	MatE	MatE. The I	.	GO:0016021|	GO:0015297|an	K03327
Manes.06G14380(	0.5295914	1.58E-07	down	no	BAG family r	pfam02179	BAG	BAG domai	.	GO:0005739|	.	.
Manes.06G14400(	0.6864061	0.00909	down	no	.	pfam11152	DUF2930	Protein of u	.	.	.	.
Manes.06G14410(	1.4513708	5.11E-05	up	no	Probable tran	pfam01803	LIM_bind	LIM-domain	GO:0030154|	GO:0005634|	.	.
Manes.06G14420(	0.6927448	0.00056	down	no	.	pfam12937	F-box-like	F-box-like. '	.	.	.	.
Manes.06G14460(	1.2330786	0.29386	up	no	Probable (S)-	pfam00067	p450	Cytochrome	.	GO:0005789|	GO:0020037|he	.
Manes.06G14490(	0.9154362	0.39107	down	no	Protein FAR1	pfam03101	FAR1	FAR1 DNA	GO:0006355|	GO:0005634|	GO:0008270|zi	.
Manes.06G14500(	0.522609	1.16E-10	down	no	.	pfam13178	DUF4005	Protein of u	.	.	.	.
Manes.06G14520(	1.1932871	0.1129	up	no	Inactive rhon	pfam01694	Rhomboid	Rhomboid f	GO:0016477|	GO:0005789|	.	.
Manes.06G14550(	0.6457226	0.00012	down	no	Homeobox-le	pfam01852	START	START dor	GO:0043481|	GO:0005634|	GO:0008289|lip	.
Manes.06G14560(	0.9528171	0.65256	down	no	E3 ubiquitin-	pfam12796	Ank_2	Ankyrin rep	GO:0016567|	.	GO:0016874|lig	K19044
Manes.06G14590(	0.460277	4.60E-08	down	yes	ADP-ribosyla	pfam00025	Arf	ADP-ribosy	GO:0007369|	GO:0016020|	GO:0005525|G'	K07952
Manes.06G14610(	3.7541995	0.02429	up	yes	.	pfam04842	DUF639	Plant protei	.	.	.	.
Manes.06G14620(	0.2807835	0.0035	down	yes	.	pfam08320	PIG-X	PIG-X / PB	.	.	.	.
Manes.06G14630(	1.7641374	4.70E-10	up	no	Membrane st	pfam00173	Cyt-b5	Cytochrome	.	GO:0009535|	GO:0020037|he	K17278
Manes.06G14660(	Inf	0.35135	up	no	Transcription	pfam00249	Myb_DNA-binding	Myb-like DI	GO:0010018|	GO:0016607|	GO:0003677|DI	K09422
Manes.06G14680(	1.4329995	0.00037	up	no	Remorin	pfam03763	Remorin_C	Remorin, C-	GO:0007267|	GO:0005886|	GO:0048032|ga	.
Manes.06G14690(	1.5951991	4.36E-07	up	no	Transmembra	pfam03188	Cytochrom_B561	Eukaryotic	GO:0055114|	GO:0009507|	GO:0046872|me	K08360
Manes.06G14720(	3.1515423	6.29E-16	up	yes	Low-tempera	pfam03251	Tymo_45kd_70kd	Tymovirus 4	GO:0009738|	.	.	.
Manes.06G14750(	0.862715	0.26924	down	no	.	pfam00403	HMA	Heavy-meta	.	.	.	.
Manes.06G14760(	0.4171991	6.33E-06	down	yes	O-glucosyltra	pfam05686	Glyco_transf_90	Glycosyl tra	GO:0006486|	GO:0005576|	GO:0016757|tra	K13667
Manes.06G14840(	0.2797321	0.00183	down	yes	.	pfam00131	Metallothio	Metallothior	.	.	.	.
Manes.06G14850(	0.7094792	0.0009	down	no	Tripeptidyl-p	pfam12580	TPPII	Tripeptidyl	GO:0006508|	GO:0009507|	GO:0004177|an	K01280
Manes.06G14860(	0.6711413	5.62E-05	down	no	Tripeptidyl-p	pfam14403	CP_ATPgrasp_2	Circularly pe	GO:0006508|	GO:0009507|	GO:0004177|an	K01280
Manes.06G14870(	0.3949217	9.81E-09	down	yes	.	pfam03283	PAE	Pectinacetyl	.	.	.	.
Manes.06G14900(	1.0072054	0.9216	up	no	Cysteine and	pfam04968	CHORD	CHORD. Cl	GO:0042742|	.	GO:0051879|H:	K13458
Manes.06G14920(	0.9302549	0.45275	down	no	.	pfam13868	Trichoplein	tumor suppr	.	.	.	.
Manes.06G14980(	0.8272014	0.12557	down	no	.	pfam00096	zf-C2H2	Zinc finger,	.	.	.	.
Manes.06G15010(	1.3167907	0.00341	up	no	Short-chain d	pfam00106	adh_short	short chain	GO:0015031|	GO:0009706|	GO:0016491|ox	.

Manes.06G15050(	177.72769	2.57E-39	up	yes	Putative trans	pfam03600	CitMHS	Citrate trans	GO:0098656|a	GO:0005887|	GO:0015446|ar	.
Manes.06G15080(	2.2850843	2.16E-20	up	yes	Uncharacteriz	pfam03154	Atrophin-1	Atrophin-1 f	.	GO:0005886|	.	.
Manes.06G15090(	2.6680547	1.65E-13	up	yes	Sec-independ	pfam02416	MttA_Hcf106	mttA/Hcf10	GO:0045038|p	GO:0009535|	GO:0009977|pr	.
Manes.06G15100(	0.9597228	0.67117	down	no	BAHD acyltr	pfam02458	Transferase	Transferase	GO:0010143|c	GO:0005737|	GO:0016747|tra	.
Manes.06G15120(	0.7259238	0.00624	down	no	Mediator of I	pfam01269	Fibrillarin	Fibrillarin.	GO:0000494|I	GO:0031428|	GO:1990259|his	K14563
Manes.06G15180(	0.5620425	0.0003	down	no	Probable RN	pfam14259	RRM_6	RNA recogr	.	.	GO:0000166|nu	.
Manes.06G15190(	0.6510399	1.40E-05	down	no	Probable rece	pfam12819	Malectin_like	Carbohydrat	.	GO:0016021|	GO:0005524|A	.
Manes.06G15200(	1.8084244	2.21E-07	up	no	DeSI-like prc	pfam05903	Peptidase_C97	PPPDE puta	.	.	GO:0008233|pe	.
Manes.06G15210(	0.5281872	2.01E-06	down	no	.	pfam01198	Ribosomal_L31e	Ribosomal p	.	.	.	.
Manes.06G15220(	1.9929487	4.28E-11	up	no	Beta-carotene	pfam04116	FA_hydroxylase	Fatty acid hy	GO:0016117|c	GO:0031969|	GO:0016787|hy	.
Manes.06G15230(	NA	NA	--	no	Protein SPIR	pfam10327	7TM_GPCR_Sri	Serpentine t	.	GO:0005874|	.	K18635
Manes.06G15250(	0.6930163	0.009	down	no	Glutaminyl-p	pfam05096	Glu_cyclase_2	Glutamine c	GO:0017186|p	GO:0005789|	GO:0016603|glu	.
Manes.06G15260(	0.8217304	0.10644	down	no	Glutaminyl-p	pfam05096	Glu_cyclase_2	Glutamine c	GO:0017186|p	GO:0005789|	GO:0016603|glu	.
Manes.06G15290(	0.8409354	0.07542	down	no	40S ribosoma	pfam03501	S10_plectin	Plectin/S10	.	GO:0005618|	.	K02947
Manes.06G15310(	21.458442	5.11E-39	up	yes	.	pfam00403	HMA	Heavy-meta	.	.	.	.
Manes.06G15360(	5.3308736	5.05E-06	up	yes	Probable WR	pfam03106	WRKY	WRKY DN.	GO:0042742|c	GO:0005634|	GO:0043565|se	.
Manes.06G15380(	0.3985611	0.00551	down	yes	Pentatricopep	pfam13041	PPR_2	PPR repeat	.	GO:0009507|	.	.
Manes.06G15440(	1.3857301	0.00793	up	no	Uncharacteriz	pfam00004	AAA	ATPase fam	GO:0031122|c	GO:0005741|	GO:0005524|A	.
Manes.06G15480(	0.2162352	7.67E-06	down	yes	G-type lectin	pfam00069	Pkinase	Protein kina	GO:0048544|r	GO:0016021|	GO:0005524|A	.
Manes.06G15560(	2.8094809	0.00146	up	yes	Acidic endoc	pfam00704	Glyco_hydro_18	Glycosyl hy	GO:0042631|c	GO:0005576|	GO:0008843|en	K01183
Manes.06G15600(	0.9591373	0.72601	down	no	.	pfam00627	UBA	UBA/TS-N	.	.	.	.
Manes.06G15640(	5.7819049	1.08E-22	up	yes	Ethylene-resp	pfam00847	AP2	AP2 domain	GO:0009736|c	GO:0005737|	GO:0003677|D	.
Manes.06G15650(	0.8346909	0.16618	down	no	Plastid divisio	pfam14643	DUF4455	Domain of u	GO:0010020|c	GO:0009707|	.	.
Manes.06G15670(	Inf	0.38426	up	no	Inorganic pho	pfam00083	Sugar_tr	Sugar (and c	GO:0006817|p	GO:0016021|	GO:0005315|in	K08176
Manes.06G15680(	0.8306164	0.10615	down	no	Mitochondria	pfam00153	Mito_carr	Mitochondr	GO:0006828|n	GO:0016021|	GO:0016530|m	K15119
Manes.06G15700(	0.5903201	0.0005	down	no	.	pfam10325	7TM_GPCR_Srz	Serpentine t	.	.	.	.
Manes.06G15710(	0.5768553	4.68E-05	down	no	Folylpolyglut	pfam08245	Mur_ligase_M	Mur ligase r	GO:0006730|c	.	GO:0005524|A	K11754
Manes.06G15760(	0.6631083	1	down	no	.	pfam01534	Frizzled	Frizzled/Sm	.	.	.	.
Manes.06G15810(	0.7953504	0.03687	down	no	Abscisic acid	pfam10604	Polyketide_cyc2	Polyketide c	GO:0009738|a	GO:0005737|	GO:0010427|ab	K14496
Manes.06G15860(	1.8927616	5.35E-13	up	no	Cyclic nucleo	pfam00027	cNMP_binding	Cyclic nucle	GO:0071805|p	GO:0005887|	GO:0030552|cA	K05391
Manes.06G15870(	1.1142709	0.46649	up	no	Spermine syn	pfam01564	Spermine_synth	Spermine/sp	GO:0006597|s	.	GO:0016768|sp	K00797
Manes.06G15880(	0.9481341	1	down	no	Probable prot	pfam00481	PP2C	Protein pho	GO:0006470|p	.	GO:0046872|m	K14497
Manes.06G15890(	0.4263012	0.00078	down	yes	Glycerophosp	pfam00069	Pkinase	Protein kina	GO:0006968|c	GO:0016021|	GO:0005524|A	.
Manes.06G15910(	5.870301	1.59E-10	up	yes	RING-H2 fin	pfam13639	zf-RING_2	Ring finger	GO:0016567|p	GO:0016021|	GO:0008270|zi	.
Manes.06G15920(	0.6080532	0.00687	down	no	Succinate del	pfam07896	DUF1674	Protein of u	.	GO:0005759|	.	.
Manes.06G15950(	0.3071767	4.87E-06	down	yes	Vesicle transp	pfam04178	Got1	Got1/Sft2-li	GO:0015031|p	GO:0070062|	.	.
Manes.06G15960(	1.5395697	1.40E-06	up	no	Splicing facto	pfam11931	DUF3449	Domain of u	GO:0010467|g	GO:0071013|	GO:0044822|pc	K12827
Manes.06G15970(	0.6927982	0.00713	down	no	Probable plas	pfam00069	Pkinase	Protein kina	.	GO:0009507|	GO:0005524|A	.
Manes.06G15990(	0.6343837	0.04288	down	no	Zinc finger pr	pfam06203	CCT	CCT motif.	GO:0009909|r	GO:0005634|	GO:0003700|tra	.
Manes.06G16000(	0.8934989	0.38604	down	no	Protein BOB	pfam04969	CS	CS domain.	GO:0032502|c	GO:0005737|	.	.
Manes.06G16030(	0.2862684	2.49E-09	down	yes	.	pfam03181	BURP	BURP doma	.	.	.	.
Manes.06G16040(	0.3442411	0.00076	down	yes	Protein BOB	pfam04969	CS	CS domain.	GO:0032502|c	GO:0005737|	.	.
Manes.06G16110(	1.1377735	0.15354	up	no	Nucleosome-	pfam00628	PHD	PHD-finger.	GO:0006325|c	GO:0016589|	GO:0003677|D	K11728
Manes.06G16120(	0.8355055	0.09714	down	no	.	pfam07200	Mod_r	Modifier of	.	.	.	.
Manes.06G16130(	0.3828559	0.00028	down	yes	Ubiquitin-cor	pfam00179	UQ_con	Ubiquitin-cc	GO:0031145|a	GO:0005737|	GO:0005524|A	K06688
Manes.06G16140(	2.6276965	4.86E-14	up	yes	Hexokinase-3	pfam00349	Hexokinase_1	Hexokinase.	GO:0001678|c	GO:0005829|	GO:0005524|A	K00844
Manes.06G16160(	11.231235	7.71E-07	up	yes	.	pfam08243	SPT2	SPT2 chrom	.	.	.	.
Manes.06G16170(	0.8722521	0.16688	down	no	DnaJ homolo	pfam00226	DnaJ	DnaJ domai	.	GO:0005737|	.	K09527
Manes.06G16190(	0.6222975	1.19E-05	down	no	Zinc finger C	pfam14259	RRM_6	RNA recogr	.	.	GO:0003677|D	.
Manes.06G16200(	0.6778083	0.00017	down	no	Zinc finger C	pfam14259	RRM_6	RNA recogr	.	.	GO:0003677|D	.
Manes.06G16230(	0.4214388	0.00095	down	yes	.	pfam13360	PQQ_2	PQQ-like dc	.	.	.	.
Manes.06G16250(	0.8339956	0.08696	down	no	NAD(H) kina	pfam01513	NAD_kinase	ATP-NAD k	GO:0019674|l	.	GO:0005524|A	.
Manes.06G16260(	Inf	0.00209	up	yes	.	pfam04646	DUF604	Protein of u	.	.	.	.
Manes.06G16280(	0.4905046	0.13777	down	no	Phospholipas	pfam01764	Lipase_3	Lipase (clas	GO:0016042|l	GO:0009507|	GO:0047714|ga	.
Manes.06G16350(	0.6005845	0.00017	down	no	Photosystem	pfam00119	ATP-synt_A	ATP syntha	GO:0015979|p	GO:0009535|	.	.
Manes.06G16360(	3.5673322	1.28E-11	up	yes	Sulfite reduct	pfam01077	NIR_SIR	Nitrite and s	GO:0006323|l	GO:0042644|	GO:0051539|4 i	.
Manes.06G16380(	1.7143824	5.27E-09	up	no	Translocatior	pfam03839	Sec62	Translocatic	GO:0006620|p	GO:0005783|	GO:0008565|pr	K12275
Manes.06G16390(	0.8279696	0.16658	down	no	Nuclear trans	pfam02045	CBFB_NFYA	CCAAT-bin	GO:0009793|e	GO:0016602|	GO:0003677|D	K08064
Manes.06G16410(	0.5874461	0.00175	down	no	Heterogeneo	pfam14259	RRM_6	RNA recogr	GO:0006397|r	GO:0005654|	GO:0000166|nu	K12898
Manes.06G16420(	0.9328431	0.52914	down	no	.	pfam02341	RcbX	RbcX protei	.	.	.	.
Manes.06G16440(	0.6073753	2.23E-06	down	no	.	pfam00514	Arm	Armadillo/b	.	.	.	.
Manes.06G16450(	0.7698722	0.06539	down	no	MATE efflux	pfam01554	MatE	MatE. The N	GO:0006811|i	GO:0016021|	GO:0015297|an	.
Manes.06G16460(	1.8809017	1.39E-08	up	no	Dicarboxylate	pfam00939	Na_sulph_symp	Sodium:sulf	GO:0015742|a	GO:0009507|	GO:0015139|alp	.
Manes.06G16480(	2.6072225	0.00049	up	yes	Transcription	pfam00249	Myb_DNA-binding	Myb-like DI	GO:0050832|c	GO:0005634|	GO:0003677|D	K09422
Manes.06G16490(	1.9205791	2.53E-07	up	no	Nucleolar GT	pfam06858	NOG1	Nucleolar G	GO:0042254|r	GO:0016020|	GO:0005525|G	K06943
Manes.06G16500(	0.829244	0.10217	down	no	tRNA-specific	pfam03054	tRNA_Me_trans	tRNA methy	GO:0006400|t	GO:0005737|	GO:0005524|A	K00566
Manes.06G16550(	0.8972051	0.25282	down	no	Xanthine deh	pfam02738	Ald_Xan_dh_C2	Molybdopte	GO:0006145|p	GO:0005829|	GO:0051537|2 i	K00106
Manes.06G16670(	0.4226616	0.00108	down	yes	Serine/threon	pfam00069	Pkinase	Protein kina	.	.	GO:0005524|A	.
Manes.06G16690(	0.8029891	0.1129	down	no	.	pfam04707	PRELI	PRELI-like	.	.	.	.
Manes.06G16710(	0.3898938	6.47E-10	down	yes	Serine/threon	pfam00069	Pkinase	Protein kina	.	GO:0005737|	GO:0005524|A	.
Manes.06G16730(	0.5048823	0.00041	down	no	.	pfam07944	DUF1680	Putative gly	.	.	.	.
Manes.06G16750(	2.5868793	1.14E-11	up	yes	DNA-damage	pfam08263	LRRNT_2	Leucine rich	GO:0006281|l	GO:0009507|	GO:0000166|nu	.
Manes.06G16770(	1.0112546	0.87477	up	no	Transcription	pfam00010	HLH	Helix-loop-l	GO:0048573|p	GO:0005634|	GO:0003677|D	.
Manes.06G16810(	1.3990971	0.01655	up	no	.	pfam08626	TRAPPC9-Trs120	Transport pr	.	.	.	.
Manes.06G16860(	11.021567	7.79E-60	up	yes	Putative lysin	pfam13621	Cupin_8	Cupin-like d	GO:0007623|c	GO:0005737|	GO:0051213|dio	.
Manes.06G16880(	0.8779396	0.24395	down	no	ETHYLENE	pfam04873	EIN3	Ethylene ins	GO:0071281|c	GO:0005634|	GO:0003677|D	K14514
Manes.06G16890(	0.4607837	0.0004	down	yes	Extended syn	pfam08372	PRT_C	Plant phosp	GO:0006897|c	GO:0005783|	GO:0005509|ca	.
Manes.06G16900(	3.9948614	0.01705	up	yes	Beta-glucosic	pfam00232	Glyco_hydro_1	Glycosyl hy	GO:0005975|c	.	GO:0008422|be	.
Manes.06G16910(	2.8695389	7.24E-33	up	yes	.	pfam05296	TAS2R	Mammalian	.	.	.	.
Manes.06G16930(	0.5766293	0.00342	down	no	.	pfam06687	SUR7	SUR7/PalI f	.	.	.	.
Manes.06G16960(	0.196527	3.32E-11	down	yes	.	pfam12695	Abhydrolase_5	Alpha/beta l	.	.	.	.
Manes.06G16970(	0.2999279	1.16E-08	down	yes	.	pfam00439	Bromodomain	Bromodoma	.	.	.	.
Manes.06G16980(	3.6808315	8.22E-17	up	yes	.	pfam10269	Tmemb_185A	Transmembr	.	.	.	.
Manes.06G16990(	0.4498579	0.03669	down	no	.	pfam14712	Snapin_Pallidin	Snapin/Palli	.	.	.	.
Manes.06G17010(	0.1338107	1.93E-09	down	yes	.	pfam14712	Snapin_Pallidin	Snapin/Palli	.	.	.	.
Manes.06G17040(	0.3094908	0.00035	down	yes	.	pfam14712	Snapin_Pallidin	Snapin/Palli	.	.	.	.
Manes.06G17070(	0.2487113	0.00857	down	yes	.	pfam14712	Snapin_Pallidin	Snapin/Palli	.	.	.	.
Manes.06G17090(	NA	NA	--	no	.	pfam09595	Metaviral_G	Metaviral_C	.	.	.	.

Manes.06G17110(	0.3098343	3.83E-08	down	yes	.	pfam14712	Snapin_Pallidin	Snapin/Palli	.	.	.	.
Manes.06G17120(	0.4804781	1.61E-07	down	yes	.	pfam14712	Snapin_Pallidin	Snapin/Palli	.	.	.	.
Manes.06G17130(	0.9711755	0.79061	down	no	Protein PAT1	pfam09770	PAT1	Topoisomer	GO:0033962|	GO:0030014|	GO:0044822|pc	K12617
Manes.06G17140(	2.0531108	8.88E-14	up	yes	Ribosome-bi	pfam06071	YchF-GTPase_C	Protein of u	.	.	GO:0005524|A	K06942
Manes.06G17160(	2.3517313	1.45E-09	up	yes	ATPase famil	pfam12037	DUF3523	Domain of u	GO:0007005|	GO:0016021|	GO:0005524|A	K17681
Manes.06G17180(	0.8721159	0.16073	down	no	Serine/argini	pfam00076	RRM_1	RNA recogn	GO:0006397|	GO:0016607|	GO:0000166|nu	K14325
Manes.06G17200(	0.1097951	6.23E-11	down	yes	Protein Iojap	pfam02410	Oligomerization	Oligomeriza	.	GO:0009507|	.	.
Manes.06G17210(	1.6684769	1.83E-07	up	no	60S ribosoma	pfam01777	Ribosomal_L27e	Ribosomal L	GO:0006412|	GO:0005840|	GO:0003735|stı	.
Manes.06G17250(	0.7820067	0.02122	down	no	KRR1 small s	pfam02739	5_3_exonuc_N	5'-3' exonuc	GO:0006364|	GO:0005730|	GO:0003723|Rl	K06961
Manes.06G17290(	106.85656	2.80E-57	up	yes	Transcription	pfam02365	NAM	No apical m	GO:0009718|	GO:0005634|	GO:0003677|D	.
Manes.06G17300(	0.6828507	0.02729	down	no	Molybdenum	pfam00266	Aminotran_5	Aminotransf	GO:0009688|	GO:0005622|	GO:0008265|M	K15631
Manes.06G17380(	0.631105	0.00035	down	no	Peptidyl-prol	pfam00160	Pro_isomerase	Cyclophilin	GO:0006457|	GO:0005829|	GO:0000166|nu	K12735
Manes.06G17400(	0.7781574	0.00991	down	no	Cyclin-T1-3	pfam00134	Cyclin_N	Cyclin, N-te	GO:0007049|	GO:0005634|	.	K15188
Manes.06G17410(	0.9261618	0.81918	down	no	.	pfam15095	IL33	Interleukin 3	.	.	.	.
Manes.06G17420(	1.1400672	0.35222	up	no	Protein unc-4	pfam13414	TPR_11	TPR repeat.	GO:0030154|	GO:0005794|	.	.
Manes.06G17460(	0.5743196	0.00088	down	no	Probable LRI	pfam11721	Malectin	Di-glucose l	.	GO:0016021|	GO:0005524|A	.
Manes.06G17470(	2.4408969	1.15E-07	up	yes	Probable LRI	pfam11721	Malectin	Di-glucose l	.	GO:0016021|	GO:0005524|A	.
Manes.06G17480(	0.8468661	0.73617	down	no	Auxin-induce	pfam02519	Auxin_inducible	Auxin respo	GO:0009734|	.	.	.
Manes.06G17500(	13.985412	#######	up	yes	.	pfam13840	ACT_7	ACT domain	.	.	.	.
Manes.06G17540(	NA	NA	--	no	.	pfam13009	Phage_Integr_2	Putative pha	.	.	.	.
Manes.06G17600(	0.6263724	0.00027	down	no	Probable seri	pfam07714	Pkinase_Tyr	Protein tyro	.	GO:0016021|	GO:0005524|A	.
Manes.06G17610(	0.6705294	0.00018	down	no	Nuclear trans	pfam00808	CBFD_NFYB_HN	Histone-like	GO:0045893|	GO:0016602|	GO:0043565|se	.
Manes.06G17620(	0.3386287	0.02105	down	yes	.	pfam02322	Cyto_ox_2	Cytochrome	.	.	.	.
Manes.06G17630(	1.0660454	0.47626	up	no	ATP-depende	pfam00004	AAA	ATPase fam	.	GO:0009507|	GO:0005524|A	K03798
Manes.06G17660(	9.7833208	0.03145	up	no	.	pfam11917	DUF3435	Protein of u	.	.	.	.
Manes.06G17670(	2.3257966	3.38E-20	up	yes	Glyceraldehy	pfam02800	Gp_dh_C	Glyceraldeh	GO:0080144|	GO:0009570|	GO:0004365|gl	K00134
Manes.06G17680(	0.4589217	2.94E-12	down	yes	Uncharacteriz	pfam02652	Lactate_perm	L-lactate pe	.	GO:0016021|	.	.
Manes.06G17690(	0.602825	1.20E-05	down	no	Uncharacteriz	pfam13863	DUF4200	Domain of u	.	GO:0016021|	.	.
Manes.06G17710(	0.5884865	4.84E-05	down	no	E3 ubiquitin-	pfam12906	RINGv	RING-varia	GO:0002495|	GO:0030659|	GO:0016874|lig	K10656
Manes.06G17730(	0.7925049	0.02462	down	no	Mediator of I	pfam09606	Med15	ARC105 or	GO:0050832|	GO:0016592|	.	.
Manes.06G17740(	NA	NA	--	no	Protein FAM	pfam09586	YfhO	Bacterial mo	GO:0006915|	GO:0005634|	.	K13120
Manes.06G17750(	1.2289816	0.02326	up	no	V-type proto	pfam00006	ATP-synt_ab	ATP syntha	GO:0015991|	GO:0033180|	GO:0005524|A	.
Manes.06G17800(	0.9391077	0.49824	down	no	Protein PHR	pfam14379	Myb_CC_LHEQL	MYB-CC ty	GO:0006351|	GO:0005634|	GO:0003677|D	.
Manes.06G17810(	1.7883509	5.29E-11	up	no	Beta-amyrin s	pfam13249	Prenyltrans_2	Prenyltransf	GO:0016104|	.	GO:0042300|be	.
Manes.06G17820(	0.8185275	0.17087	down	no	Protein STRU	pfam00069	Pkinase	Protein kina	GO:0009755|	GO:0016021|	GO:0005524|A	.
Manes.06G17870(	3.6085476	1.52E-07	up	yes	U-box domai	pfam00069	Pkinase	Protein kina	.	.	GO:0005524|A	.
Manes.06G17880(	1.4896632	0.33398	up	no	.	pfam15489	CTC1	CST, telome	.	.	.	.
Manes.06G17940(	0.8619575	0.12254	down	no	Helicase sen1	pfam13087	AAA_12	AAA domai	GO:0032508|	GO:0035649|	GO:0005524|A	K10706
Manes.07G00050(	1.4792639	0.00089	up	no	Citrate syntha	pfam00285	Citrate_synt	Citrate synt	GO:0044262|	GO:0005759|	GO:0004108|cit	.
Manes.07G00060(	1.9781664	2.05E-10	up	no	Homeobox-le	pfam02183	HALZ	Homeobox a	GO:0006351|	GO:0005634|	GO:0043565|se	K09338
Manes.07G00120(	1.6149625	1.60E-07	up	no	Probable seri	pfam00069	Pkinase	Protein kina	.	.	GO:0005524|A	K08853
Manes.07G00320(	1.4438321	0.00016	up	no	Clustered mit	pfam13236	CLU	Clustered m	GO:0048312|	GO:0005737|	.	K03255
Manes.07G00350(	Inf	0.9009	up	no	Cytochrome	pfam00067	p450	Cytochrome	.	GO:0016020|	GO:0020037|he	.
Manes.07G00370(	1.1911687	0.19635	up	no	Ras-related p	pfam00071	Ras	Ras family.	GO:0015031|	GO:0005622|	GO:0005525|G	.
Manes.07G00380(	0.5584747	4.01E-06	down	no	Transcription	pfam00010	HLH	Helix-loop-l	GO:0048573|	GO:0005634|	GO:0003677|D	.
Manes.07G00390(	1.1548428	0.11274	up	no	SURP and G-	pfam01805	Surp	Surp modul	GO:0006397|	GO:0005634|	GO:0003723|R	.
Manes.07G00450(	0.9125272	0.355	down	no	Zinc finger A	pfam01428	zf-AN1	AN1-like Zi	.	.	GO:0003677|D	.
Manes.07G00500(	0.8421152	0.07896	down	no	Zinc finger C	pfam13639	zf-RING_2	Ring finger	.	GO:0005634|	GO:0003677|D	.
Manes.07G00520(	15.217348	9.44E-88	up	yes	.	pfam10320	7TM_GPCR_Srsx	Serpentine t	.	.	.	.
Manes.07G00560(	0.681859	0.03737	down	no	.	pfam03723	Hemocyanin_C	Hemocyanin	.	.	.	.
Manes.07G00570(	0.2117247	2.84E-11	down	yes	.	pfam14897	EpsG	EpsG family	.	.	.	.
Manes.07G00630(	3.8424334	0.00116	up	yes	.	pfam06177	QueT	QueT transp	.	.	.	.
Manes.07G00660(	1.0233632	0.91276	up	no	Nuclear trans	pfam02045	CBFB_NFYA	CCAAT-bin	GO:0009793|	GO:0016602|	GO:0003677|D	K08064
Manes.07G00760(	1.8534541	1.13E-05	up	no	.	pfam04862	DUF642	Protein of u	.	.	.	.
Manes.07G00770(	1.1898299	0.09553	up	no	Cytochrome	pfam00173	Cyt-b5	Cytochrome	GO:0010311|	GO:0005829|	GO:0020037|he	.
Manes.07G00780(	1.3473249	0.00154	up	no	Cycloartenol-	pfam08498	Sterol_MT_C	Sterol methy	GO:0009793|	GO:0005783|	GO:0003838|ste	K00559
Manes.07G00850(	1.6884547	0.00186	up	no	Notchless pro	pfam08154	NLE	NLE (NUC	GO:0009553|	GO:0080008|	.	K14855
Manes.07G00860(	0.5068174	3.00E-05	down	no	Tetraketide a	pfam01370	Epimerase	NAD depen	GO:0009555|	GO:0005783|	GO:0050662|co	.
Manes.07G00900(	0.8676043	0.67651	down	no	.	pfam07976	Phe_hydrox_dim	Phenol hydr	.	.	.	.
Manes.07G00920(	0.325726	7.02E-11	down	yes	Cinnamoyl-C	pfam01370	Epimerase	NAD depen	GO:0007623|	GO:0005829|	GO:0016621|cin	.
Manes.07G00930(	0.6351285	0.086	down	no	Tetraketide a	pfam01370	Epimerase	NAD depen	GO:0009555|	GO:0005783|	GO:0050662|co	.
Manes.07G00950(	0.6325828	2.23E-05	down	no	Putative dise	pfam00931	NB-ARC	NB-ARC do	GO:0006952|	.	GO:0043531|A	.
Manes.07G01040(	1.1452085	0.15004	up	no	.	pfam11891	DUF3411	Domain of u	.	.	.	.
Manes.07G01080(	0.9336264	0.5799	down	no	.	pfam12937	F-box-like	F-box-like.	.	.	.	.
Manes.07G01120(	8.7577261	0.04719	up	no	.	pfam02010	REJ	REJ domain	.	.	.	.
Manes.07G01150(	30.186736	#######	up	yes	.	pfam05633	DUF793	Protein of u	.	.	.	.
Manes.07G01200(	0.2288833	1.40E-32	down	yes	Serine/threon	pfam07714	Pkinase_Tyr	Protein tyro	.	GO:0005576|	GO:0005524|A	.
Manes.07G01220(	0.6904295	0.04545	down	no	Structure-spe	pfam01541	GIY-YIG	GIY-YIG ca	GO:0000737|	GO:0030874|	GO:0017108|5'-	.
Manes.07G01260(	2.0568786	3.22E-11	up	yes	.	pfam03168	LEA_2	Late embryo	.	.	.	.
Manes.07G01270(	NA	NA	--	no	Probable WR	pfam03106	WRKY	WRKY DN	GO:0006351|	GO:0005634|	GO:0043565|se	.
Manes.07G01280(	1.9813195	3.94E-10	up	no	Mitogen-acti	pfam00069	Pkinase	Protein kina	GO:0000186|	GO:0005634|	GO:0005524|A	K13414
Manes.07G01310(	2.0302414	5.26E-14	up	yes	.	pfam07800	DUF1644	Protein of u	.	.	.	.
Manes.07G01330(	0.8115372	0.07638	down	no	mRNA turno	pfam00466	Ribosomal_L10	Ribosomal p	GO:0042254|	GO:0005737|	GO:0044822|pc	K14815
Manes.07G01380(	0.6204991	0.00288	down	no	Partner of Y1	pfam09282	Mago-bind	Mago bindi	GO:0000184|	GO:0005737|	.	K14294
Manes.07G01420(	0.4680033	0.00787	down	yes	Plasma memb	pfam02322	Cyto_ox_2	Cytochrome	GO:0006754|	GO:0016021|	GO:0005524|A	.
Manes.07G01470(	0.7986974	0.02744	down	no	.	pfam04842	DUF639	Plant protei	.	.	.	.
Manes.07G01480(	0.540984	0.00403	down	no	Pathogenesis	pfam00314	Thaumatin	Thaumatin f	GO:0031540|	GO:0048046|	.	.
Manes.07G01490(	0.7413971	0.41421	down	no	Dynamin-like	pfam14840	DNA_pol3_delt_C	Processivity	GO:0010020|	GO:0009507|	GO:0005525|G	.
Manes.07G01500(	3.4476724	3.58E-10	up	yes	Elongation o	pfam01151	ELO	GNS1/SUR	GO:0019368|	GO:0005789|	GO:0009922|fa	K10245
Manes.07G01530(	2.702282	0.009	up	yes	Probable LRI	pfam00069	Pkinase	Protein kina	.	GO:0016021|	GO:0005524|A	.
Manes.07G01540(	0.4102215	1.11E-05	down	yes	F-box protei	pfam00646	F-box	F-box doma	.	GO:0009506|	.	.
Manes.07G01560(	2.4973833	3.91E-18	up	yes	.	pfam01793	Glyco_transf_15	Glycolipid 2	.	.	.	.
Manes.07G01570(	0.7063254	0.00487	down	no	Uncharacteriz	pfam15346	ARGLU	Arginine and	.	.	.	K13173
Manes.07G01580(	0.1813185	2.05E-10	down	yes	.	pfam07079	DUF1347	Protein of u	.	.	.	.
Manes.07G01620(	0.7115349	0.0181	down	no	.	pfam05084	GRA6	Granule anti	.	.	.	.
Manes.07G01630(	1.1176221	0.2341	up	no	PsbP-like pro	pfam01789	PsbP	PsbP. This f	GO:0015979|	GO:0009507|	GO:0005509|ca	K02717
Manes.07G01650(	1.1779768	0.09568	up	no	Pentatricopep	pfam13041	PPR_2	PPR repeat	.	GO:0009507|	.	.
Manes.07G01760(	1.5396935	1.16E-06	up	no	Mediator-ass	pfam04504	DUF573	Protein of u	GO:0006355|	GO:0005507|	.	.

Manes.07G01780(	0.3923329	0.00462	down	yes	Probable rece	pfam00069	Pkinase	Protein kina	.	GO:0016021|	GO:0005524|A'	.
Manes.07G01810(	1.5897448	0.0291	up	no	Cytochrome	pfam00067	p450	Cytochrome	GO:0071555|	GO:0005783|	GO:0020037|he	K15402
Manes.07G01820(	5.8087056	0.00055	up	yes	Anthocyanidi	pfam00201	UDPGT	UDP-glucor	GO:0009718|	.	GO:0047213|an	.
Manes.07G01840(	15.715449	#######	up	yes	Anthocyanidi	pfam00201	UDPGT	UDP-glucor	GO:0009718|	.	GO:0047213|an	.
Manes.07G01860(	0.8594128	0.25803	down	no	Anthocyanidi	pfam00201	UDPGT	UDP-glucor	GO:0009718|	.	GO:0047213|an	.
Manes.07G01870(	21.754154	4.32E-67	up	yes	Anthocyanidi	pfam00201	UDPGT	UDP-glucor	GO:0009718|	.	GO:0047213|an	.
Manes.07G01890(	0.4006795	9.85E-08	down	yes	Potassium ch	pfam00520	Ion_trans	Ion transpor	GO:0071805|	GO:0005789|	GO:0042802|id	.
Manes.07G01900(	0.5809825	1.54E-07	down	no	Serine hydro	pfam00464	SHMT	Serine hydro	GO:0006544|	GO:0005737|	GO:0004372|gl	K00600
Manes.07G01920(	1.2464261	0.01582	up	no	T-complex pr	pfam05794	Tcp11	T-complex	.	GO:0005874|	.	.
Manes.07G01950(	0.9263107	0.45436	down	no	Serine/threon	pfam00069	Pkinase	Protein kina	.	.	GO:0005524|A'	.
Manes.07G02030(	1.0319222	0.72485	up	no	Magnesium t	pfam01544	CorA	CorA-like M	GO:0098655|	GO:0016021|	GO:0015095|m	K16075
Manes.07G02180(	Inf	0.17669	up	no	.	pfam02362	B3	B3 DNA bir	.	.	.	.
Manes.07G02230(	2.8415822	3.48E-14	up	yes	Serine/threon	pfam00069	Pkinase	Protein kina	.	.	GO:0005524|A'	.
Manes.07G02260(	0.7139081	0.26096	down	no	.	pfam07429	Glyco_transf_56	4-alpha-L-fu	.	.	.	.
Manes.07G02290(	1.2327295	0.02053	up	no	ATP phospho	pfam01634	HisG	ATP phosph	GO:0000105|	GO:0009507|	GO:0003879|A'	K00765
Manes.07G02310(	24.128578	2.18E-91	up	yes	Protein argor	pfam02171	Piwi	Piwi domain	GO:0042742|	GO:0030529|	GO:0035197|si	K11593
Manes.07G02380(	0.8051101	0.02708	down	no	Bromodomai	pfam03004	Transposase_24	Plant transp	GO:0006974|	GO:0005694|	GO:0003682|ch	K11722
Manes.07G02390(	0.6781174	0.00058	down	no	DEAD-box A	pfam00270	DEAD	DEAD/DEA	GO:0000398|	GO:0005634|	GO:0005524|A'	K12811
Manes.07G02410(	1.0213452	0.82237	up	no	YTH domain	pfam04146	YTH	YT521-B-li	GO:0043488|	GO:0000932|	GO:1990247|N	.
Manes.07G02430(	0.1542696	3.04E-14	down	yes	60S ribosoma	pfam00297	Ribosomal_L3	Ribosomal p	GO:0006412|	GO:0022625|	GO:0003735|str	K02925
Manes.07G02440(	0.3522471	0.00014	down	yes	60S ribosoma	pfam00297	Ribosomal_L3	Ribosomal p	GO:0006412|	GO:0022625|	GO:0003735|str	K02925
Manes.07G02450(	0.3282061	1.35E-07	down	yes	.	pfam03619	Solute_trans_a	Organic solu	.	.	.	.
Manes.07G02460(	0.5297547	1.12E-07	down	no	MACPF dom	pfam01823	MACPF	MAC/Perfo	GO:0009626|	GO:0005886|	.	.
Manes.07G02480(	0.787137	0.01324	down	no	Phosphatidyl	pfam02666	PS_Dcarbxylase	Phosphatidy	GO:0006646|	GO:0009705|	GO:0005509|ca	K01613
Manes.07G02490(	0.511741	8.12E-05	down	no	.	pfam00564	PB1	PB1 domain	.	.	.	.
Manes.07G02500(	0.4419501	1.66E-08	down	yes	.	pfam07543	PGA2	Protein traff	.	.	.	.
Manes.07G02540(	Inf	0.00411	up	yes	.	pfam13962	PGG	Domain of u	.	.	.	.
Manes.07G02550(	0.5676987	2.70E-08	down	no	Shaggy-relat	pfam00069	Pkinase	Protein kina	.	GO:0005886|	GO:0005524|A'	.
Manes.07G02570(	1.1849792	0.07025	up	no	Regulator of	pfam03467	Smg4_UPF3	Smg-4/UPF	GO:0042742|	GO:0005737|	GO:0000166|nu	K14328
Manes.07G02610(	1.646199	8.87E-07	up	no	Ras-related p	pfam00071	Ras	Ras family.	GO:0000911|	GO:0009504|	GO:0005525|G'	K07904
Manes.07G02650(	8.1211492	0.00136	up	yes	Probable seri	pfam00069	Pkinase	Protein kina	.	.	GO:0005524|A'	.
Manes.07G02660(	1.3395404	0.00126	up	no	.	pfam03606	DcuC	C4-dicarbox	.	.	.	.
Manes.07G02680(	13.004209	0.10442	up	no	Mitogen-acti	pfam00069	Pkinase	Protein kina	GO:0043622|	GO:0005737|	GO:0005524|A'	.
Manes.07G02690(	#NAME?	0.43239	down	no	.	pfam02362	B3	B3 DNA bir	.	.	.	.
Manes.07G02820(	1.9484404	3.46E-08	up	no	Pentatricope	pfam09606	Med15	ARC105 or	GO:0051301|	GO:0005739|	.	.
Manes.07G02850(	0.8595464	0.12565	down	no	.	pfam04339	DUF482	Protein of u	.	.	.	.
Manes.07G02870(	0.8264964	0.0761	down	no	NADPH-dep	pfam00106	adh_short	short chain	GO:0006760|	GO:0005829|	GO:0016616|ox	.
Manes.07G02920(	2.6779944	0.00393	up	yes	Probable glut	pfam13417	GST_N_3	Glutathione	.	.	GO:0004364|gl	K00799
Manes.07G02930(	0.7919297	0.27626	down	no	.	pfam06298	PsbY	Photosysten	.	.	.	.
Manes.07G02950(	Inf	0.9009	up	no	U-box domai	pfam00825	Ribonuclease_P	Ribonucleas	GO:0016567|	GO:0005737|	GO:0016874|lig	.
Manes.07G03030(	3.3300784	2.16E-11	up	yes	.	pfam06317	Arena_RNA_pol	Arenavirus	.	.	.	.
Manes.07G03060(	1.0011463	0.97639	up	no	Survival of m	pfam06003	SMN	Survival mo	GO:0006397|	GO:0015030|	GO:0003723|R	K12839
Manes.07G03070(	0.4868174	1.36E-06	down	yes	.	pfam06105	Aph-1	Aph-1 prote	.	.	.	.
Manes.07G03080(	1.360778	0.00099	up	no	Cullin-associ	pfam08623	TIP120	TATA-bindi	GO:0016567|	GO:0005618|	.	K17263
Manes.07G03090(	4.5802301	1.10E-54	up	yes	.	pfam04884	DUF647	Vitamin B6	.	.	.	.
Manes.07G03120(	0.9327633	0.48416	down	no	Squamous ce	pfam00076	RRM_1	RNA recogr	GO:0000902|	GO:0015030|	GO:0042393|his	.
Manes.07G03160(	0.6724248	0.01705	down	no	Tobamovirus	pfam06454	DUF1084	Protein of u	GO:0046786|	GO:0016021|	.	.
Manes.07G03170(	2.3526636	1.91E-15	up	yes	Outer envelo	pfam01425	Amidase	Amidase.	GO:0006626|	GO:0016021|	GO:0016884|ca	.
Manes.07G03210(	0.3551128	4.18E-05	down	yes	Metacaspase-	pfam00656	Peptidase_C14	Caspase dor	GO:0006952|	.	GO:0004197|cy	.
Manes.07G03220(	0.3208683	1.67E-09	down	yes	Metacaspase-	pfam00656	Peptidase_C14	Caspase dor	GO:0006952|	.	GO:0004197|cy	.
Manes.07G03240(	0.4250593	0.9154	down	no	Metacaspase-	pfam00656	Peptidase_C14	Caspase dor	GO:0006952|	.	GO:0004197|cy	.
Manes.07G03260(	0.8775291	0.21774	down	no	Methyl-CpG-	pfam13894	zf-C2H2_4	C2H2-type	GO:0006355|	GO:0005634|	GO:0008327|m	.
Manes.07G03370(	0.5704161	0.00016	down	no	7-deoxylogar	pfam00201	UDPGT	UDP-glucor	.	.	GO:0035251|U	.
Manes.07G03390(	3.2124434	1.76E-05	up	yes	Protein EXO	pfam04674	Phi_1	Phosphate-i	.	GO:0048046|	.	.
Manes.07G03450(	0.4273882	0.00011	down	yes	ABC transpo	pfam01061	ABC2_membrane	ABC-2 type	GO:0010588|	GO:0009897|	GO:0005524|A'	.
Manes.07G03460(	0.3931235	1.14E-08	down	yes	ABC transpo	pfam01061	ABC2_membrane	ABC-2 type	GO:0010588|	GO:0009897|	GO:0005524|A'	.
Manes.07G03470(	0.3202039	1.28E-20	down	yes	1,4-dihydrox	pfam01040	UbiA	UbiA prenyl	GO:0009772|	GO:0009507|	GO:0046428|1,	K02548
Manes.07G03530(	0.5904432	7.83E-07	down	no	Dicarboxylat	pfam00939	Na_sulph_symp	Sodium:sulf	GO:0019676|	GO:0009507|	GO:0005313|L-	.
Manes.07G03540(	3.85772	1.28E-47	up	yes	Bifunctional	pfam00926	DHBP_synthase	3,4-dihydro	GO:0009231|	GO:0009507|	GO:0008686|3,	K14652
Manes.07G03550(	4.9013554	1.26E-38	up	yes	.	pfam14264	Glucos_trans_II	Glucosyl tra	.	.	.	.
Manes.07G03560(	2.4358831	0.09294	up	no	.	pfam15598	Imm37	Immunity pr	.	.	.	.
Manes.07G03660(	0.8300556	0.05807	down	no	Transcription	pfam12157	DUF3591	Protein of u	GO:0016568|	GO:0005634|	.	K03125
Manes.07G03670(	1.0575406	0.59259	up	no	.	pfam06020	Roughex	Drosophila	.	.	.	.
Manes.07G03710(	0.5488488	3.98E-09	down	no	Root phototr	pfam03000	NPH3	NPH3 famil	GO:0009638|	GO:0005886|	GO:0004871|si	.
Manes.07G03730(	Inf	0.9009	up	no	Cell division	pfam07065	D123	D123. This	GO:0007049|	GO:0005737|	.	.
Manes.07G03760(	0.5203614	4.91E-07	down	no	Probable 2-o	pfam03171	2OG-FeII_Oxy	2OG-Fe(II)	.	.	GO:0005506|iro	.
Manes.07G03830(	0.491233	4.92E-05	down	yes	Probable ubic	pfam02902	Peptidase_C48	Ulp1 protea	GO:0016926|	.	GO:0016929|SU	K08596
Manes.07G03840(	4.3092862	5.48E-11	up	yes	Guanosine n	pfam00996	GDI	GDP dissoc	GO:0015031|	GO:0048046|	GO:0005096|G'	K17255
Manes.07G03880(	0.9029312	0.32306	down	no	Beta carboni	pfam00484	Pro_CA	Carbonic an	GO:0015976|	GO:0009507|	GO:0004089|ca	K01673
Manes.07G03970(	Inf	0.35135	up	no	.	pfam14895	PPPI_inhib	Protein phos	.	.	.	.
Manes.07G04040(	Inf	0.9009	up	no	.	pfam02956	TT_ORF1	TT viral orf	.	.	.	.
Manes.07G04070(	NA	NA	--	no	.	pfam05695	DUF825	Plant protei	.	.	.	.
Manes.07G04090(	2.0420574	0.00051	up	yes	Putative glut	pfam00117	GATase	Glutamine a	GO:0006878|	GO:0005737|	GO:0016740|tra	.
Manes.07G04100(	1.9232188	1.68E-13	up	no	.	pfam06747	CHCH	CHCH dom	.	.	.	.
Manes.07G04160(	3.7978614	6.67E-05	up	yes	Putative UDI	pfam00201	UDPGT	UDP-glucor	.	GO:0016021|	GO:0016758|tra	.
Manes.07G04180(	0.4690462	2.34E-11	down	yes	Beta-ureidop	pfam00795	CN_hydrolase	Carbon-nitr	GO:0019483|	GO:0045335|	GO:0003837|be	K01431
Manes.07G04190(	0.8505954	0.48896	down	no	.	pfam08507	COPI_assoc	COPI assoc	.	.	.	.
Manes.07G04240(	0.721526	0.06656	down	no	NAC transcri	pfam02365	NAM	No apical m	GO:0009908|	GO:0005634|	GO:0003677|D	.
Manes.07G04260(	0.8251781	0.77253	down	no	.	pfam13413	HTH_25	Helix-turn-h	.	.	.	.
Manes.07G04310(	0.7867868	0.10897	down	no	HVA22-like	pfam03134	TB2_DP1_HVA2	TB2/DP1, F	GO:0042538|	GO:0016021|	.	.
Manes.07G04320(	0.8075925	0.11084	down	no	Methylmalon	pfam00171	Aldedh	Aldehyde de	GO:0006979|	GO:0005739|	GO:0004029|ald	.
Manes.07G04330(	0.4935169	0.00098	down	yes	.	pfam04576	Zein-binding	Zein-binding	.	.	.	.
Manes.07G04340(	0.2962923	1.19E-06	down	yes	.	pfam15390	DUF4613	Domain of u	.	.	.	.
Manes.07G04350(	0.9642849	0.75755	down	no	.	pfam07264	EI24	Etoposide-i	.	.	.	.
Manes.07G04380(	2.0386179	2.17E-09	up	yes	Putative dise	pfam00931	NB-ARC	NB-ARC dc	GO:0006952|	.	GO:0043531|A	.
Manes.07G04390(	Inf	0.0238	up	yes	.	pfam00240	ubiquitin	Ubiquitin fa	.	.	.	.
Manes.07G04400(	2.7611553	2.12E-06	up	yes	.	pfam05936	DUF876	Bacterial pr	.	.	.	.
Manes.07G04410(	2.2723041	8.13E-15	up	yes	Putative dise	pfam00931	NB-ARC	NB-ARC dc	GO:0006952|	.	GO:0043531|A	.

Manes.07G04420(	3.24041	4.74E-13	up	yes	.	pfam10317	7TM_GPCR_Srd	Serpentine t	.	.	.	.
Manes.07G04430(	4.9131335	1.36E-22	up	yes	Putative disea	pfam00931	NB-ARC	NB-ARC dc	GO:0006952|(	.	GO:0043531|Al	.
Manes.07G04440(	Inf	0.1623	up	no	.	pfam04276	DUF443	Protein of u	.	.	.	.
Manes.07G04450(	5.7261477	1.26E-16	up	yes	.	pfam07226	DUF1422	Protein of u	.	.	.	.
Manes.07G04460(	3.847665	6.28E-06	up	yes	.	pfam05927	Penaeidin	Penaeidin. T	.	.	.	.
Manes.07G04470(	7.8369038	1.72E-25	up	yes	Putative disea	pfam00931	NB-ARC	NB-ARC dc	GO:0006952|(	.	GO:0043531|Al	.
Manes.07G04490(	0.9220735	0.53828	down	no	Mitogen-activ	pfam00069	Pkinase	Protein kina	GO:0000186|a	GO:0005634|	GO:0005524|A'	K13414
Manes.07G04510(	0.3987434	0.0017	down	yes	Tetraketide a	pfam01370	Epimerase	NAD depen	GO:0009555|p	GO:0005783|	GO:0050662|co	.
Manes.07G04530(	2.8849512	2.52E-05	up	yes	.	pfam05927	Penaeidin	Penaeidin. T	.	.	.	.
Manes.07G04540(	3.3758825	6.03E-18	up	yes	Putative disea	pfam00931	NB-ARC	NB-ARC dc	GO:0006952|(	.	GO:0043531|Al	.
Manes.07G04620(	4.156065	1.28E-05	up	yes	Uncharacteriz	pfam10839	DUF2647	Protein of u	.	GO:0009507|	.	.
Manes.07G04720(	4.0378077	0.00999	up	yes	.	pfam14093	DUF4271	Domain of u	.	.	.	.
Manes.07G04790(	NA	NA	--	no	L-type lectin-	pfam00069	Pkinase	Protein kina	GO:0009755|l	GO:0016021|	GO:0005524|A'	.
Manes.07G04830(	1.2267152	0.0269	up	no	Soluble inorg	pfam00719	Pyrophosphatase	Inorganic py	GO:0042742|(	GO:0009507|	GO:0004427|ino	K01507
Manes.07G04860(	0.1551792	6.47E-11	down	yes	Transcription	pfam00010	HLH	Helix-loop-l	GO:0006355|r	GO:0005634|	GO:0003677|DI	.
Manes.07G04900(	0.4828391	1.74E-07	down	yes	.	pfam06552	TOM20_plant	Plant specifi	.	.	.	.
Manes.07G04920(	4.0168959	1.22E-08	up	yes	Receptor-like	pfam00069	Pkinase	Protein kina	GO:0009960|(	GO:0016021|	GO:0005524|A'	.
Manes.07G04970(	0.3700677	2.58E-16	down	yes	.	pfam03108	DBD_Tnp_Mut	MuDR fami	.	.	.	.
Manes.07G04990(	1.7754851	7.61E-09	up	no	.	pfam08622	Svf1	Svf1-like. F	.	.	.	.
Manes.07G05000(	2.9412351	2.21E-33	up	yes	DUF21 doma	pfam01595	DUF21	Domain of u	.	GO:0016021|	.	K16302
Manes.07G05010(	0.9443667	0.71879	down	no	Uncharacteriz	pfam08241	Methyltransf_11	Methyltrans	.	GO:0009535|	.	.
Manes.07G05050(	Inf	0.38692	up	no	DNA-directe	pfam14700	RPOL_N	DNA-direct	GO:0006351|t	GO:0009507|	GO:0003677|DI	K10908
Manes.07G05150(	21.690992	2.17E-75	up	yes	.	pfam06219	DUF1005	Protein of u	.	.	.	.
Manes.07G05160(	10.626299	4.65E-08	up	yes	.	pfam03478	DUF295	Protein of u	.	.	.	.
Manes.07G05170(	NA	NA	--	no	Agamous-like	pfam00319	SRF-TF	SRF-type tr	GO:0009960|(	GO:0005634|	GO:0003677|DI	.
Manes.07G05180(	0.1269163	5.23E-12	down	yes	Secoisolaricir	pfam13561	adh_short_C2	Enoyl-(Acyl	.	.	GO:0000166|nu	.
Manes.07G05210(	0.7456312	0.02275	down	no	Secoisolaricir	pfam13561	adh_short_C2	Enoyl-(Acyl	.	.	GO:0000166|nu	.
Manes.07G05230(	1.22908	0.03085	up	no	Aspartate--tR	pfam00152	tRNA-synt_2	tRNA synth	GO:0006418|t	GO:0005737|	GO:0050560|as	K01876
Manes.07G05350(	0.3522025	7.68E-05	down	yes	Cytosolic sul	pfam00685	Sulfotransfer_1	Sulfotransfe	GO:0019761|g	GO:0005737|	GO:0047364|de	.
Manes.07G05420(	4.249056	1.17E-26	up	yes	.	pfam03613	EIID-AGA	PTS system	.	.	.	.
Manes.07G05580(	1.0043213	0.9333	up	no	Serine/threon	pfam11865	DUF3385	Domain of u	GO:0000724|(	GO:0005737|	GO:0016303|1-	.
Manes.07G05640(	0.8546426	0.14104	down	no	.	pfam09852	DUF2079	Predicted m	.	.	.	.
Manes.07G05650(	0.1612066	0.00083	down	yes	.	.	.	.	.	.	.	.
Manes.07G05670(	1.7595433	3.45E-09	up	no	F-box proteir	pfam12937	F-box-like	F-box-like. '	GO:0016567|p	GO:0005634|	.	K10293
Manes.07G05680(	0.0190072	7.53E-07	down	yes	.	pfam14309	DUF4378	Domain of u	.	.	.	.
Manes.07G05710(	0.8396802	0.13396	down	no	Sec1 family d	pfam00995	Sec1	Sec1 family.	GO:0051604|p	GO:0005789|	.	.
Manes.07G05880(	110.75756	0	up	yes	.	pfam06634	DUF1156	Protein of u	.	.	.	.
Manes.07G05900(	3.4201603	0.00027	up	yes	.	pfam07086	DUF1352	Protein of u	.	.	.	.
Manes.07G05930(	0.5471698	5.31E-06	down	no	Endo-1,4-bet	pfam00331	Glyco_hydro_10	Glycosyl hy	GO:0045493|:	GO:0005576|	GO:0031176|en	K01181
Manes.07G05970(	0.869711	0.26485	down	no	ADP-ribosyla	pfam00025	Arf	ADP-ribosy	GO:0051301|(	GO:0031902|	GO:0005525|G'	K07955
Manes.07G06060(	0.7392856	0.00754	down	no	.	pfam03798	TRAM_LAG1_CI	TLC domain	.	.	.	.
Manes.07G06070(	0.3724439	3.63E-17	down	yes	Disease resist	pfam00931	NB-ARC	NB-ARC dc	GO:0006952|(	.	GO:0043531|Al	.
Manes.07G06110(	0.4039281	3.34E-05	down	yes	Protein SNO'	pfam04484	DUF566	Family of ur	GO:0009658|(	GO:0005777|	.	.
Manes.07G06130(	0.492984	2.18E-08	down	yes	Disease resist	pfam00931	NB-ARC	NB-ARC dc	GO:0006952|(	.	GO:0043531|Al	.
Manes.07G06140(	0.5470765	6.00E-05	down	no	.	pfam04484	DUF566	Family of ur	.	.	.	.
Manes.07G06150(	0.5981482	6.37E-05	down	no	Disease resist	pfam00931	NB-ARC	NB-ARC dc	GO:0006952|(	.	GO:0043531|Al	.
Manes.07G06160(	1.2872616	0.05603	up	no	BTB/POZ do	pfam03000	NPH3	NPH3 famil	GO:0009958|p	GO:0071944|	.	.
Manes.07G06170(	115.47535	0	up	yes	Zinc finger pi	pfam13912	zf-C2H2_6	C2H2-type :	GO:0009738|a	GO:0005634|	GO:0003677|DI	.
Manes.07G06180(	1.1245214	0.19453	up	no	ATP-depende	pfam00574	CLP_protease	Clp proteas	.	GO:0009507|	GO:0004252|se	K01358
Manes.07G06190(	0.3298576	1.41E-06	down	yes	Receptor-like	pfam08263	LRRNT_2	Leucine rich	GO:0009755|l	GO:0016021|	GO:0004672|pr	.
Manes.07G06210(	0.6253949	0.00045	down	no	Vacuolar-sor	pfam02225	PA	PA domain.	GO:0006623|p	GO:0030665|	GO:0005509|ca	.
Manes.07G06230(	0.4731735	9.22E-08	down	yes	.	pfam04484	DUF566	Family of ur	.	.	.	.
Manes.07G06270(	0.4897434	0.00026	down	yes	.	pfam08045	CDC14	Cell division	.	.	.	.
Manes.07G06280(	0.2839213	4.67E-05	down	yes	.	pfam07666	MpPF26	M penetrans	.	.	.	.
Manes.07G06290(	0.5353953	0.02465	down	no	.	pfam15560	Imm8	Immunity pr	.	.	.	.
Manes.07G06310(	0.513476	2.58E-07	down	no	Disease resist	pfam00931	NB-ARC	NB-ARC dc	GO:0006952|(	.	GO:0043531|Al	.
Manes.07G06320(	2.861392	4.56E-27	up	yes	E3 ubiquitin-	pfam13639	zf-RING_2	Ring finger	GO:0043433|r	GO:0005737|	GO:0016874|lig	K16271
Manes.07G06330(	2.8381719	1.17E-23	up	yes	Probable prot	pfam00481	PP2C	Protein pho	GO:0006470|p	.	GO:0046872|me	.
Manes.07G06370(	3.6573267	3.76E-07	up	yes	.	pfam13891	zf-C3Hc3H	Potential DI	.	.	.	.
Manes.07G06400(	0.860612	0.11728	down	no	.	pfam12907	zf-met2	Zinc-binding	.	.	.	.
Manes.07G06450(	Inf	0.9009	up	no	Uncharacteriz	pfam00462	Glutaredoxin	Glutaredoxi	GO:0045454|(	GO:0005886|	GO:0009055|ele	.
Manes.07G06460(	0.651413	0.14084	down	no	.	pfam00023	Ank	Ankyrin rep	.	.	.	.
Manes.07G06480(	0.0696536	1.49E-06	down	yes	Protein MID'	pfam12222	PNGaseA	Peptide N-a	GO:0006816|(	GO:0005887|	.	.
Manes.07G06540(	0.5679813	0.0361	down	no	Snakin-1	pfam02704	GASA	Gibberellin	GO:0006952|(	GO:0005618|	.	.
Manes.07G06570(	0.6505669	0.1789	down	no	.	pfam08334	T2SG	Type II secr	.	.	.	.
Manes.07G06650(	0.9183507	0.39995	down	no	TIP41-like pi	pfam04176	TIP41	TIP41-like f	.	.	.	K17607
Manes.07G06660(	0.6270514	0.00012	down	no	Zinc finger pi	pfam13912	zf-C2H2_6	C2H2-type :	GO:0006355|r	GO:0005737|	GO:0046872|me	.
Manes.07G06670(	1.6513657	0.00048	up	no	.	pfam08696	Dna2	DNA replica	.	.	.	.
Manes.07G06680(	3.7608404	7.11E-50	up	yes	.	pfam04788	DUF620	Protein of u	.	.	.	.
Manes.07G06700(	0.3349708	0.00792	down	yes	Uncharacteriz	pfam13855	LRR_8	Leucine rich	.	GO:0005576|	.	.
Manes.07G06710(	0.4233828	0.00136	down	yes	Pentatricopep	pfam13041	PPR_2	PPR repeat	.	GO:0005739|	.	.
Manes.07G06720(	0.5120667	0.0008	down	no	Pentatricopep	pfam13041	PPR_2	PPR repeat	.	GO:0005739|	.	.
Manes.07G06740(	0.68172	0.02015	down	no	Pentatricopep	pfam13041	PPR_2	PPR repeat	.	GO:0005739|	.	.
Manes.07G06790(	3.9266131	9.30E-15	up	yes	Kynurenine f	pfam04199	Cyclase	Putative cyc	GO:0043420|a	.	GO:0004061|ar	K07130
Manes.07G06850(	0.747165	0.12102	down	no	.	pfam00646	F-box	F-box doma	.	.	.	.
Manes.07G06860(	0.9010154	0.28741	down	no	DNA-directe	pfam04801	Sin_N	Sin-like pro	GO:0006383|t	GO:0005829|	GO:0003899|DI	K14721
Manes.07G06960(	0.8528264	0.40512	down	no	D-3-phospho	pfam00389	2-Hacid_dh	D-isomer sp	GO:0009793|(	GO:0009507|	GO:0016597|an	K00058
Manes.07G06970(	0.8253194	0.04611	down	no	Na(+)/H(+) a	pfam03600	CitMHS	Citrate trans	GO:0006814|s	GO:0016021|	GO:0015297|an	.
Manes.07G07100(	0.6175282	0.09166	down	no	Methylthiorit	pfam01636	APH	Phosphotrar	GO:0071369|(	GO:0005829|	GO:0005524|A'	K00899
Manes.07G07140(	2.0710112	9.14E-06	up	yes	LRR receptor	pfam13855	LRR_8	Leucine rich	GO:0009793|(	GO:0016021|	GO:0005524|A'	.
Manes.07G07190(	0.2359696	3.03E-07	down	yes	Probable LRI	pfam00069	Pkinase	Protein kina	.	GO:0016021|	GO:0005524|A'	.
Manes.07G07200(	0.8599876	0.66936	down	no	.	pfam03154	Atrophin-1	Atrophin-1 f	.	.	.	.
Manes.07G07210(	0.4066995	5.39E-14	down	yes	Receptor pro	pfam00069	Pkinase	Protein kina	.	GO:0016021|	GO:0005524|A'	.
Manes.07G07250(	4.5076421	6.87E-66	up	yes	U-box domai	pfam04564	U-box	U-box doma	.	.	GO:0016874|lig	.
Manes.07G07280(	0.9595151	0.66792	down	no	Probable E3	pfam12906	RINGv	RING-varia	GO:0042335|(	GO:0016021|	GO:0016874|lig	K10661
Manes.07G07340(	#NAME?	0.01254	down	yes	Protein PMR	pfam13839	PC-Esterase	GDSL/SGN	GO:0006952|(	GO:0005783|	.	.
Manes.07G07360(	0.2415215	2.01E-21	down	yes	.	pfam12697	Abhydrolase_6	Alpha/beta l	.	.	.	.
Manes.07G07450(	0.263213	1.44E-07	down	yes	.	pfam09451	ATG27	Autophagy-	.	.	.	.

Manes.07G07460(	0.4979181	7.41E-12	down	yes	DEAD-box A	pfam00270	DEAD	DEAD/DEA	.	.	GO:0005524|A	K14809
Manes.07G07500(	Inf	0.9009	up	no	Polygalacturo	pfam00295	Glyco_hydro_28	Glycosyl hy	GO:0005975|	GO:0005618|	GO:0004650|pc	.
Manes.07G07510(	0.9031346	0.60263	down	no	O-acyltransfe	pfam06974	DUF1298	Protein of u	GO:0019432|	GO:0005783|	GO:0004144|di	K15406
Manes.07G07600(	2.0240403	3.89E-11	up	yes	.	pfam03699	UPF0182	Uncharacter	.	.	.	.
Manes.07G07610(	0.5705267	5.24E-06	down	no	Protein PLAI	pfam04749	PLAC8	PLAC8 fam	.	GO:0016021|	.	.
Manes.07G07620(	0.6156219	0.07279	down	no	Uncharacteriz	pfam12799	LRR_4	Leucine Ric	.	GO:0005576|	.	.
Manes.07G07630(	4.5497048	9.85E-15	up	yes	Scopoletin gl	pfam00201	UDPGT	UDP-glucor	.	.	GO:0042802|id	.
Manes.07G07670(	1.3784384	0.00714	up	no	DNA-directe	pfam01193	RNA_pol_L	RNA polym	GO:0006366|	GO:0005737|	GO:0003677|D	K03011
Manes.07G07680(	0.3071064	2.19E-08	down	yes	Cyclin-D3-1	pfam00134	Cyclin_N	Cyclin, N-te	GO:0051301|	GO:0005634|	.	K14505
Manes.07G07720(	0.3131342	0.00858	down	yes	O-acyltransfe	pfam03007	WES_acyltransf	Wax ester sy	GO:0019432|	GO:0005783|	GO:0004144|di	K15406
Manes.07G07790(	0.8387185	0.36626	down	no	Abscisic acid	pfam00067	p450	Cytochrome	GO:0046345|	GO:0016021|	GO:0010295|(+	K09843
Manes.07G07800(	1.4372067	0.0018	up	no	Glycoprotein	pfam00852	Glyco_transf_10	Glycosyltrar	GO:0071555|	GO:0005794|	GO:0046920|al	K00753
Manes.07G07830(	0.3749312	4.05E-07	down	yes	Inactive leuci	pfam00069	Pkinase	Protein kina	.	GO:0016021|	GO:0005524|A	.
Manes.07G07840(	0.3831329	0.00048	down	yes	Inactive leuci	pfam00069	Pkinase	Protein kina	.	GO:0016021|	GO:0005524|A	.
Manes.07G07850(	0.5297289	6.23E-10	down	no	Inactive poly	pfam12174	RST	RCD1-SRO	GO:0009816|	GO:0005737|	GO:0003950|N	.
Manes.07G07890(	NA	NA	--	no	Polygalacturo	pfam00295	Glyco_hydro_28	Glycosyl hy	GO:0005975|	GO:0005618|	GO:0004650|pc	.
Manes.07G07990(	1.4069245	1	up	no	Polygalacturo	pfam00295	Glyco_hydro_28	Glycosyl hy	GO:0005975|	GO:0005618|	GO:0004650|pc	.
Manes.07G08040(	1.5706956	1.68E-05	up	no	Aldo-keto re	pfam00248	Aldo_ket_red	Aldo/keto r	GO:0055114|	GO:0009507|	GO:0008106|al	.
Manes.07G08110(	88.919248	6.14E-33	up	yes	Abscisic acid	pfam00067	p450	Cytochrome	GO:0046345|	GO:0016021|	GO:0010295|(+	K09843
Manes.07G08120(	1.1549807	0.13065	up	no	YTH domain	pfam04146	YTH	YT521-B-li	GO:0043488|	GO:0000932|	GO:1990247|N	.
Manes.07G08130(	0.623233	9.26E-06	down	no	Expansin-A1	pfam01357	Pollen_allerg_1	Pollen allerg	GO:0009664|	GO:0005618|	.	.
Manes.07G08260(	2.6302961	8.77E-25	up	yes	Calmodulin-li	pfam13499	EF-hand_7	EF-hand do	.	GO:0005739|	GO:0005509|ca	K13448
Manes.07G08270(	1.5376375	0.00085	up	no	Protein disulf	pfam06074	DUF935	Protein of u	GO:0009658|	GO:0009507|	GO:0046872|m	.
Manes.07G08410(	1.0011326	0.97996	up	no	Vacuolar pro	pfam04157	EAP30	EAP30/Vps	GO:0043328|	GO:0000814|	GO:0032266|ph	K12190
Manes.07G08430(	Inf	0.9009	up	no	Probable pect	pfam01095	Pectinesterase	Pectinestera	GO:0042545|	GO:0005618|	GO:0045330|as	.
Manes.07G08440(	5.9570814	0.00579	up	yes	.	pfam06636	DUF1157	Protein of u	.	.	.	.
Manes.07G08460(	0.4952002	4.73E-10	down	yes	Protein FAR	pfam10551	MULE	MULE trans	GO:0006355|	GO:0005634|	GO:0008270|zi	.
Manes.07G08470(	Inf	0.00038	up	yes	.	pfam03179	V-ATPase_G	Vacuolar (H	.	.	.	.
Manes.07G08590(	0.5567945	8.86E-06	down	no	Chaperone pı	pfam02861	Clp_N	Clp amino t	GO:0045727|	GO:0009941|	GO:0005524|A	K03695
Manes.07G08620(	0.844175	0.11183	down	no	Exocyst com	pfam03081	Exo70	Exo70 exoc	GO:0006887|	GO:0005618|	.	.
Manes.07G08630(	0.7750138	0.03205	down	no	Cationic amir	pfam13520	AA_permease_2	Amino acid	GO:0003333|	GO:0031969|	GO:0015297|an	.
Manes.07G08680(	1.312965	0.0171	up	no	.	pfam14536	DUF4441	Domain of u	.	.	.	.
Manes.07G08690(	1.1600773	0.13754	up	no	.	pfam01814	Hemerythrin	Hemerythrir	.	.	.	.
Manes.07G08760(	4.304758	0.00041	up	yes	.	pfam04505	Dispanin	Interferon-ir	.	.	.	.
Manes.07G08780(	0.8300765	0.07243	down	no	R3H domain-	pfam12752	SUZ	SUZ domai	.	GO:0005634|	GO:0044822|pc	.
Manes.07G08810(	0.8687608	0.20039	down	no	.	pfam13041	PPR_2	PPR repeat	.	.	.	.
Manes.07G08860(	0.977298	0.87237	down	no	.	pfam12796	Ank_2	Ankyrin rep	.	.	.	.
Manes.07G08890(	1.044643	0.62161	up	no	Transcription	pfam05793	TFIIF_alpha	Transcriptio	GO:0032968|	GO:0005634|	GO:0003824|ca	K03138
Manes.07G08940(	1.7628032	5.80E-07	up	no	Tether contai	pfam00789	UBX	UBX domai	GO:0042593|	GO:0009898|	.	K15627
Manes.07G08970(	3.8503746	0.39483	up	no	.	.	.	.	.	.	.	.
Manes.07G09000(	2.4016588	0.76613	up	no	Beta-D-gluco	pfam00201	UDPGT	UDP-glucor	.	.	GO:0016758|tra	.
Manes.07G09010(	1.3112409	0.00411	up	no	.	pfam00261	Tropomyosin	Tropomyosi	.	.	.	.
Manes.07G09200(	1.6884903	4.75E-09	up	no	.	pfam05003	DUF668	Protein of u	.	.	.	.
Manes.07G09300(	0.6268762	0.0002	down	no	3-phosphoinc	pfam00069	Pkinase	Protein kina	.	GO:0005737|	GO:0005524|A	K06276
Manes.07G09310(	0.5995403	6.64E-05	down	no	Probable phy	pfam01148	CTP_transf_1	Cytidylyltrai	GO:0010189|	GO:0031969|	GO:0016301|ki	K18678
Manes.07G09320(	1.8733423	4.41E-12	up	no	.	pfam00534	Glycos_transf_1	Glycosyl tra	.	.	.	.
Manes.07G09340(	4.7824164	0.00354	up	yes	.	pfam02118	Srg	Srg family c	.	.	.	.
Manes.07G09350(	5.458358	9.75E-60	up	yes	.	pfam15504	DUF4647	Domain of u	.	.	.	.
Manes.07G09380(	0.8388302	0.08444	down	no	Protein neura	pfam13920	zf-C3HC4_3	Zinc finger,	GO:0007498|	GO:0005634|	GO:0003677|D	.
Manes.07G09390(	1.5689656	5.29E-06	up	no	Ribosome pro	pfam04427	Brix	Brix domain	GO:0006364|	GO:0005730|	GO:0003723|R	K14846
Manes.07G09430(	1.2085255	0.16892	up	no	Uncharacteriz	pfam03266	NTPase_1	NTPase. Th	.	GO:0009507|	GO:0005524|A	.
Manes.07G09440(	NA	NA	--	no	.	pfam06813	Nodulin-like	Nodulin-like	.	.	.	.
Manes.07G09470(	2.1286582	5.16E-16	up	yes	Probable pect	pfam00544	Pec_lyase_C	Pectate lyase	GO:0042547|	GO:0031225|	GO:0046872|m	K01728
Manes.07G09510(	3.6961308	2.81E-06	up	yes	Zinc finger pı	pfam13912	zf-C2H2_6	C2H2-type	GO:0006355|	GO:0005634|	GO:0046872|m	.
Manes.07G09520(	0.4482942	0.00348	down	yes	Monocopper	pfam00394	Cu-oxidase	Multicopper	GO:0009932|	GO:0031225|	GO:0005507|co	.
Manes.07G09540(	2.3692529	1.00E-07	up	yes	Vacuolar ami	pfam01490	Aa_trans	Transmemb	GO:0003333|	GO:0000324|	GO:0015186|L-	K15015
Manes.07G09550(	1.7496466	6.29E-09	up	no	Pumilio hom	pfam07990	NABP	Nucleic acid	GO:0006417|	GO:0005737|	GO:0003729|m	.
Manes.07G09560(	0.2804191	3.44E-09	down	yes	.	pfam11955	PORR	Plant organe	.	.	.	.
Manes.07G09570(	0.9894351	0.96132	down	no	.	pfam14911	MMS22L_C	S-phase gen	.	.	.	.
Manes.07G09590(	0.2970462	4.32E-11	down	yes	.	pfam11955	PORR	Plant organe	.	.	.	.
Manes.07G09710(	NA	NA	--	no	.	pfam07415	Herpes_LMP2	Gammaherp	.	.	.	.
Manes.07G09720(	0.5904256	0.02169	down	no	LysM domair	pfam07714	Pkinase_Tyr	Protein tyro	GO:0050832|	GO:0016021|	GO:0005524|A	.
Manes.07G09770(	0.2518434	0.00019	down	yes	.	pfam13966	zf-RVT	zinc-binding	.	.	.	.
Manes.07G09780(	0.8576262	0.26443	down	no	.	pfam11221	Med21	Subunit 21	.	.	.	.
Manes.07G09880(	1.0903922	0.44873	up	no	Synaptotagm	pfam00168	C2	C2 domain.	.	GO:0005783|	GO:0046872|m	.
Manes.07G09920(	0.3247639	1.89E-12	down	yes	Protein ENH	pfam07059	DUF1336	Protein of u	GO:0009873|	GO:0005789|	GO:0070273|ph	.
Manes.07G09950(	0.3733038	2.79E-11	down	yes	Auxin respon	pfam06507	Auxin_resp	Auxin respo	GO:0009734|	GO:0005634|	GO:0003677|D	.
Manes.07G09960(	1.6879886	1.05E-08	up	no	GATA transc	pfam00320	GATA	GATA zinc	GO:0007623|	GO:0005634|	GO:0043565|se	.
Manes.07G09970(	1.3091089	0.00262	up	no	Eukaryotic tr	pfam10255	Paf67	RNA polym	GO:0001731|	GO:0016282|	GO:0003743|tra	K15029
Manes.07G09980(	0.5119087	0.00174	down	no	Myb-related	pfam00249	Myb_DNA-binding	Myb-like D	.	GO:0005634|	GO:0003677|D	.
Manes.07G09990(	0.1973818	1.01E-06	down	yes	La protein 1	pfam08777	RRM_3	RNA bindin	GO:0034470|	GO:0005618|	GO:0000166|nu	K11090
Manes.07G10010(	1.2233696	0.41414	up	no	Ent-kaur-16-	pfam03936	Terpene_synth_C	Terpene syn	GO:0009686|	GO:0009507|	GO:0009899|en	.
Manes.07G10020(	0.718086	0.00477	down	no	.	pfam00583	Acetyltransf_1	Acetyltransf	.	.	.	.
Manes.07G10100(	Inf	0.9009	up	no	.	pfam02665	Nitrate_red_gam	Nitrate redu	.	.	.	.
Manes.07G10150(	0.3955347	1.95E-17	down	yes	Ethylene rece	pfam02518	HATPase_c	Histidine kir	GO:0009873|	GO:0005789|	GO:0005524|A	.
Manes.07G10160(	1.174204	0.07888	up	no	Regulator of	pfam02854	MIF4G	MIF4G dom	GO:0042742|	GO:0005737|	GO:0003723|R	K14327
Manes.07G10180(	0.8653231	0.1379	down	no	40S ribosom	pfam00164	Ribosom_S12_S23	Ribosomal p	GO:0006412|	GO:0015935|	GO:0003735|str	.
Manes.07G10200(	1.493405	0.65209	up	no	Low tempera	pfam01679	Pmp3	Proteolipid	.	GO:0016021|	.	.
Manes.07G10260(	20.258319	7.14E-11	up	yes	.	pfam05424	Duffy_binding	Duffy bindir	.	.	.	.
Manes.07G10270(	0.6369221	8.12E-05	down	no	S-adenosylme	pfam00153	Mito_carr	Mitochondr	GO:1901962|	GO:0016021|	GO:0000095|S-	K15111
Manes.07G10330(	Inf	0.0131	up	yes	.	pfam14666	RICTOR_M	Rapamycin-	.	.	.	.
Manes.07G10340(	2.3599287	1.36E-05	up	yes	.	pfam07111	HCR	Alpha helica	.	.	.	.
Manes.07G10350(	0.7870821	0.05381	down	no	RNA pseudo	pfam00849	PseudoU_synth_2	RNA pseudo	GO:0001522|	.	GO:0009982|ps	.
Manes.07G10400(	0.5877742	0.00602	down	no	Tetraspanin-	pfam00335	Tetraspannin	Tetraspanin	.	GO:0016021|	.	.
Manes.07G10410(	8.5036369	3.02E-26	up	yes	MLO-like pro	pfam03094	Mlo	Mlo family.	GO:0006952|	GO:0016021|	.	K08472
Manes.07G10440(	0.8971663	0.27584	down	no	Uncharacteriz	pfam03109	ABC1	ABC1 famil	.	.	GO:0005524|A	.
Manes.07G10450(	0.4809957	0.09192	down	no	.	pfam03190	Thioredox_DsbH	Protein of u	.	.	.	.
Manes.07G10470(	1.6015144	5.24E-07	up	no	Protein TON	pfam09398	FOP_dimer	FOP N term	GO:0030865|	GO:0030981|	.	.

Manes.07G10490(	0.5675139	1.26E-06	down	no	Two-compor	pfam09586	YfhO	Bacterial mo	GO:0000160|]	GO:0005634|	.	K12131
Manes.07G10510(	0.6795847	9.56E-05	down	no	Two-compor	pfam06203	CCT	CCT motif.	GO:0000160|]	GO:0005634|	.	.
Manes.07G10540(	1.9423777	2.33E-10	up	no	Serine/threon	pfam07714	Pkinase_Tyr	Protein tyro	GO:0009742|l	GO:0005886|	GO:0005524|A'	.
Manes.07G10560(	0.952201	0.68771	down	no	CDP-diacylgl	pfam01066	CDP-OH_P_transf	CDP-alcoho	GO:0006655|]	GO:0009507|	GO:0008444|Cl	K00995
Manes.07G10570(	0.9221733	0.41468	down	no	Protein IN2-1	pfam13417	GST_N_3	Glutathione	.	.	.	K00799
Manes.07G10580(	0.7446415	0.03413	down	no	Probable prot	pfam00481	PP2C	Protein phos	GO:0006470|]	.	GO:0046872|me	.
Manes.07G10620(	0.9094153	0.41668	down	no	Macrophage	pfam10607	CLTH	CTLH/CRA	GO:0007049|	GO:0005737|	.	K18624
Manes.07G10700(	2.5842531	2.27E-27	up	yes	Probable rece	pfam12819	Malectin_like	Carbohydrat	.	GO:0016021|	GO:0005524|A'	.
Manes.07G10710(	23.878051	0.01328	up	yes	Probable LRI	pfam12819	Malectin_like	Carbohydrat	GO:0009793|	GO:0016021|	GO:0005524|A'	.
Manes.07G10720(	0.6404201	4.42E-05	down	no	Chalcone--fla	pfam02431	Chalcone	Chalcone-fla	GO:0009813|]	.	GO:0045430|ch	.
Manes.07G10740(	1.1533167	0.6151	up	no	Probable mer	pfam09468	RNase_H2-Ydr27	Ydr279p pro	.	GO:0005886|	.	.
Manes.07G10750(	0.3179518	1.59E-05	down	yes	.	pfam01010	Oxidored_q1_C	NADH-Ubi	.	.	.	.
Manes.07G10780(	0.5249959	2.35E-06	down	no	Putative disea	pfam00931	NB-ARC	NB-ARC do	GO:0006952|	.	GO:0043531|Al	.
Manes.07G10790(	0.7727394	0.4012	down	no	Protein kinas	pfam00069	Pkinase	Protein kina	.	GO:0009507|	GO:0005524|A'	.
Manes.07G10840(	111.45908	1.94E-17	up	yes	Protein kinas	pfam00069	Pkinase	Protein kina	.	GO:0009507|	GO:0005524|A'	.
Manes.07G10850(	3.1793055	5.99E-24	up	yes	Protein kinas	pfam00069	Pkinase	Protein kina	.	GO:0009507|	GO:0005524|A'	.
Manes.07G10860(	27.797213	1.84E-05	up	yes	.	pfam03408	Foamy_virus_ENV	Foamy virus	.	.	.	.
Manes.07G10880(	0.7493894	0.04694	down	no	Protein kinas	pfam00069	Pkinase	Protein kina	.	GO:0009507|	GO:0005524|A'	.
Manes.07G10910(	0.9690414	0.79272	down	no	Splicing facto	pfam03178	CPSF_A	CPSF A sub	GO:0000398|]	GO:0071013|	GO:0003723|Rl	K12830
Manes.07G11010(	0.4824511	0.74402	down	no	.	pfam09713	A_thal_3526	Plant protein	.	.	.	.
Manes.07G11020(	1.2035246	0.04208	up	no	Sulfate transp	pfam00916	Sulfate_transp	Sulfate trans	.	GO:0031969|	GO:0008271|se	K18059
Manes.07G11040(	1.8540086	2.59E-12	up	no	Protein-tyros	pfam00782	DSPc	Dual specifi	GO:0006952|	GO:0005829|	GO:0017017|M	.
Manes.07G11050(	3.6788201	5.97E-17	up	yes	Auxin-induce	pfam02519	Auxin_inducible	Auxin respo	GO:0009734|a	.	.	K14488
Manes.07G11070(	3.6747497	0.00556	up	yes	.	pfam05056	DUF674	Protein of u	.	.	.	.
Manes.07G11110(	1.1797183	0.07774	up	no	PsbP-like pro	pfam01789	PsbP	PsbP. This f	GO:0015979|]	GO:0009507|	GO:0005509|ca	K02717
Manes.07G11120(	0.6565856	2.70E-05	down	no	3-hexulose-6	pfam01380	SIS	SIS domain.	GO:0005975|	.	GO:0030246|ca	K08094
Manes.07G11190(	13.8175	5.82E-15	up	yes	Zinc finger pr	pfam13912	zf-C2H2_6	C2H2-type z	GO:0071289|	GO:0005634|	GO:0046872|me	.
Manes.07G11200(	0.6553957	0.00034	down	no	.	pfam03871	RNA_pol_Rpb5_N	RNA polym	.	.	.	.
Manes.07G11250(	1.2329944	0.10786	up	no	Actin-depoly	pfam00241	Cofilin_ADF	Cofilin/trop	GO:0030042|a	GO:0015629|	.	.
Manes.07G11260(	2.7154801	6.28E-13	up	yes	.	pfam13041	PPR_2	PPR repeat	.	.	.	.
Manes.07G11310(	0.7315402	0.05948	down	no	B3 domain-co	pfam02362	B3	B3 DNA bin	GO:0009909|]	GO:0005654|	GO:0003677|Dl	.
Manes.07G11340(	0.4321653	0.0089	down	yes	THO comple	pfam00076	RRM_1	RNA recogn	GO:0051028|]	GO:0005654|	GO:0000166|nu	K12881
Manes.07G11360(	0.4245324	8.17E-05	down	yes	3-hydroxypro	pfam00378	ECH	Enoyl-CoA	GO:0006631|]	.	GO:0043956|3-	K15019
Manes.07G11380(	0.7531379	0.026	down	no	.	pfam08238	Sel1	Sel1 repeat.	.	.	.	.
Manes.07G11410(	2.4401673	5.99E-15	up	yes	Heat shock co	pfam00012	HSP70	Hsp70 prote	.	.	GO:0005524|A'	.
Manes.07G11420(	0.8211625	0.18185	down	no	Heat shock co	pfam00012	HSP70	Hsp70 prote	.	.	GO:0005524|A'	.
Manes.07G11430(	0.8906671	0.24861	down	no	.	pfam13019	Telomere_Sde2	Telomere st	.	.	.	.
Manes.07G11540(	3.2058078	3.00E-23	up	yes	Dof zinc fing	pfam02701	zf-Dof	Dof domain	GO:0006355|]	GO:0005634|	GO:0003677|Dl	.
Manes.07G11560(	4.8400499	0.00395	up	yes	.	pfam07649	C1_3	C1-like dom	.	.	.	.
Manes.07G11580(	0.8511163	0.14209	down	no	Very-long-ch	pfam02544	Steroid_dh	3-oxo-5-alp	GO:0006633|]	GO:0005783|	GO:0009922|fa	K10258
Manes.07G11610(	1.0311617	0.74344	up	no	Dolichyl-pho	pfam00535	Glycos_transf_2	Glycosyl tra	GO:0044267|	GO:0005789|	GO:0004581|do	K00729
Manes.07G11650(	6.3990841	0.00063	up	yes	Glucan endo-	pfam00332	Glyco_hydro_17	Glycosyl hy	GO:0005975|	.	GO:0042973|gl	.
Manes.07G11670(	1.3064287	0.00362	up	no	Triosephosph	pfam00121	TIM	Triosephosp	GO:0006094|]	GO:0005737|	GO:0004807|tri	.
Manes.07G11680(	4.327045	7.68E-61	up	yes	Protein kinas	pfam00069	Pkinase	Protein kina	.	GO:0009507|	GO:0005524|A'	.
Manes.07G11690(	4.0519647	1.22E-06	up	yes	Elicitor-respo	pfam00168	C2	C2 domain.	.	GO:0005737|	GO:0005544|ca	.
Manes.07G11720(	0.402434	1.18E-06	down	yes	Expansin-A4	pfam01357	Pollen_allerg_1	Pollen allerg	GO:0009664|]	GO:0005829|	.	.
Manes.07G11730(	1.927513	7.54E-13	up	no	Telomere rep	pfam00249	Myb_DNA-binding	Myb-like Dl	GO:0006355|]	GO:0000784|	GO:0008301|Dl	.
Manes.07G11740(	0.7317223	0.00193	down	no	Scarecrow-lil	pfam03514	GRAS	GRAS doma	GO:0006355|]	GO:0005634|	GO:0003700|tra	.
Manes.07G11770(	0.6335372	1	down	no	.	pfam05290	Baculo_IE-1	Baculovirus	.	.	.	.
Manes.07G11790(	0.5149776	3.44E-10	down	no	Peptidyl-prol	pfam00254	FKBP_C	FKBP-type	GO:0006457|]	.	GO:0003755|pe	K01802
Manes.07G11800(	1.0189867	0.82812	up	no	E3 ubiquitin-	pfam13639	zf-RING_2	Ring finger	GO:0009789|]	GO:0005783|	GO:0016874|lig	K16283
Manes.07G11830(	6.868391	2.92E-13	up	yes	E3 ubiquitin-	pfam06547	DUF1117	Protein of u	GO:0016567|]	.	GO:0016874|lig	.
Manes.07G11850(	0.3489069	6.30E-05	down	yes	.	pfam00314	Thaumatin	Thaumatin f	.	.	.	.
Manes.07G11870(	3.600702	0.0122	up	yes	AT-hook mo	pfam03479	DUF296	Domain of u	GO:0006325|	GO:0005634|	GO:0003680|A'	.
Manes.07G11880(	0.8034658	0.08636	down	no	Thylakoid lur	pfam00805	Pentapeptide	Pentapeptide	.	GO:0009507|	.	.
Manes.07G11890(	0.0724536	6.44E-28	down	yes	.	pfam04646	DUF604	Protein of u	.	.	.	.
Manes.07G11920(	1.0876021	0.37338	up	no	.	pfam00106	adh_short	short chain	.	.	.	.
Manes.07G11930(	3.4665923	0.07617	up	no	.	pfam10265	DUF2217	Uncharacter	.	.	.	.
Manes.07G11940(	7.5653907	2.20E-75	up	yes	Protein phosp	pfam00481	PP2C	Protein phos	GO:0009788|]	GO:0005886|	GO:0046872|me	K14497
Manes.07G11950(	1.1552493	0.12719	up	no	Probable met	pfam03141	Methyltransf_29	Putative S-a	.	GO:0005768|	GO:0008168|me	.
Manes.07G11980(	1.3696015	0.07688	up	no	Ultraviolet-B	pfam00415	RCC1	Regulator o	GO:0009649|	GO:0000785|	GO:0003682|ch	.
Manes.07G12040(	0.564844	0.00146	down	no	CASP-like pr	pfam04535	DUF588	Domain of u	.	GO:0016021|	.	.
Manes.07G12060(	1.875177	7.25E-11	up	no	Uncharacteriz	pfam02330	MAM33	Mitochondr	.	GO:0005759|	.	K15414
Manes.07G12070(	0.9718293	0.78788	down	no	Delta-1-pyrro	pfam00696	AA_kinase	Amino acid	GO:0055129|]	GO:0005737|	GO:0005524|A'	.
Manes.07G12100(	0.8862172	0.31267	down	no	PRA1 family	pfam03208	PRA1	PRA1 family	GO:0016192|	GO:0005783|	.	.
Manes.07G12110(	0.4149425	0.00473	down	yes	Putative disea	pfam00931	NB-ARC	NB-ARC do	GO:0006952|	GO:0009506|	GO:0043531|Al	.
Manes.07G12180(	0.3853063	0.00026	down	yes	Protein ABC	pfam02361	CbiQ	Cobalt trans	.	GO:0009507|	.	.
Manes.07G12190(	0.4816424	7.26E-05	down	yes	Protein DA1-	pfam12315	DUF3633	Protein of u	GO:0010088|]	.	GO:0043130|ub	.
Manes.07G12200(	0.9419862	0.63309	down	no	Mitochondria	pfam00153	Mito_carr	Mitochondr	GO:0055085|]	GO:0016021|	GO:0005509|ca	K14684
Manes.07G12210(	0.2124273	0.00042	down	yes	BEL1-like ho	pfam07526	POX	Associated v	GO:0048457|]	GO:0005829|	GO:0043565|se	.
Manes.07G12240(	Inf	0.9009	up	no	.	pfam09984	DUF2222	Uncharacter	.	.	.	.
Manes.07G12280(	0.2788626	2.77E-06	down	yes	Rop guanine	pfam03759	PRONE	PRONE (Pl	GO:0043547|]	.	GO:0005089|Rl	.
Manes.07G12290(	0.7658618	0.05552	down	no	.	pfam09778	Guanylate_cyc_2	Guanylylate	.	.	.	.
Manes.07G12310(	1.6558592	0.01271	up	no	.	pfam10328	7TM_GPCR_Srx	Serpentine t	.	.	.	.
Manes.07G12360(	0.6677399	0.00043	down	no	.	pfam12906	RINGv	RING-varia	.	.	.	.
Manes.07G12370(	0.2069549	1.58E-09	down	yes	.	pfam02271	UCR_14kD	Ubiquinol-c	.	.	.	.
Manes.07G12430(	0.8475825	0.26646	down	no	Immediate ea	pfam08571	Yos1	Yos1-like. I	.	GO:0005789|	.	.
Manes.07G12490(	2.9387067	1.19E-08	up	yes	.	pfam10604	Polyketide_cyc2	Polyketide c	.	.	.	.
Manes.07G12540(	2.8387119	1.18E-31	up	yes	.	pfam05999	Herpes_U5	Herpesvirus	.	.	.	.
Manes.07G12570(	1.0484453	0.64613	up	no	Peroxisomal	pfam00153	Mito_carr	Mitochondr	GO:0043132|]	GO:0046861|	GO:0015297|an	K13354
Manes.07G12660(	0.9167537	0.43142	down	no	Probable aqu	pfam00230	MIP	Major intrin	.	GO:0016021|	GO:0005215|tra	.
Manes.07G12670(	0.4154784	5.03E-11	down	yes	Glycosyltrans	pfam09258	Glyco_transf_64	Glycosyl tra	GO:0009738|a	GO:0005768|	GO:0046872|me	.
Manes.07G12690(	14.171127	1.27E-80	up	yes	Nematode res	pfam07014	Hs1pro-1_C	Hs1pro-1 pr	GO:0009816|	GO:0005737|	.	.
Manes.07G12710(	33.137187	0.00548	up	yes	Probable LRI	pfam00069	Pkinase	Protein kina	.	GO:0016021|	GO:0005524|A'	.
Manes.07G12760(	0.4223325	4.65E-07	down	yes	.	pfam10440	WIYLD	Ubiquitin-bi	.	.	.	.
Manes.07G12790(	0.7937765	0.06017	down	no	Anthranilate s	pfam00425	Chorismate_bind	chorismate l	GO:0010600|]	GO:0009507|	GO:0004049|an	.
Manes.07G12830(	0.5474327	8.90E-08	down	no	.	pfam05109	Herpes_BLLF1	Herpes virus	.	.	.	.
Manes.07G12840(	0.6853412	0.01747	down	no	.	pfam03226	Yippee-Mis18	Yippee zinc	.	.	.	.

Manes.07G12870(	2.3039097	1.97E-15	up	yes	Cytochrome	pfam00067	p450	Cytochrome	GO:0048657|a	GO:0016021|	GO:0020037|he	.
Manes.07G12890(	1.1837606	0.14554	up	no	Putative ABC	pfam03109	ABC1	ABC1 famil	GO:0006810|t	.	GO:0005215|tra	K08869
Manes.07G12940(	0.269185	0.12566	down	no	.	pfam08393	DHC_N2	Dynein heav	.	.	.	.
Manes.07G12960(	13.979153	4.66E-08	up	yes	Protein EAR	pfam07011	DUF1313	Protein of u	GO:0009649|e	GO:0005634|	GO:0042803|pr	.
Manes.07G12970(	Inf	0.08088	up	no	.	pfam09364	XFP_N	XFP N-term	.	.	.	.
Manes.07G13010(	1.5971403	2.25E-07	up	no	Chaperone pr	pfam07724	AAA_2	AAA domai	GO:0016485|p	GO:0005737|	GO:0005524|A	K03695
Manes.07G13020(	0.9487924	0.65465	down	no	PHD finger p	pfam12165	DUF3594	Domain of u	GO:0016568|c	GO:0005634|	GO:0035064|me	.
Manes.07G13030(	2.9721012	6.93E-06	up	yes	Leucoanthoc	pfam14226	DIOX_N	non-haem di	GO:0009718|a	.	GO:0031418|L-	.
Manes.07G13050(	0.3064833	1.19E-06	down	yes	Pentatricoper	pfam13041	PPR_2	PPR repeat	GO:0006397|r	GO:0009507|	.	.
Manes.07G13060(	15.470138	#######	up	yes	Zinc finger C	pfam12796	Ank_2	Ankyrin rep	GO:0050832|d	GO:0005634|	GO:0003677|D	.
Manes.07G13070(	1.8783055	3.12E-08	up	no	Omega-3 fatt	pfam11960	DUF3474	Domain of u	GO:0006636|u	GO:0031969|	GO:0016717|ox	K10257
Manes.07G13080(	0.7169092	0.0401	down	no	Methyltransfe	pfam01564	Spermine_synth	Spermine/sp	.	.	GO:0008168|me	.
Manes.07G13150(	2.2743369	4.85E-15	up	yes	GDP-Man:M	pfam00534	Glycos_transf_1	Glycosyl tra	GO:0097502|n	GO:0005783|	GO:0004377|G	K03844
Manes.07G13180(	0.2024281	0.04911	down	no	Transcription	pfam00249	Myb_DNA-binding	Myb-like DI	GO:0009738|a	GO:0005634|	GO:0003677|D	K09422
Manes.07G13210(	0.8784743	0.17903	down	no	Trihelix trans	pfam13837	Myb_DNA-bind_4	Myb/SANT-	GO:0009793|e	GO:0005634|	GO:0003677|D	.
Manes.07G13220(	0.2515642	2.69E-20	down	yes	.	pfam03195	DUF260	Protein of u	.	.	.	.
Manes.07G13250(	1.3021919	0.16515	up	no	Putative Myb	pfam00249	Myb_DNA-binding	Myb-like DI	GO:0006355|r	GO:0005634|	GO:0003677|D	.
Manes.07G13300(	NA	NA	--	no	Uncharacteriz	pfam00462	Glutaredoxin	Glutaredoxi	GO:0045454|c	GO:0005886|	GO:0009055|ele	.
Manes.07G13370(	NA	NA	--	no	Uncharacteriz	pfam00462	Glutaredoxin	Glutaredoxi	GO:0045454|c	GO:0005886|	GO:0009055|ele	.
Manes.07G13410(	0.4296501	3.90E-11	down	yes	Ankyrin repe	pfam12796	Ank_2	Ankyrin rep	GO:0035556|i	GO:0005622|	.	K10325
Manes.07G13440(	0.3540776	0.00034	down	yes	Protein MID	pfam04749	PLAC8	PLAC8 fam	GO:0006816|c	GO:0005887|	.	.
Manes.07G13450(	0.9370607	0.5891	down	no	Multiple inos	pfam00328	His_Phos_2	Histidine ph	GO:0016311|c	GO:0005829|	GO:0003993|ac	K03103
Manes.07G13480(	0.5910692	3.63E-07	down	no	DEAD-box A	pfam00270	DEAD	DEAD/DEA	.	.	GO:0005524|A	K14809
Manes.07G13520(	7.4513455	9.39E-20	up	yes	Abscisic acid	pfam10604	Polyketide_cyc2	Polyketide c	GO:0009738|a	GO:0005737|	GO:0010427|ab	K14496
Manes.07G13530(	29.175856	2.06E-52	up	yes	Dehydration-	pfam00847	AP2	AP2 domair	GO:0009738|a	GO:0005634|	GO:0003677|D	.
Manes.07G13540(	0.2366659	0.49873	down	no	Laccase-5	pfam07732	Cu-oxidase_3	Multicopper	GO:0046274|l	GO:0048046|	GO:0005507|co	K05909
Manes.07G13640(	1.1461077	0.30051	up	no	.	pfam00403	HMA	Heavy-meta	.	.	.	.
Manes.07G13650(	1.5013806	1.12E-05	up	no	Staphylococc	pfam00565	SNase	Staphylococ	GO:0006308|l	GO:0005886|	GO:0004520|en	.
Manes.07G13660(	0.1023868	6.47E-06	down	yes	.	pfam06681	DUF1182	Protein of u	.	.	.	.
Manes.07G13680(	Inf	5.27E-05	up	yes	.	pfam04525	Tub_2	Tubby C 2.	.	.	.	.
Manes.07G13690(	18.60569	#######	up	yes	.	pfam07107	WI12	Wound-indu	.	.	.	.
Manes.07G13700(	1.2288653	0.4292	up	no	.	pfam11204	DUF2985	Protein of u	.	.	.	.
Manes.07G13710(	1.3497306	0.00286	up	no	Alpha-soluble	pfam14938	SNAP	Soluble NSI	GO:0006886|i	GO:0016020|	GO:0005483|so	.
Manes.07G13780(	0.45712	5.02E-14	down	yes	CBL-interact	pfam00069	Pkinase	Protein kina	GO:0007165|s	.	GO:0005524|A	.
Manes.07G13790(	0.4868562	3.70E-05	down	yes	.	pfam03113	RSV_NS2	Respiratory	.	.	.	.
Manes.07G13800(	0.747347	0.27387	down	no	Uroporphyrin	pfam01208	URO-D	Uroporphyr	GO:0015995|c	GO:0009507|	GO:0004853|ur	.
Manes.07G13820(	0.0958103	4.56E-08	down	yes	High mobility	pfam00505	HMG_box	HMG (high	GO:0006338|c	GO:0005634|	GO:0003682|ch	.
Manes.07G13860(	0.293708	0.05761	down	no	Peroxidase 4	pfam00141	peroxidase	Peroxidase.	GO:0042744|l	GO:0005576|	GO:0020037|he	K00430
Manes.07G13940(	0.8849491	0.28505	down	no	.	pfam07767	Nop53	Nop53 (60S	.	.	.	.
Manes.07G13950(	1.1570786	0.11531	up	no	.	pfam05349	GATA-N	GATA-type	.	.	.	.
Manes.07G13960(	0.33303	2.68E-24	down	yes	Potassium tra	pfam02705	K_trans	K+ potassiu	.	GO:0016021|	GO:0015079|po	K03549
Manes.07G13970(	0.4443475	5.32E-06	down	yes	.	pfam14204	Ribosomal_L18_c	Ribosomal l	.	.	.	.
Manes.07G13990(	0.3365997	4.26E-10	down	yes	.	pfam04479	RTA1	RTA1 like p	.	.	.	.
Manes.07G14000(	43.506905	#######	up	yes	.	pfam05346	DUF747	Eukaryotic r	.	.	.	.
Manes.07G14010(	1.529979	0.00429	up	no	Alpha-galact	pfam02065	Melibiase	Melibiase. C	GO:0005975|c	.	GO:0052692|ra	.
Manes.07G14020(	2.5927435	1.81E-21	up	yes	.	pfam04928	PAP_central	Poly(A) pol	.	.	.	.
Manes.07G14030(	1.1283766	0.43015	up	no	tRNA (guani	pfam02005	TRM	N2,N2-dime	.	.	GO:0004809|tR	K00555
Manes.07G14060(	0.6058257	4.81E-05	down	no	.	pfam01661	Macro	Macro doma	.	.	.	.
Manes.07G14070(	0.2744595	8.19E-12	down	yes	Expansin-A8	pfam01357	Pollen_allerg_1	Pollen allerg	GO:0009664|p	GO:0005618|	.	.
Manes.07G14080(	0.3338793	2.38E-22	down	yes	RING-H2 fin	pfam13639	zf-RING_2	Ring finger	GO:0016567|p	GO:0016021|	GO:0008270|zin	.
Manes.07G14100(	0.4601439	1.73E-12	down	yes	.	pfam10433	MMS1_N	Mono-funct	.	.	.	.
Manes.07G14110(	0.7181688	0.0219	down	no	Probable met	pfam01588	tRNA_bind	Putative tRN	GO:0006431|r	GO:0005737|	GO:0005524|A	.
Manes.07G14120(	33.587408	2.40E-06	up	yes	.	pfam00625	Guanylate_kin	Guanylate k	.	.	.	.
Manes.07G14200(	0.9768596	0.96147	down	no	.	pfam11331	DUF3133	Protein of u	.	.	.	.
Manes.07G14210(	1.2626658	0.01005	up	no	Uncharacteriz	pfam03109	ABC1	ABC1 famil	.	GO:0009507|	GO:0005524|A	K08869
Manes.07G14260(	0.8613511	0.11781	down	no	Probable inac	pfam00069	Pkinase	Protein kina	.	GO:0005813|	GO:0005524|A	K08876
Manes.07G14280(	2.0523821	0.00137	up	yes	.	pfam05263	DUF722	Protein of u	.	.	.	.
Manes.07G14300(	0.9769919	0.79147	down	no	Methionine--	pfam01588	tRNA_bind	Putative tRN	GO:0006431|r	GO:0005829|	GO:0005524|A	K01874
Manes.07G14360(	0.4177747	2.48E-16	down	yes	.	pfam04735	Baculo_helicase	Baculovirus	.	.	.	.
Manes.08G00020(	2.4137405	3.55E-22	up	yes	Serine/argini	pfam00076	RRM_1	RNA recogr	GO:0000398|r	GO:0016607|	GO:0003676|nu	K12896
Manes.08G00040(	2.5052474	4.01E-09	up	yes	.	pfam11508	DUF3218	Protein of u	.	.	.	.
Manes.08G00060(	Inf	0.04449	up	no	Pathogenesis	pfam00188	CAP	Cysteine-ric	GO:0006952|c	GO:0005576|	.	.
Manes.08G00120(	6.0839356	2.91E-05	up	yes	.	pfam13150	DUF3989	Protein of u	.	.	.	.
Manes.08G00140(	1.2980182	0.3899	up	no	TBC1 domai	pfam00566	RabGAP-TBC	Rab-GTPase	.	GO:0016021|	GO:0005509|ca	.
Manes.08G00150(	2.112125	1.21E-06	up	yes	WRKY trans	pfam03106	WRKY	WRKY DN.	GO:0009957|c	GO:0005634|	GO:0043565|se	.
Manes.08G00160(	0.5444055	2.27E-09	down	no	Two-compor	pfam00072	Response_reg	Response re	GO:0007623|c	GO:0005739|	GO:0003677|D	K12129
Manes.08G00170(	0.2695366	1.17E-15	down	yes	Thioredoxin-	pfam13911	AhpC-TSA_2	AhpC/TSA	.	GO:0009507|	.	.
Manes.08G00180(	1.0972556	0.52028	up	no	Pentatricoper	pfam13041	PPR_2	PPR repeat	.	GO:0009507|	.	.
Manes.08G00190(	0.5481824	1.54E-06	down	no	Zinc finger C	pfam14897	EpsG	EpsG family	.	.	GO:0003677|D	.
Manes.08G00210(	0.4117749	7.04E-08	down	yes	.	pfam14239	RRXRR	RRXRR pro	.	.	.	.
Manes.08G00250(	10.188417	0.1034	up	no	Uncharacteriz	pfam00462	Glutaredoxin	Glutaredoxi	GO:0045454|c	GO:0005886|	GO:0009055|ele	.
Manes.08G00300(	1.0293475	0.74902	up	no	E3 ubiquitin-	pfam00632	HECT	HECT-dom	GO:0042787|p	GO:0005737|	GO:0016874|lig	K10590
Manes.08G00310(	1.0849812	0.34206	up	no	Pentatricoper	pfam13041	PPR_2	PPR repeat	GO:0006397|r	GO:0009570|	GO:0003729|m	.
Manes.08G00340(	0.6342888	5.40E-05	down	no	.	pfam10323	7TM_GPCR_Srv	Serpentine t	.	.	.	.
Manes.08G00380(	1.4466801	0.00011	up	no	Protein IQ-D	pfam13178	DUF4005	Protein of u	.	GO:0005634|	.	.
Manes.08G00390(	0.5766237	0.06336	down	no	Probable beta	pfam03360	Glyco_transf_43	Glycosyltrar	GO:0071555|c	GO:0005794|	GO:0015018|ga	.
Manes.08G00400(	0.7401398	0.0138	down	no	Importin sub	pfam00514	Arm	Armadillo/b	GO:0000278|r	GO:0005829|	GO:0008565|pr	.
Manes.08G00420(	0.5796631	0.00108	down	no	.	pfam14897	EpsG	EpsG family	.	.	.	.
Manes.08G00430(	0.5374923	3.27E-05	down	no	.	pfam12072	DUF3552	Domain of u	.	.	.	.
Manes.08G00480(	0.7963097	0.04113	down	no	L-type lectin-	pfam00139	Lectin_legB	Legume lect	GO:0009755|l	GO:0016021|	GO:0005524|A	.
Manes.08G00520(	0.7747453	0.01347	down	no	E3 ubiquitin-	pfam12692	Methyltransf_17	S-adenosyl-	GO:0009908|f	.	GO:0016874|lig	.
Manes.08G00530(	0.3747687	8.03E-07	down	yes	.	pfam00843	Arena_nucleocap	Arenavirus r	.	.	.	.
Manes.08G00580(	0.5600623	7.26E-05	down	no	DnaJ protein	pfam00226	DnaJ	DnaJ domai	GO:0044794|p	GO:0005783|	.	K09523
Manes.08G00610(	0.6982277	0.00039	down	no	Probable E3	pfam12906	RINGv	RING-varia	GO:0016567|p	.	GO:0016874|lig	K10665
Manes.08G00620(	0.6639615	0.00231	down	no	.	pfam05140	ResB	ResB-like fa	.	.	.	.
Manes.08G00640(	1.8367262	7.89E-12	up	no	Probable E3	pfam13920	zf-C3HC4_3	Zinc finger,	GO:1901527|a	GO:0005886|	GO:0016874|lig	.
Manes.08G00650(	0.5450744	0.00376	down	no	Putative seri	pfam00069	Pkinase	Protein kina	.	.	GO:0005524|A	.
Manes.08G00670(	0.7488725	0.06116	down	no	Serine/threon	pfam07714	Pkinase_Tyr	Protein tyro	GO:0009755|l	GO:0016021|	GO:0005524|A	.

Manes.08G00680(	0.3759635	2.59E-13	down	yes	Aquaporin Pl	pfam00230	MIP	Major intrin	.	GO:0016021|	GO:0005215|tra	K09872
Manes.08G00740(	5.3003711	1.23E-63	up	yes	CASP-like pr	pfam04535	DUF588	Domain of u	.	GO:0016021|	.	.
Manes.08G00770(	0.4472703	0.07601	down	no	BTB/POZ do	pfam03000	NPH3	NPH3 famil	GO:0016567|p	.	.	.
Manes.08G00780(	0.4273133	3.59E-11	down	yes	Cytokinin rib	pfam03641	Lysine_decarbox	Possible lysi	GO:0009691|c	GO:0005737|c	GO:0016799|hy	.
Manes.08G00790(	Inf	2.66E-25	up	yes	Putative cycli	pfam00027	cNMP_binding	Cyclic nucle	GO:0071805|p	GO:0005887|i	GO:0030552|cA	K05391
Manes.08G00840(	2.6567935	7.50E-23	up	yes	Phenylalanine	pfam00221	Lyase_aromatic	Aromatic an	GO:0009800|c	GO:0005737|c	GO:0045548|ph	.
Manes.08G00880(	10.31458	0.00197	up	yes	.	pfam14745	WASH-7_N	WASH com	.	.	.	.
Manes.08G00890(	0.7248017	0.00136	down	no	Telomere rep	pfam00249	Myb_DNA-binding	Myb-like DI	GO:0006355|r	GO:0000784|	GO:0003691|dc	.
Manes.08G00910(	0.7036162	0.01771	down	no	OTU domain	pfam02338	OTU	OTU-like cy	.	.	GO:0008234|cy	.
Manes.08G00940(	0.0206494	7.45E-08	down	yes	.	pfam08137	DVL	DVL family	.	.	.	.
Manes.08G00960(	0.5897117	0.00192	down	no	UDP-glycosy	pfam00201	UDPGT	UDP-glucor	.	.	GO:0016758|tra	.
Manes.08G00970(	0.8296763	0.09647	down	no	TSL-kinase i	pfam00249	Myb_DNA-binding	Myb-like DI	GO:0006355|r	GO:0005634|r	GO:0003677|DI	.
Manes.08G01010(	0.021151	1.01E-16	down	yes	Auxin-induce	pfam02519	Auxin_inducible	Auxin respo	GO:0009734|a	.	.	.
Manes.08G01060(	0.382612	3.99E-06	down	yes	.	pfam00403	HMA	Heavy-meta	.	.	.	.
Manes.08G01070(	0.9426941	0.5576	down	no	Histidine--tR	pfam13393	tRNA-synt_His	Histidyl-tRN	GO:0006427|l	GO:0005737|c	GO:0005524|A1	K01892
Manes.08G01100(	0.2494627	0.00041	down	yes	.	pfam12698	ABC2_membrane_	ABC-2 fami	.	.	.	.
Manes.08G01110(	0.5645269	1.62E-06	down	no	ABC transpo	pfam00664	ABC_membrane	ABC transp	GO:0010541|a	GO:0016021|	GO:0005524|A1	K05658
Manes.08G01130(	0.5054422	0.06672	down	no	Transcription	pfam00249	Myb_DNA-binding	Myb-like DI	GO:0007275|r	GO:0005634|r	GO:0003677|DI	K09422
Manes.08G01140(	0.5291902	1.51E-08	down	no	.	pfam15175	SPATA24	Spermatoge	.	.	.	.
Manes.08G01160(	0.5915708	0.00024	down	no	Thioredoxin-	pfam00085	Thioredoxin	Thioredoxin	GO:0045454|c	GO:0005737|c	GO:0016671|ox	K03671
Manes.08G01170(	0.5814104	0.00165	down	no	.	pfam01842	ACT	ACT domai	.	.	.	.
Manes.08G01180(	0.3509257	1.46E-17	down	yes	Cytochrome	pfam00067	p450	Cytochrome	.	GO:0005789|c	GO:0020037|he	.
Manes.08G01190(	0.3377488	4.50E-15	down	yes	Probable xylo	pfam00722	Glyco_hydro_16	Glycosyl hy	GO:0042546|c	GO:0048046|c	GO:0004553|hy	.
Manes.08G01220(	0.7376273	0.00381	down	no	Serine/threon	pfam01603	B56	Protein pho	GO:0007165|s	GO:0000159|r	GO:0008601|pr	K11584
Manes.08G01250(	0.865865	1	down	no	.	pfam01139	RtcB	tRNA-splici	.	.	.	.
Manes.08G01270(	0.4475526	0.00011	down	yes	RING finger	pfam14599	zinc_ribbon_6	Zinc-ribbon.	GO:0032436|p	GO:0005737|c	GO:0016874|lig	K10144
Manes.08G01360(	0.6728071	0.00203	down	no	Protein fluG	pfam00120	Gln-synt_C	Glutamine s	GO:0001896|a	GO:0005737|c	GO:0003824|ca	.
Manes.08G01370(	0.8946076	0.27517	down	no	Protein fluG	pfam00120	Gln-synt_C	Glutamine s	GO:0001896|a	GO:0005737|c	GO:0003824|ca	.
Manes.08G01480(	0.6806656	8.07E-05	down	no	.	pfam12695	Abhydrolase_5	Alpha/beta l	.	.	.	.
Manes.08G01500(	0.6773626	0.01111	down	no	snRNA-activ	pfam12251	zf-SNAP50_C	snRNA-acti	GO:0009926|a	GO:0005634|r	GO:0003677|DI	K15210
Manes.08G01510(	0.3910198	2.87E-14	down	yes	.	pfam13639	zf-RING_2	Ring finger	.	.	.	.
Manes.08G01520(	0.3162292	2.11E-08	down	yes	.	pfam04043	PMEI	Plant inverta	.	.	.	.
Manes.08G01530(	0.7302547	0.00121	down	no	Probable gala	pfam01501	Glyco_transf_8	Glycosyl tra	GO:0071555|c	GO:0005794|c	GO:0047262|po	K13648
Manes.08G01570(	1.2973472	0.00385	up	no	DEAD-box A	pfam00270	DEAD	DEAD/DEA	GO:0016973|p	GO:0005737|c	GO:0005524|A1	K18655
Manes.08G01580(	1.084189	0.48242	up	no	.	pfam09350	DUF1992	Domain of u	.	.	.	.
Manes.08G01590(	0.9061229	0.4927	down	no	Vacuolar pro	pfam07200	Mod_r	Modifier of	GO:0015031|p	GO:0000813|l	.	K12185
Manes.08G01610(	0.6058323	0.58243	down	no	RING-H2 fin	pfam13639	zf-RING_2	Ring finger	GO:0016567|p	GO:0016021|	GO:0008270|zi	.
Manes.08G01620(	NA	NA	--	no	Probable 2-o:	pfam03171	2OG-FeII_Oxy	2OG-Fe(II)	.	.	GO:0005506|irc	.
Manes.08G01640(	1.684042	1.31E-08	up	no	Caffeic acid 3	pfam00891	Methyltransf_2	O-methyltra	GO:0009809|l	.	GO:0047763|ca	.
Manes.08G01650(	6.1473023	4.14E-10	up	yes	Probable pect	pfam01095	Pectinesterase	Pectinestera	GO:0042545|c	GO:0005618|c	GO:0045330|as	.
Manes.08G01690(	0.477124	0.01439	down	yes	UDP-glycosy	pfam00201	UDPGT	UDP-glucor	.	.	GO:0016758|tra	K13496
Manes.08G01750(	0.6254135	0.03237	down	no	GDSL estera	pfam00657	Lipase_GDSL	GDSL-like l	GO:0016042|l	GO:0005576|c	GO:0016298|lip	.
Manes.08G01760(	1.0765582	0.41068	up	no	E3 ubiquitin-	pfam00632	HECT	HECT-dom	GO:0042787|p	GO:0005737|c	GO:0016874|lig	K10588
Manes.08G01770(	0.4444016	5.69E-14	down	yes	Pentatricoper	pfam14432	DYW_deaminase	DYW family	GO:0009793|c	.	.	.
Manes.08G01790(	2.3605806	0.0045	up	yes	Probable glyc	pfam03016	Exostosin	Exostosin fa	GO:0071555|c	GO:0000139|c	GO:0016757|tra	.
Manes.08G01800(	1.2891721	1	up	no	Embryonic pr	pfam02987	LEA_4	Late embryo	.	GO:0005618|c	.	.
Manes.08G01810(	0.8356145	0.45702	down	no	.	pfam01925	TauE	Sulfite expo	.	.	.	.
Manes.08G01820(	1.8260498	8.68E-11	up	no	Protein kinas	pfam00069	Pkinase	Protein kina	GO:0051301|c	GO:0032153|c	GO:0005524|A1	K08832
Manes.08G01870(	Inf	0.9009	up	no	.	pfam11902	DUF3422	Protein of u	.	.	.	.
Manes.08G01890(	0.5756125	0.00328	down	no	Probable 3-de	pfam04413	Glycos_transf_N	3-Deoxy-D-	GO:0036104|l	GO:0005739|c	GO:0016740|tra	K02527
Manes.08G01900(	0.0965802	0.00024	down	yes	Cytochrome	pfam00067	p450	Cytochrome	.	.	GO:0020037|he	.
Manes.08G01940(	0.4994014	0.02475	down	yes	Cytochrome	pfam00067	p450	Cytochrome	.	.	GO:0020037|he	.
Manes.08G01950(	2.3921625	1.92E-20	up	yes	Cytochrome	pfam00067	p450	Cytochrome	.	.	GO:0020037|he	.
Manes.08G01960(	0.678568	0.01208	down	no	.	pfam05003	DUF668	Protein of u	.	.	.	.
Manes.08G02010(	0.9505274	0.61745	down	no	Glucomannar	pfam13641	Glyco_tranf_2_3	Glycosyltrar	GO:0071555|c	GO:0000139|c	GO:0047259|glu	K13680
Manes.08G02020(	1.2707647	0.00882	up	no	Enolase 2	pfam00113	Enolase_C	Enolase, C-t	GO:0006096|g	GO:0000015|p	GO:0000287|ma	.
Manes.08G02030(	0.6985672	0.00069	down	no	Splicing facto	pfam00642	zf-CCCH	Zinc finger C	GO:0006397|r	GO:0016607|r	GO:0003677|DI	.
Manes.08G02070(	13.846391	#######	up	yes	Heat stress tr	pfam00447	HSF_DNA-bind	HSF-type D	GO:0006355|r	GO:0005634|r	GO:0003677|DI	K09419
Manes.08G02080(	8.7850393	0.05118	up	no	.	pfam00199	Catalase	Catalase.	.	.	.	.
Manes.08G02090(	1.1973411	0.05087	up	no	Ribosome pro	pfam04427	Brix	Brix domain	.	GO:0005730|r	.	K14847
Manes.08G02220(	1.6414519	3.04E-08	up	no	.	pfam04949	Transcrip_act	Transcriptio	.	.	.	.
Manes.08G02300(	0.3402897	1.83E-15	down	yes	Serine/threon	pfam00069	Pkinase	Protein kina	GO:0035556|i	GO:0005737|c	GO:0005524|A1	.
Manes.08G02310(	0.6819375	0.11112	down	no	.	pfam09508	Lact_bio_phlase	Lacto-N-bic	.	.	.	.
Manes.08G02320(	1.6931848	4.09E-06	up	no	Monodehydro	pfam07992	Pyr_redox_2	Pyridine nuc	GO:0045454|c	GO:0005737|c	GO:0050660|fla	.
Manes.08G02330(	0.8528993	0.33738	down	no	60S acidic rib	pfam00428	Ribosomal_60s	60s Acidic r	GO:0006414|t	GO:0005840|r	GO:0003735|str	.
Manes.08G02340(	0.4932653	1.23E-08	down	yes	.	pfam00612	IQ	IQ calmodu	.	.	.	.
Manes.08G02380(	0.4364952	1.38E-06	down	yes	Zinc finger A	pfam01428	zf-AN1	AN1-like Zi	.	.	GO:0003677|DI	.
Manes.08G02400(	3.2847526	2.96E-42	up	yes	Purple acid p	pfam00149	Metallophos	Calcineurin-	GO:0016311|c	GO:0005576|c	GO:0003993|ac	.
Manes.08G02490(	0.3878011	1.32E-10	down	yes	.	pfam12280	BSMAP	Brain specif	.	.	.	.
Manes.08G02500(	0.7004905	0.00348	down	no	Probable cyto	pfam00400	WD40	WD domain	GO:0016226|i	GO:0097361|c	.	.
Manes.08G02530(	3.4058914	2.45E-06	up	yes	Glucosamine	pfam00583	Acetyltransf_1	Acetyltransf	GO:0006044|l	GO:0005783|c	GO:0004343|glu	K00621
Manes.08G02610(	0.7477126	0.01432	down	no	.	pfam12937	F-box-like	F-box-like.	.	.	.	.
Manes.08G02620(	1.877205	4.93E-12	up	no	Heterogeneou	pfam00076	RRM_1	RNA recogr	GO:0007623|c	GO:0070937|c	GO:0000166|nu	K13160
Manes.08G02650(	0.3518229	8.27E-07	down	yes	Putative pent	pfam14432	DYW_deaminase	DYW family	GO:0016554|c	.	.	.
Manes.08G02700(	0.5944445	2.29E-05	down	no	.	pfam13136	DUF3984	Protein of u	.	.	.	.
Manes.08G02750(	2.5185851	1.06E-05	up	yes	.	pfam12697	Abhydrolase_6	Alpha/beta l	.	.	.	.
Manes.08G02760(	1.4147246	0.00177	up	no	.	pfam12494	DUF3695	Protein of u	.	.	.	.
Manes.08G02800(	NA	NA	--	no	NAC domain	pfam02365	NAM	No apical m	GO:0006351|t	GO:0005634|r	GO:0003677|DI	.
Manes.08G02840(	0.9732945	0.80071	down	no	Probable UD	pfam13844	Glyco_transf_41	Glycosyl tra	GO:0009740|p	GO:0005634|r	GO:0016757|tra	.
Manes.08G02850(	Inf	0.00129	up	yes	Casparian str	pfam04535	DUF588	Domain of u	GO:0071555|c	GO:0016021|	.	.
Manes.08G02860(	Inf	0.9009	up	no	Casparian str	pfam04535	DUF588	Domain of u	GO:0071555|c	GO:0016021|	.	.
Manes.08G02890(	0.9744108	0.79608	down	no	.	pfam05793	TFIIF_alpha	Transcriptio	.	.	.	.
Manes.08G02900(	0.9757612	0.85924	down	no	Protein tricho	pfam13839	PC-Esterase	GDSL/SGN	.	GO:0016021|	.	.
Manes.08G02910(	0.9730612	0.824	down	no	.	pfam15433	MRP-S31	Mitochondr	.	.	.	.
Manes.08G02920(	6.3579857	3.22E-57	up	yes	B3 domain-co	pfam02362	B3	B3 DNA bir	GO:0045892|r	GO:0005634|r	GO:0003677|DI	.
Manes.08G02930(	3.8465463	0.00088	up	yes	Lysosomal Pr	pfam05577	Peptidase_S28	Serine carbc	GO:0043535|p	GO:0005764|l	GO:0004180|ca	K01285
Manes.08G02940(	0.7090752	0.00483	down	no	Uncharacteriz	pfam01576	Myosin_tail_1	Myosin tail.	.	GO:0009507|c	.	.
Manes.08G02950(	0.3869556	2.33E-06	down	yes	IAA-amino a	pfam01546	Peptidase_M20	Peptidase fa	.	.	GO:0016787|hy	K14664

Manes.08G02960(	0.2209068	1.52E-08	down	yes	IAA-amino a	pfam13289	SIR2_2	SIR2-like d	.	.	GO:0016787|hy	.
Manes.08G02970(	0.4344433	1.68E-12	down	yes	Tropinone re	pfam13561	adh_short_C2	Enoyl-(Acyl	.	.	GO:0016491|ox	.
Manes.08G03000(	Inf	0.9009	up	no	Secoisolaricii	pfam00106	adh_short	short chain	.	.	GO:0016491|ox	.
Manes.08G03020(	0.5668411	2.34E-07	down	no	Secoisolaricii	pfam00106	adh_short	short chain	.	.	GO:0000166|nu	.
Manes.08G03030(	0.5300978	0.00764	down	no	Secoisolaricii	pfam00106	adh_short	short chain	.	.	GO:0016491|ox	.
Manes.08G03050(	0.9952066	0.95806	down	no	TLD domain	pfam07534	TLD	TLD. This c	.	GO:0005765|	.	.
Manes.08G03060(	0.816461	0.06151	down	no	.	pfam14643	DUF4455	Domain of u	.	.	.	.
Manes.08G03140(	1.5051183	0.17536	up	no	.	pfam03134	TB2_DP1_HVA2	TB2/DP1, H	.	.	.	.
Manes.08G03170(	0.5044118	8.40E-05	down	no	.	pfam02536	mTERF	mTERF. Th	.	.	.	.
Manes.08G03210(	0.5573468	2.48E-06	down	no	Aspartic prot	pfam14541	TAXi_C	Xylanase inl	.	GO:0005576|	GO:0004190|as	.
Manes.08G03220(	0.7214623	0.00149	down	no	Protein CLE(	pfam09758	FPL	Uncharacter	GO:0006914|	GO:0010008|	.	.
Manes.08G03260(	0.5514988	0.00282	down	no	.	pfam14659	Phage_int_SAM_	Phage integ	.	.	.	.
Manes.08G03270(	6.3005197	0.00711	up	yes	Protein YLS	pfam03168	LEA_2	Late embryo	GO:0051607|	GO:0009507|	.	.
Manes.08G03290(	0.2512727	2.41E-13	down	yes	.	pfam05296	TAS2R	Mammalian	.	.	.	.
Manes.08G03300(	0.4656446	5.61E-05	down	yes	.	pfam07520	SrfB	Virulence fa	.	.	.	.
Manes.08G03310(	0.3825293	0.00028	down	yes	Protein CHR	pfam13880	Acetyltransf_13	ESCO1/2 ac	GO:0048653|	GO:0005737|	GO:0016407|ac	K11268
Manes.08G03340(	0.2494159	8.51E-28	down	yes	Receptor pro	pfam07714	Pkinase_Tyr	Protein tyro	GO:0009755|	GO:0005576|	GO:0005524|A	K00924
Manes.08G03370(	1.0102533	0.88017	up	no	Digalactosyl	pfam00534	Glycos_transf_1	Glycosyl tra	GO:0009058|	GO:0009707|	GO:0046481|di	.
Manes.08G03380(	39.137579	4.36E-08	up	yes	Aluminum-ac	pfam11744	ALMT	Aluminium	GO:0015743|	GO:0016021|	.	.
Manes.08G03400(	9.4157595	2.26E-07	up	yes	E3 ubiquitin-	pfam04564	U-box	U-box doma	GO:0006952|	.	GO:0016874|lig	.
Manes.08G03410(	40.319052	6.31E-08	up	yes	E3 ubiquitin-	pfam04564	U-box	U-box doma	GO:0006952|	GO:0005829|	GO:0016874|lig	.
Manes.08G03450(	0.5403266	0.00126	down	no	Putative deo	pfam01026	TatD_DNase	TatD relatec	.	GO:0005634|	GO:0016888|en	K03424
Manes.08G03460(	2.3939754	1.74E-14	up	yes	33 kDa ribon	pfam00076	RRM_1	RNA recogi	GO:0006397|	GO:0009507|	GO:0000166|nu	.
Manes.08G03510(	Inf	0.35135	up	no	Agamous-lik	pfam00319	SRF-TF	SRF-type tr	GO:0006351|	GO:0005634|	GO:0003677|D	.
Manes.08G03520(	1.6242043	1.16E-05	up	no	.	pfam10204	DuoxA	Dual oxidas	.	.	.	.
Manes.08G03550(	3.3441655	2.78E-08	up	yes	Uncharacteri	pfam00462	Glutaredoxin	Glutaredoxi	GO:0045454|	GO:0005886|	GO:0009055|el	.
Manes.08G03560(	2.5943936	1.37E-24	up	yes	.	pfam10358	NT-C2	N-terminal	.	.	.	.
Manes.08G03570(	0.4993752	2.38E-08	down	yes	Protein YAB	pfam04690	YABBY	YABBY pro	GO:0007275|	GO:0005634|	GO:0046872|m	.
Manes.08G03580(	1.7651383	2.36E-08	up	no	Dihydroneop	pfam02152	FolB	Dihydroneo	GO:0046656|	GO:0005829|	GO:0004150|di	K01633
Manes.08G03600(	0.9694712	0.83738	down	no	.	pfam14184	YrvL	Regulatory	.	.	.	.
Manes.08G03610(	0.9562445	0.73612	down	no	Iron-sulfur cl	pfam07743	HSCB_C	HSCB C-ter	GO:0016226|	GO:0005813|	GO:0051087|ch	K04082
Manes.08G03640(	1.756997	2.37E-10	up	no	Protein WAV	pfam06886	TPX2	Targeting p	GO:0007163|	GO:0055028|	.	.
Manes.08G03650(	1.8953396	5.99E-12	up	no	Fasciclin-like	pfam02469	Fasciclin	Fasciclin do	.	GO:0005576|	.	.
Manes.08G03660(	0.7713948	0.00729	down	no	E3 ubiquitin-	pfam13639	zf-RING_2	Ring finger	GO:0009561|	GO:0005886|	GO:0016874|lig	K19043
Manes.08G03670(	0.5640808	3.97E-05	down	no	.	pfam03006	HlyIII	Haemolysin	.	.	.	.
Manes.08G03710(	0.862119	0.12579	down	no	Prolycopene	pfam01593	Amino_oxidase	Flavin conta	GO:0016117|	GO:0031969|	GO:0046608|ca	K09835
Manes.08G03720(	0.7267997	0.01439	down	no	ABC transpo	pfam01061	ABC2_membrane	ABC-2 type	GO:0009414|	GO:0016021|	GO:0005524|A	.
Manes.08G03730(	0.2500353	3.01E-07	down	yes	Calcium-dep	pfam00069	Pkinase	Protein kina	GO:0009738|	GO:0005737|	GO:0005524|A	K13412
Manes.08G03780(	0.6388155	9.28E-05	down	no	.	pfam04576	Zein-binding	Zein-binding	.	.	.	.
Manes.08G03790(	4.9133147	6.72E-32	up	yes	Probable carl	pfam07859	Abhydrolase_3	alpha/beta h	.	.	GO:0052689|ca	.
Manes.08G03820(	0.7393545	0.06439	down	no	Proton-coupl	pfam01490	Aa_trans	Transmemb	GO:0003333|	GO:0016021|	GO:0015171|an	K14209
Manes.08G03830(	0.7904717	0.02822	down	no	Probable glut	pfam04389	Peptidase_M28	Peptidase fa	GO:0009793|	GO:0016021|	GO:0004180|ca	K01301
Manes.08G03880(	1.1542164	0.14121	up	no	Rhomboid-lik	pfam01694	Rhomboid	Rhomboid f	.	GO:0016021|	GO:0004252|se	.
Manes.08G03940(	0.8254836	0.06007	down	no	Homogentisa	pfam01040	UbiA	UbiA prenyl	.	GO:0031969|	GO:0004659|pr	K12501
Manes.08G03960(	0.6911958	0.00019	down	no	Eukaryotic tr	pfam05470	eIF-3c_N	Eukaryotic t	GO:0001731|	GO:0016282|	GO:0003743|tra	.
Manes.08G03980(	1.2898277	0.01265	up	no	TNF recepto	pfam02176	zf-TRAF	TRAF-type	.	GO:0005737|	GO:0004842|ub	.
Manes.08G03990(	0.7461757	0.01265	down	no	Mediator of I	pfam05983	Med7	MED7 prote	GO:0006351|	GO:0016592|	GO:0001104|R	K15148
Manes.08G04000(	0.6905596	0.00015	down	no	Splicing facto	pfam10433	MMS1_N	Mono-funct	GO:0000398|	GO:0071013|	GO:0003723|R	K12830
Manes.08G04030(	0.798279	0.15127	down	no	Histone-lysin	pfam00856	SET	SET domair	.	GO:0005634|	GO:0018024|his	K11426
Manes.08G04050(	2.2726448	1.31E-19	up	yes	Lipid phosph	pfam01569	PAP2	PAP2 super	GO:0009737|	GO:0005789|	GO:0016787|hy	.
Manes.08G04060(	0.870826	0.15704	down	no	Serine/threon	pfam00149	Metallophos	Calcineurin-	GO:0006470|	GO:0005737|	GO:0046872|m	K04382
Manes.08G04070(	2.2741988	5.83E-20	up	yes	DEAD-box A	pfam00270	DEAD	DEAD/DEA	.	GO:0005777|	GO:0005524|A	K11594
Manes.08G04090(	1.1595382	0.09954	up	no	Carbon catab	pfam03372	Exo_endo_phos	Endonuclea	GO:0006397|	GO:0000932|	GO:0046872|m	K12603
Manes.08G04190(	0.4466079	0.00018	down	yes	Zinc finger p	pfam15315	FRG2	Facioscapul	GO:0007275|	GO:0005737|	GO:0046872|m	.
Manes.08G04210(	0.1649349	4.70E-18	down	yes	Dirigent prot	pfam03018	Dirigent	Dirigent-like	.	GO:0048046|	.	.
Manes.08G04230(	0.7714397	0.0074	down	no	Kinesin-relat	pfam00225	Kinesin	Kinesin mot	GO:0034613|	GO:0005737|	GO:0005524|A	K11498
Manes.08G04240(	0.9163453	0.53395	down	no	Transmembra	pfam01956	DUF106	Integral mer	.	GO:0005789|	.	.
Manes.08G04250(	2.1272403	3.42E-08	up	yes	Metal toleran	pfam01545	Cation_efflux	Cation efflu	.	GO:0016021|	GO:0008324|ca	.
Manes.08G04270(	0.9492112	0.60938	down	no	.	pfam14688	DUF4461	Domain of u	.	.	.	.
Manes.08G04280(	0.4798161	6.21E-08	down	yes	Activator of	pfam09229	Aha1_N	Activator of	GO:0032781|	GO:0005737|	GO:0001671|A	.
Manes.08G04290(	0.687208	0.00462	down	no	DNA-3-meth	pfam02245	Pur_DNA_glyco	Methylpurin	GO:0006284|	GO:0005634|	GO:0003677|D	K03652
Manes.08G04320(	0.8159258	0.12163	down	no	Nudix hydrol	pfam00293	NUDIX	NUDIX dor	GO:0042726|	GO:0009507|	GO:0047631|A	K18453
Manes.08G04380(	0.5227454	8.39E-07	down	no	E3 ubiquitin-	pfam13923	zf-C3HC4_2	Zinc finger,	.	GO:0005783|	GO:0016874|lig	K10666
Manes.08G04490(	2.935052	0.21285	up	no	11 kDa late e	pfam03760	LEA_1	Late embryo	GO:0009790|	.	.	.
Manes.08G04510(	1.546881	0.00062	up	no	G patch dom	pfam13821	DUF4187	Domain of u	.	.	GO:0003676|nu	.
Manes.08G04530(	0.6199962	0.00042	down	no	UBP1-associ	pfam00076	RRM_1	RNA recogi	GO:0009738|	GO:0005829|	GO:0017091|A	K12741
Manes.08G04550(	0.1064469	0.00099	down	yes	.	pfam05553	DUF761	Cotton fibre	.	.	.	.
Manes.08G04580(	1.3049507	0.00519	up	no	.	pfam03735	ENT	ENT domai	.	.	.	.
Manes.08G04640(	3.2643849	6.91E-33	up	yes	Zinc finger C	pfam00013	KH_1	KH domain.	.	.	GO:0003677|D	.
Manes.08G04660(	1.3409226	0.00135	up	no	TBCC domai	pfam07986	TBCC	Tubulin binc	GO:0000902|	GO:0005737|	.	K16810
Manes.08G04690(	NA	NA	--	no	Cytochrome	pfam00067	p450	Cytochrome	.	GO:0016020|	GO:0020037|he	.
Manes.08G04710(	18.440498	0.0009	up	yes	Ras-related p	pfam00071	Ras	Ras family.	GO:0006886|	GO:0045177|	GO:0005525|G	K07904
Manes.08G04740(	1.250932	0.02706	up	no	Transcription	pfam00010	HLH	Helix-loop-l	GO:0006355|	GO:0005634|	GO:0003677|D	.
Manes.08G04770(	3.6031076	1.15E-13	up	yes	Pentatricope	pfam13041	PPR_2	PPR repeat	.	GO:0009507|	.	.
Manes.08G04840(	0.4442085	0.0006	down	yes	.	pfam10325	7TM_GPCR_Srz	Serpentine t	.	.	.	.
Manes.08G04850(	0.3675164	0.00919	down	yes	Dehydration-	pfam00847	AP2	AP2 domair	GO:0006351|	GO:0005634|	GO:0003677|D	.
Manes.08G04900(	2.0393218	3.57E-11	up	yes	Guanylate ki	pfam00625	Guanylate_kin	Guanylate k	GO:0048229|	.	GO:0005524|A	.
Manes.08G04930(	0.5664153	0.05761	down	no	Remorin	pfam03763	Remorin_C	Remorin, C-	GO:0007267|	GO:0005886|	GO:0048032|ga	.
Manes.08G04950(	1.0239439	0.83109	up	no	Calcium-dep	pfam00069	Pkinase	Protein kina	GO:0009738|	GO:0005737|	GO:0005524|A	K13412
Manes.08G04960(	2.6075043	7.76E-06	up	yes	Probable gala	pfam05691	Raffinose_syn	Raffinose sy	GO:0034484|	GO:0009506|	GO:0052692|ra	K06617
Manes.08G04990(	0.2148011	0.00043	down	yes	.	pfam10322	7TM_GPCR_Sru	Serpentine t	.	.	.	.
Manes.08G05050(	0.9044083	0.43428	down	no	Exosome cor	pfam01138	RNase_PH	3' exoribon	GO:0043928|	GO:0005737|	GO:0017091|A	K12586
Manes.08G05080(	4.1542101	9.86E-10	up	yes	.	pfam09520	RE_TdeIII	Type II rest	.	.	.	.
Manes.08G05090(	1.1796362	0.07275	up	no	Zinc-metallo	pfam00675	Peptidase_M16	Insulinase (I	GO:0043171|	GO:0005739|	GO:0004222|m	K01408
Manes.08G05130(	0.1024578	3.00E-08	down	yes	.	pfam15449	Retinal	Retinal prot	.	.	.	.
Manes.08G05140(	0.9335843	0.50141	down	no	.	pfam13041	PPR_2	PPR repeat	.	.	.	.
Manes.08G05170(	1.7363825	5.93E-10	up	no	NADPH-dep	pfam07992	Pyr_redox_2	Pyridine nuc	GO:0045454|	GO:0009507|	GO:0008047|en	K00384
Manes.08G05210(	0.3666464	1.07E-15	down	yes	.	pfam11996	DUF3491	Protein of u	.	.	.	.

Manes.08G05240(	0.2530222	1.94E-28	down	yes	.	pfam07498	Rho_N	Rho termina	.	.	.	.
Manes.08G05250(	0.8063598	0.11689	down	no	Probable suga	pfam03151	TPT	Triose-phos	GO:0008643|	GO:0016021|	.	.
Manes.08G05290(	7.8788062	3.46E-90	up	yes	.	pfam04577	DUF563	Protein of u	.	.	.	.
Manes.08G05340(	0.7798896	0.01189	down	no	Folylpolyglut	pfam08245	Mur_ligase_M	Mur ligase r	GO:0006730|	GO:0005743|	GO:0005524|A	K01930
Manes.08G05400(	5.2574251	3.22E-32	up	yes	.	pfam13496	DUF4120	Domain of u	.	.	.	.
Manes.08G05500(	6.1685754	2.90E-32	up	yes	Protein EARI	pfam04647	AgrB	Accessory g	GO:0009617|	GO:0005737|	.	.
Manes.08G05510(	1.348731	0.27218	up	no	.	pfam13962	PGG	Domain of u	.	.	.	.
Manes.08G05520(	Inf	0.35135	up	no	.	pfam03772	Competence	Competence	.	.	.	.
Manes.08G05530(	35.038098	1.55E-52	up	yes	.	pfam06472	ABC_membrane_2	ABC transp	.	.	.	.
Manes.08G05570(	1.1301239	0.25272	up	no	Uric acid deg	pfam09349	OHCU_decarbox	OHCU deca	GO:0019428|	GO:0005829|	GO:0051997|2-	K13484
Manes.08G05600(	1.2454308	0.0224	up	no	Triacylglycer	pfam11815	DUF3336	Domain of u	GO:0006071|	GO:0016021|	GO:0004806|tri	K14674
Manes.08G05620(	0.7071409	0.03504	down	no	.	pfam01348	Intron_maturas2	Type II intro	.	.	.	.
Manes.08G05640(	0.501302	9.98E-07	down	no	Probable met	pfam03141	Methyltransf_29	Putative S-a	.	GO:0005768|	GO:0008168|me	.
Manes.08G05650(	1.1890156	0.06314	up	no	Regulatory p	pfam12796	Ank_2	Ankyrin rep	GO:0010434|	GO:0005737|	.	K14508
Manes.08G05680(	Inf	0.18198	up	no	.	pfam13963	Transpos_assoc	Transposase	.	.	.	.
Manes.08G05710(	24.478316	#######	up	yes	Putative meth	pfam08241	Methyltransf_11	Methyltrans	GO:0032259|	GO:0045335|	GO:0008757|S-	.
Manes.08G05740(	0.2770132	0.01936	down	yes	DNA-directe	pfam03871	RNA_pol_Rpb5_N	RNA polym	GO:0006351|	GO:0000419|	GO:0003677|D	K03013
Manes.08G05780(	0.9025622	0.38266	down	no	50S ribosom	pfam00298	Ribosomal_L11	Ribosomal p	GO:0006412|	GO:0009507|	GO:0019843|rR	K02867
Manes.08G05790(	131.16501	4.32E-17	up	yes	.	pfam12937	F-box-like	F-box-like.	.	.	.	.
Manes.08G05810(	0.7418354	0.00574	down	no	Chloroplast p	pfam10502	Peptidase_S26	Signal pepti	GO:0051604|	GO:0009507|	GO:0008233|pe	K03100
Manes.08G05820(	3.4724388	1.14E-33	up	yes	Two-compor	pfam00072	Response_reg	Response re	GO:0007623|	GO:0005634|	GO:0000156|ph	K14492
Manes.08G05840(	1.4007141	0.00022	up	no	Haloacid deh	pfam13419	HAD_2	Haloacid de	.	GO:0005739|	GO:0016787|hy	.
Manes.08G05850(	11.259934	0.01725	up	yes	Zinc finger p	pfam13912	zf-C2H2_6	C2H2-type	GO:0048235|	GO:0005634|	GO:0046872|me	.
Manes.08G05870(	0.3767646	2.58E-09	down	yes	Transcription	pfam00010	HLH	Helix-loop-l	GO:0010106|	GO:0005634|	GO:0003677|D	.
Manes.08G05880(	0.3000306	1.11E-13	down	yes	L-type lectin-	pfam00139	Lectin_legB	Legume lect	GO:0009755|	GO:0016021|	GO:0005524|A	.
Manes.08G05910(	16.556236	1.25E-41	up	yes	.	pfam12069	DUF3549	Protein of u	.	.	.	.
Manes.08G05990(	1.1955075	0.35321	up	no	Vacuolar ami	pfam01490	Aa_trans	Transmemb	GO:0003333|	GO:0000324|	GO:0015186|L-	K15015
Manes.08G06000(	0.9695058	0.78092	down	no	Vacuolar ami	pfam01490	Aa_trans	Transmemb	GO:0003333|	GO:0000324|	GO:0015186|L-	K15015
Manes.08G06010(	1.08069	0.72324	up	no	.	pfam01490	Aa_trans	Transmemb	.	.	.	.
Manes.08G06030(	1.1358834	0.22712	up	no	CDPK-relate	pfam00069	Pkinase	Protein kina	GO:0009738|	GO:0005737|	GO:0005524|A	.
Manes.08G06040(	0.3793507	3.43E-10	down	yes	.	pfam00368	HMG-CoA_red	Hydroxyme	.	.	.	.
Manes.08G06050(	0.4465665	2.58E-13	down	yes	Pentatricope	pfam13812	PPR_3	Pentatricope	.	GO:0009941|	.	.
Manes.08G06060(	0.7387987	0.32674	down	no	Alpha-glucos	pfam01055	Glyco_hydro_31	Glycosyl hy	.	.	GO:0004558|al	K15922
Manes.08G06070(	1.6594903	2.83E-06	up	no	Microtubule-	pfam07058	Myosin_HC-like	Myosin II h	GO:0007010|	GO:0005737|	GO:0008017|mi	.
Manes.08G06130(	1.9076203	9.38E-06	up	no	Serine carbox	pfam00450	Peptidase_S10	Serine carbo	.	GO:0005576|	GO:0004185|se	K16297
Manes.08G06140(	2.6503136	2.63E-27	up	yes	Probable me	pfam08711	Med26	TFIIS helica	GO:0006355|	GO:0005634|	GO:0003677|D	.
Manes.08G06150(	0.7412482	0.03741	down	no	ABC transpo	pfam00664	ABC_membrane	ABC transp	GO:0010541|	GO:0016021|	GO:0005524|A	K05658
Manes.08G06180(	1.0760353	0.46615	up	no	.	pfam05557	MAD	Mitotic chec	.	.	.	.
Manes.08G06210(	0.9237263	0.42871	down	no	Calmodulin-i	pfam00004	AAA	ATPase fam	.	GO:0009507|	GO:0005524|A	.
Manes.08G06220(	0.928956	0.52281	down	no	Transcription	pfam00249	Myb_DNA-binding	Myb-like D	GO:0007623|	GO:0005634|	GO:0003677|D	.
Manes.08G06240(	0.5751674	0.01975	down	no	.	pfam12752	SUZ	SUZ domain	.	.	.	.
Manes.08G06280(	1.9337401	6.14E-12	up	no	TGACG-sequ	pfam00170	bZIP_1	bZIP transc	.	GO:0005634|	GO:0043565|se	.
Manes.08G06290(	0.4981731	7.80E-07	down	yes	Exocyst com	pfam04091	Sec15	Exocyst con	GO:0006904|	GO:0005829|	.	.
Manes.08G06380(	22.988843	1.27E-20	up	yes	Ankyrin repe	pfam13962	PGG	Domain of u	.	GO:0005783|	.	.
Manes.08G06390(	1.1492674	0.26743	up	no	Alcohol dehy	pfam08240	ADH_N	Alcohol deh	.	GO:0005737|	GO:0004022|al	K00121
Manes.08G06400(	1.074945	0.45236	up	no	.	pfam04640	PLATZ	PLATZ tran	.	.	.	.
Manes.08G06420(	0.8740711	0.16365	down	no	V-type proto	pfam03224	V-ATPase_H_N	V-ATPase s	GO:0015991|	GO:0009507|	GO:0046961|pr	K02144
Manes.08G06460(	2.3623119	4.31E-07	up	yes	Inositol-tetra	pfam05770	Ins134_P3_kin	Inositol 1, 3	GO:0052746|	GO:0005829|	GO:0005524|A	K00913
Manes.08G06470(	1.9884284	5.53E-14	up	no	THO comple	pfam05615	THOC7	Tho comple	GO:0006397|	GO:0000347|	GO:0003723|R	K13176
Manes.08G06500(	2.5310938	0.03408	up	no	NAC domain	pfam02365	NAM	No apical m	GO:0006355|	GO:0005634|	GO:0003677|D	.
Manes.08G06530(	0.7541891	0.03127	down	no	40S ribosom	pfam01251	Ribosomal_S7e	Ribosomal p	GO:0006412|	GO:0005840|	GO:0003735|str	.
Manes.08G06570(	2.1503859	3.28E-17	up	yes	Protein PAT1	pfam09770	PAT1	Topoisomer	GO:0033962|	GO:0000932|	GO:0003723|R	K12617
Manes.08G06580(	0.8882479	0.38211	down	no	Protein Mpv1	pfam04117	Mpv17_PMP22	Mpv17 / PM	.	GO:0016021|	.	K13348
Manes.08G06590(	3.4987407	8.70E-38	up	yes	.	pfam05678	VQ	VQ motif. T	.	.	.	.
Manes.08G06630(	1.741253	3.64E-09	up	no	Cellulose syn	pfam03552	Cellulose_synt	Cellulose sy	GO:0071555|	GO:0005783|	GO:0016760|ce	.
Manes.08G06640(	0.537239	1.52E-05	down	no	Retrovirus-re	pfam07727	RVT_2	Reverse tran	GO:0015074|	.	GO:0004190|as	.
Manes.08G06730(	0.3660788	2.52E-05	down	yes	.	pfam14383	VARLMGL	DUF761-ass	.	.	.	.
Manes.08G06780(	1.4691903	0.00433	up	no	.	pfam14547	Hydrophob_seed	Hydrophobi	.	.	.	.
Manes.08G06800(	0.6015164	0.00175	down	no	RNA polyme	pfam03031	NIF	NLI interact	GO:0006355|	GO:0005634|	GO:0046872|me	K18999
Manes.08G06820(	0.1819282	3.26E-09	down	yes	.	pfam13041	PPR_2	PPR repeat	.	.	.	.
Manes.08G06830(	0.7356146	0.01165	down	no	E3 ubiquitin-	pfam00240	ubiquitin	Ubiquitin fa	GO:0010150|	GO:0005737|	GO:0030246|ca	K10591
Manes.08G06850(	6.4953968	0.14757	up	no	.	pfam05701	WEMBL	Weak chlor	.	.	.	.
Manes.08G06860(	1.7060238	5.29E-07	up	no	Acetyl-CoA a	pfam00108	Thiolase_N	Thiolase, N-	GO:0008299|	GO:0005829|	GO:0003985|ac	K00626
Manes.08G06900(	0.6422854	0.00045	down	no	.	pfam15253	STIL_N	SCL-interru	.	.	.	.
Manes.08G06910(	1.1063939	0.26608	up	no	Gamma amin	pfam00202	Aminotran_3	Aminotrans	.	GO:0009507|	GO:0030170|py	K16871
Manes.08G06990(	0.540114	3.94E-07	down	no	SNF1-related	pfam04739	AMPKBI	5'-AMP-acti	GO:0005975|	.	GO:0005524|A	.
Manes.08G07060(	2.6334811	0.21919	up	no	.	pfam03542	Tuberin	Tuberin. Tu	.	.	.	.
Manes.08G07070(	0.4534187	0.01135	down	yes	CASP-like pr	pfam04535	DUF588	Domain of u	.	GO:0016021|	.	.
Manes.08G07080(	1.4233715	0.00023	up	no	Heterogeneo	pfam00076	RRM_1	RNA recogn	GO:0006397|	GO:0005737|	GO:0000166|nu	K14411
Manes.08G07140(	1.7322416	0.0006	up	no	Chaperone p	pfam06022	Cir_Bir_Yir	Plasmodium	.	GO:0016021|	.	.
Manes.08G07230(	1.527135	0.00227	up	no	.	pfam01039	Carboxyl_trans	Carboxyl tra	.	.	.	.
Manes.08G07270(	NA	NA	--	no	.	pfam14392	zf-CCHC_4	Zinc knuckl	.	.	.	.
Manes.08G07280(	4.3926298	3.40E-08	up	yes	Probable me	pfam03000	NPH3	NPH3 famil	.	GO:0005886|	.	.
Manes.08G07320(	0.4697725	0.00427	down	yes	.	pfam13868	Trichoplein	tumor suppr	.	.	.	.
Manes.08G07340(	0.9338962	0.60029	down	no	Glucan endo-	pfam00332	Glyco_hydro_17	Glycosyl hy	GO:0005975|	GO:0031225|	GO:0042973|gl	.
Manes.08G07350(	32.281417	1.53E-07	up	yes	.	pfam15323	Ashwin	Developme	.	.	.	.
Manes.08G07360(	0.2123053	0.37476	down	no	E3 ubiquitin-	pfam07002	Copine	Copine. Thi	GO:0009850|	GO:0005634|	GO:0016874|lig	K16280
Manes.08G07430(	1.6853837	1.46E-08	up	no	GPN-loop G	pfam03029	ATP_bind_1	Conserved	GO:0007264|	GO:0005737|	GO:0005525|G	K06883
Manes.08G07440(	0.7057639	0.57909	down	no	.	pfam14093	DUF4271	Domain of u	.	.	.	.
Manes.08G07460(	0.2550916	2.37E-14	down	yes	Copper trans	pfam00403	HMA	Heavy-meta	GO:0006825|	GO:0000785|	GO:0046872|me	K07213
Manes.08G07540(	0.8542414	0.09568	down	no	Serine/threon	pfam00069	Pkinase	Protein kina	GO:0006355|	.	GO:0005524|A	K08287
Manes.08G07620(	0.8695051	0.21508	down	no	Retinal-bindi	pfam00650	CRAL_TRIO	CRAL/TRIO	GO:0050896|	GO:0016021|	.	.
Manes.08G07650(	1.1681419	0.18855	up	no	Farnesyl pyro	pfam00348	polyprenyl_synt	Polyprenyl s	GO:0006695|	GO:0005737|	GO:0004161|di	.
Manes.08G07670(	1.4514638	4.28E-05	up	no	.	pfam02500	DNA_pack_N	Probable D	.	.	.	.
Manes.08G07700(	0.9036982	0.29931	down	no	Ras-related p	pfam00071	Ras	Ras family.	GO:0009873|	GO:0000139|	GO:0005525|G	K07901
Manes.08G07720(	0.6513106	0.00032	down	no	WAT1-relate	pfam00892	EamA	EamA-like t	.	GO:0016021|	GO:0022857|tra	.
Manes.08G07730(	0.6751252	0.0001	down	no	.	pfam00520	Ion_trans	Ion transpo	.	.	.	.
Manes.08G07790(	1.7190965	1.82E-09	up	no	Myb-related	pfam00249	Myb_DNA-binding	Myb-like D	.	GO:0005634|	GO:0003677|D	.

Manes.08G07800(	2.0447909	5.56E-14	up	yes	U-box domai	pfam04564	U-box	U-box doma	GO:0042742|(	GO:0005737|(	GO:0016874|lig	.
Manes.08G07870(	0.5967732	0.00078	down	no	Protein NRT	pfam00854	PTR2	POT family.	GO:0006857|(	GO:0016021|	GO:0005215|tra	.
Manes.08G07900(	0.6817557	0.00354	down	no	Metallothion(	pfam01439	Metallothio_2	Metallothio	.	.	GO:0046872|m(	.
Manes.08G07960(	0.6580074	0.01244	down	no	Aldo-keto re(	pfam00248	Aldo_ket_red	Aldo/keto r(	GO:0055114|(	GO:0009507|(	GO:0008106|al(	.
Manes.08G08010(	0.5714227	5.75E-05	down	no	Trafficking p	pfam04099	Sybindin	Sybindin-lik	GO:0006888|(	GO:0005801|(	GO:0017112|Ra	.
Manes.08G08020(	1.2052102	0.12607	up	no	ABC transpo	pfam00005	ABC_tran	ABC transp	GO:0006810|t	GO:0005737|(	GO:0005524|A'	.
Manes.08G08030(	0.2499146	1.02E-15	down	yes	Expansin-A4	pfam01357	Pollen_allerg_1	Pollen allerg	GO:0009664|(	GO:0005829|(	.	.
Manes.08G08040(	0.3598197	1.42E-06	down	yes	Ultraviolet-B	pfam00415	RCC1	Regulator o	GO:0009649|(	GO:0000785|(	GO:0003682|ch	.
Manes.08G08060(	0.4631457	0.36963	down	no	.	pfam11153	DUF2931	Protein of u	.	.	.	.
Manes.08G08120(	0.6905757	0.03556	down	no	Vacuolar ami	pfam01490	Aa_trans	Transmemb	GO:0003333|(	GO:0000324|	GO:0015186|L-	K15015
Manes.08G08140(	4.4933384	4.17E-26	up	yes	E3 ubiquitin-	pfam06547	DUF1117	Protein of u	GO:0016567|(	.	GO:0016874|lig	.
Manes.08G08180(	0.6113659	0.01523	down	no	.	pfam11279	DUF3080	Protein of u	.	.	.	.
Manes.08G08200(	0.6018511	0.00011	down	no	Probable DE	pfam06273	eIF-4B	Plant specifi	.	.	GO:0005524|A'	.
Manes.08G08220(	1.5667304	0.00291	up	no	.	pfam04196	Bunya_RdRp	Bunyavirus	.	.	.	.
Manes.08G08290(	2.1413302	3.99E-17	up	yes	Phosphatidyl	pfam14360	PAP2_C	PAP2 super	GO:0030148|(	GO:0016021|	GO:0045140|in(	.
Manes.08G08320(	0.570794	0.00067	down	no	Transcription	pfam13921	Myb_DNA-bind_6	Myb-like DI	GO:0009738|(	GO:0005634|	GO:0003677|DI	K09422
Manes.08G08370(	0.7565505	0.00475	down	no	.	pfam02357	NusG	Transcriptio	.	.	.	.
Manes.08G08410(	0.5216894	0.02565	down	no	Fructokinase-	pfam08153	NGP1NT	NGP1NT (N	GO:0009658|(	GO:0009507|(	GO:0016301|ki	.
Manes.08G08420(	0.6022445	0.00099	down	no	Uncharacteri	pfam10250	O-FucT	GDP-fucose	.	GO:0005768|(	GO:0016757|tra	.
Manes.08G08440(	6.2964528	2.71E-36	up	yes	Uncharacteri	pfam13419	HAD_2	Haloacid de	GO:0016311|(	GO:0005737|(	GO:0008252|nu	K18551
Manes.08G08500(	1.0092218	0.92587	up	no	Transmembra	pfam05915	DUF872	Eukaryotic	.	GO:0016021|	.	.
Manes.08G08600(	0.7504965	0.03884	down	no	Mitochondria	pfam00153	Mito_carr	Mitochondr	GO:0055085|t	GO:0016021|	GO:0005509|ca	K14684
Manes.08G08650(	0.5966707	0.00145	down	no	Aldo-keto re(	pfam00248	Aldo_ket_red	Aldo/keto r(	GO:0055114|(	GO:0009507|(	GO:0008106|al(	.
Manes.08G08660(	1.8030892	2.50E-08	up	no	Uncharacteri	pfam02330	MAM33	Mitochondr	.	GO:0005759|	.	K15414
Manes.08G08690(	2.4904088	2.29E-14	up	yes	E3 ubiquitin-	pfam09770	PAT1	Topoisomer	GO:0007162|(	.	GO:0016874|lig	K15685
Manes.08G08700(	0.3027171	2.23E-20	down	yes	Reticulon-lik(	pfam02453	Reticulon	Reticulon. F	.	GO:0005789|(	.	.
Manes.08G08730(	0.7004214	0.00067	down	no	mRNA-cappi	pfam01331	mRNA_cap_enzyr	mRNA capp	GO:0006370|'	GO:0005634|	GO:0005525|G'	K13917
Manes.08G08740(	0.8977757	0.35516	down	no	mRNA-cappi	pfam01331	mRNA_cap_enzyr	mRNA capp	GO:0006370|'	GO:0005654|	GO:0005525|G'	K13917
Manes.08G08750(	0.5265785	9.53E-05	down	no	.	pfam06127	DUF962	Protein of u	.	.	.	.
Manes.08G08760(	1.4078513	0.00886	up	no	Probable LRI	pfam00069	Pkinase	Protein kina	.	GO:0016021|	GO:0005524|A'	.
Manes.08G08790(	0.797108	0.14778	down	no	.	pfam13041	PPR_2	PPR repeat	.	.	.	.
Manes.08G08820(	0.9105884	0.39126	down	no	.	pfam05641	Agenet	Agenet dom	.	.	.	.
Manes.08G08900(	0.7475209	0.03723	down	no	Scarecrow-lil	pfam03514	GRAS	GRAS doma	GO:0006355|(	GO:0005634|	GO:0003700|tra	.
Manes.08G08910(	6.0635329	7.23E-25	up	yes	BAHD acyltr	pfam02458	Transferase	Transferase	GO:0010143|(	GO:0005737|(	GO:0016747|tra	.
Manes.08G08920(	1.50928	5.74E-06	up	no	GDP-mannos	pfam00534	Glycos_transf_1	Glycosyl tra	GO:0040007|(	.	GO:0016757|tra	K12583
Manes.08G08930(	0.2259766	9.84E-07	down	yes	Nucleobase-a	pfam00860	Xan_ur_permease	Permease fa	GO:0055085|t	GO:0016021|	GO:0005215|tra	.
Manes.08G08940(	1.3469617	0.00482	up	no	Pentatricope	pfam13041	PPR_2	PPR repeat	.	GO:0009507|(	.	.
Manes.08G08990(	1.0001772	1	up	no	Glycerol-3-pl	pfam01553	Acyltransferase	Acyltransfer	GO:0010143|(	GO:0016021|	GO:0090447|gl	K13508
Manes.08G09030(	0.9747327	1	down	no	Probable LRI	pfam00069	Pkinase	Protein kina	.	GO:0016021|	GO:0005524|A'	.
Manes.08G09040(	26.028171	1.76E-08	up	yes	Lactosylcera	pfam04572	Gb3_synth	Alpha 1,4-g	GO:0001576|(	GO:0070062|(	GO:0008378|ga	K01988
Manes.08G09050(	Inf	0.00126	up	yes	.	pfam08664	YcbB	YcbB doma	.	.	.	.
Manes.08G09080(	0.7591848	0.75793	down	no	Probable LRI	pfam00069	Pkinase	Protein kina	.	GO:0016021|	GO:0005524|A'	.
Manes.08G09110(	0.9746591	0.79156	down	no	Alpha-amylas	pfam00686	CBM_20	Starch bindi	GO:0005975|(	.	GO:0004556|al	.
Manes.08G09140(	0.7066205	0.01429	down	no	.	pfam15402	Spc7_N	N-terminus	.	.	.	.
Manes.08G09170(	0.700921	0.01601	down	no	Chlorophyll a	pfam00504	Chloroa_b-bind	Chlorophyll	GO:0009765|(	GO:0009507|(	GO:0016168|ch	K08915
Manes.08G09180(	0.8103869	0.15458	down	no	High mobility	pfam00505	HMG_box	HMG (high	GO:0006333|(	GO:0000785|(	GO:0003682|ch	K10802
Manes.08G09200(	2.8510545	2.16E-07	up	yes	RING finger	pfam13639	zf-RING_2	Ring finger	GO:0051865|(	.	GO:0003677|DI	.
Manes.08G09220(	1.7526468	1.13E-08	up	no	GDSL estera	pfam00657	Lipase_GDSL	GDSL-like l	GO:0016042|l	GO:0048046|(	GO:0016788|hy	.
Manes.08G09230(	0.5387735	7.17E-09	down	no	Rhodanese-li	pfam00581	Rhodanese	Rhodanese-l	.	GO:0009507|(	.	.
Manes.08G09240(	0.5087513	4.88E-10	down	no	Serine/threon	pfam07714	Pkinase_Tyr	Protein tyro	.	.	GO:0005524|A'	.
Manes.08G09250(	Inf	0.1623	up	no	Serine/threon	pfam07714	Pkinase_Tyr	Protein tyro	.	.	GO:0005524|A'	.
Manes.08G09260(	0.7999882	0.02573	down	no	PHD finger p	pfam12165	DUF3594	Domain of u	GO:0016568|(	GO:0005634|	GO:0035064|m(	.
Manes.08G09270(	Inf	0.00217	up	yes	Protein SRG	pfam03171	2OG-FeII_Oxy	2OG-Fe(II)	GO:0010260|(	.	GO:0046872|m(	.
Manes.08G09290(	7.8801871	1.63E-13	up	yes	Trihelix trans	pfam13837	Myb_DNA-bind_4	Myb/SANT-	GO:0006355|(	GO:0016592|(	GO:0043565|se	.
Manes.08G09320(	0.7650839	0.00647	down	no	Acyl-coenzyr	pfam02771	Acyl-CoA_dh_N	Acyl-CoA d	GO:0009793|(	GO:0009514|	GO:0003995|ac	K00232
Manes.08G09350(	0.9296041	0.52673	down	no	Phosphoinosi	pfam02383	Syja_N	SacI homolo	.	GO:0005783|(	GO:0052866|ph	.
Manes.08G09360(	0.6760143	0.00021	down	no	Bifunctional	pfam00462	Glutaredoxin	Glutaredoxi	GO:0006812|(	GO:0009507|(	GO:0051537|2	.
Manes.08G09380(	0.5578505	8.15E-05	down	no	.	pfam02030	Lipoprotein_8	Hypothetica	.	.	.	.
Manes.08G09390(	0.7105579	0.00055	down	no	AMP deamin	pfam00962	A_deaminase	Adenosine/A	GO:0009793|(	GO:0005829|(	GO:0003876|Al	K01490
Manes.08G09430(	0.6797133	0.00177	down	no	DnaJ protein	pfam00226	DnaJ	DnaJ domai	GO:0034605|(	GO:0005788|(	GO:0016491|ox	.
Manes.08G09460(	0.4709419	0.00706	down	yes	Receptor-like	pfam13855	LRR_8	Leucine rich	GO:0009755|l	GO:0016021|	GO:0004672|pr	.
Manes.08G09480(	0.2143264	0.00466	down	yes	Transcription	pfam00010	HLH	Helix-loop-l	GO:0007275|(	GO:0005634|	GO:0003677|DI	.
Manes.08G09490(	1.146452	0.1703	up	no	Pre-mRNA-s	pfam02847	MA3	MA3 domai	GO:0006397|(	GO:0005681|(	GO:0003723|RI	K13100
Manes.08G09500(	11.326648	5.42E-19	up	yes	Ammonium t	pfam00909	Ammonium_trans	Ammonium	GO:0015696|(	GO:0016021|	GO:0015398|hi	K03320
Manes.08G09510(	0.2596883	1.90E-08	down	yes	.	pfam04936	DUF658	Protein of u	.	.	.	.
Manes.08G09550(	0.8507749	0.86916	down	no	Nodulation-s	pfam03514	GRAS	GRAS doma	GO:0009877|(	GO:0005783|(	.	.
Manes.08G09560(	3.3987783	0.00145	up	yes	Gamma-inter	pfam03227	GILT	Gamma inte	GO:0042590|(	GO:0030054|(	GO:0016667|ox	K08059
Manes.08G09580(	9.3010054	1.86E-05	up	yes	Ferritin-3, ch	pfam00210	Ferritin	Ferritin-like	GO:0006879|(	GO:0009507|(	GO:0008199|fe	K00522
Manes.08G09660(	0.6962053	0.00044	down	no	MATE efflux	pfam00223	PsaA_PsaB	Photosysten	.	GO:0031969|(	GO:0015297|an	.
Manes.08G09670(	Inf	0.9009	up	no	.	pfam00434	VP7	Glycoprotei	.	.	.	.
Manes.08G09690(	13.71563	6.13E-97	up	yes	Dehydration-	pfam00847	AP2	AP2 domair	GO:0045893|(	GO:0005634|	GO:0003677|DI	.
Manes.08G09710(	4.4412222	0.00576	up	yes	Macrophage	pfam01187	MIF	Macrophage	.	GO:0005615|(	GO:0004167|dc	.
Manes.08G09720(	0.8674483	0.15832	down	no	Patellin-6	pfam00650	CRAL_TRIO	CRAL/TRIC	GO:0007049|(	GO:0005737|(	GO:0008289|lip	.
Manes.08G09730(	Inf	0.9009	up	no	Aldose reduc	pfam00248	Aldo_ket_red	Aldo/keto r(	.	.	GO:0004032|al(	.
Manes.08G09760(	1.5266616	6.68E-06	up	no	.	pfam05038	Cytochrom_B558a	Cytochrome	.	.	.	.
Manes.08G09780(	1.1577739	0.1472	up	no	.	pfam07082	DUF1350	Protein of u	.	.	.	.
Manes.08G09790(	1.6238999	1.18E-07	up	no	.	pfam10649	DUF2478	Protein of u	.	.	.	.
Manes.08G09800(	Inf	0.17828	up	no	.	pfam10896	DUF2714	Protein of u	.	.	.	.
Manes.08G09810(	0.7660709	0.01336	down	no	Hsp70 nucle(	pfam08609	Fes1	Nucleotide (	GO:0071629|(	GO:0022626|(	GO:0000774|ad	.
Manes.08G09870(	0.6036997	1.38E-06	down	no	Photosystem	pfam13326	PSII_Pbs27	Photosysten	GO:0010207|(	GO:0009507|(	.	K08902
Manes.08G09910(	13.700718	#######	up	yes	.	pfam14009	DUF4228	Domain of u	.	.	.	.
Manes.08G09920(	0.5999694	0.00918	down	no	Heavy metal-	pfam00403	HMA	Heavy-meta	GO:0010286|l	GO:0031965|(	GO:0046872|m(	.
Manes.08G09940(	3.6787376	0.00014	up	yes	.	pfam05553	DUF761	Cotton fibre	.	.	.	.
Manes.08G09960(	0.6189544	0.01941	down	no	Protein vip1	pfam14259	RRM_6	RNA recogi	.	GO:0005829|(	GO:0000166|nu	.
Manes.08G09990(	2.9561018	0.00225	up	yes	Nudix hydrol	pfam00293	NUDIX	NUDIX dor	.	GO:0005739|(	GO:0016787|hy	.
Manes.08G10020(	0.9340327	0.50088	down	no	.	pfam14351	DUF4401	Domain of u	.	.	.	.
Manes.08G10040(	0.8328283	0.1196	down	no	Sterol 14-der	pfam00067	p450	Cytochrome	GO:0070988|(	GO:0005783|(	GO:0020037|he	K05917
Manes.08G10070(	0.8276648	0.40352	down	no	Probable beta	pfam03016	Exostosin	Exostosin fa	GO:0071555|(	GO:0000139|(	GO:0080116|gl	.

Manes.08G10090(	2.4528023	8.10E-21	up	yes	Cytochrome l	pfam00355	Rieske	Rieske [2Fe	.	GO:0016021|	GO:0051537|2	.
Manes.08G10130(	3.4589466	2.90E-37	up	yes	Ethylene-resp	pfam00847	AP2	AP2 domair	GO:0071497|(	GO:0005634|	GO:0003677|D	.
Manes.08G10160(	0.6325156	3.29E-06	down	no	Protein MEI2	pfam04059	RRM_2	RNA recogn	.	.	GO:0000166|nu	.
Manes.08G10170(	0.9173742	0.6128	down	no	.	pfam13837	Myb_DNA-bind_4	Myb/SANT-	.	.	.	.
Manes.08G10180(	0.7085487	0.00197	down	no	.	pfam03323	GerA	Bacillus/Clo	.	.	.	.
Manes.08G10190(	0.7213169	0.00077	down	no	ADP-ribosyla	pfam01412	ArfGap	Putative GT	GO:0006897|(	GO:0005829|	GO:0005096|G	K12489
Manes.08G10200(	0.350231	1.60E-14	down	yes	Inactive leuci	pfam00069	Pkinase	Protein kina	GO:0009755|l	GO:0016021|	GO:0005524|A	.
Manes.08G10230(	8.9444134	2.13E-17	up	yes	.	pfam00405	Transferrin	Transferrin.	.	.	.	.
Manes.08G10250(	0.8891091	0.24922	down	no	.	pfam00536	SAM_1	SAM domai	.	.	.	.
Manes.08G10330(	1.4158359	0.60144	up	no	.	pfam09402	MSC	Man1-Src1p	.	.	.	.
Manes.08G10360(	0.4969024	3.06E-05	down	yes	Somatic emb	pfam08263	LRRNT_2	Leucine rich	GO:0009742|l	GO:0005789|	GO:0005524|A	K13418
Manes.08G10370(	0.8623323	0.17489	down	no	Uncharacteriz	pfam03114	BAR	BAR domai	.	GO:0005886|	.	.
Manes.08G10390(	6.6660947	0.07712	up	no	.	pfam01529	zf-DHHC	DHHC palm	.	.	.	.
Manes.08G10410(	0.5785653	4.00E-08	down	no	Zinc finger C	pfam00642	zf-CCCH	Zinc finger (	.	GO:0005634|	GO:0003677|D	.
Manes.08G10440(	0.4105207	0.00235	down	yes	Putative auxi	pfam03547	Mem_trans	Membrane t	GO:0010315|a	GO:0005783|	GO:0010329|au	K13947
Manes.08G10450(	1.5103154	4.19E-06	up	no	CBS domain-	pfam05216	UNC-50	UNC-50 far	.	GO:0016021|	.	.
Manes.08G10470(	0.9793461	0.85823	down	no	Protein TRIG	pfam00005	ABC_tran	ABC transp	GO:0006869|l	GO:0009507|	GO:0005524|A	.
Manes.08G10490(	0.9408415	0.55957	down	no	Protein PELG	pfam03465	eRF1_3	eRF1 domai	GO:0070966|r	GO:0005737|	GO:0004519|en	K06965
Manes.08G10500(	1.5116666	0.02525	up	no	.	pfam06011	TRP	Transient re	.	.	.	.
Manes.08G10530(	0.4765184	3.26E-12	down	yes	Protein CHR	pfam00628	PHD	PHD-finger.	GO:0016568|c	GO:0005634|	GO:0005524|A	.
Manes.08G10540(	3.0242215	5.15E-07	up	yes	.	pfam14009	DUF4228	Domain of u	.	.	.	.
Manes.08G10580(	1.9402746	1.60E-13	up	no	Transmembra	pfam03619	Solute_trans_a	Organic solu	.	GO:0016021|	.	.
Manes.08G10590(	2.2637804	2.94E-09	up	yes	.	pfam02355	SecD_SecF	Protein expo	.	.	.	.
Manes.08G10640(	0.7594823	0.02709	down	no	Germin-like p	pfam00190	Cupin_1	Cupin. This	.	GO:0048046|	GO:0030145|ma	.
Manes.08G10650(	0.8136789	0.09618	down	no	Putative mem	pfam03062	MBOAT	MBOAT, m	GO:0015793|g	GO:0005829|	GO:0008374|O-	.
Manes.08G10660(	1.2098102	0.08053	up	no	Flap endonuc	pfam00867	XPG_I	XPG I-regic	GO:0006284|l	GO:0005739|	GO:0008409|5'-	K04799
Manes.08G10680(	0.5190251	1.44E-05	down	no	Palmitoleoyl-	pfam03283	PAE	Pectinacetyl	GO:0030178|n	GO:0005576|	GO:1990699|pa	.
Manes.08G10690(	5.5756257	5.81E-83	up	yes	Transcription	pfam00249	Myb_DNA-binding	Myb-like DI	GO:0006355|r	GO:0005634|	GO:0003677|D	K09422
Manes.08G10700(	1.7668627	4.64E-10	up	no	.	pfam09528	Ehrlichia_rpt	Ehrlichia tar	.	.	.	.
Manes.08G10750(	0.8017119	0.06767	down	no	G patch doma	pfam01585	G-patch	G-patch dor	.	.	GO:0046872|me	.
Manes.08G10760(	0.780952	0.05062	down	no	Putative L-cy	pfam00266	Aminotran_5	Aminotransf	.	.	GO:0016829|ly	.
Manes.08G10770(	1.7008299	3.46E-05	up	no	.	pfam14672	LCE	Late cornific	.	.	.	.
Manes.08G10780(	2.0989078	1.60E-16	up	yes	Meiosis arres	pfam01936	NYN	NYN domai	GO:0051321|r	GO:0005777|	GO:0000166|nu	.
Manes.08G10790(	0.4594405	5.86E-13	down	yes	.	pfam01576	Myosin_tail_1	Myosin tail.	.	.	.	.
Manes.08G10810(	0.4685085	2.98E-13	down	yes	Probable rece	pfam00069	Pkinase	Protein kina	.	GO:0016021|	GO:0005524|A	.
Manes.08G10820(	3.1344469	5.57E-32	up	yes	S-adenosylme	pfam02772	S-AdoMet_synt_M	S-adenosyln	GO:0006730|c	GO:0005737|	GO:0005524|A	.
Manes.08G10850(	0.9195216	0.39752	down	no	Shaggy-relate	pfam00069	Pkinase	Protein kina	GO:0042538|l	GO:0005829|	GO:0005524|A	K00924
Manes.08G10880(	Inf	1.50E-11	up	yes	Polygalacturo	pfam00295	Glyco_hydro_28	Glycosyl hy	GO:0005975|c	GO:0005618|	GO:0004650|pc	.
Manes.08G10920(	0.5326271	0.0018	down	no	WAT1-relate	pfam00892	EamA	EamA-like t	.	GO:0016021|	GO:0022857|tra	.
Manes.08G10930(	31.444078	2.57E-85	up	yes	Galactinol sy	pfam01501	Glyco_transf_8	Glycosyl tra	GO:0006012|g	GO:0005737|	GO:0047216|in	K18819
Manes.08G10940(	1.392657	0.36902	up	no	Galactinol sy	pfam01501	Glyco_transf_8	Glycosyl tra	GO:0006012|g	GO:0005737|	GO:0047216|in	K18819
Manes.08G10950(	1.0490943	0.78965	up	no	Golgi apparat	pfam05832	DUF846	Eukaryotic p	GO:0007030|(	GO:0031901|	.	.
Manes.08G10990(	1.461652	0.00178	up	no	.	pfam04398	DUF538	Protein of u	.	.	.	.
Manes.08G11000(	0.971766	0.84514	down	no	.	pfam03959	FSH1	Serine hydro	.	.	.	.
Manes.08G11010(	1.8733844	1.77E-09	up	no	Importin sub	pfam01749	IBB	Importin bet	GO:0006606|l	GO:0005634|	GO:0008565|pr	.
Manes.08G11020(	0.7592031	0.01559	down	no	Zinc phospho	pfam12706	Lactamase_B_2	Beta-lactam	GO:0072684|r	GO:0005739|	GO:0004519|en	K00784
Manes.08G11040(	0.2764835	0.03924	down	no	.	pfam13896	Glyco_transf_49	Glycosyl-tra	.	.	.	.
Manes.08G11070(	0.589764	1.73E-07	down	no	Solute carrier	pfam06963	FPN1	Ferroportin1	GO:0006879|c	GO:0009941|	GO:0005381|irc	.
Manes.08G11090(	0.6235997	0.00148	down	no	.	pfam04795	PAPA-1	PAPA-1-lik	.	.	.	.
Manes.08G11100(	0.2913016	1.89E-06	down	yes	Homeobox p	pfam03791	KNOX2	KNOX2 dor	GO:0006355|r	GO:0005634|	GO:0043565|se	.
Manes.08G11130(	0.3377467	2.08E-06	down	yes	.	pfam13959	DUF4217	Domain of u	.	.	.	.
Manes.08G11150(	1.9194002	4.30E-12	up	no	Lon protease	pfam05362	Lon_C	Lon proteas	GO:0034599|c	GO:0005759|	GO:0005524|A	K08675
Manes.08G11160(	5.0329084	2.05E-21	up	yes	.	pfam10595	UPF0564	Uncharacter	.	.	.	.
Manes.08G11250(	0.4889251	2.53E-10	down	yes	.	pfam07816	DUF1645	Protein of u	.	.	.	.
Manes.08G11280(	7.4906812	3.33E-32	up	yes	.	pfam07887	Calmodulin_bind	Calmodulin	.	.	.	.
Manes.08G11300(	1.1928361	0.33503	up	no	DnaJ homolo	pfam00226	DnaJ	DnaJ domai	.	GO:0005634|	.	.
Manes.08G11310(	0.9804842	0.86169	down	no	Importin sub	pfam01749	IBB	Importin bet	GO:0006606|l	GO:0005618|	GO:0008565|pr	.
Manes.08G11320(	6.9074675	1.17E-10	up	yes	.	pfam10328	7TM_GPCR_Srx	Serpentine t	.	.	.	.
Manes.08G11330(	0.4323717	0.04029	down	no	GATA transc	pfam00320	GATA	GATA zinc	GO:0006351|t	GO:0005634|	GO:0043565|se	.
Manes.08G11370(	2.0148087	4.38E-13	up	yes	E3 ubiquitin-	pfam13639	zf-RING_2	Ring finger	GO:0051299|c	GO:0005652|	GO:0005521|la	K10631
Manes.08G11400(	0.6077872	6.09E-07	down	no	Probable prot	pfam00481	PP2C	Protein pho	GO:0006470|p	GO:0005886|	GO:0046872|me	.
Manes.08G11410(	5.0044149	0.22618	up	no	Wall-associat	pfam13947	GUB_WAK_bind	Wall-associa	.	GO:0016021|	GO:0005524|A	.
Manes.08G11430(	Inf	0.9009	up	no	Wall-associat	pfam13947	GUB_WAK_bind	Wall-associa	.	GO:0016021|	GO:0005524|A	.
Manes.08G11440(	0.7626513	0.00615	down	no	Eukaryotic tr	pfam13868	Trichoplein	tumor suppr	GO:0001731|l	GO:0005829|	GO:0003743|tra	K03254
Manes.08G11450(	1.4321736	7.48E-05	up	no	Topless-relat	pfam00400	WD40	WD domain	GO:0006355|r	GO:0005634|	.	.
Manes.08G11470(	2.6677339	5.56E-11	up	yes	Phospholipas	pfam13091	PLDc_2	PLD-like do	GO:0016042|l	.	GO:0070290|N-	K01115
Manes.08G11480(	1.2092064	0.14872	up	no	.	pfam13414	TPR_11	TPR repeat.	.	.	.	.
Manes.08G11490(	20.567459	1.05E-79	up	yes	F-box protein	pfam14299	PP2	Phloem prot	.	.	GO:0030246|ca	.
Manes.08G11500(	35.517596	9.15E-38	up	yes	F-box protein	pfam14299	PP2	Phloem prot	.	.	GO:0030246|ca	.
Manes.08G11510(	0.5811159	0.00013	down	no	.	pfam07173	DUF1399	Protein of u	.	.	.	.
Manes.08G11550(	Inf	0.9009	up	no	Probable pect	pfam01095	Pectinesterase	Pectinestera	GO:0042545|c	GO:0005618|	GO:0045330|as	K01051
Manes.08G11560(	0.7485436	0.02545	down	no	.	pfam09135	Alb1	Alb1. Alb1 i	.	.	.	.
Manes.08G11580(	12.717628	0.00034	up	yes	Putative inve	pfam04043	PMEI	Plant inverta	.	GO:0005576|	GO:0004857|en	.
Manes.08G11600(	NA	NA	--	no	Receptor-like	pfam13855	LRR_8	Leucine rich	GO:0009755|l	GO:0016021|	GO:0004672|pr	.
Manes.08G11640(	2.2744516	0.00028	up	yes	Serine/threon	pfam00149	Metallophos	Calcineurin-	GO:0016311|c	.	GO:0046872|me	K06269
Manes.08G11660(	0.5941831	0.00044	down	no	.	pfam05564	Auxin_repressed	Dormancy/a	.	.	.	.
Manes.08G11690(	1.2954564	0.00398	up	no	.	pfam04588	HIG_1_N	Hypoxia ind	.	.	.	.
Manes.08G11750(	1.1291415	0.43406	up	no	Tafazzin	pfam01553	Acyltransferase	Acyltransfer	GO:0035965|c	GO:0016021|	GO:0008374|O-	K13511
Manes.08G11780(	1.4525364	7.62E-05	up	no	REF/SRPP-li	pfam05755	REF	Rubber elon	GO:0019915|l	GO:0005811|	.	.
Manes.08G11800(	6.1557464	6.34E-36	up	yes	Protein RALI	pfam05498	RALF	Rapid ALka	GO:0019722|c	GO:0048046|	GO:0005179|ho	.
Manes.08G11840(	0.8840109	0.26049	down	no	Copper trans	pfam00403	HMA	Heavy-meta	GO:0006825|c	GO:0000785|	GO:0046872|me	K07213
Manes.08G11850(	13.664494	0.00645	up	yes	Respiratory b	pfam08030	NAD_binding_6	Ferric reduc	.	GO:0016021|	GO:0005509|ca	K13447
Manes.08G11900(	2.4774792	4.04E-17	up	yes	Putative prot	pfam13365	Trypsin_2	Trypsin-like	.	.	GO:0004252|se	.
Manes.08G11910(	1.0710232	0.44859	up	no	Autophagy-re	pfam00519	PPV_E1_C	Papillomavi	GO:0048102|a	GO:0005829|	GO:0080025|ph	.
Manes.08G11920(	7.5878954	1.25E-33	up	yes	.	pfam00168	C2	C2 domain.	.	.	.	.
Manes.08G11930(	0.8253058	0.07892	down	no	Acyl-CoA-bi	pfam00887	ACBP	Acyl CoA b	GO:0006869|l	GO:0005737|	GO:0000062|fa	.
Manes.08G11940(	0.7459711	0.00296	down	no	Lysine-specif	pfam10497	zf-4CXXC_R1	Zinc-finger	GO:0033169|l	GO:0005634|	GO:0051213|di	.
Manes.08G11950(	0.4218651	8.71E-12	down	yes	PsbP domain	pfam01789	PsbP	PsbP. This f	GO:0015979|l	GO:0009507|	GO:0005509|ca	.

Manes.08G11980(	NA	NA	--	no	Nuclear trans	pfam00808	CBFD_NFYB_HN	Histone-like	GO:0006355|r	GO:0005634|	GO:0043565|se	.
Manes.08G12010(	0.4578097	4.00E-08	down	yes	Sucrose nonf	pfam00571	CBS	CBS domain	GO:0032147|a	GO:0005737|	GO:0030295|pr	.
Manes.08G12020(	0.4934545	4.16E-12	down	yes	Signal recogn	pfam12796	Ank_2	Ankyrin rep	GO:0045038|p	GO:0009507|	GO:0042802|id	K12271
Manes.08G12030(	1.551478	6.18E-05	up	no	Probable lino	pfam00305	Lipoxygenase	Lipoxygenas	GO:0031408|o	GO:0005737|	GO:0046872|m	K15718
Manes.08G12040(	2.2751401	1.33E-20	up	yes	F-box proteir	pfam01476	LysM	LysM doma	GO:0016567|p	.	.	.
Manes.08G12050(	0.8042751	0.02449	down	no	Mannosylglyc	pfam00703	Glyco_hydro_2	Glycosyl hyc	GO:0005975|c	.	GO:0033947|m	.
Manes.08G12120(	0.6862389	0.00015	down	no	Mitochondria	pfam08356	EF_assoc_2	EF hand ass	GO:0007005|r	GO:0031307|	GO:0005509|ca	K07870
Manes.08G12140(	1.0594957	0.58764	up	no	Protein YIPF	pfam04893	Yip1	Yip1 domain	.	GO:0016021|	.	.
Manes.08G12160(	4.6199849	3.16E-26	up	yes	.	pfam08601	PAP1	Transcriptio	.	.	.	.
Manes.08G12230(	0.5033696	1.87E-05	down	no	Mevalonate k	pfam08544	GHMP_kinases_C	GHMP kina	GO:0019287|i	GO:0005829|	GO:0005524|A	K00869
Manes.08G12260(	0.7588915	0.00547	down	no	.	pfam12776	Myb_DNA-bind_3	Myb/SANT-	.	.	.	.
Manes.08G12290(	0.3037626	1.36E-12	down	yes	Serine/threon	pfam00069	Pkinase	Protein kina	GO:0009742|b	GO:0005886|	GO:0005524|A	.
Manes.08G12360(	0.495979	1.48E-06	down	yes	.	pfam06364	DUF1068	Protein of u	.	.	.	.
Manes.08G12410(	0.6736508	6.91E-05	down	no	Sugar transpo	pfam00083	Sugar_tr	Sugar (and c	GO:0008643|c	GO:0016021|	GO:0022891|su	.
Manes.08G12430(	0.0975359	9.15E-15	down	yes	Putative suga	pfam00083	Sugar_tr	Sugar (and c	GO:0008643|c	GO:0016021|	GO:0022891|su	.
Manes.08G12450(	1.0061995	0.96389	up	no	Autophagy-re	pfam12490	BCAS3	Breast carci	GO:0006914|a	GO:0005737|	.	.
Manes.08G12460(	2.2678876	1.78E-15	up	yes	.	pfam01576	Myosin_tail_1	Myosin tail.	.	.	.	.
Manes.08G12490(	1.6142243	3.03E-07	up	no	.	pfam02163	Peptidase_M50	Peptidase fa	.	.	.	.
Manes.08G12550(	31.791431	7.09E-70	up	yes	.	pfam05701	WEMBL	Weak chloro	.	.	.	.
Manes.08G12570(	0.629202	2.55E-05	down	no	Vesicle-assoc	pfam00635	Motile_Sperm	MSP (Major	GO:0046907|i	GO:0005789|	.	.
Manes.08G12580(	2.3610526	5.43E-13	up	yes	Sodium/hydro	pfam00999	Na_H_Exchanger	Sodium/hyd	GO:0055075|p	GO:0016021|	GO:0015386|pc	.
Manes.08G12610(	1.890723	2.32E-05	up	no	Probable nuc	pfam01798	Nop	Putative sno	GO:0042254|r	GO:0016020|	.	K14565
Manes.08G12620(	0.2139885	1.73E-06	down	yes	Double-stranc	pfam00035	dsrm	Double-stra	.	.	GO:0003725|dc	.
Manes.08G12670(	1.5479254	9.99E-07	up	no	Serine/threon	pfam14381	EDR1	Ethylene-res	GO:0009738|a	GO:0005829|	GO:0005524|A	.
Manes.08G12750(	0.5815828	8.14E-08	down	no	Rubredoxin	pfam00301	Rubredoxin	Rubredoxin.	GO:0055114|c	.	GO:0005506|irc	.
Manes.08G12760(	1.5430611	1.28E-06	up	no	Protein transp	pfam04811	Sec23_trunk	Sec23/Sec24	GO:0006888|I	GO:0030127|	GO:0008270|zi	K14007
Manes.08G12770(	0.9073784	0.45194	down	no	Guanine dear	pfam00383	dCMP_cyt_deam_	Cytidine anc	GO:0006147|g	.	GO:0008892|gu	K01487
Manes.08G12800(	0.4566724	1.61E-11	down	yes	Serine/threon	pfam00069	Pkinase	Protein kina	GO:0007623|c	GO:0016020|	GO:0005524|A	.
Manes.08G12860(	3.6014785	6.92E-44	up	yes	.	pfam03096	Ndr	Ndr family.	.	.	.	.
Manes.08G12970(	0.7185702	0.08011	down	no	Cellulose syn	pfam03552	Cellulose_synt	Cellulose sy	GO:0071555|c	GO:0005783|	GO:0016760|ce	.
Manes.08G12980(	1.765475	0.00034	up	no	Cellulose syn	pfam03552	Cellulose_synt	Cellulose sy	GO:0071555|c	GO:0000139|	GO:0016760|ce	.
Manes.08G13230(	0.490978	3.31E-08	down	yes	Multisubstrat	pfam01142	TruD	tRNA pseuc	GO:0000455|e	GO:0005737|	GO:0009982|ps	K06176
Manes.08G13260(	0.8495023	0.1426	down	no	Serine/threon	pfam00069	Pkinase	Protein kina	GO:0006468|p	.	GO:0005524|A	K08857
Manes.08G13290(	1.2514408	0.01415	up	no	Probable ribo	pfam06026	Rib_5-P_isom_A	Ribose 5-ph	GO:0042742|c	GO:0009507|	GO:0004751|rit	K01807
Manes.08G13340(	3.0450333	9.25E-06	up	yes	Perakine redu	pfam00248	Aldo_ket_red	Aldo/keto re	GO:0009820|a	.	GO:0016491|ox	.
Manes.08G13360(	0.2480165	9.14E-13	down	yes	Perakine redu	pfam00248	Aldo_ket_red	Aldo/keto re	GO:0009820|a	.	GO:0016491|ox	.
Manes.08G13390(	0.4179909	0.00168	down	yes	.	pfam03162	Y_phosphatase2	Tyrosine ph	.	.	.	.
Manes.08G13400(	3.3614523	1.59E-08	up	yes	Calcium-tran	pfam00689	Cation_ATPase_C	Cation trans	.	GO:0016021|	GO:0005524|A	K01537
Manes.08G13430(	0.3401052	0.00243	down	yes	.	pfam05192	MutS_III	MutS doma	.	.	.	.
Manes.08G13450(	6.3233906	2.40E-21	up	yes	Light-inducib	pfam12498	bZIP_C	Basic leucin	GO:0006351|t	GO:0005634|	GO:0043565|se	.
Manes.08G13490(	6.3743312	0.01114	up	yes	.	pfam06450	NhaB	Bacterial Na	.	.	.	.
Manes.08G13500(	1.1224955	0.22425	up	no	Auxin-respor	pfam02309	AUX_IAA	AUX/IAA f	GO:0009734|a	GO:0005634|	GO:0003700|tra	K14484
Manes.08G13580(	0.5804297	2.71E-06	down	no	.	pfam00583	Acetyltransf_1	Acetyltransf	.	.	.	.
Manes.08G13590(	6.673454	7.61E-16	up	yes	Transcription	pfam00400	WD40	WD domain	GO:0030154|c	GO:0005634|	.	.
Manes.08G13640(	0.6736481	0.00018	down	no	.	pfam13450	NAD_binding_8	NAD(P)-bir	.	.	.	.
Manes.08G13660(	1.2149518	0.63557	up	no	Uncharacteriz	pfam01918	Alba	Alba. Alba i	.	GO:0005829|	GO:0003676|nu	.
Manes.08G13680(	1.310106	0.0099	up	no	.	pfam04576	Zein-binding	Zein-binding	.	.	.	.
Manes.08G13710(	Inf	0.9009	up	no	.	pfam05678	VQ	VQ motif. T	.	.	.	.
Manes.08G13720(	4.2522875	6.78E-15	up	yes	AT-hook mo	pfam03479	DUF296	Domain of u	GO:0006325|c	GO:0005634|	GO:0003680|A	.
Manes.08G13790(	21.911307	1.94E-13	up	yes	.	pfam03763	Remorin_C	Remorin, C-	.	.	.	.
Manes.08G13830(	10.134576	5.09E-26	up	yes	Phosphoenol	pfam00069	Pkinase	Protein kina	GO:0009738|a	GO:0005737|	GO:0005524|A	.
Manes.08G13870(	7.3765045	0.00011	up	yes	Myb-related	pfam00249	Myb_DNA-binding	Myb-like DI	GO:0006355|r	GO:0005634|	GO:0003677|DI	K09422
Manes.08G13920(	2.674596	7.00E-06	up	yes	Probable low	pfam01212	Beta_elim_lyase	Beta-elimina	GO:0006545|g	GO:0005829|	GO:0008732|L-	K01620
Manes.08G13940(	1.2069871	0.04032	up	no	Putative lysin	pfam02373	JmjC	JmjC domai	GO:0016568|c	GO:0005634|	GO:0051213|dic	K11446
Manes.08G13950(	0.796763	0.07718	down	no	Protein preny	pfam01239	PPTA	Protein pren	GO:0018342|p	GO:0005737|	GO:0008318|pr	K14137
Manes.08G14040(	Inf	0.04703	up	no	Histone-lysin	pfam00856	SET	SET domain	.	GO:0005694|	GO:0018024|his	.
Manes.08G14080(	1.5930722	2.81E-07	up	no	.	pfam08387	FBD	FBD. This r	.	.	.	.
Manes.08G14100(	Inf	0.17828	up	no	.	pfam00644	PARP	Poly(ADP-r	.	.	.	.
Manes.08G14110(	4.5880148	1.28E-60	up	yes	.	pfam06101	DUF946	Plant protein	.	.	.	.
Manes.08G14140(	2.9248141	3.72E-32	up	yes	Inositol phos	pfam01501	Glyco_transf_8	Glycosyl tra	GO:0046513|c	GO:0005768|	GO:0015020|glu	.
Manes.08G14190(	0.3267348	1.57E-10	down	yes	DNA-directe	pfam03874	RNA_pol_Rpb4	RNA polym	GO:0031990|r	GO:0000932|	GO:0003899|DI	K03012
Manes.08G14210(	1.1565838	0.20451	up	no	Cytoplasmic	pfam01171	ATP_bind_3	PP-loop fan	GO:0032447|p	GO:0005737|	GO:0016779|nu	.
Manes.08G14230(	1.5046228	9.62E-06	up	no	.	pfam03080	DUF239	Domain of u	.	.	.	.
Manes.08G14240(	1.1197146	0.27509	up	no	GDSL estera	pfam00657	Lipase_GDSL	GDSL-like I	GO:0042538|h	GO:0005576|	GO:0016788|hy	.
Manes.08G14250(	0.8232445	0.05444	down	no	GDSL estera	pfam00657	Lipase_GDSL	GDSL-like I	GO:0016042|l	GO:0005576|	GO:0016788|hy	.
Manes.08G14270(	0.398574	1.43E-08	down	yes	Probable leuc	pfam00069	Pkinase	Protein kina	.	GO:0016021|	GO:0005524|A	.
Manes.08G14280(	1.0027056	0.95667	up	no	Probable UD	pfam13844	Glyco_transf_41	Glycosyl tra	GO:0007275|r	.	GO:0016757|tra	K09667
Manes.08G14290(	NA	NA	--	no	Germin-like p	pfam00190	Cupin_1	Cupin. This	.	GO:0048046|	GO:0030145|m	.
Manes.08G14300(	NA	NA	--	no	Germin-like p	pfam00190	Cupin_1	Cupin. This	.	GO:0048046|	GO:0030145|m	.
Manes.08G14390(	0.9875484	0.91303	down	no	Casein kinase	pfam00069	Pkinase	Protein kina	GO:0018105|p	GO:0005813|	GO:0005524|A	K08957
Manes.08G14420(	10.609733	0.02893	up	yes	.	pfam01578	Cytochrom_C_asn	Cytochrome	.	.	.	.
Manes.08G14430(	1.5950588	0.0017	up	no	.	pfam13398	Peptidase_M50B	Peptidase M	.	.	.	.
Manes.08G14490(	4.7643972	8.28E-12	up	yes	.	pfam14897	EpsG	EpsG family	.	.	.	.
Manes.08G14500(	1.2807132	0.35144	up	no	.	pfam09770	PAT1	Topoisomer	.	.	.	.
Manes.08G14540(	10.229103	3.67E-23	up	yes	Ethylene-resp	pfam00847	AP2	AP2 domain	GO:0035865|c	GO:0005634|	GO:0043565|se	K09286
Manes.08G14570(	1.7630953	9.12E-09	up	no	Uncharacteriz	pfam10250	O-FucT	GDP-fucose	.	GO:0005768|	GO:0016757|tra	.
Manes.08G14580(	0.634964	0.00245	down	no	Syntaxin-132	pfam05739	SNARE	SNARE dor	GO:0006886|i	GO:0016021|	.	.
Manes.08G14650(	NA	NA	--	no	Germin-like p	pfam00190	Cupin_1	Cupin. This	.	GO:0048046|	GO:0030145|m	.
Manes.08G14660(	1.1081526	0.62135	up	no	Probable UD	pfam13844	Glyco_transf_41	Glycosyl tra	GO:0007275|r	.	GO:0016757|tra	K09667
Manes.08G14750(	0.8710113	0.19916	down	no	Apyrase 2	pfam01150	GDA1_CD39	GDA1/CD3	.	GO:0000139|	GO:0005524|A	.
Manes.08G14760(	4.3356615	0.0002	up	yes	.	pfam13000	Acatn	Acetyl-coen	.	.	.	.
Manes.08G14770(	2.8696514	0.00017	up	yes	.	pfam09348	DUF1990	Domain of u	.	.	.	.
Manes.08G14780(	19.386118	#######	up	yes	NAC transcri	pfam02365	NAM	No apical m	GO:0006355|r	GO:0005634|	GO:0003677|DI	.
Manes.08G14790(	1.1861844	0.07598	up	no	.	pfam14200	RicinB_lectin_2	Ricin-type b	.	.	.	.
Manes.08G14800(	13.25008	4.46E-24	up	yes	NAC domain	pfam02365	NAM	No apical m	GO:0006351|t	GO:0005634|	GO:0003677|DI	.
Manes.08G14810(	0.7762802	0.02687	down	no	U11/U12 sma	pfam00076	RRM_1	RNA recogn	GO:0010229|i	GO:0005634|	GO:0000166|nu	K13157
Manes.08G14820(	1.6980815	5.03E-07	up	no	Thioredoxin-	pfam00085	Thioredoxin	Thioredoxin	GO:0045454|c	GO:0009507|	GO:0016671|ox	.
Manes.08G14840(	6.9056966	2.68E-21	up	yes	.	pfam02891	zf-MIZ	MIZ/SP-RII	.	.	.	.

Manes.08G14850(	2.9720354	4.19E-12	up	yes	Expansin-A1:	pfam01357	Pollen_allerg_1	Pollen allerg	GO:0009664|	GO:0005618|	.	.
Manes.08G14890(	0.9062691	0.55998	down	no	.	pfam00209	SNF	Sodium:neu	.	.	.	.
Manes.08G14900(	2.0504767	7.15E-16	up	yes	Early nodulin	pfam03386	ENOD93	Early noduli	GO:0009877|	GO:0016021|	.	.
Manes.08G14910(	2.0280495	4.02E-12	up	yes	TATA-box-b	pfam00352	TBP	Transcriptio	GO:0006352|	GO:0005634|	GO:0003677|D	K03120
Manes.08G14920(	0.8273873	0.18114	down	no	.	pfam01844	HNH	HNH endon	.	.	.	.
Manes.08G14940(	0.5504739	2.81E-09	down	no	CCR4-NOT t	pfam04065	Not3	Not1 N-terr	GO:0010467|	GO:0030014|	.	K12580
Manes.08G14960(	0.5420175	0.04495	down	no	Myb family t	pfam14379	Myb_CC_LHEQL	MYB-CC ty	GO:0010088|	GO:0005634|	GO:0003677|D	.
Manes.08G14990(	0.4371887	5.24E-08	down	yes	.	pfam14432	DYW_deaminase	DYW family	.	.	.	.
Manes.08G15030(	#NAME?	0.64948	down	no	.	pfam09586	YfhO	Bacterial me	.	.	.	.
Manes.08G15040(	0.7113821	0.09266	down	no	Protein TRA	pfam01554	MatE	MatE. The M	GO:0009813|	GO:0016021|	GO:0015238|dr	K03327
Manes.08G15050(	1.1355654	0.35302	up	no	Protein GrpE	pfam01025	GrpE	GrpE.	GO:0006457|	GO:0005737|	GO:0000774|ad	K03687
Manes.08G15060(	1.05242	0.5722	up	no	Probable AD	pfam01412	ArfGap	Putative GT	.	.	GO:0005096|G	.
Manes.08G15110(	0.6644115	0.00691	down	no	Myb-related	pfam00249	Myb_DNA-binding	Myb-like DI	GO:0006355|	GO:0005634|	GO:0003677|D	.
Manes.08G15120(	1.4896944	8.97E-06	up	no	Probable S-s	pfam00291	PALP	Pyridoxal-pl	GO:0006535|	GO:0009507|	GO:0004124|cy	K01738
Manes.08G15160(	0.6018256	8.26E-05	down	no	.	pfam12697	Abhydrolase_6	Alpha/beta l	.	.	.	.
Manes.08G15170(	1.0112433	0.86863	up	no	Zinc finger p	pfam00641	zf-RanBP	Zn-finger in	GO:1900871|	GO:0009507|	GO:0008270|zi	.
Manes.08G15200(	NA	NA	--	no	Peroxidase 5	pfam00141	peroxidase	Peroxidase.	GO:0042744|	GO:0005618|	GO:0020037|he	K00430
Manes.08G15210(	3.1243918	1.10E-08	up	yes	Cyclic dof fa	pfam02701	zf-Dof	Dof domain	GO:0009908|	GO:0005634|	GO:0003677|D	.
Manes.08G15220(	1.0944522	0.58894	up	no	.	pfam05832	DUF846	Eukaryotic	.	.	.	.
Manes.08G15260(	0.4821257	4.06E-09	down	yes	Xylosyltransf	pfam02485	Branch	Core-2/I-Br	GO:0034605|	GO:0005789|	GO:0008375|ac	K00771
Manes.08G15280(	0.775291	0.04612	down	no	.	pfam00684	DnaJ_CXXCXGX	DnaJ centra	.	.	.	.
Manes.08G15290(	9.6339252	#######	up	yes	Cation/calciu	pfam01699	Na_Ca_ex	Sodium/calc	GO:0006813|	GO:0016021|	GO:0015297|an	.
Manes.08G15330(	0.4762914	9.42E-05	down	yes	Microfibrillar	pfam06991	Prp19_bind	Splicing fact	GO:0030198|	GO:0005576|	GO:0044822|pc	K13110
Manes.08G15350(	0.5914221	0.01128	down	no	.	pfam11951	Fungal_trans_2	Fungal spec	.	.	.	.
Manes.08G15360(	0.4834482	0.00053	down	yes	.	pfam04789	DUF621	Protein of u	.	.	.	.
Manes.08G15370(	1.3222133	0.00197	up	no	Cationic amir	pfam13520	AA_permease_2	Amino acid	GO:0003333|	GO:0005887|	GO:0015297|an	.
Manes.08G15390(	5.8735168	1.28E-86	up	yes	.	pfam10225	DUF2215	Uncharacter	.	.	.	.
Manes.08G15400(	0.8290264	0.20651	down	no	.	pfam07802	GCK	GCK domai	.	.	.	.
Manes.08G15420(	1.8022259	2.88E-11	up	no	Ubiquitin-cor	pfam00179	UQ_con	Ubiquitin-cc	GO:0006281|	GO:0005737|	GO:0005524|A	K10573
Manes.08G15430(	2.0927251	4.82E-17	up	yes	Exosome cor	pfam01612	DNA_pol_A_exo1	3'-5' exonuc	GO:0071034|	GO:0005737|	GO:0008408|3'-	K12591
Manes.08G15550(	1.5213469	4.09E-06	up	no	ELMO doma	pfam04727	ELMO_CED12	ELMO/CEI	GO:0048870|	GO:0005737|	GO:0045159|m	.
Manes.08G15570(	1.0570958	0.52548	up	no	.	pfam09528	Ehrlichia_rpt	Ehrlichia tar	.	.	.	.
Manes.08G15580(	0.8660795	0.14912	down	no	TATA-bindir	pfam12054	DUF3535	Domain of u	GO:1902185|	GO:0005634|	GO:0005524|A	.
Manes.08G15630(	0.5800183	6.37E-08	down	no	RNA polyme	pfam04542	Sigma70_r2	Sigma-70 re	GO:0071482|	GO:0009507|	GO:0003677|D	.
Manes.08G15650(	0.9728291	0.80941	down	no	.	pfam07928	Vps54	Vps54-like	.	.	.	.
Manes.08G15660(	Inf	1.54E-14	up	yes	Beta-amyrin	pfam00067	p450	Cytochrome	.	GO:0016021|	GO:0020037|he	.
Manes.08G15670(	0.4834231	5.20E-10	down	yes	.	pfam06219	DUF1005	Protein of u	.	.	.	.
Manes.08G15680(	0.5320895	5.49E-06	down	no	Tubulin-foldi	pfam02970	TBCA	Tubulin binc	GO:0007021|	GO:0005829|	.	K17292
Manes.08G15690(	0.8257536	0.14623	down	no	.	pfam00754	F5_F8_type_C	F5/8 type C	.	.	.	.
Manes.08G15700(	0.873127	0.15926	down	no	.	pfam07420	DUF1509	Protein of u	.	.	.	.
Manes.08G15710(	0.4872855	6.03E-05	down	yes	.	pfam06813	Nodulin-like	Nodulin-like	.	.	.	.
Manes.08G15770(	0.3400151	3.04E-08	down	yes	.	pfam05622	HOOK	HOOK prot	.	.	.	.
Manes.08G15790(	1.3411121	0.04056	up	no	Non-specific	pfam04185	Phosphoesterase	Phosphoeste	GO:0016036|	GO:0016020|	GO:0052642|ly	K01114
Manes.08G15810(	5.4453991	0.00164	up	yes	BTB/POZ do	pfam03000	NPH3	NPH3 famil	GO:0016567|	.	.	.
Manes.08G15840(	NA	NA	--	no	.	pfam10321	7TM_GPCR_Srt	Serpentine t	.	.	.	.
Manes.08G15890(	0.8576378	0.14753	down	no	Serine/threon	pfam00069	Pkinase	Protein kina	GO:0007165|	GO:0051286|	GO:0005524|A	K18670
Manes.08G15900(	0.7783788	0.0099	down	no	RNA polyme	pfam04542	Sigma70_r2	Sigma-70 re	GO:0071482|	GO:0009507|	GO:0003677|D	K03086
Manes.08G15910(	26.646345	1.79E-18	up	yes	.	pfam04910	Tcf25	Transcriptio	.	.	.	.
Manes.08G15930(	0.2237827	0.00013	down	yes	.	pfam04740	LXG	LXG domai	.	.	.	.
Manes.08G15940(	0.3678657	6.05E-11	down	yes	Pentatricope	pfam13041	PPR_2	PPR repeat	.	GO:0005739|	.	.
Manes.08G15960(	0.4759434	4.64E-05	down	yes	Probable fola	pfam03092	BT1	BT1 family.	GO:0006810|	GO:0016021|	.	.
Manes.08G15980(	0.5710471	0.00028	down	no	Cytochrome	pfam00067	p450	Cytochrome	GO:0044550|	GO:0016021|	GO:0020037|he	.
Manes.08G16030(	0.4524517	5.68E-10	down	yes	.	pfam01576	Myosin_tail_1	Myosin tail.	.	.	.	.
Manes.08G16050(	1.4589999	9.98E-05	up	no	.	pfam03124	EXS	EXS family.	.	.	.	.
Manes.08G16080(	1.9192281	2.01E-05	up	no	Growth-regu	pfam08879	WRC	WRC. The	GO:0032502|	GO:0005634|	GO:0005524|A	.
Manes.08G16090(	0.7399047	0.01129	down	no	GPCR-type (	pfam12430	ABA_GPCR	Abscisic aci	.	GO:0005789|	GO:0005525|G	.
Manes.08G16100(	0.2817417	8.70E-17	down	yes	F-box/kelch-	pfam01344	Kelch_1	Kelch motif.	.	GO:0005634|	.	.
Manes.08G16110(	0.7692339	0.00684	down	no	Bromodomai	pfam00628	PHD	PHD-finger.	GO:0000183|	GO:0005677|	GO:0003677|D	K15224
Manes.08G16180(	2.4777105	1.95E-05	up	yes	Werner Synd	pfam01612	DNA_pol_A_exo1	3'-5' exonuc	GO:0090305|	GO:0005737|	GO:0008408|3'-	.
Manes.08G16260(	1.112697	0.28959	up	no	D-xylose-pro	pfam00083	Sugar_tr	Sugar (and	GO:0008643|	GO:0016021|	GO:0022891|su	.
Manes.08G16270(	0.8347991	0.12782	down	no	Probable NA	pfam05071	NDUFA12	NADH ubiq	GO:0009735|	GO:0005747|	GO:0050897|co	.
Manes.08G16280(	1.348543	0.00097	up	no	ERAD-assoc	pfam13639	zf-RING_2	Ring finger	GO:0030433|	GO:0005789|	GO:0016874|lig	K10601
Manes.08G16290(	2.2194852	1.58E-11	up	yes	ER lumen pr	pfam00810	ER_lumen_recept	ER lumen p	GO:0006621|	GO:0005789|	GO:0046923|EI	.
Manes.08G16310(	1.786743	1.56E-10	up	no	Exportin-1	pfam08767	CRM1_C	CRM1 C ter	GO:0051028|	GO:0005642|	GO:0005487|nu	K14290
Manes.08G16320(	0.5702621	0.00025	down	no	Anthocyanidi	pfam00201	UDPGT	UDP-glucor	GO:0009718|	.	GO:0047213|an	.
Manes.08G16340(	0.655275	0.09571	down	no	ADP-ribosyla	pfam00025	Arf	ADP-ribosy	GO:0015031|	GO:0005794|	GO:0005525|G	.
Manes.08G16360(	2.0245477	1.63E-07	up	yes	Cytochrome	pfam00067	p450	Cytochrome	GO:0044550|	GO:0016021|	GO:0020037|he	.
Manes.08G16380(	0.7338856	0.02213	down	no	Serine/threon	pfam09184	PPP4R2	PPP4R2. PF	GO:0000278|	GO:0000775|	.	.
Manes.08G16410(	Inf	0.9009	up	no	.	pfam11260	Spidroin_MaSp	Major ampu	.	.	.	.
Manes.08G16420(	0.4773629	0.00018	down	yes	.	pfam10034	Dpy19	Q-cell neurc	.	.	.	.
Manes.08G16430(	0.9849403	0.89522	down	no	.	pfam00855	PWWP	PWWP dom	.	.	.	.
Manes.08G16440(	0.8538544	0.20095	down	no	Altered inher	pfam03803	Scramblase	Scramblase.	.	GO:0005739|	.	.
Manes.08G16470(	2.7208812	1.70E-08	up	yes	.	pfam06749	DUF1218	Protein of u	.	.	.	.
Manes.08G16490(	1.3883472	0.00344	up	no	.	pfam12588	PSDC	Phophatidyl	.	.	.	.
Manes.08G16500(	2.3458577	1.55E-05	up	yes	.	pfam02028	BCCT	BCCT famil	.	.	.	.
Manes.08G16510(	0.8543771	0.17589	down	no	Pentatricope	pfam13041	PPR_2	PPR repeat	.	GO:0009507|	.	.
Manes.08G16570(	0.8745146	0.20247	down	no	Probable gala	pfam01501	Glyco_transf_8	Glycosyl tra	GO:0071555|	GO:0005768|	GO:0047262|pc	K13648
Manes.08G16580(	0.2920264	7.45E-07	down	yes	Protein SCA	pfam03514	GRAS	GRAS doma	GO:0007275|	GO:0005634|	.	.
Manes.08G16610(	0.1980669	3.55E-10	down	yes	Ethanolamine	pfam01467	CTP_transf_2	Cytidylyltra	GO:0006646|	GO:0031307|	GO:0004306|et	K00967
Manes.08G16620(	2.355775	0.00464	up	yes	.	pfam04525	Tub_2	Tubby C 2.	.	.	.	.
Manes.08G16630(	2.3226872	2.59E-11	up	yes	.	pfam13327	T3SS_LEE_assoc	Type III sec	.	.	.	.
Manes.08G16640(	1.3171939	0.00287	up	no	Serine/threon	pfam00069	Pkinase	Protein kina	.	GO:0005886|	GO:0005524|A	.
Manes.08G16650(	1.1739518	0.15616	up	no	50S ribosoma	pfam01196	Ribosomal_L17	Ribosomal p	GO:0006412|	GO:0009507|	GO:0019843|rR	K02879
Manes.08G16660(	3.5162508	3.48E-37	up	yes	.	pfam04525	Tub_2	Tubby C 2.	.	.	.	.
Manes.08G16730(	0.7947139	0.18617	down	no	.	pfam10962	DUF2764	Protein of u	.	.	.	.
Manes.08G16740(	9.2557931	1.07E-14	up	yes	Calcium-bind	pfam13833	EF-hand_8	EF-hand do	GO:0010091|	.	GO:0005509|ca	K13448
Manes.08G16790(	0.4074405	1.63E-12	down	yes	.	pfam06697	DUF1191	Protein of u	.	.	.	.
Manes.08G16800(	0.6421103	0.03264	down	no	NADH dehy	pfam03073	TspO_MBR	TspO/MBR	GO:0055114|	GO:0016021|	.	K03945

Manes.08G16810(	1.6918739	4.71E-08	up	no	KH domain-c	pfam00013	KH_1	KH domain.	.	GO:0005829|	GO:0003723|RI	K14945
Manes.08G16820(	1.9364028	1.17E-08	up	no	.	pfam11891	DUF3411	Domain of u	.	.	.	.
Manes.08G16830(	0.8093927	0.0474	down	no	Putative zinc	pfam02535	Zip	ZIP Zinc tra	GO:0006829|	GO:0016021|	GO:0046873|m	.
Manes.08G16840(	0.5521324	0.01018	down	no	Tubulin-foldi	pfam09106	SelB-wing_2	Elongation f	GO:0009793|	GO:0005829|	GO:0048487|be	.
Manes.08G16880(	0.7382818	0.04054	down	no	Probable LRI	pfam07714	Pkinase_Tyr	Protein tyro	.	GO:0016021|	GO:0005524|A	.
Manes.08G16890(	0.8273833	0.05404	down	no	Acetyltransfe	pfam13508	Acetyltransf_7	Acetyltransf	GO:0030187|	GO:0009507|	GO:0004059|ar	.
Manes.08G16910(	0.711344	0.03079	down	no	BTB/POZ do	pfam03000	NPH3	NPH3 famil	GO:0016567|	.	.	.
Manes.08G16940(	0.7150405	0.00251	down	no	.	pfam07999	RHSP	Retrotransp	.	.	.	.
Manes.08G17010(	4.1936948	1.02E-57	up	yes	Receptor-like	pfam12819	Malectin_like	Carbohydrat	GO:0009738|	GO:0043680|	GO:0005524|A	.
Manes.08G17030(	0.4414899	0.00104	down	yes	Serine/threon	pfam00069	Pkinase	Protein kina	GO:0045727|	GO:0005634|	GO:0005524|A	K04688
Manes.08G17040(	1.873768	5.16E-07	up	no	Transcription	pfam07500	TFIIS_M	Transcriptio	GO:0006354|	GO:0005813|	GO:0003677|D	K03145
Manes.08G17050(	0.5351907	2.87E-09	down	no	Phospholipid	pfam00122	E1-E2_ATPase	E1-E2 ATP	GO:0006886|	GO:0005783|	GO:0005524|A	K14802
Manes.08G17070(	0.8295685	0.06395	down	no	Transmembra	pfam06814	Lung_7-TM_R	Lung seven	.	GO:0016021|	.	.
Manes.08G17080(	1.2635624	0.00997	up	no	Vacuolar-sor	pfam02225	PA	PA domain.	GO:0006896|	GO:0030665|	GO:0009940|an	.
Manes.08G17090(	0.8657968	0.28316	down	no	.	pfam07464	ApoLp-III	Apolipopho	.	.	.	.
Manes.08G17100(	2.5533846	2.52E-21	up	yes	Probably inac	pfam07714	Pkinase_Tyr	Protein tyro	.	GO:0016021|	GO:0005524|A	.
Manes.08G17110(	3.1086532	9.87E-24	up	yes	.	pfam06493	DUF1096	Protein of u	.	.	.	.
Manes.08G17160(	33.256897	5.89E-45	up	yes	.	pfam02906	Fe_hyd_lg_C	Iron only hy	.	.	.	.
Manes.08G17170(	1.0493659	0.58166	up	no	Non-specific	pfam00234	Tryp_alpha_amyl	Protease inh	GO:0006869|	.	GO:0008289|lip	.
Manes.08G17180(	1.474749	1.41E-05	up	no	Non-specific	pfam00234	Tryp_alpha_amyl	Protease inh	GO:0006869|	.	GO:0008289|lip	.
Manes.08G17190(	1.2903824	0.01137	up	no	Non-specific	pfam00234	Tryp_alpha_amyl	Protease inh	GO:0006869|	.	GO:0008289|lip	.
Manes.08G17200(	9.9256146	4.03E-11	up	yes	Expansin-A8	pfam01357	Pollen_allerg_1	Pollen allerg	GO:0009664|	GO:0005618|	.	.
Manes.08G17210(	0.4154622	2.91E-08	down	yes	MACPF dom	pfam01823	MACPF	MAC/Perfor	GO:0008219|	.	.	.
Manes.08G17220(	0.5479116	4.27E-07	down	no	Serine/threon	pfam07714	Pkinase_Tyr	Protein tyro	.	.	GO:0005524|A	.
Manes.08G17250(	0.4598291	1.84E-05	down	yes	Aspartate am	pfam00155	Aminotran_1_2	Aminotransf	GO:0006103|	GO:0005759|	GO:0005507|co	K14455
Manes.08G17300(	Inf	0.9009	up	no	.	pfam01567	Hanta_G1	Hantavirus g	.	.	.	.
Manes.08G17340(	0.8672089	0.20512	down	no	CBL-interact	pfam00069	Pkinase	Protein kina	GO:0007165|	GO:0005634|	GO:0005524|A	.
Manes.08G17370(	0.3754575	0.00031	down	yes	Transcription	pfam04844	Ovate	Transcriptio	GO:0045892|	GO:0005634|	GO:0003677|D	.
Manes.08G17380(	3.680143	0.00034	up	yes	.	pfam08429	PLU-1	PLU-1-like	.	.	.	.
Manes.08G17390(	2.3572624	1.19E-14	up	yes	Probable seri	pfam00069	Pkinase	Protein kina	GO:0048573|	.	GO:0005524|A	K08867
Manes.08G17420(	0.568406	0.00013	down	no	.	pfam03025	Papilloma_E5	Papillomavi	.	.	.	.
Manes.08G17440(	1.0609034	0.50478	up	no	Regulatory-a	pfam14538	Raptor_N	Raptor N-te	GO:0016049|	GO:0080008|	.	K07204
Manes.09G00010(	1.4874548	0.00119	up	no	Membrane-as	pfam00173	Cyt-b5	Cytochrome	.	GO:0012505|	GO:0020037|he	.
Manes.09G00080(	1.4244909	0.00057	up	no	Calmodulin	pfam13499	EF-hand_7	EF-hand do	.	.	GO:0005509|ca	.
Manes.09G00090(	0.7291341	0.00882	down	no	Probable gala	pfam01501	Glyco_transf_8	Glycosyl tra	GO:0071555|	GO:0005768|	GO:0047262|pc	.
Manes.09G00110(	1.4968466	0.0016	up	no	.	pfam05678	VQ	VQ motif. T	.	.	.	.
Manes.09G00130(	0.5750374	8.63E-05	down	no	Chaperonin C	pfam13347	MFS_2	MFS/sugar	GO:0042026|	GO:0005739|	GO:0005524|A	K04077
Manes.09G00180(	2.1890229	1.17E-10	up	yes	Probable pep	pfam07690	MFS_1	Major Facili	GO:0009624|	GO:0016021|	.	.
Manes.09G00210(	1.4365135	7.34E-05	up	no	Endoplasmic	pfam07970	COPIIcoated_ERV	Endoplasmi	GO:0016192|	GO:0005789|	.	.
Manes.09G00270(	0.7333633	0.00366	down	no	.	pfam15619	Lebercilin	Ciliary prote	.	.	.	.
Manes.09G00290(	0.8116976	0.04214	down	no	Putative SWI	pfam00176	SNF2_N	SNF2 family	GO:0016568|	GO:0005634|	GO:0005524|A	K15711
Manes.09G00310(	1.0426538	0.74736	up	no	Hydroxymeth	pfam00682	HMGL-like	HMGL-like.	.	GO:0005759|	GO:0004419|hy	K01640
Manes.09G00340(	1.5378873	2.66E-06	up	no	Ribosome bio	pfam04950	DUF663	Protein of u	GO:0042255|	GO:0005730|	GO:0005524|A	K14569
Manes.09G00350(	2.2997093	1.99E-20	up	yes	CDPK-relate	pfam00069	Pkinase	Protein kina	GO:0009738|	GO:0005737|	GO:0005524|A	.
Manes.09G00360(	1.6018696	6.61E-07	up	no	Dual specifici	pfam00782	DSPc	Dual specifi	GO:0006464|	GO:0005737|	GO:0004725|pr	K14819
Manes.09G00390(	3.1208171	4.75E-39	up	yes	.	pfam12937	F-box-like	F-box-like.	.	.	.	.
Manes.09G00420(	2.5321861	2.98E-09	up	yes	Pentatricope	pfam01535	PPR	PPR repeat.	.	GO:0009941|	.	.
Manes.09G00520(	1.9196875	8.46E-08	up	no	Transcription	pfam01849	NAC	NAC domai	GO:0001701|	GO:0005737|	GO:0044822|pc	K01527
Manes.09G00570(	2.7786287	0.00344	up	yes	.	pfam14690	zf-ISL3	zinc-finger c	.	.	.	.
Manes.09G00590(	NA	NA	--	no	.	pfam14671	DSPn	Dual specifi	.	.	.	.
Manes.09G00640(	17.17254	2.55E-06	up	yes	Zinc finger pi	pfam13912	zf-C2H2_6	C2H2-type	GO:0048235|	GO:0005634|	GO:0046872|m	.
Manes.09G00650(	0.7706892	0.01377	down	no	Haloacid deh	pfam13419	HAD_2	Haloacid de	.	GO:0005739|	GO:0016787|hy	.
Manes.09G00660(	1.4644647	2.76E-05	up	no	.	pfam13041	PPR_2	PPR repeat	.	.	.	.
Manes.09G00670(	0.9036437	0.49721	down	no	Protein STRI	pfam03088	Str_synth	Strictosidin	GO:0009820|	GO:0005794|	GO:0016829|ly	K01757
Manes.09G00680(	0.4044531	3.28E-11	down	yes	Two-compor	pfam00072	Response_reg	Response re	GO:0007623|	GO:0005634|	GO:0000156|ph	K14492
Manes.09G00720(	0.9061121	0.34657	down	no	Tubulin-foldi	pfam14560	Ubiquitin_2	Ubiquitin-lil	GO:0051301|	GO:0005737|	.	K17262
Manes.09G00730(	0.6641413	3.32E-05	down	no	Pyruvate kina	pfam00224	PK	Pyruvate kir	GO:0006096|	GO:0005737|	GO:0005524|A	K00873
Manes.09G00760(	0.7349141	0.00721	down	no	DNA-directe	pfam01191	RNA_pol_Rpb5_C	RNA polym	GO:0006351|	GO:0000419|	GO:0003677|D	K03013
Manes.09G00770(	1.2017127	0.21358	up	no	Uncharacteri	pfam00462	Glutaredoxin	Glutaredoxi	GO:0045454|	GO:0005886|	GO:0009055|el	.
Manes.09G00780(	0.6105648	3.10E-06	down	no	.	pfam10267	Tmemb_cc2	Predicted tr	.	.	.	.
Manes.09G00820(	0.4075912	9.74E-18	down	yes	Lycopene bet	pfam05834	Lycopene_cycl	Lycopene cy	GO:0016117|	GO:0009507|	GO:0016853|isc	.
Manes.09G00830(	1.6322491	2.27E-07	up	no	.	pfam13716	CRAL_TRIO_2	Divergent C	.	.	.	.
Manes.09G00840(	2.0498078	6.75E-12	up	yes	Regulatory pi	pfam12796	Ank_2	Ankyrin rep	GO:0010434|	GO:0005737|	.	K14508
Manes.09G00860(	0.8071673	0.06112	down	no	Probable met	pfam03141	Methyltransf_29	Putative S-a	.	GO:0005768|	GO:0008168|m	.
Manes.09G00910(	2.2083216	2.07E-08	up	yes	Probable calc	pfam13833	EF-hand_8	EF-hand do	GO:0050832|	GO:0005886|	GO:0005509|ca	K13448
Manes.09G00920(	0.3384495	0.08369	down	no	.	pfam09418	DUF2009	Protein of u	.	.	.	.
Manes.09G00930(	0.626423	0.00046	down	no	Triacylglycer	pfam11815	DUF3336	Domain of u	GO:0006071|	GO:0016021|	GO:0004806|tri	K14674
Manes.09G00960(	Inf	0.00021	up	yes	.	pfam03028	Dynein_heavy	Dynein heav	.	.	.	.
Manes.09G01000(	0.6440498	0.0001	down	no	N-acetylgluc	pfam05024	Gpi1	N-acetylglu	GO:0006506|	GO:0000506|	GO:0017176|ph	K03860
Manes.09G01050(	1.1201632	0.21124	up	no	DnaJ homolo	pfam00226	DnaJ	DnaJ domai	GO:0070389|	GO:0005737|	GO:0031072|he	K09527
Manes.09G01060(	0.925338	0.46916	down	no	.	pfam03647	Tmemb_14	Transmemb	.	.	.	.
Manes.09G01100(	0.6559637	0.05159	down	no	.	pfam02593	dTMP_synthase	Thymidylate	.	.	.	.
Manes.09G01130(	0.4563356	2.12E-06	down	yes	.	pfam13041	PPR_2	PPR repeat	.	.	.	.
Manes.09G01150(	0.6528072	0.00125	down	no	Pentatricope	pfam13041	PPR_2	PPR repeat	.	GO:0005739|	.	.
Manes.09G01170(	0.6792736	0.03694	down	no	.	pfam13041	PPR_2	PPR repeat	.	.	.	.
Manes.09G01250(	0.4449497	7.31E-05	down	yes	Putative pent	pfam13041	PPR_2	PPR repeat	.	GO:0005739|	.	.
Manes.09G01280(	0.4268651	6.94E-05	down	yes	.	pfam06060	Mesothelin	Pre-pro-meg	.	.	.	.
Manes.09G01290(	0.5767655	0.02106	down	no	Pentatricope	pfam13041	PPR_2	PPR repeat	GO:0000966|	GO:0005739|	GO:0003723|RI	.
Manes.09G01300(	0.1734165	0.03071	down	no	Pentatricope	pfam13041	PPR_2	PPR repeat	GO:0000966|	GO:0005739|	GO:0003723|RI	.
Manes.09G01330(	NA	NA	--	no	Auxin-induce	pfam02519	Auxin_inducible	Auxin respo	GO:0009734|	.	.	.
Manes.09G01410(	0.6480508	0.00808	down	no	Pentatricope	pfam13041	PPR_2	PPR repeat	GO:0006397|	GO:0009507|	.	.
Manes.09G01460(	0.568867	0.01851	down	no	Putative pent	pfam13041	PPR_2	PPR repeat	.	GO:0005739|	.	.
Manes.09G01560(	0.5769352	0.00182	down	no	Pentatricope	pfam13041	PPR_2	PPR repeat	.	GO:0005739|	.	.
Manes.09G01580(	1.7139719	8.24E-05	up	no	.	pfam04712	Radial_spoke	Radial spok	.	.	.	.
Manes.09G01600(	0.5116476	1.11E-07	down	no	DNA-(apurin	pfam03372	Exo_endo_phos	Endonuclea	GO:0006284|	GO:0042644|	GO:0003677|D	.
Manes.09G01610(	0.8847199	0.38821	down	no	Glycosyltrans	pfam13704	Glyco_tranf_2_4	Glycosyl tra	GO:0007049|	GO:0005737|	GO:0016757|tra	.
Manes.09G01620(	1.499213	0.00512	up	no	Folylpolyglut	pfam03273	Baculo_gp64	Baculovirus	GO:0006730|	GO:0005743|	GO:0005524|A	K01930
Manes.09G01640(	0.7626529	0.0379	down	no	Peptide chain	pfam03462	PCRF	PCRF doma	GO:0009658|	GO:0009507|	GO:0003730|m	.

Manes.09G01660(	Inf	4.16E-05	up	yes	.	pfam00646	F-box	F-box doma	.	.	.	.
Manes.09G01690(	0.6662285	0.00721	down	no	Pentatricoper	pfam13041	PPR_2	PPR repeat	GO:0006397|r	GO:0009507|	.	.
Manes.09G01720(	0.7110113	0.0692	down	no	Pentatricoper	pfam13041	PPR_2	PPR repeat	.	GO:0005739|	.	.
Manes.09G01810(	0.8383322	0.07167	down	no	DNA helicase	pfam00176	SNF2_N	SNF2 family	GO:0006338|	GO:0031011|	GO:0005524|A'	.
Manes.09G01850(	2.290172	0.00403	up	yes	.	pfam02786	CPSase_L_D2	Carbamoyl-p	.	.	.	.
Manes.09G01870(	0.5622266	0.00018	down	no	.	pfam13041	PPR_2	PPR repeat	.	.	.	.
Manes.09G01880(	1.5324076	4.04E-06	up	no	CCAAT/enha	pfam03914	CBF	CBF/Mak21	GO:0045944|	GO:0005634|	GO:0003677|D	K14832
Manes.09G01900(	NA	NA	--	no	Calcium-trans	pfam00689	Cation_ATPase_C	Cation trans	GO:0070588|	GO:0012505|	GO:0005524|A'	K01537
Manes.09G01920(	0.9672001	0.84409	down	no	Probable glyc	pfam07479	NAD_Gly3P_dh_(	NAD-depen	GO:0005975|	GO:0005829|	GO:0004367|gl	K00006
Manes.09G01960(	0.4503868	0.00043	down	yes	ABC transpo	pfam00005	ABC_tran	ABC transp	GO:0080167|	GO:0016021|	GO:0005524|A'	.
Manes.09G01970(	2.8358017	1.38E-22	up	yes	Disease resist	pfam00931	NB-ARC	NB-ARC do	GO:0006952|	.	GO:0043531|A	.
Manes.09G01990(	2.9655713	3.85E-16	up	yes	Putative disea	pfam00931	NB-ARC	NB-ARC do	GO:0006952|	.	GO:0043531|A	.
Manes.09G02000(	3.3418332	3.27E-06	up	yes	.	pfam02076	STE3	Pheromone	.	.	.	.
Manes.09G02070(	0.722098	0.00575	down	no	AP2-like ethy	pfam00847	AP2	AP2 domair	GO:0009873|	GO:0005634|	GO:0003677|D	.
Manes.09G02090(	0.7081284	0.0013	down	no	.	pfam14612	Ino80_Iec3	IEC3 subun	.	.	.	.
Manes.09G02130(	0.8313808	0.08333	down	no	Protein FAR1	pfam03101	FAR1	FAR1 DNA	GO:0006355|	GO:0005634|	GO:0008270|zi	K17604
Manes.09G02150(	0.9775018	0.84829	down	no	Zinc-metallo	pfam00675	Peptidase_M16	Insulinase (I	GO:0043171|	GO:0005739|	GO:0004222|m	K01408
Manes.09G02170(	0.271861	0.0001	down	yes	.	pfam04468	PSP1	PSP1 C-term	.	.	.	.
Manes.09G02180(	0.3769535	1.07E-07	down	yes	Glutathione S	pfam13417	GST_N_3	Glutathione	GO:0046686|	GO:0005829|	GO:0004364|gl	K00799
Manes.09G02190(	1.557431	2.17E-06	up	no	.	pfam03385	DUF288	Protein of u	.	.	.	.
Manes.09G02200(	1.6845565	2.31E-05	up	no	Cytoplasmic	pfam10288	DUF2392	Protein of u	GO:0010311|	GO:0005737|	GO:0016779|nu	K14169
Manes.09G02210(	0.549537	0.00377	down	no	Protein N-ter	pfam09764	Nt_Gln_amidase	N-terminal g	.	.	GO:0016811|hy	.
Manes.09G02220(	0.2149564	3.88E-06	down	yes	.	pfam03942	DTW	DTW doma	.	.	.	.
Manes.09G02230(	0.4536882	3.73E-14	down	yes	Leucine-rich	pfam00069	Pkinase	Protein kina	GO:0009755|	GO:0016021|	GO:0005524|A'	.
Manes.09G02250(	0.5327656	0.06895	down	no	Putative disea	pfam00931	NB-ARC	NB-ARC do	GO:0006952|	.	GO:0043531|A	.
Manes.09G02370(	1.1665382	0.10802	up	no	Cellulose syn	pfam03552	Cellulose_synt	Cellulose sy	GO:0071555|	GO:0005768|	GO:0016760|ce	K10999
Manes.09G02420(	1.2852513	0.06181	up	no	.	pfam14432	DYW_deaminase	DYW family	.	.	.	.
Manes.09G02430(	1.0805674	0.66547	up	no	Probable gala	pfam05691	Raffinose_syn	Raffinose sy	GO:0034484|	GO:0009506|	GO:0052692|ra	K06617
Manes.09G02470(	0.6454612	0.00169	down	no	Remorin	pfam03763	Remorin_C	Remorin, C-	GO:0007267|	GO:0005886|	GO:0048032|ga	.
Manes.09G02480(	1.0706854	0.62633	up	no	Putative disea	pfam00931	NB-ARC	NB-ARC do	GO:0006952|	.	GO:0043531|A	.
Manes.09G02520(	0.8370211	0.55851	down	no	Nuclear trans	pfam02045	CBFB_NFYA	CCAAT-bin	GO:0009793|	GO:0016602|	GO:0003677|D	K08064
Manes.09G02550(	4.7783117	2.04E-32	up	yes	Disease resist	pfam00931	NB-ARC	NB-ARC do	GO:0006952|	.	GO:0043531|A	.
Manes.09G02570(	10.300336	8.70E-06	up	yes	.	pfam04078	Rcd1	Cell differen	.	.	.	.
Manes.09G02640(	0.2673199	1.04E-27	down	yes	.	pfam00249	Myb_DNA-binding	Myb-like DI	.	.	.	.
Manes.09G02680(	0.8560383	0.11611	down	no	.	pfam12717	Cnd1	non-SMC m	.	.	.	.
Manes.09G02690(	0.6137334	0.00716	down	no	MATE efflux	pfam01554	MatE	MatE. The I	GO:0006811|	GO:0016021|	GO:0015297|an	.
Manes.09G02700(	0.6308321	0.26352	down	no	MATE efflux	pfam01554	MatE	MatE. The I	GO:0006811|	GO:0016021|	GO:0015297|an	.
Manes.09G02770(	0.4478345	0.005	down	yes	MATE efflux	pfam01554	MatE	MatE. The I	GO:0006811|	GO:0016021|	GO:0015297|an	.
Manes.09G02860(	1.1835236	0.07877	up	no	DNA-binding	pfam13087	AAA_12	AAA domai	GO:0032508|	GO:0030424|	GO:0005524|A'	.
Manes.09G02870(	0.5752673	4.33E-06	down	no	.	pfam02536	mTERF	mTERF. Th	.	.	.	.
Manes.09G02890(	0.956772	0.70115	down	no	Probable gluc	pfam03016	Exostosin	Exostosin fa	GO:0071555|	GO:0005794|	GO:0016757|tra	.
Manes.09G02950(	0.7203638	0.05765	down	no	Carboxyl-tern	pfam03572	Peptidase_S41	Peptidase fa	.	GO:0009543|	GO:0008236|se	.
Manes.09G02970(	2.3578661	0.09535	up	no	.	pfam01578	Cytochrom_C_asn	Cytochrome	.	.	.	.
Manes.09G02990(	1.6049892	1.09E-07	up	no	Serine/threon	pfam00069	Pkinase	Protein kina	GO:0043622|	.	GO:0005524|A'	K08857
Manes.09G03030(	1.1817461	0.06421	up	no	.	pfam07964	Red1	Rec10 / Rec	.	.	.	.
Manes.09G03060(	1.1135195	0.33499	up	no	Transcription	pfam10322	7TM_GPCR_Sru	Serpentine t	GO:0006355|	GO:0005634|	GO:0003677|D	.
Manes.09G03080(	0.9547491	0.64965	down	no	OTU domain	pfam02338	OTU	OTU-like cy	.	GO:0009507|	.	.
Manes.09G03150(	7.0296487	3.11E-32	up	yes	Polygalacturo	pfam08263	LRRNT_2	Leucine rich	.	GO:0048046|	.	.
Manes.09G03290(	0.5682365	0.00043	down	no	.	pfam02544	Steroid_dh	3-oxo-5-alp	.	.	.	.
Manes.09G03310(	0.8472931	0.17277	down	no	.	pfam02536	mTERF	mTERF. Th	.	.	.	.
Manes.09G03320(	0.6656971	0.0107	down	no	UBP1-associ	pfam00076	RRM_1	RNA recogn	GO:0008219|	GO:0005730|	GO:0000166|nu	K12741
Manes.09G03360(	0.2902727	0.00063	down	yes	.	pfam05553	DUF761	Cotton fibre	.	.	.	.
Manes.09G03420(	Inf	0.1623	up	no	.	pfam06876	SCRL	Plant self-in	.	.	.	.
Manes.09G03440(	1.6788084	2.18E-06	up	no	.	pfam03735	ENT	ENT domain	.	.	.	.
Manes.09G03470(	1.9610924	1.01E-07	up	no	Disease resist	pfam00931	NB-ARC	NB-ARC do	GO:0006952|	GO:0005886|	GO:0043531|A	.
Manes.09G03480(	1.8263971	0.0015	up	no	.	pfam03760	LEA_1	Late embryo	.	.	.	.
Manes.09G03500(	0.252041	1.58E-06	down	yes	.	pfam09764	Nt_Gln_amidase	N-terminal g	.	.	.	.
Manes.09G03560(	1.7600698	0.00037	up	no	Actin	pfam00022	Actin	Actin.	.	GO:0005737|	GO:0005524|A'	.
Manes.09G03590(	2.6223788	0.09604	up	no	Probable L-ty	pfam00139	Lectin_legB	Legume lect	GO:0009755|	GO:0016021|	GO:0005524|A'	.
Manes.09G03610(	0.4757886	0.00034	down	yes	Ribosomal pr	pfam00338	Ribosomal_S10	Ribosomal p	GO:0006412|	GO:0005739|	GO:0003735|str	.
Manes.09G03650(	2.4818509	6.89E-09	up	yes	Homeobox-le	pfam02183	HALZ	Homeobox a	GO:0006351|	GO:0005634|	GO:0043565|se	K09338
Manes.09G03690(	1.837493	8.58E-12	up	no	Thioredoxin-	pfam00085	Thioredoxin	Thioredoxin	GO:0045454|	GO:0009570|	GO:0015035|pr	.
Manes.09G03720(	0.8793158	0.22882	down	no	E3 ubiquitin-	pfam03145	Sina	Seven in abs	GO:0007275|	GO:0005634|	GO:0016874|lig	K04506
Manes.09G03740(	0.3193724	4.33E-17	down	yes	Activator of	pfam09229	Aha1_N	Activator of	GO:0032781|	GO:0005737|	GO:0001671|A'	.
Manes.09G03750(	0.6148634	1.40E-05	down	no	.	pfam09637	Med18	Med18 prot	.	.	.	.
Manes.09G03790(	NA	NA	--	no	.	pfam13912	zf-C2H2_6	C2H2-type	.	.	.	.
Manes.09G03830(	0.3572806	8.71E-22	down	yes	Uncharacteriz	pfam07035	Mic1	Colon cance	.	GO:0005765|	.	.
Manes.09G03840(	0.3159712	9.43E-12	down	yes	.	pfam05895	DUF859	Siphovirus p	.	.	.	.
Manes.09G03860(	0.353374	1.03E-19	down	yes	Zinc finger pr	pfam13894	zf-C2H2_4	C2H2-type	GO:0007275|	GO:0005737|	GO:0046872|m	.
Manes.09G03880(	0.7809202	0.04166	down	no	60S ribosoma	pfam00297	Ribosomal_L3	Ribosomal p	GO:0006412|	GO:0022625|	GO:0003735|str	K02925
Manes.09G03910(	0.3452625	4.55E-05	down	yes	Dirigent prote	pfam03018	Dirigent	Dirigent-lik	.	GO:0048046|	.	.
Manes.09G03940(	0.9661461	0.88405	down	no	Cysteine prot	pfam00031	Cystatin	Cystatin dor	GO:0006952|	GO:0005618|	GO:0004869|cy	.
Manes.09G03950(	0.9942061	0.97443	down	no	Carbon catab	pfam03372	Exo_endo_phos	Endonuclea	GO:0006397|	GO:0000932|	GO:0046872|m	K12603
Manes.09G03980(	1.9080671	1.06E-12	up	no	DEAD-box A	pfam00270	DEAD	DEAD/DEA	.	.	GO:0005524|A'	K11594
Manes.09G04000(	0.5954349	8.59E-06	down	no	Lipid phosph	pfam01569	PAP2	PAP2 super	GO:0009737|	GO:0005789|	GO:0016787|hy	.
Manes.09G04010(	4.5185717	1.48E-31	up	yes	F-box/kelch-r	pfam01344	Kelch_1	Kelch motif	GO:0080037|	GO:0005829|	.	.
Manes.09G04020(	0.4144613	2.75E-13	down	yes	Serine/argini	pfam00076	RRM_1	RNA recogn	GO:0006397|	GO:0005737|	GO:0042802|id	K12900
Manes.09G04030(	1.7303247	2.43E-09	up	no	Protein RRP5	pfam00575	S1	S1 RNA bin	GO:0006397|	GO:0005829|	GO:0044822|po	K14792
Manes.09G04060(	0.3045333	1.84E-08	down	yes	TNF recepto	pfam02176	zf-TRAF	TRAF-type	GO:0006955|	GO:0005737|	GO:0004842|ub	.
Manes.09G04070(	0.8527199	0.11227	down	no	Eukaryotic tr	pfam05470	eIF-3c_N	Eukaryotic t	GO:0001731|	GO:0016282|	GO:0003743|tra	.
Manes.09G04110(	0.8181878	0.14657	down	no	Pentatricoper	pfam12569	NARP1	NMDA rece	.	GO:0005739|	.	.
Manes.09G04160(	0.8640562	0.19886	down	no	.	pfam04576	Zein-binding	Zein-binding	.	.	.	.
Manes.09G04190(	1.3432916	0.04233	up	no	ABC transpo	pfam01061	ABC2_membrane	ABC-2 type	GO:0009414|	GO:0016021|	GO:0005524|A'	.
Manes.09G04200(	2.1260884	0.01243	up	yes	.	pfam03104	DNA_pol_B_exo1	DNA polym	.	.	.	.
Manes.09G04210(	0.7935863	0.04816	down	no	Sucrose-phos	pfam05116	S6PP	Sucrose-6F-	GO:0005986|	.	GO:0000287|m	.
Manes.09G04230(	0.610093	0.04779	down	no	.	pfam02411	MerT	MerT mercu	.	.	.	.
Manes.09G04250(	0.7983163	0.02039	down	no	Fasciclin-like	pfam02469	Fasciclin	Fasciclin do	.	GO:0005576|	.	.
Manes.09G04280(	6.566274	1.71E-61	up	yes	.	pfam00009	GTP_EFTU	Elongation f	.	.	.	.

Manes.09G04290(	Inf	0.00119	up	yes	.	pfam14857	TMEM151	TMEM151	.	.	.	.
Manes.09G04330(	1.272919	0.00892	up	no	Ubiquitin carl	pfam00443	UCH	Ubiquitin ca	GO:0006511|u	.	GO:0008234|cy	K11835
Manes.09G04340(	2.2935803	1.05E-20	up	yes	.	pfam03798	TRAM_LAG1_CI	TLC domain	.	.	.	.
Manes.09G04350(	0.3012989	5.57E-08	down	yes	Uncharacteriz	pfam00462	Glutaredoxin	Glutaredoxi	GO:0045454|	GO:0005886|	GO:0009055|ele	.
Manes.09G04390(	Inf	0.9009	up	no	Secoisolaricir	pfam13561	adh_short_C2	Enoyl-(Acyl	.	.	GO:0016491|ox	.
Manes.09G04460(	7.6903355	1.64E-87	up	yes	Syntaxin-121	pfam05739	SNARE	SNARE dor	GO:0006952|	GO:0009504|	GO:0043495|pr	K08486
Manes.09G04470(	1.7494687	4.89E-08	up	no	Testis-expres	pfam12333	Ipi1_N	Rix1 comple	.	GO:0005737|	.	K14827
Manes.09G04480(	1.0266701	0.93811	up	no	.	pfam08538	DUF1749	Protein of u	.	.	.	.
Manes.09G04490(	15.07653	8.48E-99	up	yes	.	pfam05985	EutC	Ethanolamir	.	.	.	.
Manes.09G04510(	1.0272392	0.78281	up	no	Testis-expres	pfam12333	Ipi1_N	Rix1 comple	.	GO:0005737|	.	K14827
Manes.09G04520(	0.2438259	1.99E-35	down	yes	Receptor pro	pfam07714	Pkinase_Tyr	Protein tyro	GO:0009755|l	GO:0005576|	GO:0005524|A	K00924
Manes.09G04570(	1.3397914	0.01797	up	no	BTB/POZ do	pfam00651	BTB	BTB/POZ d	GO:0016567|	.	.	.
Manes.09G04610(	58.55955	2.09E-23	up	yes	Dof zinc fing	pfam02701	zf-Dof	Dof domain	GO:0006355|	GO:0005634|	GO:0003677|D	.
Manes.09G04620(	0.9032082	0.31846	down	no	.	pfam10998	DUF2838	Protein of u	.	.	.	.
Manes.09G04640(	110.0543	5.03E-40	up	yes	L-type lectin-	pfam00139	Lectin_legB	Legume lect	GO:0009755|l	GO:0016021|	GO:0005524|A	.
Manes.09G04660(	0.490713	9.20E-05	down	yes	.	pfam03168	LEA_2	Late embryc	.	.	.	.
Manes.09G04730(	0.4541784	0.00386	down	yes	Aspartic prot	pfam14541	TAXi_C	Xylanase inl	.	GO:0005576|	GO:0004190|as	.
Manes.09G04750(	1.112034	0.25799	up	no	2-Cys peroxi	pfam00578	AhpC-TSA	AhpC/TSA	GO:0042742|	GO:0048046|	GO:0004601|pe	K03386
Manes.09G04770(	0.6830309	0.03385	down	no	Protein CHU	pfam08317	Spc7	Spc7 kineto	GO:0009902|	GO:0009507|	.	.
Manes.09G04800(	Inf	0.9009	up	no	Putative ribo	pfam13966	zf-RVT	zinc-binding	.	.	GO:0046872|m	.
Manes.09G04820(	3.9224764	2.84E-14	up	yes	.	pfam02175	7TM_GPCR_Srb	Serpentine t	.	.	.	.
Manes.09G04830(	1.2643141	0.00999	up	no	.	pfam00628	PHD	PHD-finger.	.	.	.	.
Manes.09G04900(	0.580995	0.0101	down	no	Probable CCl	pfam04857	CAF1	CAF1 family	GO:0042742|	GO:0030014|	GO:0008408|3'-	K12581
Manes.09G04950(	1.8116887	4.16E-06	up	no	.	pfam13198	DUF4014	Protein of u	.	.	.	.
Manes.09G04970(	0.8114042	0.07471	down	no	40S ribosoma	pfam00833	Ribosomal_S17e	Ribosomal S	GO:0006412|t	GO:0005840|	GO:0003735|str	K02962
Manes.09G05020(	NA	NA	--	no	Pumilio homo	pfam05938	Self-incomp_S1	Plant self-in	GO:0006417|	GO:0005737|	GO:0003723|R	.
Manes.09G05050(	0.4823605	1.00E-12	down	yes	Secoisolaricir	pfam00106	adh_short	short chain	.	.	GO:0016491|ox	.
Manes.09G05060(	0.7952918	0.02134	down	no	Secoisolaricir	pfam13561	adh_short_C2	Enoyl-(Acyl	.	.	GO:0016491|ox	.
Manes.09G05080(	1.3571359	0.04602	up	no	.	pfam13041	PPR_2	PPR repeat	.	.	.	.
Manes.09G05110(	0.4540842	4.58E-09	down	yes	Uncharacteriz	pfam01576	Myosin_tail_1	Myosin tail.	.	GO:0009507|	.	.
Manes.09G05120(	0.4207384	6.21E-06	down	yes	Lysosomal Pr	pfam05577	Peptidase_S28	Serine carbc	GO:0043535|	GO:0005764|	GO:0004180|ca	K01285
Manes.09G05140(	3.7120729	3.71E-18	up	yes	B3 domain-c	pfam02362	B3	B3 DNA bir	GO:0045892|	GO:0005634|	GO:0003677|D	.
Manes.09G05160(	2.356242	1.27E-07	up	yes	.	pfam12937	F-box-like	F-box-like.	.	.	.	.
Manes.09G05230(	0.7806732	0.01043	down	no	Probable UD	pfam13844	Glyco_transf_41	Glycosyl tra	GO:0009740|	GO:0005634|	GO:0016757|tra	.
Manes.09G05240(	4.2207736	0.3795	up	no	WD repeat-c	pfam00400	WD40	WD domain	GO:0009718|	GO:0005634|	GO:0044212|tra	K11805
Manes.09G05260(	NA	NA	--	no	NAC domain	pfam02365	NAM	No apical m	GO:0006351|t	GO:0005634|	GO:0003677|D	.
Manes.09G05270(	0.5346645	1.21E-07	down	no	Cyclin-depen	pfam00069	Pkinase	Protein kina	GO:0007049|	GO:0005634|	GO:0005524|A	K02208
Manes.09G05280(	1.4156229	0.61142	up	no	NAC domain	pfam02365	NAM	No apical m	GO:0006351|t	GO:0005634|	GO:0003677|D	.
Manes.09G05290(	3.7150768	7.43E-08	up	yes	.	pfam04520	Senescence_reg	Senescence	.	.	.	.
Manes.09G05300(	1.603544	2.15E-07	up	no	Sorting nexin	pfam00787	PX	PX domain.	GO:0010252|	GO:0005829|	GO:0035091|ph	K17917
Manes.09G05310(	2.4845767	4.54E-15	up	yes	Cholesterol o	pfam05199	GMC_oxred_C	GMC oxido	GO:0008203|	GO:0005622|	GO:0016995|ch	K03333
Manes.09G05330(	1.9278202	1.34E-10	up	no	Alpha/beta hy	pfam12697	Abhydrolase_6	Alpha/beta l	.	GO:0005739|	GO:0016787|hy	.
Manes.09G05390(	Inf	0.9009	up	no	.	pfam12869	tRNA_anti-like	tRNA_anti-	.	.	.	.
Manes.09G05400(	1.0197202	0.83206	up	no	Rho GTPase-	pfam00620	RhoGAP	RhoGAP do	GO:0007165|	.	GO:0005096|G	.
Manes.09G05440(	1.5391542	2.29E-06	up	no	Heterogeneou	pfam00076	RRM_1	RNA recogr	GO:0007623|	GO:0070937|	GO:0000166|nu	K13160
Manes.09G05500(	12.354119	3.42E-23	up	yes	.	pfam14009	DUF4228	Domain of u	.	.	.	.
Manes.09G05510(	0.2785943	6.86E-06	down	yes	.	pfam13041	PPR_2	PPR repeat	.	.	.	.
Manes.09G05570(	0.5019903	3.76E-10	down	no	.	pfam03039	IL12	Interleukin-	.	.	.	.
Manes.09G05580(	0.8110841	0.02864	down	no	Cell division	pfam12327	FtsZ_C	FtsZ family,	GO:0010020|	GO:0009507|	GO:0005525|G	K03531
Manes.09G05590(	0.5120502	3.62E-05	down	no	Iron-sulfur as	pfam01521	Fe-S_biosyn	Iron-sulphur	GO:0016226|i	GO:0005739|	GO:0051536|irc	.
Manes.09G05600(	0.6703306	0.02351	down	no	.	pfam00646	F-box	F-box doma	.	.	.	.
Manes.09G05610(	1.1767636	0.72823	up	no	Protein ABS	pfam00170	bZIP_1	bZIP transcr	GO:0009738|	GO:0005634|	GO:0043565|se	K14432
Manes.09G05630(	3.7533112	0.39312	up	no	Protein TERI	pfam01161	PBP	Phosphatidy	GO:0030154|	GO:0005737|	GO:0003712|tra	.
Manes.09G05650(	0.638536	0.0024	down	no	CAAX preny	pfam02517	Abi	CAAX prot	GO:0071586|	GO:0005783|	GO:0004197|cy	K08658
Manes.09G05660(	3.9539544	2.60E-10	up	yes	.	pfam09311	Rab5-bind	Rabaptin-lik	.	.	.	.
Manes.09G05670(	1.9767152	6.42E-12	up	no	Transcription	pfam03847	TFIID_20kDa	Transcriptio	GO:0006352|	GO:0005669|	.	K03126
Manes.09G05700(	0.738125	0.04488	down	no	GDSL estera	pfam00657	Lipase_GDSL	GDSL-like l	GO:0016042|	GO:0005576|	GO:0016788|hy	.
Manes.09G05740(	0.6873895	0.00276	down	no	Transcription	pfam00170	bZIP_1	bZIP transcr	GO:0006351|t	GO:0005634|	GO:0043565|se	.
Manes.09G05770(	0.2851306	5.58E-29	down	yes	ABC transpo	pfam00664	ABC_membrane	ABC transp	GO:0010541|	GO:0016021|	GO:0005524|A	K05658
Manes.09G05780(	2.0271537	5.53E-15	up	yes	.	pfam01925	TauE	Sulfite expo	.	.	.	.
Manes.09G05820(	1.1902327	0.17022	up	no	SRSF protein	pfam00069	Pkinase	Protein kina	GO:0007059|	GO:0005737|	GO:0005524|A	K15409
Manes.09G05830(	1.687465	0.0004	up	no	Pentatricope	pfam13041	PPR_2	PPR repeat	.	GO:0005739|	GO:0008270|zi	.
Manes.09G05850(	0.3710539	2.96E-07	down	yes	Serine/threon	pfam00069	Pkinase	Protein kina	GO:0035556|i	GO:0005737|	GO:0005524|A	.
Manes.09G05880(	1.4349441	5.43E-05	up	no	Monodehydro	pfam07992	Pyr_redox_2	Pyridine nuc	.	GO:0005737|	GO:0050660|fla	K08232
Manes.09G05940(	0.6009505	0.01585	down	no	1,4-alpha-glu	pfam02806	Alpha-amylase_C	Alpha amyla	GO:0009790|	GO:0009501|	GO:0003844|1,	.
Manes.09G05950(	1.7273454	6.22E-06	up	no	Growth-regu	pfam08879	WRC	WRC. The	GO:0048366|	GO:0005634|	GO:0005524|A	.
Manes.09G05970(	0.9747145	0.81701	down	no	Photosystem	pfam00421	PSII	Photosysten	GO:0009772|	GO:0009535|	GO:0016168|ch	.
Manes.09G06000(	1.3467164	0.00092	up	no	Enolase 2	pfam00113	Enolase_C	Enolase, C-	GO:0006096|	GO:0000015|	GO:0000287|ma	.
Manes.09G06020(	0.981426	0.87638	down	no	Splicing facto	pfam00642	zf-CCCH	Zinc finger	GO:0006397|	GO:0016607|	GO:0003677|D	K12836
Manes.09G06040(	0.9199928	0.40417	down	no	Histone deac	pfam12171	zf-C2H2_jaz	Zinc-finger	GO:0016568|	GO:0005730|	GO:0016787|hy	.
Manes.09G06060(	4.0443029	5.64E-11	up	yes	Heat stress tr	pfam00447	HSF_DNA-bind	HSF-type D	GO:0006355|	GO:0005634|	GO:0003677|D	K09419
Manes.09G06080(	0.5191218	2.30E-06	down	no	Protein ROS	pfam15628	RRM_DME	RRM in Der	GO:0006284|l	GO:0005730|	GO:0051539|4	.
Manes.09G06100(	0.6850643	0.00685	down	no	Uridylate kin	pfam00696	AA_kinase	Amino acid	GO:0009793|	GO:0005634|	GO:0003677|D	K09903
Manes.09G06150(	0.4747724	4.12E-11	down	yes	Protein kinas	pfam00069	Pkinase	Protein kina	GO:0051301|	GO:0032153|	GO:0005524|A	K08832
Manes.09G06220(	2.9334465	0.00049	up	yes	Zinc finger pr	pfam13912	zf-C2H2_6	C2H2-type	GO:0006355|	GO:0005634|	GO:0046872|m	.
Manes.09G06280(	Inf	0.38692	up	no	Aspartic prot	pfam14543	TAXi_N	Xylanase inl	.	GO:0005576|	GO:0004190|as	.
Manes.09G06300(	20.398456	0.00025	up	yes	Transcription	pfam00249	Myb_DNA-binding	Myb-like DI	GO:0010438|	GO:0005634|	GO:0003677|D	K09422
Manes.09G06370(	6.8016841	1.71E-19	up	yes	Phenylalanine	pfam00221	Lyase_aromatic	Aromatic an	GO:0009800|	GO:0005737|	GO:0045548|ph	.
Manes.09G06390(	0.6418325	0.00081	down	no	.	pfam14897	EpsG	EpsG family	.	.	.	.
Manes.09G06420(	1.1726962	0.12009	up	no	Translin	pfam01997	Translin	Translin fam	.	GO:0005634|	GO:0004519|en	.
Manes.09G06480(	0.6731535	0.00767	down	no	Probable xylo	pfam00722	Glyco_hydro_16	Glycosyl hy	GO:0042546|	GO:0048046|	GO:0004553|hy	.
Manes.09G06520(	18.807404	#######	up	yes	S-adenosylme	pfam02772	S-AdoMet_synt_M	S-adenosyln	GO:0006730|	GO:0005737|	GO:0005524|A	.
Manes.09G06550(	0.9180174	0.6375	down	no	4-coumarate-	pfam00501	AMP-binding	AMP-bindin	GO:0009695|	GO:0005777|	GO:0005524|A	K01904
Manes.09G06560(	1.0221925	1	up	no	.	pfam10150	RNase_E_G	Ribonucleas	.	.	.	.
Manes.09G06570(	0.5921074	1.23E-07	down	no	ABC transpo	pfam00664	ABC_membrane	ABC transp	GO:0010541|	GO:0016021|	GO:0005524|A	K05658
Manes.09G06600(	0.661918	0.00068	down	no	Transcription	pfam08781	DP	Transcriptio	GO:0042023|	GO:0005737|	GO:0003677|D	.
Manes.09G06630(	9.6777692	#######	up	yes	.	pfam00403	HMA	Heavy-meta	.	.	.	.
Manes.09G06640(	0.1828254	0.00126	down	yes	.	pfam02928	zf-C5HC2	C5HC2 zinc	.	.	.	.

Manes.09G06700(	0.4290907	7.78E-06	down	yes	.	pfam15479	DUF4639	Domain of u	.	.	.	.
Manes.09G06720(	0.812805	0.04967	down	no	TSL-kinase i	pfam00249	Myb_DNA-binding	Myb-like DI	GO:0006355|	GO:0005634|	GO:0003677|DI	.
Manes.09G06760(	3.8424265	1.43E-43	up	yes	OTU domain	pfam02338	OTU	OTU-like cy	.	.	GO:0008234|cy	.
Manes.09G06770(	1.1599933	0.12332	up	no	Probable rece	pfam00069	Pkinase	Protein kina	.	GO:0016021|	GO:0005524|A'	.
Manes.09G06780(	1.5672792	3.27E-06	up	no	RNA-binding	pfam00076	RRM_1	RNA recogi	GO:0045087|	GO:0009507|	GO:0000166|nu	K11294
Manes.09G06810(	0.5233616	4.22E-07	down	no	Cytokinin rib	pfam03641	Lysine_decarbox	Possible lysi	GO:0009691|	GO:0005737|	GO:0016799|hy	.
Manes.09G06830(	1.2322706	0.02615	up	no	Ubiquitin-like	pfam00240	ubiquitin	Ubiquitin fa	GO:0006464|	GO:0005737|	GO:0031386|pr	K13113
Manes.09G06850(	5.5871867	3.84E-06	up	yes	.	pfam05086	Dicty_REP	Dictyosteliu	.	.	.	.
Manes.09G06880(	1.871616	0.00436	up	no	Probable aqu	pfam00230	MIP	Major intrin	.	GO:0016021|	GO:0005215|tra	K09872
Manes.09G06900(	0.8460124	0.3153	down	no	Probable E3	pfam13920	zf-C3HC4_3	Zinc finger,	GO:1901527|	GO:0005886|	GO:0016874|lig	.
Manes.09G06920(	0.8530853	0.27435	down	no	Probable cata	pfam00400	WD40	WD domain	GO:0045013|	GO:0005829|	.	.
Manes.09G06940(	0.6704937	0.00377	down	no	.	pfam01490	Aa_trans	Transmemb	.	.	.	.
Manes.09G06950(	0.4315822	0.00058	down	yes	DnaJ protein	pfam00226	DnaJ	DnaJ domai	GO:0044794|	GO:0005783|	.	K09523
Manes.09G06960(	2.4293352	1.03E-10	up	yes	Zinc finger p	pfam00096	zf-C2H2	Zinc finger,	GO:0051302|	GO:0005634|	GO:0003677|DI	.
Manes.09G06980(	0.7587763	0.25038	down	no	Beta-xylosida	pfam00933	Glyco_hydro_3	Glycosyl hy	GO:0045493|	GO:0005578|	GO:0046556|al	.
Manes.09G07020(	Inf	0.04209	up	no	.	pfam12786	GBV-C_env	GB virus C	.	.	.	.
Manes.09G07050(	4.0376374	2.04E-07	up	yes	.	pfam14547	Hydrophob_seed	Hydrophobi	.	.	.	.
Manes.09G07060(	0.5405687	5.68E-09	down	no	Zinc finger C	pfam04192	Utp21	Utp21 speci	.	.	GO:0003677|DI	.
Manes.09G07100(	0.7946662	0.32231	down	no	Serine/threon	pfam07714	Pkinase_Tyr	Protein tyro	GO:0009742|	GO:0005886|	GO:0005524|A'	.
Manes.09G07110(	1.0727041	0.4489	up	no	Glutathione S	pfam13417	GST_N_3	Glutathione	GO:0046686|	GO:0005829|	GO:0004364|gl	K00799
Manes.09G07150(	0.2692849	0.00026	down	yes	.	pfam07062	Clc-like	Clc-like. Th	.	.	.	.
Manes.09G07180(	0.8944538	0.38453	down	no	Dirigent prot	pfam10324	7TM_GPCR_Srw	Serpentine t	.	GO:0048046|	.	.
Manes.09G07230(	1.2681674	0.01385	up	no	Ras-related p	pfam00071	Ras	Ras family.	GO:0006888|	GO:0005789|	GO:0005525|G	K07877
Manes.09G07280(	0.6350998	0.00059	down	no	.	pfam00300	His_Phos_1	Histidine ph	.	.	.	.
Manes.09G07350(	2.8141334	2.23E-12	up	yes	.	pfam01194	RNA_pol_N	RNA polym	.	.	.	.
Manes.09G07360(	1.5790727	1.65E-05	up	no	Calmodulin	pfam13499	EF-hand_7	EF-hand do	.	.	GO:0005509|ca	.
Manes.09G07370(	1.4280068	0.00029	up	no	Probable prot	pfam01529	zf-DHHC	DHHC paln	.	GO:0016021|	GO:0019706|pr	.
Manes.09G07390(	0.6370546	0.01994	down	no	.	pfam03745	DUF309	Domain of u	.	.	.	.
Manes.09G07400(	1.8495084	8.99E-07	up	no	Probable aqu	pfam00230	MIP	Major intrin	.	GO:0005789|	GO:0005215|tra	.
Manes.09G07410(	1.5956915	0.00036	up	no	Probable aqu	pfam09335	SNARE_assoc	SNARE ass	.	GO:0005789|	GO:0005215|tra	.
Manes.09G07450(	0.9419301	0.58437	down	no	Alpha-L-arab	pfam06964	Alpha-L-AF_C	Alpha-L-ara	GO:0046373|	GO:0048046|	GO:0046556|al	K01209
Manes.09G07470(	0.4781026	0.00012	down	yes	DNA topoiso	pfam06839	zf-GRF	GRF zinc fi	GO:0006265|	GO:0005694|	GO:0003677|DI	K03165
Manes.09G07490(	5.3674798	6.39E-76	up	yes	.	pfam11893	DUF3413	Domain of u	.	.	.	.
Manes.09G07510(	2.6831772	0.00107	up	yes	Inositol trans	pfam00083	Sugar_tr	Sugar (and	GO:0046323|	GO:0005887|	GO:0005355|gl	K08150
Manes.09G07550(	0.9158677	0.40395	down	no	Cytochrome	pfam00067	p450	Cytochrome	.	GO:0031969|	GO:0020037|he	.
Manes.09G07560(	0.6001475	0.0002	down	no	Pentatricope	pfam13041	PPR_2	PPR repeat	GO:0009793|	.	.	.
Manes.09G07590(	1.1935205	0.55039	up	no	Cytochrome	pfam04526	DUF568	Protein of u	GO:0055114|	GO:0016021|	GO:0046872|m	.
Manes.09G07610(	Inf	0.9009	up	no	.	pfam10393	Matrilin_ccoil	Trimeric coi	.	.	.	.
Manes.09G07620(	0.8206876	0.06229	down	no	Putative vacu	pfam05090	VKG_Carbox	Vitamin K-c	GO:0045053|	GO:0019898|	.	.
Manes.09G07630(	0.7567764	0.00425	down	no	Putative vacu	pfam12624	Chorein_N	N-terminal	GO:0045053|	GO:0019898|	.	.
Manes.09G07720(	2.0266252	8.75E-16	up	yes	E3 ubiquitin-	pfam00632	HECT	HECT-dom	GO:0010150|	GO:0005737|	GO:0030246|ca	K10591
Manes.09G07740(	0.6540723	0.00015	down	no	F-box protei	pfam00646	F-box	F-box doma	GO:0031348|	GO:0005737|	.	.
Manes.09G07760(	NA	NA	--	no	Exopolygala	pfam00295	Glyco_hydro_28	Glycosyl hy	GO:0071555|	GO:0005618|	GO:0047911|ga	.
Manes.09G07780(	NA	NA	--	no	ATP-depend	pfam00365	PFK	Phosphofru	GO:0046835|	GO:0005829|	GO:0003872|6-	K00850
Manes.09G07790(	0.4998645	1.42E-06	down	yes	Glucan endo-	pfam00332	Glyco_hydro_17	Glycosyl hy	GO:0005975|	GO:0031225|	GO:0042973|gl	.
Manes.09G07900(	1.0251704	0.95862	up	no	CASP-like p	pfam04535	DUF588	Domain of u	.	GO:0016021|	.	.
Manes.09G07930(	NA	NA	--	no	Poly(A)-spec	pfam04857	CAF1	CAF1 famil	GO:0006952|	GO:0005737|	GO:0046872|m	K01148
Manes.09G07940(	48.594751	8.17E-11	up	yes	Premnaspiro	pfam00067	p450	Cytochrome	.	GO:0016021|	GO:0020037|he	.
Manes.09G08010(	2.43708	1.03E-07	up	yes	Floral homeo	pfam00847	AP2	AP2 domai	GO:0030154|	GO:0005634|	GO:0003677|DI	K09284
Manes.09G08020(	3.4778596	1.07E-05	up	yes	.	pfam01567	Hanta_G1	Hantavirus	.	.	.	.
Manes.09G08040(	1.4512208	4.15E-05	up	no	CCR4-NOT	pfam14570	zf-RING_4	RING/Ubox	GO:0010467|	GO:0005829|	GO:0016874|lig	K10643
Manes.09G08140(	1.841636	6.34E-12	up	no	Protein IQ-D	pfam13178	DUF4005	Protein of u	.	GO:0005634|	.	.
Manes.09G08210(	0.7629743	0.22922	down	no	.	pfam15499	Peptidase_C98	Ubiquitin-sp	.	.	.	.
Manes.09G08290(	1.0057812	0.92825	up	no	Oligopeptide	pfam03169	OPT	OPT oligop	GO:0007275|	GO:0016021|	.	.
Manes.09G08300(	5.7538268	1.79E-08	up	yes	.	pfam01190	Pollen_Ole_e_I	Pollen prote	.	.	.	.
Manes.09G08470(	0.7147174	0.00116	down	no	Nuclear inhib	pfam00498	FHA	FHA domai	GO:0006397|	GO:0016607|	GO:0003677|DI	K13216
Manes.09G08500(	0.9859995	0.93803	down	no	DNA-directe	pfam03874	RNA_pol_Rpb4	RNA polym	GO:0006351|	GO:0000418|	GO:0003899|DI	K03012
Manes.09G08510(	0.4000937	1.69E-08	down	yes	Glucan endo-	pfam00332	Glyco_hydro_17	Glycosyl hy	GO:0005975|	GO:0031225|	GO:0042973|gl	.
Manes.09G08560(	0.3746438	2.20E-09	down	yes	Copper trans	pfam00403	HMA	Heavy-meta	GO:0006825|	GO:0000785|	GO:0046872|m	K07213
Manes.09G08580(	1.4416447	0.00047	up	no	.	pfam14559	TPR_19	Tetratricope	.	.	.	.
Manes.09G08610(	1.5615649	0.67668	up	no	Probable glut	pfam13417	GST_N_3	Glutathione	GO:0009734|	.	GO:0004364|gl	.
Manes.09G08630(	NA	NA	--	no	Probable glut	pfam00043	GST_C	Glutathione	GO:0009734|	.	GO:0004364|gl	.
Manes.09G08640(	1.1505551	1	up	no	Probable glut	pfam13417	GST_N_3	Glutathione	GO:0009734|	.	GO:0004364|gl	.
Manes.09G08650(	0.5066247	7.36E-08	down	no	.	pfam14905	OMP_b-brl_3	Outer memb	.	.	.	.
Manes.09G08660(	1.6488449	5.42E-08	up	no	Ras-related p	pfam00071	Ras	Ras family.	GO:0009873|	GO:0000139|	GO:0005525|G	K07901
Manes.09G08670(	2.4147382	5.39E-13	up	yes	.	pfam06423	GWT1	GWT1. Gly	.	.	.	.
Manes.09G08690(	1.2712529	0.05707	up	no	Glycine-rich	pfam00076	RRM_1	RNA recog	GO:0009651|	GO:0005768|	GO:0000166|nu	.
Manes.09G08710(	0.8000256	0.0453	down	no	UNC93-like	pfam05978	UNC-93	Ion channel	.	GO:0016021|	.	.
Manes.09G08770(	NA	NA	--	no	Exopolygala	pfam00295	Glyco_hydro_28	Glycosyl hy	GO:0071555|	GO:0005618|	GO:0047911|ga	.
Manes.09G08890(	1.0709874	0.44563	up	no	Chromodoma	pfam00628	PHD	PHD-finger.	GO:0043044|	GO:0005813|	GO:0005524|A'	K11643
Manes.09G08950(	1.073261	0.50641	up	no	Protein trans	pfam07732	Cu-oxidase_3	Multicoppe	GO:0006888|	GO:0030127|	GO:0008270|zi	.
Manes.09G08980(	0.2671518	2.45E-05	down	yes	.	pfam02028	BCCT	BCCT famil	.	.	.	.
Manes.09G09000(	0.3456868	6.38E-08	down	yes	RING-H2 fin	pfam13639	zf-RING_2	Ring finger	GO:0016567|	GO:0016021|	GO:0008270|zi	.
Manes.09G09020(	0.8987284	0.40944	down	no	Cation/H(+)	pfam00999	Na_H_Exchanger	Sodium/hyd	GO:0030007|	GO:0012505|	GO:0015299|so	.
Manes.09G09060(	0.6163163	0.01569	down	no	Diaminopime	pfam01678	DAP_epimerase	Diaminopim	GO:0009089|	GO:0009507|	GO:0008837|di	K01778
Manes.09G09090(	1.7497134	0.4644	up	no	Probable glut	pfam13417	GST_N_3	Glutathione	GO:0009734|	.	GO:0004364|gl	.
Manes.09G09110(	0.8753023	0.19776	down	no	Palmitoleoyl-	pfam03283	PAE	Pectinacetyl	GO:0030178|	GO:0005576|	GO:1990699|pa	.
Manes.09G09160(	0.9604086	0.69633	down	no	E3 ubiquitin-	pfam13639	zf-RING_2	Ring finger	GO:0009790|	GO:0005737|	GO:0016874|lig	.
Manes.09G09170(	0.9327394	0.4847	down	no	E3 ubiquitin-	pfam02207	zf-UBR	Putative zin	GO:0071233|	GO:0005737|	GO:0070728|le	K10625
Manes.09G09200(	0.4176688	5.66E-15	down	yes	.	pfam03753	HHV6-IE	Human herp	.	.	.	.
Manes.09G09240(	1.2424486	0.69589	up	no	.	pfam01807	zf-CHC2	CHC2 zinc	.	.	.	.
Manes.09G09250(	1.0319501	0.74548	up	no	ATP-depend	pfam01434	Peptidase_M41	Peptidase fa	.	GO:0016021|	GO:0005524|A'	K08956
Manes.09G09260(	2.1627047	6.24E-09	up	yes	Uncharacteri	pfam13419	HAD_2	Haloacid de	GO:0016311|	GO:0005737|	GO:0008252|nu	K18551
Manes.09G09270(	0.820302	0.05118	down	no	Uncharacteri	pfam13460	NAD_binding_10	NADH(P)-l	GO:0042742|	GO:0048046|	GO:0005507|co	.
Manes.09G09290(	0.5704269	3.59E-08	down	no	Transcription	pfam00249	Myb_DNA-binding	Myb-like DI	GO:0008356|	GO:0000793|	GO:0003677|DI	K09422
Manes.09G09300(	1.8103494	2.96E-10	up	no	Probable seri	pfam00069	Pkinase	Protein kina	GO:0009755|	GO:0005886|	GO:0005524|A'	.
Manes.09G09320(	0.2187014	8.86E-22	down	yes	Expansin-A4	pfam01357	Pollen_allerg_1	Pollen aller	GO:0009664|	GO:0005829|	.	.
Manes.09G09350(	Inf	0.9009	up	no	Exopolygala	pfam00295	Glyco_hydro_28	Glycosyl hy	GO:0005975|	GO:0005618|	GO:0047911|ga	.

Manes.09G09430(	0.3301906	2.22E-09	down	yes	Aspartic prot	pfam14543	TAXi_N	Xylanase inl	GO:0012501|]	GO:0005783|	GO:0004190|as	.
Manes.09G09470(	3.2024092	7.50E-20	up	yes	L-type lectin-	pfam00139	Lectin_legB	Legume lect	GO:0009755|l	GO:0016021|	GO:0005524|A'	.
Manes.09G09490(	2.9490779	4.36E-09	up	yes	L-type lectin-	pfam00139	Lectin_legB	Legume lect	GO:0009755|l	GO:0016021|	GO:0005524|A'	.
Manes.09G09540(	1.8165855	0.20332	up	no	Tropinone re	pfam00106	adh_short	short chain	GO:0009710|t	.	GO:0050356|tro	.
Manes.09G09560(	0.1651685	1.70E-25	down	yes	Ultraviolet-B	pfam00415	RCC1	Regulator o	GO:0009649|	GO:0000785|	GO:0003682|ch	.
Manes.09G09580(	3.6495146	3.31E-16	up	yes	.	pfam11175	DUF2961	Protein of u	.	.	.	.
Manes.09G09600(	3.9918975	0.01521	up	yes	Aldo-keto re	pfam00248	Aldo_ket_red	Aldo/keto re	GO:0055114|	GO:0009507|	GO:0008106|alc	.
Manes.09G09610(	0.7384477	0.00827	down	no	Characteriz	pfam02330	MAM33	Mitochondr	.	GO:0005759|	.	K15414
Manes.09G09650(	#NAME?	0.09758	down	no	.	pfam10271	Tmp39	Putative trai	.	.	.	.
Manes.09G09660(	3.0338068	6.67E-05	up	yes	.	pfam03542	Tuberin	Tuberin. Tu	.	.	.	.
Manes.09G09690(	1.070858	0.75499	up	no	Putative rece	pfam00069	Pkinase	Protein kina	GO:0048544|	GO:0016021|	GO:0005524|A'	.
Manes.09G09740(	23.585906	9.41E-77	up	yes	.	pfam02232	Alpha_TIF	Alpha trans-	.	.	.	.
Manes.09G09780(	5.3338397	1.37E-44	up	yes	Protein NRT	pfam00854	PTR2	POT family.	GO:0006857|	GO:0016021|	GO:0005215|tra	.
Manes.09G09810(	1.2049844	0.48558	up	no	Protein NRT	pfam00854	PTR2	POT family.	GO:0006857|	GO:0016021|	GO:0005215|tra	.
Manes.09G09840(	4.8522718	0.01337	up	yes	.	pfam09654	DUF2396	Protein of u	.	.	.	.
Manes.09G09850(	1.5079234	1.25E-05	up	no	.	pfam12273	RCR	Chitin synth	.	.	.	.
Manes.09G09900(	0.8504422	0.29636	down	no	Universal stre	pfam00582	Usp	Universal st	GO:0006950|	GO:0005886|	GO:0016208|Al	.
Manes.09G09910(	3.3750286	1.43E-41	up	yes	.	pfam03798	TRAM_LAG1_CI	TLC domain	.	.	.	.
Manes.09G09960(	0.5031699	2.03E-05	down	no	Phosphatidyli	pfam14360	PAP2_C	PAP2 super	GO:0030148|	GO:0016021|	GO:0045140|in	.
Manes.09G09980(	0.6062224	0.01301	down	no	.	pfam12906	RINGv	RING-varia	.	.	.	.
Manes.09G09990(	0.1952428	0.37125	down	no	.	pfam06521	PAR1	PAR1 prote	.	.	.	.
Manes.09G10000(	0.6350579	0.01174	down	no	Fructose-1,6-	pfam00316	FBPase	Fructose-1-(	GO:0019253|	GO:0009570|	GO:0042132|fru	.
Manes.09G10010(	0.3783031	1.33E-14	down	yes	Group II intr	pfam00078	RVT_1	Reverse tran	GO:0006314|i	.	GO:0004519|en	.
Manes.09G10020(	0.4302803	1.64E-06	down	yes	.	pfam05109	Herpes_BLLF1	Herpes virus	.	.	.	.
Manes.09G10050(	0.2788788	7.18E-06	down	yes	.	pfam14897	EpsG	EpsG family	.	.	.	.
Manes.09G10060(	0.5237228	7.13E-06	down	no	Heme-binding	pfam04832	SOUL	SOUL heme	.	GO:0009507|	.	.
Manes.09G10080(	2.5240804	0.00037	up	yes	Characteriz	pfam10250	O-FucT	GDP-fucose	.	GO:0005768|	GO:0016757|tra	.
Manes.09G10110(	Inf	2.93E-05	up	yes	.	pfam14264	Glucos_trans_II	Glucosyl tra	.	.	.	.
Manes.09G10140(	4.0361473	1.53E-07	up	yes	Reticulon-like	pfam02453	Reticulon	Reticulon. F	.	GO:0005789|	.	.
Manes.09G10150(	1.1038086	0.41635	up	no	Ataxin-3 hon	pfam02099	Josephin	Josephin.	GO:0006355|	GO:0005634|	GO:0008234|cy	K11863
Manes.09G10160(	0.9836344	0.88004	down	no	OTU domain	pfam02338	OTU	OTU-like cy	.	GO:0009507|	.	.
Manes.09G10180(	0.9616554	0.696	down	no	mRNA-cappi	pfam01331	mRNA_cap_enzyr	mRNA capp	GO:0006370|	GO:0005654|	GO:0005525|G'	K13917
Manes.09G10200(	0.5907729	2.17E-06	down	no	.	pfam06127	DUF962	Protein of u	.	.	.	.
Manes.09G10240(	0.5971438	1.94E-06	down	no	.	pfam00769	ERM	Ezrin/radixi	.	.	.	.
Manes.09G10250(	1.1752497	0.35406	up	no	Protein NRT	pfam00854	PTR2	POT family.	GO:0042938|	GO:0016021|	GO:0042936|dip	.
Manes.09G10270(	0.495106	1.64E-09	down	yes	Transcription	pfam00249	Myb_DNA-binding	Myb-like DI	GO:0048262|	GO:0005634|	GO:0003677|DI	.
Manes.09G10290(	0.8773181	0.18113	down	no	APO protein	pfam05634	APO_RNA-bind	APO RNA-l	.	GO:0009507|	GO:0003723|RI	.
Manes.09G10300(	2.8087505	8.72E-28	up	yes	GDP-mannos	pfam00534	Glycos_transf_1	Glycosyl tra	GO:0009247|	.	GO:0000030|ma	K12583
Manes.09G10330(	1.1682087	0.08967	up	no	26S proteaso	pfam13012	MitMem_reg	Maintenance	GO:0045087|	GO:0005829|	.	.
Manes.09G10380(	1.2254547	0.03771	up	no	Thylakoid AI	pfam00153	Mito_carr	Mitochondr	GO:0015867|	GO:0009507|	GO:0005347|A'	K14684
Manes.09G10410(	0.4935858	5.20E-05	down	yes	Probable LRI	pfam00069	Pkinase	Protein kina	.	GO:0016021|	GO:0005524|A'	.
Manes.09G10430(	1.1399378	0.15237	up	no	RNA polyme	pfam00035	dsrm	Double-stra	GO:0009734|	GO:0005737|	GO:0046872|m	.
Manes.09G10440(	0.4205249	0.01089	down	yes	.	pfam12292	DUF3624	Protein of u	.	.	.	.
Manes.09G10450(	0.7690622	0.01433	down	no	.	pfam00686	CBM_20	Starch bindi	.	.	.	.
Manes.09G10470(	3.5192091	4.01E-09	up	yes	GDSL estera	pfam13472	Lipase_GDSL_2	GDSL-like l	GO:0016042|	GO:0048046|	GO:0016788|hy	.
Manes.09G10480(	0.9043622	0.28707	down	no	LIMR family	pfam04791	LMBR1	LMBR1-like	.	GO:0016021|	.	K14617
Manes.09G10490(	Inf	0.9009	up	no	(+)-menthofu	pfam03635	Vps35	Vacuolar pr	GO:0016114|	GO:0016021|	GO:0052582|(+	.
Manes.09G10500(	1.1187158	0.44923	up	no	Putative E3 u	pfam13920	zf-C3HC4_3	Zinc finger,	.	GO:0005737|	GO:0016874|lig	.
Manes.09G10580(	Inf	8.74E-25	up	yes	Protein PHR	pfam14379	Myb_CC_LHEQL	MYB-CC ty	GO:0006351|	GO:0005634|	GO:0003677|DI	.
Manes.09G10600(	0.511039	6.94E-08	down	no	Calcium-depe	pfam00069	Pkinase	Protein kina	GO:0009738|	GO:0005737|	GO:0005524|A'	K13412
Manes.09G10610(	1.7098374	0.11835	up	no	Trihelix trans	pfam13837	Myb_DNA-bind_4	Myb/SANT-	GO:0006355|	GO:0005634|	GO:0042802|id	.
Manes.09G10650(	1.5557095	9.72E-07	up	no	Importin-11	pfam03810	IBN_N	Importin-be	GO:0006610|	GO:0005737|	GO:0008565|pr	.
Manes.09G10660(	1.5279303	2.24E-06	up	no	Ran-binding	pfam00638	Ran_BP1	RanBP1 dor	GO:0051028|	GO:0005643|	.	K15306
Manes.09G10670(	1.5532361	3.56E-06	up	no	Proliferation-	pfam00557	Peptidase_M24	Metallopept	GO:0007049|	GO:0005737|	GO:0003677|DI	.
Manes.09G10680(	2.3964665	0.14296	up	no	.	pfam00477	LEA_5	Small hydro	.	.	.	.
Manes.09G10700(	0.6159171	0.01226	down	no	.	pfam13965	SID-1_RNA_chan	dsRNA-gate	.	.	.	.
Manes.09G10750(	0.5998983	0.01189	down	no	PHD finger-li	pfam03660	PHF5	PHF5-like p	.	.	.	K12834
Manes.09G10790(	0.6136106	0.06587	down	no	30S ribosom	pfam05437	AzlD	Branched-cl	GO:0032544|	GO:0009507|	.	K19033
Manes.09G10840(	0.384403	2.84E-09	down	yes	.	pfam02232	Alpha_TIF	Alpha trans-	.	.	.	.
Manes.09G10850(	7.1812991	2.64E-09	up	yes	Receptor-like	pfam13855	LRR_8	Leucine rich	GO:0009755|	GO:0016021|	GO:0004672|pr	.
Manes.09G10880(	0.7698123	0.06342	down	no	BAG family r	pfam02179	BAG	BAG domai	GO:0009409|	GO:0005829|	.	.
Manes.09G10900(	1.5415644	0.00156	up	no	Ammonium t	pfam00909	Ammonium_transp	Ammonium	GO:0015696|	GO:0016021|	GO:0015398|hi	K03320
Manes.09G10950(	2.6000338	2.82E-07	up	yes	.	pfam05097	DUF688	Protein of u	.	.	.	.
Manes.09G10990(	1.2765664	0.00876	up	no	Ubiquitin-act	pfam09358	UBA_e1_C	Ubiquitin-ac	GO:0016567|	GO:0005829|	GO:0016881|ac	K03178
Manes.09G11010(	0.6434752	0.0219	down	no	.	pfam04310	MukB	MukB N-ter	.	.	.	.
Manes.09G11020(	NA	NA	--	no	Gamma-inter	pfam03227	GILT	Gamma inte	GO:0042590|	GO:0005576|	GO:0016667|ox	K08059
Manes.09G11080(	5.1285189	1.63E-61	up	yes	Abscisic acid	pfam10604	Polyketide_cyc2	Polyketide c	GO:0009738|	GO:0005737|	GO:0010427|ab	K14496
Manes.09G11180(	0.3533353	1.75E-12	down	yes	Zeaxanthin e	pfam00498	FHA	FHA domai	GO:0009688|	GO:0031969|	GO:0052663|an	.
Manes.09G11190(	0.7772807	0.03375	down	no	Integrator co	pfam02118	Srg	Srg family c	.	GO:0005634|	.	K13141
Manes.09G11200(	0.8302418	0.12471	down	no	Kelch-like pr	pfam10539	Dev_Cell_Death	Developmer	GO:0016567|	GO:0031463|	.	K10446
Manes.09G11230(	Inf	0.00737	up	yes	Cation/H(+) a	pfam00999	Na_H_Exchanger	Sodium/hyd	GO:0006813|	GO:0016021|	GO:0005451|m	.
Manes.09G11240(	21.411463	7.46E-09	up	yes	Probable prot	pfam00481	PP2C	Protein phos	GO:0006470|	.	GO:0046872|m	.
Manes.09G11250(	2.6023366	1.37E-27	up	yes	.	pfam05050	Methyltransf_21	Methyltrans	.	.	.	.
Manes.09G11270(	48.605497	0	up	yes	Probable WR	pfam03106	WRKY	WRKY DN.	GO:0010120|	GO:0005634|	GO:0043565|se	K13424
Manes.09G11300(	2.177574	0.00153	up	yes	Solute carrie	pfam06963	FPN1	Ferroportin1	GO:0010106|	GO:0016021|	GO:0015087|co	K14685
Manes.09G11310(	1.9622059	0.00493	up	no	Solute carrie	pfam06963	FPN1	Ferroportin1	GO:0006824|	GO:0016021|	GO:0005381|iro	K14685
Manes.09G11320(	1.4491032	0.00058	up	no	.	pfam08238	Sel1	Sel1 repeat.	.	.	.	.
Manes.09G11340(	1.3202219	0.03176	up	no	.	pfam14432	DYW_deaminase	DYW family	.	.	.	.
Manes.09G11360(	1.4055121	0.00017	up	no	Protein SCAI	pfam09528	Ehrlichia_rpt	Ehrlichia tar	GO:0030036|	GO:0005737|	.	.
Manes.09G11370(	11.257308	#######	up	yes	Short-chain d	pfam00106	adh_short	short chain	.	.	GO:0016491|ox	.
Manes.09G11390(	0.8803178	0.42952	down	no	D-alanine--D	pfam01820	Dala_Dala_lig_N	D-ala D-ala	GO:0071555|	GO:0005737|	GO:0005524|A'	K01921
Manes.09G11400(	0.2709614	1.54E-13	down	yes	.	pfam05734	DUF832	Herpesvirus	.	.	.	.
Manes.09G11410(	0.667665	0.02851	down	no	Pentatricope	pfam13041	PPR_2	PPR repeat	.	GO:0005739|	.	.
Manes.09G11420(	0.7902559	0.0132	down	no	Protein LUR	pfam04525	Tub_2	Tubby C 2.	.	GO:0009507|	.	.
Manes.09G11460(	0.6989823	0.00026	down	no	Regulatory-a	pfam14538	Raptor_N	Raptor N-te	GO:0016049|	GO:0080008|	.	K07204
Manes.09G11490(	1.8913548	3.28E-12	up	no	Alanine--glyc	pfam00202	Aminotran_3	Aminotransf	GO:0009853|	GO:0005739|	GO:0008453|ala	.
Manes.09G11510(	0.6550695	0.11401	down	no	G-type lectin	pfam01453	B_lectin	D-mannose	GO:0009755|	GO:0016021|	GO:0005524|A'	.
Manes.09G11570(	1.3740694	0.00059	up	no	Prosaposin	pfam05184	SapB_1	Saposin-like	GO:0007193|	GO:0070062|	GO:0008047|en	K12382

Manes.09G11590(	0.5969808	0.49048	down	no	.	pfam07420	DUF1509	Protein of u	.	.	.	.
Manes.09G11600(	0.8742912	0.15968	down	no	CBL-interact	pfam00069	Pkinase	Protein kina	GO:0007165|s	.	GO:0005524|A'	.
Manes.09G11610(	1.0382984	0.6741	up	no	.	pfam11600	CAF-1_p150	Chromatin a	.	.	.	.
Manes.09G11620(	0.5797552	2.02E-05	down	no	CBL-interact	pfam00069	Pkinase	Protein kina	GO:0007165|s	GO:0005634|	GO:0005524|A'	.
Manes.09G11640(	1.0041191	1	up	no	Transcription	pfam04844	Ovate	Transcriptio	GO:0006281|i	GO:0005856|	GO:0003677|DI	.
Manes.09G11650(	1.3186471	0.34653	up	no	.	pfam14226	DIOX_N	non-haem di	.	.	.	.
Manes.09G11670(	1.2630763	0.01051	up	no	.	pfam01909	NTP_transf_2	Nucleotidylt	.	.	.	.
Manes.09G11680(	0.9758802	0.81614	down	no	.	pfam13497	DUF4121	Domain of u	.	.	.	.
Manes.09G11700(	#NAME?	0.06822	down	no	MATE efflux	pfam01554	MatE	MatE. The I	.	GO:0016021|	GO:0015297|an	K03327
Manes.09G11710(	0.6410983	0.00021	down	no	Serine/threon	pfam07714	Pkinase_Tyr	Protein tyro	.	.	GO:0005524|A'	.
Manes.09G11720(	1.3326998	0.00173	up	no	MACPF dom	pfam01823	MACPF	MAC/Perfo	GO:0008219|c	.	.	.
Manes.09G11740(	8.4774021	3.41E-27	up	yes	RING-H2 fin	pfam13639	zf-RING_2	Ring finger	GO:0016567||	GO:0016021|	GO:0008270|zi	.
Manes.09G11760(	2.8866419	2.62E-29	up	yes	Probable rece	pfam07714	Pkinase_Tyr	Protein tyro	.	GO:0016021|	GO:0005524|A'	.
Manes.09G11770(	1.5388316	2.35E-05	up	no	B3 domain-co	pfam02362	B3	B3 DNA bir	GO:0006355|i	GO:0005634|	GO:0003677|DI	.
Manes.09G11820(	10.267666	0.02867	up	yes	.	pfam14111	DUF4283	Domain of u	.	.	.	.
Manes.09G11840(	1.1303054	0.1829	up	no	Vacuolar-sor	pfam02225	PA	PA domain.	GO:0006896|(	GO:0030665|	GO:0009940|an	.
Manes.09G11850(	0.9543841	0.65283	down	no	Transmembra	pfam06814	Lung_7-TM_R	Lung seven	.	GO:0016021|	.	.
Manes.09G11880(	0.6014658	3.15E-07	down	no	5'-3' exoribor	pfam03159	XRN_N	XRN 5'-3' e	GO:0010587|i	GO:0005634|	GO:0004534|5'-	K12619
Manes.09G11900(	0.524066	2.14E-07	down	no	SWR1 compl	pfam05764	YL1	YL1 nuclear	GO:0030154|c	GO:0005634|	.	K11664
Manes.09G11940(	0.3286621	1.30E-21	down	yes	Protein SCAI	pfam03514	GRAS	GRAS doma	GO:0007275|i	GO:0005634|	.	.
Manes.09G11950(	0.5303196	6.91E-06	down	no	Bifunctional	pfam02882	THF_DHG_CYH_	Tetrahydrof	GO:0009396|i	GO:0005739|	GO:0004477|m	.
Manes.09G11960(	0.7002365	0.00059	down	no	.	pfam15035	Rootletin	Ciliary rootl	.	.	.	.
Manes.09G11980(	5.7265387	0.00159	up	yes	.	pfam04525	Tub_2	Tubby C 2.	.	.	.	.
Manes.09G11990(	2.8112258	2.30E-10	up	yes	.	pfam04525	Tub_2	Tubby C 2.	.	.	.	.
Manes.09G12000(	0.917047	0.38582	down	no	2,3-bisphospl	pfam01676	Metalloenzyme	Metalloenzy	GO:0006007|g	GO:0005737|	GO:0030145|ma	.
Manes.09G12010(	1.3310848	0.09499	up	no	Serine/threon	pfam00149	Metallophos	Calcineurin-	.	.	GO:0046872|m	.
Manes.09G12040(	2.4123107	3.00E-05	up	yes	.	pfam03168	LEA_2	Late embryc	.	.	.	.
Manes.09G12050(	1.3424103	0.05488	up	no	.	pfam03168	LEA_2	Late embryc	.	.	.	.
Manes.09G12070(	3.8141217	3.06E-09	up	yes	Calcium-bind	pfam13499	EF-hand_7	EF-hand do	GO:0010091|t	.	GO:0005509|ca	K13448
Manes.09G12080(	0.2209726	0.00226	down	yes	.	pfam15177	IL28A	Interleukin-2	.	.	.	.
Manes.09G12090(	1.8128903	5.50E-05	up	no	Cytochrome	pfam00067	p450	Cytochrome	.	GO:0005789|	GO:0020037|he	.
Manes.09G12100(	0.7127798	0.00119	down	no	.	pfam03547	Mem_trans	Membrane t	.	.	.	.
Manes.09G12120(	0.1185224	0.00033	down	yes	Bromodomai	pfam00439	Bromodomain	Bromodoma	GO:0007596|l	GO:0005829|	GO:0008270|zi	K11350
Manes.09G12140(	1.6378103	5.71E-08	up	no	RING finger	pfam13639	zf-RING_2	Ring finger	.	GO:0016021|	GO:0008270|zi	.
Manes.09G12150(	0.9588585	0.76141	down	no	.	pfam06697	DUF1191	Protein of u	.	.	.	.
Manes.09G12170(	1.9978284	1.10E-11	up	no	KH domain-c	pfam00013	KH_1	KH domain.	.	GO:0005829|	GO:0003723|RI	K14945
Manes.09G12180(	3.9955557	0.00026	up	yes	.	pfam14111	DUF4283	Domain of u	.	.	.	.
Manes.09G12190(	0.7022677	0.0103	down	no	.	pfam11891	DUF3411	Domain of u	.	.	.	.
Manes.09G12200(	1.1862625	0.08255	up	no	Putative zinc	pfam02535	Zip	ZIP Zinc tra	GO:0006829|z	GO:0016021|	GO:0046873|m	.
Manes.09G12220(	0.4208565	3.17E-06	down	yes	BTB/POZ do	pfam03000	NPH3	NPH3 famil	GO:0016567||	.	.	.
Manes.09G12290(	1.9718997	1.97E-13	up	no	Receptor-like	pfam12819	Malectin_like	Carbohydra	GO:0009738|a	GO:0043680|	GO:0005524|A'	.
Manes.09G12310(	1.9251847	1.59E-12	up	no	Serine/threon	pfam00069	Pkinase	Protein kina	GO:0045727||	GO:0005634|	GO:0005524|A'	K04688
Manes.09G12340(	0.7592736	0.00626	down	no	Transcription	pfam07500	TFIIS_M	Transcriptio	GO:0006354|l	GO:0005813|	GO:0003677|DI	K03145
Manes.09G12360(	0.7627371	0.00707	down	no	Phospholipid	pfam00122	E1-E2_ATPase	E1-E2 ATP	GO:0006886|i	GO:0005783|	GO:0005524|A'	K14802
Manes.09G12390(	2.6736035	1.90E-18	up	yes	.	pfam13634	Nucleoporin_FG	Nucleoporin	.	.	.	.
Manes.09G12420(	0.5792993	5.94E-06	down	no	.	pfam12128	DUF3584	Protein of u	.	.	.	.
Manes.09G12440(	0.7788286	0.00904	down	no	Probable GT	pfam13328	HD_4	HD domain.	GO:0015969|g	GO:0009507|	GO:0005524|A'	.
Manes.09G12450(	1.9878158	5.35E-12	up	no	ADP-ribosyla	pfam00025	Arf	ADP-ribosy	GO:0015031|i	GO:0005794|	GO:0005525|G'	.
Manes.09G12460(	1.4918013	1.17E-05	up	no	Exportin-1	pfam08767	CRM1_C	CRM1 C ter	GO:0051028|i	GO:0005642|	GO:0005487|nu	K14290
Manes.09G12470(	1.3861423	0.00077	up	no	ERAD-assoc	pfam13639	zf-RING_2	Ring finger	GO:0030433|i	GO:0005789|	GO:0016874|lig	K10601
Manes.09G12500(	5.7291966	1.98E-06	up	yes	NAC domain	pfam02365	NAM	No apical m	GO:0006355|i	GO:0005634|	GO:0003677|DI	.
Manes.09G12530(	0.4177912	0.33277	down	no	Probable calc	pfam13499	EF-hand_7	EF-hand do	GO:0043269|i	GO:0000325|	GO:0005509|ca	K13448
Manes.09G12660(	1.6499699	2.66E-08	up	no	TOM1-like p	pfam00790	VHS	VHS domai	GO:0006886|i	GO:0070062|	GO:0030276|cl	.
Manes.09G12690(	Inf	0.00073	up	yes	.	pfam02469	Fasciclin	Fasciclin do	.	.	.	.
Manes.09G12740(	0.8399773	0.06626	down	no	.	pfam00069	Pkinase	Protein kina	.	.	.	.
Manes.09G12750(	1.5844385	1.79E-06	up	no	Histone acety	pfam00583	Acetyltransf_1	Acetyltransf	GO:0007059|c	.	GO:0004402|his	.
Manes.09G12760(	151.45748	1.07E-65	up	yes	Protein EXO	pfam04674	Phi_1	Phosphate-i	.	GO:0048046|	.	.
Manes.09G12790(	1.5345514	1.72E-06	up	no	Transducin b	pfam08625	Utp13	Utp13 speci	GO:0000462|i	GO:0005730|	GO:0044822|pc	K14555
Manes.09G12820(	3.670291	0.00053	up	yes	.	pfam05641	Agenet	Agenet dom	.	.	.	.
Manes.09G12830(	1.0598517	0.5811	up	no	.	pfam08378	NERD	Nuclease-re	.	.	.	.
Manes.09G12840(	0.8325065	0.0734	down	no	WEB family	pfam05701	WEMBL	Weak chlorc	.	GO:0009507|	.	.
Manes.09G12860(	1.4739132	1.46E-05	up	no	V-type proto	pfam03224	V-ATPase_H_N	V-ATPase s	GO:0015991|,	GO:0009507|	GO:0046961|pr	K02144
Manes.09G12870(	1.6070557	0.01408	up	no	Transcription	pfam11682	DUF3279	Protein of u	GO:0009567|c	GO:0005634|	GO:0003677|DI	.
Manes.09G12880(	0.7025278	0.0009	down	no	Uncharacteriz	pfam00294	PfkB	pfkB family	.	.	GO:0016301|ki	.
Manes.09G12890(	0.9430265	0.56143	down	no	.	pfam04640	PLATZ	PLATZ tran	.	.	.	.
Manes.09G12910(	0.4191057	2.75E-10	down	yes	Chaperone pr	pfam00226	DnaJ	DnaJ domai	GO:0006260|l	GO:0005737|	GO:0005524|A'	K03686
Manes.09G12940(	2.1931965	3.62E-17	up	yes	.	pfam12732	YtxH	YtxH-like p	.	.	.	.
Manes.09G12950(	0.9782923	0.83895	down	no	Bromodomai	pfam00628	PHD	PHD-finger.	GO:0006338|c	GO:0016590|	GO:0008270|zi	K11655
Manes.09G12960(	0.7261596	0.00414	down	no	F-box/kelch-r	pfam01344	Kelch_1	Kelch motif.	.	GO:0005634|	.	.
Manes.09G13000(	0.7014802	0.00383	down	no	.	pfam01576	Myosin_tail_1	Myosin tail.	.	.	.	.
Manes.09G13010(	2.1286934	3.08E-17	up	yes	.	pfam15249	GLTSCR1	Glioma tum	.	.	.	.
Manes.09G13020(	22.835985	3.06E-62	up	yes	Cytochrome	pfam00067	p450	Cytochrome	GO:0044550|s	GO:0016021|	GO:0020037|he	.
Manes.09G13060(	1.6205233	1.19E-06	up	no	Septin and tu	pfam07842	GCFC	GC-rich seq	GO:0000398|i	GO:0031981|	GO:0003677|DI	K13103
Manes.09G13070(	7.2941916	0.18853	up	no	.	pfam02005	TRM	N2,N2-dime	.	.	.	.
Manes.09G13100(	0.8367736	0.06484	down	no	Serine/threon	pfam00069	Pkinase	Protein kina	GO:0007165|s	GO:0051286|	GO:0005524|A'	K18670
Manes.09G13110(	0.571019	4.99E-06	down	no	Benzyl alcoh	pfam02458	Transferase	Transferase	.	.	GO:0016747|tra	.
Manes.09G13120(	2.477578	0.00023	up	yes	Benzyl alcoh	pfam02458	Transferase	Transferase	.	.	GO:0016747|tra	.
Manes.09G13180(	0.9594259	0.67597	down	no	.	pfam05384	DegS	Sensor prot	.	.	.	.
Manes.09G13220(	0.494246	0.3129	down	no	Probable glut	pfam13417	GST_N_3	Glutathione	GO:0009734|a	.	GO:0004364|gl	.
Manes.09G13230(	0.4723532	0.00042	down	yes	Probable glut	pfam13417	GST_N_3	Glutathione	.	.	GO:0004364|gl	K00799
Manes.09G13270(	3.8503746	0.39483	up	no	Aspartic prot	pfam14541	TAXi_C	Xylanase inl	GO:0042742|c	GO:0048046|	GO:0004190|as	.
Manes.09G13300(	0.3676435	9.92E-08	down	yes	.	pfam06219	DUF1005	Protein of u	.	.	.	.
Manes.09G13310(	0.6623954	0.0049	down	no	.	pfam06394	Pepsin-I3	Pepsin inhib	.	.	.	.
Manes.09G13330(	2.4026765	1.72E-23	up	yes	.	pfam14813	NADH_B2	NADH dehy	.	.	.	.
Manes.09G13380(	2.3246169	0.00062	up	yes	Dynamin-rela	pfam02212	GED	Dynamin G	.	GO:0005737|	GO:0005525|G'	.
Manes.09G13390(	7.5567807	4.99E-20	up	yes	Dynamin-rela	pfam00350	Dynamin_N	Dynamin far	.	GO:0005737|	GO:0005525|G'	.
Manes.09G13420(	0.4839181	1.29E-06	down	yes	CRAL-TRIO	pfam00650	CRAL_TRIO	CRAL/TRIC	GO:0006696|c	GO:0005886|	GO:0008526|ph	.
Manes.09G13440(	0.5096351	1.34E-08	down	no	.	pfam14817	HAUS5	HAUS augn	.	.	.	.

Manes.09G13450(	0.7775531	0.01387	down	no	Regulation o	pfam04818	CTD_bind	RNA polym	GO:0070940|	GO:0016591|	.	.
Manes.09G13460(	0.8195993	0.04057	down	no	.	pfam14264	Glucos_trans_II	Glucosyl tra	.	.	.	.
Manes.09G13470(	0.6958355	0.01127	down	no	ELMO doma	pfam04727	ELMO_CED12	ELMO/CEI	GO:0048870|	GO:0005737|	GO:0045159|m	.
Manes.09G13520(	6.5602699	2.48E-09	up	yes	Protein TRA	pfam01554	MatE	MatE. The	GO:0009813|	GO:0016021|	GO:0015238|dr	K03327
Manes.09G13530(	0.3787272	6.56E-17	down	yes	Protein TRA	pfam01554	MatE	MatE. The	GO:0009813|	GO:0016021|	GO:0015238|dr	K03327
Manes.09G13540(	1.1637616	0.09505	up	no	Protein GrpE	pfam01025	GrpE	GrpE.	GO:0006457|	GO:0005737|	GO:0000774|ad	K03687
Manes.09G13580(	13.176802	0.00125	up	yes	Probable ara	pfam03016	Exostosin	Exostosin fa	GO:0071555|	GO:0005768|	GO:0016757|tra	.
Manes.09G13610(	0.4534529	0.00182	down	yes	Transcription	pfam13921	Myb_DNA-bind_6	Myb-like D	GO:0009738|	GO:0005634|	GO:0003677|D	K09422
Manes.09G13630(	2.2464774	8.71E-17	up	yes	Cyclic dof fa	pfam02701	zf-Dof	Dof domain	GO:0009908|	GO:0005634|	GO:0003677|D	.
Manes.09G13640(	1.0831337	0.76263	up	no	.	pfam13595	DUF4138	Domain of u	.	.	.	.
Manes.09G13650(	2.2876541	8.79E-05	up	yes	Protein PNS1	pfam04515	Choline_transpo	Plasma-mem	GO:0006810|	GO:0016021|	.	.
Manes.09G13660(	1.2837568	0.35925	up	no	Cation/calciu	pfam01699	Na_Ca_ex	Sodium/calc	GO:0006813|	GO:0016021|	GO:0015297|an	.
Manes.09G13730(	3.0646303	3.53E-21	up	yes	Histone-lysin	pfam00856	SET	SET domain	.	GO:0005694|	GO:0018024|his	.
Manes.09G13740(	5.3722512	0.0073	up	yes	LOB domain	pfam03195	DUF260	Protein of u	GO:0009867|	.	.	.
Manes.09G13770(	2.5974927	2.35E-21	up	yes	Membrin-11	pfam12352	V-SNARE_C	Snare regio	GO:0006888|	GO:0005789|	GO:0005484|SN	.
Manes.09G13810(	1.4684276	0.01595	up	no	.	pfam03395	Pox_P4A	Poxvirus P4	.	.	.	.
Manes.09G13820(	1.1191674	0.29104	up	no	5-methyltetra	pfam01717	Meth_synt_2	Cobalamin-i	GO:0009086|	GO:0005737|	GO:0003871|5-	.
Manes.09G13910(	0.9530816	0.63896	down	no	Inhibitor of E	pfam12796	Ank_2	Ankyrin rep	.	GO:0005737|	.	.
Manes.09G13920(	0.329095	6.10E-09	down	yes	.	pfam12043	DUF3527	Domain of u	.	.	.	.
Manes.09G13960(	1.1587841	0.16226	up	no	Syntaxin-132	pfam05739	SNARE	SNARE dor	GO:0006886|	GO:0016021|	.	.
Manes.09G13990(	2.5614929	0.12414	up	no	Transcription	pfam00249	Myb_DNA-binding	Myb-like D	GO:0048354|	GO:0005634|	GO:0003677|D	K09422
Manes.09G14010(	14.772718	0.00132	up	yes	.	pfam01122	Cobalamin_bind	Eukaryotic	.	.	.	.
Manes.09G14030(	0.7280528	0.01523	down	no	Eukaryotic tr	pfam01652	IF4E	Eukaryotic i	GO:0006417|	GO:0005737|	GO:0003743|tra	.
Manes.09G14040(	1.8119328	5.02E-11	up	no	5'-adenylylsul	pfam00085	Thioredoxin	Thioredoxin	GO:0045454|	GO:0005623|	GO:0003756|pr	.
Manes.09G14050(	0.9582636	0.68245	down	no	.	pfam05704	Caps_synth	Capsular po	.	.	.	.
Manes.09G14060(	3.4661261	3.32E-05	up	yes	.	pfam13361	UvrD_C	UvrD-like h	.	.	.	.
Manes.09G14070(	1.8071035	2.19E-06	up	no	.	pfam06716	DUF1201	Protein of u	.	.	.	.
Manes.09G14090(	1.1836782	0.06837	up	no	.	pfam10615	DUF2470	Protein of u	.	.	.	.
Manes.09G14110(	0.6841937	0.00127	down	no	Adenylyl-sulf	pfam01583	APS_kinase	Adenylylsul	GO:0019344|	GO:0005829|	GO:0004020|ad	K00860
Manes.09G14130(	2.7498343	1.49E-11	up	yes	.	pfam12931	Sec16_C	Sec23-bindi	.	.	.	.
Manes.09G14150(	3.0358995	2.07E-07	up	yes	Glutamate de	pfam00208	ELFV_dehydrog	Glutamate/I	GO:0006520|	GO:0005739|	GO:0005524|A	K00261
Manes.09G14160(	0.7287207	0.0247	down	no	ADP-glucose	pfam01087	GalP_UDP_transf	Galactose-1	GO:0005975|	.	GO:0043531|A	K00965
Manes.09G14170(	2.8687581	7.38E-23	up	yes	YTH domain	pfam04146	YTH	YT521-B-li	GO:0043488|	GO:0000932|	GO:1990247|N	.
Manes.09G14270(	3.2684035	2.98E-13	up	yes	.	pfam01844	HNH	HNH endon	.	.	.	.
Manes.09G14290(	0.8337622	0.11734	down	no	Early nodulin	pfam03386	ENOD93	Early noduli	GO:0009877|	GO:0016021|	.	.
Manes.09G14320(	1.6842076	0.00204	up	no	Putative dise	pfam00931	NB-ARC	NB-ARC do	GO:0006952|	.	GO:0043531|A	.
Manes.09G14340(	0.847404	0.19952	down	no	.	pfam14634	zf-RING_5	zinc-RING	.	.	.	.
Manes.09G14430(	1.0019073	1	up	no	Apyrase 2	pfam01150	GDA1_CD39	GDA1/CD3	.	GO:0000139|	GO:0005524|A	.
Manes.09G14460(	0.6429746	1.38E-05	down	no	Violaxanthin	pfam07137	VDE	Violaxanthi	.	GO:0009535|	GO:0046422|vi	.
Manes.09G14470(	1.1516944	0.1728	up	no	.	pfam08317	Spc7	Spc7 kineto	.	.	.	.
Manes.09G14490(	0.2899187	2.22E-08	down	yes	.	pfam14093	DUF4271	Domain of u	.	.	.	.
Manes.09G14500(	0.4836904	0.00663	down	yes	U-box domai	pfam04564	U-box	U-box dom	GO:0009738|	GO:0005737|	GO:0016874|lig	.
Manes.09G14510(	0.8008224	0.02481	down	no	U11/U12 sm	pfam05253	zf-U11-48K	U11-48K-li	GO:0006397|	GO:0005681|	GO:0046872|m	K13156
Manes.09G14540(	1.2881577	0.00613	up	no	Probable UD	pfam13844	Glyco_transf_41	Glycosyl tra	GO:0007275|	.	GO:0016757|tra	K09667
Manes.09G14560(	1.0090534	0.88851	up	no	.	pfam02037	SAP	SAP domain	.	.	.	.
Manes.09G14580(	0.2286211	2.28E-16	down	yes	GDSL estera	pfam00657	Lipase_GDSL	GDSL-like	GO:0016042|	GO:0005576|	GO:0016788|hy	.
Manes.09G14610(	NA	NA	--	no	Dehydration-	pfam00847	AP2	AP2 domain	GO:0006351|	GO:0005634|	GO:0003677|D	.
Manes.09G14650(	1.1279605	0.22554	up	no	DNA-directe	pfam03874	RNA_pol_Rpb4	RNA polym	GO:0031990|	GO:0000932|	GO:0003899|D	K03012
Manes.09G14660(	0.9047668	0.30514	down	no	.	pfam05208	ALG3	ALG3 prote	.	.	.	.
Manes.09G14760(	0.8724079	0.17651	down	no	Ankyrin repe	pfam11904	GPCR_chapero_1	GPCR-chap	.	GO:0005769|	.	.
Manes.09G14780(	0.727299	0.02995	down	no	Casein kinase	pfam00069	Pkinase	Protein kina	GO:0006897|	GO:0005737|	GO:0005524|A	K02218
Manes.09G14790(	1.0062796	0.93127	up	no	Exportin-4	pfam09531	Ndc1_Nup	Nucleoporin	GO:0015031|	GO:0005737|	.	.
Manes.09G14820(	1.7521174	0.02188	up	no	Protein LIGH	pfam04852	DUF640	Protein of u	GO:0009299|	GO:0005634|	GO:0003677|D	.
Manes.09G14830(	2.8869917	0.0002	up	yes	Probable low	pfam01212	Beta_elim_lyase	Beta-elimina	GO:0006545|	GO:0005829|	GO:0008732|L-	K01620
Manes.09G14860(	2.1637451	0.01374	up	yes	Phosphoenol	pfam00069	Pkinase	Protein kina	GO:0009738|	GO:0005737|	GO:0005524|A	.
Manes.09G14870(	0.7186602	0.00366	down	no	Serine/threon	pfam00149	Metallophos	Calcineurin-	.	.	GO:0046872|m	K06269
Manes.09G14890(	1.3991586	0.59781	up	no	.	pfam02714	DUF221	Domain of u	.	.	.	.
Manes.09G14900(	6.008417	0.09585	up	no	Ethylene-resp	pfam00847	AP2	AP2 domain	GO:0006952|	GO:0005634|	GO:0003677|D	K14516
Manes.09G14960(	0.665974	0.01448	down	no	Methyltransf	pfam13489	Methyltransf_23	Methyltrans	.	.	GO:0008168|m	K00599
Manes.09G14980(	1.4814343	0.0017	up	no	.	pfam03479	DUF296	Domain of u	.	.	.	.
Manes.09G15160(	0.60376	0.00039	down	no	Transcription	pfam00400	WD40	WD domain	GO:0030154|	GO:0005634|	.	.
Manes.09G15170(	0.3843792	2.47E-07	down	yes	PRA1 family	pfam03208	PRA1	PRA1 famil	GO:0016192|	GO:0005783|	.	.
Manes.09G15180(	0.6765592	0.00453	down	no	Probable pre	pfam01920	Prefoldin_2	Prefoldin su	GO:0006457|	GO:0005829|	.	K09550
Manes.09G15190(	1.036721	0.76431	up	no	E3 ubiquitin	pfam13923	zf-C3HC4_2	Zinc finger,	GO:0051865|	.	GO:0016874|lig	K16277
Manes.09G15200(	Inf	0.08944	up	no	.	pfam06728	PIG-U	GPI transam	.	.	.	.
Manes.09G15210(	0.7136061	0.00369	down	no	Photosystem	pfam07123	PsbW	Photosysten	GO:0015979|	GO:0009535|	.	.
Manes.09G15240(	Inf	0.35135	up	no	.	pfam03361	Herpes_IE2_3	Herpes virus	.	.	.	.
Manes.09G15270(	2.3524136	9.13E-21	up	yes	Auxin-induce	pfam02309	AUX_IAA	AUX/IAA f	GO:0009734|	GO:0005634|	.	.
Manes.09G15280(	1.6996039	7.55E-09	up	no	Mediator of I	pfam08638	Med14	Mediator co	GO:0009631|	GO:0016592|	GO:0001104|R	K15156
Manes.09G15290(	0.5306537	0.00141	down	no	Pentatricope	pfam13041	PPR_2	PPR repeat	.	GO:0005739|	.	.
Manes.09G15320(	0.1060377	9.50E-09	down	yes	Pentatricope	pfam13041	PPR_2	PPR repeat	GO:0080156|	GO:0005739|	.	.
Manes.09G15370(	15.311443	5.78E-09	up	yes	Calcium-tran	pfam00689	Cation_ATPase_C	Cation trans	.	GO:0016021|	GO:0005524|A	K01537
Manes.09G15410(	8.7980822	1.17E-06	up	yes	.	pfam06247	Plasmod_Pvs28	Plasmodium	.	.	.	.
Manes.09G15420(	1.9298308	2.18E-09	up	no	Mitochondria	pfam02466	Tim17	Tim17/Tim2	GO:0006886|	GO:0016021|	GO:0015450|P-	K17794
Manes.09G15430(	1.7120264	8.78E-09	up	no	Trihelix trans	pfam13837	Myb_DNA-bind_4	Myb/SANT	GO:0045893|	GO:0005634|	GO:0005516|ca	.
Manes.09G15440(	6.524498	0.15093	up	no	.	pfam13863	DUF4200	Domain of u	.	.	.	.
Manes.09G15460(	1.5975682	7.53E-07	up	no	Multisubstrat	pfam01142	TruD	tRNA pseud	GO:0000455|	GO:0005737|	GO:0009982|ps	K06176
Manes.09G15510(	Inf	0.04608	up	no	Calcium-tran	pfam00122	E1-E2_ATPase	E1-E2 ATP	.	GO:0016021|	GO:0005524|A	K01537
Manes.09G15520(	1.6692266	4.94E-08	up	no	.	pfam03096	Ndr	Ndr family.	.	.	.	.
Manes.09G15600(	0.6830337	0.01314	down	no	.	pfam10184	DUF2358	Uncharacter	.	.	.	.
Manes.09G15630(	0.7903288	0.0156	down	no	Serine/threon	pfam00069	Pkinase	Protein kina	GO:0007623|	GO:0016020|	GO:0005524|A	.
Manes.09G15650(	1.1863556	0.0678	up	no	40S ribosom	pfam01282	Ribosomal_S24e	Ribosomal	GO:0006412|	GO:0005618|	GO:0000166|nu	K02974
Manes.09G15670(	3.7786227	6.83E-47	up	yes	Guanine dea	pfam00383	dCMP_cyt_deam_	Cytidine and	GO:0006147|	.	GO:0008892|gu	K01487
Manes.09G15680(	1.505873	6.19E-06	up	no	Protein trans	pfam04811	Sec23_trunk	Sec23/Sec2	GO:0006888|	GO:0030127|	GO:0008270|zi	K14007
Manes.09G15690(	1.9311901	1.82E-13	up	no	Mediator-ass	pfam04504	DUF573	Protein of u	GO:0006355|	GO:0009507|	.	.
Manes.09G15700(	0.7983899	0.06275	down	no	Rubredoxin	pfam00301	Rubredoxin	Rubredoxin.	GO:0055114|	.	GO:0005506|irc	.
Manes.09G15710(	0.916137	0.3798	down	no	Cysteine synt	pfam00291	PALP	Pyridoxal-p	GO:0006535|	GO:0005737|	GO:0047458|be	.
Manes.09G15730(	Inf	0.00013	up	yes	Cysteine synt	pfam00291	PALP	Pyridoxal-p	GO:0006535|	GO:0005737|	GO:0047458|be	.

Manes.09G15750(	14.079535	8.86E-05	up	yes	.	pfam04927	SMP	Seed matura	.	.	.	.
Manes.09G15770(	2.2846699	0.00175	up	yes	Putative defe	pfam07333	SLR1-BP	S locus-relat	GO:0050832|	GO:0005576|	.	.
Manes.09G15790(	0.9711192	0.81209	down	no	.	pfam08242	Methyltransf_12	Methyltrans	.	.	.	.
Manes.09G15800(	0.7866086	0.015	down	no	Kinesin-like	pfam00225	Kinesin	Kinesin mot	GO:0007018|	GO:0005871|	GO:0043531|Al	.
Manes.09G15810(	0.8460482	0.08912	down	no	WD repeat-c	pfam11816	DUF3337	Domain of u	GO:0000724|	GO:0005764|	.	K15361
Manes.09G15820(	1.1416772	0.15539	up	no	Acyltransfera	pfam12697	Abhydrolase_6	Alpha/beta l	GO:0033306|	GO:0009507|	GO:0004144|di	.
Manes.09G15840(	0.4553277	3.49E-08	down	yes	Acyltransfera	pfam12697	Abhydrolase_6	Alpha/beta l	GO:0033306|	GO:0009507|	GO:0004144|di	.
Manes.09G15850(	0.4385589	5.16E-10	down	yes	TMV resistar	pfam00931	NB-ARC	NB-ARC dc	GO:0009626|	GO:0005737|	GO:0043531|Al	.
Manes.09G15870(	0.7181037	0.02495	down	no	.	pfam13041	PPR_2	PPR repeat	.	.	.	.
Manes.09G15880(	2.1349005	4.98E-15	up	yes	Double-stran	pfam00035	dsrm	Double-stra	.	.	GO:0003725|dc	.
Manes.09G15890(	1.4310657	5.98E-05	up	no	Probable nuc	pfam01798	Nop	Putative sno	GO:0042254|	GO:0016020|	.	K14565
Manes.09G15900(	1.2414196	0.03941	up	no	Probable seri	pfam00069	Pkinase	Protein kina	.	.	GO:0005524|A	.
Manes.09G15930(	Inf	0.00041	up	yes	.	pfam02438	Adeno_100	Late 100kD	.	.	.	.
Manes.09G16040(	0.3713168	2.97E-11	down	yes	Superoxide d	pfam00080	Sod_Cu	Copper/zinc	.	GO:0005737|	GO:0046872|m	.
Manes.09G16050(	1.0129276	0.90929	up	no	Trafficking p	pfam04051	TRAPP	Transport p	GO:0016192|	GO:0005783|	.	.
Manes.09G16080(	0.7151906	0.00167	down	no	FRIGIDA-lik	pfam07899	Frigida	Frigida-like	GO:0030154|	.	.	.
Manes.09G16090(	0.7483165	0.18073	down	no	Bifunctional	pfam01487	DHquinase_I	Type I 3-del	GO:0009073|	GO:0009507|	GO:0003855|3-	K13832
Manes.09G16160(	0.5831242	8.53E-07	down	no	Gibberellin re	pfam07859	Abhydrolase_3	alpha/beta h	GO:0048444|	GO:0005634|	GO:0016787|hy	K14493
Manes.09G16190(	1.5758304	3.63E-06	up	no	Autophagy-re	pfam12490	BCAS3	Breast carci	GO:0006914|	GO:0005737|	.	.
Manes.09G16250(	0.519546	3.20E-07	down	no	Sugar transp	pfam00083	Sugar_tr	Sugar (and	GO:0008643|	GO:0016021|	GO:0022891|su	.
Manes.09G16270(	0.5756525	0.00025	down	no	Piezo-type m	pfam12166	DUF3595	Protein of u	.	GO:0016021|	GO:0008381|m	.
Manes.09G16290(	0.9113929	0.35127	down	no	Glutathione s	pfam03917	GSH_synth_ATP	Eukaryotic	.	GO:0009507|	GO:0005524|A	.
Manes.09G16320(	0.3857728	0.00027	down	yes	Probable aral	pfam03016	Exostosin	Exostosin fa	GO:0071555|	GO:0005768|	GO:0016757|tra	.
Manes.09G16330(	2.1510311	6.79E-07	up	yes	Sodium/hydr	pfam00999	Na_H_Exchanger	Sodium/hyd	GO:0055075|	GO:0016021|	GO:0015386|pc	.
Manes.09G16340(	0.6050016	1.78E-05	down	no	.	pfam01789	PsbP	PsbP. This f	.	.	.	.
Manes.09G16350(	1.2005809	0.04978	up	no	UPF0603 prc	pfam04536	TPM	TLP18.3, Ps	GO:0016311|	GO:0009507|	GO:0003993|ac	.
Manes.09G16410(	0.33994	1.54E-06	down	yes	Transcription	pfam00010	HLH	Helix-loop-l	GO:0006351|	GO:0005634|	GO:0016597|an	.
Manes.09G16420(	5.8290751	5.82E-17	up	yes	E3 ubiquitin-	pfam13639	zf-RING_2	Ring finger	GO:0009816|	GO:0016021|	GO:0016874|lig	K10664
Manes.09G16430(	0.8096936	0.07839	down	no	DNA excisio	pfam03834	Rad10	Binding don	GO:0000724|	GO:0005634|	GO:0017108|5'-	K10849
Manes.09G16440(	0.591294	0.00012	down	no	Serine/threon	pfam07714	Pkinase_Tyr	Protein tyro	GO:0009742|	GO:0005886|	GO:0005524|A	.
Manes.09G16450(	0.6692257	0.00024	down	no	.	pfam00403	HMA	Heavy-meta	.	.	.	.
Manes.09G16480(	0.6215268	0.00244	down	no	Signal peptid	pfam04573	SPC22	Signal pepti	GO:0006465|	GO:0005618|	GO:0008233|pe	K12948
Manes.09G16490(	2.455641	5.72E-11	up	yes	Cation/calciu	pfam01699	Na_Ca_ex	Sodium/calc	GO:0010163|	GO:0016021|	GO:0015297|an	.
Manes.09G16530(	0.4992123	0.00217	down	yes	.	pfam03154	Atrophin-1	Atrophin-1	.	.	.	.
Manes.09G16560(	0.5276233	1.78E-08	down	no	Amidase 1	pfam01425	Amidase	Amidase.	GO:0009684|	GO:0005737|	GO:0004040|an	.
Manes.09G16570(	1.1828113	0.11718	up	no	Mevalonate k	pfam08544	GHMP_kinases_C	GHMP kina	GO:0019287|	GO:0005829|	GO:0005524|A	K00869
Manes.09G16580(	Inf	0.01236	up	yes	Glucose and	pfam13561	adh_short_C2	Enoyl-(Acyl	.	.	GO:0016491|ox	.
Manes.09G16650(	2.0393719	1.98E-08	up	yes	Peroxisomal	pfam00153	Mito_carr	Mitochondr	GO:0015866|	GO:0016021|	GO:0015217|Al	.
Manes.09G16660(	1.0078114	0.90563	up	no	Mitochondria	pfam08356	EF_assoc_2	EF hand ass	GO:0007005|	GO:0031307|	GO:0005509|ca	K07870
Manes.09G16670(	7.8507146	0.08808	up	no	Putative UDI	pfam01501	Glyco_transf_8	Glycosyl tra	GO:0071555|	GO:0005794|	GO:0015020|gl	.
Manes.09G16720(	0.5745105	0.00019	down	no	Chaperone p	pfam00226	DnaJ	DnaJ domai	GO:0006260|	GO:0005737|	GO:0005524|A	K03686
Manes.09G16730(	1.4177891	0.07458	up	no	.	pfam13041	PPR_2	PPR repeat	.	.	.	.
Manes.09G16740(	0.864221	0.2042	down	no	Long chain a	pfam00501	AMP-binding	AMP-bindin	GO:0006631|	GO:0005777|	GO:0005524|A	K01897
Manes.09G16770(	0.7462808	0.01317	down	no	Probable lino	pfam00305	Lipoxygenase	Lipoxygena	GO:0031408|	GO:0005737|	GO:0046872|m	K15718
Manes.09G16790(	1.6171664	2.76E-07	up	no	Zinc finger p	pfam00641	zf-RanBP	Zn-finger in	GO:1900871|	GO:0009507|	GO:0008270|zi	.
Manes.09G16910(	1.1942124	0.05259	up	no	Acyl-CoA-bi	pfam00887	ACBP	Acyl CoA b	GO:0006869|	GO:0005737|	GO:0000062|fa	.
Manes.09G16930(	2.1597528	1.25E-13	up	yes	Autophagy-re	pfam15548	DUF4655	Domain of u	GO:0048102|	GO:0005829|	GO:0080025|ph	.
Manes.09G16960(	0.1929874	3.47E-23	down	yes	Formin-like p	pfam02181	FH2	Formin Hon	.	GO:0016021|	GO:0003779|ac	.
Manes.09G16970(	0.4779379	0.00023	down	yes	Copper trans	pfam00403	HMA	Heavy-meta	GO:0006878|	GO:0048046|	GO:0016531|co	K07213
Manes.09G16990(	2.3766786	3.93E-06	up	yes	Protein RAL	pfam05498	RALF	Rapid ALka	GO:0019722|	GO:0048046|	GO:0005179|ho	.
Manes.09G17000(	0.7064827	0.01445	down	no	.	pfam05755	REF	Rubber elon	.	.	.	.
Manes.09G17050(	0.4474291	0.00105	down	yes	.	pfam04235	DUF418	Protein of u	.	.	.	.
Manes.09G17070(	1.1009534	0.31801	up	no	26S protease	pfam00004	AAA	ATPase fam	GO:0009553|	GO:0005737|	GO:0005524|A	K03065
Manes.09G17100(	0.6873522	0.00029	down	no	Serine/argini	pfam00076	RRM_1	RNA recogi	GO:0006376|	GO:0005737|	GO:0003729|m	K12890
Manes.09G17120(	Inf	4.96E-95	up	yes	3-ketoacyl-C	pfam08392	FAE1_CUT1_Rpp	FAE1/Type	GO:0006633|	GO:0016021|	GO:0016747|tra	K15397
Manes.09G17130(	2.4436992	7.96E-07	up	yes	50S ribosom	pfam00861	Ribosomal_L18p	Ribosomal I	GO:0006412|	GO:0005840|	GO:0019843|rR	.
Manes.09G17150(	15.456289	2.96E-16	up	yes	.	pfam03024	Folate_rec	Folate recep	.	.	.	.
Manes.09G17160(	3.9675494	3.05E-50	up	yes	Serine/threon	pfam00149	Metallophos	Calcineurin-	GO:0016311|	.	GO:0046872|m	K06269
Manes.09G17200(	0.6701723	0.18434	down	no	Bidirectional	pfam03083	MtN3_slv	Sugar efflux	GO:0034219|	GO:0005887|	GO:0051119|su	K15382
Manes.09G17250(	2.837857	0.00057	up	yes	Respiratory b	pfam08030	NAD_binding_6	Ferric reduc	.	GO:0016021|	GO:0005509|ca	K13447
Manes.09G17330(	0.7234665	0.32722	down	no	Nudix hydrol	pfam00293	NUDIX	NUDIX dor	.	GO:0005739|	GO:0034432|bi	.
Manes.09G17350(	Inf	0.9009	up	no	Putative inve	pfam04043	PMEI	Plant inverta	.	GO:0005576|	GO:0004857|en	.
Manes.09G17370(	1.4717665	0.00587	up	no	F-box/LRR-r	pfam15449	Retinal	Retinal prot	.	.	.	K10268
Manes.09G17400(	0.4676928	2.85E-06	down	yes	Probable pro	pfam00481	PP2C	Protein phos	GO:0006470|	.	GO:0046872|m	.
Manes.09G17430(	1.0245959	0.76181	up	no	Eukaryotic tr	pfam13868	Trichoplein	tumor suppr	GO:0001731|	GO:0005829|	GO:0003743|tra	K03254
Manes.09G17460(	0.9646144	0.73522	down	no	Protein DEH	pfam14571	Di19_C	Stress-induc	GO:0045893|	GO:0005634|	GO:0003677|D	.
Manes.09G17480(	0.5559997	0.00036	down	no	Exonuclease	pfam00929	RNase_T	Exonuclease	GO:0000738|	GO:0009507|	GO:0008296|3'-	.
Manes.09G17490(	3.7430296	6.71E-25	up	yes	GATA transc	pfam00320	GATA	GATA zinc	GO:0006351|	GO:0005634|	GO:0043565|se	.
Manes.09G17510(	0.5357577	6.57E-05	down	no	Exopolyphos	pfam02541	Ppx-GppA	Ppx/GppA	GO:0071978|	.	GO:0004309|ex	K01524
Manes.09G17520(	3.5649427	0.07683	up	no	.	pfam12023	DUF3511	Domain of u	.	.	.	.
Manes.09G17530(	1.308373	0.12786	up	no	ATP-depende	pfam00270	DEAD	DEAD/DEA	GO:0070417|	GO:0005634|	GO:0005524|A	K10901
Manes.09G17550(	0.5427687	2.16E-08	down	no	.	pfam07816	DUF1645	Protein of u	.	.	.	.
Manes.09G17570(	1.0981025	0.37246	up	no	Zinc finger m	pfam12874	zf-met	Zinc-finger	.	GO:0005634|	GO:0003677|D	K12848
Manes.09G17580(	8.7384676	3.53E-21	up	yes	.	pfam02537	CRCB	CrcB-like p	.	.	.	.
Manes.09G17590(	Inf	1.50E-05	up	yes	.	pfam11588	DUF3243	Protein of u	.	.	.	.
Manes.09G17600(	48.256586	0.00431	up	yes	.	pfam03694	Erg28	Erg28 like p	.	.	.	.
Manes.09G17640(	0.6360814	0.00329	down	no	RNA pseudo	pfam00849	PseudoU_synth_2	RNA pseudo	GO:0006457|	.	GO:0009982|ps	.
Manes.09G17660(	Inf	0.04209	up	no	Lon protease	pfam05362	Lon_C	Lon proteas	GO:0034599|	GO:0005759|	GO:0005524|A	K08675
Manes.09G17670(	0.6917591	0.02351	down	no	.	pfam11991	Trp_DMAT	Tryptophan	.	.	.	.
Manes.09G17710(	0.5434485	1.03E-08	down	no	Pentatricope	pfam14432	DYW_deaminase	DYW family	GO:0009451|	GO:0009507|	.	.
Manes.09G17730(	NA	NA	--	no	Probable pec	pfam01095	Pectinesterase	Pectinestera	GO:0042545|	GO:0005618|	GO:0045330|as	.
Manes.09G17740(	0.6188713	0.01105	down	no	Rab9 effecto	pfam13418	Kelch_4	Galactose o	.	GO:0010008|	.	.
Manes.09G17760(	0.8408642	0.15009	down	no	Transcription	pfam03342	Rhabdo_M1	Rhabdoviru	GO:0006351|	GO:0005634|	GO:0003677|D	.
Manes.09G17800(	0.3098746	5.10E-05	down	yes	.	pfam00002	7tm_2	7 transmem	.	.	.	.
Manes.09G17870(	1.7671619	7.24E-10	up	no	Alkaline/neut	pfam12899	Glyco_hydro_100	Alkaline anc	GO:0048364|	GO:0005829|	GO:0004564|be	.
Manes.09G17880(	0.9370886	0.67664	down	no	AP-3 comple	pfam00928	Adap_comp_sub	Adaptor cor	GO:0009630|	GO:0030131|	.	K12398
Manes.09G17890(	0.4664928	0.13058	down	no	Golgi appara	pfam05832	DUF846	Eukaryotic	GO:0007030|	GO:0031901|	.	.
Manes.09G17910(	3.171182	2.49E-13	up	yes	Galactinol sy	pfam01501	Glyco_transf_8	Glycosyl tra	GO:0006012|	GO:0005737|	GO:0047216|in	K18819

Manes.09G17930(	#NAME?	0.29342	down	no	WAT1-relate	pfam00892	EamA	EamA-like t	.	GO:0016021|	GO:0022857|tra	.
Manes.09G17940(	0.6874494	0.0163	down	no	GDSL estera	pfam00657	Lipase_GDSL	GDSL-like l	GO:0016042|l	GO:0009570|	GO:0016788|hy	.
Manes.09G17950(	0.5169408	0.00186	down	no	.	pfam10332	DUF2418	Protein of u	.	.	.	.
Manes.09G17970(	0.6023911	1.69E-06	down	no	Glucose-6-pl	pfam02781	G6PD_C	Glucose-6-p	GO:0006006|	GO:0009507|	GO:0004345|gl	.
Manes.09G17980(	1.1129559	0.24657	up	no	Pyrrolidone-c	pfam01470	Peptidase_C15	Pyroglutamy	.	GO:0005829|	GO:0008234|cy	K01304
Manes.09G18000(	0.9064058	0.30951	down	no	Shaggy-relate	pfam00069	Pkinase	Protein kina	.	.	GO:0005524|A'	.
Manes.09G18010(	0.9232221	0.40955	down	no	ATP-citrate s	pfam08442	ATP-grasp_2	ATP-grasp c	GO:0006629|	GO:0005829|	GO:0005524|A'	K01648
Manes.09G18020(	0.8111531	0.06527	down	no	E3 ubiquitin-	pfam13920	zf-C3HC4_3	Zinc finger,	.	GO:0005737|	GO:0016874|lig	.
Manes.09G18030(	0.3842197	1.60E-14	down	yes	Probable rece	pfam00069	Pkinase	Protein kina	.	GO:0016021|	GO:0005524|A'	.
Manes.09G18060(	0.3615017	1.28E-13	down	yes	.	pfam01576	Myosin_tail_1	Myosin tail.	.	.	.	.
Manes.09G18070(	1.0419619	0.6424	up	no	Meiosis arres	pfam01936	NYN	NYN domai	GO:0051321|	GO:0005777|	GO:0000166|nu	.
Manes.09G18080(	0.66414	0.0155	down	no	Pentatricopep	pfam13041	PPR_2	PPR repeat	GO:0009793|	GO:0009507|	.	.
Manes.09G18120(	0.8395163	0.4773	down	no	Aspartic prot	pfam00026	Asp	Eukaryotic a	GO:0006629|	GO:0005773|	GO:0004190|as	K08245
Manes.09G18160(	0.559875	0.01476	down	no	Anaphase-pro	pfam12861	zf-Apc11	Anaphase-p	GO:0051301|	GO:0005737|	GO:0008270|zi	K03358
Manes.09G18170(	1.7785886	1.30E-05	up	no	Hydrophobic	pfam01679	Pmp3	Proteolipid	.	GO:0016021|	.	.
Manes.09G18180(	1.6012961	1.59E-07	up	no	Transcription	pfam00010	HLH	Helix-loop-l	GO:0009704|	GO:0005634|	GO:0003677|D	K12126
Manes.09G18210(	0.7782851	0.01235	down	no	.	pfam03283	PAE	Pectinacetyl	.	.	.	.
Manes.09G18340(	0.4841747	1.33E-08	down	yes	.	pfam00628	PHD	PHD-finger.	.	.	.	.
Manes.09G18380(	1.054798	0.57	up	no	.	pfam13657	Couple_hipA	HipA N-term	.	.	.	.
Manes.09G18410(	1.8120672	7.77E-07	up	no	ALBINO3-lil	pfam02096	60KD_IMP	60Kd inner	GO:0051205|	GO:0016021|	.	.
Manes.09G18430(	0.8652977	0.24262	down	no	.	pfam01424	R3H	R3H domain	.	.	.	.
Manes.09G18450(	0.3394592	8.38E-12	down	yes	CBS domain-	pfam15449	Retinal	Retinal prot	.	GO:0016021|	.	.
Manes.09G18470(	0.2801933	1.17E-23	down	yes	.	pfam05212	DUF707	Protein of u	.	.	.	.
Manes.09G18480(	14.625602	1.62E-06	up	yes	.	pfam04348	LppC	LppC putati	.	.	.	.
Manes.09G18490(	1.2528101	0.06957	up	no	.	pfam06682	DUF1183	Protein of u	.	.	.	.
Manes.09G18520(	0.6105479	1.18E-05	down	no	Zinc finger C	pfam00642	zf-CCCH	Zinc finger C	.	GO:0005634|	GO:0003677|D	.
Manes.09G18530(	0.2269534	8.79E-38	down	yes	NAC transcri	pfam02365	NAM	No apical m	GO:0009908|	GO:0005634|	GO:0003677|D	.
Manes.09G18540(	0.9272216	0.46189	down	no	Pyruvate deh	pfam02779	Transket_pyr	Transketola	GO:0006086|	GO:0048046|	GO:0004739|py	K00162
Manes.09G18560(	0.5390241	4.43E-06	down	no	Somatic emb	pfam08263	LRRNT_2	Leucine rich	GO:0009742|	GO:0005789|	GO:0005524|A'	K13418
Manes.09G18580(	6.5907536	6.52E-39	up	yes	.	pfam10601	zf-LITAF-like	LITAF-like	.	.	.	.
Manes.09G18590(	1.9888152	9.26E-08	up	no	.	pfam07295	DUF1451	Protein of u	.	.	.	.
Manes.09G18600(	143.93649	3.90E-05	up	yes	.	pfam11443	DUF2828	Domain of u	.	.	.	.
Manes.09G18610(	7.2192796	#######	up	yes	.	pfam11443	DUF2828	Domain of u	.	.	.	.
Manes.09G18630(	0.7920885	0.4538	down	no	E3 ubiquitin	pfam11816	DUF3337	Domain of u	GO:0051865|	GO:0005634|	GO:0016874|lig	K16277
Manes.09G18640(	0.5796215	0.00075	down	no	.	pfam00536	SAM_1	SAM domai	.	.	.	.
Manes.09G18650(	0.8613338	0.12599	down	no	RNA polyme	pfam03031	NIF	NLI interact	GO:0009788|	GO:0005634|	GO:0046872|m	K18999
Manes.09G18690(	0.7216801	0.00658	down	no	.	pfam05266	DUF724	Protein of u	.	.	.	.
Manes.09G18730(	0.7174388	0.00264	down	no	Light-regulat	pfam07207	Lir1	Light regula	GO:0030003|	GO:0009507|	.	.
Manes.09G18740(	0.6504732	0.02663	down	no	Inactive leuci	pfam00069	Pkinase	Protein kina	GO:0009755|	GO:0016021|	GO:0005524|A'	.
Manes.09G18760(	0.3000574	9.13E-06	down	yes	.	pfam15264	TSSC4	tumor suppr	.	.	.	.
Manes.09G18780(	0.741832	0.01535	down	no	Histone-lysin	pfam05033	Pre-SET	Pre-SET mo	GO:0051567|	GO:0000775|	GO:0010385|dc	K11420
Manes.09G18790(	0.7091903	0.00172	down	no	Protein MEI2	pfam04059	RRM_2	RNA recogr	.	.	GO:0000166|nu	.
Manes.09G18800(	0.7341607	0.00499	down	no	Ubiquitin thio	pfam10275	Peptidase_C65	Peptidase C	GO:0016579|	.	GO:0008234|cy	K09602
Manes.09G18820(	1.2641953	0.01134	up	no	Glycine--tRN	pfam00587	tRNA-synt_2b	tRNA synth	GO:0015966|	GO:0005829|	GO:0005524|A'	K01880
Manes.09G18840(	0.5453367	1.70E-07	down	no	.	pfam00650	CRAL_TRIO	CRAL/TRIC	.	.	.	.
Manes.09G18880(	0.3959566	0.00479	down	yes	Sterol 14-der	pfam00067	p450	Cytochrome	GO:0070988|	GO:0005783|	GO:0020037|he	K05917
Manes.09G18900(	0.6239695	3.54E-06	down	no	Protein NRT	pfam00854	PTR2	POT family.	GO:0006857|	GO:0016021|	GO:0005215|tra	.
Manes.10G00030(	1.442349	0.2272	up	no	Superoxide d	pfam02777	Sod_Fe_C	Iron/mangar	.	GO:0005759|	GO:0046872|m	.
Manes.10G00060(	0.7050788	0.0078	down	no	Reticulon-lik	pfam02453	Reticulon	Reticulon. F	.	GO:0005789|	.	.
Manes.10G00070(	18.508077	6.43E-30	up	yes	O-acetyl-ADI	pfam01661	Macro	Macro doma	GO:0060701|	.	GO:0019213|de	.
Manes.10G00100(	0.4198712	1.33E-06	down	yes	Folic acid syr	pfam00809	Pterin_bind	Pterin bindi	GO:0046656|	.	GO:0003848|2-	.
Manes.10G00130(	0.2232422	1.52E-09	down	yes	.	pfam14967	FAM70	FAM70 pro	.	.	.	.
Manes.10G00150(	0.5041909	8.65E-07	down	no	U-box domai	pfam04564	U-box	U-box doma	.	.	GO:0016874|lig	.
Manes.10G00160(	0.7503803	0.17781	down	no	.	pfam09971	DUF2206	Predicted m	.	.	.	.
Manes.10G00200(	0.8705725	0.14321	down	no	Probable inac	pfam00069	Pkinase	Protein kina	.	GO:0005813|	GO:0005524|A'	K08876
Manes.10G00230(	0.4901859	7.15E-06	down	yes	.	pfam08387	FBD	FBD. This r	.	.	.	.
Manes.10G00310(	0.9902479	0.94986	down	no	E3 ubiquitin-	pfam00176	SNF2_N	SNF2 family	GO:0006281|	GO:0000786|	GO:0005524|A'	K15710
Manes.10G00370(	10.565137	2.89E-09	up	yes	Serine/threon	pfam00069	Pkinase	Protein kina	GO:0045727|	GO:0005634|	GO:0005524|A'	K04688
Manes.10G00390(	0.5488674	2.06E-07	down	no	Probable inac	pfam00069	Pkinase	Protein kina	.	GO:0016021|	GO:0005524|A'	.
Manes.10G00410(	0.4704374	1.69E-10	down	yes	.	pfam04633	Herpes_BMRF2	Herpesvirus	.	.	.	.
Manes.10G00490(	0.6160929	0.00013	down	no	.	pfam03219	TLC	TLC ATP/A	.	.	.	.
Manes.10G00500(	0.9638662	0.74434	down	no	Phosphogluc	pfam00782	DSPc	Dual specifi	GO:0046838|	GO:0009507|	GO:0019203|ca	.
Manes.10G00510(	0.1632593	9.87E-05	down	yes	Adenine/guar	pfam00860	Xan_ur_permease	Permease fa	GO:0015853|	GO:0016021|	GO:0005345|pu	K06901
Manes.10G00520(	Inf	0.00667	up	yes	CBL-interact	pfam00069	Pkinase	Protein kina	GO:0007165|	.	GO:0005524|A'	.
Manes.10G00540(	1.0189693	0.85094	up	no	Fructose-1-pl	pfam13419	HAD_2	Haloacid de	GO:0016311|	.	GO:0008801|be	.
Manes.10G00550(	0.5311885	5.72E-06	down	no	.	pfam05701	WEMBL	Weak chlorc	.	.	.	.
Manes.10G00560(	0.4153016	2.78E-07	down	yes	.	pfam05097	DUF688	Protein of u	.	.	.	.
Manes.10G00580(	0.7053657	0.25732	down	no	Peroxidase 4	pfam00141	peroxidase	Peroxidase.	GO:0042744|	GO:0005576|	GO:0020037|he	K00430
Manes.10G00600(	1.1912503	0.78926	up	no	Peroxidase 4	pfam00141	peroxidase	Peroxidase.	GO:0042744|	GO:0005576|	GO:0020037|he	K00430
Manes.10G00630(	0.6784998	0.00027	down	no	APO protein	pfam05634	APO_RNA-bind	APO RNA-l	.	GO:0009507|	GO:0003723|R	.
Manes.10G00650(	0.3985722	4.61E-09	down	yes	.	pfam00503	G-alpha	G-protein al	.	.	.	.
Manes.10G00680(	10.769841	#######	up	yes	.	pfam07107	WI12	Wound-indu	.	.	.	.
Manes.10G00690(	NA	NA	--	no	.	pfam04525	Tub_2	Tubby C 2.	.	.	.	.
Manes.10G00710(	2.2910042	2.34E-06	up	yes	.	pfam13041	PPR_2	PPR repeat	.	.	.	.
Manes.10G00750(	1.4767141	0.00092	up	no	PRA1 family	pfam03208	PRA1	PRA1 family	GO:0016192|	GO:0005783|	.	.
Manes.10G00790(	4.4175342	1.38E-07	up	yes	Abscisic acid	pfam10604	Polyketide_cyc2	Polyketide c	GO:0009738|	GO:0005737|	GO:0010427|ab	K14496
Manes.10G00820(	5.9401629	5.17E-38	up	yes	Putative dise	pfam00931	NB-ARC	NB-ARC do	GO:0006952|	.	GO:0043531|A	.
Manes.10G00870(	0.520329	1.96E-07	down	no	Cytosolic enc	pfam03644	Glyco_hydro_85	Glycosyl hy	.	GO:0005829|	GO:0033925|m	K01227
Manes.10G00900(	0.5801043	3.58E-06	down	no	Multiple inos	pfam00328	His_Phos_2	Histidine ph	GO:0016311|	GO:0005829|	GO:0003993|ac	K03103
Manes.10G00920(	0.9430496	0.61242	down	no	Peptide-N4-(	pfam12222	PNGaseA	Peptide N-a	.	.	GO:0000224|pe	.
Manes.10G00960(	2.4705309	7.40E-25	up	yes	Inactive rece	pfam07714	Pkinase_Tyr	Protein tyro	.	GO:0016021|	GO:0005524|A'	.
Manes.10G00970(	Inf	1.62E-13	up	yes	.	pfam01190	Pollen_Ole_e_I	Pollen prote	.	.	.	.
Manes.10G01010(	2.8615705	3.48E-21	up	yes	.	pfam01704	UDPGP	UTP--gluco	.	.	.	.
Manes.10G01040(	4.7846395	9.83E-09	up	yes	RING-H2 fin	pfam13639	zf-RING_2	Ring finger	GO:0016567|	GO:0016021|	GO:0008270|zi	.
Manes.10G01060(	6.1963543	1.04E-68	up	yes	Probable pro	pfam00481	PP2C	Protein phos	GO:0009738|	GO:0005634|	GO:0046872|m	.
Manes.10G01080(	10.58911	1.27E-05	up	yes	Protein ESKI	pfam13839	PC-Esterase	GDSL/SGN	GO:0045893|	GO:0005794|	GO:0016413|O-	.
Manes.10G01090(	0.4428506	2.52E-13	down	yes	Protein ESKI	pfam13839	PC-Esterase	GDSL/SGN	GO:0045893|	GO:0005794|	GO:0016413|O-	.
Manes.10G01100(	0.3375053	2.04E-17	down	yes	Protein ESKI	pfam13839	PC-Esterase	GDSL/SGN	GO:0045893|	GO:0005794|	GO:0016413|O-	.

Manes.10G01120(	1.0923512	0.33059	up	no	Syntaxin-binc	pfam00957	Synaptobrevin	Synaptobrev	GO:0006887|(	GO:0005737|	.	K08518
Manes.10G01130(	0.6551269	1.70E-05	down	no	Omega-3 fatt	pfam11960	DUF3474	Domain of u	GO:0006636|(	GO:0031969|	GO:0016717|ox	K10257
Manes.10G01160(	3.4653	0.14835	up	no	.	pfam14184	YrvL	Regulatory	.	.	.	.
Manes.10G01170(	17.252053	#######	up	yes	Zinc finger C	pfam12796	Ank_2	Ankyrin rep	GO:0050832|	GO:0005634|	GO:0003677|DI	.
Manes.10G01190(	0.4528452	1.18E-11	down	yes	Serine/threon	pfam00069	Pkinase	Protein kina	GO:0007165|	GO:0051286|	GO:0005524|A'	K18670
Manes.10G01200(	1.6882141	5.16E-08	up	no	Mitochondria	pfam03031	NIF	NLI interact	GO:0007006|	GO:0016021|	GO:0005134|int	K17496
Manes.10G01210(	2.4913036	3.76E-09	up	yes	Putative serir	pfam00069	Pkinase	Protein kina	GO:0009755|l	GO:0016021|	GO:0005524|A'	.
Manes.10G01220(	0.7645681	0.01699	down	no	NF-X1-type z	pfam01422	zf-NF-X1	NF-X1 type	GO:0016567||	GO:0016021|	GO:0016874|lig	K15683
Manes.10G01260(	0.1856082	3.93E-07	down	yes	Putative serir	pfam00069	Pkinase	Protein kina	GO:0009755|l	GO:0016021|	GO:0005524|A'	.
Manes.10G01270(	0.9892151	0.92609	down	no	Phosphoinosi	pfam00388	PI-PLC-X	Phosphatidy	GO:0035556|i	GO:0005622|	GO:0004435|ph	K05857
Manes.10G01280(	0.0927427	1.11E-05	down	yes	Phosphoinosi	pfam00388	PI-PLC-X	Phosphatidy	GO:0035556|i	GO:0005622|	GO:0004435|ph	K05857
Manes.10G01320(	0.7473509	0.07819	down	no	GDSL estera	pfam00657	Lipase_GDSL	GDSL-like l	GO:0016042|l	GO:0048046|	GO:0016788|hy	.
Manes.10G01340(	0.721849	0.03074	down	no	Elongator co	pfam05625	PAXNEB	PAXNEB p	GO:0009738|a	GO:0005737|	GO:0016746|tra	K11375
Manes.10G01350(	9.4251419	1.91E-17	up	yes	.	pfam09068	EF-hand_2	EF hand. M	.	.	.	.
Manes.10G01380(	0.8707992	0.37118	down	no	.	pfam03226	Yippee-Mis18	Yippee zinc	.	.	.	.
Manes.10G01410(	0.432934	0.00115	down	yes	Clathrin light	pfam01086	Clathrin_lg_ch	Clathrin ligh	GO:0072583|	GO:0009504|	GO:0032050|cl	.
Manes.10G01420(	0.6035687	9.54E-05	down	no	.	pfam03959	FSH1	Serine hydrc	.	.	.	.
Manes.10G01500(	Inf	1.40E-08	up	yes	.	pfam00745	GlutR_dimer	Glutamyl-tR	.	.	.	.
Manes.10G01520(	2.797232	4.24E-20	up	yes	.	pfam13634	Nucleoporin_FG	Nucleoporin	.	.	.	.
Manes.10G01540(	Inf	0.08088	up	no	.	pfam14121	DUF4289	Domain of u	.	.	.	.
Manes.10G01550(	5.3929936	1.49E-14	up	yes	Probable LRI	pfam00069	Pkinase	Protein kina	.	GO:0016021|	GO:0005524|A'	.
Manes.10G01590(	0.4014947	4.18E-05	down	yes	LRR recepto	pfam00069	Pkinase	Protein kina	GO:0010204|	GO:0012505|	GO:0005524|A'	K13428
Manes.10G01620(	33.319553	#######	up	yes	Nematode res	pfam07014	Hs1pro-1_C	Hs1pro-1 pr	GO:0009816|	GO:0005737|	.	.
Manes.10G01630(	1.4813113	0.27687	up	no	.	pfam13414	TPR_11	TPR repeat.	.	.	.	.
Manes.10G01640(	0.7615753	0.0162	down	no	Glycosyltrans	pfam09258	Glyco_transf_64	Glycosyl tra	GO:0009738|a	GO:0005768|	GO:0046872|me	.
Manes.10G01660(	0.9673264	0.81028	down	no	Probable aqu	pfam00230	MIP	Major intrin	GO:0009992|	GO:0005887|	GO:0015254|gl	.
Manes.10G01690(	120.39448	4.43E-31	up	yes	Uncharacteriz	pfam02458	Transferase	Transferase	.	.	GO:0016747|tra	.
Manes.10G01710(	0.7497873	0.09256	down	no	.	pfam06762	LMF1	Lipase matu	.	.	.	.
Manes.10G01740(	1.1517514	0.2026	up	no	Peroxisomal	pfam00153	Mito_carr	Mitochondr	GO:0043132|	GO:0046861|	GO:0015297|an	K13354
Manes.10G01780(	3.4126398	4.37E-42	up	yes	.	pfam06123	CreD	Inner memb	.	.	.	.
Manes.10G01790(	1.1501508	0.11944	up	no	Coronatine-ir	pfam02346	Vac_Fusion	Chordopoxv	GO:0009901|a	.	.	K13463
Manes.10G01800(	3.5679775	1.13E-33	up	yes	Transcription	pfam00249	Myb_DNA-binding	Myb-like DI	GO:0048262|	GO:0005634|	GO:0003677|DI	.
Manes.10G01810(	0.8052642	0.02908	down	no	Sedoheptulos	pfam00316	FBPase	Fructose-1-(	GO:0019253|	GO:0009507|	GO:0042132|fru	.
Manes.10G01820(	0.6504636	0.00055	down	no	Adenylosucci	pfam08328	ASL_C	Adenylosuc	GO:0044208|	.	GO:0070626|(S	K01756
Manes.10G01830(	0.6409642	0.00209	down	no	Uncharacteriz	pfam03767	Acid_phosphat_B	HAD superf	GO:0046686|	GO:0016021|	GO:0003993|ac	.
Manes.10G01850(	0.8260671	0.08365	down	no	.	pfam10521	DUF2454	Protein of u	.	.	.	.
Manes.10G01860(	1.201891	0.12082	up	no	LIM domain-	pfam00412	LIM	LIM domair	.	GO:0005737|	GO:0008270|zin	.
Manes.10G01870(	0.0377592	9.94E-05	down	yes	.	pfam05518	Totivirus_coat	Totivirus co	.	.	.	.
Manes.10G01890(	0.7959181	0.18409	down	no	Protein ASPA	pfam14543	TAXi_N	Xylanase inl	GO:0030163|	GO:0005783|	GO:0004190|as	.
Manes.10G01910(	0.8404386	0.13433	down	no	Syntaxin-81	pfam10496	Syntaxin-18_N	SNARE-cor	GO:0061025|	GO:0005783|	GO:0005484|SI	K08492
Manes.10G01940(	3.2646477	6.67E-20	up	yes	Probable sug	pfam03151	TPT	Triose-phos	GO:0008643|	GO:0005783|	.	.
Manes.10G01950(	0.9619366	0.70772	down	no	Proline transp	pfam01490	Aa_trans	Transmembr	GO:0035524|	GO:0016021|	GO:0015193|L-	.
Manes.10G01960(	0.6585733	0.00048	down	no	.	pfam00139	Lectin_legB	Legume lect	.	.	.	.
Manes.10G01980(	0.4033223	3.34E-07	down	yes	.	pfam12906	RINGv	RING-varia	.	.	.	.
Manes.10G01990(	4.1146287	3.88E-09	up	yes	.	pfam04788	DUF620	Protein of u	.	.	.	.
Manes.10G02000(	6.7153971	5.48E-51	up	yes	17.3 kDa clas	pfam00011	HSP20	Hsp20/alpha	.	GO:0005737|	.	K13993
Manes.10G02010(	43.545754	#######	up	yes	18.2 kDa clas	pfam00011	HSP20	Hsp20/alpha	.	GO:0005737|	.	.
Manes.10G02020(	0.6050309	0.00239	down	no	Probable met	pfam03141	Methyltransf_29	Putative S-a	.	GO:0005768|	GO:0008168|me	.
Manes.10G02030(	6.7468617	1.30E-20	up	yes	7-deoxylogar	pfam10156	Med17	Subunit 17 (	GO:1900994|(	.	GO:0035251|UI	.
Manes.10G02040(	2.284176	1.09E-20	up	yes	7-deoxylogar	pfam00201	UDPGT	UDP-glucor	GO:1900994|(	.	GO:0035251|UI	.
Manes.10G02050(	0.6005352	0.0303	down	no	UDP-glycosy	pfam00201	UDPGT	UDP-glucor	.	.	GO:0016758|tra	.
Manes.10G02120(	3.2586143	8.23E-15	up	yes	.	pfam04276	DUF443	Protein of u	.	.	.	.
Manes.10G02130(	14.133529	0.00011	up	yes	.	pfam11581	Argos	Antagonist (	.	.	.	.
Manes.10G02180(	Inf	0.1623	up	no	Cucumisin	pfam00082	Peptidase_S8	Subtilase far	.	GO:0005576|	GO:0004252|se	.
Manes.10G02210(	0.2454738	2.12E-26	down	yes	Protein FAR1	pfam03101	FAR1	FAR1 DNA	GO:0006355|	GO:0005634|	GO:0008270|zin	.
Manes.10G02250(	1.6434197	5.70E-08	up	no	Disease resist	pfam00931	NB-ARC	NB-ARC dc	GO:0006952|	GO:0005886|	GO:0043531|AI	.
Manes.10G02290(	0.6946906	0.0528	down	no	Folylpolyglut	pfam14093	DUF4271	Domain of u	GO:0006730|	GO:0009507|	GO:0005524|A'	K01930
Manes.10G02320(	1.6743842	1.64E-05	up	no	Putative disea	pfam00931	NB-ARC	NB-ARC dc	GO:0006952|	GO:0009506|	GO:0043531|AI	.
Manes.10G02360(	0.5786683	0.00411	down	no	Probable glyc	pfam03016	Exostosin	Exostosin fa	GO:0071555|	GO:0000139|	GO:0016757|tra	.
Manes.10G02400(	4.7015193	0.04218	up	no	Probably inac	pfam13855	LRR_8	Leucine rich	GO:0009755|l	GO:0005783|	GO:0005524|A'	.
Manes.10G02450(	Inf	0.35135	up	no	PRA1 family	pfam03208	PRA1	PRA1 family	GO:0016192|	GO:0005783|	.	.
Manes.10G02480(	7.0074556	#######	up	yes	Delta-1-pyrro	pfam00696	AA_kinase	Amino acid	GO:0055129|	GO:0005737|	GO:0005524|A'	.
Manes.10G02490(	4.5602286	5.12E-38	up	yes	CASP-like pr	pfam04535	DUF588	Domain of u	.	GO:0016021|	.	.
Manes.10G02560(	Inf	0.04209	up	no	.	pfam02891	zf-MIZ	MIZ/SP-RII	.	.	.	.
Manes.10G02580(	Inf	0.08088	up	no	.	pfam03248	Rer1	Rer1 family.	.	.	.	.
Manes.10G02620(	1.6606252	2.71E-07	up	no	Probable met	pfam03141	Methyltransf_29	Putative S-a	.	GO:0005768|	GO:0008168|me	.
Manes.10G02640(	1.9275737	1.13E-08	up	no	Protein phosp	pfam00481	PP2C	Protein phos	GO:0009738|a	GO:0005829|	GO:0046872|me	K14497
Manes.10G02650(	1.3361284	0.15496	up	no	.	pfam00106	adh_short	short chain (	.	.	.	.
Manes.10G02660(	0.6923252	0.00151	down	no	.	pfam00106	adh_short	short chain (	.	.	.	.
Manes.10G02680(	0.3209365	2.80E-08	down	yes	.	pfam00106	adh_short	short chain (	.	.	.	.
Manes.10G02690(	0.4908006	0.00106	down	yes	.	pfam04646	DUF604	Protein of u	.	.	.	.
Manes.10G02700(	0.4537584	4.90E-05	down	yes	AT-hook mo	pfam03479	DUF296	Domain of u	GO:0006325|	GO:0005634|	GO:0003680|A'	.
Manes.10G02780(	1.0643707	0.50161	up	no	Pumilio homc	pfam07990	NABP	Nucleic acid	GO:0006417|	GO:0005737|	GO:0003729|ml	K17943
Manes.10G02790(	4.1226605	1.44E-11	up	yes	E3 ubiquitin-	pfam06547	DUF1117	Protein of u	GO:0016567||	.	GO:0016874|lig	.
Manes.10G02800(	2.8773383	1.80E-14	up	yes	Short-chain-e	pfam00378	ECH	Enoyl-CoA	GO:0019605|l	.	GO:0016829|ly	K01715
Manes.10G02840(	0.8737843	0.42932	down	no	Probable dise	pfam00931	NB-ARC	NB-ARC dc	GO:0006952|	.	GO:0043531|AI	.
Manes.10G02850(	2.589881	0.09835	up	no	Receptor-like	pfam13855	LRR_8	Leucine rich	GO:0009755|l	GO:0016021|	GO:0004672|pr	.
Manes.10G02860(	0.9295364	0.66028	down	no	Disease resist	pfam00931	NB-ARC	NB-ARC dc	GO:0006952|	.	GO:0043531|AI	.
Manes.10G02890(	6.1704366	7.44E-12	up	yes	.	pfam10320	7TM_GPCR_Srsx	Serpentine t	.	.	.	.
Manes.10G02910(	1.6276111	0.00098	up	no	Ribosomal R	pfam01728	FtsJ	FtsJ-like me	.	GO:0005737|	GO:0008650|rR	K02427
Manes.10G02930(	2.0646629	1.60E-16	up	yes	Telomere rep	pfam14571	Di19_C	Stress-induc	GO:0006355|	GO:0000784|	GO:0008301|DI	.
Manes.10G02970(	1.6505624	9.12E-08	up	no	Probable dua	pfam04055	Radical_SAM	Radical SAN	GO:0070475|	GO:0005737|	GO:0051539|4	K06941
Manes.10G02980(	2.5847536	1.49E-11	up	yes	Protein kinas	pfam00069	Pkinase	Protein kina	.	GO:0009507|	GO:0005524|A'	.
Manes.10G02990(	8.1735346	4.30E-08	up	yes	Probable dise	pfam00931	NB-ARC	NB-ARC dc	GO:0006952|	.	GO:0043531|AI	.
Manes.10G03000(	7.1174348	7.00E-25	up	yes	Probable dise	pfam00931	NB-ARC	NB-ARC dc	GO:0006952|	.	GO:0043531|AI	.
Manes.10G03010(	5.3736569	5.32E-79	up	yes	.	pfam04720	DUF506	Protein of u	.	.	.	.
Manes.10G03040(	0.3877993	2.79E-06	down	yes	Dolichyl-pho	pfam00535	Glycos_transf_2	Glycosyl tra	GO:0040003|	GO:0005783|	GO:0004581|do	K00729
Manes.10G03110(	80.575673	2.26E-16	up	yes	Probable nuc	pfam03107	C1_2	C1 domain.	GO:0045454|	GO:0005623|	GO:0047134|pr	K17609

Manes.10G03140(	0.537996	7.58E-07	down	no	Heat shock c	pfam00012	HSP70	Hsp70 prote	.	.	GO:0005524|A	.
Manes.10G03200(	0.72795	0.00208	down	no	Heat shock c	pfam00012	HSP70	Hsp70 prote	.	.	GO:0005524|A	.
Manes.10G03240(	0.2379538	0.0011	down	yes	Meiotic recor	pfam04406	TP6A_N	Type IIB DI	GO:0051026|	GO:0005694|	GO:0005524|A	K10878
Manes.10G03250(	1.8038746	5.13E-11	up	no	UDP-glucuro	pfam01370	Epimerase	NAD depen	GO:0033320|	GO:0005737|	GO:0050662|co	K08678
Manes.10G03270(	2.2456965	7.10E-20	up	yes	.	pfam01842	ACT	ACT domai	.	.	.	.
Manes.10G03280(	1.0114147	0.9011	up	no	Cellular nucle	pfam00098	zf-CCHC	Zinc knuckle	GO:0008284|	GO:0005829|	GO:0003677|D	K09250
Manes.10G03290(	1.857085	0.00089	up	no	.	pfam00378	ECH	Enoyl-CoA	.	.	.	.
Manes.10G03300(	1.4643622	2.31E-05	up	no	.	pfam05145	AmoA	Putative am	.	.	.	.
Manes.10G03320(	20.914438	1.88E-31	up	yes	B3 domain-c	pfam02362	B3	B3 DNA bin	GO:0009909|	GO:0005654|	GO:0003677|D	.
Manes.10G03330(	0.9697362	0.78607	down	no	B3 domain-c	pfam02362	B3	B3 DNA bin	GO:0009909|	GO:0005654|	GO:0003677|D	.
Manes.10G03340(	4.4347606	0.10819	up	no	B3 domain-c	pfam02362	B3	B3 DNA bin	GO:0009909|	GO:0005654|	GO:0003677|D	.
Manes.10G03360(	1.4600988	2.60E-05	up	no	Actin-depoly	pfam00241	Cofilin_ADF	Cofilin/trop	GO:0030042|	GO:0015629|	.	.
Manes.10G03370(	#NAME?	0.29366	down	no	.	pfam04043	PMEI	Plant inverta	.	.	.	.
Manes.10G03410(	Inf	0.17988	up	no	.	pfam03281	Mab-21	Mab-21 pro	.	.	.	.
Manes.10G03420(	11.950453	2.49E-06	up	yes	Scopoletin gl	pfam00201	UDPGT	UDP-glucor	.	.	GO:0042802|id	.
Manes.10G03440(	0.8449725	0.09866	down	no	2-oxoglutarat	pfam00676	E1_dh	Dehydrogen	GO:0006096|	GO:0009353|	GO:0004591|ox	K00164
Manes.10G03470(	1.1900655	0.09097	up	no	.	pfam14905	OMP_b-brl_3	Outer memb	.	.	.	.
Manes.10G03480(	0.7181219	0.00175	down	no	Photosynthet	pfam01789	PsbP	PsbP. This f	GO:0015979|	GO:0009507|	GO:0005509|ca	.
Manes.10G03500(	0.3197619	1.91E-06	down	yes	Aquaporin TI	pfam00230	MIP	Major intrin	GO:0009992|	GO:0042807|	GO:0015254|gl	K09873
Manes.10G03510(	0.4547426	8.37E-13	down	yes	.	pfam00646	F-box	F-box doma	.	.	.	.
Manes.10G03560(	Inf	0.9009	up	no	Pectinesteras	pfam01095	Pectinesterase	Pectinestera	GO:0042545|	GO:0005618|	GO:0045330|as	K01051
Manes.10G03590(	1.7173755	0.00014	up	no	Protein gar2	pfam00076	RRM_1	RNA recogn	GO:0006364|	GO:0001651|	GO:0000166|nu	K11294
Manes.10G03600(	Inf	0.9009	up	no	.	pfam01284	MARVEL	Membrane-a	.	.	.	.
Manes.10G03630(	NA	NA	--	no	Exocyst comp	pfam03081	Exo70	Exo70 exoc	GO:0006887|	GO:0005618|	.	.
Manes.10G03640(	11.723406	1.65E-06	up	yes	Caffeoylshiki	pfam12146	Hydrolase_4	Putative lyso	GO:0009809|	GO:0005886|	GO:0003846|2-	K18368
Manes.10G03660(	15.165518	0.00394	up	yes	Dirigent prot	pfam03018	Dirigent	Dirigent-like	.	GO:0048046|	.	.
Manes.10G03730(	0.1955597	7.40E-10	down	yes	.	pfam09713	A_thal_3526	Plant protei	.	.	.	.
Manes.10G03750(	0.8709868	0.31011	down	no	Sulfate transp	pfam00916	Sulfate_transp	Sulfate trans	.	GO:0031969|	GO:0008271|se	K18059
Manes.10G03790(	0.4710957	0.0018	down	yes	.	pfam12730	ABC2_membrane_	ABC-2 fami	.	.	.	.
Manes.10G03800(	0.5269488	0.00253	down	no	.	pfam05056	DUF674	Protein of u	.	.	.	.
Manes.10G03810(	0.4433885	0.0006	down	yes	.	pfam05056	DUF674	Protein of u	.	.	.	.
Manes.10G03820(	0.5327784	1.68E-05	down	no	Protein kinas	pfam00069	Pkinase	Protein kina	.	GO:0009507|	GO:0005524|A	.
Manes.10G03830(	2.0234005	2.66E-13	up	yes	Ankyrin repe	pfam12796	Ank_2	Ankyrin rep	GO:0034613|	GO:0016021|	.	.
Manes.10G03860(	0.9288353	0.4536	down	no	.	pfam10174	Cast	RIM-binding	.	.	.	.
Manes.10G03900(	0.5515466	0.02033	down	no	ABC transpo	pfam01061	ABC2_membrane	ABC-2 type	GO:0010208|	GO:0016021|	GO:0005524|A	.
Manes.10G03950(	1.2135549	0.03406	up	no	Macrophage	pfam10607	CLTH	CTLH/CRA	GO:0007049|	GO:0005737|	.	K18624
Manes.10G03960(	0.6502987	1.13E-05	down	no	.	pfam00504	Chloroa_b-bind	Chlorophyll	.	.	.	.
Manes.10G03970(	0.3754412	0.38549	down	no	.	pfam14392	zf-CCHC_4	Zinc knuckle	.	.	.	.
Manes.10G04040(	1.5817614	0.00049	up	no	Adenylate kin	pfam00406	ADK	Adenylate k	GO:0008652|	GO:0009507|	GO:0004017|ad	K00939
Manes.10G04060(	0.5422621	2.95E-05	down	no	.	pfam13344	Hydrolase_6	Haloacid de	.	.	.	.
Manes.10G04110(	2.9799557	3.01E-33	up	yes	Floral homeo	pfam00847	AP2	AP2 domair	GO:0009873|	GO:0005634|	GO:0003677|D	K09284
Manes.10G04120(	1.1338157	0.18688	up	no	.	pfam02582	DUF155	Uncharacter	.	.	.	.
Manes.10G04170(	0.7059096	0.00058	down	no	Serine/threon	pfam00855	PWWP	PWWP dom	GO:0006974|	GO:0005634|	GO:0005524|A	K04728
Manes.10G04200(	1.4818004	0.01996	up	no	Putative pent	pfam13041	PPR_2	PPR repeat	.	GO:0005739|	.	.
Manes.10G04220(	0.7051544	0.02809	down	no	40S ribosoma	pfam00164	Ribosom_S12_S2	Ribosomal p	GO:0006412|	GO:0015935|	GO:0003735|str	.
Manes.10G04230(	3.5301053	1.61E-05	up	yes	.	pfam08288	PIGA	PIGA (GPI	.	.	.	.
Manes.10G04240(	7.6600371	1.42E-06	up	yes	.	pfam03936	Terpene_synth_C	Terpene syn	.	.	.	.
Manes.10G04250(	Inf	0.9009	up	no	Low tempera	pfam01679	Pmp3	Proteolipid	.	GO:0016021|	.	.
Manes.10G04320(	1.0534909	0.68982	up	no	Mitochondria	pfam00226	DnaJ	DnaJ domai	.	GO:0016021|	.	K09539
Manes.10G04350(	#NAME?	0.43239	down	no	.	pfam14436	EndoU_bacteria	Bacterial En	.	.	.	.
Manes.10G04370(	0.9713927	0.8091	down	no	Nucleolar cor	pfam03715	Noc2	Noc2p famil	.	GO:0005634|	.	K14833
Manes.10G04460(	2.1259532	0.26654	up	no	MLO-like pro	pfam03094	Mlo	Mlo family.	GO:0006952|	GO:0016021|	.	K08472
Manes.10G04490(	0.7245914	0.00412	down	no	Chlorophyll a	pfam00504	Chloroa_b-bind	Chlorophyll	GO:0009765|	GO:0009535|	GO:0016168|ch	K08907
Manes.10G04500(	0.6819556	0.00013	down	no	Ethylene rece	pfam02518	HATPase_c	Histidine kir	GO:0009873|	GO:0005789|	GO:0005524|A	.
Manes.10G04530(	1.0258254	0.89651	up	no	Patatin-like p	pfam01734	Patatin	Patatin-like	GO:0019374|	GO:0005886|	GO:0047617|ac	.
Manes.10G04550(	1.3804742	0.00048	up	no	V-type proto	pfam03179	V-ATPase_G	Vacuolar (H	GO:0015992|	GO:0016471|	GO:0016820|hy	.
Manes.10G04580(	1.3816933	0.00069	up	no	Caffeoylshiki	pfam12697	Abhydrolase_6	Alpha/beta l	GO:0009809|	GO:0005886|	GO:0003846|2-	K18368
Manes.10G04640(	0.8534611	0.10661	down	no	Endoplasmic	pfam04389	Peptidase_M28	Peptidase fa	.	GO:0005789|	GO:0046872|m	.
Manes.10G04660(	0.9951558	0.98363	down	no	Phosphomanı	pfam02878	PGM_PMM_I	Phosphoglu	GO:0042121|	.	GO:0000287|m	K15778
Manes.10G04670(	1.0085155	0.90914	up	no	Auxin respon	pfam06507	Auxin_resp	Auxin respo	GO:0009734|	GO:0005634|	GO:0003677|D	.
Manes.10G04680(	2.5562199	7.92E-27	up	yes	GATA transc	pfam00320	GATA	GATA zinc	GO:0007623|	GO:0005634|	GO:0043565|se	.
Manes.10G04720(	2.629861	0.02554	up	yes	.	pfam13703	PepSY_TM_2	PepSY-asso	.	.	.	.
Manes.10G04730(	1.6976535	7.20E-08	up	no	Uncharacteri	pfam14624	Vwaint	VWA / Hh	.	.	.	K07114
Manes.10G04810(	0.1514645	0.00038	down	yes	.	pfam13705	TRC8_N	TRC8 N-ter	.	.	.	.
Manes.10G04820(	0.3142005	2.68E-10	down	yes	Probable acet	pfam00583	Acetyltransf_1	Acetyltransf	.	GO:0009507|	GO:0008080|N	.
Manes.10G04870(	0.80387	0.05732	down	no	ABC transpo	pfam00005	ABC_tran	ABC transp	GO:0006810|	GO:0016021|	GO:0005524|A	.
Manes.10G04900(	0.9449501	0.57601	down	no	Pumilio homo	pfam07990	NABP	Nucleic acid	GO:0006417|	GO:0005737|	GO:0003729|m	.
Manes.10G04910(	Inf	8.33E-08	up	yes	Polyubiquitin	pfam00240	ubiquitin	Ubiquitin fa	GO:0006511|	GO:0005737|	.	K08770
Manes.10G04950(	21.029212	5.92E-19	up	yes	.	pfam05975	EcsB	Bacterial AI	.	.	.	.
Manes.10G04960(	2.7786368	2.99E-09	up	yes	.	pfam04647	AgrB	Accessory g	.	.	.	.
Manes.10G04970(	3.3046035	2.67E-06	up	yes	.	pfam03025	Papilloma_E5	Papillomavi	.	.	.	.
Manes.10G04980(	12.33612	4.52E-19	up	yes	.	.	.	.	.	.	.	.
Manes.10G05000(	1.1884781	0.06255	up	no	Ras-related p	pfam00071	Ras	Ras family.	GO:0045022|	GO:0010009|	GO:0005525|G	.
Manes.10G05050(	0.4782092	0.12507	down	no	.	pfam15551	DUF4656	Domain of u	.	.	.	.
Manes.10G05070(	1.1241393	0.32541	up	no	Serine carbox	pfam00450	Peptidase_S10	Serine carbo	.	GO:0005829|	GO:0004185|se	K16298
Manes.10G05080(	Inf	2.65E-08	up	yes	Vinorine synt	pfam02458	Transferase	Transferase	GO:0009820|	.	GO:0050636|vi	.
Manes.10G05100(	0.8588849	0.25751	down	no	Glycine-rich	pfam00076	RRM_1	RNA recogn	GO:0009409|	GO:0005634|	GO:0003677|D	K12885
Manes.10G05110(	0.7299802	0.09574	down	no	.	pfam09511	RNA_lig_T4_1	RNA ligase.	.	.	.	.
Manes.10G05120(	0.9513116	0.6999	down	no	Glycine-rich	pfam00076	RRM_1	RNA recogn	GO:0009409|	GO:0005730|	GO:0003677|D	K12885
Manes.10G05130(	12.423371	3.88E-18	up	yes	.	pfam06813	Nodulin-like	Nodulin-like	.	.	.	.
Manes.10G05150(	2.006607	1.08E-13	up	yes	Probable pect	pfam00544	Pec_lyase_C	Pectate lyas	GO:0045490|	GO:0031225|	GO:0046872|m	K01728
Manes.10G05170(	0.9414046	0.54442	down	no	Pullulanase 1	pfam11852	DUF3372	Domain of u	GO:0019252|	GO:0009507|	GO:0043169|ca	.
Manes.10G05210(	1.9594857	0.00017	up	no	Ras-related p	pfam00071	Ras	Ras family.	GO:0006886|	GO:0005794|	GO:0005525|G	K07910
Manes.10G05230(	NA	NA	--	no	Ras-related p	pfam00071	Ras	Ras family.	GO:0006886|	GO:0005794|	GO:0005525|G	K07910
Manes.10G05240(	1.3012726	0.00604	up	no	.	pfam08614	ATG16	Autophagy	.	.	.	.
Manes.10G05300(	2.3072192	8.47E-21	up	yes	.	pfam01576	Myosin_tail_1	Myosin tail.	.	.	.	.
Manes.10G05340(	0.632984	0.00059	down	no	.	pfam08395	7tm_7	7tm Chemos	.	.	.	.
Manes.10G05350(	1.9850308	3.31E-07	up	no	Phosphogluca	pfam00782	DSPc	Dual specifi	GO:0007623|	GO:0009501|	GO:0019203|ca	.

Manes.10G05380(	3.4508034	6.49E-17	up	yes	SEC12-like p	pfam00400	WD40	WD domain	GO:0016036|	GO:0005783|	.	K14003
Manes.10G05390(	5.5188119	4.63E-81	up	yes	Glycosyltrans	pfam09258	Glyco_transf_64	Glycosyl tra	GO:0006024|	GO:0016021|	GO:0046872|m	.
Manes.10G05580(	2.3124087	3.06E-17	up	yes	Lysine--tRNA	pfam00152	tRNA-synt_2	tRNA synth	GO:0006430|	GO:0005829|	GO:0005524|A	K04567
Manes.10G05650(	0.6984059	0.00204	down	no	Acetylglutam	pfam00696	AA_kinase	Amino acid	GO:0042450|	GO:0009507|	GO:0003991|ac	K00930
Manes.10G05680(	35.749536	#######	up	yes	.	pfam07939	DUF1685	Protein of u	.	.	.	.
Manes.10G05740(	1.9801721	1.73E-13	up	no	.	pfam10513	EPL1	Enhancer of	.	.	.	.
Manes.10G05750(	0.5270725	1.98E-07	down	no	.	pfam13041	PPR_2	PPR repeat	.	.	.	.
Manes.10G05760(	0.5539521	0.10535	down	no	Serine/threon	pfam00069	Pkinase	Protein kina	GO:0009734|	GO:0005737|	GO:0005524|A	K08286
Manes.10G05770(	0.9310767	0.46211	down	no	Alkaline/neut	pfam12899	Glyco_hydro_100	Alkaline anc	GO:0048364|	GO:0005829|	GO:0004564|be	.
Manes.10G05780(	5.7249239	3.36E-13	up	yes	GDSL estera	pfam00657	Lipase_GDSL	GDSL-like l	GO:0016042|	GO:0005576|	GO:0016788|hy	.
Manes.10G05790(	1.3721046	0.00046	up	no	Glycine-rich l	pfam00076	RRM_1	RNA recogn	GO:0009409|	GO:0005739|	GO:0003690|dc	.
Manes.10G05810(	0.4007483	0.00375	down	yes	.	pfam10317	7TM_GPCR_Srd	Serpentine t	.	.	.	.
Manes.10G05830(	1.1444364	0.14905	up	no	cAMP-regula	pfam12752	SUZ	SUZ domain	GO:0034605|	GO:0005737|	GO:0003676|nu	.
Manes.10G05840(	19.19818	2.67E-10	up	yes	.	pfam07558	Shugoshin_N	Shugoshin N	.	.	.	.
Manes.10G05850(	0.9433832	0.61766	down	no	Probable asp	pfam02127	Peptidase_M18	Aminopepti	.	GO:0005737|	GO:0004177|an	K01267
Manes.10G05900(	1.16208	0.1201	up	no	3-hydroxyiso	pfam13766	ECH_C	2-enoyl-Co/	GO:0009409|	GO:0005777|	GO:0003860|3-	K05605
Manes.10G05920(	0.8861315	0.32301	down	no	7-methylguar	pfam05822	UMPH-1	Pyrimidine 5	GO:0009117|	GO:0005737|	GO:0008253|5'-	.
Manes.10G05930(	0.4807412	0.00039	down	yes	.	pfam10358	NT-C2	N-terminal C	.	.	.	.
Manes.10G05980(	1.8567062	2.69E-12	up	no	Calcium-depe	pfam00069	Pkinase	Protein kina	GO:0009738|	GO:0005737|	GO:0005524|A	K13412
Manes.10G05990(	1.1908171	0.05517	up	no	Uricase-2	pfam01014	Uricase	Uricase.	GO:0009877|	GO:0005777|	GO:0004846|ur	.
Manes.10G06020(	0.7992506	0.06167	down	no	.	pfam02485	Branch	Core-2/I-Br	.	.	.	.
Manes.10G06040(	2.5832561	1.18E-09	up	yes	Pentatricoper	pfam13041	PPR_2	PPR repeat	.	GO:0005739|	.	.
Manes.10G06150(	1.2219645	0.10628	up	no	Ferredoxin-3,	pfam00111	Fer2	2Fe-2S iron	GO:0022900|	GO:0009507|	GO:0051537|2	K02639
Manes.10G06160(	2.5894974	2.76E-05	up	yes	Protein TIC 2	pfam01757	Acyl_transf_3	Acyltransfer	GO:0045037|	GO:0009507|	.	.
Manes.10G06220(	0.5057338	0.00174	down	no	.	pfam01814	Hemerythrin	Hemerythrin	.	.	.	.
Manes.10G06240(	1.8047813	8.39E-11	up	no	Vesicle-assoc	pfam00957	Synaptobrevin	Synaptobrev	GO:0006887|	GO:0005768|	GO:0005484|SN	K08511
Manes.10G06270(	1.6650762	1.86E-07	up	no	Multiple mye	pfam10159	MMtag	Kinase phos	.	.	GO:0044822|pc	.
Manes.10G06280(	0.7119205	0.07206	down	no	Transcription	pfam00249	Myb_DNA-binding	Myb-like DI	GO:0048262|	GO:0005634|	GO:0003677|DI	.
Manes.10G06300(	0.7442381	0.01441	down	no	.	pfam08145	BOP1NT	BOP1NT (N	.	.	.	.
Manes.10G06310(	0.7061671	0.00906	down	no	.	pfam01576	Myosin_tail_1	Myosin tail.	.	.	.	.
Manes.10G06360(	0.6542565	0.00214	down	no	Expansin-A1	pfam01357	Pollen_allerg_1	Pollen allerg	GO:0009664|	GO:0005618|	.	.
Manes.10G06370(	0.7610304	0.00778	down	no	TPR and ank	pfam13087	AAA_12	AAA domai	.	.	GO:0005524|A	.
Manes.10G06400(	2.1842982	1.04E-10	up	yes	Putative pent	pfam13041	PPR_2	PPR repeat	.	.	.	K17710
Manes.10G06420(	2.132003	0.00031	up	yes	Probable pect	pfam01095	Pectinesterase	Pectinestera	GO:0042545|	GO:0005618|	GO:0045330|as	K01051
Manes.10G06440(	2.0485761	0.00062	up	yes	Uncharacteriz	pfam00462	Glutaredoxin	Glutaredoxi	GO:0045454|	GO:0005886|	GO:0009055|el	.
Manes.10G06460(	Inf	0.9009	up	no	.	pfam03419	Peptidase_U4	Sporulation	.	.	.	.
Manes.10G06500(	Inf	0.00725	up	yes	.	pfam01646	Herpes_UL24	Herpes virus	.	.	.	.
Manes.10G06510(	0.5489614	5.85E-06	down	no	BTB/POZ do	pfam00651	BTB	BTB/POZ d	GO:0016567|	.	.	.
Manes.10G06570(	0.6817023	0.00025	down	no	Zinc-binding	pfam00107	ADH_zinc_N	Zinc-binding	.	GO:0005739|	GO:0016491|ox	K07119
Manes.10G06620(	0.8583814	0.14869	down	no	Presequence	pfam08367	M16C_assoc	Peptidase M	GO:0016485|	GO:0048046|	GO:0004222|m	.
Manes.10G06640(	0.8882438	0.22595	down	no	Probable carl	pfam07859	Abhydrolase_3	alpha/beta h	.	GO:0005737|	GO:0052689|ca	.
Manes.10G06660(	1.4113355	0.00304	up	no	2-hydroxyiso	pfam07859	Abhydrolase_3	alpha/beta h	GO:0009813|	.	GO:0033987|2-	.
Manes.10G06670(	0.5300942	0.03278	down	no	.	pfam07942	N2227	N2227-like	.	.	.	.
Manes.10G06680(	0.4707214	6.34E-07	down	yes	.	pfam02536	mTERF	mTERF. Th	.	.	.	.
Manes.10G06750(	1.1669309	0.20973	up	no	.	pfam11062	DUF2863	Protein of u	.	.	.	.
Manes.10G06770(	106.50226	#######	up	yes	Abscisic acid	pfam00067	p450	Cytochrome	GO:0046345|	GO:0016021|	GO:0010295|(+	K09843
Manes.10G06780(	0.7945418	0.04135	down	no	YTH domain	pfam04146	YTH	YT521-B-lil	GO:0006959|	GO:0000932|	GO:1990247|N	.
Manes.10G06830(	0.6999003	0.00563	down	no	.	pfam03763	Remorin_C	Remorin, C-	.	.	.	.
Manes.10G06840(	0.9889993	0.9307	down	no	Sodium/hydr	pfam00999	Na_H_Exchanger	Sodium/hyd	GO:0006885|	GO:0005768|	GO:0015385|so	K14724
Manes.10G06860(	0.5361188	3.90E-07	down	no	Inactive leuc	pfam00069	Pkinase	Protein kina	.	GO:0016021|	GO:0005524|A	.
Manes.10G06890(	Inf	0.1623	up	no	Chloroplast e	pfam03040	CemA	CemA famil	.	GO:0009706|	GO:0015078|hy	.
Manes.10G06900(	4.0751214	0.02064	up	yes	Gibberellin 2-	pfam03171	2OG-FeII_Oxy	2OG-Fe(II)	GO:0009686|	.	GO:0052635|C-	.
Manes.10G06910(	NA	NA	--	no	.	pfam07916	TraG_N	TraG-like pr	.	.	.	.
Manes.10G06920(	1.4969957	6.57E-06	up	no	ADP-ribosyla	pfam01412	ArfGap	Putative GT	.	GO:0005737|	GO:0005096|G	K12489
Manes.10G06930(	0.6974202	0.00023	down	no	Inactive poly	pfam12174	RST	RCD1-SRO	GO:0009816|	GO:0005737|	GO:0003950|N	.
Manes.10G06940(	1.4677418	0.06888	up	no	Protein ZINC	pfam07690	MFS_1	Major Facili	GO:0010540|	GO:0016021|	GO:0022821|pc	.
Manes.10G06970(	0.3599901	2.64E-10	down	yes	Pentatricoper	pfam13041	PPR_2	PPR repeat	.	GO:0005739|	.	.
Manes.10G07000(	0.4790812	0.09471	down	no	Limonoid UI	pfam00201	UDPGT	UDP-glucor	.	.	GO:0050645|lin	.
Manes.10G07100(	1.206787	0.09749	up	no	Ubiquitin car	pfam12436	USP7_ICP0_bdg	ICP0-bindin	GO:0006511|	GO:0005829|	GO:0008234|cy	.
Manes.10G07120(	0.4607249	1.81E-10	down	yes	Sucrose-phos	pfam05116	S6PP	Sucrose-6F-	GO:0005986|	.	GO:0000287|m	.
Manes.10G07160(	0.1500799	0.0053	down	yes	Abscisic acid	pfam00067	p450	Cytochrome	GO:0046345|	GO:0016021|	GO:0010295|(+	K09843
Manes.10G07200(	0.5290022	1.21E-07	down	no	Protein DEFI	pfam05517	p25-alpha	p25-alpha. T	GO:0006306|	GO:0000419|	GO:0042803|pr	.
Manes.10G07250(	1.6508095	3.04E-07	up	no	Putative UPF	pfam03140	DUF247	Plant protei	.	GO:0016021|	.	.
Manes.10G07340(	0.8424278	0.28789	down	no	Origin recogr	pfam14629	ORC4_C	Origin recog	GO:0006270|	GO:0015629|	GO:0005524|A	K02606
Manes.10G07360(	0.3004581	4.41E-23	down	yes	Receptor pro	pfam00069	Pkinase	Protein kina	.	GO:0016021|	GO:0005524|A	.
Manes.10G07370(	0.8104287	0.04096	down	no	Probable carl	pfam03629	DUF303	Domain of u	.	.	GO:0016787|hy	.
Manes.10G07420(	0.3656328	1.46E-13	down	yes	Putative disea	pfam00931	NB-ARC	NB-ARC dc	GO:0006952|	.	GO:0043531|A	.
Manes.10G07430(	1.3574991	0.00596	up	no	.	pfam15341	SLX9	Ribosome b	.	.	.	.
Manes.10G07440(	1.5548119	8.66E-07	up	no	.	pfam13041	PPR_2	PPR repeat	.	.	.	.
Manes.10G07450(	2.3573019	6.13E-11	up	yes	3-oxo-Delta(	pfam13460	NAD_binding_10	NADH(P)-b	GO:0009611|	GO:0005829|	GO:0047787|de	.
Manes.10G07470(	2.5841326	2.78E-19	up	yes	Probable prot	pfam00481	PP2C	Protein phos	GO:0006470|	.	GO:0046872|m	.
Manes.10G07570(	1.0226858	0.82469	up	no	Alpha-L-fuco	pfam14498	Glyco_hyd_65N_2	Glycosyl hy	.	GO:0048046|	GO:0047513|1,	K15923
Manes.10G07580(	1.1446487	0.1755	up	no	Alpha-L-fuco	pfam14498	Glyco_hyd_65N_2	Glycosyl hy	.	GO:0048046|	GO:0047513|1,	K15923
Manes.10G07590(	Inf	0.9009	up	no	.	pfam00223	PsaA_PsaB	Photosysten	.	.	.	.
Manes.10G07620(	0.4297584	3.53E-05	down	yes	Pentatricoper	pfam13041	PPR_2	PPR repeat	GO:0031425|	GO:0009507|	.	.
Manes.10G07700(	0.8015007	0.23239	down	no	Scopoletin gl	pfam00201	UDPGT	UDP-glucor	.	.	GO:0042802|id	.
Manes.10G07710(	0.7907435	0.02307	down	no	Phospholipas	pfam01764	Lipase_3	Lipase (clas	GO:0071493|	GO:0009507|	GO:0008970|ph	.
Manes.10G07720(	5.5901974	7.45E-79	up	yes	Cysteine prot	pfam00112	Peptidase_C1	Papain famil	GO:0050832|	GO:0048046|	GO:0004197|cy	.
Manes.10G07740(	3.4535652	4.05E-46	up	yes	Elicitor-resp	pfam00168	C2	C2 domain.	.	GO:0005737|	GO:0005544|ca	.
Manes.10G07760(	1.0442528	0.64392	up	no	Aladin	pfam00400	WD40	WD domain	GO:0051028|	GO:0005783|	.	K14320
Manes.10G07790(	0.5930115	1.62E-05	down	no	Scopoletin gl	pfam00201	UDPGT	UDP-glucor	.	.	GO:0042802|id	.
Manes.10G07800(	1.8945951	9.23E-05	up	no	Scopoletin gl	pfam00201	UDPGT	UDP-glucor	.	.	GO:0042802|id	.
Manes.10G07850(	0.9921588	0.93613	down	no	Probable E3	pfam12906	RINGv	RING-varia	GO:0042335|	GO:0016021|	GO:0016874|lig	K10661
Manes.10G07870(	1.3031106	0.03195	up	no	40S ribosom	pfam00380	Ribosomal_S9	Ribosomal p	GO:0006412|	GO:0005840|	GO:0003735|st	.
Manes.10G07890(	Inf	0.9009	up	no	.	pfam05970	PIF1	PIF1-like he	.	.	.	.
Manes.10G07900(	1.0505891	0.67774	up	no	.	pfam13839	PC-Esterase	GDSL/SGN	.	.	.	.
Manes.10G07910(	0.6579377	0.00025	down	no	.	pfam01936	NYN	NYN domai	.	.	.	.
Manes.10G08000(	56.827926	0	up	yes	Zinc finger p	pfam13912	zf-C2H2_6	C2H2-type	GO:0035264|	GO:0005634|	GO:0046872|m	.

Manes.10G08010(	25.848336	8.87E-31	up	yes	.	pfam05637	Glyco_transf_34	galactosyl tr	.	.	.	.
Manes.10G08070(	0.8397561	0.1048	down	no	ABSCISIC A	pfam00170	bZIP_1	bZIP transcr	GO:0009738|	GO:0005634|	GO:0043565|se	K14432
Manes.10G08100(	0.8122192	0.22606	down	no	.	pfam10838	DUF2677	Protein of u	.	.	.	.
Manes.10G08140(	0.6564759	0.00033	down	no	.	pfam11600	CAF-1_p150	Chromatin a	.	.	.	.
Manes.10G08150(	0.6124942	0.00179	down	no	DNA polyme	pfam00817	IMS	impB/mucB	GO:0006974|	GO:0005829|	GO:0003684|da	K03511
Manes.10G08160(	0.4849516	2.02E-05	down	yes	.	pfam06505	XylR_N	Activator of	.	.	.	.
Manes.10G08230(	0.3686759	0.00444	down	yes	.	pfam11929	DUF3447	Domain of u	.	.	.	.
Manes.10G08240(	1.0369785	0.71129	up	no	Telomere rep	pfam00249	Myb_DNA-binding	Myb-like DI	GO:0006334|	GO:0000785|	GO:0003691|dc	K09422
Manes.10G08250(	Inf	0.9009	up	no	LIM domain-	pfam00412	LIM	LIM domain	GO:0051017|	GO:0005737|	GO:0051015|ac	.
Manes.10G08260(	0.9256093	0.47948	down	no	Zinc finger pi	pfam00097	zf-C3HC4	Zinc finger,	.	GO:0016021|	GO:0008270|zi	.
Manes.10G08410(	0.7091591	0.04078	down	no	.	pfam13041	PPR_2	PPR repeat	.	.	.	.
Manes.10G08450(	0.3286946	2.21E-06	down	yes	Cyclin-D1-1	pfam00134	Cyclin_N	Cyclin, N-te	GO:0007049|	GO:0005634|	.	K18810
Manes.10G08480(	1.0785377	0.425	up	no	Magnesium tı	pfam01544	CorA	CorA-like M	GO:0098655|	GO:0016021|	GO:0015095|ma	.
Manes.10G08530(	NA	NA	--	no	Cytosolic sul	pfam00685	Sulfotransfer_1	Sulfotransfe	GO:0019761|	GO:0005737|	GO:0047364|de	.
Manes.10G08540(	0.2287636	1.43E-19	down	yes	Cytosolic sul	pfam00685	Sulfotransfer_1	Sulfotransfe	GO:0019761|	GO:0005737|	GO:0047364|de	.
Manes.10G08570(	0.0518209	2.80E-07	down	yes	Cytosolic sul	pfam00685	Sulfotransfer_1	Sulfotransfe	GO:0019761|	GO:0005737|	GO:0047364|de	.
Manes.10G08590(	0.7062961	0.001	down	no	Serine carbox	pfam00450	Peptidase_S10	Serine carbo	.	GO:0005576|	GO:0004185|se	K16296
Manes.10G08600(	Inf	0.9009	up	no	.	pfam05729	NACHT	NACHT dor	.	.	.	.
Manes.10G08660(	3.5334934	0.00091	up	yes	Thymocyte n	pfam01878	EVE	EVE domain	.	GO:0005737|	.	.
Manes.10G08670(	19.229806	0.00082	up	yes	Agamous-like	pfam00319	SRF-TF	SRF-type tr	GO:0009960|	GO:0005634|	GO:0003677|DI	.
Manes.10G08680(	2.442372	1.40E-05	up	yes	.	pfam00095	WAP	WAP-type (	.	.	.	.
Manes.10G08700(	0.5820013	0.05777	down	no	Probable zinc	pfam02163	Peptidase_M50	Peptidase fa	GO:0009408|	GO:0031969|	GO:0008237|me	.
Manes.10G08720(	1.7154372	5.23E-08	up	no	Tubulin alpha	pfam03953	Tubulin_C	Tubulin C-te	GO:0007017|	GO:0005737|	GO:0005525|G'	.
Manes.10G08730(	1.2283501	0.05762	up	no	Tubulin alpha	pfam03953	Tubulin_C	Tubulin C-te	GO:0007017|	GO:0005737|	GO:0005525|G'	.
Manes.10G08770(	0.8968625	0.37475	down	no	.	pfam04109	APG9	Autophagy p	.	.	.	.
Manes.10G08800(	14.510111	1.52E-30	up	yes	.	pfam06219	DUF1005	Protein of u	.	.	.	.
Manes.10G08810(	8.1871688	2.68E-23	up	yes	.	pfam08211	dCMP_cyt_deam_	Cytidine anc	.	.	.	.
Manes.10G08870(	0.3989047	0.25401	down	no	Squalene mo	pfam08491	SE	Squalene ep	.	GO:0016021|	GO:0050660|fla	.
Manes.10G08960(	0.540247	0.00031	down	no	Protein trich	pfam13839	PC-Esterase	GDSL/SGN	.	GO:0005794|	.	.
Manes.10G09040(	0.3555492	0.00806	down	yes	Probable cati	pfam02386	TrkH	Cation trans	GO:0030003|	GO:0016021|	GO:0015079|pc	.
Manes.10G09130(	0.7269623	0.03421	down	no	Telomerase r	pfam12009	Telomerase_RBD	Telomerase	GO:0050000|	GO:0000781|	GO:0046872|me	K11126
Manes.10G09160(	7.1611481	3.28E-07	up	yes	Disease resist	pfam00931	NB-ARC	NB-ARC dc	GO:0006952|	.	GO:0043531|Al	.
Manes.10G09200(	3.9618862	7.94E-38	up	yes	Glutamyl-tRN	pfam05201	GlutR_N	Glutamyl-tR	GO:0015995|	GO:0009507|	GO:0008883|gl	K02492
Manes.10G09210(	0.7691995	0.17327	down	no	.	pfam10607	CLTH	CTLH/CRA	.	.	.	.
Manes.10G09220(	0.5975119	0.00067	down	no	.	pfam01061	ABC2_membrane	ABC-2 type	.	.	.	.
Manes.10G09350(	0.2378826	1.87E-13	down	yes	Oligopeptide	pfam03169	OPT	OPT oligop	GO:0015833|	GO:0016021|	.	.
Manes.10G09370(	0.9435425	0.96955	down	no	Agamous-like	pfam01486	K-box	K-box regio	GO:0006351|	GO:0005634|	GO:0003677|DI	K09264
Manes.10G09430(	12.870098	5.69E-39	up	yes	.	pfam06022	Cir_Bir_Yir	Plasmodium	.	.	.	.
Manes.10G09440(	3.9838899	4.11E-43	up	yes	Transcription	pfam00439	Bromodomain	Bromodoma	GO:0006355|	GO:0005634|	.	.
Manes.10G09470(	0.6157225	2.83E-06	down	no	Phosphoglyc	pfam00300	His_Phos_1	Histidine ph	.	GO:0005829|	.	.
Manes.10G09530(	27.098893	7.41E-25	up	yes	.	pfam03478	DUF295	Protein of u	.	.	.	.
Manes.10G09550(	113.14843	1.80E-10	up	yes	.	pfam03478	DUF295	Protein of u	.	.	.	.
Manes.10G09580(	0.5890115	8.25E-05	down	no	Methylmalon	pfam00171	Aldedh	Aldehyde de	GO:0006979|	GO:0005739|	GO:0004029|al	.
Manes.10G09590(	1.1235864	0.20822	up	no	Methylmalon	pfam00171	Aldedh	Aldehyde de	GO:0006979|	GO:0005739|	GO:0004029|al	.
Manes.10G09690(	1.2300432	0.07424	up	no	Putative glut	pfam00117	GATase	Glutamine a	GO:0006878|	GO:0005737|	GO:0016740|tra	.
Manes.10G09700(	0.9197282	0.57638	down	no	Anthocyanidi	pfam00201	UDPGT	UDP-glucor	GO:0009718|	.	GO:0047213|an	.
Manes.10G09730(	1.5042475	6.94E-05	up	no	Dynamin-rela	pfam01031	Dynamin_M	Dynamin cei	GO:0007049|	GO:0005739|	GO:0005525|G'	.
Manes.10G09740(	0.9461059	0.57053	down	no	GATA transc	pfam00320	GATA	GATA zinc	GO:0006351|	GO:0005634|	GO:0043565|se	.
Manes.10G09750(	0.7407293	0.00214	down	no	KH domain-c	pfam00013	KH_1	KH domain.	.	GO:0005634|	GO:0003723|RI	K13162
Manes.10G09760(	0.7833409	0.01673	down	no	Fructose-1,6-	pfam00316	FBPase	Fructose-1-(	GO:0019253|	GO:0009507|	GO:0042132|fru	.
Manes.10G09780(	0.5801884	0.00129	down	no	.	pfam12470	SUFU_C	Suppressor (	.	.	.	.
Manes.10G09790(	0.5186057	0.00201	down	no	.	pfam02519	Auxin_inducible	Auxin respo	.	.	.	.
Manes.10G09800(	0.0593423	2.57E-06	down	yes	Auxin-induce	pfam02519	Auxin_inducible	Auxin respo	GO:0009734|	.	.	K14488
Manes.10G09850(	0.488453	0.00015	down	yes	.	pfam01576	Myosin_tail_1	Myosin tail.	.	.	.	.
Manes.10G09860(	2.73393	4.77E-13	up	yes	Magnesium-c	pfam12689	Acid_PPase	Acid Phospl	GO:0030389|	GO:0070062|	GO:0046872|me	K17619
Manes.10G09870(	0.8544985	0.15088	down	no	Probable ubi	pfam02902	Peptidase_C48	Ulp1 protea	GO:0016926|	.	GO:0016929|SU	K08596
Manes.10G09890(	7.6006707	2.73E-26	up	yes	Guanosine nu	pfam00996	GDI	GDP dissoc	GO:0015031|	GO:0048046|	GO:0005096|G'	K17255
Manes.10G09900(	0.5853895	2.91E-07	down	no	MADS-box p	pfam00319	SRF-TF	SRF-type tr	GO:0007275|	GO:0005634|	GO:0003677|DI	.
Manes.10G09950(	0.764864	0.03585	down	no	.	pfam00226	DnaJ	DnaJ domai	.	.	.	.
Manes.10G09960(	0.8367566	0.10187	down	no	Beta carbonic	pfam00484	Pro_CA	Carbonic an	GO:0015976|	GO:0009507|	GO:0004089|ca	K01673
Manes.10G09990(	NA	NA	--	no	.	pfam06235	NAD4L	NADH dehy	.	.	.	.
Manes.10G10000(	NA	NA	--	no	.	pfam11667	DUF3267	Protein of u	.	.	.	.
Manes.10G10040(	Inf	0.35135	up	no	Agamous-like	pfam00319	SRF-TF	SRF-type tr	GO:0009960|	GO:0005634|	GO:0003677|DI	.
Manes.10G10140(	0.3943244	5.23E-05	down	yes	.	pfam02928	zf-C5HC2	C5HC2 zinc	.	.	.	.
Manes.10G10150(	0.3933421	0.01598	down	yes	.	pfam06232	ATS3	Embryo-spe	.	.	.	.
Manes.10G10160(	35.475722	2.60E-50	up	yes	Mitochondria	pfam00153	Mito_carr	Mitochondr	GO:0006835|	GO:0016021|	GO:0005310|di	K15104
Manes.10G10170(	40.89357	2.81E-20	up	yes	Mitochondria	pfam00153	Mito_carr	Mitochondr	GO:0006835|	GO:0016021|	GO:0005310|di	K15104
Manes.10G10180(	54.874277	1.48E-50	up	yes	Mitochondria	pfam00153	Mito_carr	Mitochondr	GO:0006835|	GO:0016021|	GO:0005310|di	K15104
Manes.10G10190(	56.746765	8.55E-07	up	yes	Mitochondria	pfam00153	Mito_carr	Mitochondr	GO:0006835|	GO:0016021|	GO:0005310|di	K15104
Manes.10G10200(	1.4104778	0.00186	up	no	.	pfam10319	7TM_GPCR_Srj	Serpentine t	.	.	.	.
Manes.10G10210(	1.4055724	0.00022	up	no	Transcription	pfam04437	RINT1_TIP1	RINT-1 / TI	GO:0006355|	GO:0005634|	GO:0003677|DI	.
Manes.10G10220(	0.4799794	1.47E-06	down	yes	Root phototr	pfam03000	NPH3	NPH3 famil	GO:0009638|	GO:0005886|	GO:0004871|sig	.
Manes.10G10230(	0.4298378	4.74E-13	down	yes	Serine/threon	pfam00069	Pkinase	Protein kina	GO:0009738|	GO:0005737|	GO:0005524|A'	.
Manes.10G10240(	0.6806541	0.00578	down	no	Pentatricopep	pfam13041	PPR_2	PPR repeat	.	GO:0005739|	.	.
Manes.10G10250(	5.1902372	0.00691	up	yes	Disease resist	pfam00931	NB-ARC	NB-ARC dc	GO:0009626|	GO:0012505|	GO:0043531|Al	K13457
Manes.10G10310(	7.9320561	6.65E-09	up	yes	TMV resistar	pfam00931	NB-ARC	NB-ARC dc	GO:0009626|	GO:0005737|	GO:0043531|Al	.
Manes.10G10440(	1.0665998	0.47092	up	no	Splicing facto	pfam08920	SF3b1	Splicing fact	GO:0006397|	GO:0005681|	.	K12828
Manes.10G10470(	521.97765	4.09E-61	up	yes	Feruloyl CoA	pfam14226	DIOX_N	non-haem di	GO:0009805|	.	GO:0051213|di	K06892
Manes.10G10480(	Inf	1.38E-39	up	yes	Feruloyl CoA	pfam14226	DIOX_N	non-haem di	GO:0009805|	.	GO:0051213|di	K06892
Manes.10G10490(	305.01472	8.05E-05	up	yes	Feruloyl CoA	pfam14226	DIOX_N	non-haem di	GO:0009805|	.	GO:0051213|di	K06892
Manes.10G10510(	0.3085539	1.83E-05	down	yes	ABC transpo	pfam01061	ABC2_membrane	ABC-2 type	GO:0010588|	GO:0009897|	GO:0005524|A'	.
Manes.10G10540(	0.0545475	4.89E-08	down	yes	Metacaspase-	pfam00656	Peptidase_C14	Caspase dor	GO:0006952|	.	GO:0004197|cy	.
Manes.10G10550(	0.7221654	0.00461	down	no	Metacaspase-	pfam00656	Peptidase_C14	Caspase dor	GO:0006952|	.	GO:0004197|cy	.
Manes.10G10560(	0.7577896	0.16583	down	no	.	pfam09912	DUF2141	Uncharacter	.	.	.	.
Manes.10G10600(	0.3413374	1.06E-09	down	yes	Zinc-finger h	pfam04770	ZF-HD_dimer	ZF-HD prot	GO:0006355|	GO:0005634|	GO:0003677|DI	.
Manes.10G10620(	0.8862532	0.24461	down	no	ATP-depende	pfam07724	AAA_2	AAA domai	GO:0006457|	.	GO:0005524|A'	K03544
Manes.10G10640(	0.6032529	9.39E-07	down	no	Survival of m	pfam06003	SMN	Survival mo	GO:0006397|	GO:0015030|	GO:0003723|RI	K12839
Manes.10G10690(	NA	NA	--	no	Ubiquitin car	pfam00917	MATH	MATH dom	GO:0006511|	GO:0005829|	GO:0008234|cy	.

Manes.10G10710(	4.2712925	1.01E-05	up	yes	.	pfam02936	COX4	Cytochrome	.	.	.	.
Manes.10G10720(	0.7127353	0.01199	down	no	.	pfam11906	DUF3426	Protein of u	.	.	.	.
Manes.10G10760(	0.4485095	0.00015	down	yes	.	pfam15272	BBP1_C	Spindle pole	.	.	.	.
Manes.10G10780(	20.112572	0.00079	up	yes	.	pfam05436	MF_alpha_N	Mating facto	.	.	.	.
Manes.10G10800(	0.6026901	0.00227	down	no	Cationic amir	pfam13520	AA_permease_2	Amino acid	GO:0003333|	GO:0016021|	GO:0015171|an	.
Manes.10G10810(	1.4874753	1.13E-05	up	no	Auxin respon	pfam06507	Auxin_resp	Auxin respo	GO:0009734|	GO:0005634|	GO:0003677|D	K14486
Manes.10G11010(	2.4299424	7.16E-24	up	yes	Chloride char	pfam00654	Voltage_CLC	Voltage gat	.	GO:0034707|	GO:0005247|vo	K05016
Manes.10G11020(	0.7226983	0.07194	down	no	.	pfam02485	Branch	Core-2/I-Br	.	.	.	.
Manes.10G11040(	1.4583802	0.00695	up	no	.	pfam05212	DUF707	Protein of u	.	.	.	.
Manes.10G11060(	0.3989163	3.90E-12	down	yes	Protein FAR1	pfam03101	FAR1	FAR1 DNA	GO:0006355|	GO:0005634|	GO:0008270|zi	.
Manes.10G11080(	2.3614037	4.80E-13	up	yes	Uncharacteriz	pfam03126	Plus-3	Plus-3 doma	GO:0006352|	GO:0005634|	GO:0003677|D	.
Manes.10G11090(	NA	NA	--	no	.	pfam00931	NB-ARC	NB-ARC dc	.	.	.	.
Manes.10G11170(	0.4914268	1.53E-06	down	yes	BAG family r	pfam02179	BAG	BAG domai	.	GO:0005739|	.	.
Manes.10G11240(	0.6025722	0.0001	down	no	Mitogen-activ	pfam00069	Pkinase	Protein kina	GO:0000186|	GO:0005634|	GO:0005524|A	K13414
Manes.10G11260(	0.6171588	0.35811	down	no	Mitogen-activ	pfam00069	Pkinase	Protein kina	GO:0000186|	GO:0005634|	GO:0005524|A	K13414
Manes.10G11350(	9.5832879	1.17E-63	up	yes	Glutathione t	pfam13417	GST_N_3	Glutathione	GO:0006952|	.	GO:0004364|gl	K00799
Manes.10G11400(	2.4951015	1.94E-17	up	yes	Putative dise	pfam00931	NB-ARC	NB-ARC dc	GO:0006952|	.	GO:0043531|A	.
Manes.10G11460(	NA	NA	--	no	Probable LRI	pfam00069	Pkinase	Protein kina	.	GO:0016021|	GO:0005524|A	.
Manes.10G11500(	0.7849662	0.07981	down	no	Non-lysosom	pfam04685	DUF608	Protein of u	GO:0008206|	GO:0005789|	GO:0008422|be	K17108
Manes.10G11540(	3.0659611	1.28E-36	up	yes	.	pfam03706	UPF0104	Uncharacter	.	.	.	.
Manes.10G11590(	1.6152886	4.84E-07	up	no	Ras-related p	pfam00071	Ras	Ras family.	GO:0000911|	GO:0009504|	GO:0005525|G	K07904
Manes.10G11600(	1.5298255	0.00018	up	no	Elongation fa	pfam00647	EF1G	Elongation f	.	.	GO:0003746|tr	.
Manes.10G11610(	0.3927432	0.00259	down	yes	.	pfam01984	dsDNA_bind	Double-stra	.	.	.	.
Manes.10G11620(	2.675503	1.84E-13	up	yes	.	pfam14897	EpsG	EpsG family	.	.	.	.
Manes.10G11640(	1.035813	0.70857	up	no	Regulator of	pfam03467	Smg4_UPF3	Smg-4/UPF	GO:0042742|	GO:0005737|	GO:0000166|nu	K14328
Manes.10G11650(	7.662494	4.41E-07	up	yes	.	pfam04987	PigN	Phosphatidy	.	.	.	.
Manes.10G11660(	0.6012664	5.52E-07	down	no	Shaggy-relate	pfam00069	Pkinase	Protein kina	.	GO:0005886|	GO:0005524|A	.
Manes.10G11670(	61.698822	2.61E-10	up	yes	.	pfam07133	Merozoite_SPAM	Merozoite s	.	.	.	.
Manes.10G11710(	8.8092161	#######	up	yes	.	pfam00564	PB1	PB1 domain	.	.	.	.
Manes.10G11730(	0.781627	0.02588	down	no	Stromal 70 k	pfam00012	HSP70	Hsp70 prote	GO:0006457|	GO:0009570|	GO:0005524|A	.
Manes.10G11740(	1.1016286	0.34949	up	no	MACPF dom	pfam01823	MACPF	MAC/Perfo	GO:0009626|	GO:0005886|	.	.
Manes.10G11770(	0.4517844	2.43E-05	down	yes	.	pfam13041	PPR_2	PPR repeat	.	.	.	.
Manes.10G11790(	16.700455	0.03368	up	no	.	pfam14631	FancD2	Fanconi ana	.	.	.	.
Manes.10G11860(	Inf	0.9009	up	no	.	pfam15213	CDRT4	CMT1A duj	.	.	.	.
Manes.10G11890(	0.6992388	0.00296	down	no	Probable sod	pfam01758	SBF	Sodium Bile	GO:0019761|	GO:0009941|	GO:0050833|py	K03453
Manes.10G11900(	6.0454801	1.76E-15	up	yes	.	pfam00903	Glyoxalase	Glyoxalase/	.	.	.	.
Manes.10G11960(	4.1085394	3.26E-33	up	yes	Floral homeo	pfam03006	HlyIII	Haemolysin	GO:0009873|	GO:0005634|	GO:0003677|D	K09284
Manes.10G11970(	0.872174	0.15796	down	no	ATP-depende	pfam04408	HA2	Helicase ass	.	GO:0016282|	GO:0005524|A	K18995
Manes.10G11990(	1.0323603	0.73871	up	no	NifU-like pro	pfam01106	NifU	NifU-like dc	GO:0009658|	GO:0009507|	GO:0051537|2	.
Manes.10G12010(	1.0288393	0.78514	up	no	ATP phospho	pfam01634	HisG	ATP phospl	GO:0000105|	GO:0009507|	GO:0003879|A	K00765
Manes.10G12020(	1.7250303	2.14E-07	up	no	Probable mag	pfam05653	Mg_trans_NIPA	Magnesium	GO:0015693|	GO:0005769|	GO:0015095|m	.
Manes.10G12040(	0.6669221	0.00014	down	no	Transcription	pfam03634	TCP	TCP family	GO:0006355|	GO:0005634|	GO:0001047|co	.
Manes.10G12180(	42.940586	#######	up	yes	Anthocyanidi	pfam00201	UDPGT	UDP-glucor	GO:0009718|	.	GO:0047213|an	.
Manes.10G12190(	0.8761812	0.18867	down	no	UDP-glucose	pfam06427	UDP-g_GGTase	UDP-glucos	GO:0006486|	GO:0005788|	GO:0003980|U	K11718
Manes.10G12200(	0.9725661	0.78507	down	no	Potassium ch	pfam00520	Ion_trans	Ion transpor	GO:0071805|	GO:0005789|	GO:0042802|id	.
Manes.10G12220(	1.5087747	0.01361	up	no	.	pfam11595	DUF3245	Protein of u	.	.	.	.
Manes.10G12230(	0.3779229	0.00292	down	yes	.	pfam09726	Macoilin	Transmemb	.	.	.	.
Manes.10G12240(	5.8376963	1.90E-07	up	yes	.	pfam07649	C1_3	C1-like dom	.	.	.	.
Manes.10G12250(	2.2532226	9.87E-13	up	yes	Polyubiquitin	pfam00240	ubiquitin	Ubiquitin fa	.	GO:0005737|	.	.
Manes.10G12260(	4.8499071	2.53E-25	up	yes	Polyubiquitin	pfam00240	ubiquitin	Ubiquitin fa	GO:0006511|	GO:0005634|	.	K08770
Manes.10G12270(	14.389837	2.45E-06	up	yes	.	pfam02362	B3	B3 DNA bir	.	.	.	.
Manes.10G12350(	2.8655354	6.37E-05	up	yes	Phospholipas	pfam12357	PLD_C	Phospholipa	GO:0016042|	GO:0016020|	GO:0005509|ca	K01115
Manes.10G12360(	1.4918266	1.76E-05	up	no	Cleavage and	pfam11718	CPSF73-100_C	Pre-mRNA	GO:0006397|	GO:0005634|	GO:0004518|nu	K14403
Manes.10G12370(	0.4857612	0.00159	down	yes	Putative pent	pfam14432	DYW_deaminase	DYW family	.	GO:0005739|	.	.
Manes.10G12380(	0.8998596	0.27038	down	no	.	pfam00170	bZIP_1	bZIP transcı	.	.	.	.
Manes.10G12510(	0.495726	0.00276	down	yes	.	pfam13041	PPR_2	PPR repeat	.	.	.	.
Manes.10G12520(	2.7234045	8.77E-21	up	yes	TMV resistar	pfam01582	TIR	TIR domain	GO:0009626|	GO:0005737|	GO:0043531|A	.
Manes.10G12530(	3.5978117	0.00096	up	yes	.	pfam08022	FAD_binding_8	FAD-bindin	.	.	.	.
Manes.10G12580(	Inf	0.17619	up	no	DnaJ homolo	pfam01556	CTDII	DnaJ C tern	GO:0006457|	.	.	K09519
Manes.10G12590(	0.9498418	0.60803	down	no	.	pfam09746	Membralin	tumor-assoc	.	.	.	.
Manes.10G12610(	0.4291803	0.00055	down	yes	60S ribosoma	pfam01294	Ribosomal_L13e	Ribosomal p	GO:0006412|	GO:0005840|	GO:0003735|st	.
Manes.10G12650(	Inf	0.1623	up	no	.	pfam12796	Ank_2	Ankyrin rep	.	.	.	.
Manes.10G12660(	2.5894002	5.09E-06	up	yes	Putative dise	pfam00931	NB-ARC	NB-ARC dc	GO:0006952|	GO:0005739|	GO:0043531|A	.
Manes.10G12690(	2.8818893	4.58E-13	up	yes	TMV resistar	pfam01582	TIR	TIR domain	GO:0009626|	GO:0005737|	GO:0043531|A	.
Manes.10G12710(	5.2388294	1.01E-16	up	yes	Probable WR	pfam13855	LRR_8	Leucine rich	GO:0006952|	GO:0009941|	GO:0043531|A	.
Manes.10G12720(	0.4479601	0.00323	down	yes	.	pfam13041	PPR_2	PPR repeat	.	.	.	.
Manes.10G12840(	0.599793	0.01538	down	no	Legumin type	pfam00190	Cupin_1	Cupin. This	.	.	GO:0045735|nu	.
Manes.10G12850(	2.1009758	3.01E-05	up	yes	Legumin type	pfam00190	Cupin_1	Cupin. This	.	.	GO:0045735|nu	.
Manes.10G12900(	0.8275368	0.08728	down	no	Phosphatidyli	pfam01504	PIP5K	Phosphatidy	.	.	GO:0016308|1-	.
Manes.10G12970(	0.8166046	0.15345	down	no	.	.	.	.	.	.	.	.
Manes.10G12980(	0.5701295	4.03E-05	down	no	Rhomboid pr	pfam01694	Rhomboid	Rhomboid f	.	GO:0016021|	GO:0004252|se	K19225
Manes.10G13040(	0.4302342	7.10E-05	down	yes	Elongation of	pfam01151	ELO	GNS1/SUR	GO:0071215|	GO:0016021|	GO:0009922|fa	.
Manes.10G13050(	0.5217663	0.00018	down	no	Putative elon	pfam01151	ELO	GNS1/SUR	GO:0006633|	GO:0030176|	GO:0016740|tr	.
Manes.10G13130(	0.5462852	0.00637	down	no	Heavy metal-	pfam00403	HMA	Heavy-meta	GO:0010286|	GO:0031965|	GO:0046872|m	.
Manes.10G13140(	0.4940143	2.18E-07	down	yes	Sterol 3-beta	pfam03033	Glyco_transf_28	Glycosyltrar	GO:0009813|	GO:0005774|	GO:0016906|st	K05841
Manes.10G13160(	0.5834769	1.69E-06	down	no	Protein SUPI	pfam03468	XS	XS domain.	GO:0031047|	GO:0048471|	.	.
Manes.10G13200(	0.98632	0.94877	down	no	Partner of Y1	pfam09282	Mago-bind	Mago bindir	GO:0000184|	GO:0005737|	GO:0043022|ril	K14294
Manes.10G13210(	14.196146	0.00655	up	yes	Pectinesteras	pfam01095	Pectinesterase	Pectinestera	GO:0042545|	GO:0005618|	GO:0045330|as	K01051
Manes.10G13230(	2.8225302	9.04E-29	up	yes	.	pfam07800	DUF1644	Protein of u	.	.	.	.
Manes.10G13260(	1.8681921	1.24E-09	up	no	Mitogen-activ	pfam00069	Pkinase	Protein kina	GO:0000186|	GO:0005634|	GO:0005524|A	K13414
Manes.10G13270(	3.6138531	4.26E-46	up	yes	.	pfam03999	MAP65_ASE1	Microtubule	.	.	.	.
Manes.10G13290(	0.8109	0.03019	down	no	.	pfam04696	Pinin_SDK_memA	pinin/SDK/r	.	.	.	.
Manes.10G13300(	1.5894124	2.31E-05	up	no	.	pfam03168	LEA_2	Late embryc	.	.	.	.
Manes.10G13310(	1.904466	7.64E-08	up	no	Mitogen-activ	pfam00069	Pkinase	Protein kina	GO:0000186|	GO:0005634|	GO:0005524|A	K13414
Manes.10G13320(	23.159421	2.14E-12	up	yes	.	pfam12937	F-box-like	F-box-like.	.	.	.	.
Manes.10G13450(	0.3000198	1.65E-07	down	yes	Acyl-CoA--st	pfam13813	MBOAT_2	Membrane b	GO:0016127|	GO:0016021|	GO:0047196|lo	.
Manes.10G13460(	3.0189786	3.63E-15	up	yes	Acyl-CoA--st	pfam13813	MBOAT_2	Membrane b	GO:0016127|	GO:0016021|	GO:0047196|lo	.
Manes.10G13470(	2.4079304	3.42E-05	up	yes	Putative rece	pfam00069	Pkinase	Protein kina	GO:0048544|	GO:0016021|	GO:0005524|A	.

Manes.10G13530(	25.657975	1.03E-13	up	yes	Arogenate de	pfam00800	PDT	Prephenate (	GO:0009094|l	GO:0009507|	GO:0016597|an	K05359
Manes.10G13560(	2.1284955	3.00E-07	up	yes	Serine carbo›	pfam00450	Peptidase_S10	Serine carbo	.	GO:0005576|	GO:0004185|se	K16297
Manes.10G13570(	2.4242808	7.57E-24	up	yes	Putative disea	pfam00931	NB-ARC	NB-ARC do	GO:0006952|	.	GO:0043531|Al	.
Manes.10G13610(	0.5004064	0.0007	down	no	CTL-like pro	pfam04515	Choline_transpo	Plasma-men	.	GO:0016021|	.	.
Manes.10G13630(	0.6695659	8.23E-05	down	no	50S ribosoma	pfam00687	Ribosomal_L1	Ribosomal p	GO:0006412|	GO:0009507|	GO:0019843|rR	K02863
Manes.10G13670(	0.2933304	0.00101	down	yes	Receptor-like	pfam13855	LRR_8	Leucine rich	GO:0009755|l	GO:0016021|	GO:0004672|pr	.
Manes.10G13680(	0.2716954	5.16E-13	down	yes	LRR recepto	pfam13855	LRR_8	Leucine rich	GO:0009793|	GO:0016021|	GO:0005524|A′	.
Manes.10G13740(	0.3041674	7.37E-05	down	yes	LRR recepto	pfam13855	LRR_8	Leucine rich	GO:0009793|	GO:0016021|	GO:0005524|A′	.
Manes.10G13790(	0.9641984	0.81423	down	no	Putative disea	pfam00931	NB-ARC	NB-ARC do	GO:0006952|	.	GO:0043531|Al	.
Manes.10G13800(	0.1954277	0.10875	down	no	.	pfam04654	DUF599	Protein of u	.	.	.	.
Manes.10G13880(	1.3844954	0.00061	up	no	Tetraketide a	pfam01370	Epimerase	NAD depen	GO:0009555|	GO:0005783|	GO:0050662|co	.
Manes.10G13890(	0.5148194	1.08E-05	down	no	Tetraketide a	pfam01370	Epimerase	NAD depen	GO:0009555|	GO:0005783|	GO:0050662|co	.
Manes.10G13910(	1.3999463	0.00022	up	no	Alpha-N-acet	pfam05089	NAGLU	Alpha-N-ac	GO:0005975|	GO:0070062|	GO:0004561|al	K01205
Manes.10G13950(	0.898752	0.30587	down	no	.	pfam04862	DUF642	Protein of u	.	.	.	.
Manes.10G14020(	1.4457622	0.0027	up	no	Protein OBE	pfam07227	DUF1423	Protein of u	GO:0001708|	GO:0005634|	GO:0046872|m	.
Manes.10G14030(	1.1220827	0.2076	up	no	Protein OBE	pfam07227	DUF1423	Protein of u	GO:0001708|	GO:0005634|	GO:0046872|m	.
Manes.10G14080(	1.33176	0.00171	up	no	.	pfam06485	DUF1092	Protein of u	.	.	.	.
Manes.10G14100(	1.0310965	0.7559	up	no	.	pfam14958	DUF4506	Domain of u	.	.	.	.
Manes.10G14110(	0.4246581	3.42E-10	down	yes	Actin-related	pfam00022	Actin	Actin.	GO:0009653|	GO:0005737|	GO:0005200|str	K16615
Manes.10G14130(	1.1093395	0.27754	up	no	Carotenoid 9	pfam03055	RPE65	Retinal pign	.	.	GO:0051213|di	.
Manes.10G14140(	0.5411159	2.05E-06	down	no	Nuclear trans	pfam02045	CBFB_NFYA	CCAAT-bin	GO:0009793|	GO:0016602|	GO:0003677|D	K08064
Manes.10G14170(	2.7641985	1.88E-24	up	yes	Putative disea	pfam00931	NB-ARC	NB-ARC do	GO:0006952|	.	GO:0043531|Al	.
Manes.10G14180(	1.7784627	1.72E-09	up	no	Putative disea	pfam00931	NB-ARC	NB-ARC do	GO:0006952|	.	GO:0043531|Al	.
Manes.10G14210(	6.7220292	1.78E-10	up	yes	.	pfam12036	DUF3522	Protein of u	.	.	.	.
Manes.10G14230(	3.9554878	1.37E-14	up	yes	.	pfam05112	Baculo_p47	Baculovirus	.	.	.	.
Manes.10G14240(	0.2166504	1.17E-12	down	yes	Putative disea	pfam00931	NB-ARC	NB-ARC do	GO:0006952|	.	GO:0043531|Al	.
Manes.10G14250(	0.1274531	0.00329	down	yes	.	pfam02383	Syja_N	SacI homolo	.	.	.	.
Manes.10G14260(	1.7089183	4.02E-07	up	no	Cellulose syn	pfam03552	Cellulose_synt	Cellulose sy	GO:0071555|	GO:0005768|	GO:0016760|ce	K10999
Manes.10G14270(	1.3868297	0.00093	up	no	ESF1 homolo	pfam08159	NUC153	NUC153 do	GO:0006355|	GO:0005615|	GO:0044822|pc	.
Manes.10G14300(	0.2912888	6.49E-05	down	yes	MATE efflux	pfam01554	MatE	MatE. The I	GO:0006879|	GO:0016021|	GO:0015297|an	.
Manes.10G14360(	4.2786569	4.97E-23	up	yes	.	pfam01704	UDPGP	UTP--gluco	.	.	.	.
Manes.10G14440(	0.8736371	0.60131	down	no	Zinc finger pı	pfam13465	zf-H2C2_2	Zinc-finger (	GO:1990058|	GO:0005634|	GO:0046872|m	.
Manes.10G14460(	0.5581772	5.61E-09	down	no	Thylakoid lur	pfam01789	PsbP	PsbP. This f	GO:0015979|	GO:0009543|	GO:0005509|ca	.
Manes.10G14480(	10.093567	0.00238	up	yes	.	pfam11122	Spore-coat_CotD	Inner spore	.	.	.	.
Manes.10G14490(	6.7522211	2.77E-33	up	yes	.	pfam02705	K_trans	K+ potassiu	.	.	.	.
Manes.10G14520(	0.1484256	1.09E-10	down	yes	Malonyl-coer	pfam02458	Transferase	Transferase	GO:0009698|	.	GO:0033809|an	.
Manes.10G14560(	2.1441033	4.43E-18	up	yes	Polyadenylate	pfam00076	RRM_1	RNA recogı	.	GO:0005737|	GO:0000166|nu	K14396
Manes.10G14570(	1.6992258	2.54E-09	up	no	Zinc finger A	pfam01428	zf-AN1	AN1-like Zi	.	.	GO:0003677|D	.
Manes.10G14580(	1.865226	1.28E-06	up	no	U6 snRNA-a	pfam01423	LSM	LSM domai	GO:0043928|	GO:0005829|	GO:0017070|U	K12626
Manes.10G14620(	1.8051983	0.00112	up	no	Protein gamn	pfam08573	SAE2	DNA repair	GO:0006974|	GO:0005634|	GO:0000014|sir	K13096
Manes.10G14640(	2.5961959	1.16E-20	up	yes	Transcription	pfam05734	DUF832	Herpesvirus	GO:0048573|	GO:0005634|	GO:0003677|D	.
Manes.10G14650(	8.2142863	0.14131	up	no	Ras-related p	pfam00071	Ras	Ras family.	GO:0006886|	GO:0005768|	GO:0005525|G	K07904
Manes.10G14660(	0.4003461	4.61E-09	down	yes	Beta-amyrin	pfam00067	p450	Cytochrome	.	GO:0016021|	GO:0020037|he	.
Manes.10G14670(	4.4710461	0.00273	up	yes	Cytochrome	pfam00067	p450	Cytochrome	GO:0051554|	GO:0005783|	GO:0020037|he	.
Manes.10G14750(	5.4459491	7.83E-12	up	yes	Beta-glucosic	pfam00232	Glyco_hydro_1	Glycosyl hy	GO:0005975|	.	GO:0008422|be	K01188
Manes.10G14760(	0.3989804	4.78E-12	down	yes	Beta-glucosic	pfam00232	Glyco_hydro_1	Glycosyl hy	GO:0005975|	.	GO:0008422|be	K01188
Manes.10G14790(	0.9108706	0.40619	down	no	Chaperone pı	pfam00226	DnaJ	DnaJ domai	GO:0006457|	GO:0005739|	GO:0005524|A′	.
Manes.10G14800(	0.6411152	0.00943	down	no	Multiple C2 a	pfam08372	PRT_C	Plant phosp	GO:0019722|	GO:0016021|	GO:0005509|ca	.
Manes.10G14840(	Inf	3.13E-66	up	yes	.	pfam08657	DASH_Spc34	DASH comp	.	.	.	.
Manes.10G14850(	458.00965	4.34E-87	up	yes	.	pfam13346	ABC2_membrane_	ABC-2 fami	.	.	.	.
Manes.10G14860(	117.14526	3.43E-73	up	yes	.	pfam10327	7TM_GPCR_Sri	Serpentine t	.	.	.	.
Manes.10G14870(	1.9646029	8.13E-08	up	no	Siroheme syn	pfam00590	TP_methylase	Tetrapyrrol	GO:0009236|	.	GO:0051287|N	K02302
Manes.10G14920(	1.8477456	3.39E-08	up	no	Uncharacteriz	pfam10250	O-FucT	GDP-fucose	.	GO:0005768|	GO:0016757|tra	.
Manes.10G14960(	0.4923023	0.00016	down	yes	Beta-glucosic	pfam00232	Glyco_hydro_1	Glycosyl hy	GO:0005975|	GO:0005794|	GO:0008422|be	K01188
Manes.10G14990(	0.4854711	3.93E-07	down	yes	Beta-glucosic	pfam00232	Glyco_hydro_1	Glycosyl hy	GO:0005975|	GO:0005794|	GO:0008422|be	K01188
Manes.10G15020(	0.3758672	0.00919	down	yes	Beta-glucosic	pfam00232	Glyco_hydro_1	Glycosyl hy	GO:0005975|	GO:0048046|	GO:0008422|be	K01188
Manes.10G15030(	NA	NA	--	no	.	pfam01227	GTP_cyclohydroI	GTP cycloh	.	.	.	.
Manes.10G15040(	1.4737382	0.00115	up	no	Box C/D sno	pfam04438	zf-HIT	HIT zinc fin	GO:0042254|	.	GO:0046872|m	.
Manes.10G15070(	0.5661151	0.00144	down	no	Zeaxanthin ep	pfam01494	FAD_binding_3	FAD binding	GO:0009688|	GO:0031969|	GO:0052663|an	K09838
Manes.10G15100(	0.7972173	0.06046	down	no	Protein N-ter	pfam14736	N_Asn_amidohyd	Protein N-te	GO:0008344|	GO:0005737|	GO:0008418|pr	K14662
Manes.10G15110(	1.5493549	0.00033	up	no	.	pfam15306	LIN37	LIN37. LIN	.	.	.	.
Manes.10G15120(	3.5350696	2.15E-09	up	yes	Homeobox-le	pfam02183	HALZ	Homeobox a	GO:0006351|	GO:0005634|	GO:0043565|se	K09338
Manes.10G15160(	0.9440603	0.70113	down	no	Callose synth	pfam02364	Glucan_synthase	1,3-beta-glu	GO:0006075|	GO:0000148|	GO:0003843|1,	.
Manes.10G15170(	0.9990058	0.99689	down	no	Callose synth	pfam14288	FKS1_dom1	1,3-beta-glu	GO:0006075|	GO:0000148|	GO:0003843|1,	K11000
Manes.10G15180(	1.4976439	0.00037	up	no	.	pfam00213	OSCP	ATP syntha	.	.	.	.
Manes.10G15200(	1.7267472	4.33E-09	up	no	Citrate syntha	pfam00285	Citrate_synt	Citrate synt	GO:0044262|	GO:0005759|	GO:0004108|cit	.
Manes.10G15230(	10.61779	1.43E-16	up	yes	.	pfam13261	DUF4052	Protein of u	.	.	.	.
Manes.10G15250(	0.4154727	0.00429	down	yes	.	pfam02714	DUF221	Domain of u	.	.	.	.
Manes.10G15280(	0.9547008	1	down	no	.	pfam15395	DUF4617	Domain of u	.	.	.	.
Manes.10G15290(	0.1995909	3.91E-05	down	yes	Protein YLS	pfam14368	LTP_2	Probable lip	GO:0006869|	GO:0031225|	GO:0008289|lip	.
Manes.10G15320(	0.3064847	0.00085	down	yes	.	pfam06075	DUF936	Plant protei	.	.	.	.
Manes.10G15350(	8.6766829	4.05E-96	up	yes	.	pfam06101	DUF946	Plant protei	.	.	.	.
Manes.10G15360(	2.3906468	0.00108	up	yes	Beta-hexosar	pfam00728	Glyco_hydro_20	Glycosyl hy	GO:0005975|	GO:0005886|	GO:0004563|be	K12373
Manes.10G15370(	0.6750565	0.00026	down	no	.	pfam03080	DUF239	Domain of u	.	.	.	.
Manes.11G00030(	0.5704693	0.00481	down	no	.	pfam03577	Peptidase_C69	Peptidase fa	.	.	.	.
Manes.11G00040(	1.0781547	0.40366	up	no	Sphingoid lo	pfam00781	DAGK_cat	Diacylglyce	GO:0030148|	.	GO:0005524|A′	.
Manes.11G00060(	1.363003	0.00059	up	no	Protease 2	pfam02897	Peptidase_S9_N	Prolyl oligo	.	.	GO:0004252|se	.
Manes.11G00080(	0.8014763	0.4419	down	no	Probable prot	pfam03302	VSP	Giardia vari	GO:0034969|	GO:0005829|	GO:0008469|his	K11437
Manes.11G00110(	0.7203284	0.38259	down	no	.	pfam13456	RVT_3	Reverse trar	.	.	.	.
Manes.11G00120(	1.5600699	1.05E-06	up	no	Calumenin-B	pfam13499	EF-hand_7	EF-hand do	.	GO:0005576|	GO:0005509|ca	.
Manes.11G00140(	4.583583	6.42E-06	up	yes	.	pfam02893	GRAM	GRAM dom	.	.	.	.
Manes.11G00150(	Inf	5.11E-14	up	yes	.	pfam10998	DUF2838	Protein of u	.	.	.	.
Manes.11G00170(	6.5234807	0.00184	up	yes	.	pfam00112	Peptidase_C1	Papain famil	.	.	.	.
Manes.11G00180(	0.727727	0.00229	down	no	Probable glyc	pfam07479	NAD_Gly3P_dh_(	NAD-depen	GO:0005975|	GO:0005829|	GO:0004367|gl	K00006
Manes.11G00220(	1.2818718	0.01226	up	no	Glycosyltrans	pfam13704	Glyco_tranf_2_4	Glycosyl tra	GO:0009738|	GO:0005737|	GO:0005543|ph	.
Manes.11G00230(	0.4698956	3.20E-12	down	yes	Ubiquitin car	pfam00443	UCH	Ubiquitin ca	GO:0043161|	GO:0005634|	GO:0004197|cy	K11841
Manes.11G00250(	0.3785628	0.00123	down	yes	Scarecrow-lil	pfam03514	GRAS	GRAS doma	GO:0006355|	GO:0005634|	GO:0003700|tra	.
Manes.11G00260(	0.9928776	1	down	no	Protein tricho	pfam13839	PC-Esterase	GDSL/SGN	.	GO:0016021|	.	.

Manes.11G00270(	1.3894376	0.01464	up	no	.	pfam09811	Yae1_N	Essential pr	.	.	.	.
Manes.11G00300(	0.5554954	0.02829	down	no	.	pfam08268	FBA_3	F-box assoc	.	.	.	.
Manes.11G00360(	0.598298	0.022	down	no	U-box domai	pfam01602	Adaptin_N	Adaptin N t	.	GO:0005886|	GO:0016874|lig	.
Manes.11G00380(	0.7247222	0.0199	down	no	Annexin D4	pfam00191	Annexin	Annexin. Th	GO:0009737|	GO:0009506|	GO:0005509|ca	.
Manes.11G00410(	0.5807802	1.30E-05	down	no	E3 ubiquitin-	pfam03145	Sina	Seven in abs	GO:0007275|	GO:0005737|	GO:0016874|lig	K04506
Manes.11G00440(	0.8814714	0.72612	down	no	Probable AD	pfam01412	ArfGap	Putative GT	.	.	GO:0005096|G	K12486
Manes.11G00450(	0.9658581	0.76608	down	no	Uncharacteri	pfam00462	Glutaredoxin	Glutaredoxi	GO:0045454|	GO:0005886|	GO:0009055|ele	.
Manes.11G00540(	7.1265286	1.21E-14	up	yes	.	pfam07422	s48_45	Sexual stage	.	.	.	.
Manes.11G00550(	2.3787822	5.18E-05	up	yes	Phosphatidyli	pfam01504	PIP5K	Phosphatidy	GO:0072583|	GO:0016324|	GO:0016308|1-	K00889
Manes.11G00610(	1.6496191	1.50E-05	up	no	U-box domai	pfam00514	Arm	Armadillo/b	.	.	GO:0016874|lig	.
Manes.11G00620(	0.8285278	0.07229	down	no	Trihelix trans	pfam13837	Myb_DNA-bind_4	Myb/SANT-	GO:0009793|	GO:0005634|	GO:0003677|D	.
Manes.11G00680(	23.483806	5.55E-57	up	yes	.	pfam14798	Ca_hom_mod	Calcium hor	.	.	.	.
Manes.11G00690(	0.5954297	0.00987	down	no	Nudix hydrol	pfam01353	GFP	Green fluor	.	GO:0005739|	GO:0016787|hy	.
Manes.11G00700(	58.922128	8.96E-36	up	yes	Sigma factor	pfam05678	VQ	VQ motif. T	GO:0071482|	GO:0009507|	.	.
Manes.11G00720(	1.0003285	1	up	no	NADH dehyc	pfam13233	Complex1_LYR_2	Complex1_L	GO:0055114|	GO:0016020|	.	K03950
Manes.11G00730(	10.692154	7.90E-38	up	yes	Cation/H(+) a	pfam00999	Na_H_Exchanger	Sodium/hyd	GO:0006813|	GO:0016021|	GO:0005451|m	.
Manes.11G00770(	4.7375452	2.27E-10	up	yes	.	pfam09991	DUF2232	Predicted m	.	.	.	.
Manes.11G00780(	7.7871365	3.87E-09	up	yes	Protein MIZU	pfam04759	DUF617	Protein of u	GO:0009658|	GO:0032541|	.	.
Manes.11G00810(	1.2793474	0.02524	up	no	Probable cho	pfam01633	Choline_kinase	Choline/etha	.	.	GO:0005524|A	K14156
Manes.11G00820(	1.2530108	0.08247	up	no	.	pfam03154	Atrophin-1	Atrophin-1 f	.	.	.	.
Manes.11G00870(	1.3937694	0.56449	up	no	Cysteine prot	pfam00031	Cystatin	Cystatin dor	GO:0006952|	GO:0005576|	GO:0004869|cy	.
Manes.11G00880(	0.4760989	3.44E-10	down	yes	Dr1-associate	pfam00808	CBFD_NFYB_HM	Histone-like	GO:0000122|	GO:0005634|	GO:0043565|se	.
Manes.11G00890(	NA	NA	--	no	Zinc finger p	pfam13912	zf-C2H2_6	C2H2-type	GO:0006355|	GO:0005737|	GO:0046872|m	.
Manes.11G00930(	2.2803568	1.39E-10	up	yes	Protein ELC	pfam05743	UEV	UEV domai	GO:0051301|	GO:0005769|	GO:0043130|ub	K12183
Manes.11G00950(	0.7535836	0.04158	down	no	.	pfam12776	Myb_DNA-bind_3	Myb/SANT-	.	.	.	.
Manes.11G00970(	0.4153421	7.05E-13	down	yes	Germinal-cen	pfam03399	SAC3_GANP	SAC3/GAN	GO:0002376|	GO:0005737|	.	.
Manes.11G00980(	0.720032	0.02468	down	no	.	pfam02628	COX15-CtaA	Cytochrome	.	.	.	.
Manes.11G00990(	5.1049287	4.75E-68	up	yes	Transcription	pfam00249	Myb_DNA-binding	Myb-like D	GO:0030154|	GO:0005634|	GO:0003677|D	K09422
Manes.11G01000(	0.9825576	0.86032	down	no	Exportin-7	pfam03810	IBN_N	Importin-be	GO:0006886|	GO:0005737|	.	K18460
Manes.11G01020(	0.7327719	0.00888	down	no	CO(2)-respo	pfam00082	Peptidase_S8	Subtilase fa	GO:2000122|	GO:0048046|	GO:0004252|se	.
Manes.11G01060(	0.7586428	0.01486	down	no	Uncharacteri	pfam00149	Metallophos	Calcineurin-	GO:0030036|	GO:0005783|	GO:0016787|hy	.
Manes.11G01110(	0.2412723	6.60E-13	down	yes	Lipase	pfam01764	Lipase_3	Lipase (clas	GO:0016042|	.	GO:0004806|tri	.
Manes.11G01120(	1.0988512	0.74407	up	no	Lipase	pfam01764	Lipase_3	Lipase (clas	GO:0016042|	.	GO:0004806|tri	.
Manes.11G01130(	1.7336051	6.57E-06	up	no	Protein STRU	pfam00069	Pkinase	Protein kina	.	GO:0016021|	GO:0005524|A	.
Manes.11G01140(	1.0151874	0.84555	up	no	26S protease	pfam00004	AAA	ATPase fam	GO:0030163|	GO:0005737|	GO:0005524|A	K03061
Manes.11G01170(	0.7216018	0.00246	down	no	.	pfam10250	O-FucT	GDP-fucose	.	.	.	.
Manes.11G01220(	0.3404998	9.08E-07	down	yes	.	pfam13041	PPR_2	PPR repeat	.	.	.	.
Manes.11G01230(	0.9475333	0.6114	down	no	Probable ubi	pfam00179	UQ_con	Ubiquitin-co	GO:0030433|	GO:0005737|	GO:0005524|A	K04554
Manes.11G01250(	0.4600627	1.47E-12	down	yes	Pectinesteras	pfam01095	Pectinesterase	Pectinestera	GO:0042545|	GO:0005618|	GO:0045330|as	.
Manes.11G01260(	1.7935266	1.69E-10	up	no	Pectinesteras	pfam01095	Pectinesterase	Pectinestera	GO:0042545|	GO:0005618|	GO:0045330|as	.
Manes.11G01270(	1.7135807	1.37E-09	up	no	Alanine amin	pfam00155	Aminotran_1_2	Aminotransf	GO:0009058|	GO:0005739|	GO:0004021|L-	K00814
Manes.11G01290(	0.4730083	1.68E-05	down	yes	.	pfam03763	Remorin_C	Remorin, C-	.	.	.	.
Manes.11G01310(	0.7162647	0.00124	down	no	1-phosphatid	pfam01504	PIP5K	Phosphatidy	GO:0010256|	GO:0005768|	GO:0000285|1-	K00921
Manes.11G01400(	#NAME?	0.64948	down	no	GDSL estera	pfam00657	Lipase_GDSL	GDSL-like L	GO:0042742|	GO:0005615|	GO:0016298|lip	.
Manes.11G01450(	0.6713394	0.00134	down	no	Phosphoinosi	pfam02383	Syja_N	SacI homol	GO:0036092|	GO:0005774|	GO:0042578|ph	.
Manes.11G01470(	#NAME?	0.30365	down	no	40S ribosom	pfam00318	Ribosomal_S2	Ribosomal p	GO:0000028|	GO:0022627|	GO:0003735|str	.
Manes.11G01480(	0.170967	0.00235	down	yes	17.4 kDa clas	pfam00011	HSP20	Hsp20/alpha	GO:0009408|	GO:0005737|	.	K13993
Manes.11G01490(	6.1333342	1.01E-20	up	yes	.	pfam02519	Auxin_inducible	Auxin respo	.	.	.	.
Manes.11G01510(	0.7317235	0.01572	down	no	Probable com	pfam08547	CIA30	Complex I i	.	GO:0005739|	.	.
Manes.11G01520(	32.024755	#######	up	yes	DnaJ homolo	pfam00226	DnaJ	DnaJ domai	.	GO:0005813|	.	K09509
Manes.11G01540(	0.8684314	0.25885	down	no	Trihelix trans	pfam13837	Myb_DNA-bind_4	Myb/SANT-	GO:0009793|	GO:0005634|	GO:0003677|D	.
Manes.11G01560(	Inf	2.02E-22	up	yes	ABC transpo	pfam00664	ABC_membrane	ABC transp	GO:0010541|	GO:0016021|	GO:0005524|A	K05658
Manes.11G01570(	0.9170262	0.6452	down	no	.	pfam06075	DUF936	Plant protei	.	.	.	.
Manes.11G01610(	1.3806067	0.00178	up	no	Peroxisomal	pfam01070	FMN_dh	FMN-depen	GO:0042742|	GO:0005777|	GO:0010181|F	K11517
Manes.11G01620(	1.7391526	5.00E-06	up	no	Peroxisomal	pfam01070	FMN_dh	FMN-depen	GO:0042742|	GO:0005777|	GO:0010181|F	K11517
Manes.11G01670(	13.503395	5.17E-31	up	yes	Protein TIFY	pfam09425	CCT_2	Divergent C	GO:0042742|	GO:0005634|	.	K13464
Manes.11G01700(	1.1937881	0.09284	up	no	Transmembra	pfam06814	Lung_7-TM_R	Lung seven	.	GO:0016021|	.	.
Manes.11G01720(	0.2311194	1.31E-11	down	yes	.	pfam00902	TatC	Sec-indepen	.	.	.	.
Manes.11G01780(	5.8650627	4.33E-18	up	yes	Aldehyde del	pfam00171	Aldedh	Aldehyde de	.	.	GO:0004029|al	.
Manes.11G01790(	2.1818553	1.14E-09	up	yes	Aldehyde del	pfam00171	Aldedh	Aldehyde de	GO:0006081|	GO:0005829|	GO:0004029|al	K14085
Manes.11G01830(	NA	NA	--	no	Ubiquitin	pfam00240	ubiquitin	Ubiquitin fa	.	GO:0005737|	.	.
Manes.11G01840(	2.6328716	8.57E-14	up	yes	Aldehyde del	pfam00171	Aldedh	Aldehyde de	GO:0006081|	GO:0005829|	GO:0004029|al	K14085
Manes.11G01880(	Inf	1.35E-19	up	yes	Uncharacteri	pfam00078	RVT_1	Reverse tra	.	GO:0005739|	.	.
Manes.11G01930(	3.3695066	8.11E-05	up	yes	Putative rece	pfam07714	Pkinase_Tyr	Protein tyro	.	.	GO:0005524|A	.
Manes.11G01940(	1.8611867	1.06E-11	up	no	Exportin-T	pfam08389	Xpo1	Exportin 1-l	GO:0009908|	GO:0005829|	GO:0015932|nu	K14288
Manes.11G01950(	0.9766018	0.90837	down	no	Probable GT	pfam13328	HD_4	HD domain.	GO:0015969|	GO:0009507|	GO:0005524|A	.
Manes.11G01980(	1.5763499	1.55E-05	up	no	Baculoviral I	pfam12483	GIDE	E3 Ubiquiti	GO:1990001|	GO:0005737|	GO:0043027|cy	K16060
Manes.11G01990(	15.465249	5.50E-15	up	yes	Nodulation-s	pfam03514	GRAS	GRAS doma	GO:0009877|	GO:0005634|	GO:0003700|tra	.
Manes.11G02000(	0.7149322	0.0008	down	no	tRNA(His) g	pfam04446	Thg1	tRNAHis gu	GO:0006400|	GO:0005654|	GO:0005525|G	K10761
Manes.11G02020(	0.3644754	0.00058	down	yes	Growth-regu	pfam08879	WRC	WRC. The	GO:0048366|	GO:0005634|	GO:0005524|A	.
Manes.11G02030(	0.2001083	5.55E-10	down	yes	AP2-like ethy	pfam00847	AP2	AP2 domair	GO:0009873|	GO:0005634|	GO:0003677|D	K09285
Manes.11G02050(	0.9353547	0.71842	down	no	Ubiquitin-like	pfam04110	APG12	Ubiquitin-lil	GO:0000045|	GO:0005737|	.	K08336
Manes.11G02100(	0.731031	0.00128	down	no	.	pfam04182	B-block_TFIIIC	B-block bin	.	.	.	.
Manes.11G02140(	0.7342346	0.00734	down	no	.	pfam03169	OPT	OPT oligop	.	.	.	.
Manes.11G02150(	0.7784639	0.01521	down	no	Telomere rep	pfam00249	Myb_DNA-binding	Myb-like D	GO:0006355|	GO:0005634|	GO:0042162|tel	.
Manes.11G02160(	Inf	5.77E-07	up	yes	RING-H2 fin	pfam13639	zf-RING_2	Ring finger	GO:0016567|	GO:0016021|	GO:0008270|zi	.
Manes.11G02210(	0.8392752	0.13966	down	no	Phospholipid	pfam02450	LCAT	Lecithin:cho	GO:0006071|	GO:0005783|	GO:0008374|O	K00679
Manes.11G02270(	3.6989358	1.02E-20	up	yes	Threonine sy	pfam00291	PALP	Pyridoxal-pl	GO:0009088|	GO:0009507|	GO:0030170|py	K01733
Manes.11G02290(	6.5565165	4.33E-97	up	yes	.	pfam05553	DUF761	Cotton fibre	.	.	.	.
Manes.11G02300(	0.5793997	0.00045	down	no	.	pfam01578	Cytochrom_C_asm	Cytochrome	.	.	.	.
Manes.11G02330(	0.3599173	8.96E-22	down	yes	Protein phos	pfam00481	PP2C	Protein pho	GO:0009738|	GO:0005737|	GO:0046872|m	K14497
Manes.11G02390(	1.6368061	4.46E-08	up	no	Casein kinase	pfam00069	Pkinase	Protein kina	GO:0006897|	GO:0005737|	GO:0005524|A	K02218
Manes.11G02410(	NA	NA	--	no	.	pfam10577	UPF0560	Uncharacter	.	.	.	.
Manes.11G02420(	0.3703926	0.00117	down	yes	Coatomer sul	pfam01217	Clat_adaptor_s	Clathrin ada	GO:0015031|	GO:0030663|	.	.
Manes.11G02440(	0.5586429	1.60E-06	down	no	.	pfam13967	RSN1_TM	Late exocyt	.	.	.	.
Manes.11G02460(	0.5432544	0.00282	down	no	Protein IQ-D	pfam01137	RTC	RNA 3'-term	.	GO:0005634|	.	.
Manes.11G02540(	10.329065	#######	up	yes	Beta-fructofu	pfam00251	Glyco_hydro_32N	Glycosyl hy	GO:0005975|	GO:0048046|	GO:0004564|be	K01193
Manes.11G02550(	0.7546946	0.05213	down	no	Putative hydr	pfam12706	Lactamase_B_2	Beta-lactam	.	GO:0005829|	GO:0016787|hy	.

Manes.11G02590(	2.2813069	2.08E-16	up	yes	Mediator of I	pfam07544	Med9	RNA polym	GO:0006355|r	GO:0016592|	.	.
Manes.11G02600(	119.26829	1.01E-17	up	yes	.	pfam03195	DUF260	Protein of u	.	.	.	.
Manes.11G02630(	0.2878906	5.95E-18	down	yes	Receptor-like	pfam07714	Pkinase_Tyr	Protein tyro	GO:0030154|c	GO:0016021|	GO:0005524|A'	.
Manes.11G02690(	2.2339214	3.05E-15	up	yes	Beta-galactos	pfam01301	Glyco_hydro_35	Glycosyl hy	GO:0005975|c	GO:0048046|	GO:0004565|be	.
Manes.11G02710(	1.9627085	1.48E-13	up	no	.	pfam02919	Topoisom_I_N	Eukaryotic I	.	.	.	.
Manes.11G02730(	1.0023536	0.96711	up	no	Protein FLX-	pfam01576	Myosin_tail_1	Myosin tail.	GO:0030154|c	.	.	.
Manes.11G02740(	#NAME?	0.03394	down	no	.	pfam11014	DUF2852	Protein of u	.	.	.	.
Manes.11G02750(	0.3466836	3.58E-16	down	yes	Calcineurin E	pfam13499	EF-hand_7	EF-hand dor	GO:0042538|l	GO:0005737|	GO:0005509|ca	.
Manes.11G02770(	1.0541859	0.64204	up	no	PRA1 family	pfam03208	PRA1	PRA1 famil	GO:0016192|v	GO:0005783|	.	.
Manes.11G02780(	0.4605846	0.0031	down	yes	.	pfam07760	DUF1616	Protein of u	.	.	.	.
Manes.11G02810(	118.93713	#######	up	yes	Inorganic pyr	pfam06888	Put_Phosphatase	Putative Pho	GO:0016311|c	.	GO:0004427|in	K13248
Manes.11G02860(	1.5631477	0.00098	up	no	Dof zinc fing	pfam02701	zf-Dof	Dof domain	GO:0006355|r	GO:0005634|	GO:0046872|m	.
Manes.11G02870(	1.2760344	0.00751	up	no	Ubiquitin car	pfam00443	UCH	Ubiquitin ca	GO:0043161|p	GO:0009506|	GO:0005516|ca	.
Manes.11G02910(	51.347257	4.82E-09	up	yes	.	pfam11955	PORR	Plant organe	.	.	.	.
Manes.11G02920(	0.6824173	0.00268	down	no	Anoctamin-li	pfam04547	Anoctamin	Calcium-act	.	GO:0034707|	GO:0005254|ch	.
Manes.11G02940(	1.6229075	1.54E-05	up	no	.	pfam00646	F-box	F-box doma	.	.	.	.
Manes.11G02950(	0.76464	0.01395	down	no	IAA-amino a	pfam01546	Peptidase_M20	Peptidase fa	GO:0009611|r	GO:0005783|	GO:0010179|IA	K14664
Manes.11G02960(	0.7714195	0.04443	down	no	Uncharacteriz	pfam05958	tRNA_U5-meth_tı	tRNA (Urac	GO:0006396|l	.	GO:0051539|4 i	K03215
Manes.11G02970(	3.4212375	0.00512	up	yes	Transcription	pfam00010	HLH	Helix-loop-l	GO:0006355|r	GO:0005634|	GO:0003677|D	.
Manes.11G03250(	5.8557811	3.07E-17	up	yes	.	pfam07343	DUF1475	Protein of u	.	.	.	.
Manes.11G03270(	0.4370511	0.10446	down	no	.	pfam13178	DUF4005	Protein of u	.	.	.	.
Manes.11G03320(	1.2817966	0.00995	up	no	LysM domair	pfam07714	Pkinase_Tyr	Protein tyro	GO:0050832|c	GO:0016021|	GO:0005524|A'	.
Manes.11G03350(	1.2899408	0.00829	up	no	MMS19 nucl	pfam14500	MMS19_N	Dos2-intera	GO:0006974|c	GO:0097361|	.	K15075
Manes.11G03380(	0.2403862	0.00298	down	yes	Histidine-con	pfam01627	Hpt	Hpt domain	GO:0009736|c	GO:0005737|	GO:0004871|sig	.
Manes.11G03390(	2.9583629	4.12E-33	up	yes	Transcription	pfam00249	Myb_DNA-binding	Myb-like DI	GO:0048262|r	GO:0005634|	GO:0003677|D	.
Manes.11G03400(	0.9743701	0.79425	down	no	NADP-deper	pfam03949	Malic_M	Malic enzyn	GO:0006633|f	GO:0009507|	GO:0050897|co	K00029
Manes.11G03420(	15.637162	2.65E-13	up	yes	ABC transpo	pfam01061	ABC2_membrane	ABC-2 type	GO:0070574|c	GO:0009507|	GO:0005524|A'	.
Manes.11G03430(	0.4975546	0.00038	down	yes	1-aminocyclo	pfam03171	2OG-FeII_Oxy	2OG-Fe(II)	GO:0009693|c	.	GO:0009815|1-	.
Manes.11G03450(	1.5409843	0.02177	up	no	.	pfam05553	DUF761	Cotton fibre	.	.	.	.
Manes.11G03500(	1.5374803	0.00038	up	no	Nuclear pore	pfam05172	Nup35_RRM	Nup53/35/4	GO:0051028|r	GO:0031965|	.	K14313
Manes.11G03530(	11.425144	6.35E-10	up	yes	Early nodulin	pfam02298	Cu_bind_like	Plastocyanin	.	GO:0031225|	GO:0009055|el	.
Manes.11G03560(	0.3604676	0.00015	down	yes	Dehydration-	pfam00847	AP2	AP2 domair	GO:0009873|c	GO:0005634|	GO:0043565|se	K09286
Manes.11G03610(	0.4954679	0.00016	down	yes	.	pfam07766	LETM1	LETM1-like	.	.	.	.
Manes.11G03640(	1.1618738	0.10263	up	no	.	pfam00111	Fer2	2Fe-2S iron	.	.	.	.
Manes.11G03660(	0.7847347	0.01267	down	no	Activating sig	pfam10469	AKAP7_NLS	AKAP7 2'5'	GO:0006355|r	GO:0005737|	GO:0003723|R	K18666
Manes.11G03670(	0.6646515	0.00043	down	no	.	pfam00559	Vif	Retroviral V	.	.	.	.
Manes.11G03680(	1.1125049	0.25964	up	no	Auxin-respor	pfam02309	AUX_IAA	AUX/IAA f	GO:0009734|	GO:0005634|	GO:0003700|tra	K14484
Manes.11G03740(	1.1879049	0.07608	up	no	Malonate--C	pfam00501	AMP-binding	AMP-bindin	GO:0006631|f	GO:0005829|	GO:0090409|ma	K18660
Manes.11G03760(	0.8900163	0.22725	down	no	Golgin candi	pfam12325	TMF_TATA_bd	TATA elem	.	GO:0005794|	.	.
Manes.11G03780(	1.6974092	1.49E-06	up	no	Homeobox-le	pfam01852	START	START dor	GO:0006355|r	GO:0005634|	GO:0008289|lip	.
Manes.11G03840(	0.4621711	0.00418	down	yes	Dynein light	pfam01221	Dynein_light	Dynein light	GO:0006915|a	GO:0005868|	GO:0019899|en	K10418
Manes.11G04260(	12.603134	1.12E-10	up	yes	Proline-rich r	pfam00069	Pkinase	Protein kina	GO:0009755|l	GO:0016021|	GO:0005524|A'	.
Manes.11G04280(	1.9264979	0.00128	up	no	Receptor-like	pfam00069	Pkinase	Protein kina	GO:0009755|l	GO:0005737|	GO:0005524|A'	.
Manes.11G04290(	2.4727734	3.47E-16	up	yes	Proline-rich r	pfam00069	Pkinase	Protein kina	.	GO:0016021|	GO:0005524|A'	.
Manes.11G04340(	7.768102	1.80E-10	up	yes	Proline-rich r	pfam00069	Pkinase	Protein kina	GO:0009755|l	GO:0016021|	GO:0005524|A'	.
Manes.11G04400(	1.2109483	0.03406	up	no	Probable 28S	pfam01189	Nol1_Nop2_Fmu	NOL1/NOP	GO:0008284|p	GO:0005730|	GO:0044822|pc	K14835
Manes.11G04430(	0.8070038	0.03195	down	no	.	pfam02194	PXA	PXA domai	.	.	.	.
Manes.11G04450(	1.9838923	6.14E-08	up	no	Probable ATI	pfam06733	DEAD_2	DEAD_2. T	GO:0006139|r	GO:0005737|	GO:0051539|4 i	K11273
Manes.11G04460(	0.3934886	6.06E-06	down	yes	.	pfam01529	zf-DHHC	DHHC palm	.	.	.	.
Manes.11G04480(	0.310815	8.55E-06	down	yes	.	pfam14897	EpsG	EpsG family	.	.	.	.
Manes.11G04520(	0.8421038	0.38592	down	no	Mitochondria	pfam00153	Mito_carr	Mitochondr	GO:1903826|a	GO:0016021|	GO:0015181|ar	K15109
Manes.11G04550(	6.105648	0.00168	up	yes	G-type lectin	pfam07714	Pkinase_Tyr	Protein tyro	.	GO:0016021|	GO:0005524|A'	.
Manes.11G04560(	5.3312858	0.02116	up	yes	G-type lectin	pfam07714	Pkinase_Tyr	Protein tyro	.	GO:0016021|	GO:0005524|A'	.
Manes.11G04590(	0.5258491	0.00022	down	no	Methyltransfe	pfam10294	Methyltransf_16	Putative me	.	GO:0005634|	GO:0008168|m	.
Manes.11G04670(	1.0813802	0.38741	up	no	.	pfam04782	DUF632	Protein of u	.	.	.	.
Manes.11G04720(	3.8047735	0.02168	up	yes	.	pfam12786	GBV-C_env	GB virus C	.	.	.	.
Manes.11G04770(	0.5402435	5.81E-07	down	no	.	pfam04570	DUF581	Protein of u	.	.	.	.
Manes.11G04780(	0.7689132	0.00716	down	no	MAR-binding	pfam01576	Myosin_tail_1	Myosin tail.	.	GO:0016363|	GO:0003677|D	.
Manes.11G04790(	0.6910668	0.0198	down	no	Protein SYS1	pfam09801	SYS1	Integral mer	GO:0006895|c	GO:0030173|	.	.
Manes.11G04860(	0.8399395	0.07287	down	no	.	pfam14259	RRM_6	RNA recogr	.	.	.	.
Manes.11G04890(	0.1978109	0.02404	down	yes	.	pfam04116	FA_hydroxylase	Fatty acid hy	.	.	.	.
Manes.11G04920(	0.5984698	0.00538	down	no	Bifunctional	pfam12697	Abhydrolase_6	Alpha/beta l	GO:0019439|a	GO:0005777|	GO:0033885|10	K08726
Manes.11G04930(	1.2823824	0.00594	up	no	Plastidic ATI	pfam03219	TLC	TLC ATP/A	.	GO:0031969|	GO:0005524|A'	K03301
Manes.11G04940(	1.0358084	0.82819	up	no	Coleoptile ph	pfam03000	NPH3	NPH3 famil	GO:0043481|a	.	.	.
Manes.11G04990(	0.7138463	0.05965	down	no	ABC transpo	pfam01061	ABC2_membrane	ABC-2 type	GO:0080168|a	GO:0016021|	GO:0005524|A'	.
Manes.11G05010(	0.6388773	0.07266	down	no	Pleiotropic di	pfam01061	ABC2_membrane	ABC-2 type	GO:0006810|t	GO:0016021|	GO:0005524|A'	.
Manes.11G05050(	0.4107408	6.60E-06	down	yes	Gibberellin 3-	pfam03171	2OG-FeII_Oxy	2OG-Fe(II)	GO:0009686|g	.	GO:0016707|gil	.
Manes.11G05060(	7.2407754	0.00018	up	yes	.	pfam00008	EGF	EGF-like do	.	.	.	.
Manes.11G05080(	0.9090811	0.31685	down	no	Aspartate am	pfam00155	Aminotran_1_2	Aminotransl	GO:0009058|l	GO:0005737|	GO:0004069|L-	K00812
Manes.11G05090(	0.1913196	0.10668	down	no	.	pfam04695	Pex14_N	Peroxisomal	.	.	.	.
Manes.11G05120(	3.9198529	1.10E-16	up	yes	Auxin-respor	pfam02309	AUX_IAA	AUX/IAA f	GO:0009734|	GO:0005634|	GO:0003700|tra	K14484
Manes.11G05130(	6.5079007	5.73E-43	up	yes	Auxin-induce	pfam02309	AUX_IAA	AUX/IAA f	GO:0009734|	GO:0005634|	.	K14484
Manes.11G05190(	0.2719468	3.29E-05	down	yes	DNA-repair p	pfam00533	BRCT	BRCA1 C T	GO:0080111|l	GO:0005634|	GO:0010385|dc	K10803
Manes.11G05230(	0.5186099	0.0001	down	no	Acyl-protein	pfam02230	Abhydrolase_2	Phospholipa	GO:0006631|f	GO:0005829|	GO:0052689|ca	K06130
Manes.11G05240(	1.7238549	1.01E-08	up	no	Periodic tryp	pfam04003	Utp12	Dip2/Utp12	GO:0000462|r	GO:0005737|	GO:0044822|pc	K14558
Manes.11G05260(	12.574803	1.09E-12	up	yes	.	pfam02987	LEA_4	Late embryo	.	.	.	.
Manes.11G05280(	1.0773582	0.45176	up	no	Biotin carbox	pfam00364	Biotin_lipoyl	Biotin-requi	GO:0006633|f	GO:0009317|	GO:0003989|ac	K02160
Manes.11G05290(	2.3227011	0.00484	up	yes	Zinc finger C	pfam00642	zf-CCCH	Zinc finger (	GO:0006355|r	.	GO:0003677|D	.
Manes.11G05300(	3.6600578	3.23E-18	up	yes	Probable dise	pfam00931	NB-ARC	NB-ARC dc	GO:0006952|c	.	GO:0043531|A	.
Manes.11G05310(	7.9068644	5.57E-58	up	yes	Probable dise	pfam00931	NB-ARC	NB-ARC dc	GO:0006952|c	.	GO:0043531|A	.
Manes.11G05350(	0.7378304	0.01286	down	no	DNA replicat	pfam05916	Sld5	GINS comp	GO:0006260|l	GO:0005737|	.	.
Manes.11G05380(	0.709163	0.00287	down	no	Peptide defor	pfam01327	Pep_deformylase	Polypeptide	GO:0043686|c	GO:0009507|	GO:0005506|irc	K01462
Manes.11G05400(	1.5686658	0.32648	up	no	.	pfam05553	DUF761	Cotton fibre	.	.	.	.
Manes.11G05410(	4.200768	9.97E-05	up	yes	.	pfam00903	Glyoxalase	Glyoxalase/I	.	.	.	.
Manes.11G05480(	54.59728	2.95E-09	up	yes	Probable poly	pfam00295	Glyco_hydro_28	Glycosyl hy	GO:0005975|c	GO:0005576|	GO:0004650|pc	.
Manes.11G05510(	1.0112889	0.90741	up	no	Phospholipas	pfam12357	PLD_C	Phospholipa	GO:0016042|l	GO:0005783|	GO:0005509|ca	.
Manes.11G05570(	0.7183472	0.35327	down	no	.	pfam08733	PalH	PalH/RIM2	.	.	.	.
Manes.11G05580(	1.0689595	0.53035	up	no	.	pfam13259	DUF4050	Protein of u	.	.	.	.

Manes.11G05610(	2.4804328	1.96E-06	up	yes	.	pfam13414	TPR_11	TPR repeat.	.	.	.	.
Manes.11G05620(	0.6895764	0.00016	down	no	Methyl-CpG-	pfam01429	MBD	Methyl-CpG	GO:0006355|r	GO:0005829|	GO:0008327|m	.
Manes.11G05640(	1.9313485	0.6353	up	no	Cucumber pe	pfam02298	Cu_bind_like	Plastocyanin	GO:0055114|(	.	GO:0009055|ele	.
Manes.11G05660(	0.2671507	7.03E-06	down	yes	.	pfam14432	DYW_deaminase	DYW family	.	.	.	.
Manes.11G05690(	0.624954	0.0037	down	no	Phosphatidylo	pfam14378	PAP2_3	PAP2 super	GO:0006657|(	GO:0016021|	GO:0004142|dia	.
Manes.11G05730(	1.193671	0.04755	up	no	PTI1-like tyro	pfam00069	Pkinase	Protein kina	.	GO:0005886|	GO:0005524|A'	.
Manes.11G05740(	1.2766836	0.02237	up	no	Pre-mRNA-p	pfam08083	PROCN	PROCN (NI	GO:0000398|r	GO:0071013|	GO:0070530|K(	K12856
Manes.11G05750(	3.7154792	4.69E-40	up	yes	Adenine phos	pfam00156	Pribosyltran	Phosphoribo	GO:0006168|a	GO:0005737|	GO:0003999|ad	K00759
Manes.11G05760(	56.613019	4.43E-44	up	yes	.	pfam13994	PgaD	PgaD-like p	.	.	.	.
Manes.11G05770(	0.7567877	0.54507	down	no	.	pfam12745	HGTP_anticodon2	Anticodon b	.	.	.	.
Manes.11G05800(	1.1757305	0.07897	up	no	Chaperone pr	pfam00226	DnaJ	DnaJ domai	GO:0006260|I	GO:0005737|	GO:0005524|A'	K03686
Manes.11G05820(	1.0754891	0.43601	up	no	Signal recogr	pfam00448	SRP54	SRP54-type	GO:0006614|S	GO:0005786|	GO:0008312|7S	K03106
Manes.11G05830(	0.7850468	0.11126	down	no	.	pfam04005	Hus1	Hus1-like pr	.	.	.	.
Manes.11G05880(	1.0053027	0.96556	up	no	Clustered mit	pfam12807	eIF3_p135	Translation	GO:0048312|i	GO:0005737|	.	K03255
Manes.11G05890(	Inf	0.02254	up	yes	Putative rece	pfam00069	Pkinase	Protein kina	.	GO:0016021|	GO:0005524|A'	.
Manes.11G05990(	0.1598651	0.00169	down	yes	.	pfam08263	LRRNT_2	Leucine rich	.	.	.	.
Manes.11G06030(	0.257116	3.76E-23	down	yes	Probable CCI	pfam04857	CAF1	CAF1 family	GO:0006355|r	GO:0005737|	GO:0046872|m	K12581
Manes.11G06050(	5.1858716	0.02986	up	no	.	pfam04576	Zein-binding	Zein-binding	.	.	.	.
Manes.11G06060(	1.0380404	0.6894	up	no	Glycerol kina	pfam00370	FGGY_N	FGGY famil	GO:0042742|(	GO:0005737|	GO:0005524|A'	.
Manes.11G06070(	1.0729303	0.4741	up	no	Pyrophospha	pfam03030	H_PPase	Inorganic H	GO:0015992|	GO:0016021|	GO:0009678|hy	.
Manes.11G06100(	3.5792831	7.48E-38	up	yes	TMV resistar	pfam01582	TIR	TIR domain	GO:0009626|	GO:0005737|	GO:0043531|Al	.
Manes.11G06130(	3.9686794	2.64E-48	up	yes	Probable sod	pfam01490	Aa_trans	Transmemb	GO:0003333|a	GO:0005887|	GO:0015171|an	K14993
Manes.11G06160(	1.8203721	0.00325	up	no	.	pfam06813	Nodulin-like	Nodulin-like	.	.	.	.
Manes.11G06170(	Inf	0.17669	up	no	.	pfam11146	DUF2905	Protein of u	.	.	.	.
Manes.11G06180(	3.494421	4.29E-18	up	yes	.	pfam03251	Tymo_45kd_70kd	Tymovirus 4	.	.	.	.
Manes.11G06190(	1.1209894	0.20886	up	no	Mediator of I	pfam15449	Retinal	Retinal prote	GO:0006355|r	GO:0016592|	.	K14972
Manes.11G06290(	2.3719203	0.03197	up	no	Ethylene-resp	pfam00847	AP2	AP2 domair	GO:0009873|(	GO:0005634|	GO:0003677|DI	.
Manes.11G06320(	1.2320397	0.04697	up	no	.	pfam08610	Pex16	Peroxisomal	.	.	.	.
Manes.11G06360(	20.777098	1.45E-43	up	yes	Pleiotropic dr	pfam01061	ABC2_membrane	ABC-2 type	GO:0006810|t	GO:0016021|	GO:0005524|A'	.
Manes.11G06400(	0.7131846	0.01582	down	no	Endoribonucl	pfam14709	DND1_DSRM	double stran	GO:0007267|(	GO:0005737|	GO:0005524|A'	K11592
Manes.11G06430(	2.1934604	8.33E-16	up	yes	DCN1-like pr	pfam03556	Cullin_binding	Cullin bindir	.	GO:0005634|	.	K17824
Manes.11G06440(	0.4459709	0.00394	down	yes	DeSI-like pro	pfam05903	Peptidase_C97	PPPDE puta	.	.	GO:0008233|pe	.
Manes.11G06470(	0.1904798	2.35E-09	down	yes	Zinc finger pr	pfam13912	zf-C2H2_6	C2H2-type 2	GO:0009640|p	GO:0005737|	GO:0046872|m	.
Manes.11G06540(	0.5930398	5.91E-06	down	no	.	pfam08737	Rgp1	Rgp1. Rgp1	.	.	.	.
Manes.11G06550(	1.0994947	0.38106	up	no	Cinnamoyl-C	pfam01370	Epimerase	NAD depen	GO:0007623|(	GO:0005829|	GO:0016621|cin	.
Manes.11G06570(	NA	NA	--	no	Gibberellin 20	pfam03171	2OG-FeII_Oxy	2OG-Fe(II)	GO:0009908|f	.	GO:0045544|gil	K05282
Manes.11G06590(	0.5753166	0.00035	down	no	Probable diph	pfam00590	TP_methylase	Tetrapyrrole	GO:0017183|p	.	GO:0004164|dip	K00586
Manes.11G06600(	NA	NA	--	no	Gibberellin 20	pfam03171	2OG-FeII_Oxy	2OG-Fe(II)	GO:0009908|f	.	GO:0045544|gil	K05282
Manes.11G06610(	1.3899516	0.08234	up	no	Metal transpo	pfam01566	Nramp	Natural resis	GO:0070574|(	GO:0012505|	GO:0015086|ca	.
Manes.11G06620(	1.0893427	0.66501	up	no	Metal transpo	pfam01566	Nramp	Natural resis	GO:0070574|(	GO:0016021|	GO:0015086|ca	.
Manes.11G06640(	0.0752747	2.76E-35	down	yes	CBL-interact	pfam00069	Pkinase	Protein kina	GO:0050832|(	.	GO:0005524|A'	.
Manes.11G06650(	92.361948	1.84E-31	up	yes	Probable WR	pfam03106	WRKY	WRKY DN	GO:0042742|(	GO:0005634|	GO:0043565|se	.
Manes.11G06680(	0.7873737	0.01365	down	no	ABC transpo	pfam00664	ABC_membrane	ABC transp	.	GO:0016021|	GO:0005524|A'	.
Manes.11G06700(	0.4748148	7.60E-07	down	yes	Putative rece	pfam00069	Pkinase	Protein kina	.	GO:0016021|	GO:0005524|A'	.
Manes.11G06720(	1.3148657	0.0167	up	no	Lamin-like pr	pfam02298	Cu_bind_like	Plastocyanin	.	GO:0031225|	GO:0009055|ele	.
Manes.11G06740(	0.4708662	0.00119	down	yes	Putative leuc	pfam00069	Pkinase	Protein kina	.	GO:0016021|	GO:0005524|A'	.
Manes.11G06810(	0.3224223	0.02396	down	yes	Auxin-induce	pfam02519	Auxin_inducible	Auxin respo	GO:0009734|a	.	.	.
Manes.11G06850(	13.585535	3.76E-37	up	yes	Chaperone pr	pfam00226	DnaJ	DnaJ domai	GO:0042026|p	GO:0009507|	GO:0051087|ch	.
Manes.11G06860(	11.256598	#######	up	yes	Magnesium tr	pfam01544	CorA	CorA-like M	GO:0098655|(	GO:0016021|	GO:0015095|ma	K16075
Manes.11G06870(	0.5652189	0.00074	down	no	.	pfam09330	Lact-deh-memb	D-lactate de	.	.	.	.
Manes.11G06880(	1.5359225	0.00117	up	no	Trafficking pr	pfam04628	Sedlin_N	Sedlin, N-te	GO:0006888|I	GO:0005783|	GO:0008134|tra	.
Manes.11G06890(	NA	NA	--	no	.	pfam10323	7TM_GPCR_Srv	Serpentine t	.	.	.	.
Manes.11G06910(	1.0806799	0.62048	up	no	.	pfam15449	Retinal	Retinal prote	.	.	.	.
Manes.11G06920(	0.6995598	0.00147	down	no	.	pfam13868	Trichoplein	tumor suppr	.	.	.	.
Manes.11G06930(	77.382011	4.67E-12	up	yes	.	pfam14897	EpsG	EpsG family	.	.	.	.
Manes.11G06940(	2.4747071	8.60E-25	up	yes	.	pfam13183	Fer4_8	4Fe-4S dicl	.	.	.	.
Manes.11G06960(	0.508995	0.33139	down	no	Wall-associat	pfam00069	Pkinase	Protein kina	.	GO:0016021|	GO:0005524|A'	.
Manes.11G06970(	0.9715007	0.7877	down	no	Nudix hydrol	pfam00293	NUDIX	NUDIX dor	.	GO:0005829|	GO:0016787|hy	.
Manes.11G06980(	1.290706	0.02041	up	no	.	pfam10292	7TM_GPCR_Srab	Serpentine t	.	.	.	.
Manes.11G07070(	1.4090888	0.00014	up	no	Serine incorp	pfam03348	Serinc	Serine incor	GO:0006658|p	GO:0000139|	GO:0015194|L-	.
Manes.11G07080(	1.4518625	0.00019	up	no	Ubiquitin rec	pfam09280	XPC-binding	XPC-binding	GO:0006289|r	GO:0005737|	GO:0003684|da	.
Manes.11G07090(	0.5525033	8.76E-05	down	no	Serine/threon	pfam00069	Pkinase	Protein kina	.	.	GO:0005524|A'	K08835
Manes.11G07100(	0.4967071	0.01684	down	yes	T-complex pr	pfam00118	Cpn60_TCP1	TCP-1/cpn6	GO:0006457|p	GO:0005829|	GO:0005524|A'	K09497
Manes.11G07120(	0.3020492	1.91E-14	down	yes	.	pfam12767	SAGA-Tad1	Transcriptio	.	.	.	.
Manes.11G07140(	1.0304969	0.86842	up	no	.	pfam15279	SOBP	Sine oculis-l	.	.	.	.
Manes.11G07180(	2.0065143	1.69E-14	up	yes	4-coumarate-	pfam00501	AMP-binding	AMP-bindin	GO:0009698|p	.	GO:0016207|4-	.
Manes.11G07200(	0.8032874	0.02163	down	no	Acetyl-coenz	pfam00501	AMP-binding	AMP-bindin	GO:0006083|a	GO:0009507|	GO:0003987|ac	K01895
Manes.11G07210(	4.741435	1.02E-07	up	yes	.	pfam10186	Atg14	UV radiatio	.	.	.	.
Manes.11G07230(	1.9457139	3.94E-10	up	no	WD repeat-co	pfam00400	WD40	WD domain	GO:0009718|a	GO:0005634|	GO:0044212|tra	K11805
Manes.11G07240(	1.2156688	0.09082	up	no	Glyceraldehy	pfam00044	Gp_dh_N	Glyceraldeh	GO:0006006|g	GO:0009507|	GO:0047100|gly	.
Manes.11G07290(	0.1591868	1.84E-08	down	yes	.	pfam12072	DUF3552	Domain of u	.	.	.	.
Manes.11G07310(	2.352059	2.85E-18	up	yes	OTU domain	pfam02338	OTU	OTU-like cy	.	.	GO:0008234|cy	.
Manes.11G07320(	4.2521648	6.00E-26	up	yes	.	pfam05553	DUF761	Cotton fibre	.	.	.	.
Manes.11G07350(	3.0701657	1.73E-18	up	yes	Probable mag	pfam05653	Mg_trans_NIPA	Magnesium	GO:0015693|r	GO:0005769|	GO:0015095|ma	.
Manes.11G07360(	0.5804114	0.00061	down	no	DNA cross-li	pfam07522	DRMBL	DNA repair	GO:0006281|I	GO:0005634|	.	K15340
Manes.11G07380(	0.5745386	0.00724	down	no	.	pfam04819	DUF716	Family of ur	.	.	.	.
Manes.11G07390(	5.0132449	8.00E-22	up	yes	Transcription	pfam00270	DEAD	DEAD/DEA	GO:0006355|r	GO:0005737|	GO:0005524|A'	K03723
Manes.11G07460(	0.4254825	1.16E-06	down	yes	Nudix hydrol	pfam00293	NUDIX	NUDIX dor	.	GO:0005739|	GO:0016787|hy	.
Manes.11G07470(	0.8705343	0.31628	down	no	Calcium-bind	pfam00153	Mito_carr	Mitochondr	GO:0015867|	GO:0005737|	GO:0005347|A'	K14684
Manes.11G07520(	NA	NA	--	no	Chalcone syn	pfam00195	Chal_sti_synt_N	Chalcone an	GO:0009813|f	.	GO:0016210|na	.
Manes.11G07540(	Inf	0.1623	up	no	.	pfam08455	SNF2_assoc	Bacterial SN	.	.	.	.
Manes.11G07560(	Inf	0.9009	up	no	.	pfam14244	UBN2_3	gag-polypep	.	.	.	.
Manes.11G07570(	1.2943549	0.01415	up	no	Proline-rich r	pfam07714	Pkinase_Tyr	Protein tyro	.	GO:0016021|	GO:0005524|A'	.
Manes.11G07610(	0.9712789	0.74416	down	no	Nuclear facto	pfam04922	DIE2_ALG10	DIE2/ALG1	.	GO:0031011|	GO:0003677|DI	K11671
Manes.11G07620(	0.4476113	1.22E-12	down	yes	.	pfam12046	DUF3529	Protein of u	.	.	.	.
Manes.11G07630(	1.0319261	0.70245	up	no	Cytochrome l	pfam00355	Rieske	Rieske [2Fe	.	GO:0016021|	GO:0051537|2 i	K00411
Manes.11G07640(	NA	NA	--	no	.	pfam09726	Macoilin	Transmemb	.	.	.	.
Manes.11G07670(	2.0490876	8.36E-05	up	yes	N-alpha-acety	pfam00583	Acetyltransf_1	Acetyltransf	GO:0006474|r	GO:0005737|	GO:0004596|pe	K00670

Manes.11G07690(	0.6810103	9.85E-05	down	no	Alpha-manno	pfam01074	Glyco_hydro_38	Glycosyl hy	GO:0006013|	GO:0000326|	GO:0004559|al	.
Manes.11G07700(	1.9652049	1.80E-14	up	no	RING finger	pfam13923	zf-C3HC4_2	Zinc finger,	GO:0045893|	GO:0005737|	GO:0004842|ub	.
Manes.11G07720(	1.1738708	0.07596	up	no	Aspartokinas	pfam00696	AA_kinase	Amino acid	GO:0009089|	GO:0009507|	GO:0016597|an	K00928
Manes.11G07750(	0.7306452	0.00619	down	no	.	pfam00300	His_Phos_1	Histidine ph	.	.	.	.
Manes.11G07760(	0.3698797	3.89E-05	down	yes	.	pfam09863	DUF2090	Uncharacter	.	.	.	.
Manes.11G07770(	0.5223081	2.41E-09	down	no	Protein trich	pfam13839	PC-Esterase	GDSL/SGN	.	GO:0016021|	.	.
Manes.11G07790(	1.0833165	0.38672	up	no	30S riboson	pfam01250	Ribosomal_S6	Ribosomal p	GO:0006412|	GO:0009507|	GO:0019843|rR	K02990
Manes.11G07800(	0.7151759	0.00211	down	no	.	pfam02676	TYW3	Methyltrans	.	.	.	.
Manes.11G07850(	2.3154361	7.48E-15	up	yes	.	pfam09649	CHZ	Histone cha	.	.	.	.
Manes.11G07880(	29.436679	1.22E-98	up	yes	GEM-like pro	pfam02893	GRAM	GRAM don	GO:0009793|	.	.	.
Manes.11G07890(	0.7851952	0.01552	down	no	Translocon-a	pfam05753	TRAP_beta	Translocon-	.	GO:0005789|	.	K13250
Manes.11G07910(	0.6089852	0.0002	down	no	Zinc finger p	pfam06203	CCT	CCT motif.	GO:0006355|	GO:0005634|	GO:0003700|tra	.
Manes.11G07920(	0.4144177	2.14E-05	down	yes	.	pfam03097	BRO1	BRO1-like	.	.	.	.
Manes.11G07950(	0.1453439	5.55E-28	down	yes	Protein FAM	pfam13889	Chromosome_seg	Chromosom	.	GO:0005634|	.	.
Manes.11G07960(	0.1633518	3.13E-12	down	yes	.	pfam00876	Innexin	Innexin. Thi	.	.	.	.
Manes.11G07970(	0.9463821	0.56624	down	no	Probable inac	pfam01202	SKI	Shikimate k	GO:0009658|	GO:0009507|	.	.
Manes.11G08020(	0.5193934	0.0712	down	no	.	pfam14108	DUF4281	Domain of u	.	.	.	.
Manes.11G08030(	0.6522837	0.00766	down	no	.	pfam10018	Med4	Vitamin-D-r	.	.	.	.
Manes.11G08040(	0.5226923	4.10E-06	down	no	Double-stran	pfam00035	dsrm	Double-stra	.	.	GO:0003725|dc	.
Manes.11G08050(	0.7156598	0.00518	down	no	Probable pro	pfam01529	zf-DHHC	DHHC paln	.	GO:0016021|	GO:0019706|pr	.
Manes.11G08080(	Inf	0.35135	up	no	.	pfam13855	LRR_8	Leucine rich	.	.	.	.
Manes.11G08120(	1.1347723	0.16609	up	no	tRNA-dihydr	pfam01207	Dus	Dihydrourid	.	.	GO:0050660|fla	K05544
Manes.11G08140(	0.8912421	0.27388	down	no	Protein BASI	pfam06217	GAGA_bind	GAGA bind	GO:0050793|	GO:0005634|	GO:0003677|DI	.
Manes.11G08150(	0.9725015	0.83678	down	no	V-type proto	pfam00137	ATP-synt_C	ATP syntha	GO:0015991|	GO:0016021|	GO:0015078|hy	.
Manes.11G08160(	6.6595265	2.04E-66	up	yes	Arginine deca	pfam02784	Orn_Arg_deC_N	Pyridoxal-de	GO:0006527|	.	GO:0008792|ar	.
Manes.11G08170(	1.6422725	4.29E-05	up	no	30S ribosom	pfam01790	LGT	Prolipoprote	.	GO:0005739|	.	K19033
Manes.11G08230(	0.9871422	0.98996	down	no	.	pfam15576	DUF4661	Domain of u	.	.	.	.
Manes.11G08240(	0.6365783	8.28E-06	down	no	BEL1-like ho	pfam07526	POX	Associated	GO:0006351|	GO:0005634|	GO:0003700|tra	.
Manes.11G08290(	0.616138	0.00016	down	no	Coiled-coil d	pfam08317	Spc7	Spc7 kineto	GO:0006893|	GO:0005769|	.	.
Manes.11G08300(	1.3122345	0.00257	up	no	Transcription	pfam03634	TCP	TCP family	GO:0009653|	GO:0005634|	GO:0000987|co	.
Manes.11G08340(	0.6050171	9.46E-06	down	no	.	pfam01190	Pollen_Ole_e_I	Pollen prote	.	.	.	.
Manes.11G08440(	0.8964764	0.25469	down	no	Bifunctional	pfam00303	Thymidylat_synt	Thymidylate	GO:0006231|	.	GO:0004146|dil	K13998
Manes.11G08460(	1.0023238	0.95916	up	no	Phosphatidyli	pfam00650	CRAL_TRIO	CRAL/TRIO	GO:0015031|	GO:0000139|	GO:0005215|tra	.
Manes.11G08470(	1.1221803	0.20173	up	no	Transcription	pfam00249	Myb_DNA-binding	Myb-like DI	GO:0006383|	GO:0005634|	GO:0003677|DI	K15198
Manes.11G08490(	0.7955304	0.31001	down	no	.	pfam06040	Adeno_E3	Adenovirus	.	.	.	.
Manes.11G08500(	0.7243667	0.00786	down	no	Ocs element-	pfam00170	bZIP_1	bZIP transc	GO:0006351|	GO:0005634|	GO:0043565|se	.
Manes.11G08520(	0.630238	0.02986	down	no	.	pfam14364	DUF4408	Domain of u	.	.	.	.
Manes.11G08550(	1.4330473	0.04298	up	no	Glucose-1-ph	pfam00483	NTP_transferase	Nucleotidyl	GO:0005978|	GO:0009507|	GO:0005524|A	K00975
Manes.11G08560(	0.7335902	0.00146	down	no	F-box protein	pfam04690	YABBY	YABBY pro	GO:0009658|	.	.	.
Manes.11G08590(	0.9312446	0.62839	down	no	Probable pin	pfam05368	NmrA	NmrA-like f	.	.	GO:0010283|pi	.
Manes.11G08660(	0.4070491	6.88E-06	down	yes	Transcription	pfam00010	HLH	Helix-loop-l	GO:0009911|	GO:0005634|	GO:0003677|DI	.
Manes.11G08670(	2.0285576	1.82E-13	up	yes	rRNA-proces	pfam04900	Fcf1	Fcf1. Fcf1 i	GO:0006364|	GO:0005730|	GO:0044822|pc	K14566
Manes.11G08710(	1.230678	0.02934	up	no	Probable met	pfam03169	OPT	OPT oligop	GO:0055085|	GO:0016021|	.	.
Manes.11G08720(	2.0243546	3.38E-15	up	yes	Cysteine prot	pfam00112	Peptidase_C1	Papain famil	GO:0042742|	GO:0005615|	GO:0004197|cy	K01373
Manes.11G08730(	0.7711322	0.00694	down	no	V-type proto	pfam01496	V_ATPase_I	V-type ATP	GO:0015991|	GO:0009507|	GO:0051117|A	K02154
Manes.11G08760(	1.2049038	0.0838	up	no	.	pfam03579	SHP	Small hydro	.	.	.	.
Manes.11G08770(	0.874232	0.17505	down	no	Coatomer sul	pfam06957	COPI_C	Coatomer (C	GO:0006886|	GO:0030126|	GO:0005198|stı	K05236
Manes.11G08840(	0.3770113	0.01856	down	yes	Auxin-induce	pfam02519	Auxin_inducible	Auxin respo	GO:0009734|	.	.	K14488
Manes.11G08940(	NA	NA	--	no	COBRA-like	pfam04833	COBRA	COBRA-lik	GO:0016049|	GO:0031225|	.	.
Manes.11G08950(	NA	NA	--	no	.	pfam13961	DUF4219	Domain of u	.	.	.	.
Manes.11G08990(	1.1008866	0.30851	up	no	Triosephosph	pfam00121	TIM	Triosephosp	GO:0006096|	GO:0009507|	GO:0004807|tri	.
Manes.11G09010(	1.7352197	1.68E-06	up	no	Translocon-a	pfam03896	TRAP_alpha	Translocon-	.	GO:0009535|	.	.
Manes.11G09050(	1.9483749	8.46E-10	up	no	.	pfam06273	eIF-4B	Plant specifi	.	.	.	.
Manes.11G09110(	18.375729	0.0058	up	yes	Probable rec	pfam12819	Malectin_like	Carbohydrat	.	GO:0009507|	GO:0005524|A	.
Manes.11G09150(	17.657903	6.09E-44	up	yes	Late embryo	pfam00257	Dehydrin	Dehydrin.	GO:0006950|	.	.	.
Manes.11G09160(	1.0744366	0.47167	up	no	.	pfam13639	zf-RING_2	Ring finger	.	.	.	.
Manes.11G09190(	0.7620478	0.06548	down	no	MATE efflux	pfam01554	MatE	MatE. The I	.	GO:0009507|	GO:0015297|an	.
Manes.11G09200(	0.5797959	3.67E-07	down	no	MATE efflux	pfam01554	MatE	MatE. The I	.	GO:0009507|	GO:0015297|an	.
Manes.11G09240(	0.7448317	0.00296	down	no	.	pfam01436	NHL	NHL repeat	.	.	.	.
Manes.11G09260(	0.5578048	3.12E-08	down	no	Protein root	pfam04884	DUF647	Vitamin B6	GO:0032502|	GO:0009941|	.	.
Manes.11G09280(	0.6135466	0.00059	down	no	Endoglucana	pfam00759	Glyco_hydro_9	Glycosyl hy	GO:0071555|	GO:0005576|	GO:0008810|ce	K01179
Manes.11G09300(	2.1499851	1.19E-08	up	yes	Glycine-rich	pfam00313	CSD	'Cold-shock	GO:0006355|	.	GO:0003677|DI	.
Manes.11G09320(	Inf	0.9009	up	no	Auxin-induce	pfam02519	Auxin_inducible	Auxin respo	GO:0009734|	.	.	.
Manes.11G09370(	0.6651972	0.04839	down	no	Auxin-induce	pfam02519	Auxin_inducible	Auxin respo	GO:0009734|	.	.	.
Manes.11G09410(	1.7326387	6.73E-10	up	no	Rac-like GTI	pfam00071	Ras	Ras family.	GO:0009734|	GO:0005737|	GO:0005525|G	K04392
Manes.11G09460(	0.2982161	3.15E-09	down	yes	.	pfam00855	PWWP	PWWP dom	.	.	.	.
Manes.11G09480(	2.2912685	1.25E-19	up	yes	Myb-related	pfam00249	Myb_DNA-binding	Myb-like DI	GO:0006355|	GO:0005634|	GO:0003677|DI	.
Manes.11G09490(	1.7113397	1.61E-09	up	no	Proteasome s	pfam00227	Proteasome	Proteasome	GO:0006511|	GO:0005737|	GO:0004298|th	K02731
Manes.11G09550(	2.3206266	0.00113	up	yes	.	pfam06813	Nodulin-like	Nodulin-like	.	.	.	.
Manes.11G09560(	NA	NA	--	no	.	pfam09043	Lys-AminoMut_A	D-Lysine 5,	.	.	.	.
Manes.11G09570(	0.595785	0.00261	down	no	Endo-1,4-bet	pfam00331	Glyco_hydro_10	Glycosyl hy	GO:0045493|	GO:0005576|	GO:0030246|ca	.
Manes.11G09610(	2.7935824	7.30E-09	up	yes	Probable BO	pfam13920	zf-C3HC4_3	Zinc finger,	GO:0006952|	GO:0005634|	GO:0016874|lig	K19042
Manes.11G09630(	0.4602765	1.04E-05	down	yes	.	pfam03251	Tymo_45kd_70kd	Tymovirus	.	.	.	.
Manes.11G09650(	2.3992913	9.84E-18	up	yes	Heparan-alph	pfam01757	Acyl_transf_3	Acyltransfer	GO:0007041|	GO:0016021|	GO:0015019|he	K10532
Manes.11G09660(	1.3247948	0.044	up	no	Aquaporin PI	pfam00230	MIP	Major intrin	GO:0009992|	GO:0046658|	GO:0015254|gl	K09872
Manes.11G09670(	0.9963085	0.99422	down	no	Pre-mRNA-s	pfam08231	SYF2	SYF2 splici	GO:0006397|	GO:0005634|	.	K12868
Manes.11G09690(	2.050756	4.15E-09	up	yes	.	pfam05278	PEARLI-4	Arabidopsis	.	.	.	.
Manes.11G09700(	2.0769342	2.03E-09	up	yes	.	pfam06916	DUF1279	Protein of u	.	.	.	.
Manes.11G09730(	0.6016977	5.61E-07	down	no	Probable pro	pfam00481	PP2C	Protein pho	GO:0006470|	GO:0016021|	GO:0046872|m	.
Manes.11G09740(	Inf	1.50E-05	up	yes	.	pfam04782	DUF632	Protein of u	.	.	.	.
Manes.11G09750(	0.5874869	5.97E-06	down	no	.	pfam11833	DUF3353	Protein of u	.	.	.	.
Manes.11G09760(	0.8344393	0.14342	down	no	.	pfam01593	Amino_oxidase	Flavin conta	.	.	.	.
Manes.11G09780(	0.7084064	0.00387	down	no	INO80 comp	pfam08265	YL1_C	YL1 nuclea	GO:0006338|	GO:0031011|	.	K11667
Manes.11G09790(	1.3864168	0.00029	up	no	Transportin-1	pfam03810	IBN_N	Importin-be	GO:0006886|	GO:0005737|	.	K18752
Manes.11G09800(	0.3133603	2.83E-09	down	yes	Hippocampus	pfam07690	MFS_1	Major Facili	GO:0055085|	GO:0016021|	GO:0005215|tra	.
Manes.11G09810(	0.9585329	0.71252	down	no	DDB1- and C	pfam00400	WD40	WD domain	GO:0016567|	GO:0080008|	.	K11804
Manes.11G09820(	1.9970511	4.21E-14	up	no	Lysine-specif	pfam01593	Amino_oxidase	Flavin conta	GO:0016575|	GO:0005634|	GO:0003677|DI	K11450
Manes.11G09830(	1.1862508	0.08363	up	no	.	pfam12937	F-box-like	F-box-like.	.	.	.	.

Manes.11G09850(	0.4597534	1.81E-05	down	yes	Sulfite oxidas	pfam00174	Oxidored_molyb	Oxidoreduct	GO:0015994|(	GO:0005739|	GO:0030151|m	.
Manes.11G09880(	0.7186704	0.06185	down	no	.	pfam01529	zf-DHHC	DHHC palm	.	.	.	.
Manes.11G09890(	0.5782931	0.00013	down	no	V-type proto	pfam01813	ATP-synt_D	ATP syntha	GO:0015992||	GO:0005794|	GO:0042626|A	K02149
Manes.11G09910(	4.2423133	0.00117	up	yes	.	pfam03976	PPK2	Polyphospha	.	.	.	.
Manes.11G09940(	0.2986814	4.21E-08	down	yes	Probable rece	pfam07714	Pkinase_Tyr	Protein tyro	.	GO:0016021|	GO:0005524|A	.
Manes.11G09950(	0.2752136	2.95E-14	down	yes	DnaJ protein	pfam00226	DnaJ	DnaJ domai	GO:0006457||	GO:0005886|	GO:0005524|A	.
Manes.11G09960(	0.7570481	0.00507	down	no	Protein IQ-D	pfam13178	DUF4005	Protein of u	.	GO:0005634|	.	.
Manes.11G09970(	0.453773	0.02891	down	yes	.	pfam08058	NPCC	Nuclear por	.	.	.	.
Manes.11G09980(	Inf	0.35135	up	no	Probable E3	pfam01485	IBR	IBR domain	.	.	GO:0016874|lig	K11968
Manes.11G09990(	Inf	0.9009	up	no	Probable E3	pfam01485	IBR	IBR domain	.	.	GO:0016874|lig	K11968
Manes.11G10070(	0.8742286	0.20806	down	no	RNA polyme	pfam08621	RPAP1_N	RPAP1-like	GO:0006366||	GO:0005634|	GO:0003677|D	.
Manes.11G10100(	8.1245673	7.71E-06	up	yes	Protein CHL	pfam06203	CCT	CCT motif.	GO:0045036||	GO:0009507|	.	.
Manes.11G10130(	Inf	0.02421	up	yes	E3 ubiquitin-	pfam04564	U-box	U-box doma	GO:0006952|(	.	GO:0016874|lig	.
Manes.11G10140(	1.6212368	5.72E-07	up	no	.	pfam03215	Rad17	Rad17 cell c	.	.	.	.
Manes.11G10160(	1.4527951	9.63E-05	up	no	Zinc finger C	pfam14259	RRM_6	RNA recogn	.	.	GO:0003677|D	.
Manes.11G10190(	0.8577685	0.11704	down	no	Cytokinesis p	pfam00069	Pkinase	Protein kina	GO:0032147|	GO:0005813|	GO:0005524|A	.
Manes.11G10200(	1.6680612	1.33E-08	up	no	E3 ubiquitin l	pfam13639	zf-RING_2	Ring finger	GO:0051865||	.	GO:0016874|lig	K19045
Manes.11G10210(	1.6734771	6.65E-09	up	no	Eukaryotic in	pfam00270	DEAD	DEAD/DEA	.	.	GO:0005524|A	.
Manes.11G10280(	1.5334012	0.05457	up	no	.	pfam09545	RE_AccI	AccI restrict	.	.	.	.
Manes.11G10340(	NA	NA	--	no	Putative clath	pfam07651	ANTH	ANTH dom	GO:0048268|(	GO:0030136|	GO:0005545|1-	.
Manes.11G10350(	1.274176	0.0105	up	no	Putative axial	pfam04690	YABBY	YABBY pro	GO:0010158|	GO:0005634|	GO:0003677|D	.
Manes.11G10370(	0.9133603	0.40373	down	no	.	pfam14184	YrvL	Regulatory	.	.	.	.
Manes.11G10390(	1.5838012	4.39E-07	up	no	E3 ubiquitin-	pfam13639	zf-RING_2	Ring finger	GO:0009561|	GO:0005886|	GO:0016874|lig	K19043
Manes.11G10410(	0.4979658	2.72E-05	down	yes	Wall-associat	pfam00069	Pkinase	Protein kina	GO:0009992|	GO:0005623|	GO:0005524|A	.
Manes.11G10430(	1.5464235	4.67E-06	up	no	Serine/threon	pfam00069	Pkinase	Protein kina	.	GO:0009507|	GO:0005524|A	K08864
Manes.11G10450(	1.2119284	0.04048	up	no	DNA-binding	pfam00098	zf-CCHC	Zinc knuckle	.	GO:0005634|	GO:0003677|D	.
Manes.11G10470(	0.7003097	0.0215	down	no	Probable carb	pfam07859	Abhydrolase_3	alpha/beta h	.	.	GO:0052689|ca	.
Manes.11G10490(	1.0547154	0.59135	up	no	50S ribosoma	pfam00542	Ribosomal_L12	Ribosomal p	GO:0006412||	GO:0009507|	GO:0003735|str	K02935
Manes.11G10510(	1.0135493	0.87925	up	no	Beta-glucosid	pfam00933	Glyco_hydro_3	Glycosyl hy	GO:0085030|	GO:0042597|	GO:0008422|be	.
Manes.11G10540(	0.5749569	0.00039	down	no	Inactive prot	pfam00011	HSP20	Hsp20/alpha	GO:0006952|(	GO:0016021|	.	.
Manes.11G10580(	5.3151089	3.87E-14	up	yes	Expansin-like	pfam01357	Pollen_allerg_1	Pollen allerg	GO:0019953|	GO:0005576|	.	.
Manes.11G10590(	0.9229009	0.5244	down	no	60S ribosoma	pfam03947	Ribosomal_L2_C	Ribosomal I	GO:0006412||	GO:0015934|	GO:0003723|R	.
Manes.11G10630(	#NAME?	0.01825	down	yes	.	pfam03087	DUF241	Arabidopsis	.	.	.	.
Manes.11G10640(	1.2429105	0.01995	up	no	Probable seri	pfam00069	Pkinase	Protein kina	GO:0009742||	GO:0016020|	GO:0005524|A	K14500
Manes.11G10650(	0.7124127	0.17037	down	no	.	pfam05889	SLA_LP_auto_ag	Soluble liver	.	.	.	.
Manes.11G10660(	1.3292236	0.00172	up	no	.	pfam04782	DUF632	Protein of u	.	.	.	.
Manes.11G10680(	1.3874943	0.03741	up	no	Uncharacteri	pfam10250	O-FucT	GDP-fucose	.	GO:0005768|	GO:0016757|tra	.
Manes.11G10690(	1.0955256	0.36707	up	no	Glycerol-3-pl	pfam01553	Acyltransferase	Acyltransfer	GO:0016024|(	GO:0005783|	GO:0003841|1-	K13506
Manes.11G10720(	3.7803891	0.05842	up	no	.	pfam14111	DUF4283	Domain of u	.	.	.	.
Manes.11G10730(	5.5428822	2.19E-12	up	yes	Cytochrome	pfam00067	p450	Cytochrome	GO:0071555|	GO:0005783|	GO:0020037|he	K15402
Manes.11G10760(	0.7872271	0.01677	down	no	ENHANCER	pfam00855	PWWP	PWWP dom	GO:0030154|	GO:0005634|	GO:0003700|tra	.
Manes.11G10800(	3.9371456	5.37E-05	up	yes	Calcium-trans	pfam00122	E1-E2_ATPase	E1-E2 ATP	GO:0055081|	GO:0009507|	GO:0005524|A	K01537
Manes.11G10850(	1.2536591	0.01325	up	no	Transcription	pfam03634	TCP	TCP family	GO:0006355||	GO:0005634|	GO:0003677|D	.
Manes.11G10870(	0.7358108	0.015	down	no	.	pfam10717	ODV-E18	Occlusion-d	.	.	.	.
Manes.11G10880(	0.8862416	0.35894	down	no	.	pfam13098	Thioredoxin_2	Thioredoxin	.	.	.	.
Manes.11G10910(	0.7483044	0.03252	down	no	DNA repair p	pfam08423	Rad51	Rad51. Rad	GO:0006310||	GO:0005634|	GO:0005524|A	.
Manes.11G10920(	0.5139782	0.01418	down	no	.	pfam08387	FBD	FBD. This r	.	.	.	.
Manes.11G10970(	1.0065069	0.94897	up	no	Protein S-acy	pfam01529	zf-DHHC	DHHC palm	.	GO:0016021|	GO:0019706|pr	.
Manes.11G11000(	0.5708067	4.74E-05	down	no	Alpha-1,3-ma	pfam03071	GNT-I	GNT-I fami	GO:0006972||	GO:0005768|	GO:0003827|alp	.
Manes.11G11010(	0.6517513	0.00307	down	no	Proton-coupl	pfam01490	Aa_trans	Transmemb	GO:0015816||	GO:0016021|	GO:0015187|gl	K14209
Manes.11G11030(	27.21785	2.10E-07	up	yes	EC protein h	pfam02068	Metallothio_PEC	Plant PEC f	GO:0006829|	GO:0005737|	GO:0008270|zi	.
Manes.11G11050(	4.9115221	1.49E-71	up	yes	Probable seri	pfam07714	Pkinase_Tyr	Protein tyro	GO:0009755||	GO:0005886|	GO:0005524|A	.
Manes.11G11080(	2.3959504	1.65E-12	up	yes	.	pfam13041	PPR_2	PPR repeat	.	.	.	.
Manes.11G11090(	0.7262555	0.02928	down	no	Protein NLP	pfam02042	RWP-RK	RWP-RK do	GO:0006351||	GO:0005634|	GO:0003677|D	.
Manes.11G11110(	0.675618	0.00255	down	no	SNF1-related	pfam04739	AMPKBI	5'-AMP-acti	GO:0005975|(	.	GO:0019887|pr	.
Manes.11G11130(	8.1801411	0.01638	up	yes	Ankyrin repe	pfam12796	Ank_2	Ankyrin rep	.	GO:0005783|	.	.
Manes.11G11140(	1.0320864	0.72745	up	no	Serrate RNA	pfam12066	DUF3546	Domain of u	GO:0016568|	GO:0009507|	GO:0003677|D	.
Manes.11G11150(	1.0878784	0.34544	up	no	Protein FAR	pfam03101	FAR1	FAR1 DNA	GO:0006355||	GO:0005634|	GO:0008270|zi	.
Manes.11G11160(	0.7474163	0.01389	down	no	(+)-neomenth	pfam00106	adh_short	short chain	GO:0006952|(	.	GO:0047501|(+	.
Manes.11G11190(	1.0762232	0.4483	up	no	Non-specific	pfam14368	LTP_2	Probable lip	GO:0042335|	GO:0031225|	GO:0008289|lip	.
Manes.11G11220(	0.6533984	0.00084	down	no	.	pfam11139	DUF2910	Protein of u	.	.	.	.
Manes.11G11240(	2.3447681	1.42E-07	up	yes	.	pfam03168	LEA_2	Late embryo	.	.	.	.
Manes.11G11250(	2.2508515	7.20E-18	up	yes	rRNA-proces	pfam04900	Fcf1	Fcf1. Fcf1 is	GO:0006364|	GO:0005730|	GO:0044822|po	K14773
Manes.11G11310(	1.3149354	0.00234	up	no	Somatic emb	pfam08263	LRRNT_2	Leucine rich	GO:0009742||	GO:0005789|	GO:0005524|A	K13418
Manes.11G11350(	1.6048617	3.21E-07	up	no	Ras-related p	pfam00071	Ras	Ras family.	GO:0006886||	GO:0005829|	GO:0005525|G	K07904
Manes.11G11360(	0.5667329	0.00193	down	no	Pirin-like pro	pfam02678	Pirin	Pirin. This f	.	GO:0005634|	.	K06911
Manes.11G11390(	0.4206318	1.05E-12	down	yes	UDP-glycosy	pfam00201	UDPGT	UDP-glucor	.	.	GO:0016758|tra	.
Manes.11G11460(	3.2348949	2.11E-11	up	yes	.	pfam09801	SYS1	Integral mer	.	.	.	.
Manes.11G11470(	2.9941828	2.36E-07	up	yes	.	pfam01190	Pollen_Ole_e_I	Pollen prote	.	.	.	.
Manes.11G11480(	1.3382255	0.00139	up	no	Clathrin inter	pfam01417	ENTH	ENTH doma	GO:0015031||	GO:0030136|	.	K12471
Manes.11G11520(	32.596935	1.02E-17	up	yes	Probable xylo	pfam00722	Glyco_hydro_16	Glycosyl hy	GO:0042546|	GO:0048046|	GO:0004553|hy	K08235
Manes.11G11530(	1.009437	0.89459	up	no	Solute carrie	pfam06027	DUF914	Eukaryotic	GO:0006810||	GO:0016021|	.	K15287
Manes.11G11620(	2.3190897	5.47E-19	up	yes	CMP-sialic a	pfam04142	Nuc_sug_transp	Nucleotide-	GO:0015739|	GO:0000139|	GO:0015136|sia	.
Manes.11G11640(	4.4819886	1.03E-09	up	yes	Probable rece	pfam00069	Pkinase	Protein kina	.	GO:0016021|	GO:0005524|A	.
Manes.11G11650(	1.0527191	0.67371	up	no	Glycerophosp	pfam00069	Pkinase	Protein kina	GO:0006968|(	GO:0016021|	GO:0005524|A	.
Manes.11G11660(	0.2971897	4.93E-18	down	yes	Glycerophosp	pfam00069	Pkinase	Protein kina	GO:0006968|(	GO:0016021|	GO:0005524|A	.
Manes.11G11670(	9.7833208	0.03145	up	no	.	pfam11186	DUF2972	Protein of u	.	.	.	.
Manes.11G11740(	0.8941885	0.41931	down	no	INO80 comp	pfam13891	zf-C3Hc3H	Potential DI	GO:0006310||	GO:0005634|	.	.
Manes.11G11750(	0.9855014	0.90203	down	no	Probable glut	pfam00255	GSHPx	Glutathione	GO:0006979||	GO:0005829|	GO:0004602|gl	K00432
Manes.11G11760(	3.3627712	1.96E-13	up	yes	.	pfam07939	DUF1685	Protein of u	.	.	.	.
Manes.11G11770(	1.5744085	0.00289	up	no	Inositol trans	pfam00083	Sugar_tr	Sugar (and	GO:0015798||	GO:0016021|	GO:0005366|m	K08150
Manes.11G11780(	0.7711767	0.3422	down	no	.	pfam02876	Stap_Strp_tox_C	Staphylococ	.	.	.	.
Manes.11G11790(	0.6899613	0.44452	down	no	Probable leuc	pfam00069	Pkinase	Protein kina	.	GO:0016021|	GO:0005524|A	.
Manes.11G11800(	0.5097609	0.01425	down	no	Probable LRI	pfam00069	Pkinase	Protein kina	.	GO:0016021|	GO:0005524|A	.
Manes.11G11830(	0.9669919	0.76365	down	no	Probable E3	pfam01485	IBR	IBR domain	.	.	GO:0016874|lig	K11968
Manes.11G11870(	0.2839506	0.03069	down	no	.	pfam02537	CRCB	CrcB-like pr	.	.	.	.
Manes.11G11890(	0.6382437	0.00134	down	no	Probable leuc	pfam13855	LRR_8	Leucine rich	.	GO:0016021|	GO:0005524|A	.
Manes.11G11920(	0.444781	0.0029	down	yes	Probable leuc	pfam00069	Pkinase	Protein kina	.	GO:0016021|	GO:0005524|A	.

Manes.11G11970(	2.4725624	1.92E-15	up	yes	TMV resistar	pfam01582	TIR	TIR domain	GO:0009626|	GO:0005737|	GO:0043531|Al	.
Manes.11G11980(	1.184293	0.23115	up	no	3-oxo-Delta(	pfam07019	Rab5ip	Rab5-intera	GO:0009611|	GO:0005829|	GO:0047787|de	.
Manes.11G12000(	1.1037558	0.6281	up	no	3-oxo-Delta(	pfam01370	Epimerase	NAD depen	GO:0009611|	GO:0005829|	GO:0047787|de	.
Manes.11G12010(	#NAME?	0.43419	down	no	Probable 2-o	pfam03171	2OG-FeII_Oxy	2OG-Fe(II)	.	.	GO:0046872|m	.
Manes.11G12040(	0.6538055	6.03E-05	down	no	Tetrapyrrole-	pfam05419	GUN4	GUN4-like.	GO:0015995|	GO:0009507|	GO:0019899|en	.
Manes.11G12130(	0.8157814	0.67771	down	no	Probable LRI	pfam07714	Pkinase_Tyr	Protein tyro	.	GO:0016021|	GO:0005524|A	.
Manes.11G12160(	6.110874	0.00021	up	yes	.	pfam15455	Pro-rich_19	Proline-rich	.	.	.	.
Manes.11G12170(	0.8610572	0.26359	down	no	Protein FAR	pfam03101	FAR1	FAR1 DNA	GO:0006355|	GO:0005634|	GO:0008270|zi	.
Manes.11G12190(	1.5529655	9.33E-05	up	no	Protein YIF1	pfam03878	YIF1	YIF1. YIF1	.	GO:0016021|	.	.
Manes.11G12200(	1.3385371	0.00279	up	no	B-box zinc fi	pfam00643	zf-B_box	B-box zinc f	GO:0010100|	GO:0005634|	GO:0003700|tr	.
Manes.11G12240(	NA	NA	--	no	.	pfam06728	PIG-U	GPI transam	.	.	.	.
Manes.11G12310(	0.2588008	0.24759	down	no	.	pfam10329	DUF2417	Region of u	.	.	.	.
Manes.11G12320(	23.094629	3.64E-17	up	yes	Probable dise	pfam00931	NB-ARC	NB-ARC do	GO:0006952|	.	GO:0043531|Al	.
Manes.11G12330(	5.1632711	2.06E-26	up	yes	.	pfam13968	DUF4220	Domain of u	.	.	.	.
Manes.11G12350(	1.8352167	4.12E-11	up	no	Electron tran	pfam05187	ETF_QO	Electron tra	GO:0006552|	GO:0005743|	GO:0051539|4	K00311
Manes.11G12360(	2.1651587	4.63E-18	up	yes	Probable tran	pfam08614	ATG16	Autophagy	GO:0006351|	GO:0005737|	GO:0043565|se	.
Manes.11G12380(	0.6116624	0.00068	down	no	.	pfam02536	mTERF	mTERF. Th	.	.	.	.
Manes.11G12410(	0.5537442	2.00E-05	down	no	.	pfam02536	mTERF	mTERF. Th	.	.	.	.
Manes.11G12420(	0.8346103	0.1815	down	no	.	pfam02536	mTERF	mTERF. Th	.	.	.	.
Manes.11G12430(	0.5864303	0.00061	down	no	.	pfam02536	mTERF	mTERF. Th	.	.	.	.
Manes.11G12440(	0.6805618	0.01145	down	no	.	pfam02536	mTERF	mTERF. Th	.	.	.	.
Manes.11G12450(	0.2279371	2.75E-11	down	yes	.	pfam02536	mTERF	mTERF. Th	.	.	.	.
Manes.11G12470(	0.5700287	0.00039	down	no	.	pfam02536	mTERF	mTERF. Th	.	.	.	.
Manes.11G12490(	70.803696	2.97E-07	up	yes	S-type anion	pfam03595	SLAC1	Voltage-dep	GO:0006873|	GO:0016021|	GO:0008308|vo	.
Manes.11G12500(	14.283076	4.57E-25	up	yes	Mitogen-acti	pfam00069	Pkinase	Protein kina	GO:0043622|	GO:0005737|	GO:0005524|A	.
Manes.11G12510(	1.6986099	0.00022	up	no	Aspartic prot	pfam00026	Asp	Eukaryotic a	GO:0006629|	GO:0005829|	GO:0004190|as	K08245
Manes.11G12520(	1.1391632	0.18266	up	no	Probable hyd	pfam00753	Lactamase_B	Metallo-beta	GO:0006750|	GO:0009507|	GO:0004416|hy	K01069
Manes.11G12580(	0.6976551	0.01331	down	no	Ent-kaurenoi	pfam00067	p450	Cytochrome	GO:0009686|	GO:0005783|	GO:0051777|en	K04123
Manes.11G12590(	0.7787629	0.04695	down	no	Protein SHO	pfam03514	GRAS	GRAS doma	GO:0008356|	GO:0005769|	GO:0043565|se	.
Manes.11G12600(	0.451629	8.07E-08	down	yes	.	pfam04266	ASCH	ASCH doma	.	.	.	.
Manes.11G12670(	0.7535221	0.01724	down	no	TBC1 domai	pfam00566	RabGAP-TBC	Rab-GTPas	GO:0043547|	GO:0005737|	GO:0005096|G	.
Manes.11G12710(	Inf	0.04432	up	no	Peroxisomal	pfam00577	Usher	Outer memb	GO:0006635|	GO:0005778|	.	K13344
Manes.11G12720(	0.6347231	7.03E-06	down	no	Uncharacteri	pfam14750	INTS2	Integrator c	.	GO:0031225|	.	.
Manes.11G12750(	0.9446642	0.57469	down	no	GPI ethanola	pfam01663	Phosphodiest	Type I phos	GO:0006501|	GO:0005789|	GO:0051377|m	K05288
Manes.11G12790(	1.3448019	0.00462	up	no	DNA-directe	pfam03870	RNA_pol_Rpb8	RNA polym	GO:0006360|	GO:0005736|	.	K03016
Manes.11G12810(	4.7000904	2.02E-08	up	yes	.	pfam00520	Ion_trans	Ion transpo	.	.	.	.
Manes.11G12820(	0.2428475	0.40652	down	no	Endochitinas	pfam00182	Glyco_hydro_19	Chitinase cl	GO:0016998|	.	GO:0008061|ch	.
Manes.11G12830(	0.5734318	0.01968	down	no	Endochitinas	pfam00182	Glyco_hydro_19	Chitinase cl	GO:0016998|	.	GO:0008061|ch	.
Manes.11G12850(	0.8680077	0.16401	down	no	Acyl-[acyl-ca	pfam03405	FA_desaturase_2	Fatty acid d	GO:0006633|	GO:0009507|	GO:0045300|ac	K03921
Manes.11G12860(	0.5939257	0.00109	down	no	DNA primas	pfam01896	DNA_primase_S	Eukaryotic	.	GO:0016020|	GO:0003896|D	K02684
Manes.11G12870(	0.6448168	8.07E-06	down	no	.	pfam13178	DUF4005	Protein of u	.	.	.	.
Manes.11G12880(	0.400968	2.33E-05	down	yes	.	pfam03324	Herpes_HEPA	Herpesvirus	.	.	.	.
Manes.11G12890(	Inf	0.18039	up	no	.	pfam08886	GshA	Glutamate-c	.	.	.	.
Manes.11G12910(	1.9902197	2.08E-12	up	no	F-box/kelch-	pfam13964	Kelch_6	Kelch motif.	GO:0016567|	GO:0005634|	.	.
Manes.11G12930(	2.1006254	1.86E-06	up	yes	Transcription	pfam00010	HLH	Helix-loop-	GO:0006355|	GO:0005634|	GO:0003677|D	.
Manes.11G12970(	2.0087054	4.76E-11	up	yes	Putative dise	pfam00931	NB-ARC	NB-ARC do	GO:0006952|	.	GO:0043531|Al	.
Manes.11G12980(	0.722491	0.01508	down	no	.	pfam08387	FBD	FBD. This r	.	.	.	.
Manes.11G12990(	1.0980885	0.54063	up	no	Disease resis	pfam00931	NB-ARC	NB-ARC do	GO:0006952|	.	GO:0043531|Al	.
Manes.11G13000(	12.769788	2.91E-15	up	yes	Disease resis	pfam00931	NB-ARC	NB-ARC do	GO:0006952|	.	GO:0043531|Al	.
Manes.11G13050(	0.5665611	2.94E-07	down	no	.	pfam15489	CTC1	CST, telom	.	.	.	.
Manes.11G13070(	0.0509091	0.00043	down	yes	Endochitinas	pfam00182	Glyco_hydro_19	Chitinase cl	GO:0016998|	.	GO:0008061|ch	.
Manes.11G13080(	1.5392219	0.00012	up	no	Fasciclin-like	pfam02469	Fasciclin	Fasciclin do	GO:0007155|	GO:0031225|	GO:0030247|pc	.
Manes.11G13090(	2.4318574	1.32E-10	up	yes	Probable pro	pfam00481	PP2C	Protein pho	GO:0048366|	GO:0005634|	GO:0046872|m	.
Manes.11G13160(	0.6821456	0.00124	down	no	Dof zinc fing	pfam02701	zf-Dof	Dof domain	GO:0006355|	GO:0005634|	GO:0046872|m	.
Manes.11G13170(	0.7994953	0.15291	down	no	.	pfam03798	TRAM_LAG1_CI	TLC domai	.	.	.	.
Manes.11G13200(	0.4078504	0.00293	down	yes	CLAVATA3	pfam03650	MPC	Uncharacter	GO:0045168|	GO:0005615|	.	.
Manes.11G13220(	0.9135135	0.40069	down	no	Ubiquitin-co	pfam00179	UQ_con	Ubiquitin-co	GO:0016567|	.	GO:0005524|A	K10575
Manes.11G13260(	9.5367973	0.10882	up	no	.	pfam13456	RVT_3	Reverse tra	.	.	.	.
Manes.11G13300(	0.595377	0.00135	down	no	.	pfam03395	Pox_P4A	Poxvirus P4	.	.	.	.
Manes.11G13330(	1.783393	7.58E-11	up	no	Actin-depoly	pfam00241	Cofilin_ADF	Cofilin/trop	GO:0030042|	GO:0015629|	.	.
Manes.11G13410(	19.509239	1.37E-17	up	yes	Zinc finger p	pfam13912	zf-C2H2_6	C2H2-type	GO:0009631|	GO:0005634|	GO:0046872|m	.
Manes.11G13420(	2.0147258	3.93E-06	up	yes	.	pfam06136	DUF966	Domain of u	.	.	.	.
Manes.11G13430(	0.3420462	1.54E-07	down	yes	Transcription	pfam00249	Myb_DNA-binding	Myb-like D	GO:0006355|	GO:0005634|	GO:0003677|D	.
Manes.11G13440(	0.6060945	1.60E-06	down	no	Very-long-ch	pfam04387	PTPLA	Protein tyro	GO:0030497|	GO:0005783|	GO:0018812|3-	K10703
Manes.11G13450(	0.0947557	1.01E-10	down	yes	.	pfam05996	Fe_bilin_red	Ferredoxin-	.	.	.	.
Manes.11G13470(	1.4570852	0.16319	up	no	.	pfam07795	DUF1635	Protein of u	.	.	.	.
Manes.11G13520(	19.199125	8.75E-41	up	yes	Glutelin type	pfam00190	Cupin_1	Cupin. This	.	.	GO:0045735|nu	.
Manes.11G13580(	0.3521981	5.62E-06	down	yes	Copper trans	pfam00403	HMA	Heavy-meta	GO:0006825|	GO:0000785|	GO:0046872|m	K07213
Manes.11G13590(	3.3045788	2.26E-34	up	yes	Exocyst com	pfam03081	Exo70	Exo70 exoc	GO:0006887|	GO:0005618|	.	.
Manes.11G13600(	1.5812315	1.56E-05	up	no	3-ketoacyl-C	pfam08392	FAE1_CUT1_Rpp	FAE1/Type	GO:0006633|	GO:0005783|	GO:0016747|tr	K15397
Manes.11G13610(	0.9769143	0.86738	down	no	Nudix hydrol	pfam00293	NUDIX	NUDIX dor	.	.	GO:0016787|hy	.
Manes.11G13620(	0.5097015	4.19E-05	down	no	Ankyrin repe	pfam12796	Ank_2	Ankyrin rep	.	GO:0005783|	.	.
Manes.11G13660(	0.9873146	0.91107	down	no	Probable NO	pfam04153	NOT2_3_5	NOT2 / NO	GO:0015074|	GO:0005634|	.	.
Manes.11G13670(	0.8501426	0.66583	down	no	2-alkenal red	pfam00107	ADH_zinc_N	Zinc-binding	.	.	GO:0047655|all	.
Manes.11G13680(	0.4683418	6.60E-09	down	yes	Receptor-like	pfam12819	Malectin_like	Carbohydra	GO:0046777|	GO:0016021|	GO:0005524|A	.
Manes.11G13820(	0.7025638	0.00194	down	no	Phototropin-	pfam00069	Pkinase	Protein kina	GO:0009785|	GO:0009986|	GO:0005524|A	K08282
Manes.11G13830(	1.793618	0.00012	up	no	Probable seri	pfam07714	Pkinase_Tyr	Protein tyro	.	.	GO:0005524|A	.
Manes.11G13840(	1.0188463	0.8609	up	no	Probable tran	pfam00107	ADH_zinc_N	Zinc-binding	GO:0006633|	GO:0009507|	GO:0005524|A	K07512
Manes.11G13850(	3.8502582	6.88E-31	up	yes	.	pfam13344	Hydrolase_6	Haloacid de	.	.	.	.
Manes.11G13870(	Inf	0.17828	up	no	Receptor pro	pfam00069	Pkinase	Protein kina	GO:0009755|	GO:0005576|	GO:0005524|A	K00924
Manes.11G13880(	73.904078	#######	up	yes	.	pfam12569	NARP1	NMDA rece	.	.	.	.
Manes.11G13900(	95.514056	6.80E-24	up	yes	.	pfam02405	Permease	Permease. T	.	.	.	.
Manes.11G13910(	18.625281	6.27E-18	up	yes	.	pfam14351	DUF4401	Domain of u	.	.	.	.
Manes.11G13920(	11.33852	1.69E-11	up	yes	.	pfam14159	CAAD	CAAD dom	.	.	.	.
Manes.11G13980(	0.5999933	0.05396	down	no	Protein NRT	pfam00854	PTR2	POT family.	GO:0042128|	GO:0016021|	GO:0010542|ni	.
Manes.11G14010(	1.1499128	0.12882	up	no	V-type proto	pfam01496	V_ATPase_I	V-type ATP	GO:0015991|	GO:0005768|	GO:0051117|A	K02154
Manes.11G14050(	1.2048565	0.28377	up	no	Tetraspanin-	pfam00335	Tetraspannin	Tetraspanin	.	GO:0016021|	.	.
Manes.11G14070(	0.6560962	0.02899	down	no	tRNA-splicin	pfam01974	tRNA_int_endo	tRNA intro	GO:0006397|	GO:0005634|	GO:0016829|ly	K15322

Manes.11G14110(	0.7568282	0.02861	down	no	Mitochondria	pfam01926	MMR_HSR1	50S ribosom	GO:0006417|r	GO:0005743|	GO:0005525|G'	.
Manes.11G14140(	0.8335716	0.17756	down	no	Ribosome bio	pfam01926	MMR_HSR1	50S ribosom	GO:0042254|r	GO:0005737|	GO:0005525|G'	K14540
Manes.11G14150(	1.4114264	0.00148	up	no	Exonuclease	pfam09810	Exo5	Exonuclease	.	GO:0009507|	GO:0051539|4	K17815
Manes.11G14180(	0.5600484	0.00045	down	no	Pentatricopep	pfam13041	PPR_2	PPR repeat	.	GO:0005739|	.	.
Manes.11G14190(	0.7249312	0.01818	down	no	.	pfam05419	GUN4	GUN4-like.	.	.	.	.
Manes.11G14210(	1.5715561	7.36E-07	up	no	E3 SUMO-pr	pfam02891	zf-MIZ	MIZ/SP-RII	GO:0051301|c	GO:0016607|	GO:0016874|lig	.
Manes.11G14220(	0.7263334	0.01263	down	no	Isovaleryl-Co	pfam00441	Acyl-CoA_dh_1	Acyl-CoA d	GO:1902198|3	GO:0005739|	GO:0050660|fla	K00253
Manes.11G14230(	0.5387785	9.43E-10	down	no	Auxin respon	pfam06507	Auxin_resp	Auxin respo	GO:0010158|a	GO:0005634|	GO:0003677|D	.
Manes.11G14290(	0.7590788	0.0049	down	no	Zinc finger pr	pfam00096	zf-C2H2	Zinc finger,	GO:0006355|r	GO:0005634|	GO:0046872|me	.
Manes.11G14330(	NA	NA	--	no	Fasciclin-like	pfam02469	Fasciclin	Fasciclin do	GO:0009834|p	GO:0031225|	.	.
Manes.11G14350(	#NAME?	0.02406	down	yes	Fasciclin-like	pfam02469	Fasciclin	Fasciclin do	GO:0009834|p	GO:0031225|	.	.
Manes.11G14420(	0.7872465	0.01623	down	no	Serine/threon	pfam00069	Pkinase	Protein kina	GO:0006468|p	.	GO:0005524|A'	K07359
Manes.11G14430(	0.6538579	0.01899	down	no	F-box/kelch-r	pfam01344	Kelch_1	Kelch motif.	.	GO:0005634|	.	.
Manes.11G14440(	2.1984675	3.35E-18	up	yes	Probable E3 u	pfam13920	zf-C3HC4_3	Zinc finger,	GO:1901527|a	GO:0005886|	GO:0016874|lig	.
Manes.11G14450(	0.8603157	0.3438	down	no	.	pfam12906	RINGv	RING-varia	.	.	.	.
Manes.11G14480(	0.2771591	1.45E-06	down	yes	.	pfam13868	Trichoplein	tumor suppr	.	.	.	.
Manes.11G14490(	0.8419225	0.07883	down	no	Protein disulf	pfam13848	Thioredoxin_6	Thioredoxin	GO:0045454|c	GO:0005618|	GO:0003756|pr	.
Manes.11G14500(	0.912113	0.4197	down	no	Probable seri	pfam07714	Pkinase_Tyr	Protein tyro	GO:0009755|h	GO:0005886|	GO:0005524|A'	.
Manes.11G14550(	2.5744285	8.34E-10	up	yes	.	pfam04520	Senescence_reg	Senescence	.	.	.	.
Manes.11G14560(	0.8724106	1	down	no	.	pfam05007	Mannosyl_trans	Mannosyltra	.	.	.	.
Manes.11G14580(	0.9527624	0.62971	down	no	Vacuolar fusi	pfam03164	Mon1	Trafficking p	GO:0009306|p	GO:0005623|	.	.
Manes.11G14600(	0.6378292	1.29E-05	down	no	Homeobox-le	pfam08670	MEKHLA	MEKHLA d	GO:0030154|c	GO:0005634|	GO:0008289|lip	K09338
Manes.11G14610(	Inf	2.69E-05	up	yes	.	pfam12767	SAGA-Tad1	Transcriptio	.	.	.	.
Manes.11G14620(	0.8038326	0.10775	down	no	CASP-like pr	pfam04535	DUF588	Domain of u	.	GO:0016021|	.	.
Manes.11G14640(	3.8755413	7.09E-06	up	yes	Ubiquitin-like	pfam00240	ubiquitin	Ubiquitin fa	GO:0006464|c	GO:0005737|	GO:0031386|pr	K13113
Manes.11G14650(	1.3618077	0.0017	up	no	Auxin respon	pfam06507	Auxin_resp	Auxin respo	GO:0009734|a	GO:0005634|	GO:0003677|D	.
Manes.11G14660(	0.421692	1.92E-07	down	yes	GATA transc	pfam00320	GATA	GATA zinc	GO:0006351|t	GO:0005634|	GO:0043565|se	.
Manes.11G14680(	0.1715188	4.12E-06	down	yes	.	pfam10174	Cast	RIM-binding	.	.	.	.
Manes.11G14730(	1.4063858	0.0024	up	no	Putative cycli	pfam00520	Ion_trans	Ion transpor	GO:0071805|p	GO:0005887|	GO:0030552|cA	K05391
Manes.11G14740(	0.6882557	0.00037	down	no	Receptor-like	pfam07714	Pkinase_Tyr	Protein tyro	GO:0009755|h	GO:0012505|	GO:0005524|A'	.
Manes.11G14750(	1.0018567	1	up	no	Uncharacteriz	pfam14624	Vwaint	VWA / Hh p	.	.	.	K07114
Manes.11G14760(	0.4867518	0.01179	down	yes	.	pfam02601	Exonuc_VII_L	Exonuclease	.	.	.	.
Manes.11G14830(	Inf	0.9009	up	no	Kiwellin	pfam03330	DPBB_1	Rare lipopro	.	GO:0005576|	.	.
Manes.11G14840(	1.026759	0.80932	up	no	Kiwellin	pfam03330	DPBB_1	Rare lipopro	.	GO:0005576|	.	.
Manes.11G14880(	0.2827579	4.94E-09	down	yes	ABC transpo	pfam00646	F-box	F-box doma	GO:0006810|t	GO:0016021|	GO:0005524|A'	.
Manes.11G14890(	12.311299	6.03E-09	up	yes	ABC transpo	pfam00005	ABC_tran	ABC transpo	GO:0006810|t	GO:0016021|	GO:0005524|A'	.
Manes.11G14920(	0.5282416	9.70E-08	down	no	RuBisCO larg	pfam13921	Myb_DNA-bind_6	Myb-like DI	GO:0042026|p	GO:0009507|	GO:0005524|A'	.
Manes.11G14930(	0.7019546	0.00295	down	no	RuBisCO larg	pfam00118	Cpn60_TCP1	TCP-1/cpn6	GO:0042026|p	GO:0009507|	GO:0005524|A'	.
Manes.11G14940(	0.6536943	0.03359	down	no	.	pfam13207	AAA_17	AAA domai	.	.	.	.
Manes.11G14960(	0.339005	1.09E-06	down	yes	.	pfam05140	ResB	ResB-like fa	.	.	.	.
Manes.11G14980(	NA	NA	--	no	Cation/H(+) a	pfam00999	Na_H_Exchanger	Sodium/hyd	GO:0006813|p	GO:0016021|	GO:0005451|mo	.
Manes.11G15010(	2.0315824	3.95E-07	up	yes	.	pfam00684	DnaJ_CXXCXGX	DnaJ centra	.	.	.	.
Manes.11G15020(	5.0612558	2.71E-06	up	yes	.	pfam00777	Glyco_transf_29	Glycosyltrar	.	.	.	.
Manes.11G15030(	1.3718833	0.00047	up	no	Cell division	pfam00004	AAA	ATPase fam	GO:0007049|c	GO:0005886|	GO:0005524|A'	K13525
Manes.11G15070(	2.4654711	8.28E-06	up	yes	Auxin-induce	pfam02519	Auxin_inducible	Auxin respo	GO:0009734|a	.	.	.
Manes.11G15080(	3.6381709	1.60E-45	up	yes	Heavy metal-	pfam00403	HMA	Heavy-meta	GO:0010286|h	GO:0031965|	GO:0046872|me	.
Manes.11G15090(	1.4026562	0.05726	up	no	Heavy metal-	pfam00403	HMA	Heavy-meta	GO:0010286|h	GO:0031965|	GO:0046872|me	.
Manes.11G15120(	0.4335148	0.00024	down	yes	Alpha-L-fuco	pfam01120	Alpha_L_fucos	Alpha-L-fuc	GO:0005975|c	GO:0048046|	GO:0004560|alp	K01206
Manes.11G15130(	Inf	7.01E-06	up	yes	Serine carbox	pfam00450	Peptidase_S10	Serine carbo	.	GO:0005576|	GO:0004185|se	K16298
Manes.11G15140(	1.3955983	0.00133	up	no	Vacuolar pro	pfam03357	Snf7	Snf7. This f	GO:0070676|i	GO:0005829|	.	K12193
Manes.11G15160(	3.7133303	1.28E-24	up	yes	Probable gluc	pfam03016	Exostosin	Exostosin fa	GO:0071555|c	GO:0005794|	GO:0015020|glu	.
Manes.11G15170(	4.595837	2.47E-13	up	yes	.	pfam06813	Nodulin-like	Nodulin-like	.	.	.	.
Manes.11G15190(	0.9329306	0.73485	down	no	.	pfam07795	DUF1635	Protein of u	.	.	.	.
Manes.11G15200(	1.0783428	0.39923	up	no	DNA ligase 1	pfam01068	DNA_ligase_A_M	ATP depend	GO:0007049|c	GO:0005737|	GO:0005524|A'	K10747
Manes.11G15220(	0.6538344	0.00052	down	no	.	pfam06136	DUF966	Domain of u	.	.	.	.
Manes.11G15260(	Inf	5.33E-10	up	yes	Zinc finger pr	pfam00096	zf-C2H2	Zinc finger,	GO:0010447|r	GO:0005634|	GO:0046872|me	.
Manes.11G15300(	1.6301266	7.50E-08	up	no	Chromodoma	pfam00628	PHD	PHD-finger.	GO:0043044|a	GO:0005813|	GO:0005524|A'	K11643
Manes.11G15350(	4.6644006	9.40E-37	up	yes	.	pfam02029	Caldesmon	Caldesmon.	.	.	.	.
Manes.11G15390(	1.6663184	0.00014	up	no	Serine carbox	pfam00450	Peptidase_S10	Serine carbo	.	GO:0005576|	GO:0004185|se	K09646
Manes.11G15410(	1.2723137	0.00829	up	no	Vacuolar pro	pfam00637	Clathrin	Region in C	GO:0006886|i	GO:0005622|	GO:0008270|zin	.
Manes.11G15480(	1.3526284	0.00932	up	no	Aspartic prot	pfam00026	Asp	Eukaryotic a	.	GO:0031225|	GO:0004190|as	.
Manes.11G15510(	0.9035158	0.36053	down	no	SRSF protein	pfam00069	Pkinase	Protein kina	GO:0007059|c	GO:0005737|	GO:0005524|A'	K15409
Manes.11G15530(	6.6959609	1.95E-06	up	yes	GDSL estera	pfam00657	Lipase_GDSL	GDSL-like l	GO:0016042|l	GO:0005576|	GO:0016788|hy	.
Manes.11G15550(	2.252451	1.22E-18	up	yes	UDP-D-apios	pfam01370	Epimerase	NAD depen	GO:0071555|c	GO:0048046|	GO:0003824|ca	K12449
Manes.11G15570(	1.6000303	1.43E-07	up	no	AP-2 comple	pfam01602	Adaptin_N	Adaptin N te	GO:0006897|e	GO:0030131|	GO:0008289|lip	K11824
Manes.11G15580(	0.334067	2.60E-05	down	yes	Protein CHR	pfam02791	DDT	DDT domai	GO:0016568|c	GO:0005634|	GO:0005524|A'	.
Manes.11G15630(	0.2482515	3.70E-32	down	yes	Splicing facto	pfam13893	RRM_5	RNA recogn	GO:0006397|r	GO:0005634|	GO:0003677|D	K12836
Manes.11G15640(	0.1732856	5.56E-19	down	yes	.	pfam01559	Zein	Zein seed st	.	.	.	.
Manes.11G15700(	1.6544736	0.00096	up	no	.	pfam08324	PUL	PUL domain	.	.	.	.
Manes.11G15710(	0.1759091	6.83E-15	down	yes	Putative phyt	pfam06404	PSK	Phytosulfok	GO:0030154|c	GO:0005576|	.	.
Manes.11G15730(	5.5024999	2.58E-09	up	yes	Arogenate de	pfam00800	PDT	Prephenate c	GO:0009094|l	GO:0009507|	GO:0016597|an	K05359
Manes.11G15790(	2.3181579	7.24E-12	up	yes	.	pfam05910	DUF868	Plant protein	.	.	.	.
Manes.11G15810(	0.6934467	0.02079	down	no	.	pfam06455	NADH5_C	NADH dehy	.	.	.	.
Manes.11G15820(	1.1984203	0.04715	up	no	Uncharacteriz	pfam07876	Dabb	Stress respo	.	GO:0009507|	GO:0046872|me	.
Manes.11G15830(	0.6885445	0.60229	down	no	UPF0481 pro	pfam03140	DUF247	Plant protein	.	GO:0016021|	.	.
Manes.11G15990(	3.781902	0.00013	up	yes	Cytochrome	pfam00067	p450	Cytochrome	GO:0009611|r	GO:0016021|	GO:0018685|alk	.
Manes.11G16000(	0.6552018	0.00528	down	no	Proteinaceou	pfam11977	RNase_Zc3h12a	Zc3h12a-lik	GO:0006397|r	GO:0005634|	GO:0004526|rib	K18213
Manes.11G16010(	0.7613335	0.02296	down	no	Probable fatty	pfam07993	NAD_binding_4	Male sterility	GO:0006629|l	GO:0043231|	GO:0080019|fat	K13356
Manes.11G16070(	1.9552258	1.62E-13	up	no	Zinc finger A	pfam01428	zf-AN1	AN1-like Zi	.	.	GO:0003677|D	.
Manes.11G16080(	1.0153641	0.91969	up	no	CMP-N-acety	pfam00777	Glyco_transf_29	Glycosyltrar	GO:0006054|N	GO:0070062|	GO:0003836|be	K00780
Manes.11G16160(	1.8397359	4.32E-05	up	no	.	pfam12937	F-box-like	F-box-like.	.	.	.	.
Manes.11G16200(	2.9100679	5.80E-19	up	yes	Glucan endo-	pfam00332	Glyco_hydro_17	Glycosyl hyd	GO:0005975|c	GO:0046658|	GO:0042973|glu	.
Manes.11G16260(	0.347728	2.29E-09	down	yes	.	pfam11250	DUF3049	Protein of u	.	.	.	.
Manes.11G16310(	95.341561	4.99E-15	up	yes	Peroxidase 6(	pfam00141	peroxidase	Peroxidase.	GO:0042744|h	GO:0005576|	GO:0020037|he	K00430
Manes.11G16350(	44.991204	#######	up	yes	Transcription	pfam00249	Myb_DNA-binding	Myb-like DI	GO:0048262|c	GO:0005634|	GO:0003677|D	.
Manes.11G16360(	1.1678481	0.21634	up	no	Vesicle-assoc	pfam00957	Synaptobrevin	Synaptobrev	GO:0006887|e	GO:0005794|	GO:0005484|SN	K08515
Manes.11G16370(	2.4398803	1.87E-05	up	yes	Capsanthin/ca	pfam05834	Lycopene_cycl	Lycopene cy	GO:0016117|c	GO:0009509|	GO:0052727|ca	.
Manes.11G16380(	2.5503868	3.88E-21	up	yes	Mitochondria	pfam06258	Mito_fiss_Elm1	Mitochondri	GO:0000266|r	GO:0005741|	.	.

Manes.11G16390(	26.101325	0.00018	up	yes	Casparian str	pfam04535	DUF588	Domain of u	GO:0071555|	GO:0016021|	.	.
Manes.11G16400(	0.8291861	0.06363	down	no	OTU domain	pfam02338	OTU	OTU-like cy	GO:0071108|	.	GO:0004843|ub	.
Manes.11G16450(	2.8085488	9.25E-08	up	yes	.	pfam12937	F-box-like	F-box-like.	.	.	.	.
Manes.11G16460(	0.9804493	0.91067	down	no	.	pfam02463	SMC_N	RecF/RecN/	.	.	.	.
Manes.11G16470(	1.0080136	0.93558	up	no	Bifunctional	pfam00795	CN_hydrolase	Carbon-nitr	GO:0006807|	.	GO:0047427|cy	.
Manes.11G16480(	1.1536546	0.44004	up	no	Secoisolaricii	pfam00106	adh_short	short chain	.	.	GO:0016491|ox	.
Manes.11G16500(	9.6447562	2.51E-43	up	yes	Probable CCl	pfam04857	CAF1	CAF1 family	GO:0042742|	GO:0030014|	GO:0008408|3'-	K12581
Manes.11G16520(	0.6022746	9.56E-07	down	no	.	pfam06916	DUF1279	Protein of u	.	.	.	.
Manes.11G16530(	1.766437	5.06E-08	up	no	Transcription	pfam02319	E2F_TDP	E2F/DP fam	GO:0007049|	GO:0005737|	GO:0003677|DI	K06620
Manes.11G16570(	0.8246159	0.04703	down	no	Putative vacu	pfam12624	Chorein_N	N-terminal	GO:0031154|	GO:0019898|	.	.
Manes.12G00040(	1.0883566	0.36854	up	no	Type II inosit	pfam03372	Exo_endo_phos	Endonuclea	GO:0046856|	.	GO:0046872|m	.
Manes.12G00050(	0.6540443	9.19E-05	down	no	Beta-hexosar	pfam00728	Glyco_hydro_20	Glycosyl hy	GO:0005975|	GO:0005618|	GO:0004563|be	K12373
Manes.12G00090(	NA	NA	--	no	.	pfam09916	DUF2145	Uncharacter	.	.	.	.
Manes.12G00150(	3.7590717	2.42E-18	up	yes	Acyl-[acyl-ca	pfam03405	FA_desaturase_2	Fatty acid d	GO:0006633|	GO:0009507|	GO:0045300|ac	.
Manes.12G00170(	Inf	0.18198	up	no	.	pfam10325	7TM_GPCR_Srz	Serpentine t	.	.	.	.
Manes.12G00220(	0.5557348	0.01023	down	no	Ycf20-like pr	pfam04483	DUF565	Protein of u	GO:0010196|	GO:0009507|	.	.
Manes.12G00270(	0.6830931	0.0218	down	no	Endoribonucl	pfam04851	ResIII	Type III res	GO:0051607|	GO:0005737|	GO:0005524|A	K11592
Manes.12G00310(	1.2437181	0.02804	up	no	Peptide chain	pfam00472	RF-1	RF-1 domai	GO:0009657|	GO:0009507|	GO:0016149|tra	.
Manes.12G00370(	2.0884573	0.00377	up	yes	.	pfam00314	Thaumatin	Thaumatin f	.	.	.	.
Manes.12G00380(	8.0686509	5.32E-10	up	yes	Pathogenesis	pfam00314	Thaumatin	Thaumatin f	GO:0031540|	GO:0048046|	.	.
Manes.12G00420(	3.4312277	2.40E-43	up	yes	Glutamine--fi	pfam01380	SIS	SIS domain.	GO:0044267|	GO:0005829|	GO:0030246|ca	K00820
Manes.12G00430(	3.2184767	5.53E-35	up	yes	Aspartic prot	pfam14543	TAXi_N	Xylanase inl	.	GO:0031225|	GO:0004190|as	.
Manes.12G00470(	0.2851313	3.91E-06	down	yes	Pentatricopep	pfam13041	PPR_2	PPR repeat	GO:0006397|	GO:0009507|	.	.
Manes.12G00510(	0.7207523	0.00547	down	no	Riboflavin bi	pfam08719	DUF1768	Domain of u	GO:1901135|	GO:0009507|	GO:0008703|5-	.
Manes.12G00520(	1.4744619	1.91E-05	up	no	Basic leucine	pfam02020	W2	eIF4-gamma	GO:0030154|	GO:0016020|	.	.
Manes.12G00550(	1.2372714	0.02883	up	no	Isopentenyl-c	pfam00293	NUDIX	NUDIX dor	GO:0015995|	.	GO:0016787|hy	.
Manes.12G00620(	1.6203798	8.20E-08	up	no	.	pfam01169	UPF0016	Uncharacter	.	.	.	.
Manes.12G00650(	4.5040761	1.59E-25	up	yes	.	pfam08740	BCS1_N	BCS1 N ter	.	.	.	.
Manes.12G00660(	1.8605377	2.24E-12	up	no	Delta(3,5)-De	pfam00378	ECH	Enoyl-CoA	GO:0006635|	GO:0070062|	GO:0016853|iso	K12663
Manes.12G00670(	1.9181262	3.14E-06	up	no	Bidirectional	pfam03083	MtN3_slv	Sugar efflux	GO:0034219|	GO:0005887|	GO:0008515|su	K15382
Manes.12G00710(	0.3421518	5.63E-13	down	yes	Major pollen	pfam01190	Pollen_Ole_e_I	Pollen prote	.	GO:0005615|	.	.
Manes.12G00760(	1.6554297	1.68E-08	up	no	Bifunctional	pfam01842	ACT	ACT domai	GO:0009399|	.	GO:0008773|[p	K00990
Manes.12G00770(	0.955455	0.67579	down	no	Transcription	pfam03634	TCP	TCP family	GO:0009653|	GO:0005634|	GO:0000987|co	.
Manes.12G00780(	Inf	0.9009	up	no	.	pfam12070	DUF3550	Protein of u	.	.	.	.
Manes.12G00800(	7.125488	1.73E-43	up	yes	.	pfam05553	DUF761	Cotton fibre	.	.	.	.
Manes.12G00820(	0.6628188	0.01546	down	no	.	pfam13102	Phage_int_SAM_5	Phage integ	.	.	.	.
Manes.12G00840(	0.5375859	3.00E-06	down	no	.	pfam14033	DUF4246	Protein of u	.	.	.	.
Manes.12G00860(	1.4601197	7.22E-05	up	no	Sphingosine-	pfam00282	Pyridoxal_deC	Pyridoxal-d	GO:0019752|	GO:0005783|	GO:0016831|ca	K01634
Manes.12G00870(	1.5329698	0.24915	up	no	Probable WR	pfam03106	WRKY	WRKY DN	GO:0010055|	GO:0005634|	GO:0001046|co	.
Manes.12G00890(	2.1010667	2.65E-13	up	yes	Probable DN	pfam04104	DNA_primase_lrg	Eukaryotic	.	GO:1990077|	GO:0051539|4	.
Manes.12G00910(	5.236275	2.79E-05	up	yes	.	pfam09678	Caa3_CtaG	Cytochrome	.	.	.	.
Manes.12G00930(	4.6120377	2.53E-48	up	yes	.	pfam00124	Photo_RC	Photosynthe	.	.	.	.
Manes.12G00990(	1.0219896	0.85512	up	no	Stress-associ	pfam06624	RAMP4	Ribosome a	GO:0015031|	GO:0005789|	.	.
Manes.12G01040(	1.0358303	0.70685	up	no	RanBP2-type	pfam00641	zf-RanBP	Zn-finger in	.	.	GO:0008270|zi	.
Manes.12G01050(	0.8806389	0.27344	down	no	YTH domain	pfam04146	YTH	YT521-B-li	GO:0045948|	GO:0005737|	GO:1990247|N	.
Manes.12G01060(	1.8982255	2.19E-06	up	no	Non-specific	pfam00234	Tryp_alpha_amyl	Protease inh	GO:0042335|	GO:0031225|	GO:0008289|lip	.
Manes.12G01070(	0.1964909	0.0114	down	yes	Glucuronoxy	pfam04669	Polysacc_synt_4	Polysacchar	GO:0045491|	GO:0005794|	GO:0030775|gl	K18801
Manes.12G01080(	6.6559224	3.07E-16	up	yes	.	pfam01997	Translin	Translin fan	.	.	.	.
Manes.12G01090(	6.8201259	7.78E-05	up	yes	.	pfam11379	DUF3182	Protein of u	.	.	.	.
Manes.12G01100(	1.3004366	0.00387	up	no	.	pfam04359	DUF493	Protein of u	.	.	.	.
Manes.12G01130(	2.237135	1.06E-19	up	yes	F-box protei	pfam01753	zf-MYND	MYND fing	GO:0016567|	GO:0005634|	.	.
Manes.12G01150(	0.9888119	0.9497	down	no	REF/SRPP-li	pfam05755	REF	Rubber elon	.	GO:0005773|	.	.
Manes.12G01160(	4.4968999	1.06E-14	up	yes	HORMA dor	pfam02301	HORMA	HORMA do	GO:0042138|	GO:0005694|	.	.
Manes.12G01170(	0.9829723	0.86573	down	no	CTD small p	pfam03031	NIF	NLI interact	.	.	GO:0004721|ph	K17616
Manes.12G01200(	1.1313379	0.55449	up	no	Transcription	pfam10163	EnY2	Transcriptio	GO:0016578|	GO:0071819|	GO:0003713|tra	.
Manes.12G01220(	0.3899238	1.83E-07	down	yes	65-kDa micro	pfam03999	MAP65_ASE1	Microtubule	GO:0000910|	GO:0005737|	.	K16732
Manes.12G01260(	1.3824175	0.01469	up	no	Endoplasmic	pfam04389	Peptidase_M28	Peptidase fa	GO:0001541|	GO:0005783|	GO:0046872|m	.
Manes.12G01280(	2.9163016	1.44E-18	up	yes	Probable sign	pfam06645	SPC12	Microsomal	GO:0006465|	GO:0016021|	GO:0008233|pe	K12946
Manes.12G01290(	0.2972279	0.00029	down	yes	Putative ribo	pfam03193	DUF258	Protein of u	.	.	GO:0005525|G	K06949
Manes.12G01300(	2.0012138	5.56E-11	up	yes	.	pfam01344	Kelch_1	Kelch motif.	.	.	.	.
Manes.12G01320(	4.7311209	3.83E-09	up	yes	.	pfam00709	Adenylsucc_synt	Adenylosuc	.	.	.	.
Manes.12G01340(	2.2254048	2.63E-07	up	yes	Probable inac	pfam00069	Pkinase	Protein kina	.	GO:0016021|	GO:0005524|A	.
Manes.12G01370(	8.5443649	4.14E-07	up	yes	Probable mit	pfam14363	AAA_assoc	Domain ass	GO:0016226|	GO:0016021|	GO:0005524|A	K08900
Manes.12G01380(	0.9887401	0.91716	down	no	Mitochondria	pfam02096	60KD_IMP	60Kd inner	GO:0051205|	GO:0016021|	.	K03217
Manes.12G01420(	26.740579	8.06E-05	up	yes	.	pfam11882	DUF3402	Domain of u	.	.	.	.
Manes.12G01510(	3.0361837	7.13E-05	up	yes	.	pfam03798	TRAM_LAG1_CI	TLC domai	.	.	.	.
Manes.12G01560(	2.091219	5.57E-06	up	yes	.	pfam06522	B12D	NADH-ubiq	.	.	.	.
Manes.12G01570(	0.4996991	0.00363	down	yes	ABC transpo	pfam00664	ABC_membrane	ABC transp	.	GO:0048046|	GO:0005524|A	.
Manes.12G01580(	0.4150221	5.67E-05	down	yes	Mitotic spind	pfam02301	HORMA	HORMA do	GO:0051301|	GO:0010369|	.	.
Manes.12G01610(	0.3833536	5.25E-14	down	yes	.	pfam09588	YqaJ	YqaJ-like vi	.	.	.	.
Manes.12G01630(	3.0058722	6.73E-29	up	yes	F-box protei	pfam12937	F-box-like	F-box-like.	GO:0016567|	.	.	.
Manes.12G01750(	1.6864328	5.27E-06	up	no	Two-compor	pfam00072	Response_reg	Response re	GO:0009736|	GO:0005634|	GO:0003677|DI	K14491
Manes.12G01770(	0.1991386	2.33E-06	down	yes	.	pfam14950	DUF4502	Domain of u	.	.	.	.
Manes.12G01780(	0.2430025	5.53E-07	down	yes	Two-compor	pfam00072	Response_reg	Response re	GO:0009736|	GO:0005634|	GO:0003677|DI	K14491
Manes.12G01800(	0.5185896	0.00263	down	no	RING finger	pfam13639	zf-RING_2	Ring finger	GO:0051865|	.	GO:0003677|DI	.
Manes.12G01810(	Inf	0.38692	up	no	GDSL estera	pfam00657	Lipase_GDSL	GDSL-like	GO:0016042|	GO:0005576|	GO:0016788|hy	.
Manes.12G01820(	0.7760557	0.00866	down	no	.	pfam02463	SMC_N	RecF/RecN/	.	.	.	.
Manes.12G01830(	0.5878352	3.76E-05	down	no	.	pfam02517	Abi	CAAX prot	.	.	.	.
Manes.12G01860(	1.9031048	1.66E-09	up	no	.	pfam09726	Macoilin	Transmemb	.	.	.	.
Manes.12G01870(	1.2332927	0.45146	up	no	.	pfam08241	Methyltransf_11	Methyltrans	.	.	.	.
Manes.12G01880(	0.592285	8.74E-06	down	no	Photosystem	pfam06298	PsbY	Photosysten	GO:0015979|	GO:0009535|	GO:0030145|m	.
Manes.12G01900(	0.6684468	0.00792	down	no	Ultraviolet-B	pfam00415	RCC1	Regulator o	GO:0009649|	GO:0000785|	GO:0003682|ch	.
Manes.12G01920(	1.28191	0.00745	up	no	T-complex p	pfam00118	Cpn60_TCP1	TCP-1/cpn6	GO:0006457|	GO:0005829|	GO:0005524|A	K09497
Manes.12G01940(	0.474703	1.62E-10	down	yes	Oxysterol-bi	pfam01237	Oxysterol_BP	Oxysterol-b	GO:0006869|	.	GO:0008289|lip	.
Manes.12G01950(	1.3748068	0.0004	up	no	Protein SDA	pfam05285	SDA1	SDA1. This	GO:0030036|	GO:0005730|	.	K14856
Manes.12G01980(	8.8625913	9.49E-14	up	yes	Vignain	pfam00112	Peptidase_C1	Papain famil	.	GO:0005788|	GO:0008234|cy	.
Manes.12G01990(	1.3408254	0.00412	up	no	.	pfam09805	Nop25	Nucleolar p	.	.	.	.
Manes.12G02000(	15.366565	2.26E-45	up	yes	Myosin heav	pfam00400	WD40	WD domain	GO:0000281|	GO:0005826|	GO:0005524|A	.

Manes.12G02030(	1.2764771	0.03006	up	no	E3 ubiquitin-	pfam07002	Copine	Copine. Thi	GO:0009850|a	GO:0005634|	GO:0016874|lig	K16280
Manes.12G02070(	0.4663848	5.72E-10	down	yes	Serine/threon	pfam00069	Pkinase	Protein kina	GO:0006397|r	.	GO:0005524|A'	K08287
Manes.12G02090(	11.798632	#######	up	yes	Protein-tyros	pfam00782	DSPc	Dual specifi	GO:0006952|c	GO:0005829|	GO:0017017|M	.
Manes.12G02100(	18.52125	5.60E-08	up	yes	.	pfam05067	Mn_catalase	Manganese	.	.	.	.
Manes.12G02110(	1.4753677	0.00039	up	no	.	pfam02073	Peptidase_M29	Thermophili	.	.	.	.
Manes.12G02120(	0.8879511	0.22758	down	no	Uncharacteriz	pfam10250	O-FucT	GDP-fucose	.	GO:0005768|	GO:0016757|tra	.
Manes.12G02150(	3.9735437	6.32E-05	up	yes	GDSL estera	pfam00657	Lipase_GDSL	GDSL-like l	GO:0016042|l	GO:0005576|	GO:0016788|hy	.
Manes.12G02160(	0.5830267	7.38E-07	down	no	Alpha-L-fuco	pfam00657	Lipase_GDSL	GDSL-like l	.	GO:0048046|	GO:0004560|alp	K01206
Manes.12G02200(	2.5139851	0.00038	up	yes	Probable E3	pfam13639	zf-RING_2	Ring finger	GO:0009688|a	GO:0016021|	GO:0016874|lig	K16285
Manes.12G02210(	9.0792734	3.70E-47	up	yes	.	pfam11013	DUF2851	Protein of u	.	.	.	.
Manes.12G02220(	31.46172	#######	up	yes	.	pfam04724	Glyco_transf_17	Glycosyltrar	.	.	.	.
Manes.12G02230(	7.7007492	0.07931	up	no	.	pfam10268	Tmemb_161AB	Predicted tr	.	.	.	.
Manes.12G02240(	0.4218016	1.18E-07	down	yes	BTB/POZ do	pfam03000	NPH3	NPH3 famil	GO:0016567|r	.	.	.
Manes.12G02270(	12.886676	1.37E-26	up	yes	Non-function	pfam00248	Aldo_ket_red	Aldo/keto re	.	GO:0005829|	GO:0016491|ox	.
Manes.12G02280(	15.917675	0.00382	up	yes	E3 ubiquitin-	pfam13639	zf-RING_2	Ring finger	GO:0051301|c	GO:0016021|	GO:0016874|lig	K16286
Manes.12G02290(	0.7089496	0.01373	down	no	Integrator co	pfam10996	Beta-Casp	Beta-Casp d	GO:0016180|s	GO:0032039|	.	K13146
Manes.12G02320(	0.4281943	2.02E-15	down	yes	Inorganic pho	pfam01384	PHO4	Phosphate tr	GO:0035435|r	GO:0009507|	GO:0005315|ino	K14640
Manes.12G02370(	1.0238093	0.77973	up	no	Copper meth	pfam01179	Cu_amine_oxid	Copper ami	GO:0009308|a	.	GO:0052595|ali	.
Manes.12G02380(	9.2408557	1.36E-08	up	yes	.	pfam00514	Arm	Armadillo/b	.	.	.	.
Manes.12G02390(	1.3751855	0.01637	up	no	.	pfam08238	Sel1	Sel1 repeat.	.	.	.	.
Manes.12G02400(	2.7437527	2.38E-09	up	yes	.	pfam14897	EpsG	EpsG family	.	.	.	.
Manes.12G02410(	1.1635725	0.17424	up	no	Glutamate--g	pfam00155	Aminotran_1_2	Aminotransf	GO:0009058|l	GO:0009507|	GO:0008453|ala	K14272
Manes.12G02430(	0.8843522	0.23312	down	no	Anthranilate	pfam00591	Glycos_transf_3	Glycosyl tra	GO:0000162|t	.	GO:0004048|an	.
Manes.12G02450(	0.7056852	0.00048	down	no	AP-1 comple	pfam00928	Adap_comp_sub	Adaptor cor	GO:0006886|i	GO:0030131|	.	K12393
Manes.12G02530(	2.5587158	5.04E-07	up	yes	Cysteine-rich	pfam00069	Pkinase	Protein kina	GO:0009755|l	GO:0016021|	GO:0005524|A'	.
Manes.12G02540(	1.3137023	0.01596	up	no	Cysteine-rich	pfam00069	Pkinase	Protein kina	GO:0009755|l	GO:0016021|	GO:0005524|A'	.
[truncated: 2,176,709 more chars]
