# Supplementary material for: Genome-wide identification and functional prediction of cold and/or drought-responsive lncRNAs in cassava
Source: Sci Rep. 2017 Apr 7;7:45981. doi: 10.1038/srep45981 (PMC5384091; doi:10.1038/srep45981)

Supplementary information

Genome-wide identification and functional prediction of cold and/or drought-responsive lncRNAs in cassava

Shuxia Li[1†], Xiang Yu[2†], Ning Lei[1], Zhihao Cheng[3], Pingjuan Zhao[1], Yuke He[2], Wenquan Wang[1]& Ming Peng[1*]

Supplemental Figure S1. Physiological investigation and expression analysis of stress-responsive genes in cassava. (A) Phenotypic changes of cassava in response to PEG-induced drought stress. Bar=1 cm. (B-D) qRT-PCR analysis of the transcript levels of *MeRD26* (B), *MeRD29*(C) and *MeDREB2* (D) under 20%PEG treatment, respectively. (E, F) Relative expression levels of *MeCBF1* (E), and *MeCBF2* (E) response to cold stress, respectively. The values shown are the means ± standard deviation of three replicates. *MeACTIN* was used as the reference gene.

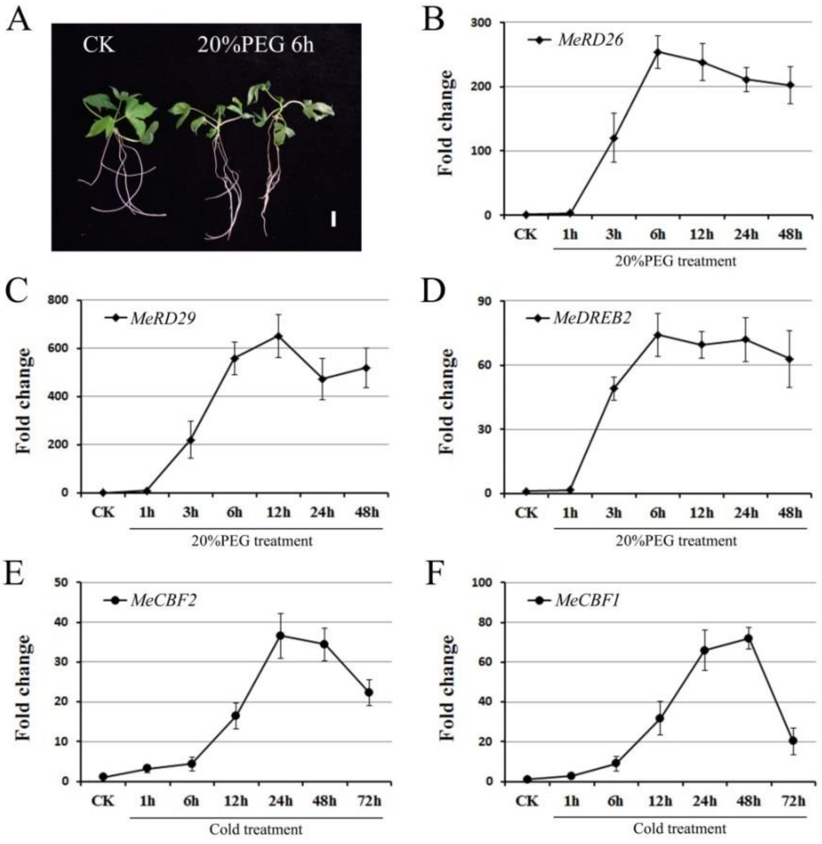

Supplemental Figure S2. GO analysis of the cold- or drought-responsive lncRNA targets.

The color of the rectangle represents q value.

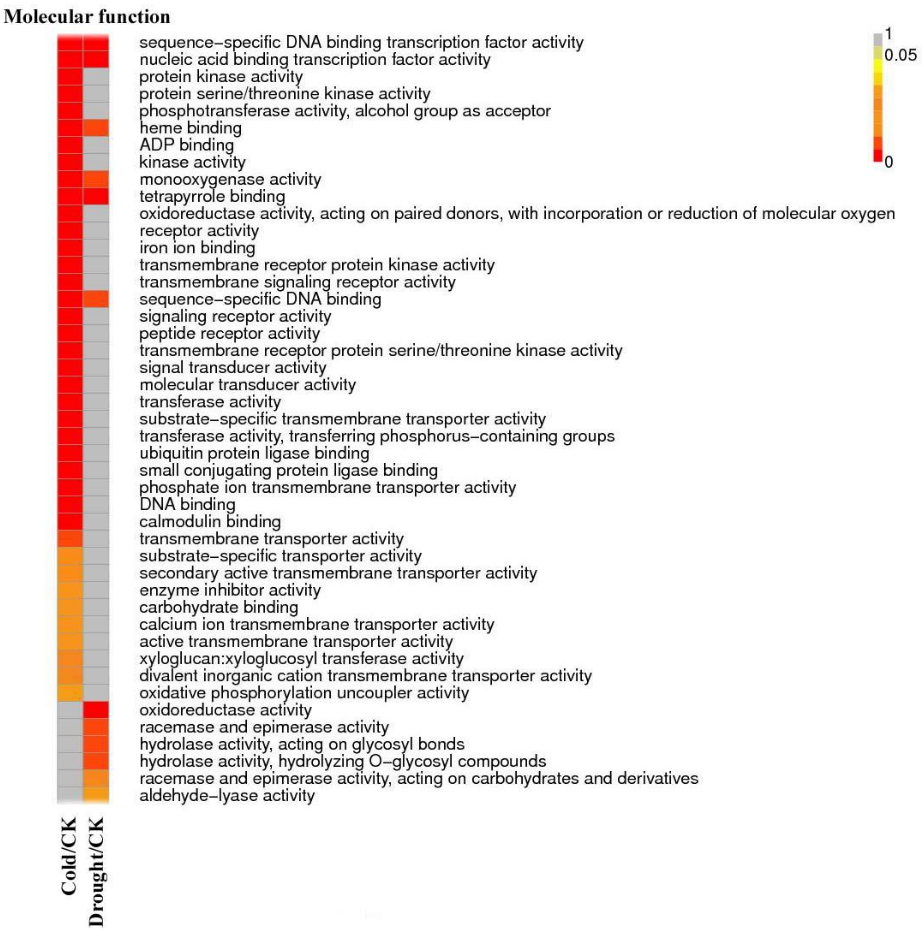

Supplement: Supplementary Figure [file srep45981-s2.pdf]
